# Life-course socioeconomic position and the gut microbiome in the Hispanic Community Health Study/Study of Latinos (HCHS/SOL)

**DOI:** 10.1080/19490976.2025.2479772

**Published:** 2025-03-18

**Authors:** Monica A. Batalha, Madison N. LeCroy, Juan Lin, Brandilyn A. Peters, Qibin Qi, Zheng Wang, Tao Wang, Linda C. Gallo, Gregory A. Talavera, Amanda C. McClain, Bharat Thyagarajan, Martha L. Daviglus, Lifang Hou, Maria Llabre, Jianwen Cai, Robert C. Kaplan, Carmen R. Isasi

**Affiliations:** a Department of Epidemiology and Population Health, Albert Einstein College of Medicine, Bronx, NY, USA; b Department of Population Health, NYU Grossman School of Medicine, New York, NY, USA; c Department of Psychology, San Diego State University, San Diego, CA, USA; d School of Exercise and Nutritional Sciences, San Diego State University, San Diego, CA, USA; e Department of Laboratory Medicine and Pathology, University of Minnesota, Minneapolis, MN, USA; f Institute for Minority Health Research, College of Medicine, University of Illinois at Chicago, Chicago, IL, USA; g Department of Preventive Medicine, Northwestern University, Chicago, IL, USA; h Department of Psychology, University of Miami, Miami, FL, USA; i Department of Biostatistics, University of North Carolina at Chapel Hill, Chapel Hill, NC, USA; j Public Health Sciences Division, Fred Hutchinson Cancer Center, Seattle, WA, USA

**Keywords:** Microbiome, sociobiome, Hispanic, Latino, socioeconomic factors, socioeconomic position, social determinants of health

## Abstract

Socioeconomic position (SEP) in childhood and beyond may influence the gut microbiome, with implications for disease risk. Studies evaluating the relationship between life-course SEP and the gut microbiome are sparse, particularly among Hispanic/Latino individuals, who have a high prevalence of low SEP. We use the Hispanic Community Health Study/Study of Latinos (HCHS/SOL), a population-based cohort study conducted in four field centers in the United States (U.S.), to evaluate the association between life-course SEP and gut microbiome composition. Life-course SEP indicators included parental education (proxy of childhood SEP), current SEP (*n* = 2174), and childhood (*n* = 988) and current economic hardship (*n* = 994). Shotgun sequencing was performed on stool samples. Analysis of Compositions of Microbiomes was used to identify associations of life-course SEP indicators with gut microbiome species and functions. Parental education and current SEP were associated with the overall gut microbiome composition; however, parental education and current education explained more the gut microbiome variance than the current SEP. A lower parental education and current SEP were associated with a lower abundance of species from genus *Bacteroides*. In stratified analysis by nativity, we found similar findings mainly among foreign-born participants. Early-life SEP may have long-term effects on gut microbiome composition underscoring another biological mechanism linking early childhood factors to adult disease.

## Introduction

The link between socioeconomic disadvantages throughout the life course and disparities in morbidity and mortality worldwide is well established.^1–3^ Socioeconomic factors affect cardiovascular and other health outcomes through multiple mechanisms, including behavioral (e.g., diet, physical activity, smoking), psychological (e.g., depression, anxiety), and biological (e.g., inflammation and stress physiology) pathways.^4^

Some evidence suggests that the gut microbiome is one of the biological factors that may explain the relationship between social factors and health-related outcomes.^5^ Further, the same social factors that contribute to health inequities may also predict gut microbiome composition.^6^ For example, several behavioral risks (e.g., smoking, nutrition) and environmental factors (e.g., living conditions, air pollution) linked to lower socioeconomic position (SEP) also affect the microbiota composition.^7–9^ Individuals with lower SEP also tend to have high levels of stress and inflammation.^10^ Previous work on the gut-brain axis observed that stress affects the neurohumoral communication between the gut and brain, altering gastrointestinal motility, secretion, and mucin production, resulting in dysregulation of microbial composition.^11,12^ In this scenario, some authors started to use the term “sociobiome” to define socio-economic factors as important components of the environmental influences that shape gut microbiome.^5,13^

Alterations in gut microbiome composition both in early and late life promote dysregulation of immune, metabolic, and neuroendocrine systems that are involved in several diseases, such as cardiovascular disease (CVD), type 2 diabetes mellitus, chronic kidney disease, and some types of cancer.^14–18^

To date, few studies have investigated the association between SEP and gut microbiome in adults, and results are mixed. In a US cohort, Kwak et al. found that lower individual educational attainment was associated with greater microbial α-diversity (Faith’s phylogenetic diversity index).^13^ However, in the same study, the occupational socioeconomic index (individual-level SEP) and two neighborhood-level SES independent indicators (neighborhood income and social deprivation index) were not associated with alpha diversity.^13^ A study in twin adults in the United Kingdom (UK) found that education was not associated with different alpha-diversity indexes (Shannon, Chao1, and Simpson index). However, the same study found that higher levels of income and area-level SEP (composite index) were positively correlated to these different alpha diversity indexes.^19^ Previous studies also found the lower neighborhood SEP (composite index) was associated with a lower alpha diversity.^20,21^ The different definitions of SEP using composite metrics (e.g., composite neighborhood index) or independent indicators (e.g., education and income) may explain these mixed results.

Extensive research has been focusing on early-life determinants of the children’s gut microbiome. However, the lifetime microbial consequences of early-life socioeconomic adversity are still largely unknown.^22^ Large studies evaluating the relationship between both childhood and current SEP/economic hardship and the gut microbiome are sparse, particularly among Hispanic/Latino individuals, who have a high prevalence of low SEP. Thus, as the first aim, we assessed whether parental education (as a proxy of childhood SEP) and current SEP are associated with gut microbiome diversity and overall composition in the Hispanic Community Health Study/Study of Latinos (HCHS/SOL). In addition, as a secondary aim, we also explored whether childhood and current economic hardship were associated with these gut microbiome characteristics in a subset of participants.

## Materials and methods

### Study design and population

The HCHS/SOL is a prospective, population-based cohort study of 16,415 hispanic/Latino adults that aims to identify the risk and protective factors for cardiovascular disease and other chronic conditions. From 2008 to 2011 (baseline), HCHS/SOL recruited participants aged 18 to 74 years old using a multi-stage probability sampling design from randomly sampled census block areas within the 4 field centers across the U.S. (Bronx, NY; Chicago, IL; Miami, FL; San Diego, CA).^23,24^ The HCHS/SOL Gut Origins of Latino Diabetes (GOLD) ancillary study was conducted to examine the role of gut microbiome composition on diabetes and other outcomes, enrolling ~ 3,000 participants from HCHS/SOL approximately concurrent with the second in-person HCHS/SOL visit cycle (2014–2017).^25^ For this manuscript, 2,713 participants had complete data for the gut microbiome. We excluded participants with <100,000 sequence reads in their microbiome sample (*N* = 173). Of the 2,540 participants with microbiome data, 2,174 participants had information on parental education and current SEP and independent variables at HCHS/SOL baseline. For our secondary analysis, we used a subset of participants that had information on childhood (*n* = 988) and current (*n* = 994) economic hardship, which was collected in the HCHS/SOL Sociocultural Ancillary Study (in a separate visit within 9 months of HCHS/SOL baseline).^26^ The study was approved by the institutional review boards of corresponding site institutions. Written informed consent was obtained from all participants.

### Life course SEP indicators

Parental education was determined using participants’ self-reported maternal or paternal educational attainment. We selected the highest education achieved by either the father or mother and created the following categories: a lower (both father and mother with <high school) and a higher (father or mother with ≥high school) parental education. In this study, we used parental education as a proxy for childhood SEP.

Current SEP was assessed using the combination of participants’ self-reported educational attainment (<high school or ≥high school) and current annual household income (<$30,000 or ≥$30,000). We selected the highest education and household income achieved by the participant and created the following categories: a lower (<high school and <$30,000) and a higher (participant with ≥high school or ≥$30,000) current SEP. We also conducted a sensitivity analysis using current education (<high school or ≥high school) as an independent SEP indicator in our study.

Childhood Economic Hardship was measured by the question, “Did your family ever experience a period of time when they had trouble paying for their basic needs, such as food, housing, medical care, and utilities, when you were a child?” and was used as a dichotomous variable (yes, no).

Current economic hardship was measured by the question, “In the past 12 months, was there ever a time when you had trouble paying for your basic needs, such as food, housing, medical care, and utilities?” and was used as a dichotomous variable (yes, no).

### Stool sample collection, sequencing, and microbiome bioinformatics processing

GOLD ancillary study participants were provided with a stool self-collection kit and a single fecal specimen was collected using a disposable paper inverted hat (Protocult collection device, ABC Medical Enterprises, Inc., Rochester, MN), as previously described.^25^ Shotgun sequencing was conducted at the Knight laboratory at the University of California San Diego using a shallow approach.^27^ The DNA was extracted from fecal samples following the Earth Microbiome Project protocol.^28^ The adapters and barcode indices were processed following the iTru adapter protocol,^29^ and the resulting libraries were purified, quantified, and normalized for sequencing on Illumina NovaSeq.

FASTQ sequence reads were processed using the standard shotgun sequencing pipeline in Qiita.^30^ Sequence adapters were removed via fastp, and sequence reads mapping to the human genome (GRCh38) were filtered via minimap2.^31,32^ The sequence reads were then aligned against the WolR1^33^ reference database of bacterial and archaeal genomes using Woltka with the Bowtie2 aligner^34^ to generate an operational genomic unit (OGU) table and a gene table. The sequence alignments were also classified at the species taxonomic rank, while functional profiles were obtained by collapsing the gene table into MetaCyc enzymatic reactions and functional pathways.^35^

### Covariates

Covariates were chosen based on previous literature.^13,19–21,36^ Sociodemographic characteristics, lifestyle behaviors, mental health, and clinical factors related to SEP indicators and gut microbiome were included in our models. The sociodemographic characteristics included age (continuous), sex (male, female), U.S. nativity (including 50 states and the District of Columbia (US-born, foreign-born)), field center (Chicago, Miami, Bronx, and San Diego), and Hispanic/Latino background (Central American, Cuban, Dominican, Mexican, Puerto Rican, South American, and Other/Mixed). We also evaluated stool type, using the Bristol scale (7 categories), and BMI (continuous). Lifestyle/behavior factors included the Alternative Healthy Eating Index 2010^37^ (AHEI 2010) score (continuous (range = 0–110) with higher scores indicating better diet quality), cigarette smoking (never, former, current), and alcohol use (never, former, current). Among mental health factors, depression symptoms were measured with the 10-item Center for Epidemiological Studies-Depression Scale^38^ (CES-D) (continuous (range = 0–30), with higher scores indicating worse depression symptoms) and anxiety symptoms were measured using the 10-item Spielberger State-Trait Anxiety Inventory^39^ (STAI) (continuous (range = 0–40), with higher scores indicating worse anxiety symptoms). Clinical factors included diabetes (defined based on American Diabetes Association criteria or self-report of antidiabetic medication (no, prediabetes, diabetes)), hypertension (defined as systolic or diastolic blood pressure ≥ 140/90 or self-report of antihypertensive medications (yes, no)), dyslipidemia (defined as LDL-c ≥160 mg/dL or HDL-c <40 mg/dL or Triglycerides ≥200 mg/dL and self-report of antihypertensive medications (no, without treatment, with treatment)), CVD (yes, no), and antibiotic use in the last 6 months (yes, no). Considering that the dataset had minimal missing values in covariates, we imputed the covariates in which the maximum number of missing values was 21 (≤1% of *n* = 2174) by replacing them with the median for continuous variables and mode for categorical variables (except for categorical variables with > 1% missing, which were set to missing in a new category).

### Statistical analysis

The primary goal of the analysis was to examine the association of SEP indicators with diversity and overall composition of the gut microbiome. We adjusted our models for age, sex, BMI, field center, Hispanic/Latino background, U.S. nativity, Bristol stool type, AHEI 2010 score, cigarette smoking, alcohol use, depression and anxiety symptoms, diabetes, hypertension, CVD, dyslipidemia, and antibiotic use. We additionally adjusted the models that the current SEP was the exposure for parental education and the models that parental education was the exposure was adjusted for current SEP.

All analyses were implemented in R (version 4.4.1). The Benjamini-Hochberg false discovery rate (FDR) method was used for multiple testing corrections at 5%. The vegan and phyloseq R packages were used to calculate α-diversity (Shannon, Inverse Simpson, Chao1 and Observed indexes) and β-diversity (Jensen-Shannon Divergence [JSD]) from the OGU table.^40,41^ The nlme R package was used to conduct linear regression models. Multivariable linear regression models were used to investigate the association of SEP indicators with the alpha diversity indexes (Shannon, Inverse Simpson, Chao1, Observed), adjusting for covariates previously described. The results of principal coordinates analysis (PCoA) were plotted to create a visual representation of the microbial community compositional differences (β-diversity). Permutational multivariate analysis of variance (PERMANOVA) was used to estimate the association of life-course SEP indicators with overall microbiome composition, as measured by the JSD, adjusting for covariates. Statistical significance was set at <0.05.

The analysis of Composition of Microbiomes (ANCOM2)^42^ was used to detect species, pathways, and enzymatic reactions for which abundance was linked to life-course SEP indicators, adjusting for covariates. We controlled the FDR at 5% and excluded species, pathways, or enzymatic reactions if they were present in less than 20% of the participants. An ANCOM2 detection level ≥ 0.7 was considered significant. Multivariable linear regression models were performed to investigate the direction and magnitude of the associations between the life-course SEP indicators (predictors) and centered log ratio (CLR)- transformed species/pathway/reaction abundance (outcomes), adjusting for the previously described covariates. Spearman’s correlations were used to assess the relationships between ANCOM-detected species, pathways, and enzymatic reaction abundance. We used ggtreeExtra R package for tree data visualization in a circular layout and to include data graph layers (e.g., heatmap) in the tree.^43^ The phylogenetic tree was downloaded from the WoL website and corresponds to the WolR1 database (https://biocore.github.io/wol/download).

#### Sensitivity analysis

We performed stratified analysis to evaluate whether the association between parental education and current SEP and gut microbiome differ by U.S. nativity (US-born and foreign-born).

In addition, we performed a sensitivity analysis including current education as independent exposure in our models to have a similar metric to the one used to define childhood SEP (based on parental education).

## Results

Approximately 14% of participants in the current study were born in the U.S. 50 states/D.C. and 64% were women. Parental education less than high school (proxy of lower childhood SEP) was reported by 64% of participants and 27% had lower current SEP. Participants with childhood or current lower SEP were more likely to be older, to have been born outside the U.S., to have healthier diets, to have higher mean scores for anxiety symptoms, or to have a higher prevalence of diabetes than those with a higher SEP (Table 1).

**Table 1.** Characteristics by parental education and current socioeconomic position (SEP) among participants in the HCHS/SOL.

		Parental education^a^			Current SEP^b^		
Characteristic^c^	Overall	Lower	Higher	p^d^	Lower	Higher	p^d^
N	2174	1391(64.0%)	783(36.0%)		592(27.2%)	1582(72.8%)	
Age, years	50.5 ± 11.0	52.2 ± 9.8	47.6 ± 12.3	<0.01	52.5 ± 10.8	49.7 ± 11.0	<0.01
Sex				0.01			0.01
Female	1396(64.2%)	921(66.2%)	475(60.7%)		405(68.4%)	991(62.6%)	
Male	778(35.8%)	470(33.8%)	308(39.3%)		187(31.6%)	591(37.4%)	
U.S. nativity^e^				<0.01			<0.01
Foreign-born	1872(86.1%)	1276(91.7%)	596(76.1%)		557(94.1%)	1315(83.1%)	
US-born	302(13.9%)	115(8.3%)	187(23.9%)		35(5.9%)	267(16.9%)	
Hispanic Background				<0.01			<0.01
Dominican	213(9.8%)	148(10.6%)	65(8.3%)		58(9.8%)	155(9.8%)	
Central American	197(9.1%)	140(10.1%)	57(7.3%)		65(11%)	132(8.3%)	
Cuban	256(11.8%)	128(9.2%)	128(16.3%)		34(5.7%)	222(14%)	
Mexican	937(43.1%)	674(48.5%)	263(33.6%)		314(53%)	623(39.4%)	
Puerto Rican	357(16.4%)	192(13.8%)	165(21.1%)		84(14.2%)	273(17.3%)	
South American	160(7.4%)	85(6.1%)	75(9.6%)		26(4.4%)	134(8.5%)	
Mixed/Other	54(2.5%)	24(1.7%)	30(3.8%)		11(1.9%)	43(2.7%)	
Parental education				<0.01			<0.01
<High School	1453(57.2%)	1453(100%)	0(0%)		489(69.8%)	902(52.7%)	
High School	452(17.8%)	0(0%)	452(55.1%)		66(9.4%)	368(21.5%)	
>High School	369(14.5%)	0(0%)	369(44.9%)		37(5.3%)	312(18.2%)	
Current Education				<0.01			<0.01
<High School	1572(61.9%)	772(53.1%)	689(83.9%)		701(100%)	1514(88.5%)	
≥High School	960(37.8%)	680(46.8%)	132(16.1%)		0(0%)	197(11.5%)	
Household income, $				<0.01			<0.01
<30K	1627(64.1%)	957(65.9%)	485(59.1%)		701(100%)	923(53.9%)	
≥30K	790(31.1%)	435(29.9%)	298(36.3%)		0(0%)	788(46.1%)	
BMI, kg/m^b^	30.1 ± 5.7	30.1 ± 5.5	30.0 ± 6.1	0.54	30.3 ± 5.7	30.0 ± 5.7	0.30
AHEI 2010^f^	50.6 ± 7.5	51.8 ± 7.2	48.5 ± 7.7	<0.01	52.2 ± 7.3	50.0 ± 7.5	<0.01
Alcohol use				<0.01			<0.01
Never	424(19.5%)	270(19.4%)	154(19.7%)		133(22.5%)	291(18.4%)	
Former	771(35.5%)	527(37.9%)	244(31.2%)		243(41%)	528(33.4%)	
Current	979(45%)	594(42.7%)	385(49.2%)		216(36.5%)	763(48.2%)	
Cigarette use				<0.01			0.55
Never	1346(61.9%)	895(64.3%)	451(57.6%)		362(61.1%)	984(62.2%)	
Former	480(22.1%)	307(22.1%)	173(22.1%)		127(21.5%)	353(22.3%)	
Current	348(16%)	189(13.6%)	159(20.3%)		103(17.4%)	245(15.5%)	
Diabetes^g^				<0.01			<0.01
No	1122(51.6%)	704(50.6%)	418(53.4%)		160(27%)	560(35.4%)	
Prediabetes	667(30.7%)	410(29.5%)	257(32.8%)		269(45.4%)	705(44.6%)	
Diabetes	385(17.7%)	277(19.9%)	108(13.8%)		163(27.5%)	317(20%)	
Dyslipidemia^h^				<0.01			<0.01
No	1122(51.6%)	704(50.6%)	418(53.4%)		278(47%)	844(53.4%)	
Without treatment	667(30.7%)	410(29.5%)	257(32.8%)		168(28.4%)	499(31.5%)	
With treatment	385(17.7%)	277(19.9%)	108(13.8%)		146(24.7%)	239(15.1%)	
Hypertension^i^	782(36%)	521(37.5%)	261(33.3%)	0.06	230(38.9%)	552(34.9%)	0.09
Prevalence CVD	130(6%)	97(7%)	33(4.2%)	0.01	38(6.4%)	92(5.8%)	0.46
Antibiotic use	1570(72.2%)	1032(74.2%)	538(68.7%)	0.01	426(72%)	1144(72.3%)	0.87
CES-D^j^	7.3 ± 6.1	7.5 ± 6.0	7.1 ± 6.2	0.06	8.8 ± 6.5	6.8 ± 5.8	<0.01
STAI^k^	17.1 ± 5.8	17.4 ± 5.8	16.6 ± 5.6	<0.01	18.8 ± 6.0	16.6 ± 5.5	<0.01

AHEI 2010: Alternative Healthy Eating Index 2010, BMI: body mass index, CES-D: Center for Epidemiological Studies-Depression Scale, CVD: cardiovascular disease, HCHS/SOL: Hispanic Community Health Study/Study of Latino, SEP: socioeconomic position, STAI: Spielberger State-Trait Anxiety Inventory.

^a^Parental education was determined using participants’ self-reported parental (mother or father) educational attainment: a lower (both father and mother with <high school) and a higher (father or mother with ≥high school).

^b^Current SEP was assessed using the combination of the highest education and household income achieved by the participant: a lower (<high school and <$30,000) and a higher (participant with ≥high school or ≥$30,000).

^c^Continuous variables are presented as means (SD). Categorical variables are presented as n (%).

^d^P-value from Wilcoxon rank-sum test for continuous variables or Chi-square test for categorical variables.

^e^U.S. nativity included 50 states and the District of Columbia.

^f^AHEI 2010 score (continuous (range = 0–110) with higher scores indicating better diet quality

^g^Diabetes was defined based on American Diabetes Association criteria or self-report of antidiabetic medication.

^h^Dyslipidemia was defined as LDL-c ≥160 mg/dL or HDL-c <40 mg/dL or Triglycerides ≥200 mg/dL and self-report of antihypertensive medications.

^i^Hypertension was defined as systolic or diastolic blood pressure ≥ 140/90 or self-report of antihypertensive medications.

^j^10-item CES-D (continuous (range = 0–30), with higher scores indicating worse depression symptoms.

^k^10-item STAI (continuous (range = 0–40), with higher scores indicating worse anxiety symptoms.

### SEP and gut microbiome diversity and overall composition

**Figure 1.**
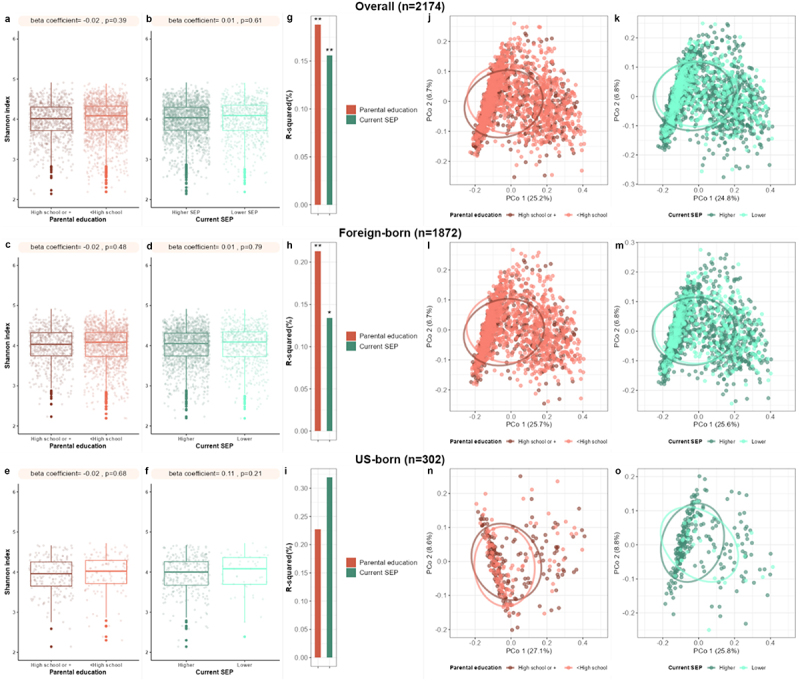
Parental education and current socioeconomic position (SEP) and gut microbiome diversity and overall composition. Models were adjusted for age, sex, BMI, field center, Hispanic/Latino background, U.S. nativity, Bristol stool type, parental education (models that current SEP or current education were the exposure) or current SEP (models that parental education was the exposure), the alternative Healthy Eating Index 2010 score, cigarette smoking, alcohol use, depressive and anxiety symptoms, diabetes, hypertension, cardiovascular disease, dyslipidemia, and antibiotic use. (a) Beta coefficients from multivariable linear regression of parental education and Shannon diversity index in overall participants. (b) Beta coefficients from multivariable linear regression of current SEP and Shannon diversity index in overall participants. (c) Beta coefficients from multivariable linear regression of parental education and Shannon diversity index in foreign-born participants. (d) Beta coefficients from multivariable linear regression of current SEP and Shannon diversity index in foreign-born participants. (d) Beta coefficients from multivariable linear regression of parental education and Shannon diversity index in us-born participants. (e) Beta coefficients from multivariable linear regression of current SEP and Shannon diversity index in us-born participants. (g) Bar plots represent R-squared values for parental education and current SEP from permanova-adjusted models of the Jensen-Shannon divergence in overall participants. *,*p* < 0.05; **,*p* ≤ 0.01. (h) Bar plots represent R-squared values for parental education and current SEP from permanova-adjusted models of the Jensen-Shannon divergence in foreign-born participants. *, *p* < 0.05; **, *p* ≤ 0.01. (i) bar plots represent R-squared values for parental education and current SEP from permanova-adjusted models of the Jensen-Shannon divergence in us-born participants. *,*p* < 0.05; **,*p* ≤ 0.01. (j) Principal coordinate analysis of the Jensen-Shannon divergence in overall participants according to parental education. Sample points are colored by parental education groups. (k) Principal coordinate analysis of the Jensen-Shannon divergence in overall participants according to current SEP. Sample points are colored by current SEP groups. (l) Principal coordinate analysis of the Jensen-Shannon divergence in foreign-born participants according to parental education. Sample points are colored by parental education groups. (m) Principal coordinate analysis of the Jensen-Shannon divergence in foreign-born participants according to current SEP. Sample points are colored by current SEP groups. (n) Principal coordinate analysis of the Jensen-Shannon divergence in us-born participants according to parental education. Sample points are colored by parental education groups. (o) Principal coordinate analysis of the Jensen-Shannon divergence in us-born participants according to current SEP. Sample points are colored by current SEP groups.

Life course SEP indicators were not associated with the alpha diversity indexes in multivariable linear regression models, except the observed index for current SEP (Figure 1(a,b); Table 2). Nevertheless, in multivariable permutational multivariate analysis of variance (PERMANOVA) models, life course SEP indicators were associated with the overall gut microbiome composition (Figure 1(g)). Differences in overall microbiome composition by life course SEP indicators were not clear in the principal coordinate analysis of the JSD (Figure 1(j,k)).

**Table 2.** Association of parental education and current SEP with gut microbiome alpha diversity indexes (Shannon, Inverse Simpson, Chao1, observed).

Exposure	index	Model^a^	p
Parental education	Shannon	−0.0192 (−0.0634, 0.0249)	0.39
Parental education	Inverse Simpson	−0.5948 (−1.8473, 0.6577)	0.35
Parental education	Chao1	8.9053 (−11.9064, 29.717)	0.40
Parental education	Observed	6.0356 (−9.9672, 22.0385)	0.46
Current SEP	Shannon	0.012 (−0.0342, 0.0581)	0.61
Current SEP	Inverse Simpson	0.6197 (−0.6887, 1.9282)	0.35
Current SEP	Chao1	20.391 (−1.4407, 42.2227)	0.07
Current SEP	Observed	17.9864 (1.2022, 34.7706)	0.04
Current education	Shannon	0.0054 (−0.038, 0.0488)	0.81
Current education	Inverse Simpson	0.4112 (−0.8229, 1.6453)	0.51
Current education	Chao1	17.8564 (−2.668, 38.3808)	0.09
Current education	Observed	16.3117 (0.5342, 32.0891)	0.04

^a^Models were adjusted for age, gender, BMI, field center, Hispanic/Latino background, U.S. nativity, Bristol stool type, parental education (models that current SEP or current education were the exposure) or current SEP (models that parental education was the exposure), the alternative Healthy Eating Index 2010 score, cigarette smoking, alcohol use, depressive and anxiety symptoms, diabetes, hypertension, cardiovascular disease, dyslipidemia, and antibiotic use.

### SEP and gut microbiome species

**Figure 2.**
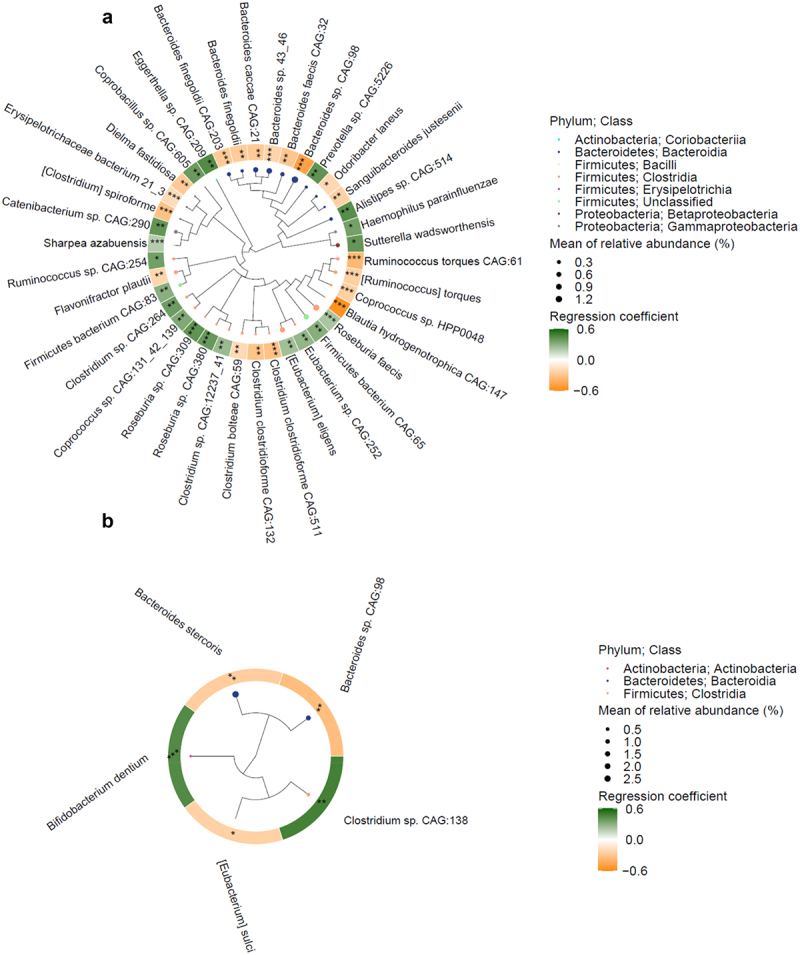
Gut microbiome species associated with parental education and current socioeconomic position (SEP). (a) Phylogenetic tree of 38 species associated with parental education. Analysis of composition of microbiomes (ANCOM2) was used to detect species associated with parental education (detection level ≥0.7), adjusted for age, sex, BMI, field center, Hispanic/Latino background, U.S. nativity, Bristol stool type, current SEP, AHEI 2010 score, cigarette smoking, alcohol use, depression and anxiety symptoms, diabetes, hypertension, cardiovascular disease, dyslipidemia, and antibiotic use. (b) Phylogenetic tree of 5 species associated with current SEP. ANCOM2 models were used to detect species associated with current SEP (detection level ≥0.7), adjusted for age, sex, BMI, field center, Hispanic/Latino background, U.S. nativity, parental education, Bristol stool type, AHEI 2010 score, cigarette smoking, alcohol use, depression and anxiety symptoms, diabetes, hypertension, cardiovascular disease, dyslipidemia, and antibiotic use. Node size represents the mean relative abundance of each species and node colors represent taxonomic class. The circular heatmap around the phylogenetic tree shows the beta coefficients from multivariable linear regression of parental education and current SEP on clr-transformed species abundance, adjusted for the same covariates listed above. *,*p* < 0.05; **,*p* ≤ 0.01; ***,*p* ≤ 0.001.

Of 1177 species tested in Analysis of Composition of Microbiomes (ANCOM2), 38 and 5 species were associated with childhood and current SEP, respectively, at a detection level ≥ 0.7 in the fully adjusted model (Figure 2). Of the 38 species related to the lower parental education (proxy of lower childhood SEP), 19 had enriched abundance and 19 had depleted abundance, including species from the genus *Bacteroides* (Figure 2(a); Supplementary Table S1).

A lower current SEP was associated with higher abundance of *Bifidobacterium dentium* and *Clostridium* sp. CAG:138, and with lower abundance of *[Eubacterium] sulci, Bacteroides stercoris*, and *Bacteroides* species CAG:98 (Figure 2(b); Supplementary Table S2). This latter species had also a lower abundance in participants who reported a lower parental education (Figure 2(a)).

### SEP and gut microbiome functions

Of 658 functional pathways tested in ANCOM2, three pathways were associated with a lower parental education (lower childhood SEP) at a detection level ≥ 0.7 in the fully adjusted model (Supplementary Table S3). A lower parental education was associated with a lower abundance of GDP-6-deoxy-D-altro-heptose biosynthesis, tRNA-uridine 2-thiolation (thermophilic bacteria), and conversion of succinate to propanoate functions (Supplementary Table S3). Of 1063 enzymatic reactions tested, lower parental education was related to lower abundance of GDP-6-deoxy-4-keto-D-arabino-heptose 4-reductase, [sulfur carrier protein TuB] adenylyltransferase, and propionyl-CoA:succinate CoA transferase reactions (Supplementary Table S5). We did not detect functions that differed in abundance by current SEP (Supplementary Tables S4 and S6).

**Figure 3.**
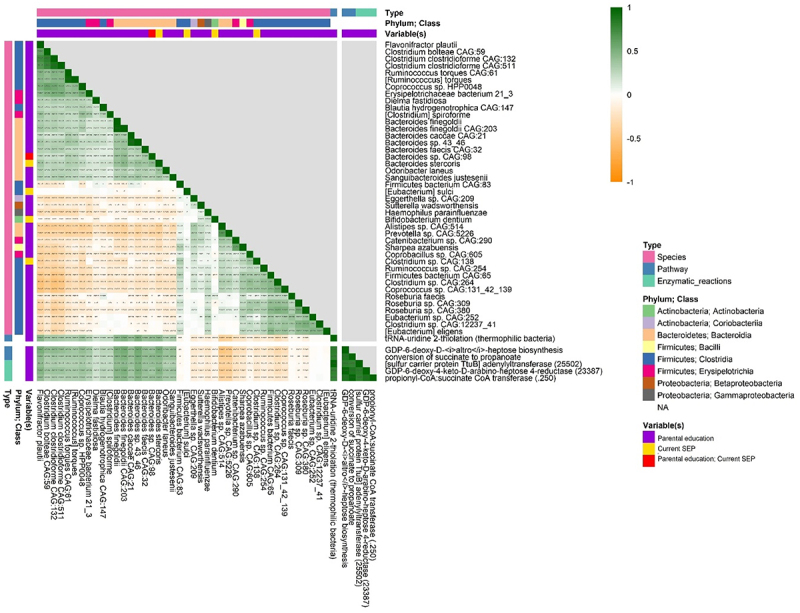
Correlation between gut microbiome species and functions. We used ANCOM2 to identify MetaCyc functional pathways and enzymatic reactions for which abundance was associated with parental education and current SEP. Only sep-related species and functions were included in the matrix (ANCOM2 detection level ≥0.7). We conducted Spearman’s correlations of sep-related clr-transformed species, pathways, and enzymatic reactions. *,*p* < 0.05; **,*p* ≤ 0.01; ***,*p* ≤ 0.001; ****,*p* ≤ 0.0001.

We observed moderate (*r* ≥ 0.40) positive correlations of the above-mentioned functional pathways and enzymatic reactions with *Flavonifractor plautii*, *Bacteroides finegoldii, Bacteroides finegoldii* CAG:203, *Bacteroides* sp. 43_46, and *Bacteroides stercoris* (Figure 3).

### Childhood and current economic hardship

Childhood and current economic hardship were reported by 50.3% and 52.9% of the participants, respectively (Table 3). Childhood and current economic hardship were not associated with the alpha diversity indexes (Supplementary Table S7). In PERMANOVA models, participants did not differ in overall gut microbiome composition by childhood or current economic hardship (Supplementary Table S8). Childhood economic hardship was associated with a higher abundance of *Haemophilus parainfluenzae (*also enriched in participants with a lower parental education), *Haemophilus sputorum*, and *Granulicatella adiacens* and with a lower abundance of *Lachnospiraceae* bacterium NLAE-zl-G231 (Supplementary Table S9). Childhood economic hardship was also associated with a higher abundance of the molybdenum cofactor biosynthesis functional pathway (Supplementary Table S10) and with a lower abundance of methylthioalkylmalate dehydrogenase, isopropylmalate dehydrogenase, and fumarase reactions (Supplementary Table S11). We did not find differences in species, functional pathways, and enzymatic reactions abundance by current economic hardship (Supplementary Tables S12,S13,S14).

**Table 3.** Characteristics by childhood and current economic hardship among participants in the HCHS/SOL study.

		Childhood economic hardship(*n* = 988)^a^			Current economic hardship(*n* = 994)^b^		
Characteristic^c^		Yes	No	p^d^	Yes	No	p^d^
N		497(50.3%)	491(49.7%)		526 (52.9%)	468(47.1%)	
Age, years	51.3 ± 11	51.3 ± 10.8	51.3 ± 11.2	0.78	49.9 ± 10.5	53 ± 11.3	<0.01
Sex				0.84			0.59
Female	646(65%)	325(65.4%)	318(64.8%)		346(65.8%)	300(64.1%)	
Male	348(35%)	172(34.6%)	173(35.2%)		180(34.2%)	168(35.9%)	
U.S. nativity^e^				0.79			0.72
Foreign-born	846 (85.1%)	425 (85.5%)	416 (84.7%)		450 (85.6%)	396 (84.6%)	
US-born	148(14.9%)	72(14.5%)	75(15.3%)		76(14.4%)	72(15.4%)	
Hispanic Background				0.01			0.67
Dominican	122(12.3%)	61(12.3%)	61(12.4%)		68(12.9%)	54(11.5%)	
Central American	87(8.8%)	49(9.9%)	37(7.5%)		49(9.3%)	38(8.1%)	
Cuban	134(13.5%)	49(9.9%)	85(17.3%)		75(14.3%)	59(12.6%)	
Mexican	394(39.6%)	216(43.5%)	175(35.6%)		204(38.8%)	190(40.6%)	
Puerto Rican	174(17.5%)	81(16.3%)	92(18.7%)		83(15.8%)	91(19.4%)	
South American	64(6.4%)	34(6.8%)	29(5.9%)		37(7%)	27(5.8%)	
Mixed/Other	19(1.9%)	7(1.4%)	12(2.4%)		10(1.9%)	9(1.9%)	
Parental education				<0.01			0.73
<High School	561(56.4%)	313(63%)	244(49.7%)		296(56.3%)	265(56.6%)	
High School	175(17.6%)	76(15.3%)	98(20%)		98(18.6%)	77(16.5%)	
>High School	160(16.1%)	64(12.9%)	95(19.3%)		84(16%)	76(16.2%)	
Current Education				<0.01			0.39
<High School	356(35.8%)	214(43.1%)	141(28.7%)		195(37.1%)	161(34.4%)	
≥High School	638(64.2%)	283(56.9%)	350(71.3%)		331(62.9%)	307(65.6%)	
Household Income				0.10			<0.01
<30K	674(67.8%)	352(70.8%)	319(65%)		404(76.8%)	270(57.7%)	
≥30K	273(27.5%)	126(25.4%)	145(29.5%)		99(18.8%)	174(37.2%)	
BMI, kg/m^b^	30.1 ± 5.9	30.3 ± 6	29.9 ± 5.8	0.43	30.4 ± 5.9	29.8 ± 5.9	0.06
AHEI 2010^f^	50.2 ± 7.6	50.7 ± 7.5	49.7 ± 7.7	0.03	49.4 ± 7.4	51 ± 7.7	<0.01
Alcohol use				0.47			0.15
Never	208(20.9%)	97(19.5%)	108(22.0%)		119(22.6%)	89(19.0%)	
Former	341(34.3%)	179(36%)	161(32.8%)		186(35.4%)	155(33.1%)	
Current	445(44.8%)	221(44.5%)	222(45.2%)		221(42%)	224(47.9%)	
Cigarette use				0.27			0.01
Never	616(62.0%)	320(64.4%)	293(59.7%)		308(58.6%)	308(65.8%)	
Former	213(21.4%)	97(19.5%)	114(23.2%)		114(21.7%)	99(21.2%)	
Current	165(16.6%)	80(16.1%)	84(17.1%)		104(19.8%)	61(13.0%)	
Diabetes^g^				0.40			0.21
No	329(33.1%)	157(31.6%)	168(34.2%)		182(34.6%)	147(31.4%)	
Prediabetes	421(42.4%)	209(42.1%)	211(43%)		209(39.7%)	212(45.3%)	
Diabetes	244(24.5%)	131(26.4%)	112(22.8%)		135(25.7%)	109(23.3%)	
Dyslipidemia^h^				0.19			0.26
No	491 (49.4%)	232 (46.7%)	256 (52.1%)		248 (47.1%)	243 (51.9%)	
Without treatment	301(30.3%)	155(31.2%)	144(29.3%)		170(32.3%)	131(28.0%)	
With treatment	202(20.3%)	110(22.1%)	91(18.5%)		108(20.5%)	94(20.1%)	
Hypertension^i^	411(41.3%)	204(41.0%)	205(41.8%)	0.85	203(38.6%)	208(44.4%)	0.07
Prevalence CVD	65(6.5%)	29(5.8%)	35(7.1%)	0.44	30(5.7%)	35(7.5%)	0.30
Antibiotic use	281(28.3%)	146(29.4%)	135(27.5%)	0.53	156(29.7%)	125(26.7%)	0.32
CES-D score^j^	7.9 ± 6.5	8.5 ± 6.4	7.4 ± 6.6	<0.01	9.3 ± 6.7	6.4 ± 5.9	<0.01
STAI score^k^	17.5 ± 6	18.2 ± 6.1	16.9 ± 5.9	<0.01	18.6 ± 6.4	16.3 ± 5.3	<0.01

AHEI 2010: Alternative Healthy Eating Index 2010, BMI: body mass index, CES-D: Center for Epidemiological Studies-Depression Scale, CVD: cardiovascular disease, HCHS/SOL: Hispanic Community Health Study/Study of Latino, STAI: Spielberger State-Trait Anxiety Inventory.

^a^Childhood economic hardship was determined using the following question: Did your family ever experience a period of time when they had trouble paying for their basic needs, such as food, housing, medical care, and utilities, when you were a child?

^b^Current economic hardship was determined using the following question: In the past 12 months, was there ever a time when you had trouble paying for your basic needs, such as food, housing, medical care, and utilities?

^c^Continuous variables are presented as means (SD). Categorical variables are presented as n (%).

^d^P-value from Wilcoxon rank-sum test for continuous variables or Chi-square test for categorical variables.

^e^U.S. nativity included 50 states and District of Columbia.

^f^AHEI 2010 score (continuous (range = 0–110) with higher scores indicating better diet quality

^g^Diabetes was defined based on American Diabetes Association criteria or self-report of antidiabetic medication.

^h^Dyslipidemia was defined as LDL-c ≥160 mg/dL or HDL-c <40 mg/dL or Triglycerides ≥200 mg/dL and self-report of antihypertensive medications.

^i^Hypertension was defined as systolic or diastolic blood pressure ≥ 140/90 or self-report of antihypertensive medications.

^j^10-item CES-D (continuous (range = 0–30), with higher scores indicating worse depression symptoms.

^k^10-item STAI (continuous (range = 0–40), with higher scores indicating worse anxiety symptoms.

### Sensitivity analysis

In the analysis stratified by U.S. nativity, we did not find association between parental education and current SEP and the Shannon diversity index (Figures 1(c-f)); however, these SEP indicators were associated with differences in the overall gut microbiome composition only in foreign-born participants (Figures 1(h,i)). The associations of parental education and current SEP with species abundance appeared to be largely driven by place of birth, as the associations were mainly present among the foreign-born participants (Supplementary Tables S18, S19, S20,S21).

Our sensitivity analysis using current education as exposure showed similar results to the current SEP. Current education was not associated with the alpha diversity indexes in multivariable linear regression models, except for the observed index similar to what we found regarding current SEP. In multivariable PERMANOVA models, current education was associated with the overall gut microbiome composition (R-squared = 0.144, *p* = 0.003) and explained the variability of the gut microbiome slightly better than the current SEP (R-squared = 0.100, *p* = 0.017). The results related to the species, pathways, and enzymatic reactions related to current education were similar to current SEP (Supplementary Tables 15, 16, and 17).

## Discussion

In this study of Hispanic/Latino adults from the HCHS/SOL cohort, we observed that parental education (used as a proxy of childhood SEP) and current SEP were significantly associated with overall gut microbiome composition. Parental education explained a higher proportion of the gut microbiome variance compared to current SEP, suggesting the persistent influence of early-life SEP on later-life gut microbiome. This study expands the literature on SEP and human gut microbiome by connecting life-course SEP indicators to taxonomic features and their functions. We detected several species (19 enriched and 19 depleted), functions (3 depleted), and enzymatic reactions (3 depleted) that differed in participants with lower and higher parental education. In addition, the abundance of five species differed in participants with lower and higher current SEP. Of these five species, one of them also had lower abundance for those that reported a lower compared to a higher parental education. Participants with low parental education or lower current SEP were more likely to be older, foreign-born, have healthier diets, and present more anxiety symptoms than those with a higher SEP. These adult factors were adjusted for in the models. However, childhood factors such as diet, infections, or antibiotic use at a period of gut microbiome maturation, were not available and may explain the differences in the relative abundance of some species. When we stratified our analysis by U.S. nativity, we found that the results of the overall sample remained consistent only among the foreign-born participants. We also detected 4 species (3 enriched and 1 depleted) that differed in participants with and without childhood economic hardship.

Previous studies have assessed the relationship between SEP and human gut microbiome with mixed results. The mixed results could be explained by differences in the size and characteristics of the sample, the sequencing approaches (16S rRNA gene or shotgun metagenomic sequencing), and the models’ adjustment for potential confounders. In addition, the SEP definition in each study varies, including both individual (education, income, and occupation) and area (neighborhood SEP and social vulnerability index) level indicators.^13,19,20,36^

In line with our findings, a previous study in children did not observe an association between family SEP (using paternal and maternal education as a proxy) and alpha diversity.^36^ In contrast, two studies conducted in adults in the UK (*n* = 1,672) and U.S. (*n* = 825) observed associations of SEP (using individual and neighborhood-level indicators) with lower and greater alpha diversity, respectively.^13,19^ These aforementioned studies also found that SEP indicators were associated with gut microbiome composition. The study conducted in the U.S. observed a higher abundance of species from genus *Catenibacterium* and *Prevotella* in those with low SEP, similar to our findings.^13^ Higher abundance of species from *Catenibacterium* was previously detected in individuals with polycystic ovary syndrome and HIV infection, thus, potentially related to increased inflammatory response.^44,45^ Western lifestyle (including high-fat and low-fiber diets) have been linked to depleted abundance of species from *Prevotella* ^46^; however, the relationship of these species with health and diseases is still not clear. There was one species (Bacteroides species CAG:98) that overlap with parental education and current SEP, with statistically significant association at those two life span periods. However, there was a large proportion of species (62%) associated with current SEP that were significantly associated with childhood SEP in the same direction, but without reaching statistical significance. This consistency of findings may indicate that there may be a similar mechanism involved in the association of childhood and current SEP and the composition of the gut microbiome.

Overall, lower parental education and current SEP were associated with depleted abundance of species from genus *Bacteroides*. *Bacteroides* are gram-negative and participate in the degradation of polysaccharides to oligosaccharides and monosaccharides, forming various metabolites in the gut, including short-chain fatty acids (mainly acetate and propionate).^47,48^ In the current study, species from the genus *Bacteroides* were positively correlated with the conversion of succinate to propanoate functional pathway. This pathway was also depleted in participants with a lower parental education. Previous research has shown that the phylum *Bacteroidetes* use succinate pathway via methylmalonyl-CoA for propionate production.^49^ Previous studies suggested that propionate has potential beneficial health effects, including weight control and anti-inflammatory properties.^50,51^

*Flavonifractor plautii*, a gram-positive member of Firmicutes, was depleted in participants with a lower parental education. A recent study observed that *Flavonifractor plautii* was also depleted among individuals with elevated arterial stiffness.^52^ A previous study using HCHS/SOL data found that this species was associated with a better cardiometabolic profile, but also with a poor diet quality.^53^ Participants with lower parental education and childhood economic hardship were enriched in *Haemophilus parainfluenzae*, a pathogen that has been linked to some infections and Crohn’s disease.^54^ Participants with lower parental education had a higher abundance of species from the genus *Eubacterium* and *Roseburia* compared with those with higher parental education. These bacteria have been related to gut health and immune defense through butyrate production.^55,56^ In the present study, those with lower parental education have better diet quality compared to those with higher SEP. Even though we adjusted our models for diet quality, there could be unmeasured diet-related confounding that could explain the association between lower childhood SEP and these species.

In our study, the association of life-course SEP and gut microbiome overall composition differed by U.S. nativity. Parental education and current SEP remained associated with gut microbiome overall composition mainly among foreign-born participants. Previous findings from HCHS/SOL study have shown differences in gut microbiome regarding nativity and time living in the US.^25,57^ One of these studies showed that the Prevotella to Bacteroides ratio was lowest among those born in the US mainland and that the Prevotella to Bacteroides ratio increased monotonically with the increasing relocation age among those born in Latin America.^25^ In addition, the adoption of US dominant culture diet (dietary acculturation) by Hispanic/Latinos immigrants also increased with years of living in the US and was associated with differences in gut microbiome composition.^57^ These studies highlight the role of the environmental conditions at a time of gut microbiome maturation as well as life course influences later in life. The factors that explain the links observed between childhood and adult SEP and gut microbiome mostly among the foreign born cannot be directly addressed in this study. As the proportion of US-born individuals in the sample was relatively small, power may be one of the issues for the lack of association among US-born individuals.

The strengths of the current study include the large sample size and the inclusion of life-course SEP indicators, thus, allowing the evaluation of life-course SEP and gut microbiome in a large diverse cohort of Hispanic/Latino heritage. In addition, we collected detailed dietary data using two 24-hour dietary recalls to evaluate diet quality. However, we acknowledge that the current study has some limitations. First, the cross-sectional analyses prevented us from inferring a causal relationship between SEP and gut microbiome. Second, our childhood indicator of SEP relied only on parental educational attainment. We did not have information to fully characterize the environmental conditions, such as diet and nutritional status during early life, which may be involved in programming the gut microbiome composition. However, in a subset of the sample we examined whether childhood and current economic hardship influence the gut microbiome. Economic hardship refers to ever experiencing a period of time when they had trouble paying for basic needs, giving a glimpse of adequate living conditions. Even though we adjusted our models for several potential confounders, we cannot eliminate the possibility of residual confounding. In addition, it is important to mention that overall cohort participants are predominantly of low SEP and that we used a low threshold to classify individuals as lower or higher income ($30K), which in practice more closely classify individuals as being below or above the poverty line. Therefore, these findings may not generalize to populations with a much wider income distribution. Lastly, as the current study was performed in the Hispanic/Latino population, the findings may not be generalizable to other ethnic groups.

In summary, the current study found that among this sample of Hispanic/Latino adults, parental education (proxy of childhood SEP) and current SEP were associated with overall gut microbiome composition. Early-life SEP may have long-term effects on gut microbiome composition, underscoring another biological mechanism that may link early childhood factors to adult chronic diseases. Future studies should further test whether gut microbiome is another manifestation of the biological embedding of disparities that puts minoritized individuals at higher risk of CVD and other chronic conditions.

## Supplementary Material

supplementarymaterial.docx

## Data Availability

HCHS/SOL data are archived at the National Institutes of Health repositories dbGap and BIOLINCC. Sequence data from the samples described in this study is deposited in QIITA (study ID 11,666). HCHS/SOL has established a process for the scientific community to apply for access to participant data and materials with such requests reviewed by the project’s Steering Committee. These policies are described at https://sites.cscc.unc.edu/hchs/.
