# Supplementary material for: Life-course socioeconomic position and the gut microbiome in the Hispanic Community Health Study/Study of Latinos (HCHS/SOL)
Source: Gut Microbes. 2025 Mar 18;17(1):2479772. doi: 10.1080/19490976.2025.2479772 (PMC12834079; doi:10.1080/19490976.2025.2479772)
Supplement: supplementarymaterial.docx [file KGMI_A_2479772_SM8234.docx]

| **Supplementary Table 1.** Association of parental education (High school or more vs. less than High school) with species in overall participants (n=2174). Analysis of Compositions of Microbiomes (ANCOM2) was used to identify species related to parental education. Regression coefficients were obtained by modeling the effect of parental education on centered log ratio (CLR)-transformed species in multivariable linear regression adjusted for age, gender, BMI, field center, Hispanic/Latino background, U.S. nativity, Bristol stool type, current SEP, the alternative Healthy Eating Index 2010 score, cigarette smoking, alcohol use, depressive and anxiety symptoms, diabetes, hypertension, cardiovascular disease, dyslipidemia, and antibiotic use. | | | | | | |
| --- | --- | --- | --- | --- | --- | --- |
| **species** | **W_stat** | **ANCOM** | **beta** | **l95ci** | **u95ci** | **qval** |
| Alistipes sp. CAG:514 | 921 | 0.7 | 0.434 | 0.146 | 0.722 | 0.003 |
| Bacteroides caccae CAG:21 | 931 | 0.7 | -0.276 | -0.461 | -0.090 | 0.004 |
| Bacteroides faecis CAG:32 | 992 | 0.8 | -0.299 | -0.479 | -0.119 | 0.001 |
| Bacteroides finegoldii | 974 | 0.8 | -0.272 | -0.437 | -0.106 | 0.001 |
| Bacteroides finegoldii CAG:203 | 1028 | 0.8 | -0.276 | -0.432 | -0.121 | 0.001 |
| Bacteroides sp. 43_46 | 916 | 0.7 | -0.219 | -0.346 | -0.093 | 0.001 |
| Bacteroides sp. CAG:98 | 1163 | 0.9 | -0.492 | -0.721 | -0.264 | 0.000 |
| Blautia hydrogenotrophica CAG:147 | 1132 | 0.9 | -0.565 | -0.795 | -0.336 | 0.000 |
| Catenibacterium sp. CAG:290 | 842 | 0.7 | 0.404 | 0.098 | 0.710 | 0.010 |
| Clostridium bolteae CAG:59 | 837 | 0.7 | -0.199 | -0.329 | -0.068 | 0.003 |
| Clostridium clostridioforme CAG:132 | 1032 | 0.8 | -0.307 | -0.492 | -0.123 | 0.001 |
| Clostridium clostridioforme CAG:511 | 1034 | 0.8 | -0.322 | -0.490 | -0.154 | 0.000 |
| Clostridium sp. CAG:12237_41 | 872 | 0.7 | 0.261 | 0.103 | 0.419 | 0.001 |
| Clostridium sp. CAG:264 | 848 | 0.7 | 0.376 | 0.143 | 0.610 | 0.002 |
| Coprobacillus sp. CAG:605 | 846 | 0.7 | 0.387 | 0.134 | 0.639 | 0.003 |
| Coprococcus sp. CAG:131_42_139 | 905 | 0.7 | 0.327 | 0.101 | 0.552 | 0.005 |
| Coprococcus sp. HPP0048 | 1017 | 0.8 | -0.245 | -0.360 | -0.130 | 0.000 |
| Dielma fastidiosa | 844 | 0.7 | -0.274 | -0.447 | -0.102 | 0.002 |
| Eggerthella sp. CAG:209 | 924 | 0.7 | 0.447 | 0.137 | 0.757 | 0.005 |
| Erysipelotrichaceae bacterium 21_3 | 855 | 0.7 | -0.184 | -0.276 | -0.092 | 0.000 |
| Eubacterium sp. CAG:252 | 883 | 0.7 | 0.278 | 0.104 | 0.453 | 0.002 |
| Firmicutes bacterium CAG:65 | 960 | 0.8 | 0.309 | 0.122 | 0.496 | 0.001 |
| Firmicutes bacterium CAG:83 | 889 | 0.7 | 0.302 | 0.096 | 0.507 | 0.004 |
| Flavonifractor plautii | 861 | 0.7 | -0.214 | -0.356 | -0.071 | 0.003 |
| Haemophilus parainfluenzae | 913 | 0.7 | 0.359 | 0.073 | 0.646 | 0.014 |
| Odoribacter laneus | 826 | 0.7 | -0.205 | -0.361 | -0.049 | 0.010 |
| Prevotella sp. CAG:5226 | 916 | 0.7 | 0.414 | 0.130 | 0.699 | 0.004 |
| Roseburia faecis | 852 | 0.7 | 0.219 | 0.091 | 0.347 | 0.001 |
| Roseburia sp. CAG:309 | 969 | 0.8 | 0.411 | 0.190 | 0.633 | 0.000 |
| Roseburia sp. CAG:380 | 1039 | 0.8 | 0.393 | 0.187 | 0.598 | 0.000 |
| Ruminococcus sp. CAG:254 | 836 | 0.7 | 0.366 | 0.050 | 0.683 | 0.023 |
| Ruminococcus torques CAG:61 | 1063 | 0.9 | -0.367 | -0.578 | -0.156 | 0.001 |
| Sanguibacteroides justesenii | 856 | 0.7 | -0.238 | -0.396 | -0.081 | 0.003 |
| Sharpea azabuensis | 840 | 0.7 | 0.192 | 0.107 | 0.278 | 0.000 |
| Sutterella wadsworthensis | 891 | 0.7 | 0.401 | 0.096 | 0.706 | 0.010 |
| [Clostridium] spiroforme | 1038 | 0.8 | -0.319 | -0.493 | -0.145 | 0.000 |
| [Eubacterium] eligens | 878 | 0.7 | 0.243 | 0.093 | 0.393 | 0.002 |

| [Ruminococcus] torques | 995 | 0.8 | -0.245 | -0.369 | -0.121 | 0.000 |
| --- | --- | --- | --- | --- | --- | --- |
| Abiotrophia defectiva | 1 | Not detected | 0.009 | -0.172 | 0.191 | 0.919 |
| Acetanaerobacterium elongatum | 28 | Not detected | 0.020 | -0.106 | 0.147 | 0.752 |
| Acetitomaculum ruminis | 19 | Not detected | 0.002 | -0.071 | 0.075 | 0.948 |
| Acetivibrio cellulolyticus | 8 | Not detected | -0.003 | -0.151 | 0.145 | 0.967 |
| Acetivibrio ethanolgignens | 11 | Not detected | -0.023 | -0.085 | 0.039 | 0.460 |
| Acetobacter sp. 46_36 | 0 | Not detected | -0.133 | -0.417 | 0.150 | 0.357 |
| Acetobacter sp. CAG:267 | 0 | Not detected | -0.030 | -0.287 | 0.227 | 0.818 |
| Acholeplasma axanthum | 3 | Not detected | 0.030 | -0.097 | 0.157 | 0.644 |
| Acholeplasma equifetale | 2 | Not detected | -0.085 | -0.258 | 0.088 | 0.335 |
| Acidaminococcus fermentans | 0 | Not detected | 0.029 | -0.096 | 0.155 | 0.647 |
| Acidaminococcus intestini | 0 | Not detected | -0.024 | -0.253 | 0.205 | 0.837 |
| Acidaminococcus intestini CAG:325 | 0 | Not detected | -0.005 | -0.364 | 0.355 | 0.980 |
| Acinetobacter baumannii | 13 | Not detected | 0.121 | -0.028 | 0.270 | 0.111 |
| Acinetobacter bereziniae | 2 | Not detected | 0.025 | -0.123 | 0.173 | 0.742 |
| Acinetobacter johnsonii | 1 | Not detected | -0.011 | -0.173 | 0.151 | 0.894 |
| Acinetobacter sp. CAG:196 | 0 | Not detected | -0.021 | -0.328 | 0.287 | 0.896 |
| Acinetobacter sp. CAG:196_36_41 | 0 | Not detected | 0.000 | -0.299 | 0.298 | 0.999 |
| Acinetobacter sp. N54.MGS-139 | 0 | Not detected | -0.078 | -0.237 | 0.081 | 0.337 |
| Acinetobacter venetianus | 2 | Not detected | 0.012 | -0.169 | 0.192 | 0.900 |
| Actinobaculum sp. oral taxon 183 | 129 | Not detected | 0.194 | -0.018 | 0.405 | 0.073 |
| Actinobaculum suis | 1 | Not detected | -0.022 | -0.174 | 0.131 | 0.779 |
| Actinomyces dentalis | 2 | Not detected | 0.064 | -0.155 | 0.282 | 0.568 |
| Actinomyces gerencseriae | 2 | Not detected | -0.027 | -0.225 | 0.170 | 0.787 |
| Actinomyces graevenitzii | 0 | Not detected | 0.044 | -0.099 | 0.186 | 0.549 |
| Actinomyces hongkongensis | 0 | Not detected | -0.127 | -0.323 | 0.069 | 0.204 |
| Actinomyces israelii | 2 | Not detected | 0.042 | -0.164 | 0.247 | 0.690 |
| Actinomyces johnsonii | 6 | Not detected | 0.011 | -0.188 | 0.210 | 0.914 |
| Actinomyces massiliensis | 3 | Not detected | 0.072 | -0.130 | 0.275 | 0.484 |
| Actinomyces meyeri | 1 | Not detected | -0.102 | -0.273 | 0.069 | 0.243 |
| Actinomyces odontolyticus | 0 | Not detected | -0.022 | -0.174 | 0.130 | 0.781 |
| Actinomyces oris | 3 | Not detected | 0.001 | -0.180 | 0.182 | 0.992 |
| Actinomyces sp. HMSC065F12 | 42 | Not detected | -0.013 | -0.132 | 0.105 | 0.826 |
| Actinomyces sp. HMSC06A08 | 59 | Not detected | 0.062 | -0.058 | 0.182 | 0.314 |
| Actinomyces sp. oral taxon 181 | 0 | Not detected | -0.083 | -0.277 | 0.112 | 0.404 |
| Actinomyces sp. oral taxon 448 | 0 | Not detected | 0.044 | -0.186 | 0.274 | 0.709 |
| Actinomyces sp. oral taxon 848 | 115 | Not detected | 0.137 | -0.016 | 0.290 | 0.080 |
| Actinomyces sp. ph3 | 0 | Not detected | -0.114 | -0.302 | 0.074 | 0.236 |
| Actinomyces timonensis | 3 | Not detected | -0.077 | -0.215 | 0.061 | 0.273 |
| Actinomyces turicensis | 0 | Not detected | -0.073 | -0.272 | 0.125 | 0.470 |
| Adlercreutzia equolifaciens | 0 | Not detected | -0.077 | -0.313 | 0.159 | 0.523 |
| Aeromonas salmonicida | 1 | Not detected | -0.034 | -0.225 | 0.157 | 0.727 |
| Aggregatibacter aphrophilus | 8 | Not detected | 0.169 | -0.044 | 0.381 | 0.120 |
| Akkermansia glycaniphila | 0 | Not detected | -0.137 | -0.333 | 0.059 | 0.170 |

| Akkermansia muciniphila | 0 | Not detected | -0.171 | -0.423 | 0.081 | 0.185 |
| --- | --- | --- | --- | --- | --- | --- |
| Akkermansia muciniphila CAG:154 | 0 | Not detected | -0.099 | -0.429 | 0.232 | 0.559 |
| Akkermansia sp. 54_46 | 0 | Not detected | -0.150 | -0.494 | 0.194 | 0.393 |
| Akkermansia sp. CAG:344 | 0 | Not detected | -0.229 | -0.578 | 0.120 | 0.199 |
| Akkermansia sp. KLE1605 | 0 | Not detected | -0.225 | -0.522 | 0.072 | 0.138 |
| Akkermansia sp. KLE1797 | 0 | Not detected | -0.230 | -0.527 | 0.068 | 0.131 |
| Akkermansia sp. KLE1798 | 0 | Not detected | -0.229 | -0.526 | 0.069 | 0.132 |
| Akkermansia sp. Phil8 | 0 | Not detected | -0.147 | -0.486 | 0.193 | 0.397 |
| Akkermansia sp. UNK.MGS-1 | 0 | Not detected | -0.149 | -0.490 | 0.191 | 0.391 |
| Alistipes finegoldii | 0 | Not detected | -0.063 | -0.199 | 0.072 | 0.357 |
| Alistipes ihumii | 0 | Not detected | -0.096 | -0.328 | 0.135 | 0.415 |
| Alistipes indistinctus | 0 | Not detected | 0.179 | -0.141 | 0.499 | 0.273 |
| Alistipes inops | 0 | Not detected | -0.179 | -0.430 | 0.073 | 0.164 |
| Alistipes obesi | 0 | Not detected | -0.012 | -0.160 | 0.135 | 0.869 |
| Alistipes putredinis | 678 | Not detected | -0.238 | -0.430 | -0.046 | 0.015 |
| Alistipes senegalensis | 2 | Not detected | 0.018 | -0.103 | 0.139 | 0.771 |
| Alistipes shahii | 0 | Not detected | -0.054 | -0.188 | 0.079 | 0.426 |
| Alistipes sp. CAG:435 | 0 | Not detected | 0.213 | -0.104 | 0.530 | 0.189 |
| Alistipes sp. Zagget8 | 0 | Not detected | 0.038 | -0.158 | 0.235 | 0.702 |
| Alistipes timonensis | 0 | Not detected | 0.005 | -0.200 | 0.211 | 0.959 |
| Alkaliflexus imshenetskii | 450 | Not detected | -0.257 | -0.407 | -0.106 | 0.001 |
| Allisonella histaminiformans | 2 | Not detected | 0.312 | 0.010 | 0.613 | 0.043 |
| Allobaculum stercoricanis | 23 | Not detected | 0.040 | -0.031 | 0.112 | 0.270 |
| Allofustis seminis | 1 | Not detected | -0.041 | -0.211 | 0.129 | 0.636 |
| Alloprevotella rava | 2 | Not detected | 0.006 | -0.127 | 0.139 | 0.929 |
| Alloprevotella tannerae | 80 | Not detected | -0.093 | -0.186 | 0.000 | 0.050 |
| Alloscardovia criceti | 3 | Not detected | 0.094 | -0.073 | 0.261 | 0.272 |
| Alloscardovia omnicolens | 54 | Not detected | 0.166 | -0.012 | 0.345 | 0.068 |
| Amphibacillus xylanus | 2 | Not detected | -0.114 | -0.290 | 0.063 | 0.207 |
| Anaerobium acetethylicum | 21 | Not detected | -0.026 | -0.099 | 0.047 | 0.490 |
| Anaerococcus hydrogenalis | 58 | Not detected | -0.145 | -0.275 | -0.015 | 0.029 |
| Anaerococcus lactolyticus | 2 | Not detected | -0.119 | -0.300 | 0.062 | 0.197 |
| Anaerococcus obesiensis | 579 | Not detected | -0.200 | -0.352 | -0.047 | 0.010 |
| Anaerocolumna aminovalerica | 19 | Not detected | 0.043 | -0.110 | 0.195 | 0.581 |
| Anaerocolumna xylanovorans | 19 | Not detected | -0.060 | -0.198 | 0.079 | 0.401 |
| Anaerofustis stercorihominis | 0 | Not detected | -0.071 | -0.294 | 0.152 | 0.532 |
| Anaeroglobus geminatus | 0 | Not detected | -0.086 | -0.268 | 0.096 | 0.355 |
| Anaeromassilibacillus senegalensis | 20 | Not detected | 0.047 | -0.079 | 0.173 | 0.466 |
| Anaerophaga thermohalophila | 4 | Not detected | -0.051 | -0.167 | 0.065 | 0.391 |
| Anaerosalibacter massiliensis | 41 | Not detected | -0.194 | -0.379 | -0.010 | 0.039 |
| Anaerosporobacter mobilis | 5 | Not detected | -0.064 | -0.147 | 0.018 | 0.126 |
| Anaerosporomusa subterranea | 3 | Not detected | 0.011 | -0.117 | 0.139 | 0.866 |
| Anaerostipes caccae | 139 | Not detected | -0.075 | -0.150 | -0.001 | 0.047 |
| Anaerostipes hadrus | 0 | Not detected | -0.057 | -0.184 | 0.069 | 0.376 |

| Anaerostipes sp. CAG:276 | 0 | Not detected | -0.232 | -0.494 | 0.031 | 0.084 |
| --- | --- | --- | --- | --- | --- | --- |
| Anaerotruncus colihominis | 92 | Not detected | -0.074 | -0.156 | 0.008 | 0.076 |
| Anaerotruncus sp. CAG:390 | 2 | Not detected | 0.156 | -0.075 | 0.387 | 0.186 |
| Anaerotruncus sp. CAG:528 | 547 | Not detected | 0.245 | 0.056 | 0.434 | 0.011 |
| Anaerotruncus sp. G3(2012) | 20 | Not detected | 0.003 | -0.059 | 0.065 | 0.927 |
| Anaerovorax odorimutans | 25 | Not detected | 0.084 | -0.061 | 0.229 | 0.257 |
| Angelakisella massiliensis | 5 | Not detected | -0.011 | -0.080 | 0.059 | 0.766 |
| Aphanizomenon flos-aquae | 87 | Not detected | 0.137 | 0.007 | 0.267 | 0.039 |
| Aquabacterium parvum | 3 | Not detected | 0.008 | -0.148 | 0.165 | 0.918 |
| Arcanobacterium haemolyticum | 13 | Not detected | 0.015 | -0.143 | 0.173 | 0.854 |
| Asaccharospora irregularis | 5 | Not detected | 0.108 | -0.070 | 0.285 | 0.235 |
| Atlantibacter hermannii | 0 | Not detected | 0.042 | -0.185 | 0.268 | 0.720 |
| Atopobium deltae | 4 | Not detected | -0.085 | -0.256 | 0.087 | 0.332 |
| Atopobium minutum | 3 | Not detected | -0.137 | -0.310 | 0.036 | 0.121 |
| Atopobium parvulum | 0 | Not detected | -0.063 | -0.256 | 0.130 | 0.520 |
| Atopobium rimae | 3 | Not detected | 0.040 | -0.118 | 0.199 | 0.618 |
| Atopobium sp. BS2 | 0 | Not detected | -0.066 | -0.254 | 0.122 | 0.489 |
| Atopobium sp. HMSC064B08 | 4 | Not detected | -0.035 | -0.171 | 0.102 | 0.618 |
| Atopobium sp. ICM42b | 0 | Not detected | -0.074 | -0.264 | 0.116 | 0.443 |
| Atopobium vaginae | 2 | Not detected | 0.054 | -0.125 | 0.232 | 0.556 |
| Atopostipes suicloacalis | 9 | Not detected | 0.083 | -0.083 | 0.250 | 0.327 |
| Avibacterium paragallinarum | 8 | Not detected | 0.099 | -0.054 | 0.251 | 0.204 |
| Azospirillum sp. 47_25 | 0 | Not detected | -0.168 | -0.496 | 0.161 | 0.317 |
| Azospirillum sp. CAG:260 | 0 | Not detected | -0.194 | -0.516 | 0.129 | 0.239 |
| Bacillus acidiproducens | 256 | Not detected | -0.193 | -0.381 | -0.005 | 0.044 |
| Bacillus andreraoultii | 571 | Not detected | -0.228 | -0.415 | -0.042 | 0.017 |
| Bacillus azotoformans | 120 | Not detected | -0.205 | -0.375 | -0.036 | 0.018 |
| Bacillus bataviensis | 152 | Not detected | -0.205 | -0.369 | -0.042 | 0.014 |
| Bacillus coahuilensis | 0 | Not detected | -0.012 | -0.201 | 0.177 | 0.900 |
| Bacillus rubiinfantis | 22 | Not detected | -0.110 | -0.276 | 0.057 | 0.198 |
| Bacillus sp. CAG:988 | 0 | Not detected | -0.022 | -0.181 | 0.136 | 0.783 |
| Bacillus thermotolerans | 338 | Not detected | -0.202 | -0.383 | -0.021 | 0.029 |
| Bacteroidales bacterium 45-6 | 41 | Not detected | 0.094 | -0.084 | 0.272 | 0.300 |
| Bacteroidales bacterium 55_9 | 4 | Not detected | -0.060 | -0.150 | 0.030 | 0.192 |
| Bacteroidales bacterium Bact_08 | 2 | Not detected | -0.001 | -0.147 | 0.144 | 0.987 |
| Bacteroidales bacterium Bact_18 | 7 | Not detected | 0.031 | -0.115 | 0.176 | 0.680 |
| Bacteroidales bacterium Bact_23 | 1 | Not detected | -0.091 | -0.279 | 0.097 | 0.342 |
| Bacteroidales bacterium CF | 1 | Not detected | -0.041 | -0.199 | 0.117 | 0.610 |
| Bacteroidales bacterium H10 | 0 | Not detected | -0.109 | -0.247 | 0.028 | 0.119 |
| Bacteroidales bacterium K1 | 1 | Not detected | 0.001 | -0.160 | 0.162 | 0.991 |
| Bacteroidales bacterium K10 | 175 | Not detected | -0.169 | -0.326 | -0.013 | 0.034 |
| Bacteroidales bacterium KHT7 | 1 | Not detected | 0.028 | -0.155 | 0.212 | 0.763 |
| Bacteroides barnesiae | 9 | Not detected | -0.073 | -0.162 | 0.015 | 0.105 |
| Bacteroides cellulosilyticus | 0 | Not detected | -0.072 | -0.233 | 0.089 | 0.381 |

| Bacteroides coprocola | 680 | Not detected | -0.175 | -0.299 | -0.051 | 0.006 |
| --- | --- | --- | --- | --- | --- | --- |
| Bacteroides coprophilus | 0 | Not detected | -0.052 | -0.139 | 0.036 | 0.245 |
| Bacteroides coprosuis | 0 | Not detected | 0.030 | -0.107 | 0.167 | 0.670 |
| Bacteroides dorei CAG:222 | 0 | Not detected | -0.148 | -0.294 | -0.003 | 0.046 |
| Bacteroides faecichinchillae | 788 | 0.6 | -0.162 | -0.250 | -0.073 | 0.000 |
| Bacteroides fluxus | 757 | 0.6 | -0.158 | -0.252 | -0.064 | 0.001 |
| Bacteroides fragilis | 744 | 0.6 | -0.185 | -0.311 | -0.059 | 0.004 |
| Bacteroides fragilis CAG:47 | 0 | Not detected | -0.200 | -0.403 | 0.004 | 0.055 |
| Bacteroides graminisolvens | 2 | Not detected | -0.126 | -0.324 | 0.072 | 0.212 |
| Bacteroides neonati | 42 | Not detected | -0.073 | -0.145 | -0.002 | 0.045 |
| Bacteroides oleiciplenus | 665 | Not detected | -0.158 | -0.264 | -0.051 | 0.004 |
| Bacteroides ovatus | 57 | Not detected | -0.141 | -0.263 | -0.018 | 0.025 |
| Bacteroides ovatus CAG:22 | 44 | Not detected | -0.153 | -0.287 | -0.019 | 0.026 |
| Bacteroides paurosaccharolyticus | 765 | 0.6 | -0.201 | -0.327 | -0.075 | 0.002 |
| Bacteroides pectinophilus CAG:437 | 98 | Not detected | 0.100 | -0.018 | 0.219 | 0.098 |
| Bacteroides plebeius | 412 | Not detected | -0.162 | -0.292 | -0.033 | 0.014 |
| Bacteroides propionicifaciens | 2 | Not detected | -0.058 | -0.227 | 0.112 | 0.505 |
| Bacteroides pyogenes | 360 | Not detected | -0.138 | -0.242 | -0.035 | 0.009 |
| Bacteroides reticulotermitis | 713 | 0.6 | -0.172 | -0.283 | -0.061 | 0.002 |
| Bacteroides salanitronis | 0 | Not detected | -0.052 | -0.141 | 0.038 | 0.260 |
| Bacteroides salyersiae | 0 | Not detected | -0.098 | -0.222 | 0.026 | 0.123 |
| Bacteroides sp. 41_26 | 27 | Not detected | -0.160 | -0.306 | -0.013 | 0.033 |
| Bacteroides sp. CAG:1060 | 0 | Not detected | -0.006 | -0.237 | 0.226 | 0.961 |
| Bacteroides sp. CAG:1076 | 1 | Not detected | -0.138 | -0.287 | 0.011 | 0.069 |
| Bacteroides sp. CAG:530 | 2 | Not detected | 0.125 | -0.010 | 0.261 | 0.070 |
| Bacteroides sp. CAG:545 | 2 | Not detected | 0.319 | 0.023 | 0.614 | 0.035 |
| Bacteroides sp. CAG:714 | 0 | Not detected | -0.004 | -0.215 | 0.207 | 0.968 |
| Bacteroides sp. CAG:770 | 0 | Not detected | 0.049 | -0.112 | 0.210 | 0.553 |
| Bacteroides sp. D2 | 31 | Not detected | -0.159 | -0.300 | -0.019 | 0.026 |
| Bacteroides sp. Ga6A1 | 0 | Not detected | 0.067 | -0.140 | 0.274 | 0.525 |
| Bacteroides sp. Ga6A2 | 0 | Not detected | -0.025 | -0.231 | 0.181 | 0.811 |
| Bacteroides stercoris | 0 | Not detected | -0.135 | -0.297 | 0.027 | 0.103 |
| Bacteroides thetaiotaomicron | 0 | Not detected | -0.032 | -0.124 | 0.060 | 0.492 |
| Bacteroides thetaiotaomicron CAG:40 | 0 | Not detected | -0.044 | -0.189 | 0.101 | 0.549 |
| Bacteroides uniformis | 0 | Not detected | -0.084 | -0.215 | 0.046 | 0.206 |
| Bacteroides vulgatus | 0 | Not detected | -0.118 | -0.241 | 0.004 | 0.058 |
| Bacteroides vulgatus CAG:6 | 0 | Not detected | -0.132 | -0.278 | 0.014 | 0.078 |
| Bacteroidetes bacterium OLB8 | 2 | Not detected | -0.010 | -0.182 | 0.163 | 0.912 |
| Bacteroidetes oral taxon 274 | 22 | Not detected | -0.121 | -0.244 | 0.002 | 0.054 |
| Bariatricus massiliensis | 4 | Not detected | 0.000 | -0.066 | 0.066 | 0.999 |
| Barnesiella intestinihominis | 0 | Not detected | -0.098 | -0.259 | 0.063 | 0.234 |
| Barnesiella viscericola | 167 | Not detected | -0.093 | -0.168 | -0.018 | 0.015 |
| Bavariicoccus seileri | 0 | Not detected | -0.152 | -0.307 | 0.003 | 0.055 |
| Beduini massiliensis | 4 | Not detected | -0.020 | -0.120 | 0.080 | 0.696 |

| Bibersteinia trehalosi | 2 | Not detected | 0.016 | -0.168 | 0.200 | 0.867 |
| --- | --- | --- | --- | --- | --- | --- |
| Bifidobacterium actinocoloniiforme | 2 | Not detected | 0.001 | -0.176 | 0.178 | 0.992 |
| Bifidobacterium adolescentis | 0 | Not detected | 0.203 | -0.070 | 0.475 | 0.145 |
| Bifidobacterium aesculapii | 9 | Not detected | 0.092 | -0.052 | 0.236 | 0.211 |
| Bifidobacterium angulatum | 9 | Not detected | 0.211 | -0.015 | 0.436 | 0.067 |
| Bifidobacterium animalis | 0 | Not detected | 0.011 | -0.182 | 0.205 | 0.907 |
| Bifidobacterium asteroides | 2 | Not detected | 0.025 | -0.161 | 0.210 | 0.796 |
| Bifidobacterium biavatii | 9 | Not detected | 0.027 | -0.045 | 0.099 | 0.459 |
| Bifidobacterium bifidum | 0 | Not detected | 0.048 | -0.210 | 0.305 | 0.717 |
| Bifidobacterium bohemicum | 20 | Not detected | 0.111 | -0.057 | 0.280 | 0.195 |
| Bifidobacterium bombi | 3 | Not detected | -0.017 | -0.184 | 0.150 | 0.843 |
| Bifidobacterium breve | 0 | Not detected | 0.130 | -0.123 | 0.382 | 0.314 |
| Bifidobacterium callitrichos | 4 | Not detected | 0.178 | -0.046 | 0.402 | 0.120 |
| Bifidobacterium choerinum | 15 | Not detected | 0.109 | -0.087 | 0.306 | 0.274 |
| Bifidobacterium coryneforme | 2 | Not detected | 0.005 | -0.173 | 0.183 | 0.957 |
| Bifidobacterium crudilactis | 3 | Not detected | 0.037 | -0.112 | 0.185 | 0.628 |
| Bifidobacterium cuniculi | 2 | Not detected | 0.032 | -0.158 | 0.221 | 0.743 |
| Bifidobacterium dentium | 0 | Not detected | 0.074 | -0.169 | 0.318 | 0.549 |
| Bifidobacterium gallicum | 1 | Not detected | -0.014 | -0.206 | 0.177 | 0.884 |
| Bifidobacterium gallinarum | 0 | Not detected | 0.082 | -0.146 | 0.310 | 0.482 |
| Bifidobacterium longum | 0 | Not detected | 0.110 | -0.141 | 0.361 | 0.390 |
| Bifidobacterium magnum | 72 | Not detected | 0.081 | -0.039 | 0.200 | 0.184 |
| Bifidobacterium minimum | 1 | Not detected | 0.060 | -0.175 | 0.294 | 0.618 |
| Bifidobacterium mongoliense | 3 | Not detected | 0.073 | -0.107 | 0.254 | 0.427 |
| Bifidobacterium pseudolongum | 1 | Not detected | -0.028 | -0.231 | 0.176 | 0.790 |
| Bifidobacterium psychraerophilum | 3 | Not detected | 0.009 | -0.128 | 0.145 | 0.902 |
| Bifidobacterium reuteri | 2 | Not detected | 0.102 | -0.080 | 0.283 | 0.273 |
| Bifidobacterium saguini | 2 | Not detected | 0.116 | -0.128 | 0.360 | 0.351 |
| Bifidobacterium scardovii | 0 | Not detected | 0.125 | -0.116 | 0.367 | 0.308 |
| Bifidobacterium stellenboschense | 2 | Not detected | 0.009 | -0.071 | 0.089 | 0.822 |
| Bifidobacterium subtile | 2 | Not detected | 0.032 | -0.182 | 0.246 | 0.770 |
| Bifidobacterium thermophilum | 38 | Not detected | 0.074 | -0.043 | 0.191 | 0.217 |
| Bifidobacterium tsurumiense | 4 | Not detected | 0.099 | -0.093 | 0.291 | 0.313 |
| Bilophila wadsworthia | 0 | Not detected | 0.075 | -0.216 | 0.366 | 0.615 |
| Bittarella massiliensis | 40 | Not detected | -0.043 | -0.112 | 0.027 | 0.227 |
| Blautia hansenii | 118 | Not detected | -0.088 | -0.169 | -0.006 | 0.035 |
| Blautia hydrogenotrophica | 729 | 0.6 | -0.139 | -0.212 | -0.065 | 0.000 |
| Blautia obeum | 5 | Not detected | -0.025 | -0.100 | 0.051 | 0.520 |
| Blautia producta | 2 | Not detected | -0.095 | -0.198 | 0.008 | 0.071 |
| Blautia schinkii | 41 | Not detected | -0.022 | -0.078 | 0.033 | 0.435 |
| Blautia sp. CAG:237 | 2 | Not detected | 0.099 | -0.061 | 0.258 | 0.226 |
| Blautia sp. CAG:37 | 2 | Not detected | 0.075 | -0.074 | 0.224 | 0.324 |
| Blautia sp. CAG:37_48_57 | 3 | Not detected | 0.052 | -0.063 | 0.167 | 0.377 |
| Blautia sp. KLE 1732 | 1 | Not detected | -0.013 | -0.106 | 0.081 | 0.791 |

| Brenneria goodwinii | 3 | Not detected | 0.007 | -0.138 | 0.152 | 0.924 |
| --- | --- | --- | --- | --- | --- | --- |
| Bulleidia extructa | 2 | Not detected | 0.050 | -0.132 | 0.232 | 0.589 |
| Burkholderiales bacterium GJ-E10 | 73 | Not detected | 0.207 | 0.038 | 0.375 | 0.016 |
| Buttiauxella ferragutiae | 3 | Not detected | 0.009 | -0.154 | 0.172 | 0.914 |
| Butyricicoccus desmolans | 16 | Not detected | -0.021 | -0.082 | 0.040 | 0.493 |
| Butyricicoccus pullicaecorum | 17 | Not detected | -0.051 | -0.129 | 0.028 | 0.206 |
| Butyricicoccus sp. K4410.MGS-46 | 6 | Not detected | 0.062 | -0.042 | 0.166 | 0.242 |
| Butyricimonas sp. Marseille-P2440 | 11 | Not detected | -0.166 | -0.319 | -0.014 | 0.033 |
| Butyricimonas synergistica | 0 | Not detected | -0.102 | -0.289 | 0.084 | 0.283 |
| Butyricimonas virosa | 0 | Not detected | -0.052 | -0.207 | 0.104 | 0.515 |
| Butyrivibrio crossotus | 558 | Not detected | 0.181 | 0.053 | 0.309 | 0.006 |
| Butyrivibrio crossotus CAG:259 | 2 | Not detected | 0.194 | 0.000 | 0.388 | 0.050 |
| Butyrivibrio fibrisolvens | 64 | Not detected | 0.108 | -0.051 | 0.267 | 0.183 |
| Butyrivibrio hungatei | 145 | Not detected | 0.114 | -0.016 | 0.244 | 0.085 |
| Butyrivibrio proteoclasticus | 25 | Not detected | 0.004 | -0.085 | 0.092 | 0.932 |
| Butyrivibrio sp. AD3002 | 5 | Not detected | -0.031 | -0.141 | 0.079 | 0.585 |
| Butyrivibrio sp. AE2005 | 41 | Not detected | 0.031 | -0.090 | 0.152 | 0.619 |
| Butyrivibrio sp. AE3003 | 44 | Not detected | -0.003 | -0.091 | 0.085 | 0.948 |
| Butyrivibrio sp. AE3004 | 33 | Not detected | -0.019 | -0.104 | 0.065 | 0.656 |
| Butyrivibrio sp. AE3009 | 46 | Not detected | 0.107 | -0.034 | 0.247 | 0.138 |
| Butyrivibrio sp. FCS006 | 96 | Not detected | 0.131 | -0.012 | 0.275 | 0.074 |
| Butyrivibrio sp. INlla14 | 9 | Not detected | 0.026 | -0.090 | 0.142 | 0.663 |
| Butyrivibrio sp. INlla16 | 45 | Not detected | 0.048 | -0.077 | 0.172 | 0.452 |
| Butyrivibrio sp. INlla21 | 40 | Not detected | 0.073 | -0.051 | 0.196 | 0.251 |
| Butyrivibrio sp. LB2008 | 64 | Not detected | 0.106 | -0.035 | 0.247 | 0.141 |
| Butyrivibrio sp. M55 | 45 | Not detected | 0.046 | -0.086 | 0.178 | 0.495 |
| Butyrivibrio sp. NC2002 | 5 | Not detected | -0.028 | -0.168 | 0.112 | 0.693 |
| Butyrivibrio sp. NC2007 | 20 | Not detected | 0.010 | -0.118 | 0.138 | 0.879 |
| Butyrivibrio sp. NC3005 | 489 | Not detected | 0.304 | 0.122 | 0.487 | 0.001 |
| Butyrivibrio sp. VCD2006 | 110 | Not detected | 0.076 | -0.055 | 0.207 | 0.256 |
| Butyrivibrio sp. WCD2001 | 76 | Not detected | 0.047 | -0.084 | 0.177 | 0.482 |
| Butyrivibrio sp. WCD3002 | 27 | Not detected | -0.009 | -0.132 | 0.115 | 0.888 |
| Butyrivibrio sp. ob235 | 44 | Not detected | 0.060 | -0.094 | 0.214 | 0.444 |
| Campylobacter coli | 4 | Not detected | -0.054 | -0.154 | 0.047 | 0.296 |
| Campylobacter hominis | 637 | Not detected | -0.266 | -0.465 | -0.067 | 0.009 |
| Candidatus Azobacteroides pseudotricho | 4 | Not detected | 0.004 | -0.125 | 0.134 | 0.949 |
| Candidatus Gastranaerophilales bacterium | 4 | Not detected | -0.132 | -0.303 | 0.038 | 0.129 |
| Candidatus Melainabacteria bacterium LE | 2 | Not detected | 0.004 | -0.116 | 0.125 | 0.946 |
| Candidatus Melainabacteria bacterium M | 2 | Not detected | 0.191 | -0.054 | 0.436 | 0.126 |
| Candidatus Saccharibacteria oral taxon T | 6 | Not detected | 0.058 | -0.091 | 0.208 | 0.445 |
| Candidatus Soleaferrea massiliensis | 9 | Not detected | -0.017 | -0.123 | 0.089 | 0.750 |
| Candidatus Stoquefichus sp. SB1 | 3 | Not detected | -0.033 | -0.133 | 0.067 | 0.519 |
| Capnocytophaga granulosa | 2 | Not detected | -0.005 | -0.162 | 0.151 | 0.946 |
| Capnocytophaga sp. oral taxon 329 | 3 | Not detected | 0.077 | -0.143 | 0.297 | 0.494 |

| Capnocytophaga sp. oral taxon 863 | 381 | Not detected | -0.299 | -0.489 | -0.109 | 0.002 |
| --- | --- | --- | --- | --- | --- | --- |
| Capnocytophaga sputigena | 5 | Not detected | -0.035 | -0.159 | 0.089 | 0.577 |
| Carnobacterium gallinarum | 16 | Not detected | -0.218 | -0.401 | -0.035 | 0.020 |
| Catabacter hongkongensis | 11 | Not detected | -0.072 | -0.217 | 0.072 | 0.327 |
| Catenibacterium mitsuokai | 553 | Not detected | 0.319 | 0.071 | 0.568 | 0.012 |
| Catonella morbi | 4 | Not detected | -0.015 | -0.100 | 0.070 | 0.732 |
| Cedecea neteri | 3 | Not detected | 0.119 | -0.083 | 0.322 | 0.248 |
| Cellulomonas carbonis | 310 | Not detected | 0.226 | 0.049 | 0.404 | 0.013 |
| Cellulomonas flavigena | 0 | Not detected | -0.120 | -0.316 | 0.076 | 0.230 |
| Cellulosilyticum lentocellum | 81 | Not detected | -0.145 | -0.286 | -0.004 | 0.044 |
| Cellulosilyticum ruminicola | 21 | Not detected | 0.022 | -0.128 | 0.173 | 0.772 |
| Chlamydia psittaci | 3 | Not detected | 0.009 | -0.153 | 0.171 | 0.917 |
| Christensenella sp. AF73-05CM02 | 0 | Not detected | -0.069 | -0.259 | 0.121 | 0.479 |
| Christensenellaceae bacterium Phil1 | 0 | Not detected | 0.049 | -0.082 | 0.181 | 0.460 |
| Christensenellaceae bacterium Phil10 | 2 | Not detected | 0.117 | -0.077 | 0.311 | 0.238 |
| Christensenellaceae bacterium Phil7 | 2 | Not detected | 0.169 | -0.032 | 0.369 | 0.100 |
| Chryseobacterium gallinarum | 0 | Not detected | -0.052 | -0.282 | 0.178 | 0.657 |
| Chryseobacterium greenlandense | 0 | Not detected | -0.141 | -0.356 | 0.074 | 0.199 |
| Citrobacter amalonaticus | 0 | Not detected | 0.019 | -0.239 | 0.277 | 0.888 |
| Citrobacter freundii | 0 | Not detected | 0.008 | -0.258 | 0.273 | 0.955 |
| Cloacibacillus evryensis | 0 | Not detected | -0.092 | -0.341 | 0.157 | 0.470 |
| Cloacibacillus porcorum | 0 | Not detected | -0.136 | -0.347 | 0.074 | 0.203 |
| Cloacibacterium normanense | 0 | Not detected | -0.220 | -0.427 | -0.012 | 0.038 |
| Clostridia bacterium UC5.1-1C12 | 796 | 0.6 | -0.375 | -0.609 | -0.141 | 0.002 |
| Clostridia bacterium UC5.1-1D1 | 61 | Not detected | -0.068 | -0.142 | 0.005 | 0.069 |
| Clostridia bacterium UC5.1-1E11 | 10 | Not detected | 0.018 | -0.053 | 0.089 | 0.619 |
| Clostridiales bacterium 1_7_47FAA | 781 | 0.6 | -0.146 | -0.227 | -0.065 | 0.000 |
| Clostridiales bacterium 59_14 | 374 | Not detected | 0.159 | 0.038 | 0.280 | 0.010 |
| Clostridiales bacterium DTU035 | 31 | Not detected | 0.085 | -0.069 | 0.238 | 0.279 |
| Clostridiales bacterium DTU036 | 9 | Not detected | 0.060 | -0.082 | 0.203 | 0.406 |
| Clostridiales bacterium DTU089 | 2 | Not detected | 0.043 | -0.086 | 0.172 | 0.513 |
| Clostridiales bacterium Firm_03 | 0 | Not detected | -0.024 | -0.200 | 0.152 | 0.786 |
| Clostridiales bacterium Firm_16 | 0 | Not detected | -0.011 | -0.210 | 0.187 | 0.911 |
| Clostridiales bacterium NK3B98 | 2 | Not detected | 0.020 | -0.143 | 0.183 | 0.811 |
| Clostridiales bacterium S5-A14a | 25 | Not detected | 0.023 | -0.048 | 0.095 | 0.524 |
| Clostridiales bacterium SIT11 | 3 | Not detected | -0.087 | -0.251 | 0.077 | 0.300 |
| Clostridiales bacterium VE202-08 | 4 | Not detected | -0.027 | -0.109 | 0.056 | 0.526 |
| Clostridioides difficile | 747 | 0.6 | -0.139 | -0.208 | -0.070 | 0.000 |
| Clostridioides mangenotii | 3 | Not detected | -0.003 | -0.161 | 0.154 | 0.966 |
| Clostridium baratii | 0 | Not detected | -0.020 | -0.205 | 0.165 | 0.832 |
| Clostridium beijerinckii | 4 | Not detected | 0.102 | -0.067 | 0.271 | 0.238 |
| Clostridium botulinum | 4 | Not detected | 0.095 | -0.086 | 0.277 | 0.304 |
| Clostridium butyricum | 3 | Not detected | 0.053 | -0.129 | 0.236 | 0.568 |
| Clostridium cadaveris | 42 | Not detected | 0.067 | -0.086 | 0.221 | 0.391 |

| Clostridium celatum | 0 | Not detected | 0.099 | -0.168 | 0.366 | 0.468 |
| --- | --- | --- | --- | --- | --- | --- |
| Clostridium cellulovorans | 159 | Not detected | 0.073 | -0.034 | 0.179 | 0.182 |
| Clostridium colicanis | 4 | Not detected | 0.050 | -0.091 | 0.192 | 0.488 |
| Clostridium disporicum | 0 | Not detected | -0.019 | -0.150 | 0.113 | 0.779 |
| Clostridium ihumii | 2 | Not detected | 0.005 | -0.152 | 0.161 | 0.955 |
| Clostridium intestinale | 3 | Not detected | 0.010 | -0.118 | 0.139 | 0.875 |
| Clostridium kluyveri | 210 | Not detected | -0.169 | -0.348 | 0.010 | 0.064 |
| Clostridium leptum CAG:27 | 0 | Not detected | -0.076 | -0.281 | 0.130 | 0.470 |
| Clostridium lundense | 86 | Not detected | -0.195 | -0.372 | -0.017 | 0.032 |
| Clostridium neonatale | 14 | Not detected | 0.112 | -0.042 | 0.265 | 0.155 |
| Clostridium nexile CAG:348 | 0 | Not detected | -0.097 | -0.229 | 0.034 | 0.147 |
| Clostridium paraputrificum | 3 | Not detected | 0.081 | -0.139 | 0.300 | 0.471 |
| Clostridium perfringens | 0 | Not detected | 0.125 | -0.144 | 0.395 | 0.361 |
| Clostridium phoceensis | 0 | Not detected | -0.039 | -0.144 | 0.067 | 0.470 |
| Clostridium polynesiense | 1 | Not detected | -0.035 | -0.210 | 0.140 | 0.695 |
| Clostridium saccharobutylicum | 3 | Not detected | 0.045 | -0.113 | 0.202 | 0.577 |
| Clostridium saccharoperbutylacetonicum | 3 | Not detected | 0.031 | -0.122 | 0.184 | 0.690 |
| Clostridium sartagoforme | 2 | Not detected | 0.024 | -0.164 | 0.213 | 0.800 |
| Clostridium sp. 44_14 | 163 | Not detected | 0.158 | -0.003 | 0.319 | 0.055 |
| Clostridium sp. ASF356 | 3 | Not detected | -0.105 | -0.288 | 0.077 | 0.258 |
| Clostridium sp. Ade.TY | 2 | Not detected | 0.013 | -0.148 | 0.174 | 0.876 |
| Clostridium sp. CAG:1193 | 2 | Not detected | 0.104 | -0.091 | 0.300 | 0.297 |
| Clostridium sp. CAG:122 | 735 | 0.6 | 0.306 | 0.099 | 0.514 | 0.004 |
| Clostridium sp. CAG:127 | 2 | Not detected | 0.266 | 0.033 | 0.500 | 0.026 |
| Clostridium sp. CAG:138 | 0 | Not detected | 0.182 | -0.133 | 0.497 | 0.258 |
| Clostridium sp. CAG:167 | 251 | Not detected | 0.403 | 0.129 | 0.677 | 0.004 |
| Clostridium sp. CAG:226 | 0 | Not detected | 0.030 | -0.208 | 0.268 | 0.806 |
| Clostridium sp. CAG:230 | 187 | Not detected | 0.205 | 0.038 | 0.372 | 0.016 |
| Clostridium sp. CAG:242 | 0 | Not detected | -0.091 | -0.332 | 0.149 | 0.457 |
| Clostridium sp. CAG:245 | 535 | Not detected | 0.386 | 0.028 | 0.743 | 0.035 |
| Clostridium sp. CAG:245_30_32 | 5 | Not detected | 0.116 | -0.032 | 0.265 | 0.126 |
| Clostridium sp. CAG:277 | 332 | Not detected | 0.198 | 0.035 | 0.361 | 0.017 |
| Clostridium sp. CAG:288 | 2 | Not detected | 0.224 | -0.019 | 0.466 | 0.071 |
| Clostridium sp. CAG:299 | 0 | Not detected | -0.095 | -0.306 | 0.115 | 0.375 |
| Clostridium sp. CAG:307_30_263 | 6 | Not detected | 0.123 | -0.015 | 0.262 | 0.080 |
| Clostridium sp. CAG:349 | 0 | Not detected | -0.039 | -0.360 | 0.282 | 0.813 |
| Clostridium sp. CAG:352 | 2 | Not detected | 0.075 | -0.128 | 0.278 | 0.470 |
| Clostridium sp. CAG:413 | 2 | Not detected | 0.258 | 0.017 | 0.499 | 0.036 |
| Clostridium sp. CAG:448 | 2 | Not detected | 0.163 | -0.097 | 0.423 | 0.220 |
| Clostridium sp. CAG:451 | 0 | Not detected | 0.082 | -0.187 | 0.351 | 0.550 |
| Clostridium sp. CAG:465 | 0 | Not detected | 0.108 | -0.145 | 0.361 | 0.403 |
| Clostridium sp. CAG:505 | 0 | Not detected | -0.127 | -0.374 | 0.121 | 0.315 |
| Clostridium sp. CAG:568 | 2 | Not detected | 0.200 | -0.059 | 0.459 | 0.130 |
| Clostridium sp. CAG:58 | 9 | Not detected | -0.168 | -0.342 | 0.007 | 0.060 |

| Clostridium sp. CAG:590 | 128 | Not detected | 0.168 | 0.039 | 0.297 | 0.011 |
| --- | --- | --- | --- | --- | --- | --- |
| Clostridium sp. CAG:594 | 16 | Not detected | 0.325 | 0.039 | 0.612 | 0.026 |
| Clostridium sp. CAG:628 | 775 | 0.6 | 0.434 | 0.163 | 0.704 | 0.002 |
| Clostridium sp. CAG:62_40_43 | 605 | Not detected | 0.354 | 0.072 | 0.636 | 0.014 |
| Clostridium sp. CAG:632 | 595 | Not detected | 0.179 | 0.012 | 0.347 | 0.036 |
| Clostridium sp. CAG:678 | 1 | Not detected | 0.015 | -0.182 | 0.212 | 0.881 |
| Clostridium sp. CAG:762 | 26 | Not detected | 0.228 | 0.002 | 0.454 | 0.048 |
| Clostridium sp. CAG:768 | 66 | Not detected | 0.197 | 0.046 | 0.349 | 0.011 |
| Clostridium sp. CAG:780 | 0 | Not detected | 0.085 | -0.202 | 0.372 | 0.562 |
| Clostridium sp. CAG:793 | 2 | Not detected | 0.152 | -0.080 | 0.384 | 0.199 |
| Clostridium sp. CAG:81 | 33 | Not detected | 0.118 | -0.008 | 0.244 | 0.067 |
| Clostridium sp. CAG:91 | 239 | Not detected | 0.251 | 0.045 | 0.456 | 0.017 |
| Clostridium sp. CAG:964 | 0 | Not detected | -0.022 | -0.148 | 0.104 | 0.731 |
| Clostridium sp. CL-6 | 95 | Not detected | 0.093 | -0.043 | 0.229 | 0.180 |
| Clostridium sp. HGF2 | 291 | Not detected | -0.089 | -0.162 | -0.017 | 0.015 |
| Clostridium sp. K4410.MGS-306 | 0 | Not detected | -0.055 | -0.149 | 0.038 | 0.246 |
| Clostridium sp. KLE 1755 | 148 | Not detected | -0.119 | -0.231 | -0.006 | 0.038 |
| Clostridium sp. KNHs205 | 45 | Not detected | 0.017 | -0.044 | 0.078 | 0.579 |
| Clostridium sp. KNHs209 | 61 | Not detected | 0.045 | -0.015 | 0.106 | 0.143 |
| Clostridium sp. KNHs214 | 2 | Not detected | 0.004 | -0.100 | 0.109 | 0.936 |
| Clostridium sp. L2-50 | 83 | Not detected | 0.094 | 0.002 | 0.186 | 0.046 |
| Clostridium sp. LF2 | 3 | Not detected | 0.012 | -0.113 | 0.136 | 0.856 |
| Clostridium sp. M62/1 | 23 | Not detected | 0.004 | -0.056 | 0.064 | 0.896 |
| Clostridium sp. ND2 | 2 | Not detected | 0.008 | -0.144 | 0.160 | 0.918 |
| Clostridium sp. SY8519 | 29 | Not detected | 0.039 | -0.028 | 0.106 | 0.252 |
| Clostridium sulfidigenes | 5 | Not detected | -0.047 | -0.170 | 0.077 | 0.459 |
| Collinsella aerofaciens | 0 | Not detected | 0.082 | -0.102 | 0.267 | 0.381 |
| Collinsella intestinalis | 2 | Not detected | 0.125 | -0.085 | 0.335 | 0.245 |
| Collinsella sp. CAG:166 | 0 | Not detected | 0.087 | -0.120 | 0.294 | 0.410 |
| Collinsella sp. MS5 | 2 | Not detected | 0.096 | -0.045 | 0.236 | 0.182 |
| Collinsella stercoris | 2 | Not detected | 0.090 | -0.098 | 0.277 | 0.347 |
| Collinsella tanakaei | 0 | Not detected | -0.039 | -0.174 | 0.097 | 0.576 |
| Comamonas kerstersii | 2 | Not detected | 0.103 | -0.113 | 0.320 | 0.349 |
| Coprobacillus sp. D6 | 9 | Not detected | -0.059 | -0.150 | 0.031 | 0.200 |
| Coprobacter secundus | 2 | Not detected | -0.028 | -0.110 | 0.054 | 0.505 |
| Coprococcus comes | 6 | Not detected | 0.033 | -0.041 | 0.106 | 0.385 |
| Coprococcus eutactus | 226 | Not detected | 0.129 | 0.024 | 0.234 | 0.016 |
| Coprococcus eutactus CAG:665 | 644 | Not detected | 0.302 | 0.069 | 0.534 | 0.011 |
| Coprococcus sp. 43_8 | 2 | Not detected | 0.059 | -0.085 | 0.204 | 0.422 |
| Coprococcus sp. CAG:131 | 657 | Not detected | 0.302 | 0.078 | 0.526 | 0.008 |
| Coriobacteriaceae bacterium 68-1-3 | 8 | Not detected | 0.110 | -0.038 | 0.258 | 0.144 |
| Coriobacteriaceae bacterium BV3Ac1 | 1 | Not detected | 0.012 | -0.182 | 0.205 | 0.906 |
| Coriobacteriales bacterium DNF00809 | 8 | Not detected | 0.068 | -0.103 | 0.239 | 0.434 |
| Coriobacterium glomerans | 4 | Not detected | -0.072 | -0.231 | 0.086 | 0.371 |

| Corynebacterium amycolatum | 42 | Not detected | 0.071 | -0.055 | 0.198 | 0.270 |
| --- | --- | --- | --- | --- | --- | --- |
| Corynebacterium durum | 7 | Not detected | 0.071 | -0.118 | 0.260 | 0.463 |
| Corynebacterium glucuronolyticum | 37 | Not detected | 0.074 | -0.076 | 0.224 | 0.335 |
| Corynebacterium singulare | 3 | Not detected | 0.032 | -0.109 | 0.173 | 0.654 |
| Corynebacterium urealyticum | 7 | Not detected | 0.097 | -0.059 | 0.253 | 0.224 |
| Criibacterium bergeronii | 0 | Not detected | 0.013 | -0.092 | 0.117 | 0.813 |
| Cronobacter sakazakii | 0 | Not detected | 0.030 | -0.223 | 0.284 | 0.815 |
| Cryptobacterium sp. CAG:338 | 0 | Not detected | 0.148 | -0.114 | 0.410 | 0.269 |
| Culturomica massiliensis | 0 | Not detected | -0.035 | -0.129 | 0.059 | 0.464 |
| Cutibacterium acnes | 2 | Not detected | -0.123 | -0.297 | 0.051 | 0.166 |
| Defluviitalea phaphyphila | 5 | Not detected | 0.007 | -0.122 | 0.136 | 0.910 |
| Dehalobacter restrictus | 9 | Not detected | 0.070 | -0.062 | 0.201 | 0.298 |
| Dendrosporobacter quercicolus | 36 | Not detected | 0.103 | -0.024 | 0.231 | 0.113 |
| Denitrobacterium detoxificans | 68 | Not detected | 0.044 | -0.088 | 0.176 | 0.512 |
| Desnuesiella massiliensis | 7 | Not detected | 0.072 | -0.063 | 0.207 | 0.295 |
| Desulfovibrio desulfuricans | 0 | Not detected | 0.005 | -0.229 | 0.239 | 0.967 |
| Desulfovibrio fairfieldensis | 0 | Not detected | 0.084 | -0.184 | 0.352 | 0.540 |
| Desulfovibrio piger | 0 | Not detected | -0.114 | -0.443 | 0.215 | 0.498 |
| Desulfovibrio vulgaris | 4 | Not detected | -0.049 | -0.202 | 0.104 | 0.532 |
| Devosia insulae | 5 | Not detected | -0.010 | -0.118 | 0.098 | 0.860 |
| Dialister invisus | 0 | Not detected | 0.055 | -0.295 | 0.406 | 0.757 |
| Dialister invisus CAG:218 | 0 | Not detected | 0.005 | -0.353 | 0.363 | 0.978 |
| Dialister micraerophilus | 1 | Not detected | 0.107 | -0.108 | 0.322 | 0.331 |
| Dialister sp. CAG:357 | 0 | Not detected | 0.218 | -0.124 | 0.561 | 0.211 |
| Dialister sp. CAG:486 | 2 | Not detected | 0.278 | -0.032 | 0.588 | 0.079 |
| Dialister sp. CAG:588 | 1 | Not detected | -0.181 | -0.368 | 0.007 | 0.059 |
| Dialister succinatiphilus | 2 | Not detected | 0.132 | -0.057 | 0.321 | 0.173 |
| Dickeya dadantii | 3 | Not detected | -0.006 | -0.153 | 0.141 | 0.935 |
| Dorea formicigenerans | 372 | Not detected | 0.112 | 0.044 | 0.179 | 0.001 |
| Dorea longicatena | 32 | Not detected | 0.083 | -0.014 | 0.181 | 0.094 |
| Dorea sp. 42_8 | 2 | Not detected | 0.050 | -0.063 | 0.163 | 0.388 |
| Dorea sp. 5-2 | 32 | Not detected | 0.006 | -0.045 | 0.058 | 0.817 |
| Dorea sp. CAG:105 | 2 | Not detected | 0.074 | -0.074 | 0.222 | 0.329 |
| Draconibacterium orientale | 5 | Not detected | -0.088 | -0.213 | 0.036 | 0.165 |
| Drancourtella massiliensis | 223 | Not detected | -0.143 | -0.268 | -0.018 | 0.025 |
| Dubosiella newyorkensis | 3 | Not detected | -0.048 | -0.184 | 0.088 | 0.489 |
| Dysgonomonas gadei | 4 | Not detected | -0.076 | -0.191 | 0.038 | 0.190 |
| Dysgonomonas mossii | 158 | Not detected | -0.167 | -0.305 | -0.029 | 0.017 |
| Dysgonomonas sp. BGC7 | 3 | Not detected | -0.150 | -0.319 | 0.020 | 0.084 |
| Dysgonomonas sp. HGC4 | 26 | Not detected | -0.134 | -0.280 | 0.012 | 0.073 |
| Edwardsiella ictaluri | 3 | Not detected | 0.049 | -0.101 | 0.199 | 0.525 |
| Eggerthella lenta | 0 | Not detected | -0.082 | -0.261 | 0.098 | 0.373 |
| Eggerthella sp. 51_9 | 489 | Not detected | 0.236 | 0.035 | 0.437 | 0.022 |
| Eggerthella sp. CAG:368 | 0 | Not detected | 0.090 | -0.130 | 0.311 | 0.421 |

| Eggerthella sp. YY7918 | 69 | Not detected | -0.187 | -0.337 | -0.037 | 0.015 |
| --- | --- | --- | --- | --- | --- | --- |
| Eggerthellaceae bacterium AT8 | 21 | Not detected | 0.054 | -0.039 | 0.147 | 0.254 |
| Eggerthia catenaformis | 4 | Not detected | 0.076 | -0.113 | 0.266 | 0.431 |
| Eisenbergiella tayi | 117 | Not detected | -0.157 | -0.304 | -0.010 | 0.036 |
| Elizabethkingia anophelis | 0 | Not detected | -0.165 | -0.362 | 0.031 | 0.100 |
| Emergencia timonensis | 214 | Not detected | -0.078 | -0.146 | -0.010 | 0.024 |
| Empedobacter brevis | 601 | Not detected | -0.306 | -0.559 | -0.053 | 0.018 |
| Enhygromyxa salina | 2 | Not detected | 0.003 | -0.162 | 0.168 | 0.971 |
| Enorma massiliensis | 20 | Not detected | 0.069 | -0.027 | 0.164 | 0.161 |
| Enorma timonensis | 121 | Not detected | 0.131 | 0.008 | 0.254 | 0.036 |
| Enterobacter cloacae | 2 | Not detected | 0.167 | -0.098 | 0.433 | 0.217 |
| Enterobacter lignolyticus | 0 | Not detected | 0.004 | -0.237 | 0.245 | 0.976 |
| Enterobacter sp. 638 | 0 | Not detected | 0.006 | -0.217 | 0.229 | 0.958 |
| Enterobacteriaceae bacterium LSJC7 | 2 | Not detected | -0.003 | -0.165 | 0.159 | 0.971 |
| Enterococcus avium | 3 | Not detected | -0.001 | -0.160 | 0.158 | 0.989 |
| Enterococcus cecorum | 0 | Not detected | -0.092 | -0.229 | 0.045 | 0.190 |
| Enterococcus columbae | 0 | Not detected | -0.109 | -0.288 | 0.069 | 0.229 |
| Enterococcus durans | 0 | Not detected | -0.173 | -0.379 | 0.033 | 0.100 |
| Enterococcus faecalis | 0 | Not detected | 0.006 | -0.149 | 0.162 | 0.937 |
| Enterococcus faecium | 55 | Not detected | 0.121 | -0.042 | 0.284 | 0.147 |
| Enterococcus gallinarum | 0 | Not detected | -0.182 | -0.344 | -0.020 | 0.027 |
| Enterococcus gilvus | 133 | Not detected | 0.104 | -0.047 | 0.256 | 0.178 |
| Enterococcus italicus | 2 | Not detected | -0.225 | -0.411 | -0.038 | 0.018 |
| Enterococcus massiliensis | 15 | Not detected | -0.218 | -0.399 | -0.038 | 0.018 |
| Enterococcus sp. HMSC064A12 | 0 | Not detected | -0.103 | -0.287 | 0.081 | 0.273 |
| Enterorhabdus caecimuris | 1 | Not detected | -0.114 | -0.300 | 0.072 | 0.230 |
| Enterorhabdus mucosicola | 2 | Not detected | -0.028 | -0.188 | 0.133 | 0.735 |
| Epulopiscium sp. 'N.t. morphotype B' | 15 | Not detected | 0.053 | -0.068 | 0.174 | 0.391 |
| Erwinia billingiae | 7 | Not detected | 0.045 | -0.101 | 0.191 | 0.548 |
| Erwinia oleae | 1 | Not detected | 0.033 | -0.156 | 0.223 | 0.732 |
| Erysipelatoclostridium ramosum | 0 | Not detected | -0.137 | -0.357 | 0.083 | 0.222 |
| Erysipelotrichaceae bacterium 5_2_54FA | 123 | Not detected | -0.063 | -0.137 | 0.012 | 0.100 |
| Erysipelotrichaceae bacterium CAG:64 | 615 | Not detected | -0.228 | -0.393 | -0.063 | 0.007 |
| Erysipelotrichaceae bacterium I46 | 803 | 0.6 | -0.157 | -0.244 | -0.071 | 0.000 |
| Escherichia coli | 0 | Not detected | -0.063 | -0.306 | 0.180 | 0.610 |
| Escherichia vulneris | 0 | Not detected | 0.008 | -0.205 | 0.222 | 0.940 |
| Ethanoligenens harbinense | 105 | Not detected | 0.100 | -0.065 | 0.266 | 0.234 |
| Eubacterium dolichum CAG:375 | 0 | Not detected | -0.257 | -0.507 | -0.008 | 0.043 |
| Eubacterium limosum | 14 | Not detected | -0.106 | -0.217 | 0.005 | 0.061 |
| Eubacterium plexicaudatum | 56 | Not detected | 0.043 | -0.018 | 0.103 | 0.166 |
| Eubacterium pyruvativorans | 114 | Not detected | 0.058 | -0.020 | 0.135 | 0.145 |
| Eubacterium ramulus | 6 | Not detected | -0.011 | -0.080 | 0.059 | 0.765 |
| Eubacterium sp. 14-2 | 30 | Not detected | -0.026 | -0.089 | 0.037 | 0.422 |
| Eubacterium sp. 3_1_31 | 56 | Not detected | -0.085 | -0.171 | 0.001 | 0.054 |

| Eubacterium sp. 45_250 | 0 | Not detected | 0.060 | -0.183 | 0.303 | 0.630 |
| --- | --- | --- | --- | --- | --- | --- |
| Eubacterium sp. AB3007 | 0 | Not detected | -0.145 | -0.372 | 0.083 | 0.212 |
| Eubacterium sp. CAG:115 | 0 | Not detected | 0.098 | -0.264 | 0.460 | 0.596 |
| Eubacterium sp. CAG:146 | 2 | Not detected | 0.071 | -0.083 | 0.225 | 0.367 |
| Eubacterium sp. CAG:180 | 511 | Not detected | 0.369 | 0.063 | 0.676 | 0.018 |
| Eubacterium sp. CAG:192 | 2 | Not detected | 0.255 | 0.027 | 0.484 | 0.029 |
| Eubacterium sp. CAG:202 | 2 | Not detected | 0.216 | -0.016 | 0.447 | 0.068 |
| Eubacterium sp. CAG:248 | 815 | 0.6 | 0.267 | 0.075 | 0.460 | 0.007 |
| Eubacterium sp. CAG:251 | 0 | Not detected | 0.164 | -0.117 | 0.446 | 0.253 |
| Eubacterium sp. CAG:581 | 0 | Not detected | 0.043 | -0.200 | 0.286 | 0.729 |
| Eubacterium sp. CAG:603 | 769 | 0.6 | 0.212 | 0.085 | 0.338 | 0.001 |
| Eubacterium sp. CAG:786 | 0 | Not detected | -0.011 | -0.294 | 0.272 | 0.938 |
| Eubacterium sp. CAG:841 | 0 | Not detected | 0.189 | -0.080 | 0.458 | 0.169 |
| Eubacterium sp. ER2 | 14 | Not detected | -0.028 | -0.088 | 0.033 | 0.372 |
| Eubacterium ventriosum | 2 | Not detected | 0.013 | -0.076 | 0.103 | 0.774 |
| Eubacterium xylanophilum | 367 | Not detected | 0.187 | 0.046 | 0.327 | 0.009 |
| Facklamia hominis | 1 | Not detected | -0.001 | -0.173 | 0.170 | 0.987 |
| Facklamia ignava | 1 | Not detected | -0.002 | -0.171 | 0.166 | 0.980 |
| Facklamia languida | 2 | Not detected | -0.027 | -0.187 | 0.134 | 0.745 |
| Faecalibacterium prausnitzii | 5 | Not detected | 0.092 | -0.022 | 0.206 | 0.113 |
| Faecalibacterium sp. CAG:1138 | 2 | Not detected | 0.144 | -0.092 | 0.380 | 0.232 |
| Faecalibacterium sp. CAG:82-related_59 | 0 | Not detected | 0.027 | -0.145 | 0.198 | 0.760 |
| Faecalibaculum rodentium | 4 | Not detected | -0.006 | -0.188 | 0.176 | 0.945 |
| Faecalicoccus pleomorphus | 48 | Not detected | 0.065 | -0.030 | 0.160 | 0.177 |
| Faecalitalea cylindroides | 21 | Not detected | 0.006 | -0.054 | 0.065 | 0.854 |
| Fenollaria massiliensis | 395 | Not detected | -0.186 | -0.390 | 0.018 | 0.075 |
| Fermentimonas caenicola | 2 | Not detected | -0.203 | -0.375 | -0.030 | 0.021 |
| Fibrobacter sp. UWB12 | 2 | Not detected | 0.027 | -0.071 | 0.125 | 0.590 |
| Fibrobacter sp. UWCM | 7 | Not detected | 0.127 | -0.006 | 0.261 | 0.062 |
| Fibrobacter sp. UWOV1 | 8 | Not detected | 0.011 | -0.088 | 0.109 | 0.833 |
| Fibrobacterales bacterium Fibro_02 | 282 | Not detected | 0.119 | 0.010 | 0.227 | 0.032 |
| Fibrobacteria bacterium AD80 | 281 | Not detected | 0.115 | 0.005 | 0.224 | 0.040 |
| Filifactor alocis | 1 | Not detected | -0.077 | -0.260 | 0.105 | 0.404 |
| Finegoldia magna | 341 | Not detected | -0.203 | -0.390 | -0.016 | 0.034 |
| Firmicutes bacterium ASF500 | 24 | Not detected | 0.019 | -0.046 | 0.083 | 0.570 |
| Firmicutes bacterium CAG:102 | 2 | Not detected | 0.168 | -0.070 | 0.406 | 0.167 |
| Firmicutes bacterium CAG:137 | 0 | Not detected | -0.132 | -0.414 | 0.150 | 0.360 |
| Firmicutes bacterium CAG:137_57_8 | 0 | Not detected | -0.081 | -0.294 | 0.132 | 0.454 |
| Firmicutes bacterium CAG:145 | 75 | Not detected | -0.257 | -0.509 | -0.004 | 0.047 |
| Firmicutes bacterium CAG:176 | 0 | Not detected | 0.286 | -0.016 | 0.588 | 0.064 |
| Firmicutes bacterium CAG:176_59_8 | 0 | Not detected | 0.228 | -0.047 | 0.503 | 0.104 |
| Firmicutes bacterium CAG:227 | 0 | Not detected | -0.094 | -0.294 | 0.107 | 0.361 |
| Firmicutes bacterium CAG:238 | 0 | Not detected | 0.173 | -0.061 | 0.407 | 0.147 |
| Firmicutes bacterium CAG:24 | 2 | Not detected | 0.113 | -0.053 | 0.278 | 0.182 |

| Firmicutes bacterium CAG:240 | 0 | Not detected | 0.072 | -0.193 | 0.336 | 0.596 |
| --- | --- | --- | --- | --- | --- | --- |
| Firmicutes bacterium CAG:270 | 0 | Not detected | -0.115 | -0.310 | 0.080 | 0.247 |
| Firmicutes bacterium CAG:272 | 0 | Not detected | 0.121 | -0.125 | 0.368 | 0.335 |
| Firmicutes bacterium CAG:272_52_7 | 0 | Not detected | 0.018 | -0.165 | 0.202 | 0.844 |
| Firmicutes bacterium CAG:308 | 0 | Not detected | -0.094 | -0.254 | 0.066 | 0.249 |
| Firmicutes bacterium CAG:313 | 0 | Not detected | 0.038 | -0.289 | 0.364 | 0.822 |
| Firmicutes bacterium CAG:41 | 459 | Not detected | 0.243 | 0.028 | 0.459 | 0.027 |
| Firmicutes bacterium CAG:466 | 0 | Not detected | -0.079 | -0.251 | 0.093 | 0.366 |
| Firmicutes bacterium CAG:475 | 0 | Not detected | -0.030 | -0.266 | 0.206 | 0.801 |
| Firmicutes bacterium CAG:536 | 745 | 0.6 | 0.333 | 0.146 | 0.521 | 0.000 |
| Firmicutes bacterium CAG:552 | 2 | Not detected | 0.182 | -0.045 | 0.410 | 0.116 |
| Firmicutes bacterium CAG:555 | 0 | Not detected | 0.017 | -0.204 | 0.237 | 0.882 |
| Firmicutes bacterium CAG:791 | 118 | Not detected | 0.159 | 0.040 | 0.277 | 0.009 |
| Firmicutes bacterium CAG:882 | 25 | Not detected | 0.113 | -0.034 | 0.260 | 0.131 |
| Firmicutes bacterium CAG:884 | 44 | Not detected | 0.223 | 0.001 | 0.446 | 0.050 |
| Firmicutes bacterium CAG:94 | 0 | Not detected | -0.090 | -0.319 | 0.139 | 0.443 |
| Firmicutes bacterium M10-2 | 329 | Not detected | 0.210 | 0.025 | 0.395 | 0.026 |
| Firmicutes bacterium ZOR0006 | 0 | Not detected | -0.029 | -0.226 | 0.167 | 0.771 |
| Fournierella massiliensis | 9 | Not detected | 0.012 | -0.058 | 0.083 | 0.730 |
| Franconibacter pulveris | 0 | Not detected | 0.014 | -0.187 | 0.214 | 0.892 |
| Fretibacterium fastidiosum | 4 | Not detected | -0.073 | -0.205 | 0.060 | 0.283 |
| Fusicatenibacter saccharivorans | 22 | Not detected | 0.039 | -0.044 | 0.121 | 0.358 |
| Fusobacterium mortiferum | 0 | Not detected | -0.128 | -0.383 | 0.128 | 0.328 |
| Fusobacterium necrophorum | 37 | Not detected | -0.149 | -0.323 | 0.025 | 0.093 |
| Fusobacterium nucleatum | 0 | Not detected | -0.022 | -0.268 | 0.224 | 0.861 |
| Fusobacterium periodonticum | 2 | Not detected | 0.070 | -0.143 | 0.282 | 0.521 |
| Gabonia massiliensis | 0 | Not detected | -0.092 | -0.227 | 0.044 | 0.185 |
| Gabonibacter massiliensis | 0 | Not detected | -0.124 | -0.283 | 0.036 | 0.129 |
| Gallibacterium anatis | 44 | Not detected | 0.060 | -0.067 | 0.187 | 0.355 |
| Gardnerella vaginalis | 404 | Not detected | -0.185 | -0.377 | 0.007 | 0.060 |
| Gemella haemolysans | 3 | Not detected | -0.033 | -0.214 | 0.148 | 0.721 |
| Gemella sanguinis | 3 | Not detected | 0.053 | -0.167 | 0.274 | 0.634 |
| Gemmata obscuriglobus | 40 | Not detected | 0.066 | -0.090 | 0.222 | 0.405 |
| Geofilum rubicundum | 282 | Not detected | -0.185 | -0.334 | -0.036 | 0.015 |
| Gilliamella apicola | 7 | Not detected | 0.095 | -0.052 | 0.243 | 0.206 |
| Globicatella sanguinis | 3 | Not detected | 0.081 | -0.090 | 0.251 | 0.354 |
| Gordonibacter pamelaeae | 7 | Not detected | 0.075 | -0.052 | 0.201 | 0.249 |
| Granulicatella adiacens | 3 | Not detected | 0.056 | -0.157 | 0.269 | 0.607 |
| Granulicatella elegans | 0 | Not detected | -0.075 | -0.254 | 0.104 | 0.411 |
| Haemophilus influenzae | 355 | Not detected | 0.323 | 0.085 | 0.561 | 0.008 |
| Haemophilus sputorum | 97 | Not detected | 0.268 | 0.022 | 0.514 | 0.033 |
| Hafnia sp. HMSC23F03 | 2 | Not detected | 0.084 | -0.096 | 0.263 | 0.362 |
| Hathewaya proteolytica | 3 | Not detected | -0.050 | -0.223 | 0.123 | 0.570 |
| Heliobacterium sp. N15.MGS-306 | 7 | Not detected | 0.025 | -0.050 | 0.101 | 0.509 |

| Hespellia stercorisuis | 12 | Not detected | -0.036 | -0.106 | 0.034 | 0.313 |
| --- | --- | --- | --- | --- | --- | --- |
| Holdemanella biformis | 2 | Not detected | 0.070 | -0.058 | 0.199 | 0.284 |
| Holdemania filiformis | 146 | Not detected | -0.071 | -0.149 | 0.008 | 0.079 |
| Holdemania massiliensis | 0 | Not detected | -0.030 | -0.134 | 0.075 | 0.579 |
| Holdemania sp. Marseille-P2844 | 4 | Not detected | -0.039 | -0.132 | 0.055 | 0.416 |
| Hungatella hathewayi | 663 | Not detected | -0.129 | -0.213 | -0.044 | 0.003 |
| Hydrogenoanaerobacterium saccharovora | 10 | Not detected | 0.016 | -0.097 | 0.129 | 0.781 |
| Idiomarina zobellii | 70 | Not detected | 0.266 | 0.053 | 0.478 | 0.015 |
| Ileibacterium valens | 2 | Not detected | 0.067 | -0.126 | 0.259 | 0.498 |
| Intestinibacter bartlettii | 0 | Not detected | -0.055 | -0.219 | 0.109 | 0.512 |
| Intestinimonas butyriciproducens | 2 | Not detected | 0.038 | -0.063 | 0.139 | 0.463 |
| Intestinimonas massiliensis | 7 | Not detected | 0.048 | -0.043 | 0.140 | 0.301 |
| Izhakiella sp. D4N98 | 1 | Not detected | -0.028 | -0.185 | 0.129 | 0.728 |
| Jeotgalibaca sp. PTS2502 | 1 | Not detected | -0.014 | -0.153 | 0.124 | 0.839 |
| Johnsonella ignava | 27 | Not detected | 0.056 | -0.028 | 0.140 | 0.189 |
| Kallipyga massiliensis | 307 | Not detected | -0.265 | -0.437 | -0.093 | 0.003 |
| Kandleria vitulina | 577 | Not detected | 0.305 | 0.059 | 0.551 | 0.015 |
| Kiloniella litopenaei | 3 | Not detected | 0.068 | -0.100 | 0.236 | 0.425 |
| Kingella kingae | 2 | Not detected | 0.047 | -0.112 | 0.207 | 0.560 |
| Klebsiella oxytoca | 0 | Not detected | -0.026 | -0.311 | 0.259 | 0.858 |
| Klebsiella pneumoniae | 0 | Not detected | 0.068 | -0.221 | 0.357 | 0.644 |
| Klebsiella sp. RIT-PI-d | 0 | Not detected | -0.042 | -0.256 | 0.172 | 0.701 |
| Kluyvera ascorbata | 2 | Not detected | 0.155 | -0.109 | 0.418 | 0.250 |
| Kluyvera cryocrescens | 0 | Not detected | 0.108 | -0.162 | 0.378 | 0.433 |
| Kluyvera intermedia | 0 | Not detected | 0.082 | -0.169 | 0.334 | 0.520 |
| Kocuria sp. ZOR0020 | 138 | Not detected | -0.207 | -0.350 | -0.064 | 0.005 |
| Kosakonia sacchari | 0 | Not detected | -0.018 | -0.246 | 0.209 | 0.875 |
| Lachnoanaerobaculum saburreum | 45 | Not detected | 0.076 | -0.012 | 0.163 | 0.091 |
| Lachnoanaerobaculum sp. OBRC5-5 | 3 | Not detected | 0.024 | -0.058 | 0.106 | 0.564 |
| Lachnobacterium bovis | 22 | Not detected | 0.028 | -0.053 | 0.109 | 0.496 |
| Lachnoclostridium phytofermentans | 42 | Not detected | 0.011 | -0.052 | 0.073 | 0.733 |
| Lachnospira multipara | 409 | Not detected | 0.139 | 0.023 | 0.255 | 0.019 |
| Lachnospiraceae bacterium 10-1 | 72 | Not detected | 0.046 | -0.006 | 0.099 | 0.082 |
| Lachnospiraceae bacterium 28-4 | 28 | Not detected | 0.015 | -0.040 | 0.070 | 0.588 |
| Lachnospiraceae bacterium 2_1_46FAA | 623 | Not detected | -0.124 | -0.204 | -0.043 | 0.003 |
| Lachnospiraceae bacterium 3-1 | 10 | Not detected | -0.008 | -0.071 | 0.056 | 0.811 |
| Lachnospiraceae bacterium 3-2 | 51 | Not detected | -0.043 | -0.106 | 0.019 | 0.173 |
| Lachnospiraceae bacterium A2 | 20 | Not detected | 0.009 | -0.056 | 0.074 | 0.785 |
| Lachnospiraceae bacterium A4 | 32 | Not detected | -0.019 | -0.077 | 0.039 | 0.521 |
| Lachnospiraceae bacterium AB2028 | 131 | Not detected | 0.143 | -0.037 | 0.323 | 0.121 |
| Lachnospiraceae bacterium AC2012 | 129 | Not detected | 0.064 | -0.010 | 0.138 | 0.090 |
| Lachnospiraceae bacterium AC2014 | 23 | Not detected | 0.072 | -0.043 | 0.187 | 0.221 |
| Lachnospiraceae bacterium AC2028 | 79 | Not detected | 0.143 | 0.016 | 0.269 | 0.027 |
| Lachnospiraceae bacterium AC2029 | 59 | Not detected | 0.023 | -0.058 | 0.104 | 0.579 |

| Lachnospiraceae bacterium AC2031 | 66 | Not detected | 0.070 | -0.048 | 0.187 | 0.246 |
| --- | --- | --- | --- | --- | --- | --- |
| Lachnospiraceae bacterium AC3007 | 37 | Not detected | 0.064 | -0.086 | 0.214 | 0.402 |
| Lachnospiraceae bacterium AD3010 | 3 | Not detected | 0.044 | -0.130 | 0.217 | 0.622 |
| Lachnospiraceae bacterium C6A11 | 181 | Not detected | 0.128 | 0.015 | 0.240 | 0.026 |
| Lachnospiraceae bacterium COE1 | 24 | Not detected | 0.047 | -0.035 | 0.129 | 0.260 |
| Lachnospiraceae bacterium FD2005 | 122 | Not detected | 0.133 | -0.016 | 0.281 | 0.080 |
| Lachnospiraceae bacterium FE2018 | 22 | Not detected | -0.165 | -0.341 | 0.011 | 0.067 |
| Lachnospiraceae bacterium G41 | 96 | Not detected | 0.158 | 0.006 | 0.310 | 0.041 |
| Lachnospiraceae bacterium KHCPX20 | 51 | Not detected | 0.114 | -0.057 | 0.285 | 0.193 |
| Lachnospiraceae bacterium M18-1 | 8 | Not detected | -0.006 | -0.068 | 0.056 | 0.840 |
| Lachnospiraceae bacterium NC2004 | 6 | Not detected | 0.048 | -0.116 | 0.211 | 0.570 |
| Lachnospiraceae bacterium NC2008 | 121 | Not detected | 0.119 | -0.032 | 0.269 | 0.122 |
| Lachnospiraceae bacterium ND2006 | 74 | Not detected | 0.113 | -0.043 | 0.269 | 0.157 |
| Lachnospiraceae bacterium NK3A20 | 2 | Not detected | -0.055 | -0.230 | 0.121 | 0.542 |
| Lachnospiraceae bacterium NK4A179 | 60 | Not detected | 0.115 | -0.044 | 0.273 | 0.156 |
| Lachnospiraceae bacterium NLAE-zl-G2 | 271 | Not detected | -0.192 | -0.356 | -0.028 | 0.022 |
| Lachnospiraceae bacterium P6B14 | 73 | Not detected | 0.071 | -0.056 | 0.197 | 0.272 |
| Lachnospiraceae bacterium TF01-11 | 322 | Not detected | 0.123 | 0.039 | 0.206 | 0.004 |
| Lachnospiraceae bacterium TWA4 | 75 | Not detected | 0.006 | -0.081 | 0.093 | 0.897 |
| Lachnospiraceae bacterium V9D3004 | 100 | Not detected | 0.178 | 0.025 | 0.331 | 0.022 |
| Lachnospiraceae bacterium YSB2008 | 157 | Not detected | 0.120 | 0.008 | 0.233 | 0.037 |
| Lachnospiraceae bacterium mt14 | 13 | Not detected | 0.050 | -0.095 | 0.195 | 0.502 |
| Lachnospiraceae bacterium oral taxon 50 | 43 | Not detected | 0.049 | -0.017 | 0.115 | 0.145 |
| Lactobacillus acidophilus | 2 | Not detected | 0.024 | -0.103 | 0.152 | 0.709 |
| Lactobacillus amylotrophicus | 3 | Not detected | -0.096 | -0.257 | 0.065 | 0.245 |
| Lactobacillus animalis | 0 | Not detected | -0.033 | -0.195 | 0.129 | 0.692 |
| Lactobacillus antri | 2 | Not detected | -0.141 | -0.318 | 0.036 | 0.120 |
| Lactobacillus aviarius | 58 | Not detected | 0.017 | -0.073 | 0.107 | 0.713 |
| Lactobacillus casei | 0 | Not detected | -0.163 | -0.389 | 0.063 | 0.157 |
| Lactobacillus curvatus | 2 | Not detected | -0.144 | -0.320 | 0.033 | 0.111 |
| Lactobacillus delbrueckii | 0 | Not detected | -0.175 | -0.374 | 0.024 | 0.085 |
| Lactobacillus equi | 3 | Not detected | -0.144 | -0.318 | 0.030 | 0.105 |
| Lactobacillus fermentum | 0 | Not detected | -0.090 | -0.345 | 0.165 | 0.489 |
| Lactobacillus gasseri | 0 | Not detected | -0.170 | -0.427 | 0.088 | 0.196 |
| Lactobacillus helveticus | 0 | Not detected | -0.049 | -0.237 | 0.140 | 0.613 |
| Lactobacillus ingluviei | 2 | Not detected | -0.021 | -0.172 | 0.130 | 0.787 |
| Lactobacillus jensenii | 497 | Not detected | -0.215 | -0.377 | -0.053 | 0.009 |
| Lactobacillus kefiranofaciens | 2 | Not detected | -0.087 | -0.234 | 0.060 | 0.245 |
| Lactobacillus manihotivorans | 6 | Not detected | 0.053 | -0.111 | 0.217 | 0.527 |
| Lactobacillus mucosae | 1 | Not detected | 0.015 | -0.157 | 0.186 | 0.869 |
| Lactobacillus paracasei | 0 | Not detected | -0.107 | -0.339 | 0.125 | 0.365 |
| Lactobacillus pasteurii | 29 | Not detected | 0.084 | -0.056 | 0.223 | 0.240 |
| Lactobacillus plantarum | 0 | Not detected | -0.050 | -0.245 | 0.145 | 0.615 |
| Lactobacillus rhamnosus | 0 | Not detected | -0.154 | -0.363 | 0.054 | 0.147 |

| Lactobacillus ruminis | 57 | Not detected | 0.182 | 0.012 | 0.351 | 0.036 |
| --- | --- | --- | --- | --- | --- | --- |
| Lactobacillus ruminis CAG:367 | 2 | Not detected | 0.026 | -0.142 | 0.195 | 0.761 |
| Lactobacillus sakei | 0 | Not detected | -0.070 | -0.242 | 0.102 | 0.426 |
| Lactobacillus salivarius | 0 | Not detected | 0.033 | -0.214 | 0.280 | 0.795 |
| Lactobacillus vaginalis | 0 | Not detected | -0.112 | -0.330 | 0.106 | 0.314 |
| Lactococcus garvieae | 0 | Not detected | -0.146 | -0.323 | 0.031 | 0.106 |
| Lactococcus lactis | 361 | Not detected | -0.291 | -0.562 | -0.021 | 0.035 |
| Lactococcus piscium | 2 | Not detected | -0.148 | -0.329 | 0.032 | 0.107 |
| Lactonifactor longoviformis | 52 | Not detected | -0.033 | -0.088 | 0.022 | 0.236 |
| Lamprocystis purpurea | 0 | Not detected | -0.035 | -0.251 | 0.181 | 0.752 |
| Lascolabacillus massiliensis | 5 | Not detected | -0.115 | -0.262 | 0.032 | 0.125 |
| Lautropia mirabilis | 3 | Not detected | 0.124 | -0.056 | 0.304 | 0.176 |
| Lawsonia intracellularis | 2 | Not detected | -0.062 | -0.238 | 0.114 | 0.491 |
| Leclercia adecarboxylata | 0 | Not detected | 0.117 | -0.137 | 0.371 | 0.368 |
| Lelliottia amnigena | 0 | Not detected | 0.126 | -0.151 | 0.404 | 0.373 |
| Lentimicrobium saccharophilum | 2 | Not detected | -0.051 | -0.242 | 0.140 | 0.601 |
| Leuconostoc citreum | 1 | Not detected | -0.022 | -0.182 | 0.137 | 0.783 |
| Leuconostoc lactis | 0 | Not detected | 0.000 | -0.191 | 0.190 | 0.997 |
| Leuconostoc mesenteroides | 2 | Not detected | 0.011 | -0.192 | 0.214 | 0.918 |
| Levyella massiliensis | 3 | Not detected | 0.035 | -0.055 | 0.126 | 0.446 |
| Libanicoccus massiliensis | 2 | Not detected | 0.125 | -0.037 | 0.287 | 0.130 |
| Luteimonas sp. FCS-9 | 5 | Not detected | -0.006 | -0.142 | 0.130 | 0.929 |
| Lutispora thermophila | 23 | Not detected | 0.045 | -0.113 | 0.202 | 0.581 |
| Macellibacteroides sp. HH-ZS | 575 | Not detected | -0.181 | -0.303 | -0.059 | 0.004 |
| Macrococcus caseolyticus | 14 | Not detected | -0.196 | -0.369 | -0.022 | 0.027 |
| Mageeibacillus indolicus | 11 | Not detected | -0.113 | -0.241 | 0.015 | 0.085 |
| Mangrovibacter sp. MFB070 | 0 | Not detected | 0.022 | -0.179 | 0.223 | 0.830 |
| Mannheimia haemolytica | 33 | Not detected | 0.147 | -0.019 | 0.314 | 0.082 |
| Marvinbryantia formatexigens | 22 | Not detected | -0.046 | -0.125 | 0.033 | 0.255 |
| Massilioclostridium coli | 4 | Not detected | 0.001 | -0.079 | 0.080 | 0.986 |
| Massiliomicrobiota timonensis | 3 | Not detected | 0.060 | -0.120 | 0.241 | 0.511 |
| Mediterranea massiliensis | 2 | Not detected | 0.020 | -0.083 | 0.123 | 0.705 |
| Megamonas funiformis | 0 | Not detected | -0.001 | -0.315 | 0.314 | 0.997 |
| Megasphaera cerevisiae | 2 | Not detected | 0.058 | -0.176 | 0.292 | 0.629 |
| Megasphaera elsdenii | 0 | Not detected | -0.134 | -0.435 | 0.166 | 0.381 |
| Megasphaera genomosp. type_1 | 57 | Not detected | 0.043 | -0.016 | 0.102 | 0.155 |
| Megasphaera massiliensis | 0 | Not detected | -0.167 | -0.449 | 0.116 | 0.248 |
| Megasphaera micronuciformis | 0 | Not detected | -0.159 | -0.370 | 0.053 | 0.142 |
| Megasphaera sp. BV3C16-1 | 2 | Not detected | 0.092 | -0.088 | 0.271 | 0.316 |
| Megasphaera sp. UPII 135-E | 38 | Not detected | 0.018 | -0.043 | 0.079 | 0.555 |
| Merdibacter massiliensis | 6 | Not detected | -0.020 | -0.088 | 0.048 | 0.563 |
| Methanobrevibacter smithii | 0 | Not detected | -0.080 | -0.439 | 0.279 | 0.662 |
| Methanobrevibacter smithii CAG:186 | 0 | Not detected | -0.055 | -0.390 | 0.280 | 0.747 |
| Methanocorpusculaceae archaeon Phil4 | 2 | Not detected | 0.199 | -0.035 | 0.433 | 0.096 |

| Methanosarcina mazei | 2 | Not detected | -0.006 | -0.212 | 0.200 | 0.954 |
| --- | --- | --- | --- | --- | --- | --- |
| Methylobacterium sp. ZNC0032 | 55 | Not detected | -0.206 | -0.351 | -0.061 | 0.005 |
| Methyloglobulus morosus | 3 | Not detected | 0.022 | -0.149 | 0.193 | 0.801 |
| Microcystis aeruginosa | 3 | Not detected | 0.095 | -0.082 | 0.272 | 0.294 |
| Mitsuokella multacida | 0 | Not detected | 0.154 | -0.152 | 0.461 | 0.323 |
| Mobilibacterium timonense | 25 | Not detected | 0.078 | -0.089 | 0.246 | 0.360 |
| Mobiluncus curtisii | 44 | Not detected | 0.014 | -0.056 | 0.084 | 0.697 |
| Mobiluncus mulieris | 53 | Not detected | 0.012 | -0.069 | 0.094 | 0.771 |
| Mogibacterium timidum | 9 | Not detected | 0.089 | -0.108 | 0.287 | 0.377 |
| Moraxella porci | 3 | Not detected | 0.039 | -0.102 | 0.179 | 0.590 |
| Morganella morganii | 0 | Not detected | -0.082 | -0.273 | 0.109 | 0.399 |
| Morococcus cerebrosus | 2 | Not detected | 0.044 | -0.111 | 0.199 | 0.578 |
| Mucinivorans hirudinis | 36 | Not detected | -0.179 | -0.319 | -0.040 | 0.012 |
| Murdochiella sp. Marseille-P2341 | 6 | Not detected | -0.029 | -0.120 | 0.063 | 0.541 |
| Mycoplasma sp. CAG:877 | 2 | Not detected | 0.228 | -0.016 | 0.473 | 0.068 |
| Mycoplasma sp. CAG:956 | 25 | Not detected | 0.185 | -0.030 | 0.401 | 0.092 |
| Myroides injenensis | 0 | Not detected | -0.148 | -0.358 | 0.062 | 0.168 |
| Myroides odoratimimus | 0 | Not detected | -0.111 | -0.332 | 0.110 | 0.326 |
| Necropsobacter massiliensis | 5 | Not detected | 0.092 | -0.102 | 0.287 | 0.351 |
| Negativicoccus massiliensis | 3 | Not detected | 0.059 | -0.094 | 0.213 | 0.450 |
| Neglecta timonensis | 15 | Not detected | -0.068 | -0.173 | 0.037 | 0.207 |
| Neisseria cinerea | 3 | Not detected | -0.101 | -0.268 | 0.067 | 0.238 |
| Neisseria mucosa | 43 | Not detected | 0.157 | -0.014 | 0.328 | 0.072 |
| Nissabacter archeti | 3 | Not detected | 0.098 | -0.098 | 0.293 | 0.327 |
| Novibacillus thermophilus | 3 | Not detected | -0.079 | -0.255 | 0.098 | 0.381 |
| Obesumbacterium proteus | 0 | Not detected | 0.030 | -0.203 | 0.263 | 0.803 |
| Oceanospirillum linum | 3 | Not detected | 0.054 | -0.111 | 0.220 | 0.519 |
| Odoribacter laneus CAG:561 | 0 | Not detected | -0.173 | -0.381 | 0.036 | 0.104 |
| Odoribacter sp. 43_10 | 1 | Not detected | 0.026 | -0.103 | 0.155 | 0.690 |
| Odoribacter sp. CAG:788 | 0 | Not detected | -0.118 | -0.325 | 0.089 | 0.265 |
| Odoribacter splanchnicus | 1 | Not detected | -0.021 | -0.117 | 0.074 | 0.664 |
| Odoribacter splanchnicus CAG:14 | 2 | Not detected | 0.081 | -0.061 | 0.223 | 0.265 |
| Oligella ureolytica | 3 | Not detected | 0.036 | -0.132 | 0.203 | 0.677 |
| Olsenella scatoligenes | 370 | Not detected | 0.266 | 0.083 | 0.448 | 0.004 |
| Olsenella sp. Marseille-P3197 | 2 | Not detected | 0.117 | -0.064 | 0.299 | 0.204 |
| Olsenella sp. oral taxon 809 | 7 | Not detected | 0.054 | -0.073 | 0.182 | 0.403 |
| Olsenella uli | 44 | Not detected | 0.120 | -0.062 | 0.302 | 0.198 |
| Olsenella umbonata | 3 | Not detected | -0.001 | -0.149 | 0.147 | 0.992 |
| Oribacterium parvum | 73 | Not detected | 0.049 | -0.010 | 0.107 | 0.104 |
| Oribacterium sinus | 3 | Not detected | 0.057 | -0.082 | 0.197 | 0.420 |
| Oribacterium sp. WCC10 | 3 | Not detected | 0.109 | -0.075 | 0.293 | 0.246 |
| Oribacterium sp. oral taxon 078 | 31 | Not detected | 0.040 | -0.055 | 0.135 | 0.409 |
| Oscillibacter ruminantium | 2 | Not detected | 0.017 | -0.113 | 0.147 | 0.796 |
| Oscillibacter sp. 1-3 | 7 | Not detected | 0.000 | -0.071 | 0.071 | 0.999 |

| Oscillibacter sp. 57_20 | 518 | Not detected | 0.290 | 0.060 | 0.519 | 0.013 |
| --- | --- | --- | --- | --- | --- | --- |
| Oscillibacter sp. ER4 | 233 | Not detected | 0.163 | 0.027 | 0.299 | 0.019 |
| Oscillibacter sp. KLE 1745 | 2 | Not detected | -0.086 | -0.202 | 0.030 | 0.144 |
| Oscillibacter valericigenes | 8 | Not detected | 0.051 | -0.065 | 0.167 | 0.393 |
| Oscillospiraceae bacterium Zagget12 | 2 | Not detected | -0.026 | -0.168 | 0.115 | 0.715 |
| Oxalobacter formigenes | 2 | Not detected | 0.056 | -0.089 | 0.201 | 0.450 |
| Paeniclostridium sordellii | 2 | Not detected | 0.041 | -0.156 | 0.238 | 0.683 |
| Paludibacter propionicigenes | 5 | Not detected | -0.019 | -0.154 | 0.116 | 0.780 |
| Pantoea agglomerans | 1 | Not detected | -0.025 | -0.188 | 0.138 | 0.765 |
| Pantoea ananatis | 3 | Not detected | -0.008 | -0.157 | 0.141 | 0.915 |
| Pantoea rwandensis | 3 | Not detected | 0.033 | -0.124 | 0.189 | 0.683 |
| Pantoea sp. IMH | 7 | Not detected | 0.058 | -0.101 | 0.217 | 0.476 |
| Pantoea sp. PSNIH2 | 0 | Not detected | 0.040 | -0.207 | 0.288 | 0.749 |
| Parabacteroides distasonis | 77 | Not detected | -0.152 | -0.285 | -0.019 | 0.025 |
| Parabacteroides goldsteinii | 0 | Not detected | -0.082 | -0.226 | 0.062 | 0.265 |
| Parabacteroides gordonii | 736 | 0.6 | -0.174 | -0.292 | -0.056 | 0.004 |
| Parabacteroides johnsonii | 35 | Not detected | -0.112 | -0.217 | -0.007 | 0.037 |
| Parabacteroides merdae CAG:48 | 0 | Not detected | -0.079 | -0.272 | 0.114 | 0.422 |
| Paraburkholderia sacchari | 149 | Not detected | -0.176 | -0.337 | -0.016 | 0.032 |
| Paraclostridium bifermentans | 4 | Not detected | 0.121 | -0.056 | 0.298 | 0.179 |
| Paraprevotella clara CAG:116 | 0 | Not detected | 0.114 | -0.120 | 0.349 | 0.338 |
| Paraprevotella xylaniphila | 36 | Not detected | 0.119 | -0.007 | 0.244 | 0.063 |
| Parascardovia denticolens | 0 | Not detected | -0.024 | -0.209 | 0.160 | 0.797 |
| Parasporobacterium paucivorans | 47 | Not detected | -0.004 | -0.113 | 0.105 | 0.941 |
| Parasutterella excrementihominis | 0 | Not detected | -0.018 | -0.251 | 0.214 | 0.878 |
| Parasutterella excrementihominis CAG:2 | 0 | Not detected | -0.101 | -0.456 | 0.254 | 0.577 |
| Parvimonas micra | 2 | Not detected | -0.172 | -0.378 | 0.033 | 0.100 |
| Pasteurella bettyae | 83 | Not detected | 0.150 | -0.013 | 0.313 | 0.071 |
| Pectobacterium carotovorum | 3 | Not detected | 0.009 | -0.141 | 0.158 | 0.911 |
| Pedobacter glucosidilyticus | 3 | Not detected | 0.027 | -0.129 | 0.184 | 0.733 |
| Pelosinus fermentans | 21 | Not detected | 0.113 | -0.037 | 0.263 | 0.139 |
| Peptoanaerobacter stomatis | 56 | Not detected | 0.146 | -0.009 | 0.301 | 0.065 |
| Peptococcus niger | 3 | Not detected | -0.070 | -0.219 | 0.080 | 0.360 |
| Peptoniphilus duerdenii | 177 | Not detected | -0.131 | -0.257 | -0.006 | 0.041 |
| Peptoniphilus grossensis | 733 | 0.6 | -0.302 | -0.468 | -0.137 | 0.000 |
| Peptoniphilus harei | 285 | Not detected | -0.168 | -0.355 | 0.020 | 0.080 |
| Peptoniphilus lacrimalis | 501 | Not detected | -0.188 | -0.389 | 0.013 | 0.067 |
| Peptoniphilus obesi | 457 | Not detected | -0.228 | -0.404 | -0.051 | 0.012 |
| Peptoniphilus rhinitidis | 3 | Not detected | -0.083 | -0.256 | 0.091 | 0.349 |
| Peptoniphilus senegalensis | 2 | Not detected | -0.082 | -0.269 | 0.106 | 0.394 |
| Peptoniphilus sp. BV3C26 | 2 | Not detected | -0.123 | -0.325 | 0.079 | 0.234 |
| Peptoniphilus sp. ING2-D1G | 3 | Not detected | -0.115 | -0.289 | 0.058 | 0.193 |
| Peptoniphilus sp. oral taxon 375 | 7 | Not detected | -0.011 | -0.095 | 0.073 | 0.795 |
| Peptostreptococcaceae bacterium VA2 | 5 | Not detected | 0.167 | -0.045 | 0.379 | 0.122 |

| Peptostreptococcus anaerobius | 5 | Not detected | -0.029 | -0.095 | 0.037 | 0.385 |
| --- | --- | --- | --- | --- | --- | --- |
| Peptostreptococcus anaerobius CAG:621 | 3 | Not detected | -0.071 | -0.220 | 0.078 | 0.350 |
| Peptostreptococcus stomatis | 2 | Not detected | -0.115 | -0.295 | 0.065 | 0.210 |
| Petrimonas mucosa | 2 | Not detected | 0.072 | -0.097 | 0.240 | 0.404 |
| Phaeodactylibacter xiamenensis | 1 | Not detected | -0.242 | -0.467 | -0.016 | 0.036 |
| Phascolarctobacterium sp. CAG:207 | 0 | Not detected | -0.065 | -0.404 | 0.274 | 0.708 |
| Phascolarctobacterium sp. CAG:266 | 0 | Not detected | -0.128 | -0.365 | 0.109 | 0.290 |
| Phascolarctobacterium succinatutens | 2 | Not detected | 0.148 | -0.024 | 0.319 | 0.093 |
| Phocaeicola abscessus | 47 | Not detected | -0.142 | -0.282 | -0.001 | 0.048 |
| Phocea massiliensis | 2 | Not detected | 0.006 | -0.082 | 0.095 | 0.887 |
| Piscirickettsia salmonis | 2 | Not detected | -0.015 | -0.159 | 0.129 | 0.838 |
| Plautia stali symbiont | 7 | Not detected | 0.063 | -0.086 | 0.211 | 0.408 |
| Pluralibacter gergoviae | 0 | Not detected | 0.004 | -0.230 | 0.238 | 0.973 |
| Pontibacillus litoralis | 495 | Not detected | -0.216 | -0.430 | -0.001 | 0.049 |
| Porphyromonadaceae bacterium COT-18 | 4 | Not detected | -0.105 | -0.262 | 0.052 | 0.190 |
| Porphyromonadaceae bacterium KH3CP3 | 422 | Not detected | -0.211 | -0.364 | -0.058 | 0.007 |
| Porphyromonadaceae bacterium KH3R12 | 6 | Not detected | -0.024 | -0.173 | 0.125 | 0.755 |
| Porphyromonas asaccharolytica | 753 | 0.6 | -0.266 | -0.459 | -0.074 | 0.007 |
| Porphyromonas bennonis | 148 | Not detected | -0.156 | -0.340 | 0.027 | 0.095 |
| Porphyromonas cangingivalis | 1 | Not detected | -0.041 | -0.209 | 0.128 | 0.638 |
| Porphyromonas canoris | 3 | Not detected | -0.104 | -0.273 | 0.065 | 0.229 |
| Porphyromonas catoniae | 5 | Not detected | -0.042 | -0.172 | 0.088 | 0.526 |
| Porphyromonas crevioricanis | 43 | Not detected | 0.096 | -0.020 | 0.212 | 0.106 |
| Porphyromonas endodontalis | 0 | Not detected | -0.127 | -0.313 | 0.060 | 0.183 |
| Porphyromonas gingivalis | 0 | Not detected | -0.077 | -0.230 | 0.075 | 0.319 |
| Porphyromonas gingivicanis | 25 | Not detected | -0.129 | -0.251 | -0.006 | 0.040 |
| Porphyromonas levii | 0 | Not detected | -0.101 | -0.323 | 0.122 | 0.376 |
| Porphyromonas macacae | 2 | Not detected | -0.058 | -0.223 | 0.108 | 0.496 |
| Porphyromonas somerae | 2 | Not detected | 0.007 | -0.073 | 0.087 | 0.862 |
| Porphyromonas sp. CAG:1061 | 8 | Not detected | -0.171 | -0.373 | 0.031 | 0.097 |
| Porphyromonas sp. COT-239 OH1446 | 2 | Not detected | 0.011 | -0.196 | 0.217 | 0.918 |
| Porphyromonas sp. COT-290 OH3588 | 0 | Not detected | 0.037 | -0.164 | 0.238 | 0.719 |
| Prevotella albensis | 571 | Not detected | 0.359 | 0.071 | 0.647 | 0.015 |
| Prevotella amnii | 2 | Not detected | 0.004 | -0.072 | 0.080 | 0.920 |
| Prevotella aurantiaca | 0 | Not detected | 0.093 | -0.094 | 0.280 | 0.329 |
| Prevotella baroniae | 0 | Not detected | -0.022 | -0.111 | 0.068 | 0.634 |
| Prevotella bergensis | 2 | Not detected | 0.120 | -0.022 | 0.261 | 0.099 |
| Prevotella bivia | 2 | Not detected | 0.080 | -0.018 | 0.178 | 0.111 |
| Prevotella bryantii | 0 | Not detected | 0.203 | -0.086 | 0.491 | 0.169 |
| Prevotella buccae | 7 | Not detected | 0.230 | 0.010 | 0.450 | 0.041 |
| Prevotella buccalis | 2 | Not detected | 0.027 | -0.055 | 0.110 | 0.519 |
| Prevotella copri | 0 | Not detected | 0.268 | -0.038 | 0.574 | 0.086 |
| Prevotella corporis | 4 | Not detected | -0.049 | -0.130 | 0.032 | 0.237 |
| Prevotella dentalis | 2 | Not detected | 0.260 | 0.034 | 0.487 | 0.025 |

| Prevotella dentasini | 2 | Not detected | 0.182 | -0.044 | 0.407 | 0.114 |
| --- | --- | --- | --- | --- | --- | --- |
| Prevotella denticola | 0 | Not detected | 0.162 | -0.089 | 0.414 | 0.206 |
| Prevotella disiens | 0 | Not detected | 0.001 | -0.086 | 0.088 | 0.983 |
| Prevotella enoeca | 2 | Not detected | 0.172 | -0.047 | 0.390 | 0.125 |
| Prevotella fusca | 2 | Not detected | 0.218 | -0.031 | 0.468 | 0.087 |
| Prevotella histicola | 0 | Not detected | 0.027 | -0.127 | 0.181 | 0.728 |
| Prevotella intermedia | 0 | Not detected | 0.003 | -0.151 | 0.158 | 0.965 |
| Prevotella loescheii | 2 | Not detected | 0.154 | -0.055 | 0.362 | 0.149 |
| Prevotella maculosa | 2 | Not detected | 0.076 | -0.105 | 0.257 | 0.412 |
| Prevotella marshii | 0 | Not detected | -0.053 | -0.140 | 0.034 | 0.233 |
| Prevotella melaninogenica | 2 | Not detected | 0.165 | -0.012 | 0.343 | 0.068 |
| Prevotella micans | 2 | Not detected | 0.141 | -0.068 | 0.351 | 0.187 |
| Prevotella multiformis | 82 | Not detected | 0.139 | 0.009 | 0.269 | 0.036 |
| Prevotella multisaccharivorax | 2 | Not detected | 0.324 | 0.040 | 0.609 | 0.025 |
| Prevotella nanceiensis | 0 | Not detected | -0.008 | -0.176 | 0.160 | 0.925 |
| Prevotella nigrescens | 2 | Not detected | 0.194 | -0.011 | 0.399 | 0.064 |
| Prevotella oralis | 2 | Not detected | 0.163 | -0.055 | 0.381 | 0.143 |
| Prevotella oris | 0 | Not detected | 0.095 | -0.148 | 0.338 | 0.444 |
| Prevotella oryzae | 2 | Not detected | 0.300 | 0.048 | 0.553 | 0.020 |
| Prevotella oulorum | 0 | Not detected | 0.253 | -0.008 | 0.515 | 0.058 |
| Prevotella pallens | 2 | Not detected | 0.039 | -0.058 | 0.137 | 0.428 |
| Prevotella paludivivens | 7 | Not detected | 0.160 | -0.013 | 0.333 | 0.071 |
| Prevotella ruminicola | 2 | Not detected | 0.185 | -0.057 | 0.427 | 0.134 |
| Prevotella saccharolytica | 0 | Not detected | 0.106 | -0.128 | 0.341 | 0.375 |
| Prevotella salivae | 2 | Not detected | 0.031 | -0.059 | 0.121 | 0.495 |
| Prevotella scopos | 2 | Not detected | 0.029 | -0.092 | 0.151 | 0.636 |
| Prevotella sp. 10(H) | 44 | Not detected | 0.077 | -0.075 | 0.228 | 0.321 |
| Prevotella sp. 109 | 2 | Not detected | -0.066 | -0.152 | 0.020 | 0.131 |
| Prevotella sp. AGR2160 | 0 | Not detected | 0.194 | -0.080 | 0.468 | 0.164 |
| Prevotella sp. BP1-148 | 2 | Not detected | 0.019 | -0.108 | 0.145 | 0.770 |
| Prevotella sp. CAG:1058 | 0 | Not detected | -0.148 | -0.374 | 0.078 | 0.200 |
| Prevotella sp. CAG:1092 | 2 | Not detected | 0.306 | 0.020 | 0.591 | 0.036 |
| Prevotella sp. CAG:1124 | 42 | Not detected | -0.258 | -0.489 | -0.027 | 0.028 |
| Prevotella sp. CAG:1185 | 0 | Not detected | 0.020 | -0.223 | 0.262 | 0.874 |
| Prevotella sp. CAG:1320 | 0 | Not detected | -0.143 | -0.327 | 0.042 | 0.130 |
| Prevotella sp. CAG:255 | 0 | Not detected | -0.034 | -0.183 | 0.115 | 0.658 |
| Prevotella sp. CAG:386 | 0 | Not detected | 0.288 | -0.027 | 0.603 | 0.074 |
| Prevotella sp. CAG:487 | 0 | Not detected | -0.009 | -0.161 | 0.142 | 0.904 |
| Prevotella sp. CAG:520 | 0 | Not detected | 0.235 | -0.025 | 0.494 | 0.077 |
| Prevotella sp. CAG:604 | 0 | Not detected | 0.329 | -0.010 | 0.669 | 0.058 |
| Prevotella sp. CAG:732 | 780 | 0.6 | 0.408 | 0.102 | 0.713 | 0.009 |
| Prevotella sp. CAG:891 | 2 | Not detected | 0.217 | -0.003 | 0.437 | 0.053 |
| Prevotella sp. FD3004 | 60 | Not detected | 0.208 | 0.014 | 0.402 | 0.036 |
| Prevotella sp. HUN102 | 45 | Not detected | 0.078 | -0.049 | 0.204 | 0.230 |

| Prevotella sp. MSX73 | 2 | Not detected | 0.157 | -0.064 | 0.377 | 0.164 |
| --- | --- | --- | --- | --- | --- | --- |
| Prevotella sp. P4-76 | 2 | Not detected | 0.149 | -0.012 | 0.309 | 0.069 |
| Prevotella sp. P6B1 | 58 | Not detected | 0.166 | 0.032 | 0.301 | 0.016 |
| Prevotella sp. P6B4 | 7 | Not detected | 0.124 | -0.008 | 0.256 | 0.065 |
| Prevotella sp. RM4 | 3 | Not detected | 0.125 | -0.040 | 0.290 | 0.139 |
| Prevotella sp. S7 MS 2 | 2 | Not detected | 0.028 | -0.058 | 0.114 | 0.519 |
| Prevotella sp. S7-1-8 | 2 | Not detected | 0.042 | -0.039 | 0.123 | 0.305 |
| Prevotella sp. kh1p2 | 2 | Not detected | 0.163 | -0.058 | 0.383 | 0.149 |
| Prevotella sp. khp1 | 7 | Not detected | 0.164 | -0.005 | 0.333 | 0.058 |
| Prevotella sp. ne3005 | 3 | Not detected | 0.085 | -0.063 | 0.233 | 0.259 |
| Prevotella sp. oral taxon 473 | 1 | Not detected | 0.137 | -0.084 | 0.357 | 0.224 |
| Prevotella stercorea | 2 | Not detected | 0.071 | -0.049 | 0.192 | 0.246 |
| Prevotella stercorea CAG:629 | 2 | Not detected | 0.327 | 0.057 | 0.598 | 0.018 |
| Prevotella timonensis | 2 | Not detected | 0.012 | -0.073 | 0.096 | 0.783 |
| Prevotellamassilia timonensis | 234 | Not detected | 0.177 | 0.035 | 0.318 | 0.015 |
| Propionibacterium acidifaciens | 0 | Not detected | -0.020 | -0.198 | 0.159 | 0.829 |
| Proteiniclasticum ruminis | 51 | Not detected | -0.144 | -0.294 | 0.007 | 0.062 |
| Proteiniphilum acetatigenes | 247 | Not detected | -0.153 | -0.309 | 0.003 | 0.054 |
| Proteiniphilum saccharofermentans | 351 | Not detected | -0.150 | -0.322 | 0.022 | 0.087 |
| Proteiniphilum sp. EBM-41 | 2 | Not detected | -0.010 | -0.225 | 0.205 | 0.927 |
| Proteobacteria bacterium CAG:139 | 0 | Not detected | -0.137 | -0.478 | 0.204 | 0.431 |
| Proteobacteria bacterium CAG:495 | 0 | Not detected | 0.041 | -0.250 | 0.333 | 0.782 |
| Providencia rettgeri | 2 | Not detected | 0.077 | -0.110 | 0.263 | 0.420 |
| Pseudobacteroides cellulosolvens | 19 | Not detected | 0.049 | -0.090 | 0.187 | 0.491 |
| Pseudobutyrivibrio ruminis | 54 | Not detected | 0.055 | -0.015 | 0.125 | 0.125 |
| Pseudobutyrivibrio sp. ACV-2 | 367 | Not detected | 0.266 | 0.107 | 0.425 | 0.001 |
| Pseudobutyrivibrio sp. JW11 | 71 | Not detected | 0.135 | -0.021 | 0.291 | 0.090 |
| Pseudobutyrivibrio sp. MD2005 | 21 | Not detected | 0.035 | -0.132 | 0.202 | 0.680 |
| Pseudobutyrivibrio sp. UC1225 | 356 | Not detected | 0.123 | 0.017 | 0.229 | 0.023 |
| Pseudobutyrivibrio sp. YE44 | 15 | Not detected | 0.043 | -0.113 | 0.200 | 0.587 |
| Pseudobutyrivibrio xylanivorans | 94 | Not detected | 0.142 | -0.019 | 0.302 | 0.084 |
| Pseudoflavonifractor capillosus | 15 | Not detected | -0.019 | -0.093 | 0.055 | 0.617 |
| Pseudopropionibacterium propionicum | 0 | Not detected | -0.106 | -0.287 | 0.074 | 0.249 |
| Pyramidobacter piscolens | 0 | Not detected | 0.109 | -0.128 | 0.345 | 0.369 |
| Rahnella aquatilis | 3 | Not detected | 0.020 | -0.128 | 0.168 | 0.793 |
| Ralstonia mannitolilytica | 0 | Not detected | -0.159 | -0.367 | 0.048 | 0.132 |
| Ralstonia pickettii | 2 | Not detected | 0.054 | -0.141 | 0.249 | 0.587 |
| Raoultella ornithinolytica | 0 | Not detected | 0.156 | -0.129 | 0.440 | 0.284 |
| Rappaport israeli | 2 | Not detected | 0.023 | -0.135 | 0.181 | 0.776 |
| Rhodococcus defluvii | 45 | Not detected | 0.081 | -0.072 | 0.234 | 0.298 |
| Rhodopseudomonas palustris | 5 | Not detected | 0.074 | -0.098 | 0.245 | 0.398 |
| Rhodospirillum sp. UNK.MGS-17 | 0 | Not detected | -0.137 | -0.463 | 0.189 | 0.410 |
| Riemerella columbina | 35 | Not detected | -0.069 | -0.149 | 0.011 | 0.090 |
| Rikenella microfusus | 10 | Not detected | -0.061 | -0.142 | 0.021 | 0.143 |

| Risungbinella massiliensis | 5 | Not detected | -0.005 | -0.140 | 0.131 | 0.947 |
| --- | --- | --- | --- | --- | --- | --- |
| Robinsoniella peoriensis | 62 | Not detected | 0.042 | -0.016 | 0.099 | 0.155 |
| Rodentibacter pneumotropicus | 7 | Not detected | 0.068 | -0.073 | 0.208 | 0.346 |
| Roseburia hominis | 2 | Not detected | 0.019 | -0.062 | 0.101 | 0.642 |
| Roseburia intestinalis | 324 | Not detected | 0.158 | 0.035 | 0.281 | 0.012 |
| Roseburia inulinivorans | 352 | Not detected | 0.138 | 0.037 | 0.238 | 0.007 |
| Roseburia sp. CAG:197_41_10 | 169 | Not detected | 0.125 | 0.023 | 0.228 | 0.017 |
| Rothia dentocariosa | 1 | Not detected | 0.026 | -0.195 | 0.247 | 0.820 |
| Rothia mucilaginosa | 0 | Not detected | 0.110 | -0.121 | 0.342 | 0.349 |
| Ruminococcaceae bacterium AB4001 | 3 | Not detected | -0.031 | -0.172 | 0.110 | 0.664 |
| Ruminococcaceae bacterium AE2021 | 10 | Not detected | 0.022 | -0.115 | 0.160 | 0.750 |
| Ruminococcaceae bacterium D16 | 12 | Not detected | -0.021 | -0.090 | 0.048 | 0.548 |
| Ruminococcaceae bacterium YRB3002 | 3 | Not detected | 0.034 | -0.132 | 0.201 | 0.686 |
| Ruminococcus albus | 36 | Not detected | 0.087 | -0.053 | 0.228 | 0.224 |
| Ruminococcus bicirculans | 0 | Not detected | 0.071 | -0.139 | 0.281 | 0.506 |
| Ruminococcus callidus | 67 | Not detected | 0.154 | 0.012 | 0.297 | 0.034 |
| Ruminococcus champanellensis | 2 | Not detected | 0.187 | 0.011 | 0.364 | 0.038 |
| Ruminococcus faecis | 2 | Not detected | 0.010 | -0.084 | 0.103 | 0.837 |
| Ruminococcus flavefaciens | 48 | Not detected | 0.113 | -0.017 | 0.242 | 0.089 |
| Ruminococcus gauvreauii | 61 | Not detected | 0.056 | -0.019 | 0.131 | 0.144 |
| Ruminococcus lactaris | 0 | Not detected | -0.034 | -0.141 | 0.073 | 0.534 |
| Ruminococcus obeum CAG:39 | 9 | Not detected | -0.069 | -0.192 | 0.054 | 0.274 |
| Ruminococcus sp. AT10 | 694 | Not detected | -0.131 | -0.199 | -0.063 | 0.000 |
| Ruminococcus sp. CAG:108-related_41_ | 0 | Not detected | 0.033 | -0.189 | 0.255 | 0.770 |
| Ruminococcus sp. CAG:177 | 0 | Not detected | 0.365 | 0.020 | 0.709 | 0.038 |
| Ruminococcus sp. CAG:330 | 2 | Not detected | 0.296 | -0.019 | 0.610 | 0.066 |
| Ruminococcus sp. CAG:379 | 682 | Not detected | 0.308 | 0.074 | 0.542 | 0.010 |
| Ruminococcus sp. CAG:382 | 0 | Not detected | 0.146 | -0.152 | 0.444 | 0.338 |
| Ruminococcus sp. CAG:403 | 2 | Not detected | 0.230 | -0.021 | 0.481 | 0.073 |
| Ruminococcus sp. CAG:488 | 2 | Not detected | 0.129 | -0.078 | 0.336 | 0.223 |
| Ruminococcus sp. CAG:563 | 78 | Not detected | 0.328 | 0.099 | 0.556 | 0.005 |
| Ruminococcus sp. CAG:60 | 6 | Not detected | 0.093 | -0.039 | 0.225 | 0.168 |
| Ruminococcus sp. CAG:624 | 0 | Not detected | 0.048 | -0.181 | 0.277 | 0.680 |
| Ruminococcus sp. CAG:724 | 0 | Not detected | 0.094 | -0.160 | 0.348 | 0.469 |
| Ruminococcus sp. FC2018 | 2 | Not detected | 0.049 | -0.102 | 0.200 | 0.528 |
| Ruminococcus sp. HUN007 | 14 | Not detected | 0.012 | -0.112 | 0.135 | 0.854 |
| Ruminococcus sp. NK3A76 | 7 | Not detected | 0.089 | -0.064 | 0.242 | 0.253 |
| Ruminococcus sp. Phil15 | 0 | Not detected | -0.043 | -0.244 | 0.158 | 0.674 |
| Ruminococcus sp. Zagget7 | 1 | Not detected | -0.102 | -0.217 | 0.012 | 0.079 |
| Ruthenibacterium lactatiformans | 361 | Not detected | -0.123 | -0.221 | -0.024 | 0.015 |
| Salmonella enterica | 0 | Not detected | -0.006 | -0.257 | 0.244 | 0.960 |
| Scardovia inopinata | 1 | Not detected | -0.075 | -0.226 | 0.075 | 0.326 |
| Scardovia wiggsiae | 0 | Not detected | 0.005 | -0.192 | 0.202 | 0.958 |
| Selenomonas bovis | 3 | Not detected | 0.032 | -0.096 | 0.161 | 0.624 |

| Selenomonas noxia | 3 | Not detected | 0.074 | -0.076 | 0.224 | 0.335 |
| --- | --- | --- | --- | --- | --- | --- |
| Selenomonas ruminantium | 25 | Not detected | 0.160 | -0.024 | 0.344 | 0.088 |
| Selenomonas sputigena | 8 | Not detected | 0.090 | -0.069 | 0.249 | 0.267 |
| Sellimonas intestinalis | 43 | Not detected | -0.095 | -0.195 | 0.006 | 0.064 |
| Senegalimassilia anaerobia | 2 | Not detected | 0.132 | -0.049 | 0.313 | 0.152 |
| Serratia fonticola | 27 | Not detected | 0.093 | -0.050 | 0.235 | 0.204 |
| Serratia liquefaciens | 3 | Not detected | 0.046 | -0.105 | 0.198 | 0.550 |
| Serratia marcescens | 7 | Not detected | 0.044 | -0.121 | 0.209 | 0.602 |
| Serratia odorifera | 3 | Not detected | 0.030 | -0.127 | 0.186 | 0.710 |
| Serratia rubidaea | 1 | Not detected | -0.026 | -0.187 | 0.135 | 0.750 |
| Shigella dysenteriae | 0 | Not detected | -0.051 | -0.305 | 0.202 | 0.692 |
| Shigella flexneri | 0 | Not detected | -0.052 | -0.305 | 0.200 | 0.686 |
| Shimwellia blattae | 0 | Not detected | -0.103 | -0.312 | 0.107 | 0.338 |
| Shuttleworthia satelles | 41 | Not detected | 0.061 | -0.035 | 0.158 | 0.214 |
| Siccibacter turicensis | 0 | Not detected | 0.015 | -0.207 | 0.236 | 0.897 |
| Slackia exigua | 2 | Not detected | -0.013 | -0.178 | 0.152 | 0.875 |
| Slackia heliotrinireducens | 3 | Not detected | -0.005 | -0.151 | 0.141 | 0.948 |
| Slackia piriformis | 0 | Not detected | -0.009 | -0.254 | 0.236 | 0.943 |
| Solobacterium moorei | 2 | Not detected | 0.013 | -0.086 | 0.112 | 0.798 |
| Sphaerochaeta sp. Phil3 | 59 | Not detected | 0.149 | -0.029 | 0.326 | 0.102 |
| Sphingobacterium wenxiniae | 269 | Not detected | -0.242 | -0.421 | -0.063 | 0.008 |
| Spirochaetales bacterium Spiro_07 | 55 | Not detected | 0.093 | -0.053 | 0.238 | 0.211 |
| Sporobacter termitidis | 2 | Not detected | 0.069 | -0.074 | 0.212 | 0.347 |
| Sporomusa ovata | 26 | Not detected | 0.112 | -0.052 | 0.276 | 0.181 |
| Staphylococcus pseudintermedius | 3 | Not detected | 0.034 | -0.135 | 0.203 | 0.693 |
| Staphylococcus sp. CAG:324 | 0 | Not detected | -0.064 | -0.370 | 0.243 | 0.684 |
| Staphylococcus sp. DORA_6_22 | 0 | Not detected | -0.064 | -0.229 | 0.101 | 0.445 |
| Staphylococcus warneri | 3 | Not detected | -0.051 | -0.234 | 0.131 | 0.581 |
| Stomatobaculum longum | 63 | Not detected | 0.046 | -0.009 | 0.101 | 0.100 |
| Streptobacillus hongkongensis | 2 | Not detected | -0.030 | -0.190 | 0.130 | 0.716 |
| Streptobacillus notomytis | 0 | Not detected | -0.114 | -0.293 | 0.066 | 0.215 |
| Streptococcus agalactiae | 0 | Not detected | -0.023 | -0.208 | 0.162 | 0.808 |
| Streptococcus anginosus | 0 | Not detected | -0.062 | -0.268 | 0.145 | 0.560 |
| Streptococcus caballi | 1 | Not detected | -0.124 | -0.314 | 0.066 | 0.200 |
| Streptococcus cristatus | 0 | Not detected | -0.012 | -0.174 | 0.151 | 0.887 |
| Streptococcus dysgalactiae | 0 | Not detected | -0.077 | -0.264 | 0.109 | 0.418 |
| Streptococcus entericus | 28 | Not detected | -0.202 | -0.353 | -0.051 | 0.009 |
| Streptococcus gallolyticus | 0 | Not detected | -0.018 | -0.246 | 0.209 | 0.875 |
| Streptococcus gordonii | 11 | Not detected | 0.232 | 0.015 | 0.450 | 0.036 |
| Streptococcus halotolerans | 1 | Not detected | -0.027 | -0.180 | 0.127 | 0.733 |
| Streptococcus henryi | 0 | Not detected | -0.111 | -0.293 | 0.072 | 0.234 |
| Streptococcus ictaluri | 0 | Not detected | -0.048 | -0.210 | 0.113 | 0.557 |
| Streptococcus infantis | 2 | Not detected | 0.065 | -0.097 | 0.228 | 0.431 |
| Streptococcus iniae | 2 | Not detected | -0.121 | -0.285 | 0.044 | 0.152 |

| Streptococcus intermedius | 0 | Not detected | -0.020 | -0.230 | 0.191 | 0.854 |
| --- | --- | --- | --- | --- | --- | --- |
| Streptococcus massiliensis | 0 | Not detected | -0.080 | -0.250 | 0.090 | 0.355 |
| Streptococcus minor | 4 | Not detected | -0.044 | -0.172 | 0.083 | 0.495 |
| Streptococcus mitis | 0 | Not detected | -0.084 | -0.206 | 0.039 | 0.182 |
| Streptococcus mutans | 0 | Not detected | 0.127 | -0.122 | 0.376 | 0.318 |
| Streptococcus oralis | 4 | Not detected | 0.118 | -0.074 | 0.309 | 0.228 |
| Streptococcus orisratti | 8 | Not detected | -0.119 | -0.300 | 0.063 | 0.200 |
| Streptococcus ovis | 1 | Not detected | -0.037 | -0.204 | 0.130 | 0.665 |
| Streptococcus parasanguinis | 2 | Not detected | 0.148 | -0.048 | 0.343 | 0.140 |
| Streptococcus parauberis | 2 | Not detected | -0.144 | -0.313 | 0.025 | 0.094 |
| Streptococcus phocae | 4 | Not detected | -0.072 | -0.198 | 0.055 | 0.266 |
| Streptococcus plurextorum | 9 | Not detected | -0.017 | -0.101 | 0.067 | 0.690 |
| Streptococcus porci | 5 | Not detected | -0.019 | -0.120 | 0.082 | 0.716 |
| Streptococcus pseudoporcinus | 2 | Not detected | -0.102 | -0.283 | 0.079 | 0.271 |
| Streptococcus pyogenes | 0 | Not detected | -0.026 | -0.211 | 0.160 | 0.787 |
| Streptococcus salivarius | 237 | Not detected | 0.224 | 0.044 | 0.403 | 0.015 |
| Streptococcus sanguinis | 8 | Not detected | 0.140 | -0.072 | 0.353 | 0.196 |
| Streptococcus sobrinus | 65 | Not detected | -0.125 | -0.319 | 0.069 | 0.208 |
| Streptococcus sp. X13SY08 | 1 | Not detected | -0.015 | -0.147 | 0.116 | 0.820 |
| Streptococcus suis | 3 | Not detected | -0.079 | -0.167 | 0.008 | 0.076 |
| Streptococcus thermophilus | 0 | Not detected | -0.064 | -0.251 | 0.124 | 0.507 |
| Streptococcus uberis | 2 | Not detected | -0.145 | -0.307 | 0.016 | 0.078 |
| Streptococcus urinalis | 0 | Not detected | -0.025 | -0.209 | 0.160 | 0.794 |
| Streptomyces albulus | 8 | Not detected | 0.040 | -0.111 | 0.192 | 0.601 |
| Streptomyces cattleya | 0 | Not detected | -0.041 | -0.216 | 0.134 | 0.646 |
| Streptomyces kanamyceticus | 3 | Not detected | 0.018 | -0.097 | 0.133 | 0.763 |
| Subdoligranulum sp. CAG:314 | 0 | Not detected | 0.051 | -0.303 | 0.405 | 0.777 |
| Subdoligranulum variabile | 5 | Not detected | 0.005 | -0.062 | 0.072 | 0.878 |
| Succinatimonas sp. CAG:777 | 0 | Not detected | 0.222 | -0.069 | 0.514 | 0.135 |
| Succinispira mobilis | 1 | Not detected | 0.002 | -0.171 | 0.175 | 0.979 |
| Succinivibrio sp. Phil9 | 2 | Not detected | 0.174 | -0.104 | 0.451 | 0.221 |
| Sulfolobus acidocaldarius | 1 | Not detected | -0.224 | -0.443 | -0.004 | 0.046 |
| Sutterella parvirubra | 365 | Not detected | 0.258 | 0.068 | 0.448 | 0.008 |
| Sutterella sp. CAG:397 | 2 | Not detected | 0.360 | -0.012 | 0.731 | 0.058 |
| Sutterella sp. CAG:521 | 0 | Not detected | -0.022 | -0.257 | 0.213 | 0.853 |
| Sutterella wadsworthensis CAG:135 | 1 | Not detected | 0.431 | 0.049 | 0.813 | 0.027 |
| Sutterellaceae bacterium ND3 | 0 | Not detected | -0.094 | -0.326 | 0.138 | 0.428 |
| Synergistales bacterium Syner_02 | 0 | Not detected | -0.017 | -0.215 | 0.182 | 0.869 |
| Synergistes sp. 3_1_syn1 | 0 | Not detected | -0.110 | -0.361 | 0.141 | 0.391 |
| Synergistes sp. Zagget9 | 161 | Not detected | -0.168 | -0.324 | -0.011 | 0.036 |
| Tannerella forsythia | 2 | Not detected | 0.010 | -0.106 | 0.126 | 0.862 |
| Tatumella morbirosei | 3 | Not detected | 0.019 | -0.130 | 0.168 | 0.802 |
| Tepidanaerobacter sp. EBM-49 | 0 | Not detected | -0.002 | -0.244 | 0.239 | 0.985 |
| Terrisporobacter glycolicus | 4 | Not detected | 0.076 | -0.130 | 0.282 | 0.469 |

| Thermophagus xiamenensis | 2 | Not detected | -0.006 | -0.192 | 0.180 | 0.951 |
| --- | --- | --- | --- | --- | --- | --- |
| Thermosinus carboxydivorans | 12 | Not detected | 0.120 | -0.021 | 0.260 | 0.094 |
| Tidjanibacter massiliensis | 0 | Not detected | -0.205 | -0.450 | 0.039 | 0.100 |
| Tissierellia bacterium S7-1-4 | 12 | Not detected | -0.168 | -0.356 | 0.020 | 0.079 |
| Trabulsiella odontotermitis | 0 | Not detected | 0.016 | -0.216 | 0.248 | 0.891 |
| Traorella massiliensis | 22 | Not detected | 0.027 | -0.039 | 0.093 | 0.427 |
| Treponema denticola | 207 | Not detected | 0.149 | 0.013 | 0.286 | 0.032 |
| Treponema medium | 597 | Not detected | -0.316 | -0.542 | -0.090 | 0.006 |
| Treponema putidum | 179 | Not detected | 0.125 | 0.004 | 0.245 | 0.043 |
| Treponema socranskii | 68 | Not detected | 0.053 | -0.086 | 0.192 | 0.454 |
| Treponema succinifaciens | 4 | Not detected | 0.018 | -0.059 | 0.096 | 0.643 |
| Treponema vincentii | 116 | Not detected | 0.097 | -0.029 | 0.224 | 0.131 |
| Trichococcus flocculiformis | 51 | Not detected | 0.084 | -0.067 | 0.235 | 0.277 |
| Trueperella pyogenes | 2 | Not detected | 0.032 | -0.122 | 0.186 | 0.685 |
| Tumebacillus flagellatus | 1 | Not detected | -0.087 | -0.238 | 0.065 | 0.261 |
| Turicibacter sanguinis | 0 | Not detected | -0.039 | -0.308 | 0.230 | 0.776 |
| Turicibacter sp. H121 | 0 | Not detected | -0.061 | -0.166 | 0.044 | 0.253 |
| Tyzzerella nexilis | 135 | Not detected | -0.067 | -0.128 | -0.006 | 0.032 |
| Urmitella timonensis | 20 | Not detected | 0.063 | -0.054 | 0.181 | 0.290 |
| Vaginella massiliensis | 0 | Not detected | -0.020 | -0.245 | 0.206 | 0.865 |
| Varibaculum cambriense | 28 | Not detected | 0.027 | -0.067 | 0.120 | 0.574 |
| Varibaculum sp. Marseille-P2802 | 48 | Not detected | 0.097 | -0.054 | 0.247 | 0.208 |
| Veillonella magna | 63 | Not detected | 0.049 | -0.019 | 0.117 | 0.157 |
| Veillonella parvula | 7 | Not detected | 0.262 | 0.036 | 0.488 | 0.023 |
| Veillonella seminalis | 286 | Not detected | 0.124 | 0.046 | 0.202 | 0.002 |
| Veillonella sp. ACP1 | 0 | Not detected | 0.111 | -0.161 | 0.383 | 0.424 |
| Veillonella sp. CAG:933 | 2 | Not detected | 0.056 | -0.076 | 0.189 | 0.402 |
| Veillonella sp. oral taxon 780 | 3 | Not detected | 0.025 | -0.145 | 0.195 | 0.770 |
| Waddlia chondrophila | 91 | Not detected | -0.276 | -0.489 | -0.063 | 0.011 |
| Weissella cibaria | 8 | Not detected | 0.177 | -0.033 | 0.388 | 0.099 |
| Weissella confusa | 71 | Not detected | 0.202 | -0.038 | 0.443 | 0.100 |
| Xanthomonas sp. Mitacek01 | 3 | Not detected | 0.020 | -0.105 | 0.146 | 0.749 |
| Yersinia enterocolitica | 2 | Not detected | -0.035 | -0.236 | 0.166 | 0.736 |
| Yersinia pestis | 0 | Not detected | -0.060 | -0.318 | 0.197 | 0.646 |
| Yokenella regensburgei | 0 | Not detected | 0.052 | -0.177 | 0.280 | 0.657 |
| [Bacteroides] pectinophilus | 141 | Not detected | 0.082 | 0.011 | 0.154 | 0.025 |
| [Clostridium] aminophilum | 57 | Not detected | 0.054 | -0.010 | 0.119 | 0.099 |
| [Clostridium] asparagiforme | 107 | Not detected | -0.095 | -0.188 | -0.001 | 0.048 |
| [Clostridium] bolteae | 813 | 0.6 | -0.164 | -0.250 | -0.077 | 0.000 |
| [Clostridium] celerecrescens | 30 | Not detected | -0.016 | -0.075 | 0.043 | 0.598 |
| [Clostridium] cellobioparum | 30 | Not detected | -0.084 | -0.221 | 0.053 | 0.230 |
| [Clostridium] cellulolyticum | 15 | Not detected | -0.110 | -0.267 | 0.046 | 0.167 |
| [Clostridium] cellulosi | 9 | Not detected | -0.050 | -0.206 | 0.106 | 0.529 |
| [Clostridium] citroniae | 711 | 0.6 | -0.125 | -0.194 | -0.056 | 0.000 |

| [Clostridium] clariflavum | 63 | Not detected | 0.089 | -0.047 | 0.224 | 0.199 |
| --- | --- | --- | --- | --- | --- | --- |
| [Clostridium] clostridioforme | 676 | Not detected | -0.140 | -0.233 | -0.046 | 0.003 |
| [Clostridium] dakarense | 2 | Not detected | 0.075 | -0.132 | 0.283 | 0.477 |
| [Clostridium] glycyrrhizinilyticum | 70 | Not detected | -0.049 | -0.109 | 0.012 | 0.116 |
| [Clostridium] hiranonis | 43 | Not detected | -0.207 | -0.397 | -0.018 | 0.032 |
| [Clostridium] hylemonae | 105 | Not detected | 0.097 | -0.038 | 0.232 | 0.161 |
| [Clostridium] innocuum | 553 | Not detected | -0.107 | -0.171 | -0.042 | 0.001 |
| [Clostridium] josui | 2 | Not detected | -0.134 | -0.294 | 0.025 | 0.098 |
| [Clostridium] lactatifermentans | 189 | Not detected | -0.090 | -0.170 | -0.010 | 0.028 |
| [Clostridium] leptum | 13 | Not detected | -0.076 | -0.180 | 0.027 | 0.147 |
| [Clostridium] methylpentosum | 38 | Not detected | 0.067 | -0.039 | 0.172 | 0.215 |
| [Clostridium] papyrosolvens | 24 | Not detected | 0.049 | -0.071 | 0.169 | 0.426 |
| [Clostridium] saccharogumia | 1 | Not detected | -0.035 | -0.127 | 0.058 | 0.460 |
| [Clostridium] saccharolyticum | 44 | Not detected | 0.074 | -0.011 | 0.160 | 0.090 |
| [Clostridium] scindens | 9 | Not detected | -0.055 | -0.130 | 0.020 | 0.154 |
| [Clostridium] sporosphaeroides | 56 | Not detected | 0.072 | -0.066 | 0.211 | 0.308 |
| [Clostridium] symbiosum | 20 | Not detected | -0.053 | -0.129 | 0.022 | 0.165 |
| [Clostridium] viride | 4 | Not detected | -0.011 | -0.144 | 0.121 | 0.865 |
| [Enterobacter] aerogenes | 0 | Not detected | 0.125 | -0.164 | 0.415 | 0.397 |
| [Eubacterium] brachy | 2 | Not detected | 0.065 | -0.136 | 0.266 | 0.525 |
| [Eubacterium] cellulosolvens | 85 | Not detected | 0.083 | 0.000 | 0.165 | 0.051 |
| [Eubacterium] contortum | 37 | Not detected | -0.041 | -0.097 | 0.016 | 0.163 |
| [Eubacterium] dolichum | 27 | Not detected | -0.040 | -0.099 | 0.019 | 0.187 |
| [Eubacterium] fissicatena | 27 | Not detected | -0.074 | -0.151 | 0.003 | 0.059 |
| [Eubacterium] hallii | 5 | Not detected | 0.048 | -0.052 | 0.149 | 0.347 |
| [Eubacterium] nodatum | 2 | Not detected | -0.038 | -0.220 | 0.145 | 0.685 |
| [Eubacterium] rectale | 2 | Not detected | 0.060 | -0.086 | 0.205 | 0.421 |
| [Eubacterium] siraeum | 0 | Not detected | 0.022 | -0.202 | 0.247 | 0.845 |
| [Eubacterium] sulci | 1 | Not detected | -0.120 | -0.319 | 0.078 | 0.235 |
| [Eubacterium] yurii | 5 | Not detected | -0.031 | -0.146 | 0.084 | 0.596 |
| [Haemophilus] ducreyi | 3 | Not detected | 0.055 | -0.087 | 0.197 | 0.445 |
| [Haemophilus] parasuis | 87 | Not detected | 0.187 | 0.016 | 0.358 | 0.032 |
| [Ruminococcus] gnavus | 609 | Not detected | -0.156 | -0.271 | -0.041 | 0.008 |
| bacterium F082 | 2 | Not detected | 0.002 | -0.097 | 0.102 | 0.962 |
| bacterium LF-3 | 2 | Not detected | 0.081 | -0.071 | 0.233 | 0.296 |
| bacterium MS4 | 22 | Not detected | 0.018 | -0.111 | 0.147 | 0.785 |
| bacterium OL-1 | 2 | Not detected | 0.142 | -0.099 | 0.384 | 0.249 |
| bacterium P201 | 2 | Not detected | 0.106 | -0.049 | 0.261 | 0.179 |
| bacterium P3 | 80 | Not detected | 0.241 | 0.060 | 0.422 | 0.009 |

| **Supplementary Table 2.** Association of current SEP (higher SEP vs. Lower SEP) with species in overall participants (n=2174). Analysis of Compositions of Microbiomes (ANCOM2) was used to identify species related to current SEP. Regression coefficients were obtained by modeling the effect of current SEP on centered log ratio (CLR)-transformed species in multivariable linear regression adjusted for age, gender, BMI, field center, Hispanic/Latino background, U.S. nativity, Bristol stool type, parental education, the alternative Healthy Eating Index 2010 score, cigarette smoking, alcohol use, depressive and anxiety symptoms, diabetes, hypertension, cardiovascular disease, dyslipidemia, and antibiotic use. | | | | | | |
| --- | --- | --- | --- | --- | --- | --- |
| **species** | **W_stat** | **ANCOM** | **beta** | **l95ci** | **u95ci** | **qval** |
| Bacteroides sp. CAG:98 | 1020 | 0.8 | -0.339 | -0.584 | -0.095 | 0.007 |
| Bacteroides stercoris | 930 | 0.7 | -0.258 | -0.431 | -0.084 | 0.004 |
| Bifidobacterium dentium | 990 | 0.8 | 0.448 | 0.188 | 0.708 | 0.001 |
| Clostridium sp. CAG:138 | 1054 | 0.8 | 0.485 | 0.148 | 0.822 | 0.005 |
| [Eubacterium] sulci | 862 | 0.7 | -0.238 | -0.452 | -0.024 | 0.029 |
| Abiotrophia defectiva | 0 | Not detected | -0.081 | -0.275 | 0.112 | 0.411 |
| Acetanaerobacterium elongatum | 0 | Not detected | 0.172 | 0.037 | 0.308 | 0.013 |
| Acetitomaculum ruminis | 0 | Not detected | 0.063 | -0.015 | 0.140 | 0.114 |
| Acetivibrio cellulolyticus | 0 | Not detected | 0.073 | -0.085 | 0.231 | 0.364 |
| Acetivibrio ethanolgignens | 0 | Not detected | 0.010 | -0.058 | 0.078 | 0.778 |
| Acetobacter sp. 46_36 | 0 | Not detected | 0.051 | -0.251 | 0.354 | 0.739 |
| Acetobacter sp. CAG:267 | 0 | Not detected | -0.302 | -0.576 | -0.027 | 0.031 |
| Acholeplasma axanthum | 0 | Not detected | 0.165 | 0.027 | 0.302 | 0.019 |
| Acholeplasma equifetale | 0 | Not detected | 0.053 | -0.132 | 0.239 | 0.574 |
| Acidaminococcus fermentans | 0 | Not detected | -0.002 | -0.136 | 0.133 | 0.980 |
| Acidaminococcus intestini | 0 | Not detected | -0.128 | -0.369 | 0.114 | 0.301 |
| Acidaminococcus intestini CAG:325 | 0 | Not detected | -0.362 | -0.742 | 0.017 | 0.062 |
| Acinetobacter baumannii | 0 | Not detected | 0.007 | -0.150 | 0.164 | 0.929 |
| Acinetobacter bereziniae | 0 | Not detected | 0.105 | -0.054 | 0.264 | 0.196 |
| Acinetobacter johnsonii | 0 | Not detected | 0.101 | -0.071 | 0.274 | 0.250 |
| Acinetobacter sp. CAG:196 | 0 | Not detected | 0.025 | -0.301 | 0.351 | 0.882 |
| Acinetobacter sp. CAG:196_36_41 | 0 | Not detected | 0.026 | -0.290 | 0.342 | 0.873 |
| Acinetobacter sp. N54.MGS-139 | 0 | Not detected | -0.070 | -0.238 | 0.098 | 0.415 |
| Acinetobacter venetianus | 0 | Not detected | 0.152 | -0.036 | 0.340 | 0.114 |
| Actinobaculum sp. oral taxon 183 | 0 | Not detected | -0.112 | -0.336 | 0.113 | 0.329 |
| Actinobaculum suis | 0 | Not detected | -0.017 | -0.181 | 0.147 | 0.837 |
| Actinomyces dentalis | 0 | Not detected | 0.205 | -0.027 | 0.438 | 0.084 |
| Actinomyces gerencseriae | 0 | Not detected | 0.115 | -0.096 | 0.326 | 0.285 |
| Actinomyces graevenitzii | 0 | Not detected | 0.038 | -0.115 | 0.190 | 0.628 |
| Actinomyces hongkongensis | 0 | Not detected | -0.051 | -0.260 | 0.157 | 0.629 |
| Actinomyces israelii | 0 | Not detected | 0.143 | -0.078 | 0.363 | 0.205 |
| Actinomyces johnsonii | 0 | Not detected | -0.004 | -0.216 | 0.208 | 0.971 |
| Actinomyces massiliensis | 0 | Not detected | 0.024 | -0.193 | 0.240 | 0.830 |
| Actinomyces meyeri | 0 | Not detected | -0.084 | -0.268 | 0.100 | 0.370 |
| Actinomyces odontolyticus | 0 | Not detected | 0.063 | -0.098 | 0.224 | 0.442 |

| Actinomyces oris | 0 | Not detected | 0.123 | -0.073 | 0.320 | 0.220 |
| --- | --- | --- | --- | --- | --- | --- |
| Actinomyces sp. HMSC065F12 | 0 | Not detected | -0.053 | -0.178 | 0.072 | 0.406 |
| Actinomyces sp. HMSC06A08 | 0 | Not detected | -0.095 | -0.222 | 0.032 | 0.142 |
| Actinomyces sp. oral taxon 181 | 0 | Not detected | 0.072 | -0.136 | 0.280 | 0.500 |
| Actinomyces sp. oral taxon 448 | 0 | Not detected | 0.185 | -0.061 | 0.432 | 0.141 |
| Actinomyces sp. oral taxon 848 | 0 | Not detected | 0.059 | -0.105 | 0.224 | 0.479 |
| Actinomyces sp. ph3 | 0 | Not detected | 0.002 | -0.198 | 0.203 | 0.983 |
| Actinomyces timonensis | 0 | Not detected | 0.009 | -0.138 | 0.156 | 0.901 |
| Actinomyces turicensis | 0 | Not detected | 0.141 | -0.072 | 0.355 | 0.194 |
| Adlercreutzia equolifaciens | 0 | Not detected | 0.050 | -0.206 | 0.306 | 0.701 |
| Aeromonas salmonicida | 0 | Not detected | 0.075 | -0.131 | 0.281 | 0.477 |
| Aggregatibacter aphrophilus | 0 | Not detected | 0.033 | -0.195 | 0.261 | 0.776 |
| Akkermansia glycaniphila | 0 | Not detected | -0.051 | -0.260 | 0.158 | 0.632 |
| Akkermansia muciniphila | 0 | Not detected | 0.041 | -0.230 | 0.312 | 0.766 |
| Akkermansia muciniphila CAG:154 | 0 | Not detected | 0.023 | -0.332 | 0.378 | 0.899 |
| Akkermansia sp. 54_46 | 0 | Not detected | 0.004 | -0.366 | 0.373 | 0.984 |
| Akkermansia sp. CAG:344 | 0 | Not detected | 0.091 | -0.282 | 0.464 | 0.634 |
| Akkermansia sp. KLE1605 | 0 | Not detected | -0.177 | -0.495 | 0.142 | 0.276 |
| Akkermansia sp. KLE1797 | 0 | Not detected | -0.177 | -0.496 | 0.142 | 0.276 |
| Akkermansia sp. KLE1798 | 0 | Not detected | -0.173 | -0.491 | 0.146 | 0.288 |
| Akkermansia sp. Phil8 | 0 | Not detected | 0.014 | -0.351 | 0.380 | 0.939 |
| Akkermansia sp. UNK.MGS-1 | 0 | Not detected | -0.008 | -0.373 | 0.358 | 0.966 |
| Alistipes finegoldii | 0 | Not detected | -0.036 | -0.180 | 0.109 | 0.629 |
| Alistipes ihumii | 0 | Not detected | -0.023 | -0.271 | 0.225 | 0.856 |
| Alistipes indistinctus | 0 | Not detected | -0.065 | -0.407 | 0.278 | 0.712 |
| Alistipes inops | 0 | Not detected | 0.029 | -0.240 | 0.299 | 0.831 |
| Alistipes obesi | 0 | Not detected | -0.125 | -0.285 | 0.035 | 0.127 |
| Alistipes putredinis | 0 | Not detected | -0.124 | -0.331 | 0.082 | 0.238 |
| Alistipes senegalensis | 0 | Not detected | 0.041 | -0.089 | 0.170 | 0.539 |
| Alistipes shahii | 0 | Not detected | 0.014 | -0.131 | 0.158 | 0.853 |
| Alistipes sp. CAG:435 | 0 | Not detected | 0.194 | -0.148 | 0.536 | 0.266 |
| Alistipes sp. CAG:514 | 0 | Not detected | 0.227 | -0.081 | 0.535 | 0.149 |
| Alistipes sp. Zagget8 | 0 | Not detected | 0.055 | -0.159 | 0.269 | 0.613 |
| Alistipes timonensis | 0 | Not detected | 0.041 | -0.184 | 0.266 | 0.722 |
| Alkaliflexus imshenetskii | 0 | Not detected | -0.060 | -0.221 | 0.100 | 0.461 |
| Allisonella histaminiformans | 0 | Not detected | 0.169 | -0.154 | 0.492 | 0.306 |
| Allobaculum stercoricanis | 0 | Not detected | 0.028 | -0.050 | 0.105 | 0.482 |
| Allofustis seminis | 0 | Not detected | 0.228 | 0.046 | 0.410 | 0.014 |
| Alloprevotella rava | 0 | Not detected | 0.020 | -0.121 | 0.161 | 0.783 |
| Alloprevotella tannerae | 0 | Not detected | -0.032 | -0.131 | 0.067 | 0.526 |
| Alloscardovia criceti | 0 | Not detected | 0.131 | -0.047 | 0.309 | 0.151 |
| Alloscardovia omnicolens | 0 | Not detected | 0.026 | -0.166 | 0.217 | 0.794 |
| Amphibacillus xylanus | 0 | Not detected | 0.051 | -0.138 | 0.240 | 0.600 |
| Anaerobium acetethylicum | 0 | Not detected | 0.056 | -0.021 | 0.133 | 0.153 |

| Anaerococcus hydrogenalis | 0 | Not detected | -0.043 | -0.180 | 0.094 | 0.536 |
| --- | --- | --- | --- | --- | --- | --- |
| Anaerococcus lactolyticus | 0 | Not detected | -0.026 | -0.221 | 0.168 | 0.791 |
| Anaerococcus obesiensis | 0 | Not detected | -0.052 | -0.215 | 0.110 | 0.529 |
| Anaerocolumna aminovalerica | 0 | Not detected | -0.046 | -0.209 | 0.117 | 0.583 |
| Anaerocolumna xylanovorans | 0 | Not detected | -0.031 | -0.179 | 0.117 | 0.681 |
| Anaerofustis stercorihominis | 0 | Not detected | -0.031 | -0.271 | 0.208 | 0.797 |
| Anaeroglobus geminatus | 0 | Not detected | -0.086 | -0.282 | 0.110 | 0.390 |
| Anaeromassilibacillus senegalensis | 0 | Not detected | 0.057 | -0.079 | 0.193 | 0.413 |
| Anaerophaga thermohalophila | 0 | Not detected | 0.036 | -0.088 | 0.159 | 0.571 |
| Anaerosalibacter massiliensis | 0 | Not detected | 0.030 | -0.168 | 0.228 | 0.765 |
| Anaerosporobacter mobilis | 0 | Not detected | 0.037 | -0.051 | 0.125 | 0.406 |
| Anaerosporomusa subterranea | 0 | Not detected | -0.064 | -0.201 | 0.073 | 0.361 |
| Anaerostipes caccae | 0 | Not detected | 0.014 | -0.066 | 0.094 | 0.731 |
| Anaerostipes hadrus | 0 | Not detected | -0.064 | -0.200 | 0.072 | 0.356 |
| Anaerostipes sp. CAG:276 | 0 | Not detected | -0.226 | -0.507 | 0.054 | 0.114 |
| Anaerotruncus colihominis | 0 | Not detected | -0.007 | -0.095 | 0.082 | 0.882 |
| Anaerotruncus sp. CAG:390 | 0 | Not detected | 0.182 | -0.067 | 0.430 | 0.153 |
| Anaerotruncus sp. CAG:528 | 0 | Not detected | 0.184 | -0.018 | 0.386 | 0.075 |
| Anaerotruncus sp. G3(2012) | 0 | Not detected | -0.023 | -0.091 | 0.044 | 0.496 |
| Anaerovorax odorimutans | 0 | Not detected | -0.038 | -0.194 | 0.117 | 0.629 |
| Angelakisella massiliensis | 0 | Not detected | 0.013 | -0.062 | 0.089 | 0.729 |
| Aphanizomenon flos-aquae | 0 | Not detected | 0.090 | -0.049 | 0.230 | 0.204 |
| Aquabacterium parvum | 0 | Not detected | 0.180 | 0.013 | 0.346 | 0.035 |
| Arcanobacterium haemolyticum | 0 | Not detected | -0.111 | -0.280 | 0.058 | 0.199 |
| Asaccharospora irregularis | 0 | Not detected | 0.086 | -0.105 | 0.277 | 0.377 |
| Atlantibacter hermannii | 0 | Not detected | 0.121 | -0.122 | 0.363 | 0.331 |
| Atopobium deltae | 0 | Not detected | 0.065 | -0.118 | 0.249 | 0.485 |
| Atopobium minutum | 0 | Not detected | 0.076 | -0.109 | 0.261 | 0.420 |
| Atopobium parvulum | 0 | Not detected | 0.071 | -0.137 | 0.279 | 0.505 |
| Atopobium rimae | 0 | Not detected | 0.052 | -0.119 | 0.222 | 0.554 |
| Atopobium sp. BS2 | 0 | Not detected | 0.004 | -0.198 | 0.207 | 0.966 |
| Atopobium sp. HMSC064B08 | 0 | Not detected | 0.054 | -0.094 | 0.202 | 0.474 |
| Atopobium sp. ICM42b | 0 | Not detected | 0.025 | -0.179 | 0.229 | 0.810 |
| Atopobium vaginae | 0 | Not detected | 0.149 | -0.042 | 0.339 | 0.126 |
| Atopostipes suicloacalis | 1 | Not detected | 0.237 | 0.058 | 0.416 | 0.010 |
| Avibacterium paragallinarum | 0 | Not detected | -0.057 | -0.221 | 0.107 | 0.496 |
| Azospirillum sp. 47_25 | 0 | Not detected | 0.059 | -0.294 | 0.411 | 0.743 |
| Azospirillum sp. CAG:260 | 0 | Not detected | 0.043 | -0.304 | 0.389 | 0.809 |
| Bacillus acidiproducens | 0 | Not detected | 0.131 | -0.069 | 0.332 | 0.199 |
| Bacillus andreraoultii | 0 | Not detected | 0.156 | -0.043 | 0.355 | 0.125 |
| Bacillus azotoformans | 0 | Not detected | -0.005 | -0.186 | 0.177 | 0.959 |
| Bacillus bataviensis | 0 | Not detected | -0.041 | -0.216 | 0.134 | 0.644 |
| Bacillus coahuilensis | 0 | Not detected | 0.058 | -0.143 | 0.259 | 0.572 |
| Bacillus rubiinfantis | 0 | Not detected | 0.113 | -0.065 | 0.291 | 0.212 |

| Bacillus sp. CAG:988 | 0 | Not detected | 0.099 | -0.070 | 0.268 | 0.252 |
| --- | --- | --- | --- | --- | --- | --- |
| Bacillus thermotolerans | 0 | Not detected | 0.097 | -0.096 | 0.291 | 0.323 |
| Bacteroidales bacterium 45-6 | 0 | Not detected | -0.124 | -0.314 | 0.066 | 0.201 |
| Bacteroidales bacterium 55_9 | 0 | Not detected | -0.030 | -0.126 | 0.066 | 0.544 |
| Bacteroidales bacterium Bact_08 | 0 | Not detected | -0.068 | -0.222 | 0.085 | 0.384 |
| Bacteroidales bacterium Bact_18 | 0 | Not detected | 0.086 | -0.069 | 0.240 | 0.278 |
| Bacteroidales bacterium Bact_23 | 0 | Not detected | -0.112 | -0.312 | 0.089 | 0.274 |
| Bacteroidales bacterium CF | 0 | Not detected | 0.110 | -0.060 | 0.279 | 0.205 |
| Bacteroidales bacterium H10 | 0 | Not detected | 0.017 | -0.132 | 0.165 | 0.826 |
| Bacteroidales bacterium K1 | 0 | Not detected | -0.020 | -0.191 | 0.152 | 0.821 |
| Bacteroidales bacterium K10 | 0 | Not detected | -0.099 | -0.266 | 0.068 | 0.245 |
| Bacteroidales bacterium KHT7 | 0 | Not detected | 0.096 | -0.099 | 0.290 | 0.334 |
| Bacteroides barnesiae | 0 | Not detected | -0.072 | -0.167 | 0.023 | 0.139 |
| Bacteroides caccae CAG:21 | 0 | Not detected | -0.001 | -0.201 | 0.200 | 0.994 |
| Bacteroides cellulosilyticus | 0 | Not detected | -0.103 | -0.275 | 0.069 | 0.240 |
| Bacteroides coprocola | 411 | Not detected | -0.182 | -0.314 | -0.051 | 0.007 |
| Bacteroides coprophilus | 0 | Not detected | -0.049 | -0.143 | 0.045 | 0.307 |
| Bacteroides coprosuis | 0 | Not detected | 0.087 | -0.059 | 0.232 | 0.243 |
| Bacteroides dorei CAG:222 | 0 | Not detected | -0.084 | -0.239 | 0.071 | 0.286 |
| Bacteroides faecichinchillae | 0 | Not detected | -0.082 | -0.177 | 0.012 | 0.089 |
| Bacteroides faecis CAG:32 | 0 | Not detected | -0.071 | -0.262 | 0.120 | 0.467 |
| Bacteroides finegoldii | 0 | Not detected | -0.216 | -0.394 | -0.038 | 0.018 |
| Bacteroides finegoldii CAG:203 | 0 | Not detected | -0.190 | -0.357 | -0.023 | 0.026 |
| Bacteroides fluxus | 0 | Not detected | -0.020 | -0.120 | 0.080 | 0.697 |
| Bacteroides fragilis | 0 | Not detected | -0.080 | -0.214 | 0.054 | 0.242 |
| Bacteroides fragilis CAG:47 | 0 | Not detected | -0.014 | -0.232 | 0.204 | 0.901 |
| Bacteroides graminisolvens | 0 | Not detected | -0.046 | -0.258 | 0.166 | 0.670 |
| Bacteroides neonati | 0 | Not detected | -0.079 | -0.155 | -0.002 | 0.044 |
| Bacteroides oleiciplenus | 0 | Not detected | -0.130 | -0.243 | -0.018 | 0.024 |
| Bacteroides ovatus | 0 | Not detected | -0.083 | -0.215 | 0.048 | 0.215 |
| Bacteroides ovatus CAG:22 | 0 | Not detected | -0.108 | -0.251 | 0.036 | 0.141 |
| Bacteroides paurosaccharolyticus | 0 | Not detected | -0.134 | -0.267 | -0.001 | 0.048 |
| Bacteroides pectinophilus CAG:437 | 0 | Not detected | 0.030 | -0.100 | 0.159 | 0.653 |
| Bacteroides plebeius | 0 | Not detected | -0.140 | -0.278 | -0.003 | 0.045 |
| Bacteroides propionicifaciens | 0 | Not detected | -0.033 | -0.214 | 0.147 | 0.718 |
| Bacteroides pyogenes | 0 | Not detected | -0.106 | -0.217 | 0.005 | 0.061 |
| Bacteroides reticulotermitis | 0 | Not detected | -0.077 | -0.196 | 0.042 | 0.203 |
| Bacteroides salanitronis | 0 | Not detected | -0.075 | -0.171 | 0.020 | 0.121 |
| Bacteroides salyersiae | 593 | Not detected | -0.189 | -0.323 | -0.056 | 0.005 |
| Bacteroides sp. 41_26 | 0 | Not detected | -0.143 | -0.299 | 0.013 | 0.073 |
| Bacteroides sp. 43_46 | 0 | Not detected | -0.019 | -0.156 | 0.118 | 0.785 |
| Bacteroides sp. CAG:1060 | 0 | Not detected | 0.092 | -0.155 | 0.340 | 0.465 |
| Bacteroides sp. CAG:1076 | 0 | Not detected | -0.030 | -0.190 | 0.129 | 0.709 |
| Bacteroides sp. CAG:530 | 2 | Not detected | 0.162 | 0.018 | 0.306 | 0.028 |

| Bacteroides sp. CAG:545 | 0 | Not detected | 0.109 | -0.209 | 0.428 | 0.501 |
| --- | --- | --- | --- | --- | --- | --- |
| Bacteroides sp. CAG:714 | 0 | Not detected | -0.019 | -0.245 | 0.206 | 0.866 |
| Bacteroides sp. CAG:770 | 0 | Not detected | 0.052 | -0.118 | 0.222 | 0.548 |
| Bacteroides sp. D2 | 0 | Not detected | -0.117 | -0.266 | 0.032 | 0.125 |
| Bacteroides sp. Ga6A1 | 0 | Not detected | 0.019 | -0.201 | 0.238 | 0.867 |
| Bacteroides sp. Ga6A2 | 0 | Not detected | 0.022 | -0.197 | 0.242 | 0.843 |
| Bacteroides thetaiotaomicron | 0 | Not detected | -0.058 | -0.157 | 0.040 | 0.246 |
| Bacteroides thetaiotaomicron CAG:40 | 0 | Not detected | -0.080 | -0.235 | 0.075 | 0.311 |
| Bacteroides uniformis | 0 | Not detected | -0.099 | -0.239 | 0.041 | 0.166 |
| Bacteroides vulgatus | 0 | Not detected | -0.066 | -0.196 | 0.064 | 0.319 |
| Bacteroides vulgatus CAG:6 | 0 | Not detected | -0.073 | -0.228 | 0.082 | 0.355 |
| Bacteroidetes bacterium OLB8 | 0 | Not detected | -0.114 | -0.298 | 0.070 | 0.224 |
| Bacteroidetes oral taxon 274 | 0 | Not detected | 0.026 | -0.105 | 0.157 | 0.695 |
| Bariatricus massiliensis | 0 | Not detected | -0.025 | -0.096 | 0.045 | 0.480 |
| Barnesiella intestinihominis | 0 | Not detected | -0.095 | -0.268 | 0.078 | 0.280 |
| Barnesiella viscericola | 0 | Not detected | -0.065 | -0.146 | 0.015 | 0.110 |
| Bavariicoccus seileri | 0 | Not detected | -0.001 | -0.167 | 0.166 | 0.993 |
| Beduini massiliensis | 0 | Not detected | 0.091 | -0.018 | 0.201 | 0.101 |
| Bibersteinia trehalosi | 0 | Not detected | 0.099 | -0.098 | 0.295 | 0.326 |
| Bifidobacterium actinocoloniiforme | 0 | Not detected | 0.221 | 0.032 | 0.411 | 0.022 |
| Bifidobacterium adolescentis | 0 | Not detected | 0.164 | -0.126 | 0.453 | 0.269 |
| Bifidobacterium aesculapii | 0 | Not detected | 0.136 | -0.018 | 0.289 | 0.084 |
| Bifidobacterium angulatum | 511 | Not detected | 0.310 | 0.070 | 0.550 | 0.012 |
| Bifidobacterium animalis | 0 | Not detected | 0.029 | -0.176 | 0.235 | 0.780 |
| Bifidobacterium asteroides | 0 | Not detected | 0.178 | -0.022 | 0.377 | 0.081 |
| Bifidobacterium biavatii | 267 | Not detected | 0.105 | 0.028 | 0.182 | 0.008 |
| Bifidobacterium bifidum | 0 | Not detected | 0.226 | -0.050 | 0.503 | 0.109 |
| Bifidobacterium bohemicum | 0 | Not detected | 0.201 | 0.021 | 0.381 | 0.029 |
| Bifidobacterium bombi | 0 | Not detected | 0.219 | 0.040 | 0.398 | 0.017 |
| Bifidobacterium breve | 0 | Not detected | 0.312 | 0.046 | 0.579 | 0.022 |
| Bifidobacterium callitrichos | 0 | Not detected | 0.250 | 0.012 | 0.489 | 0.040 |
| Bifidobacterium choerinum | 0 | Not detected | 0.185 | -0.025 | 0.395 | 0.084 |
| Bifidobacterium coryneforme | 0 | Not detected | 0.182 | -0.009 | 0.372 | 0.062 |
| Bifidobacterium crudilactis | 0 | Not detected | 0.126 | -0.033 | 0.285 | 0.121 |
| Bifidobacterium cuniculi | 0 | Not detected | 0.232 | 0.029 | 0.435 | 0.026 |
| Bifidobacterium gallicum | 0 | Not detected | 0.189 | -0.016 | 0.393 | 0.071 |
| Bifidobacterium gallinarum | 0 | Not detected | 0.286 | 0.045 | 0.528 | 0.020 |
| Bifidobacterium longum | 0 | Not detected | 0.272 | 0.006 | 0.537 | 0.045 |
| Bifidobacterium magnum | 0 | Not detected | 0.106 | -0.022 | 0.234 | 0.104 |
| Bifidobacterium minimum | 0 | Not detected | 0.209 | -0.041 | 0.459 | 0.101 |
| Bifidobacterium mongoliense | 9 | Not detected | 0.326 | 0.133 | 0.520 | 0.001 |
| Bifidobacterium pseudolongum | 0 | Not detected | 0.208 | -0.010 | 0.426 | 0.061 |
| Bifidobacterium psychraerophilum | 0 | Not detected | 0.066 | -0.080 | 0.212 | 0.378 |
| Bifidobacterium reuteri | 0 | Not detected | 0.168 | -0.023 | 0.360 | 0.085 |

| Bifidobacterium saguini | 0 | Not detected | 0.247 | -0.011 | 0.505 | 0.061 |
| --- | --- | --- | --- | --- | --- | --- |
| Bifidobacterium scardovii | 0 | Not detected | 0.327 | 0.071 | 0.583 | 0.012 |
| Bifidobacterium stellenboschense | 340 | Not detected | 0.127 | 0.041 | 0.213 | 0.004 |
| Bifidobacterium subtile | 0 | Not detected | 0.215 | -0.014 | 0.443 | 0.066 |
| Bifidobacterium thermophilum | 0 | Not detected | 0.121 | -0.005 | 0.247 | 0.061 |
| Bifidobacterium tsurumiense | 0 | Not detected | 0.155 | -0.051 | 0.360 | 0.140 |
| Bilophila wadsworthia | 0 | Not detected | 0.114 | -0.198 | 0.426 | 0.474 |
| Bittarella massiliensis | 0 | Not detected | -0.001 | -0.075 | 0.073 | 0.975 |
| Blautia hansenii | 0 | Not detected | -0.057 | -0.145 | 0.030 | 0.200 |
| Blautia hydrogenotrophica | 0 | Not detected | -0.043 | -0.122 | 0.035 | 0.281 |
| Blautia hydrogenotrophica CAG:147 | 0 | Not detected | -0.030 | -0.277 | 0.218 | 0.815 |
| Blautia obeum | 0 | Not detected | -0.012 | -0.093 | 0.069 | 0.776 |
| Blautia producta | 0 | Not detected | -0.140 | -0.251 | -0.029 | 0.013 |
| Blautia schinkii | 0 | Not detected | 0.004 | -0.056 | 0.063 | 0.904 |
| Blautia sp. CAG:237 | 0 | Not detected | -0.022 | -0.192 | 0.149 | 0.803 |
| Blautia sp. CAG:37 | 0 | Not detected | -0.049 | -0.211 | 0.112 | 0.549 |
| Blautia sp. CAG:37_48_57 | 0 | Not detected | -0.067 | -0.191 | 0.057 | 0.287 |
| Blautia sp. KLE 1732 | 0 | Not detected | 0.027 | -0.072 | 0.126 | 0.592 |
| Brenneria goodwinii | 0 | Not detected | -0.006 | -0.162 | 0.149 | 0.938 |
| Bulleidia extructa | 0 | Not detected | 0.037 | -0.157 | 0.232 | 0.706 |
| Burkholderiales bacterium GJ-E10 | 0 | Not detected | -0.138 | -0.318 | 0.041 | 0.131 |
| Buttiauxella ferragutiae | 0 | Not detected | 0.070 | -0.104 | 0.244 | 0.431 |
| Butyricicoccus desmolans | 0 | Not detected | -0.040 | -0.105 | 0.025 | 0.231 |
| Butyricicoccus pullicaecorum | 0 | Not detected | 0.083 | -0.001 | 0.167 | 0.052 |
| Butyricicoccus sp. K4410.MGS-46 | 0 | Not detected | -0.053 | -0.166 | 0.060 | 0.357 |
| Butyricimonas sp. Marseille-P2440 | 0 | Not detected | -0.018 | -0.183 | 0.147 | 0.828 |
| Butyricimonas synergistica | 0 | Not detected | 0.136 | -0.065 | 0.336 | 0.185 |
| Butyricimonas virosa | 0 | Not detected | 0.037 | -0.130 | 0.205 | 0.663 |
| Butyrivibrio crossotus | 0 | Not detected | -0.025 | -0.164 | 0.114 | 0.729 |
| Butyrivibrio crossotus CAG:259 | 0 | Not detected | -0.007 | -0.216 | 0.203 | 0.950 |
| Butyrivibrio fibrisolvens | 0 | Not detected | -0.048 | -0.219 | 0.124 | 0.586 |
| Butyrivibrio hungatei | 0 | Not detected | -0.077 | -0.216 | 0.061 | 0.273 |
| Butyrivibrio proteoclasticus | 0 | Not detected | -0.049 | -0.145 | 0.047 | 0.318 |
| Butyrivibrio sp. AD3002 | 0 | Not detected | 0.027 | -0.091 | 0.144 | 0.659 |
| Butyrivibrio sp. AE2005 | 0 | Not detected | 0.024 | -0.107 | 0.155 | 0.719 |
| Butyrivibrio sp. AE3003 | 0 | Not detected | 0.011 | -0.085 | 0.106 | 0.829 |
| Butyrivibrio sp. AE3004 | 0 | Not detected | -0.056 | -0.148 | 0.036 | 0.230 |
| Butyrivibrio sp. AE3009 | 0 | Not detected | 0.068 | -0.082 | 0.219 | 0.374 |
| Butyrivibrio sp. FCS006 | 0 | Not detected | -0.014 | -0.167 | 0.139 | 0.857 |
| Butyrivibrio sp. INlla14 | 0 | Not detected | 0.020 | -0.105 | 0.144 | 0.757 |
| Butyrivibrio sp. INlla16 | 0 | Not detected | -0.036 | -0.169 | 0.097 | 0.596 |
| Butyrivibrio sp. INlla21 | 0 | Not detected | -0.013 | -0.146 | 0.119 | 0.846 |
| Butyrivibrio sp. LB2008 | 0 | Not detected | 0.064 | -0.086 | 0.214 | 0.406 |
| Butyrivibrio sp. M55 | 0 | Not detected | 0.001 | -0.141 | 0.143 | 0.990 |

| Butyrivibrio sp. NC2002 | 0 | Not detected | 0.002 | -0.148 | 0.153 | 0.977 |
| --- | --- | --- | --- | --- | --- | --- |
| Butyrivibrio sp. NC2007 | 0 | Not detected | 0.008 | -0.130 | 0.145 | 0.914 |
| Butyrivibrio sp. NC3005 | 0 | Not detected | -0.026 | -0.221 | 0.168 | 0.791 |
| Butyrivibrio sp. VCD2006 | 0 | Not detected | 0.035 | -0.105 | 0.175 | 0.624 |
| Butyrivibrio sp. WCD2001 | 0 | Not detected | -0.022 | -0.161 | 0.117 | 0.755 |
| Butyrivibrio sp. WCD3002 | 0 | Not detected | -0.050 | -0.182 | 0.082 | 0.459 |
| Butyrivibrio sp. ob235 | 0 | Not detected | 0.108 | -0.055 | 0.271 | 0.195 |
| Campylobacter coli | 0 | Not detected | -0.103 | -0.210 | 0.004 | 0.059 |
| Campylobacter hominis | 0 | Not detected | -0.084 | -0.296 | 0.128 | 0.439 |
| Candidatus Azobacteroides pseudotrichonym | 0 | Not detected | -0.056 | -0.194 | 0.082 | 0.425 |
| Candidatus Gastranaerophilales bacterium M | 0 | Not detected | -0.128 | -0.311 | 0.054 | 0.168 |
| Candidatus Melainabacteria bacterium LEY3 | 0 | Not detected | -0.055 | -0.186 | 0.076 | 0.411 |
| Candidatus Melainabacteria bacterium MEL. | 0 | Not detected | 0.067 | -0.197 | 0.331 | 0.620 |
| Candidatus Saccharibacteria oral taxon TM7x | 0 | Not detected | -0.007 | -0.167 | 0.153 | 0.929 |
| Candidatus Soleaferrea massiliensis | 0 | Not detected | 0.103 | -0.014 | 0.220 | 0.084 |
| Candidatus Stoquefichus sp. SB1 | 0 | Not detected | -0.015 | -0.121 | 0.091 | 0.776 |
| Capnocytophaga granulosa | 0 | Not detected | 0.016 | -0.152 | 0.184 | 0.854 |
| Capnocytophaga sp. oral taxon 329 | 0 | Not detected | 0.035 | -0.203 | 0.273 | 0.773 |
| Capnocytophaga sp. oral taxon 863 | 0 | Not detected | -0.114 | -0.318 | 0.089 | 0.270 |
| Capnocytophaga sputigena | 0 | Not detected | -0.037 | -0.170 | 0.096 | 0.589 |
| Carnobacterium gallinarum | 0 | Not detected | 0.108 | -0.087 | 0.304 | 0.277 |
| Catabacter hongkongensis | 0 | Not detected | 0.001 | -0.156 | 0.159 | 0.986 |
| Catenibacterium mitsuokai | 0 | Not detected | 0.268 | 0.000 | 0.536 | 0.050 |
| Catenibacterium sp. CAG:290 | 0 | Not detected | 0.283 | -0.045 | 0.610 | 0.091 |
| Catonella morbi | 0 | Not detected | -0.049 | -0.140 | 0.043 | 0.296 |
| Cedecea neteri | 0 | Not detected | 0.124 | -0.093 | 0.341 | 0.264 |
| Cellulomonas carbonis | 0 | Not detected | 0.053 | -0.134 | 0.240 | 0.578 |
| Cellulomonas flavigena | 0 | Not detected | 0.027 | -0.183 | 0.237 | 0.802 |
| Cellulosilyticum lentocellum | 0 | Not detected | 0.090 | -0.061 | 0.241 | 0.241 |
| Cellulosilyticum ruminicola | 0 | Not detected | -0.020 | -0.182 | 0.141 | 0.808 |
| Chlamydia psittaci | 0 | Not detected | 0.173 | -0.001 | 0.346 | 0.051 |
| Christensenella sp. AF73-05CM02 | 0 | Not detected | 0.008 | -0.196 | 0.213 | 0.936 |
| Christensenellaceae bacterium Phil1 | 358 | Not detected | 0.179 | 0.036 | 0.323 | 0.014 |
| Christensenellaceae bacterium Phil10 | 0 | Not detected | 0.029 | -0.181 | 0.239 | 0.787 |
| Christensenellaceae bacterium Phil7 | 0 | Not detected | 0.190 | -0.026 | 0.406 | 0.084 |
| Chryseobacterium gallinarum | 0 | Not detected | -0.099 | -0.343 | 0.145 | 0.427 |
| Chryseobacterium greenlandense | 0 | Not detected | -0.147 | -0.377 | 0.084 | 0.212 |
| Citrobacter amalonaticus | 0 | Not detected | 0.092 | -0.182 | 0.366 | 0.510 |
| Citrobacter freundii | 0 | Not detected | 0.066 | -0.218 | 0.351 | 0.648 |
| Cloacibacillus evryensis | 0 | Not detected | 0.210 | -0.057 | 0.477 | 0.123 |
| Cloacibacillus porcorum | 0 | Not detected | 0.048 | -0.175 | 0.271 | 0.674 |
| Cloacibacterium normanense | 0 | Not detected | -0.112 | -0.334 | 0.110 | 0.322 |
| Clostridia bacterium UC5.1-1C12 | 0 | Not detected | -0.141 | -0.392 | 0.109 | 0.269 |
| Clostridia bacterium UC5.1-1D1 | 0 | Not detected | 0.024 | -0.056 | 0.103 | 0.561 |

| Clostridia bacterium UC5.1-1E11 | 0 | Not detected | 0.043 | -0.034 | 0.120 | 0.276 |
| --- | --- | --- | --- | --- | --- | --- |
| Clostridiales bacterium 1_7_47FAA | 0 | Not detected | 0.016 | -0.071 | 0.103 | 0.722 |
| Clostridiales bacterium 59_14 | 0 | Not detected | 0.052 | -0.079 | 0.183 | 0.436 |
| Clostridiales bacterium DTU035 | 0 | Not detected | 0.023 | -0.140 | 0.186 | 0.780 |
| Clostridiales bacterium DTU036 | 0 | Not detected | -0.090 | -0.242 | 0.063 | 0.249 |
| Clostridiales bacterium DTU089 | 0 | Not detected | -0.003 | -0.142 | 0.136 | 0.967 |
| Clostridiales bacterium Firm_03 | 0 | Not detected | 0.008 | -0.182 | 0.197 | 0.936 |
| Clostridiales bacterium Firm_16 | 0 | Not detected | -0.016 | -0.226 | 0.194 | 0.884 |
| Clostridiales bacterium NK3B98 | 0 | Not detected | 0.122 | -0.054 | 0.298 | 0.176 |
| Clostridiales bacterium S5-A14a | 0 | Not detected | 0.028 | -0.051 | 0.106 | 0.490 |
| Clostridiales bacterium SIT11 | 0 | Not detected | 0.221 | 0.044 | 0.398 | 0.014 |
| Clostridiales bacterium VE202-08 | 0 | Not detected | -0.030 | -0.119 | 0.059 | 0.507 |
| Clostridioides difficile | 0 | Not detected | -0.034 | -0.106 | 0.038 | 0.357 |
| Clostridioides mangenotii | 0 | Not detected | 0.054 | -0.116 | 0.224 | 0.535 |
| Clostridium baratii | 0 | Not detected | 0.120 | -0.080 | 0.319 | 0.241 |
| Clostridium beijerinckii | 0 | Not detected | 0.095 | -0.088 | 0.277 | 0.309 |
| Clostridium bolteae CAG:59 | 0 | Not detected | -0.169 | -0.310 | -0.029 | 0.018 |
| Clostridium botulinum | 0 | Not detected | 0.253 | 0.059 | 0.448 | 0.011 |
| Clostridium butyricum | 0 | Not detected | 0.079 | -0.121 | 0.278 | 0.441 |
| Clostridium cadaveris | 0 | Not detected | -0.016 | -0.182 | 0.149 | 0.845 |
| Clostridium celatum | 0 | Not detected | 0.253 | -0.033 | 0.538 | 0.083 |
| Clostridium cellulovorans | 0 | Not detected | -0.056 | -0.169 | 0.058 | 0.337 |
| Clostridium clostridioforme CAG:132 | 0 | Not detected | -0.212 | -0.407 | -0.018 | 0.033 |
| Clostridium clostridioforme CAG:511 | 0 | Not detected | -0.250 | -0.428 | -0.073 | 0.006 |
| Clostridium colicanis | 0 | Not detected | -0.014 | -0.166 | 0.138 | 0.854 |
| Clostridium disporicum | 0 | Not detected | 0.159 | 0.018 | 0.301 | 0.027 |
| Clostridium ihumii | 3 | Not detected | 0.257 | 0.090 | 0.424 | 0.003 |
| Clostridium kluyveri | 0 | Not detected | 0.162 | -0.030 | 0.354 | 0.097 |
| Clostridium leptum CAG:27 | 0 | Not detected | 0.020 | -0.203 | 0.244 | 0.859 |
| Clostridium lundense | 0 | Not detected | 0.061 | -0.129 | 0.251 | 0.532 |
| Clostridium neonatale | 0 | Not detected | 0.053 | -0.116 | 0.222 | 0.540 |
| Clostridium nexile CAG:348 | 0 | Not detected | -0.011 | -0.153 | 0.132 | 0.883 |
| Clostridium paraputrificum | 0 | Not detected | -0.038 | -0.274 | 0.198 | 0.754 |
| Clostridium perfringens | 0 | Not detected | 0.137 | -0.152 | 0.425 | 0.353 |
| Clostridium phoceensis | 51 | Not detected | -0.146 | -0.261 | -0.032 | 0.012 |
| Clostridium polynesiense | 0 | Not detected | 0.201 | 0.013 | 0.390 | 0.036 |
| Clostridium saccharobutylicum | 0 | Not detected | 0.078 | -0.092 | 0.248 | 0.369 |
| Clostridium saccharoperbutylacetonicum | 0 | Not detected | 0.141 | -0.024 | 0.306 | 0.094 |
| Clostridium sartagoforme | 0 | Not detected | 0.117 | -0.087 | 0.321 | 0.260 |
| Clostridium sp. 44_14 | 0 | Not detected | 0.052 | -0.121 | 0.226 | 0.555 |
| Clostridium sp. ASF356 | 0 | Not detected | -0.068 | -0.265 | 0.129 | 0.500 |
| Clostridium sp. Ade.TY | 0 | Not detected | 0.067 | -0.106 | 0.241 | 0.446 |
| Clostridium sp. CAG:1193 | 0 | Not detected | 0.241 | 0.032 | 0.451 | 0.024 |
| Clostridium sp. CAG:122 | 0 | Not detected | 0.066 | -0.158 | 0.290 | 0.562 |

| Clostridium sp. CAG:12237_41 | 0 | Not detected | 0.084 | -0.086 | 0.255 | 0.332 |
| --- | --- | --- | --- | --- | --- | --- |
| Clostridium sp. CAG:127 | 0 | Not detected | 0.210 | -0.042 | 0.463 | 0.102 |
| Clostridium sp. CAG:167 | 0 | Not detected | -0.088 | -0.381 | 0.205 | 0.557 |
| Clostridium sp. CAG:226 | 0 | Not detected | 0.234 | -0.027 | 0.494 | 0.079 |
| Clostridium sp. CAG:230 | 0 | Not detected | 0.036 | -0.146 | 0.218 | 0.700 |
| Clostridium sp. CAG:242 | 0 | Not detected | -0.025 | -0.283 | 0.233 | 0.849 |
| Clostridium sp. CAG:245 | 0 | Not detected | 0.075 | -0.306 | 0.456 | 0.700 |
| Clostridium sp. CAG:245_30_32 | 0 | Not detected | 0.031 | -0.128 | 0.190 | 0.703 |
| Clostridium sp. CAG:264 | 0 | Not detected | 0.075 | -0.179 | 0.329 | 0.563 |
| Clostridium sp. CAG:277 | 0 | Not detected | -0.038 | -0.215 | 0.139 | 0.674 |
| Clostridium sp. CAG:288 | 0 | Not detected | 0.116 | -0.148 | 0.380 | 0.391 |
| Clostridium sp. CAG:299 | 0 | Not detected | -0.102 | -0.329 | 0.125 | 0.379 |
| Clostridium sp. CAG:307_30_263 | 0 | Not detected | 0.068 | -0.081 | 0.216 | 0.372 |
| Clostridium sp. CAG:349 | 0 | Not detected | -0.127 | -0.471 | 0.217 | 0.470 |
| Clostridium sp. CAG:352 | 0 | Not detected | 0.016 | -0.202 | 0.234 | 0.886 |
| Clostridium sp. CAG:413 | 0 | Not detected | 0.178 | -0.083 | 0.439 | 0.181 |
| Clostridium sp. CAG:448 | 0 | Not detected | 0.217 | -0.064 | 0.497 | 0.130 |
| Clostridium sp. CAG:451 | 0 | Not detected | 0.239 | -0.051 | 0.528 | 0.106 |
| Clostridium sp. CAG:465 | 0 | Not detected | 0.148 | -0.124 | 0.421 | 0.286 |
| Clostridium sp. CAG:505 | 0 | Not detected | -0.192 | -0.459 | 0.074 | 0.157 |
| Clostridium sp. CAG:568 | 0 | Not detected | -0.115 | -0.396 | 0.166 | 0.422 |
| Clostridium sp. CAG:58 | 0 | Not detected | -0.019 | -0.208 | 0.170 | 0.845 |
| Clostridium sp. CAG:590 | 0 | Not detected | 0.079 | -0.061 | 0.219 | 0.269 |
| Clostridium sp. CAG:594 | 0 | Not detected | 0.258 | -0.049 | 0.565 | 0.099 |
| Clostridium sp. CAG:628 | 0 | Not detected | 0.239 | -0.051 | 0.529 | 0.106 |
| Clostridium sp. CAG:62_40_43 | 0 | Not detected | 0.055 | -0.248 | 0.358 | 0.722 |
| Clostridium sp. CAG:632 | 0 | Not detected | 0.169 | -0.014 | 0.351 | 0.071 |
| Clostridium sp. CAG:678 | 0 | Not detected | 0.071 | -0.142 | 0.284 | 0.516 |
| Clostridium sp. CAG:762 | 0 | Not detected | 0.267 | 0.025 | 0.509 | 0.031 |
| Clostridium sp. CAG:768 | 0 | Not detected | -0.010 | -0.173 | 0.153 | 0.905 |
| Clostridium sp. CAG:780 | 0 | Not detected | 0.149 | -0.159 | 0.457 | 0.343 |
| Clostridium sp. CAG:793 | 0 | Not detected | 0.008 | -0.238 | 0.254 | 0.948 |
| Clostridium sp. CAG:81 | 0 | Not detected | -0.050 | -0.187 | 0.087 | 0.474 |
| Clostridium sp. CAG:91 | 0 | Not detected | -0.059 | -0.283 | 0.164 | 0.602 |
| Clostridium sp. CAG:964 | 0 | Not detected | 0.019 | -0.118 | 0.156 | 0.781 |
| Clostridium sp. CL-6 | 0 | Not detected | 0.050 | -0.098 | 0.197 | 0.508 |
| Clostridium sp. HGF2 | 0 | Not detected | 0.035 | -0.043 | 0.112 | 0.381 |
| Clostridium sp. K4410.MGS-306 | 0 | Not detected | -0.018 | -0.120 | 0.083 | 0.721 |
| Clostridium sp. KLE 1755 | 0 | Not detected | -0.047 | -0.168 | 0.073 | 0.438 |
| Clostridium sp. KNHs205 | 0 | Not detected | -0.057 | -0.122 | 0.008 | 0.083 |
| Clostridium sp. KNHs209 | 0 | Not detected | -0.041 | -0.106 | 0.023 | 0.211 |
| Clostridium sp. KNHs214 | 603 | Not detected | 0.174 | 0.061 | 0.286 | 0.003 |
| Clostridium sp. L2-50 | 0 | Not detected | 0.018 | -0.082 | 0.118 | 0.726 |
| Clostridium sp. LF2 | 0 | Not detected | -0.005 | -0.140 | 0.130 | 0.944 |

| Clostridium sp. M62/1 | 0 | Not detected | 0.029 | -0.037 | 0.095 | 0.386 |
| --- | --- | --- | --- | --- | --- | --- |
| Clostridium sp. ND2 | 0 | Not detected | 0.084 | -0.081 | 0.248 | 0.318 |
| Clostridium sp. SY8519 | 0 | Not detected | 0.047 | -0.026 | 0.120 | 0.205 |
| Clostridium sulfidigenes | 0 | Not detected | 0.133 | 0.000 | 0.266 | 0.051 |
| Collinsella aerofaciens | 0 | Not detected | 0.125 | -0.073 | 0.323 | 0.216 |
| Collinsella intestinalis | 0 | Not detected | -0.063 | -0.287 | 0.161 | 0.580 |
| Collinsella sp. CAG:166 | 0 | Not detected | 0.124 | -0.096 | 0.345 | 0.270 |
| Collinsella sp. MS5 | 0 | Not detected | 0.144 | -0.006 | 0.293 | 0.060 |
| Collinsella stercoris | 0 | Not detected | -0.027 | -0.226 | 0.172 | 0.789 |
| Collinsella tanakaei | 0 | Not detected | -0.086 | -0.233 | 0.062 | 0.257 |
| Comamonas kerstersii | 0 | Not detected | 0.113 | -0.119 | 0.346 | 0.340 |
| Coprobacillus sp. CAG:605 | 0 | Not detected | 0.163 | -0.107 | 0.432 | 0.237 |
| Coprobacillus sp. D6 | 0 | Not detected | 0.046 | -0.052 | 0.144 | 0.356 |
| Coprobacter secundus | 0 | Not detected | -0.063 | -0.151 | 0.026 | 0.164 |
| Coprococcus comes | 0 | Not detected | 0.027 | -0.053 | 0.106 | 0.509 |
| Coprococcus eutactus | 0 | Not detected | 0.030 | -0.082 | 0.143 | 0.598 |
| Coprococcus eutactus CAG:665 | 0 | Not detected | 0.095 | -0.154 | 0.344 | 0.456 |
| Coprococcus sp. 43_8 | 0 | Not detected | 0.073 | -0.085 | 0.230 | 0.366 |
| Coprococcus sp. CAG:131 | 0 | Not detected | 0.050 | -0.190 | 0.290 | 0.684 |
| Coprococcus sp. CAG:131_42_139 | 0 | Not detected | 0.168 | -0.074 | 0.409 | 0.174 |
| Coprococcus sp. HPP0048 | 0 | Not detected | -0.001 | -0.124 | 0.123 | 0.989 |
| Coriobacteriaceae bacterium 68-1-3 | 0 | Not detected | -0.041 | -0.199 | 0.117 | 0.613 |
| Coriobacteriaceae bacterium BV3Ac1 | 0 | Not detected | 0.026 | -0.182 | 0.233 | 0.808 |
| Coriobacteriales bacterium DNF00809 | 0 | Not detected | -0.016 | -0.199 | 0.168 | 0.865 |
| Coriobacterium glomerans | 0 | Not detected | 0.101 | -0.069 | 0.271 | 0.244 |
| Corynebacterium amycolatum | 0 | Not detected | -0.035 | -0.171 | 0.100 | 0.610 |
| Corynebacterium durum | 0 | Not detected | -0.053 | -0.255 | 0.149 | 0.609 |
| Corynebacterium glucuronolyticum | 0 | Not detected | 0.031 | -0.131 | 0.192 | 0.708 |
| Corynebacterium singulare | 0 | Not detected | 0.100 | -0.051 | 0.250 | 0.196 |
| Corynebacterium urealyticum | 0 | Not detected | 0.078 | -0.089 | 0.244 | 0.360 |
| Criibacterium bergeronii | 0 | Not detected | 0.058 | -0.054 | 0.171 | 0.308 |
| Cronobacter sakazakii | 0 | Not detected | 0.113 | -0.158 | 0.384 | 0.413 |
| Cryptobacterium sp. CAG:338 | 776 | 0.6 | -0.354 | -0.632 | -0.075 | 0.013 |
| Culturomica massiliensis | 0 | Not detected | -0.091 | -0.192 | 0.010 | 0.077 |
| Cutibacterium acnes | 0 | Not detected | 0.051 | -0.134 | 0.236 | 0.590 |
| Defluviitalea phaphyphila | 0 | Not detected | -0.109 | -0.246 | 0.029 | 0.122 |
| Dehalobacter restrictus | 0 | Not detected | 0.120 | -0.020 | 0.261 | 0.094 |
| Dendrosporobacter quercicolus | 0 | Not detected | -0.088 | -0.225 | 0.050 | 0.213 |
| Denitrobacterium detoxificans | 0 | Not detected | -0.005 | -0.147 | 0.136 | 0.943 |
| Desnuesiella massiliensis | 0 | Not detected | 0.041 | -0.104 | 0.187 | 0.580 |
| Desulfovibrio desulfuricans | 0 | Not detected | 0.198 | -0.050 | 0.445 | 0.118 |
| Desulfovibrio fairfieldensis | 0 | Not detected | -0.008 | -0.292 | 0.277 | 0.959 |
| Desulfovibrio piger | 0 | Not detected | -0.121 | -0.472 | 0.230 | 0.500 |
| Desulfovibrio vulgaris | 0 | Not detected | 0.133 | -0.032 | 0.297 | 0.114 |

| Devosia insulae | 0 | Not detected | -0.017 | -0.133 | 0.099 | 0.775 |
| --- | --- | --- | --- | --- | --- | --- |
| Dialister invisus | 0 | Not detected | -0.050 | -0.424 | 0.324 | 0.794 |
| Dialister invisus CAG:218 | 0 | Not detected | -0.097 | -0.479 | 0.286 | 0.620 |
| Dialister micraerophilus | 0 | Not detected | 0.051 | -0.180 | 0.282 | 0.666 |
| Dialister sp. CAG:357 | 0 | Not detected | 0.122 | -0.244 | 0.488 | 0.513 |
| Dialister sp. CAG:486 | 0 | Not detected | 0.149 | -0.180 | 0.477 | 0.375 |
| Dialister sp. CAG:588 | 0 | Not detected | -0.007 | -0.207 | 0.194 | 0.949 |
| Dialister succinatiphilus | 0 | Not detected | 0.089 | -0.118 | 0.296 | 0.400 |
| Dickeya dadantii | 0 | Not detected | 0.061 | -0.097 | 0.219 | 0.450 |
| Dielma fastidiosa | 0 | Not detected | -0.194 | -0.378 | -0.009 | 0.039 |
| Dorea formicigenerans | 0 | Not detected | 0.007 | -0.067 | 0.080 | 0.858 |
| Dorea longicatena | 0 | Not detected | 0.044 | -0.061 | 0.150 | 0.413 |
| Dorea sp. 42_8 | 0 | Not detected | 0.027 | -0.096 | 0.150 | 0.667 |
| Dorea sp. 5-2 | 0 | Not detected | -0.005 | -0.060 | 0.050 | 0.864 |
| Dorea sp. CAG:105 | 0 | Not detected | 0.019 | -0.143 | 0.181 | 0.817 |
| Draconibacterium orientale | 0 | Not detected | 0.081 | -0.053 | 0.214 | 0.236 |
| Drancourtella massiliensis | 0 | Not detected | 0.003 | -0.132 | 0.137 | 0.970 |
| Dubosiella newyorkensis | 0 | Not detected | 0.125 | -0.021 | 0.271 | 0.094 |
| Dysgonomonas gadei | 0 | Not detected | -0.051 | -0.173 | 0.072 | 0.419 |
| Dysgonomonas mossii | 0 | Not detected | -0.059 | -0.206 | 0.089 | 0.435 |
| Dysgonomonas sp. BGC7 | 0 | Not detected | -0.078 | -0.259 | 0.102 | 0.394 |
| Dysgonomonas sp. HGC4 | 0 | Not detected | 0.010 | -0.147 | 0.166 | 0.901 |
| Edwardsiella ictaluri | 0 | Not detected | -0.021 | -0.182 | 0.139 | 0.795 |
| Eggerthella lenta | 0 | Not detected | -0.163 | -0.355 | 0.029 | 0.095 |
| Eggerthella sp. 51_9 | 0 | Not detected | 0.061 | -0.155 | 0.277 | 0.581 |
| Eggerthella sp. CAG:209 | 0 | Not detected | 0.014 | -0.316 | 0.343 | 0.936 |
| Eggerthella sp. CAG:368 | 0 | Not detected | 0.050 | -0.185 | 0.284 | 0.678 |
| Eggerthella sp. YY7918 | 0 | Not detected | -0.034 | -0.196 | 0.129 | 0.683 |
| Eggerthellaceae bacterium AT8 | 0 | Not detected | -0.002 | -0.100 | 0.095 | 0.961 |
| Eggerthia catenaformis | 0 | Not detected | 0.158 | -0.046 | 0.362 | 0.129 |
| Eisenbergiella tayi | 0 | Not detected | 0.003 | -0.157 | 0.163 | 0.968 |
| Elizabethkingia anophelis | 0 | Not detected | -0.077 | -0.287 | 0.134 | 0.477 |
| Emergencia timonensis | 0 | Not detected | 0.024 | -0.048 | 0.097 | 0.511 |
| Empedobacter brevis | 0 | Not detected | -0.156 | -0.429 | 0.116 | 0.261 |
| Enhygromyxa salina | 0 | Not detected | 0.016 | -0.160 | 0.192 | 0.860 |
| Enorma massiliensis | 0 | Not detected | 0.006 | -0.101 | 0.113 | 0.912 |
| Enorma timonensis | 0 | Not detected | 0.010 | -0.122 | 0.142 | 0.880 |
| Enterobacter cloacae | 0 | Not detected | -0.019 | -0.303 | 0.265 | 0.896 |
| Enterobacter lignolyticus | 0 | Not detected | 0.178 | -0.080 | 0.436 | 0.176 |
| Enterobacter sp. 638 | 0 | Not detected | 0.090 | -0.149 | 0.330 | 0.461 |
| Enterobacteriaceae bacterium LSJC7 | 0 | Not detected | 0.036 | -0.138 | 0.209 | 0.688 |
| Enterococcus avium | 0 | Not detected | 0.020 | -0.153 | 0.193 | 0.822 |
| Enterococcus cecorum | 0 | Not detected | 0.046 | -0.100 | 0.193 | 0.536 |
| Enterococcus columbae | 0 | Not detected | -0.008 | -0.199 | 0.182 | 0.934 |

| Enterococcus durans | 0 | Not detected | 0.180 | -0.040 | 0.399 | 0.108 |
| --- | --- | --- | --- | --- | --- | --- |
| Enterococcus faecalis | 0 | Not detected | -0.011 | -0.179 | 0.156 | 0.896 |
| Enterococcus faecium | 0 | Not detected | 0.159 | -0.015 | 0.332 | 0.073 |
| Enterococcus gallinarum | 0 | Not detected | 0.042 | -0.131 | 0.215 | 0.636 |
| Enterococcus gilvus | 0 | Not detected | 0.075 | -0.087 | 0.238 | 0.365 |
| Enterococcus italicus | 0 | Not detected | 0.078 | -0.123 | 0.278 | 0.448 |
| Enterococcus massiliensis | 0 | Not detected | 0.183 | -0.011 | 0.376 | 0.065 |
| Enterococcus sp. HMSC064A12 | 0 | Not detected | -0.010 | -0.209 | 0.189 | 0.920 |
| Enterorhabdus caecimuris | 0 | Not detected | -0.040 | -0.239 | 0.160 | 0.697 |
| Enterorhabdus mucosicola | 0 | Not detected | 0.011 | -0.162 | 0.184 | 0.901 |
| Erwinia billingiae | 0 | Not detected | 0.032 | -0.124 | 0.188 | 0.686 |
| Erwinia oleae | 0 | Not detected | 0.088 | -0.113 | 0.290 | 0.390 |
| Erysipelatoclostridium ramosum | 0 | Not detected | -0.115 | -0.352 | 0.122 | 0.343 |
| Erysipelotrichaceae bacterium 21_3 | 0 | Not detected | 0.033 | -0.065 | 0.132 | 0.509 |
| Erysipelotrichaceae bacterium 5_2_54FAA | 0 | Not detected | 0.022 | -0.059 | 0.102 | 0.599 |
| Erysipelotrichaceae bacterium CAG:64 | 0 | Not detected | 0.075 | -0.102 | 0.252 | 0.406 |
| Erysipelotrichaceae bacterium I46 | 0 | Not detected | 0.007 | -0.086 | 0.100 | 0.890 |
| Escherichia coli | 0 | Not detected | 0.173 | -0.085 | 0.432 | 0.189 |
| Escherichia vulneris | 0 | Not detected | 0.098 | -0.131 | 0.327 | 0.402 |
| Ethanoligenens harbinense | 0 | Not detected | 0.137 | -0.041 | 0.314 | 0.132 |
| Eubacterium dolichum CAG:375 | 0 | Not detected | 0.035 | -0.231 | 0.300 | 0.798 |
| Eubacterium limosum | 0 | Not detected | 0.089 | -0.029 | 0.207 | 0.141 |
| Eubacterium plexicaudatum | 0 | Not detected | 0.000 | -0.065 | 0.065 | 0.994 |
| Eubacterium pyruvativorans | 0 | Not detected | 0.037 | -0.048 | 0.122 | 0.397 |
| Eubacterium ramulus | 0 | Not detected | -0.020 | -0.095 | 0.055 | 0.599 |
| Eubacterium sp. 14-2 | 0 | Not detected | -0.029 | -0.096 | 0.038 | 0.393 |
| Eubacterium sp. 3_1_31 | 0 | Not detected | -0.029 | -0.121 | 0.064 | 0.544 |
| Eubacterium sp. 45_250 | 0 | Not detected | 0.139 | -0.124 | 0.402 | 0.300 |
| Eubacterium sp. AB3007 | 0 | Not detected | 0.260 | 0.018 | 0.502 | 0.036 |
| Eubacterium sp. CAG:115 | 0 | Not detected | 0.113 | -0.273 | 0.500 | 0.566 |
| Eubacterium sp. CAG:146 | 0 | Not detected | 0.090 | -0.077 | 0.258 | 0.291 |
| Eubacterium sp. CAG:180 | 0 | Not detected | 0.051 | -0.278 | 0.380 | 0.762 |
| Eubacterium sp. CAG:192 | 0 | Not detected | -0.056 | -0.303 | 0.190 | 0.654 |
| Eubacterium sp. CAG:202 | 0 | Not detected | 0.113 | -0.136 | 0.362 | 0.375 |
| Eubacterium sp. CAG:248 | 0 | Not detected | 0.037 | -0.169 | 0.243 | 0.726 |
| Eubacterium sp. CAG:251 | 0 | Not detected | 0.247 | -0.058 | 0.552 | 0.112 |
| Eubacterium sp. CAG:252 | 0 | Not detected | 0.042 | -0.146 | 0.230 | 0.660 |
| Eubacterium sp. CAG:581 | 0 | Not detected | 0.105 | -0.156 | 0.365 | 0.431 |
| Eubacterium sp. CAG:603 | 0 | Not detected | 0.082 | -0.056 | 0.219 | 0.244 |
| Eubacterium sp. CAG:786 | 0 | Not detected | 0.074 | -0.229 | 0.377 | 0.632 |
| Eubacterium sp. CAG:841 | 0 | Not detected | 0.066 | -0.224 | 0.355 | 0.656 |
| Eubacterium sp. ER2 | 0 | Not detected | 0.006 | -0.060 | 0.071 | 0.861 |
| Eubacterium ventriosum | 0 | Not detected | -0.011 | -0.107 | 0.085 | 0.824 |
| Eubacterium xylanophilum | 0 | Not detected | -0.038 | -0.190 | 0.113 | 0.618 |

| Facklamia hominis | 0 | Not detected | 0.218 | 0.033 | 0.403 | 0.021 |
| --- | --- | --- | --- | --- | --- | --- |
| Facklamia ignava | 0 | Not detected | 0.143 | -0.039 | 0.324 | 0.124 |
| Facklamia languida | 0 | Not detected | -0.078 | -0.250 | 0.094 | 0.374 |
| Faecalibacterium prausnitzii | 0 | Not detected | 0.038 | -0.084 | 0.161 | 0.540 |
| Faecalibacterium sp. CAG:1138 | 0 | Not detected | -0.094 | -0.348 | 0.159 | 0.466 |
| Faecalibacterium sp. CAG:82-related_59_9 | 0 | Not detected | 0.151 | -0.035 | 0.337 | 0.112 |
| Faecalibaculum rodentium | 324 | Not detected | 0.338 | 0.143 | 0.532 | 0.001 |
| Faecalicoccus pleomorphus | 0 | Not detected | 0.049 | -0.053 | 0.151 | 0.350 |
| Faecalitalea cylindroides | 0 | Not detected | 0.013 | -0.052 | 0.077 | 0.695 |
| Fenollaria massiliensis | 0 | Not detected | 0.204 | -0.014 | 0.422 | 0.067 |
| Fermentimonas caenicola | 0 | Not detected | -0.014 | -0.199 | 0.171 | 0.881 |
| Fibrobacter sp. UWB12 | 0 | Not detected | -0.019 | -0.124 | 0.087 | 0.729 |
| Fibrobacter sp. UWCM | 0 | Not detected | 0.070 | -0.073 | 0.213 | 0.337 |
| Fibrobacter sp. UWOV1 | 0 | Not detected | -0.013 | -0.117 | 0.091 | 0.808 |
| Fibrobacterales bacterium Fibro_02 | 0 | Not detected | -0.098 | -0.213 | 0.018 | 0.098 |
| Fibrobacteria bacterium AD80 | 0 | Not detected | -0.049 | -0.163 | 0.065 | 0.400 |
| Filifactor alocis | 519 | Not detected | -0.303 | -0.497 | -0.108 | 0.002 |
| Finegoldia magna | 0 | Not detected | -0.071 | -0.270 | 0.128 | 0.485 |
| Firmicutes bacterium ASF500 | 0 | Not detected | 0.021 | -0.049 | 0.090 | 0.560 |
| Firmicutes bacterium CAG:102 | 0 | Not detected | -0.138 | -0.394 | 0.117 | 0.289 |
| Firmicutes bacterium CAG:137 | 0 | Not detected | 0.198 | -0.103 | 0.500 | 0.198 |
| Firmicutes bacterium CAG:137_57_8 | 0 | Not detected | 0.021 | -0.209 | 0.251 | 0.858 |
| Firmicutes bacterium CAG:145 | 0 | Not detected | 0.101 | -0.169 | 0.372 | 0.463 |
| Firmicutes bacterium CAG:176 | 0 | Not detected | 0.271 | -0.051 | 0.592 | 0.099 |
| Firmicutes bacterium CAG:176_59_8 | 0 | Not detected | 0.264 | -0.032 | 0.560 | 0.081 |
| Firmicutes bacterium CAG:227 | 0 | Not detected | 0.040 | -0.172 | 0.252 | 0.710 |
| Firmicutes bacterium CAG:238 | 0 | Not detected | 0.181 | -0.072 | 0.433 | 0.161 |
| Firmicutes bacterium CAG:24 | 0 | Not detected | 0.079 | -0.100 | 0.257 | 0.387 |
| Firmicutes bacterium CAG:240 | 0 | Not detected | 0.209 | -0.076 | 0.495 | 0.151 |
| Firmicutes bacterium CAG:270 | 0 | Not detected | -0.037 | -0.245 | 0.170 | 0.724 |
| Firmicutes bacterium CAG:272 | 0 | Not detected | 0.089 | -0.178 | 0.355 | 0.515 |
| Firmicutes bacterium CAG:272_52_7 | 0 | Not detected | 0.102 | -0.096 | 0.301 | 0.312 |
| Firmicutes bacterium CAG:308 | 0 | Not detected | 0.101 | -0.072 | 0.273 | 0.252 |
| Firmicutes bacterium CAG:313 | 0 | Not detected | 0.087 | -0.265 | 0.440 | 0.627 |
| Firmicutes bacterium CAG:41 | 0 | Not detected | -0.056 | -0.286 | 0.173 | 0.630 |
| Firmicutes bacterium CAG:466 | 0 | Not detected | -0.042 | -0.227 | 0.143 | 0.655 |
| Firmicutes bacterium CAG:475 | 0 | Not detected | 0.117 | -0.136 | 0.370 | 0.364 |
| Firmicutes bacterium CAG:536 | 0 | Not detected | 0.266 | 0.064 | 0.467 | 0.010 |
| Firmicutes bacterium CAG:552 | 0 | Not detected | -0.170 | -0.411 | 0.071 | 0.167 |
| Firmicutes bacterium CAG:555 | 0 | Not detected | 0.071 | -0.169 | 0.312 | 0.561 |
| Firmicutes bacterium CAG:65 | 0 | Not detected | 0.138 | -0.064 | 0.340 | 0.181 |
| Firmicutes bacterium CAG:791 | 244 | Not detected | 0.180 | 0.052 | 0.309 | 0.006 |
| Firmicutes bacterium CAG:83 | 0 | Not detected | -0.008 | -0.232 | 0.216 | 0.945 |
| Firmicutes bacterium CAG:882 | 0 | Not detected | 0.013 | -0.147 | 0.173 | 0.872 |

| Firmicutes bacterium CAG:884 | 0 | Not detected | 0.118 | -0.122 | 0.359 | 0.335 |
| --- | --- | --- | --- | --- | --- | --- |
| Firmicutes bacterium CAG:94 | 0 | Not detected | 0.118 | -0.129 | 0.365 | 0.349 |
| Firmicutes bacterium M10-2 | 0 | Not detected | 0.115 | -0.084 | 0.314 | 0.258 |
| Firmicutes bacterium ZOR0006 | 0 | Not detected | 0.181 | -0.028 | 0.390 | 0.090 |
| Flavonifractor plautii | 0 | Not detected | -0.117 | -0.272 | 0.037 | 0.137 |
| Fournierella massiliensis | 0 | Not detected | 0.030 | -0.046 | 0.106 | 0.442 |
| Franconibacter pulveris | 0 | Not detected | 0.045 | -0.170 | 0.260 | 0.684 |
| Fretibacterium fastidiosum | 0 | Not detected | 0.062 | -0.079 | 0.204 | 0.389 |
| Fusicatenibacter saccharivorans | 0 | Not detected | -0.049 | -0.138 | 0.039 | 0.275 |
| Fusobacterium mortiferum | 0 | Not detected | -0.114 | -0.390 | 0.162 | 0.419 |
| Fusobacterium necrophorum | 0 | Not detected | -0.028 | -0.217 | 0.160 | 0.769 |
| Fusobacterium nucleatum | 0 | Not detected | -0.048 | -0.312 | 0.215 | 0.720 |
| Fusobacterium periodonticum | 0 | Not detected | -0.206 | -0.434 | 0.022 | 0.077 |
| Gabonia massiliensis | 0 | Not detected | -0.124 | -0.267 | 0.019 | 0.090 |
| Gabonibacter massiliensis | 0 | Not detected | -0.058 | -0.227 | 0.112 | 0.505 |
| Gallibacterium anatis | 0 | Not detected | -0.037 | -0.172 | 0.099 | 0.596 |
| Gardnerella vaginalis | 0 | Not detected | 0.298 | 0.095 | 0.501 | 0.004 |
| Gemella haemolysans | 0 | Not detected | -0.030 | -0.224 | 0.164 | 0.759 |
| Gemella sanguinis | 373 | Not detected | -0.299 | -0.535 | -0.064 | 0.013 |
| Gemmata obscuriglobus | 0 | Not detected | 0.026 | -0.141 | 0.193 | 0.760 |
| Geofilum rubicundum | 0 | Not detected | -0.121 | -0.280 | 0.038 | 0.135 |
| Gilliamella apicola | 0 | Not detected | 0.075 | -0.081 | 0.230 | 0.345 |
| Globicatella sanguinis | 0 | Not detected | 0.104 | -0.079 | 0.288 | 0.264 |
| Gordonibacter pamelaeae | 0 | Not detected | -0.091 | -0.226 | 0.045 | 0.192 |
| Granulicatella adiacens | 0 | Not detected | 0.006 | -0.222 | 0.234 | 0.957 |
| Granulicatella elegans | 0 | Not detected | 0.044 | -0.148 | 0.235 | 0.655 |
| Haemophilus influenzae | 0 | Not detected | 0.022 | -0.233 | 0.277 | 0.864 |
| Haemophilus parainfluenzae | 0 | Not detected | 0.006 | -0.304 | 0.316 | 0.971 |
| Haemophilus sputorum | 0 | Not detected | -0.085 | -0.348 | 0.178 | 0.527 |
| Hafnia sp. HMSC23F03 | 0 | Not detected | 0.025 | -0.167 | 0.218 | 0.796 |
| Hathewaya proteolytica | 3 | Not detected | 0.263 | 0.077 | 0.449 | 0.006 |
| Heliobacterium sp. N15.MGS-306 | 0 | Not detected | 0.021 | -0.062 | 0.103 | 0.626 |
| Hespellia stercorisuis | 0 | Not detected | 0.034 | -0.041 | 0.108 | 0.378 |
| Holdemanella biformis | 624 | Not detected | 0.190 | 0.050 | 0.330 | 0.008 |
| Holdemania filiformis | 0 | Not detected | -0.012 | -0.097 | 0.072 | 0.775 |
| Holdemania massiliensis | 0 | Not detected | -0.008 | -0.121 | 0.104 | 0.883 |
| Holdemania sp. Marseille-P2844 | 0 | Not detected | -0.070 | -0.169 | 0.029 | 0.168 |
| Hungatella hathewayi | 0 | Not detected | 0.060 | -0.031 | 0.151 | 0.196 |
| Hydrogenoanaerobacterium saccharovorans | 0 | Not detected | 0.081 | -0.043 | 0.205 | 0.199 |
| Idiomarina zobellii | 0 | Not detected | 0.116 | -0.108 | 0.341 | 0.310 |
| Ileibacterium valens | 0 | Not detected | 0.188 | -0.018 | 0.393 | 0.074 |
| Intestinibacter bartlettii | 0 | Not detected | 0.057 | -0.120 | 0.233 | 0.529 |
| Intestinimonas butyriciproducens | 0 | Not detected | 0.034 | -0.075 | 0.144 | 0.540 |
| Intestinimonas massiliensis | 0 | Not detected | -0.028 | -0.127 | 0.071 | 0.577 |

| Izhakiella sp. D4N98 | 0 | Not detected | 0.094 | -0.076 | 0.265 | 0.278 |
| --- | --- | --- | --- | --- | --- | --- |
| Jeotgalibaca sp. PTS2502 | 0 | Not detected | 0.162 | 0.014 | 0.310 | 0.032 |
| Johnsonella ignava | 0 | Not detected | 0.056 | -0.034 | 0.147 | 0.222 |
| Kallipyga massiliensis | 0 | Not detected | 0.039 | -0.145 | 0.222 | 0.679 |
| Kandleria vitulina | 311 | Not detected | 0.413 | 0.149 | 0.677 | 0.002 |
| Kiloniella litopenaei | 0 | Not detected | 0.080 | -0.098 | 0.258 | 0.376 |
| Kingella kingae | 0 | Not detected | -0.018 | -0.187 | 0.152 | 0.838 |
| Klebsiella oxytoca | 0 | Not detected | 0.119 | -0.186 | 0.424 | 0.445 |
| Klebsiella pneumoniae | 0 | Not detected | 0.036 | -0.271 | 0.344 | 0.818 |
| Klebsiella sp. RIT-PI-d | 0 | Not detected | 0.116 | -0.114 | 0.345 | 0.323 |
| Kluyvera ascorbata | 0 | Not detected | 0.120 | -0.161 | 0.402 | 0.402 |
| Kluyvera cryocrescens | 0 | Not detected | 0.111 | -0.178 | 0.400 | 0.452 |
| Kluyvera intermedia | 0 | Not detected | 0.090 | -0.177 | 0.357 | 0.507 |
| Kocuria sp. ZOR0020 | 5 | Not detected | -0.105 | -0.258 | 0.048 | 0.179 |
| Kosakonia sacchari | 0 | Not detected | 0.159 | -0.085 | 0.403 | 0.201 |
| Lachnoanaerobaculum saburreum | 0 | Not detected | -0.088 | -0.181 | 0.005 | 0.063 |
| Lachnoanaerobaculum sp. OBRC5-5 | 0 | Not detected | 0.042 | -0.046 | 0.130 | 0.351 |
| Lachnobacterium bovis | 0 | Not detected | -0.016 | -0.103 | 0.071 | 0.715 |
| Lachnoclostridium phytofermentans | 0 | Not detected | -0.036 | -0.103 | 0.031 | 0.293 |
| Lachnospira multipara | 0 | Not detected | -0.086 | -0.211 | 0.040 | 0.181 |
| Lachnospiraceae bacterium 10-1 | 0 | Not detected | 0.025 | -0.031 | 0.081 | 0.377 |
| Lachnospiraceae bacterium 28-4 | 0 | Not detected | -0.003 | -0.063 | 0.057 | 0.915 |
| Lachnospiraceae bacterium 2_1_46FAA | 0 | Not detected | -0.035 | -0.120 | 0.051 | 0.427 |
| Lachnospiraceae bacterium 3-1 | 0 | Not detected | 0.013 | -0.056 | 0.082 | 0.704 |
| Lachnospiraceae bacterium 3-2 | 0 | Not detected | -0.004 | -0.070 | 0.062 | 0.900 |
| Lachnospiraceae bacterium A2 | 0 | Not detected | -0.018 | -0.089 | 0.052 | 0.612 |
| Lachnospiraceae bacterium A4 | 0 | Not detected | -0.007 | -0.069 | 0.055 | 0.827 |
| Lachnospiraceae bacterium AB2028 | 0 | Not detected | 0.022 | -0.172 | 0.215 | 0.827 |
| Lachnospiraceae bacterium AC2012 | 0 | Not detected | 0.025 | -0.055 | 0.105 | 0.545 |
| Lachnospiraceae bacterium AC2014 | 0 | Not detected | 0.051 | -0.073 | 0.175 | 0.419 |
| Lachnospiraceae bacterium AC2028 | 0 | Not detected | 0.018 | -0.117 | 0.153 | 0.795 |
| Lachnospiraceae bacterium AC2029 | 0 | Not detected | 0.016 | -0.071 | 0.104 | 0.716 |
| Lachnospiraceae bacterium AC2031 | 0 | Not detected | 0.062 | -0.064 | 0.188 | 0.332 |
| Lachnospiraceae bacterium AC3007 | 0 | Not detected | 0.075 | -0.086 | 0.236 | 0.362 |
| Lachnospiraceae bacterium AD3010 | 0 | Not detected | 0.159 | -0.026 | 0.345 | 0.093 |
| Lachnospiraceae bacterium C6A11 | 0 | Not detected | -0.022 | -0.144 | 0.100 | 0.724 |
| Lachnospiraceae bacterium COE1 | 0 | Not detected | 0.069 | -0.020 | 0.157 | 0.127 |
| Lachnospiraceae bacterium FD2005 | 0 | Not detected | -0.012 | -0.171 | 0.148 | 0.887 |
| Lachnospiraceae bacterium FE2018 | 0 | Not detected | 0.000 | -0.190 | 0.189 | 0.996 |
| Lachnospiraceae bacterium G41 | 0 | Not detected | -0.007 | -0.169 | 0.156 | 0.935 |
| Lachnospiraceae bacterium KHCPX20 | 0 | Not detected | 0.091 | -0.092 | 0.274 | 0.330 |
| Lachnospiraceae bacterium M18-1 | 0 | Not detected | 0.026 | -0.040 | 0.093 | 0.440 |
| Lachnospiraceae bacterium MC2017 | 0 | Not detected | -0.040 | -0.173 | 0.093 | 0.557 |
| Lachnospiraceae bacterium NC2004 | 0 | Not detected | 0.073 | -0.103 | 0.248 | 0.419 |

| Lachnospiraceae bacterium NC2008 | 0 | Not detected | 0.033 | -0.129 | 0.194 | 0.691 |
| --- | --- | --- | --- | --- | --- | --- |
| Lachnospiraceae bacterium ND2006 | 0 | Not detected | -0.056 | -0.224 | 0.112 | 0.517 |
| Lachnospiraceae bacterium NK3A20 | 0 | Not detected | 0.060 | -0.127 | 0.248 | 0.529 |
| Lachnospiraceae bacterium NK4A179 | 0 | Not detected | -0.007 | -0.177 | 0.163 | 0.936 |
| Lachnospiraceae bacterium NLAE-zl-G231 | 0 | Not detected | -0.034 | -0.212 | 0.144 | 0.709 |
| Lachnospiraceae bacterium P6B14 | 0 | Not detected | 0.015 | -0.121 | 0.151 | 0.829 |
| Lachnospiraceae bacterium TF01-11 | 0 | Not detected | 0.027 | -0.062 | 0.117 | 0.552 |
| Lachnospiraceae bacterium TWA4 | 0 | Not detected | 0.015 | -0.079 | 0.110 | 0.751 |
| Lachnospiraceae bacterium V9D3004 | 0 | Not detected | 0.097 | -0.067 | 0.262 | 0.245 |
| Lachnospiraceae bacterium YSB2008 | 0 | Not detected | -0.014 | -0.137 | 0.108 | 0.820 |
| Lachnospiraceae bacterium mt14 | 0 | Not detected | 0.042 | -0.114 | 0.198 | 0.599 |
| Lachnospiraceae bacterium oral taxon 500 | 0 | Not detected | 0.018 | -0.052 | 0.089 | 0.611 |
| Lactobacillus acidophilus | 0 | Not detected | 0.066 | -0.073 | 0.206 | 0.352 |
| Lactobacillus amylotrophicus | 0 | Not detected | 0.025 | -0.147 | 0.198 | 0.775 |
| Lactobacillus animalis | 0 | Not detected | 0.035 | -0.136 | 0.207 | 0.685 |
| Lactobacillus antri | 219 | Not detected | 0.304 | 0.115 | 0.494 | 0.002 |
| Lactobacillus aviarius | 0 | Not detected | -0.001 | -0.096 | 0.094 | 0.986 |
| Lactobacillus casei | 0 | Not detected | -0.039 | -0.280 | 0.202 | 0.752 |
| Lactobacillus curvatus | 0 | Not detected | 0.111 | -0.077 | 0.300 | 0.247 |
| Lactobacillus delbrueckii | 0 | Not detected | -0.062 | -0.277 | 0.153 | 0.572 |
| Lactobacillus equi | 0 | Not detected | 0.087 | -0.099 | 0.274 | 0.360 |
| Lactobacillus fermentum | 0 | Not detected | 0.139 | -0.132 | 0.411 | 0.315 |
| Lactobacillus gasseri | 0 | Not detected | 0.258 | -0.017 | 0.534 | 0.067 |
| Lactobacillus helveticus | 0 | Not detected | 0.116 | -0.087 | 0.319 | 0.265 |
| Lactobacillus ingluviei | 0 | Not detected | 0.062 | -0.100 | 0.225 | 0.453 |
| Lactobacillus jensenii | 0 | Not detected | 0.068 | -0.105 | 0.241 | 0.443 |
| Lactobacillus kefiranofaciens | 0 | Not detected | 0.067 | -0.092 | 0.225 | 0.410 |
| Lactobacillus manihotivorans | 0 | Not detected | 0.108 | -0.068 | 0.284 | 0.229 |
| Lactobacillus mucosae | 0 | Not detected | -0.061 | -0.244 | 0.123 | 0.518 |
| Lactobacillus paracasei | 0 | Not detected | -0.003 | -0.251 | 0.245 | 0.982 |
| Lactobacillus pasteurii | 0 | Not detected | 0.070 | -0.081 | 0.221 | 0.364 |
| Lactobacillus plantarum | 0 | Not detected | 0.023 | -0.182 | 0.229 | 0.824 |
| Lactobacillus rhamnosus | 0 | Not detected | 0.005 | -0.217 | 0.228 | 0.962 |
| Lactobacillus ruminis | 0 | Not detected | 0.030 | -0.153 | 0.212 | 0.750 |
| Lactobacillus ruminis CAG:367 | 0 | Not detected | 0.035 | -0.148 | 0.218 | 0.708 |
| Lactobacillus sakei | 0 | Not detected | 0.137 | -0.046 | 0.321 | 0.142 |
| Lactobacillus salivarius | 0 | Not detected | 0.214 | -0.049 | 0.478 | 0.111 |
| Lactobacillus vaginalis | 0 | Not detected | 0.146 | -0.088 | 0.380 | 0.222 |
| Lactococcus garvieae | 0 | Not detected | 0.042 | -0.146 | 0.231 | 0.659 |
| Lactococcus lactis | 0 | Not detected | -0.090 | -0.378 | 0.199 | 0.542 |
| Lactococcus piscium | 0 | Not detected | -0.093 | -0.285 | 0.099 | 0.342 |
| Lactonifactor longoviformis | 0 | Not detected | 0.012 | -0.046 | 0.071 | 0.680 |
| Lamprocystis purpurea | 0 | Not detected | 0.191 | -0.038 | 0.421 | 0.102 |
| Lascolabacillus massiliensis | 0 | Not detected | 0.018 | -0.139 | 0.176 | 0.820 |

| Lautropia mirabilis | 0 | Not detected | 0.063 | -0.131 | 0.256 | 0.525 |
| --- | --- | --- | --- | --- | --- | --- |
| Lawsonia intracellularis | 0 | Not detected | 0.117 | -0.072 | 0.306 | 0.224 |
| Leclercia adecarboxylata | 0 | Not detected | 0.090 | -0.182 | 0.362 | 0.517 |
| Lelliottia amnigena | 0 | Not detected | 0.144 | -0.154 | 0.441 | 0.343 |
| Lentimicrobium saccharophilum | 0 | Not detected | -0.110 | -0.313 | 0.094 | 0.291 |
| Leuconostoc citreum | 0 | Not detected | 0.014 | -0.156 | 0.184 | 0.870 |
| Leuconostoc lactis | 0 | Not detected | -0.050 | -0.253 | 0.152 | 0.626 |
| Leuconostoc mesenteroides | 0 | Not detected | -0.050 | -0.268 | 0.167 | 0.649 |
| Levyella massiliensis | 0 | Not detected | 0.033 | -0.064 | 0.131 | 0.504 |
| Libanicoccus massiliensis | 0 | Not detected | -0.025 | -0.197 | 0.148 | 0.781 |
| Luteimonas sp. FCS-9 | 0 | Not detected | 0.018 | -0.127 | 0.164 | 0.806 |
| Lutispora thermophila | 0 | Not detected | 0.144 | -0.024 | 0.311 | 0.094 |
| Macellibacteroides sp. HH-ZS | 8 | Not detected | -0.150 | -0.277 | -0.023 | 0.021 |
| Macrococcus caseolyticus | 0 | Not detected | 0.052 | -0.133 | 0.238 | 0.581 |
| Mageeibacillus indolicus | 141 | Not detected | 0.198 | 0.060 | 0.335 | 0.005 |
| Mangrovibacter sp. MFB070 | 0 | Not detected | 0.090 | -0.125 | 0.305 | 0.414 |
| Mannheimia haemolytica | 0 | Not detected | 0.066 | -0.112 | 0.244 | 0.466 |
| Marvinbryantia formatexigens | 0 | Not detected | 0.014 | -0.072 | 0.100 | 0.749 |
| Massilioclostridium coli | 0 | Not detected | -0.017 | -0.102 | 0.067 | 0.687 |
| Massiliomicrobiota timonensis | 0 | Not detected | 0.140 | -0.054 | 0.335 | 0.158 |
| Mediterranea massiliensis | 0 | Not detected | 0.001 | -0.108 | 0.110 | 0.981 |
| Megamonas funiformis | 0 | Not detected | 0.000 | -0.334 | 0.334 | 1.000 |
| Megasphaera cerevisiae | 0 | Not detected | 0.100 | -0.151 | 0.350 | 0.435 |
| Megasphaera elsdenii | 0 | Not detected | 0.081 | -0.238 | 0.400 | 0.619 |
| Megasphaera genomosp. type_1 | 0 | Not detected | 0.039 | -0.025 | 0.102 | 0.230 |
| Megasphaera massiliensis | 0 | Not detected | 0.251 | -0.048 | 0.550 | 0.100 |
| Megasphaera micronuciformis | 0 | Not detected | 0.152 | -0.075 | 0.379 | 0.190 |
| Megasphaera sp. BV3C16-1 | 0 | Not detected | 0.189 | -0.004 | 0.381 | 0.055 |
| Megasphaera sp. UPII 135-E | 0 | Not detected | 0.014 | -0.052 | 0.079 | 0.686 |
| Merdibacter massiliensis | 0 | Not detected | 0.023 | -0.050 | 0.095 | 0.542 |
| Methanobrevibacter smithii | 0 | Not detected | 0.343 | -0.044 | 0.730 | 0.082 |
| Methanobrevibacter smithii CAG:186 | 0 | Not detected | 0.335 | -0.026 | 0.696 | 0.069 |
| Methanocorpusculaceae archaeon Phil4 | 0 | Not detected | 0.180 | -0.072 | 0.432 | 0.161 |
| Methanosarcina mazei | 0 | Not detected | 0.142 | -0.079 | 0.363 | 0.207 |
| Methylobacterium sp. ZNC0032 | 104 | Not detected | -0.167 | -0.321 | -0.013 | 0.034 |
| Methyloglobulus morosus | 0 | Not detected | 0.142 | -0.040 | 0.324 | 0.127 |
| Microcystis aeruginosa | 0 | Not detected | 0.021 | -0.167 | 0.208 | 0.829 |
| Mitsuokella multacida | 0 | Not detected | 0.180 | -0.148 | 0.509 | 0.282 |
| Mobilibacterium timonense | 0 | Not detected | -0.087 | -0.265 | 0.091 | 0.337 |
| Mobiluncus curtisii | 0 | Not detected | -0.009 | -0.085 | 0.068 | 0.825 |
| Mobiluncus mulieris | 0 | Not detected | -0.039 | -0.127 | 0.049 | 0.382 |
| Mogibacterium timidum | 0 | Not detected | -0.007 | -0.219 | 0.204 | 0.947 |
| Moraxella porci | 0 | Not detected | 0.044 | -0.105 | 0.193 | 0.563 |
| Morganella morganii | 0 | Not detected | 0.040 | -0.163 | 0.243 | 0.698 |

| Morococcus cerebrosus | 0 | Not detected | 0.039 | -0.128 | 0.207 | 0.643 |
| --- | --- | --- | --- | --- | --- | --- |
| Mucinivorans hirudinis | 0 | Not detected | 0.080 | -0.069 | 0.229 | 0.293 |
| Murdochiella sp. Marseille-P2341 | 0 | Not detected | 0.018 | -0.081 | 0.117 | 0.722 |
| Mycoplasma sp. CAG:877 | 0 | Not detected | 0.304 | 0.042 | 0.566 | 0.023 |
| Mycoplasma sp. CAG:956 | 189 | Not detected | 0.277 | 0.044 | 0.509 | 0.020 |
| Myroides injenensis | 0 | Not detected | -0.103 | -0.327 | 0.120 | 0.365 |
| Myroides odoratimimus | 0 | Not detected | -0.087 | -0.322 | 0.148 | 0.470 |
| Necropsobacter massiliensis | 0 | Not detected | -0.066 | -0.273 | 0.140 | 0.530 |
| Negativicoccus massiliensis | 0 | Not detected | -0.086 | -0.252 | 0.079 | 0.305 |
| Neglecta timonensis | 0 | Not detected | -0.007 | -0.120 | 0.106 | 0.903 |
| Neisseria cinerea | 0 | Not detected | -0.079 | -0.256 | 0.099 | 0.383 |
| Neisseria mucosa | 0 | Not detected | -0.174 | -0.357 | 0.009 | 0.062 |
| Nissabacter archeti | 0 | Not detected | 0.086 | -0.123 | 0.295 | 0.420 |
| Novibacillus thermophilus | 0 | Not detected | 0.022 | -0.167 | 0.211 | 0.820 |
| Obesumbacterium proteus | 0 | Not detected | 0.164 | -0.084 | 0.412 | 0.195 |
| Oceanospirillum linum | 0 | Not detected | 0.110 | -0.067 | 0.287 | 0.223 |
| Odoribacter laneus | 0 | Not detected | -0.155 | -0.322 | 0.012 | 0.069 |
| Odoribacter laneus CAG:561 | 0 | Not detected | -0.077 | -0.303 | 0.148 | 0.501 |
| Odoribacter sp. 43_10 | 0 | Not detected | -0.032 | -0.171 | 0.107 | 0.651 |
| Odoribacter sp. CAG:788 | 0 | Not detected | -0.016 | -0.235 | 0.202 | 0.883 |
| Odoribacter splanchnicus | 0 | Not detected | -0.050 | -0.152 | 0.051 | 0.330 |
| Odoribacter splanchnicus CAG:14 | 0 | Not detected | 0.013 | -0.139 | 0.165 | 0.868 |
| Oligella ureolytica | 0 | Not detected | 0.149 | -0.031 | 0.330 | 0.105 |
| Olsenella scatoligenes | 0 | Not detected | -0.015 | -0.211 | 0.180 | 0.878 |
| Olsenella sp. Marseille-P3197 | 0 | Not detected | 0.182 | -0.012 | 0.376 | 0.066 |
| Olsenella sp. oral taxon 809 | 0 | Not detected | 0.009 | -0.127 | 0.145 | 0.900 |
| Olsenella uli | 0 | Not detected | 0.030 | -0.165 | 0.225 | 0.764 |
| Olsenella umbonata | 0 | Not detected | 0.079 | -0.080 | 0.238 | 0.331 |
| Oribacterium parvum | 0 | Not detected | 0.011 | -0.052 | 0.073 | 0.734 |
| Oribacterium sinus | 0 | Not detected | -0.013 | -0.160 | 0.135 | 0.866 |
| Oribacterium sp. WCC10 | 0 | Not detected | 0.054 | -0.143 | 0.251 | 0.593 |
| Oribacterium sp. oral taxon 078 | 0 | Not detected | 0.031 | -0.068 | 0.130 | 0.539 |
| Oscillibacter ruminantium | 0 | Not detected | 0.084 | -0.055 | 0.223 | 0.234 |
| Oscillibacter sp. 1-3 | 0 | Not detected | 0.064 | -0.014 | 0.142 | 0.106 |
| Oscillibacter sp. 57_20 | 0 | Not detected | 0.018 | -0.229 | 0.265 | 0.887 |
| Oscillibacter sp. ER4 | 0 | Not detected | 0.148 | 0.002 | 0.294 | 0.048 |
| Oscillibacter sp. KLE 1745 | 0 | Not detected | -0.038 | -0.163 | 0.088 | 0.555 |
| Oscillibacter valericigenes | 0 | Not detected | 0.057 | -0.068 | 0.183 | 0.371 |
| Oscillospiraceae bacterium Zagget12 | 0 | Not detected | 0.003 | -0.151 | 0.157 | 0.969 |
| Oxalobacter formigenes | 0 | Not detected | 0.061 | -0.096 | 0.219 | 0.445 |
| Paeniclostridium sordellii | 0 | Not detected | 0.132 | -0.078 | 0.342 | 0.218 |
| Paludibacter propionicigenes | 10 | Not detected | -0.130 | -0.275 | 0.014 | 0.077 |
| Pantoea agglomerans | 0 | Not detected | 0.042 | -0.134 | 0.217 | 0.642 |
| Pantoea ananatis | 0 | Not detected | 0.097 | -0.063 | 0.256 | 0.235 |

| Pantoea rwandensis | 0 | Not detected | 0.039 | -0.129 | 0.206 | 0.653 |
| --- | --- | --- | --- | --- | --- | --- |
| Pantoea sp. IMH | 0 | Not detected | 0.014 | -0.157 | 0.185 | 0.875 |
| Pantoea sp. PSNIH2 | 0 | Not detected | 0.099 | -0.163 | 0.362 | 0.459 |
| Parabacteroides distasonis | 0 | Not detected | -0.113 | -0.255 | 0.030 | 0.122 |
| Parabacteroides goldsteinii | 0 | Not detected | -0.007 | -0.163 | 0.149 | 0.929 |
| Parabacteroides gordonii | 0 | Not detected | -0.067 | -0.194 | 0.060 | 0.302 |
| Parabacteroides johnsonii | 0 | Not detected | -0.120 | -0.233 | -0.008 | 0.036 |
| Parabacteroides merdae CAG:48 | 0 | Not detected | -0.100 | -0.306 | 0.107 | 0.344 |
| Paraburkholderia sacchari | 0 | Not detected | -0.059 | -0.232 | 0.113 | 0.499 |
| Paraclostridium bifermentans | 0 | Not detected | 0.110 | -0.079 | 0.299 | 0.256 |
| Paraprevotella clara CAG:116 | 0 | Not detected | -0.081 | -0.332 | 0.169 | 0.525 |
| Paraprevotella xylaniphila | 0 | Not detected | 0.032 | -0.102 | 0.166 | 0.637 |
| Parascardovia denticolens | 0 | Not detected | 0.291 | 0.093 | 0.488 | 0.004 |
| Parasporobacterium paucivorans | 0 | Not detected | -0.026 | -0.144 | 0.093 | 0.669 |
| Parasutterella excrementihominis | 0 | Not detected | -0.103 | -0.352 | 0.146 | 0.416 |
| Parasutterella excrementihominis CAG:233 | 0 | Not detected | -0.033 | -0.413 | 0.348 | 0.867 |
| Parvimonas micra | 0 | Not detected | -0.058 | -0.277 | 0.161 | 0.605 |
| Pasteurella bettyae | 0 | Not detected | -0.051 | -0.228 | 0.125 | 0.569 |
| Pectobacterium carotovorum | 0 | Not detected | 0.042 | -0.119 | 0.202 | 0.611 |
| Pedobacter glucosidilyticus | 0 | Not detected | 0.054 | -0.113 | 0.220 | 0.528 |
| Pelosinus fermentans | 0 | Not detected | -0.137 | -0.297 | 0.023 | 0.094 |
| Peptoanaerobacter stomatis | 0 | Not detected | -0.101 | -0.268 | 0.067 | 0.238 |
| Peptococcus niger | 0 | Not detected | -0.028 | -0.184 | 0.129 | 0.727 |
| Peptoniphilus duerdenii | 0 | Not detected | 0.116 | -0.019 | 0.251 | 0.092 |
| Peptoniphilus grossensis | 0 | Not detected | -0.001 | -0.177 | 0.175 | 0.991 |
| Peptoniphilus harei | 0 | Not detected | 0.015 | -0.186 | 0.216 | 0.885 |
| Peptoniphilus lacrimalis | 0 | Not detected | 0.122 | -0.093 | 0.337 | 0.267 |
| Peptoniphilus obesi | 0 | Not detected | -0.028 | -0.218 | 0.162 | 0.770 |
| Peptoniphilus rhinitidis | 0 | Not detected | 0.099 | -0.088 | 0.286 | 0.300 |
| Peptoniphilus senegalensis | 0 | Not detected | 0.143 | -0.058 | 0.344 | 0.162 |
| Peptoniphilus sp. BV3C26 | 0 | Not detected | 0.161 | -0.056 | 0.378 | 0.147 |
| Peptoniphilus sp. ING2-D1G | 0 | Not detected | -0.063 | -0.249 | 0.123 | 0.506 |
| Peptoniphilus sp. oral taxon 375 | 0 | Not detected | 0.034 | -0.057 | 0.125 | 0.459 |
| Peptostreptococcaceae bacterium VA2 | 0 | Not detected | 0.167 | -0.057 | 0.392 | 0.144 |
| Peptostreptococcus anaerobius | 0 | Not detected | -0.003 | -0.074 | 0.068 | 0.928 |
| Peptostreptococcus anaerobius CAG:621 | 0 | Not detected | -0.005 | -0.163 | 0.153 | 0.948 |
| Peptostreptococcus stomatis | 0 | Not detected | 0.056 | -0.137 | 0.250 | 0.567 |
| Petrimonas mucosa | 731 | 0.6 | 0.255 | 0.071 | 0.439 | 0.007 |
| Phaeodactylibacter xiamenensis | 0 | Not detected | -0.159 | -0.401 | 0.082 | 0.196 |
| Phascolarctobacterium sp. CAG:207 | 0 | Not detected | -0.276 | -0.640 | 0.088 | 0.137 |
| Phascolarctobacterium sp. CAG:266 | 0 | Not detected | -0.153 | -0.409 | 0.103 | 0.242 |
| Phascolarctobacterium succinatutens | 0 | Not detected | 0.128 | -0.057 | 0.312 | 0.175 |
| Phocaeicola abscessus | 0 | Not detected | -0.037 | -0.189 | 0.114 | 0.630 |
| Phocea massiliensis | 0 | Not detected | -0.044 | -0.138 | 0.051 | 0.368 |

| Piscirickettsia salmonis | 0 | Not detected | 0.009 | -0.146 | 0.163 | 0.914 |
| --- | --- | --- | --- | --- | --- | --- |
| Plautia stali symbiont | 0 | Not detected | 0.003 | -0.156 | 0.161 | 0.974 |
| Pluralibacter gergoviae | 0 | Not detected | 0.083 | -0.166 | 0.333 | 0.513 |
| Pontibacillus litoralis | 0 | Not detected | -0.134 | -0.364 | 0.097 | 0.255 |
| Porphyromonadaceae bacterium COT-184 OH | 0 | Not detected | -0.018 | -0.186 | 0.149 | 0.830 |
| Porphyromonadaceae bacterium KH3CP3RA | 0 | Not detected | -0.083 | -0.249 | 0.083 | 0.329 |
| Porphyromonadaceae bacterium KH3R12 | 0 | Not detected | -0.105 | -0.265 | 0.054 | 0.196 |
| Porphyromonas asaccharolytica | 0 | Not detected | -0.130 | -0.335 | 0.075 | 0.213 |
| Porphyromonas bennonis | 0 | Not detected | -0.122 | -0.316 | 0.073 | 0.221 |
| Porphyromonas cangingivalis | 0 | Not detected | 0.087 | -0.093 | 0.266 | 0.345 |
| Porphyromonas canoris | 0 | Not detected | -0.113 | -0.293 | 0.067 | 0.217 |
| Porphyromonas catoniae | 0 | Not detected | -0.048 | -0.187 | 0.090 | 0.494 |
| Porphyromonas crevioricanis | 0 | Not detected | 0.015 | -0.108 | 0.139 | 0.806 |
| Porphyromonas endodontalis | 0 | Not detected | 0.031 | -0.169 | 0.231 | 0.762 |
| Porphyromonas gingivalis | 0 | Not detected | 0.036 | -0.128 | 0.199 | 0.671 |
| Porphyromonas gingivicanis | 0 | Not detected | -0.006 | -0.137 | 0.125 | 0.930 |
| Porphyromonas levii | 0 | Not detected | 0.034 | -0.203 | 0.272 | 0.777 |
| Porphyromonas macacae | 0 | Not detected | 0.060 | -0.118 | 0.238 | 0.510 |
| Porphyromonas somerae | 0 | Not detected | -0.087 | -0.173 | -0.001 | 0.048 |
| Porphyromonas sp. CAG:1061 | 0 | Not detected | -0.053 | -0.269 | 0.162 | 0.628 |
| Porphyromonas sp. COT-239 OH1446 | 445 | Not detected | -0.236 | -0.455 | -0.016 | 0.035 |
| Porphyromonas sp. COT-290 OH3588 | 0 | Not detected | 0.085 | -0.130 | 0.300 | 0.438 |
| Prevotella albensis | 0 | Not detected | 0.112 | -0.196 | 0.421 | 0.475 |
| Prevotella amnii | 2 | Not detected | -0.094 | -0.176 | -0.013 | 0.024 |
| Prevotella aurantiaca | 0 | Not detected | 0.074 | -0.125 | 0.273 | 0.466 |
| Prevotella baroniae | 0 | Not detected | -0.009 | -0.106 | 0.088 | 0.853 |
| Prevotella bergensis | 0 | Not detected | -0.015 | -0.167 | 0.136 | 0.845 |
| Prevotella bivia | 0 | Not detected | 0.014 | -0.090 | 0.117 | 0.798 |
| Prevotella bryantii | 0 | Not detected | 0.256 | -0.053 | 0.565 | 0.104 |
| Prevotella buccae | 0 | Not detected | 0.026 | -0.208 | 0.261 | 0.826 |
| Prevotella buccalis | 0 | Not detected | 0.012 | -0.076 | 0.100 | 0.783 |
| Prevotella copri | 0 | Not detected | 0.245 | -0.081 | 0.571 | 0.141 |
| Prevotella corporis | 1 | Not detected | -0.092 | -0.179 | -0.005 | 0.037 |
| Prevotella dentalis | 0 | Not detected | 0.188 | -0.054 | 0.430 | 0.128 |
| Prevotella dentasini | 0 | Not detected | 0.193 | -0.048 | 0.434 | 0.117 |
| Prevotella denticola | 0 | Not detected | 0.180 | -0.088 | 0.448 | 0.188 |
| Prevotella disiens | 0 | Not detected | -0.056 | -0.149 | 0.038 | 0.244 |
| Prevotella enoeca | 0 | Not detected | 0.072 | -0.161 | 0.305 | 0.545 |
| Prevotella fusca | 0 | Not detected | 0.209 | -0.057 | 0.475 | 0.124 |
| Prevotella histicola | 0 | Not detected | 0.011 | -0.151 | 0.173 | 0.891 |
| Prevotella intermedia | 0 | Not detected | 0.092 | -0.072 | 0.257 | 0.272 |
| Prevotella loescheii | 0 | Not detected | 0.163 | -0.059 | 0.386 | 0.150 |
| Prevotella maculosa | 0 | Not detected | 0.006 | -0.186 | 0.197 | 0.955 |
| Prevotella marshii | 0 | Not detected | -0.023 | -0.118 | 0.071 | 0.632 |

| Prevotella melaninogenica | 0 | Not detected | 0.121 | -0.067 | 0.310 | 0.206 |
| --- | --- | --- | --- | --- | --- | --- |
| Prevotella micans | 0 | Not detected | 0.195 | -0.029 | 0.418 | 0.088 |
| Prevotella multiformis | 0 | Not detected | 0.004 | -0.134 | 0.142 | 0.955 |
| Prevotella multisaccharivorax | 0 | Not detected | 0.087 | -0.215 | 0.389 | 0.573 |
| Prevotella nanceiensis | 0 | Not detected | 0.000 | -0.177 | 0.178 | 0.997 |
| Prevotella nigrescens | 0 | Not detected | 0.186 | -0.032 | 0.404 | 0.095 |
| Prevotella oralis | 0 | Not detected | 0.152 | -0.081 | 0.385 | 0.200 |
| Prevotella oris | 0 | Not detected | 0.148 | -0.110 | 0.407 | 0.261 |
| Prevotella oryzae | 0 | Not detected | 0.205 | -0.064 | 0.474 | 0.135 |
| Prevotella oulorum | 0 | Not detected | 0.187 | -0.091 | 0.465 | 0.188 |
| Prevotella pallens | 0 | Not detected | -0.059 | -0.163 | 0.045 | 0.266 |
| Prevotella paludivivens | 0 | Not detected | 0.075 | -0.109 | 0.259 | 0.424 |
| Prevotella ruminicola | 0 | Not detected | 0.201 | -0.058 | 0.460 | 0.128 |
| Prevotella saccharolytica | 0 | Not detected | 0.184 | -0.066 | 0.434 | 0.150 |
| Prevotella salivae | 0 | Not detected | -0.041 | -0.137 | 0.055 | 0.404 |
| Prevotella scopos | 0 | Not detected | 0.041 | -0.089 | 0.171 | 0.539 |
| Prevotella sp. 10(H) | 0 | Not detected | 0.012 | -0.149 | 0.173 | 0.882 |
| Prevotella sp. 109 | 0 | Not detected | 0.008 | -0.085 | 0.100 | 0.873 |
| Prevotella sp. AGR2160 | 0 | Not detected | 0.289 | -0.003 | 0.581 | 0.053 |
| Prevotella sp. BP1-148 | 0 | Not detected | 0.091 | -0.044 | 0.227 | 0.186 |
| Prevotella sp. CAG:1058 | 0 | Not detected | -0.072 | -0.317 | 0.172 | 0.563 |
| Prevotella sp. CAG:1092 | 0 | Not detected | 0.330 | 0.023 | 0.637 | 0.036 |
| Prevotella sp. CAG:1124 | 0 | Not detected | 0.023 | -0.225 | 0.271 | 0.857 |
| Prevotella sp. CAG:1185 | 0 | Not detected | -0.165 | -0.424 | 0.094 | 0.212 |
| Prevotella sp. CAG:1320 | 0 | Not detected | 0.032 | -0.166 | 0.230 | 0.752 |
| Prevotella sp. CAG:255 | 0 | Not detected | 0.027 | -0.134 | 0.187 | 0.745 |
| Prevotella sp. CAG:386 | 0 | Not detected | 0.278 | -0.059 | 0.615 | 0.106 |
| Prevotella sp. CAG:487 | 0 | Not detected | -0.052 | -0.212 | 0.108 | 0.524 |
| Prevotella sp. CAG:520 | 0 | Not detected | 0.153 | -0.124 | 0.430 | 0.280 |
| Prevotella sp. CAG:5226 | 0 | Not detected | 0.107 | -0.197 | 0.411 | 0.490 |
| Prevotella sp. CAG:604 | 0 | Not detected | 0.257 | -0.107 | 0.621 | 0.167 |
| Prevotella sp. CAG:732 | 0 | Not detected | 0.293 | -0.033 | 0.619 | 0.078 |
| Prevotella sp. CAG:891 | 0 | Not detected | 0.060 | -0.177 | 0.297 | 0.620 |
| Prevotella sp. FD3004 | 0 | Not detected | 0.276 | 0.069 | 0.483 | 0.009 |
| Prevotella sp. HUN102 | 0 | Not detected | 0.075 | -0.058 | 0.209 | 0.270 |
| Prevotella sp. MSX73 | 0 | Not detected | -0.010 | -0.245 | 0.225 | 0.935 |
| Prevotella sp. P4-76 | 0 | Not detected | 0.153 | -0.018 | 0.323 | 0.080 |
| Prevotella sp. P6B1 | 0 | Not detected | 0.177 | 0.034 | 0.321 | 0.016 |
| Prevotella sp. P6B4 | 0 | Not detected | 0.221 | 0.081 | 0.361 | 0.002 |
| Prevotella sp. RM4 | 0 | Not detected | 0.087 | -0.089 | 0.263 | 0.333 |
| Prevotella sp. S7 MS 2 | 0 | Not detected | -0.038 | -0.130 | 0.055 | 0.423 |
| Prevotella sp. S7-1-8 | 0 | Not detected | 0.006 | -0.080 | 0.093 | 0.884 |
| Prevotella sp. kh1p2 | 0 | Not detected | 0.195 | -0.040 | 0.430 | 0.104 |
| Prevotella sp. khp1 | 0 | Not detected | 0.106 | -0.074 | 0.286 | 0.249 |

| Prevotella sp. ne3005 | 0 | Not detected | 0.091 | -0.067 | 0.249 | 0.261 |
| --- | --- | --- | --- | --- | --- | --- |
| Prevotella sp. oral taxon 473 | 0 | Not detected | 0.039 | -0.198 | 0.277 | 0.745 |
| Prevotella stercorea | 0 | Not detected | 0.015 | -0.113 | 0.143 | 0.817 |
| Prevotella stercorea CAG:629 | 0 | Not detected | 0.126 | -0.162 | 0.414 | 0.391 |
| Prevotella timonensis | 0 | Not detected | -0.035 | -0.125 | 0.056 | 0.456 |
| Prevotellamassilia timonensis | 0 | Not detected | 0.078 | -0.072 | 0.229 | 0.308 |
| Propionibacterium acidifaciens | 238 | Not detected | 0.263 | 0.070 | 0.456 | 0.008 |
| Proteiniclasticum ruminis | 0 | Not detected | 0.018 | -0.144 | 0.180 | 0.831 |
| Proteiniphilum acetatigenes | 0 | Not detected | -0.025 | -0.195 | 0.144 | 0.768 |
| Proteiniphilum saccharofermentans | 0 | Not detected | -0.072 | -0.258 | 0.115 | 0.451 |
| Proteiniphilum sp. EBM-41 | 0 | Not detected | -0.071 | -0.300 | 0.159 | 0.546 |
| Proteobacteria bacterium CAG:139 | 0 | Not detected | 0.011 | -0.352 | 0.374 | 0.954 |
| Proteobacteria bacterium CAG:495 | 0 | Not detected | 0.021 | -0.288 | 0.331 | 0.893 |
| Providencia rettgeri | 0 | Not detected | -0.002 | -0.201 | 0.198 | 0.987 |
| Pseudobacteroides cellulosolvens | 13 | Not detected | -0.140 | -0.288 | 0.008 | 0.063 |
| Pseudobutyrivibrio ruminis | 0 | Not detected | 0.016 | -0.060 | 0.091 | 0.686 |
| Pseudobutyrivibrio sp. ACV-2 | 0 | Not detected | -0.051 | -0.221 | 0.119 | 0.556 |
| Pseudobutyrivibrio sp. JW11 | 0 | Not detected | -0.114 | -0.281 | 0.053 | 0.181 |
| Pseudobutyrivibrio sp. MD2005 | 0 | Not detected | 0.079 | -0.101 | 0.258 | 0.391 |
| Pseudobutyrivibrio sp. UC1225 | 0 | Not detected | 0.060 | -0.055 | 0.175 | 0.307 |
| Pseudobutyrivibrio sp. YE44 | 0 | Not detected | 0.017 | -0.150 | 0.185 | 0.840 |
| Pseudobutyrivibrio xylanivorans | 0 | Not detected | -0.004 | -0.176 | 0.168 | 0.967 |
| Pseudoflavonifractor capillosus | 0 | Not detected | 0.033 | -0.048 | 0.114 | 0.422 |
| Pseudopropionibacterium propionicum | 0 | Not detected | -0.022 | -0.215 | 0.171 | 0.822 |
| Pyramidobacter piscolens | 0 | Not detected | 0.011 | -0.241 | 0.264 | 0.929 |
| Rahnella aquatilis | 0 | Not detected | 0.027 | -0.131 | 0.186 | 0.737 |
| Ralstonia mannitolilytica | 0 | Not detected | 0.130 | -0.089 | 0.350 | 0.245 |
| Ralstonia pickettii | 0 | Not detected | 0.025 | -0.182 | 0.232 | 0.813 |
| Raoultella ornithinolytica | 0 | Not detected | 0.091 | -0.212 | 0.395 | 0.555 |
| Rappaport israeli | 0 | Not detected | 0.033 | -0.133 | 0.200 | 0.696 |
| Rhodococcus defluvii | 0 | Not detected | 0.012 | -0.150 | 0.174 | 0.887 |
| Rhodopseudomonas palustris | 0 | Not detected | -0.026 | -0.210 | 0.158 | 0.781 |
| Rhodospirillum sp. UNK.MGS-17 | 0 | Not detected | -0.014 | -0.364 | 0.336 | 0.939 |
| Riemerella columbina | 0 | Not detected | -0.082 | -0.168 | 0.004 | 0.062 |
| Rikenella microfusus | 0 | Not detected | -0.070 | -0.157 | 0.017 | 0.117 |
| Risungbinella massiliensis | 0 | Not detected | -0.037 | -0.182 | 0.108 | 0.615 |
| Robinsoniella peoriensis | 0 | Not detected | -0.034 | -0.095 | 0.027 | 0.277 |
| Rodentibacter pneumotropicus | 0 | Not detected | -0.083 | -0.235 | 0.068 | 0.281 |
| Roseburia faecis | 0 | Not detected | 0.021 | -0.116 | 0.159 | 0.759 |
| Roseburia hominis | 0 | Not detected | 0.085 | -0.002 | 0.173 | 0.056 |
| Roseburia intestinalis | 0 | Not detected | -0.043 | -0.176 | 0.089 | 0.520 |
| Roseburia inulinivorans | 0 | Not detected | 0.018 | -0.089 | 0.126 | 0.740 |
| Roseburia sp. CAG:197_41_10 | 0 | Not detected | 0.002 | -0.109 | 0.114 | 0.967 |
| Roseburia sp. CAG:309 | 0 | Not detected | -0.138 | -0.378 | 0.102 | 0.260 |

| Roseburia sp. CAG:380 | 0 | Not detected | -0.012 | -0.231 | 0.208 | 0.918 |
| --- | --- | --- | --- | --- | --- | --- |
| Rothia dentocariosa | 2 | Not detected | -0.150 | -0.386 | 0.085 | 0.210 |
| Rothia mucilaginosa | 0 | Not detected | 0.054 | -0.195 | 0.302 | 0.671 |
| Ruminiclostridium thermocellum | 0 | Not detected | 0.043 | -0.072 | 0.158 | 0.463 |
| Ruminococcaceae bacterium AB4001 | 0 | Not detected | 0.101 | -0.051 | 0.254 | 0.192 |
| Ruminococcaceae bacterium AE2021 | 2 | Not detected | 0.222 | 0.075 | 0.370 | 0.003 |
| Ruminococcaceae bacterium D16 | 0 | Not detected | 0.050 | -0.024 | 0.125 | 0.188 |
| Ruminococcaceae bacterium YRB3002 | 0 | Not detected | -0.009 | -0.186 | 0.169 | 0.924 |
| Ruminococcus albus | 0 | Not detected | 0.075 | -0.076 | 0.227 | 0.330 |
| Ruminococcus bicirculans | 0 | Not detected | 0.087 | -0.138 | 0.311 | 0.449 |
| Ruminococcus callidus | 0 | Not detected | 0.113 | -0.039 | 0.265 | 0.147 |
| Ruminococcus champanellensis | 0 | Not detected | 0.096 | -0.097 | 0.290 | 0.330 |
| Ruminococcus faecis | 0 | Not detected | 0.079 | -0.020 | 0.179 | 0.119 |
| Ruminococcus flavefaciens | 0 | Not detected | 0.078 | -0.062 | 0.218 | 0.275 |
| Ruminococcus gauvreauii | 0 | Not detected | -0.018 | -0.098 | 0.061 | 0.651 |
| Ruminococcus lactaris | 0 | Not detected | 0.058 | -0.056 | 0.171 | 0.321 |
| Ruminococcus obeum CAG:39 | 0 | Not detected | -0.005 | -0.138 | 0.129 | 0.947 |
| Ruminococcus sp. AT10 | 0 | Not detected | -0.057 | -0.130 | 0.016 | 0.125 |
| Ruminococcus sp. CAG:108-related_41_35 | 0 | Not detected | -0.022 | -0.260 | 0.215 | 0.854 |
| Ruminococcus sp. CAG:177 | 0 | Not detected | 0.175 | -0.196 | 0.546 | 0.357 |
| Ruminococcus sp. CAG:254 | 0 | Not detected | -0.119 | -0.459 | 0.221 | 0.494 |
| Ruminococcus sp. CAG:330 | 0 | Not detected | 0.248 | -0.091 | 0.587 | 0.152 |
| Ruminococcus sp. CAG:379 | 0 | Not detected | 0.009 | -0.242 | 0.260 | 0.946 |
| Ruminococcus sp. CAG:382 | 0 | Not detected | 0.122 | -0.201 | 0.444 | 0.460 |
| Ruminococcus sp. CAG:403 | 0 | Not detected | 0.138 | -0.130 | 0.407 | 0.312 |
| Ruminococcus sp. CAG:488 | 311 | Not detected | 0.279 | 0.053 | 0.506 | 0.016 |
| Ruminococcus sp. CAG:563 | 0 | Not detected | 0.150 | -0.099 | 0.400 | 0.237 |
| Ruminococcus sp. CAG:60 | 0 | Not detected | 0.042 | -0.101 | 0.184 | 0.565 |
| Ruminococcus sp. CAG:624 | 0 | Not detected | 0.085 | -0.159 | 0.329 | 0.496 |
| Ruminococcus sp. CAG:724 | 0 | Not detected | 0.203 | -0.071 | 0.476 | 0.147 |
| Ruminococcus sp. FC2018 | 0 | Not detected | 0.079 | -0.083 | 0.241 | 0.340 |
| Ruminococcus sp. HUN007 | 0 | Not detected | 0.089 | -0.047 | 0.225 | 0.199 |
| Ruminococcus sp. NK3A76 | 0 | Not detected | 0.085 | -0.079 | 0.249 | 0.311 |
| Ruminococcus sp. Phil15 | 0 | Not detected | -0.089 | -0.306 | 0.127 | 0.418 |
| Ruminococcus sp. Zagget7 | 0 | Not detected | 0.034 | -0.088 | 0.155 | 0.589 |
| Ruminococcus torques CAG:61 | 0 | Not detected | -0.180 | -0.406 | 0.045 | 0.117 |
| Ruthenibacterium lactatiformans | 0 | Not detected | -0.020 | -0.128 | 0.088 | 0.719 |
| Salmonella enterica | 0 | Not detected | 0.098 | -0.169 | 0.366 | 0.471 |
| Sanguibacteroides justesenii | 0 | Not detected | -0.050 | -0.217 | 0.117 | 0.556 |
| Scardovia inopinata | 0 | Not detected | 0.106 | -0.055 | 0.267 | 0.196 |
| Scardovia wiggsiae | 0 | Not detected | 0.156 | -0.055 | 0.367 | 0.148 |
| Selenomonas bovis | 0 | Not detected | 0.000 | -0.138 | 0.138 | 1.000 |
| Selenomonas noxia | 0 | Not detected | 0.027 | -0.134 | 0.189 | 0.740 |
| Selenomonas ruminantium | 0 | Not detected | 0.118 | -0.078 | 0.314 | 0.238 |

| Selenomonas sputigena | 0 | Not detected | -0.015 | -0.185 | 0.154 | 0.860 |
| --- | --- | --- | --- | --- | --- | --- |
| Sellimonas intestinalis | 0 | Not detected | 0.035 | -0.073 | 0.143 | 0.528 |
| Senegalimassilia anaerobia | 0 | Not detected | 0.107 | -0.087 | 0.301 | 0.280 |
| Serratia fonticola | 0 | Not detected | 0.007 | -0.145 | 0.160 | 0.927 |
| Serratia liquefaciens | 0 | Not detected | 0.120 | -0.043 | 0.282 | 0.148 |
| Serratia marcescens | 0 | Not detected | 0.094 | -0.082 | 0.271 | 0.296 |
| Serratia odorifera | 0 | Not detected | 0.119 | -0.050 | 0.288 | 0.166 |
| Serratia rubidaea | 0 | Not detected | 0.052 | -0.120 | 0.224 | 0.554 |
| Sharpea azabuensis | 0 | Not detected | 0.085 | -0.007 | 0.177 | 0.072 |
| Shigella dysenteriae | 0 | Not detected | 0.180 | -0.091 | 0.452 | 0.194 |
| Shigella flexneri | 0 | Not detected | 0.192 | -0.078 | 0.462 | 0.164 |
| Shimwellia blattae | 0 | Not detected | 0.119 | -0.105 | 0.343 | 0.299 |
| Shuttleworthia satelles | 0 | Not detected | 0.052 | -0.054 | 0.157 | 0.338 |
| Siccibacter turicensis | 0 | Not detected | 0.076 | -0.162 | 0.314 | 0.532 |
| Slackia exigua | 0 | Not detected | 0.025 | -0.151 | 0.200 | 0.784 |
| Slackia heliotrinireducens | 0 | Not detected | -0.050 | -0.206 | 0.106 | 0.527 |
| Slackia piriformis | 0 | Not detected | 0.285 | 0.024 | 0.546 | 0.033 |
| Solobacterium moorei | 0 | Not detected | 0.061 | -0.045 | 0.168 | 0.261 |
| Sphaerochaeta sp. Phil3 | 0 | Not detected | 0.061 | -0.130 | 0.251 | 0.533 |
| Sphingobacterium wenxiniae | 0 | Not detected | -0.066 | -0.260 | 0.127 | 0.503 |
| Spirochaetales bacterium Spiro_07 | 0 | Not detected | -0.066 | -0.222 | 0.089 | 0.403 |
| Sporobacter termitidis | 0 | Not detected | 0.146 | -0.008 | 0.301 | 0.064 |
| Sporomusa ovata | 14 | Not detected | -0.196 | -0.371 | -0.021 | 0.028 |
| Staphylococcus pseudintermedius | 0 | Not detected | 0.215 | 0.033 | 0.397 | 0.021 |
| Staphylococcus sp. CAG:324 | 0 | Not detected | 0.086 | -0.239 | 0.411 | 0.604 |
| Staphylococcus sp. DORA_6_22 | 0 | Not detected | 0.034 | -0.144 | 0.212 | 0.708 |
| Staphylococcus warneri | 0 | Not detected | 0.064 | -0.132 | 0.261 | 0.522 |
| Stomatobaculum longum | 0 | Not detected | 0.012 | -0.047 | 0.071 | 0.686 |
| Streptobacillus hongkongensis | 0 | Not detected | 0.070 | -0.101 | 0.241 | 0.424 |
| Streptobacillus notomytis | 0 | Not detected | 0.024 | -0.169 | 0.217 | 0.808 |
| Streptococcus agalactiae | 0 | Not detected | 0.183 | -0.017 | 0.383 | 0.073 |
| Streptococcus anginosus | 213 | Not detected | 0.298 | 0.079 | 0.518 | 0.008 |
| Streptococcus caballi | 0 | Not detected | 0.023 | -0.181 | 0.227 | 0.827 |
| Streptococcus cristatus | 1 | Not detected | 0.154 | -0.021 | 0.328 | 0.084 |
| Streptococcus dysgalactiae | 0 | Not detected | 0.124 | -0.076 | 0.324 | 0.223 |
| Streptococcus entericus | 0 | Not detected | 0.121 | -0.042 | 0.283 | 0.145 |
| Streptococcus gallolyticus | 0 | Not detected | 0.099 | -0.145 | 0.343 | 0.426 |
| Streptococcus gordonii | 0 | Not detected | 0.110 | -0.123 | 0.343 | 0.355 |
| Streptococcus halotolerans | 0 | Not detected | -0.032 | -0.197 | 0.133 | 0.705 |
| Streptococcus henryi | 0 | Not detected | 0.018 | -0.179 | 0.214 | 0.859 |
| Streptococcus ictaluri | 0 | Not detected | -0.056 | -0.229 | 0.117 | 0.523 |
| Streptococcus infantis | 0 | Not detected | 0.034 | -0.140 | 0.207 | 0.702 |
| Streptococcus iniae | 0 | Not detected | -0.060 | -0.237 | 0.118 | 0.510 |
| Streptococcus intermedius | 0 | Not detected | 0.203 | -0.023 | 0.428 | 0.078 |

| Streptococcus massiliensis | 0 | Not detected | 0.018 | -0.165 | 0.200 | 0.849 |
| --- | --- | --- | --- | --- | --- | --- |
| Streptococcus minor | 0 | Not detected | -0.012 | -0.149 | 0.124 | 0.859 |
| Streptococcus mitis | 0 | Not detected | 0.052 | -0.079 | 0.184 | 0.434 |
| Streptococcus mutans | 0 | Not detected | 0.153 | -0.116 | 0.421 | 0.265 |
| Streptococcus oralis | 0 | Not detected | 0.079 | -0.127 | 0.285 | 0.452 |
| Streptococcus orisratti | 0 | Not detected | 0.196 | 0.003 | 0.388 | 0.046 |
| Streptococcus ovis | 0 | Not detected | 0.057 | -0.122 | 0.236 | 0.532 |
| Streptococcus parasanguinis | 0 | Not detected | 0.117 | -0.092 | 0.327 | 0.273 |
| Streptococcus parauberis | 0 | Not detected | -0.087 | -0.268 | 0.094 | 0.345 |
| Streptococcus phocae | 0 | Not detected | -0.060 | -0.196 | 0.076 | 0.386 |
| Streptococcus plurextorum | 0 | Not detected | -0.029 | -0.121 | 0.063 | 0.537 |
| Streptococcus porci | 0 | Not detected | 0.010 | -0.100 | 0.120 | 0.857 |
| Streptococcus pseudoporcinus | 0 | Not detected | 0.184 | -0.011 | 0.379 | 0.065 |
| Streptococcus pyogenes | 0 | Not detected | 0.075 | -0.125 | 0.274 | 0.463 |
| Streptococcus salivarius | 0 | Not detected | 0.028 | -0.165 | 0.220 | 0.777 |
| Streptococcus sanguinis | 0 | Not detected | 0.157 | -0.070 | 0.384 | 0.176 |
| Streptococcus sobrinus | 0 | Not detected | 0.136 | -0.071 | 0.343 | 0.199 |
| Streptococcus sp. X13SY08 | 0 | Not detected | 0.026 | -0.116 | 0.167 | 0.721 |
| Streptococcus suis | 0 | Not detected | 0.067 | -0.027 | 0.162 | 0.162 |
| Streptococcus thermophilus | 0 | Not detected | -0.089 | -0.292 | 0.114 | 0.390 |
| Streptococcus uberis | 0 | Not detected | 0.012 | -0.161 | 0.185 | 0.892 |
| Streptococcus urinalis | 0 | Not detected | -0.034 | -0.231 | 0.164 | 0.739 |
| Streptomyces albulus | 0 | Not detected | 0.029 | -0.133 | 0.190 | 0.727 |
| Streptomyces cattleya | 0 | Not detected | 0.085 | -0.102 | 0.271 | 0.374 |
| Streptomyces kanamyceticus | 0 | Not detected | -0.014 | -0.135 | 0.108 | 0.827 |
| Subdoligranulum sp. CAG:314 | 0 | Not detected | 0.185 | -0.198 | 0.567 | 0.344 |
| Subdoligranulum variabile | 0 | Not detected | 0.055 | -0.018 | 0.127 | 0.140 |
| Succinatimonas sp. CAG:777 | 0 | Not detected | 0.170 | -0.145 | 0.485 | 0.290 |
| Succinispira mobilis | 0 | Not detected | -0.172 | -0.358 | 0.013 | 0.068 |
| Succinivibrio sp. Phil9 | 0 | Not detected | 0.266 | -0.032 | 0.565 | 0.081 |
| Sulfolobus acidocaldarius | 0 | Not detected | -0.100 | -0.333 | 0.133 | 0.400 |
| Sutterella parvirubra | 0 | Not detected | -0.149 | -0.351 | 0.054 | 0.150 |
| Sutterella sp. CAG:397 | 0 | Not detected | -0.149 | -0.552 | 0.253 | 0.467 |
| Sutterella sp. CAG:521 | 0 | Not detected | -0.164 | -0.420 | 0.091 | 0.208 |
| Sutterella wadsworthensis | 0 | Not detected | 0.117 | -0.209 | 0.443 | 0.483 |
| Sutterella wadsworthensis CAG:135 | 0 | Not detected | 0.056 | -0.354 | 0.465 | 0.790 |
| Sutterellaceae bacterium ND3 | 0 | Not detected | 0.171 | -0.078 | 0.420 | 0.179 |
| Synergistales bacterium Syner_02 | 0 | Not detected | 0.102 | -0.110 | 0.315 | 0.346 |
| Synergistes sp. 3_1_syn1 | 0 | Not detected | 0.184 | -0.085 | 0.453 | 0.181 |
| Synergistes sp. Zagget9 | 0 | Not detected | -0.099 | -0.266 | 0.068 | 0.245 |
| Tannerella forsythia | 0 | Not detected | -0.011 | -0.137 | 0.116 | 0.870 |
| Tatumella morbirosei | 0 | Not detected | 0.116 | -0.044 | 0.275 | 0.154 |
| Tepidanaerobacter sp. EBM-49 | 0 | Not detected | 0.000 | -0.259 | 0.258 | 0.998 |
| Terrisporobacter glycolicus | 0 | Not detected | 0.100 | -0.123 | 0.322 | 0.380 |

| Thermophagus xiamenensis | 0 | Not detected | -0.090 | -0.289 | 0.109 | 0.376 |
| --- | --- | --- | --- | --- | --- | --- |
| Thermosinus carboxydivorans | 0 | Not detected | -0.102 | -0.252 | 0.049 | 0.186 |
| Tidjanibacter massiliensis | 0 | Not detected | 0.039 | -0.224 | 0.302 | 0.774 |
| Tissierellia bacterium S7-1-4 | 0 | Not detected | -0.198 | -0.396 | -0.001 | 0.049 |
| Trabulsiella odontotermitis | 0 | Not detected | 0.052 | -0.197 | 0.300 | 0.684 |
| Traorella massiliensis | 0 | Not detected | -0.013 | -0.084 | 0.058 | 0.728 |
| Treponema denticola | 0 | Not detected | 0.110 | -0.038 | 0.259 | 0.145 |
| Treponema medium | 0 | Not detected | -0.259 | -0.501 | -0.017 | 0.036 |
| Treponema putidum | 0 | Not detected | 0.082 | -0.049 | 0.212 | 0.220 |
| Treponema socranskii | 0 | Not detected | -0.014 | -0.163 | 0.135 | 0.852 |
| Treponema succinifaciens | 1 | Not detected | 0.084 | 0.001 | 0.168 | 0.048 |
| Treponema vincentii | 0 | Not detected | -0.001 | -0.137 | 0.136 | 0.991 |
| Trichococcus flocculiformis | 0 | Not detected | -0.063 | -0.226 | 0.099 | 0.445 |
| Trueperella pyogenes | 0 | Not detected | -0.096 | -0.260 | 0.068 | 0.252 |
| Tumebacillus flagellatus | 0 | Not detected | 0.180 | 0.018 | 0.342 | 0.029 |
| Turicibacter sanguinis | 0 | Not detected | 0.006 | -0.282 | 0.294 | 0.968 |
| Turicibacter sp. H121 | 0 | Not detected | 0.092 | -0.020 | 0.204 | 0.107 |
| Tyzzerella nexilis | 0 | Not detected | -0.028 | -0.094 | 0.038 | 0.403 |
| Urmitella timonensis | 0 | Not detected | -0.054 | -0.179 | 0.071 | 0.398 |
| Vaginella massiliensis | 0 | Not detected | -0.136 | -0.377 | 0.105 | 0.269 |
| Varibaculum cambriense | 0 | Not detected | -0.017 | -0.118 | 0.085 | 0.750 |
| Varibaculum sp. Marseille-P2802 | 0 | Not detected | 0.040 | -0.122 | 0.202 | 0.629 |
| Veillonella magna | 0 | Not detected | -0.061 | -0.134 | 0.012 | 0.104 |
| Veillonella parvula | 0 | Not detected | 0.103 | -0.140 | 0.346 | 0.406 |
| Veillonella seminalis | 0 | Not detected | -0.008 | -0.092 | 0.077 | 0.860 |
| Veillonella sp. ACP1 | 0 | Not detected | 0.026 | -0.267 | 0.319 | 0.864 |
| Veillonella sp. CAG:933 | 0 | Not detected | -0.062 | -0.208 | 0.084 | 0.406 |
| Veillonella sp. oral taxon 780 | 6 | Not detected | -0.160 | -0.343 | 0.023 | 0.087 |
| Waddlia chondrophila | 0 | Not detected | -0.083 | -0.309 | 0.144 | 0.475 |
| Weissella cibaria | 0 | Not detected | 0.037 | -0.187 | 0.261 | 0.745 |
| Weissella confusa | 0 | Not detected | -0.027 | -0.282 | 0.228 | 0.835 |
| Xanthomonas sp. Mitacek01 | 0 | Not detected | -0.005 | -0.140 | 0.130 | 0.945 |
| Yersinia enterocolitica | 0 | Not detected | 0.094 | -0.122 | 0.311 | 0.393 |
| Yersinia pestis | 0 | Not detected | 0.182 | -0.093 | 0.456 | 0.194 |
| Yokenella regensburgei | 0 | Not detected | 0.111 | -0.134 | 0.355 | 0.376 |
| [Bacteroides] pectinophilus | 0 | Not detected | 0.020 | -0.058 | 0.098 | 0.613 |
| [Clostridium] aminophilum | 0 | Not detected | 0.031 | -0.038 | 0.101 | 0.373 |
| [Clostridium] asparagiforme | 0 | Not detected | -0.101 | -0.203 | 0.000 | 0.050 |
| [Clostridium] bolteae | 0 | Not detected | -0.062 | -0.156 | 0.031 | 0.193 |
| [Clostridium] celerecrescens | 0 | Not detected | -0.063 | -0.127 | 0.000 | 0.052 |
| [Clostridium] cellobioparum | 0 | Not detected | 0.041 | -0.107 | 0.189 | 0.586 |
| [Clostridium] cellulolyticum | 0 | Not detected | 0.117 | -0.051 | 0.284 | 0.172 |
| [Clostridium] cellulosi | 0 | Not detected | 0.136 | -0.032 | 0.303 | 0.113 |
| [Clostridium] citroniae | 0 | Not detected | -0.048 | -0.122 | 0.026 | 0.207 |

| [Clostridium] clariflavum | 0 | Not detected | 0.037 | -0.108 | 0.181 | 0.617 |
| --- | --- | --- | --- | --- | --- | --- |
| [Clostridium] clostridioforme | 0 | Not detected | -0.126 | -0.226 | -0.026 | 0.014 |
| [Clostridium] dakarense | 0 | Not detected | 0.183 | -0.040 | 0.405 | 0.108 |
| [Clostridium] glycyrrhizinilyticum | 0 | Not detected | -0.005 | -0.069 | 0.060 | 0.886 |
| [Clostridium] hiranonis | 0 | Not detected | 0.180 | -0.023 | 0.382 | 0.082 |
| [Clostridium] hylemonae | 0 | Not detected | -0.110 | -0.253 | 0.033 | 0.132 |
| [Clostridium] innocuum | 13 | Not detected | 0.075 | 0.006 | 0.145 | 0.034 |
| [Clostridium] josui | 0 | Not detected | 0.019 | -0.152 | 0.189 | 0.828 |
| [Clostridium] lactatifermentans | 0 | Not detected | -0.001 | -0.087 | 0.085 | 0.981 |
| [Clostridium] leptum | 0 | Not detected | 0.011 | -0.101 | 0.122 | 0.852 |
| [Clostridium] methylpentosum | 0 | Not detected | -0.021 | -0.133 | 0.091 | 0.719 |
| [Clostridium] papyrosolvens | 0 | Not detected | 0.008 | -0.122 | 0.137 | 0.905 |
| [Clostridium] saccharogumia | 0 | Not detected | -0.033 | -0.133 | 0.066 | 0.514 |
| [Clostridium] saccharolyticum | 0 | Not detected | -0.030 | -0.122 | 0.062 | 0.526 |
| [Clostridium] scindens | 0 | Not detected | 0.011 | -0.070 | 0.092 | 0.790 |
| [Clostridium] spiroforme | 0 | Not detected | -0.059 | -0.243 | 0.125 | 0.528 |
| [Clostridium] sporosphaeroides | 0 | Not detected | 0.049 | -0.101 | 0.200 | 0.520 |
| [Clostridium] symbiosum | 0 | Not detected | -0.039 | -0.121 | 0.043 | 0.350 |
| [Clostridium] viride | 0 | Not detected | 0.069 | -0.073 | 0.211 | 0.342 |
| [Enterobacter] aerogenes | 0 | Not detected | 0.185 | -0.126 | 0.495 | 0.244 |
| [Eubacterium] brachy | 0 | Not detected | 0.057 | -0.158 | 0.272 | 0.603 |
| [Eubacterium] cellulosolvens | 0 | Not detected | -0.058 | -0.148 | 0.032 | 0.205 |
| [Eubacterium] contortum | 0 | Not detected | 0.004 | -0.057 | 0.065 | 0.908 |
| [Eubacterium] dolichum | 0 | Not detected | 0.042 | -0.021 | 0.105 | 0.188 |
| [Eubacterium] eligens | 0 | Not detected | 0.015 | -0.144 | 0.175 | 0.852 |
| [Eubacterium] fissicatena | 0 | Not detected | -0.013 | -0.095 | 0.069 | 0.754 |
| [Eubacterium] hallii | 0 | Not detected | 0.053 | -0.056 | 0.162 | 0.342 |
| [Eubacterium] nodatum | 0 | Not detected | 0.059 | -0.137 | 0.255 | 0.553 |
| [Eubacterium] rectale | 0 | Not detected | 0.128 | -0.029 | 0.285 | 0.109 |
| [Eubacterium] siraeum | 0 | Not detected | 0.138 | -0.103 | 0.379 | 0.262 |
| [Eubacterium] yurii | 0 | Not detected | 0.046 | -0.078 | 0.170 | 0.468 |
| [Haemophilus] ducreyi | 0 | Not detected | -0.095 | -0.247 | 0.056 | 0.219 |
| [Haemophilus] parasuis | 0 | Not detected | -0.013 | -0.197 | 0.171 | 0.889 |
| [Ruminococcus] gnavus | 0 | Not detected | -0.054 | -0.177 | 0.070 | 0.396 |
| [Ruminococcus] torques | 0 | Not detected | -0.080 | -0.214 | 0.054 | 0.240 |
| bacterium F082 | 0 | Not detected | 0.037 | -0.070 | 0.145 | 0.497 |
| bacterium LF-3 | 0 | Not detected | -0.022 | -0.183 | 0.139 | 0.791 |
| bacterium MS4 | 0 | Not detected | 0.077 | -0.065 | 0.218 | 0.288 |
| bacterium OL-1 | 0 | Not detected | -0.066 | -0.325 | 0.193 | 0.617 |
| bacterium P201 | 0 | Not detected | 0.135 | -0.031 | 0.300 | 0.111 |
| bacterium P3 | 0 | Not detected | 0.191 | -0.003 | 0.384 | 0.053 |

| **Supplementary Table 3**. Association of parental education (High school or more vs. less than High school) with functional modules in overall participants (n=2174). Analysis of Compositions of Microbiomes (ANCOM2) was used to identify functional modules related to parental education. Regression coefficients were obtained by modeling the effect of parental education on centered log ratio (CLR)-transformed species in multivariable linear regression adjusted for age, gender, BMI, field center, Hispanic/Latino background,  U.S. nativity, Bristol stool type, current SEP, the alternative Healthy Eating Index 2010 score, cigarette smoking, alcohol use,  depressive and anxiety symptoms, diabetes, hypertension, cardiovascular disease, dyslipidemia, and antibiotic use. | | | | | | |
| --- | --- | --- | --- | --- | --- | --- |
| **otu.names** | **pathname** | **ANCOM** | **beta** | **l95ci** | **u95ci** | **qval** |
| PWY.7610 | GDP-6-deoxy-D-<i>altro</i>-heptose biosynthes | 0.7 | -0.233 | -0.391 | -0.076 | 0.004 |
| PWY.7904 | tRNA-uridine 2-thiolation (thermophilic bacteria | 0.8 | -0.346 | -0.542 | -0.150 | 0.001 |
| PWY0.43 | conversion of succinate to propanoate | 0.8 | -0.243 | -0.377 | -0.110 | 0.000 |
| ADENOSYL | L-methionine salvage from L-homocysteine | Not detected | -0.035 | -0.109 | 0.039 | 0.351 |
| ALANINE.V | L-alanine biosynthesis I | Not detected | -0.108 | -0.288 | 0.071 | 0.238 |
| ANAGLYCO | glycolysis III (from glucose) | Not detected | 0.007 | -0.059 | 0.073 | 0.840 |
| APYRASE.R | NA | Not detected | 0.005 | -0.065 | 0.074 | 0.898 |
| ARABCAT.P | L-arabinose degradation I | Not detected | -0.008 | -0.081 | 0.065 | 0.838 |
| ARGDEG.III | L-arginine degradation IV (arginine decarboxylas | Not detected | -0.037 | -0.140 | 0.067 | 0.486 |
| ARGSYN.PW | L-arginine biosynthesis I (via L-ornithine) | Not detected | -0.037 | -0.150 | 0.075 | 0.517 |
| ARGSYNBS | L-arginine biosynthesis II (acetyl cycle) | Not detected | -0.021 | -0.130 | 0.089 | 0.713 |
| AST.PWY | L-arginine degradation II (AST pathway) | Not detected | 0.019 | -0.193 | 0.232 | 0.858 |
| BETSYN.PW | glycine betaine biosynthesis I (Gram-negative ba | Not detected | -0.175 | -0.369 | 0.020 | 0.079 |
| CALVIN.PW | Calvin-Benson-Bassham cycle | Not detected | 0.001 | -0.064 | 0.066 | 0.976 |
| CARBPSYN. | NA | Not detected | 0.051 | -0.086 | 0.189 | 0.463 |
| CARNMET.P | L-carnitine degradation I | Not detected | 0.009 | -0.210 | 0.229 | 0.933 |
| CENTFERM. | pyruvate fermentation to butanoate | Not detected | 0.002 | -0.085 | 0.089 | 0.960 |
| CHLOROPH | 3,8-divinyl-chlorophyllide <i>a</i> biosynthesis | Not detected | 0.126 | -0.087 | 0.340 | 0.246 |
| CHOLINE.B | choline degradation I | Not detected | -0.175 | -0.369 | 0.020 | 0.079 |
| COBALSYN. | superpathway of adenosylcobalamin salvage from | Not detected | -0.025 | -0.191 | 0.140 | 0.764 |
| CODH.PWY | reductive acetyl coenzyme A pathway I (homoac | Not detected | -0.115 | -0.221 | -0.008 | 0.036 |
| COLANSYN | colanic acid building blocks biosynthesis | Not detected | -0.063 | -0.193 | 0.068 | 0.347 |
| DAPLYSINE | L-lysine biosynthesis I | Not detected | -0.180 | -0.393 | 0.033 | 0.098 |
| DARABCAT | D-arabinose degradation I | Not detected | -0.121 | -0.355 | 0.113 | 0.310 |
| DENITRIFIC | nitrate reduction I (denitrification) | Not detected | -0.011 | -0.202 | 0.180 | 0.912 |
| DETOX1.PW | superoxide radicals degradation | Not detected | -0.179 | -0.393 | 0.035 | 0.101 |
| DETOX1.PW | reactive oxygen species degradation | Not detected | -0.191 | -0.410 | 0.028 | 0.087 |
| DHGLUCON | glucose degradation (oxidative) | Not detected | -0.084 | -0.299 | 0.132 | 0.447 |
| DISSULFRE | dissimilatory sulfate reduction I (to hydrogen suf | Not detected | -0.079 | -0.293 | 0.135 | 0.470 |
| DTDPRHAM | dTDP-L-rhamnose biosynthesis | Not detected | 0.016 | -0.054 | 0.085 | 0.661 |
| ECASYN.PW | enterobacterial common antigen biosynthesis | Not detected | -0.007 | -0.231 | 0.217 | 0.953 |
| ETOH.ACET | ethanol degradation I | Not detected | -0.343 | -0.582 | -0.104 | 0.005 |
| FAO.PWY | fatty acid &beta;-oxidation I | Not detected | -0.098 | -0.369 | 0.173 | 0.478 |
| FASYN.ELO | fatty acid elongation -- saturated | Not detected | 0.181 | -0.057 | 0.420 | 0.137 |
| FERMENTA | mixed acid fermentation | Not detected | -0.014 | -0.080 | 0.052 | 0.673 |
| FHLMULTI. | NA | Not detected | -0.058 | -0.271 | 0.155 | 0.593 |

| FUCCAT.PW | fucose degradation | Not detected | -0.121 | -0.355 | 0.113 | 0.310 |
| --- | --- | --- | --- | --- | --- | --- |
| GALACTAR | D-galactarate degradation I | Not detected | -0.066 | -0.200 | 0.068 | 0.333 |
| GALACTCA | D-galactonate degradation | Not detected | -0.058 | -0.282 | 0.167 | 0.615 |
| GALACTUR | D-galacturonate degradation I | Not detected | 0.001 | -0.067 | 0.069 | 0.982 |
| GCVMULTI. | NA | Not detected | 0.049 | -0.125 | 0.223 | 0.583 |
| GDPRHAMS | GDP-D-rhamnose biosynthesis | Not detected | 0.301 | 0.067 | 0.534 | 0.012 |
| GLUCARDE | <i>D</i>-glucarate degradation I | Not detected | -0.062 | -0.201 | 0.078 | 0.386 |
| GLUCONEO | gluconeogenesis I | Not detected | 0.012 | -0.055 | 0.079 | 0.733 |
| GLUCOSE1P | glucose and glucose-1-phosphate degradation | Not detected | 0.113 | 0.009 | 0.216 | 0.034 |
| GLUDEG.I.P | GABA shunt | Not detected | -0.014 | -0.213 | 0.185 | 0.889 |
| GLUTATHIO | glutathione biosynthesis | Not detected | 0.026 | -0.159 | 0.210 | 0.785 |
| GLUTORN.P | L-ornithine biosynthesis I | Not detected | 0.172 | -0.036 | 0.380 | 0.104 |
| GLYCLEAV. | glycine cleavage | Not detected | 0.049 | -0.125 | 0.223 | 0.583 |
| GLYCOCAT | glycogen degradation I | Not detected | 0.085 | -0.015 | 0.185 | 0.094 |
| GLYCOGEN | glycogen biosynthesis I (from ADP-D-Glucose) | Not detected | 0.033 | -0.055 | 0.121 | 0.457 |
| GLYCOLAT | glycolate and glyoxylate degradation I | Not detected | -0.037 | -0.250 | 0.176 | 0.735 |
| GLYCOLYSI | glycolysis I (from glucose 6-phosphate) | Not detected | 0.007 | -0.060 | 0.073 | 0.841 |
| GLYCOLYSI | superpathway of glycolysis and the Entner-Doud | Not detected | 0.016 | -0.174 | 0.207 | 0.867 |
| GLYOXDEG | glycolate and glyoxylate degradation II | Not detected | -0.002 | -0.221 | 0.217 | 0.984 |
| GLYOXYLA | glyoxylate cycle | Not detected | -0.022 | -0.097 | 0.052 | 0.561 |
| HCAMHPDE | 3-phenylpropanoate and 3-(3-hydroxyphenyl)pro | Not detected | -0.148 | -0.338 | 0.041 | 0.125 |
| HEME.BIOS | heme <i>b</i> biosynthesis I (aerobic) | Not detected | 0.140 | -0.075 | 0.354 | 0.201 |
| HEMESYN2. | heme <i>b</i> biosynthesis II (anaerobic) | Not detected | 0.186 | -0.031 | 0.402 | 0.093 |
| HISDEG.PW | L-histidine degradation I | Not detected | 0.028 | -0.170 | 0.227 | 0.780 |
| HISHP.PWY | L-histidine degradation VI | Not detected | 0.028 | -0.170 | 0.227 | 0.780 |
| HISTSYN.P | L-histidine biosynthesis | Not detected | -0.003 | -0.074 | 0.068 | 0.933 |
| HOMOSER. | L-methionine biosynthesis I | Not detected | -0.034 | -0.102 | 0.034 | 0.331 |
| HOMOSER.T | L-threonine biosynthesis | Not detected | -0.018 | -0.241 | 0.204 | 0.872 |
| HOMOSERS | L-homoserine biosynthesis | Not detected | -0.205 | -0.416 | 0.005 | 0.056 |
| HSERMETA | L-methionine biosynthesis III | Not detected | -0.032 | -0.102 | 0.038 | 0.369 |
| ILEUSYN.P | L-isoleucine biosynthesis I (from threonine) | Not detected | 0.002 | -0.082 | 0.085 | 0.964 |
| KDO.LIPAS | (Kdo)<sub>2</sub>-lipid A biosynthesis I | Not detected | -0.055 | -0.243 | 0.133 | 0.568 |
| KDO.NAGLI | superpathway of (Kdo)<SUB>2</SUB>-lipid A | Not detected | -0.035 | -0.246 | 0.177 | 0.748 |
| KDOSYN.PW | Kdo transfer to lipid IV<sub>A</sub> I | Not detected | -0.006 | -0.226 | 0.214 | 0.956 |
| KETOGLUC | ketogluconate metabolism | Not detected | 0.044 | -0.138 | 0.226 | 0.635 |
| LACTOSEC | lactose and galactose degradation I | Not detected | -0.102 | -0.324 | 0.119 | 0.365 |
| LARABITOL | xylitol degradation | Not detected | -0.046 | -0.284 | 0.191 | 0.703 |
| LEUSYN.PW | L-leucine biosynthesis | Not detected | -0.078 | -0.191 | 0.035 | 0.179 |
| LIPA.CORES | lipid A-core biosynthesis (<i>E. coli</i> K-12) | Not detected | 0.081 | -0.155 | 0.317 | 0.503 |
| LYSDEGII.P | L-lysine degradation III | Not detected | 0.029 | -0.140 | 0.199 | 0.735 |
| LYXMET.PW | L-lyxose degradation | Not detected | -0.002 | -0.068 | 0.063 | 0.944 |
| MALATE.AS | L-aspartate degradation II | Not detected | -0.012 | -0.254 | 0.231 | 0.926 |
| MANNCAT. | D-mannose degradation | Not detected | -0.026 | -0.228 | 0.175 | 0.799 |
| MANNIDEG | mannitol degradation I | Not detected | -0.090 | -0.304 | 0.124 | 0.408 |

| MENAQUIN | menaquinol-8 biosynthesis | Not detected | 0.146 | -0.071 | 0.363 | 0.186 |
| --- | --- | --- | --- | --- | --- | --- |
| METH.ACET | methanogenesis from acetate | Not detected | -0.014 | -0.077 | 0.048 | 0.648 |
| NAGLIPASY | lipid IV<sub>A</sub> biosynthesis | Not detected | 0.289 | 0.066 | 0.513 | 0.011 |
| NONMEVIP | methylerythritol phosphate pathway I | Not detected | -0.037 | -0.110 | 0.037 | 0.326 |
| NONOXIPE | pentose phosphate pathway (non-oxidative branc | Not detected | 0.113 | -0.033 | 0.260 | 0.130 |
| NPGLUCAT. | Entner-Doudoroff pathway II (non-phosphorylati | Not detected | -0.022 | -0.201 | 0.157 | 0.812 |
| OANTIGEN. | <i>O</i>-antigen building blocks biosynthesis (< | Not detected | -0.030 | -0.112 | 0.051 | 0.465 |
| OXIDATIVE | pentose phosphate pathway (oxidative branch) I | Not detected | -0.091 | -0.230 | 0.048 | 0.198 |
| P101.PWY | ectoine biosynthesis | Not detected | -0.206 | -0.416 | 0.005 | 0.056 |
| P105.PWY | TCA cycle IV (2-oxoglutarate decarboxylase) | Not detected | -0.017 | -0.088 | 0.054 | 0.639 |
| P108.PWY | pyruvate fermentation to propanoate I | Not detected | -0.113 | -0.200 | -0.026 | 0.011 |
| P121.PWY | adenine and adenosine salvage I | Not detected | 0.156 | -0.057 | 0.369 | 0.151 |
| P122.PWY | heterolactic fermentation | Not detected | -0.006 | -0.069 | 0.056 | 0.846 |
| P124.PWY | Bifidobacterium shunt | Not detected | 0.006 | -0.055 | 0.067 | 0.846 |
| P161.PWY | acetylene degradation (anaerobic) | Not detected | -0.014 | -0.072 | 0.044 | 0.627 |
| P162.PWY | L-glutamate degradation V (via hydroxyglutarate | Not detected | 0.002 | -0.085 | 0.089 | 0.960 |
| P163.PWY | L-lysine fermentation to acetate and butanoate | Not detected | -0.012 | -0.077 | 0.053 | 0.715 |
| P164.PWY | purine nucleobases degradation I (anaerobic) | Not detected | -0.054 | -0.119 | 0.010 | 0.100 |
| P181.PWY | nicotine degradation I (pyridine pathway) | Not detected | -0.009 | -0.198 | 0.180 | 0.925 |
| P185.PWY | formaldehyde assimilation III (dihydroxyacetone | Not detected | 0.002 | -0.063 | 0.067 | 0.947 |
| P2.PWY | citrate lyase activation | Not detected | 0.033 | -0.157 | 0.223 | 0.733 |
| P21.PWY | pentose phosphate pathway (partial) | Not detected | 0.113 | -0.033 | 0.260 | 0.130 |
| P23.PWY | reductive TCA cycle I | Not detected | -0.024 | -0.102 | 0.055 | 0.556 |
| P3.PWY | gallate degradation III (anaerobic) | Not detected | -0.008 | -0.072 | 0.056 | 0.804 |
| P341.PWY | glycolysis V (Pyrococcus) | Not detected | 0.009 | -0.060 | 0.078 | 0.790 |
| P42.PWY | incomplete reductive TCA cycle | Not detected | -0.020 | -0.119 | 0.079 | 0.693 |
| P562.PWY | <I>myo</I>-inositol degradation I | Not detected | 0.095 | -0.042 | 0.233 | 0.174 |
| PEPTIDOGL | peptidoglycan biosynthesis I (<I>meso</I>-diam | Not detected | 0.123 | -0.082 | 0.328 | 0.238 |
| PHOSLIPSY | superpathway of phospholipid biosynthesis II (pl | Not detected | 0.051 | -0.147 | 0.248 | 0.616 |
| PHOSPHON | 2-aminoethylphosphonate degradation I | Not detected | -0.326 | -0.554 | -0.097 | 0.005 |
| PPGPPMET. | ppGpp biosynthesis | Not detected | 0.065 | 0.005 | 0.124 | 0.033 |
| PUTDEG.PW | putrescine degradation I | Not detected | -0.080 | -0.248 | 0.088 | 0.351 |
| PWY.1042 | glycolysis IV (plant cytosol) | Not detected | 0.014 | -0.052 | 0.080 | 0.684 |
| PWY.1186 | L-homomethionine biosynthesis | Not detected | 0.146 | -0.059 | 0.351 | 0.163 |
| PWY.1269 | CMP-3-deoxy-D-<I>manno</I>-octulosonate bi | Not detected | 0.288 | 0.069 | 0.507 | 0.010 |
| PWY.1281 | sulfoacetaldehyde degradation I | Not detected | -0.023 | -0.099 | 0.054 | 0.558 |
| PWY.1341 | phenylacetate degradation II (anaerobic) | Not detected | -0.121 | -0.232 | -0.011 | 0.032 |
| PWY.1361 | benzoyl-CoA degradation I (aerobic) | Not detected | -0.006 | -0.204 | 0.193 | 0.954 |
| PWY.1622 | formaldehyde assimilation I (serine pathway) | Not detected | -0.073 | -0.212 | 0.066 | 0.306 |
| PWY.1722 | formate assimilation into 5,10-methylenetetrahyd | Not detected | -0.115 | -0.221 | -0.008 | 0.036 |
| PWY.1861 | formaldehyde assimilation II (assimilatory RuMP | Not detected | 0.014 | -0.064 | 0.091 | 0.730 |
| PWY.2 | putrescine degradation IV | Not detected | -0.080 | -0.248 | 0.088 | 0.351 |
| PWY.2161 | folate polyglutamylation | Not detected | 0.105 | -0.117 | 0.326 | 0.354 |
| PWY.2201 | folate transformations I | Not detected | -0.043 | -0.113 | 0.026 | 0.224 |

| PWY.2221 | Entner-Doudoroff pathway III (semi-phosphoryla | Not detected | 0.026 | -0.039 | 0.090 | 0.432 |
| --- | --- | --- | --- | --- | --- | --- |
| PWY.2361 | 3-oxoadipate degradation | Not detected | 0.012 | -0.151 | 0.175 | 0.886 |
| PWY.2661 | trehalose biosynthesis V | Not detected | 0.056 | -0.184 | 0.295 | 0.648 |
| PWY.2722 | trehalose degradation IV | Not detected | 0.135 | -0.051 | 0.321 | 0.154 |
| PWY.2723 | trehalose degradation V | Not detected | 0.113 | 0.002 | 0.225 | 0.046 |
| PWY.2781 | <i>cis</i>-zeatin biosynthesis | Not detected | 0.040 | -0.161 | 0.241 | 0.698 |
| PWY.2941 | L-lysine biosynthesis II | Not detected | -0.205 | -0.416 | 0.005 | 0.056 |
| PWY.2942 | L-lysine biosynthesis III | Not detected | -0.205 | -0.416 | 0.005 | 0.056 |
| PWY.3 | putrescine degradation V | Not detected | -0.074 | -0.243 | 0.095 | 0.394 |
| PWY.3801 | sucrose degradation II (sucrose synthase) | Not detected | 0.000 | -0.063 | 0.062 | 0.991 |
| PWY.3821 | D-galactose detoxification | Not detected | -0.065 | -0.172 | 0.041 | 0.231 |
| PWY.3841 | folate transformations II | Not detected | -0.042 | -0.111 | 0.027 | 0.232 |
| PWY.3861 | mannitol degradation II | Not detected | -0.170 | -0.381 | 0.042 | 0.116 |
| PWY.3881 | mannitol biosynthesis | Not detected | -0.085 | -0.297 | 0.127 | 0.431 |
| PWY.3941 | &beta;-alanine biosynthesis II | Not detected | -0.113 | -0.337 | 0.112 | 0.326 |
| PWY.4041 | &gamma;-glutamyl cycle | Not detected | 0.102 | -0.115 | 0.319 | 0.356 |
| PWY.4101 | D-sorbitol degradation I | Not detected | -0.109 | -0.320 | 0.103 | 0.315 |
| PWY.43 | putrescine biosynthesis II | Not detected | -0.037 | -0.140 | 0.067 | 0.486 |
| PWY.4321 | L-glutamate degradation IV | Not detected | -0.041 | -0.239 | 0.157 | 0.686 |
| PWY.4361 | <i>S</i>-methyl-5-thio-&alpha;-D-ribose 1-phos | Not detected | 0.106 | -0.114 | 0.327 | 0.345 |
| PWY.4381 | fatty acid biosynthesis initiation I | Not detected | 0.258 | 0.052 | 0.465 | 0.014 |
| PWY.4702 | phytate degradation I | Not detected | 0.051 | -0.163 | 0.265 | 0.640 |
| PWY.4983 | nitric oxide biosynthesis II (mammals) | Not detected | -0.037 | -0.150 | 0.076 | 0.519 |
| PWY.4984 | urea cycle | Not detected | 0.038 | -0.054 | 0.131 | 0.419 |
| PWY.5022 | 4-aminobutanoate degradation V | Not detected | 0.015 | -0.066 | 0.095 | 0.723 |
| PWY.5028 | L-histidine degradation II | Not detected | 0.034 | -0.166 | 0.233 | 0.741 |
| PWY.5030 | L-histidine degradation III | Not detected | -0.111 | -0.213 | -0.009 | 0.033 |
| PWY.5041 | <i>S</i>-adenosyl-L-methionine cycle II | Not detected | 0.057 | -0.167 | 0.280 | 0.620 |
| PWY.5054 | D-sorbitol biosynthesis I | Not detected | -0.008 | -0.078 | 0.062 | 0.824 |
| PWY.5097 | L-lysine biosynthesis VI | Not detected | -0.205 | -0.416 | 0.005 | 0.056 |
| PWY.5101 | L-isoleucine biosynthesis II | Not detected | -0.065 | -0.276 | 0.146 | 0.544 |
| PWY.5103 | L-isoleucine biosynthesis III | Not detected | 0.002 | -0.082 | 0.085 | 0.964 |
| PWY.5104 | L-isoleucine biosynthesis IV | Not detected | -0.116 | -0.342 | 0.109 | 0.313 |
| PWY.5122 | geranyl diphosphate biosynthesis | Not detected | -0.082 | -0.276 | 0.111 | 0.405 |
| PWY.5123 | <i>trans, trans</i>-farnesyl diphosphate biosynth | Not detected | -0.078 | -0.306 | 0.149 | 0.500 |
| PWY.5136 | fatty acid &beta;-oxidation II (peroxisome) | Not detected | -0.074 | -0.323 | 0.176 | 0.561 |
| PWY.5137 | fatty acid &beta;-oxidation III (unsaturated, odd | Not detected | -0.080 | -0.274 | 0.114 | 0.417 |
| PWY.5138 | unsaturated, even numbered fatty acid &beta;-oxi | Not detected | -0.099 | -0.322 | 0.123 | 0.381 |
| PWY.5154 | L-arginine biosynthesis III (via <i>N</i>-acetyl- | Not detected | -0.020 | -0.130 | 0.089 | 0.715 |
| PWY.5162 | 2-hydroxypenta-2,4-dienoate degradation | Not detected | -0.287 | -0.515 | -0.058 | 0.014 |
| PWY.5177 | glutaryl-CoA degradation | Not detected | -0.011 | -0.132 | 0.109 | 0.852 |
| PWY.5188 | tetrapyrrole biosynthesis I (from glutamate) | Not detected | 0.076 | -0.027 | 0.179 | 0.148 |
| PWY.5189 | tetrapyrrole biosynthesis II (from glycine) | Not detected | 0.130 | -0.023 | 0.282 | 0.095 |
| PWY.5265 | peptidoglycan biosynthesis II (staphylococci) | Not detected | 0.001 | -0.120 | 0.122 | 0.988 |

| PWY.5269 | cardiolipin biosynthesis II | Not detected | 0.056 | -0.141 | 0.254 | 0.576 |
| --- | --- | --- | --- | --- | --- | --- |
| PWY.5280 | L-lysine degradation IV | Not detected | 0.016 | -0.158 | 0.190 | 0.859 |
| PWY.5306 | superpathway of thiosulfate metabolism (<i>Des | Not detected | -0.079 | -0.293 | 0.135 | 0.470 |
| PWY.5316 | nicotine biosynthesis | Not detected | -0.157 | -0.411 | 0.097 | 0.226 |
| PWY.5344 | L-homocysteine biosynthesis | Not detected | 0.027 | -0.048 | 0.101 | 0.485 |
| PWY.5352 | thiosulfate disproportionation II (cytochrome) | Not detected | -0.174 | -0.376 | 0.029 | 0.093 |
| PWY.5360 | superpathway of tetrathionate reduction (<i>Salm | Not detected | -0.079 | -0.293 | 0.135 | 0.470 |
| PWY.5381 | pyridine nucleotide cycling (plants) | Not detected | -0.029 | -0.238 | 0.179 | 0.782 |
| PWY.5384 | sucrose degradation IV (sucrose phosphorylase) | Not detected | 0.000 | -0.062 | 0.063 | 0.989 |
| PWY.5386 | methylglyoxal degradation I | Not detected | -0.021 | -0.230 | 0.189 | 0.846 |
| PWY.5392 | reductive TCA cycle II | Not detected | -0.027 | -0.107 | 0.053 | 0.502 |
| PWY.5394 | betalamic acid biosynthesis | Not detected | -0.087 | -0.241 | 0.067 | 0.270 |
| PWY.5436 | L-threonine degradation IV | Not detected | -0.326 | -0.554 | -0.097 | 0.005 |
| PWY.5437 | L-threonine degradation I | Not detected | -0.198 | -0.423 | 0.027 | 0.085 |
| PWY.5453 | methylglyoxal degradation III | Not detected | -0.044 | -0.109 | 0.020 | 0.178 |
| PWY.5458 | methylglyoxal degradation V | Not detected | 0.174 | -0.030 | 0.378 | 0.096 |
| PWY.5459 | methylglyoxal degradation IV | Not detected | 0.179 | -0.025 | 0.384 | 0.086 |
| PWY.5480 | pyruvate fermentation to ethanol I | Not detected | -0.343 | -0.582 | -0.104 | 0.005 |
| PWY.5482 | pyruvate fermentation to acetate II | Not detected | -0.014 | -0.077 | 0.048 | 0.648 |
| PWY.5484 | glycolysis II (from fructose 6-phosphate) | Not detected | 0.014 | -0.051 | 0.080 | 0.663 |
| PWY.5485 | pyruvate fermentation to acetate IV | Not detected | -0.014 | -0.077 | 0.048 | 0.648 |
| PWY.5486 | pyruvate fermentation to ethanol II | Not detected | -0.123 | -0.320 | 0.074 | 0.220 |
| PWY.5497 | purine nucleobases degradation II (anaerobic) | Not detected | -0.035 | -0.099 | 0.028 | 0.275 |
| PWY.5509 | adenosylcobalamin biosynthesis from adenosylco | Not detected | -0.025 | -0.191 | 0.140 | 0.764 |
| PWY.5512 | UDP-<i>N</i>-acetyl-D-galactosamine biosynth | Not detected | -0.228 | -0.494 | 0.038 | 0.094 |
| PWY.5514 | UDP-<i>N</i>-acetyl-D-galactosamine biosynth | Not detected | -0.014 | -0.085 | 0.057 | 0.702 |
| PWY.5523 | 5,6-dimethylbenzimidazole biosynthesis I (aerobi | Not detected | 0.025 | -0.166 | 0.217 | 0.794 |
| PWY.5531 | 3,8-divinyl-chlorophyllide <i>a</i> biosynthesis | Not detected | 0.163 | -0.053 | 0.378 | 0.140 |
| PWY.5532 | nucleoside and nucleotide degradation (archaea) | Not detected | 0.111 | -0.054 | 0.276 | 0.187 |
| PWY.5536 | acetate formation from acetyl-CoA (succinate) | Not detected | 0.007 | -0.137 | 0.151 | 0.925 |
| PWY.561 | superpathway of glyoxylate cycle and fatty acid d | Not detected | 0.022 | -0.047 | 0.091 | 0.524 |
| PWY.5659 | GDP-mannose biosynthesis | Not detected | -0.003 | -0.068 | 0.062 | 0.926 |
| PWY.5661 | GDP-glucose biosynthesis | Not detected | 0.113 | 0.002 | 0.225 | 0.046 |
| PWY.5663 | erythro-tetrahydrobiopterin biosynthesis I | Not detected | 0.027 | -0.096 | 0.150 | 0.665 |
| PWY.5664 | <i>erythro</i>-tetrahydrobiopterin biosynthesis I | Not detected | 0.027 | -0.096 | 0.150 | 0.665 |
| PWY.5667 | CDP-diacylglycerol biosynthesis I | Not detected | -0.066 | -0.240 | 0.109 | 0.461 |
| PWY.5668 | cardiolipin biosynthesis I | Not detected | -0.070 | -0.297 | 0.158 | 0.548 |
| PWY.5669 | phosphatidylserine and phosphatidylethanolamin | Not detected | 0.051 | -0.147 | 0.248 | 0.616 |
| PWY.5676 | acetyl-CoA fermentation to butanoate II | Not detected | -0.003 | -0.083 | 0.076 | 0.935 |
| PWY.5677 | succinate fermentation to butanoate | Not detected | 0.017 | -0.067 | 0.101 | 0.691 |
| PWY.5686 | UMP biosynthesis I | Not detected | -0.024 | -0.094 | 0.047 | 0.514 |
| PWY.5690 | TCA cycle II (plants and fungi) | Not detected | -0.028 | -0.108 | 0.052 | 0.493 |
| PWY.5692 | allantoin degradation to glyoxylate II | Not detected | 0.063 | -0.128 | 0.255 | 0.518 |
| PWY.5698 | allantoin degradation to ureidoglycolate II (amm | Not detected | 0.063 | -0.128 | 0.255 | 0.518 |

| PWY.5723 | Rubisco shunt | Not detected | 0.050 | -0.021 | 0.121 | 0.171 |
| --- | --- | --- | --- | --- | --- | --- |
| PWY.5738 | GDP-6-deoxy-D-talose biosynthesis | Not detected | 0.301 | 0.067 | 0.534 | 0.012 |
| PWY.5739 | GDP-D-perosamine biosynthesis | Not detected | 0.301 | 0.067 | 0.534 | 0.012 |
| PWY.5740 | GDP-L-colitose biosynthesis | Not detected | 0.301 | 0.067 | 0.534 | 0.012 |
| PWY.5741 | ethylmalonyl-CoA pathway | Not detected | -0.056 | -0.239 | 0.128 | 0.553 |
| PWY.5743 | 3-hydroxypropanoate cycle | Not detected | -0.029 | -0.120 | 0.062 | 0.531 |
| PWY.5744 | glyoxylate assimilation | Not detected | -0.030 | -0.121 | 0.062 | 0.526 |
| PWY.5747 | 2-methylcitrate cycle II | Not detected | -0.123 | -0.366 | 0.120 | 0.321 |
| PWY.5757 | fosfomycin biosynthesis | Not detected | 0.015 | -0.244 | 0.275 | 0.909 |
| PWY.5785 | di-<i>trans</i>,poly-<i>cis</i>-undecaprenyl ph | Not detected | -0.002 | -0.199 | 0.194 | 0.980 |
| PWY.5789 | 3-hydroxypropanoate/4-hydroxybutanate cycle | Not detected | -0.013 | -0.092 | 0.066 | 0.751 |
| PWY.5794 | malonate degradation I (biotin-independent) | Not detected | 0.066 | -0.141 | 0.272 | 0.534 |
| PWY.5796 | malonate decarboxylase activation | Not detected | 0.019 | -0.145 | 0.182 | 0.823 |
| PWY.5837 | 2-carboxy-1,4-naphthoquinol biosynthesis | Not detected | -0.039 | -0.113 | 0.036 | 0.306 |
| PWY.5852 | demethylmenaquinol-8 biosynthesis I | Not detected | 0.011 | -0.167 | 0.189 | 0.901 |
| PWY.5870 | ubiquinol-8 biosynthesis (eukaryotic) | Not detected | 0.078 | -0.148 | 0.304 | 0.498 |
| PWY.5875 | staphyloxanthin biosynthesis | Not detected | -0.238 | -0.471 | -0.004 | 0.046 |
| PWY.5901 | 2,3-dihydroxybenzoate biosynthesis | Not detected | -0.034 | -0.201 | 0.133 | 0.688 |
| PWY.5913 | partial TCA cycle (obligate autotrophs) | Not detected | -0.024 | -0.102 | 0.054 | 0.549 |
| PWY.5921 | glutaminyl-tRNA<sup>gln</sup> biosynthesis vi | Not detected | 0.110 | -0.129 | 0.349 | 0.366 |
| PWY.5940 | streptomycin biosynthesis | Not detected | 0.108 | -0.001 | 0.216 | 0.052 |
| PWY.5941 | glycogen degradation II | Not detected | 0.113 | 0.002 | 0.225 | 0.046 |
| PWY.5971 | palmitate biosynthesis II (bacteria and plants) | Not detected | 0.043 | -0.101 | 0.187 | 0.555 |
| PWY.5981 | CDP-diacylglycerol biosynthesis III | Not detected | -0.196 | -0.368 | -0.025 | 0.025 |
| PWY.5994 | palmitate biosynthesis I (animals and fungi) | Not detected | 0.017 | -0.144 | 0.177 | 0.839 |
| PWY.6012 | acyl carrier protein metabolism | Not detected | 0.041 | -0.116 | 0.198 | 0.609 |
| PWY.6019 | pseudouridine degradation | Not detected | -0.006 | -0.197 | 0.184 | 0.947 |
| PWY.6053 | dimethylsulfoniopropanoate biosynthesis III (alg | Not detected | 0.146 | -0.059 | 0.351 | 0.163 |
| PWY.6060 | malonate degradation II (biotin-dependent) | Not detected | -0.139 | -0.264 | -0.015 | 0.028 |
| PWY.6121 | 5-aminoimidazole ribonucleotide biosynthesis I | Not detected | 0.094 | 0.014 | 0.175 | 0.022 |
| PWY.6122 | 5-aminoimidazole ribonucleotide biosynthesis II | Not detected | 0.027 | -0.037 | 0.090 | 0.408 |
| PWY.6123 | inosine-5'-phosphate biosynthesis I | Not detected | 0.062 | -0.068 | 0.192 | 0.350 |
| PWY.6124 | inosine-5'-phosphate biosynthesis II | Not detected | 0.062 | -0.068 | 0.192 | 0.350 |
| PWY.6138 | CMP-<i>N</i>-acetylneuraminate biosynthesis I | Not detected | -0.003 | -0.180 | 0.175 | 0.976 |
| PWY.6140 | CMP-2-keto-3-deoxy-D-<i>glycero</i>-D-<i>ga | Not detected | -0.315 | -0.559 | -0.071 | 0.011 |
| PWY.6142 | gluconeogenesis II (<i>Methanobacterium therm | Not detected | 0.010 | -0.059 | 0.079 | 0.784 |
| PWY.6143 | CMP-pseudaminate biosynthesis | Not detected | -0.033 | -0.199 | 0.132 | 0.691 |
| PWY.6146 | <i>Methanobacterium thermoautotrophicum</i> | Not detected | 0.008 | -0.206 | 0.223 | 0.940 |
| PWY.6147 | 6-hydroxymethyl-dihydropterin diphosphate bios | Not detected | 0.019 | -0.085 | 0.122 | 0.726 |
| PWY.6148 | tetrahydromethanopterin biosynthesis | Not detected | 0.122 | -0.053 | 0.296 | 0.173 |
| PWY.6151 | <i>S</i>-adenosyl-L-methionine cycle I | Not detected | 0.132 | -0.100 | 0.364 | 0.265 |
| PWY.6153 | autoinducer AI-2 biosynthesis I | Not detected | 0.214 | 0.040 | 0.387 | 0.016 |
| PWY.6154 | autoinducer AI-2 biosynthesis II (<i>Vibrio</i>) | Not detected | 0.214 | 0.040 | 0.387 | 0.016 |
| PWY.6160 | 3-dehydroquinate biosynthesis II (archaea) | Not detected | -0.187 | -0.397 | 0.023 | 0.081 |

| PWY.6163 | chorismate biosynthesis from 3-dehydroquinate | Not detected | 0.041 | -0.118 | 0.201 | 0.610 |
| --- | --- | --- | --- | --- | --- | --- |
| PWY.6164 | 3-dehydroquinate biosynthesis I | Not detected | 0.139 | -0.068 | 0.346 | 0.187 |
| PWY.6167 | flavin biosynthesis II (archaea) | Not detected | 0.007 | -0.060 | 0.074 | 0.837 |
| PWY.6168 | flavin biosynthesis III (fungi) | Not detected | 0.006 | -0.063 | 0.075 | 0.869 |
| PWY.621 | sucrose degradation III (sucrose invertase) | Not detected | -0.008 | -0.074 | 0.058 | 0.803 |
| PWY.622 | starch biosynthesis | Not detected | 0.002 | -0.060 | 0.064 | 0.949 |
| PWY.6269 | superpathway of adenosylcobalamin salvage from | Not detected | -0.017 | -0.201 | 0.167 | 0.858 |
| PWY.6277 | superpathway of 5-aminoimidazole ribonucleotid | Not detected | 0.027 | -0.037 | 0.090 | 0.408 |
| PWY.6317 | D-galactose degradation I (Leloir pathway) | Not detected | -0.003 | -0.070 | 0.064 | 0.929 |
| PWY.6318 | L-phenylalanine degradation IV (mammalian, via | Not detected | -0.121 | -0.232 | -0.011 | 0.032 |
| PWY.6322 | phosphinothricin tripeptide biosynthesis | Not detected | 0.010 | -0.225 | 0.246 | 0.931 |
| PWY.6328 | L-lysine degradation X | Not detected | 0.016 | -0.158 | 0.190 | 0.859 |
| PWY.6333 | acetaldehyde biosynthesis I | Not detected | -0.123 | -0.320 | 0.074 | 0.220 |
| PWY.6383 | mono-<i>trans</i>, poly-<i>cis</i> decaprenyl p | Not detected | -0.066 | -0.272 | 0.139 | 0.528 |
| PWY.6386 | UDP-<i>N</i>-acetylmuramoyl-pentapeptide bio | Not detected | 0.003 | -0.059 | 0.064 | 0.936 |
| PWY.6387 | UDP-<i>N</i>-acetylmuramoyl-pentapeptide bio | Not detected | 0.008 | -0.050 | 0.066 | 0.794 |
| PWY.6397 | mycolyl-arabinogalactan-peptidoglycan complex | Not detected | -0.028 | -0.109 | 0.053 | 0.497 |
| PWY.6406 | salicylate biosynthesis I | Not detected | -0.034 | -0.201 | 0.133 | 0.688 |
| PWY.6416 | quinate degradation II | Not detected | 0.040 | -0.159 | 0.239 | 0.693 |
| PWY.6461 | peptidoglycan cross-bridge biosynthesis II (E. fae | Not detected | 0.324 | 0.081 | 0.567 | 0.009 |
| PWY.6471 | peptidoglycan biosynthesis IV (Enterococcus fae | Not detected | 0.002 | -0.118 | 0.122 | 0.971 |
| PWY.6473 | 4-aminobutanoate degradation IV | Not detected | -0.108 | -0.276 | 0.060 | 0.208 |
| PWY.6478 | GDP-D-<i>glycero</i>-&alpha;-D-<i>manno</i | Not detected | 0.002 | -0.196 | 0.200 | 0.987 |
| PWY.6497 | D-galactarate degradation II | Not detected | -0.066 | -0.277 | 0.145 | 0.537 |
| PWY.6499 | D-glucarate degradation II | Not detected | -0.235 | -0.443 | -0.026 | 0.027 |
| PWY.6507 | 4-deoxy-L-<i>threo</i>-hex-4-enopyranuronate | Not detected | 0.006 | -0.062 | 0.074 | 0.861 |
| PWY.6517 | <i>N</i>-acetylglucosamine degradation II | Not detected | -0.049 | -0.129 | 0.032 | 0.238 |
| PWY.6518 | glycocholate metabolism (bacteria) | Not detected | 0.219 | -0.012 | 0.449 | 0.063 |
| PWY.6519 | 8-amino-7-oxononanoate biosynthesis I | Not detected | -0.004 | -0.186 | 0.179 | 0.968 |
| PWY.6527 | stachyose degradation | Not detected | 0.000 | -0.079 | 0.078 | 0.994 |
| PWY.6531 | mannitol cycle | Not detected | -0.145 | -0.342 | 0.053 | 0.150 |
| PWY.6535 | 4-aminobutanoate degradation I | Not detected | -0.041 | -0.239 | 0.157 | 0.686 |
| PWY.6536 | 4-aminobutanoate degradation III | Not detected | -0.028 | -0.201 | 0.144 | 0.747 |
| PWY.6537 | 4-aminobutanoate degradation II | Not detected | 0.029 | -0.141 | 0.199 | 0.737 |
| PWY.6545 | pyrimidine deoxyribonucleotides <i>de novo</i> | Not detected | 0.052 | -0.029 | 0.134 | 0.211 |
| PWY.6549 | L-glutamine biosynthesis III | Not detected | -0.027 | -0.109 | 0.054 | 0.510 |
| PWY.6556 | pyrimidine ribonucleosides salvage II | Not detected | 0.039 | -0.126 | 0.204 | 0.645 |
| PWY.6559 | spermidine biosynthesis II | Not detected | 0.011 | -0.066 | 0.088 | 0.783 |
| PWY.6562 | norspermidine biosynthesis | Not detected | -0.200 | -0.403 | 0.003 | 0.053 |
| PWY.6572 | chondroitin sulfate degradation I (bacterial) | Not detected | -0.289 | -0.508 | -0.069 | 0.010 |
| PWY.6575 | juvenile hormone III biosynthesis I | Not detected | 0.035 | -0.145 | 0.215 | 0.706 |
| PWY.6583 | pyruvate fermentation to butanol I | Not detected | -0.011 | -0.095 | 0.073 | 0.795 |
| PWY.6587 | pyruvate fermentation to ethanol III | Not detected | -0.343 | -0.582 | -0.104 | 0.005 |
| PWY.6596 | adenosine nucleotides degradation I | Not detected | 0.076 | -0.130 | 0.282 | 0.469 |

| PWY.66 | GDP-L-fucose biosynthesis I (from GDP-D-man | Not detected | 0.301 | 0.067 | 0.534 | 0.012 |
| --- | --- | --- | --- | --- | --- | --- |
| PWY.6605 | adenine and adenosine salvage II | Not detected | 0.177 | -0.037 | 0.390 | 0.105 |
| PWY.6606 | guanosine nucleotides degradation II | Not detected | 0.042 | -0.161 | 0.245 | 0.683 |
| PWY.6607 | guanosine nucleotides degradation I | Not detected | 0.078 | -0.110 | 0.265 | 0.416 |
| PWY.6608 | guanosine nucleotides degradation III | Not detected | 0.167 | -0.049 | 0.383 | 0.130 |
| PWY.6610 | adenine salvage | Not detected | 0.194 | -0.023 | 0.411 | 0.079 |
| PWY.6613 | tetrahydrofolate salvage from 5,10-methenyltetra | Not detected | -0.112 | -0.209 | -0.015 | 0.023 |
| PWY.6614 | tetrahydrofolate biosynthesis | Not detected | 0.136 | -0.059 | 0.331 | 0.171 |
| PWY.6620 | guanine and guanosine salvage | Not detected | 0.104 | -0.119 | 0.327 | 0.360 |
| PWY.6626 | cytidine-5'-diphosphate-glycerol biosynthesis | Not detected | 0.026 | -0.172 | 0.223 | 0.799 |
| PWY.6637 | sulfolactate degradation II | Not detected | -0.023 | -0.099 | 0.054 | 0.558 |
| PWY.6654 | phosphopantothenate biosynthesis III (archaebac | Not detected | 0.049 | -0.116 | 0.214 | 0.559 |
| PWY.6672 | <i>cis</i>-genanyl-CoA degradation | Not detected | -0.114 | -0.327 | 0.098 | 0.292 |
| PWY.6682 | dehydrophos biosynthesis | Not detected | 0.013 | -0.223 | 0.249 | 0.913 |
| PWY.6690 | cinnamate and 3-hydroxycinnamate degradation t | Not detected | -0.148 | -0.338 | 0.041 | 0.125 |
| PWY.6700 | queuosine biosynthesis | Not detected | 0.031 | -0.079 | 0.141 | 0.583 |
| PWY.6703 | preQ<sub>0</sub> biosynthesis | Not detected | 0.013 | -0.094 | 0.119 | 0.817 |
| PWY.6707 | gallate biosynthesis | Not detected | 0.040 | -0.159 | 0.239 | 0.693 |
| PWY.6708 | ubiquinol-8 biosynthesis (prokaryotic) | Not detected | 0.075 | -0.151 | 0.302 | 0.514 |
| PWY.6717 | (1,4)-&beta;-D-xylan degradation | Not detected | 0.194 | 0.014 | 0.373 | 0.035 |
| PWY.6724 | starch degradation II | Not detected | -0.070 | -0.286 | 0.146 | 0.527 |
| PWY.6728 | methylaspartate cycle | Not detected | -0.024 | -0.102 | 0.054 | 0.548 |
| PWY.6731 | starch degradation III | Not detected | 0.115 | 0.003 | 0.227 | 0.045 |
| PWY.6737 | starch degradation V | Not detected | 0.114 | 0.002 | 0.226 | 0.046 |
| PWY.6748 | nitrate reduction VII (denitrification) | Not detected | 0.049 | -0.169 | 0.268 | 0.660 |
| PWY.6749 | CMP-legionaminate biosynthesis I | Not detected | 0.123 | -0.067 | 0.313 | 0.205 |
| PWY.6771 | rhamnogalacturonan type I degradation II (bacter | Not detected | -0.165 | -0.309 | -0.022 | 0.024 |
| PWY.6772 | hydrogen production V | Not detected | -0.057 | -0.271 | 0.156 | 0.598 |
| PWY.6784 | cellulose and hemicellulose degradation (cellulol | Not detected | 0.198 | 0.017 | 0.380 | 0.032 |
| PWY.6786 | detoxification of reactive carbonyls in chloroplas | Not detected | -0.045 | -0.109 | 0.020 | 0.173 |
| PWY.6788 | cellulose degradation II (fungi) | Not detected | 0.037 | -0.159 | 0.233 | 0.711 |
| PWY.6799 | fatty acid biosynthesis (plant mitochondria) | Not detected | 0.273 | 0.066 | 0.480 | 0.010 |
| PWY.6808 | dTDP-D-forosamine biosynthesis | Not detected | 0.021 | -0.048 | 0.090 | 0.548 |
| PWY.6823 | molybdenum cofactor biosynthesis | Not detected | 0.152 | -0.059 | 0.363 | 0.158 |
| PWY.6839 | 2-aminoethylphosphonate biosynthesis | Not detected | 0.013 | -0.223 | 0.249 | 0.913 |
| PWY.6840 | homoglutathione biosynthesis | Not detected | 0.026 | -0.159 | 0.210 | 0.785 |
| PWY.6842 | glutathione-mediated detoxification II | Not detected | 0.016 | -0.168 | 0.200 | 0.868 |
| PWY.6854 | ethylene biosynthesis III (microbes) | Not detected | -0.030 | -0.255 | 0.194 | 0.790 |
| PWY.6855 | chitin degradation I (archaea) | Not detected | -0.005 | -0.203 | 0.192 | 0.958 |
| PWY.6859 | <i>all-trans</i>-farnesol biosynthesis | Not detected | -0.078 | -0.306 | 0.149 | 0.500 |
| PWY.6863 | pyruvate fermentation to hexanol (engineered) | Not detected | -0.029 | -0.119 | 0.060 | 0.519 |
| PWY.6871 | 3-methylbutanol biosynthesis (engineered) | Not detected | -0.078 | -0.191 | 0.035 | 0.179 |
| PWY.6883 | pyruvate fermentation to butanol II (engineered) | Not detected | -0.029 | -0.118 | 0.060 | 0.526 |
| PWY.6886 | 1-butanol autotrophic biosynthesis (engineered) | Not detected | 0.020 | -0.045 | 0.085 | 0.543 |

| PWY.6890 | 4-amino-2-methyl-5-diphosphomethylpyrimidine | Not detected | -0.028 | -0.111 | 0.055 | 0.507 |
| --- | --- | --- | --- | --- | --- | --- |
| PWY.6891 | thiazole biosynthesis II (aerobic bacteria) | Not detected | -0.010 | -0.082 | 0.062 | 0.782 |
| PWY.6892 | thiazole biosynthesis I (facultative anaerobic bact | Not detected | -0.002 | -0.062 | 0.058 | 0.948 |
| PWY.6893 | thiamine diphosphate biosynthesis II (Bacillus) | Not detected | -0.002 | -0.172 | 0.168 | 0.985 |
| PWY.6894 | thiamine diphosphate biosynthesis I (E. coli) | Not detected | 0.011 | -0.174 | 0.197 | 0.904 |
| PWY.6896 | thiamine salvage I | Not detected | -0.035 | -0.221 | 0.151 | 0.710 |
| PWY.6897 | thiamine salvage II | Not detected | 0.011 | -0.174 | 0.197 | 0.904 |
| PWY.6901 | superpathway of glucose and xylose degradation | Not detected | 0.021 | -0.044 | 0.085 | 0.531 |
| PWY.6902 | chitin degradation II (Vibrio) | Not detected | -0.012 | -0.207 | 0.184 | 0.908 |
| PWY.6906 | chitin derivatives degradation | Not detected | -0.038 | -0.106 | 0.030 | 0.268 |
| PWY.6938 | NADH repair | Not detected | -0.060 | -0.252 | 0.132 | 0.538 |
| PWY.6942 | dTDP-D-desosamine biosynthesis | Not detected | 0.017 | -0.052 | 0.086 | 0.624 |
| PWY.6944 | androstenedione degradation | Not detected | -0.073 | -0.281 | 0.135 | 0.491 |
| PWY.6949 | DIBOA-glucoside biosynthesis | Not detected | -0.063 | -0.219 | 0.093 | 0.429 |
| PWY.6953 | dTDP-3-acetamido-&alpha;-D-fucose biosynthes | Not detected | 0.021 | -0.048 | 0.091 | 0.546 |
| PWY.6961 | L-ascorbate degradation II (bacterial, aerobic) | Not detected | -0.003 | -0.067 | 0.060 | 0.921 |
| PWY.6969 | TCA cycle V (2-oxoglutarate:ferredoxin oxidore | Not detected | -0.017 | -0.088 | 0.055 | 0.649 |
| PWY.6973 | dTDP-D-olivose, dTDP-D-oliose and dTDP-D-m | Not detected | 0.021 | -0.048 | 0.090 | 0.548 |
| PWY.6974 | dTDP-L-olivose biosynthesis | Not detected | 0.021 | -0.048 | 0.090 | 0.548 |
| PWY.6976 | dTDP-L-mycarose biosynthesis | Not detected | 0.021 | -0.047 | 0.090 | 0.544 |
| PWY.6981 | chitin biosynthesis | Not detected | 0.000 | -0.066 | 0.066 | 0.999 |
| PWY.6983 | <i>threo</i>-tetrahydrobiopterin biosynthesis | Not detected | 0.027 | -0.096 | 0.150 | 0.665 |
| PWY.6984 | lipoate salvage II | Not detected | 0.003 | -0.155 | 0.162 | 0.966 |
| PWY.6986 | alginate degradation | Not detected | 0.003 | -0.069 | 0.076 | 0.927 |
| PWY.6987 | lipoate biosynthesis and incorporation III (Bacill | Not detected | -0.113 | -0.302 | 0.076 | 0.241 |
| PWY.6992 | 1,5-anhydrofructose degradation | Not detected | -0.006 | -0.074 | 0.062 | 0.860 |
| PWY.7003 | glycerol degradation to butanol | Not detected | 0.021 | -0.044 | 0.085 | 0.530 |
| PWY.7007 | methyl ketone biosynthesis (engineered) | Not detected | -0.092 | -0.314 | 0.131 | 0.420 |
| PWY.7013 | (<i>S</i>)-propane-1,2-diol degradation | Not detected | -0.228 | -0.446 | -0.010 | 0.040 |
| PWY.702 | L-methionine biosynthesis II (plants) | Not detected | 0.057 | -0.167 | 0.280 | 0.620 |
| PWY.7031 | protein <i>N</i>-glycosylation (bacterial) | Not detected | -0.127 | -0.350 | 0.097 | 0.267 |
| PWY.7037 | protein <i>O</i>-glycosylation (Neisseria) | Not detected | -0.124 | -0.348 | 0.100 | 0.279 |
| PWY.7077 | <i>N</i>-acetyl-D-galactosamine degradation | Not detected | -0.102 | -0.311 | 0.108 | 0.340 |
| PWY.7085 | triethylamine degradation | Not detected | -0.326 | -0.554 | -0.097 | 0.005 |
| PWY.7090 | UDP-2,3-diacetamido-2,3-dideoxy-&alpha;-D-m | Not detected | -0.144 | -0.304 | 0.015 | 0.077 |
| PWY.7096 | triclosan resistance | Not detected | 0.181 | -0.057 | 0.420 | 0.137 |
| PWY.7102 | bisabolene biosynthesis (engineered) | Not detected | -0.078 | -0.306 | 0.149 | 0.500 |
| PWY.7104 | dTDP-L-megosamine biosynthesis | Not detected | 0.021 | -0.048 | 0.090 | 0.548 |
| PWY.7111 | pyruvate fermentation to isobutanol (engineered) | Not detected | 0.005 | -0.075 | 0.085 | 0.904 |
| PWY.7115 | C4 photosynthetic carbon assimilation cycle, NA | Not detected | -0.012 | -0.254 | 0.231 | 0.926 |
| PWY.7117 | C4 photosynthetic carbon assimilation cycle, PEP | Not detected | 0.026 | -0.045 | 0.097 | 0.480 |
| PWY.7118 | chitin degradation to ethanol | Not detected | -0.181 | -0.419 | 0.058 | 0.137 |
| PWY.7124 | ethylene biosynthesis V (engineered) | Not detected | 0.004 | -0.065 | 0.074 | 0.900 |
| PWY.7127 | CDP-D-mannitol biosynthesis | Not detected | -0.030 | -0.266 | 0.207 | 0.806 |

| PWY.7130 | L-glucose degradation | Not detected | 0.053 | -0.140 | 0.246 | 0.590 |
| --- | --- | --- | --- | --- | --- | --- |
| PWY.7131 | CMP-legionaminate biosynthesis II | Not detected | 0.045 | -0.166 | 0.255 | 0.677 |
| PWY.7141 | (3<i>S</i>)-linalool biosynthesis | Not detected | -0.082 | -0.276 | 0.111 | 0.405 |
| PWY.7153 | grixazone biosynthesis | Not detected | -0.205 | -0.416 | 0.005 | 0.056 |
| PWY.7159 | 3,8-divinyl-chlorophyllide <i>a</i> biosynthesis | Not detected | 0.126 | -0.087 | 0.340 | 0.246 |
| PWY.7165 | L-ascorbate biosynthesis VI (engineered pathway | Not detected | -0.062 | -0.290 | 0.165 | 0.592 |
| PWY.7174 | <i>S</i>-methyl-5-thio-&alpha;-D-ribose 1-phos | Not detected | 0.126 | -0.107 | 0.359 | 0.289 |
| PWY.7176 | UTP and CTP <i>de novo</i> biosynthesis | Not detected | 0.008 | -0.064 | 0.079 | 0.832 |
| PWY.7177 | UTP and CTP dephosphorylation II | Not detected | 0.001 | -0.072 | 0.074 | 0.975 |
| PWY.7180 | 2'-deoxy-&alpha;-D-ribose 1-phosphate degradat | Not detected | -0.105 | -0.316 | 0.105 | 0.327 |
| PWY.7182 | linalool biosynthesis I | Not detected | -0.082 | -0.276 | 0.111 | 0.405 |
| PWY.7184 | pyrimidine deoxyribonucleotides <i>de novo</i> | Not detected | 0.052 | -0.029 | 0.134 | 0.210 |
| PWY.7185 | UTP and CTP dephosphorylation I | Not detected | 0.001 | -0.072 | 0.074 | 0.975 |
| PWY.7187 | pyrimidine deoxyribonucleotides <i>de novo</i> | Not detected | 0.053 | -0.026 | 0.132 | 0.187 |
| PWY.7193 | pyrimidine ribonucleosides salvage I | Not detected | 0.266 | 0.030 | 0.502 | 0.027 |
| PWY.7195 | pyrimidine ribonucleosides salvage III | Not detected | 0.039 | -0.126 | 0.204 | 0.645 |
| PWY.7197 | pyrimidine deoxyribonucleotide phosphorylation | Not detected | 0.054 | -0.026 | 0.134 | 0.186 |
| PWY.7198 | pyrimidine deoxyribonucleotides <i>de novo</i> | Not detected | 0.053 | -0.030 | 0.136 | 0.214 |
| PWY.7204 | pyridoxal 5'-phosphate salvage II (plants) | Not detected | -0.063 | -0.268 | 0.141 | 0.544 |
| PWY.7205 | CMP phosphorylation | Not detected | 0.054 | -0.023 | 0.130 | 0.172 |
| PWY.7210 | pyrimidine deoxyribonucleotides biosynthesis fro | Not detected | 0.053 | -0.030 | 0.136 | 0.214 |
| PWY.7216 | (R)- and (S)-3-hydroxybutanoate biosynthesis (e | Not detected | -0.051 | -0.178 | 0.077 | 0.439 |
| PWY.7218 | photosynthetic 3-hydroxybutanoate biosynthesis | Not detected | 0.048 | -0.026 | 0.121 | 0.206 |
| PWY.7219 | adenosine ribonucleotides <i>de novo</i> biosyn | Not detected | 0.052 | -0.030 | 0.134 | 0.213 |
| PWY.7221 | guanosine ribonucleotides <i>de novo</i> biosy | Not detected | 0.051 | -0.031 | 0.133 | 0.223 |
| PWY.7222 | guanosine deoxyribonucleotides <i>de novo</i> | Not detected | 0.051 | -0.031 | 0.133 | 0.223 |
| PWY.7224 | purine deoxyribonucleosides salvage | Not detected | 0.051 | -0.031 | 0.133 | 0.223 |
| PWY.7226 | guanosine deoxyribonucleotides <i>de novo</i> | Not detected | 0.051 | -0.031 | 0.133 | 0.223 |
| PWY.7234 | inosine-5'-phosphate biosynthesis III | Not detected | 0.061 | -0.059 | 0.181 | 0.319 |
| PWY.7237 | <i>myo</i>-, <i>chiro</i>- and <i>scyllo</i>-in | Not detected | 0.115 | -0.085 | 0.315 | 0.259 |
| PWY.7238 | sucrose biosynthesis II | Not detected | 0.001 | -0.063 | 0.065 | 0.987 |
| PWY.7241 | <I>myo</I>-inositol degradation II | Not detected | 0.127 | -0.073 | 0.328 | 0.213 |
| PWY.7242 | D-fructuronate degradation | Not detected | -0.029 | -0.090 | 0.032 | 0.351 |
| PWY.7247 | &beta;-D-glucuronide and D-glucuronate degrad | Not detected | 0.002 | -0.068 | 0.073 | 0.948 |
| PWY.7248 | pectin degradation II | Not detected | 0.002 | -0.068 | 0.073 | 0.950 |
| PWY.7250 | [2Fe-2S] iron-sulfur cluster biosynthesis | Not detected | 0.179 | -0.024 | 0.382 | 0.085 |
| PWY.7254 | TCA cycle VII (acetate-producers) | Not detected | -0.019 | -0.093 | 0.054 | 0.607 |
| PWY.7255 | ergothioneine biosynthesis I (bacteria) | Not detected | 0.026 | -0.159 | 0.210 | 0.785 |
| PWY.7268 | NAD/NADP-NADH/NADPH cytosolic interconv | Not detected | -0.038 | -0.204 | 0.128 | 0.657 |
| PWY.7269 | NAD/NADP-NADH/NADPH mitochondrial inte | Not detected | 0.049 | -0.130 | 0.228 | 0.594 |
| PWY.7279 | aerobic respiration II (cytochrome c) (yeast) | Not detected | 0.053 | -0.126 | 0.232 | 0.563 |
| PWY.7282 | 4-amino-2-methyl-5-diphosphomethylpyrimidine | Not detected | 0.032 | -0.118 | 0.182 | 0.680 |
| PWY.7288 | fatty acid &beta;-oxidation (peroxisome, yeast) | Not detected | 0.027 | -0.192 | 0.246 | 0.807 |
| PWY.7290 | <i>Escherichia coli</i> serotype O86 <i>O</i>- | Not detected | 0.020 | -0.220 | 0.260 | 0.872 |

| PWY.7294 | D-xylose degradation IV | Not detected | -0.095 | -0.325 | 0.135 | 0.420 |
| --- | --- | --- | --- | --- | --- | --- |
| PWY.7295 | L-arabinose degradation IV | Not detected | -0.002 | -0.221 | 0.217 | 0.984 |
| PWY.7301 | dTDP-4-<i>O</i>-demethyl-&beta;-L-noviose b | Not detected | 0.016 | -0.054 | 0.085 | 0.661 |
| PWY.7312 | dTDP-D-&beta;-fucofuranose biosynthesis | Not detected | 0.021 | -0.047 | 0.090 | 0.541 |
| PWY.7315 | dTDP-<i>N</i>-acetylthomosamine biosynthesis | Not detected | 0.023 | -0.043 | 0.089 | 0.490 |
| PWY.7316 | dTDP-<i>N</i>-acetylviosamine biosynthesis | Not detected | 0.017 | -0.052 | 0.086 | 0.624 |
| PWY.7318 | dTDP-3-acetamido-3,6-dideoxy-&alpha;-D-gluc | Not detected | 0.022 | -0.047 | 0.091 | 0.525 |
| PWY.7328 | superpathway of UDP-glucose-derived O-antigen | Not detected | -0.028 | -0.109 | 0.053 | 0.498 |
| PWY.7330 | UDP-<i>N</i>-acetyl-&beta;-L-fucosamine bios | Not detected | -0.076 | -0.231 | 0.080 | 0.341 |
| PWY.7331 | UDP-<i>N</i>-acetyl-&beta;-L-quinovosamine | Not detected | -0.030 | -0.184 | 0.124 | 0.703 |
| PWY.7332 | superpathway of UDP-<I>N</i>-acetylglucosami | Not detected | -0.034 | -0.199 | 0.131 | 0.689 |
| PWY.7335 | UDP-<i>N</i>-acetyl-&alpha;-D-mannosaminou | Not detected | 0.022 | -0.093 | 0.136 | 0.713 |
| PWY.7336 | UDP-<i>N</i>-acetyl-&alpha;-D-galactosaminur | Not detected | -0.170 | -0.336 | -0.003 | 0.046 |
| PWY.7342 | superpathway of nicotine biosynthesis | Not detected | -0.157 | -0.411 | 0.097 | 0.226 |
| PWY.7343 | UDP-&alpha;-D-glucose biosynthesis I | Not detected | 0.036 | -0.039 | 0.111 | 0.352 |
| PWY.7344 | UDP-&alpha;-D-galactose biosynthesis | Not detected | -0.063 | -0.193 | 0.068 | 0.347 |
| PWY.7346 | UDP-&alpha;-D-glucuronate biosynthesis (from | Not detected | -0.179 | -0.363 | 0.005 | 0.057 |
| PWY.7347 | sucrose biosynthesis III | Not detected | -0.013 | -0.088 | 0.063 | 0.740 |
| PWY.7377 | cob(II)yrinate <i>a,c</i>-diamide biosynthesis I | Not detected | 0.061 | -0.029 | 0.151 | 0.182 |
| PWY.7378 | aminopropanol phosphate biosynthesis II | Not detected | 0.030 | -0.190 | 0.249 | 0.791 |
| PWY.7383 | anaerobic energy metabolism (invertebrates, cyto | Not detected | 0.036 | -0.200 | 0.272 | 0.763 |
| PWY.7384 | anaerobic energy metabolism (invertebrates, mito | Not detected | -0.108 | -0.187 | -0.028 | 0.008 |
| PWY.7385 | 1,3-propanediol biosynthesis (engineered) | Not detected | 0.002 | -0.065 | 0.068 | 0.961 |
| PWY.7388 | octanoyl-[acyl-carrier protein] biosynthesis (mito | Not detected | 0.267 | 0.060 | 0.474 | 0.012 |
| PWY.7395 | D-galactosamine and <i>N</i>-acetyl-D-galactos | Not detected | -0.102 | -0.311 | 0.108 | 0.340 |
| PWY.7396 | butanol and isobutanol biosynthesis (engineered) | Not detected | -0.019 | -0.112 | 0.073 | 0.681 |
| PWY.7399 | methylphosphonate degradation II | Not detected | -0.247 | -0.460 | -0.033 | 0.024 |
| PWY.7400 | L-arginine biosynthesis IV (archaebacteria) | Not detected | -0.037 | -0.150 | 0.075 | 0.517 |
| PWY.7401 | crotonate fermentation (to acetate and cyclohexan | Not detected | -0.011 | -0.131 | 0.109 | 0.856 |
| PWY.7410 | ipsdienol biosynthesis | Not detected | -0.082 | -0.276 | 0.111 | 0.405 |
| PWY.7413 | dTDP-6-deoxy-&alpha;-D-allose biosynthesis | Not detected | 0.021 | -0.048 | 0.090 | 0.548 |
| PWY.7414 | dTDP-&alpha;-D-mycaminose biosynthesis | Not detected | 0.022 | -0.047 | 0.091 | 0.525 |
| PWY.7419 | FR-900098 and FR-33289 antibiotics biosynthes | Not detected | 0.013 | -0.223 | 0.249 | 0.913 |
| PWY.7425 | 2-chloroacrylate degradation I | Not detected | -0.008 | -0.192 | 0.176 | 0.931 |
| PWY.7431 | aromatic biogenic amine degradation (bacteria) | Not detected | -0.111 | -0.285 | 0.062 | 0.208 |
| PWY.7440 | dTDP-&beta;-L-4-<i>epi</i>-vancosamine biosy | Not detected | 0.021 | -0.048 | 0.090 | 0.548 |
| PWY.7442 | drosopterin and aurodrosopterin biosynthesis | Not detected | 0.027 | -0.096 | 0.150 | 0.665 |
| PWY.7446 | sulfoquinovose degradation I | Not detected | -0.164 | -0.401 | 0.073 | 0.175 |
| PWY.7456 | &beta;-(1,4)-mannan degradation | Not detected | -0.033 | -0.145 | 0.078 | 0.559 |
| PWY.7459 | kojibiose degradation | Not detected | 0.002 | -0.191 | 0.194 | 0.987 |
| PWY.7467 | 2-acetamido-4-amino-2,4,6-trideoxy-&alpha;-D- | Not detected | -0.069 | -0.244 | 0.107 | 0.442 |
| PWY.7510 | rhizocticin A and B biosynthesis | Not detected | 0.013 | -0.223 | 0.249 | 0.913 |
| PWY.7530 | &beta;-D-galactosaminyl-(1&rarr;3)-<i>N</i>-a | Not detected | 0.020 | -0.220 | 0.261 | 0.869 |
| PWY.7539 | 6-hydroxymethyl-dihydropterin diphosphate bios | Not detected | 0.003 | -0.072 | 0.078 | 0.940 |

| PWY.7541 | 1,2-propanediol biosynthesis from lactate (engine | Not detected | -0.204 | -0.442 | 0.034 | 0.093 |
| --- | --- | --- | --- | --- | --- | --- |
| PWY.7552 | heme <i>b</i> biosynthesis III (from siroheme) | Not detected | -0.003 | -0.225 | 0.220 | 0.981 |
| PWY.7559 | glutathione degradation (DUG pathway - yeast) | Not detected | 0.102 | -0.115 | 0.319 | 0.356 |
| PWY.7560 | methylerythritol phosphate pathway II | Not detected | -0.068 | -0.161 | 0.024 | 0.146 |
| PWY.7562 | 3,6-anhydro-&alpha;-L-galactopyranose degrada | Not detected | 0.003 | -0.065 | 0.071 | 0.926 |
| PWY.7564 | bacimethrin and bacimethrin pyrophosphate bios | Not detected | 0.062 | -0.073 | 0.196 | 0.368 |
| PWY.7568 | L-galactose degradation | Not detected | -0.238 | -0.414 | -0.062 | 0.008 |
| PWY.7573 | GDP-mycosamine biosynthesis | Not detected | 0.301 | 0.067 | 0.534 | 0.012 |
| PWY.7581 | <i>N</i>-acetylneuraminate and <i>N</i>-acety | Not detected | -0.192 | -0.344 | -0.040 | 0.013 |
| PWY.7586 | &beta;-1,4-D-mannosyl-<i>N</i>-acetyl-D-gluc | Not detected | -0.002 | -0.195 | 0.191 | 0.985 |
| PWY.7588 | ursodeoxycholate biosynthesis (bacteria) | Not detected | -0.136 | -0.330 | 0.058 | 0.170 |
| PWY.7616 | methanol oxidation to carbon dioxide | Not detected | -0.062 | -0.276 | 0.151 | 0.566 |
| PWY.7622 | UDP-&alpha;-D-galactofuranose biosynthesis | Not detected | -0.030 | -0.112 | 0.051 | 0.465 |
| PWY.7644 | heparin degradation | Not detected | -0.205 | -0.416 | 0.006 | 0.057 |
| PWY.7645 | hyaluronan degradation | Not detected | -0.258 | -0.452 | -0.064 | 0.009 |
| PWY.7646 | dermatan sulfate degradation I (bacterial) | Not detected | -0.253 | -0.446 | -0.061 | 0.010 |
| PWY.7651 | heparan sulfate degradation | Not detected | -0.127 | -0.400 | 0.146 | 0.363 |
| PWY.7657 | dTDP-&beta;-L-digitoxose biosynthesis | Not detected | 0.021 | -0.048 | 0.090 | 0.548 |
| PWY.7659 | viridicatumtoxin biosynthesis | Not detected | -0.082 | -0.276 | 0.111 | 0.405 |
| PWY.7663 | gondoate biosynthesis (anaerobic) | Not detected | -0.039 | -0.125 | 0.046 | 0.370 |
| PWY.7664 | oleate biosynthesis IV (anaerobic) | Not detected | -0.040 | -0.126 | 0.047 | 0.367 |
| PWY.7674 | CMP-8-amino-3,8-dideoxy-D-<I>manno</I>-oct | Not detected | 0.017 | -0.187 | 0.222 | 0.868 |
| PWY.7675 | Kdo transfer to lipid IV<sub>A</sub> II | Not detected | -0.033 | -0.228 | 0.162 | 0.737 |
| PWY.7684 | &alpha;-diglucosyldiacylglycerol biosynthesis | Not detected | 0.032 | -0.166 | 0.231 | 0.752 |
| PWY.7688 | dTDP-D-ravidosamine and dTDP-4-acetyl-D-rav | Not detected | 0.021 | -0.048 | 0.091 | 0.546 |
| PWY.7709 | (3<i>R</i>)-linalool biosynthesis | Not detected | -0.082 | -0.276 | 0.111 | 0.405 |
| PWY.7716 | penicillin G and penicillin V biosynthesis | Not detected | -0.121 | -0.232 | -0.011 | 0.032 |
| PWY.7719 | CMP-diacetamido-8-epilegionaminic acid biosyn | Not detected | -0.056 | -0.201 | 0.089 | 0.445 |
| PWY.7721 | methyl phomopsenoate biosynthesis | Not detected | -0.091 | -0.288 | 0.107 | 0.367 |
| PWY.7736 | stellatic acid biosynthesis | Not detected | -0.109 | -0.333 | 0.116 | 0.343 |
| PWY.7754 | bile acids degradation | Not detected | -0.064 | -0.343 | 0.215 | 0.651 |
| PWY.7755 | iso-bile acids biosynthesis I | Not detected | 0.248 | -0.012 | 0.509 | 0.061 |
| PWY.7756 | iso-bile acids biosynthesis II | Not detected | -0.040 | -0.201 | 0.121 | 0.628 |
| PWY.7766 | heme <i>b</i> biosynthesis IV (Gram-positive b | Not detected | 0.127 | -0.087 | 0.340 | 0.245 |
| PWY.7769 | phosalacine biosynthesis | Not detected | 0.010 | -0.225 | 0.246 | 0.931 |
| PWY.7778 | 2-methylpropene degradation | Not detected | -0.051 | -0.179 | 0.077 | 0.438 |
| PWY.7779 | methyl <i>tert</i>-butyl ether degradation | Not detected | -0.051 | -0.179 | 0.077 | 0.438 |
| PWY.7790 | UMP biosynthesis II | Not detected | -0.028 | -0.108 | 0.053 | 0.501 |
| PWY.7791 | UMP biosynthesis III | Not detected | -0.028 | -0.108 | 0.053 | 0.501 |
| PWY.7793 | dimethyl sulfide biosynthesis from methionine | Not detected | -0.119 | -0.321 | 0.083 | 0.248 |
| PWY.7805 | (aminomethyl)phosphonate degradation | Not detected | -0.059 | -0.271 | 0.153 | 0.588 |
| PWY.7807 | glyphosate degradation III | Not detected | 0.050 | -0.154 | 0.255 | 0.629 |
| PWY.7808 | tetracycline resistance | Not detected | -0.357 | -0.681 | -0.034 | 0.031 |
| PWY.7814 | dTDP-L-daunosamine biosynthesis | Not detected | 0.016 | -0.054 | 0.085 | 0.661 |

| PWY.7815 | poly(ribitol phosphate) wall teichoic acid biosynt | Not detected | 0.058 | -0.041 | 0.158 | 0.251 |
| --- | --- | --- | --- | --- | --- | --- |
| PWY.7816 | poly(ribitol phosphate) wall teichoic acid biosynt | Not detected | 0.064 | -0.036 | 0.165 | 0.210 |
| PWY.7817 | type I lipoteichoic acid biosynthesis (<i>S. aureu | Not detected | 0.022 | -0.072 | 0.117 | 0.640 |
| PWY.7818 | type IV lipoteichoic acid biosynthesis (<i>S. pne | Not detected | 0.149 | -0.018 | 0.317 | 0.081 |
| PWY.7819 | poly(3-O-&beta;-D-glucopyranosyl-<i>N</i>-ac | Not detected | 0.022 | -0.093 | 0.136 | 0.712 |
| PWY.7820 | teichuronic acid biosynthesis (<i>B. subtilis</i> | Not detected | -0.187 | -0.368 | -0.007 | 0.042 |
| PWY.7822 | chitin degradation III (Serratia) | Not detected | -0.005 | -0.201 | 0.191 | 0.958 |
| PWY.7852 | 6-hydroxymethyl-dihydropterin diphosphate bios | Not detected | 0.022 | -0.087 | 0.130 | 0.696 |
| PWY.7854 | crotonyl-CoA/ethylmalonyl-CoA/hydroxybutyryl | Not detected | 0.006 | -0.213 | 0.225 | 0.957 |
| PWY.7856 | heme <i>a</i> biosynthesis | Not detected | -0.057 | -0.221 | 0.107 | 0.494 |
| PWY.7858 | (5Z)-dodecenoate biosynthesis II | Not detected | 0.014 | -0.070 | 0.097 | 0.752 |
| PWY.7863 | roseoflavin biosynthesis | Not detected | 0.026 | -0.145 | 0.196 | 0.768 |
| PWY.7864 | 8-methylmenaquinone biosynthesis | Not detected | 0.167 | -0.078 | 0.412 | 0.181 |
| PWY.7873 | D-erythronate degradation II | Not detected | 0.027 | -0.151 | 0.205 | 0.766 |
| PWY.7874 | L-threonate degradation | Not detected | -0.009 | -0.208 | 0.191 | 0.931 |
| PWY.7883 | anhydromuropeptides recycling II | Not detected | 0.230 | 0.021 | 0.440 | 0.031 |
| PWY.7884 | lipoprotein posttranslational modification | Not detected | 0.019 | -0.219 | 0.257 | 0.876 |
| PWY.7892 | tRNA-uridine 2-thiolation (bacteria) | Not detected | -0.003 | -0.154 | 0.148 | 0.965 |
| PWY.7900 | glycogen biosynthesis III (from &alpha;-maltose | Not detected | 0.060 | -0.028 | 0.149 | 0.180 |
| PWY.7902 | glucosylglycerol biosynthesis | Not detected | 0.044 | -0.054 | 0.142 | 0.381 |
| PWY.7909 | formaldehyde oxidation VII (THF pathway) | Not detected | -0.115 | -0.221 | -0.008 | 0.036 |
| PWY.7928 | nickel cofactor biosynthesis | Not detected | -0.049 | -0.196 | 0.098 | 0.514 |
| PWY.7953 | UDP-<i>N</i>-acetylmuramoyl-pentapeptide bio | Not detected | 0.010 | -0.051 | 0.070 | 0.754 |
| PWY.7975 | adenosylcobalamin biosynthesis from adenosylco | Not detected | -0.025 | -0.191 | 0.140 | 0.764 |
| PWY.7977 | L-methionine biosynthesis IV (archaea) | Not detected | -0.040 | -0.113 | 0.033 | 0.281 |
| PWY.7978 | <i>N</i>-methylpyrrolidone degradation | Not detected | -0.108 | -0.276 | 0.060 | 0.208 |
| PWY.7981 | &alpha;-dystroglycan glycosylation | Not detected | 0.311 | 0.077 | 0.544 | 0.009 |
| PWY.7991 | toxoflavin biosynthesis | Not detected | 0.006 | -0.064 | 0.076 | 0.870 |
| PWY.8004 | Entner-Doudoroff pathway I | Not detected | -0.002 | -0.068 | 0.063 | 0.941 |
| PWY.801 | homocysteine and cysteine interconversion | Not detected | -0.008 | -0.234 | 0.217 | 0.942 |
| PWY.8010 | L-cysteine biosynthesis IX (Trichomonas vaginal | Not detected | 0.037 | -0.056 | 0.129 | 0.436 |
| PWY.8012 | mupirocin biosynthesis | Not detected | 0.273 | 0.066 | 0.480 | 0.010 |
| PWY.8013 | UDP-<i>N</i>-acetyl-D-galactosamine biosynth | Not detected | -0.013 | -0.088 | 0.063 | 0.740 |
| PWY.8015 | glycine degradation (Stickland reaction) | Not detected | -0.003 | -0.072 | 0.065 | 0.920 |
| PWY.8040 | ansatrienin biosynthesis | Not detected | 0.060 | -0.168 | 0.288 | 0.605 |
| PWY.882 | L-ascorbate biosynthesis I (L-galactose pathway) | Not detected | -0.024 | -0.216 | 0.168 | 0.808 |
| PWY.I9 | L-cysteine biosynthesis VI (from L-methionine) | Not detected | 0.081 | -0.147 | 0.310 | 0.485 |
| PWY0.1182 | trehalose degradation II (cytosolic) | Not detected | -0.079 | -0.311 | 0.153 | 0.506 |
| PWY0.1221 | putrescine degradation II | Not detected | -0.131 | -0.325 | 0.064 | 0.189 |
| PWY0.1241 | ADP-L-<i>glycero</i>-&beta;-D-<i>manno</i> | Not detected | 0.025 | -0.172 | 0.221 | 0.805 |
| PWY0.1261 | anhydromuropeptides recycling I | Not detected | 0.032 | -0.025 | 0.088 | 0.270 |
| PWY0.1264 | biotin-carboxyl carrier protein assembly | Not detected | -0.024 | -0.110 | 0.062 | 0.582 |
| PWY0.1275 | lipoate biosynthesis and incorporation II | Not detected | -0.077 | -0.241 | 0.088 | 0.362 |
| PWY0.1296 | purine ribonucleosides degradation | Not detected | 0.071 | -0.113 | 0.255 | 0.447 |

| PWY0.1300 | 2-<I>O</I>-&alpha;-mannosyl-D-glycerate degr | Not detected | -0.035 | -0.261 | 0.190 | 0.759 |
| --- | --- | --- | --- | --- | --- | --- |
| PWY0.1306 | L-galactonate degradation | 0.6 | -0.256 | -0.404 | -0.109 | 0.001 |
| PWY0.1312 | acetate and ATP formation from acetyl-CoA I | Not detected | -0.014 | -0.077 | 0.048 | 0.648 |
| PWY0.1313 | acetate conversion to acetyl-CoA | Not detected | -0.114 | -0.327 | 0.098 | 0.292 |
| PWY0.1317 | L-lactaldehyde degradation (aerobic) | Not detected | 0.126 | -0.068 | 0.320 | 0.204 |
| PWY0.1319 | CDP-diacylglycerol biosynthesis II | Not detected | -0.074 | -0.242 | 0.093 | 0.384 |
| PWY0.1324 | <i>N</i>-acetylneuraminate and <i>N</i>-acety | Not detected | -0.161 | -0.295 | -0.028 | 0.018 |
| PWY0.1337 | oleate &beta;-oxidation | Not detected | -0.126 | -0.325 | 0.073 | 0.215 |
| PWY0.1338 | polymyxin resistance | Not detected | -0.005 | -0.220 | 0.209 | 0.962 |
| PWY0.1415 | superpathway of heme <i>b</i> biosynthesis fro | Not detected | 0.140 | -0.075 | 0.354 | 0.201 |
| PWY0.1433 | tetrahydromonapterin biosynthesis | Not detected | 0.046 | -0.062 | 0.154 | 0.407 |
| PWY0.1465 | D-malate degradation | Not detected | -0.204 | -0.406 | -0.003 | 0.047 |
| PWY0.1466 | trehalose degradation VI (periplasmic) | Not detected | -0.096 | -0.310 | 0.118 | 0.379 |
| PWY0.1471 | uracil degradation III | Not detected | 0.002 | -0.188 | 0.191 | 0.987 |
| PWY0.1477 | ethanolamine utilization | Not detected | -0.014 | -0.072 | 0.044 | 0.627 |
| PWY0.1479 | tRNA processing | Not detected | 0.067 | -0.158 | 0.291 | 0.559 |
| PWY0.1533 | methylphosphonate degradation I | Not detected | -0.265 | -0.474 | -0.056 | 0.013 |
| PWY0.1545 | cardiolipin biosynthesis III | Not detected | 0.139 | -0.074 | 0.352 | 0.201 |
| PWY0.1546 | muropeptide degradation | Not detected | 0.012 | -0.170 | 0.195 | 0.895 |
| PWY0.1565 | D-lactate to cytochrome <i>bo</i> oxidase electr | Not detected | -0.008 | -0.192 | 0.176 | 0.931 |
| PWY0.1567 | NADH to cytochrome <i>bo</i> oxidase electro | Not detected | 0.053 | -0.126 | 0.232 | 0.563 |
| PWY0.1568 | NADH to cytochrome <i>bd</i> oxidase electro | Not detected | 0.053 | -0.126 | 0.232 | 0.563 |
| PWY0.1569 | autoinducer AI-2 degradation | Not detected | -0.100 | -0.287 | 0.086 | 0.291 |
| PWY0.1573 | nitrate reduction VIIIb (dissimilatory) | Not detected | 0.053 | -0.126 | 0.232 | 0.563 |
| PWY0.1585 | formate to nitrite electron transfer | Not detected | 0.183 | -0.013 | 0.380 | 0.068 |
| PWY0.1586 | peptidoglycan maturation (<i>meso</i>-diamino | Not detected | -0.127 | -0.392 | 0.138 | 0.347 |
| PWY0.1590 | NADH to hydrogen peroxide electron transfer | Not detected | -0.053 | -0.255 | 0.149 | 0.608 |
| PWY0.1591 | glycerol-3-phosphate to hydrogen peroxide electr | Not detected | -0.053 | -0.255 | 0.149 | 0.608 |
| PWY0.1597 | prenylated FMNH<sub>2</sub> biosynthesis | Not detected | -0.002 | -0.160 | 0.156 | 0.976 |
| PWY0.166 | superpathway of pyrimidine deoxyribonucleotide | Not detected | 0.054 | -0.028 | 0.135 | 0.196 |
| PWY0.301 | L-ascorbate degradation I (bacterial, anaerobic) | Not detected | -0.003 | -0.067 | 0.060 | 0.921 |
| PWY0.321 | phenylacetate degradation I (aerobic) | Not detected | -0.100 | -0.197 | -0.003 | 0.044 |
| PWY0.42 | 2-methylcitrate cycle I | Not detected | -0.150 | -0.397 | 0.097 | 0.233 |
| PWY0.44 | D-allose degradation | Not detected | 0.019 | -0.218 | 0.256 | 0.875 |
| PWY0.461 | L-lysine degradation I | Not detected | 0.027 | -0.176 | 0.230 | 0.794 |
| PWY0.501 | lipoate biosynthesis and incorporation I | Not detected | -0.057 | -0.222 | 0.109 | 0.503 |
| PWY0.522 | lipoate salvage I | Not detected | 0.014 | -0.169 | 0.198 | 0.880 |
| PWY0.662 | PRPP biosynthesis | Not detected | 0.005 | -0.087 | 0.096 | 0.918 |
| PWY0.862 | (5Z)-dodecenoate biosynthesis I | Not detected | 0.014 | -0.070 | 0.098 | 0.747 |
| PWY1.3 | polyhydroxybutanoate biosynthesis | Not detected | -0.062 | -0.230 | 0.106 | 0.469 |
| PWY3O.4107 | NAD salvage pathway V (PNC V cycle) | Not detected | -0.058 | -0.253 | 0.137 | 0.558 |
| PWY4FS.7 | phosphatidylglycerol biosynthesis I (plastidic) | Not detected | 0.056 | -0.141 | 0.254 | 0.576 |
| PWY4FS.8 | phosphatidylglycerol biosynthesis II (non-plastid | Not detected | 0.056 | -0.141 | 0.254 | 0.576 |
| PWY4LZ.257 | superpathway of fermentation (<i>Chlamydomon | Not detected | -0.014 | -0.072 | 0.043 | 0.624 |

| PWY66.161 | ethanol degradation III | Not detected | -0.132 | -0.345 | 0.081 | 0.224 |
| --- | --- | --- | --- | --- | --- | --- |
| PWY66.162 | ethanol degradation IV | Not detected | -0.132 | -0.345 | 0.081 | 0.224 |
| PWY66.21 | ethanol degradation II | Not detected | -0.264 | -0.490 | -0.039 | 0.022 |
| PWY66.366 | flavin biosynthesis IV (mammalian) | Not detected | 0.028 | -0.170 | 0.226 | 0.781 |
| PWY66.391 | fatty acid &beta;-oxidation VI (peroxisome) | Not detected | -0.082 | -0.331 | 0.168 | 0.520 |
| PWY66.398 | TCA cycle III (animals) | Not detected | -0.028 | -0.108 | 0.052 | 0.494 |
| PWY66.399 | gluconeogenesis III | Not detected | -0.003 | -0.072 | 0.066 | 0.929 |
| PWY66.422 | D-galactose degradation V (Leloir pathway) | Not detected | -0.003 | -0.070 | 0.064 | 0.929 |
| PWY66.426 | hydrogen sulfide biosynthesis II (mammalian) | Not detected | 0.176 | -0.017 | 0.369 | 0.073 |
| PYRIDNUCS | NAD salvage pathway I (PNC VI cycle) | Not detected | -0.038 | -0.236 | 0.159 | 0.703 |
| PYRIDNUCS | NAD <i>de novo</i> biosynthesis I (from aspart | Not detected | -0.165 | -0.421 | 0.091 | 0.208 |
| PYRIDOXSY | pyridoxal 5'-phosphate biosynthesis I | Not detected | -0.224 | -0.479 | 0.031 | 0.085 |
| PYRUVDEH | NA | Not detected | 0.088 | -0.123 | 0.299 | 0.412 |
| PYRUVDEH | pyruvate decarboxylation to acetyl CoA | Not detected | 0.088 | -0.123 | 0.299 | 0.412 |
| QUINATEDE | quinate degradation I | Not detected | 0.042 | -0.147 | 0.231 | 0.663 |
| REDCITCYC | TCA cycle VIII (Helicobacter) | Not detected | -0.024 | -0.102 | 0.055 | 0.554 |
| RHAMCAT.P | L-rhamnose degradation I | Not detected | 0.055 | -0.136 | 0.247 | 0.570 |
| RIBOSYN2.P | flavin biosynthesis I (bacteria and plants) | Not detected | 0.006 | -0.063 | 0.075 | 0.868 |
| RUMP.PWY | formaldehyde oxidation I | Not detected | -0.005 | -0.072 | 0.061 | 0.872 |
| RXN.12588 | NA | Not detected | 0.122 | -0.102 | 0.347 | 0.285 |
| RXN.13163 | NA | Not detected | -0.275 | -0.529 | -0.021 | 0.034 |
| RXN.14047 | NA | Not detected | -0.033 | -0.118 | 0.051 | 0.437 |
| RXN.14201 | NA | Not detected | 0.005 | -0.049 | 0.058 | 0.867 |
| RXN.14249 | NA | Not detected | 0.030 | -0.190 | 0.249 | 0.791 |
| RXN.14314 | NA | Not detected | 0.129 | -0.087 | 0.345 | 0.241 |
| RXN.17127 | NA | Not detected | 0.014 | -0.169 | 0.198 | 0.880 |
| RXN.20025 | NA | Not detected | -0.012 | -0.184 | 0.160 | 0.893 |
| RXN0.1141 | NA | Not detected | 0.008 | -0.150 | 0.167 | 0.920 |
| RXN0.6511 | NA | Not detected | 0.014 | -0.189 | 0.218 | 0.890 |
| SERSYN.PW | L-serine biosynthesis I | Not detected | 0.036 | -0.044 | 0.116 | 0.382 |
| SUCROSEU | sucrose degradation VII (sucrose 3-dehydrogenas | Not detected | -0.109 | -0.320 | 0.103 | 0.315 |
| SUCSYN.PW | sucrose biosynthesis I (from photosynthesis) | Not detected | -0.005 | -0.074 | 0.064 | 0.893 |
| SUCUTIL.P | sucrose degradation I (sucrose phosphotransferas | Not detected | -0.086 | -0.294 | 0.121 | 0.416 |
| SULFMETII. | assimilatory sulfate reduction II | Not detected | 0.223 | -0.026 | 0.472 | 0.079 |
| TCA | TCA cycle I (prokaryotic) | Not detected | -0.021 | -0.095 | 0.054 | 0.588 |
| TEICHOICA | poly(glycerol phosphate) wall teichoic acid biosy | Not detected | 0.028 | -0.076 | 0.132 | 0.595 |
| THRDLCTC | L-threonine degradation III (to methylglyoxal) | Not detected | 0.030 | -0.190 | 0.249 | 0.791 |
| THREONINE | L-threonine degradation II | Not detected | 0.030 | -0.190 | 0.249 | 0.791 |
| TREDEGLO | trehalose degradation I (low osmolarity) | Not detected | -0.055 | -0.142 | 0.033 | 0.221 |
| TRESYN.PW | trehalose biosynthesis I | Not detected | 0.027 | -0.159 | 0.213 | 0.777 |
| TRIGLSYN.P | diacylglycerol and triacylglycerol biosynthesis | Not detected | 0.181 | -0.052 | 0.413 | 0.128 |
| TRNA.CHAR | tRNA charging | Not detected | 0.015 | -0.036 | 0.065 | 0.574 |
| TRPSYN.PW | L-tryptophan biosynthesis | Not detected | 0.061 | -0.177 | 0.299 | 0.613 |
| TRYPSYN.R | NA | Not detected | -0.074 | -0.285 | 0.136 | 0.490 |

| UDPNACET | UDP-<i>N</i>-acetyl-D-glucosamine biosynthes | Not detected | -0.009 | -0.080 | 0.062 | 0.802 |
| --- | --- | --- | --- | --- | --- | --- |
| UDPNAGSY | UDP-<i>N</i>-acetyl-D-glucosamine biosynthes | Not detected | -0.005 | -0.074 | 0.064 | 0.888 |
| VALSYN.PW | L-valine biosynthesis | Not detected | 0.005 | -0.075 | 0.085 | 0.904 |
| X1.1.1.39.RX | NA | Not detected | -0.012 | -0.254 | 0.231 | 0.926 |
| X1CMET2.P | <i>N</i><sup>10</sup>-formyl-tetrahydrofolate | Not detected | -0.042 | -0.111 | 0.027 | 0.232 |
| X3.HYDROX | 4-hydroxyphenylacetate degradation | Not detected | -0.258 | -0.459 | -0.058 | 0.012 |

| **Supplementary Table 4.** Association of current SEP (higher SEP vs. Lower SEP) with functional modules in overall participants (n=2174). Analysis of Compositions of Microbiomes (ANCOM2) was used to identify functional modules related to current SEP. Regression coefficients were obtained by modelling the effect of current SEP on centered log ratio (CLR)-transformed species in multivariable linear regression adjusted for age, gender, BMI, field center, Hispanic/Latino background, U.S. nativity, Bristol stool type, parental education, the alternative Healthy Eating Index 2010 score, cigarette smoking, alcohol use, depressive and anxiety symptoms, diabetes, hypertension, cardiovascular disease, dyslipidemia, and antibiotic use. | | | | | | | |
| --- | --- | --- | --- | --- | --- | --- | --- |
| **otu.names** | **pathname** | **W_stat** | **ANCOM** | **beta** | **l95ci** | **u95ci** | **qval** |
| ADENOSYLHOM | L-methionine salvage from L-homocysteine | 0 | Not detected | -0.028 | -0.106 | 0.050 | 0.480 |
| ALANINE.VALIN | L-alanine biosynthesis I | 0 | Not detected | 0.038 | -0.151 | 0.226 | 0.696 |
| ANAGLYCOLYSI | glycolysis III (from glucose) | 0 | Not detected | 0.001 | -0.069 | 0.070 | 0.985 |
| APYRASE.RXN | NA | 0 | Not detected | 0.008 | -0.066 | 0.081 | 0.837 |
| ARABCAT.PWY | L-arabinose degradation I | 0 | Not detected | 0.005 | -0.072 | 0.081 | 0.899 |
| ARGDEG.III.PWY | L-arginine degradation IV (arginine decarboxyla | 0 | Not detected | -0.018 | -0.127 | 0.090 | 0.742 |
| ARGSYN.PWY | L-arginine biosynthesis I (via L-ornithine) | 0 | Not detected | -0.066 | -0.184 | 0.052 | 0.273 |
| ARGSYNBSUB.P | L-arginine biosynthesis II (acetyl cycle) | 0 | Not detected | -0.059 | -0.175 | 0.056 | 0.311 |
| AST.PWY | L-arginine degradation II (AST pathway) | 0 | Not detected | 0.010 | -0.213 | 0.232 | 0.933 |
| BETSYN.PWY | glycine betaine biosynthesis I (Gram-negative ba | 0 | Not detected | 0.120 | -0.084 | 0.324 | 0.249 |
| CALVIN.PWY | Calvin-Benson-Bassham cycle | 0 | Not detected | -0.001 | -0.070 | 0.067 | 0.969 |
| CARBPSYN.RXN | NA | 0 | Not detected | 0.103 | -0.041 | 0.246 | 0.163 |
| CARNMET.PWY | L-carnitine degradation I | 0 | Not detected | 0.074 | -0.156 | 0.304 | 0.530 |
| CENTFERM.PWY | pyruvate fermentation to butanoate | 0 | Not detected | 0.040 | -0.051 | 0.132 | 0.387 |
| CHLOROPHYLL.S | 3,8-divinyl-chlorophyllide <i>a</i> biosynthesis | 0 | Not detected | 0.024 | -0.200 | 0.248 | 0.833 |
| CHOLINE.BETAI | choline degradation I | 0 | Not detected | 0.120 | -0.084 | 0.324 | 0.249 |
| COBALSYN.PWY | superpathway of adenosylcobalamin salvage fro | 0 | Not detected | -0.020 | -0.194 | 0.153 | 0.819 |
| CODH.PWY | reductive acetyl coenzyme A pathway I (homoac | 0 | Not detected | -0.111 | -0.223 | 0.001 | 0.052 |
| COLANSYN.PWY | colanic acid building blocks biosynthesis | 0 | Not detected | -0.051 | -0.188 | 0.086 | 0.466 |
| DAPLYSINESYN. | L-lysine biosynthesis I | 0 | Not detected | -0.035 | -0.258 | 0.188 | 0.759 |
| DARABCATK12.P | D-arabinose degradation I | 0 | Not detected | -0.059 | -0.304 | 0.187 | 0.640 |
| DENITRIFICATIO | nitrate reduction I (denitrification) | 0 | Not detected | 0.039 | -0.161 | 0.239 | 0.703 |
| DETOX1.PWY | superoxide radicals degradation | 0 | Not detected | 0.012 | -0.212 | 0.237 | 0.915 |
| DETOX1.PWY.1 | reactive oxygen species degradation | 0 | Not detected | 0.021 | -0.209 | 0.250 | 0.860 |
| DHGLUCONATE. | glucose degradation (oxidative) | 0 | Not detected | 0.093 | -0.132 | 0.319 | 0.417 |
| DISSULFRED.PW | dissimilatory sulfate reduction I (to hydrogen suf | 0 | Not detected | 0.190 | -0.034 | 0.414 | 0.097 |
| DTDPRHAMSYN. | dTDP-L-rhamnose biosynthesis | 0 | Not detected | -0.008 | -0.081 | 0.065 | 0.839 |
| ECASYN.PWY | enterobacterial common antigen biosynthesis | 0 | Not detected | 0.122 | -0.113 | 0.357 | 0.308 |
| ETOH.ACETYLCO | ethanol degradation I | 0 | Not detected | 0.105 | -0.146 | 0.355 | 0.414 |
| FAO.PWY | fatty acid &beta;-oxidation I | 0 | Not detected | 0.081 | -0.203 | 0.366 | 0.576 |
| FASYN.ELONG.P | fatty acid elongation -- saturated | 0 | Not detected | -0.138 | -0.389 | 0.112 | 0.278 |
| FERMENTATION. | mixed acid fermentation | 0 | Not detected | -0.002 | -0.072 | 0.067 | 0.947 |
| FHLMULTI.RXN | NA | 0 | Not detected | -0.025 | -0.248 | 0.199 | 0.829 |
| FUCCAT.PWY | fucose degradation | 0 | Not detected | -0.059 | -0.304 | 0.187 | 0.640 |
| GALACTARDEG. | D-galactarate degradation I | 0 | Not detected | -0.061 | -0.201 | 0.079 | 0.397 |
| GALACTCAT.PW | D-galactonate degradation | 0 | Not detected | 0.071 | -0.165 | 0.306 | 0.556 |
| GALACTUROCAT | D-galacturonate degradation I | 0 | Not detected | -0.017 | -0.088 | 0.055 | 0.644 |
| GCVMULTI.RXN | NA | 0 | Not detected | -0.006 | -0.189 | 0.176 | 0.947 |

| GDPRHAMSYN.P | GDP-D-rhamnose biosynthesis | 0 | Not detected | -0.049 | -0.294 | 0.195 | 0.693 |
| --- | --- | --- | --- | --- | --- | --- | --- |
| GLUCARDEG.PW | <i>D</i>-glucarate degradation I | 0 | Not detected | -0.138 | -0.284 | 0.008 | 0.064 |
| GLUCONEO.PWY | gluconeogenesis I | 0 | Not detected | 0.001 | -0.069 | 0.072 | 0.968 |
| GLUCOSE1PMET | glucose and glucose-1-phosphate degradation | 0 | Not detected | 0.029 | -0.080 | 0.138 | 0.605 |
| GLUDEG.I.PWY | GABA shunt | 0 | Not detected | 0.015 | -0.194 | 0.223 | 0.890 |
| GLUTATHIONES | glutathione biosynthesis | 0 | Not detected | -0.025 | -0.219 | 0.168 | 0.798 |
| GLUTORN.PWY | L-ornithine biosynthesis I | 0 | Not detected | 0.068 | -0.149 | 0.286 | 0.538 |
| GLYCLEAV.PWY | glycine cleavage | 0 | Not detected | -0.006 | -0.189 | 0.176 | 0.947 |
| GLYCOCAT.PWY | glycogen degradation I | 0 | Not detected | 0.042 | -0.063 | 0.147 | 0.430 |
| GLYCOGENSYNT | glycogen biosynthesis I (from ADP-D-Glucose) | 0 | Not detected | 0.077 | -0.016 | 0.169 | 0.103 |
| GLYCOLATEMET | glycolate and glyoxylate degradation I | 0 | Not detected | -0.014 | -0.237 | 0.210 | 0.905 |
| GLYCOLYSIS | glycolysis I (from glucose 6-phosphate) | 0 | Not detected | 0.001 | -0.069 | 0.071 | 0.977 |
| GLYCOLYSIS.E.D | superpathway of glycolysis and the Entner-Doud | 0 | Not detected | 0.027 | -0.173 | 0.226 | 0.794 |
| GLYOXDEG.PWY | glycolate and glyoxylate degradation II | 0 | Not detected | 0.060 | -0.169 | 0.289 | 0.608 |
| GLYOXYLATE.B | glyoxylate cycle | 0 | Not detected | 0.006 | -0.072 | 0.084 | 0.889 |
| HCAMHPDEG.PW | 3-phenylpropanoate and 3-(3-hydroxyphenyl)pro | 0 | Not detected | -0.030 | -0.229 | 0.168 | 0.764 |
| HEME.BIOSYNTH | heme <i>b</i> biosynthesis I (aerobic) | 0 | Not detected | 0.069 | -0.156 | 0.294 | 0.550 |
| HEMESYN2.PWY | heme <i>b</i> biosynthesis II (anaerobic) | 0 | Not detected | 0.187 | -0.040 | 0.414 | 0.107 |
| HISDEG.PWY | L-histidine degradation I | 0 | Not detected | -0.040 | -0.248 | 0.168 | 0.708 |
| HISHP.PWY | L-histidine degradation VI | 0 | Not detected | -0.040 | -0.248 | 0.168 | 0.708 |
| HISTSYN.PWY | L-histidine biosynthesis | 0 | Not detected | -0.021 | -0.096 | 0.053 | 0.575 |
| HOMOSER.METS | L-methionine biosynthesis I | 0 | Not detected | -0.017 | -0.089 | 0.054 | 0.632 |
| HOMOSER.THRE | L-threonine biosynthesis | 0 | Not detected | -0.002 | -0.235 | 0.231 | 0.986 |
| HOMOSERSYN.P | L-homoserine biosynthesis | 0 | Not detected | -0.018 | -0.238 | 0.203 | 0.875 |
| HSERMETANA.P | L-methionine biosynthesis III | 0 | Not detected | -0.026 | -0.099 | 0.047 | 0.489 |
| ILEUSYN.PWY | L-isoleucine biosynthesis I (from threonine) | 0 | Not detected | 0.028 | -0.060 | 0.116 | 0.532 |
| KDO.LIPASYN.P | (Kdo)<sub>2</sub>-lipid A biosynthesis I | 0 | Not detected | -0.006 | -0.203 | 0.191 | 0.951 |
| KDO.NAGLIPASY | superpathway of (Kdo)<SUB>2</SUB>-lipid A | 0 | Not detected | -0.026 | -0.248 | 0.195 | 0.815 |
| KDOSYN.PWY | Kdo transfer to lipid IV<sub>A</sub> I | 0 | Not detected | 0.070 | -0.160 | 0.301 | 0.550 |
| KETOGLUCONM | ketogluconate metabolism | 0 | Not detected | 0.042 | -0.149 | 0.233 | 0.665 |
| LACTOSECAT.PW | lactose and galactose degradation I | 0 | Not detected | 0.193 | -0.039 | 0.425 | 0.104 |
| LARABITOLUTIL | xylitol degradation | 0 | Not detected | 0.112 | -0.137 | 0.361 | 0.379 |
| LEUSYN.PWY | L-leucine biosynthesis | 0 | Not detected | -0.109 | -0.228 | 0.009 | 0.071 |
| LIPA.CORESYN.P | lipid A-core biosynthesis (<i>E. coli</i> K-12) | 0 | Not detected | 0.087 | -0.160 | 0.335 | 0.489 |
| LYSDEGII.PWY | L-lysine degradation III | 0 | Not detected | -0.085 | -0.263 | 0.093 | 0.350 |
| LYXMET.PWY | L-lyxose degradation | 0 | Not detected | 0.004 | -0.065 | 0.072 | 0.911 |
| MALATE.ASPART | L-aspartate degradation II | 0 | Not detected | -0.059 | -0.313 | 0.195 | 0.647 |
| MANNCAT.PWY | D-mannose degradation | 0 | Not detected | 0.028 | -0.183 | 0.240 | 0.793 |
| MANNIDEG.PWY | mannitol degradation I | 0 | Not detected | 0.057 | -0.167 | 0.281 | 0.621 |
| MENAQUINONES | menaquinol-8 biosynthesis | 0 | Not detected | 0.029 | -0.198 | 0.256 | 0.802 |
| METH.ACETATE. | methanogenesis from acetate | 0 | Not detected | 0.004 | -0.062 | 0.069 | 0.913 |
| NAGLIPASYN.PW | lipid IV<sub>A</sub> biosynthesis | 0 | Not detected | -0.088 | -0.323 | 0.146 | 0.460 |
| NONMEVIPP.PW | methylerythritol phosphate pathway I | 0 | Not detected | 0.000 | -0.077 | 0.076 | 0.992 |
| NONOXIPENT.PW | pentose phosphate pathway (non-oxidative branc | 0 | Not detected | 0.007 | -0.147 | 0.160 | 0.933 |

| NPGLUCAT.PWY | Entner-Doudoroff pathway II (non-phosphorylat | 0 | Not detected | -0.027 | -0.215 | 0.161 | 0.778 |
| --- | --- | --- | --- | --- | --- | --- | --- |
| OANTIGEN.PWY | <i>O</i>-antigen building blocks biosynthesis ( | 0 | Not detected | -0.037 | -0.123 | 0.048 | 0.391 |
| OXIDATIVEPENT | pentose phosphate pathway (oxidative branch) I | 0 | Not detected | -0.045 | -0.191 | 0.100 | 0.542 |
| P101.PWY | ectoine biosynthesis | 0 | Not detected | -0.019 | -0.240 | 0.201 | 0.864 |
| P105.PWY | TCA cycle IV (2-oxoglutarate decarboxylase) | 0 | Not detected | -0.001 | -0.076 | 0.073 | 0.973 |
| P108.PWY | pyruvate fermentation to propanoate I | 0 | Not detected | -0.092 | -0.183 | -0.001 | 0.048 |
| P121.PWY | adenine and adenosine salvage I | 0 | Not detected | 0.017 | -0.206 | 0.240 | 0.879 |
| P122.PWY | heterolactic fermentation | 0 | Not detected | -0.003 | -0.069 | 0.062 | 0.924 |
| P124.PWY | Bifidobacterium shunt | 0 | Not detected | -0.003 | -0.067 | 0.062 | 0.937 |
| P161.PWY | acetylene degradation (anaerobic) | 0 | Not detected | 0.000 | -0.060 | 0.061 | 0.991 |
| P162.PWY | L-glutamate degradation V (via hydroxyglutarate | 0 | Not detected | 0.040 | -0.051 | 0.132 | 0.387 |
| P163.PWY | L-lysine fermentation to acetate and butanoate | 0 | Not detected | 0.014 | -0.054 | 0.082 | 0.693 |
| P164.PWY | purine nucleobases degradation I (anaerobic) | 0 | Not detected | -0.040 | -0.108 | 0.027 | 0.242 |
| P181.PWY | nicotine degradation I (pyridine pathway) | 0 | Not detected | 0.009 | -0.190 | 0.207 | 0.930 |
| P185.PWY | formaldehyde assimilation III (dihydroxyacetone | 0 | Not detected | -0.001 | -0.069 | 0.067 | 0.977 |
| P2.PWY | citrate lyase activation | 0 | Not detected | 0.074 | -0.125 | 0.273 | 0.469 |
| P21.PWY | pentose phosphate pathway (partial) | 0 | Not detected | 0.007 | -0.147 | 0.160 | 0.933 |
| P23.PWY | reductive TCA cycle I | 0 | Not detected | -0.002 | -0.084 | 0.080 | 0.958 |
| P3.PWY | gallate degradation III (anaerobic) | 0 | Not detected | 0.012 | -0.055 | 0.080 | 0.723 |
| P341.PWY | glycolysis V (Pyrococcus) | 0 | Not detected | 0.004 | -0.068 | 0.077 | 0.906 |
| P42.PWY | incomplete reductive TCA cycle | 0 | Not detected | -0.059 | -0.163 | 0.044 | 0.263 |
| P562.PWY | <I>myo</I>-inositol degradation I | 0 | Not detected | 0.011 | -0.134 | 0.155 | 0.886 |
| PEPTIDOGLYCAN | peptidoglycan biosynthesis I (<I>meso</I>-diam | 0 | Not detected | -0.081 | -0.296 | 0.134 | 0.461 |
| PHOSLIPSYN2.PW | superpathway of phospholipid biosynthesis II (pl | 0 | Not detected | -0.034 | -0.241 | 0.172 | 0.745 |
| PHOSPHONOTAS | 2-aminoethylphosphonate degradation I | 0 | Not detected | 0.127 | -0.112 | 0.367 | 0.297 |
| PPGPPMET.PWY | ppGpp biosynthesis | 0 | Not detected | -0.049 | -0.111 | 0.013 | 0.122 |
| PUTDEG.PWY | putrescine degradation I | 0 | Not detected | 0.061 | -0.115 | 0.236 | 0.499 |
| PWY.1042 | glycolysis IV (plant cytosol) | 0 | Not detected | 0.001 | -0.069 | 0.070 | 0.986 |
| PWY.1186 | L-homomethionine biosynthesis | 0 | Not detected | 0.024 | -0.191 | 0.239 | 0.825 |
| PWY.1269 | CMP-3-deoxy-D-<I>manno</I>-octulosonate bi | 0 | Not detected | -0.075 | -0.305 | 0.155 | 0.522 |
| PWY.1281 | sulfoacetaldehyde degradation I | 0 | Not detected | 0.008 | -0.073 | 0.088 | 0.852 |
| PWY.1341 | phenylacetate degradation II (anaerobic) | 0 | Not detected | -0.084 | -0.201 | 0.032 | 0.155 |
| PWY.1361 | benzoyl-CoA degradation I (aerobic) | 0 | Not detected | -0.093 | -0.301 | 0.115 | 0.381 |
| PWY.1622 | formaldehyde assimilation I (serine pathway) | 0 | Not detected | -0.053 | -0.198 | 0.093 | 0.480 |
| PWY.1722 | formate assimilation into 5,10-methylenetetrahyd | 0 | Not detected | -0.111 | -0.223 | 0.001 | 0.052 |
| PWY.181 | photorespiration | 0 | Not detected | -0.060 | -0.224 | 0.104 | 0.475 |
| PWY.1861 | formaldehyde assimilation II (assimilatory RuM | 0 | Not detected | 0.013 | -0.069 | 0.094 | 0.759 |
| PWY.2 | putrescine degradation IV | 0 | Not detected | 0.061 | -0.115 | 0.236 | 0.499 |
| PWY.2161 | folate polyglutamylation | 0 | Not detected | -0.017 | -0.249 | 0.215 | 0.888 |
| PWY.2201 | folate transformations I | 0 | Not detected | -0.030 | -0.103 | 0.043 | 0.427 |
| PWY.2221 | Entner-Doudoroff pathway III (semi-phosphoryl | 0 | Not detected | -0.008 | -0.075 | 0.060 | 0.827 |
| PWY.2361 | 3-oxoadipate degradation | 0 | Not detected | -0.137 | -0.308 | 0.034 | 0.116 |
| PWY.2661 | trehalose biosynthesis V | 0 | Not detected | 0.226 | -0.026 | 0.477 | 0.079 |
| PWY.2722 | trehalose degradation IV | 0 | Not detected | -0.013 | -0.208 | 0.182 | 0.895 |

| PWY.2723 | trehalose degradation V | 0 | Not detected | 0.042 | -0.074 | 0.159 | 0.478 |
| --- | --- | --- | --- | --- | --- | --- | --- |
| PWY.2781 | <i>cis</i>-zeatin biosynthesis | 0 | Not detected | -0.118 | -0.329 | 0.092 | 0.271 |
| PWY.2941 | L-lysine biosynthesis II | 0 | Not detected | -0.018 | -0.238 | 0.203 | 0.875 |
| PWY.2942 | L-lysine biosynthesis III | 0 | Not detected | -0.018 | -0.238 | 0.203 | 0.875 |
| PWY.3 | putrescine degradation V | 0 | Not detected | 0.061 | -0.116 | 0.238 | 0.500 |
| PWY.3801 | sucrose degradation II (sucrose synthase) | 0 | Not detected | 0.003 | -0.063 | 0.068 | 0.931 |
| PWY.3821 | D-galactose detoxification | 0 | Not detected | -0.050 | -0.162 | 0.061 | 0.378 |
| PWY.3841 | folate transformations II | 0 | Not detected | -0.029 | -0.101 | 0.043 | 0.426 |
| PWY.3861 | mannitol degradation II | 0 | Not detected | 0.029 | -0.193 | 0.250 | 0.801 |
| PWY.3881 | mannitol biosynthesis | 0 | Not detected | 0.025 | -0.196 | 0.247 | 0.823 |
| PWY.3941 | &beta;-alanine biosynthesis II | 0 | Not detected | -0.009 | -0.244 | 0.227 | 0.941 |
| PWY.4041 | &gamma;-glutamyl cycle | 0 | Not detected | -0.060 | -0.287 | 0.167 | 0.605 |
| PWY.4101 | D-sorbitol degradation I | 0 | Not detected | 0.113 | -0.109 | 0.335 | 0.318 |
| PWY.43 | putrescine biosynthesis II | 0 | Not detected | -0.018 | -0.127 | 0.090 | 0.742 |
| PWY.4321 | L-glutamate degradation IV | 0 | Not detected | 0.027 | -0.180 | 0.234 | 0.799 |
| PWY.4361 | <i>S</i>-methyl-5-thio-&alpha;-D-ribose 1-pho | 0 | Not detected | -0.063 | -0.295 | 0.168 | 0.590 |
| PWY.4381 | fatty acid biosynthesis initiation I | 0 | Not detected | 0.099 | -0.118 | 0.315 | 0.372 |
| PWY.4702 | phytate degradation I | 0 | Not detected | 0.067 | -0.157 | 0.291 | 0.557 |
| PWY.4983 | nitric oxide biosynthesis II (mammals) | 0 | Not detected | -0.066 | -0.184 | 0.052 | 0.274 |
| PWY.4984 | urea cycle | 0 | Not detected | 0.056 | -0.041 | 0.153 | 0.261 |
| PWY.5022 | 4-aminobutanoate degradation V | 0 | Not detected | 0.018 | -0.066 | 0.103 | 0.667 |
| PWY.5028 | L-histidine degradation II | 0 | Not detected | -0.036 | -0.245 | 0.173 | 0.736 |
| PWY.5030 | L-histidine degradation III | 0 | Not detected | -0.093 | -0.200 | 0.013 | 0.087 |
| PWY.5041 | <i>S</i>-adenosyl-L-methionine cycle II | 0 | Not detected | 0.031 | -0.204 | 0.265 | 0.797 |
| PWY.5049 | rosmarinic acid biosynthesis II | 0 | Not detected | -0.040 | -0.209 | 0.129 | 0.644 |
| PWY.5054 | D-sorbitol biosynthesis I | 0 | Not detected | 0.002 | -0.072 | 0.075 | 0.964 |
| PWY.5097 | L-lysine biosynthesis VI | 0 | Not detected | -0.010 | -0.231 | 0.211 | 0.931 |
| PWY.5101 | L-isoleucine biosynthesis II | 0 | Not detected | -0.026 | -0.247 | 0.195 | 0.817 |
| PWY.5103 | L-isoleucine biosynthesis III | 0 | Not detected | 0.028 | -0.060 | 0.116 | 0.532 |
| PWY.5104 | L-isoleucine biosynthesis IV | 0 | Not detected | 0.050 | -0.187 | 0.287 | 0.679 |
| PWY.5122 | geranyl diphosphate biosynthesis | 0 | Not detected | 0.046 | -0.157 | 0.248 | 0.660 |
| PWY.5123 | <i>trans, trans</i>-farnesyl diphosphate biosynt | 0 | Not detected | 0.073 | -0.166 | 0.312 | 0.550 |
| PWY.5136 | fatty acid &beta;-oxidation II (peroxisome) | 0 | Not detected | 0.117 | -0.144 | 0.379 | 0.379 |
| PWY.5137 | fatty acid &beta;-oxidation III (unsaturated, odd | 0 | Not detected | 0.037 | -0.166 | 0.240 | 0.722 |
| PWY.5138 | unsaturated, even numbered fatty acid &beta;-ox | 0 | Not detected | 0.053 | -0.180 | 0.286 | 0.655 |
| PWY.5154 | L-arginine biosynthesis III (via <i>N</i>-acetyl- | 0 | Not detected | -0.059 | -0.174 | 0.056 | 0.312 |
| PWY.5162 | 2-hydroxypenta-2,4-dienoate degradation | 0 | Not detected | 0.136 | -0.104 | 0.376 | 0.266 |
| PWY.5177 | glutaryl-CoA degradation | 0 | Not detected | 0.014 | -0.112 | 0.140 | 0.826 |
| PWY.5188 | tetrapyrrole biosynthesis I (from glutamate) | 0 | Not detected | 0.007 | -0.101 | 0.115 | 0.897 |
| PWY.5189 | tetrapyrrole biosynthesis II (from glycine) | 0 | Not detected | 0.112 | -0.048 | 0.271 | 0.170 |
| PWY.5265 | peptidoglycan biosynthesis II (staphylococci) | 0 | Not detected | -0.016 | -0.143 | 0.111 | 0.808 |
| PWY.5269 | cardiolipin biosynthesis II | 0 | Not detected | 0.023 | -0.184 | 0.230 | 0.825 |
| PWY.5280 | L-lysine degradation IV | 0 | Not detected | -0.089 | -0.271 | 0.093 | 0.338 |
| PWY.5306 | superpathway of thiosulfate metabolism (<i>Des | 0 | Not detected | 0.190 | -0.034 | 0.414 | 0.097 |

| PWY.5316 | nicotine biosynthesis | 0 | Not detected | -0.032 | -0.298 | 0.235 | 0.814 |
| --- | --- | --- | --- | --- | --- | --- | --- |
| PWY.5344 | L-homocysteine biosynthesis | 0 | Not detected | 0.010 | -0.069 | 0.088 | 0.809 |
| PWY.5352 | thiosulfate disproportionation II (cytochrome) | 0 | Not detected | 0.170 | -0.042 | 0.382 | 0.116 |
| PWY.5360 | superpathway of tetrathionate reduction (<i>Sal | 0 | Not detected | 0.190 | -0.034 | 0.414 | 0.097 |
| PWY.5381 | pyridine nucleotide cycling (plants) | 0 | Not detected | -0.103 | -0.322 | 0.116 | 0.356 |
| PWY.5384 | sucrose degradation IV (sucrose phosphorylase) | 0 | Not detected | 0.005 | -0.060 | 0.071 | 0.879 |
| PWY.5386 | methylglyoxal degradation I | 0 | Not detected | 0.090 | -0.130 | 0.309 | 0.424 |
| PWY.5392 | reductive TCA cycle II | 0 | Not detected | -0.001 | -0.084 | 0.083 | 0.990 |
| PWY.5394 | betalamic acid biosynthesis | 0 | Not detected | -0.064 | -0.225 | 0.097 | 0.438 |
| PWY.5436 | L-threonine degradation IV | 0 | Not detected | 0.127 | -0.112 | 0.367 | 0.297 |
| PWY.5437 | L-threonine degradation I | 0 | Not detected | 0.225 | -0.011 | 0.461 | 0.062 |
| PWY.5453 | methylglyoxal degradation III | 0 | Not detected | -0.023 | -0.090 | 0.045 | 0.505 |
| PWY.5458 | methylglyoxal degradation V | 0 | Not detected | 0.027 | -0.188 | 0.241 | 0.809 |
| PWY.5459 | methylglyoxal degradation IV | 0 | Not detected | 0.026 | -0.188 | 0.241 | 0.810 |
| PWY.5480 | pyruvate fermentation to ethanol I | 0 | Not detected | 0.105 | -0.146 | 0.355 | 0.414 |
| PWY.5482 | pyruvate fermentation to acetate II | 0 | Not detected | 0.004 | -0.062 | 0.069 | 0.913 |
| PWY.5484 | glycolysis II (from fructose 6-phosphate) | 0 | Not detected | 0.000 | -0.068 | 0.069 | 0.993 |
| PWY.5485 | pyruvate fermentation to acetate IV | 0 | Not detected | 0.004 | -0.062 | 0.069 | 0.913 |
| PWY.5486 | pyruvate fermentation to ethanol II | 0 | Not detected | -0.112 | -0.319 | 0.095 | 0.288 |
| PWY.5497 | purine nucleobases degradation II (anaerobic) | 0 | Not detected | -0.016 | -0.083 | 0.051 | 0.636 |
| PWY.5509 | adenosylcobalamin biosynthesis from adenosylc | 0 | Not detected | -0.020 | -0.194 | 0.153 | 0.819 |
| PWY.5512 | UDP-<i>N</i>-acetyl-D-galactosamine biosynth | 0 | Not detected | -0.169 | -0.448 | 0.110 | 0.236 |
| PWY.5514 | UDP-<i>N</i>-acetyl-D-galactosamine biosynth | 0 | Not detected | -0.002 | -0.077 | 0.072 | 0.949 |
| PWY.5523 | 5,6-dimethylbenzimidazole biosynthesis I (aerob | 0 | Not detected | -0.102 | -0.303 | 0.099 | 0.319 |
| PWY.5531 | 3,8-divinyl-chlorophyllide <i>a</i> biosynthesis | 0 | Not detected | 0.152 | -0.074 | 0.378 | 0.189 |
| PWY.5532 | nucleoside and nucleotide degradation (archaea) | 0 | Not detected | -0.006 | -0.179 | 0.166 | 0.943 |
| PWY.5536 | acetate formation from acetyl-CoA (succinate) | 0 | Not detected | -0.133 | -0.283 | 0.018 | 0.084 |
| PWY.561 | superpathway of glyoxylate cycle and fatty acid | 0 | Not detected | -0.007 | -0.079 | 0.065 | 0.851 |
| PWY.5659 | GDP-mannose biosynthesis | 0 | Not detected | 0.005 | -0.064 | 0.073 | 0.894 |
| PWY.5661 | GDP-glucose biosynthesis | 0 | Not detected | 0.042 | -0.074 | 0.159 | 0.478 |
| PWY.5663 | erythro-tetrahydrobiopterin biosynthesis I | 0 | Not detected | 0.057 | -0.072 | 0.186 | 0.386 |
| PWY.5664 | <i>erythro</i>-tetrahydrobiopterin biosynthesis | 0 | Not detected | 0.057 | -0.072 | 0.186 | 0.386 |
| PWY.5667 | CDP-diacylglycerol biosynthesis I | 0 | Not detected | 0.059 | -0.124 | 0.242 | 0.529 |
| PWY.5668 | cardiolipin biosynthesis I | 0 | Not detected | 0.027 | -0.211 | 0.266 | 0.822 |
| PWY.5669 | phosphatidylserine and phosphatidylethanolamin | 0 | Not detected | -0.034 | -0.241 | 0.172 | 0.745 |
| PWY.5676 | acetyl-CoA fermentation to butanoate II | 0 | Not detected | 0.029 | -0.055 | 0.112 | 0.499 |
| PWY.5677 | succinate fermentation to butanoate | 0 | Not detected | 0.001 | -0.087 | 0.089 | 0.979 |
| PWY.5686 | UMP biosynthesis I | 0 | Not detected | 0.015 | -0.059 | 0.089 | 0.683 |
| PWY.5690 | TCA cycle II (plants and fungi) | 0 | Not detected | -0.001 | -0.084 | 0.083 | 0.988 |
| PWY.5692 | allantoin degradation to glyoxylate II | 0 | Not detected | -0.034 | -0.235 | 0.167 | 0.737 |
| PWY.5698 | allantoin degradation to ureidoglycolate II (amm | 0 | Not detected | -0.034 | -0.235 | 0.167 | 0.737 |
| PWY.5723 | Rubisco shunt | 0 | Not detected | -0.017 | -0.091 | 0.058 | 0.665 |
| PWY.5738 | GDP-6-deoxy-D-talose biosynthesis | 0 | Not detected | -0.049 | -0.294 | 0.195 | 0.693 |
| PWY.5739 | GDP-D-perosamine biosynthesis | 0 | Not detected | -0.049 | -0.294 | 0.195 | 0.693 |

| PWY.5740 | GDP-L-colitose biosynthesis | 0 | Not detected | -0.049 | -0.294 | 0.195 | 0.693 |
| --- | --- | --- | --- | --- | --- | --- | --- |
| PWY.5741 | ethylmalonyl-CoA pathway | 0 | Not detected | -0.058 | -0.251 | 0.134 | 0.552 |
| PWY.5743 | 3-hydroxypropanoate cycle | 0 | Not detected | -0.019 | -0.115 | 0.077 | 0.695 |
| PWY.5744 | glyoxylate assimilation | 0 | Not detected | -0.019 | -0.115 | 0.077 | 0.703 |
| PWY.5747 | 2-methylcitrate cycle II | 0 | Not detected | -0.009 | -0.263 | 0.245 | 0.944 |
| PWY.5757 | fosfomycin biosynthesis | 0 | Not detected | -0.037 | -0.309 | 0.235 | 0.790 |
| PWY.5785 | di-<i>trans</i>,poly-<i>cis</i>-undecaprenyl ph | 0 | Not detected | 0.074 | -0.132 | 0.279 | 0.483 |
| PWY.5789 | 3-hydroxypropanoate/4-hydroxybutanate cycle | 0 | Not detected | 0.013 | -0.070 | 0.096 | 0.756 |
| PWY.5794 | malonate degradation I (biotin-independent) | 0 | Not detected | -0.065 | -0.281 | 0.152 | 0.557 |
| PWY.5796 | malonate decarboxylase activation | 0 | Not detected | 0.080 | -0.091 | 0.252 | 0.357 |
| PWY.5837 | 2-carboxy-1,4-naphthoquinol biosynthesis | 0 | Not detected | 0.014 | -0.064 | 0.092 | 0.727 |
| PWY.5852 | demethylmenaquinol-8 biosynthesis I | 0 | Not detected | -0.030 | -0.217 | 0.157 | 0.753 |
| PWY.5870 | ubiquinol-8 biosynthesis (eukaryotic) | 0 | Not detected | 0.140 | -0.096 | 0.377 | 0.246 |
| PWY.5875 | staphyloxanthin biosynthesis | 0 | Not detected | 0.086 | -0.158 | 0.331 | 0.489 |
| PWY.5901 | 2,3-dihydroxybenzoate biosynthesis | 0 | Not detected | -0.143 | -0.318 | 0.032 | 0.110 |
| PWY.5913 | partial TCA cycle (obligate autotrophs) | 0 | Not detected | -0.002 | -0.084 | 0.080 | 0.956 |
| PWY.5921 | glutaminyl-tRNA<sup>gln</sup> biosynthesis v | 0 | Not detected | 0.096 | -0.154 | 0.347 | 0.451 |
| PWY.5940 | streptomycin biosynthesis | 0 | Not detected | 0.037 | -0.077 | 0.151 | 0.524 |
| PWY.5941 | glycogen degradation II | 0 | Not detected | 0.042 | -0.074 | 0.159 | 0.478 |
| PWY.5971 | palmitate biosynthesis II (bacteria and plants) | 0 | Not detected | -0.024 | -0.175 | 0.127 | 0.755 |
| PWY.5981 | CDP-diacylglycerol biosynthesis III | 0 | Not detected | 0.175 | -0.005 | 0.355 | 0.057 |
| PWY.5994 | palmitate biosynthesis I (animals and fungi) | 0 | Not detected | -0.052 | -0.220 | 0.117 | 0.547 |
| PWY.6012 | acyl carrier protein metabolism | 0 | Not detected | -0.027 | -0.192 | 0.137 | 0.744 |
| PWY.6019 | pseudouridine degradation | 0 | Not detected | 0.102 | -0.098 | 0.301 | 0.317 |
| PWY.6053 | dimethylsulfoniopropanoate biosynthesis III (alg | 0 | Not detected | 0.024 | -0.191 | 0.239 | 0.825 |
| PWY.6060 | malonate degradation II (biotin-dependent) | 0 | Not detected | -0.169 | -0.300 | -0.039 | 0.011 |
| PWY.6121 | 5-aminoimidazole ribonucleotide biosynthesis I | 0 | Not detected | -0.033 | -0.118 | 0.052 | 0.448 |
| PWY.6122 | 5-aminoimidazole ribonucleotide biosynthesis II | 0 | Not detected | -0.042 | -0.108 | 0.025 | 0.220 |
| PWY.6123 | inosine-5'-phosphate biosynthesis I | 0 | Not detected | 0.078 | -0.058 | 0.214 | 0.262 |
| PWY.6124 | inosine-5'-phosphate biosynthesis II | 0 | Not detected | 0.078 | -0.058 | 0.214 | 0.262 |
| PWY.6138 | CMP-<i>N</i>-acetylneuraminate biosynthesis I | 0 | Not detected | 0.026 | -0.160 | 0.212 | 0.787 |
| PWY.6140 | CMP-2-keto-3-deoxy-D-<i>glycero</i>-D-<i>g | 0 | Not detected | -0.317 | -0.573 | -0.062 | 0.015 |
| PWY.6142 | gluconeogenesis II (<i>Methanobacterium therm | 0 | Not detected | 0.002 | -0.070 | 0.074 | 0.961 |
| PWY.6143 | CMP-pseudaminate biosynthesis | 0 | Not detected | -0.033 | -0.206 | 0.140 | 0.711 |
| PWY.6146 | <i>Methanobacterium thermoautotrophicum</i> | 0 | Not detected | -0.021 | -0.245 | 0.204 | 0.856 |
| PWY.6147 | 6-hydroxymethyl-dihydropterin diphosphate bio | 0 | Not detected | 0.041 | -0.067 | 0.150 | 0.455 |
| PWY.6148 | tetrahydromethanopterin biosynthesis | 0 | Not detected | 0.114 | -0.069 | 0.297 | 0.221 |
| PWY.6151 | <i>S</i>-adenosyl-L-methionine cycle I | 0 | Not detected | 0.061 | -0.182 | 0.304 | 0.623 |
| PWY.6153 | autoinducer AI-2 biosynthesis I | 0 | Not detected | 0.054 | -0.128 | 0.235 | 0.564 |
| PWY.6154 | autoinducer AI-2 biosynthesis II (<i>Vibrio</i>) | 0 | Not detected | 0.054 | -0.128 | 0.235 | 0.564 |
| PWY.6160 | 3-dehydroquinate biosynthesis II (archaea) | 0 | Not detected | -0.020 | -0.240 | 0.200 | 0.859 |
| PWY.6163 | chorismate biosynthesis from 3-dehydroquinate | 0 | Not detected | 0.024 | -0.143 | 0.192 | 0.776 |
| PWY.6164 | 3-dehydroquinate biosynthesis I | 0 | Not detected | -0.058 | -0.274 | 0.159 | 0.602 |
| PWY.6167 | flavin biosynthesis II (archaea) | 0 | Not detected | -0.017 | -0.087 | 0.053 | 0.630 |

| PWY.6168 | flavin biosynthesis III (fungi) | 0 | Not detected | -0.018 | -0.090 | 0.055 | 0.635 |
| --- | --- | --- | --- | --- | --- | --- | --- |
| PWY.6174 | mevalonate pathway II (archaea) | 0 | Not detected | 0.044 | -0.117 | 0.205 | 0.592 |
| PWY.621 | sucrose degradation III (sucrose invertase) | 0 | Not detected | 0.000 | -0.070 | 0.069 | 0.991 |
| PWY.622 | starch biosynthesis | 0 | Not detected | 0.005 | -0.060 | 0.070 | 0.886 |
| PWY.6269 | superpathway of adenosylcobalamin salvage fro | 0 | Not detected | 0.060 | -0.133 | 0.253 | 0.543 |
| PWY.6277 | superpathway of 5-aminoimidazole ribonucleotid | 0 | Not detected | -0.042 | -0.108 | 0.025 | 0.220 |
| PWY.6317 | D-galactose degradation I (Leloir pathway) | 0 | Not detected | -0.020 | -0.090 | 0.050 | 0.576 |
| PWY.6318 | L-phenylalanine degradation IV (mammalian, vi | 0 | Not detected | -0.084 | -0.201 | 0.032 | 0.155 |
| PWY.6322 | phosphinothricin tripeptide biosynthesis | 0 | Not detected | -0.017 | -0.264 | 0.229 | 0.890 |
| PWY.6328 | L-lysine degradation X | 0 | Not detected | -0.089 | -0.271 | 0.093 | 0.338 |
| PWY.6333 | acetaldehyde biosynthesis I | 0 | Not detected | -0.112 | -0.319 | 0.095 | 0.288 |
| PWY.6383 | mono-<i>trans</i>, poly-<i>cis</i> decaprenyl | 0 | Not detected | 0.102 | -0.114 | 0.317 | 0.354 |
| PWY.6386 | UDP-<i>N</i>-acetylmuramoyl-pentapeptide bi | 0 | Not detected | 0.054 | -0.011 | 0.119 | 0.103 |
| PWY.6387 | UDP-<i>N</i>-acetylmuramoyl-pentapeptide bi | 0 | Not detected | 0.051 | -0.010 | 0.111 | 0.102 |
| PWY.6397 | mycolyl-arabinogalactan-peptidoglycan complex | 0 | Not detected | -0.049 | -0.134 | 0.036 | 0.257 |
| PWY.6406 | salicylate biosynthesis I | 0 | Not detected | -0.143 | -0.318 | 0.032 | 0.110 |
| PWY.6416 | quinate degradation II | 0 | Not detected | 0.028 | -0.180 | 0.237 | 0.790 |
| PWY.6461 | peptidoglycan cross-bridge biosynthesis II (E. fa | 0 | Not detected | 0.059 | -0.196 | 0.313 | 0.652 |
| PWY.6471 | peptidoglycan biosynthesis IV (Enterococcus fae | 0 | Not detected | -0.011 | -0.137 | 0.115 | 0.863 |
| PWY.6473 | 4-aminobutanoate degradation IV | 0 | Not detected | 0.085 | -0.091 | 0.262 | 0.343 |
| PWY.6478 | GDP-D-<i>glycero</i>-&alpha;-D-<i>manno</i | 0 | Not detected | 0.132 | -0.076 | 0.339 | 0.214 |
| PWY.6497 | D-galactarate degradation II | 0 | Not detected | 0.058 | -0.163 | 0.279 | 0.609 |
| PWY.6499 | D-glucarate degradation II | 0 | Not detected | -0.197 | -0.416 | 0.021 | 0.077 |
| PWY.6507 | 4-deoxy-L-<i>threo</i>-hex-4-enopyranuronate | 0 | Not detected | -0.021 | -0.092 | 0.051 | 0.568 |
| PWY.6517 | <i>N</i>-acetylglucosamine degradation II | 0 | Not detected | -0.057 | -0.142 | 0.028 | 0.186 |
| PWY.6518 | glycocholate metabolism (bacteria) | 0 | Not detected | 0.079 | -0.163 | 0.321 | 0.522 |
| PWY.6519 | 8-amino-7-oxononanoate biosynthesis I | 0 | Not detected | 0.031 | -0.160 | 0.223 | 0.747 |
| PWY.6527 | stachyose degradation | 0 | Not detected | -0.026 | -0.109 | 0.056 | 0.533 |
| PWY.6531 | mannitol cycle | 0 | Not detected | 0.127 | -0.080 | 0.334 | 0.230 |
| PWY.6535 | 4-aminobutanoate degradation I | 0 | Not detected | 0.027 | -0.180 | 0.234 | 0.799 |
| PWY.6536 | 4-aminobutanoate degradation III | 0 | Not detected | -0.093 | -0.274 | 0.088 | 0.312 |
| PWY.6537 | 4-aminobutanoate degradation II | 0 | Not detected | -0.084 | -0.262 | 0.093 | 0.352 |
| PWY.6545 | pyrimidine deoxyribonucleotides <i>de novo</i> | 0 | Not detected | -0.046 | -0.132 | 0.039 | 0.288 |
| PWY.6549 | L-glutamine biosynthesis III | 0 | Not detected | -0.001 | -0.086 | 0.084 | 0.980 |
| PWY.6556 | pyrimidine ribonucleosides salvage II | 0 | Not detected | -0.082 | -0.255 | 0.091 | 0.354 |
| PWY.6559 | spermidine biosynthesis II | 0 | Not detected | 0.004 | -0.077 | 0.084 | 0.929 |
| PWY.6562 | norspermidine biosynthesis | 0 | Not detected | -0.053 | -0.266 | 0.159 | 0.624 |
| PWY.6572 | chondroitin sulfate degradation I (bacterial) | 0 | Not detected | -0.125 | -0.355 | 0.104 | 0.285 |
| PWY.6575 | juvenile hormone III biosynthesis I | 0 | Not detected | 0.036 | -0.153 | 0.224 | 0.711 |
| PWY.6583 | pyruvate fermentation to butanol I | 0 | Not detected | 0.028 | -0.060 | 0.116 | 0.534 |
| PWY.6587 | pyruvate fermentation to ethanol III | 0 | Not detected | 0.105 | -0.146 | 0.355 | 0.414 |
| PWY.6596 | adenosine nucleotides degradation I | 0 | Not detected | -0.051 | -0.267 | 0.164 | 0.640 |
| PWY.66 | GDP-L-fucose biosynthesis I (from GDP-D-man | 0 | Not detected | -0.049 | -0.294 | 0.195 | 0.693 |
| PWY.6605 | adenine and adenosine salvage II | 0 | Not detected | -0.106 | -0.330 | 0.117 | 0.352 |

| PWY.6606 | guanosine nucleotides degradation II | 0 | Not detected | -0.071 | -0.284 | 0.142 | 0.511 |
| --- | --- | --- | --- | --- | --- | --- | --- |
| PWY.6607 | guanosine nucleotides degradation I | 0 | Not detected | -0.045 | -0.242 | 0.151 | 0.652 |
| PWY.6608 | guanosine nucleotides degradation III | 0 | Not detected | -0.181 | -0.407 | 0.046 | 0.117 |
| PWY.6610 | adenine salvage | 0 | Not detected | 0.031 | -0.197 | 0.258 | 0.792 |
| PWY.6613 | tetrahydrofolate salvage from 5,10-methenyltetra | 0 | Not detected | -0.110 | -0.212 | -0.008 | 0.034 |
| PWY.6614 | tetrahydrofolate biosynthesis | 0 | Not detected | -0.001 | -0.205 | 0.204 | 0.995 |
| PWY.6620 | guanine and guanosine salvage | 0 | Not detected | -0.205 | -0.439 | 0.029 | 0.085 |
| PWY.6626 | cytidine-5'-diphosphate-glycerol biosynthesis | 0 | Not detected | 0.168 | -0.040 | 0.375 | 0.113 |
| PWY.6637 | sulfolactate degradation II | 0 | Not detected | 0.008 | -0.073 | 0.088 | 0.852 |
| PWY.6654 | phosphopantothenate biosynthesis III (archaebac | 0 | Not detected | -0.003 | -0.176 | 0.169 | 0.969 |
| PWY.6672 | <i>cis</i>-genanyl-CoA degradation | 0 | Not detected | 0.035 | -0.188 | 0.258 | 0.761 |
| PWY.6682 | dehydrophos biosynthesis | 0 | Not detected | -0.042 | -0.289 | 0.205 | 0.740 |
| PWY.6690 | cinnamate and 3-hydroxycinnamate degradation | 0 | Not detected | -0.030 | -0.229 | 0.168 | 0.764 |
| PWY.6700 | queuosine biosynthesis | 0 | Not detected | -0.067 | -0.182 | 0.049 | 0.256 |
| PWY.6703 | preQ<sub>0</sub> biosynthesis | 0 | Not detected | 0.048 | -0.063 | 0.160 | 0.395 |
| PWY.6707 | gallate biosynthesis | 0 | Not detected | 0.028 | -0.180 | 0.237 | 0.790 |
| PWY.6708 | ubiquinol-8 biosynthesis (prokaryotic) | 0 | Not detected | 0.128 | -0.110 | 0.365 | 0.292 |
| PWY.6717 | (1,4)-&beta;-D-xylan degradation | 0 | Not detected | 0.112 | -0.076 | 0.300 | 0.244 |
| PWY.6724 | starch degradation II | 0 | Not detected | 0.261 | 0.034 | 0.487 | 0.024 |
| PWY.6728 | methylaspartate cycle | 0 | Not detected | -0.002 | -0.085 | 0.080 | 0.955 |
| PWY.6731 | starch degradation III | 0 | Not detected | 0.045 | -0.072 | 0.163 | 0.452 |
| PWY.6737 | starch degradation V | 0 | Not detected | 0.045 | -0.073 | 0.163 | 0.454 |
| PWY.6748 | nitrate reduction VII (denitrification) | 0 | Not detected | -0.028 | -0.257 | 0.201 | 0.810 |
| PWY.6749 | CMP-legionaminate biosynthesis I | 0 | Not detected | -0.041 | -0.240 | 0.158 | 0.685 |
| PWY.6771 | rhamnogalacturonan type I degradation II (bacter | 0 | Not detected | -0.025 | -0.176 | 0.126 | 0.747 |
| PWY.6772 | hydrogen production V | 0 | Not detected | -0.024 | -0.248 | 0.199 | 0.830 |
| PWY.6784 | cellulose and hemicellulose degradation (cellulol | 0 | Not detected | 0.109 | -0.081 | 0.299 | 0.261 |
| PWY.6786 | detoxification of reactive carbonyls in chloroplas | 0 | Not detected | -0.022 | -0.090 | 0.045 | 0.515 |
| PWY.6788 | cellulose degradation II (fungi) | 0 | Not detected | 0.081 | -0.125 | 0.286 | 0.441 |
| PWY.6799 | fatty acid biosynthesis (plant mitochondria) | 0 | Not detected | 0.097 | -0.120 | 0.314 | 0.382 |
| PWY.6808 | dTDP-D-forosamine biosynthesis | 0 | Not detected | -0.006 | -0.078 | 0.066 | 0.870 |
| PWY.6823 | molybdenum cofactor biosynthesis | 0 | Not detected | 0.129 | -0.092 | 0.351 | 0.253 |
| PWY.6839 | 2-aminoethylphosphonate biosynthesis | 0 | Not detected | -0.042 | -0.289 | 0.205 | 0.740 |
| PWY.6840 | homoglutathione biosynthesis | 0 | Not detected | -0.025 | -0.219 | 0.168 | 0.798 |
| PWY.6842 | glutathione-mediated detoxification II | 0 | Not detected | 0.069 | -0.124 | 0.262 | 0.481 |
| PWY.6854 | ethylene biosynthesis III (microbes) | 0 | Not detected | 0.028 | -0.207 | 0.263 | 0.817 |
| PWY.6855 | chitin degradation I (archaea) | 0 | Not detected | 0.147 | -0.060 | 0.353 | 0.164 |
| PWY.6859 | <i>all-trans</i>-farnesol biosynthesis | 0 | Not detected | 0.073 | -0.166 | 0.312 | 0.550 |
| PWY.6863 | pyruvate fermentation to hexanol (engineered) | 0 | Not detected | 0.019 | -0.074 | 0.113 | 0.686 |
| PWY.6871 | 3-methylbutanol biosynthesis (engineered) | 0 | Not detected | -0.109 | -0.228 | 0.009 | 0.071 |
| PWY.6883 | pyruvate fermentation to butanol II (engineered) | 0 | Not detected | 0.020 | -0.074 | 0.114 | 0.676 |
| PWY.6886 | 1-butanol autotrophic biosynthesis (engineered) | 0 | Not detected | -0.010 | -0.077 | 0.058 | 0.783 |
| PWY.6890 | 4-amino-2-methyl-5-diphosphomethylpyrimidin | 0 | Not detected | -0.004 | -0.091 | 0.083 | 0.927 |
| PWY.6891 | thiazole biosynthesis II (aerobic bacteria) | 0 | Not detected | -0.044 | -0.119 | 0.031 | 0.252 |

| PWY.6892 | thiazole biosynthesis I (facultative anaerobic bac | 0 | Not detected | -0.041 | -0.104 | 0.022 | 0.206 |
| --- | --- | --- | --- | --- | --- | --- | --- |
| PWY.6893 | thiamine diphosphate biosynthesis II (Bacillus) | 0 | Not detected | 0.006 | -0.172 | 0.185 | 0.945 |
| PWY.6894 | thiamine diphosphate biosynthesis I (E. coli) | 0 | Not detected | 0.058 | -0.137 | 0.252 | 0.562 |
| PWY.6896 | thiamine salvage I | 0 | Not detected | 0.067 | -0.128 | 0.262 | 0.499 |
| PWY.6897 | thiamine salvage II | 0 | Not detected | 0.058 | -0.137 | 0.252 | 0.562 |
| PWY.6901 | superpathway of glucose and xylose degradation | 0 | Not detected | -0.009 | -0.077 | 0.058 | 0.788 |
| PWY.6902 | chitin degradation II (Vibrio) | 0 | Not detected | 0.156 | -0.049 | 0.362 | 0.135 |
| PWY.6906 | chitin derivatives degradation | 0 | Not detected | -0.046 | -0.117 | 0.025 | 0.205 |
| PWY.6938 | NADH repair | 0 | Not detected | 0.070 | -0.131 | 0.271 | 0.496 |
| PWY.6942 | dTDP-D-desosamine biosynthesis | 0 | Not detected | -0.008 | -0.080 | 0.064 | 0.831 |
| PWY.6944 | androstenedione degradation | 0 | Not detected | -0.100 | -0.318 | 0.118 | 0.370 |
| PWY.6949 | DIBOA-glucoside biosynthesis | 0 | Not detected | 0.012 | -0.151 | 0.176 | 0.882 |
| PWY.6952 | glycerophosphodiester degradation | 0 | Not detected | 0.001 | -0.161 | 0.163 | 0.991 |
| PWY.6953 | dTDP-3-acetamido-&alpha;-D-fucose biosynthe | 0 | Not detected | -0.010 | -0.083 | 0.062 | 0.778 |
| PWY.6961 | L-ascorbate degradation II (bacterial, aerobic) | 0 | Not detected | 0.008 | -0.059 | 0.074 | 0.825 |
| PWY.6969 | TCA cycle V (2-oxoglutarate:ferredoxin oxidore | 0 | Not detected | 0.001 | -0.074 | 0.075 | 0.988 |
| PWY.6973 | dTDP-D-olivose, dTDP-D-oliose and dTDP-D-m | 0 | Not detected | -0.006 | -0.078 | 0.066 | 0.870 |
| PWY.6974 | dTDP-L-olivose biosynthesis | 0 | Not detected | -0.006 | -0.078 | 0.066 | 0.870 |
| PWY.6976 | dTDP-L-mycarose biosynthesis | 0 | Not detected | -0.007 | -0.079 | 0.065 | 0.852 |
| PWY.6981 | chitin biosynthesis | 0 | Not detected | 0.005 | -0.064 | 0.075 | 0.884 |
| PWY.6983 | <i>threo</i>-tetrahydrobiopterin biosynthesis | 0 | Not detected | 0.057 | -0.072 | 0.186 | 0.386 |
| PWY.6984 | lipoate salvage II | 0 | Not detected | 0.046 | -0.120 | 0.213 | 0.585 |
| PWY.6986 | alginate degradation | 0 | Not detected | -0.015 | -0.091 | 0.061 | 0.703 |
| PWY.6987 | lipoate biosynthesis and incorporation III (Bacill | 0 | Not detected | -0.112 | -0.311 | 0.086 | 0.267 |
| PWY.6992 | 1,5-anhydrofructose degradation | 0 | Not detected | 0.001 | -0.070 | 0.072 | 0.975 |
| PWY.7003 | glycerol degradation to butanol | 0 | Not detected | -0.009 | -0.077 | 0.058 | 0.790 |
| PWY.7007 | methyl ketone biosynthesis (engineered) | 0 | Not detected | 0.045 | -0.188 | 0.278 | 0.707 |
| PWY.7013 | (<i>S</i>)-propane-1,2-diol degradation | 0 | Not detected | 0.180 | -0.048 | 0.408 | 0.123 |
| PWY.702 | L-methionine biosynthesis II (plants) | 0 | Not detected | 0.031 | -0.204 | 0.265 | 0.797 |
| PWY.7031 | protein <i>N</i>-glycosylation (bacterial) | 0 | Not detected | -0.257 | -0.492 | -0.023 | 0.032 |
| PWY.7037 | protein <i>O</i>-glycosylation (Neisseria) | 0 | Not detected | -0.247 | -0.482 | -0.012 | 0.039 |
| PWY.7077 | <i>N</i>-acetyl-D-galactosamine degradation | 0 | Not detected | 0.203 | -0.017 | 0.422 | 0.070 |
| PWY.7085 | triethylamine degradation | 0 | Not detected | 0.127 | -0.112 | 0.367 | 0.297 |
| PWY.7090 | UDP-2,3-diacetamido-2,3-dideoxy-&alpha;-D-m | 0 | Not detected | -0.140 | -0.307 | 0.027 | 0.100 |
| PWY.7096 | triclosan resistance | 0 | Not detected | -0.138 | -0.389 | 0.112 | 0.278 |
| PWY.7102 | bisabolene biosynthesis (engineered) | 0 | Not detected | 0.073 | -0.166 | 0.312 | 0.550 |
| PWY.7104 | dTDP-L-megosamine biosynthesis | 0 | Not detected | -0.006 | -0.078 | 0.066 | 0.870 |
| PWY.7111 | pyruvate fermentation to isobutanol (engineered) | 0 | Not detected | 0.021 | -0.063 | 0.106 | 0.617 |
| PWY.7115 | C4 photosynthetic carbon assimilation cycle, NA | 0 | Not detected | -0.059 | -0.313 | 0.195 | 0.647 |
| PWY.7117 | C4 photosynthetic carbon assimilation cycle, PE | 0 | Not detected | -0.008 | -0.083 | 0.066 | 0.826 |
| PWY.7118 | chitin degradation to ethanol | 0 | Not detected | 0.041 | -0.209 | 0.291 | 0.747 |
| PWY.7124 | ethylene biosynthesis V (engineered) | 0 | Not detected | -0.002 | -0.075 | 0.072 | 0.968 |
| PWY.7127 | CDP-D-mannitol biosynthesis | 0 | Not detected | 0.056 | -0.192 | 0.304 | 0.658 |
| PWY.7130 | L-glucose degradation | 0 | Not detected | 0.078 | -0.125 | 0.281 | 0.452 |

| PWY.7131 | CMP-legionaminate biosynthesis II | 0 | Not detected | -0.162 | -0.382 | 0.059 | 0.151 |
| --- | --- | --- | --- | --- | --- | --- | --- |
| PWY.7141 | (3<i>S</i>)-linalool biosynthesis | 0 | Not detected | 0.046 | -0.157 | 0.248 | 0.660 |
| PWY.7153 | grixazone biosynthesis | 0 | Not detected | -0.018 | -0.238 | 0.203 | 0.875 |
| PWY.7159 | 3,8-divinyl-chlorophyllide <i>a</i> biosynthesis | 0 | Not detected | 0.024 | -0.200 | 0.248 | 0.833 |
| PWY.7165 | L-ascorbate biosynthesis VI (engineered pathwa | 0 | Not detected | 0.044 | -0.194 | 0.283 | 0.715 |
| PWY.7174 | <i>S</i>-methyl-5-thio-&alpha;-D-ribose 1-pho | 0 | Not detected | -0.126 | -0.370 | 0.118 | 0.313 |
| PWY.7176 | UTP and CTP <i>de novo</i> biosynthesis | 0 | Not detected | 0.007 | -0.068 | 0.082 | 0.850 |
| PWY.7177 | UTP and CTP dephosphorylation II | 0 | Not detected | 0.011 | -0.066 | 0.088 | 0.780 |
| PWY.7180 | 2'-deoxy-&alpha;-D-ribose 1-phosphate degrada | 0 | Not detected | 0.067 | -0.154 | 0.287 | 0.555 |
| PWY.7182 | linalool biosynthesis I | 0 | Not detected | 0.046 | -0.157 | 0.248 | 0.660 |
| PWY.7184 | pyrimidine deoxyribonucleotides <i>de novo</i> | 0 | Not detected | -0.046 | -0.132 | 0.039 | 0.288 |
| PWY.7185 | UTP and CTP dephosphorylation I | 0 | Not detected | 0.011 | -0.066 | 0.088 | 0.780 |
| PWY.7187 | pyrimidine deoxyribonucleotides <i>de novo</i> | 0 | Not detected | -0.043 | -0.126 | 0.040 | 0.310 |
| PWY.7193 | pyrimidine ribonucleosides salvage I | 0 | Not detected | 0.033 | -0.215 | 0.280 | 0.794 |
| PWY.7195 | pyrimidine ribonucleosides salvage III | 0 | Not detected | -0.082 | -0.255 | 0.091 | 0.354 |
| PWY.7197 | pyrimidine deoxyribonucleotide phosphorylation | 0 | Not detected | -0.047 | -0.131 | 0.037 | 0.276 |
| PWY.7198 | pyrimidine deoxyribonucleotides <i>de novo</i> | 0 | Not detected | -0.048 | -0.135 | 0.039 | 0.282 |
| PWY.7204 | pyridoxal 5'-phosphate salvage II (plants) | 0 | Not detected | -0.012 | -0.226 | 0.203 | 0.915 |
| PWY.7205 | CMP phosphorylation | 0 | Not detected | -0.042 | -0.122 | 0.039 | 0.309 |
| PWY.7210 | pyrimidine deoxyribonucleotides biosynthesis fr | 0 | Not detected | -0.048 | -0.135 | 0.039 | 0.282 |
| PWY.7216 | (R)- and (S)-3-hydroxybutanoate biosynthesis (e | 0 | Not detected | 0.000 | -0.134 | 0.135 | 0.996 |
| PWY.7218 | photosynthetic 3-hydroxybutanoate biosynthesis | 0 | Not detected | -0.014 | -0.091 | 0.063 | 0.726 |
| PWY.7219 | adenosine ribonucleotides <i>de novo</i> biosy | 0 | Not detected | -0.044 | -0.130 | 0.042 | 0.319 |
| PWY.7221 | guanosine ribonucleotides <i>de novo</i> biosy | 0 | Not detected | -0.044 | -0.130 | 0.042 | 0.314 |
| PWY.7222 | guanosine deoxyribonucleotides <i>de novo</i> | 0 | Not detected | -0.044 | -0.130 | 0.042 | 0.314 |
| PWY.7224 | purine deoxyribonucleosides salvage | 0 | Not detected | -0.044 | -0.130 | 0.042 | 0.314 |
| PWY.7226 | guanosine deoxyribonucleotides <i>de novo</i> | 0 | Not detected | -0.044 | -0.130 | 0.042 | 0.314 |
| PWY.7234 | inosine-5'-phosphate biosynthesis III | 0 | Not detected | 0.062 | -0.064 | 0.188 | 0.334 |
| PWY.7237 | <i>myo</i>-, <i>chiro</i>- and <i>scyllo</i>-in | 0 | Not detected | -0.105 | -0.314 | 0.105 | 0.328 |
| PWY.7238 | sucrose biosynthesis II | 0 | Not detected | 0.003 | -0.064 | 0.070 | 0.921 |
| PWY.7241 | <I>myo</I>-inositol degradation II | 0 | Not detected | -0.068 | -0.278 | 0.142 | 0.527 |
| PWY.7242 | D-fructuronate degradation | 0 | Not detected | -0.040 | -0.104 | 0.024 | 0.222 |
| PWY.7247 | &beta;-D-glucuronide and D-glucuronate degrad | 0 | Not detected | -0.018 | -0.092 | 0.057 | 0.644 |
| PWY.7248 | pectin degradation II | 0 | Not detected | -0.018 | -0.092 | 0.057 | 0.643 |
| PWY.7250 | [2Fe-2S] iron-sulfur cluster biosynthesis | 0 | Not detected | 0.176 | -0.037 | 0.388 | 0.106 |
| PWY.7254 | TCA cycle VII (acetate-producers) | 0 | Not detected | -0.009 | -0.086 | 0.068 | 0.809 |
| PWY.7255 | ergothioneine biosynthesis I (bacteria) | 0 | Not detected | -0.025 | -0.219 | 0.168 | 0.798 |
| PWY.7268 | NAD/NADP-NADH/NADPH cytosolic intercon | 0 | Not detected | -0.018 | -0.192 | 0.156 | 0.837 |
| PWY.7269 | NAD/NADP-NADH/NADPH mitochondrial inte | 0 | Not detected | 0.042 | -0.146 | 0.230 | 0.664 |
| PWY.7279 | aerobic respiration II (cytochrome c) (yeast) | 0 | Not detected | 0.041 | -0.146 | 0.229 | 0.666 |
| PWY.7282 | 4-amino-2-methyl-5-diphosphomethylpyrimidin | 0 | Not detected | -0.088 | -0.246 | 0.069 | 0.270 |
| PWY.7288 | fatty acid &beta;-oxidation (peroxisome, yeast) | 0 | Not detected | 0.078 | -0.152 | 0.307 | 0.507 |
| PWY.7290 | <i>Escherichia coli</i> serotype O86 <i>O</i>- | 0 | Not detected | -0.230 | -0.482 | 0.022 | 0.074 |
| PWY.7294 | D-xylose degradation IV | 0 | Not detected | 0.199 | -0.042 | 0.441 | 0.106 |

| PWY.7295 | L-arabinose degradation IV | 0 | Not detected | 0.060 | -0.169 | 0.289 | 0.608 |
| --- | --- | --- | --- | --- | --- | --- | --- |
| PWY.7301 | dTDP-4-<i>O</i>-demethyl-&beta;-L-noviose b | 0 | Not detected | -0.008 | -0.081 | 0.065 | 0.839 |
| PWY.7312 | dTDP-D-&beta;-fucofuranose biosynthesis | 0 | Not detected | -0.006 | -0.078 | 0.065 | 0.863 |
| PWY.7315 | dTDP-<i>N</i>-acetylthomosamine biosynthesi | 0 | Not detected | -0.005 | -0.074 | 0.064 | 0.884 |
| PWY.7316 | dTDP-<i>N</i>-acetylviosamine biosynthesis | 0 | Not detected | -0.008 | -0.080 | 0.065 | 0.837 |
| PWY.7318 | dTDP-3-acetamido-3,6-dideoxy-&alpha;-D-gluc | 0 | Not detected | -0.007 | -0.079 | 0.065 | 0.845 |
| PWY.7328 | superpathway of UDP-glucose-derived O-antige | 0 | Not detected | -0.049 | -0.134 | 0.036 | 0.256 |
| PWY.7330 | UDP-<i>N</i>-acetyl-&beta;-L-fucosamine bios | 0 | Not detected | 0.002 | -0.162 | 0.165 | 0.986 |
| PWY.7331 | UDP-<i>N</i>-acetyl-&beta;-L-quinovosamine | 0 | Not detected | -0.055 | -0.216 | 0.107 | 0.509 |
| PWY.7332 | superpathway of UDP-<I>N</i>-acetylglucosam | 0 | Not detected | -0.035 | -0.208 | 0.138 | 0.695 |
| PWY.7335 | UDP-<i>N</i>-acetyl-&alpha;-D-mannosamino | 0 | Not detected | 0.013 | -0.107 | 0.134 | 0.827 |
| PWY.7336 | UDP-<i>N</i>-acetyl-&alpha;-D-galactosaminu | 0 | Not detected | -0.155 | -0.330 | 0.020 | 0.082 |
| PWY.7342 | superpathway of nicotine biosynthesis | 0 | Not detected | -0.032 | -0.298 | 0.235 | 0.814 |
| PWY.7343 | UDP-&alpha;-D-glucose biosynthesis I | 0 | Not detected | 0.019 | -0.060 | 0.098 | 0.636 |
| PWY.7344 | UDP-&alpha;-D-galactose biosynthesis | 0 | Not detected | -0.051 | -0.188 | 0.086 | 0.466 |
| PWY.7346 | UDP-&alpha;-D-glucuronate biosynthesis (from | 0 | Not detected | -0.107 | -0.300 | 0.087 | 0.280 |
| PWY.7347 | sucrose biosynthesis III | 0 | Not detected | 0.005 | -0.074 | 0.084 | 0.907 |
| PWY.7377 | cob(II)yrinate <i>a,c</i>-diamide biosynthesis I | 0 | Not detected | -0.005 | -0.099 | 0.089 | 0.916 |
| PWY.7378 | aminopropanol phosphate biosynthesis II | 0 | Not detected | 0.037 | -0.193 | 0.267 | 0.751 |
| PWY.7383 | anaerobic energy metabolism (invertebrates, cyto | 0 | Not detected | 0.065 | -0.182 | 0.313 | 0.605 |
| PWY.7384 | anaerobic energy metabolism (invertebrates, mit | 0 | Not detected | -0.082 | -0.165 | 0.002 | 0.056 |
| PWY.7385 | 1,3-propanediol biosynthesis (engineered) | 0 | Not detected | 0.002 | -0.068 | 0.072 | 0.954 |
| PWY.7388 | octanoyl-[acyl-carrier protein] biosynthesis (mito | 0 | Not detected | 0.084 | -0.133 | 0.301 | 0.446 |
| PWY.7391 | isoprene biosynthesis II (engineered) | 0 | Not detected | 0.044 | -0.117 | 0.205 | 0.592 |
| PWY.7395 | D-galactosamine and <i>N</i>-acetyl-D-galacto | 0 | Not detected | 0.203 | -0.017 | 0.422 | 0.070 |
| PWY.7396 | butanol and isobutanol biosynthesis (engineered) | 0 | Not detected | 0.005 | -0.093 | 0.102 | 0.921 |
| PWY.7399 | methylphosphonate degradation II | 0 | Not detected | 0.154 | -0.069 | 0.378 | 0.177 |
| PWY.7400 | L-arginine biosynthesis IV (archaebacteria) | 0 | Not detected | -0.066 | -0.184 | 0.052 | 0.273 |
| PWY.7401 | crotonate fermentation (to acetate and cyclohexa | 0 | Not detected | 0.014 | -0.112 | 0.140 | 0.831 |
| PWY.7409 | phospholipid remodeling (phosphatidylethanola | 0 | Not detected | 0.001 | -0.161 | 0.163 | 0.991 |
| PWY.7410 | ipsdienol biosynthesis | 0 | Not detected | 0.046 | -0.157 | 0.248 | 0.660 |
| PWY.7413 | dTDP-6-deoxy-&alpha;-D-allose biosynthesis | 0 | Not detected | -0.006 | -0.078 | 0.066 | 0.870 |
| PWY.7414 | dTDP-&alpha;-D-mycaminose biosynthesis | 0 | Not detected | -0.007 | -0.079 | 0.065 | 0.845 |
| PWY.7419 | FR-900098 and FR-33289 antibiotics biosynthes | 0 | Not detected | -0.042 | -0.289 | 0.205 | 0.740 |
| PWY.7425 | 2-chloroacrylate degradation I | 0 | Not detected | 0.012 | -0.181 | 0.205 | 0.905 |
| PWY.7431 | aromatic biogenic amine degradation (bacteria) | 0 | Not detected | 0.089 | -0.092 | 0.271 | 0.335 |
| PWY.7440 | dTDP-&beta;-L-4-<i>epi</i>-vancosamine bios | 0 | Not detected | -0.006 | -0.078 | 0.066 | 0.870 |
| PWY.7442 | drosopterin and aurodrosopterin biosynthesis | 0 | Not detected | 0.057 | -0.072 | 0.186 | 0.386 |
| PWY.7446 | sulfoquinovose degradation I | 0 | Not detected | 0.015 | -0.233 | 0.264 | 0.903 |
| PWY.7456 | &beta;-(1,4)-mannan degradation | 0 | Not detected | -0.127 | -0.244 | -0.010 | 0.033 |
| PWY.7459 | kojibiose degradation | 0 | Not detected | 0.220 | 0.018 | 0.422 | 0.033 |
| PWY.7467 | 2-acetamido-4-amino-2,4,6-trideoxy-&alpha;-D- | 0 | Not detected | -0.096 | -0.280 | 0.088 | 0.309 |
| PWY.7510 | rhizocticin A and B biosynthesis | 0 | Not detected | -0.042 | -0.289 | 0.205 | 0.740 |
| PWY.7524 | mevalonate pathway III (archaea) | 0 | Not detected | 0.044 | -0.117 | 0.205 | 0.592 |

| PWY.7530 | &beta;-D-galactosaminyl-(1&rarr;3)-<i>N</i>-a | 0 | Not detected | -0.228 | -0.480 | 0.023 | 0.075 |
| --- | --- | --- | --- | --- | --- | --- | --- |
| PWY.7539 | 6-hydroxymethyl-dihydropterin diphosphate bio | 0 | Not detected | -0.017 | -0.096 | 0.061 | 0.663 |
| PWY.7541 | 1,2-propanediol biosynthesis from lactate (engin | 0 | Not detected | -0.108 | -0.358 | 0.142 | 0.399 |
| PWY.7552 | heme <i>b</i> biosynthesis III (from siroheme) | 0 | Not detected | 0.111 | -0.122 | 0.344 | 0.352 |
| PWY.7559 | glutathione degradation (DUG pathway - yeast) | 0 | Not detected | -0.060 | -0.287 | 0.167 | 0.605 |
| PWY.7560 | methylerythritol phosphate pathway II | 0 | Not detected | 0.017 | -0.080 | 0.114 | 0.732 |
| PWY.7562 | 3,6-anhydro-&alpha;-L-galactopyranose degrada | 0 | Not detected | -0.018 | -0.089 | 0.053 | 0.620 |
| PWY.7564 | bacimethrin and bacimethrin pyrophosphate bios | 0 | Not detected | -0.162 | -0.303 | -0.021 | 0.024 |
| PWY.7568 | L-galactose degradation | 0 | Not detected | -0.134 | -0.319 | 0.050 | 0.153 |
| PWY.7573 | GDP-mycosamine biosynthesis | 0 | Not detected | -0.049 | -0.294 | 0.195 | 0.693 |
| PWY.7581 | <i>N</i>-acetylneuraminate and <i>N</i>-acety | 0 | Not detected | -0.189 | -0.348 | -0.030 | 0.020 |
| PWY.7586 | &beta;-1,4-D-mannosyl-<i>N</i>-acetyl-D-gluc | 0 | Not detected | -0.023 | -0.225 | 0.179 | 0.823 |
| PWY.7588 | ursodeoxycholate biosynthesis (bacteria) | 0 | Not detected | -0.003 | -0.207 | 0.201 | 0.977 |
| PWY.7610 | GDP-6-deoxy-D-<i>altro</i>-heptose biosynthe | 0 | Not detected | -0.150 | -0.315 | 0.015 | 0.075 |
| PWY.7616 | methanol oxidation to carbon dioxide | 0 | Not detected | -0.025 | -0.249 | 0.198 | 0.824 |
| PWY.7622 | UDP-&alpha;-D-galactofuranose biosynthesis | 0 | Not detected | -0.037 | -0.123 | 0.048 | 0.391 |
| PWY.7644 | heparin degradation | 317 | Not detected | -0.260 | -0.481 | -0.039 | 0.021 |
| PWY.7645 | hyaluronan degradation | 0 | Not detected | -0.073 | -0.276 | 0.130 | 0.482 |
| PWY.7646 | dermatan sulfate degradation I (bacterial) | 0 | Not detected | -0.108 | -0.310 | 0.094 | 0.294 |
| PWY.7651 | heparan sulfate degradation | 0 | Not detected | -0.206 | -0.493 | 0.080 | 0.158 |
| PWY.7657 | dTDP-&beta;-L-digitoxose biosynthesis | 0 | Not detected | -0.006 | -0.078 | 0.066 | 0.870 |
| PWY.7659 | viridicatumtoxin biosynthesis | 0 | Not detected | 0.046 | -0.157 | 0.248 | 0.660 |
| PWY.7663 | gondoate biosynthesis (anaerobic) | 0 | Not detected | 0.003 | -0.087 | 0.092 | 0.950 |
| PWY.7664 | oleate biosynthesis IV (anaerobic) | 0 | Not detected | 0.007 | -0.084 | 0.097 | 0.882 |
| PWY.7674 | CMP-8-amino-3,8-dideoxy-D-<I>manno</I>-oc | 0 | Not detected | -0.035 | -0.249 | 0.180 | 0.751 |
| PWY.7675 | Kdo transfer to lipid IV<sub>A</sub> II | 0 | Not detected | 0.080 | -0.125 | 0.284 | 0.446 |
| PWY.7684 | &alpha;-diglucosyldiacylglycerol biosynthesis | 0 | Not detected | -0.040 | -0.248 | 0.168 | 0.707 |
| PWY.7688 | dTDP-D-ravidosamine and dTDP-4-acetyl-D-rav | 0 | Not detected | -0.010 | -0.083 | 0.062 | 0.778 |
| PWY.7709 | (3<i>R</i>)-linalool biosynthesis | 0 | Not detected | 0.046 | -0.157 | 0.248 | 0.660 |
| PWY.7716 | penicillin G and penicillin V biosynthesis | 0 | Not detected | -0.084 | -0.201 | 0.032 | 0.155 |
| PWY.7719 | CMP-diacetamido-8-epilegionaminic acid biosy | 0 | Not detected | -0.086 | -0.238 | 0.066 | 0.269 |
| PWY.7721 | methyl phomopsenoate biosynthesis | 0 | Not detected | 0.042 | -0.165 | 0.250 | 0.688 |
| PWY.7736 | stellatic acid biosynthesis | 0 | Not detected | 0.047 | -0.189 | 0.282 | 0.697 |
| PWY.7754 | bile acids degradation | 0 | Not detected | 0.262 | -0.031 | 0.555 | 0.079 |
| PWY.7755 | iso-bile acids biosynthesis I | 0 | Not detected | 0.101 | -0.172 | 0.373 | 0.468 |
| PWY.7756 | iso-bile acids biosynthesis II | 0 | Not detected | 0.073 | -0.096 | 0.242 | 0.395 |
| PWY.7766 | heme <i>b</i> biosynthesis IV (Gram-positive b | 0 | Not detected | 0.025 | -0.199 | 0.249 | 0.827 |
| PWY.7769 | phosalacine biosynthesis | 0 | Not detected | -0.017 | -0.264 | 0.229 | 0.890 |
| PWY.7778 | 2-methylpropene degradation | 0 | Not detected | 0.000 | -0.134 | 0.135 | 0.995 |
| PWY.7779 | methyl <i>tert</i>-butyl ether degradation | 0 | Not detected | 0.000 | -0.134 | 0.135 | 0.995 |
| PWY.7790 | UMP biosynthesis II | 0 | Not detected | 0.016 | -0.067 | 0.100 | 0.700 |
| PWY.7791 | UMP biosynthesis III | 0 | Not detected | 0.016 | -0.067 | 0.100 | 0.700 |
| PWY.7793 | dimethyl sulfide biosynthesis from methionine | 0 | Not detected | 0.004 | -0.207 | 0.216 | 0.967 |
| PWY.7805 | (aminomethyl)phosphonate degradation | 0 | Not detected | 0.050 | -0.172 | 0.272 | 0.659 |

| PWY.7807 | glyphosate degradation III | 0 | Not detected | -0.159 | -0.373 | 0.056 | 0.147 |
| --- | --- | --- | --- | --- | --- | --- | --- |
| PWY.7808 | tetracycline resistance | 0 | Not detected | -0.048 | -0.387 | 0.292 | 0.783 |
| PWY.7814 | dTDP-L-daunosamine biosynthesis | 0 | Not detected | -0.008 | -0.081 | 0.065 | 0.839 |
| PWY.7815 | poly(ribitol phosphate) wall teichoic acid biosyn | 0 | Not detected | 0.067 | -0.037 | 0.172 | 0.207 |
| PWY.7816 | poly(ribitol phosphate) wall teichoic acid biosyn | 0 | Not detected | 0.070 | -0.036 | 0.175 | 0.195 |
| PWY.7817 | type I lipoteichoic acid biosynthesis (<i>S. aureu | 0 | Not detected | 0.026 | -0.073 | 0.124 | 0.608 |
| PWY.7818 | type IV lipoteichoic acid biosynthesis (<i>S. pne | 0 | Not detected | 0.233 | 0.057 | 0.408 | 0.010 |
| PWY.7819 | poly(3-O-&beta;-D-glucopyranosyl-<i>N</i>-ac | 0 | Not detected | 0.013 | -0.107 | 0.133 | 0.828 |
| PWY.7820 | teichuronic acid biosynthesis (<i>B. subtilis</i> | 0 | Not detected | -0.149 | -0.338 | 0.040 | 0.123 |
| PWY.7822 | chitin degradation III (Serratia) | 0 | Not detected | 0.158 | -0.047 | 0.363 | 0.132 |
| PWY.7852 | 6-hydroxymethyl-dihydropterin diphosphate bio | 0 | Not detected | 0.051 | -0.063 | 0.165 | 0.377 |
| PWY.7854 | crotonyl-CoA/ethylmalonyl-CoA/hydroxybutyry | 0 | Not detected | 0.061 | -0.169 | 0.290 | 0.604 |
| PWY.7856 | heme <i>a</i> biosynthesis | 0 | Not detected | 0.048 | -0.124 | 0.220 | 0.585 |
| PWY.7858 | (5Z)-dodecenoate biosynthesis II | 0 | Not detected | -0.001 | -0.089 | 0.087 | 0.982 |
| PWY.7863 | roseoflavin biosynthesis | 0 | Not detected | -0.097 | -0.276 | 0.082 | 0.288 |
| PWY.7864 | 8-methylmenaquinone biosynthesis | 0 | Not detected | -0.107 | -0.363 | 0.150 | 0.414 |
| PWY.7873 | D-erythronate degradation II | 0 | Not detected | 0.093 | -0.093 | 0.280 | 0.328 |
| PWY.7874 | L-threonate degradation | 0 | Not detected | 0.047 | -0.162 | 0.256 | 0.662 |
| PWY.7883 | anhydromuropeptides recycling II | 0 | Not detected | -0.131 | -0.351 | 0.088 | 0.241 |
| PWY.7884 | lipoprotein posttranslational modification | 0 | Not detected | 0.005 | -0.245 | 0.254 | 0.970 |
| PWY.7892 | tRNA-uridine 2-thiolation (bacteria) | 0 | Not detected | 0.041 | -0.117 | 0.199 | 0.611 |
| PWY.7900 | glycogen biosynthesis III (from &alpha;-maltose | 0 | Not detected | 0.041 | -0.052 | 0.133 | 0.389 |
| PWY.7902 | glucosylglycerol biosynthesis | 0 | Not detected | 0.037 | -0.065 | 0.140 | 0.474 |
| PWY.7904 | tRNA-uridine 2-thiolation (thermophilic bacteria | 0 | Not detected | -0.136 | -0.341 | 0.070 | 0.195 |
| PWY.7909 | formaldehyde oxidation VII (THF pathway) | 0 | Not detected | -0.111 | -0.223 | 0.001 | 0.052 |
| PWY.7928 | nickel cofactor biosynthesis | 0 | Not detected | 0.021 | -0.133 | 0.176 | 0.787 |
| PWY.7953 | UDP-<i>N</i>-acetylmuramoyl-pentapeptide bi | 0 | Not detected | 0.050 | -0.014 | 0.113 | 0.125 |
| PWY.7975 | adenosylcobalamin biosynthesis from adenosylc | 0 | Not detected | -0.020 | -0.194 | 0.153 | 0.819 |
| PWY.7977 | L-methionine biosynthesis IV (archaea) | 0 | Not detected | -0.019 | -0.095 | 0.058 | 0.632 |
| PWY.7978 | <i>N</i>-methylpyrrolidone degradation | 0 | Not detected | 0.085 | -0.091 | 0.262 | 0.343 |
| PWY.7981 | &alpha;-dystroglycan glycosylation | 0 | Not detected | 0.086 | -0.159 | 0.331 | 0.492 |
| PWY.7991 | toxoflavin biosynthesis | 0 | Not detected | -0.016 | -0.090 | 0.057 | 0.660 |
| PWY.8004 | Entner-Doudoroff pathway I | 0 | Not detected | -0.016 | -0.084 | 0.053 | 0.656 |
| PWY.801 | homocysteine and cysteine interconversion | 0 | Not detected | 0.127 | -0.109 | 0.363 | 0.290 |
| PWY.8010 | L-cysteine biosynthesis IX (Trichomonas vagina | 0 | Not detected | 0.057 | -0.040 | 0.154 | 0.247 |
| PWY.8012 | mupirocin biosynthesis | 0 | Not detected | 0.091 | -0.126 | 0.308 | 0.411 |
| PWY.8013 | UDP-<i>N</i>-acetyl-D-galactosamine biosynth | 0 | Not detected | 0.005 | -0.074 | 0.084 | 0.907 |
| PWY.8015 | glycine degradation (Stickland reaction) | 0 | Not detected | -0.002 | -0.074 | 0.069 | 0.953 |
| PWY.8040 | ansatrienin biosynthesis | 0 | Not detected | 0.061 | -0.178 | 0.300 | 0.619 |
| PWY.882 | L-ascorbate biosynthesis I (L-galactose pathway) | 0 | Not detected | -0.012 | -0.214 | 0.189 | 0.906 |
| PWY.922 | mevalonate pathway I | 0 | Not detected | 0.044 | -0.117 | 0.205 | 0.592 |
| PWY.I9 | L-cysteine biosynthesis VI (from L-methionine) | 0 | Not detected | 0.094 | -0.145 | 0.333 | 0.441 |
| PWY0.1182 | trehalose degradation II (cytosolic) | 0 | Not detected | 0.096 | -0.148 | 0.340 | 0.439 |
| PWY0.1221 | putrescine degradation II | 0 | Not detected | 0.164 | -0.040 | 0.368 | 0.116 |

| PWY0.1241 | ADP-L-<i>glycero</i>-&beta;-D-<i>manno</i> | 0 | Not detected | 0.027 | -0.180 | 0.233 | 0.800 |
| --- | --- | --- | --- | --- | --- | --- | --- |
| PWY0.1261 | anhydromuropeptides recycling I | 0 | Not detected | 0.032 | -0.027 | 0.091 | 0.282 |
| PWY0.1264 | biotin-carboxyl carrier protein assembly | 0 | Not detected | -0.045 | -0.134 | 0.045 | 0.331 |
| PWY0.1275 | lipoate biosynthesis and incorporation II | 0 | Not detected | -0.070 | -0.243 | 0.103 | 0.427 |
| PWY0.1296 | purine ribonucleosides degradation | 0 | Not detected | -0.055 | -0.248 | 0.137 | 0.573 |
| PWY0.1300 | 2-<I>O</I>-&alpha;-mannosyl-D-glycerate degr | 0 | Not detected | -0.029 | -0.266 | 0.207 | 0.808 |
| PWY0.1306 | L-galactonate degradation | 0 | Not detected | -0.111 | -0.265 | 0.043 | 0.159 |
| PWY0.1312 | acetate and ATP formation from acetyl-CoA I | 0 | Not detected | 0.004 | -0.062 | 0.069 | 0.913 |
| PWY0.1313 | acetate conversion to acetyl-CoA | 0 | Not detected | 0.035 | -0.188 | 0.258 | 0.761 |
| PWY0.1317 | L-lactaldehyde degradation (aerobic) | 0 | Not detected | 0.064 | -0.140 | 0.268 | 0.538 |
| PWY0.1319 | CDP-diacylglycerol biosynthesis II | 0 | Not detected | 0.082 | -0.093 | 0.258 | 0.357 |
| PWY0.1324 | <i>N</i>-acetylneuraminate and <i>N</i>-acety | 0 | Not detected | -0.190 | -0.330 | -0.050 | 0.008 |
| PWY0.1337 | oleate &beta;-oxidation | 0 | Not detected | 0.012 | -0.196 | 0.221 | 0.907 |
| PWY0.1338 | polymyxin resistance | 0 | Not detected | 0.033 | -0.192 | 0.258 | 0.773 |
| PWY0.1415 | superpathway of heme <i>b</i> biosynthesis fro | 0 | Not detected | 0.069 | -0.156 | 0.294 | 0.550 |
| PWY0.1433 | tetrahydromonapterin biosynthesis | 0 | Not detected | 0.041 | -0.072 | 0.155 | 0.474 |
| PWY0.1465 | D-malate degradation | 0 | Not detected | -0.263 | -0.475 | -0.052 | 0.015 |
| PWY0.1466 | trehalose degradation VI (periplasmic) | 0 | Not detected | 0.132 | -0.093 | 0.357 | 0.251 |
| PWY0.1471 | uracil degradation III | 0 | Not detected | 0.000 | -0.199 | 0.198 | 0.998 |
| PWY0.1477 | ethanolamine utilization | 0 | Not detected | 0.000 | -0.060 | 0.061 | 0.991 |
| PWY0.1479 | tRNA processing | 0 | Not detected | 0.010 | -0.226 | 0.245 | 0.936 |
| PWY0.1533 | methylphosphonate degradation I | 0 | Not detected | 0.163 | -0.056 | 0.382 | 0.145 |
| PWY0.1545 | cardiolipin biosynthesis III | 0 | Not detected | 0.053 | -0.170 | 0.276 | 0.641 |
| PWY0.1546 | muropeptide degradation | 0 | Not detected | -0.002 | -0.194 | 0.190 | 0.984 |
| PWY0.1565 | D-lactate to cytochrome <i>bo</i> oxidase elect | 0 | Not detected | 0.012 | -0.181 | 0.205 | 0.905 |
| PWY0.1567 | NADH to cytochrome <i>bo</i> oxidase electro | 0 | Not detected | 0.041 | -0.146 | 0.229 | 0.666 |
| PWY0.1568 | NADH to cytochrome <i>bd</i> oxidase electro | 0 | Not detected | 0.041 | -0.146 | 0.229 | 0.666 |
| PWY0.1569 | autoinducer AI-2 degradation | 0 | Not detected | 0.209 | 0.013 | 0.404 | 0.036 |
| PWY0.1573 | nitrate reduction VIIIb (dissimilatory) | 0 | Not detected | 0.041 | -0.146 | 0.229 | 0.666 |
| PWY0.1585 | formate to nitrite electron transfer | 0 | Not detected | 0.007 | -0.200 | 0.213 | 0.950 |
| PWY0.1586 | peptidoglycan maturation (<i>meso</i>-diamino | 0 | Not detected | 0.105 | -0.173 | 0.383 | 0.459 |
| PWY0.1590 | NADH to hydrogen peroxide electron transfer | 0 | Not detected | -0.111 | -0.323 | 0.101 | 0.304 |
| PWY0.1591 | glycerol-3-phosphate to hydrogen peroxide elect | 0 | Not detected | -0.111 | -0.323 | 0.101 | 0.304 |
| PWY0.1597 | prenylated FMNH<sub>2</sub> biosynthesis | 0 | Not detected | -0.039 | -0.204 | 0.127 | 0.646 |
| PWY0.166 | superpathway of pyrimidine deoxyribonucleotide | 0 | Not detected | -0.047 | -0.132 | 0.038 | 0.280 |
| PWY0.301 | L-ascorbate degradation I (bacterial, anaerobic) | 0 | Not detected | 0.008 | -0.059 | 0.074 | 0.825 |
| PWY0.321 | phenylacetate degradation I (aerobic) | 0 | Not detected | -0.094 | -0.195 | 0.008 | 0.072 |
| PWY0.42 | 2-methylcitrate cycle I | 0 | Not detected | -0.002 | -0.260 | 0.257 | 0.990 |
| PWY0.43 | conversion of succinate to propanoate | 0 | Not detected | -0.125 | -0.265 | 0.015 | 0.080 |
| PWY0.44 | D-allose degradation | 0 | Not detected | -0.040 | -0.288 | 0.208 | 0.751 |
| PWY0.461 | L-lysine degradation I | 0 | Not detected | -0.020 | -0.233 | 0.193 | 0.856 |
| PWY0.501 | lipoate biosynthesis and incorporation I | 0 | Not detected | -0.106 | -0.280 | 0.067 | 0.229 |
| PWY0.522 | lipoate salvage I | 0 | Not detected | 0.049 | -0.144 | 0.241 | 0.620 |
| PWY0.662 | PRPP biosynthesis | 0 | Not detected | -0.027 | -0.123 | 0.069 | 0.581 |

| PWY0.862 | (5Z)-dodecenoate biosynthesis I | 0 | Not detected | -0.004 | -0.092 | 0.084 | 0.930 |
| --- | --- | --- | --- | --- | --- | --- | --- |
| PWY1.3 | polyhydroxybutanoate biosynthesis | 0 | Not detected | 0.050 | -0.126 | 0.226 | 0.579 |
| PWY3O.4107 | NAD salvage pathway V (PNC V cycle) | 0 | Not detected | -0.042 | -0.247 | 0.162 | 0.684 |
| PWY4FS.7 | phosphatidylglycerol biosynthesis I (plastidic) | 0 | Not detected | 0.023 | -0.184 | 0.230 | 0.825 |
| PWY4FS.8 | phosphatidylglycerol biosynthesis II (non-plastid | 0 | Not detected | 0.023 | -0.184 | 0.230 | 0.825 |
| PWY4LZ.257 | superpathway of fermentation (<i>Chlamydomo | 0 | Not detected | 0.000 | -0.060 | 0.061 | 0.993 |
| PWY66.161 | ethanol degradation III | 0 | Not detected | 0.056 | -0.167 | 0.279 | 0.624 |
| PWY66.162 | ethanol degradation IV | 0 | Not detected | 0.056 | -0.167 | 0.279 | 0.624 |
| PWY66.21 | ethanol degradation II | 0 | Not detected | 0.055 | -0.181 | 0.292 | 0.646 |
| PWY66.366 | flavin biosynthesis IV (mammalian) | 0 | Not detected | -0.114 | -0.322 | 0.094 | 0.283 |
| PWY66.391 | fatty acid &beta;-oxidation VI (peroxisome) | 0 | Not detected | 0.126 | -0.136 | 0.387 | 0.346 |
| PWY66.398 | TCA cycle III (animals) | 0 | Not detected | -0.001 | -0.084 | 0.083 | 0.989 |
| PWY66.399 | gluconeogenesis III | 0 | Not detected | -0.002 | -0.074 | 0.071 | 0.966 |
| PWY66.422 | D-galactose degradation V (Leloir pathway) | 0 | Not detected | -0.020 | -0.090 | 0.050 | 0.576 |
| PWY66.426 | hydrogen sulfide biosynthesis II (mammalian) | 0 | Not detected | 0.082 | -0.120 | 0.284 | 0.427 |
| PYRIDNUCSAL.P | NAD salvage pathway I (PNC VI cycle) | 0 | Not detected | -0.050 | -0.257 | 0.157 | 0.636 |
| PYRIDNUCSYN.P | NAD <i>de novo</i> biosynthesis I (from aspart | 0 | Not detected | 0.017 | -0.251 | 0.286 | 0.899 |
| PYRIDOXSYN.PW | pyridoxal 5'-phosphate biosynthesis I | 0 | Not detected | 0.001 | -0.266 | 0.269 | 0.992 |
| PYRUVDEH.RXN | NA | 0 | Not detected | -0.012 | -0.234 | 0.209 | 0.913 |
| PYRUVDEHYD.P | pyruvate decarboxylation to acetyl CoA | 0 | Not detected | -0.012 | -0.234 | 0.209 | 0.913 |
| QUINATEDEG.PW | quinate degradation I | 0 | Not detected | -0.014 | -0.211 | 0.184 | 0.893 |
| REDCITCYC | TCA cycle VIII (Helicobacter) | 0 | Not detected | -0.003 | -0.086 | 0.079 | 0.941 |
| RHAMCAT.PWY | L-rhamnose degradation I | 0 | Not detected | 0.108 | -0.092 | 0.309 | 0.290 |
| RIBOSYN2.PWY | flavin biosynthesis I (bacteria and plants) | 0 | Not detected | -0.018 | -0.090 | 0.055 | 0.635 |
| RUMP.PWY | formaldehyde oxidation I | 0 | Not detected | 0.001 | -0.069 | 0.071 | 0.976 |
| RXN.12588 | NA | 0 | Not detected | 0.351 | 0.116 | 0.586 | 0.003 |
| RXN.13163 | NA | 0 | Not detected | -0.006 | -0.272 | 0.260 | 0.965 |
| RXN.14047 | NA | 0 | Not detected | -0.001 | -0.089 | 0.088 | 0.987 |
| RXN.14201 | NA | 0 | Not detected | -0.006 | -0.062 | 0.051 | 0.840 |
| RXN.14249 | NA | 0 | Not detected | 0.037 | -0.193 | 0.267 | 0.751 |
| RXN.14314 | NA | 0 | Not detected | -0.023 | -0.250 | 0.203 | 0.840 |
| RXN.17127 | NA | 0 | Not detected | 0.049 | -0.144 | 0.241 | 0.620 |
| RXN.20025 | NA | 0 | Not detected | 0.039 | -0.141 | 0.219 | 0.668 |
| RXN0.1141 | NA | 0 | Not detected | 0.045 | -0.121 | 0.211 | 0.597 |
| RXN0.6511 | NA | 0 | Not detected | -0.077 | -0.290 | 0.137 | 0.482 |
| SERSYN.PWY | L-serine biosynthesis I | 0 | Not detected | 0.051 | -0.033 | 0.135 | 0.235 |
| SUCROSEUTIL2.P | sucrose degradation VII (sucrose 3-dehydrogena | 0 | Not detected | 0.113 | -0.109 | 0.335 | 0.318 |
| SUCSYN.PWY | sucrose biosynthesis I (from photosynthesis) | 0 | Not detected | 0.001 | -0.071 | 0.074 | 0.971 |
| SUCUTIL.PWY | sucrose degradation I (sucrose phosphotransferas | 0 | Not detected | 0.218 | 0.001 | 0.435 | 0.049 |
| SULFMETII.PWY | assimilatory sulfate reduction II | 0 | Not detected | 0.066 | -0.195 | 0.328 | 0.619 |
| TCA | TCA cycle I (prokaryotic) | 0 | Not detected | -0.004 | -0.082 | 0.074 | 0.919 |
| TEICHOICACID.P | poly(glycerol phosphate) wall teichoic acid biosy | 0 | Not detected | 0.042 | -0.067 | 0.151 | 0.452 |
| THRDLCTCAT.PW | L-threonine degradation III (to methylglyoxal) | 0 | Not detected | 0.037 | -0.193 | 0.267 | 0.751 |
| THREONINE.DEG | L-threonine degradation II | 0 | Not detected | 0.037 | -0.193 | 0.267 | 0.751 |

| TREDEGLOW.PW | trehalose degradation I (low osmolarity) | 0 | Not detected | -0.052 | -0.144 | 0.040 | 0.268 |
| --- | --- | --- | --- | --- | --- | --- | --- |
| TRESYN.PWY | trehalose biosynthesis I | 0 | Not detected | 0.087 | -0.108 | 0.282 | 0.382 |
| TRIGLSYN.PWY | diacylglycerol and triacylglycerol biosynthesis | 0 | Not detected | -0.050 | -0.293 | 0.194 | 0.691 |
| TRNA.CHARGING | tRNA charging | 0 | Not detected | -0.002 | -0.056 | 0.051 | 0.932 |
| TRPSYN.PWY | L-tryptophan biosynthesis | 0 | Not detected | 0.067 | -0.183 | 0.316 | 0.599 |
| TRYPSYN.RXN | NA | 0 | Not detected | 0.070 | -0.151 | 0.290 | 0.537 |
| UDPNACETYLGA | UDP-<i>N</i>-acetyl-D-glucosamine biosynthe | 0 | Not detected | 0.002 | -0.073 | 0.076 | 0.964 |
| UDPNAGSYN.PW | UDP-<i>N</i>-acetyl-D-glucosamine biosynthe | 0 | Not detected | -0.001 | -0.072 | 0.071 | 0.989 |
| VALSYN.PWY | L-valine biosynthesis | 0 | Not detected | 0.021 | -0.063 | 0.106 | 0.617 |
| X1.1.1.39.RXN | NA | 0 | Not detected | -0.059 | -0.313 | 0.195 | 0.647 |
| X1CMET2.PWY | <i>N</i><sup>10</sup>-formyl-tetrahydrofolat | 0 | Not detected | -0.029 | -0.101 | 0.043 | 0.426 |
| X3.HYDROXYPH | 4-hydroxyphenylacetate degradation | 0 | Not detected | 0.011 | -0.199 | 0.222 | 0.916 |

| **Supplementary Table 5.** Association of parental education (High school or more vs. less than High school) with enzymatic reactions in overall participants (n=2174). Analysis of Compositions of Microbiomes (ANCOM2) was used to identify enzymatic reactions related to parental education. Regression coefficients were obtained by modeling the effect of parental education on centered log ratio (CLR)-transformed species in multivariable linear regression adjusted for age, gender, BMI, field center, Hispanic/Latino background, U.S. nativity, Bristol stool type, current SEP, the alternative Healthy Eating Index 2010 score, cigarette smoking, alcohol use, depressive and anxiety symptoms, diabetes, hypertension, cardiovascular disease, dyslipidemia, and antibiotic use. | | | | | | | |
| --- | --- | --- | --- | --- | --- | --- | --- |
| **otu.names** | **V2** | **W_stat** | **ANCOM** | **beta** | **l95ci** | **u95ci** | **qval** |
| ENZRXN.23 | GDP-6-deoxy-4-keto-D-arabi | 824 | 0.7 | -0.254 | -0.445 | -0.064 | 0.009 |
| ENZRXN.25 | [sulfur carrier protein TtuB] | 960 | 0.9 | -0.405 | -0.653 | -0.157 | 0.001 |
| ENZRXN0.2 | propionyl-CoA:succinate Co | 942 | 0.8 | -0.266 | -0.437 | -0.095 | 0.002 |
| ACETATEKI | acetate kinase | 0 | Not detected | -0.067 | -0.266 | 0.132 | 0.509 |
| ACS.ENZRX | acetyl-CoA synthetase (AMP | 0 | Not detected | -0.181 | -0.416 | 0.054 | 0.132 |
| ACYLACPS | acyl-ACP synthetase | 0 | Not detected | 0.029 | -0.182 | 0.241 | 0.787 |
| ACYLCOAD | acyl-CoA dehydrogenase | 0 | Not detected | -0.104 | -0.323 | 0.114 | 0.349 |
| ACYLGPEA | 2-acylglycerophosphoethanol | 0 | Not detected | 0.029 | -0.182 | 0.241 | 0.787 |
| ADENYL.KI | adenylate kinase | 4 | Not detected | 0.042 | -0.078 | 0.163 | 0.491 |
| ADENYLAT | adenylate cyclase | 0 | Not detected | -0.041 | -0.274 | 0.193 | 0.733 |
| ADOMET.D | S-adenosylmethionine:2-dem | 0 | Not detected | 0.170 | -0.085 | 0.425 | 0.192 |
| AICARTRAN | AICAR transformylase | 0 | Not detected | 0.079 | -0.096 | 0.253 | 0.378 |
| ALDOSE1EP | galactose-1-epimerase | 0 | Not detected | 0.105 | -0.076 | 0.287 | 0.255 |
| ALPHA.AM | Î±-amylase | 0 | Not detected | 0.061 | -0.118 | 0.240 | 0.504 |
| ALPHA.AM | Î±-amylase | 0 | Not detected | -0.149 | -0.359 | 0.061 | 0.166 |
| ALTRO.OXI | tagaturonate reductase | 0 | Not detected | -0.019 | -0.206 | 0.168 | 0.840 |
| ALTRODEH | D-altronate dehydratase | 0 | Not detected | -0.011 | -0.126 | 0.103 | 0.845 |
| AMYLOMA | amylomaltase | 0 | Not detected | -0.001 | -0.226 | 0.225 | 0.995 |
| ARGS.ENZR | arginine&mdash;tRNA ligase | 0 | Not detected | 0.047 | -0.191 | 0.285 | 0.698 |
| ARGSUCCIN | argininosuccinate lyase | 0 | Not detected | -0.053 | -0.194 | 0.087 | 0.455 |
| BIOTINLIG. | biotin-[acetyl-CoA-carboxyla | 0 | Not detected | -0.053 | -0.228 | 0.123 | 0.557 |
| CARDIOLIP | cardiolipin synthase | 0 | Not detected | -0.146 | -0.322 | 0.029 | 0.101 |
| CHD.ENZRX | choline dehydrogenase | 0 | Not detected | -0.230 | -0.438 | -0.022 | 0.030 |
| CMPKI.ENZ | CMP kinase | 0 | Not detected | 0.093 | -0.130 | 0.315 | 0.413 |
| CPM.KDOSY | 3-deoxy-manno-octulosonate | 0 | Not detected | 0.335 | 0.077 | 0.593 | 0.011 |
| CROBETRE | crotonobetainyl-CoA reducta | 0 | Not detected | 0.024 | -0.190 | 0.239 | 0.824 |
| CYCPHOSD | 2',3'-cyclic nucleotide 2'-phos | 0 | Not detected | -0.004 | -0.237 | 0.229 | 0.974 |
| CYSS.ENZR | cysteine&mdash;tRNA ligase | 489 | Not detected | 0.364 | 0.112 | 0.616 | 0.005 |
| CYTIDINEK | cytidine kinase | 0 | Not detected | 0.271 | 0.022 | 0.520 | 0.033 |
| D.PPENTOM | deoxyribose 1,5-phosphomut | 0 | Not detected | 0.022 | -0.251 | 0.295 | 0.875 |
| DALADALA | D-alanine&mdash;D-alanine | 0 | Not detected | 0.012 | -0.159 | 0.183 | 0.887 |
| DARABKIN. | D-ribulokinase | 0 | Not detected | -0.158 | -0.423 | 0.107 | 0.242 |
| DEHYDDEO | 2-dehydro-3-deoxygalactono | 0 | Not detected | 0.062 | -0.120 | 0.244 | 0.507 |
| DEOXYGLU | 2-dehydro-3-deoxygluconoki | 0 | Not detected | 0.046 | -0.128 | 0.220 | 0.607 |
| DIENOYLCO | 2,4-dienoyl-CoA reductase | 0 | Not detected | -0.035 | -0.252 | 0.181 | 0.750 |
| DIHYDROF | dihydrofolate synthetase | 0 | Not detected | 0.087 | -0.103 | 0.276 | 0.370 |

| DIHYDROO | dihydroorotate dehydrogenas | 0 | Not detected | 0.000 | -0.282 | 0.282 | 0.999 |
| --- | --- | --- | --- | --- | --- | --- | --- |
| DLACTDEH | D-lactate dehydrogenase (qui | 0 | Not detected | -0.022 | -0.214 | 0.170 | 0.819 |
| DMK.ENZR | 1,4-dihydroxy-2-naphthoate | 0 | Not detected | -0.004 | -0.194 | 0.186 | 0.970 |
| DSERDEAM | D-serine ammonia-lyase | 0 | Not detected | 0.018 | -0.207 | 0.243 | 0.877 |
| DTDPDEHY | dTDP-4-dehydrorhamnose 3, | 0 | Not detected | -0.016 | -0.145 | 0.112 | 0.802 |
| DTDPGLUC | dTDP-glucose 4,6-dehydrata | 180 | Not detected | 0.114 | -0.014 | 0.242 | 0.081 |
| ENTC.ENZR | isochorismate synthase | 0 | Not detected | -0.049 | -0.222 | 0.123 | 0.577 |
| ENZRXN.10 | L-cysteine-S-conjugate thiol- | 0 | Not detected | 0.033 | -0.194 | 0.260 | 0.776 |
| ENZRXN.11 | imidazolone-5-propionate hy | 0 | Not detected | 0.070 | -0.175 | 0.316 | 0.575 |
| ENZRXN.11 | ammonia-dependent carbamo | 0 | Not detected | 0.041 | -0.131 | 0.212 | 0.643 |
| ENZRXN.11 | cystathionine &gamma;-lyase | 0 | Not detected | -0.045 | -0.313 | 0.224 | 0.745 |
| ENZRXN.12 | citrate synthase | 0 | Not detected | 0.171 | -0.140 | 0.482 | 0.282 |
| ENZRXN.12 | citrate hydro-lyase (cis-aconi | 0 | Not detected | 0.015 | -0.274 | 0.304 | 0.920 |
| ENZRXN.12 | D-threo-isocitrate hydro-lyas | 0 | Not detected | 0.015 | -0.274 | 0.304 | 0.920 |
| ENZRXN.12 | malate dehydrogenase | 0 | Not detected | -0.023 | -0.318 | 0.272 | 0.879 |
| ENZRXN.12 | 1-deoxy-D-xylulose 5-phosp | 0 | Not detected | -0.084 | -0.230 | 0.063 | 0.264 |
| ENZRXN.12 | Pfl-activating enzyme | 0 | Not detected | -0.131 | -0.387 | 0.125 | 0.316 |
| ENZRXN.12 | phenylacetate-CoA ligase | 20 | Not detected | -0.131 | -0.275 | 0.012 | 0.073 |
| ENZRXN.12 | xylitol dehydrogenase | 0 | Not detected | -0.075 | -0.364 | 0.215 | 0.614 |
| ENZRXN.12 | UDP-N-acetylenolpyruvoylgl | 0 | Not detected | -0.016 | -0.248 | 0.216 | 0.895 |
| ENZRXN.12 | UDP-N-acetylmuramoyl-L-al | 0 | Not detected | 0.202 | -0.097 | 0.501 | 0.186 |
| ENZRXN.12 | 3,5-diaminohexanoate dehyd | 0 | Not detected | -0.218 | -0.467 | 0.030 | 0.086 |
| ENZRXN.12 | 3-aminobutyryl-CoA deamin | 2 | Not detected | -0.330 | -0.603 | -0.058 | 0.018 |
| ENZRXN.13 | thiosulfate reductase (cytochr | 0 | Not detected | -0.220 | -0.473 | 0.034 | 0.090 |
| ENZRXN.13 | Enzyme IIscr | 0 | Not detected | 0.170 | -0.108 | 0.448 | 0.230 |
| ENZRXN.13 | Enzyme IIscr | 0 | Not detected | -0.060 | -0.276 | 0.157 | 0.587 |
| ENZRXN.13 | fructokinase | 0 | Not detected | 0.016 | -0.159 | 0.191 | 0.854 |
| ENZRXN.13 | phosphoribosyl-formimino-5 | 0 | Not detected | -0.045 | -0.200 | 0.110 | 0.570 |
| ENZRXN.13 | imidazoleglycerolphosphate | 0 | Not detected | 0.033 | -0.189 | 0.256 | 0.769 |
| ENZRXN.13 | sucrose phosphorylase | 0 | Not detected | 0.163 | -0.147 | 0.473 | 0.303 |
| ENZRXN.13 | phosphoserine aminotransfer | 0 | Not detected | 0.027 | -0.105 | 0.159 | 0.689 |
| ENZRXN.13 | phosphoglucose isomerase | 0 | Not detected | -0.130 | -0.404 | 0.143 | 0.350 |
| ENZRXN.13 | 3-phosphoglycerate kinase | 0 | Not detected | -0.028 | -0.163 | 0.107 | 0.683 |
| ENZRXN.13 | myo-inosose-2 dehydratase | 0 | Not detected | 0.041 | -0.181 | 0.263 | 0.716 |
| ENZRXN.13 | glucose-6-phosphate isomera | 0 | Not detected | 0.102 | -0.174 | 0.377 | 0.471 |
| ENZRXN.13 | fructokinase | 0 | Not detected | 0.001 | -0.230 | 0.233 | 0.993 |
| ENZRXN.13 | glucose-6-phosphate isomera | 0 | Not detected | -0.023 | -0.144 | 0.098 | 0.711 |
| ENZRXN.13 | phosphotransacetylase | 0 | Not detected | -0.040 | -0.242 | 0.162 | 0.699 |
| ENZRXN.14 | UDP-N-acetylglucosamine 4- | 0 | Not detected | -0.269 | -0.587 | 0.049 | 0.098 |
| ENZRXN.14 | UDP-galactose 4-epimerase | 0 | Not detected | -0.269 | -0.587 | 0.049 | 0.098 |
| ENZRXN.14 | cobalt precorrin-4 (C11)-met | 5 | Not detected | 0.064 | -0.076 | 0.205 | 0.368 |
| ENZRXN.14 | cobalt-precorrin-3 (C17)-met | 0 | Not detected | 0.142 | -0.123 | 0.407 | 0.293 |
| ENZRXN.14 | GDP-D-mannose pyrophosph | 0 | Not detected | -0.009 | -0.247 | 0.229 | 0.943 |
| ENZRXN.14 | phosphomannose isomerase | 0 | Not detected | -0.009 | -0.247 | 0.229 | 0.943 |

| ENZRXN.14 | succinyl-CoA:CoA transferas | 0 | Not detected | -0.025 | -0.241 | 0.191 | 0.819 |
| --- | --- | --- | --- | --- | --- | --- | --- |
| ENZRXN.14 | 4-hydroxybutyrate coenzyme | 0 | Not detected | -0.055 | -0.319 | 0.209 | 0.683 |
| ENZRXN.14 | crotonase | 0 | Not detected | -0.042 | -0.325 | 0.240 | 0.769 |
| ENZRXN.14 | butyryl-CoA dehydrogenase | 0 | Not detected | 0.056 | -0.151 | 0.262 | 0.597 |
| ENZRXN.14 | phosphate acetyltransferase | 0 | Not detected | -0.026 | -0.150 | 0.097 | 0.676 |
| ENZRXN.14 | acetate kinase | 0 | Not detected | 0.094 | -0.155 | 0.342 | 0.460 |
| ENZRXN.14 | GDP-Î±-D-mannose 4,6-dehy | 25 | Not detected | 0.296 | 0.028 | 0.564 | 0.030 |
| ENZRXN.14 | aconitase | 86 | Not detected | -0.129 | -0.280 | 0.021 | 0.092 |
| ENZRXN.14 | phosphoenolpyruvate phosph | 0 | Not detected | -0.001 | -0.284 | 0.283 | 0.996 |
| ENZRXN.14 | naphthoyl-CoA synthase | 0 | Not detected | -0.067 | -0.202 | 0.068 | 0.329 |
| ENZRXN.14 | N-acetylhexosamine kinase | 0 | Not detected | -0.176 | -0.457 | 0.106 | 0.221 |
| ENZRXN.14 | glutamyl-tRNAGln synthetas | 0 | Not detected | 0.127 | -0.160 | 0.413 | 0.387 |
| ENZRXN.15 | glutamyl-tRNAGlu synthetas | 0 | Not detected | 0.127 | -0.160 | 0.413 | 0.387 |
| ENZRXN.15 | 1-acylglycerol-3-phosphate a | 0 | Not detected | -0.075 | -0.289 | 0.140 | 0.495 |
| ENZRXN.15 | biotin sulfoxide reductase | 0 | Not detected | -0.079 | -0.302 | 0.144 | 0.486 |
| ENZRXN.15 | malonate decarboxylase acety | 0 | Not detected | -0.149 | -0.345 | 0.047 | 0.135 |
| ENZRXN.15 | acyl-carrier protein: acetate li | 0 | Not detected | 0.203 | -0.086 | 0.492 | 0.169 |
| ENZRXN.15 | acetyl-[acyl-carrier protein]:m | 0 | Not detected | 0.145 | -0.130 | 0.420 | 0.301 |
| ENZRXN.15 | carboxybiotin decarboxylase | 486 | Not detected | -0.281 | -0.498 | -0.063 | 0.012 |
| ENZRXN.15 | N-succinylornithine carbamo | 0 | Not detected | -0.061 | -0.203 | 0.080 | 0.393 |
| ENZRXN.15 | 5-monosubstituted-hydantoin | 0 | Not detected | 0.124 | -0.146 | 0.394 | 0.368 |
| ENZRXN.15 | phosphoglycerate mutase | 2 | Not detected | 0.258 | -0.020 | 0.537 | 0.069 |
| ENZRXN.15 | 2-keto-3-deoxy-D-glycero-D- | 0 | Not detected | -0.272 | -0.591 | 0.046 | 0.094 |
| ENZRXN.15 | 2-keto-3-deoxy-D-glycero-D- | 0 | Not detected | -0.285 | -0.582 | 0.012 | 0.060 |
| ENZRXN.16 | Î²-D-galactosyl-(1->4)-L-rha | 0 | Not detected | -0.135 | -0.437 | 0.167 | 0.381 |
| ENZRXN.17 | UDP-N-acetylglucosamine en | 0 | Not detected | -0.091 | -0.383 | 0.201 | 0.543 |
| ENZRXN.17 | UDP-galactopyranose mutase | 0 | Not detected | 0.080 | -0.230 | 0.390 | 0.613 |
| ENZRXN.17 | 3-dehydroquinate dehydratas | 0 | Not detected | 0.033 | -0.205 | 0.272 | 0.785 |
| ENZRXN.17 | UDP-N-acetylglucosamine-- | 0 | Not detected | -0.017 | -0.186 | 0.153 | 0.848 |
| ENZRXN.17 | UDP-N-acetylmuramoyl--L-a | 0 | Not detected | -0.144 | -0.401 | 0.113 | 0.273 |
| ENZRXN.17 | UDP-N-acetylglucosamine 1- | 0 | Not detected | -0.001 | -0.199 | 0.198 | 0.995 |
| ENZRXN.17 | UDP-N-acetylglucosamine 1- | 0 | Not detected | 0.023 | -0.142 | 0.189 | 0.781 |
| ENZRXN.17 | undecaprenyl-pyrophosphory | 0 | Not detected | 0.210 | -0.087 | 0.507 | 0.166 |
| ENZRXN.17 | asparagine synthase | 2 | Not detected | 0.341 | 0.048 | 0.633 | 0.023 |
| ENZRXN.17 | undecaprenyl-pyrophosphory | 0 | Not detected | 0.210 | -0.087 | 0.507 | 0.166 |
| ENZRXN.17 | D-sedoheptulose 7-phosphate | 0 | Not detected | 0.020 | -0.246 | 0.286 | 0.884 |
| ENZRXN.17 | D-glycero-Î±-D-manno-hepto | 0 | Not detected | -0.010 | -0.312 | 0.293 | 0.950 |
| ENZRXN.17 | methylthioalkylmalate dehyd | 0 | Not detected | 0.011 | -0.126 | 0.148 | 0.877 |
| ENZRXN.17 | methylthioalkylmalate dehyd | 0 | Not detected | 0.011 | -0.126 | 0.148 | 0.877 |
| ENZRXN.17 | methylthioalkylmalate dehyd | 0 | Not detected | 0.011 | -0.126 | 0.148 | 0.877 |
| ENZRXN.17 | methylthioalkylmalate dehyd | 0 | Not detected | 0.011 | -0.126 | 0.148 | 0.877 |
| ENZRXN.17 | methylthioalkylmalate dehyd | 0 | Not detected | 0.011 | -0.126 | 0.148 | 0.877 |
| ENZRXN.17 | lipoate-ATP adenylate transfe | 0 | Not detected | -0.008 | -0.166 | 0.150 | 0.921 |
| ENZRXN.17 | (2Z,6E)-farnesyl diphosphate | 0 | Not detected | 0.189 | -0.100 | 0.479 | 0.200 |

| ENZRXN.18 | 3-(hydroxyamino)phenol mut | 0 | Not detected | 0.185 | -0.111 | 0.482 | 0.220 |
| --- | --- | --- | --- | --- | --- | --- | --- |
| ENZRXN.18 | butanal dehydrogenase | 0 | Not detected | -0.028 | -0.159 | 0.103 | 0.675 |
| ENZRXN.18 | butanol dehydrogenase | 0 | Not detected | -0.028 | -0.159 | 0.103 | 0.675 |
| ENZRXN.18 | glycerol-2-phosphotransferas | 0 | Not detected | -0.007 | -0.254 | 0.239 | 0.953 |
| ENZRXN.18 | DsrC-trisulfide reductase | 0 | Not detected | -0.107 | -0.374 | 0.160 | 0.432 |
| ENZRXN.18 | dCMP kinase | 0 | Not detected | 0.093 | -0.130 | 0.315 | 0.413 |
| ENZRXN.18 | 3-hydroxy-trans-cinnamate h | 0 | Not detected | -0.134 | -0.323 | 0.054 | 0.164 |
| ENZRXN.18 | 1-deoxy-D-xylulose 5-phosp | 0 | Not detected | -0.175 | -0.465 | 0.115 | 0.236 |
| ENZRXN.18 | rhamnogalacturonan endolya | 0 | Not detected | -0.205 | -0.428 | 0.017 | 0.071 |
| ENZRXN.18 | unsaturated chondroitin 6-sul | 75 | Not detected | -0.334 | -0.569 | -0.100 | 0.005 |
| ENZRXN.18 | legionaminate synthase | 0 | Not detected | 0.033 | -0.228 | 0.294 | 0.803 |
| ENZRXN.18 | rhamnogalacturonan exolyase | 0 | Not detected | -0.095 | -0.339 | 0.149 | 0.444 |
| ENZRXN.18 | isoamylase | 0 | Not detected | -0.086 | -0.355 | 0.183 | 0.532 |
| ENZRXN.18 | formate dehydrogenase | 0 | Not detected | -0.076 | -0.322 | 0.170 | 0.544 |
| ENZRXN.19 | cellobiose phosphorylase | 7 | Not detected | 0.079 | -0.065 | 0.222 | 0.283 |
| ENZRXN.19 | GTP 3',8'-cyclase | 0 | Not detected | 0.133 | -0.053 | 0.320 | 0.161 |
| ENZRXN.19 | endo-Î±-N-acetylgalactosami | 0 | Not detected | -0.279 | -0.580 | 0.023 | 0.070 |
| ENZRXN.19 | trans-2-enoyl-CoA reductase | 0 | Not detected | -0.159 | -0.394 | 0.076 | 0.184 |
| ENZRXN.19 | crotonyl-CoA reductase | 0 | Not detected | -0.159 | -0.394 | 0.076 | 0.184 |
| ENZRXN.19 | trans-2-hexenoyl-CoA reduct | 0 | Not detected | -0.159 | -0.394 | 0.076 | 0.184 |
| ENZRXN.19 | hexanal dehydrogenase | 0 | Not detected | -0.028 | -0.159 | 0.103 | 0.675 |
| ENZRXN.19 | hexanol dehydrogenase | 0 | Not detected | -0.028 | -0.159 | 0.103 | 0.675 |
| ENZRXN.19 | carboxy-adenylated-[ThiS su | 0 | Not detected | 0.108 | -0.099 | 0.315 | 0.306 |
| ENZRXN.19 | molybdopterin synthase sulfu | 0 | Not detected | -0.015 | -0.182 | 0.151 | 0.857 |
| ENZRXN.19 | N,N'-diacetylchitobiose phos | 0 | Not detected | -0.177 | -0.456 | 0.101 | 0.212 |
| ENZRXN.19 | 12Î±-hydroxy-3-oxochol-4,6- | 0 | Not detected | 0.030 | -0.242 | 0.303 | 0.828 |
| ENZRXN.19 | dTDP-glucose 4,6-dehydrata | 0 | Not detected | -0.064 | -0.281 | 0.154 | 0.568 |
| ENZRXN.19 | glucose-1-phosphate thymidy | 0 | Not detected | 0.009 | -0.119 | 0.138 | 0.887 |
| ENZRXN.19 | dTDP-3-amino-3,6-dideoxy-Î | 0 | Not detected | -0.071 | -0.365 | 0.222 | 0.634 |
| ENZRXN.19 | 2-oxoglutarate decarboxylase | 0 | Not detected | -0.130 | -0.431 | 0.171 | 0.397 |
| ENZRXN.20 | deoxyribonuclease IV | 103 | Not detected | -0.220 | -0.395 | -0.046 | 0.013 |
| ENZRXN.20 | uracil-DNA glycosylase | 1 | Not detected | -0.069 | -0.183 | 0.046 | 0.239 |
| ENZRXN.20 | DNA glycosylase | 0 | Not detected | 0.020 | -0.245 | 0.284 | 0.884 |
| ENZRXN.20 | DNA-formamidopyrimidine | 0 | Not detected | 0.049 | -0.128 | 0.225 | 0.588 |
| ENZRXN.20 | adenine glycosylase | 0 | Not detected | 0.054 | -0.135 | 0.242 | 0.575 |
| ENZRXN.20 | glycerol-3-phosphate dehydr | 0 | Not detected | -0.212 | -0.476 | 0.053 | 0.117 |
| ENZRXN.20 | 3-isopropylmalate dehydratas | 79 | Not detected | -0.327 | -0.634 | -0.019 | 0.037 |
| ENZRXN.20 | (S)-NADPHX dehydratase | 0 | Not detected | -0.086 | -0.287 | 0.115 | 0.401 |
| ENZRXN.20 | NADPHX epimerase | 0 | Not detected | -0.086 | -0.287 | 0.115 | 0.401 |
| ENZRXN.20 | butanol dehydrogenase | 0 | Not detected | 0.028 | -0.105 | 0.160 | 0.684 |
| ENZRXN.20 | CoA-dependent propionaldeh | 0 | Not detected | -0.113 | -0.344 | 0.118 | 0.339 |
| ENZRXN.20 | phosphate propanoyltransfera | 0 | Not detected | -0.232 | -0.475 | 0.011 | 0.061 |
| ENZRXN.20 | phosphate acetyltransferase | 0 | Not detected | -0.232 | -0.475 | 0.011 | 0.061 |
| ENZRXN.20 | propionate kinase | 0 | Not detected | -0.036 | -0.254 | 0.183 | 0.749 |

| ENZRXN.20 | thymine oxygenase | 0 | Not detected | 0.044 | -0.122 | 0.210 | 0.605 |
| --- | --- | --- | --- | --- | --- | --- | --- |
| ENZRXN.20 | hydroxybutyryl-coA dehydro | 0 | Not detected | -0.047 | -0.216 | 0.121 | 0.582 |
| ENZRXN.20 | biotin carboxylase | 0 | Not detected | 0.008 | -0.252 | 0.267 | 0.954 |
| ENZRXN.20 | N,N'-diacetylbacilliosaminyl- | 0 | Not detected | -0.151 | -0.422 | 0.120 | 0.276 |
| ENZRXN.20 | carboxyspermidine dehydrog | 3 | Not detected | 0.008 | -0.114 | 0.130 | 0.899 |
| ENZRXN.20 | N-carbamoylputrescine amid | 0 | Not detected | -0.043 | -0.185 | 0.099 | 0.554 |
| ENZRXN.20 | 4,4'-diaponeurosporenal dehy | 0 | Not detected | -0.262 | -0.549 | 0.026 | 0.074 |
| ENZRXN.20 | superoxide dismutase | 0 | Not detected | -0.269 | -0.536 | -0.003 | 0.047 |
| ENZRXN.20 | UDP-N-acetyl-2-amino-2-de | 0 | Not detected | -0.009 | -0.306 | 0.289 | 0.955 |
| ENZRXN.20 | UDP-2,3-diacetamido-2,3-di | 0 | Not detected | -0.241 | -0.536 | 0.055 | 0.111 |
| ENZRXN.20 | enoyl-[acyl-carrier-protein] r | 0 | Not detected | 0.199 | -0.091 | 0.488 | 0.178 |
| ENZRXN.20 | enoyl-[acyl-carrier-protein] r | 0 | Not detected | 0.199 | -0.091 | 0.488 | 0.178 |
| ENZRXN.20 | crotonyl-[acyl-carrier-protein | 0 | Not detected | 0.199 | -0.091 | 0.488 | 0.178 |
| ENZRXN.20 | endo-1,4-Î²-xylanase | 0 | Not detected | 0.177 | -0.044 | 0.398 | 0.116 |
| ENZRXN.20 | D-fructose-6-phosphate cytid | 0 | Not detected | -0.064 | -0.347 | 0.219 | 0.658 |
| ENZRXN.21 | acetaldehyde dehydrogenase | 0 | Not detected | -0.157 | -0.398 | 0.084 | 0.203 |
| ENZRXN.21 | homoserine O-acetyltransfera | 0 | Not detected | 0.023 | -0.214 | 0.261 | 0.847 |
| ENZRXN.21 | methylthioribose-1-phosphat | 0 | Not detected | 0.099 | -0.181 | 0.379 | 0.487 |
| ENZRXN.21 | oligosaccharide reducing-end | 0 | Not detected | 0.045 | -0.156 | 0.246 | 0.659 |
| ENZRXN.21 | Î²-L-arabinofuranosidase | 0 | Not detected | 0.101 | -0.187 | 0.388 | 0.493 |
| ENZRXN.21 | uridine kinase | 0 | Not detected | 0.271 | 0.022 | 0.520 | 0.033 |
| ENZRXN.21 | cytidine kinase | 0 | Not detected | 0.271 | 0.022 | 0.520 | 0.033 |
| ENZRXN.21 | urocanate reductase | 46 | Not detected | 0.499 | 0.199 | 0.800 | 0.001 |
| ENZRXN.21 | 3D-(3,5/4)-trihydroxycyclohe | 0 | Not detected | 0.129 | -0.062 | 0.320 | 0.185 |
| ENZRXN.21 | myo-inositol 2-dehydrogenas | 0 | Not detected | 0.167 | -0.070 | 0.404 | 0.168 |
| ENZRXN.21 | D-mannonate dehydrogenase | 0 | Not detected | -0.034 | -0.174 | 0.106 | 0.637 |
| ENZRXN.21 | D-mannonate dehydratase | 0 | Not detected | -0.043 | -0.176 | 0.090 | 0.523 |
| ENZRXN.21 | 2-keto-3-deoxygluconate kin | 0 | Not detected | -0.019 | -0.146 | 0.107 | 0.766 |
| ENZRXN.21 | [sulfur carrier protein CysO]- | 0 | Not detected | 0.042 | -0.230 | 0.315 | 0.760 |
| ENZRXN.21 | N-acetyl-Î±-D-glucosaminyl- | 0 | Not detected | 0.012 | -0.278 | 0.303 | 0.934 |
| ENZRXN.21 | glucose-1-phosphate thymidy | 0 | Not detected | -0.215 | -0.432 | 0.003 | 0.053 |
| ENZRXN.21 | dTDP-glucose 4,6-dehydrata | 35 | Not detected | -0.253 | -0.460 | -0.045 | 0.017 |
| ENZRXN.21 | dTDP-4-amino-4,6-dideoxy- | 0 | Not detected | -0.161 | -0.467 | 0.145 | 0.303 |
| ENZRXN.21 | UDP-glucose 6-dehydrogena | 0 | Not detected | -0.083 | -0.360 | 0.194 | 0.556 |
| ENZRXN.21 | UDP-N-acetylglucosamine 4, | 0 | Not detected | -0.102 | -0.346 | 0.142 | 0.413 |
| ENZRXN.21 | UDP-2-acetamido-2,6-dideox | 0 | Not detected | -0.337 | -0.627 | -0.047 | 0.023 |
| ENZRXN.21 | UDP-N-acetyl-Î²-L-rhamnosa | 0 | Not detected | -0.124 | -0.382 | 0.134 | 0.346 |
| ENZRXN.22 | UDP-N-acetylglucosamine 4, | 0 | Not detected | -0.099 | -0.363 | 0.165 | 0.461 |
| ENZRXN.22 | Î²-L-arabinobiosidase | 0 | Not detected | 0.025 | -0.302 | 0.352 | 0.881 |
| ENZRXN.22 | butyryl-CoA dehydrogenase | 0 | Not detected | 0.005 | -0.129 | 0.139 | 0.944 |
| ENZRXN.22 | succinyl-CoA:acetate CoA-tr | 0 | Not detected | -0.022 | -0.320 | 0.276 | 0.884 |
| ENZRXN.22 | propionyl-CoA:succinate Co | 0 | Not detected | -0.022 | -0.320 | 0.276 | 0.884 |
| ENZRXN.22 | N-acetyl-Î²-neuraminate:H+ s | 0 | Not detected | 0.000 | -0.190 | 0.191 | 0.996 |
| ENZRXN.22 | 1,4-Î²-mannanase | 0 | Not detected | -0.244 | -0.499 | 0.012 | 0.062 |

| ENZRXN.22 | mannobiose 2-epimerase | 0 | Not detected | -0.184 | -0.402 | 0.034 | 0.099 |
| --- | --- | --- | --- | --- | --- | --- | --- |
| ENZRXN.22 | cellobiose 2-epimerase | 0 | Not detected | -0.184 | -0.402 | 0.034 | 0.099 |
| ENZRXN.22 | cellobiose 2-epimerase | 0 | Not detected | -0.017 | -0.271 | 0.237 | 0.897 |
| ENZRXN.22 | UDP-acetamido-4-amino-6-d | 0 | Not detected | -0.200 | -0.495 | 0.095 | 0.184 |
| ENZRXN.22 | 2-oxo-4-methylthiobutanoate | 0 | Not detected | 0.018 | -0.210 | 0.245 | 0.880 |
| ENZRXN.23 | CoA-dependent (R)-lactaldeh | 0 | Not detected | -0.113 | -0.344 | 0.118 | 0.339 |
| ENZRXN.23 | CoA-dependent (S)-lactaldeh | 0 | Not detected | -0.113 | -0.344 | 0.118 | 0.339 |
| ENZRXN.23 | (R)-2,3-dihydroxy-3-methylb | 0 | Not detected | -0.007 | -0.132 | 0.118 | 0.910 |
| ENZRXN.23 | (R)-2,3-dihydroxy-3-methylp | 0 | Not detected | -0.007 | -0.132 | 0.118 | 0.910 |
| ENZRXN.23 | (R)-2,3-dihydroxy-3-methylb | 0 | Not detected | -0.085 | -0.343 | 0.173 | 0.517 |
| ENZRXN.23 | (R)-2,3-dihydroxy-3-methylp | 0 | Not detected | -0.085 | -0.343 | 0.173 | 0.517 |
| ENZRXN.23 | Fe-coproporphyrin synthase | 0 | Not detected | -0.020 | -0.293 | 0.254 | 0.887 |
| ENZRXN.23 | bacimethrin diphosphokinase | 3 | Not detected | 0.053 | -0.118 | 0.224 | 0.543 |
| ENZRXN.23 | L-galactose dehydrogenase | 79 | Not detected | -0.278 | -0.492 | -0.065 | 0.011 |
| ENZRXN.23 | L-galactonate 5-dehydrogena | 547 | Not detected | -0.303 | -0.511 | -0.095 | 0.004 |
| ENZRXN.23 | N-acetylneuraminate lyase | 599 | Not detected | -0.252 | -0.445 | -0.058 | 0.011 |
| ENZRXN.23 | N-acetylglucosamine kinase | 0 | Not detected | -0.060 | -0.197 | 0.076 | 0.386 |
| ENZRXN.23 | 7-oxolithocholate reductase | 0 | Not detected | -0.165 | -0.407 | 0.077 | 0.182 |
| ENZRXN.23 | 7-oxolithocholate reductase | 0 | Not detected | -0.165 | -0.407 | 0.077 | 0.182 |
| ENZRXN.23 | UDP-N-acetylglucosamine 6- | 0 | Not detected | -0.194 | -0.394 | 0.007 | 0.059 |
| ENZRXN.23 | UDP-N-acetylgalactosamine | 0 | Not detected | -0.194 | -0.394 | 0.007 | 0.059 |
| ENZRXN.23 | Kdo2-lipid A phosphotransfe | 0 | Not detected | -0.027 | -0.256 | 0.202 | 0.819 |
| ENZRXN.23 | Kdo2-lipid A phosphoethano | 0 | Not detected | -0.022 | -0.231 | 0.187 | 0.837 |
| ENZRXN.23 | phosphomannomutase | 0 | Not detected | 0.278 | -0.004 | 0.559 | 0.054 |
| ENZRXN.23 | phosphoglucomutase | 0 | Not detected | 0.278 | -0.004 | 0.559 | 0.054 |
| ENZRXN.23 | heparin lyase | 0 | Not detected | -0.181 | -0.544 | 0.182 | 0.328 |
| ENZRXN.23 | heparan sulfate lyase | 0 | Not detected | -0.181 | -0.544 | 0.182 | 0.328 |
| ENZRXN.23 | heparin lyase | 0 | Not detected | -0.242 | -0.512 | 0.029 | 0.080 |
| ENZRXN.23 | unsaturated hyaluronan disac | 75 | Not detected | -0.334 | -0.569 | -0.100 | 0.005 |
| ENZRXN.23 | unsaturated chondroitin disac | 75 | Not detected | -0.334 | -0.569 | -0.100 | 0.005 |
| ENZRXN.23 | 4-deoxy-Î²-L-erythro-hex-4-e | 0 | Not detected | -0.057 | -0.345 | 0.231 | 0.697 |
| ENZRXN.23 | 4-deoxy-Î²-D-gluc-4-enurono | 0 | Not detected | -0.057 | -0.345 | 0.231 | 0.697 |
| ENZRXN.23 | 4-deoxy-Î²-D-gluc-4-enurono | 0 | Not detected | -0.057 | -0.345 | 0.231 | 0.697 |
| ENZRXN.23 | 4-deoxy-Î²-L-erythro-hex-4-e | 0 | Not detected | -0.057 | -0.345 | 0.231 | 0.697 |
| ENZRXN.23 | (5Z)-3-oxo-tetradec-5-enoyl- | 0 | Not detected | -0.055 | -0.185 | 0.075 | 0.407 |
| ENZRXN.23 | (5Z)-3-oxo-tetradec-5-enoyl- | 0 | Not detected | 0.110 | -0.127 | 0.346 | 0.363 |
| ENZRXN.23 | (2E,5Z)-tetradeca-2,5-dienoy | 0 | Not detected | -0.017 | -0.221 | 0.188 | 0.873 |
| ENZRXN.23 | (3R)-3-hydroxydecanoyl-[acp | 0 | Not detected | 0.015 | -0.185 | 0.215 | 0.882 |
| ENZRXN.23 | (2E)-dec-2-enoyl-[acp] isome | 0 | Not detected | 0.015 | -0.185 | 0.215 | 0.882 |
| ENZRXN.23 | (5Z)-3-oxo-dodec-5-enoyl-[a | 0 | Not detected | 0.002 | -0.123 | 0.128 | 0.970 |
| ENZRXN.23 | (7Z)-3-oxo-hexadec-7-enoyl- | 0 | Not detected | -0.055 | -0.185 | 0.075 | 0.407 |
| ENZRXN.23 | (9Z)-3-oxo-octadec-9-enoyl-[ | 0 | Not detected | -0.055 | -0.185 | 0.075 | 0.407 |
| ENZRXN.23 | (7Z)-3-oxo-hexadec-7-enoyl- | 0 | Not detected | 0.110 | -0.127 | 0.346 | 0.363 |
| ENZRXN.23 | (9Z)-3-oxo-octadec-9-enoyl-[ | 0 | Not detected | 0.110 | -0.127 | 0.346 | 0.363 |

| ENZRXN.23 | (11Z)-3-oxo-icos-11-enoyl-[a | 0 | Not detected | 0.110 | -0.127 | 0.346 | 0.363 |
| --- | --- | --- | --- | --- | --- | --- | --- |
| ENZRXN.23 | (11Z)-3-oxo-icos-11-enoyl-[a | 0 | Not detected | -0.055 | -0.185 | 0.075 | 0.407 |
| ENZRXN.23 | (2E,7Z)-hexadeca-2,7-dienoy | 0 | Not detected | -0.017 | -0.221 | 0.188 | 0.873 |
| ENZRXN.23 | (2E,9Z)-octadeca-2,9-dienoy | 0 | Not detected | -0.017 | -0.221 | 0.188 | 0.873 |
| ENZRXN.23 | (2E,11Z)-icosa-2,11-dienoyl- | 0 | Not detected | -0.017 | -0.221 | 0.188 | 0.873 |
| ENZRXN.23 | lactose 2-epimerase | 0 | Not detected | -0.017 | -0.271 | 0.237 | 0.897 |
| ENZRXN.23 | 8-amino-3,8-dideoxy-manno- | 0 | Not detected | 0.015 | -0.235 | 0.266 | 0.904 |
| ENZRXN.24 | scaffold protein for iron-sulfu | 0 | Not detected | 0.117 | -0.131 | 0.366 | 0.355 |
| ENZRXN.24 | lactate racemase | 0 | Not detected | -0.120 | -0.345 | 0.106 | 0.298 |
| ENZRXN.24 | UDP-4-amino-4,6-dideoxy-N | 0 | Not detected | 0.041 | -0.265 | 0.348 | 0.792 |
| ENZRXN.24 | guanosine phosphorylase | 0 | Not detected | 0.115 | -0.170 | 0.401 | 0.429 |
| ENZRXN.24 | deoxycholate 3Î±-dehydroge | 0 | Not detected | -0.052 | -0.248 | 0.144 | 0.605 |
| ENZRXN.24 | deoxycholate 3Î±-dehydroge | 0 | Not detected | 0.287 | 0.000 | 0.573 | 0.050 |
| ENZRXN.24 | 3-oxochol-4,6-dien-24-oyl-C | 0 | Not detected | 0.030 | -0.242 | 0.303 | 0.828 |
| ENZRXN.24 | lithocholate 3Î±-dehydrogena | 0 | Not detected | 0.287 | 0.000 | 0.573 | 0.050 |
| ENZRXN.24 | chenodeoxycholate 3Î±-dehy | 0 | Not detected | 0.287 | 0.000 | 0.573 | 0.050 |
| ENZRXN.24 | cholate 3Î±-dehydrogenase | 0 | Not detected | 0.287 | 0.000 | 0.573 | 0.050 |
| ENZRXN.24 | lithocholate 3Î±-dehydrogena | 0 | Not detected | -0.052 | -0.248 | 0.144 | 0.605 |
| ENZRXN.24 | cholate 3Î±-dehydrogenase | 0 | Not detected | -0.052 | -0.248 | 0.144 | 0.605 |
| ENZRXN.24 | chenodeoxycholate 3Î±-dehy | 0 | Not detected | -0.052 | -0.248 | 0.144 | 0.605 |
| ENZRXN.24 | Î²-1,2-mannosidase | 0 | Not detected | -0.184 | -0.424 | 0.056 | 0.133 |
| ENZRXN.24 | L-galacto-1,5-lactonase | 0 | Not detected | -0.147 | -0.350 | 0.057 | 0.158 |
| ENZRXN.24 | succinate:malate antiport | 0 | Not detected | 0.139 | -0.104 | 0.383 | 0.262 |
| ENZRXN.24 | succinate:L-aspartate antiport | 0 | Not detected | 0.139 | -0.104 | 0.383 | 0.262 |
| ENZRXN.24 | malate:succinate antiporter | 0 | Not detected | 0.119 | -0.090 | 0.328 | 0.264 |
| ENZRXN.24 | L-aspartate:succinate antiport | 0 | Not detected | 0.119 | -0.090 | 0.328 | 0.264 |
| ENZRXN.24 | dihydroorotate dehydrogenas | 0 | Not detected | 0.000 | -0.282 | 0.282 | 0.999 |
| ENZRXN.24 | methionine &gamma;-lyase | 0 | Not detected | -0.154 | -0.406 | 0.099 | 0.233 |
| ENZRXN.24 | [HypE]-S-carboxamide dehy | 0 | Not detected | -0.014 | -0.208 | 0.180 | 0.888 |
| ENZRXN.25 | Î±-D-ribose-1-(2-N-acetamid | 0 | Not detected | -0.148 | -0.337 | 0.042 | 0.127 |
| ENZRXN.25 | carbon-phosphorous lyase | 0 | Not detected | -0.248 | -0.467 | -0.029 | 0.026 |
| ENZRXN.25 | Î±-D-ribose-1-[N-(phosphon | 0 | Not detected | -0.032 | -0.243 | 0.178 | 0.764 |
| ENZRXN.25 | Î±-D-ribose-1-[N-(phosphon | 0 | Not detected | -0.078 | -0.327 | 0.172 | 0.542 |
| ENZRXN.25 | adenine phosphoribosyltransf | 5 | Not detected | 0.217 | -0.052 | 0.486 | 0.115 |
| ENZRXN.25 | tetracycline 11a-oxygenase | 0 | Not detected | -0.399 | -0.772 | -0.027 | 0.036 |
| ENZRXN.25 | tigecycline 11a-momooxygen | 0 | Not detected | -0.399 | -0.772 | -0.027 | 0.036 |
| ENZRXN.25 | UDP-N-acetylglucosamine 2- | 0 | Not detected | -0.191 | -0.479 | 0.096 | 0.192 |
| ENZRXN.25 | ribitol-5-phosphate cytidylylt | 71 | Not detected | 0.342 | 0.056 | 0.628 | 0.019 |
| ENZRXN.25 | ribitol-5-phosphate 1-dehydr | 0 | Not detected | 0.232 | -0.053 | 0.517 | 0.110 |
| ENZRXN.25 | ribitol-5-phosphate 1-dehydr | 0 | Not detected | -0.100 | -0.397 | 0.196 | 0.507 |
| ENZRXN.25 | UTP--glucose-1-phosphate u | 0 | Not detected | 0.176 | -0.096 | 0.449 | 0.205 |
| ENZRXN.25 | undecaprenyl-phosphate 2-ac | 0 | Not detected | 0.158 | -0.105 | 0.421 | 0.239 |
| ENZRXN.25 | D-alanine--[D-alanyl carrier | 0 | Not detected | 0.074 | -0.201 | 0.350 | 0.597 |
| ENZRXN.25 | PG:teichoic acid D-alanyltran | 0 | Not detected | 0.031 | -0.222 | 0.284 | 0.809 |

| ENZRXN.25 | D-alanine carrier protein:PG | 0 | Not detected | -0.010 | -0.262 | 0.241 | 0.937 |
| --- | --- | --- | --- | --- | --- | --- | --- |
| ENZRXN.25 | 1,2-diacylglycerol 3-Î±-gluco | 0 | Not detected | -0.023 | -0.263 | 0.217 | 0.851 |
| ENZRXN.25 | isopropylmalate dehydrogena | 0 | Not detected | 0.011 | -0.126 | 0.148 | 0.877 |
| ENZRXN.25 | tRNA uridine 4-sulfurtransfe | 0 | Not detected | 0.108 | -0.099 | 0.315 | 0.306 |
| ENZRXN.25 | aminobutyraldehyde dehydro | 0 | Not detected | -0.115 | -0.293 | 0.064 | 0.207 |
| ENZRXN.25 | succinate-semialdehyde dehy | 0 | Not detected | -0.115 | -0.293 | 0.064 | 0.207 |
| ENZRXN.25 | 7,8-dihydro-8-oxoguanine D | 0 | Not detected | 0.049 | -0.128 | 0.225 | 0.588 |
| ENZRXN.25 | trans-4-hydroxy-L-proline de | 0 | Not detected | -0.085 | -0.244 | 0.073 | 0.293 |
| ENZRXN.25 | menaquinone C8-methyltrans | 0 | Not detected | 0.200 | -0.099 | 0.499 | 0.189 |
| ENZRXN.25 | quinol--ferredoxin oxidoredu | 0 | Not detected | 0.188 | -0.108 | 0.484 | 0.213 |
| ENZRXN.25 | dissimilatory sulfite reductase | 0 | Not detected | 0.328 | 0.024 | 0.633 | 0.035 |
| ENZRXN.25 | 4-phospho-D-erythronate 3-d | 0 | Not detected | -0.280 | -0.543 | -0.017 | 0.037 |
| ENZRXN.25 | UDP-N-acetylmuramate--L-a | 0 | Not detected | 0.294 | -0.006 | 0.594 | 0.055 |
| ENZRXN.25 | methenyltetrahydrofolate cyc | 1 | Not detected | -0.126 | -0.267 | 0.015 | 0.080 |
| ENZRXN.25 | methylenetetrahydrofolate de | 1 | Not detected | -0.126 | -0.267 | 0.015 | 0.080 |
| ENZRXN.25 | deoxyribodipyrimidine photo | 0 | Not detected | -0.082 | -0.272 | 0.108 | 0.398 |
| ENZRXN.25 | deoxyribodipyrimidine photo | 0 | Not detected | -0.082 | -0.272 | 0.108 | 0.398 |
| ENZRXN.25 | pyridinium-3,5-biscarboxylic | 0 | Not detected | -0.055 | -0.240 | 0.129 | 0.558 |
| ENZRXN.26 | acetate kinase | 0 | Not detected | -0.042 | -0.204 | 0.120 | 0.615 |
| ENZRXN.26 | acetaldehyde dehydrogenase | 0 | Not detected | -0.193 | -0.427 | 0.042 | 0.107 |
| ENZRXN.26 | glyoxal reductase | 0 | Not detected | 0.195 | -0.051 | 0.440 | 0.121 |
| ENZRXN.26 | methylglyoxal reductase | 0 | Not detected | 0.195 | -0.051 | 0.440 | 0.121 |
| ENZRXN.26 | 3-oxochol-4-en-24-oyl-CoA | 0 | Not detected | 0.030 | -0.242 | 0.303 | 0.828 |
| ENZRXN.26 | 12Î±-hydroxy-3-oxochol-4-e | 0 | Not detected | 0.030 | -0.242 | 0.303 | 0.828 |
| ENZRXN.26 | 3-hydroxyhexadecanoyl-CoA | 0 | Not detected | -0.062 | -0.201 | 0.078 | 0.388 |
| ENZRXN.26 | diaminohydroxyphosphoribo | 0 | Not detected | 0.165 | -0.083 | 0.414 | 0.193 |
| ENZRXN.26 | 5-amino-6-(5-phosphoribosyl | 0 | Not detected | 0.165 | -0.083 | 0.414 | 0.193 |
| ENZRXN.26 | 1,2-Î±-L-arabinofuranosidase | 0 | Not detected | 0.005 | -0.125 | 0.134 | 0.944 |
| ENZRXN.26 | 2-hydroxyethylphosphonate c | 0 | Not detected | -0.001 | -0.284 | 0.283 | 0.996 |
| ENZRXN.26 | acetate kinase | 0 | Not detected | -0.040 | -0.189 | 0.109 | 0.597 |
| ENZRXN.26 | thiol-disulfide exchange | 0 | Not detected | -0.021 | -0.231 | 0.189 | 0.844 |
| ENZRXN.26 | phosphofructokinase | 0 | Not detected | -0.021 | -0.185 | 0.142 | 0.797 |
| ENZRXN.28 | fructose-bisphosphate aldolas | 0 | Not detected | -0.002 | -0.131 | 0.127 | 0.973 |
| ENZRXN.29 | phospho-Î²-galactosidase | 0 | Not detected | -0.127 | -0.399 | 0.146 | 0.363 |
| ENZRXN.33 | aspartokinase | 0 | Not detected | -0.173 | -0.450 | 0.105 | 0.224 |
| ENZRXN.44 | GDP-4-keto-6-deoxymannos | 0 | Not detected | 0.160 | -0.115 | 0.436 | 0.255 |
| ENZRXN.58 | isoamylase | 0 | Not detected | 0.071 | -0.220 | 0.363 | 0.631 |
| ENZRXN.60 | trehalose-6-phosphate hydrol | 0 | Not detected | -0.082 | -0.230 | 0.066 | 0.279 |
| ENZRXN.61 | 16S rRNA m2G1207 methylt | 0 | Not detected | 0.055 | -0.124 | 0.235 | 0.545 |
| ENZRXN.62 | Î±-phosphoglucomutase | 53 | Not detected | 0.298 | 0.071 | 0.524 | 0.010 |
| ENZRXN.63 | UDP-glucose-4-epimerase | 0 | Not detected | 0.046 | -0.142 | 0.234 | 0.632 |
| ENZRXN.67 | N-succinyldiaminopimelate-a | 0 | Not detected | 0.162 | -0.102 | 0.425 | 0.229 |
| ENZRXN.68 | aspartate kinase | 0 | Not detected | -0.135 | -0.378 | 0.108 | 0.276 |
| ENZRXN.71 | 6-phosphogluconate dehydro | 0 | Not detected | -0.117 | -0.311 | 0.077 | 0.238 |

| ENZRXN.78 | phosphoribosylamidoimidazo | 0 | Not detected | 0.251 | -0.030 | 0.533 | 0.080 |
| --- | --- | --- | --- | --- | --- | --- | --- |
| ENZRXN.80 | nitric oxide dioxygenase | 8 | Not detected | -0.214 | -0.389 | -0.039 | 0.016 |
| ENZRXN.89 | UDP-N-acetylglucosamine 2- | 0 | Not detected | 0.075 | -0.087 | 0.236 | 0.364 |
| ENZRXN.90 | UTP-glucose-1-phosphate ur | 0 | Not detected | -0.028 | -0.211 | 0.155 | 0.765 |
| ENZRXN.93 | NAD-dependent glyceraldehy | 0 | Not detected | 0.143 | -0.129 | 0.415 | 0.302 |
| ENZRXN.95 | O-acetylhomoserine sulfhydr | 3 | Not detected | 0.015 | -0.104 | 0.135 | 0.805 |
| ENZRXN0.1 | lipoyl-adenylate--protein liga | 0 | Not detected | -0.008 | -0.166 | 0.150 | 0.921 |
| ENZRXN0.1 | 23S rRNA m5U1939 methylt | 0 | Not detected | -0.071 | -0.255 | 0.113 | 0.449 |
| ENZRXN0.1 | GTPase | 0 | Not detected | 0.033 | -0.163 | 0.230 | 0.740 |
| ENZRXN0.1 | lipoate acetyltransferase | 0 | Not detected | 0.082 | -0.146 | 0.310 | 0.480 |
| ENZRXN0.1 | lipoate-apo-GcvH protein lig | 0 | Not detected | -0.008 | -0.166 | 0.150 | 0.921 |
| ENZRXN0.1 | protein-(glutamine-N5) meth | 0 | Not detected | 0.160 | -0.007 | 0.326 | 0.060 |
| ENZRXN0.1 | protein-glutamine N-methyltr | 0 | Not detected | -0.010 | -0.186 | 0.166 | 0.908 |
| ENZRXN0.1 | propanoate:CoA ligase (AMP | 0 | Not detected | -0.181 | -0.416 | 0.054 | 0.132 |
| ENZRXN0.1 | tRNA-dihydrouridine synthas | 0 | Not detected | 0.293 | 0.041 | 0.544 | 0.023 |
| ENZRXN0.1 | tRNA-dihydrouridine synthas | 0 | Not detected | 0.085 | -0.102 | 0.273 | 0.373 |
| ENZRXN0.1 | tRNA-dihydrouridine16 synt | 0 | Not detected | 0.040 | -0.136 | 0.217 | 0.657 |
| ENZRXN0.1 | tRNA(m5U54)methyltransfer | 0 | Not detected | 0.092 | -0.109 | 0.293 | 0.371 |
| ENZRXN0.1 | deoxyribodipyrimidine photo | 0 | Not detected | -0.082 | -0.272 | 0.108 | 0.398 |
| ENZRXN0.1 | O-6-methylguanine-DNA me | 0 | Not detected | -0.167 | -0.338 | 0.005 | 0.057 |
| ENZRXN0.1 | CCA tRNA nucleotidyltransf | 0 | Not detected | 0.090 | -0.110 | 0.291 | 0.377 |
| ENZRXN0.1 | DNA adenine methyltransfer | 0 | Not detected | 0.146 | -0.041 | 0.334 | 0.126 |
| ENZRXN0.1 | tRNA dimethylallyltransferas | 0 | Not detected | 0.034 | -0.191 | 0.260 | 0.765 |
| ENZRXN0.1 | tRNA preQ134 S-adenosylm | 0 | Not detected | 0.044 | -0.099 | 0.187 | 0.546 |
| ENZRXN0.1 | ribose 1,5-bisphosphate phos | 0 | Not detected | -0.023 | -0.176 | 0.129 | 0.765 |
| ENZRXN0.1 | TDP-N-acetylfucosamine:lipi | 0 | Not detected | -0.024 | -0.210 | 0.162 | 0.799 |
| ENZRXN0.1 | ferroxidase | 0 | Not detected | -0.099 | -0.292 | 0.094 | 0.316 |
| ENZRXN0.1 | inosine:H+ antiporter | 0 | Not detected | -0.024 | -0.210 | 0.162 | 0.798 |
| ENZRXN0.2 | hydroxylamine reductase | 0 | Not detected | 0.064 | -0.132 | 0.260 | 0.520 |
| ENZRXN0.2 | diadenosine tetraphosphatase | 0 | Not detected | 0.065 | -0.113 | 0.242 | 0.476 |
| ENZRXN0.2 | DNA-cytosine methyltransfer | 0 | Not detected | -0.027 | -0.207 | 0.152 | 0.766 |
| ENZRXN0.2 | phosphatidylglycerol&mdash | 0 | Not detected | -0.015 | -0.243 | 0.214 | 0.900 |
| ENZRXN0.2 | enterochelin esterase | 0 | Not detected | -0.105 | -0.279 | 0.069 | 0.236 |
| ENZRXN0.2 | limit dextrin Î±-1,6-glucohyd | 0 | Not detected | -0.103 | -0.328 | 0.123 | 0.373 |
| ENZRXN0.2 | ferric dihyroxybenzoylserine | 0 | Not detected | -0.105 | -0.292 | 0.083 | 0.273 |
| ENZRXN0.2 | UDP-4-amino-4-deoxy-L-ara | 0 | Not detected | 0.031 | -0.142 | 0.204 | 0.726 |
| ENZRXN0.2 | guanosine:H+ antiporter | 0 | Not detected | -0.024 | -0.210 | 0.162 | 0.798 |
| ENZRXN0.2 | Î²-N-acetylglucosaminidase | 0 | Not detected | 0.036 | -0.151 | 0.222 | 0.706 |
| ENZRXN0.2 | guanine deaminase | 0 | Not detected | -0.063 | -0.267 | 0.142 | 0.548 |
| ENZRXN0.2 | crotonyl-CoA hydratase | 0 | Not detected | 0.004 | -0.188 | 0.197 | 0.964 |
| ENZRXN0.2 | L-ribulose-5-phosphate 4-epi | 0 | Not detected | -0.053 | -0.225 | 0.118 | 0.541 |
| ENZRXN0.2 | dihydropteridine reductase | 8 | Not detected | -0.214 | -0.389 | -0.039 | 0.016 |
| ENZRXN0.2 | 23S rRNA pseudouridine746 | 0 | Not detected | 0.092 | -0.083 | 0.267 | 0.301 |
| ENZRXN0.2 | 23S rRNA pseudouridine260 | 0 | Not detected | -0.032 | -0.189 | 0.125 | 0.689 |

| ENZRXN0.2 | 23S rRNA pseudouridine245 | 0 | Not detected | -0.008 | -0.222 | 0.205 | 0.939 |
| --- | --- | --- | --- | --- | --- | --- | --- |
| ENZRXN0.2 | 23S rRNA pseudouridine955 | 184 | Not detected | 0.271 | 0.065 | 0.477 | 0.010 |
| ENZRXN0.2 | inositol-phosphate phosphata | 0 | Not detected | 0.034 | -0.168 | 0.237 | 0.739 |
| ENZRXN0.2 | tRNAIle-lysidine synthetase | 0 | Not detected | -0.040 | -0.231 | 0.151 | 0.680 |
| ENZRXN0.2 | 23S rRNA pseudouridine191 | 0 | Not detected | 0.129 | -0.079 | 0.336 | 0.225 |
| ENZRXN0.2 | lipid IVA 4-amino-4-deoxy-L | 0 | Not detected | -0.041 | -0.227 | 0.145 | 0.665 |
| ENZRXN0.2 | 3-hydroxyacyl-CoA dehydro | 0 | Not detected | -0.107 | -0.311 | 0.097 | 0.304 |
| ENZRXN0.2 | dodecenoyl-CoA D-isomeras | 0 | Not detected | -0.107 | -0.311 | 0.097 | 0.304 |
| ENZRXN0.2 | 3-hydroxybutyryl-CoA epime | 0 | Not detected | -0.107 | -0.311 | 0.097 | 0.304 |
| ENZRXN0.2 | acetyl-CoA C-acyltransferase | 0 | Not detected | 0.001 | -0.181 | 0.183 | 0.993 |
| ENZRXN0.2 | short chain acyl-CoA synthet | 0 | Not detected | 0.046 | -0.182 | 0.274 | 0.694 |
| ENZRXN0.2 | enoyl-CoA hydratase | 0 | Not detected | -0.107 | -0.311 | 0.097 | 0.304 |
| ENZRXN0.2 | tyrosine kinase | 0 | Not detected | 0.082 | -0.113 | 0.277 | 0.411 |
| ENZRXN0.2 | tRNA-specific 2-thiouridylas | 0 | Not detected | -0.013 | -0.213 | 0.187 | 0.897 |
| ENZRXN0.2 | 3-hydroxyadipyl-CoA dehydr | 0 | Not detected | -0.053 | -0.226 | 0.120 | 0.548 |
| ENZRXN0.2 | D-allulose-6-phosphate 3-epi | 0 | Not detected | 0.006 | -0.269 | 0.281 | 0.967 |
| ENZRXN0.2 | NADH cupric reductase | 0 | Not detected | 0.045 | -0.147 | 0.237 | 0.645 |
| ENZRXN0.2 | DNA ligase | 0 | Not detected | 0.126 | -0.083 | 0.335 | 0.239 |
| ENZRXN0.2 | DNA ligase | 0 | Not detected | 0.133 | -0.142 | 0.408 | 0.344 |
| ENZRXN0.2 | peptidyl-prolyl cis-trans isom | 0 | Not detected | 0.156 | -0.094 | 0.407 | 0.221 |
| ENZRXN0.2 | peptidyl-prolyl cis-trans isom | 0 | Not detected | 0.066 | -0.183 | 0.316 | 0.603 |
| ENZRXN0.2 | UDP-2,3-diacylglucosamine | 0 | Not detected | -0.002 | -0.162 | 0.158 | 0.978 |
| ENZRXN0.2 | pyruvate-flavodoxin oxidored | 0 | Not detected | -0.397 | -0.713 | -0.080 | 0.014 |
| ENZRXN0.2 | malate:quinone oxidoreducta | 0 | Not detected | -0.009 | -0.207 | 0.189 | 0.930 |
| ENZRXN0.2 | fructose-6-phosphate aldolas | 0 | Not detected | -0.013 | -0.168 | 0.141 | 0.865 |
| ENZRXN0.2 | tRNA m7G46 methyltransfer | 0 | Not detected | 0.149 | -0.080 | 0.379 | 0.201 |
| ENZRXN0.2 | 10-formyltetrahydrofolate:L- | 0 | Not detected | 0.131 | -0.114 | 0.376 | 0.296 |
| ENZRXN0.3 | RNase R | 0 | Not detected | 0.018 | -0.231 | 0.267 | 0.887 |
| ENZRXN0.3 | tRNA 2-selenouridine syntha | 0 | Not detected | -0.092 | -0.267 | 0.084 | 0.307 |
| ENZRXN0.3 | endo-1,4-D-glucanase | 0 | Not detected | -0.017 | -0.193 | 0.160 | 0.852 |
| ENZRXN0.3 | UDP-N-acetylmuramate:L-al | 0 | Not detected | 0.218 | -0.039 | 0.475 | 0.096 |
| ENZRXN0.3 | 23S rRNA m5U747 methyltr | 0 | Not detected | -0.075 | -0.267 | 0.117 | 0.445 |
| ENZRXN0.3 | indoleglycerol phosphate ald | 0 | Not detected | -0.099 | -0.267 | 0.069 | 0.247 |
| ENZRXN0.3 | L-threonine:H+ antiporter | 0 | Not detected | -0.012 | -0.225 | 0.200 | 0.909 |
| ENZRXN0.3 | L-homoserine:H+ antiporter | 0 | Not detected | -0.012 | -0.225 | 0.200 | 0.909 |
| ENZRXN0.3 | Fe2+:H+ symporter | 0 | Not detected | 0.123 | -0.046 | 0.293 | 0.154 |
| ENZRXN0.3 | N-acetylmuramyl-L-alanine a | 0 | Not detected | 0.127 | -0.058 | 0.312 | 0.179 |
| ENZRXN0.3 | tRNA pseudouridine13 synth | 0 | Not detected | -0.113 | -0.287 | 0.061 | 0.203 |
| ENZRXN0.3 | tRNA pseudouridine55 synth | 0 | Not detected | 0.005 | -0.178 | 0.188 | 0.955 |
| ENZRXN0.3 | tRNA pseudouridine65 synth | 0 | Not detected | 0.094 | -0.071 | 0.259 | 0.266 |
| ENZRXN0.3 | methionine aminopeptidase | 0 | Not detected | 0.083 | -0.184 | 0.349 | 0.543 |
| ENZRXN0.3 | peptide deformylase | 0 | Not detected | 0.000 | -0.202 | 0.202 | 0.997 |
| ENZRXN0.3 | DNA polymerase | 0 | Not detected | 0.184 | -0.001 | 0.368 | 0.051 |
| ENZRXN0.3 | 16S rRNA m62A1518,m62A | 0 | Not detected | 0.019 | -0.188 | 0.226 | 0.859 |

| ENZRXN0.3 | undecaprenyl pyrophosphate | 0 | Not detected | 0.022 | -0.185 | 0.229 | 0.836 |
| --- | --- | --- | --- | --- | --- | --- | --- |
| ENZRXN0.3 | 16S rRNA m5C967 methyltr | 0 | Not detected | 0.069 | -0.144 | 0.282 | 0.527 |
| ENZRXN0.3 | topoisomerase | 0 | Not detected | 0.107 | -0.149 | 0.363 | 0.413 |
| ENZRXN0.4 | topoisomerase | 0 | Not detected | 0.047 | -0.163 | 0.256 | 0.664 |
| ENZRXN0.4 | methylene-tetrahydrofolylpol | 0 | Not detected | 0.087 | -0.103 | 0.276 | 0.370 |
| ENZRXN0.4 | 2,3-dihydroxybenzoate:oxyge | 0 | Not detected | -0.099 | -0.292 | 0.094 | 0.316 |
| ENZRXN0.4 | cuprous oxidase | 0 | Not detected | -0.099 | -0.292 | 0.094 | 0.316 |
| ENZRXN0.4 | uridine nucleosidase | 0 | Not detected | 0.030 | -0.144 | 0.204 | 0.734 |
| ENZRXN0.4 | oligopeptidase | 0 | Not detected | -0.031 | -0.218 | 0.155 | 0.742 |
| ENZRXN0.4 | oligopeptidase | 0 | Not detected | 0.169 | -0.085 | 0.423 | 0.193 |
| ENZRXN0.4 | tail-specific protease | 0 | Not detected | 0.102 | -0.117 | 0.320 | 0.361 |
| ENZRXN0.4 | protease | 0 | Not detected | -0.008 | -0.240 | 0.223 | 0.944 |
| ENZRXN0.4 | alanyl aminopeptidase | 0 | Not detected | 0.102 | -0.133 | 0.337 | 0.395 |
| ENZRXN0.4 | alkaline phosphatase aminop | 0 | Not detected | -0.043 | -0.212 | 0.125 | 0.613 |
| ENZRXN0.4 | peptidyl-dipeptidase | 0 | Not detected | -0.054 | -0.243 | 0.135 | 0.578 |
| ENZRXN0.4 | formate dehydrogenase | 0 | Not detected | -0.076 | -0.322 | 0.170 | 0.544 |
| ENZRXN0.4 | meso-diaminopimelic acid-D | 0 | Not detected | -0.071 | -0.261 | 0.120 | 0.467 |
| ENZRXN0.4 | exoribonuclease II | 0 | Not detected | 0.157 | -0.063 | 0.376 | 0.162 |
| ENZRXN0.5 | methionine-S-sulfoxide reduc | 0 | Not detected | -0.079 | -0.302 | 0.144 | 0.486 |
| ENZRXN0.5 | 5'-nucleotidase | 0 | Not detected | 0.049 | -0.127 | 0.225 | 0.587 |
| ENZRXN0.5 | 3'-nucleotidase | 0 | Not detected | 0.049 | -0.127 | 0.225 | 0.587 |
| ENZRXN0.5 | &gamma;-glutamylputrescine | 0 | Not detected | -0.117 | -0.294 | 0.060 | 0.194 |
| ENZRXN0.5 | &gamma;-glutamyl-&gamma | 0 | Not detected | -0.115 | -0.293 | 0.064 | 0.207 |
| ENZRXN0.5 | octanoul-ACP synthetase | 0 | Not detected | 0.029 | -0.182 | 0.241 | 0.787 |
| ENZRXN0.5 | 4-aminobutyrate aminotransf | 0 | Not detected | 0.024 | -0.159 | 0.206 | 0.797 |
| ENZRXN0.5 | endochitinase | 0 | Not detected | -0.022 | -0.239 | 0.196 | 0.846 |
| ENZRXN0.5 | serine/threonine:Na+ sympor | 0 | Not detected | 0.017 | -0.095 | 0.129 | 0.767 |
| ENZRXN0.5 | ribonuclease | 0 | Not detected | 0.051 | -0.108 | 0.209 | 0.529 |
| ENZRXN0.5 | RNase | 0 | Not detected | -0.002 | -0.289 | 0.286 | 0.991 |
| ENZRXN0.5 | methylglyoxal reductase | 1 | Not detected | -0.057 | -0.171 | 0.057 | 0.328 |
| ENZRXN0.5 | methylglyoxal reductase | 0 | Not detected | -0.124 | -0.393 | 0.146 | 0.368 |
| ENZRXN0.5 | Kdo2-lipid VIA phosphoetha | 0 | Not detected | -0.022 | -0.231 | 0.187 | 0.837 |
| ENZRXN0.5 | anhydro-N-acetylmuramic ac | 0 | Not detected | 0.067 | -0.113 | 0.247 | 0.465 |
| ENZRXN0.5 | RNase | 0 | Not detected | -0.021 | -0.262 | 0.220 | 0.866 |
| ENZRXN0.6 | DNA polymerase | 0 | Not detected | 0.098 | -0.157 | 0.353 | 0.451 |
| ENZRXN0.6 | 3' to 5' proofreading exonucle | 0 | Not detected | 0.051 | -0.119 | 0.222 | 0.553 |
| ENZRXN0.6 | DNA primase | 0 | Not detected | 0.058 | -0.168 | 0.283 | 0.618 |
| ENZRXN0.6 | DNA polymerase | 0 | Not detected | 0.026 | -0.191 | 0.242 | 0.815 |
| ENZRXN0.6 | DNA polymerase | 0 | Not detected | 0.026 | -0.191 | 0.242 | 0.815 |
| ENZRXN0.6 | peptidyl-prolyl cis-trans isom | 0 | Not detected | -0.024 | -0.210 | 0.162 | 0.798 |
| ENZRXN0.6 | cAMP phosphodiesterase | 0 | Not detected | 0.022 | -0.155 | 0.199 | 0.811 |
| ENZRXN0.6 | DNA polymerase | 0 | Not detected | 0.021 | -0.235 | 0.277 | 0.873 |
| ENZRXN0.6 | 3' to 5' proofreading exonucle | 0 | Not detected | 0.021 | -0.235 | 0.277 | 0.873 |
| ENZRXN0.6 | 5' to 3' exonuclease | 0 | Not detected | 0.021 | -0.235 | 0.277 | 0.873 |

| ENZRXN0.6 | ADP-heptose:Kdo2-lipid IV | 0 | Not detected | 0.163 | -0.024 | 0.351 | 0.088 |
| --- | --- | --- | --- | --- | --- | --- | --- |
| ENZRXN0.6 | ADP-heptose&mdash;LPS h | 0 | Not detected | 0.087 | -0.109 | 0.282 | 0.385 |
| ENZRXN0.6 | isopentenyl-adenosine tRNA | 0 | Not detected | 0.266 | 0.012 | 0.521 | 0.040 |
| ENZRXN0.6 | xanthine:H+ symporter XanP | 403 | Not detected | -0.134 | -0.278 | 0.010 | 0.069 |
| ENZRXN0.6 | 1-deoxy-D-xylulose 5-phosp | 0 | Not detected | -0.026 | -0.146 | 0.093 | 0.665 |
| ENZRXN0.6 | octanoate-non-lipoylated apo | 0 | Not detected | -0.008 | -0.166 | 0.150 | 0.921 |
| ENZRXN0.6 | 16S rRNA m5C1407 methylt | 0 | Not detected | -0.023 | -0.201 | 0.155 | 0.801 |
| ENZRXN0.6 | Î²-D-glucoside glucohydrolas | 0 | Not detected | 0.013 | -0.196 | 0.222 | 0.903 |
| ENZRXN0.6 | ADP-heptose:Kdo2-lipid A h | 0 | Not detected | 0.163 | -0.024 | 0.351 | 0.088 |
| ENZRXN0.6 | transport of D-serine | 0 | Not detected | -0.172 | -0.398 | 0.055 | 0.137 |
| ENZRXN0.6 | methylglyoxal reductase | 0 | Not detected | -0.189 | -0.470 | 0.092 | 0.187 |
| ENZRXN0.6 | 5-methylaminomethyl-2-thio | 0 | Not detected | -0.139 | -0.340 | 0.062 | 0.174 |
| ENZRXN0.6 | 2-isopropylmalate synthase | 0 | Not detected | -0.063 | -0.208 | 0.082 | 0.396 |
| ENZRXN0.6 | 6-phosphogluconate phospha | 0 | Not detected | -0.085 | -0.244 | 0.074 | 0.297 |
| ENZRXN0.6 | sugar phosphatase | 0 | Not detected | -0.064 | -0.244 | 0.117 | 0.489 |
| ENZRXN0.6 | pyridoxal phosphatase | 0 | Not detected | 0.016 | -0.152 | 0.183 | 0.852 |
| ENZRXN0.6 | phosphosugar phosphatase | 0 | Not detected | 0.016 | -0.152 | 0.183 | 0.852 |
| ENZRXN0.6 | 23S rRNA m2G1835 methylt | 0 | Not detected | -0.027 | -0.207 | 0.153 | 0.769 |
| ENZRXN0.6 | 23S rRNA m2G2445 methylt | 0 | Not detected | 0.080 | -0.149 | 0.309 | 0.492 |
| ENZRXN0.6 | 3 methyl-adenine DNA glyco | 0 | Not detected | -0.182 | -0.375 | 0.011 | 0.064 |
| ENZRXN0.6 | peptidyl-prolyl cis-trans isom | 0 | Not detected | -0.017 | -0.232 | 0.198 | 0.880 |
| ENZRXN0.6 | lysine 2,3-aminomutase | 0 | Not detected | -0.009 | -0.203 | 0.185 | 0.927 |
| ENZRXN0.6 | alanyl aminopeptidase | 0 | Not detected | 0.102 | -0.133 | 0.337 | 0.395 |
| ENZRXN0.6 | D-malate dehydrogenase (dec | 0 | Not detected | -0.263 | -0.496 | -0.030 | 0.027 |
| ENZRXN0.6 | poly(A) polymerase | 0 | Not detected | 0.028 | -0.170 | 0.226 | 0.781 |
| ENZRXN0.6 | 2-oxobutanoate aminotransfe | 1 | Not detected | -0.137 | -0.324 | 0.049 | 0.149 |
| ENZRXN0.6 | exodeoxyribonuclease I | 266 | Not detected | 0.226 | 0.025 | 0.428 | 0.028 |
| ENZRXN0.6 | transport of Î²-alanine | 0 | Not detected | -0.172 | -0.398 | 0.055 | 0.137 |
| ENZRXN0.6 | transport of L-alanine | 0 | Not detected | -0.172 | -0.398 | 0.055 | 0.137 |
| ENZRXN0.6 | transport of D-cycloserine | 0 | Not detected | -0.172 | -0.398 | 0.055 | 0.137 |
| ENZRXN0.6 | 3' &rarr; 5' exonuclease | 0 | Not detected | 0.055 | -0.108 | 0.218 | 0.508 |
| ENZRXN0.6 | Zn2+-exporting ATPase | 0 | Not detected | 0.054 | -0.172 | 0.281 | 0.639 |
| ENZRXN0.6 | S-ribosylhomocysteine lyase | 0 | Not detected | 0.180 | -0.008 | 0.368 | 0.061 |
| ENZRXN0.6 | D-gluconate transporter | 0 | Not detected | -0.139 | -0.328 | 0.051 | 0.151 |
| ENZRXN0.6 | high-affinity gluconate transp | 0 | Not detected | 0.062 | -0.172 | 0.296 | 0.602 |
| ENZRXN0.6 | 2-O-(6-phospho-Î±-mannosy | 0 | Not detected | 0.007 | -0.200 | 0.215 | 0.944 |
| ENZRXN0.6 | putrescine:H+ symporter | 0 | Not detected | 0.364 | 0.091 | 0.636 | 0.009 |
| ENZRXN0.6 | putrescine:L-ornithine antipo | 0 | Not detected | 0.364 | 0.091 | 0.636 | 0.009 |
| ENZRXN0.6 | cadaverine:H+ symporter | 0 | Not detected | 0.063 | -0.183 | 0.309 | 0.618 |
| ENZRXN0.6 | serine/threonine protein kinas | 0 | Not detected | 0.098 | -0.077 | 0.273 | 0.272 |
| ENZRXN0.6 | tRNA pseudouridine32 synth | 0 | Not detected | 0.092 | -0.083 | 0.267 | 0.301 |
| ENZRXN0.6 | transport of GlcNAc-1,6-anh | 0 | Not detected | 0.051 | -0.134 | 0.235 | 0.590 |
| ENZRXN0.6 | 1,6-anhydro-N-acetylmuramo | 0 | Not detected | -0.068 | -0.249 | 0.114 | 0.464 |
| ENZRXN0.6 | glcNAc-1,6-anhMurNAcami | 0 | Not detected | 0.036 | -0.151 | 0.222 | 0.706 |

| ENZRXN0.6 | galactonate:H+ symporter | 0 | Not detected | -0.001 | -0.194 | 0.192 | 0.992 |
| --- | --- | --- | --- | --- | --- | --- | --- |
| ENZRXN0.6 | L-galactonate oxidoreductase | 0 | Not detected | 0.000 | -0.180 | 0.181 | 0.999 |
| ENZRXN0.6 | transport of L-aspartate | 0 | Not detected | 0.251 | -0.005 | 0.507 | 0.054 |
| ENZRXN0.6 | succinate:fumarate antiporter | 0 | Not detected | -0.019 | -0.222 | 0.185 | 0.857 |
| ENZRXN0.6 | 16S rRNA m7G527 methyltr | 0 | Not detected | 0.107 | -0.067 | 0.282 | 0.228 |
| ENZRXN0.6 | transport of L-lyxose | 0 | Not detected | 0.015 | -0.161 | 0.192 | 0.865 |
| ENZRXN0.6 | L-idarate epimerase | 3 | Not detected | -0.283 | -0.528 | -0.039 | 0.023 |
| ENZRXN0.6 | biotin transporter | 0 | Not detected | 0.013 | -0.157 | 0.183 | 0.883 |
| ENZRXN0.6 | arbutin 6-phosphate glucohyd | 0 | Not detected | -0.214 | -0.461 | 0.033 | 0.090 |
| ENZRXN0.6 | arbutin 6-phosphate glucohyd | 0 | Not detected | -0.014 | -0.123 | 0.096 | 0.808 |
| ENZRXN0.6 | arbutin-6-phosphate glucohy | 0 | Not detected | -0.095 | -0.240 | 0.050 | 0.201 |
| ENZRXN0.6 | salicin 6-phosphate glucohyd | 0 | Not detected | -0.214 | -0.461 | 0.033 | 0.090 |
| ENZRXN0.6 | transport of N-acetyl-Î²-D-glu | 0 | Not detected | 0.051 | -0.134 | 0.235 | 0.590 |
| ENZRXN0.6 | 23S rRNA m2A2503 methylt | 0 | Not detected | 0.134 | -0.126 | 0.395 | 0.312 |
| ENZRXN0.6 | protein kinase | 0 | Not detected | 0.037 | -0.158 | 0.232 | 0.709 |
| ENZRXN0.6 | L-2-hydroxyglutarate dehydr | 0 | Not detected | -0.006 | -0.187 | 0.176 | 0.952 |
| ENZRXN0.6 | undecaprenyl-pyrophosphate | 0 | Not detected | -0.027 | -0.256 | 0.202 | 0.819 |
| ENZRXN0.6 | NAD(P)H:quinone oxidoredu | 0 | Not detected | -0.064 | -0.249 | 0.120 | 0.496 |
| ENZRXN0.6 | anthranilate phosphoribosyl t | 0 | Not detected | 0.056 | -0.140 | 0.251 | 0.577 |
| ENZRXN0.6 | nigerose hydrolase | 0 | Not detected | -0.110 | -0.316 | 0.096 | 0.294 |
| ENZRXN0.6 | peptidoglycan glycosyltransfe | 0 | Not detected | -0.112 | -0.321 | 0.098 | 0.297 |
| ENZRXN0.6 | peptidoglycan glycosyltransfe | 0 | Not detected | 0.075 | -0.111 | 0.262 | 0.429 |
| ENZRXN0.6 | murein transpeptidase | 0 | Not detected | 0.073 | -0.158 | 0.305 | 0.535 |
| ENZRXN0.6 | glycerol-2-phosphatase | 0 | Not detected | 0.034 | -0.168 | 0.237 | 0.739 |
| ENZRXN0.6 | L-glyceraldehyde 3-phosphat | 1 | Not detected | -0.057 | -0.171 | 0.057 | 0.328 |
| ENZRXN0.6 | poly-Î²-1,6-N-acetyl-D-gluco | 0 | Not detected | -0.035 | -0.227 | 0.158 | 0.723 |
| ENZRXN0.6 | tRNAMet cytidine acetyltran | 0 | Not detected | -0.107 | -0.310 | 0.095 | 0.299 |
| ENZRXN0.6 | ribosomal protein L11 methy | 0 | Not detected | 0.049 | -0.156 | 0.254 | 0.643 |
| ENZRXN0.6 | glutamyl-Q tRNA(Asp) synth | 0 | Not detected | -0.053 | -0.221 | 0.116 | 0.540 |
| ENZRXN0.6 | 3-hydroxypropionaldehyde d | 0 | Not detected | -0.115 | -0.293 | 0.064 | 0.207 |
| ENZRXN0.6 | lipid III flippase | 0 | Not detected | -0.059 | -0.255 | 0.136 | 0.551 |
| ENZRXN0.6 | glucose dehydrogenase | 0 | Not detected | -0.096 | -0.320 | 0.129 | 0.404 |
| ENZRXN0.6 | autoinducer-2 kinase | 0 | Not detected | -0.152 | -0.353 | 0.049 | 0.139 |
| ENZRXN0.6 | lipid II flippase | 0 | Not detected | -0.155 | -0.339 | 0.030 | 0.100 |
| ENZRXN0.6 | GTPase | 0 | Not detected | 0.012 | -0.197 | 0.220 | 0.912 |
| ENZRXN0.6 | transport of a dipeptide | 0 | Not detected | 0.071 | -0.128 | 0.270 | 0.484 |
| ENZRXN0.6 | acyl-[acp]:1-acyl-sn-glycerol | 0 | Not detected | -0.062 | -0.234 | 0.111 | 0.482 |
| ENZRXN0.6 | acyl-acp:sn-glycerol-3-phosp | 0 | Not detected | 0.220 | -0.024 | 0.465 | 0.078 |
| ENZRXN0.6 | 23S rRNA 2'-O-ribose C2498 | 0 | Not detected | 0.061 | -0.128 | 0.251 | 0.525 |
| ENZRXN0.7 | fructoselysine transporter | 0 | Not detected | -0.259 | -0.519 | 0.001 | 0.051 |
| ENZRXN0.7 | psicoselysine transporter | 0 | Not detected | -0.259 | -0.519 | 0.001 | 0.051 |
| ENZRXN0.7 | L-ribulose-5-phosphate 4-epi | 0 | Not detected | -0.021 | -0.139 | 0.097 | 0.726 |
| ENZRXN0.7 | L-ribulose-5-phosphate 3-epi | 0 | Not detected | -0.073 | -0.304 | 0.158 | 0.535 |
| ENZRXN0.7 | transport of a dipeptide | 0 | Not detected | -0.025 | -0.214 | 0.164 | 0.795 |

| ENZRXN0.7 | non-chaperonin molecular ch | 0 | Not detected | 0.197 | -0.054 | 0.448 | 0.124 |
| --- | --- | --- | --- | --- | --- | --- | --- |
| ENZRXN0.7 | protein methylthiotransferase | 0 | Not detected | 0.299 | 0.047 | 0.552 | 0.020 |
| ENZRXN0.7 | dipeptide transport | 0 | Not detected | 0.058 | -0.130 | 0.246 | 0.544 |
| ENZRXN0.7 | (S)-ureidoglycine aminohydr | 0 | Not detected | 0.057 | -0.167 | 0.282 | 0.616 |
| ENZRXN0.7 | chitobiose transport | 0 | Not detected | 0.013 | -0.168 | 0.194 | 0.885 |
| ENZRXN0.7 | transport of adenine | 0 | Not detected | -0.087 | -0.291 | 0.117 | 0.403 |
| ENZRXN0.7 | guanosine 3'-diphosphate 5'-t | 0 | Not detected | 0.045 | -0.209 | 0.300 | 0.727 |
| ENZRXN0.7 | GTPase | 0 | Not detected | 0.169 | -0.100 | 0.438 | 0.217 |
| ENZRXN0.7 | GTPase | 0 | Not detected | -0.141 | -0.354 | 0.072 | 0.195 |
| ENZRXN0.7 | carbamoyl--[HypE] ligase | 0 | Not detected | 0.039 | -0.187 | 0.265 | 0.736 |
| ENZRXN0.7 | 2-iminoacetate synthase | 0 | Not detected | -0.014 | -0.198 | 0.169 | 0.879 |
| ENZRXN0.7 | transport of malate | 0 | Not detected | 0.251 | -0.005 | 0.507 | 0.054 |
| ENZRXN0.7 | methionine-oxo-acid transam | 0 | Not detected | 0.120 | -0.059 | 0.299 | 0.188 |
| ENZRXN0.7 | p-aminobenzoyl glutamate:H | 0 | Not detected | 0.036 | -0.134 | 0.207 | 0.678 |
| ENZRXN0.7 | uracil oxygenase | 0 | Not detected | 0.044 | -0.122 | 0.210 | 0.605 |
| ENZRXN0.7 | RNA helicase | 0 | Not detected | -0.108 | -0.282 | 0.066 | 0.224 |
| ENZRXN0.7 | exoribonuclease II | 0 | Not detected | 0.157 | -0.063 | 0.376 | 0.162 |
| ENZRXN0.7 | peptidoglycan DD transpepti | 0 | Not detected | 0.095 | -0.130 | 0.319 | 0.410 |
| ENZRXN0.7 | L-aspartate oxidase | 0 | Not detected | -0.201 | -0.491 | 0.088 | 0.173 |
| ENZRXN0.7 | exoribonuclease II | 0 | Not detected | 0.157 | -0.063 | 0.376 | 0.162 |
| ENZRXN0.7 | adenosine nucleosidase | 0 | Not detected | 0.030 | -0.144 | 0.204 | 0.734 |
| ENZRXN0.7 | xanthosine nucleosidase | 0 | Not detected | 0.030 | -0.144 | 0.204 | 0.734 |
| ENZRXN0.7 | inosine nucleosidase | 0 | Not detected | 0.030 | -0.144 | 0.204 | 0.734 |
| ENZRXN0.7 | cytidine nucleosidase | 0 | Not detected | 0.030 | -0.144 | 0.204 | 0.734 |
| ENZRXN0.7 | dihydroorotate dehydrogenas | 0 | Not detected | 0.000 | -0.282 | 0.282 | 0.999 |
| ENZRXN0.7 | transport of 3-hydroxy-trans- | 0 | Not detected | -0.073 | -0.250 | 0.105 | 0.422 |
| ENZRXN0.7 | epoxyqueuosine reductase | 0 | Not detected | -0.005 | -0.189 | 0.180 | 0.961 |
| ENZRXN0.7 | putrescine:H+ symporter Pla | 0 | Not detected | 0.075 | -0.104 | 0.254 | 0.411 |
| ENZRXN0.7 | acyl-[acp]:1-acyl-sn-glycerol | 0 | Not detected | -0.062 | -0.234 | 0.111 | 0.482 |
| ENZRXN0.7 | putative inorganic phosphate | 0 | Not detected | -0.004 | -0.209 | 0.202 | 0.972 |
| ENZRXN0.7 | S-methyl-L-methionine trans | 0 | Not detected | 0.207 | -0.067 | 0.480 | 0.139 |
| ENZRXN0.7 | NADHX epimerase | 0 | Not detected | -0.086 | -0.287 | 0.115 | 0.401 |
| ENZRXN0.7 | (S)-NADHX dehydratase | 0 | Not detected | -0.086 | -0.287 | 0.115 | 0.401 |
| ENZRXN0.7 | transport of inosine | 0 | Not detected | 0.093 | -0.121 | 0.308 | 0.393 |
| ENZRXN0.7 | transport of deoxyinosine | 0 | Not detected | 0.093 | -0.121 | 0.308 | 0.393 |
| ENZRXN0.7 | 16S rRNA m2G1516 methylt | 0 | Not detected | 0.018 | -0.158 | 0.193 | 0.845 |
| ENZRXN0.7 | Î±-D-ribose-1-methylphosph | 0 | Not detected | -0.148 | -0.337 | 0.042 | 0.127 |
| ENZRXN0.7 | carbon-phosphorous lyase | 0 | Not detected | -0.248 | -0.467 | -0.029 | 0.026 |
| ENZRXN0.7 | peptidoglycan DD-carboxype | 0 | Not detected | -0.056 | -0.234 | 0.123 | 0.542 |
| ENZRXN0.7 | peptidoglycan DD-endopepti | 0 | Not detected | -0.056 | -0.234 | 0.123 | 0.542 |
| ENZRXN0.7 | ferric enterobactin esterase | 0 | Not detected | -0.105 | -0.279 | 0.069 | 0.236 |
| ENZRXN0.7 | (S)-lactate transporter | 0 | Not detected | -0.227 | -0.474 | 0.021 | 0.073 |
| ENZRXN0.7 | 23S rRNA m7G2069 methylt | 0 | Not detected | 0.080 | -0.149 | 0.309 | 0.492 |
| ENZRXN0.7 | enterobactin exporter EntS | 0 | Not detected | -0.073 | -0.258 | 0.112 | 0.439 |

| ENZRXN0.7 | D-tartrate:succinate antiporte | 0 | Not detected | 0.119 | -0.090 | 0.328 | 0.264 |
| --- | --- | --- | --- | --- | --- | --- | --- |
| ENZRXN0.7 | GTPase | 0 | Not detected | 0.006 | -0.236 | 0.248 | 0.963 |
| ENZRXN0.7 | fructose-6-phosphate:phosph | 0 | Not detected | -0.119 | -0.316 | 0.078 | 0.237 |
| ENZRXN0.7 | mannose-6-phosphate:phosph | 0 | Not detected | -0.119 | -0.316 | 0.078 | 0.237 |
| ENZRXN0.7 | propionate transporter | 0 | Not detected | 0.168 | -0.086 | 0.422 | 0.195 |
| ENZRXN0.7 | metalloprotease | 0 | Not detected | 0.021 | -0.157 | 0.199 | 0.818 |
| ENZRXN0.7 | (R)-lactate transporter | 0 | Not detected | -0.143 | -0.358 | 0.073 | 0.195 |
| ENZRXN0.7 | glycolate transporter | 0 | Not detected | -0.143 | -0.358 | 0.073 | 0.195 |
| ENZRXN0.7 | (R)-lactate transporter | 0 | Not detected | -0.227 | -0.474 | 0.021 | 0.073 |
| ENZRXN0.7 | transport of a C4-dicarboxyla | 0 | Not detected | 0.251 | -0.005 | 0.507 | 0.054 |
| ENZRXN0.7 | methyl Î²-D-glucoside 6-phos | 0 | Not detected | -0.014 | -0.123 | 0.096 | 0.808 |
| ENZRXN0.7 | D-galactarate transporter | 0 | Not detected | 0.053 | -0.129 | 0.235 | 0.567 |
| ENZRXN0.7 | D-glucarate transporter | 0 | Not detected | 0.053 | -0.129 | 0.235 | 0.567 |
| ENZRXN0.7 | D-glycerate transporter | 0 | Not detected | 0.053 | -0.129 | 0.235 | 0.567 |
| ENZRXN0.7 | propionyl-CoA synthetase | 0 | Not detected | 0.010 | -0.189 | 0.209 | 0.922 |
| ENZRXN0.7 | 23S rRNA m6A2030 methylt | 0 | Not detected | -0.032 | -0.210 | 0.147 | 0.727 |
| ENZRXN0.7 | tRNA m2A37 methyltransfer | 0 | Not detected | 0.134 | -0.126 | 0.395 | 0.312 |
| ENZRXN0.7 | inorganic triphosphatase | 0 | Not detected | 0.062 | -0.123 | 0.248 | 0.509 |
| ENZRXN0.7 | L-glyceraldehyde-3-phosphat | 0 | Not detected | -0.119 | -0.316 | 0.078 | 0.237 |
| ENZRXN0.7 | 6-phospho-Î²-D-glucosyl-(1,4 | 0 | Not detected | -0.095 | -0.240 | 0.050 | 0.201 |
| ENZRXN0.7 | aldehyde reductase | 0 | Not detected | -0.013 | -0.192 | 0.165 | 0.883 |
| ENZRXN0.7 | cysteinylglycine dipeptidase | 0 | Not detected | 0.102 | -0.133 | 0.337 | 0.395 |
| ENZRXN0.7 | UDP-N-acetylmuramate:L-al | 0 | Not detected | 0.218 | -0.039 | 0.475 | 0.096 |
| ENZRXN0.7 | 6-phospho-Î²-D-glucosyl-(1-4 | 0 | Not detected | -0.214 | -0.461 | 0.033 | 0.090 |
| ENZRXN0.7 | guanosine 2'3'-cyclic phosph | 0 | Not detected | -0.004 | -0.237 | 0.229 | 0.974 |
| ENZRXN0.7 | uridine 2'3'-cyclic phosphodi | 0 | Not detected | -0.004 | -0.237 | 0.229 | 0.974 |
| ENZRXN0.7 | cytidine 2'3'-cyclic phosphod | 0 | Not detected | -0.004 | -0.237 | 0.229 | 0.974 |
| ENZRXN0.7 | adenosine 2'3'-cyclic phospho | 0 | Not detected | -0.004 | -0.237 | 0.229 | 0.974 |
| ENZRXN0.7 | cytidine 3'-phosphate phosph | 0 | Not detected | 0.049 | -0.127 | 0.225 | 0.587 |
| ENZRXN0.7 | adenosine 3'-phosphate phos | 0 | Not detected | 0.049 | -0.127 | 0.225 | 0.587 |
| ENZRXN0.7 | dGMP 5'-phosphate phospho | 0 | Not detected | 0.049 | -0.127 | 0.225 | 0.587 |
| ENZRXN0.7 | arsenate:H+ symporter | 0 | Not detected | 0.097 | -0.108 | 0.302 | 0.355 |
| ENZRXN0.7 | succinate transporter | 0 | Not detected | 0.054 | -0.154 | 0.262 | 0.610 |
| ENZRXN0.7 | fumarate transporter | 0 | Not detected | 0.054 | -0.154 | 0.262 | 0.610 |
| ENZRXN0.7 | aspartate transport | 0 | Not detected | 0.054 | -0.154 | 0.262 | 0.610 |
| ENZRXN0.7 | amylomaltase | 0 | Not detected | -0.001 | -0.226 | 0.225 | 0.995 |
| ENZRXN0.7 | amylomaltase | 0 | Not detected | -0.001 | -0.226 | 0.225 | 0.995 |
| ENZRXN0.7 | maltopentaose glucosidase | 0 | Not detected | -0.011 | -0.209 | 0.187 | 0.911 |
| ENZRXN0.7 | maltohexaose glucosidase | 0 | Not detected | -0.011 | -0.209 | 0.187 | 0.911 |
| ENZRXN0.7 | maltoheptaose glucosidase | 0 | Not detected | -0.011 | -0.209 | 0.187 | 0.911 |
| ENZRXN0.7 | GTPase | 0 | Not detected | -0.019 | -0.192 | 0.155 | 0.834 |
| ENZRXN0.7 | phosphoethanolamine transfe | 0 | Not detected | 0.001 | -0.196 | 0.199 | 0.991 |
| ENZRXN0.7 | phosphoethanolamine transfe | 0 | Not detected | -0.156 | -0.354 | 0.042 | 0.123 |
| ENZRXN0.7 | cytidine 3' nucleotidase | 0 | Not detected | -0.004 | -0.237 | 0.229 | 0.974 |

| ENZRXN0.7 | guanosine 3' nucleotidase | 0 | Not detected | -0.004 | -0.237 | 0.229 | 0.974 |
| --- | --- | --- | --- | --- | --- | --- | --- |
| ENZRXN0.7 | adenosine 3' nucleotidase | 0 | Not detected | -0.004 | -0.237 | 0.229 | 0.974 |
| ENZRXN0.7 | uridine 3' nucleotidase | 0 | Not detected | -0.004 | -0.237 | 0.229 | 0.974 |
| ENZRXN0.7 | 5'-deoxyribonucleotidase | 0 | Not detected | 0.063 | -0.158 | 0.284 | 0.575 |
| ENZRXN0.7 | 2-octaprenylphenol hydroxyl | 0 | Not detected | 0.001 | -0.178 | 0.180 | 0.990 |
| ENZRXN0.7 | DNA-AP site lyase | 0 | Not detected | 0.020 | -0.245 | 0.284 | 0.884 |
| ENZRXN0.7 | transport of D-glucose | 0 | Not detected | -0.018 | -0.204 | 0.168 | 0.849 |
| ENZRXN0.7 | AMP kinase (GTP) | 4 | Not detected | 0.042 | -0.078 | 0.163 | 0.491 |
| ENZRXN0.7 | protein-lysine deacetylase | 0 | Not detected | 0.036 | -0.165 | 0.238 | 0.724 |
| ENZRXN0.7 | transport of glucose | 0 | Not detected | -0.056 | -0.250 | 0.138 | 0.571 |
| ENZRXN0.7 | transport of maltose | 0 | Not detected | -0.056 | -0.250 | 0.138 | 0.571 |
| ENZRXN0.7 | guanine transport | 0 | Not detected | 0.113 | -0.096 | 0.322 | 0.290 |
| ENZRXN0.7 | hypoxanthine transport | 0 | Not detected | 0.113 | -0.096 | 0.322 | 0.290 |
| ENZRXN0.7 | 5-aminomethyl-2-thiouridine | 0 | Not detected | -0.139 | -0.340 | 0.062 | 0.174 |
| ENZRXN0.7 | phosphoethanolamine transfe | 0 | Not detected | -0.046 | -0.233 | 0.141 | 0.629 |
| ENZRXN0.7 | 6-deoxy-6-sulfofructose kina | 0 | Not detected | -0.124 | -0.300 | 0.051 | 0.166 |
| ENZRXN0.7 | 6-deoxy-6-sulfofructose-1-ph | 0 | Not detected | -0.089 | -0.266 | 0.087 | 0.320 |
| ENZRXN0.7 | sulfoquinovose transporter | 0 | Not detected | -0.008 | -0.211 | 0.195 | 0.938 |
| ENZRXN0.7 | uracil:H+ symporter | 0 | Not detected | 0.046 | -0.211 | 0.303 | 0.727 |
| ENZRXN0.7 | transport of a tripeptide | 0 | Not detected | 0.071 | -0.128 | 0.270 | 0.484 |
| ENZRXN0.7 | transport of a tripeptide | 0 | Not detected | -0.025 | -0.214 | 0.164 | 0.795 |
| ENZRXN0.7 | L-threonine dehydrogenase | 0 | Not detected | 0.017 | -0.243 | 0.277 | 0.897 |
| ENZRXN0.7 | 11-deoxycorticosterone reduc | 0 | Not detected | 0.050 | -0.132 | 0.233 | 0.589 |
| ENZRXN0.7 | D-gluconate 5-dehydrogenas | 0 | Not detected | 0.050 | -0.132 | 0.233 | 0.589 |
| ENZRXN0.7 | 2-keto-3-deoxy-D-gluconate | 0 | Not detected | 0.050 | -0.132 | 0.233 | 0.589 |
| ENZRXN0.7 | sodium dodecyl sulfatase | 0 | Not detected | -0.111 | -0.319 | 0.097 | 0.296 |
| ENZRXN0.7 | isobutyraldehyde reductase | 0 | Not detected | -0.104 | -0.355 | 0.146 | 0.414 |
| ENZRXN0.7 | 1-hydroxy-2-methyl-2-(E)-bu | 1 | Not detected | -0.089 | -0.204 | 0.027 | 0.132 |
| ENZRXN0.7 | 3' &rarr; 5' ssDNA exonuclea | 0 | Not detected | -0.060 | -0.223 | 0.104 | 0.476 |
| ENZRXN0.7 | 3' &rarr; 5' RNA exonuclease | 0 | Not detected | -0.060 | -0.223 | 0.104 | 0.476 |
| ENZRXN0.7 | ATP pyrophosphatase | 0 | Not detected | 0.145 | -0.072 | 0.362 | 0.192 |
| ENZRXN0.7 | 3-isopropylmalate dehydroge | 0 | Not detected | -0.263 | -0.496 | -0.030 | 0.027 |
| ENZRXN0.7 | L-tartrate dehydrogenase | 0 | Not detected | -0.263 | -0.496 | -0.030 | 0.027 |
| ENZRXN0.7 | glycerophosphoglycerol acylt | 0 | Not detected | -0.059 | -0.236 | 0.119 | 0.515 |
| ENZRXN0.7 | NADH:menaquinone oxidore | 0 | Not detected | 0.045 | -0.147 | 0.237 | 0.645 |
| ENZRXN0.7 | polyphenol oxidase | 0 | Not detected | 0.084 | -0.088 | 0.255 | 0.339 |
| ENZRXN0.7 | PII-UMP deuridylylation | 0 | Not detected | -0.044 | -0.266 | 0.178 | 0.699 |
| ENZRXN0.7 | D-ribulose 5-phosphate 1-deh | 0 | Not detected | -0.214 | -0.428 | 0.001 | 0.051 |
| ENZRXN0.7 | flavin mononucleotide (FMN | 0 | Not detected | 0.027 | -0.148 | 0.202 | 0.762 |
| ENZRXN0.7 | flavin adenine dinucleotide ( | 0 | Not detected | 0.027 | -0.148 | 0.202 | 0.762 |
| ENZRXN0.7 | L,D-carboxypeptidase | 0 | Not detected | -0.014 | -0.208 | 0.180 | 0.885 |
| ENZRXN0.7 | L,D-transpeptidase | 0 | Not detected | -0.014 | -0.208 | 0.180 | 0.885 |
| ENZRXN0.7 | L,D transpeptidase | 0 | Not detected | -0.014 | -0.195 | 0.168 | 0.881 |
| ENZRXN0.7 | L,D-transpeptidase | 0 | Not detected | -0.014 | -0.208 | 0.180 | 0.885 |

| ENZRXN0.7 | 5' &rarr; 3' exonuclease | 0 | Not detected | 0.121 | -0.045 | 0.286 | 0.153 |
| --- | --- | --- | --- | --- | --- | --- | --- |
| ENZRXN0.7 | 3',5'-nucleoside bisphosphate | 0 | Not detected | 0.121 | -0.045 | 0.286 | 0.153 |
| ENZRXN0.7 | ATPase | 0 | Not detected | -0.006 | -0.121 | 0.110 | 0.925 |
| ENZRXN0.7 | acyl-acp:sn-glycerol-3-phosp | 0 | Not detected | 0.220 | -0.024 | 0.465 | 0.078 |
| ENZRXN0.7 | acyl-acp:sn-glycerol-3-phosp | 0 | Not detected | 0.220 | -0.024 | 0.465 | 0.078 |
| ENZRXN0.7 | acyl-acp:sn-glycerol-3-phosp | 0 | Not detected | 0.220 | -0.024 | 0.465 | 0.078 |
| ENZRXN0.7 | acyl-[acp]:1-acyl-sn-glycerol | 0 | Not detected | -0.062 | -0.234 | 0.111 | 0.482 |
| ENZRXN0.7 | acyl-[acp]:1-acyl-sn-glycerol | 0 | Not detected | -0.062 | -0.234 | 0.111 | 0.482 |
| ENZRXN0.7 | acyl-[acp]:1-acyl-sn-glycerol | 0 | Not detected | -0.062 | -0.234 | 0.111 | 0.482 |
| ENZRXN0.7 | acyl-[acp]:1-acyl-sn-glycerol | 0 | Not detected | -0.062 | -0.234 | 0.111 | 0.482 |
| ENZRXN0.7 | acyl-[acp]:1-acyl-sn-glycerol | 0 | Not detected | -0.062 | -0.234 | 0.111 | 0.482 |
| ENZRXN0.8 | magnesium hydrogenphospha | 0 | Not detected | 0.097 | -0.108 | 0.302 | 0.355 |
| ENZRXN0.8 | calcium hydrogenphosphate: | 0 | Not detected | 0.097 | -0.108 | 0.302 | 0.355 |
| ENZRXN0.8 | zinc hydrogenphosphate:H+ | 0 | Not detected | 0.097 | -0.108 | 0.302 | 0.355 |
| ENZRXN0.8 | tellurite:H+ symporter | 0 | Not detected | 0.097 | -0.108 | 0.302 | 0.355 |
| ENZRXN0.8 | L-methionine:H+ antiport | 0 | Not detected | 0.026 | -0.158 | 0.211 | 0.780 |
| ENZRXN0.8 | L-leucine:H+ antiport | 0 | Not detected | 0.026 | -0.158 | 0.211 | 0.780 |
| ENZRXN0.8 | L-isoleucine:H+ antiport | 0 | Not detected | 0.026 | -0.158 | 0.211 | 0.780 |
| ENZRXN0.8 | L-valine:H+ antiport | 0 | Not detected | 0.026 | -0.158 | 0.211 | 0.780 |
| ENZRXN0.8 | farnesyl diphosphate phospha | 0 | Not detected | 0.022 | -0.185 | 0.229 | 0.836 |
| ENZRXN0.8 | L-cystine:H+ symporter | 0 | Not detected | 0.138 | -0.052 | 0.329 | 0.155 |
| ENZRXN0.8 | D-cystine:H+ symporter | 0 | Not detected | 0.138 | -0.052 | 0.329 | 0.155 |
| ENZRXN0.8 | homocystine:H+ symporter | 0 | Not detected | 0.138 | -0.052 | 0.329 | 0.155 |
| ENZRXN0.8 | apolipoprotein N-acyltransfer | 0 | Not detected | -0.049 | -0.236 | 0.138 | 0.611 |
| ENZRXN0.8 | peptidoglycan lytic exotransg | 0 | Not detected | -0.008 | -0.183 | 0.167 | 0.928 |
| ENZRXN0.8 | peptidoglycan lytic exotransg | 0 | Not detected | -0.074 | -0.243 | 0.096 | 0.396 |
| ENZRXN0.8 | peptidoglycan lytic exotransg | 0 | Not detected | -0.055 | -0.257 | 0.147 | 0.593 |
| ENZRXN0.8 | peptidoglycan lytic endotrans | 0 | Not detected | -0.055 | -0.257 | 0.147 | 0.593 |
| ENZRXN0.8 | peptidoglycan lytic endotrans | 0 | Not detected | -0.074 | -0.243 | 0.096 | 0.396 |
| ENZRXN0.8 | peptidoglycan lytic exotransg | 0 | Not detected | 0.096 | -0.085 | 0.277 | 0.300 |
| ENZRXN0.8 | peptidoglycan lytic endotrans | 0 | Not detected | 0.096 | -0.085 | 0.277 | 0.300 |
| ENZRXN0.8 | peptidoglycan lytic endotrans | 0 | Not detected | -0.113 | -0.322 | 0.096 | 0.290 |
| ENZRXN0.8 | peptidoglycan lytic exotransg | 0 | Not detected | -0.113 | -0.322 | 0.096 | 0.290 |
| ENZRXN0.8 | peptidoglycan lytic exotransg | 0 | Not detected | -0.066 | -0.260 | 0.128 | 0.507 |
| ENZRXN0.8 | GTPase, ribosome-stimulated | 0 | Not detected | -0.011 | -0.125 | 0.102 | 0.844 |
| ENZRXN0.8 | transport of 2-acyl-1-lysopho | 0 | Not detected | -0.120 | -0.300 | 0.061 | 0.194 |
| ENZRXN0.8 | transport of lyso-phosphatidy | 0 | Not detected | -0.120 | -0.300 | 0.061 | 0.194 |
| ENZRXN0.8 | biotin-ATP ligase | 0 | Not detected | -0.053 | -0.228 | 0.123 | 0.557 |
| ENZRXN0.8 | sulfoquinovosidase | 0 | Not detected | -0.202 | -0.496 | 0.092 | 0.177 |
| ENZRXN0.8 | sulfoquinovosidase | 0 | Not detected | -0.202 | -0.496 | 0.092 | 0.177 |
| ENZRXN0.8 | (S)-2-hydroxybutanoate dehy | 0 | Not detected | 0.147 | -0.061 | 0.356 | 0.165 |
| ENZRXN0.8 | (S)-2-hydroxybutanoate trans | 0 | Not detected | -0.143 | -0.358 | 0.073 | 0.195 |
| ENZRXN0.8 | (S)-2-hydroxybutanoate trans | 0 | Not detected | -0.227 | -0.474 | 0.021 | 0.073 |
| ENZRXN0.8 | choline dehydrogenase | 0 | Not detected | -0.230 | -0.438 | -0.022 | 0.030 |

| ENZRXN0.8 | betaine aldehyde dehydrogen | 0 | Not detected | -0.230 | -0.438 | -0.022 | 0.030 |
| --- | --- | --- | --- | --- | --- | --- | --- |
| ENZRXN0.8 | short chain acyl-CoA synthet | 0 | Not detected | 0.046 | -0.182 | 0.274 | 0.694 |
| ENZRXN0.8 | cis-vaccenoyl-CoA synthetas | 0 | Not detected | 0.046 | -0.182 | 0.274 | 0.694 |
| ENZRXN0.8 | oleoyl-CoA synthetase (trans | 0 | Not detected | 0.046 | -0.182 | 0.274 | 0.694 |
| ENZRXN0.8 | inositol monophosphatase | 0 | Not detected | 0.034 | -0.168 | 0.237 | 0.739 |
| ENZRXN0.8 | fumarate:H+ symporter | 0 | Not detected | 0.119 | -0.090 | 0.328 | 0.264 |
| ENZRXN0.8 | fumarate:H+ symporter | 0 | Not detected | -0.019 | -0.222 | 0.185 | 0.857 |
| ENZRXN0.8 | acyl-CoA dehydrogenase | 0 | Not detected | -0.104 | -0.323 | 0.114 | 0.349 |
| ENZRXN0.8 | acyl-CoA dehydrogenase | 0 | Not detected | -0.104 | -0.323 | 0.114 | 0.349 |
| ENZRXN0.8 | acyl-CoA dehydrogenase | 0 | Not detected | -0.104 | -0.323 | 0.114 | 0.349 |
| ENZRXN0.8 | acyl-CoA dehydrogenase | 0 | Not detected | -0.104 | -0.323 | 0.114 | 0.349 |
| ENZRXN0.8 | acyl-CoA dehydrogenase | 0 | Not detected | -0.104 | -0.323 | 0.114 | 0.349 |
| ENZRXN0.8 | acyl-CoA dehydrogenase | 0 | Not detected | -0.104 | -0.323 | 0.114 | 0.349 |
| ENZRXN0.8 | acyl-CoA dehydrogenase | 0 | Not detected | -0.104 | -0.323 | 0.114 | 0.349 |
| ENZRXN0.8 | enoyl-CoA hydratase | 0 | Not detected | -0.107 | -0.311 | 0.097 | 0.304 |
| ENZRXN0.8 | enoyl-CoA hydratase | 0 | Not detected | -0.107 | -0.311 | 0.097 | 0.304 |
| ENZRXN0.8 | enoyl-CoA hydratase | 0 | Not detected | -0.107 | -0.311 | 0.097 | 0.304 |
| ENZRXN0.8 | enoyl-CoA hydratase | 0 | Not detected | -0.107 | -0.311 | 0.097 | 0.304 |
| ENZRXN0.8 | enoyl-CoA hydratase | 0 | Not detected | -0.107 | -0.311 | 0.097 | 0.304 |
| ENZRXN0.8 | enoyl-CoA hydratase | 0 | Not detected | -0.107 | -0.311 | 0.097 | 0.304 |
| ENZRXN0.8 | enoyl-CoA hydratase | 0 | Not detected | -0.107 | -0.311 | 0.097 | 0.304 |
| ENZRXN0.8 | 3-hydroxyacyl-CoA dehydro | 0 | Not detected | -0.107 | -0.311 | 0.097 | 0.304 |
| ENZRXN0.8 | 3-hydroxyacyl-CoA dehydro | 0 | Not detected | -0.107 | -0.311 | 0.097 | 0.304 |
| ENZRXN0.8 | 3-hydroxyacyl-CoA dehydro | 0 | Not detected | -0.107 | -0.311 | 0.097 | 0.304 |
| ENZRXN0.8 | 3-hydroxyacyl-CoA dehydro | 0 | Not detected | -0.107 | -0.311 | 0.097 | 0.304 |
| ENZRXN0.8 | 3-hydroxyacyl-CoA dehydro | 0 | Not detected | -0.107 | -0.311 | 0.097 | 0.304 |
| ENZRXN0.8 | 3-hydroxyacyl-CoA dehydro | 0 | Not detected | -0.107 | -0.311 | 0.097 | 0.304 |
| ENZRXN0.8 | 3-hydroxyacyl-CoA dehydro | 0 | Not detected | -0.107 | -0.311 | 0.097 | 0.304 |
| ENZRXN0.8 | acetyl-CoA C-acyltransferase | 0 | Not detected | 0.001 | -0.181 | 0.183 | 0.993 |
| ENZRXN0.8 | acetyl-CoA C-acyltransferase | 0 | Not detected | 0.001 | -0.181 | 0.183 | 0.993 |
| ENZRXN0.8 | acetyl-CoA C-acyltransferase | 0 | Not detected | 0.001 | -0.181 | 0.183 | 0.993 |
| ENZRXN0.8 | acetyl-CoA C-acyltransferase | 0 | Not detected | 0.001 | -0.181 | 0.183 | 0.993 |
| ENZRXN0.8 | acetyl-CoA C-acyltransferase | 0 | Not detected | 0.001 | -0.181 | 0.183 | 0.993 |
| ENZRXN0.8 | acetyl-CoA C-acyltransferase | 0 | Not detected | 0.001 | -0.181 | 0.183 | 0.993 |
| ENZRXN0.8 | acetyl-CoA C-acyltransferase | 0 | Not detected | 0.001 | -0.181 | 0.183 | 0.993 |
| ENZRXN0.8 | palmitoleoyl-CoA synthetase | 0 | Not detected | 0.046 | -0.182 | 0.274 | 0.694 |
| ENZRXN0.8 | fumarate:H+ symport | 0 | Not detected | 0.139 | -0.104 | 0.383 | 0.262 |
| ENZRXN0.8 | succinate:H+ symporter | 0 | Not detected | -0.019 | -0.222 | 0.185 | 0.857 |
| ENZRXN0.8 | methylphosphotriester-DNA- | 0 | Not detected | -0.167 | -0.338 | 0.005 | 0.057 |
| ENZRXN0.8 | O-4-methylthymine-DNA me | 0 | Not detected | -0.167 | -0.338 | 0.005 | 0.057 |
| ENZRXN0.8 | 2,6-dimethoxyphenol:oxygen | 0 | Not detected | -0.099 | -0.292 | 0.094 | 0.316 |
| ENZRXN0.8 | 4,5-DOPA dioxygenase extra | 0 | Not detected | -0.123 | -0.282 | 0.037 | 0.132 |
| ENZRXN0.8 | ssDNA-specific exonuclease | 0 | Not detected | 0.090 | -0.134 | 0.315 | 0.430 |
| ENZRXN0.8 | nitrophenyl phosphatase | 0 | Not detected | 0.063 | -0.158 | 0.284 | 0.575 |

| ENZRXN0.8 | diguanylate cyclase | 0 | Not detected | -0.081 | -0.252 | 0.091 | 0.356 |
| --- | --- | --- | --- | --- | --- | --- | --- |
| ENZRXN0.8 | P1,P3-bis(5'-adenosyl) tripho | 0 | Not detected | 0.063 | -0.158 | 0.284 | 0.575 |
| ENZRXN0.8 | CDP-glycerol hydrolase | 0 | Not detected | 0.063 | -0.158 | 0.284 | 0.575 |
| ENZRXN0.8 | tRNATyr pseudouridine35 sy | 0 | Not detected | -0.032 | -0.189 | 0.125 | 0.689 |
| ENZRXN0.8 | GTPase | 0 | Not detected | 0.137 | -0.074 | 0.348 | 0.204 |
| ENZRXN0.8 | recombination-promoting nu | 0 | Not detected | -0.069 | -0.288 | 0.151 | 0.540 |
| ENZRXN0.8 | S-sulfocysteine:H+ symporte | 0 | Not detected | 0.138 | -0.052 | 0.329 | 0.155 |
| ENZRXN0.8 | periplasmic nitrite reductase | 0 | Not detected | 0.212 | -0.002 | 0.425 | 0.052 |
| ENZRXN0.8 | signal peptide peptidase | 0 | Not detected | 0.137 | -0.074 | 0.348 | 0.202 |
| ENZRXN0.8 | lipoprotein signal peptide pep | 0 | Not detected | 0.137 | -0.074 | 0.348 | 0.202 |
| ENZRXN0.8 | L-cysteine desulfidase | 0 | Not detected | 0.202 | -0.002 | 0.407 | 0.053 |
| ENZRXN0.8 | rhomboid protease | 0 | Not detected | -0.055 | -0.220 | 0.110 | 0.512 |
| ENZRXN0.8 | 3' to 5' ATP-dependent DNA | 0 | Not detected | -0.177 | -0.420 | 0.067 | 0.155 |
| ENZRXN0.8 | 5' to 3' ATP-dependent DNA | 0 | Not detected | 0.035 | -0.173 | 0.242 | 0.744 |
| ENZRXN0.8 | cytochrome c peroxidase | 0 | Not detected | -0.086 | -0.306 | 0.134 | 0.445 |
| ENZRXN0.8 | Î²-barrel assembly-enhancing | 0 | Not detected | -0.057 | -0.250 | 0.135 | 0.558 |
| ENZRXN0.8 | 2-acyl lysophospholipase | 0 | Not detected | -0.059 | -0.236 | 0.119 | 0.515 |
| ENZRXN0.8 | 1-acyl lysophospholipase | 0 | Not detected | -0.059 | -0.236 | 0.119 | 0.515 |
| ENZRXN0.8 | pyruvate:H+ symporter | 0 | Not detected | -0.108 | -0.357 | 0.140 | 0.393 |
| ENZRXN0.8 | Na+ : H+ antiporter | 0 | Not detected | -0.012 | -0.200 | 0.176 | 0.900 |
| ENZRXN0.8 | K+ : H+ antiporter | 0 | Not detected | -0.012 | -0.200 | 0.176 | 0.900 |
| ENZRXN0.8 | glucosylglycerate phosphoryl | 0 | Not detected | -0.093 | -0.274 | 0.089 | 0.316 |
| ENZRXN0.8 | carbonylcyanide m-chlorophe | 0 | Not detected | -0.044 | -0.229 | 0.140 | 0.637 |
| ENZRXN0.8 | bicozamycin:H+ antiporter | 0 | Not detected | 0.085 | -0.086 | 0.257 | 0.329 |
| ENZRXN0.8 | chloramphenicol:H+ antiport | 0 | Not detected | -0.081 | -0.273 | 0.111 | 0.408 |
| ENZRXN0.8 | fosfomycin:H+ antiporter | 0 | Not detected | 0.137 | -0.045 | 0.319 | 0.141 |
| ENZRXN0.8 | Fe2+ transporter | 0 | Not detected | -0.138 | -0.366 | 0.090 | 0.235 |
| ENZRXN0.8 | trimethylamine N-oxide redu | 0 | Not detected | -0.040 | -0.243 | 0.162 | 0.697 |
| ENZRXN0.8 | trimethylamine N-oxide redu | 0 | Not detected | -0.097 | -0.294 | 0.101 | 0.338 |
| ENZRXN0.8 | UDP kinase | 4 | Not detected | 0.042 | -0.078 | 0.163 | 0.491 |
| ENZRXN0.8 | dTDP kinase | 4 | Not detected | 0.042 | -0.078 | 0.163 | 0.491 |
| ENZRXN0.8 | CDP kinase | 4 | Not detected | 0.042 | -0.078 | 0.163 | 0.491 |
| ENZRXN0.8 | dCDP kinase | 4 | Not detected | 0.042 | -0.078 | 0.163 | 0.491 |
| ENZRXN0.8 | dGDP kinase | 4 | Not detected | 0.042 | -0.078 | 0.163 | 0.491 |
| ENZRXN0.8 | GDP kinase | 4 | Not detected | 0.042 | -0.078 | 0.163 | 0.491 |
| ENZRXN0.8 | celulose phosphoethanolamin | 0 | Not detected | -0.029 | -0.232 | 0.175 | 0.782 |
| ENZRXN0.8 | thymine:H+ symporter | 0 | Not detected | 0.046 | -0.211 | 0.303 | 0.727 |
| ENZRXN0.8 | xanthine:H+ symporter | 0 | Not detected | 0.046 | -0.211 | 0.303 | 0.727 |
| ENZRXN0.8 | c-di-GMP phosphodiesterase | 0 | Not detected | -0.013 | -0.207 | 0.182 | 0.898 |
| ENZRXN0.8 | apolipoprotein N-acyltransfer | 0 | Not detected | -0.049 | -0.236 | 0.138 | 0.611 |
| ENZRXN0.8 | hydroquinone:hydrogen pero | 0 | Not detected | -0.086 | -0.306 | 0.134 | 0.445 |
| ENZRXN0.8 | menadiol:hydrogen peroxide | 0 | Not detected | -0.086 | -0.306 | 0.134 | 0.445 |
| ENZRXN0.8 | kojibiose phosphorylase | 0 | Not detected | -0.071 | -0.267 | 0.125 | 0.476 |
| ENZRXN0.8 | endo-poly-Î²-1,6-D-glucosam | 0 | Not detected | -0.035 | -0.227 | 0.158 | 0.723 |

| ENZRXN0.8 | L-aspartate:fumarate antiport | 0 | Not detected | 0.139 | -0.104 | 0.383 | 0.262 |
| --- | --- | --- | --- | --- | --- | --- | --- |
| ENZRXN0.8 | thiol-disulfide exchange | 0 | Not detected | -0.021 | -0.231 | 0.189 | 0.844 |
| ENZRXN0.8 | ferric enterobactin esterase | 0 | Not detected | -0.105 | -0.279 | 0.069 | 0.236 |
| ENZRXN0.8 | thiol-disulfide exchange | 0 | Not detected | -0.021 | -0.231 | 0.189 | 0.844 |
| ENZRXN0.8 | 5-aminovalerate aminotransfe | 0 | Not detected | 0.024 | -0.159 | 0.206 | 0.797 |
| ENZRXN0.8 | protein-histidine auto-kinase | 0 | Not detected | 0.144 | -0.039 | 0.327 | 0.123 |
| ENZRXN0.8 | ATP-dependent 3' &rarr; 5" h | 0 | Not detected | 0.183 | -0.052 | 0.419 | 0.127 |
| ENZRXN0.8 | 5-aminopentanoate:H+ symp | 0 | Not detected | 0.005 | -0.175 | 0.185 | 0.956 |
| ENZRXN0.8 | 5' &rarr; 3' helicase | 0 | Not detected | 0.065 | -0.143 | 0.273 | 0.541 |
| ENZRXN0.8 | apurinic/apyrimidinic endonu | 0 | Not detected | 0.055 | -0.108 | 0.218 | 0.508 |
| ENZRXN0.8 | 3'-phosphodiesterase | 0 | Not detected | 0.055 | -0.108 | 0.218 | 0.508 |
| ENZRXN0.8 | 3'-phosphomonoesterase | 0 | Not detected | 0.055 | -0.108 | 0.218 | 0.508 |
| ENZRXN0.8 | uracil DNA glycosylase | 0 | Not detected | 0.020 | -0.245 | 0.284 | 0.884 |
| ENZRXN0.8 | thymine glycol DNA glycosy | 0 | Not detected | 0.020 | -0.245 | 0.284 | 0.884 |
| ENZRXN0.8 | protein-histidine autokinase | 0 | Not detected | 0.139 | -0.051 | 0.329 | 0.152 |
| ENZRXN185 | 1,4-alpha-glucan branching e | 0 | Not detected | 0.023 | -0.183 | 0.229 | 0.828 |
| ENZRXN185 | phosphoglucomutase | 0 | Not detected | 0.139 | -0.172 | 0.450 | 0.380 |
| ENZRXN1F. | glutamate-1-semialdehyde am | 0 | Not detected | -0.060 | -0.361 | 0.240 | 0.693 |
| ENZRXN3O. | aconitate hydratase | 0 | Not detected | 0.280 | -0.133 | 0.694 | 0.184 |
| ENZRXN3O. | aconitate hydratase | 0 | Not detected | 0.280 | -0.133 | 0.694 | 0.184 |
| ENZRXN490 | enolase | 0 | Not detected | -0.001 | -0.269 | 0.267 | 0.994 |
| ENZRXN66. | pyruvate carboxylase | 0 | Not detected | 0.003 | -0.256 | 0.262 | 0.982 |
| ENZRXN66. | citrate dehydratase | 0 | Not detected | -0.139 | -0.377 | 0.098 | 0.250 |
| ENZRXN66. | aconitate hydratase | 0 | Not detected | -0.139 | -0.377 | 0.098 | 0.250 |
| ENZRXN8J2 | 1-deoxyxylulose-5-phosphate | 0 | Not detected | -0.274 | -0.582 | 0.035 | 0.082 |
| ENZRXN8J2 | GTP cyclohydrolase | 0 | Not detected | 0.014 | -0.147 | 0.175 | 0.864 |
| ENZRXN8J2 | orotate phosphoribosyltransfe | 0 | Not detected | -0.049 | -0.179 | 0.080 | 0.453 |
| ENZRXN8J2 | orotidine 5'-phosphate decarb | 0 | Not detected | 0.014 | -0.159 | 0.187 | 0.874 |
| ENZRXN8J2 | aspartate carbamoyltransferas | 0 | Not detected | 0.116 | -0.160 | 0.392 | 0.411 |
| ENZRXN8J2 | UDP-glucose 6-dehydrogena | 0 | Not detected | -0.204 | -0.449 | 0.040 | 0.101 |
| ENZRXN8J2 | 5-dehydro-2-deoxy-D-glucon | 0 | Not detected | 0.203 | -0.053 | 0.459 | 0.121 |
| ENZRXN8J2 | methionine-glutamine aminot | 0 | Not detected | 0.018 | -0.210 | 0.245 | 0.880 |
| ENZRXN8J2 | lipoyl synthase | 0 | Not detected | -0.116 | -0.346 | 0.114 | 0.323 |
| ENZRXN8J2 | 4-nitrobenzaldehyde reductas | 0 | Not detected | 0.195 | -0.051 | 0.440 | 0.121 |
| ENZRXN8J2 | CTP synthetase | 0 | Not detected | -0.009 | -0.127 | 0.109 | 0.882 |
| ENZRXN8J2 | UTP-glucose-1-phosphate ur | 0 | Not detected | -0.028 | -0.211 | 0.155 | 0.765 |
| ENZRXN8J2 | 1,5-Î±-L-arabinofuranosidase | 0 | Not detected | 0.000 | -0.204 | 0.204 | 0.999 |
| ENZRXN8J2 | 1,3-Î±-L-arabinofuranosidase | 0 | Not detected | 0.005 | -0.125 | 0.134 | 0.944 |
| ENZRXNI.14 | fumarase | 0 | Not detected | 0.027 | -0.202 | 0.256 | 0.817 |
| ENZRXNIO2 | alcohol dehydrogenase | 0 | Not detected | -0.157 | -0.398 | 0.084 | 0.203 |
| ENZRXNME | oxepin-CoA hydrolase | 0 | Not detected | 0.004 | -0.188 | 0.197 | 0.964 |
| ENZRXNME | 3-oxo-5,6-dehydrosuberyl-Co | 0 | Not detected | 0.004 | -0.188 | 0.197 | 0.964 |
| ENZRXNQT | phosphomethylpyrimidine sy | 0 | Not detected | 0.232 | -0.028 | 0.491 | 0.081 |
| ENZRXNQT | biotin carboxylase | 0 | Not detected | -0.053 | -0.192 | 0.086 | 0.458 |

| ENZRXNQT | H+-exporting diphosphatase | 0 | Not detected | 0.012 | -0.259 | 0.283 | 0.932 |
| --- | --- | --- | --- | --- | --- | --- | --- |
| ENZRXNQT | NADP+-dependent isocitrate | 0 | Not detected | -0.016 | -0.251 | 0.218 | 0.892 |
| ENZRXNQT | tryptophan synthase | 0 | Not detected | -0.102 | -0.359 | 0.156 | 0.438 |
| ENZRXNQT | aconitase | 0 | Not detected | -0.044 | -0.311 | 0.223 | 0.748 |
| ENZRXNQT | 3,4 dihydroxy-2-butanone-4- | 0 | Not detected | -0.010 | -0.132 | 0.111 | 0.868 |
| ENZRXNQT | GTP cyclohydrolase | 0 | Not detected | -0.010 | -0.132 | 0.111 | 0.868 |
| ENZRXNT9. | aconitase | 0 | Not detected | -0.044 | -0.311 | 0.223 | 0.748 |
| FADSYN.EN | FMN adenylyltransferase | 0 | Not detected | 0.016 | -0.167 | 0.199 | 0.863 |
| FGAMSYN.E | phosphoribosylformylglycina | 0 | Not detected | 0.281 | 0.066 | 0.495 | 0.011 |
| FOLYLPOLY | folylpoly-&gamma;-glutamat | 0 | Not detected | 0.087 | -0.103 | 0.276 | 0.370 |
| FORMYLTH | 10-formyl-tetrahydrofolylpol | 0 | Not detected | 0.087 | -0.103 | 0.276 | 0.370 |
| FPPSYN.EN | farnesyl diphosphate synthas | 0 | Not detected | -0.113 | -0.318 | 0.092 | 0.280 |
| FUCULOKIN | L-fuculokinase | 0 | Not detected | -0.158 | -0.423 | 0.107 | 0.242 |
| GALACTAR | galactarate dehydratase | 0 | Not detected | -0.101 | -0.347 | 0.145 | 0.421 |
| GALACTOK | galactokinase | 0 | Not detected | 0.067 | -0.126 | 0.260 | 0.494 |
| GALACTON | D-galactonate dehydratase | 0 | Not detected | -0.076 | -0.304 | 0.152 | 0.514 |
| GALACTUR | D-galacturonate isomerase | 0 | Not detected | -0.008 | -0.121 | 0.106 | 0.894 |
| GALPMUT.E | UDP-galactopyranose mutase | 0 | Not detected | -0.038 | -0.161 | 0.086 | 0.551 |
| GARTRANS | phosphoribosylglycinamide f | 0 | Not detected | -0.028 | -0.224 | 0.169 | 0.783 |
| GARTSIDE.E | acetate kinase | 0 | Not detected | -0.028 | -0.224 | 0.169 | 0.783 |
| GCVT.ENZR | aminomethyltransferase | 0 | Not detected | 0.042 | -0.140 | 0.224 | 0.653 |
| GDPPYPHO | GDP pyrophosphokinase | 0 | Not detected | 0.176 | -0.059 | 0.412 | 0.142 |
| GDPPYPHO | GDP diphosphokinase | 0 | Not detected | 0.045 | -0.209 | 0.300 | 0.727 |
| GKI.ENZRX | glycerate 2-kinase | 0 | Not detected | 0.031 | -0.190 | 0.252 | 0.783 |
| GLNS.ENZR | glutamine&mdash;tRNA liga | 0 | Not detected | -0.005 | -0.119 | 0.109 | 0.930 |
| GLU6PDEH | NADP+-dependent glucose-6 | 0 | Not detected | 0.010 | -0.164 | 0.183 | 0.914 |
| GLUCARDE | D-glucarate dehydratase | 3 | Not detected | -0.283 | -0.528 | -0.039 | 0.023 |
| GLUCUROIS | D-glucuronate isomerase | 0 | Not detected | -0.008 | -0.121 | 0.106 | 0.894 |
| GLURS.ENZ | glutamate&mdash;tRNA liga | 693 | 0.6 | 0.388 | 0.122 | 0.654 | 0.004 |
| GLUTCYSLI | glutamate&mdash;cysteine li | 0 | Not detected | 0.017 | -0.175 | 0.210 | 0.861 |
| GLYCEROL. | acyl-CoA:sn-glycerol-3-phos | 0 | Not detected | 0.220 | -0.024 | 0.465 | 0.078 |
| GLYCOGEN | 1,4-Î±-glucan branching enzy | 0 | Not detected | -0.165 | -0.369 | 0.039 | 0.113 |
| GLYCOGEN | glycogen synthase | 0 | Not detected | -0.066 | -0.271 | 0.138 | 0.526 |
| GLYRIBON | phosphoribosylamine&mdash | 0 | Not detected | 0.079 | -0.048 | 0.206 | 0.222 |
| GPPSYN.EN | geranyl diphosphate synthase | 0 | Not detected | -0.113 | -0.318 | 0.092 | 0.280 |
| GSADENYL | glutamine synthetase adenyly | 0 | Not detected | 0.065 | -0.186 | 0.316 | 0.612 |
| GSDEADEN | glutamine synthetase deadeny | 0 | Not detected | 0.065 | -0.186 | 0.316 | 0.612 |
| GTPPYPHOS | GTP pyrophosphokinase | 0 | Not detected | 0.176 | -0.059 | 0.412 | 0.142 |
| HEMEOSYN | heme O synthase | 0 | Not detected | -0.080 | -0.252 | 0.091 | 0.359 |
| HEMN.ENZR | coproporphyrinogen III dehy | 0 | Not detected | 0.152 | -0.071 | 0.374 | 0.182 |
| HISTCYCLO | phosphoribosyl-AMP cycloh | 0 | Not detected | 0.009 | -0.119 | 0.136 | 0.895 |
| HISTPRATP | phosphoribosyl-ATP pyropho | 0 | Not detected | 0.009 | -0.119 | 0.136 | 0.895 |
| HOMOCYSM | cobalamin-independent homo | 0 | Not detected | 0.108 | -0.137 | 0.354 | 0.386 |
| HOMOCYSM | methionine synthase | 0 | Not detected | -0.046 | -0.161 | 0.069 | 0.429 |

| IGPSYN.EN | indole-3-glycerol phosphate s | 0 | Not detected | 0.153 | -0.044 | 0.349 | 0.127 |
| --- | --- | --- | --- | --- | --- | --- | --- |
| ILES.ENZRX | isoleucine&mdash;tRNA liga | 0 | Not detected | 0.120 | -0.159 | 0.400 | 0.399 |
| IMPCYCLO | IMP cyclohydrolase | 0 | Not detected | 0.079 | -0.096 | 0.253 | 0.378 |
| KDOTRANS | 3-deoxy-D-manno-octuloson | 0 | Not detected | -0.020 | -0.225 | 0.185 | 0.849 |
| KDOTRANS | KDO transferase | 0 | Not detected | -0.020 | -0.225 | 0.185 | 0.849 |
| L.ASPARTA | L-aspartate oxidase | 0 | Not detected | -0.201 | -0.491 | 0.088 | 0.173 |
| L.LACTDEH | L-lactate dehydrogenase | 0 | Not detected | 0.147 | -0.061 | 0.356 | 0.165 |
| LAUROYLA | lauroyl acyltransferase | 0 | Not detected | -0.030 | -0.204 | 0.145 | 0.738 |
| LEUS.ENZR | leucine&mdash;tRNA ligase | 0 | Not detected | 0.209 | -0.070 | 0.488 | 0.142 |
| LSERINEDE | L-serine deaminase | 0 | Not detected | -0.060 | -0.339 | 0.219 | 0.672 |
| LSERINEDE | L-serine deaminase | 0 | Not detected | -0.124 | -0.321 | 0.072 | 0.215 |
| MALATESY | malate synthase A | 0 | Not detected | -0.090 | -0.292 | 0.111 | 0.380 |
| MALONYL. | [acyl-carrier-protein] S-malo | 0 | Not detected | 0.309 | 0.064 | 0.553 | 0.013 |
| MALSYNG.E | malate synthase G | 0 | Not detected | 0.070 | -0.133 | 0.274 | 0.498 |
| MALTODEX | maltodextrin glucosidase | 0 | Not detected | -0.011 | -0.209 | 0.187 | 0.911 |
| MANNOND | D-mannonate dehydratase | 88 | Not detected | -0.151 | -0.304 | 0.003 | 0.054 |
| MANNONO | D-mannonate oxidoreductase | 0 | Not detected | -0.069 | -0.294 | 0.157 | 0.551 |
| MANNPDEH | mannitol-1-phosphate 5-dehy | 0 | Not detected | -0.112 | -0.363 | 0.139 | 0.381 |
| MHPHYDRO | 3-(3-hydroxyphenyl)propano | 0 | Not detected | -0.134 | -0.323 | 0.054 | 0.164 |
| N.ACETYLG | N-acetylglutamylphosphate r | 0 | Not detected | 0.188 | -0.043 | 0.420 | 0.111 |
| NACGLCTR | N-acetylglucosaminyl transfe | 0 | Not detected | -0.010 | -0.195 | 0.176 | 0.919 |
| NACMURLA | N-acetylmuramoyl-L-alanine | 0 | Not detected | -0.117 | -0.310 | 0.075 | 0.233 |
| NADH.DHII. | NADH:ubiquinone oxidored | 0 | Not detected | 0.045 | -0.147 | 0.237 | 0.645 |
| NANK.ENZR | N-acetylmannosamine kinase | 0 | Not detected | -0.027 | -0.202 | 0.149 | 0.765 |
| NICOTINAT | nicotinate phosphoribosyltran | 0 | Not detected | 0.000 | -0.173 | 0.173 | 0.999 |
| O.SUCCINY | o-succinylbenzoate synthase | 0 | Not detected | 0.120 | -0.064 | 0.304 | 0.200 |
| OCTPRNL.M | 2-octaprenyl-3-methyl-6-met | 0 | Not detected | -0.028 | -0.208 | 0.152 | 0.759 |
| OHMETHYL | hydroxymethylbilane synthas | 0 | Not detected | 0.122 | -0.064 | 0.307 | 0.198 |
| PEPCARBO | phosphoenolpyruvate carbox | 3 | Not detected | 0.015 | -0.100 | 0.131 | 0.792 |
| PGLYCERO | phosphoglycerol transferase | 0 | Not detected | 0.012 | -0.201 | 0.226 | 0.909 |
| PGMI.ENZR | 2,3-bisphosphoglycerate-inde | 9 | Not detected | 0.038 | -0.076 | 0.153 | 0.510 |
| PHOSGLUC | phosphoglucosamine mutase | 0 | Not detected | 0.161 | -0.086 | 0.409 | 0.202 |
| PHOSGLYC | phosphoglycerate kinase | 658 | 0.6 | 0.356 | 0.120 | 0.592 | 0.003 |
| PHOSMANM | phosphomannomutase | 0 | Not detected | 0.007 | -0.268 | 0.281 | 0.962 |
| PHOSNACM | phospho-N-acetylmuramoyl- | 0 | Not detected | 0.123 | -0.123 | 0.368 | 0.327 |
| PHOSPHASE | phosphatidylserine synthase | 0 | Not detected | 0.049 | -0.165 | 0.263 | 0.653 |
| PHOSPHOG | phosphoglucomutase | 0 | Not detected | 0.095 | -0.140 | 0.329 | 0.430 |
| PPENTOMU | Î±-D-ribose 1,5-phosphomut | 0 | Not detected | 0.022 | -0.251 | 0.295 | 0.875 |
| PRAISOM.E | phosphoribosylanthranilate is | 0 | Not detected | 0.153 | -0.044 | 0.349 | 0.127 |
| PRIBFAICA | 1-(5-phosphoribosyl)-5-[(5-p | 23 | Not detected | 0.270 | 0.034 | 0.506 | 0.025 |
| PROPKIN.E | propionate kinase | 0 | Not detected | 0.037 | -0.136 | 0.210 | 0.678 |
| PROPKIN2.E | propionate kinase | 0 | Not detected | -0.067 | -0.266 | 0.132 | 0.509 |
| PROTOHEM | ferrochelatase | 0 | Not detected | 0.110 | -0.080 | 0.300 | 0.255 |
| PRPPSYN.E | ribose-phosphate diphosphok | 0 | Not detected | -0.011 | -0.137 | 0.115 | 0.864 |

| PSERPHOSP | phosphoserine phosphatase | 0 | Not detected | 0.038 | -0.150 | 0.226 | 0.691 |
| --- | --- | --- | --- | --- | --- | --- | --- |
| PSII.ENZRX | guanosine-3',5'-bis(diphosph | 0 | Not detected | 0.045 | -0.209 | 0.300 | 0.727 |
| RHAMNULO | rhamnulokinase | 0 | Not detected | 0.050 | -0.151 | 0.251 | 0.624 |
| RIBOFLAVI | riboflavin kinase | 0 | Not detected | 0.016 | -0.167 | 0.199 | 0.863 |
| RIBULP3EPI | ribulose-phosphate 3-epimera | 0 | Not detected | 0.118 | -0.061 | 0.297 | 0.197 |
| SHIKIMATE | shikimate dehydrogenase | 0 | Not detected | 0.004 | -0.166 | 0.175 | 0.959 |
| SHIKIMATE | shikimate kinase | 0 | Not detected | 0.004 | -0.166 | 0.174 | 0.965 |
| SUCCGLUA | succinylglutamate semialdeh | 0 | Not detected | -0.005 | -0.200 | 0.189 | 0.957 |
| TETRAACY | tetraacyldisaccharide 4'-kinas | 0 | Not detected | -0.007 | -0.176 | 0.161 | 0.932 |
| THI.P.KIN.E | thiamine monophosphate kin | 0 | Not detected | -0.017 | -0.196 | 0.163 | 0.856 |
| THIC.ENZR | phosphomethylpyrimidine sy | 0 | Not detected | -0.047 | -0.168 | 0.074 | 0.445 |
| THRESYN.E | threonine synthase | 0 | Not detected | 0.002 | -0.222 | 0.225 | 0.989 |
| TRANS.ENZ | transport of arabinose | 0 | Not detected | 0.142 | -0.098 | 0.382 | 0.247 |
| TRANS.ENZ | (S)-lactate transporter | 0 | Not detected | -0.143 | -0.358 | 0.073 | 0.195 |
| TRANS.ENZ | glycolate transporter | 0 | Not detected | -0.227 | -0.474 | 0.021 | 0.073 |
| TRANS.ENZ | succinate:fumarate antiport | 0 | Not detected | 0.139 | -0.104 | 0.383 | 0.262 |
| TRANS.ENZ | fumarate:succinate antiporter | 0 | Not detected | 0.119 | -0.090 | 0.328 | 0.264 |
| TRANS.ENZ | transport of adenosine | 0 | Not detected | 0.093 | -0.121 | 0.308 | 0.393 |
| TRANS.ENZ | transport of cytidine | 0 | Not detected | 0.093 | -0.121 | 0.308 | 0.393 |
| TRANS.ENZ | transport of deoxyadenosine | 0 | Not detected | 0.093 | -0.121 | 0.308 | 0.393 |
| TRANS.ENZ | transport of deoxycytidine | 0 | Not detected | 0.093 | -0.121 | 0.308 | 0.393 |
| TRANS.ENZ | transport of deoxyuridine | 0 | Not detected | 0.093 | -0.121 | 0.308 | 0.393 |
| TRANS.ENZ | transport of thymidine | 0 | Not detected | 0.093 | -0.121 | 0.308 | 0.393 |
| TRANS.ENZ | transport of uridine | 0 | Not detected | 0.093 | -0.121 | 0.308 | 0.393 |
| TRANS.ENZ | transport of rhamnose | 0 | Not detected | 0.015 | -0.161 | 0.192 | 0.865 |
| TRANS.ENZ | phosphate:H+ symporter | 0 | Not detected | 0.097 | -0.108 | 0.302 | 0.355 |
| TRANS.ENZ | cytosine transporter | 0 | Not detected | -0.012 | -0.229 | 0.205 | 0.916 |
| TRANS.ENZ | pantothenate:Na+ symporter | 0 | Not detected | 0.128 | -0.094 | 0.350 | 0.259 |
| TRANS.ENZ | proline:Na+ symporter | 0 | Not detected | 0.168 | -0.086 | 0.422 | 0.195 |
| TRANS.ENZ | transport of succinate | 0 | Not detected | 0.251 | -0.005 | 0.507 | 0.054 |
| TRANS.ENZ | transport of malate | 0 | Not detected | 0.251 | -0.005 | 0.507 | 0.054 |
| TRANS.ENZ | transport of fumarate | 0 | Not detected | 0.251 | -0.005 | 0.507 | 0.054 |
| TRANS.ENZ | transport of orotate | 0 | Not detected | 0.251 | -0.005 | 0.507 | 0.054 |
| TRANS.ENZ | L-glutamate:H+ symporter | 0 | Not detected | -0.012 | -0.204 | 0.180 | 0.899 |
| TRANS.ENZ | L-aspartate:H+ symporter | 0 | Not detected | -0.012 | -0.204 | 0.180 | 0.899 |
| TRANS.ENZ | transport of glutamate | 0 | Not detected | 0.024 | -0.190 | 0.239 | 0.824 |
| TRANS.ENZ | branched chain amino acid tr | 0 | Not detected | -0.040 | -0.226 | 0.145 | 0.672 |
| TRANS.ENZ | branched chain amino acid tr | 0 | Not detected | -0.040 | -0.226 | 0.145 | 0.672 |
| TRANS.ENZ | branched chain amino acid tr | 0 | Not detected | -0.040 | -0.226 | 0.145 | 0.672 |
| TRANS.ENZ | L-tartrate:succinate antiporter | 0 | Not detected | -0.128 | -0.308 | 0.053 | 0.165 |
| TRANS.ENZ | Na+:H+ antiporter | 0 | Not detected | 0.114 | -0.113 | 0.340 | 0.326 |
| TRANS.ENZ | Mg2+ transporter | 0 | Not detected | -0.071 | -0.265 | 0.123 | 0.472 |
| TRANS.ENZ | Co2+ transporter | 0 | Not detected | -0.071 | -0.265 | 0.123 | 0.472 |
| TRANS.ENZ | Ni2+ transporter | 0 | Not detected | -0.071 | -0.265 | 0.123 | 0.472 |

| TRANS.ENZ | K+ : H+ antiporter KefB | 0 | Not detected | -0.062 | -0.270 | 0.146 | 0.560 |
| --- | --- | --- | --- | --- | --- | --- | --- |
| TRANS.ENZ | K+:H+ symporter Kup | 0 | Not detected | -0.070 | -0.280 | 0.140 | 0.512 |
| TRANS.ENZ | fucose:H+ symporter | 0 | Not detected | 0.047 | -0.129 | 0.223 | 0.598 |
| TRANS.ENZ | fosmidomycin efflux pump | 0 | Not detected | -0.051 | -0.193 | 0.090 | 0.477 |
| TRANS.ENZ | transport of 3-(3-hydroxyphe | 0 | Not detected | -0.073 | -0.250 | 0.105 | 0.422 |
| TRANS.ENZ | sugar exporter SetA | 0 | Not detected | 0.032 | -0.145 | 0.209 | 0.721 |
| TRANS.ENZ | sugar exporter SetB | 0 | Not detected | 0.029 | -0.163 | 0.221 | 0.767 |
| TRANS.ENZ | transport of D-galactose | 0 | Not detected | -0.018 | -0.204 | 0.168 | 0.849 |
| TRANS.ENZ | Mn2+:H+ symporter | 0 | Not detected | 0.123 | -0.046 | 0.293 | 0.154 |
| TRANS.ENZ | L-threonine:H+ antiporter | 0 | Not detected | -0.032 | -0.229 | 0.165 | 0.752 |
| TRANS.ENZ | K+ transporter TrkH | 0 | Not detected | 0.102 | -0.111 | 0.314 | 0.349 |
| TRANS.ENZ | sn-glycerol 3-phosphate:phos | 0 | Not detected | -0.088 | -0.318 | 0.143 | 0.456 |
| TRANS.ENZ | transport of 2-oxoglutarate | 0 | Not detected | -0.099 | -0.279 | 0.081 | 0.279 |
| TRANS.ENZ | N-acetylneuraminate:H+ sym | 0 | Not detected | 0.000 | -0.190 | 0.191 | 0.996 |
| TRANS.ENZ | transport of a nucleoside | 0 | Not detected | 0.011 | -0.274 | 0.296 | 0.940 |
| TRANS.ENZ | transport of D-xylose | 0 | Not detected | -0.164 | -0.350 | 0.022 | 0.083 |
| TRANS.ENZ | xanthosine:H+ symporter Xa | 0 | Not detected | -0.188 | -0.382 | 0.005 | 0.057 |
| TRANS.ENZ | glucose-6-phosphate:phospha | 0 | Not detected | -0.119 | -0.316 | 0.078 | 0.237 |
| TRANS.ENZ | D-glucuronate transporter | 0 | Not detected | 0.061 | -0.125 | 0.246 | 0.522 |
| TRANS.ENZ | D-galacturonate transporter | 0 | Not detected | 0.061 | -0.125 | 0.246 | 0.522 |
| TRANS.ENZ | L-phenylalanine:H+ symport | 0 | Not detected | -0.026 | -0.248 | 0.197 | 0.822 |
| TRANS.ENZ | L-tyrosine:H+ symporter | 0 | Not detected | -0.026 | -0.248 | 0.197 | 0.822 |
| TRANS.ENZ | L-tryptophan:H+ symporter | 0 | Not detected | -0.026 | -0.248 | 0.197 | 0.822 |
| TRANS.ENZ | 4-aminobutanoate:H+ sympo | 0 | Not detected | 0.005 | -0.175 | 0.185 | 0.956 |
| TRANS.ENZ | lysine:H+ symporter | 0 | Not detected | 0.145 | -0.120 | 0.410 | 0.283 |
| TRANS.ENZ | phenylalanine:H+ symporter | 0 | Not detected | 0.194 | -0.044 | 0.431 | 0.110 |
| TRANS.ENZ | transport of D-alanine | 0 | Not detected | -0.172 | -0.398 | 0.055 | 0.137 |
| TRANS.ENZ | transport of glycine | 0 | Not detected | -0.172 | -0.398 | 0.055 | 0.137 |
| TRANS.ENZ | lysine:cadaverine antiporter | 0 | Not detected | 0.063 | -0.183 | 0.309 | 0.618 |
| TRANS.ENZ | serine:H+ symporter SdaC | 0 | Not detected | -0.104 | -0.332 | 0.123 | 0.370 |
| TRANS.ENZ | L-threonine:H+ symporter | 0 | Not detected | 0.067 | -0.117 | 0.252 | 0.475 |
| TRANS.ENZ | L-serine:H+ symporter | 0 | Not detected | 0.067 | -0.117 | 0.252 | 0.475 |
| TRANS.ENZ | tryptophan:H+ symporter | 0 | Not detected | 0.060 | -0.147 | 0.268 | 0.569 |
| TRANS.ENZ | indole:H+ symporter | 0 | Not detected | 0.060 | -0.147 | 0.268 | 0.569 |
| TRANS.ENZ | choline:H+ symporter | 0 | Not detected | -0.101 | -0.313 | 0.112 | 0.353 |
| TRE6PHYDR | trehalose-6-phosphate hydrol | 511 | Not detected | -0.401 | -0.657 | -0.145 | 0.002 |
| TREHALAC | trehalase | 0 | Not detected | -0.185 | -0.386 | 0.016 | 0.072 |
| TREHALAPE | trehalase | 0 | Not detected | -0.055 | -0.236 | 0.126 | 0.555 |
| TREHALOSE | trehalose-6-phosphate syntha | 0 | Not detected | 0.058 | -0.115 | 0.232 | 0.511 |
| TSA.REDUC | tartronate semialdehyde redu | 0 | Not detected | -0.158 | -0.368 | 0.052 | 0.141 |
| UDP.NACM | UDP-N-acetylmuramoyl-L-al | 0 | Not detected | 0.118 | -0.090 | 0.326 | 0.267 |
| UDP.NACM | D-alanyl-D-alanine-adding e | 0 | Not detected | 0.117 | -0.087 | 0.320 | 0.261 |
| UDP.NACM | UDP-N-acetylmuramoyl-L-al | 0 | Not detected | 0.093 | -0.122 | 0.308 | 0.397 |
| UDPACYLG | UDP-3-O-acyl-N-acetylgluco | 0 | Not detected | 0.322 | 0.061 | 0.584 | 0.016 |

| UDPNACET | UDP-N-acetylglucosamine 1- | 0 | Not detected | 0.074 | -0.159 | 0.307 | 0.533 |
| --- | --- | --- | --- | --- | --- | --- | --- |
| UDPNACET | UDP-N-acetylenolpyruvoylgl | 0 | Not detected | -0.001 | -0.192 | 0.189 | 0.989 |
| UDPSUGAR | UDP-sugar hydrolase | 0 | Not detected | 0.063 | -0.158 | 0.284 | 0.575 |
| URITRANS. | [protein-PII] uridylyltransfer | 0 | Not detected | -0.044 | -0.266 | 0.178 | 0.699 |
| URKI.ENZR | uridine kinase | 0 | Not detected | 0.271 | 0.022 | 0.520 | 0.033 |
| UROGENDE | uroporphyrinogen decarboxy | 0 | Not detected | 0.155 | -0.095 | 0.405 | 0.225 |
| VALINE.PY | valine&mdash;pyruvate amin | 1 | Not detected | -0.137 | -0.324 | 0.049 | 0.149 |
| VALS.ENZR | valine&mdash;tRNA ligase | 0 | Not detected | 0.165 | -0.120 | 0.450 | 0.258 |
| X1.ACYLGL | acyl-CoA:1-acyl-sn-glycerol- | 0 | Not detected | -0.062 | -0.234 | 0.111 | 0.482 |
| X2.OCTAPR | 2-octaprenyl-6-methoxy-1,4- | 0 | Not detected | 0.170 | -0.085 | 0.425 | 0.192 |
| X2.OCTPRN | 2-octaprenyl-6-methoxyphen | 0 | Not detected | -0.003 | -0.186 | 0.180 | 0.977 |
| X3.DEHYDR | 3-dehydroquinate synthase | 0 | Not detected | 0.152 | -0.075 | 0.379 | 0.190 |
| X3.NUCLEO | 3'-nucleotidase | 0 | Not detected | -0.004 | -0.237 | 0.229 | 0.974 |
| X3.P.SHIK.1 | 3-phosphoshikimate 1-carbox | 0 | Not detected | 0.078 | -0.131 | 0.288 | 0.463 |
| X5.NUCLEO | 5'-ribonucleotidase | 0 | Not detected | 0.063 | -0.158 | 0.284 | 0.575 |

| **Supplementary Table 6.** Association of current SEP with enzymatic reactions in overall participants (n=2174). Analysis of Compositions of Microbiomes (ANCOM2) was used to identify enzymatic reactions related to current SEP. Regression coefficients were obtained by modeling the effect of current SEP on centered log ratio (CLR)-transformed species in multivariable linear regression adjusted for age, gender, BMI, field center, Hispanic/Latino background, U.S. nativity, Bristol stool type, parental education, the alternative Healthy Eating Index 2010 score, cigarette smoking, alcohol use, depressive and anxiety symptoms, diabetes, hypertension, cardiovascular disease, dyslipidemia, and antibiotic use. | | | | | | | |
| --- | --- | --- | --- | --- | --- | --- | --- |
| **otu.names** | **V2** | **W_stat** | **ANCOM** | **beta** | **l95ci** | **u95ci** | **qval** |
| ACETATEKI | acetate kinase | 0 | Not detected | 0.164 | -0.044 | 0.372 | 0.123 |
| ACS.ENZRX | acetyl-CoA synthetase (AMP-forming) | 0 | Not detected | 0.028 | -0.218 | 0.275 | 0.823 |
| ACYLACPS | acyl-ACP synthetase | 0 | Not detected | 0.053 | -0.169 | 0.275 | 0.641 |
| ACYLCOAD | acyl-CoA dehydrogenase | 0 | Not detected | 0.059 | -0.170 | 0.288 | 0.611 |
| ACYLGPEA | 2-acylglycerophosphoethanolamine acyl | 0 | Not detected | 0.053 | -0.169 | 0.275 | 0.641 |
| ADENYL.KI | adenylate kinase | 0 | Not detected | -0.051 | -0.177 | 0.075 | 0.430 |
| ADENYLAT | adenylate cyclase | 1 | Not detected | 0.231 | -0.013 | 0.476 | 0.064 |
| ADOMET.D | S-adenosylmethionine:2-demethylquino | 0 | Not detected | 0.032 | -0.235 | 0.300 | 0.812 |
| AICARTRAN | AICAR transformylase | 0 | Not detected | 0.092 | -0.091 | 0.275 | 0.324 |
| ALDOSE1EP | galactose-1-epimerase | 0 | Not detected | -0.003 | -0.193 | 0.188 | 0.978 |
| ALPHA.AM | Î±-amylase | 0 | Not detected | 0.021 | -0.166 | 0.209 | 0.825 |
| ALPHA.AM | Î±-amylase | 0 | Not detected | 0.174 | -0.046 | 0.395 | 0.121 |
| ALTRO.OXI | tagaturonate reductase | 0 | Not detected | 0.057 | -0.139 | 0.253 | 0.570 |
| ALTRODEH | D-altronate dehydratase | 0 | Not detected | -0.014 | -0.134 | 0.107 | 0.825 |
| AMYLOMA | amylomaltase | 0 | Not detected | 0.071 | -0.165 | 0.307 | 0.556 |
| ARGS.ENZR | arginine&mdash;tRNA ligase | 0 | Not detected | -0.095 | -0.344 | 0.155 | 0.458 |
| ARGSUCCIN | argininosuccinate lyase | 0 | Not detected | -0.079 | -0.226 | 0.068 | 0.294 |
| ARGSUCCT | arginine N-succinyltransferase | 0 | Not detected | 0.001 | -0.174 | 0.176 | 0.992 |
| BIOTINLIG. | biotin-[acetyl-CoA-carboxylase] ligase | 0 | Not detected | 0.103 | -0.081 | 0.288 | 0.271 |
| CARDIOLIP | cardiolipin synthase | 0 | Not detected | 0.068 | -0.115 | 0.252 | 0.467 |
| CDPDIGLYP | CDP-diacylglycerol diphosphatase | 0 | Not detected | -0.014 | -0.179 | 0.152 | 0.872 |
| CHD.ENZRX | choline dehydrogenase | 0 | Not detected | 0.131 | -0.087 | 0.349 | 0.240 |
| CMPKI.ENZ | CMP kinase | 0 | Not detected | 0.093 | -0.141 | 0.326 | 0.437 |
| CPM.KDOSY | 3-deoxy-manno-octulosonate cytidylyltr | 0 | Not detected | -0.091 | -0.362 | 0.180 | 0.510 |
| CROBETRE | crotonobetainyl-CoA reductase | 0 | Not detected | 0.041 | -0.184 | 0.266 | 0.721 |
| CYCPHOSD | 2',3'-cyclic nucleotide 2'-phosphodiester | 0 | Not detected | 0.031 | -0.213 | 0.275 | 0.804 |
| CYSS.ENZR | cysteine&mdash;tRNA ligase | 0 | Not detected | -0.155 | -0.419 | 0.109 | 0.251 |
| CYTIDINEK | cytidine kinase | 0 | Not detected | 0.029 | -0.232 | 0.290 | 0.829 |
| D.PPENTOM | deoxyribose 1,5-phosphomutase | 0 | Not detected | -0.030 | -0.316 | 0.257 | 0.838 |
| DALADALA | D-alanine&mdash;D-alanine ligase | 0 | Not detected | 0.049 | -0.131 | 0.228 | 0.593 |
| DARABKIN. | D-ribulokinase | 0 | Not detected | -0.071 | -0.349 | 0.207 | 0.615 |
| DEHYDDEO | 2-dehydro-3-deoxygalactonokinase | 0 | Not detected | 0.055 | -0.136 | 0.246 | 0.570 |
| DEOXYGLU | 2-dehydro-3-deoxygluconokinase | 0 | Not detected | -0.022 | -0.205 | 0.160 | 0.811 |
| DEOXYRIB | deoxyribose-phosphate aldolase | 0 | Not detected | 0.083 | -0.085 | 0.251 | 0.334 |
| DIENOYLCO | 2,4-dienoyl-CoA reductase | 0 | Not detected | 0.159 | -0.069 | 0.386 | 0.171 |
| DIHYDROF | dihydrofolate synthetase | 0 | Not detected | -0.017 | -0.216 | 0.182 | 0.867 |
| DIHYDROO | dihydroorotate dehydrogenase | 0 | Not detected | 0.002 | -0.294 | 0.297 | 0.992 |

| DLACTDEH | D-lactate dehydrogenase (quionone) | 0 | Not detected | 0.006 | -0.195 | 0.208 | 0.950 |
| --- | --- | --- | --- | --- | --- | --- | --- |
| DMK.ENZR | 1,4-dihydroxy-2-naphthoate octaprenylt | 0 | Not detected | -0.037 | -0.236 | 0.162 | 0.715 |
| DSERDEAM | D-serine ammonia-lyase | 0 | Not detected | -0.020 | -0.256 | 0.216 | 0.870 |
| DTDPDEHY | dTDP-4-dehydrorhamnose 3,5-epimeras | 0 | Not detected | 0.011 | -0.123 | 0.145 | 0.873 |
| DTDPGLUC | dTDP-glucose 4,6-dehydratase | 0 | Not detected | -0.019 | -0.153 | 0.116 | 0.786 |
| ENTC.ENZR | isochorismate synthase | 4 | Not detected | -0.181 | -0.361 | 0.000 | 0.050 |
| ENZRXN.10 | L-cysteine-S-conjugate thiol-lyase (deam | 0 | Not detected | 0.082 | -0.156 | 0.320 | 0.499 |
| ENZRXN.11 | imidazolone-5-propionate hydrolase | 0 | Not detected | -0.104 | -0.361 | 0.154 | 0.430 |
| ENZRXN.11 | ammonia-dependent carbamoyl-phospha | 0 | Not detected | 0.102 | -0.078 | 0.282 | 0.265 |
| ENZRXN.11 | cystathionine &gamma;-lyase | 0 | Not detected | 0.122 | -0.159 | 0.404 | 0.395 |
| ENZRXN.12 | citrate synthase | 0 | Not detected | -0.045 | -0.372 | 0.281 | 0.785 |
| ENZRXN.12 | citrate hydro-lyase (cis-aconitate-formin | 0 | Not detected | 0.208 | -0.095 | 0.511 | 0.178 |
| ENZRXN.12 | D-threo-isocitrate hydro-lyase (cis-aconi | 0 | Not detected | 0.208 | -0.095 | 0.511 | 0.178 |
| ENZRXN.12 | malate dehydrogenase | 0 | Not detected | -0.068 | -0.377 | 0.241 | 0.667 |
| ENZRXN.12 | 1-deoxy-D-xylulose 5-phosphate reduct | 0 | Not detected | 0.060 | -0.094 | 0.214 | 0.446 |
| ENZRXN.12 | Pfl-activating enzyme | 0 | Not detected | -0.184 | -0.453 | 0.084 | 0.179 |
| ENZRXN.12 | phenylacetate-CoA ligase | 0 | Not detected | -0.090 | -0.240 | 0.061 | 0.242 |
| ENZRXN.12 | xylitol dehydrogenase | 0 | Not detected | 0.142 | -0.161 | 0.445 | 0.359 |
| ENZRXN.12 | UDP-N-acetylenolpyruvoylglucosamine | 0 | Not detected | 0.098 | -0.145 | 0.341 | 0.430 |
| ENZRXN.12 | UDP-N-acetylmuramoyl-L-alanyl-D-glu | 0 | Not detected | 0.187 | -0.127 | 0.501 | 0.244 |
| ENZRXN.12 | 3,5-diaminohexanoate dehydrogenase | 0 | Not detected | 0.134 | -0.127 | 0.395 | 0.314 |
| ENZRXN.12 | 3-aminobutyryl-CoA deaminase | 0 | Not detected | 0.017 | -0.269 | 0.303 | 0.906 |
| ENZRXN.13 | thiosulfate reductase (cytochrome) | 0 | Not detected | 0.200 | -0.066 | 0.466 | 0.141 |
| ENZRXN.13 | Enzyme IIscr | 0 | Not detected | -0.054 | -0.346 | 0.237 | 0.715 |
| ENZRXN.13 | Enzyme IIscr | 0 | Not detected | 0.159 | -0.068 | 0.386 | 0.170 |
| ENZRXN.13 | fructokinase | 0 | Not detected | 0.026 | -0.158 | 0.209 | 0.783 |
| ENZRXN.13 | phosphoribosyl-formimino-5-aminoimid | 0 | Not detected | 0.109 | -0.053 | 0.271 | 0.189 |
| ENZRXN.13 | imidazoleglycerolphosphate dehydratase | 0 | Not detected | -0.136 | -0.370 | 0.097 | 0.253 |
| ENZRXN.13 | sucrose phosphorylase | 0 | Not detected | 0.271 | -0.055 | 0.596 | 0.103 |
| ENZRXN.13 | phosphoserine aminotransferase | 0 | Not detected | 0.057 | -0.081 | 0.196 | 0.418 |
| ENZRXN.13 | phosphoglucose isomerase | 0 | Not detected | -0.101 | -0.388 | 0.185 | 0.488 |
| ENZRXN.13 | 3-phosphoglycerate kinase | 0 | Not detected | -0.010 | -0.152 | 0.131 | 0.885 |
| ENZRXN.13 | myo-inosose-2 dehydratase | 0 | Not detected | 0.119 | -0.114 | 0.352 | 0.317 |
| ENZRXN.13 | glucose-6-phosphate isomerase | 0 | Not detected | 0.128 | -0.161 | 0.417 | 0.386 |
| ENZRXN.13 | fructokinase | 0 | Not detected | -0.085 | -0.328 | 0.158 | 0.493 |
| ENZRXN.13 | glucose-6-phosphate isomerase | 0 | Not detected | 0.004 | -0.123 | 0.130 | 0.952 |
| ENZRXN.13 | phosphotransacetylase | 0 | Not detected | 0.210 | -0.002 | 0.422 | 0.052 |
| ENZRXN.14 | UDP-N-acetylglucosamine 4-epimerase | 0 | Not detected | -0.187 | -0.521 | 0.147 | 0.272 |
| ENZRXN.14 | UDP-galactose 4-epimerase | 0 | Not detected | -0.187 | -0.521 | 0.147 | 0.272 |
| ENZRXN.14 | cobalt precorrin-4 (C11)-methyltransfer | 0 | Not detected | 0.002 | -0.145 | 0.149 | 0.983 |
| ENZRXN.14 | cobalt-precorrin-3 (C17)-methyltransfer | 0 | Not detected | -0.024 | -0.301 | 0.253 | 0.865 |
| ENZRXN.14 | GDP-D-mannose pyrophosphorylase | 0 | Not detected | -0.006 | -0.256 | 0.243 | 0.962 |
| ENZRXN.14 | phosphomannose isomerase | 0 | Not detected | -0.006 | -0.256 | 0.243 | 0.962 |
| ENZRXN.14 | succinyl-CoA:CoA transferase | 0 | Not detected | -0.184 | -0.411 | 0.042 | 0.111 |

| ENZRXN.14 | 4-hydroxybutyrate coenzyme A transfer | 0 | Not detected | -0.192 | -0.468 | 0.085 | 0.175 |
| --- | --- | --- | --- | --- | --- | --- | --- |
| ENZRXN.14 | crotonase | 0 | Not detected | 0.045 | -0.251 | 0.341 | 0.768 |
| ENZRXN.14 | butyryl-CoA dehydrogenase | 0 | Not detected | 0.023 | -0.193 | 0.239 | 0.836 |
| ENZRXN.14 | phosphate acetyltransferase | 0 | Not detected | -0.005 | -0.135 | 0.125 | 0.945 |
| ENZRXN.14 | acetate kinase | 0 | Not detected | 0.013 | -0.248 | 0.274 | 0.921 |
| ENZRXN.14 | GDP-Î±-D-mannose 4,6-dehydratase | 0 | Not detected | -0.108 | -0.389 | 0.173 | 0.452 |
| ENZRXN.14 | aconitase | 0 | Not detected | -0.042 | -0.199 | 0.116 | 0.606 |
| ENZRXN.14 | phosphoenolpyruvate phosphomutase | 0 | Not detected | -0.039 | -0.336 | 0.258 | 0.797 |
| ENZRXN.14 | naphthoyl-CoA synthase | 0 | Not detected | 0.008 | -0.134 | 0.149 | 0.917 |
| ENZRXN.14 | N-acetylhexosamine kinase | 0 | Not detected | 0.093 | -0.201 | 0.388 | 0.535 |
| ENZRXN.14 | glutamyl-tRNAGln synthetase | 0 | Not detected | 0.110 | -0.191 | 0.410 | 0.475 |
| ENZRXN.15 | glutamyl-tRNAGlu synthetase | 0 | Not detected | 0.110 | -0.191 | 0.410 | 0.475 |
| ENZRXN.15 | acyl phosphate:glycerol-3-phosphate acy | 0 | Not detected | 0.056 | -0.154 | 0.265 | 0.603 |
| ENZRXN.15 | 1-acylglycerol-3-phosphate acyltransfera | 0 | Not detected | 0.043 | -0.182 | 0.268 | 0.710 |
| ENZRXN.15 | biotin sulfoxide reductase | 0 | Not detected | 0.068 | -0.166 | 0.302 | 0.570 |
| ENZRXN.15 | malonate decarboxylase acetyl-[acp]:ma | 0 | Not detected | 0.088 | -0.118 | 0.293 | 0.402 |
| ENZRXN.15 | acyl-carrier protein: acetate ligase | 0 | Not detected | -0.149 | -0.452 | 0.154 | 0.336 |
| ENZRXN.15 | acetyl-[acyl-carrier protein]:malonate [a | 0 | Not detected | -0.093 | -0.382 | 0.195 | 0.526 |
| ENZRXN.15 | carboxybiotin decarboxylase | 0 | Not detected | -0.315 | -0.543 | -0.087 | 0.007 |
| ENZRXN.15 | N-succinylornithine carbamoyltransferas | 0 | Not detected | -0.020 | -0.168 | 0.128 | 0.792 |
| ENZRXN.15 | 5-monosubstituted-hydantoin racemase | 0 | Not detected | 0.198 | -0.085 | 0.481 | 0.170 |
| ENZRXN.15 | phosphoglycerate mutase | 0 | Not detected | -0.221 | -0.513 | 0.071 | 0.138 |
| ENZRXN.15 | 2-keto-3-deoxy-D-glycero-D-galacto-no | 0 | Not detected | -0.363 | -0.697 | -0.028 | 0.034 |
| ENZRXN.15 | 2-keto-3-deoxy-D-glycero-D-galacto-no | 0 | Not detected | -0.232 | -0.543 | 0.080 | 0.145 |
| ENZRXN.16 | Î²-D-galactosyl-(1->4)-L-rhamnose phos | 0 | Not detected | -0.107 | -0.423 | 0.209 | 0.507 |
| ENZRXN.17 | UDP-N-acetylglucosamine enolpyruvyl | 0 | Not detected | 0.247 | -0.059 | 0.553 | 0.114 |
| ENZRXN.17 | UDP-galactopyranose mutase | 0 | Not detected | 0.253 | -0.072 | 0.578 | 0.127 |
| ENZRXN.17 | 3-dehydroquinate dehydratase | 0 | Not detected | 0.026 | -0.224 | 0.276 | 0.837 |
| ENZRXN.17 | UDP-N-acetylglucosamine--N-acetylmu | 0 | Not detected | -0.085 | -0.263 | 0.092 | 0.346 |
| ENZRXN.17 | UDP-N-acetylmuramoyl--L-alanine liga | 0 | Not detected | 0.183 | -0.087 | 0.452 | 0.184 |
| ENZRXN.17 | UDP-N-acetylglucosamine 1-carboxyvin | 0 | Not detected | -0.091 | -0.299 | 0.117 | 0.392 |
| ENZRXN.17 | UDP-N-acetylglucosamine 1-carboxyvin | 0 | Not detected | 0.122 | -0.051 | 0.296 | 0.166 |
| ENZRXN.17 | undecaprenyl-pyrophosphoryl-MurNAc | 0 | Not detected | 0.065 | -0.246 | 0.376 | 0.682 |
| ENZRXN.17 | asparagine synthase | 0 | Not detected | 0.011 | -0.296 | 0.318 | 0.945 |
| ENZRXN.17 | undecaprenyl-pyrophosphoryl-MurNAc | 0 | Not detected | 0.065 | -0.246 | 0.376 | 0.682 |
| ENZRXN.17 | D-sedoheptulose 7-phosphate isomerase | 0 | Not detected | -0.015 | -0.294 | 0.263 | 0.914 |
| ENZRXN.17 | D-glycero-Î±-D-manno-heptose-7-phosp | 0 | Not detected | 0.114 | -0.203 | 0.431 | 0.480 |
| ENZRXN.17 | methylthioalkylmalate dehydrogenase | 0 | Not detected | 0.035 | -0.108 | 0.179 | 0.629 |
| ENZRXN.17 | methylthioalkylmalate dehydrogenase | 0 | Not detected | 0.035 | -0.108 | 0.179 | 0.629 |
| ENZRXN.17 | methylthioalkylmalate dehydrogenase | 0 | Not detected | 0.035 | -0.108 | 0.179 | 0.629 |
| ENZRXN.17 | methylthioalkylmalate dehydrogenase | 0 | Not detected | 0.035 | -0.108 | 0.179 | 0.629 |
| ENZRXN.17 | methylthioalkylmalate dehydrogenase | 0 | Not detected | 0.035 | -0.108 | 0.179 | 0.629 |
| ENZRXN.17 | lipoate-ATP adenylate transferase | 0 | Not detected | -0.011 | -0.177 | 0.155 | 0.897 |
| ENZRXN.17 | pimeloyl-[acp] methyl ester esterase | 0 | Not detected | 0.081 | -0.090 | 0.253 | 0.353 |

| ENZRXN.17 | (2Z,6E)-farnesyl diphosphate mono-tran | 0 | Not detected | 0.239 | -0.065 | 0.543 | 0.123 |
| --- | --- | --- | --- | --- | --- | --- | --- |
| ENZRXN.18 | 3-(hydroxyamino)phenol mutase | 0 | Not detected | -0.176 | -0.486 | 0.135 | 0.268 |
| ENZRXN.18 | butanal dehydrogenase | 0 | Not detected | 0.002 | -0.136 | 0.139 | 0.981 |
| ENZRXN.18 | butanol dehydrogenase | 0 | Not detected | 0.002 | -0.136 | 0.139 | 0.981 |
| ENZRXN.18 | glycerol-2-phosphotransferase | 0 | Not detected | 0.228 | -0.030 | 0.486 | 0.084 |
| ENZRXN.18 | DsrC-trisulfide reductase | 0 | Not detected | 0.209 | -0.071 | 0.489 | 0.144 |
| ENZRXN.18 | dCMP kinase | 0 | Not detected | 0.093 | -0.141 | 0.326 | 0.437 |
| ENZRXN.18 | 3-hydroxy-trans-cinnamate hydroxylase | 0 | Not detected | -0.026 | -0.224 | 0.172 | 0.797 |
| ENZRXN.18 | 1-deoxy-D-xylulose 5-phosphate reduct | 0 | Not detected | -0.092 | -0.396 | 0.211 | 0.551 |
| ENZRXN.18 | rhamnogalacturonan endolyase | 0 | Not detected | -0.114 | -0.348 | 0.119 | 0.338 |
| ENZRXN.18 | unsaturated chondroitin 6-sulfate disacc | 0 | Not detected | -0.135 | -0.381 | 0.110 | 0.281 |
| ENZRXN.18 | legionaminate synthase | 0 | Not detected | -0.212 | -0.486 | 0.062 | 0.129 |
| ENZRXN.18 | rhamnogalacturonan exolyase | 0 | Not detected | -0.104 | -0.360 | 0.151 | 0.424 |
| ENZRXN.18 | isoamylase | 0 | Not detected | 0.310 | 0.028 | 0.592 | 0.031 |
| ENZRXN.18 | formate dehydrogenase | 0 | Not detected | -0.044 | -0.301 | 0.214 | 0.741 |
| ENZRXN.19 | cellobiose phosphorylase | 0 | Not detected | 0.021 | -0.129 | 0.172 | 0.783 |
| ENZRXN.19 | GTP 3',8'-cyclase | 0 | Not detected | -0.030 | -0.225 | 0.166 | 0.766 |
| ENZRXN.19 | endo-Î±-N-acetylgalactosaminidase | 0 | Not detected | -0.084 | -0.400 | 0.233 | 0.604 |
| ENZRXN.19 | trans-2-enoyl-CoA reductase (NAD+) | 0 | Not detected | -0.086 | -0.332 | 0.160 | 0.492 |
| ENZRXN.19 | crotonyl-CoA reductase | 0 | Not detected | -0.086 | -0.332 | 0.160 | 0.492 |
| ENZRXN.19 | trans-2-hexenoyl-CoA reductase | 0 | Not detected | -0.086 | -0.332 | 0.160 | 0.492 |
| ENZRXN.19 | hexanal dehydrogenase | 0 | Not detected | 0.002 | -0.136 | 0.139 | 0.981 |
| ENZRXN.19 | hexanol dehydrogenase | 0 | Not detected | 0.002 | -0.136 | 0.139 | 0.981 |
| ENZRXN.19 | carboxy-adenylated-[ThiS sulfur-carrier | 0 | Not detected | -0.064 | -0.281 | 0.152 | 0.561 |
| ENZRXN.19 | molybdopterin synthase sulfurtransferas | 0 | Not detected | 0.181 | 0.006 | 0.356 | 0.042 |
| ENZRXN.19 | N,N'-diacetylchitobiose phosphorylase | 0 | Not detected | -0.047 | -0.339 | 0.244 | 0.751 |
| ENZRXN.19 | 12Î±-hydroxy-3-oxochol-4,6-dien-24-oy | 0 | Not detected | 0.222 | -0.064 | 0.508 | 0.128 |
| ENZRXN.19 | dTDP-glucose 4,6-dehydratase | 0 | Not detected | -0.040 | -0.268 | 0.189 | 0.732 |
| ENZRXN.19 | glucose-1-phosphate thymidylyltransfer | 0 | Not detected | 0.018 | -0.117 | 0.153 | 0.794 |
| ENZRXN.19 | dTDP-3-amino-3,6-dideoxy-Î±-D-galact | 0 | Not detected | -0.108 | -0.415 | 0.200 | 0.493 |
| ENZRXN.19 | 2-oxoglutarate decarboxylase | 0 | Not detected | -0.074 | -0.390 | 0.241 | 0.645 |
| ENZRXN.20 | deoxyribonuclease IV | 0 | Not detected | -0.155 | -0.338 | 0.027 | 0.095 |
| ENZRXN.20 | uracil-DNA glycosylase | 0 | Not detected | -0.051 | -0.171 | 0.068 | 0.401 |
| ENZRXN.20 | DNA glycosylase | 0 | Not detected | -0.026 | -0.303 | 0.252 | 0.855 |
| ENZRXN.20 | DNA-formamidopyrimidine glycosylase | 0 | Not detected | -0.069 | -0.254 | 0.116 | 0.466 |
| ENZRXN.20 | adenine glycosylase | 0 | Not detected | -0.077 | -0.274 | 0.121 | 0.446 |
| ENZRXN.20 | glycerol-3-phosphate dehydrogenase | 0 | Not detected | 0.129 | -0.148 | 0.407 | 0.361 |
| ENZRXN.20 | 3-isopropylmalate dehydratase | 0 | Not detected | -0.006 | -0.328 | 0.317 | 0.973 |
| ENZRXN.20 | (S)-NADPHX dehydratase | 0 | Not detected | 0.083 | -0.128 | 0.293 | 0.443 |
| ENZRXN.20 | NADPHX epimerase | 0 | Not detected | 0.083 | -0.128 | 0.293 | 0.443 |
| ENZRXN.20 | butanol dehydrogenase | 0 | Not detected | 0.004 | -0.135 | 0.143 | 0.953 |
| ENZRXN.20 | CoA-dependent propionaldehyde dehyd | 0 | Not detected | -0.096 | -0.339 | 0.146 | 0.436 |
| ENZRXN.20 | phosphate propanoyltransferase | 0 | Not detected | -0.046 | -0.300 | 0.209 | 0.725 |
| ENZRXN.20 | phosphate acetyltransferase | 0 | Not detected | -0.046 | -0.300 | 0.209 | 0.725 |

| ENZRXN.20 | propionate kinase | 0 | Not detected | 0.124 | -0.105 | 0.353 | 0.288 |
| --- | --- | --- | --- | --- | --- | --- | --- |
| ENZRXN.20 | thymine oxygenase | 0 | Not detected | -0.043 | -0.217 | 0.132 | 0.633 |
| ENZRXN.20 | hydroxybutyryl-coA dehydrogenase | 0 | Not detected | -0.020 | -0.197 | 0.157 | 0.825 |
| ENZRXN.20 | biotin carboxylase | 0 | Not detected | -0.106 | -0.378 | 0.166 | 0.446 |
| ENZRXN.20 | N,N'-diacetylbacilliosaminyl-1-phospha | 0 | Not detected | -0.285 | -0.569 | 0.000 | 0.050 |
| ENZRXN.20 | carboxyspermidine dehydrogenase | 0 | Not detected | -0.004 | -0.132 | 0.124 | 0.951 |
| ENZRXN.20 | N-carbamoylputrescine amidohydrolase | 0 | Not detected | -0.022 | -0.172 | 0.127 | 0.769 |
| ENZRXN.20 | 4,4'-diaponeurosporenal dehydrogenase | 0 | Not detected | 0.083 | -0.218 | 0.384 | 0.590 |
| ENZRXN.20 | superoxide dismutase | 0 | Not detected | 0.003 | -0.276 | 0.282 | 0.984 |
| ENZRXN.20 | UDP-N-acetyl-2-amino-2-deoxyglucuro | 0 | Not detected | -0.219 | -0.531 | 0.093 | 0.169 |
| ENZRXN.20 | UDP-2,3-diacetamido-2,3-dideoxyglucu | 0 | Not detected | 0.166 | -0.144 | 0.475 | 0.294 |
| ENZRXN.20 | enoyl-[acyl-carrier-protein] reductase (N | 0 | Not detected | -0.163 | -0.466 | 0.141 | 0.293 |
| ENZRXN.20 | enoyl-[acyl-carrier-protein] reductase (N | 0 | Not detected | -0.163 | -0.466 | 0.141 | 0.293 |
| ENZRXN.20 | crotonyl-[acyl-carrier-protein] reductase | 0 | Not detected | -0.163 | -0.466 | 0.141 | 0.293 |
| ENZRXN.20 | endo-1,4-Î²-xylanase | 0 | Not detected | 0.165 | -0.067 | 0.396 | 0.163 |
| ENZRXN.20 | D-fructose-6-phosphate cytidylyltransfe | 0 | Not detected | 0.081 | -0.216 | 0.378 | 0.593 |
| ENZRXN.21 | acetaldehyde dehydrogenase | 0 | Not detected | -0.095 | -0.348 | 0.158 | 0.462 |
| ENZRXN.21 | homoserine O-acetyltransferase | 0 | Not detected | 0.001 | -0.247 | 0.250 | 0.991 |
| ENZRXN.21 | methylthioribose-1-phosphate isomerase | 0 | Not detected | -0.105 | -0.398 | 0.188 | 0.483 |
| ENZRXN.21 | oligosaccharide reducing-end xylanase | 0 | Not detected | 0.131 | -0.079 | 0.342 | 0.221 |
| ENZRXN.21 | Î²-L-arabinofuranosidase | 0 | Not detected | 0.087 | -0.214 | 0.388 | 0.569 |
| ENZRXN.21 | uridine kinase | 0 | Not detected | 0.029 | -0.232 | 0.290 | 0.829 |
| ENZRXN.21 | cytidine kinase | 0 | Not detected | 0.029 | -0.232 | 0.290 | 0.829 |
| ENZRXN.21 | urocanate reductase | 0 | Not detected | 0.006 | -0.310 | 0.321 | 0.972 |
| ENZRXN.21 | 3D-(3,5/4)-trihydroxycyclohexane-1,2-d | 0 | Not detected | -0.051 | -0.251 | 0.149 | 0.615 |
| ENZRXN.21 | myo-inositol 2-dehydrogenase | 0 | Not detected | -0.007 | -0.256 | 0.241 | 0.954 |
| ENZRXN.21 | D-mannonate dehydrogenase | 0 | Not detected | -0.016 | -0.163 | 0.131 | 0.827 |
| ENZRXN.21 | D-mannonate dehydratase | 0 | Not detected | 0.029 | -0.110 | 0.169 | 0.679 |
| ENZRXN.21 | 2-keto-3-deoxygluconate kinase | 0 | Not detected | -0.002 | -0.135 | 0.130 | 0.973 |
| ENZRXN.21 | [sulfur carrier protein CysO]--sulfur liga | 0 | Not detected | 0.051 | -0.235 | 0.336 | 0.729 |
| ENZRXN.21 | N-acetyl-Î±-D-glucosaminyl-diphospho- | 0 | Not detected | -0.268 | -0.572 | 0.036 | 0.085 |
| ENZRXN.21 | glucose-1-phosphate thymidylyltransfer | 0 | Not detected | -0.161 | -0.389 | 0.067 | 0.167 |
| ENZRXN.21 | dTDP-glucose 4,6-dehydratase | 0 | Not detected | -0.011 | -0.228 | 0.207 | 0.923 |
| ENZRXN.21 | dTDP-4-amino-4,6-dideoxy-D-glucose t | 0 | Not detected | -0.119 | -0.440 | 0.202 | 0.468 |
| ENZRXN.21 | UDP-glucose 6-dehydrogenase | 0 | Not detected | -0.093 | -0.383 | 0.198 | 0.531 |
| ENZRXN.21 | UDP-N-acetylglucosamine 4,6-dehydrat | 0 | Not detected | 0.055 | -0.202 | 0.311 | 0.676 |
| ENZRXN.21 | UDP-2-acetamido-2,6-dideoxy-Î²-L-talo | 0 | Not detected | 0.176 | -0.128 | 0.480 | 0.256 |
| ENZRXN.21 | UDP-N-acetyl-Î²-L-rhamnosamine 2-epi | 0 | Not detected | -0.060 | -0.331 | 0.210 | 0.662 |
| ENZRXN.22 | UDP-N-acetylglucosamine 4,6-dehydrat | 0 | Not detected | 0.047 | -0.229 | 0.324 | 0.738 |
| ENZRXN.22 | Î²-L-arabinobiosidase | 0 | Not detected | 0.212 | -0.131 | 0.555 | 0.226 |
| ENZRXN.22 | butyryl-CoA dehydrogenase | 0 | Not detected | 0.038 | -0.103 | 0.179 | 0.597 |
| ENZRXN.22 | succinyl-CoA:acetate CoA-transferase | 0 | Not detected | 0.049 | -0.263 | 0.362 | 0.757 |
| ENZRXN.22 | propionyl-CoA:succinate CoA transfera | 0 | Not detected | 0.049 | -0.263 | 0.362 | 0.757 |
| ENZRXN.22 | N-acetyl-Î²-neuraminate:H+ symporter | 0 | Not detected | 0.180 | -0.020 | 0.379 | 0.078 |

| ENZRXN.22 | 1,4-Î²-mannanase | 0 | Not detected | -0.158 | -0.426 | 0.110 | 0.248 |
| --- | --- | --- | --- | --- | --- | --- | --- |
| ENZRXN.22 | mannobiose 2-epimerase | 0 | Not detected | -0.262 | -0.490 | -0.033 | 0.025 |
| ENZRXN.22 | cellobiose 2-epimerase | 0 | Not detected | -0.262 | -0.490 | -0.033 | 0.025 |
| ENZRXN.22 | cellobiose 2-epimerase | 0 | Not detected | 0.131 | -0.135 | 0.398 | 0.335 |
| ENZRXN.22 | UDP-acetamido-4-amino-6-deoxygalact | 0 | Not detected | -0.187 | -0.496 | 0.123 | 0.237 |
| ENZRXN.22 | 2-oxo-4-methylthiobutanoate-glutamine | 0 | Not detected | -0.010 | -0.249 | 0.229 | 0.934 |
| ENZRXN.23 | CoA-dependent (R)-lactaldehyde dehyd | 0 | Not detected | -0.096 | -0.339 | 0.146 | 0.436 |
| ENZRXN.23 | CoA-dependent (S)-lactaldehyde dehydr | 0 | Not detected | -0.096 | -0.339 | 0.146 | 0.436 |
| ENZRXN.23 | (R)-lactaldehyde reductase | 0 | Not detected | -0.023 | -0.192 | 0.146 | 0.791 |
| ENZRXN.23 | (S)-lactaldehyde reductase | 0 | Not detected | -0.023 | -0.192 | 0.146 | 0.791 |
| ENZRXN.23 | (R)-2,3-dihydroxy-3-methylbutanoate is | 0 | Not detected | 0.027 | -0.104 | 0.159 | 0.684 |
| ENZRXN.23 | (R)-2,3-dihydroxy-3-methylpentanoate i | 0 | Not detected | 0.027 | -0.104 | 0.159 | 0.684 |
| ENZRXN.23 | (R)-2,3-dihydroxy-3-methylbutanoate d | 0 | Not detected | -0.023 | -0.294 | 0.248 | 0.868 |
| ENZRXN.23 | (R)-2,3-dihydroxy-3-methylpentanoate d | 0 | Not detected | -0.023 | -0.294 | 0.248 | 0.868 |
| ENZRXN.23 | Fe-coproporphyrin synthase | 0 | Not detected | 0.127 | -0.159 | 0.414 | 0.384 |
| ENZRXN.23 | bacimethrin diphosphokinase | 0 | Not detected | -0.182 | -0.361 | -0.003 | 0.046 |
| ENZRXN.23 | L-galactose dehydrogenase | 0 | Not detected | -0.156 | -0.379 | 0.067 | 0.171 |
| ENZRXN.23 | L-galactonate 5-dehydrogenase | 0 | Not detected | -0.178 | -0.396 | 0.039 | 0.109 |
| ENZRXN.23 | N-acetylneuraminate lyase | 0 | Not detected | -0.234 | -0.436 | -0.031 | 0.024 |
| ENZRXN.23 | N-acetylglucosamine kinase | 0 | Not detected | -0.053 | -0.196 | 0.090 | 0.465 |
| ENZRXN.23 | 7-oxolithocholate reductase | 0 | Not detected | -0.023 | -0.277 | 0.231 | 0.858 |
| ENZRXN.23 | 7-oxolithocholate reductase | 0 | Not detected | -0.023 | -0.277 | 0.231 | 0.858 |
| ENZRXN.23 | GDP-6-deoxy-4-keto-D-arabino-heptose | 0 | Not detected | -0.162 | -0.362 | 0.038 | 0.112 |
| ENZRXN.23 | UDP-N-acetylglucosamine 6-dehydroge | 0 | Not detected | -0.159 | -0.370 | 0.051 | 0.138 |
| ENZRXN.23 | UDP-N-acetylgalactosamine 6-dehydrog | 0 | Not detected | -0.159 | -0.370 | 0.051 | 0.138 |
| ENZRXN.23 | Kdo2-lipid A phosphotransferase | 0 | Not detected | -0.113 | -0.352 | 0.127 | 0.357 |
| ENZRXN.23 | Kdo2-lipid A phosphoethanolamine 7''-t | 0 | Not detected | 0.180 | -0.039 | 0.399 | 0.107 |
| ENZRXN.23 | phosphomannomutase | 0 | Not detected | -0.061 | -0.357 | 0.234 | 0.684 |
| ENZRXN.23 | phosphoglucomutase | 0 | Not detected | -0.061 | -0.357 | 0.234 | 0.684 |
| ENZRXN.23 | heparin lyase | 0 | Not detected | -0.291 | -0.672 | 0.090 | 0.134 |
| ENZRXN.23 | heparan sulfate lyase | 0 | Not detected | -0.291 | -0.672 | 0.090 | 0.134 |
| ENZRXN.23 | heparin lyase | 0 | Not detected | -0.341 | -0.625 | -0.057 | 0.019 |
| ENZRXN.23 | unsaturated hyaluronan disaccharide hyd | 0 | Not detected | -0.135 | -0.381 | 0.110 | 0.281 |
| ENZRXN.23 | unsaturated chondroitin disaccharide hy | 0 | Not detected | -0.135 | -0.381 | 0.110 | 0.281 |
| ENZRXN.23 | 4-deoxy-Î²-L-erythro-hex-4-enopyranuro | 0 | Not detected | 0.170 | -0.132 | 0.472 | 0.270 |
| ENZRXN.23 | 4-deoxy-Î²-D-gluc-4-enuronosyl-(1,43)- | 0 | Not detected | 0.170 | -0.132 | 0.472 | 0.270 |
| ENZRXN.23 | 4-deoxy-Î²-D-gluc-4-enuronosyl-(1,4)-N | 0 | Not detected | 0.170 | -0.132 | 0.472 | 0.270 |
| ENZRXN.23 | 4-deoxy-Î²-L-erythro-hex-4-enopyranuro | 0 | Not detected | 0.170 | -0.132 | 0.472 | 0.270 |
| ENZRXN.23 | (5Z)-3-oxo-tetradec-5-enoyl-[acyl-carrie | 0 | Not detected | -0.019 | -0.156 | 0.117 | 0.781 |
| ENZRXN.23 | (5Z)-3-oxo-tetradec-5-enoyl-[acyl-carrie | 0 | Not detected | 0.154 | -0.093 | 0.402 | 0.222 |
| ENZRXN.23 | (2E,5Z)-tetradeca-2,5-dienoyl-[acp] red | 0 | Not detected | 0.029 | -0.185 | 0.244 | 0.791 |
| ENZRXN.23 | (3R)-3-hydroxydecanoyl-[acp] dehydrat | 0 | Not detected | -0.048 | -0.257 | 0.162 | 0.657 |
| ENZRXN.23 | (2E)-dec-2-enoyl-[acp] isomerase | 0 | Not detected | -0.048 | -0.257 | 0.162 | 0.657 |
| ENZRXN.23 | (5Z)-3-oxo-dodec-5-enoyl-[acp] synthas | 0 | Not detected | 0.001 | -0.131 | 0.133 | 0.984 |

| ENZRXN.23 | (7Z)-3-oxo-hexadec-7-enoyl-[acp] synth | 0 | Not detected | -0.019 | -0.156 | 0.117 | 0.781 |
| --- | --- | --- | --- | --- | --- | --- | --- |
| ENZRXN.23 | (9Z)-3-oxo-octadec-9-enoyl-[acp] synth | 0 | Not detected | -0.019 | -0.156 | 0.117 | 0.781 |
| ENZRXN.23 | (7Z)-3-oxo-hexadec-7-enoyl-[acp] reduc | 0 | Not detected | 0.154 | -0.093 | 0.402 | 0.222 |
| ENZRXN.23 | (9Z)-3-oxo-octadec-9-enoyl-[acp] reduc | 0 | Not detected | 0.154 | -0.093 | 0.402 | 0.222 |
| ENZRXN.23 | (11Z)-3-oxo-icos-11-enoyl-[acp] reduct | 0 | Not detected | 0.154 | -0.093 | 0.402 | 0.222 |
| ENZRXN.23 | (11Z)-3-oxo-icos-11-enoyl-[acp] syntha | 0 | Not detected | -0.019 | -0.156 | 0.117 | 0.781 |
| ENZRXN.23 | (2E,7Z)-hexadeca-2,7-dienoyl-[acp] | 0 | Not detected | 0.029 | -0.185 | 0.244 | 0.791 |
| ENZRXN.23 | (2E,9Z)-octadeca-2,9-dienoyl-[acp] redu | 0 | Not detected | 0.029 | -0.185 | 0.244 | 0.791 |
| ENZRXN.23 | (2E,11Z)-icosa-2,11-dienoyl-[acp] redu | 0 | Not detected | 0.029 | -0.185 | 0.244 | 0.791 |
| ENZRXN.23 | lactose 2-epimerase | 0 | Not detected | 0.131 | -0.135 | 0.398 | 0.335 |
| ENZRXN.23 | 8-amino-3,8-dideoxy-manno-octulosona | 0 | Not detected | -0.058 | -0.321 | 0.205 | 0.666 |
| ENZRXN.24 | scaffold protein for iron-sulfur cluster as | 0 | Not detected | -0.319 | -0.580 | -0.058 | 0.017 |
| ENZRXN.24 | lactate racemase | 0 | Not detected | 0.045 | -0.191 | 0.281 | 0.708 |
| ENZRXN.24 | UDP-4-amino-4,6-dideoxy-N-acetyl-Î²- | 0 | Not detected | -0.123 | -0.444 | 0.199 | 0.454 |
| ENZRXN.24 | guanosine phosphorylase | 0 | Not detected | -0.253 | -0.552 | 0.047 | 0.098 |
| ENZRXN.24 | deoxycholate 3Î±-dehydrogenase | 0 | Not detected | 0.097 | -0.108 | 0.302 | 0.355 |
| ENZRXN.24 | deoxycholate 3Î±-dehydrogenase | 0 | Not detected | 0.059 | -0.242 | 0.359 | 0.701 |
| ENZRXN.24 | 3-oxochol-4,6-dien-24-oyl-CoA 6-reduc | 0 | Not detected | 0.222 | -0.064 | 0.508 | 0.128 |
| ENZRXN.24 | lithocholate 3Î±-dehydrogenase | 0 | Not detected | 0.059 | -0.242 | 0.359 | 0.701 |
| ENZRXN.24 | chenodeoxycholate 3Î±-dehydrogenase | 0 | Not detected | 0.059 | -0.242 | 0.359 | 0.701 |
| ENZRXN.24 | cholate 3Î±-dehydrogenase | 0 | Not detected | 0.059 | -0.242 | 0.359 | 0.701 |
| ENZRXN.24 | lithocholate 3Î±-dehydrogenase | 0 | Not detected | 0.097 | -0.108 | 0.302 | 0.355 |
| ENZRXN.24 | cholate 3Î±-dehydrogenase | 0 | Not detected | 0.097 | -0.108 | 0.302 | 0.355 |
| ENZRXN.24 | chenodeoxycholate 3Î±-dehydrogenase | 0 | Not detected | 0.097 | -0.108 | 0.302 | 0.355 |
| ENZRXN.24 | Î²-1,2-mannosidase | 0 | Not detected | -0.248 | -0.500 | 0.004 | 0.053 |
| ENZRXN.24 | L-galacto-1,5-lactonase | 0 | Not detected | -0.238 | -0.451 | -0.024 | 0.029 |
| ENZRXN.24 | succinate:malate antiport | 0 | Not detected | -0.048 | -0.303 | 0.207 | 0.711 |
| ENZRXN.24 | succinate:L-aspartate antiport | 0 | Not detected | -0.048 | -0.303 | 0.207 | 0.711 |
| ENZRXN.24 | malate:succinate antiporter | 0 | Not detected | -0.114 | -0.333 | 0.105 | 0.307 |
| ENZRXN.24 | L-aspartate:succinate antiporter | 0 | Not detected | -0.114 | -0.333 | 0.105 | 0.307 |
| ENZRXN.24 | dihydroorotate dehydrogenase | 0 | Not detected | 0.002 | -0.294 | 0.297 | 0.992 |
| ENZRXN.24 | methionine &gamma;-lyase | 0 | Not detected | 0.004 | -0.260 | 0.269 | 0.976 |
| ENZRXN.24 | [HypE]-S-carboxamide dehydratase | 0 | Not detected | 0.059 | -0.145 | 0.262 | 0.573 |
| ENZRXN.25 | Î±-D-ribose-1-(2-N-acetamidomethylph | 0 | Not detected | 0.196 | -0.003 | 0.394 | 0.053 |
| ENZRXN.25 | carbon-phosphorous lyase | 0 | Not detected | 0.133 | -0.096 | 0.363 | 0.255 |
| ENZRXN.25 | Î±-D-ribose-1-[N-(phosphonomethyl)gl | 0 | Not detected | -0.026 | -0.246 | 0.194 | 0.817 |
| ENZRXN.25 | Î±-D-ribose-1-[N-(phosphonomethyl)gl | 0 | Not detected | -0.258 | -0.520 | 0.003 | 0.053 |
| ENZRXN.25 | adenine phosphoribosyltransferase | 0 | Not detected | 0.017 | -0.265 | 0.299 | 0.906 |
| ENZRXN.25 | tetracycline 11a-oxygenase | 0 | Not detected | -0.043 | -0.434 | 0.348 | 0.830 |
| ENZRXN.25 | tigecycline 11a-momooxygenase | 0 | Not detected | -0.043 | -0.434 | 0.348 | 0.830 |
| ENZRXN.25 | UDP-N-acetylglucosamine 2-epimerase | 0 | Not detected | 0.059 | -0.242 | 0.361 | 0.699 |
| ENZRXN.25 | ribitol-5-phosphate cytidylyltransferase | 0 | Not detected | 0.076 | -0.224 | 0.376 | 0.618 |
| ENZRXN.25 | ribitol-5-phosphate 1-dehydrogenase | 0 | Not detected | 0.122 | -0.176 | 0.421 | 0.423 |
| ENZRXN.25 | ribitol-5-phosphate 1-dehydrogenase | 0 | Not detected | 0.060 | -0.251 | 0.370 | 0.707 |

| ENZRXN.25 | UTP--glucose-1-phosphate uridylyltrans | 0 | Not detected | -0.078 | -0.364 | 0.207 | 0.592 |
| --- | --- | --- | --- | --- | --- | --- | --- |
| ENZRXN.25 | undecaprenyl-phosphate 2-acetamido-4- | 0 | Not detected | -0.019 | -0.294 | 0.257 | 0.895 |
| ENZRXN.25 | D-alanine--[D-alanyl carrier protein] lig | 0 | Not detected | 0.068 | -0.222 | 0.357 | 0.647 |
| ENZRXN.25 | PG:teichoic acid D-alanyltransferase | 0 | Not detected | 0.099 | -0.166 | 0.365 | 0.463 |
| ENZRXN.25 | D-alanine carrier protein:PG D-alanyltra | 0 | Not detected | 0.006 | -0.258 | 0.269 | 0.965 |
| ENZRXN.25 | 1,2-diacylglycerol 3-Î±-glucosyltransfer | 0 | Not detected | -0.055 | -0.306 | 0.197 | 0.670 |
| ENZRXN.25 | isopropylmalate dehydrogenase | 0 | Not detected | 0.035 | -0.108 | 0.179 | 0.629 |
| ENZRXN.25 | tRNA uridine 4-sulfurtransferase | 0 | Not detected | -0.064 | -0.281 | 0.152 | 0.561 |
| ENZRXN.25 | aminobutyraldehyde dehydrogenase | 0 | Not detected | 0.102 | -0.085 | 0.289 | 0.285 |
| ENZRXN.25 | succinate-semialdehyde dehydrogenase | 0 | Not detected | 0.102 | -0.085 | 0.289 | 0.285 |
| ENZRXN.25 | 7,8-dihydro-8-oxoguanine DNA glycosy | 0 | Not detected | -0.069 | -0.254 | 0.116 | 0.466 |
| ENZRXN.25 | [sulfur carrier protein TtuB] adenylyltra | 0 | Not detected | -0.206 | -0.466 | 0.054 | 0.121 |
| ENZRXN.25 | trans-4-hydroxy-L-proline dehydratase | 0 | Not detected | -0.079 | -0.245 | 0.087 | 0.351 |
| ENZRXN.25 | menaquinone C8-methyltransferase | 0 | Not detected | -0.115 | -0.428 | 0.198 | 0.471 |
| ENZRXN.25 | quinol--ferredoxin oxidoreductase | 0 | Not detected | -0.019 | -0.330 | 0.291 | 0.903 |
| ENZRXN.25 | dissimilatory sulfite reductase | 0 | Not detected | 0.009 | -0.310 | 0.328 | 0.956 |
| ENZRXN.25 | L-threonate dehydrogenase | 0 | Not detected | -0.059 | -0.238 | 0.121 | 0.523 |
| ENZRXN.25 | 2-dehydro-D-threonate isomerase | 0 | Not detected | -0.008 | -0.176 | 0.160 | 0.927 |
| ENZRXN.25 | 2-dehydro-L-threonate isomerase | 0 | Not detected | -0.008 | -0.176 | 0.160 | 0.927 |
| ENZRXN.25 | 4-phospho-D-erythronate 3-dehydrogen | 0 | Not detected | -0.017 | -0.293 | 0.260 | 0.906 |
| ENZRXN.25 | UDP-N-acetylmuramate--L-alanyl-gam | 0 | Not detected | -0.217 | -0.532 | 0.097 | 0.176 |
| ENZRXN.25 | methenyltetrahydrofolate cyclohydrolase | 0 | Not detected | -0.115 | -0.263 | 0.033 | 0.128 |
| ENZRXN.25 | methylenetetrahydrofolate dehydrogenas | 0 | Not detected | -0.115 | -0.263 | 0.033 | 0.128 |
| ENZRXN.25 | deoxyribodipyrimidine photolyase | 0 | Not detected | 0.043 | -0.156 | 0.242 | 0.671 |
| ENZRXN.25 | deoxyribodipyrimidine photolyase | 0 | Not detected | 0.043 | -0.156 | 0.242 | 0.671 |
| ENZRXN.25 | pyridinium-3,5-biscarboxylic acid mono | 0 | Not detected | 0.020 | -0.174 | 0.213 | 0.843 |
| ENZRXN.26 | acetate kinase | 0 | Not detected | 0.006 | -0.164 | 0.176 | 0.941 |
| ENZRXN.26 | acetaldehyde dehydrogenase (acetylating | 0 | Not detected | 0.068 | -0.178 | 0.313 | 0.589 |
| ENZRXN.26 | glyoxal reductase | 0 | Not detected | 0.023 | -0.235 | 0.281 | 0.859 |
| ENZRXN.26 | methylglyoxal reductase | 0 | Not detected | 0.023 | -0.235 | 0.281 | 0.859 |
| ENZRXN.26 | 3-oxochol-4-en-24-oyl-CoA 4-reductase | 0 | Not detected | 0.222 | -0.064 | 0.508 | 0.128 |
| ENZRXN.26 | 12Î±-hydroxy-3-oxochol-4-en-24-oyl-C | 0 | Not detected | 0.222 | -0.064 | 0.508 | 0.128 |
| ENZRXN.26 | 3-hydroxyhexadecanoyl-CoA:glycine 3- | 0 | Not detected | -0.012 | -0.159 | 0.134 | 0.868 |
| ENZRXN.26 | diaminohydroxyphosphoribosylaminopy | 0 | Not detected | 0.057 | -0.204 | 0.317 | 0.671 |
| ENZRXN.26 | 5-amino-6-(5-phosphoribosylamino)ura | 0 | Not detected | 0.057 | -0.204 | 0.317 | 0.671 |
| ENZRXN.26 | 1,2-Î±-L-arabinofuranosidase | 0 | Not detected | -0.002 | -0.138 | 0.134 | 0.979 |
| ENZRXN.26 | 2-hydroxyethylphosphonate cytidylyltra | 0 | Not detected | -0.039 | -0.336 | 0.258 | 0.797 |
| ENZRXN.26 | acetate kinase | 0 | Not detected | 0.014 | -0.143 | 0.170 | 0.864 |
| ENZRXN.26 | thiol-disulfide exchange | 0 | Not detected | 0.129 | -0.091 | 0.350 | 0.249 |
| ENZRXN.26 | phosphofructokinase | 0 | Not detected | 0.093 | -0.078 | 0.264 | 0.288 |
| ENZRXN.28 | fructose-bisphosphate aldolase | 0 | Not detected | 0.020 | -0.115 | 0.155 | 0.772 |
| ENZRXN.29 | phospho-Î²-galactosidase | 0 | Not detected | 0.211 | -0.075 | 0.497 | 0.148 |
| ENZRXN.33 | aspartokinase | 0 | Not detected | -0.124 | -0.415 | 0.168 | 0.406 |
| ENZRXN.44 | GDP-4-keto-6-deoxymannose-3,5-epim | 0 | Not detected | -0.035 | -0.323 | 0.254 | 0.815 |

| ENZRXN.58 | isoamylase | 0 | Not detected | 0.274 | -0.032 | 0.579 | 0.080 |
| --- | --- | --- | --- | --- | --- | --- | --- |
| ENZRXN.60 | trehalose-6-phosphate hydrolase | 0 | Not detected | -0.079 | -0.234 | 0.076 | 0.319 |
| ENZRXN.61 | 16S rRNA m2G1207 methyltransferase | 0 | Not detected | -0.060 | -0.248 | 0.128 | 0.532 |
| ENZRXN.62 | Î±-phosphoglucomutase | 0 | Not detected | 0.017 | -0.221 | 0.254 | 0.891 |
| ENZRXN.63 | UDP-glucose-4-epimerase | 0 | Not detected | 0.084 | -0.114 | 0.281 | 0.406 |
| ENZRXN.67 | N-succinyldiaminopimelate-aminotransf | 0 | Not detected | -0.072 | -0.348 | 0.204 | 0.609 |
| ENZRXN.68 | aspartate kinase | 0 | Not detected | -0.091 | -0.345 | 0.164 | 0.485 |
| ENZRXN.71 | 6-phosphogluconate dehydrogenase (de | 0 | Not detected | -0.097 | -0.300 | 0.107 | 0.351 |
| ENZRXN.78 | phosphoribosylamidoimidazole-succino | 0 | Not detected | 0.019 | -0.275 | 0.314 | 0.898 |
| ENZRXN.80 | nitric oxide dioxygenase | 0 | Not detected | -0.113 | -0.296 | 0.071 | 0.228 |
| ENZRXN.89 | UDP-N-acetylglucosamine 2-epimerase | 0 | Not detected | -0.010 | -0.179 | 0.159 | 0.905 |
| ENZRXN.90 | UTP-glucose-1-phosphate uridylyltransf | 0 | Not detected | 0.025 | -0.168 | 0.217 | 0.801 |
| ENZRXN.93 | NAD-dependent glyceraldehyde-3-phos | 0 | Not detected | -0.115 | -0.401 | 0.170 | 0.427 |
| ENZRXN.95 | O-acetylhomoserine sulfhydrylase | 0 | Not detected | 0.008 | -0.117 | 0.134 | 0.895 |
| ENZRXN0.1 | lipoyl-adenylate--protein ligase | 0 | Not detected | -0.011 | -0.177 | 0.155 | 0.897 |
| ENZRXN0.1 | 23S rRNA m5U1939 methyltransferase | 0 | Not detected | 0.102 | -0.091 | 0.294 | 0.301 |
| ENZRXN0.1 | GTPase | 0 | Not detected | -0.103 | -0.308 | 0.103 | 0.329 |
| ENZRXN0.1 | lipoate acetyltransferase | 0 | Not detected | -0.019 | -0.258 | 0.220 | 0.877 |
| ENZRXN0.1 | lipoate-apo-GcvH protein ligase | 0 | Not detected | -0.011 | -0.177 | 0.155 | 0.897 |
| ENZRXN0.1 | protein-(glutamine-N5) methyltransferas | 0 | Not detected | 0.100 | -0.075 | 0.274 | 0.263 |
| ENZRXN0.1 | protein-glutamine N-methyltransferase | 0 | Not detected | -0.081 | -0.265 | 0.104 | 0.392 |
| ENZRXN0.1 | propanoate:CoA ligase (AMP-forming) | 0 | Not detected | 0.028 | -0.218 | 0.275 | 0.823 |
| ENZRXN0.1 | tRNA-dihydrouridine synthase | 0 | Not detected | -0.052 | -0.315 | 0.211 | 0.699 |
| ENZRXN0.1 | tRNA-dihydrouridine synthase | 0 | Not detected | -0.149 | -0.346 | 0.047 | 0.136 |
| ENZRXN0.1 | tRNA-dihydrouridine16 synthase | 0 | Not detected | 0.069 | -0.116 | 0.255 | 0.464 |
| ENZRXN0.1 | tRNA(m5U54)methyltransferase | 0 | Not detected | 0.058 | -0.153 | 0.269 | 0.590 |
| ENZRXN0.1 | deoxyribodipyrimidine photolyase | 0 | Not detected | 0.043 | -0.156 | 0.242 | 0.671 |
| ENZRXN0.1 | O-6-methylguanine-DNA methyltransfe | 0 | Not detected | 0.100 | -0.079 | 0.280 | 0.275 |
| ENZRXN0.1 | CCA tRNA nucleotidyltransferase | 0 | Not detected | -0.042 | -0.252 | 0.169 | 0.699 |
| ENZRXN0.1 | DNA adenine methyltransferase | 0 | Not detected | -0.208 | -0.405 | -0.012 | 0.038 |
| ENZRXN0.1 | tRNA dimethylallyltransferase | 0 | Not detected | -0.145 | -0.382 | 0.091 | 0.228 |
| ENZRXN0.1 | tRNA preQ134 S-adenosylmethionine ri | 0 | Not detected | -0.102 | -0.252 | 0.048 | 0.185 |
| ENZRXN0.1 | ribose 1,5-bisphosphate phosphokinase | 0 | Not detected | 0.022 | -0.138 | 0.182 | 0.787 |
| ENZRXN0.1 | TDP-N-acetylfucosamine:lipid II N-acet | 0 | Not detected | 0.130 | -0.065 | 0.326 | 0.192 |
| ENZRXN0.1 | ferroxidase | 0 | Not detected | 0.155 | -0.047 | 0.358 | 0.133 |
| ENZRXN0.1 | inosine:H+ antiporter | 0 | Not detected | -0.001 | -0.196 | 0.194 | 0.995 |
| ENZRXN0.2 | carboxylesterase | 0 | Not detected | 0.081 | -0.090 | 0.253 | 0.353 |
| ENZRXN0.2 | hydroxylamine reductase | 0 | Not detected | 0.113 | -0.092 | 0.319 | 0.280 |
| ENZRXN0.2 | diadenosine tetraphosphatase | 0 | Not detected | 0.032 | -0.154 | 0.218 | 0.736 |
| ENZRXN0.2 | DNA-cytosine methyltransferase | 0 | Not detected | 0.073 | -0.116 | 0.261 | 0.450 |
| ENZRXN0.2 | phosphatidylglycerol&mdash;prolipopro | 0 | Not detected | 0.042 | -0.197 | 0.281 | 0.729 |
| ENZRXN0.2 | enterochelin esterase | 0 | Not detected | 0.012 | -0.170 | 0.194 | 0.897 |
| ENZRXN0.2 | limit dextrin Î±-1,6-glucohydrolase | 0 | Not detected | -0.028 | -0.264 | 0.209 | 0.819 |
| ENZRXN0.2 | ferric dihyroxybenzoylserine outer mem | 0 | Not detected | 0.035 | -0.161 | 0.232 | 0.725 |

| ENZRXN0.2 | UDP-4-amino-4-deoxy-L-arabinose ami | 0 | Not detected | 0.048 | -0.133 | 0.230 | 0.600 |
| --- | --- | --- | --- | --- | --- | --- | --- |
| ENZRXN0.2 | guanosine:H+ antiporter | 0 | Not detected | -0.001 | -0.196 | 0.194 | 0.995 |
| ENZRXN0.2 | Î²-N-acetylglucosaminidase | 0 | Not detected | -0.008 | -0.203 | 0.188 | 0.939 |
| ENZRXN0.2 | guanine deaminase | 0 | Not detected | -0.040 | -0.254 | 0.175 | 0.716 |
| ENZRXN0.2 | crotonyl-CoA hydratase | 0 | Not detected | -0.077 | -0.278 | 0.125 | 0.457 |
| ENZRXN0.2 | L-ribulose-5-phosphate 4-epimerase | 0 | Not detected | 0.065 | -0.115 | 0.245 | 0.477 |
| ENZRXN0.2 | dihydropteridine reductase | 0 | Not detected | -0.113 | -0.296 | 0.071 | 0.228 |
| ENZRXN0.2 | 23S rRNA pseudouridine746 synthase | 0 | Not detected | -0.191 | -0.374 | -0.007 | 0.042 |
| ENZRXN0.2 | 23S rRNA pseudouridine2604 synthase | 0 | Not detected | -0.014 | -0.179 | 0.150 | 0.864 |
| ENZRXN0.2 | 23S rRNA pseudouridine2457 synthase | 0 | Not detected | 0.163 | -0.061 | 0.387 | 0.153 |
| ENZRXN0.2 | 23S rRNA pseudouridine955/2504/2580 | 0 | Not detected | 0.026 | -0.189 | 0.242 | 0.812 |
| ENZRXN0.2 | inositol-phosphate phosphatase | 0 | Not detected | 0.067 | -0.145 | 0.279 | 0.536 |
| ENZRXN0.2 | Î²-keto ester reductase | 0 | Not detected | -0.020 | -0.191 | 0.151 | 0.816 |
| ENZRXN0.2 | propionyl-CoA:succinate CoA transfera | 0 | Not detected | -0.158 | -0.337 | 0.021 | 0.083 |
| ENZRXN0.2 | tRNAIle-lysidine synthetase | 0 | Not detected | 0.054 | -0.146 | 0.254 | 0.597 |
| ENZRXN0.2 | 23S rRNA pseudouridine1911/1915/191 | 0 | Not detected | -0.033 | -0.251 | 0.185 | 0.768 |
| ENZRXN0.2 | lipid IVA 4-amino-4-deoxy-L-arabinosy | 0 | Not detected | -0.012 | -0.208 | 0.183 | 0.901 |
| ENZRXN0.2 | 3-hydroxyacyl-CoA dehydrogenase | 0 | Not detected | 0.042 | -0.172 | 0.256 | 0.702 |
| ENZRXN0.2 | dodecenoyl-CoA D-isomerase | 0 | Not detected | 0.042 | -0.172 | 0.256 | 0.702 |
| ENZRXN0.2 | 3-hydroxybutyryl-CoA epimerase | 0 | Not detected | 0.042 | -0.172 | 0.256 | 0.702 |
| ENZRXN0.2 | acetyl-CoA C-acyltransferase | 0 | Not detected | -0.043 | -0.234 | 0.148 | 0.659 |
| ENZRXN0.2 | short chain acyl-CoA synthetase | 0 | Not detected | 0.136 | -0.104 | 0.375 | 0.267 |
| ENZRXN0.2 | enoyl-CoA hydratase | 0 | Not detected | 0.042 | -0.172 | 0.256 | 0.702 |
| ENZRXN0.2 | tyrosine kinase | 0 | Not detected | 0.173 | -0.031 | 0.378 | 0.097 |
| ENZRXN0.2 | tRNA-specific 2-thiouridylase | 0 | Not detected | 0.056 | -0.154 | 0.266 | 0.600 |
| ENZRXN0.2 | 3-hydroxyadipyl-CoA dehydrogenase | 0 | Not detected | -0.081 | -0.263 | 0.100 | 0.379 |
| ENZRXN0.2 | D-allulose-6-phosphate 3-epimerase | 0 | Not detected | -0.086 | -0.375 | 0.202 | 0.557 |
| ENZRXN0.2 | cardiolipin synthase | 0 | Not detected | 0.115 | -0.060 | 0.291 | 0.198 |
| ENZRXN0.2 | NADH cupric reductase | 0 | Not detected | 0.041 | -0.161 | 0.242 | 0.693 |
| ENZRXN0.2 | DNA ligase | 0 | Not detected | 0.015 | -0.204 | 0.235 | 0.891 |
| ENZRXN0.2 | DNA ligase | 0 | Not detected | -0.091 | -0.380 | 0.197 | 0.534 |
| ENZRXN0.2 | peptidyl-prolyl cis-trans isomerase | 0 | Not detected | -0.084 | -0.347 | 0.178 | 0.529 |
| ENZRXN0.2 | peptidyl-prolyl cis-trans isomerase | 0 | Not detected | 0.061 | -0.200 | 0.323 | 0.645 |
| ENZRXN0.2 | UDP-2,3-diacylglucosamine diphosphat | 0 | Not detected | -0.043 | -0.211 | 0.125 | 0.615 |
| ENZRXN0.2 | pyruvate-flavodoxin oxidoreductase | 0 | Not detected | 0.159 | -0.173 | 0.491 | 0.347 |
| ENZRXN0.2 | malate:quinone oxidoreductase | 0 | Not detected | 0.049 | -0.159 | 0.257 | 0.645 |
| ENZRXN0.2 | fructose-6-phosphate aldolase | 0 | Not detected | 0.049 | -0.113 | 0.210 | 0.556 |
| ENZRXN0.2 | tRNA m7G46 methyltransferase | 0 | Not detected | 0.038 | -0.202 | 0.278 | 0.756 |
| ENZRXN0.2 | 10-formyltetrahydrofolate:L-methionyl-t | 0 | Not detected | -0.195 | -0.452 | 0.062 | 0.137 |
| ENZRXN0.2 | tRNA (Gm18) 2'-O-methyltransferase | 0 | Not detected | 0.034 | -0.131 | 0.198 | 0.689 |
| ENZRXN0.3 | RNase R | 0 | Not detected | -0.022 | -0.282 | 0.239 | 0.871 |
| ENZRXN0.3 | fructokinase | 0 | Not detected | 0.013 | -0.158 | 0.185 | 0.878 |
| ENZRXN0.3 | tRNA 2-selenouridine synthase | 0 | Not detected | 0.164 | -0.021 | 0.348 | 0.082 |
| ENZRXN0.3 | endo-1,4-D-glucanase | 0 | Not detected | 0.034 | -0.152 | 0.219 | 0.723 |

| ENZRXN0.3 | UDP-N-acetylmuramate:L-alanyl-&gam | 0 | Not detected | -0.195 | -0.464 | 0.074 | 0.155 |
| --- | --- | --- | --- | --- | --- | --- | --- |
| ENZRXN0.3 | 23S rRNA m5U747 methyltransferase | 0 | Not detected | -0.047 | -0.248 | 0.155 | 0.651 |
| ENZRXN0.3 | indoleglycerol phosphate aldolase | 0 | Not detected | 0.013 | -0.163 | 0.189 | 0.883 |
| ENZRXN0.3 | L-threonine:H+ antiporter | 0 | Not detected | 0.095 | -0.128 | 0.317 | 0.405 |
| ENZRXN0.3 | L-homoserine:H+ antiporter | 0 | Not detected | 0.095 | -0.128 | 0.317 | 0.405 |
| ENZRXN0.3 | Fe2+:H+ symporter | 0 | Not detected | -0.008 | -0.186 | 0.170 | 0.929 |
| ENZRXN0.3 | N-acetylmuramyl-L-alanine amidase | 0 | Not detected | -0.087 | -0.281 | 0.107 | 0.380 |
| ENZRXN0.3 | tRNA pseudouridine13 synthase | 0 | Not detected | 0.122 | -0.060 | 0.304 | 0.188 |
| ENZRXN0.3 | tRNA pseudouridine55 synthase | 0 | Not detected | -0.118 | -0.310 | 0.074 | 0.227 |
| ENZRXN0.3 | tRNA pseudouridine65 synthase | 0 | Not detected | -0.094 | -0.267 | 0.079 | 0.287 |
| ENZRXN0.3 | methionine aminopeptidase | 0 | Not detected | 0.145 | -0.134 | 0.424 | 0.307 |
| ENZRXN0.3 | peptide deformylase | 0 | Not detected | -0.100 | -0.311 | 0.112 | 0.357 |
| ENZRXN0.3 | DNA polymerase | 0 | Not detected | 0.128 | -0.065 | 0.322 | 0.193 |
| ENZRXN0.3 | 16S rRNA m62A1518,m62A1519 dime | 0 | Not detected | 0.054 | -0.163 | 0.272 | 0.623 |
| ENZRXN0.3 | undecaprenyl pyrophosphate phosphatas | 0 | Not detected | 0.026 | -0.191 | 0.243 | 0.814 |
| ENZRXN0.3 | 16S rRNA m5C967 methyltransferase | 0 | Not detected | 0.048 | -0.175 | 0.271 | 0.675 |
| ENZRXN0.3 | topoisomerase | 0 | Not detected | -0.033 | -0.302 | 0.235 | 0.808 |
| ENZRXN0.4 | topoisomerase | 0 | Not detected | -0.090 | -0.309 | 0.130 | 0.425 |
| ENZRXN0.4 | methylene-tetrahydrofolylpolyglutamate | 0 | Not detected | -0.017 | -0.216 | 0.182 | 0.867 |
| ENZRXN0.4 | 2,3-dihydroxybenzoate:oxygen oxidored | 0 | Not detected | 0.155 | -0.047 | 0.358 | 0.133 |
| ENZRXN0.4 | cuprous oxidase | 0 | Not detected | 0.155 | -0.047 | 0.358 | 0.133 |
| ENZRXN0.4 | uridine nucleosidase | 0 | Not detected | -0.104 | -0.287 | 0.078 | 0.263 |
| ENZRXN0.4 | oligopeptidase | 0 | Not detected | 0.109 | -0.087 | 0.304 | 0.277 |
| ENZRXN0.4 | 23S rRNA 2'-O-ribose U2552 methyltra | 0 | Not detected | 0.030 | -0.169 | 0.230 | 0.764 |
| ENZRXN0.4 | oligopeptidase | 0 | Not detected | 0.043 | -0.224 | 0.309 | 0.754 |
| ENZRXN0.4 | tail-specific protease | 0 | Not detected | -0.181 | -0.410 | 0.048 | 0.122 |
| ENZRXN0.4 | protease | 0 | Not detected | 0.008 | -0.234 | 0.251 | 0.947 |
| ENZRXN0.4 | alanyl aminopeptidase | 0 | Not detected | -0.075 | -0.321 | 0.172 | 0.553 |
| ENZRXN0.4 | alkaline phosphatase aminopeptidase | 0 | Not detected | 0.143 | -0.033 | 0.320 | 0.111 |
| ENZRXN0.4 | peptidyl-dipeptidase | 0 | Not detected | 0.141 | -0.058 | 0.339 | 0.165 |
| ENZRXN0.4 | formate dehydrogenase | 0 | Not detected | -0.044 | -0.301 | 0.214 | 0.741 |
| ENZRXN0.4 | meso-diaminopimelic acid-D-alanyl end | 0 | Not detected | -0.051 | -0.251 | 0.148 | 0.613 |
| ENZRXN0.4 | D-alanyl-D-alanine carboxypeptidase | 0 | Not detected | 0.090 | -0.086 | 0.266 | 0.318 |
| ENZRXN0.4 | exoribonuclease II | 0 | Not detected | -0.032 | -0.262 | 0.198 | 0.786 |
| ENZRXN0.5 | methionine-S-sulfoxide reductase | 0 | Not detected | 0.068 | -0.166 | 0.302 | 0.570 |
| ENZRXN0.5 | 5'-nucleotidase | 0 | Not detected | 0.098 | -0.086 | 0.283 | 0.296 |
| ENZRXN0.5 | 3'-nucleotidase | 0 | Not detected | 0.098 | -0.086 | 0.283 | 0.296 |
| ENZRXN0.5 | pyrimidine 5'-nucleotidase | 0 | Not detected | 0.000 | -0.164 | 0.164 | 0.997 |
| ENZRXN0.5 | &gamma;-glutamylputrescine oxidase | 0 | Not detected | 0.144 | -0.042 | 0.329 | 0.129 |
| ENZRXN0.5 | &gamma;-glutamyl-&gamma;-aminobut | 0 | Not detected | 0.102 | -0.085 | 0.289 | 0.285 |
| ENZRXN0.5 | octanoul-ACP synthetase | 0 | Not detected | 0.053 | -0.169 | 0.275 | 0.641 |
| ENZRXN0.5 | 4-aminobutyrate aminotransferase | 0 | Not detected | -0.095 | -0.286 | 0.096 | 0.331 |
| ENZRXN0.5 | endochitinase | 0 | Not detected | 0.160 | -0.068 | 0.388 | 0.169 |
| ENZRXN0.5 | serine/threonine:Na+ symporter | 0 | Not detected | -0.002 | -0.120 | 0.116 | 0.971 |

| ENZRXN0.5 | ribonuclease | 0 | Not detected | 0.087 | -0.079 | 0.253 | 0.305 |
| --- | --- | --- | --- | --- | --- | --- | --- |
| ENZRXN0.5 | RNase | 0 | Not detected | 0.076 | -0.226 | 0.377 | 0.622 |
| ENZRXN0.5 | methylglyoxal reductase | 0 | Not detected | -0.028 | -0.148 | 0.093 | 0.653 |
| ENZRXN0.5 | methylglyoxal reductase | 0 | Not detected | -0.020 | -0.191 | 0.151 | 0.816 |
| ENZRXN0.5 | methylglyoxal reductase | 0 | Not detected | -0.009 | -0.292 | 0.273 | 0.948 |
| ENZRXN0.5 | Kdo2-lipid VIA phosphoethanolamine 7 | 0 | Not detected | 0.180 | -0.039 | 0.399 | 0.107 |
| ENZRXN0.5 | anhydro-N-acetylmuramic acid kinase | 0 | Not detected | -0.044 | -0.233 | 0.144 | 0.644 |
| ENZRXN0.5 | RNase | 0 | Not detected | 0.144 | -0.109 | 0.397 | 0.265 |
| ENZRXN0.6 | DNA polymerase | 0 | Not detected | -0.061 | -0.329 | 0.206 | 0.653 |
| ENZRXN0.6 | 3' to 5' proofreading exonuclease | 0 | Not detected | 0.008 | -0.171 | 0.186 | 0.933 |
| ENZRXN0.6 | DNA primase | 258 | Not detected | -0.340 | -0.577 | -0.104 | 0.005 |
| ENZRXN0.6 | DNA polymerase | 0 | Not detected | 0.198 | -0.029 | 0.424 | 0.088 |
| ENZRXN0.6 | DNA polymerase | 0 | Not detected | 0.198 | -0.029 | 0.424 | 0.088 |
| ENZRXN0.6 | peptidyl-prolyl cis-trans isomerase | 0 | Not detected | -0.088 | -0.283 | 0.107 | 0.378 |
| ENZRXN0.6 | cAMP phosphodiesterase | 0 | Not detected | -0.139 | -0.325 | 0.046 | 0.141 |
| ENZRXN0.6 | DNA polymerase | 0 | Not detected | -0.042 | -0.311 | 0.226 | 0.758 |
| ENZRXN0.6 | 3' to 5' proofreading exonuclease | 0 | Not detected | -0.042 | -0.311 | 0.226 | 0.758 |
| ENZRXN0.6 | 5' to 3' exonuclease | 0 | Not detected | -0.042 | -0.311 | 0.226 | 0.758 |
| ENZRXN0.6 | ADP-heptose:Kdo2-lipid IVA-heptosylt | 0 | Not detected | -0.026 | -0.223 | 0.171 | 0.794 |
| ENZRXN0.6 | ADP-heptose&mdash;LPS heptosyltran | 0 | Not detected | -0.067 | -0.272 | 0.137 | 0.520 |
| ENZRXN0.6 | isopentenyl-adenosine tRNA methylthio | 0 | Not detected | -0.174 | -0.440 | 0.093 | 0.201 |
| ENZRXN0.6 | xanthine:H+ symporter XanP | 0 | Not detected | -0.144 | -0.295 | 0.007 | 0.062 |
| ENZRXN0.6 | 1-deoxy-D-xylulose 5-phosphate:thiol s | 0 | Not detected | -0.040 | -0.165 | 0.086 | 0.534 |
| ENZRXN0.6 | octanoate-non-lipoylated apo domain lig | 0 | Not detected | -0.011 | -0.177 | 0.155 | 0.897 |
| ENZRXN0.6 | 16S rRNA m5C1407 methyltransferase | 0 | Not detected | -0.024 | -0.211 | 0.162 | 0.798 |
| ENZRXN0.6 | Î²-D-glucoside glucohydrolase | 0 | Not detected | 0.015 | -0.205 | 0.234 | 0.896 |
| ENZRXN0.6 | ADP-heptose:Kdo2-lipid A heptosyltran | 0 | Not detected | -0.026 | -0.223 | 0.171 | 0.794 |
| ENZRXN0.6 | transport of D-serine | 0 | Not detected | 0.116 | -0.121 | 0.353 | 0.339 |
| ENZRXN0.6 | methylglyoxal reductase | 0 | Not detected | -0.144 | -0.439 | 0.151 | 0.338 |
| ENZRXN0.6 | 5-methylaminomethyl-2-thiouridine-for | 0 | Not detected | 0.089 | -0.121 | 0.300 | 0.404 |
| ENZRXN0.6 | 2-isopropylmalate synthase | 0 | Not detected | -0.134 | -0.286 | 0.019 | 0.085 |
| ENZRXN0.6 | 6-phosphogluconate phosphatase | 0 | Not detected | -0.015 | -0.181 | 0.152 | 0.864 |
| ENZRXN0.6 | sugar phosphatase | 0 | Not detected | 0.132 | -0.057 | 0.321 | 0.172 |
| ENZRXN0.6 | pyridoxal phosphatase | 0 | Not detected | -0.083 | -0.258 | 0.093 | 0.355 |
| ENZRXN0.6 | phosphosugar phosphatase | 0 | Not detected | -0.083 | -0.258 | 0.093 | 0.355 |
| ENZRXN0.6 | 23S rRNA m2G1835 methyltransferase | 0 | Not detected | -0.011 | -0.200 | 0.177 | 0.906 |
| ENZRXN0.6 | 23S rRNA m2G2445 methyltransferase | 0 | Not detected | 0.100 | -0.140 | 0.340 | 0.414 |
| ENZRXN0.6 | 3 methyl-adenine DNA glycosylase | 0 | Not detected | -0.046 | -0.249 | 0.156 | 0.653 |
| ENZRXN0.6 | peptidyl-prolyl cis-trans isomerase | 0 | Not detected | 0.047 | -0.178 | 0.272 | 0.683 |
| ENZRXN0.6 | lysine 2,3-aminomutase | 0 | Not detected | -0.007 | -0.210 | 0.196 | 0.944 |
| ENZRXN0.6 | alanyl aminopeptidase | 0 | Not detected | -0.075 | -0.321 | 0.172 | 0.553 |
| ENZRXN0.6 | D-malate dehydrogenase (decarboxylati | 0 | Not detected | -0.310 | -0.554 | -0.066 | 0.013 |
| ENZRXN0.6 | poly(A) polymerase | 0 | Not detected | -0.091 | -0.299 | 0.116 | 0.388 |
| ENZRXN0.6 | 2-oxobutanoate aminotransferase | 0 | Not detected | 0.042 | -0.154 | 0.237 | 0.677 |

| ENZRXN0.6 | exodeoxyribonuclease I | 0 | Not detected | -0.089 | -0.300 | 0.122 | 0.410 |
| --- | --- | --- | --- | --- | --- | --- | --- |
| ENZRXN0.6 | transport of Î²-alanine | 0 | Not detected | 0.116 | -0.121 | 0.353 | 0.339 |
| ENZRXN0.6 | transport of L-alanine | 0 | Not detected | 0.116 | -0.121 | 0.353 | 0.339 |
| ENZRXN0.6 | transport of D-cycloserine | 0 | Not detected | 0.116 | -0.121 | 0.353 | 0.339 |
| ENZRXN0.6 | 3' &rarr; 5' exonuclease | 0 | Not detected | 0.010 | -0.160 | 0.181 | 0.905 |
| ENZRXN0.6 | Zn2+-exporting ATPase | 0 | Not detected | 0.027 | -0.211 | 0.264 | 0.825 |
| ENZRXN0.6 | S-ribosylhomocysteine lyase | 0 | Not detected | 0.039 | -0.158 | 0.237 | 0.697 |
| ENZRXN0.6 | 16S rRNA m2G966 methyltransferase | 0 | Not detected | 0.042 | -0.124 | 0.209 | 0.618 |
| ENZRXN0.6 | D-gluconate transporter | 0 | Not detected | 0.029 | -0.169 | 0.228 | 0.771 |
| ENZRXN0.6 | high-affinity gluconate transporter | 0 | Not detected | 0.221 | -0.024 | 0.466 | 0.077 |
| ENZRXN0.6 | 2-O-(6-phospho-Î±-mannosyl)-D-glycer | 0 | Not detected | -0.172 | -0.389 | 0.046 | 0.121 |
| ENZRXN0.6 | putrescine:H+ symporter | 0 | Not detected | -0.112 | -0.397 | 0.174 | 0.443 |
| ENZRXN0.6 | putrescine:L-ornithine antiporter | 0 | Not detected | -0.112 | -0.397 | 0.174 | 0.443 |
| ENZRXN0.6 | cadaverine:H+ symporter | 0 | Not detected | 0.242 | -0.016 | 0.500 | 0.066 |
| ENZRXN0.6 | serine/threonine protein kinase | 94 | Not detected | 0.223 | 0.040 | 0.406 | 0.017 |
| ENZRXN0.6 | tRNA pseudouridine32 synthase | 0 | Not detected | -0.191 | -0.374 | -0.007 | 0.042 |
| ENZRXN0.6 | transport of GlcNAc-1,6-anhMurNAc-L | 0 | Not detected | 0.138 | -0.055 | 0.332 | 0.161 |
| ENZRXN0.6 | 1,6-anhydro-N-acetylmuramoyl-L-alani | 0 | Not detected | 0.152 | -0.038 | 0.342 | 0.118 |
| ENZRXN0.6 | glcNAc-1,6-anhMurNAcaminidase | 0 | Not detected | -0.008 | -0.203 | 0.188 | 0.939 |
| ENZRXN0.6 | galactonate:H+ symporter | 0 | Not detected | 0.052 | -0.150 | 0.254 | 0.616 |
| ENZRXN0.6 | L-galactonate oxidoreductase | 0 | Not detected | 0.068 | -0.121 | 0.257 | 0.479 |
| ENZRXN0.6 | transport of L-aspartate | 0 | Not detected | -0.152 | -0.421 | 0.116 | 0.266 |
| ENZRXN0.6 | succinate:fumarate antiporter | 0 | Not detected | 0.048 | -0.165 | 0.262 | 0.656 |
| ENZRXN0.6 | 16S rRNA m7G527 methyltransferase | 0 | Not detected | -0.020 | -0.203 | 0.163 | 0.833 |
| ENZRXN0.6 | transport of L-lyxose | 0 | Not detected | -0.027 | -0.212 | 0.158 | 0.777 |
| ENZRXN0.6 | L-idarate epimerase | 0 | Not detected | -0.246 | -0.502 | 0.011 | 0.060 |
| ENZRXN0.6 | nitrate:nitrite antiporter | 0 | Not detected | 0.026 | -0.151 | 0.203 | 0.774 |
| ENZRXN0.6 | biotin transporter | 0 | Not detected | -0.058 | -0.236 | 0.121 | 0.527 |
| ENZRXN0.6 | arbutin 6-phosphate glucohydrolase | 0 | Not detected | 0.072 | -0.187 | 0.331 | 0.584 |
| ENZRXN0.6 | arbutin 6-phosphate glucohydrolase | 0 | Not detected | 0.022 | -0.093 | 0.137 | 0.704 |
| ENZRXN0.6 | arbutin-6-phosphate glucohydrolase | 0 | Not detected | 0.007 | -0.146 | 0.159 | 0.930 |
| ENZRXN0.6 | salicin 6-phosphate glucohydrolase | 0 | Not detected | 0.072 | -0.187 | 0.331 | 0.584 |
| ENZRXN0.6 | transport of N-acetyl-Î²-D-glucosamine( | 0 | Not detected | 0.138 | -0.055 | 0.332 | 0.161 |
| ENZRXN0.6 | 23S rRNA m2A2503 methyltransferase | 0 | Not detected | 0.008 | -0.264 | 0.281 | 0.952 |
| ENZRXN0.6 | transport of a tripeptide | 0 | Not detected | 0.049 | -0.127 | 0.226 | 0.582 |
| ENZRXN0.6 | protein kinase | 0 | Not detected | 0.074 | -0.131 | 0.278 | 0.480 |
| ENZRXN0.6 | L-2-hydroxyglutarate dehydrogenase | 0 | Not detected | -0.027 | -0.217 | 0.163 | 0.778 |
| ENZRXN0.6 | undecaprenyl-pyrophosphate pyrophosp | 0 | Not detected | -0.113 | -0.352 | 0.127 | 0.357 |
| ENZRXN0.6 | NAD(P)H:quinone oxidoreductase | 0 | Not detected | 0.055 | -0.139 | 0.248 | 0.580 |
| ENZRXN0.6 | anthranilate phosphoribosyl transferase | 0 | Not detected | 0.072 | -0.134 | 0.277 | 0.494 |
| ENZRXN0.6 | nigerose hydrolase | 0 | Not detected | 0.148 | -0.068 | 0.365 | 0.178 |
| ENZRXN0.6 | peptidoglycan glycosyltransferase | 0 | Not detected | 0.105 | -0.114 | 0.325 | 0.347 |
| ENZRXN0.6 | peptidoglycan glycosyltransferase | 0 | Not detected | 0.048 | -0.148 | 0.244 | 0.632 |
| ENZRXN0.6 | murein transpeptidase | 0 | Not detected | -0.126 | -0.369 | 0.117 | 0.309 |

| ENZRXN0.6 | glycerol-2-phosphatase | 0 | Not detected | 0.067 | -0.145 | 0.279 | 0.536 |
| --- | --- | --- | --- | --- | --- | --- | --- |
| ENZRXN0.6 | L-glyceraldehyde 3-phosphate reductase | 0 | Not detected | -0.028 | -0.148 | 0.093 | 0.653 |
| ENZRXN0.6 | poly-Î²-1,6-N-acetyl-D-glucosamine N-d | 0 | Not detected | 0.152 | -0.049 | 0.354 | 0.139 |
| ENZRXN0.6 | tRNAMet cytidine acetyltransferase | 0 | Not detected | 0.072 | -0.140 | 0.284 | 0.506 |
| ENZRXN0.6 | ribosomal protein L11 methyltransferase | 0 | Not detected | -0.153 | -0.368 | 0.062 | 0.163 |
| ENZRXN0.6 | glutamyl-Q tRNA(Asp) synthetase | 0 | Not detected | 0.009 | -0.167 | 0.186 | 0.916 |
| ENZRXN0.6 | 3-hydroxypropionaldehyde dehydrogena | 0 | Not detected | 0.102 | -0.085 | 0.289 | 0.285 |
| ENZRXN0.6 | lipid III flippase | 0 | Not detected | 0.010 | -0.195 | 0.215 | 0.924 |
| ENZRXN0.6 | glucose dehydrogenase | 0 | Not detected | 0.053 | -0.183 | 0.288 | 0.662 |
| ENZRXN0.6 | autoinducer-2 kinase | 0 | Not detected | 0.266 | 0.055 | 0.477 | 0.013 |
| ENZRXN0.6 | lipid II flippase | 0 | Not detected | -0.044 | -0.237 | 0.149 | 0.657 |
| ENZRXN0.6 | GTPase | 0 | Not detected | 0.108 | -0.110 | 0.326 | 0.331 |
| ENZRXN0.6 | transport of a dipeptide | 0 | Not detected | -0.075 | -0.284 | 0.133 | 0.479 |
| ENZRXN0.6 | acyl-[acp]:1-acyl-sn-glycerol-3-phospha | 0 | Not detected | -0.056 | -0.237 | 0.125 | 0.543 |
| ENZRXN0.6 | acyl-acp:sn-glycerol-3-phosphate 1-O-a | 0 | Not detected | -0.076 | -0.332 | 0.181 | 0.563 |
| ENZRXN0.6 | 23S rRNA 2'-O-ribose C2498 methyltra | 0 | Not detected | 0.040 | -0.158 | 0.239 | 0.691 |
| ENZRXN0.7 | fructoselysine transporter | 0 | Not detected | 0.060 | -0.212 | 0.332 | 0.666 |
| ENZRXN0.7 | psicoselysine transporter | 0 | Not detected | 0.060 | -0.212 | 0.332 | 0.666 |
| ENZRXN0.7 | L-ribulose-5-phosphate 4-epimerase | 0 | Not detected | 0.004 | -0.119 | 0.127 | 0.949 |
| ENZRXN0.7 | L-ribulose-5-phosphate 3-epimerase | 0 | Not detected | 0.002 | -0.240 | 0.244 | 0.989 |
| ENZRXN0.7 | transport of a dipeptide | 0 | Not detected | 0.066 | -0.132 | 0.263 | 0.514 |
| ENZRXN0.7 | non-chaperonin molecular chaperone A | 0 | Not detected | 0.052 | -0.211 | 0.316 | 0.696 |
| ENZRXN0.7 | protein methylthiotransferase | 0 | Not detected | 0.016 | -0.249 | 0.281 | 0.907 |
| ENZRXN0.7 | dipeptide transport | 0 | Not detected | -0.077 | -0.274 | 0.120 | 0.444 |
| ENZRXN0.7 | (S)-ureidoglycine aminohydrolase | 0 | Not detected | -0.050 | -0.285 | 0.185 | 0.676 |
| ENZRXN0.7 | chitobiose transport | 0 | Not detected | 0.032 | -0.158 | 0.222 | 0.741 |
| ENZRXN0.7 | transport of adenine | 0 | Not detected | -0.068 | -0.281 | 0.146 | 0.535 |
| ENZRXN0.7 | guanosine 3'-diphosphate 5'-triphosphat | 0 | Not detected | -0.201 | -0.467 | 0.066 | 0.140 |
| ENZRXN0.7 | GTPase | 0 | Not detected | 0.005 | -0.277 | 0.287 | 0.973 |
| ENZRXN0.7 | GTPase | 0 | Not detected | 0.186 | -0.038 | 0.409 | 0.103 |
| ENZRXN0.7 | carbamoyl--[HypE] ligase | 0 | Not detected | 0.002 | -0.235 | 0.239 | 0.986 |
| ENZRXN0.7 | 2-iminoacetate synthase | 0 | Not detected | -0.007 | -0.199 | 0.185 | 0.943 |
| ENZRXN0.7 | transport of malate | 0 | Not detected | -0.152 | -0.421 | 0.116 | 0.266 |
| ENZRXN0.7 | methionine-oxo-acid transaminase | 0 | Not detected | 0.049 | -0.139 | 0.236 | 0.611 |
| ENZRXN0.7 | p-aminobenzoyl glutamate:H+ symporte | 0 | Not detected | -0.122 | -0.301 | 0.057 | 0.182 |
| ENZRXN0.7 | uracil oxygenase | 0 | Not detected | -0.043 | -0.217 | 0.132 | 0.633 |
| ENZRXN0.7 | RNA helicase | 0 | Not detected | -0.025 | -0.208 | 0.157 | 0.785 |
| ENZRXN0.7 | exoribonuclease II | 0 | Not detected | -0.032 | -0.262 | 0.198 | 0.786 |
| ENZRXN0.7 | peptidoglycan DD transpeptidase | 0 | Not detected | 0.033 | -0.202 | 0.269 | 0.782 |
| ENZRXN0.7 | L-aspartate oxidase | 0 | Not detected | -0.034 | -0.338 | 0.269 | 0.824 |
| ENZRXN0.7 | exoribonuclease II | 0 | Not detected | -0.032 | -0.262 | 0.198 | 0.786 |
| ENZRXN0.7 | adenosine nucleosidase | 0 | Not detected | -0.104 | -0.287 | 0.078 | 0.263 |
| ENZRXN0.7 | xanthosine nucleosidase | 0 | Not detected | -0.104 | -0.287 | 0.078 | 0.263 |
| ENZRXN0.7 | inosine nucleosidase | 0 | Not detected | -0.104 | -0.287 | 0.078 | 0.263 |

| ENZRXN0.7 | cytidine nucleosidase | 0 | Not detected | -0.104 | -0.287 | 0.078 | 0.263 |
| --- | --- | --- | --- | --- | --- | --- | --- |
| ENZRXN0.7 | dihydroorotate dehydrogenase (MQ) | 0 | Not detected | 0.002 | -0.294 | 0.297 | 0.992 |
| ENZRXN0.7 | transport of 3-hydroxy-trans-cinnamate | 0 | Not detected | -0.086 | -0.272 | 0.099 | 0.362 |
| ENZRXN0.7 | epoxyqueuosine reductase | 0 | Not detected | -0.059 | -0.252 | 0.135 | 0.553 |
| ENZRXN0.7 | putrescine:H+ symporter PlaP | 0 | Not detected | 0.085 | -0.103 | 0.273 | 0.376 |
| ENZRXN0.7 | acyl-[acp]:1-acyl-sn-glycerol-3-phospha | 0 | Not detected | -0.056 | -0.237 | 0.125 | 0.543 |
| ENZRXN0.7 | putative inorganic phosphate export pro | 0 | Not detected | -0.128 | -0.343 | 0.088 | 0.247 |
| ENZRXN0.7 | S-methyl-L-methionine transporter | 0 | Not detected | 0.014 | -0.273 | 0.301 | 0.925 |
| ENZRXN0.7 | NADHX epimerase | 0 | Not detected | 0.083 | -0.128 | 0.293 | 0.443 |
| ENZRXN0.7 | (S)-NADHX dehydratase | 0 | Not detected | 0.083 | -0.128 | 0.293 | 0.443 |
| ENZRXN0.7 | transport of inosine | 0 | Not detected | 0.212 | -0.013 | 0.437 | 0.065 |
| ENZRXN0.7 | transport of deoxyinosine | 0 | Not detected | 0.212 | -0.013 | 0.437 | 0.065 |
| ENZRXN0.7 | 16S rRNA m2G1516 methyltransferase | 0 | Not detected | -0.094 | -0.278 | 0.090 | 0.316 |
| ENZRXN0.7 | Î±-D-ribose-1-methylphosphonate-5-trip | 0 | Not detected | 0.196 | -0.003 | 0.394 | 0.053 |
| ENZRXN0.7 | carbon-phosphorous lyase | 0 | Not detected | 0.133 | -0.096 | 0.363 | 0.255 |
| ENZRXN0.7 | peptidoglycan DD-carboxypeptidase | 0 | Not detected | -0.173 | -0.360 | 0.014 | 0.070 |
| ENZRXN0.7 | peptidoglycan DD-endopeptidase | 0 | Not detected | -0.173 | -0.360 | 0.014 | 0.070 |
| ENZRXN0.7 | ferric enterobactin esterase | 0 | Not detected | 0.012 | -0.170 | 0.194 | 0.897 |
| ENZRXN0.7 | (S)-lactate transporter | 0 | Not detected | 0.097 | -0.162 | 0.356 | 0.465 |
| ENZRXN0.7 | 23S rRNA m7G2069 methyltransferase | 0 | Not detected | 0.100 | -0.140 | 0.340 | 0.414 |
| ENZRXN0.7 | enterobactin exporter EntS | 0 | Not detected | -0.036 | -0.230 | 0.158 | 0.715 |
| ENZRXN0.7 | D-tartrate:succinate antiporter | 0 | Not detected | -0.114 | -0.333 | 0.105 | 0.307 |
| ENZRXN0.7 | GTPase | 0 | Not detected | -0.157 | -0.410 | 0.096 | 0.225 |
| ENZRXN0.7 | fructose-6-phosphate:phosphate antiport | 0 | Not detected | 0.023 | -0.184 | 0.229 | 0.829 |
| ENZRXN0.7 | mannose-6-phosphate:phosphate antipor | 0 | Not detected | 0.023 | -0.184 | 0.229 | 0.829 |
| ENZRXN0.7 | propionate transporter | 0 | Not detected | -0.024 | -0.290 | 0.242 | 0.861 |
| ENZRXN0.7 | metalloprotease | 0 | Not detected | 0.108 | -0.078 | 0.294 | 0.256 |
| ENZRXN0.7 | (R)-lactate transporter | 0 | Not detected | 0.133 | -0.093 | 0.359 | 0.249 |
| ENZRXN0.7 | glycolate transporter | 0 | Not detected | 0.133 | -0.093 | 0.359 | 0.249 |
| ENZRXN0.7 | (R)-lactate transporter | 0 | Not detected | 0.097 | -0.162 | 0.356 | 0.465 |
| ENZRXN0.7 | transport of a C4-dicarboxylate | 0 | Not detected | -0.152 | -0.421 | 0.116 | 0.266 |
| ENZRXN0.7 | methyl Î²-D-glucoside 6-phosphate gluc | 0 | Not detected | 0.022 | -0.093 | 0.137 | 0.704 |
| ENZRXN0.7 | D-galactarate transporter | 0 | Not detected | -0.007 | -0.198 | 0.184 | 0.946 |
| ENZRXN0.7 | D-glucarate transporter | 0 | Not detected | -0.007 | -0.198 | 0.184 | 0.946 |
| ENZRXN0.7 | D-glycerate transporter | 0 | Not detected | -0.007 | -0.198 | 0.184 | 0.946 |
| ENZRXN0.7 | propionyl-CoA synthetase | 0 | Not detected | -0.016 | -0.224 | 0.192 | 0.881 |
| ENZRXN0.7 | 23S rRNA m6A2030 methyltransferase | 0 | Not detected | -0.035 | -0.222 | 0.152 | 0.711 |
| ENZRXN0.7 | tRNA m2A37 methyltransferase | 0 | Not detected | 0.008 | -0.264 | 0.281 | 0.952 |
| ENZRXN0.7 | inorganic triphosphatase | 0 | Not detected | 0.113 | -0.081 | 0.307 | 0.255 |
| ENZRXN0.7 | L-glyceraldehyde-3-phosphate:phosphat | 0 | Not detected | 0.023 | -0.184 | 0.229 | 0.829 |
| ENZRXN0.7 | 6-phospho-Î²-D-glucosyl-(1,4)-D-glucos | 0 | Not detected | 0.007 | -0.146 | 0.159 | 0.930 |
| ENZRXN0.7 | aldehyde reductase | 0 | Not detected | -0.023 | -0.192 | 0.146 | 0.791 |
| ENZRXN0.7 | aldehyde reductase | 0 | Not detected | -0.054 | -0.241 | 0.133 | 0.571 |
| ENZRXN0.7 | 2,5-diketo-D-gluconate reductase | 0 | Not detected | -0.020 | -0.191 | 0.151 | 0.816 |

| ENZRXN0.7 | cysteinylglycine dipeptidase | 0 | Not detected | -0.075 | -0.321 | 0.172 | 0.553 |
| --- | --- | --- | --- | --- | --- | --- | --- |
| ENZRXN0.7 | UDP-N-acetylmuramate:L-alanyl-&gam | 0 | Not detected | -0.195 | -0.464 | 0.074 | 0.155 |
| ENZRXN0.7 | transport of nitrite | 0 | Not detected | 0.026 | -0.151 | 0.203 | 0.774 |
| ENZRXN0.7 | 6-phospho-Î²-D-glucosyl-(1-4)-D-glucos | 0 | Not detected | 0.072 | -0.187 | 0.331 | 0.584 |
| ENZRXN0.7 | guanosine 2'3'-cyclic phosphodiesterase | 0 | Not detected | 0.031 | -0.213 | 0.275 | 0.804 |
| ENZRXN0.7 | uridine 2'3'-cyclic phosphodiesterase | 0 | Not detected | 0.031 | -0.213 | 0.275 | 0.804 |
| ENZRXN0.7 | cytidine 2'3'-cyclic phosphodiesterase | 0 | Not detected | 0.031 | -0.213 | 0.275 | 0.804 |
| ENZRXN0.7 | adenosine 2'3'-cyclic phosphodiesterase | 0 | Not detected | 0.031 | -0.213 | 0.275 | 0.804 |
| ENZRXN0.7 | cytidine 3'-phosphate phosphohydrolase | 0 | Not detected | 0.098 | -0.086 | 0.283 | 0.296 |
| ENZRXN0.7 | adenosine 3'-phosphate phosphohydrola | 0 | Not detected | 0.098 | -0.086 | 0.283 | 0.296 |
| ENZRXN0.7 | L-1-glycerophophorylethanolamine pho | 0 | Not detected | 0.002 | -0.165 | 0.170 | 0.977 |
| ENZRXN0.7 | dGMP 5'-phosphate phosphohydrolase | 0 | Not detected | 0.098 | -0.086 | 0.283 | 0.296 |
| ENZRXN0.7 | arsenate:H+ symporter | 0 | Not detected | -0.023 | -0.238 | 0.192 | 0.836 |
| ENZRXN0.7 | glycerophosphoserine phosphodiesteras | 0 | Not detected | 0.002 | -0.165 | 0.170 | 0.977 |
| ENZRXN0.7 | glycerophosphoglycerol phosphodiester | 0 | Not detected | 0.002 | -0.165 | 0.170 | 0.977 |
| ENZRXN0.7 | succinate transporter | 0 | Not detected | 0.086 | -0.133 | 0.304 | 0.442 |
| ENZRXN0.7 | fumarate transporter | 0 | Not detected | 0.086 | -0.133 | 0.304 | 0.442 |
| ENZRXN0.7 | aspartate transport | 0 | Not detected | 0.086 | -0.133 | 0.304 | 0.442 |
| ENZRXN0.7 | amylomaltase | 0 | Not detected | 0.071 | -0.165 | 0.307 | 0.556 |
| ENZRXN0.7 | amylomaltase | 0 | Not detected | 0.071 | -0.165 | 0.307 | 0.556 |
| ENZRXN0.7 | maltopentaose glucosidase | 0 | Not detected | 0.032 | -0.176 | 0.240 | 0.764 |
| ENZRXN0.7 | maltohexaose glucosidase | 0 | Not detected | 0.032 | -0.176 | 0.240 | 0.764 |
| ENZRXN0.7 | maltoheptaose glucosidase | 0 | Not detected | 0.032 | -0.176 | 0.240 | 0.764 |
| ENZRXN0.7 | GTPase | 0 | Not detected | 0.075 | -0.107 | 0.257 | 0.421 |
| ENZRXN0.7 | Na+:H+ antiporter | 0 | Not detected | -0.039 | -0.216 | 0.137 | 0.661 |
| ENZRXN0.7 | K+:H+ antiporter | 0 | Not detected | -0.039 | -0.216 | 0.137 | 0.661 |
| ENZRXN0.7 | phosphoethanolamine transferase | 0 | Not detected | 0.137 | -0.070 | 0.344 | 0.194 |
| ENZRXN0.7 | phosphoethanolamine transferase | 0 | Not detected | -0.056 | -0.264 | 0.151 | 0.594 |
| ENZRXN0.7 | cytidine 3' nucleotidase | 0 | Not detected | 0.031 | -0.213 | 0.275 | 0.804 |
| ENZRXN0.7 | guanosine 3' nucleotidase | 0 | Not detected | 0.031 | -0.213 | 0.275 | 0.804 |
| ENZRXN0.7 | adenosine 3' nucleotidase | 0 | Not detected | 0.031 | -0.213 | 0.275 | 0.804 |
| ENZRXN0.7 | uridine 3' nucleotidase | 0 | Not detected | 0.031 | -0.213 | 0.275 | 0.804 |
| ENZRXN0.7 | 5'-deoxyribonucleotidase | 0 | Not detected | -0.135 | -0.366 | 0.096 | 0.251 |
| ENZRXN0.7 | 2-octaprenylphenol hydroxylase | 0 | Not detected | 0.101 | -0.087 | 0.288 | 0.294 |
| ENZRXN0.7 | DNA-AP site lyase | 0 | Not detected | -0.026 | -0.303 | 0.252 | 0.855 |
| ENZRXN0.7 | transport of D-glucose | 0 | Not detected | -0.041 | -0.235 | 0.154 | 0.684 |
| ENZRXN0.7 | AMP kinase (GTP) | 0 | Not detected | -0.051 | -0.177 | 0.075 | 0.430 |
| ENZRXN0.7 | protein-lysine deacetylase | 0 | Not detected | 0.202 | -0.009 | 0.413 | 0.061 |
| ENZRXN0.7 | transport of a dipeptide | 0 | Not detected | 0.049 | -0.127 | 0.226 | 0.582 |
| ENZRXN0.7 | transport of glucose | 0 | Not detected | 0.019 | -0.184 | 0.223 | 0.851 |
| ENZRXN0.7 | transport of maltose | 0 | Not detected | 0.019 | -0.184 | 0.223 | 0.851 |
| ENZRXN0.7 | guanine transport | 0 | Not detected | -0.104 | -0.323 | 0.115 | 0.352 |
| ENZRXN0.7 | hypoxanthine transport | 0 | Not detected | -0.104 | -0.323 | 0.115 | 0.352 |
| ENZRXN0.7 | 5-aminomethyl-2-thiouridine synthase | 0 | Not detected | 0.089 | -0.121 | 0.300 | 0.404 |

| ENZRXN0.7 | phosphoethanolamine transferase | 0 | Not detected | -0.016 | -0.212 | 0.180 | 0.874 |
| --- | --- | --- | --- | --- | --- | --- | --- |
| ENZRXN0.7 | 6-deoxy-6-sulfofructose kinase | 0 | Not detected | 0.138 | -0.046 | 0.322 | 0.142 |
| ENZRXN0.7 | 6-deoxy-6-sulfofructose-1-phosphate ald | 0 | Not detected | 0.206 | 0.021 | 0.391 | 0.029 |
| ENZRXN0.7 | sulfoquinovose transporter | 0 | Not detected | 0.014 | -0.198 | 0.227 | 0.896 |
| ENZRXN0.7 | uracil:H+ symporter | 0 | Not detected | -0.002 | -0.271 | 0.267 | 0.988 |
| ENZRXN0.7 | transport of a tripeptide | 0 | Not detected | -0.075 | -0.284 | 0.133 | 0.479 |
| ENZRXN0.7 | transport of a tripeptide | 0 | Not detected | 0.066 | -0.132 | 0.263 | 0.514 |
| ENZRXN0.7 | L-threonine dehydrogenase | 0 | Not detected | 0.032 | -0.241 | 0.304 | 0.821 |
| ENZRXN0.7 | 11-deoxycorticosterone reductase | 0 | Not detected | 0.016 | -0.175 | 0.208 | 0.868 |
| ENZRXN0.7 | D-gluconate 5-dehydrogenase | 0 | Not detected | 0.016 | -0.175 | 0.208 | 0.868 |
| ENZRXN0.7 | 2-keto-3-deoxy-D-gluconate dehydroge | 0 | Not detected | 0.016 | -0.175 | 0.208 | 0.868 |
| ENZRXN0.7 | sodium dodecyl sulfatase | 0 | Not detected | 0.160 | -0.058 | 0.378 | 0.150 |
| ENZRXN0.7 | isobutyraldehyde reductase | 0 | Not detected | -0.023 | -0.192 | 0.146 | 0.791 |
| ENZRXN0.7 | isobutyraldehyde reductase | 0 | Not detected | -0.020 | -0.191 | 0.151 | 0.816 |
| ENZRXN0.7 | isobutyraldehyde reductase | 0 | Not detected | -0.176 | -0.439 | 0.086 | 0.188 |
| ENZRXN0.7 | 1-hydroxy-2-methyl-2-(E)-butenyl 4-dip | 0 | Not detected | 0.019 | -0.102 | 0.140 | 0.756 |
| ENZRXN0.7 | 3' &rarr; 5' ssDNA exonuclease | 0 | Not detected | -0.005 | -0.177 | 0.166 | 0.951 |
| ENZRXN0.7 | 3' &rarr; 5' RNA exonuclease | 0 | Not detected | -0.005 | -0.177 | 0.166 | 0.951 |
| ENZRXN0.7 | ATP pyrophosphatase | 0 | Not detected | -0.090 | -0.317 | 0.138 | 0.440 |
| ENZRXN0.7 | 3-isopropylmalate dehydrogenase (decar | 0 | Not detected | -0.310 | -0.554 | -0.066 | 0.013 |
| ENZRXN0.7 | L-tartrate dehydrogenase | 0 | Not detected | -0.310 | -0.554 | -0.066 | 0.013 |
| ENZRXN0.7 | glycerophosphoglycerol acyltransferase | 0 | Not detected | 0.028 | -0.159 | 0.214 | 0.772 |
| ENZRXN0.7 | NADH:menaquinone oxidoreductase | 0 | Not detected | 0.041 | -0.161 | 0.242 | 0.693 |
| ENZRXN0.7 | polyphenol oxidase | 0 | Not detected | -0.039 | -0.219 | 0.141 | 0.670 |
| ENZRXN0.7 | PII-UMP deuridylylation | 0 | Not detected | 0.115 | -0.117 | 0.348 | 0.332 |
| ENZRXN0.7 | D-ribulose 5-phosphate 1-dehydrogenas | 11 | Not detected | 0.264 | 0.039 | 0.489 | 0.021 |
| ENZRXN0.7 | flavin mononucleotide (FMN) export | 0 | Not detected | 0.002 | -0.181 | 0.185 | 0.983 |
| ENZRXN0.7 | flavin adenine dinucleotide (FAD) expo | 0 | Not detected | 0.002 | -0.181 | 0.185 | 0.983 |
| ENZRXN0.7 | L,D-carboxypeptidase | 0 | Not detected | 0.063 | -0.140 | 0.266 | 0.544 |
| ENZRXN0.7 | L,D-transpeptidase | 0 | Not detected | 0.063 | -0.140 | 0.266 | 0.544 |
| ENZRXN0.7 | L,D transpeptidase | 0 | Not detected | -0.014 | -0.204 | 0.176 | 0.888 |
| ENZRXN0.7 | L,D-transpeptidase | 0 | Not detected | 0.063 | -0.140 | 0.266 | 0.544 |
| ENZRXN0.7 | 5' &rarr; 3' exonuclease | 0 | Not detected | 0.053 | -0.120 | 0.226 | 0.547 |
| ENZRXN0.7 | 3',5'-nucleoside bisphosphate phosphata | 0 | Not detected | 0.053 | -0.120 | 0.226 | 0.547 |
| ENZRXN0.7 | ATPase | 0 | Not detected | 0.006 | -0.115 | 0.127 | 0.922 |
| ENZRXN0.7 | acyl-acp:sn-glycerol-3-phosphate 1-O-a | 0 | Not detected | -0.076 | -0.332 | 0.181 | 0.563 |
| ENZRXN0.7 | acyl-acp:sn-glycerol-3-phosphate 1-O-a | 0 | Not detected | -0.076 | -0.332 | 0.181 | 0.563 |
| ENZRXN0.7 | acyl-acp:sn-glycerol-3-phosphate 1-O-a | 0 | Not detected | -0.076 | -0.332 | 0.181 | 0.563 |
| ENZRXN0.7 | acyl-[acp]:1-acyl-sn-glycerol-3-phospha | 0 | Not detected | -0.056 | -0.237 | 0.125 | 0.543 |
| ENZRXN0.7 | acyl-[acp]:1-acyl-sn-glycerol-3-phospha | 0 | Not detected | -0.056 | -0.237 | 0.125 | 0.543 |
| ENZRXN0.7 | acyl-[acp]:1-acyl-sn-glycerol-3-phospha | 0 | Not detected | -0.056 | -0.237 | 0.125 | 0.543 |
| ENZRXN0.7 | acyl-[acp]:1-acyl-sn-glycerol-3-phospha | 0 | Not detected | -0.056 | -0.237 | 0.125 | 0.543 |
| ENZRXN0.7 | acyl-[acp]:1-acyl-sn-glycerol-3-phospha | 0 | Not detected | -0.056 | -0.237 | 0.125 | 0.543 |
| ENZRXN0.8 | magnesium hydrogenphosphate:H+ sym | 0 | Not detected | -0.023 | -0.238 | 0.192 | 0.836 |

| ENZRXN0.8 | calcium hydrogenphosphate:H+ sympor | 0 | Not detected | -0.023 | -0.238 | 0.192 | 0.836 |
| --- | --- | --- | --- | --- | --- | --- | --- |
| ENZRXN0.8 | zinc hydrogenphosphate:H+ symporter | 0 | Not detected | -0.023 | -0.238 | 0.192 | 0.836 |
| ENZRXN0.8 | tellurite:H+ symporter | 0 | Not detected | -0.023 | -0.238 | 0.192 | 0.836 |
| ENZRXN0.8 | L-methionine:H+ antiport | 0 | Not detected | 0.095 | -0.098 | 0.288 | 0.335 |
| ENZRXN0.8 | L-leucine:H+ antiport | 0 | Not detected | 0.095 | -0.098 | 0.288 | 0.335 |
| ENZRXN0.8 | L-isoleucine:H+ antiport | 0 | Not detected | 0.095 | -0.098 | 0.288 | 0.335 |
| ENZRXN0.8 | L-valine:H+ antiport | 0 | Not detected | 0.095 | -0.098 | 0.288 | 0.335 |
| ENZRXN0.8 | farnesyl diphosphate phosphatase | 0 | Not detected | 0.026 | -0.191 | 0.243 | 0.814 |
| ENZRXN0.8 | L-cystine:H+ symporter | 0 | Not detected | 0.160 | -0.040 | 0.360 | 0.118 |
| ENZRXN0.8 | D-cystine:H+ symporter | 0 | Not detected | 0.160 | -0.040 | 0.360 | 0.118 |
| ENZRXN0.8 | homocystine:H+ symporter | 0 | Not detected | 0.160 | -0.040 | 0.360 | 0.118 |
| ENZRXN0.8 | apolipoprotein N-acyltransferase | 0 | Not detected | 0.116 | -0.081 | 0.312 | 0.249 |
| ENZRXN0.8 | peptidoglycan lytic exotransglycosylase | 0 | Not detected | 0.038 | -0.145 | 0.221 | 0.683 |
| ENZRXN0.8 | peptidoglycan lytic exotransglycosylase | 0 | Not detected | -0.064 | -0.242 | 0.114 | 0.483 |
| ENZRXN0.8 | peptidoglycan lytic exotransglycosylase | 0 | Not detected | 0.054 | -0.158 | 0.266 | 0.618 |
| ENZRXN0.8 | peptidoglycan lytic endotransglycosylas | 0 | Not detected | 0.054 | -0.158 | 0.266 | 0.618 |
| ENZRXN0.8 | peptidoglycan lytic endotransglycosylas | 0 | Not detected | -0.064 | -0.242 | 0.114 | 0.483 |
| ENZRXN0.8 | peptidoglycan lytic exotransglycosylase | 0 | Not detected | 0.012 | -0.177 | 0.202 | 0.898 |
| ENZRXN0.8 | peptidoglycan lytic endotransglycosylas | 0 | Not detected | 0.012 | -0.177 | 0.202 | 0.898 |
| ENZRXN0.8 | peptidoglycan lytic endotransglycosylas | 0 | Not detected | 0.092 | -0.127 | 0.311 | 0.411 |
| ENZRXN0.8 | peptidoglycan lytic exotransglycosylase | 0 | Not detected | 0.092 | -0.127 | 0.311 | 0.411 |
| ENZRXN0.8 | peptidoglycan lytic exotransglycosylase | 0 | Not detected | -0.144 | -0.347 | 0.059 | 0.166 |
| ENZRXN0.8 | GTPase, ribosome-stimulated | 0 | Not detected | -0.005 | -0.124 | 0.115 | 0.937 |
| ENZRXN0.8 | transport of 2-acyl-1-lysophosphatidylet | 0 | Not detected | 0.082 | -0.107 | 0.271 | 0.396 |
| ENZRXN0.8 | transport of lyso-phosphatidylglycerol | 0 | Not detected | 0.082 | -0.107 | 0.271 | 0.396 |
| ENZRXN0.8 | poly-3-hydroxybutyrate synthase | 0 | Not detected | 0.082 | -0.091 | 0.256 | 0.351 |
| ENZRXN0.8 | biotin-ATP ligase | 0 | Not detected | 0.103 | -0.081 | 0.288 | 0.271 |
| ENZRXN0.8 | sulfoquinovosidase | 0 | Not detected | -0.059 | -0.367 | 0.249 | 0.707 |
| ENZRXN0.8 | sulfoquinovosidase | 0 | Not detected | -0.059 | -0.367 | 0.249 | 0.707 |
| ENZRXN0.8 | (S)-2-hydroxybutanoate dehydrogenase | 0 | Not detected | 0.074 | -0.144 | 0.293 | 0.505 |
| ENZRXN0.8 | (S)-2-hydroxybutanoate transporter | 0 | Not detected | 0.133 | -0.093 | 0.359 | 0.249 |
| ENZRXN0.8 | (S)-2-hydroxybutanoate transporter | 0 | Not detected | 0.097 | -0.162 | 0.356 | 0.465 |
| ENZRXN0.8 | choline dehydrogenase | 0 | Not detected | 0.131 | -0.087 | 0.349 | 0.240 |
| ENZRXN0.8 | betaine aldehyde dehydrogenase | 0 | Not detected | 0.131 | -0.087 | 0.349 | 0.240 |
| ENZRXN0.8 | short chain acyl-CoA synthetase (transp | 0 | Not detected | 0.136 | -0.104 | 0.375 | 0.267 |
| ENZRXN0.8 | cis-vaccenoyl-CoA synthetase (transport | 0 | Not detected | 0.136 | -0.104 | 0.375 | 0.267 |
| ENZRXN0.8 | oleoyl-CoA synthetase (transporting) | 0 | Not detected | 0.136 | -0.104 | 0.375 | 0.267 |
| ENZRXN0.8 | inositol monophosphatase | 0 | Not detected | 0.067 | -0.145 | 0.279 | 0.536 |
| ENZRXN0.8 | fumarate:H+ symporter | 0 | Not detected | -0.114 | -0.333 | 0.105 | 0.307 |
| ENZRXN0.8 | fumarate:H+ symporter | 0 | Not detected | 0.048 | -0.165 | 0.262 | 0.656 |
| ENZRXN0.8 | acyl-CoA dehydrogenase | 0 | Not detected | 0.059 | -0.170 | 0.288 | 0.611 |
| ENZRXN0.8 | acyl-CoA dehydrogenase | 0 | Not detected | 0.059 | -0.170 | 0.288 | 0.611 |
| ENZRXN0.8 | acyl-CoA dehydrogenase | 0 | Not detected | 0.059 | -0.170 | 0.288 | 0.611 |
| ENZRXN0.8 | acyl-CoA dehydrogenase | 0 | Not detected | 0.059 | -0.170 | 0.288 | 0.611 |

| ENZRXN0.8 | acyl-CoA dehydrogenase | 0 | Not detected | 0.059 | -0.170 | 0.288 | 0.611 |
| --- | --- | --- | --- | --- | --- | --- | --- |
| ENZRXN0.8 | acyl-CoA dehydrogenase | 0 | Not detected | 0.059 | -0.170 | 0.288 | 0.611 |
| ENZRXN0.8 | acyl-CoA dehydrogenase | 0 | Not detected | 0.059 | -0.170 | 0.288 | 0.611 |
| ENZRXN0.8 | enoyl-CoA hydratase | 0 | Not detected | 0.042 | -0.172 | 0.256 | 0.702 |
| ENZRXN0.8 | enoyl-CoA hydratase | 0 | Not detected | 0.042 | -0.172 | 0.256 | 0.702 |
| ENZRXN0.8 | enoyl-CoA hydratase | 0 | Not detected | 0.042 | -0.172 | 0.256 | 0.702 |
| ENZRXN0.8 | enoyl-CoA hydratase | 0 | Not detected | 0.042 | -0.172 | 0.256 | 0.702 |
| ENZRXN0.8 | enoyl-CoA hydratase | 0 | Not detected | 0.042 | -0.172 | 0.256 | 0.702 |
| ENZRXN0.8 | enoyl-CoA hydratase | 0 | Not detected | 0.042 | -0.172 | 0.256 | 0.702 |
| ENZRXN0.8 | enoyl-CoA hydratase | 0 | Not detected | 0.042 | -0.172 | 0.256 | 0.702 |
| ENZRXN0.8 | 3-hydroxyacyl-CoA dehydrogenase | 0 | Not detected | 0.042 | -0.172 | 0.256 | 0.702 |
| ENZRXN0.8 | 3-hydroxyacyl-CoA dehydrogenase | 0 | Not detected | 0.042 | -0.172 | 0.256 | 0.702 |
| ENZRXN0.8 | 3-hydroxyacyl-CoA dehydrogenase | 0 | Not detected | 0.042 | -0.172 | 0.256 | 0.702 |
| ENZRXN0.8 | 3-hydroxyacyl-CoA dehydrogenase | 0 | Not detected | 0.042 | -0.172 | 0.256 | 0.702 |
| ENZRXN0.8 | 3-hydroxyacyl-CoA dehydrogenase | 0 | Not detected | 0.042 | -0.172 | 0.256 | 0.702 |
| ENZRXN0.8 | 3-hydroxyacyl-CoA dehydrogenase | 0 | Not detected | 0.042 | -0.172 | 0.256 | 0.702 |
| ENZRXN0.8 | 3-hydroxyacyl-CoA dehydrogenase | 0 | Not detected | 0.042 | -0.172 | 0.256 | 0.702 |
| ENZRXN0.8 | acetyl-CoA C-acyltransferase | 0 | Not detected | -0.043 | -0.234 | 0.148 | 0.659 |
| ENZRXN0.8 | acetyl-CoA C-acyltransferase | 0 | Not detected | -0.043 | -0.234 | 0.148 | 0.659 |
| ENZRXN0.8 | acetyl-CoA C-acyltransferase | 0 | Not detected | -0.043 | -0.234 | 0.148 | 0.659 |
| ENZRXN0.8 | acetyl-CoA C-acyltransferase | 0 | Not detected | -0.043 | -0.234 | 0.148 | 0.659 |
| ENZRXN0.8 | acetyl-CoA C-acyltransferase | 0 | Not detected | -0.043 | -0.234 | 0.148 | 0.659 |
| ENZRXN0.8 | acetyl-CoA C-acyltransferase | 0 | Not detected | -0.043 | -0.234 | 0.148 | 0.659 |
| ENZRXN0.8 | acetyl-CoA C-acyltransferase | 0 | Not detected | -0.043 | -0.234 | 0.148 | 0.659 |
| ENZRXN0.8 | palmitoleoyl-CoA synthetase (transporti | 0 | Not detected | 0.136 | -0.104 | 0.375 | 0.267 |
| ENZRXN0.8 | fumarate:H+ symport | 0 | Not detected | -0.048 | -0.303 | 0.207 | 0.711 |
| ENZRXN0.8 | succinate:H+ symporter | 0 | Not detected | 0.048 | -0.165 | 0.262 | 0.656 |
| ENZRXN0.8 | methylphosphotriester-DNA-protein-cys | 0 | Not detected | 0.100 | -0.079 | 0.280 | 0.275 |
| ENZRXN0.8 | O-4-methylthymine-DNA methyltransfe | 0 | Not detected | 0.100 | -0.079 | 0.280 | 0.275 |
| ENZRXN0.8 | ferredoxin-NADP+ reductase | 0 | Not detected | 0.102 | -0.066 | 0.269 | 0.234 |
| ENZRXN0.8 | 2,6-dimethoxyphenol:oxygen oxidoredu | 0 | Not detected | 0.155 | -0.047 | 0.358 | 0.133 |
| ENZRXN0.8 | 4,5-DOPA dioxygenase extradiol | 0 | Not detected | -0.083 | -0.250 | 0.085 | 0.333 |
| ENZRXN0.8 | ssDNA-specific exonuclease | 0 | Not detected | -0.154 | -0.389 | 0.081 | 0.200 |
| ENZRXN0.8 | nitrophenyl phosphatase | 0 | Not detected | -0.135 | -0.366 | 0.096 | 0.251 |
| ENZRXN0.8 | diguanylate cyclase | 0 | Not detected | 0.179 | 0.000 | 0.359 | 0.050 |
| ENZRXN0.8 | P1,P3-bis(5'-adenosyl) triphosphate hyd | 0 | Not detected | -0.135 | -0.366 | 0.096 | 0.251 |
| ENZRXN0.8 | CDP-glycerol hydrolase | 0 | Not detected | -0.135 | -0.366 | 0.096 | 0.251 |
| ENZRXN0.8 | tRNATyr pseudouridine35 synthase | 0 | Not detected | -0.014 | -0.179 | 0.150 | 0.864 |
| ENZRXN0.8 | GTPase | 0 | Not detected | 0.033 | -0.189 | 0.254 | 0.772 |
| ENZRXN0.8 | hydroxyphenyllactate dehydrogenase | 0 | Not detected | -0.053 | -0.228 | 0.121 | 0.548 |
| ENZRXN0.8 | recombination-promoting nuclease | 0 | Not detected | 0.191 | -0.039 | 0.421 | 0.103 |
| ENZRXN0.8 | S-sulfocysteine:H+ symporter | 0 | Not detected | 0.160 | -0.040 | 0.360 | 0.118 |
| ENZRXN0.8 | periplasmic nitrite reductase | 0 | Not detected | -0.005 | -0.229 | 0.219 | 0.964 |
| ENZRXN0.8 | phosphatidylglycerol synthase | 0 | Not detected | 0.115 | -0.060 | 0.291 | 0.198 |

| ENZRXN0.8 | signal peptide peptidase | 0 | Not detected | 0.024 | -0.197 | 0.245 | 0.831 |
| --- | --- | --- | --- | --- | --- | --- | --- |
| ENZRXN0.8 | lipoprotein signal peptide peptidase | 0 | Not detected | 0.024 | -0.197 | 0.245 | 0.831 |
| ENZRXN0.8 | L-cysteine desulfidase | 0 | Not detected | 0.029 | -0.186 | 0.243 | 0.794 |
| ENZRXN0.8 | rhomboid protease | 0 | Not detected | 0.082 | -0.091 | 0.255 | 0.350 |
| ENZRXN0.8 | 3' to 5' ATP-dependent DNA helicase | 0 | Not detected | 0.129 | -0.126 | 0.384 | 0.321 |
| ENZRXN0.8 | 5' to 3' ATP-dependent DNA helicase | 0 | Not detected | 0.048 | -0.170 | 0.265 | 0.669 |
| ENZRXN0.8 | cytochrome c peroxidase | 0 | Not detected | -0.145 | -0.376 | 0.086 | 0.219 |
| ENZRXN0.8 | Î²-barrel assembly-enhancing protease | 0 | Not detected | -0.022 | -0.224 | 0.179 | 0.829 |
| ENZRXN0.8 | D-alanyl-D-alanine carboxypeptidase | 0 | Not detected | 0.090 | -0.086 | 0.266 | 0.318 |
| ENZRXN0.8 | D-alanyl-D-alanine carboxypeptidase | 0 | Not detected | 0.090 | -0.086 | 0.266 | 0.318 |
| ENZRXN0.8 | Î²-lactamase | 0 | Not detected | 0.090 | -0.086 | 0.266 | 0.318 |
| ENZRXN0.8 | 2-acyl lysophospholipase | 0 | Not detected | 0.028 | -0.159 | 0.214 | 0.772 |
| ENZRXN0.8 | 1-acyl lysophospholipase | 0 | Not detected | 0.028 | -0.159 | 0.214 | 0.772 |
| ENZRXN0.8 | pyruvate:H+ symporter | 0 | Not detected | 0.039 | -0.222 | 0.300 | 0.770 |
| ENZRXN0.8 | Na+ : H+ antiporter | 0 | Not detected | -0.092 | -0.289 | 0.105 | 0.361 |
| ENZRXN0.8 | K+ : H+ antiporter | 0 | Not detected | -0.092 | -0.289 | 0.105 | 0.361 |
| ENZRXN0.8 | dequalinium:H+ antiporter | 0 | Not detected | -0.039 | -0.216 | 0.137 | 0.661 |
| ENZRXN0.8 | ethidium:H+ antiporter | 0 | Not detected | -0.039 | -0.216 | 0.137 | 0.661 |
| ENZRXN0.8 | glucosylglycerate phosphorylase | 0 | Not detected | 0.042 | -0.148 | 0.232 | 0.667 |
| ENZRXN0.8 | carbonylcyanide m-chlorophenylhydraz | 0 | Not detected | 0.070 | -0.123 | 0.263 | 0.480 |
| ENZRXN0.8 | bicozamycin:H+ antiporter | 0 | Not detected | -0.180 | -0.360 | 0.000 | 0.050 |
| ENZRXN0.8 | chloramphenicol:H+ antiporter | 0 | Not detected | 0.021 | -0.180 | 0.222 | 0.839 |
| ENZRXN0.8 | fosfomycin:H+ antiporter | 0 | Not detected | 0.015 | -0.176 | 0.206 | 0.878 |
| ENZRXN0.8 | Fe2+ transporter | 0 | Not detected | 0.125 | -0.114 | 0.364 | 0.306 |
| ENZRXN0.8 | trimethylamine N-oxide reductase | 0 | Not detected | -0.036 | -0.249 | 0.176 | 0.738 |
| ENZRXN0.8 | trimethylamine N-oxide reductase | 0 | Not detected | 0.057 | -0.150 | 0.264 | 0.587 |
| ENZRXN0.8 | UDP kinase | 0 | Not detected | -0.051 | -0.177 | 0.075 | 0.430 |
| ENZRXN0.8 | dTDP kinase | 0 | Not detected | -0.051 | -0.177 | 0.075 | 0.430 |
| ENZRXN0.8 | CDP kinase | 0 | Not detected | -0.051 | -0.177 | 0.075 | 0.430 |
| ENZRXN0.8 | dCDP kinase | 0 | Not detected | -0.051 | -0.177 | 0.075 | 0.430 |
| ENZRXN0.8 | dGDP kinase | 0 | Not detected | -0.051 | -0.177 | 0.075 | 0.430 |
| ENZRXN0.8 | GDP kinase | 0 | Not detected | -0.051 | -0.177 | 0.075 | 0.430 |
| ENZRXN0.8 | celulose phosphoethanolamine transfera | 0 | Not detected | 0.207 | -0.006 | 0.421 | 0.057 |
| ENZRXN0.8 | thymine:H+ symporter | 0 | Not detected | -0.002 | -0.271 | 0.267 | 0.988 |
| ENZRXN0.8 | xanthine:H+ symporter | 0 | Not detected | -0.002 | -0.271 | 0.267 | 0.988 |
| ENZRXN0.8 | c-di-GMP phosphodiesterase | 0 | Not detected | 0.094 | -0.110 | 0.298 | 0.367 |
| ENZRXN0.8 | apolipoprotein N-acyltransferase | 0 | Not detected | 0.116 | -0.081 | 0.312 | 0.249 |
| ENZRXN0.8 | hydroquinone:hydrogen peroxide oxidor | 0 | Not detected | -0.145 | -0.376 | 0.086 | 0.219 |
| ENZRXN0.8 | menadiol:hydrogen peroxide oxidoreduc | 0 | Not detected | -0.145 | -0.376 | 0.086 | 0.219 |
| ENZRXN0.8 | kojibiose phosphorylase | 0 | Not detected | 0.200 | -0.006 | 0.405 | 0.057 |
| ENZRXN0.8 | endo-poly-Î²-1,6-D-glucosamine hydrola | 0 | Not detected | 0.152 | -0.049 | 0.354 | 0.139 |
| ENZRXN0.8 | L-aspartate:fumarate antiport | 0 | Not detected | -0.048 | -0.303 | 0.207 | 0.711 |
| ENZRXN0.8 | thiol-disulfide exchange | 0 | Not detected | 0.129 | -0.091 | 0.350 | 0.249 |
| ENZRXN0.8 | ferric enterobactin esterase | 0 | Not detected | 0.012 | -0.170 | 0.194 | 0.897 |

| ENZRXN0.8 | thiol-disulfide exchange | 0 | Not detected | 0.129 | -0.091 | 0.350 | 0.249 |
| --- | --- | --- | --- | --- | --- | --- | --- |
| ENZRXN0.8 | 5-aminovalerate aminotransferase | 0 | Not detected | -0.095 | -0.286 | 0.096 | 0.331 |
| ENZRXN0.8 | protein-histidine auto-kinase | 0 | Not detected | 0.053 | -0.139 | 0.245 | 0.589 |
| ENZRXN0.8 | ATP-dependent 3' &rarr; 5" helicase | 0 | Not detected | -0.053 | -0.300 | 0.194 | 0.673 |
| ENZRXN0.8 | 5-aminopentanoate:H+ symporter | 0 | Not detected | -0.055 | -0.244 | 0.134 | 0.571 |
| ENZRXN0.8 | 5' &rarr; 3' helicase | 0 | Not detected | 0.078 | -0.140 | 0.297 | 0.481 |
| ENZRXN0.8 | apurinic/apyrimidinic endonuclease | 0 | Not detected | 0.010 | -0.160 | 0.181 | 0.905 |
| ENZRXN0.8 | 3'-phosphodiesterase | 0 | Not detected | 0.010 | -0.160 | 0.181 | 0.905 |
| ENZRXN0.8 | 3'-phosphomonoesterase | 0 | Not detected | 0.010 | -0.160 | 0.181 | 0.905 |
| ENZRXN0.8 | uracil DNA glycosylase | 0 | Not detected | -0.026 | -0.303 | 0.252 | 0.855 |
| ENZRXN0.8 | thymine glycol DNA glycosylase | 0 | Not detected | -0.026 | -0.303 | 0.252 | 0.855 |
| ENZRXN0.8 | protein-histidine autokinase | 0 | Not detected | -0.004 | -0.203 | 0.195 | 0.971 |
| ENZRXN185 | 1,4-alpha-glucan branching enzyme | 0 | Not detected | 0.027 | -0.189 | 0.244 | 0.804 |
| ENZRXN185 | phosphoglucomutase | 0 | Not detected | 0.254 | -0.072 | 0.580 | 0.127 |
| ENZRXN1F. | glutamate-1-semialdehyde aminotransfe | 0 | Not detected | 0.006 | -0.309 | 0.321 | 0.970 |
| ENZRXN3O. | aconitate hydratase | 0 | Not detected | 0.263 | -0.170 | 0.697 | 0.234 |
| ENZRXN3O. | aconitate hydratase | 0 | Not detected | 0.263 | -0.170 | 0.697 | 0.234 |
| ENZRXN490 | enolase | 0 | Not detected | -0.166 | -0.447 | 0.115 | 0.248 |
| ENZRXN66. | pyruvate carboxylase | 0 | Not detected | -0.018 | -0.289 | 0.253 | 0.895 |
| ENZRXN66. | citrate dehydratase | 0 | Not detected | 0.029 | -0.220 | 0.278 | 0.817 |
| ENZRXN66. | aconitate hydratase | 0 | Not detected | 0.029 | -0.220 | 0.278 | 0.817 |
| ENZRXN8J2 | 1-deoxyxylulose-5-phosphate synthase | 0 | Not detected | 0.002 | -0.322 | 0.325 | 0.991 |
| ENZRXN8J2 | GTP cyclohydrolase | 0 | Not detected | 0.064 | -0.104 | 0.233 | 0.455 |
| ENZRXN8J2 | orotate phosphoribosyltransferase | 0 | Not detected | -0.009 | -0.145 | 0.126 | 0.891 |
| ENZRXN8J2 | orotidine 5'-phosphate decarboxylase | 0 | Not detected | 0.038 | -0.143 | 0.219 | 0.684 |
| ENZRXN8J2 | aspartate carbamoyltransferase | 0 | Not detected | 0.178 | -0.112 | 0.467 | 0.229 |
| ENZRXN8J2 | UDP-glucose 6-dehydrogenase | 0 | Not detected | -0.140 | -0.396 | 0.117 | 0.286 |
| ENZRXN8J2 | 5-dehydro-2-deoxy-D-gluconate kinase | 0 | Not detected | -0.081 | -0.349 | 0.187 | 0.555 |
| ENZRXN8J2 | methionine-glutamine aminotransferase | 0 | Not detected | -0.010 | -0.249 | 0.229 | 0.934 |
| ENZRXN8J2 | lipoyl synthase | 0 | Not detected | -0.137 | -0.378 | 0.104 | 0.266 |
| ENZRXN8J2 | 4-nitrobenzaldehyde reductase | 0 | Not detected | 0.023 | -0.235 | 0.281 | 0.859 |
| ENZRXN8J2 | CTP synthetase | 0 | Not detected | 0.009 | -0.115 | 0.133 | 0.884 |
| ENZRXN8J2 | UTP-glucose-1-phosphate uridylyltransf | 0 | Not detected | 0.025 | -0.168 | 0.217 | 0.801 |
| ENZRXN8J2 | 1,5-Î±-L-arabinofuranosidase | 0 | Not detected | 0.080 | -0.134 | 0.294 | 0.463 |
| ENZRXN8J2 | 1,3-Î±-L-arabinofuranosidase | 0 | Not detected | -0.002 | -0.138 | 0.134 | 0.979 |
| ENZRXNI.14 | fumarase | 0 | Not detected | -0.024 | -0.265 | 0.216 | 0.843 |
| ENZRXNIO2 | alcohol dehydrogenase | 0 | Not detected | -0.095 | -0.348 | 0.158 | 0.462 |
| ENZRXNME | oxepin-CoA hydrolase | 0 | Not detected | -0.077 | -0.278 | 0.125 | 0.457 |
| ENZRXNME | 3-oxo-5,6-dehydrosuberyl-CoA semiald | 0 | Not detected | -0.077 | -0.278 | 0.125 | 0.457 |
| ENZRXNQT | phosphomethylpyrimidine synthase | 0 | Not detected | 0.114 | -0.158 | 0.387 | 0.411 |
| ENZRXNQT | biotin carboxylase | 0 | Not detected | -0.038 | -0.184 | 0.108 | 0.612 |
| ENZRXNQT | H+-exporting diphosphatase | 0 | Not detected | 0.044 | -0.240 | 0.328 | 0.762 |
| ENZRXNQT | NADP+-dependent isocitrate dehydroge | 0 | Not detected | -0.038 | -0.284 | 0.207 | 0.759 |
| ENZRXNQT | tryptophan synthase | 0 | Not detected | 0.144 | -0.125 | 0.414 | 0.295 |

| ENZRXNQT | aconitase | 0 | Not detected | 0.133 | -0.147 | 0.412 | 0.352 |
| --- | --- | --- | --- | --- | --- | --- | --- |
| ENZRXNQT | 3,4 dihydroxy-2-butanone-4-phosphate | 0 | Not detected | -0.018 | -0.145 | 0.110 | 0.785 |
| ENZRXNQT | GTP cyclohydrolase | 0 | Not detected | -0.018 | -0.145 | 0.110 | 0.785 |
| ENZRXNT9. | aconitase | 0 | Not detected | 0.133 | -0.147 | 0.412 | 0.352 |
| FADSYN.EN | FMN adenylyltransferase | 0 | Not detected | -0.124 | -0.316 | 0.068 | 0.204 |
| FGAMSYN.E | phosphoribosylformylglycinamide synth | 0 | Not detected | 0.073 | -0.152 | 0.299 | 0.523 |
| FLAVONAD | flavodoxin-NADP+ reductase | 0 | Not detected | 0.102 | -0.066 | 0.269 | 0.234 |
| FOLYLPOLY | folylpoly-&gamma;-glutamate synthetas | 0 | Not detected | -0.017 | -0.216 | 0.182 | 0.867 |
| FORMYLTH | 10-formyl-tetrahydrofolylpolyglutamate | 0 | Not detected | -0.017 | -0.216 | 0.182 | 0.867 |
| FPPSYN.EN | farnesyl diphosphate synthase | 0 | Not detected | 0.063 | -0.152 | 0.277 | 0.568 |
| FUCULOKIN | L-fuculokinase | 0 | Not detected | -0.071 | -0.349 | 0.207 | 0.615 |
| GALACTAR | galactarate dehydratase | 0 | Not detected | 0.076 | -0.182 | 0.334 | 0.564 |
| GALACTOK | galactokinase | 0 | Not detected | 0.096 | -0.106 | 0.298 | 0.351 |
| GALACTON | D-galactonate dehydratase | 0 | Not detected | 0.035 | -0.204 | 0.274 | 0.776 |
| GALACTUR | D-galacturonate isomerase | 0 | Not detected | -0.019 | -0.138 | 0.100 | 0.752 |
| GALPMUT.E | UDP-galactopyranose mutase | 0 | Not detected | -0.045 | -0.174 | 0.085 | 0.498 |
| GARTRANS | phosphoribosylglycinamide formyltrans | 0 | Not detected | -0.066 | -0.272 | 0.140 | 0.528 |
| GARTSIDE.E | acetate kinase | 0 | Not detected | -0.066 | -0.272 | 0.140 | 0.528 |
| GCVT.ENZR | aminomethyltransferase | 0 | Not detected | -0.018 | -0.209 | 0.173 | 0.850 |
| GDPPYPHO | GDP pyrophosphokinase | 0 | Not detected | -0.184 | -0.431 | 0.062 | 0.143 |
| GDPPYPHO | GDP diphosphokinase | 0 | Not detected | -0.201 | -0.467 | 0.066 | 0.140 |
| GKI.ENZRX | glycerate 2-kinase | 0 | Not detected | -0.030 | -0.261 | 0.201 | 0.799 |
| GLNS.ENZR | glutamine&mdash;tRNA ligase | 0 | Not detected | -0.006 | -0.125 | 0.114 | 0.927 |
| GLU6PDEH | NADP+-dependent glucose-6-phosphate | 0 | Not detected | 0.013 | -0.170 | 0.195 | 0.893 |
| GLUCARDE | D-glucarate dehydratase | 0 | Not detected | -0.246 | -0.502 | 0.011 | 0.060 |
| GLUCUROIS | D-glucuronate isomerase | 0 | Not detected | -0.019 | -0.138 | 0.100 | 0.752 |
| GLURS.ENZ | glutamate&mdash;tRNA ligase | 0 | Not detected | 0.027 | -0.252 | 0.305 | 0.851 |
| GLUTCYSLI | glutamate&mdash;cysteine ligase | 0 | Not detected | -0.027 | -0.229 | 0.175 | 0.795 |
| GLUTRACE. | glutamate racemase | 0 | Not detected | -0.039 | -0.211 | 0.134 | 0.661 |
| GLYCEROL. | acyl-CoA:sn-glycerol-3-phosphate 1-O- | 0 | Not detected | -0.076 | -0.332 | 0.181 | 0.563 |
| GLYCOGEN | 1,4-Î±-glucan branching enzyme | 0 | Not detected | 0.200 | -0.014 | 0.413 | 0.067 |
| GLYCOGEN | glycogen synthase | 0 | Not detected | 0.102 | -0.112 | 0.317 | 0.349 |
| GLYCPDIES | glycerophosphodiester phosphodiesteras | 0 | Not detected | 0.002 | -0.165 | 0.170 | 0.977 |
| GLYRIBON | phosphoribosylamine&mdash;glycine li | 0 | Not detected | -0.037 | -0.170 | 0.095 | 0.582 |
| GPH.ENZRX | phosphoglycolate phosphatase | 0 | Not detected | -0.067 | -0.236 | 0.103 | 0.441 |
| GPPSYN.EN | geranyl diphosphate synthase | 0 | Not detected | 0.063 | -0.152 | 0.277 | 0.568 |
| GSADENYL | glutamine synthetase adenylyltransferase | 0 | Not detected | -0.014 | -0.277 | 0.250 | 0.918 |
| GSDEADEN | glutamine synthetase deadenylase | 0 | Not detected | -0.014 | -0.277 | 0.250 | 0.918 |
| GTPPYPHOS | GTP pyrophosphokinase | 0 | Not detected | -0.184 | -0.431 | 0.062 | 0.143 |
| HEMEOSYN | heme O synthase | 0 | Not detected | 0.050 | -0.130 | 0.231 | 0.583 |
| HEMN.ENZR | coproporphyrinogen III dehydrogenase | 0 | Not detected | 0.104 | -0.129 | 0.337 | 0.383 |
| HISTCYCLO | phosphoribosyl-AMP cyclohydrolase | 0 | Not detected | -0.044 | -0.177 | 0.089 | 0.519 |
| HISTPRATP | phosphoribosyl-ATP pyrophosphatase | 0 | Not detected | -0.044 | -0.177 | 0.089 | 0.519 |
| HOMOCYSM | cobalamin-independent homocysteine tr | 0 | Not detected | 0.030 | -0.227 | 0.287 | 0.821 |

| HOMOCYSM | methionine synthase | 0 | Not detected | -0.030 | -0.151 | 0.091 | 0.627 |
| --- | --- | --- | --- | --- | --- | --- | --- |
| IGPSYN.EN | indole-3-glycerol phosphate synthase | 0 | Not detected | -0.002 | -0.208 | 0.204 | 0.985 |
| ILES.ENZRX | isoleucine&mdash;tRNA ligase | 0 | Not detected | -0.070 | -0.363 | 0.223 | 0.640 |
| IMPCYCLO | IMP cyclohydrolase | 0 | Not detected | 0.092 | -0.091 | 0.275 | 0.324 |
| KDOTRANS | 3-deoxy-D-manno-octulosonic acid tran | 0 | Not detected | 0.070 | -0.145 | 0.284 | 0.526 |
| KDOTRANS | KDO transferase | 0 | Not detected | 0.070 | -0.145 | 0.284 | 0.526 |
| L.ASPARTA | L-aspartate oxidase | 0 | Not detected | -0.034 | -0.338 | 0.269 | 0.824 |
| L.LACTDEH | L-lactate dehydrogenase | 0 | Not detected | 0.074 | -0.144 | 0.293 | 0.505 |
| LAUROYLA | lauroyl acyltransferase | 0 | Not detected | 0.018 | -0.165 | 0.201 | 0.848 |
| LEUS.ENZR | leucine&mdash;tRNA ligase | 0 | Not detected | -0.086 | -0.378 | 0.207 | 0.566 |
| LSERINEDE | L-serine deaminase | 0 | Not detected | -0.144 | -0.436 | 0.149 | 0.337 |
| LSERINEDE | L-serine deaminase | 0 | Not detected | -0.010 | -0.216 | 0.196 | 0.922 |
| MALATESY | malate synthase A | 0 | Not detected | -0.006 | -0.217 | 0.205 | 0.956 |
| MALONYL. | [acyl-carrier-protein] S-malonyltransfera | 0 | Not detected | 0.115 | -0.141 | 0.371 | 0.379 |
| MALSYNG.E | malate synthase G | 0 | Not detected | 0.050 | -0.164 | 0.263 | 0.649 |
| MALTODEX | maltodextrin glucosidase | 0 | Not detected | 0.032 | -0.176 | 0.240 | 0.764 |
| MANNKIN.E | mannokinase | 0 | Not detected | 0.013 | -0.158 | 0.185 | 0.878 |
| MANNOND | D-mannonate dehydratase | 0 | Not detected | -0.121 | -0.282 | 0.040 | 0.139 |
| MANNONO | D-mannonate oxidoreductase | 0 | Not detected | 0.110 | -0.126 | 0.347 | 0.361 |
| MANNPDEH | mannitol-1-phosphate 5-dehydrogenase | 0 | Not detected | 0.059 | -0.204 | 0.322 | 0.660 |
| MHPHYDRO | 3-(3-hydroxyphenyl)propanoate hydrox | 0 | Not detected | -0.026 | -0.224 | 0.172 | 0.797 |
| N.ACETYLG | N-acetylglutamylphosphate reductase | 0 | Not detected | 0.076 | -0.166 | 0.319 | 0.538 |
| NACGLCTR | N-acetylglucosaminyl transferase | 0 | Not detected | -0.034 | -0.228 | 0.161 | 0.735 |
| NACMURLA | N-acetylmuramoyl-L-alanine amidase | 0 | Not detected | 0.026 | -0.177 | 0.228 | 0.805 |
| NADH.DHII. | NADH:ubiquinone oxidoreductase | 0 | Not detected | 0.041 | -0.161 | 0.242 | 0.693 |
| NANK.ENZR | N-acetylmannosamine kinase | 0 | Not detected | -0.018 | -0.202 | 0.166 | 0.850 |
| NICOTINAT | nicotinate phosphoribosyltransferase | 0 | Not detected | -0.062 | -0.244 | 0.120 | 0.503 |
| O.SUCCINY | o-succinylbenzoate synthase | 0 | Not detected | -0.078 | -0.271 | 0.115 | 0.427 |
| OCTPRNL.M | 2-octaprenyl-3-methyl-6-methoxy-1,4-b | 0 | Not detected | -0.027 | -0.216 | 0.162 | 0.780 |
| OHMETHYL | hydroxymethylbilane synthase | 0 | Not detected | 0.159 | -0.035 | 0.354 | 0.108 |
| PEPCARBO | phosphoenolpyruvate carboxykinase (A | 0 | Not detected | -0.010 | -0.131 | 0.111 | 0.870 |
| PGLYCERO | phosphoglycerol transferase | 0 | Not detected | 0.062 | -0.162 | 0.286 | 0.586 |
| PGMI.ENZR | 2,3-bisphosphoglycerate-independent ph | 0 | Not detected | -0.014 | -0.134 | 0.106 | 0.821 |
| PHOSGLUC | phosphoglucosamine mutase | 0 | Not detected | 0.027 | -0.233 | 0.286 | 0.839 |
| PHOSGLYC | phosphoglycerate kinase | 0 | Not detected | 0.001 | -0.247 | 0.249 | 0.993 |
| PHOSMANM | phosphomannomutase | 0 | Not detected | -0.024 | -0.312 | 0.264 | 0.871 |
| PHOSNACM | phospho-N-acetylmuramoyl-pentapeptid | 0 | Not detected | -0.098 | -0.355 | 0.160 | 0.457 |
| PHOSPHASE | phosphatidylserine synthase | 0 | Not detected | -0.049 | -0.274 | 0.175 | 0.666 |
| PHOSPHOG | phosphoglucomutase | 0 | Not detected | -0.172 | -0.418 | 0.074 | 0.170 |
| PPENTOMU | Î±-D-ribose 1,5-phosphomutase | 0 | Not detected | -0.030 | -0.316 | 0.257 | 0.838 |
| PRAISOM.E | phosphoribosylanthranilate isomerase | 0 | Not detected | -0.002 | -0.208 | 0.204 | 0.985 |
| PRIBFAICA | 1-(5-phosphoribosyl)-5-[(5-phosphoribo | 0 | Not detected | 0.031 | -0.217 | 0.278 | 0.807 |
| PROPKIN.E | propionate kinase | 0 | Not detected | 0.048 | -0.133 | 0.229 | 0.603 |
| PROPKIN2.E | propionate kinase | 0 | Not detected | 0.164 | -0.044 | 0.372 | 0.123 |

| PROTOHEM | ferrochelatase | 0 | Not detected | -0.055 | -0.254 | 0.144 | 0.587 |
| --- | --- | --- | --- | --- | --- | --- | --- |
| PRPPSYN.E | ribose-phosphate diphosphokinase | 0 | Not detected | -0.030 | -0.162 | 0.101 | 0.651 |
| PSERPHOSP | phosphoserine phosphatase | 0 | Not detected | 0.025 | -0.172 | 0.222 | 0.804 |
| PSII.ENZRX | guanosine-3',5'-bis(diphosphate) 3'-diph | 0 | Not detected | -0.201 | -0.467 | 0.066 | 0.140 |
| RHAMNULO | rhamnulokinase | 0 | Not detected | 0.130 | -0.080 | 0.341 | 0.226 |
| RIBOFLAVI | riboflavin kinase | 0 | Not detected | -0.124 | -0.316 | 0.068 | 0.204 |
| RIBULP3EPI | ribulose-phosphate 3-epimerase | 0 | Not detected | 0.011 | -0.176 | 0.199 | 0.905 |
| SHIKIMATE | shikimate dehydrogenase | 0 | Not detected | -0.128 | -0.307 | 0.050 | 0.160 |
| SHIKIMATE | shikimate kinase | 0 | Not detected | 0.066 | -0.112 | 0.244 | 0.469 |
| SUCCGLUA | succinylglutamate semialdehyde dehydr | 0 | Not detected | -0.087 | -0.291 | 0.117 | 0.405 |
| TDPFUCAC | dTDP-4-amino-4,6-dideoxy-D-galactose | 0 | Not detected | -0.106 | -0.272 | 0.060 | 0.210 |
| TETRAACY | tetraacyldisaccharide 4'-kinase | 0 | Not detected | 0.048 | -0.129 | 0.225 | 0.592 |
| THI.P.KIN.E | thiamine monophosphate kinase | 0 | Not detected | 0.006 | -0.183 | 0.194 | 0.954 |
| THIC.ENZR | phosphomethylpyrimidine synthase | 0 | Not detected | 0.000 | -0.126 | 0.127 | 0.998 |
| THRESYN.E | threonine synthase | 0 | Not detected | -0.103 | -0.337 | 0.131 | 0.388 |
| TRANS.ENZ | transport of arabinose | 0 | Not detected | 0.004 | -0.248 | 0.256 | 0.974 |
| TRANS.ENZ | (S)-lactate transporter | 0 | Not detected | 0.133 | -0.093 | 0.359 | 0.249 |
| TRANS.ENZ | glycolate transporter | 0 | Not detected | 0.097 | -0.162 | 0.356 | 0.465 |
| TRANS.ENZ | succinate:fumarate antiport | 0 | Not detected | -0.048 | -0.303 | 0.207 | 0.711 |
| TRANS.ENZ | fumarate:succinate antiporter | 0 | Not detected | -0.114 | -0.333 | 0.105 | 0.307 |
| TRANS.ENZ | transport of adenosine | 0 | Not detected | 0.212 | -0.013 | 0.437 | 0.065 |
| TRANS.ENZ | transport of cytidine | 0 | Not detected | 0.212 | -0.013 | 0.437 | 0.065 |
| TRANS.ENZ | transport of deoxyadenosine | 0 | Not detected | 0.212 | -0.013 | 0.437 | 0.065 |
| TRANS.ENZ | transport of deoxycytidine | 0 | Not detected | 0.212 | -0.013 | 0.437 | 0.065 |
| TRANS.ENZ | transport of deoxyuridine | 0 | Not detected | 0.212 | -0.013 | 0.437 | 0.065 |
| TRANS.ENZ | transport of thymidine | 0 | Not detected | 0.212 | -0.013 | 0.437 | 0.065 |
| TRANS.ENZ | transport of uridine | 0 | Not detected | 0.212 | -0.013 | 0.437 | 0.065 |
| TRANS.ENZ | transport of rhamnose | 0 | Not detected | -0.027 | -0.212 | 0.158 | 0.777 |
| TRANS.ENZ | phosphate:H+ symporter | 0 | Not detected | -0.023 | -0.238 | 0.192 | 0.836 |
| TRANS.ENZ | cytosine transporter | 0 | Not detected | 0.023 | -0.205 | 0.250 | 0.846 |
| TRANS.ENZ | pantothenate:Na+ symporter | 0 | Not detected | 0.033 | -0.200 | 0.266 | 0.781 |
| TRANS.ENZ | proline:Na+ symporter | 0 | Not detected | -0.024 | -0.290 | 0.242 | 0.861 |
| TRANS.ENZ | transport of succinate | 0 | Not detected | -0.152 | -0.421 | 0.116 | 0.266 |
| TRANS.ENZ | transport of malate | 0 | Not detected | -0.152 | -0.421 | 0.116 | 0.266 |
| TRANS.ENZ | transport of fumarate | 0 | Not detected | -0.152 | -0.421 | 0.116 | 0.266 |
| TRANS.ENZ | transport of orotate | 0 | Not detected | -0.152 | -0.421 | 0.116 | 0.266 |
| TRANS.ENZ | L-glutamate:H+ symporter | 0 | Not detected | -0.015 | -0.217 | 0.186 | 0.880 |
| TRANS.ENZ | L-aspartate:H+ symporter | 0 | Not detected | -0.015 | -0.217 | 0.186 | 0.880 |
| TRANS.ENZ | transport of glutamate | 0 | Not detected | -0.042 | -0.267 | 0.183 | 0.715 |
| TRANS.ENZ | branched chain amino acid transporter B | 0 | Not detected | 0.008 | -0.187 | 0.202 | 0.938 |
| TRANS.ENZ | branched chain amino acid transporter B | 0 | Not detected | 0.008 | -0.187 | 0.202 | 0.938 |
| TRANS.ENZ | branched chain amino acid transporter B | 0 | Not detected | 0.008 | -0.187 | 0.202 | 0.938 |
| TRANS.ENZ | L-tartrate:succinate antiporter | 0 | Not detected | -0.054 | -0.243 | 0.135 | 0.578 |
| TRANS.ENZ | Na+:H+ antiporter | 0 | Not detected | -0.014 | -0.251 | 0.224 | 0.910 |

| TRANS.ENZ | Mg2+ transporter | 0 | Not detected | -0.162 | -0.365 | 0.041 | 0.119 |
| --- | --- | --- | --- | --- | --- | --- | --- |
| TRANS.ENZ | Co2+ transporter | 0 | Not detected | -0.162 | -0.365 | 0.041 | 0.119 |
| TRANS.ENZ | Ni2+ transporter | 0 | Not detected | -0.162 | -0.365 | 0.041 | 0.119 |
| TRANS.ENZ | K+ : H+ antiporter KefB | 0 | Not detected | 0.008 | -0.210 | 0.227 | 0.939 |
| TRANS.ENZ | K+:H+ symporter Kup | 0 | Not detected | 0.030 | -0.190 | 0.249 | 0.793 |
| TRANS.ENZ | fucose:H+ symporter | 0 | Not detected | 0.013 | -0.171 | 0.197 | 0.891 |
| TRANS.ENZ | shikimate:H+ symporter | 0 | Not detected | 0.039 | -0.137 | 0.214 | 0.664 |
| TRANS.ENZ | fosmidomycin efflux pump | 0 | Not detected | -0.034 | -0.182 | 0.114 | 0.650 |
| TRANS.ENZ | transport of 3-(3-hydroxyphenyl)propio | 0 | Not detected | -0.086 | -0.272 | 0.099 | 0.362 |
| TRANS.ENZ | sugar exporter SetA | 0 | Not detected | -0.167 | -0.353 | 0.018 | 0.077 |
| TRANS.ENZ | sugar exporter SetB | 0 | Not detected | -0.006 | -0.207 | 0.196 | 0.957 |
| TRANS.ENZ | transport of D-galactose | 0 | Not detected | -0.041 | -0.235 | 0.154 | 0.684 |
| TRANS.ENZ | Mn2+:H+ symporter | 0 | Not detected | -0.008 | -0.186 | 0.170 | 0.929 |
| TRANS.ENZ | L-threonine:H+ antiporter | 0 | Not detected | -0.017 | -0.224 | 0.190 | 0.872 |
| TRANS.ENZ | K+ transporter TrkH | 0 | Not detected | -0.122 | -0.344 | 0.101 | 0.284 |
| TRANS.ENZ | sn-glycerol 3-phosphate:phosphate antip | 0 | Not detected | -0.173 | -0.414 | 0.069 | 0.161 |
| TRANS.ENZ | transport of 2-oxoglutarate | 0 | Not detected | 0.090 | -0.099 | 0.278 | 0.352 |
| TRANS.ENZ | N-acetylneuraminate:H+ symporter | 0 | Not detected | 0.180 | -0.020 | 0.379 | 0.078 |
| TRANS.ENZ | transport of a nucleoside | 0 | Not detected | 0.041 | -0.257 | 0.340 | 0.786 |
| TRANS.ENZ | transport of D-xylose | 0 | Not detected | -0.125 | -0.320 | 0.070 | 0.209 |
| TRANS.ENZ | xanthosine:H+ symporter XapB | 0 | Not detected | -0.153 | -0.356 | 0.051 | 0.141 |
| TRANS.ENZ | glucose-6-phosphate:phosphate antiport | 0 | Not detected | 0.023 | -0.184 | 0.229 | 0.829 |
| TRANS.ENZ | D-glucuronate transporter | 0 | Not detected | 0.069 | -0.126 | 0.264 | 0.486 |
| TRANS.ENZ | D-galacturonate transporter | 0 | Not detected | 0.069 | -0.126 | 0.264 | 0.486 |
| TRANS.ENZ | L-arabinose exporter | 0 | Not detected | -0.049 | -0.220 | 0.121 | 0.572 |
| TRANS.ENZ | L-phenylalanine:H+ symporter | 0 | Not detected | 0.043 | -0.191 | 0.277 | 0.720 |
| TRANS.ENZ | L-tyrosine:H+ symporter | 0 | Not detected | 0.043 | -0.191 | 0.277 | 0.720 |
| TRANS.ENZ | L-tryptophan:H+ symporter | 0 | Not detected | 0.043 | -0.191 | 0.277 | 0.720 |
| TRANS.ENZ | 4-aminobutanoate:H+ symporter | 0 | Not detected | -0.055 | -0.244 | 0.134 | 0.571 |
| TRANS.ENZ | lysine:H+ symporter | 0 | Not detected | 0.168 | -0.110 | 0.445 | 0.237 |
| TRANS.ENZ | phenylalanine:H+ symporter PheP | 0 | Not detected | 0.054 | -0.194 | 0.303 | 0.668 |
| TRANS.ENZ | transport of D-alanine | 0 | Not detected | 0.116 | -0.121 | 0.353 | 0.339 |
| TRANS.ENZ | transport of glycine | 0 | Not detected | 0.116 | -0.121 | 0.353 | 0.339 |
| TRANS.ENZ | lysine:cadaverine antiporter | 0 | Not detected | 0.242 | -0.016 | 0.500 | 0.066 |
| TRANS.ENZ | serine:H+ symporter SdaC | 0 | Not detected | -0.054 | -0.292 | 0.185 | 0.658 |
| TRANS.ENZ | L-threonine:H+ symporter | 0 | Not detected | 0.021 | -0.173 | 0.214 | 0.833 |
| TRANS.ENZ | L-serine:H+ symporter | 0 | Not detected | 0.021 | -0.173 | 0.214 | 0.833 |
| TRANS.ENZ | tryptophan:H+ symporter | 0 | Not detected | -0.016 | -0.233 | 0.202 | 0.886 |
| TRANS.ENZ | indole:H+ symporter | 0 | Not detected | -0.016 | -0.233 | 0.202 | 0.886 |
| TRANS.ENZ | choline:H+ symporter | 0 | Not detected | -0.002 | -0.224 | 0.220 | 0.986 |
| TRE6PHYDR | trehalose-6-phosphate hydrolase | 0 | Not detected | -0.124 | -0.392 | 0.145 | 0.367 |
| TREHALAC | trehalase | 0 | Not detected | 0.157 | -0.054 | 0.367 | 0.146 |
| TREHALAPE | trehalase | 0 | Not detected | -0.009 | -0.199 | 0.181 | 0.926 |
| TREHALOSE | trehalose-6-phosphate synthase | 0 | Not detected | 0.113 | -0.068 | 0.295 | 0.222 |

| TSA.REDUC | tartronate semialdehyde reductase | 0 | Not detected | -0.121 | -0.341 | 0.100 | 0.283 |
| --- | --- | --- | --- | --- | --- | --- | --- |
| UDP.NACM | UDP-N-acetylmuramoyl-L-alanine&md | 0 | Not detected | -0.119 | -0.336 | 0.099 | 0.287 |
| UDP.NACM | D-alanyl-D-alanine-adding enzyme | 0 | Not detected | 0.003 | -0.210 | 0.216 | 0.980 |
| UDP.NACM | UDP-N-acetylmuramoyl-L-alanyl-D-glu | 0 | Not detected | -0.003 | -0.229 | 0.222 | 0.977 |
| UDPACYLG | UDP-3-O-acyl-N-acetylglucosamine dea | 0 | Not detected | -0.033 | -0.307 | 0.241 | 0.815 |
| UDPNACET | UDP-N-acetylglucosamine 1-carboxyvin | 0 | Not detected | 0.096 | -0.148 | 0.341 | 0.440 |
| UDPNACET | UDP-N-acetylenolpyruvoylglucosamine | 0 | Not detected | 0.036 | -0.163 | 0.236 | 0.722 |
| UDPSUGAR | UDP-sugar hydrolase | 0 | Not detected | -0.135 | -0.366 | 0.096 | 0.251 |
| URITRANS. | [protein-PII] uridylyltransferase | 0 | Not detected | 0.115 | -0.117 | 0.348 | 0.332 |
| URKI.ENZR | uridine kinase | 0 | Not detected | 0.029 | -0.232 | 0.290 | 0.829 |
| UROGENDE | uroporphyrinogen decarboxylase | 0 | Not detected | 0.038 | -0.224 | 0.300 | 0.777 |
| VALINE.PY | valine&mdash;pyruvate aminotransferas | 0 | Not detected | 0.042 | -0.154 | 0.237 | 0.677 |
| VALS.ENZR | valine&mdash;tRNA ligase | 0 | Not detected | 0.097 | -0.202 | 0.396 | 0.525 |
| X1.ACYLGL | acyl-CoA:1-acyl-sn-glycerol-3-phosphat | 0 | Not detected | -0.056 | -0.237 | 0.125 | 0.543 |
| X2.OCTAPR | 2-octaprenyl-6-methoxy-1,4-benzoquino | 0 | Not detected | 0.032 | -0.235 | 0.300 | 0.812 |
| X2.OCTPRN | 2-octaprenyl-6-methoxyphenol hydroxyl | 0 | Not detected | 0.079 | -0.113 | 0.271 | 0.419 |
| X3.DEHYDR | 3-dehydroquinate synthase | 0 | Not detected | -0.080 | -0.318 | 0.158 | 0.509 |
| X3.NUCLEO | 3'-nucleotidase | 0 | Not detected | 0.031 | -0.213 | 0.275 | 0.804 |
| X3.P.SHIK.1 | 3-phosphoshikimate 1-carboxyvinyltran | 0 | Not detected | 0.082 | -0.138 | 0.301 | 0.465 |
| X4OHBENZ | 4-hydroxybenzoate octaprenyltransferas | 0 | Not detected | 0.110 | -0.063 | 0.284 | 0.213 |
| X5.NUCLEO | 5'-ribonucleotidase | 0 | Not detected | -0.135 | -0.366 | 0.096 | 0.251 |

| **Supplementary Table 7.** Association of childhood and current economic hardship with gut microbiome Shannon diversity index. For childhood economic hardship, model was adjusted for age, gender, BMI, field center, Hispanic/Latino background, U.S. nativity, Bristol stool type, current SEP, the alternative Healthy Eating Index 2010 score, cigarette smoking, alcohol use, depressive and anxiety symptoms, diabetes, hypertension, cardiovascular disease, dyslipidemia, and antibiotic use. For current economic hardship, model was adjusted for age, gender, BMI, field center, Hispanic/Latino background, U.S. nativity, Bristol stool type, parental education, the alternative Healthy Eating Index 2010 score, cigarette smoking, alcohol use, depressive and anxiety symptoms, diabetes, hypertension, cardiovascular disease, dyslipidemia, and antibiotic use. | | | |
| --- | --- | --- | --- |
| **Exposure** | **index** | **Model 3** | **p** |
| Childhood economic hardship | Shannon | -0.0432 (-0.1024, 0.016) | 0.15 |
|  | Inverse Simpson | -1.3609 (-3.0222, 0.3003) | 0.11 |
|  | Chao1 | -14.154 (-41.9168, 13.6088) | 0.32 |
|  | Observed | -9.7531 (-31.0947, 11.5885) | 0.37 |
| Current economic hardship | Shannon | 0.0297 (-0.0325, 0.092) | 0.35 |
|  | Inverse Simpson | 0.1654 (-1.5834, 1.9142) | 0.85 |
|  | Chao1 | 12.6293 (-16.5931, 41.8518) | 0.40 |
|  | Observed | 13.1171 (-9.3147, 35.549) | 0.25 |

| **Supplementary Table 8.** Association of childhood and current economic hardship with the Jensen Shannon Divergence (JSD) in permutational multivariate analysis of variance (PERMANOVA). For childhood economic hardship, model was adjusted for age, gender, BMI, field center, Hispanic/Latino background, U.S. nativity, Bristol stool type, current SEP, the alternative Healthy Eating Index 2010 score, cigarette smoking, alcohol use, depressive and anxiety symptoms, diabetes, hypertension, cardiovascular disease, dyslipidemia, and antibiotic use. For current economic hardship, model was adjusted for age, gender, BMI, field center, Hispanic/Latino background, U.S. nativity, Bristol stool type, parental education, the alternative Healthy Eating Index 2010 score, cigarette smoking, alcohol use, depressive and anxiety symptoms, diabetes, hypertension, cardiovascular disease, dyslipidemia, and antibiotic use. | | |
| --- | --- | --- |
|  | **Model 3** | |
| **Exposure** | R-squared (%) | p |
| Childhood economic hardship | 0.092 | 0.44 |
| Current economic hardship | 0.061 | 0.69 |

| **Supplementary Table 9.** Association of childhood economic hardship with species. Analysis of Compositions of Microbiomes (ANCOM2) was used to identify species related to childhood economic hardship. Regression coefficients were obtained by modelling the effect of childhood economic hardship on centered log ratio (CLR)-transformed species in multivariable linear regression adjusted for age, gender, BMI, field center, Hispanic/Latino background, U.S. nativity, Bristol stool type, current SEP, the alternative Healthy Eating Index 2010 score, cigarette smoking, alcohol use, depressive and anxiety symptoms, diabetes, hypertension, cardiovascular disease, dyslipidemia, and antibiotic use. | | | | | | |
| --- | --- | --- | --- | --- | --- | --- |
| **species** | **W_stat** | **ANCOM** | **beta** | **l95ci** | **u95ci** | **qval** |
| Granulicatella adiacens | 835 | 0.7 | 0.436 | 0.194 | 0.678 | 0.000 |
| Haemophilus parainfluenzae | 846 | 0.7 | 0.447 | 0.084 | 0.811 | 0.016 |
| Haemophilus sputorum | 841 | 0.7 | 0.371 | 0.070 | 0.673 | 0.016 |
| Lachnospiraceae bacterium NLAE-zl-G231 | 954 | 0.8 | -0.329 | -0.541 | -0.116 | 0.002 |
| Abiotrophia defectiva | 0 | Not detected | 0.149 | -0.063 | 0.361 | 0.170 |
| Acetanaerobacterium elongatum | 0 | Not detected | -0.062 | -0.213 | 0.090 | 0.424 |
| Acetitomaculum ruminis | 0 | Not detected | -0.060 | -0.159 | 0.039 | 0.234 |
| Acetivibrio cellulolyticus | 0 | Not detected | -0.030 | -0.187 | 0.126 | 0.703 |
| Acetivibrio ethanolgignens | 0 | Not detected | -0.033 | -0.122 | 0.055 | 0.460 |
| Acetobacter sp. 46_36 | 0 | Not detected | -0.147 | -0.488 | 0.194 | 0.399 |
| Acetobacter sp. CAG:267 | 0 | Not detected | -0.120 | -0.422 | 0.183 | 0.438 |
| Acholeplasma axanthum | 0 | Not detected | 0.012 | -0.131 | 0.155 | 0.869 |
| Acholeplasma equifetale | 11 | Not detected | 0.138 | -0.059 | 0.335 | 0.171 |
| Acidaminococcus fermentans | 701 | Not detected | -0.251 | -0.419 | -0.084 | 0.003 |
| Acidaminococcus intestini | 0 | Not detected | -0.362 | -0.676 | -0.049 | 0.024 |
| Acidaminococcus intestini CAG:325 | 0 | Not detected | -0.374 | -0.827 | 0.079 | 0.106 |
| Acidaminococcus sp. CAG:542 | 0 | Not detected | -0.218 | -0.449 | 0.013 | 0.065 |
| Acinetobacter baumannii | 0 | Not detected | -0.057 | -0.228 | 0.114 | 0.511 |
| Acinetobacter bereziniae | 0 | Not detected | -0.120 | -0.288 | 0.048 | 0.161 |
| Acinetobacter johnsonii | 0 | Not detected | -0.069 | -0.255 | 0.117 | 0.466 |
| Acinetobacter sp. CAG:196 | 0 | Not detected | -0.194 | -0.535 | 0.147 | 0.265 |
| Acinetobacter sp. CAG:196_36_41 | 0 | Not detected | -0.185 | -0.515 | 0.145 | 0.272 |
| Acinetobacter sp. N54.MGS-139 | 0 | Not detected | 0.081 | -0.127 | 0.290 | 0.446 |
| Acinetobacter venetianus | 0 | Not detected | 0.155 | -0.054 | 0.365 | 0.146 |
| Actinobaculum sp. oral taxon 183 | 0 | Not detected | 0.121 | -0.123 | 0.365 | 0.330 |
| Actinobaculum suis | 0 | Not detected | -0.025 | -0.200 | 0.150 | 0.781 |
| Actinomyces dentalis | 0 | Not detected | 0.029 | -0.224 | 0.281 | 0.825 |
| Actinomyces gerencseriae | 0 | Not detected | -0.091 | -0.312 | 0.130 | 0.420 |
| Actinomyces graevenitzii | 1 | Not detected | -0.128 | -0.302 | 0.046 | 0.150 |
| Actinomyces hongkongensis | 0 | Not detected | -0.045 | -0.269 | 0.179 | 0.696 |
| Actinomyces israelii | 0 | Not detected | -0.067 | -0.303 | 0.169 | 0.579 |
| Actinomyces johnsonii | 0 | Not detected | 0.066 | -0.170 | 0.303 | 0.584 |
| Actinomyces massiliensis | 0 | Not detected | -0.074 | -0.306 | 0.157 | 0.530 |
| Actinomyces meyeri | 0 | Not detected | 0.038 | -0.152 | 0.227 | 0.697 |
| Actinomyces odontolyticus | 0 | Not detected | -0.025 | -0.216 | 0.167 | 0.801 |
| Actinomyces oris | 0 | Not detected | 0.105 | -0.122 | 0.332 | 0.366 |
| Actinomyces sp. HMSC065F12 | 0 | Not detected | -0.032 | -0.164 | 0.100 | 0.636 |

| Actinomyces sp. HMSC06A08 | 0 | Not detected | -0.015 | -0.149 | 0.119 | 0.830 |
| --- | --- | --- | --- | --- | --- | --- |
| Actinomyces sp. oral taxon 181 | 0 | Not detected | -0.032 | -0.266 | 0.202 | 0.790 |
| Actinomyces sp. oral taxon 448 | 0 | Not detected | -0.122 | -0.385 | 0.142 | 0.365 |
| Actinomyces sp. oral taxon 848 | 0 | Not detected | 0.080 | -0.095 | 0.255 | 0.370 |
| Actinomyces sp. ph3 | 0 | Not detected | -0.020 | -0.242 | 0.203 | 0.863 |
| Actinomyces timonensis | 0 | Not detected | 0.094 | -0.053 | 0.241 | 0.211 |
| Actinomyces turicensis | 0 | Not detected | 0.018 | -0.204 | 0.241 | 0.871 |
| Adlercreutzia equolifaciens | 0 | Not detected | 0.076 | -0.221 | 0.374 | 0.616 |
| Aeromonas salmonicida | 0 | Not detected | -0.201 | -0.420 | 0.017 | 0.071 |
| Aggregatibacter aphrophilus | 533 | Not detected | 0.354 | 0.108 | 0.600 | 0.005 |
| Akkermansia glycaniphila | 0 | Not detected | -0.190 | -0.411 | 0.030 | 0.091 |
| Akkermansia muciniphila | 0 | Not detected | -0.200 | -0.528 | 0.128 | 0.233 |
| Akkermansia muciniphila CAG:154 | 0 | Not detected | -0.359 | -0.777 | 0.060 | 0.093 |
| Akkermansia sp. 54_46 | 0 | Not detected | -0.420 | -0.852 | 0.012 | 0.057 |
| Akkermansia sp. CAG:344 | 0 | Not detected | -0.275 | -0.708 | 0.157 | 0.213 |
| Akkermansia sp. KLE1605 | 0 | Not detected | -0.311 | -0.663 | 0.040 | 0.083 |
| Akkermansia sp. KLE1797 | 0 | Not detected | -0.328 | -0.680 | 0.023 | 0.068 |
| Akkermansia sp. KLE1798 | 0 | Not detected | -0.317 | -0.669 | 0.035 | 0.078 |
| Akkermansia sp. Phil8 | 0 | Not detected | -0.391 | -0.821 | 0.038 | 0.075 |
| Akkermansia sp. UNK.MGS-1 | 0 | Not detected | -0.453 | -0.880 | -0.025 | 0.038 |
| Alistipes finegoldii | 0 | Not detected | -0.130 | -0.311 | 0.051 | 0.160 |
| Alistipes ihumii | 0 | Not detected | -0.312 | -0.608 | -0.015 | 0.040 |
| Alistipes indistinctus | 0 | Not detected | -0.247 | -0.643 | 0.149 | 0.222 |
| Alistipes inops | 0 | Not detected | -0.279 | -0.597 | 0.040 | 0.087 |
| Alistipes obesi | 0 | Not detected | -0.087 | -0.284 | 0.110 | 0.388 |
| Alistipes putredinis | 0 | Not detected | -0.159 | -0.416 | 0.098 | 0.225 |
| Alistipes senegalensis | 0 | Not detected | -0.063 | -0.227 | 0.100 | 0.449 |
| Alistipes shahii | 0 | Not detected | -0.204 | -0.384 | -0.024 | 0.026 |
| Alistipes sp. CAG:435 | 0 | Not detected | 0.314 | -0.083 | 0.711 | 0.121 |
| Alistipes sp. CAG:514 | 0 | Not detected | 0.011 | -0.350 | 0.372 | 0.951 |
| Alistipes sp. Zagget8 | 0 | Not detected | -0.147 | -0.398 | 0.105 | 0.252 |
| Alistipes timonensis | 0 | Not detected | -0.090 | -0.353 | 0.173 | 0.503 |
| Alkaliflexus imshenetskii | 0 | Not detected | -0.105 | -0.272 | 0.061 | 0.216 |
| Allisonella histaminiformans | 0 | Not detected | -0.237 | -0.609 | 0.134 | 0.211 |
| Allobaculum stercoricanis | 20 | Not detected | -0.091 | -0.184 | 0.002 | 0.056 |
| Allofustis seminis | 0 | Not detected | -0.026 | -0.221 | 0.168 | 0.793 |
| Alloprevotella rava | 0 | Not detected | 0.022 | -0.140 | 0.184 | 0.792 |
| Alloprevotella tannerae | 0 | Not detected | 0.009 | -0.112 | 0.131 | 0.879 |
| Alloscardovia criceti | 0 | Not detected | -0.137 | -0.326 | 0.053 | 0.158 |
| Alloscardovia omnicolens | 0 | Not detected | 0.037 | -0.175 | 0.248 | 0.735 |
| Amphibacillus xylanus | 53 | Not detected | 0.172 | -0.031 | 0.374 | 0.096 |
| Anaerobium acetethylicum | 0 | Not detected | -0.043 | -0.138 | 0.051 | 0.369 |
| Anaerococcus hydrogenalis | 0 | Not detected | -0.126 | -0.266 | 0.015 | 0.081 |
| Anaerococcus lactolyticus | 0 | Not detected | -0.028 | -0.233 | 0.177 | 0.790 |

| Anaerococcus obesiensis | 0 | Not detected | -0.115 | -0.296 | 0.067 | 0.216 |
| --- | --- | --- | --- | --- | --- | --- |
| Anaerocolumna aminovalerica | 0 | Not detected | 0.086 | -0.088 | 0.260 | 0.333 |
| Anaerocolumna xylanovorans | 0 | Not detected | -0.016 | -0.172 | 0.141 | 0.844 |
| Anaerofustis stercorihominis | 0 | Not detected | 0.018 | -0.242 | 0.279 | 0.889 |
| Anaeroglobus geminatus | 0 | Not detected | -0.210 | -0.420 | 0.000 | 0.050 |
| Anaeromassilibacillus senegalensis | 0 | Not detected | -0.128 | -0.283 | 0.028 | 0.108 |
| Anaerophaga thermohalophila | 0 | Not detected | -0.047 | -0.169 | 0.076 | 0.453 |
| Anaerosalibacter massiliensis | 0 | Not detected | 0.169 | -0.047 | 0.385 | 0.126 |
| Anaerosporobacter mobilis | 0 | Not detected | -0.024 | -0.131 | 0.082 | 0.654 |
| Anaerosporomusa subterranea | 0 | Not detected | -0.068 | -0.209 | 0.073 | 0.343 |
| Anaerostipes caccae | 0 | Not detected | 0.009 | -0.095 | 0.113 | 0.862 |
| Anaerostipes hadrus | 0 | Not detected | 0.089 | -0.080 | 0.258 | 0.303 |
| Anaerostipes sp. CAG:276 | 0 | Not detected | -0.075 | -0.391 | 0.242 | 0.645 |
| Anaerotruncus colihominis | 0 | Not detected | -0.030 | -0.140 | 0.081 | 0.598 |
| Anaerotruncus sp. CAG:390 | 0 | Not detected | -0.089 | -0.392 | 0.214 | 0.565 |
| Anaerotruncus sp. CAG:528 | 0 | Not detected | 0.039 | -0.201 | 0.278 | 0.752 |
| Anaerotruncus sp. G3(2012) | 0 | Not detected | 0.010 | -0.074 | 0.095 | 0.809 |
| Anaerovorax odorimutans | 0 | Not detected | -0.081 | -0.234 | 0.071 | 0.296 |
| Angelakisella massiliensis | 0 | Not detected | -0.053 | -0.144 | 0.039 | 0.258 |
| Aphanizomenon flos-aquae | 0 | Not detected | 0.036 | -0.109 | 0.180 | 0.628 |
| Aquabacterium parvum | 0 | Not detected | 0.016 | -0.164 | 0.196 | 0.859 |
| Arcanobacterium haemolyticum | 0 | Not detected | -0.152 | -0.324 | 0.019 | 0.082 |
| Asaccharospora irregularis | 0 | Not detected | -0.090 | -0.297 | 0.116 | 0.392 |
| Atlantibacter hermannii | 0 | Not detected | 0.311 | 0.039 | 0.583 | 0.026 |
| Atopobium deltae | 310 | Not detected | -0.277 | -0.470 | -0.084 | 0.005 |
| Atopobium minutum | 0 | Not detected | -0.150 | -0.347 | 0.046 | 0.134 |
| Atopobium parvulum | 0 | Not detected | -0.031 | -0.250 | 0.189 | 0.785 |
| Atopobium rimae | 0 | Not detected | -0.110 | -0.286 | 0.066 | 0.220 |
| Atopobium sp. BS2 | 0 | Not detected | -0.055 | -0.273 | 0.162 | 0.616 |
| Atopobium sp. HMSC064B08 | 0 | Not detected | -0.030 | -0.181 | 0.120 | 0.692 |
| Atopobium sp. ICM42b | 0 | Not detected | -0.006 | -0.226 | 0.214 | 0.957 |
| Atopobium vaginae | 277 | Not detected | -0.280 | -0.481 | -0.079 | 0.007 |
| Atopostipes suicloacalis | 0 | Not detected | 0.018 | -0.174 | 0.210 | 0.852 |
| Avibacterium paragallinarum | 10 | Not detected | 0.233 | 0.061 | 0.404 | 0.008 |
| Azospirillum sp. 47_25 | 0 | Not detected | -0.214 | -0.622 | 0.195 | 0.306 |
| Azospirillum sp. CAG:260 | 0 | Not detected | -0.177 | -0.578 | 0.225 | 0.389 |
| Bacillus acidiproducens | 0 | Not detected | 0.034 | -0.182 | 0.250 | 0.758 |
| Bacillus andreraoultii | 0 | Not detected | 0.107 | -0.108 | 0.322 | 0.329 |
| Bacillus azotoformans | 0 | Not detected | -0.121 | -0.319 | 0.077 | 0.232 |
| Bacillus bataviensis | 0 | Not detected | -0.111 | -0.300 | 0.079 | 0.252 |
| Bacillus coahuilensis | 0 | Not detected | 0.057 | -0.166 | 0.279 | 0.617 |
| Bacillus rubiinfantis | 0 | Not detected | 0.101 | -0.090 | 0.292 | 0.301 |
| Bacillus sp. CAG:988 | 0 | Not detected | -0.025 | -0.206 | 0.155 | 0.782 |
| Bacillus thermotolerans | 0 | Not detected | 0.056 | -0.154 | 0.266 | 0.602 |

| Bacteroidales bacterium 45-6 | 0 | Not detected | -0.117 | -0.322 | 0.088 | 0.264 |
| --- | --- | --- | --- | --- | --- | --- |
| Bacteroidales bacterium 55_9 | 0 | Not detected | -0.103 | -0.223 | 0.017 | 0.094 |
| Bacteroidales bacterium Bact_08 | 0 | Not detected | -0.075 | -0.237 | 0.088 | 0.367 |
| Bacteroidales bacterium Bact_18 | 35 | Not detected | 0.184 | 0.027 | 0.341 | 0.022 |
| Bacteroidales bacterium Bact_23 | 0 | Not detected | -0.035 | -0.240 | 0.171 | 0.742 |
| Bacteroidales bacterium CF | 0 | Not detected | -0.086 | -0.257 | 0.085 | 0.325 |
| Bacteroidales bacterium H10 | 0 | Not detected | 0.007 | -0.163 | 0.176 | 0.940 |
| Bacteroidales bacterium K1 | 105 | Not detected | -0.258 | -0.446 | -0.070 | 0.007 |
| Bacteroidales bacterium K10 | 0 | Not detected | -0.067 | -0.268 | 0.133 | 0.511 |
| Bacteroidales bacterium KHT7 | 0 | Not detected | -0.067 | -0.278 | 0.144 | 0.531 |
| Bacteroides barnesiae | 0 | Not detected | 0.089 | -0.031 | 0.210 | 0.148 |
| Bacteroides caccae CAG:21 | 0 | Not detected | -0.163 | -0.409 | 0.084 | 0.197 |
| Bacteroides cellulosilyticus | 0 | Not detected | -0.060 | -0.270 | 0.150 | 0.576 |
| Bacteroides coprocola | 0 | Not detected | 0.010 | -0.155 | 0.175 | 0.901 |
| Bacteroides coprophilus | 0 | Not detected | 0.050 | -0.068 | 0.167 | 0.410 |
| Bacteroides coprosuis | 0 | Not detected | 0.159 | -0.013 | 0.331 | 0.070 |
| Bacteroides dorei CAG:222 | 0 | Not detected | 0.011 | -0.182 | 0.205 | 0.909 |
| Bacteroides faecichinchillae | 0 | Not detected | -0.010 | -0.132 | 0.113 | 0.879 |
| Bacteroides faecis CAG:32 | 0 | Not detected | -0.016 | -0.249 | 0.216 | 0.890 |
| Bacteroides finegoldii | 0 | Not detected | 0.033 | -0.187 | 0.252 | 0.772 |
| Bacteroides finegoldii CAG:203 | 0 | Not detected | 0.051 | -0.162 | 0.264 | 0.641 |
| Bacteroides fluxus | 0 | Not detected | 0.086 | -0.042 | 0.214 | 0.189 |
| Bacteroides fragilis | 0 | Not detected | -0.010 | -0.181 | 0.160 | 0.904 |
| Bacteroides fragilis CAG:47 | 0 | Not detected | 0.080 | -0.188 | 0.348 | 0.559 |
| Bacteroides graminisolvens | 0 | Not detected | -0.082 | -0.321 | 0.158 | 0.503 |
| Bacteroides neonati | 0 | Not detected | 0.019 | -0.074 | 0.112 | 0.692 |
| Bacteroides oleiciplenus | 0 | Not detected | -0.064 | -0.210 | 0.083 | 0.394 |
| Bacteroides ovatus | 0 | Not detected | 0.018 | -0.144 | 0.181 | 0.827 |
| Bacteroides ovatus CAG:22 | 0 | Not detected | 0.033 | -0.143 | 0.210 | 0.711 |
| Bacteroides paurosaccharolyticus | 0 | Not detected | -0.009 | -0.174 | 0.156 | 0.916 |
| Bacteroides pectinophilus CAG:437 | 0 | Not detected | -0.003 | -0.149 | 0.143 | 0.969 |
| Bacteroides plebeius | 0 | Not detected | 0.003 | -0.170 | 0.177 | 0.969 |
| Bacteroides propionicifaciens | 0 | Not detected | -0.038 | -0.230 | 0.154 | 0.697 |
| Bacteroides pyogenes | 0 | Not detected | -0.010 | -0.148 | 0.129 | 0.892 |
| Bacteroides reticulotermitis | 0 | Not detected | -0.017 | -0.164 | 0.129 | 0.816 |
| Bacteroides salanitronis | 0 | Not detected | 0.071 | -0.050 | 0.192 | 0.248 |
| Bacteroides salyersiae | 0 | Not detected | -0.092 | -0.259 | 0.075 | 0.278 |
| Bacteroides sp. 41_26 | 0 | Not detected | -0.082 | -0.272 | 0.108 | 0.398 |
| Bacteroides sp. 43_46 | 0 | Not detected | -0.069 | -0.236 | 0.099 | 0.420 |
| Bacteroides sp. CAG:1060 | 0 | Not detected | -0.090 | -0.362 | 0.182 | 0.518 |
| Bacteroides sp. CAG:1076 | 0 | Not detected | 0.167 | -0.031 | 0.366 | 0.099 |
| Bacteroides sp. CAG:530 | 0 | Not detected | 0.189 | 0.008 | 0.370 | 0.041 |
| Bacteroides sp. CAG:545 | 0 | Not detected | 0.165 | -0.200 | 0.530 | 0.375 |
| Bacteroides sp. CAG:714 | 0 | Not detected | -0.052 | -0.322 | 0.217 | 0.703 |

| Bacteroides sp. CAG:770 | 0 | Not detected | 0.039 | -0.169 | 0.248 | 0.712 |
| --- | --- | --- | --- | --- | --- | --- |
| Bacteroides sp. CAG:98 | 0 | Not detected | -0.041 | -0.350 | 0.269 | 0.797 |
| Bacteroides sp. D2 | 0 | Not detected | -0.003 | -0.185 | 0.179 | 0.971 |
| Bacteroides sp. Ga6A1 | 0 | Not detected | -0.104 | -0.343 | 0.135 | 0.393 |
| Bacteroides sp. Ga6A2 | 0 | Not detected | -0.227 | -0.462 | 0.008 | 0.059 |
| Bacteroides stercoris | 0 | Not detected | 0.025 | -0.197 | 0.246 | 0.827 |
| Bacteroides thetaiotaomicron | 0 | Not detected | -0.001 | -0.122 | 0.119 | 0.983 |
| Bacteroides thetaiotaomicron CAG:40 | 0 | Not detected | -0.070 | -0.260 | 0.120 | 0.471 |
| Bacteroides uniformis | 0 | Not detected | -0.054 | -0.233 | 0.125 | 0.554 |
| Bacteroides vulgatus | 0 | Not detected | 0.072 | -0.093 | 0.237 | 0.394 |
| Bacteroides vulgatus CAG:6 | 0 | Not detected | 0.051 | -0.144 | 0.245 | 0.609 |
| Bacteroidetes bacterium OLB8 | 0 | Not detected | 0.006 | -0.185 | 0.197 | 0.953 |
| Bacteroidetes oral taxon 274 | 0 | Not detected | -0.119 | -0.251 | 0.013 | 0.078 |
| Bariatricus massiliensis | 0 | Not detected | -0.010 | -0.097 | 0.077 | 0.821 |
| Barnesiella intestinihominis | 0 | Not detected | -0.031 | -0.243 | 0.182 | 0.778 |
| Barnesiella viscericola | 0 | Not detected | 0.056 | -0.045 | 0.157 | 0.279 |
| Bavariicoccus seileri | 0 | Not detected | -0.004 | -0.185 | 0.176 | 0.961 |
| Beduini massiliensis | 0 | Not detected | -0.078 | -0.205 | 0.049 | 0.230 |
| Bibersteinia trehalosi | 0 | Not detected | -0.008 | -0.223 | 0.206 | 0.939 |
| Bifidobacterium actinocoloniiforme | 0 | Not detected | 0.036 | -0.166 | 0.237 | 0.727 |
| Bifidobacterium adolescentis | 0 | Not detected | -0.126 | -0.485 | 0.233 | 0.492 |
| Bifidobacterium aesculapii | 0 | Not detected | -0.017 | -0.207 | 0.173 | 0.860 |
| Bifidobacterium angulatum | 0 | Not detected | -0.012 | -0.305 | 0.282 | 0.939 |
| Bifidobacterium animalis | 0 | Not detected | 0.032 | -0.207 | 0.271 | 0.791 |
| Bifidobacterium asteroides | 0 | Not detected | -0.022 | -0.234 | 0.190 | 0.837 |
| Bifidobacterium biavatii | 0 | Not detected | 0.031 | -0.069 | 0.132 | 0.542 |
| Bifidobacterium bifidum | 0 | Not detected | 0.066 | -0.268 | 0.399 | 0.700 |
| Bifidobacterium bohemicum | 0 | Not detected | -0.010 | -0.203 | 0.182 | 0.915 |
| Bifidobacterium bombi | 0 | Not detected | 0.086 | -0.102 | 0.275 | 0.371 |
| Bifidobacterium breve | 0 | Not detected | -0.017 | -0.344 | 0.310 | 0.919 |
| Bifidobacterium callitrichos | 0 | Not detected | 0.073 | -0.194 | 0.339 | 0.594 |
| Bifidobacterium choerinum | 0 | Not detected | 0.009 | -0.217 | 0.235 | 0.939 |
| Bifidobacterium coryneforme | 0 | Not detected | 0.026 | -0.176 | 0.227 | 0.802 |
| Bifidobacterium crudilactis | 0 | Not detected | 0.017 | -0.146 | 0.180 | 0.836 |
| Bifidobacterium cuniculi | 0 | Not detected | 0.067 | -0.151 | 0.284 | 0.548 |
| Bifidobacterium dentium | 0 | Not detected | 0.075 | -0.226 | 0.377 | 0.624 |
| Bifidobacterium gallicum | 0 | Not detected | 0.044 | -0.175 | 0.264 | 0.692 |
| Bifidobacterium gallinarum | 0 | Not detected | 0.037 | -0.240 | 0.313 | 0.794 |
| Bifidobacterium longum | 0 | Not detected | -0.033 | -0.364 | 0.299 | 0.848 |
| Bifidobacterium magnum | 0 | Not detected | -0.019 | -0.162 | 0.125 | 0.801 |
| Bifidobacterium minimum | 0 | Not detected | -0.002 | -0.283 | 0.279 | 0.989 |
| Bifidobacterium mongoliense | 0 | Not detected | 0.033 | -0.172 | 0.237 | 0.755 |
| Bifidobacterium pseudolongum | 0 | Not detected | 0.065 | -0.169 | 0.298 | 0.587 |
| Bifidobacterium psychraerophilum | 0 | Not detected | -0.030 | -0.180 | 0.121 | 0.699 |

| Bifidobacterium reuteri | 0 | Not detected | 0.062 | -0.178 | 0.302 | 0.612 |
| --- | --- | --- | --- | --- | --- | --- |
| Bifidobacterium saguini | 0 | Not detected | 0.033 | -0.265 | 0.330 | 0.830 |
| Bifidobacterium scardovii | 0 | Not detected | 0.119 | -0.179 | 0.418 | 0.434 |
| Bifidobacterium stellenboschense | 0 | Not detected | 0.038 | -0.071 | 0.146 | 0.496 |
| Bifidobacterium subtile | 0 | Not detected | 0.040 | -0.207 | 0.287 | 0.750 |
| Bifidobacterium thermophilum | 0 | Not detected | -0.080 | -0.227 | 0.067 | 0.287 |
| Bifidobacterium tsurumiense | 0 | Not detected | 0.038 | -0.180 | 0.256 | 0.735 |
| Bilophila wadsworthia | 0 | Not detected | 0.006 | -0.363 | 0.375 | 0.975 |
| Bittarella massiliensis | 0 | Not detected | -0.015 | -0.107 | 0.078 | 0.754 |
| Blautia hansenii | 0 | Not detected | 0.031 | -0.087 | 0.148 | 0.610 |
| Blautia hydrogenotrophica | 0 | Not detected | -0.005 | -0.098 | 0.088 | 0.917 |
| Blautia hydrogenotrophica CAG:147 | 0 | Not detected | 0.013 | -0.270 | 0.297 | 0.927 |
| Blautia obeum | 0 | Not detected | 0.019 | -0.083 | 0.120 | 0.718 |
| Blautia producta | 0 | Not detected | 0.040 | -0.094 | 0.174 | 0.558 |
| Blautia schinkii | 0 | Not detected | 0.027 | -0.049 | 0.103 | 0.493 |
| Blautia sp. CAG:237 | 0 | Not detected | -0.006 | -0.216 | 0.204 | 0.955 |
| Blautia sp. CAG:37 | 0 | Not detected | 0.010 | -0.188 | 0.209 | 0.919 |
| Blautia sp. CAG:37_48_57 | 0 | Not detected | 0.014 | -0.139 | 0.168 | 0.854 |
| Blautia sp. KLE 1732 | 0 | Not detected | 0.046 | -0.078 | 0.170 | 0.466 |
| Brenneria goodwinii | 0 | Not detected | 0.117 | -0.057 | 0.291 | 0.189 |
| Bulleidia extructa | 0 | Not detected | 0.057 | -0.152 | 0.266 | 0.596 |
| Burkholderiales bacterium GJ-E10 | 0 | Not detected | 0.145 | -0.045 | 0.335 | 0.136 |
| Buttiauxella ferragutiae | 0 | Not detected | 0.101 | -0.091 | 0.292 | 0.303 |
| Butyricicoccus desmolans | 0 | Not detected | -0.024 | -0.105 | 0.056 | 0.556 |
| Butyricicoccus pullicaecorum | 0 | Not detected | -0.101 | -0.205 | 0.003 | 0.057 |
| Butyricicoccus sp. K4410.MGS-46 | 0 | Not detected | 0.144 | 0.010 | 0.278 | 0.036 |
| Butyricimonas sp. Marseille-P2440 | 0 | Not detected | 0.019 | -0.184 | 0.223 | 0.852 |
| Butyricimonas synergistica | 0 | Not detected | -0.139 | -0.393 | 0.115 | 0.284 |
| Butyricimonas virosa | 0 | Not detected | -0.058 | -0.266 | 0.150 | 0.587 |
| Butyrivibrio crossotus | 0 | Not detected | 0.031 | -0.136 | 0.197 | 0.718 |
| Butyrivibrio crossotus CAG:259 | 0 | Not detected | 0.098 | -0.155 | 0.351 | 0.448 |
| Butyrivibrio fibrisolvens | 0 | Not detected | 0.069 | -0.103 | 0.241 | 0.432 |
| Butyrivibrio hungatei | 0 | Not detected | -0.044 | -0.188 | 0.101 | 0.554 |
| Butyrivibrio proteoclasticus | 0 | Not detected | -0.046 | -0.155 | 0.063 | 0.406 |
| Butyrivibrio sp. AD3002 | 0 | Not detected | 0.072 | -0.070 | 0.214 | 0.321 |
| Butyrivibrio sp. AE2005 | 0 | Not detected | -0.066 | -0.209 | 0.077 | 0.366 |
| Butyrivibrio sp. AE3003 | 0 | Not detected | 0.021 | -0.086 | 0.127 | 0.704 |
| Butyrivibrio sp. AE3004 | 0 | Not detected | -0.044 | -0.147 | 0.060 | 0.409 |
| Butyrivibrio sp. AE3009 | 0 | Not detected | 0.023 | -0.129 | 0.175 | 0.766 |
| Butyrivibrio sp. FCS006 | 0 | Not detected | 0.064 | -0.093 | 0.221 | 0.425 |
| Butyrivibrio sp. INlla14 | 0 | Not detected | -0.022 | -0.145 | 0.100 | 0.721 |
| Butyrivibrio sp. INlla16 | 0 | Not detected | -0.003 | -0.139 | 0.133 | 0.964 |
| Butyrivibrio sp. INlla21 | 0 | Not detected | -0.041 | -0.181 | 0.100 | 0.571 |
| Butyrivibrio sp. LB2008 | 0 | Not detected | -0.018 | -0.171 | 0.135 | 0.821 |

| Butyrivibrio sp. M55 | 0 | Not detected | -0.005 | -0.149 | 0.139 | 0.948 |
| --- | --- | --- | --- | --- | --- | --- |
| Butyrivibrio sp. NC2002 | 0 | Not detected | -0.012 | -0.170 | 0.145 | 0.878 |
| Butyrivibrio sp. NC2007 | 0 | Not detected | 0.051 | -0.088 | 0.189 | 0.473 |
| Butyrivibrio sp. NC3005 | 0 | Not detected | -0.072 | -0.280 | 0.137 | 0.501 |
| Butyrivibrio sp. VCD2006 | 0 | Not detected | -0.040 | -0.186 | 0.107 | 0.595 |
| Butyrivibrio sp. WCD2001 | 0 | Not detected | 0.041 | -0.108 | 0.189 | 0.590 |
| Butyrivibrio sp. WCD3002 | 0 | Not detected | -0.022 | -0.156 | 0.112 | 0.744 |
| Butyrivibrio sp. ob235 | 0 | Not detected | 0.085 | -0.079 | 0.250 | 0.310 |
| Campylobacter coli | 0 | Not detected | -0.047 | -0.172 | 0.079 | 0.468 |
| Campylobacter hominis | 0 | Not detected | -0.110 | -0.339 | 0.119 | 0.345 |
| Candidatus Azobacteroides pseudotrichonym | 0 | Not detected | 0.011 | -0.131 | 0.153 | 0.882 |
| Candidatus Gastranaerophilales bacterium M | 0 | Not detected | -0.046 | -0.276 | 0.183 | 0.692 |
| Candidatus Melainabacteria bacterium LEY | 166 | Not detected | -0.156 | -0.309 | -0.003 | 0.046 |
| Candidatus Melainabacteria bacterium MEL | 0 | Not detected | -0.016 | -0.303 | 0.272 | 0.916 |
| Candidatus Saccharibacteria oral taxon TM7 | 0 | Not detected | 0.057 | -0.106 | 0.220 | 0.490 |
| Candidatus Soleaferrea massiliensis | 0 | Not detected | -0.057 | -0.192 | 0.077 | 0.405 |
| Candidatus Stoquefichus sp. SB1 | 0 | Not detected | 0.029 | -0.095 | 0.153 | 0.649 |
| Capnocytophaga granulosa | 0 | Not detected | -0.069 | -0.244 | 0.106 | 0.440 |
| Capnocytophaga sp. oral taxon 329 | 0 | Not detected | -0.006 | -0.265 | 0.252 | 0.961 |
| Capnocytophaga sp. oral taxon 863 | 0 | Not detected | -0.202 | -0.422 | 0.018 | 0.072 |
| Capnocytophaga sputigena | 0 | Not detected | -0.078 | -0.214 | 0.059 | 0.266 |
| Carnobacterium gallinarum | 0 | Not detected | -0.059 | -0.270 | 0.152 | 0.585 |
| Catabacter hongkongensis | 367 | Not detected | -0.247 | -0.430 | -0.065 | 0.008 |
| Catenibacterium mitsuokai | 0 | Not detected | 0.118 | -0.212 | 0.447 | 0.483 |
| Catenibacterium sp. CAG:290 | 0 | Not detected | 0.381 | -0.016 | 0.779 | 0.061 |
| Catonella morbi | 0 | Not detected | 0.006 | -0.109 | 0.120 | 0.921 |
| Cedecea neteri | 0 | Not detected | 0.185 | -0.056 | 0.426 | 0.133 |
| Cellulomonas carbonis | 0 | Not detected | 0.071 | -0.161 | 0.303 | 0.550 |
| Cellulomonas flavigena | 0 | Not detected | 0.048 | -0.182 | 0.279 | 0.680 |
| Cellulosilyticum lentocellum | 0 | Not detected | -0.121 | -0.277 | 0.034 | 0.126 |
| Cellulosilyticum ruminicola | 0 | Not detected | -0.060 | -0.223 | 0.103 | 0.470 |
| Chlamydia psittaci | 0 | Not detected | 0.132 | -0.053 | 0.317 | 0.163 |
| Christensenella sp. AF73-05CM02 | 0 | Not detected | -0.144 | -0.358 | 0.071 | 0.189 |
| Christensenellaceae bacterium Phil1 | 0 | Not detected | -0.111 | -0.286 | 0.064 | 0.216 |
| Christensenellaceae bacterium Phil10 | 0 | Not detected | -0.192 | -0.442 | 0.059 | 0.134 |
| Christensenellaceae bacterium Phil7 | 0 | Not detected | -0.231 | -0.483 | 0.022 | 0.074 |
| Chryseobacterium gallinarum | 0 | Not detected | 0.023 | -0.248 | 0.295 | 0.866 |
| Chryseobacterium greenlandense | 0 | Not detected | -0.178 | -0.434 | 0.079 | 0.176 |
| Citrobacter amalonaticus | 0 | Not detected | 0.198 | -0.112 | 0.507 | 0.211 |
| Citrobacter freundii | 0 | Not detected | 0.127 | -0.195 | 0.450 | 0.438 |
| Cloacibacillus evryensis | 0 | Not detected | -0.144 | -0.441 | 0.153 | 0.341 |
| Cloacibacillus porcorum | 653 | Not detected | -0.297 | -0.553 | -0.041 | 0.023 |
| Cloacibacterium normanense | 0 | Not detected | -0.287 | -0.530 | -0.044 | 0.021 |
| Clostridia bacterium UC5.1-1C12 | 0 | Not detected | -0.160 | -0.438 | 0.118 | 0.260 |

| Clostridia bacterium UC5.1-1D1 | 0 | Not detected | -0.038 | -0.137 | 0.061 | 0.453 |
| --- | --- | --- | --- | --- | --- | --- |
| Clostridia bacterium UC5.1-1E11 | 0 | Not detected | 0.001 | -0.097 | 0.099 | 0.984 |
| Clostridiales bacterium 1_7_47FAA | 0 | Not detected | -0.057 | -0.163 | 0.048 | 0.288 |
| Clostridiales bacterium 59_14 | 0 | Not detected | 0.065 | -0.102 | 0.232 | 0.446 |
| Clostridiales bacterium DTU035 | 0 | Not detected | 0.079 | -0.083 | 0.241 | 0.339 |
| Clostridiales bacterium DTU036 | 0 | Not detected | -0.016 | -0.173 | 0.141 | 0.844 |
| Clostridiales bacterium DTU089 | 0 | Not detected | -0.087 | -0.247 | 0.074 | 0.290 |
| Clostridiales bacterium Firm_03 | 0 | Not detected | -0.226 | -0.440 | -0.011 | 0.040 |
| Clostridiales bacterium Firm_16 | 0 | Not detected | -0.114 | -0.338 | 0.110 | 0.320 |
| Clostridiales bacterium NK3B98 | 0 | Not detected | 0.078 | -0.112 | 0.267 | 0.423 |
| Clostridiales bacterium S5-A14a | 0 | Not detected | -0.056 | -0.150 | 0.038 | 0.244 |
| Clostridiales bacterium SIT11 | 0 | Not detected | -0.003 | -0.189 | 0.182 | 0.971 |
| Clostridiales bacterium VE202-08 | 0 | Not detected | 0.050 | -0.059 | 0.160 | 0.367 |
| Clostridioides difficile | 0 | Not detected | -0.036 | -0.128 | 0.057 | 0.450 |
| Clostridioides mangenotii | 0 | Not detected | -0.001 | -0.172 | 0.171 | 0.995 |
| Clostridium baratii | 0 | Not detected | 0.138 | -0.075 | 0.350 | 0.205 |
| Clostridium beijerinckii | 0 | Not detected | 0.046 | -0.140 | 0.232 | 0.628 |
| Clostridium bolteae CAG:59 | 0 | Not detected | 0.072 | -0.104 | 0.248 | 0.424 |
| Clostridium botulinum | 0 | Not detected | -0.031 | -0.235 | 0.172 | 0.762 |
| Clostridium butyricum | 0 | Not detected | 0.066 | -0.138 | 0.270 | 0.524 |
| Clostridium cadaveris | 0 | Not detected | -0.062 | -0.241 | 0.117 | 0.496 |
| Clostridium celatum | 0 | Not detected | 0.111 | -0.202 | 0.424 | 0.487 |
| Clostridium cellulovorans | 0 | Not detected | -0.065 | -0.189 | 0.060 | 0.310 |
| Clostridium clostridioforme CAG:132 | 0 | Not detected | -0.073 | -0.314 | 0.169 | 0.554 |
| Clostridium clostridioforme CAG:511 | 0 | Not detected | -0.110 | -0.329 | 0.109 | 0.324 |
| Clostridium colicanis | 0 | Not detected | 0.025 | -0.130 | 0.179 | 0.755 |
| Clostridium disporicum | 0 | Not detected | 0.082 | -0.079 | 0.243 | 0.316 |
| Clostridium ihumii | 0 | Not detected | -0.028 | -0.205 | 0.149 | 0.754 |
| Clostridium intestinale | 0 | Not detected | 0.054 | -0.086 | 0.194 | 0.446 |
| Clostridium kluyveri | 0 | Not detected | 0.148 | -0.058 | 0.354 | 0.160 |
| Clostridium leptum CAG:27 | 0 | Not detected | 0.105 | -0.166 | 0.375 | 0.449 |
| Clostridium lundense | 35 | Not detected | 0.220 | 0.011 | 0.430 | 0.040 |
| Clostridium neonatale | 0 | Not detected | -0.060 | -0.239 | 0.118 | 0.509 |
| Clostridium nexile CAG:348 | 0 | Not detected | -0.026 | -0.201 | 0.149 | 0.772 |
| Clostridium paraputrificum | 0 | Not detected | -0.092 | -0.353 | 0.170 | 0.493 |
| Clostridium perfringens | 0 | Not detected | 0.093 | -0.234 | 0.420 | 0.577 |
| Clostridium phoceensis | 0 | Not detected | -0.008 | -0.153 | 0.136 | 0.909 |
| Clostridium polynesiense | 0 | Not detected | -0.192 | -0.398 | 0.014 | 0.068 |
| Clostridium saccharobutylicum | 0 | Not detected | 0.028 | -0.143 | 0.199 | 0.746 |
| Clostridium saccharoperbutylacetonicum | 0 | Not detected | 0.049 | -0.116 | 0.214 | 0.563 |
| Clostridium sartagoforme | 0 | Not detected | 0.103 | -0.110 | 0.315 | 0.343 |
| Clostridium sp. 44_14 | 0 | Not detected | 0.047 | -0.158 | 0.253 | 0.653 |
| Clostridium sp. ASF356 | 0 | Not detected | 0.093 | -0.115 | 0.301 | 0.382 |
| Clostridium sp. Ade.TY | 0 | Not detected | 0.114 | -0.064 | 0.292 | 0.210 |

| Clostridium sp. CAG:1193 | 0 | Not detected | -0.072 | -0.292 | 0.147 | 0.518 |
| --- | --- | --- | --- | --- | --- | --- |
| Clostridium sp. CAG:122 | 0 | Not detected | -0.061 | -0.318 | 0.195 | 0.639 |
| Clostridium sp. CAG:12237_41 | 0 | Not detected | 0.012 | -0.193 | 0.216 | 0.911 |
| Clostridium sp. CAG:127 | 0 | Not detected | 0.082 | -0.225 | 0.390 | 0.600 |
| Clostridium sp. CAG:138 | 0 | Not detected | 0.036 | -0.370 | 0.443 | 0.861 |
| Clostridium sp. CAG:167 | 0 | Not detected | 0.210 | -0.128 | 0.548 | 0.224 |
| Clostridium sp. CAG:226 | 0 | Not detected | -0.341 | -0.653 | -0.029 | 0.032 |
| Clostridium sp. CAG:230 | 0 | Not detected | 0.047 | -0.163 | 0.257 | 0.661 |
| Clostridium sp. CAG:242 | 0 | Not detected | -0.185 | -0.472 | 0.102 | 0.207 |
| Clostridium sp. CAG:245 | 0 | Not detected | -0.081 | -0.518 | 0.356 | 0.717 |
| Clostridium sp. CAG:245_30_32 | 0 | Not detected | 0.056 | -0.136 | 0.247 | 0.568 |
| Clostridium sp. CAG:264 | 0 | Not detected | -0.074 | -0.375 | 0.227 | 0.630 |
| Clostridium sp. CAG:277 | 0 | Not detected | -0.052 | -0.258 | 0.153 | 0.617 |
| Clostridium sp. CAG:288 | 0 | Not detected | -0.191 | -0.473 | 0.090 | 0.184 |
| Clostridium sp. CAG:299 | 0 | Not detected | 0.040 | -0.242 | 0.322 | 0.782 |
| Clostridium sp. CAG:307_30_263 | 0 | Not detected | 0.046 | -0.135 | 0.227 | 0.622 |
| Clostridium sp. CAG:349 | 0 | Not detected | -0.157 | -0.550 | 0.237 | 0.436 |
| Clostridium sp. CAG:352 | 0 | Not detected | -0.188 | -0.470 | 0.093 | 0.190 |
| Clostridium sp. CAG:413 | 0 | Not detected | -0.120 | -0.436 | 0.196 | 0.457 |
| Clostridium sp. CAG:448 | 0 | Not detected | -0.047 | -0.355 | 0.261 | 0.765 |
| Clostridium sp. CAG:451 | 0 | Not detected | -0.044 | -0.362 | 0.273 | 0.784 |
| Clostridium sp. CAG:465 | 0 | Not detected | -0.242 | -0.545 | 0.061 | 0.118 |
| Clostridium sp. CAG:505 | 0 | Not detected | -0.043 | -0.363 | 0.278 | 0.794 |
| Clostridium sp. CAG:568 | 0 | Not detected | 0.061 | -0.227 | 0.348 | 0.679 |
| Clostridium sp. CAG:58 | 0 | Not detected | -0.054 | -0.284 | 0.177 | 0.649 |
| Clostridium sp. CAG:590 | 22 | Not detected | 0.146 | -0.007 | 0.298 | 0.061 |
| Clostridium sp. CAG:594 | 0 | Not detected | 0.062 | -0.263 | 0.387 | 0.707 |
| Clostridium sp. CAG:628 | 0 | Not detected | -0.408 | -0.720 | -0.096 | 0.011 |
| Clostridium sp. CAG:62_40_43 | 0 | Not detected | -0.104 | -0.473 | 0.265 | 0.580 |
| Clostridium sp. CAG:632 | 0 | Not detected | 0.120 | -0.088 | 0.327 | 0.259 |
| Clostridium sp. CAG:678 | 0 | Not detected | -0.076 | -0.324 | 0.172 | 0.549 |
| Clostridium sp. CAG:762 | 0 | Not detected | 0.043 | -0.210 | 0.295 | 0.741 |
| Clostridium sp. CAG:768 | 0 | Not detected | -0.018 | -0.188 | 0.152 | 0.835 |
| Clostridium sp. CAG:780 | 0 | Not detected | -0.146 | -0.492 | 0.201 | 0.410 |
| Clostridium sp. CAG:793 | 0 | Not detected | -0.085 | -0.357 | 0.188 | 0.542 |
| Clostridium sp. CAG:81 | 0 | Not detected | 0.135 | -0.035 | 0.305 | 0.121 |
| Clostridium sp. CAG:91 | 0 | Not detected | -0.146 | -0.422 | 0.131 | 0.302 |
| Clostridium sp. CAG:964 | 0 | Not detected | -0.047 | -0.213 | 0.119 | 0.580 |
| Clostridium sp. CL-6 | 0 | Not detected | -0.101 | -0.243 | 0.041 | 0.163 |
| Clostridium sp. HGF2 | 0 | Not detected | 0.023 | -0.071 | 0.117 | 0.634 |
| Clostridium sp. K4410.MGS-306 | 0 | Not detected | 0.062 | -0.066 | 0.190 | 0.341 |
| Clostridium sp. KLE 1755 | 0 | Not detected | -0.033 | -0.174 | 0.108 | 0.649 |
| Clostridium sp. KNHs205 | 0 | Not detected | -0.005 | -0.086 | 0.075 | 0.897 |
| Clostridium sp. KNHs209 | 0 | Not detected | 0.011 | -0.068 | 0.090 | 0.778 |

| Clostridium sp. KNHs214 | 0 | Not detected | -0.032 | -0.175 | 0.111 | 0.660 |
| --- | --- | --- | --- | --- | --- | --- |
| Clostridium sp. L2-50 | 0 | Not detected | -0.025 | -0.146 | 0.096 | 0.688 |
| Clostridium sp. M62/1 | 0 | Not detected | 0.012 | -0.072 | 0.097 | 0.775 |
| Clostridium sp. ND2 | 0 | Not detected | -0.026 | -0.197 | 0.145 | 0.766 |
| Clostridium sp. SY8519 | 0 | Not detected | -0.056 | -0.145 | 0.033 | 0.221 |
| Clostridium sulfidigenes | 0 | Not detected | -0.018 | -0.161 | 0.126 | 0.811 |
| Collinsella aerofaciens | 0 | Not detected | 0.039 | -0.208 | 0.287 | 0.756 |
| Collinsella intestinalis | 0 | Not detected | -0.150 | -0.410 | 0.111 | 0.260 |
| Collinsella sp. CAG:166 | 0 | Not detected | 0.032 | -0.237 | 0.302 | 0.814 |
| Collinsella sp. MS5 | 0 | Not detected | -0.159 | -0.342 | 0.025 | 0.090 |
| Collinsella stercoris | 0 | Not detected | -0.090 | -0.325 | 0.146 | 0.455 |
| Collinsella tanakaei | 0 | Not detected | -0.094 | -0.270 | 0.082 | 0.296 |
| Comamonas kerstersii | 0 | Not detected | -0.234 | -0.486 | 0.019 | 0.071 |
| Coprobacillus sp. CAG:605 | 0 | Not detected | -0.271 | -0.563 | 0.021 | 0.070 |
| Coprobacillus sp. D6 | 0 | Not detected | 0.004 | -0.117 | 0.124 | 0.949 |
| Coprobacter secundus | 0 | Not detected | 0.071 | -0.038 | 0.180 | 0.200 |
| Coprococcus comes | 0 | Not detected | 0.046 | -0.056 | 0.147 | 0.377 |
| Coprococcus eutactus | 0 | Not detected | -0.007 | -0.142 | 0.128 | 0.920 |
| Coprococcus eutactus CAG:665 | 0 | Not detected | -0.007 | -0.298 | 0.284 | 0.962 |
| Coprococcus sp. 43_8 | 0 | Not detected | 0.076 | -0.117 | 0.269 | 0.440 |
| Coprococcus sp. CAG:131 | 0 | Not detected | -0.037 | -0.320 | 0.246 | 0.798 |
| Coprococcus sp. CAG:131_42_139 | 0 | Not detected | 0.021 | -0.262 | 0.305 | 0.882 |
| Coprococcus sp. HPP0048 | 0 | Not detected | -0.003 | -0.164 | 0.157 | 0.969 |
| Coriobacteriaceae bacterium 68-1-3 | 0 | Not detected | -0.098 | -0.276 | 0.080 | 0.281 |
| Coriobacteriaceae bacterium BV3Ac1 | 57 | Not detected | -0.277 | -0.496 | -0.058 | 0.013 |
| Coriobacteriales bacterium DNF00809 | 0 | Not detected | -0.153 | -0.341 | 0.036 | 0.113 |
| Coriobacterium glomerans | 1 | Not detected | -0.103 | -0.280 | 0.073 | 0.253 |
| Corynebacterium amycolatum | 0 | Not detected | 0.006 | -0.135 | 0.147 | 0.934 |
| Corynebacterium durum | 0 | Not detected | 0.184 | -0.025 | 0.393 | 0.085 |
| Corynebacterium glucuronolyticum | 0 | Not detected | -0.053 | -0.221 | 0.115 | 0.534 |
| Corynebacterium singulare | 0 | Not detected | -0.017 | -0.180 | 0.146 | 0.840 |
| Corynebacterium urealyticum | 0 | Not detected | -0.009 | -0.190 | 0.173 | 0.925 |
| Criibacterium bergeronii | 0 | Not detected | -0.087 | -0.224 | 0.049 | 0.209 |
| Cronobacter sakazakii | 0 | Not detected | 0.227 | -0.076 | 0.531 | 0.142 |
| Cryptobacterium sp. CAG:338 | 0 | Not detected | -0.042 | -0.356 | 0.271 | 0.791 |
| Culturomica massiliensis | 0 | Not detected | 0.037 | -0.088 | 0.162 | 0.560 |
| Cutibacterium acnes | 0 | Not detected | 0.088 | -0.112 | 0.289 | 0.389 |
| Defluviitalea phaphyphila | 18 | Not detected | 0.140 | 0.001 | 0.280 | 0.049 |
| Dehalobacter restrictus | 0 | Not detected | -0.004 | -0.146 | 0.137 | 0.954 |
| Dendrosporobacter quercicolus | 0 | Not detected | 0.027 | -0.115 | 0.170 | 0.708 |
| Denitrobacterium detoxificans | 0 | Not detected | -0.101 | -0.257 | 0.054 | 0.201 |
| Desnuesiella massiliensis | 0 | Not detected | 0.050 | -0.096 | 0.196 | 0.502 |
| Desulfovibrio alaskensis | 0 | Not detected | -0.070 | -0.219 | 0.078 | 0.355 |
| Desulfovibrio desulfuricans | 0 | Not detected | -0.197 | -0.460 | 0.067 | 0.145 |

| Desulfovibrio fairfieldensis | 0 | Not detected | -0.303 | -0.611 | 0.005 | 0.054 |
| --- | --- | --- | --- | --- | --- | --- |
| Desulfovibrio piger | 0 | Not detected | -0.180 | -0.570 | 0.209 | 0.364 |
| Desulfovibrio vulgaris | 0 | Not detected | -0.118 | -0.287 | 0.051 | 0.172 |
| Devosia insulae | 0 | Not detected | -0.004 | -0.123 | 0.115 | 0.948 |
| Dialister invisus | 0 | Not detected | -0.175 | -0.621 | 0.270 | 0.441 |
| Dialister invisus CAG:218 | 0 | Not detected | -0.161 | -0.614 | 0.293 | 0.488 |
| Dialister micraerophilus | 0 | Not detected | -0.193 | -0.445 | 0.058 | 0.132 |
| Dialister sp. CAG:357 | 0 | Not detected | -0.229 | -0.647 | 0.190 | 0.284 |
| Dialister sp. CAG:486 | 0 | Not detected | -0.190 | -0.585 | 0.204 | 0.345 |
| Dialister sp. CAG:588 | 4 | Not detected | -0.301 | -0.515 | -0.086 | 0.006 |
| Dialister succinatiphilus | 737 | 0.6 | -0.358 | -0.608 | -0.108 | 0.005 |
| Dickeya dadantii | 0 | Not detected | 0.128 | -0.043 | 0.299 | 0.142 |
| Dielma fastidiosa | 0 | Not detected | -0.094 | -0.317 | 0.129 | 0.408 |
| Dorea formicigenerans | 0 | Not detected | 0.017 | -0.078 | 0.112 | 0.728 |
| Dorea longicatena | 0 | Not detected | -0.003 | -0.133 | 0.126 | 0.959 |
| Dorea sp. 42_8 | 0 | Not detected | -0.070 | -0.230 | 0.090 | 0.390 |
| Dorea sp. 5-2 | 0 | Not detected | 0.038 | -0.032 | 0.108 | 0.291 |
| Dorea sp. CAG:105 | 0 | Not detected | 0.072 | -0.129 | 0.273 | 0.481 |
| Draconibacterium orientale | 0 | Not detected | -0.094 | -0.229 | 0.041 | 0.175 |
| Drancourtella massiliensis | 0 | Not detected | 0.054 | -0.114 | 0.223 | 0.528 |
| Dubosiella newyorkensis | 0 | Not detected | -0.033 | -0.179 | 0.114 | 0.663 |
| Dysgonomonas gadei | 0 | Not detected | -0.040 | -0.167 | 0.087 | 0.537 |
| Dysgonomonas mossii | 0 | Not detected | -0.149 | -0.304 | 0.006 | 0.059 |
| Dysgonomonas sp. BGC7 | 0 | Not detected | -0.136 | -0.330 | 0.059 | 0.172 |
| Dysgonomonas sp. HGC4 | 0 | Not detected | -0.017 | -0.178 | 0.144 | 0.839 |
| Edwardsiella ictaluri | 0 | Not detected | 0.126 | -0.051 | 0.303 | 0.163 |
| Eggerthella lenta | 0 | Not detected | -0.017 | -0.255 | 0.221 | 0.886 |
| Eggerthella sp. 51_9 | 0 | Not detected | 0.135 | -0.115 | 0.386 | 0.290 |
| Eggerthella sp. CAG:209 | 0 | Not detected | 0.259 | -0.108 | 0.626 | 0.167 |
| Eggerthella sp. CAG:368 | 0 | Not detected | 0.148 | -0.104 | 0.400 | 0.251 |
| Eggerthella sp. YY7918 | 0 | Not detected | -0.181 | -0.365 | 0.002 | 0.053 |
| Eggerthellaceae bacterium AT8 | 0 | Not detected | -0.082 | -0.198 | 0.035 | 0.170 |
| Eggerthia catenaformis | 4 | Not detected | 0.147 | -0.073 | 0.368 | 0.190 |
| Eisenbergiella tayi | 1 | Not detected | -0.185 | -0.376 | 0.006 | 0.058 |
| Elizabethkingia anophelis | 0 | Not detected | -0.230 | -0.457 | -0.003 | 0.047 |
| Emergencia timonensis | 0 | Not detected | -0.053 | -0.143 | 0.037 | 0.248 |
| Empedobacter brevis | 0 | Not detected | -0.163 | -0.467 | 0.142 | 0.296 |
| Enhygromyxa salina | 0 | Not detected | 0.082 | -0.110 | 0.275 | 0.401 |
| Enorma massiliensis | 500 | Not detected | -0.165 | -0.291 | -0.038 | 0.011 |
| Enorma timonensis | 133 | Not detected | -0.132 | -0.278 | 0.014 | 0.077 |
| Enterobacter cloacae | 0 | Not detected | 0.272 | -0.051 | 0.594 | 0.099 |
| Enterobacter lignolyticus | 0 | Not detected | 0.263 | -0.024 | 0.549 | 0.073 |
| Enterobacter sp. 638 | 0 | Not detected | 0.220 | -0.043 | 0.483 | 0.102 |
| Enterobacteriaceae bacterium LSJC7 | 0 | Not detected | 0.179 | -0.014 | 0.372 | 0.070 |

| Enterococcus avium | 0 | Not detected | -0.018 | -0.206 | 0.170 | 0.851 |
| --- | --- | --- | --- | --- | --- | --- |
| Enterococcus cecorum | 0 | Not detected | 0.110 | -0.065 | 0.286 | 0.218 |
| Enterococcus columbae | 0 | Not detected | 0.009 | -0.204 | 0.223 | 0.932 |
| Enterococcus durans | 0 | Not detected | -0.058 | -0.302 | 0.186 | 0.642 |
| Enterococcus faecalis | 0 | Not detected | 0.009 | -0.179 | 0.198 | 0.925 |
| Enterococcus faecium | 0 | Not detected | -0.025 | -0.240 | 0.189 | 0.816 |
| Enterococcus gallinarum | 0 | Not detected | -0.108 | -0.304 | 0.089 | 0.283 |
| Enterococcus gilvus | 1 | Not detected | -0.221 | -0.404 | -0.039 | 0.017 |
| Enterococcus italicus | 0 | Not detected | -0.206 | -0.424 | 0.011 | 0.063 |
| Enterococcus massiliensis | 0 | Not detected | -0.053 | -0.262 | 0.155 | 0.617 |
| Enterococcus sp. HMSC064A12 | 0 | Not detected | -0.037 | -0.250 | 0.176 | 0.734 |
| Enterorhabdus caecimuris | 0 | Not detected | 0.045 | -0.167 | 0.258 | 0.676 |
| Enterorhabdus mucosicola | 0 | Not detected | 0.082 | -0.112 | 0.275 | 0.409 |
| Epulopiscium sp. 'N.t. morphotype B' | 0 | Not detected | -0.062 | -0.190 | 0.065 | 0.339 |
| Erwinia billingiae | 0 | Not detected | 0.143 | -0.030 | 0.315 | 0.105 |
| Erwinia oleae | 0 | Not detected | 0.119 | -0.115 | 0.352 | 0.319 |
| Erysipelatoclostridium ramosum | 0 | Not detected | 0.039 | -0.236 | 0.315 | 0.780 |
| Erysipelotrichaceae bacterium 21_3 | 0 | Not detected | -0.067 | -0.186 | 0.053 | 0.273 |
| Erysipelotrichaceae bacterium 5_2_54FAA | 0 | Not detected | -0.015 | -0.116 | 0.086 | 0.770 |
| Erysipelotrichaceae bacterium CAG:64 | 0 | Not detected | -0.001 | -0.206 | 0.204 | 0.993 |
| Erysipelotrichaceae bacterium I46 | 0 | Not detected | -0.028 | -0.141 | 0.086 | 0.633 |
| Escherichia coli | 0 | Not detected | 0.106 | -0.214 | 0.425 | 0.518 |
| Escherichia vulneris | 0 | Not detected | 0.172 | -0.084 | 0.429 | 0.188 |
| Ethanoligenens harbinense | 0 | Not detected | -0.162 | -0.358 | 0.033 | 0.104 |
| Eubacterium dolichum CAG:375 | 0 | Not detected | -0.120 | -0.430 | 0.191 | 0.450 |
| Eubacterium limosum | 0 | Not detected | -0.076 | -0.224 | 0.071 | 0.311 |
| Eubacterium plexicaudatum | 0 | Not detected | 0.009 | -0.073 | 0.092 | 0.824 |
| Eubacterium pyruvativorans | 0 | Not detected | -0.061 | -0.162 | 0.041 | 0.242 |
| Eubacterium ramulus | 0 | Not detected | 0.016 | -0.079 | 0.110 | 0.747 |
| Eubacterium sp. 14-2 | 0 | Not detected | 0.028 | -0.056 | 0.112 | 0.511 |
| Eubacterium sp. 3_1_31 | 0 | Not detected | -0.035 | -0.146 | 0.077 | 0.543 |
| Eubacterium sp. 45_250 | 0 | Not detected | -0.213 | -0.541 | 0.116 | 0.204 |
| Eubacterium sp. AB3007 | 0 | Not detected | -0.136 | -0.400 | 0.129 | 0.314 |
| Eubacterium sp. CAG:115 | 0 | Not detected | 0.169 | -0.271 | 0.609 | 0.452 |
| Eubacterium sp. CAG:146 | 0 | Not detected | -0.067 | -0.277 | 0.144 | 0.535 |
| Eubacterium sp. CAG:180 | 0 | Not detected | -0.171 | -0.589 | 0.247 | 0.422 |
| Eubacterium sp. CAG:192 | 0 | Not detected | -0.261 | -0.555 | 0.032 | 0.081 |
| Eubacterium sp. CAG:202 | 0 | Not detected | -0.167 | -0.469 | 0.134 | 0.277 |
| Eubacterium sp. CAG:248 | 0 | Not detected | 0.093 | -0.169 | 0.355 | 0.487 |
| Eubacterium sp. CAG:251 | 0 | Not detected | -0.175 | -0.528 | 0.178 | 0.332 |
| Eubacterium sp. CAG:252 | 0 | Not detected | 0.177 | -0.052 | 0.405 | 0.130 |
| Eubacterium sp. CAG:581 | 0 | Not detected | -0.410 | -0.701 | -0.119 | 0.006 |
| Eubacterium sp. CAG:603 | 0 | Not detected | -0.103 | -0.263 | 0.057 | 0.209 |
| Eubacterium sp. CAG:786 | 0 | Not detected | 0.159 | -0.172 | 0.491 | 0.346 |

| Eubacterium sp. CAG:841 | 0 | Not detected | 0.010 | -0.315 | 0.335 | 0.952 |
| --- | --- | --- | --- | --- | --- | --- |
| Eubacterium sp. ER2 | 0 | Not detected | -0.067 | -0.149 | 0.015 | 0.110 |
| Eubacterium ventriosum | 0 | Not detected | -0.076 | -0.196 | 0.044 | 0.217 |
| Eubacterium xylanophilum | 0 | Not detected | -0.086 | -0.250 | 0.079 | 0.307 |
| Facklamia hominis | 0 | Not detected | -0.011 | -0.207 | 0.185 | 0.909 |
| Facklamia ignava | 0 | Not detected | -0.001 | -0.194 | 0.192 | 0.993 |
| Facklamia languida | 20 | Not detected | 0.127 | -0.050 | 0.304 | 0.161 |
| Faecalibacterium prausnitzii | 0 | Not detected | -0.018 | -0.171 | 0.134 | 0.816 |
| Faecalibacterium sp. CAG:1138 | 0 | Not detected | -0.044 | -0.314 | 0.227 | 0.752 |
| Faecalibacterium sp. CAG:82-related_59_9 | 0 | Not detected | 0.178 | -0.049 | 0.404 | 0.124 |
| Faecalibaculum rodentium | 0 | Not detected | 0.145 | -0.064 | 0.354 | 0.175 |
| Faecalicoccus pleomorphus | 0 | Not detected | -0.029 | -0.148 | 0.090 | 0.634 |
| Faecalitalea cylindroides | 0 | Not detected | 0.022 | -0.060 | 0.104 | 0.595 |
| Fenollaria massiliensis | 0 | Not detected | -0.104 | -0.334 | 0.125 | 0.374 |
| Fermentimonas caenicola | 0 | Not detected | 0.109 | -0.086 | 0.304 | 0.274 |
| Fibrobacter sp. UWB12 | 0 | Not detected | 0.059 | -0.071 | 0.189 | 0.373 |
| Fibrobacter sp. UWCM | 0 | Not detected | 0.094 | -0.083 | 0.271 | 0.297 |
| Fibrobacter sp. UWOV1 | 0 | Not detected | -0.085 | -0.207 | 0.037 | 0.171 |
| Fibrobacterales bacterium Fibro_02 | 0 | Not detected | -0.036 | -0.163 | 0.090 | 0.574 |
| Fibrobacteria bacterium AD80 | 0 | Not detected | -0.093 | -0.222 | 0.035 | 0.156 |
| Filifactor alocis | 0 | Not detected | -0.005 | -0.210 | 0.200 | 0.961 |
| Finegoldia magna | 0 | Not detected | -0.133 | -0.344 | 0.078 | 0.217 |
| Firmicutes bacterium ASF500 | 0 | Not detected | -0.005 | -0.093 | 0.083 | 0.915 |
| Firmicutes bacterium CAG:102 | 0 | Not detected | 0.063 | -0.242 | 0.368 | 0.686 |
| Firmicutes bacterium CAG:137 | 0 | Not detected | -0.177 | -0.514 | 0.160 | 0.304 |
| Firmicutes bacterium CAG:137_57_8 | 0 | Not detected | -0.175 | -0.434 | 0.085 | 0.188 |
| Firmicutes bacterium CAG:145 | 0 | Not detected | -0.158 | -0.460 | 0.144 | 0.306 |
| Firmicutes bacterium CAG:176 | 0 | Not detected | 0.030 | -0.355 | 0.414 | 0.880 |
| Firmicutes bacterium CAG:176_59_8 | 0 | Not detected | -0.015 | -0.355 | 0.326 | 0.933 |
| Firmicutes bacterium CAG:227 | 0 | Not detected | 0.023 | -0.237 | 0.283 | 0.863 |
| Firmicutes bacterium CAG:238 | 0 | Not detected | -0.155 | -0.444 | 0.133 | 0.292 |
| Firmicutes bacterium CAG:24 | 0 | Not detected | 0.068 | -0.153 | 0.288 | 0.547 |
| Firmicutes bacterium CAG:240 | 0 | Not detected | 0.035 | -0.302 | 0.372 | 0.840 |
| Firmicutes bacterium CAG:270 | 0 | Not detected | -0.082 | -0.333 | 0.169 | 0.520 |
| Firmicutes bacterium CAG:272 | 0 | Not detected | -0.250 | -0.549 | 0.048 | 0.100 |
| Firmicutes bacterium CAG:272_52_7 | 0 | Not detected | 0.071 | -0.155 | 0.297 | 0.537 |
| Firmicutes bacterium CAG:308 | 0 | Not detected | -0.017 | -0.216 | 0.183 | 0.871 |
| Firmicutes bacterium CAG:313 | 0 | Not detected | -0.064 | -0.457 | 0.329 | 0.750 |
| Firmicutes bacterium CAG:41 | 0 | Not detected | 0.099 | -0.175 | 0.373 | 0.477 |
| Firmicutes bacterium CAG:466 | 0 | Not detected | -0.013 | -0.244 | 0.218 | 0.912 |
| Firmicutes bacterium CAG:475 | 0 | Not detected | -0.077 | -0.370 | 0.215 | 0.604 |
| Firmicutes bacterium CAG:536 | 0 | Not detected | -0.026 | -0.266 | 0.214 | 0.833 |
| Firmicutes bacterium CAG:552 | 0 | Not detected | -0.063 | -0.318 | 0.193 | 0.632 |
| Firmicutes bacterium CAG:555 | 0 | Not detected | -0.157 | -0.417 | 0.104 | 0.239 |

| Firmicutes bacterium CAG:65 | 0 | Not detected | -0.099 | -0.350 | 0.151 | 0.438 |
| --- | --- | --- | --- | --- | --- | --- |
| Firmicutes bacterium CAG:791 | 0 | Not detected | 0.016 | -0.126 | 0.158 | 0.826 |
| Firmicutes bacterium CAG:83 | 0 | Not detected | -0.062 | -0.335 | 0.211 | 0.658 |
| Firmicutes bacterium CAG:882 | 0 | Not detected | 0.058 | -0.127 | 0.242 | 0.541 |
| Firmicutes bacterium CAG:884 | 123 | Not detected | -0.356 | -0.611 | -0.100 | 0.006 |
| Firmicutes bacterium CAG:94 | 0 | Not detected | -0.219 | -0.511 | 0.074 | 0.144 |
| Firmicutes bacterium M10-2 | 0 | Not detected | 0.068 | -0.151 | 0.287 | 0.544 |
| Firmicutes bacterium ZOR0006 | 40 | Not detected | 0.182 | -0.047 | 0.411 | 0.120 |
| Flavonifractor plautii | 0 | Not detected | 0.005 | -0.188 | 0.197 | 0.962 |
| Fournierella massiliensis | 0 | Not detected | -0.052 | -0.144 | 0.040 | 0.271 |
| Franconibacter pulveris | 0 | Not detected | 0.219 | -0.021 | 0.458 | 0.074 |
| Fretibacterium fastidiosum | 0 | Not detected | -0.150 | -0.295 | -0.004 | 0.044 |
| Fusicatenibacter saccharivorans | 0 | Not detected | 0.021 | -0.091 | 0.132 | 0.714 |
| Fusobacterium mortiferum | 0 | Not detected | 0.286 | -0.036 | 0.609 | 0.082 |
| Fusobacterium necrophorum | 0 | Not detected | -0.088 | -0.299 | 0.123 | 0.415 |
| Fusobacterium nucleatum | 0 | Not detected | 0.097 | -0.188 | 0.382 | 0.504 |
| Fusobacterium periodonticum | 17 | Not detected | 0.217 | -0.021 | 0.455 | 0.075 |
| Gabonia massiliensis | 0 | Not detected | -0.101 | -0.279 | 0.078 | 0.270 |
| Gabonibacter massiliensis | 0 | Not detected | -0.226 | -0.437 | -0.016 | 0.036 |
| Gallibacterium anatis | 0 | Not detected | -0.031 | -0.184 | 0.122 | 0.692 |
| Gardnerella vaginalis | 0 | Not detected | 0.118 | -0.102 | 0.337 | 0.294 |
| Gemella haemolysans | 0 | Not detected | 0.109 | -0.097 | 0.315 | 0.300 |
| Gemella sanguinis | 0 | Not detected | -0.003 | -0.257 | 0.251 | 0.979 |
| Gemmata obscuriglobus | 0 | Not detected | 0.082 | -0.095 | 0.258 | 0.365 |
| Geofilum rubicundum | 0 | Not detected | -0.109 | -0.273 | 0.055 | 0.193 |
| Gilliamella apicola | 0 | Not detected | 0.169 | -0.001 | 0.340 | 0.052 |
| Globicatella sanguinis | 0 | Not detected | -0.085 | -0.279 | 0.108 | 0.388 |
| Gordonibacter pamelaeae | 0 | Not detected | 0.047 | -0.127 | 0.222 | 0.596 |
| Granulicatella elegans | 10 | Not detected | 0.199 | 0.000 | 0.399 | 0.051 |
| Haemophilus influenzae | 824 | 0.6 | 0.467 | 0.187 | 0.747 | 0.001 |
| Hafnia sp. HMSC23F03 | 0 | Not detected | 0.078 | -0.127 | 0.283 | 0.455 |
| Hathewaya proteolytica | 0 | Not detected | -0.042 | -0.238 | 0.155 | 0.677 |
| Heliobacterium sp. N15.MGS-306 | 0 | Not detected | -0.001 | -0.105 | 0.104 | 0.991 |
| Hespellia stercorisuis | 0 | Not detected | -0.019 | -0.114 | 0.076 | 0.697 |
| Holdemanella biformis | 0 | Not detected | -0.003 | -0.172 | 0.167 | 0.976 |
| Holdemania filiformis | 0 | Not detected | -0.007 | -0.111 | 0.098 | 0.903 |
| Holdemania massiliensis | 0 | Not detected | -0.099 | -0.237 | 0.040 | 0.162 |
| Holdemania sp. Marseille-P2844 | 0 | Not detected | -0.063 | -0.189 | 0.063 | 0.325 |
| Hungatella hathewayi | 0 | Not detected | -0.095 | -0.208 | 0.017 | 0.096 |
| Hydrogenoanaerobacterium saccharovorans | 0 | Not detected | -0.057 | -0.196 | 0.083 | 0.424 |
| Idiomarina zobellii | 0 | Not detected | -0.103 | -0.356 | 0.149 | 0.423 |
| Ileibacterium valens | 0 | Not detected | -0.078 | -0.303 | 0.147 | 0.500 |
| Intestinibacter bartlettii | 0 | Not detected | -0.038 | -0.251 | 0.175 | 0.724 |
| Intestinimonas butyriciproducens | 0 | Not detected | -0.031 | -0.166 | 0.104 | 0.656 |

| Intestinimonas massiliensis | 0 | Not detected | -0.041 | -0.164 | 0.083 | 0.518 |
| --- | --- | --- | --- | --- | --- | --- |
| Izhakiella sp. D4N98 | 0 | Not detected | 0.151 | -0.037 | 0.340 | 0.116 |
| Jeotgalibaca sp. PTS2502 | 0 | Not detected | 0.022 | -0.134 | 0.179 | 0.779 |
| Johnsonella ignava | 0 | Not detected | 0.023 | -0.086 | 0.131 | 0.682 |
| Kallipyga massiliensis | 0 | Not detected | -0.062 | -0.260 | 0.136 | 0.538 |
| Kandleria vitulina | 0 | Not detected | 0.206 | -0.106 | 0.518 | 0.197 |
| Kiloniella litopenaei | 0 | Not detected | -0.019 | -0.216 | 0.177 | 0.848 |
| Kingella kingae | 0 | Not detected | -0.076 | -0.263 | 0.112 | 0.428 |
| Klebsiella oxytoca | 0 | Not detected | 0.181 | -0.173 | 0.535 | 0.317 |
| Klebsiella pneumoniae | 0 | Not detected | 0.313 | -0.058 | 0.684 | 0.099 |
| Klebsiella sp. RIT-PI-d | 0 | Not detected | 0.224 | -0.031 | 0.479 | 0.085 |
| Kluyvera ascorbata | 0 | Not detected | 0.171 | -0.153 | 0.495 | 0.302 |
| Kluyvera cryocrescens | 0 | Not detected | 0.254 | -0.078 | 0.585 | 0.134 |
| Kluyvera intermedia | 0 | Not detected | 0.192 | -0.112 | 0.496 | 0.216 |
| Kocuria sp. ZOR0020 | 0 | Not detected | -0.106 | -0.273 | 0.061 | 0.212 |
| Kosakonia sacchari | 0 | Not detected | 0.272 | 0.000 | 0.545 | 0.051 |
| Lachnoanaerobaculum saburreum | 0 | Not detected | 0.003 | -0.109 | 0.116 | 0.956 |
| Lachnoanaerobaculum sp. OBRC5-5 | 0 | Not detected | -0.043 | -0.146 | 0.061 | 0.418 |
| Lachnobacterium bovis | 0 | Not detected | 0.076 | -0.023 | 0.175 | 0.135 |
| Lachnoclostridium phytofermentans | 0 | Not detected | -0.006 | -0.091 | 0.079 | 0.886 |
| Lachnospira multipara | 0 | Not detected | 0.019 | -0.134 | 0.172 | 0.808 |
| Lachnospiraceae bacterium 10-1 | 0 | Not detected | 0.002 | -0.071 | 0.074 | 0.961 |
| Lachnospiraceae bacterium 28-4 | 0 | Not detected | 0.008 | -0.065 | 0.082 | 0.825 |
| Lachnospiraceae bacterium 2_1_46FAA | 0 | Not detected | 0.025 | -0.088 | 0.138 | 0.661 |
| Lachnospiraceae bacterium 3-1 | 0 | Not detected | 0.055 | -0.034 | 0.143 | 0.224 |
| Lachnospiraceae bacterium 3-2 | 0 | Not detected | 0.021 | -0.065 | 0.106 | 0.639 |
| Lachnospiraceae bacterium A2 | 0 | Not detected | -0.029 | -0.117 | 0.060 | 0.524 |
| Lachnospiraceae bacterium A4 | 0 | Not detected | 0.028 | -0.049 | 0.105 | 0.476 |
| Lachnospiraceae bacterium AB2028 | 0 | Not detected | 0.078 | -0.131 | 0.287 | 0.464 |
| Lachnospiraceae bacterium AC2012 | 0 | Not detected | 0.017 | -0.080 | 0.113 | 0.736 |
| Lachnospiraceae bacterium AC2014 | 0 | Not detected | -0.022 | -0.165 | 0.121 | 0.764 |
| Lachnospiraceae bacterium AC2028 | 0 | Not detected | 0.028 | -0.104 | 0.160 | 0.678 |
| Lachnospiraceae bacterium AC2029 | 0 | Not detected | -0.036 | -0.137 | 0.066 | 0.492 |
| Lachnospiraceae bacterium AC2031 | 0 | Not detected | 0.041 | -0.097 | 0.179 | 0.562 |
| Lachnospiraceae bacterium AC3007 | 0 | Not detected | -0.021 | -0.183 | 0.141 | 0.797 |
| Lachnospiraceae bacterium AD3010 | 0 | Not detected | 0.093 | -0.101 | 0.286 | 0.349 |
| Lachnospiraceae bacterium C6A11 | 0 | Not detected | -0.027 | -0.159 | 0.105 | 0.691 |
| Lachnospiraceae bacterium COE1 | 0 | Not detected | 0.039 | -0.070 | 0.149 | 0.482 |
| Lachnospiraceae bacterium FD2005 | 0 | Not detected | 0.055 | -0.106 | 0.215 | 0.504 |
| Lachnospiraceae bacterium FE2018 | 0 | Not detected | -0.076 | -0.277 | 0.125 | 0.459 |
| Lachnospiraceae bacterium G41 | 0 | Not detected | 0.101 | -0.066 | 0.269 | 0.237 |
| Lachnospiraceae bacterium KHCPX20 | 0 | Not detected | -0.052 | -0.257 | 0.154 | 0.622 |
| Lachnospiraceae bacterium M18-1 | 0 | Not detected | -0.038 | -0.121 | 0.046 | 0.374 |
| Lachnospiraceae bacterium MC2017 | 0 | Not detected | 0.015 | -0.125 | 0.156 | 0.830 |

| Lachnospiraceae bacterium NC2004 | 0 | Not detected | -0.016 | -0.194 | 0.163 | 0.864 |
| --- | --- | --- | --- | --- | --- | --- |
| Lachnospiraceae bacterium NC2008 | 0 | Not detected | -0.073 | -0.245 | 0.099 | 0.406 |
| Lachnospiraceae bacterium ND2006 | 0 | Not detected | -0.006 | -0.177 | 0.165 | 0.946 |
| Lachnospiraceae bacterium NK3A20 | 0 | Not detected | -0.120 | -0.322 | 0.082 | 0.245 |
| Lachnospiraceae bacterium NK4A179 | 0 | Not detected | 0.097 | -0.079 | 0.272 | 0.282 |
| Lachnospiraceae bacterium P6B14 | 0 | Not detected | -0.044 | -0.188 | 0.099 | 0.546 |
| Lachnospiraceae bacterium TF01-11 | 0 | Not detected | 0.038 | -0.070 | 0.146 | 0.489 |
| Lachnospiraceae bacterium TWA4 | 0 | Not detected | 0.017 | -0.092 | 0.125 | 0.762 |
| Lachnospiraceae bacterium V9D3004 | 0 | Not detected | 0.002 | -0.162 | 0.165 | 0.986 |
| Lachnospiraceae bacterium YSB2008 | 0 | Not detected | -0.087 | -0.221 | 0.046 | 0.201 |
| Lachnospiraceae bacterium mt14 | 0 | Not detected | -0.105 | -0.258 | 0.048 | 0.179 |
| Lachnospiraceae bacterium oral taxon 500 | 0 | Not detected | -0.016 | -0.104 | 0.071 | 0.718 |
| Lactobacillus acidophilus | 0 | Not detected | -0.137 | -0.292 | 0.018 | 0.083 |
| Lactobacillus amylotrophicus | 0 | Not detected | -0.071 | -0.256 | 0.114 | 0.451 |
| Lactobacillus animalis | 0 | Not detected | -0.177 | -0.349 | -0.005 | 0.044 |
| Lactobacillus antri | 10 | Not detected | -0.243 | -0.457 | -0.028 | 0.027 |
| Lactobacillus aviarius | 0 | Not detected | -0.018 | -0.121 | 0.085 | 0.733 |
| Lactobacillus buchneri | 0 | Not detected | -0.021 | -0.167 | 0.125 | 0.778 |
| Lactobacillus casei | 0 | Not detected | 0.025 | -0.248 | 0.298 | 0.858 |
| Lactobacillus curvatus | 0 | Not detected | -0.054 | -0.258 | 0.150 | 0.605 |
| Lactobacillus delbrueckii | 0 | Not detected | 0.068 | -0.164 | 0.300 | 0.564 |
| Lactobacillus equi | 0 | Not detected | -0.236 | -0.437 | -0.036 | 0.021 |
| Lactobacillus fermentum | 0 | Not detected | -0.028 | -0.338 | 0.282 | 0.860 |
| Lactobacillus gasseri | 0 | Not detected | -0.248 | -0.563 | 0.068 | 0.124 |
| Lactobacillus helveticus | 0 | Not detected | -0.026 | -0.249 | 0.197 | 0.819 |
| Lactobacillus ingluviei | 482 | Not detected | -0.305 | -0.477 | -0.133 | 0.001 |
| Lactobacillus jensenii | 0 | Not detected | -0.077 | -0.263 | 0.110 | 0.419 |
| Lactobacillus kefiranofaciens | 0 | Not detected | -0.175 | -0.352 | 0.001 | 0.052 |
| Lactobacillus manihotivorans | 0 | Not detected | -0.108 | -0.291 | 0.074 | 0.243 |
| Lactobacillus mucosae | 0 | Not detected | -0.210 | -0.430 | 0.010 | 0.062 |
| Lactobacillus paracasei | 0 | Not detected | 0.082 | -0.201 | 0.366 | 0.569 |
| Lactobacillus paraplantarum | 0 | Not detected | 0.066 | -0.124 | 0.257 | 0.494 |
| Lactobacillus pasteurii | 19 | Not detected | -0.174 | -0.338 | -0.011 | 0.036 |
| Lactobacillus plantarum | 0 | Not detected | -0.007 | -0.243 | 0.229 | 0.954 |
| Lactobacillus reuteri | 0 | Not detected | -0.015 | -0.233 | 0.204 | 0.896 |
| Lactobacillus rhamnosus | 0 | Not detected | -0.036 | -0.291 | 0.219 | 0.780 |
| Lactobacillus ruminis | 0 | Not detected | -0.036 | -0.259 | 0.187 | 0.754 |
| Lactobacillus ruminis CAG:367 | 0 | Not detected | 0.050 | -0.176 | 0.277 | 0.662 |
| Lactobacillus sakei | 0 | Not detected | 0.011 | -0.190 | 0.213 | 0.912 |
| Lactobacillus salivarius | 0 | Not detected | -0.116 | -0.417 | 0.184 | 0.448 |
| Lactobacillus vaginalis | 0 | Not detected | -0.185 | -0.451 | 0.081 | 0.172 |
| Lactococcus garvieae | 0 | Not detected | -0.008 | -0.216 | 0.200 | 0.943 |
| Lactococcus lactis | 0 | Not detected | -0.280 | -0.602 | 0.042 | 0.089 |
| Lactococcus piscium | 0 | Not detected | 0.077 | -0.134 | 0.287 | 0.474 |

| Lactonifactor longoviformis | 0 | Not detected | -0.003 | -0.076 | 0.071 | 0.944 |
| --- | --- | --- | --- | --- | --- | --- |
| Lamprocystis purpurea | 0 | Not detected | 0.166 | -0.092 | 0.425 | 0.207 |
| Lascolabacillus massiliensis | 0 | Not detected | 0.092 | -0.068 | 0.251 | 0.260 |
| Lautropia mirabilis | 0 | Not detected | -0.014 | -0.223 | 0.194 | 0.894 |
| Lawsonia intracellularis | 0 | Not detected | -0.127 | -0.328 | 0.074 | 0.215 |
| Leclercia adecarboxylata | 0 | Not detected | 0.230 | -0.076 | 0.536 | 0.142 |
| Lelliottia amnigena | 0 | Not detected | 0.284 | -0.054 | 0.623 | 0.100 |
| Lentimicrobium saccharophilum | 0 | Not detected | -0.021 | -0.232 | 0.190 | 0.845 |
| Leuconostoc citreum | 0 | Not detected | -0.037 | -0.217 | 0.144 | 0.689 |
| Leuconostoc lactis | 0 | Not detected | -0.063 | -0.289 | 0.164 | 0.589 |
| Leuconostoc mesenteroides | 0 | Not detected | 0.091 | -0.144 | 0.327 | 0.449 |
| Levyella massiliensis | 0 | Not detected | 0.018 | -0.096 | 0.133 | 0.754 |
| Libanicoccus massiliensis | 0 | Not detected | 0.058 | -0.148 | 0.264 | 0.580 |
| Luteimonas sp. FCS-9 | 0 | Not detected | 0.051 | -0.095 | 0.198 | 0.493 |
| Lutispora thermophila | 0 | Not detected | 0.024 | -0.151 | 0.199 | 0.790 |
| Macellibacteroides sp. HH-ZS | 0 | Not detected | 0.011 | -0.140 | 0.161 | 0.889 |
| Macrococcus caseolyticus | 0 | Not detected | -0.163 | -0.361 | 0.036 | 0.108 |
| Mageeibacillus indolicus | 0 | Not detected | 0.004 | -0.153 | 0.162 | 0.955 |
| Mangrovibacter sp. MFB070 | 423 | Not detected | 0.299 | 0.061 | 0.537 | 0.014 |
| Mannheimia haemolytica | 0 | Not detected | 0.021 | -0.166 | 0.209 | 0.823 |
| Mariniphaga anaerophila | 0 | Not detected | -0.041 | -0.191 | 0.110 | 0.596 |
| Marvinbryantia formatexigens | 0 | Not detected | -0.007 | -0.110 | 0.096 | 0.891 |
| Massilioclostridium coli | 0 | Not detected | 0.000 | -0.111 | 0.111 | 0.996 |
| Massiliomicrobiota timonensis | 0 | Not detected | 0.063 | -0.142 | 0.269 | 0.546 |
| Mediterranea massiliensis | 0 | Not detected | 0.022 | -0.111 | 0.155 | 0.749 |
| Megamonas funiformis | 0 | Not detected | -0.329 | -0.718 | 0.061 | 0.099 |
| Megasphaera cerevisiae | 0 | Not detected | -0.208 | -0.487 | 0.071 | 0.145 |
| Megasphaera elsdenii | 0 | Not detected | -0.249 | -0.621 | 0.122 | 0.189 |
| Megasphaera genomosp. type_1 | 0 | Not detected | -0.061 | -0.141 | 0.019 | 0.134 |
| Megasphaera massiliensis | 0 | Not detected | -0.162 | -0.511 | 0.188 | 0.365 |
| Megasphaera micronuciformis | 0 | Not detected | 0.117 | -0.129 | 0.362 | 0.353 |
| Megasphaera sp. BV3C16-1 | 0 | Not detected | -0.212 | -0.431 | 0.006 | 0.058 |
| Megasphaera sp. UPII 135-E | 0 | Not detected | -0.023 | -0.111 | 0.064 | 0.599 |
| Merdibacter massiliensis | 0 | Not detected | -0.052 | -0.141 | 0.037 | 0.255 |
| Methanobrevibacter smithii | 0 | Not detected | -0.196 | -0.634 | 0.241 | 0.379 |
| Methanobrevibacter smithii CAG:186 | 0 | Not detected | -0.122 | -0.525 | 0.281 | 0.553 |
| Methanocorpusculaceae archaeon Phil4 | 0 | Not detected | -0.253 | -0.522 | 0.016 | 0.066 |
| Methanosarcina mazei | 0 | Not detected | 0.129 | -0.113 | 0.371 | 0.297 |
| Methylobacterium sp. ZNC0032 | 0 | Not detected | -0.104 | -0.271 | 0.064 | 0.226 |
| Methyloglobulus morosus | 0 | Not detected | 0.031 | -0.169 | 0.231 | 0.764 |
| Microcystis aeruginosa | 0 | Not detected | 0.111 | -0.098 | 0.320 | 0.299 |
| Mitsuokella multacida | 0 | Not detected | -0.283 | -0.657 | 0.091 | 0.138 |
| Mobilibacterium timonense | 0 | Not detected | -0.073 | -0.260 | 0.114 | 0.446 |
| Mobiluncus curtisii | 0 | Not detected | -0.052 | -0.138 | 0.034 | 0.238 |

| Mobiluncus mulieris | 0 | Not detected | -0.058 | -0.155 | 0.039 | 0.243 |
| --- | --- | --- | --- | --- | --- | --- |
| Mogibacterium timidum | 0 | Not detected | 0.036 | -0.191 | 0.264 | 0.754 |
| Moraxella porci | 0 | Not detected | -0.157 | -0.314 | 0.001 | 0.051 |
| Morganella morganii | 0 | Not detected | 0.082 | -0.150 | 0.315 | 0.487 |
| Morococcus cerebrosus | 259 | Not detected | 0.263 | 0.090 | 0.435 | 0.003 |
| Mucinivorans hirudinis | 0 | Not detected | -0.120 | -0.270 | 0.030 | 0.118 |
| Murdochiella sp. Marseille-P2341 | 0 | Not detected | -0.049 | -0.166 | 0.069 | 0.418 |
| Mycoplasma sp. CAG:877 | 0 | Not detected | -0.234 | -0.513 | 0.045 | 0.101 |
| Mycoplasma sp. CAG:956 | 0 | Not detected | -0.091 | -0.337 | 0.155 | 0.469 |
| Myroides injenensis | 0 | Not detected | -0.012 | -0.252 | 0.228 | 0.920 |
| Myroides odoratimimus | 0 | Not detected | -0.151 | -0.417 | 0.115 | 0.265 |
| Necropsobacter massiliensis | 574 | Not detected | 0.310 | 0.089 | 0.532 | 0.006 |
| Negativicoccus massiliensis | 0 | Not detected | -0.110 | -0.281 | 0.062 | 0.210 |
| Neglecta timonensis | 0 | Not detected | 0.008 | -0.132 | 0.149 | 0.906 |
| Neisseria cinerea | 0 | Not detected | 0.066 | -0.125 | 0.258 | 0.498 |
| Neisseria mucosa | 0 | Not detected | 0.197 | 0.006 | 0.388 | 0.043 |
| Nissabacter archeti | 0 | Not detected | 0.171 | -0.065 | 0.408 | 0.156 |
| Novibacillus thermophilus | 133 | Not detected | 0.194 | -0.007 | 0.396 | 0.059 |
| Obesumbacterium proteus | 0 | Not detected | 0.201 | -0.075 | 0.476 | 0.154 |
| Oceanospirillum linum | 0 | Not detected | 0.146 | -0.050 | 0.343 | 0.144 |
| Odoribacter laneus | 0 | Not detected | 0.023 | -0.183 | 0.230 | 0.825 |
| Odoribacter laneus CAG:561 | 0 | Not detected | -0.002 | -0.270 | 0.266 | 0.987 |
| Odoribacter sp. 43_10 | 0 | Not detected | -0.107 | -0.282 | 0.068 | 0.231 |
| Odoribacter sp. CAG:788 | 0 | Not detected | 0.013 | -0.234 | 0.261 | 0.917 |
| Odoribacter splanchnicus | 0 | Not detected | -0.075 | -0.201 | 0.051 | 0.242 |
| Odoribacter splanchnicus CAG:14 | 0 | Not detected | -0.135 | -0.328 | 0.058 | 0.171 |
| Oligella ureolytica | 0 | Not detected | -0.092 | -0.283 | 0.099 | 0.346 |
| Olsenella sp. Marseille-P3197 | 0 | Not detected | -0.169 | -0.381 | 0.042 | 0.117 |
| Olsenella sp. oral taxon 809 | 0 | Not detected | -0.084 | -0.221 | 0.053 | 0.232 |
| Olsenella uli | 0 | Not detected | -0.003 | -0.212 | 0.206 | 0.976 |
| Olsenella umbonata | 0 | Not detected | -0.076 | -0.241 | 0.088 | 0.363 |
| Oribacterium parvum | 0 | Not detected | -0.005 | -0.079 | 0.069 | 0.896 |
| Oribacterium sinus | 0 | Not detected | 0.095 | -0.066 | 0.257 | 0.247 |
| Oribacterium sp. WCC10 | 0 | Not detected | 0.091 | -0.117 | 0.299 | 0.392 |
| Oribacterium sp. oral taxon 078 | 0 | Not detected | -0.116 | -0.238 | 0.005 | 0.061 |
| Oscillibacter ruminantium | 0 | Not detected | -0.073 | -0.241 | 0.095 | 0.392 |
| Oscillibacter sp. 1-3 | 0 | Not detected | 0.042 | -0.053 | 0.137 | 0.389 |
| Oscillibacter sp. 57_20 | 0 | Not detected | 0.229 | -0.082 | 0.540 | 0.149 |
| Oscillibacter sp. ER4 | 0 | Not detected | 0.096 | -0.088 | 0.279 | 0.307 |
| Oscillibacter sp. KLE 1745 | 0 | Not detected | -0.100 | -0.260 | 0.059 | 0.217 |
| Oscillibacter valericigenes | 0 | Not detected | -0.060 | -0.219 | 0.098 | 0.454 |
| Oscillospiraceae bacterium Zagget12 | 0 | Not detected | 0.110 | -0.064 | 0.284 | 0.215 |
| Oxalobacter formigenes | 0 | Not detected | -0.011 | -0.203 | 0.181 | 0.913 |
| Paeniclostridium sordellii | 0 | Not detected | 0.069 | -0.150 | 0.289 | 0.536 |

| Paludibacter propionicigenes | 0 | Not detected | -0.053 | -0.200 | 0.093 | 0.475 |
| --- | --- | --- | --- | --- | --- | --- |
| Pantoea agglomerans | 0 | Not detected | 0.172 | -0.018 | 0.362 | 0.076 |
| Pantoea ananatis | 0 | Not detected | 0.156 | -0.018 | 0.331 | 0.079 |
| Pantoea dispersa | 31 | Not detected | 0.181 | 0.010 | 0.353 | 0.038 |
| Pantoea rwandensis | 0 | Not detected | 0.145 | -0.043 | 0.334 | 0.132 |
| Pantoea sp. IMH | 0 | Not detected | 0.086 | -0.108 | 0.279 | 0.385 |
| Pantoea sp. PSNIH2 | 0 | Not detected | 0.177 | -0.120 | 0.474 | 0.243 |
| Parabacteroides distasonis | 0 | Not detected | 0.056 | -0.128 | 0.241 | 0.550 |
| Parabacteroides goldsteinii | 0 | Not detected | -0.068 | -0.263 | 0.127 | 0.494 |
| Parabacteroides gordonii | 0 | Not detected | -0.146 | -0.308 | 0.015 | 0.077 |
| Parabacteroides johnsonii | 0 | Not detected | -0.065 | -0.206 | 0.076 | 0.368 |
| Parabacteroides merdae CAG:48 | 0 | Not detected | -0.099 | -0.360 | 0.163 | 0.460 |
| Paraburkholderia sacchari | 0 | Not detected | 0.029 | -0.165 | 0.223 | 0.769 |
| Paraclostridium bifermentans | 0 | Not detected | -0.025 | -0.221 | 0.171 | 0.802 |
| Paraprevotella clara CAG:116 | 0 | Not detected | -0.029 | -0.340 | 0.281 | 0.853 |
| Paraprevotella xylaniphila | 0 | Not detected | 0.068 | -0.094 | 0.230 | 0.413 |
| Parascardovia denticolens | 0 | Not detected | 0.029 | -0.182 | 0.239 | 0.790 |
| Parasporobacterium paucivorans | 0 | Not detected | -0.012 | -0.135 | 0.110 | 0.842 |
| Parasutterella excrementihominis | 0 | Not detected | -0.031 | -0.339 | 0.276 | 0.841 |
| Parasutterella excrementihominis CAG:233 | 0 | Not detected | -0.119 | -0.560 | 0.323 | 0.599 |
| Parvimonas micra | 0 | Not detected | -0.099 | -0.331 | 0.132 | 0.401 |
| Pasteurella bettyae | 23 | Not detected | 0.220 | 0.036 | 0.405 | 0.019 |
| Pectobacterium carotovorum | 0 | Not detected | 0.136 | -0.040 | 0.312 | 0.131 |
| Pedobacter glucosidilyticus | 0 | Not detected | 0.034 | -0.147 | 0.215 | 0.713 |
| Pelosinus fermentans | 1 | Not detected | -0.183 | -0.351 | -0.016 | 0.032 |
| Peptoanaerobacter stomatis | 0 | Not detected | -0.065 | -0.232 | 0.101 | 0.442 |
| Peptococcus niger | 0 | Not detected | 0.077 | -0.090 | 0.244 | 0.368 |
| Peptoniphilus duerdenii | 0 | Not detected | -0.097 | -0.251 | 0.058 | 0.221 |
| Peptoniphilus grossensis | 0 | Not detected | -0.159 | -0.346 | 0.029 | 0.098 |
| Peptoniphilus harei | 0 | Not detected | -0.047 | -0.255 | 0.161 | 0.658 |
| Peptoniphilus lacrimalis | 0 | Not detected | -0.086 | -0.314 | 0.142 | 0.458 |
| Peptoniphilus obesi | 0 | Not detected | -0.014 | -0.222 | 0.194 | 0.895 |
| Peptoniphilus rhinitidis | 0 | Not detected | -0.085 | -0.284 | 0.113 | 0.400 |
| Peptoniphilus senegalensis | 0 | Not detected | 0.053 | -0.164 | 0.269 | 0.633 |
| Peptoniphilus sp. BV3C26 | 0 | Not detected | -0.025 | -0.254 | 0.205 | 0.834 |
| Peptoniphilus sp. ING2-D1G | 0 | Not detected | -0.108 | -0.302 | 0.087 | 0.278 |
| Peptoniphilus sp. oral taxon 375 | 0 | Not detected | -0.016 | -0.125 | 0.094 | 0.778 |
| Peptostreptococcaceae bacterium VA2 | 0 | Not detected | 0.162 | -0.089 | 0.412 | 0.206 |
| Peptostreptococcus anaerobius | 0 | Not detected | -0.059 | -0.145 | 0.027 | 0.177 |
| Peptostreptococcus anaerobius CAG:621 | 0 | Not detected | 0.036 | -0.143 | 0.214 | 0.693 |
| Peptostreptococcus stomatis | 0 | Not detected | -0.187 | -0.385 | 0.011 | 0.065 |
| Petrimonas mucosa | 0 | Not detected | -0.113 | -0.323 | 0.098 | 0.294 |
| Phaeodactylibacter xiamenensis | 0 | Not detected | 0.013 | -0.250 | 0.276 | 0.924 |
| Phascolarctobacterium sp. CAG:207 | 0 | Not detected | -0.159 | -0.601 | 0.283 | 0.480 |

| Phascolarctobacterium sp. CAG:266 | 0 | Not detected | 0.061 | -0.219 | 0.341 | 0.667 |
| --- | --- | --- | --- | --- | --- | --- |
| Phascolarctobacterium succinatutens | 0 | Not detected | 0.011 | -0.216 | 0.239 | 0.923 |
| Phocaeicola abscessus | 0 | Not detected | -0.046 | -0.207 | 0.116 | 0.580 |
| Phocea massiliensis | 0 | Not detected | -0.066 | -0.185 | 0.053 | 0.280 |
| Piscirickettsia salmonis | 0 | Not detected | -0.101 | -0.264 | 0.063 | 0.227 |
| Plautia stali symbiont | 19 | Not detected | 0.209 | 0.031 | 0.387 | 0.021 |
| Pluralibacter gergoviae | 0 | Not detected | 0.151 | -0.127 | 0.429 | 0.287 |
| Pontibacillus litoralis | 0 | Not detected | -0.206 | -0.476 | 0.064 | 0.135 |
| Porphyromonadaceae bacterium COT-184 O | 0 | Not detected | -0.015 | -0.197 | 0.168 | 0.874 |
| Porphyromonadaceae bacterium KH3CP3R | 0 | Not detected | -0.007 | -0.183 | 0.169 | 0.936 |
| Porphyromonadaceae bacterium KH3R12 | 0 | Not detected | -0.049 | -0.212 | 0.114 | 0.555 |
| Porphyromonas asaccharolytica | 0 | Not detected | -0.046 | -0.259 | 0.168 | 0.676 |
| Porphyromonas bennonis | 0 | Not detected | -0.017 | -0.222 | 0.188 | 0.872 |
| Porphyromonas cangingivalis | 0 | Not detected | -0.009 | -0.196 | 0.177 | 0.921 |
| Porphyromonas canoris | 0 | Not detected | -0.051 | -0.242 | 0.139 | 0.596 |
| Porphyromonas catoniae | 0 | Not detected | -0.137 | -0.273 | -0.001 | 0.049 |
| Porphyromonas crevioricanis | 0 | Not detected | 0.091 | -0.061 | 0.243 | 0.240 |
| Porphyromonas endodontalis | 0 | Not detected | -0.073 | -0.282 | 0.135 | 0.490 |
| Porphyromonas gingivalis | 0 | Not detected | -0.018 | -0.192 | 0.157 | 0.844 |
| Porphyromonas gingivicanis | 0 | Not detected | -0.121 | -0.253 | 0.012 | 0.075 |
| Porphyromonas levii | 254 | Not detected | -0.329 | -0.585 | -0.072 | 0.012 |
| Porphyromonas macacae | 0 | Not detected | -0.009 | -0.195 | 0.177 | 0.926 |
| Porphyromonas somerae | 0 | Not detected | 0.036 | -0.068 | 0.140 | 0.496 |
| Porphyromonas sp. CAG:1061 | 0 | Not detected | -0.273 | -0.506 | -0.040 | 0.022 |
| Porphyromonas sp. COT-239 OH1446 | 0 | Not detected | 0.056 | -0.206 | 0.318 | 0.674 |
| Porphyromonas sp. COT-290 OH3588 | 0 | Not detected | 0.089 | -0.138 | 0.315 | 0.442 |
| Prevotella albensis | 0 | Not detected | 0.107 | -0.259 | 0.473 | 0.566 |
| Prevotella amnii | 0 | Not detected | 0.042 | -0.058 | 0.143 | 0.410 |
| Prevotella aurantiaca | 0 | Not detected | 0.075 | -0.158 | 0.308 | 0.530 |
| Prevotella baroniae | 0 | Not detected | 0.063 | -0.056 | 0.181 | 0.300 |
| Prevotella bergensis | 0 | Not detected | 0.184 | 0.000 | 0.367 | 0.050 |
| Prevotella bivia | 0 | Not detected | 0.069 | -0.060 | 0.199 | 0.296 |
| Prevotella bryantii | 0 | Not detected | 0.067 | -0.300 | 0.434 | 0.720 |
| Prevotella buccae | 0 | Not detected | 0.070 | -0.187 | 0.328 | 0.593 |
| Prevotella buccalis | 0 | Not detected | 0.045 | -0.067 | 0.157 | 0.433 |
| Prevotella copri | 0 | Not detected | 0.164 | -0.240 | 0.568 | 0.426 |
| Prevotella corporis | 0 | Not detected | 0.018 | -0.091 | 0.127 | 0.742 |
| Prevotella dentalis | 0 | Not detected | 0.059 | -0.225 | 0.343 | 0.684 |
| Prevotella dentasini | 0 | Not detected | 0.162 | -0.107 | 0.431 | 0.239 |
| Prevotella denticola | 0 | Not detected | -0.006 | -0.310 | 0.298 | 0.970 |
| Prevotella disiens | 0 | Not detected | 0.009 | -0.107 | 0.124 | 0.884 |
| Prevotella enoeca | 0 | Not detected | 0.027 | -0.238 | 0.292 | 0.841 |
| Prevotella fusca | 0 | Not detected | -0.050 | -0.364 | 0.264 | 0.754 |
| Prevotella histicola | 0 | Not detected | 0.084 | -0.111 | 0.278 | 0.400 |

| Prevotella intermedia | 0 | Not detected | 0.103 | -0.076 | 0.282 | 0.259 |
| --- | --- | --- | --- | --- | --- | --- |
| Prevotella loescheii | 0 | Not detected | 0.048 | -0.195 | 0.291 | 0.696 |
| Prevotella maculosa | 0 | Not detected | 0.100 | -0.116 | 0.317 | 0.365 |
| Prevotella marshii | 0 | Not detected | 0.073 | -0.043 | 0.189 | 0.219 |
| Prevotella melaninogenica | 0 | Not detected | 0.111 | -0.123 | 0.346 | 0.353 |
| Prevotella micans | 0 | Not detected | 0.000 | -0.244 | 0.245 | 0.997 |
| Prevotella multiformis | 0 | Not detected | 0.063 | -0.107 | 0.233 | 0.466 |
| Prevotella multisaccharivorax | 0 | Not detected | 0.172 | -0.187 | 0.530 | 0.348 |
| Prevotella nanceiensis | 0 | Not detected | 0.119 | -0.092 | 0.330 | 0.269 |
| Prevotella nigrescens | 0 | Not detected | 0.081 | -0.159 | 0.321 | 0.508 |
| Prevotella oralis | 0 | Not detected | -0.009 | -0.264 | 0.246 | 0.946 |
| Prevotella oris | 0 | Not detected | 0.125 | -0.171 | 0.421 | 0.408 |
| Prevotella oryzae | 0 | Not detected | 0.108 | -0.201 | 0.416 | 0.493 |
| Prevotella oulorum | 0 | Not detected | 0.019 | -0.303 | 0.341 | 0.909 |
| Prevotella pallens | 0 | Not detected | 0.107 | -0.022 | 0.237 | 0.103 |
| Prevotella paludivivens | 1 | Not detected | 0.106 | -0.086 | 0.297 | 0.281 |
| Prevotella ruminicola | 0 | Not detected | 0.025 | -0.267 | 0.317 | 0.868 |
| Prevotella saccharolytica | 0 | Not detected | 0.027 | -0.251 | 0.305 | 0.850 |
| Prevotella salivae | 0 | Not detected | 0.072 | -0.048 | 0.193 | 0.240 |
| Prevotella scopos | 0 | Not detected | 0.083 | -0.077 | 0.243 | 0.311 |
| Prevotella sp. 10(H) | 0 | Not detected | -0.086 | -0.253 | 0.081 | 0.312 |
| Prevotella sp. 109 | 0 | Not detected | 0.074 | -0.042 | 0.190 | 0.212 |
| Prevotella sp. AGR2160 | 0 | Not detected | 0.154 | -0.177 | 0.485 | 0.362 |
| Prevotella sp. BP1-145 | 0 | Not detected | 0.034 | -0.086 | 0.154 | 0.582 |
| Prevotella sp. BP1-148 | 0 | Not detected | 0.036 | -0.102 | 0.174 | 0.611 |
| Prevotella sp. CAG:1058 | 0 | Not detected | 0.135 | -0.133 | 0.403 | 0.324 |
| Prevotella sp. CAG:1092 | 0 | Not detected | 0.311 | -0.046 | 0.669 | 0.088 |
| Prevotella sp. CAG:1124 | 0 | Not detected | -0.221 | -0.496 | 0.054 | 0.116 |
| Prevotella sp. CAG:1185 | 0 | Not detected | 0.113 | -0.177 | 0.404 | 0.444 |
| Prevotella sp. CAG:1320 | 0 | Not detected | 0.163 | -0.075 | 0.400 | 0.180 |
| Prevotella sp. CAG:255 | 0 | Not detected | -0.003 | -0.199 | 0.192 | 0.972 |
| Prevotella sp. CAG:386 | 0 | Not detected | 0.168 | -0.253 | 0.590 | 0.434 |
| Prevotella sp. CAG:487 | 0 | Not detected | 0.040 | -0.157 | 0.237 | 0.690 |
| Prevotella sp. CAG:520 | 0 | Not detected | 0.095 | -0.246 | 0.436 | 0.586 |
| Prevotella sp. CAG:5226 | 0 | Not detected | 0.151 | -0.218 | 0.521 | 0.423 |
| Prevotella sp. CAG:604 | 0 | Not detected | 0.261 | -0.191 | 0.713 | 0.259 |
| Prevotella sp. CAG:732 | 0 | Not detected | 0.197 | -0.213 | 0.607 | 0.346 |
| Prevotella sp. CAG:891 | 0 | Not detected | 0.158 | -0.123 | 0.439 | 0.271 |
| Prevotella sp. FD3004 | 0 | Not detected | 0.124 | -0.099 | 0.347 | 0.277 |
| Prevotella sp. HUN102 | 0 | Not detected | 0.019 | -0.143 | 0.181 | 0.820 |
| Prevotella sp. MSX73 | 0 | Not detected | 0.029 | -0.227 | 0.285 | 0.824 |
| Prevotella sp. P4-76 | 0 | Not detected | 0.064 | -0.146 | 0.274 | 0.549 |
| Prevotella sp. P6B1 | 0 | Not detected | 0.132 | -0.012 | 0.276 | 0.073 |
| Prevotella sp. P6B4 | 0 | Not detected | 0.090 | -0.054 | 0.233 | 0.220 |

| Prevotella sp. RM4 | 0 | Not detected | 0.029 | -0.155 | 0.212 | 0.758 |
| --- | --- | --- | --- | --- | --- | --- |
| Prevotella sp. S7 MS 2 | 0 | Not detected | 0.073 | -0.045 | 0.191 | 0.223 |
| Prevotella sp. S7-1-8 | 0 | Not detected | 0.020 | -0.093 | 0.132 | 0.733 |
| Prevotella sp. kh1p2 | 0 | Not detected | 0.042 | -0.215 | 0.298 | 0.751 |
| Prevotella sp. khp1 | 0 | Not detected | 0.091 | -0.099 | 0.281 | 0.348 |
| Prevotella sp. ne3005 | 6 | Not detected | 0.103 | -0.060 | 0.266 | 0.215 |
| Prevotella sp. oral taxon 473 | 0 | Not detected | -0.033 | -0.290 | 0.224 | 0.802 |
| Prevotella stercorea | 0 | Not detected | 0.172 | 0.014 | 0.330 | 0.033 |
| Prevotella stercorea CAG:629 | 0 | Not detected | 0.081 | -0.280 | 0.442 | 0.661 |
| Prevotella timonensis | 0 | Not detected | 0.066 | -0.044 | 0.177 | 0.241 |
| Prevotellamassilia timonensis | 0 | Not detected | 0.077 | -0.110 | 0.264 | 0.422 |
| Propionibacterium acidifaciens | 0 | Not detected | -0.117 | -0.320 | 0.085 | 0.257 |
| Proteiniclasticum ruminis | 0 | Not detected | 0.118 | -0.050 | 0.286 | 0.168 |
| Proteiniphilum acetatigenes | 0 | Not detected | -0.004 | -0.182 | 0.175 | 0.969 |
| Proteiniphilum saccharofermentans | 0 | Not detected | -0.031 | -0.227 | 0.165 | 0.756 |
| Proteiniphilum sp. EBM-41 | 0 | Not detected | -0.055 | -0.299 | 0.189 | 0.659 |
| Proteobacteria bacterium CAG:139 | 0 | Not detected | -0.111 | -0.532 | 0.309 | 0.605 |
| Proteobacteria bacterium CAG:495 | 0 | Not detected | -0.281 | -0.621 | 0.059 | 0.106 |
| Providencia rettgeri | 0 | Not detected | 0.033 | -0.186 | 0.251 | 0.769 |
| Pseudobacteroides cellulosolvens | 0 | Not detected | 0.004 | -0.144 | 0.152 | 0.957 |
| Pseudobutyrivibrio ruminis | 0 | Not detected | 0.026 | -0.065 | 0.116 | 0.579 |
| Pseudobutyrivibrio sp. ACV-2 | 0 | Not detected | -0.024 | -0.197 | 0.148 | 0.782 |
| Pseudobutyrivibrio sp. JW11 | 0 | Not detected | -0.014 | -0.191 | 0.162 | 0.872 |
| Pseudobutyrivibrio sp. MD2005 | 0 | Not detected | 0.105 | -0.079 | 0.288 | 0.263 |
| Pseudobutyrivibrio sp. UC1225 | 0 | Not detected | -0.088 | -0.221 | 0.045 | 0.197 |
| Pseudobutyrivibrio sp. YE44 | 0 | Not detected | 0.064 | -0.106 | 0.234 | 0.461 |
| Pseudobutyrivibrio xylanivorans | 0 | Not detected | -0.011 | -0.187 | 0.166 | 0.905 |
| Pseudoflavonifractor capillosus | 0 | Not detected | -0.033 | -0.135 | 0.069 | 0.524 |
| Pseudopropionibacterium propionicum | 0 | Not detected | -0.033 | -0.235 | 0.168 | 0.747 |
| Pyramidobacter piscolens | 0 | Not detected | -0.074 | -0.353 | 0.205 | 0.602 |
| Rahnella aquatilis | 0 | Not detected | 0.047 | -0.126 | 0.221 | 0.593 |
| Ralstonia mannitolilytica | 0 | Not detected | -0.058 | -0.300 | 0.184 | 0.639 |
| Ralstonia pickettii | 0 | Not detected | -0.118 | -0.343 | 0.107 | 0.305 |
| Raoultella ornithinolytica | 0 | Not detected | 0.238 | -0.107 | 0.582 | 0.177 |
| Rappaport israeli | 0 | Not detected | 0.189 | 0.003 | 0.375 | 0.047 |
| Rhodococcus defluvii | 0 | Not detected | -0.032 | -0.200 | 0.136 | 0.711 |
| Rhodopseudomonas palustris | 105 | Not detected | 0.229 | 0.023 | 0.434 | 0.030 |
| Rhodospirillum sp. UNK.MGS-17 | 0 | Not detected | -0.149 | -0.554 | 0.257 | 0.473 |
| Riemerella columbina | 0 | Not detected | 0.018 | -0.090 | 0.127 | 0.742 |
| Rikenella microfusus | 0 | Not detected | -0.004 | -0.113 | 0.105 | 0.940 |
| Risungbinella massiliensis | 0 | Not detected | -0.021 | -0.162 | 0.121 | 0.774 |
| Robinsoniella peoriensis | 0 | Not detected | 0.010 | -0.063 | 0.084 | 0.784 |
| Rodentibacter pneumotropicus | 0 | Not detected | 0.179 | 0.024 | 0.335 | 0.024 |
| Roseburia faecis | 0 | Not detected | 0.043 | -0.126 | 0.213 | 0.617 |

| Roseburia hominis | 0 | Not detected | -0.037 | -0.145 | 0.072 | 0.507 |
| --- | --- | --- | --- | --- | --- | --- |
| Roseburia intestinalis | 0 | Not detected | 0.137 | -0.028 | 0.302 | 0.104 |
| Roseburia inulinivorans | 0 | Not detected | 0.036 | -0.097 | 0.169 | 0.599 |
| Roseburia sp. CAG:197_41_10 | 0 | Not detected | 0.023 | -0.110 | 0.157 | 0.733 |
| Roseburia sp. CAG:309 | 0 | Not detected | 0.029 | -0.238 | 0.296 | 0.830 |
| Roseburia sp. CAG:380 | 0 | Not detected | -0.068 | -0.328 | 0.191 | 0.607 |
| Rothia dentocariosa | 0 | Not detected | 0.225 | -0.028 | 0.478 | 0.082 |
| Rothia mucilaginosa | 0 | Not detected | 0.256 | -0.025 | 0.537 | 0.075 |
| Ruminiclostridium thermocellum | 0 | Not detected | -0.117 | -0.232 | -0.001 | 0.048 |
| Ruminococcaceae bacterium AB4001 | 0 | Not detected | -0.053 | -0.207 | 0.101 | 0.502 |
| Ruminococcaceae bacterium AE2021 | 0 | Not detected | -0.006 | -0.150 | 0.139 | 0.940 |
| Ruminococcaceae bacterium D16 | 0 | Not detected | -0.039 | -0.133 | 0.054 | 0.410 |
| Ruminococcaceae bacterium YRB3002 | 0 | Not detected | -0.066 | -0.248 | 0.116 | 0.476 |
| Ruminococcus albus | 0 | Not detected | -0.126 | -0.299 | 0.047 | 0.153 |
| Ruminococcus bicirculans | 0 | Not detected | -0.222 | -0.501 | 0.056 | 0.117 |
| Ruminococcus callidus | 0 | Not detected | -0.066 | -0.255 | 0.123 | 0.493 |
| Ruminococcus champanellensis | 0 | Not detected | -0.103 | -0.341 | 0.135 | 0.397 |
| Ruminococcus faecis | 0 | Not detected | 0.009 | -0.118 | 0.136 | 0.891 |
| Ruminococcus flavefaciens | 0 | Not detected | -0.106 | -0.278 | 0.067 | 0.230 |
| Ruminococcus gauvreauii | 1 | Not detected | -0.105 | -0.200 | -0.010 | 0.030 |
| Ruminococcus lactaris | 0 | Not detected | -0.036 | -0.179 | 0.108 | 0.627 |
| Ruminococcus obeum CAG:39 | 0 | Not detected | 0.059 | -0.109 | 0.227 | 0.494 |
| Ruminococcus sp. AT10 | 0 | Not detected | 0.036 | -0.053 | 0.125 | 0.432 |
| Ruminococcus sp. CAG:108-related_41_35 | 0 | Not detected | -0.065 | -0.360 | 0.230 | 0.665 |
| Ruminococcus sp. CAG:177 | 0 | Not detected | 0.286 | -0.167 | 0.739 | 0.216 |
| Ruminococcus sp. CAG:254 | 0 | Not detected | 0.064 | -0.340 | 0.467 | 0.758 |
| Ruminococcus sp. CAG:330 | 0 | Not detected | -0.137 | -0.514 | 0.239 | 0.475 |
| Ruminococcus sp. CAG:379 | 0 | Not detected | -0.106 | -0.378 | 0.165 | 0.442 |
| Ruminococcus sp. CAG:382 | 0 | Not detected | -0.095 | -0.463 | 0.273 | 0.613 |
| Ruminococcus sp. CAG:403 | 0 | Not detected | 0.058 | -0.240 | 0.356 | 0.703 |
| Ruminococcus sp. CAG:488 | 0 | Not detected | -0.112 | -0.373 | 0.149 | 0.401 |
| Ruminococcus sp. CAG:563 | 0 | Not detected | -0.035 | -0.316 | 0.246 | 0.808 |
| Ruminococcus sp. CAG:60 | 0 | Not detected | -0.014 | -0.184 | 0.157 | 0.875 |
| Ruminococcus sp. CAG:624 | 0 | Not detected | -0.052 | -0.314 | 0.209 | 0.696 |
| Ruminococcus sp. CAG:724 | 0 | Not detected | -0.035 | -0.341 | 0.270 | 0.821 |
| Ruminococcus sp. FC2018 | 0 | Not detected | -0.214 | -0.399 | -0.030 | 0.023 |
| Ruminococcus sp. HUN007 | 0 | Not detected | -0.126 | -0.283 | 0.031 | 0.115 |
| Ruminococcus sp. NK3A76 | 0 | Not detected | -0.140 | -0.326 | 0.046 | 0.140 |
| Ruminococcus sp. Phil15 | 0 | Not detected | -0.059 | -0.295 | 0.178 | 0.626 |
| Ruminococcus sp. Zagget7 | 0 | Not detected | -0.005 | -0.153 | 0.143 | 0.946 |
| Ruminococcus torques CAG:61 | 0 | Not detected | -0.029 | -0.312 | 0.254 | 0.840 |
| Ruthenibacterium lactatiformans | 0 | Not detected | -0.077 | -0.206 | 0.052 | 0.243 |
| Salmonella enterica | 0 | Not detected | 0.249 | -0.051 | 0.548 | 0.105 |
| Sanguibacteroides justesenii | 0 | Not detected | 0.080 | -0.132 | 0.292 | 0.458 |

| Scardovia inopinata | 0 | Not detected | -0.074 | -0.245 | 0.098 | 0.400 |
| --- | --- | --- | --- | --- | --- | --- |
| Scardovia wiggsiae | 0 | Not detected | -0.097 | -0.328 | 0.134 | 0.411 |
| Selenomonas bovis | 590 | Not detected | -0.212 | -0.368 | -0.055 | 0.008 |
| Selenomonas noxia | 0 | Not detected | -0.086 | -0.257 | 0.086 | 0.328 |
| Selenomonas ruminantium | 652 | Not detected | -0.334 | -0.553 | -0.115 | 0.003 |
| Selenomonas sputigena | 102 | Not detected | -0.270 | -0.447 | -0.094 | 0.003 |
| Sellimonas intestinalis | 0 | Not detected | 0.004 | -0.131 | 0.138 | 0.959 |
| Senegalimassilia anaerobia | 0 | Not detected | 0.038 | -0.200 | 0.275 | 0.756 |
| Serratia fonticola | 0 | Not detected | 0.120 | -0.047 | 0.287 | 0.159 |
| Serratia liquefaciens | 0 | Not detected | 0.056 | -0.118 | 0.230 | 0.530 |
| Serratia marcescens | 19 | Not detected | 0.158 | -0.033 | 0.350 | 0.106 |
| Serratia odorifera | 0 | Not detected | 0.049 | -0.135 | 0.233 | 0.600 |
| Serratia rubidaea | 0 | Not detected | 0.190 | -0.004 | 0.385 | 0.055 |
| Sharpea azabuensis | 0 | Not detected | 0.017 | -0.099 | 0.134 | 0.771 |
| Shigella dysenteriae | 0 | Not detected | 0.097 | -0.230 | 0.425 | 0.560 |
| Shigella flexneri | 0 | Not detected | 0.107 | -0.220 | 0.434 | 0.520 |
| Shimwellia blattae | 0 | Not detected | 0.198 | -0.053 | 0.448 | 0.122 |
| Shuttleworthia satelles | 0 | Not detected | -0.063 | -0.180 | 0.053 | 0.288 |
| Siccibacter turicensis | 0 | Not detected | 0.187 | -0.077 | 0.452 | 0.166 |
| Slackia exigua | 0 | Not detected | -0.146 | -0.328 | 0.036 | 0.115 |
| Slackia heliotrinireducens | 0 | Not detected | -0.056 | -0.211 | 0.098 | 0.475 |
| Slackia piriformis | 0 | Not detected | 0.106 | -0.185 | 0.398 | 0.475 |
| Solobacterium moorei | 0 | Not detected | -0.058 | -0.191 | 0.075 | 0.395 |
| Sphaerochaeta sp. Phil3 | 0 | Not detected | -0.006 | -0.230 | 0.217 | 0.955 |
| Sphingobacterium wenxiniae | 0 | Not detected | -0.158 | -0.371 | 0.054 | 0.144 |
| Spirochaetales bacterium Spiro_07 | 0 | Not detected | -0.158 | -0.309 | -0.007 | 0.040 |
| Sporobacter termitidis | 0 | Not detected | -0.036 | -0.214 | 0.141 | 0.690 |
| Sporomusa ovata | 0 | Not detected | -0.099 | -0.284 | 0.087 | 0.298 |
| Staphylococcus pseudintermedius | 0 | Not detected | 0.037 | -0.157 | 0.230 | 0.711 |
| Staphylococcus sp. CAG:324 | 0 | Not detected | -0.306 | -0.665 | 0.053 | 0.095 |
| Staphylococcus sp. DORA_6_22 | 0 | Not detected | -0.006 | -0.199 | 0.187 | 0.952 |
| Staphylococcus warneri | 0 | Not detected | -0.068 | -0.279 | 0.143 | 0.528 |
| Stomatobaculum longum | 0 | Not detected | -0.003 | -0.079 | 0.073 | 0.937 |
| Streptobacillus hongkongensis | 0 | Not detected | -0.070 | -0.259 | 0.120 | 0.471 |
| Streptobacillus notomytis | 0 | Not detected | 0.121 | -0.094 | 0.336 | 0.270 |
| Streptococcus agalactiae | 0 | Not detected | -0.107 | -0.323 | 0.108 | 0.329 |
| Streptococcus anginosus | 0 | Not detected | -0.073 | -0.326 | 0.179 | 0.568 |
| Streptococcus caballi | 0 | Not detected | -0.029 | -0.245 | 0.188 | 0.794 |
| Streptococcus cristatus | 0 | Not detected | 0.020 | -0.180 | 0.221 | 0.842 |
| Streptococcus dysgalactiae | 0 | Not detected | 0.151 | -0.060 | 0.362 | 0.162 |
| Streptococcus entericus | 0 | Not detected | -0.154 | -0.328 | 0.021 | 0.084 |
| Streptococcus gallolyticus | 0 | Not detected | 0.097 | -0.177 | 0.371 | 0.487 |
| Streptococcus gordonii | 0 | Not detected | 0.111 | -0.150 | 0.371 | 0.405 |
| Streptococcus halotolerans | 1 | Not detected | -0.055 | -0.229 | 0.119 | 0.537 |

| Streptococcus henryi | 0 | Not detected | -0.006 | -0.215 | 0.203 | 0.957 |
| --- | --- | --- | --- | --- | --- | --- |
| Streptococcus ictaluri | 0 | Not detected | 0.009 | -0.176 | 0.193 | 0.926 |
| Streptococcus infantis | 0 | Not detected | 0.165 | -0.043 | 0.372 | 0.120 |
| Streptococcus iniae | 0 | Not detected | 0.004 | -0.187 | 0.196 | 0.964 |
| Streptococcus intermedius | 0 | Not detected | -0.055 | -0.298 | 0.187 | 0.655 |
| Streptococcus massiliensis | 0 | Not detected | 0.005 | -0.186 | 0.197 | 0.955 |
| Streptococcus minor | 0 | Not detected | 0.036 | -0.109 | 0.181 | 0.627 |
| Streptococcus mitis | 0 | Not detected | 0.029 | -0.133 | 0.192 | 0.726 |
| Streptococcus mutans | 0 | Not detected | -0.226 | -0.520 | 0.068 | 0.132 |
| Streptococcus oralis | 0 | Not detected | 0.169 | -0.068 | 0.405 | 0.163 |
| Streptococcus orisratti | 0 | Not detected | 0.046 | -0.161 | 0.254 | 0.661 |
| Streptococcus ovis | 0 | Not detected | 0.070 | -0.117 | 0.257 | 0.463 |
| Streptococcus parasanguinis | 0 | Not detected | 0.162 | -0.094 | 0.418 | 0.214 |
| Streptococcus parauberis | 0 | Not detected | -0.034 | -0.225 | 0.157 | 0.724 |
| Streptococcus phocae | 0 | Not detected | 0.015 | -0.129 | 0.159 | 0.834 |
| Streptococcus plurextorum | 4 | Not detected | -0.112 | -0.215 | -0.009 | 0.034 |
| Streptococcus porci | 0 | Not detected | -0.051 | -0.173 | 0.071 | 0.409 |
| Streptococcus pseudoporcinus | 0 | Not detected | -0.047 | -0.253 | 0.160 | 0.658 |
| Streptococcus pyogenes | 0 | Not detected | 0.017 | -0.194 | 0.227 | 0.877 |
| Streptococcus salivarius | 0 | Not detected | 0.094 | -0.143 | 0.331 | 0.435 |
| Streptococcus sanguinis | 0 | Not detected | 0.087 | -0.160 | 0.335 | 0.490 |
| Streptococcus sobrinus | 0 | Not detected | -0.041 | -0.261 | 0.178 | 0.712 |
| Streptococcus sp. X13SY08 | 1 | Not detected | -0.069 | -0.219 | 0.081 | 0.365 |
| Streptococcus suis | 1 | Not detected | -0.069 | -0.184 | 0.046 | 0.242 |
| Streptococcus thermophilus | 0 | Not detected | -0.072 | -0.328 | 0.184 | 0.582 |
| Streptococcus uberis | 0 | Not detected | 0.079 | -0.103 | 0.260 | 0.394 |
| Streptococcus urinalis | 1 | Not detected | -0.024 | -0.234 | 0.187 | 0.825 |
| Streptomyces albulus | 0 | Not detected | 0.040 | -0.128 | 0.208 | 0.640 |
| Streptomyces cattleya | 0 | Not detected | 0.008 | -0.200 | 0.215 | 0.942 |
| Streptomyces kanamyceticus | 0 | Not detected | -0.017 | -0.137 | 0.103 | 0.782 |
| Subdoligranulum sp. CAG:314 | 0 | Not detected | -0.126 | -0.557 | 0.304 | 0.565 |
| Subdoligranulum variabile | 0 | Not detected | -0.005 | -0.096 | 0.085 | 0.906 |
| Succinatimonas sp. CAG:777 | 0 | Not detected | 0.050 | -0.299 | 0.399 | 0.777 |
| Succinispira mobilis | 0 | Not detected | -0.061 | -0.264 | 0.142 | 0.555 |
| Succinivibrio sp. Phil9 | 0 | Not detected | -0.135 | -0.470 | 0.199 | 0.428 |
| Sulfolobus acidocaldarius | 0 | Not detected | -0.050 | -0.303 | 0.204 | 0.701 |
| Sutterella parvirubra | 0 | Not detected | -0.180 | -0.419 | 0.060 | 0.142 |
| Sutterella sp. CAG:397 | 0 | Not detected | 0.439 | -0.026 | 0.904 | 0.064 |
| Sutterella sp. CAG:521 | 0 | Not detected | -0.045 | -0.319 | 0.229 | 0.748 |
| Sutterella wadsworthensis | 0 | Not detected | -0.134 | -0.539 | 0.270 | 0.515 |
| Sutterella wadsworthensis CAG:135 | 0 | Not detected | -0.056 | -0.548 | 0.435 | 0.822 |
| Sutterellaceae bacterium ND3 | 0 | Not detected | -0.203 | -0.505 | 0.099 | 0.188 |
| Synergistales bacterium Syner_02 | 0 | Not detected | 0.065 | -0.164 | 0.294 | 0.579 |
| Synergistes sp. 3_1_syn1 | 0 | Not detected | -0.158 | -0.458 | 0.143 | 0.304 |

| Synergistes sp. Zagget9 | 0 | Not detected | -0.069 | -0.270 | 0.131 | 0.499 |
| --- | --- | --- | --- | --- | --- | --- |
| Tannerella forsythia | 0 | Not detected | 0.060 | -0.094 | 0.214 | 0.448 |
| Tatumella morbirosei | 0 | Not detected | 0.123 | -0.052 | 0.297 | 0.170 |
| Tepidanaerobacter sp. EBM-49 | 0 | Not detected | 0.002 | -0.284 | 0.287 | 0.990 |
| Terrisporobacter glycolicus | 0 | Not detected | -0.028 | -0.257 | 0.201 | 0.811 |
| Thermophagus xiamenensis | 0 | Not detected | 0.096 | -0.108 | 0.299 | 0.357 |
| Thermosinus carboxydivorans | 0 | Not detected | -0.111 | -0.269 | 0.047 | 0.168 |
| Tidjanibacter massiliensis | 0 | Not detected | -0.270 | -0.588 | 0.047 | 0.096 |
| Tissierellia bacterium S7-1-4 | 0 | Not detected | -0.102 | -0.306 | 0.102 | 0.325 |
| Trabulsiella odontotermitis | 0 | Not detected | 0.246 | -0.031 | 0.524 | 0.082 |
| Traorella massiliensis | 0 | Not detected | -0.014 | -0.104 | 0.075 | 0.755 |
| Treponema denticola | 0 | Not detected | 0.054 | -0.100 | 0.207 | 0.494 |
| Treponema medium | 0 | Not detected | 0.006 | -0.259 | 0.271 | 0.965 |
| Treponema putidum | 0 | Not detected | 0.015 | -0.124 | 0.154 | 0.831 |
| Treponema socranskii | 0 | Not detected | -0.066 | -0.225 | 0.093 | 0.414 |
| Treponema succinifaciens | 0 | Not detected | -0.064 | -0.171 | 0.042 | 0.236 |
| Treponema vincentii | 0 | Not detected | -0.024 | -0.168 | 0.120 | 0.744 |
| Trichococcus flocculiformis | 0 | Not detected | -0.042 | -0.212 | 0.128 | 0.627 |
| Trueperella pyogenes | 1 | Not detected | -0.120 | -0.302 | 0.063 | 0.200 |
| Tumebacillus flagellatus | 0 | Not detected | 0.110 | -0.069 | 0.289 | 0.229 |
| Turicibacter sanguinis | 0 | Not detected | -0.177 | -0.501 | 0.146 | 0.283 |
| Turicibacter sp. H121 | 0 | Not detected | -0.070 | -0.207 | 0.067 | 0.316 |
| Tyzzerella nexilis | 0 | Not detected | -0.008 | -0.088 | 0.073 | 0.850 |
| Urmitella timonensis | 0 | Not detected | -0.077 | -0.213 | 0.060 | 0.272 |
| Vaginella massiliensis | 0 | Not detected | -0.171 | -0.439 | 0.097 | 0.212 |
| Varibaculum cambriense | 0 | Not detected | -0.089 | -0.207 | 0.029 | 0.140 |
| Varibaculum sp. Marseille-P2802 | 0 | Not detected | -0.138 | -0.321 | 0.045 | 0.140 |
| Veillonella magna | 0 | Not detected | -0.023 | -0.109 | 0.063 | 0.606 |
| Veillonella parvula | 0 | Not detected | 0.235 | -0.052 | 0.522 | 0.109 |
| Veillonella seminalis | 0 | Not detected | -0.064 | -0.180 | 0.053 | 0.282 |
| Veillonella sp. ACP1 | 0 | Not detected | 0.297 | -0.039 | 0.632 | 0.084 |
| Veillonella sp. CAG:933 | 0 | Not detected | 0.069 | -0.111 | 0.250 | 0.450 |
| Veillonella sp. oral taxon 780 | 0 | Not detected | 0.109 | -0.086 | 0.303 | 0.274 |
| Waddlia chondrophila | 0 | Not detected | 0.081 | -0.163 | 0.325 | 0.516 |
| Weissella cibaria | 0 | Not detected | -0.002 | -0.259 | 0.254 | 0.985 |
| Weissella confusa | 0 | Not detected | -0.239 | -0.525 | 0.046 | 0.101 |
| Xanthomonas sp. Mitacek01 | 0 | Not detected | -0.096 | -0.233 | 0.041 | 0.169 |
| Yersinia enterocolitica | 0 | Not detected | 0.165 | -0.075 | 0.406 | 0.179 |
| Yersinia pestis | 0 | Not detected | 0.206 | -0.103 | 0.515 | 0.191 |
| Yokenella regensburgei | 0 | Not detected | 0.238 | -0.036 | 0.512 | 0.089 |
| [Bacteroides] pectinophilus | 0 | Not detected | 0.013 | -0.083 | 0.109 | 0.793 |
| [Clostridium] aminophilum | 0 | Not detected | -0.003 | -0.090 | 0.084 | 0.949 |
| [Clostridium] asparagiforme | 0 | Not detected | -0.015 | -0.140 | 0.110 | 0.814 |
| [Clostridium] bolteae | 0 | Not detected | 0.020 | -0.096 | 0.136 | 0.738 |

| [Clostridium] celerecrescens | 0 | Not detected | 0.013 | -0.068 | 0.094 | 0.750 |
| --- | --- | --- | --- | --- | --- | --- |
| [Clostridium] cellobioparum | 0 | Not detected | 0.038 | -0.116 | 0.191 | 0.631 |
| [Clostridium] cellulolyticum | 0 | Not detected | 0.041 | -0.144 | 0.226 | 0.664 |
| [Clostridium] cellulosi | 0 | Not detected | -0.231 | -0.408 | -0.055 | 0.010 |
| [Clostridium] citroniae | 0 | Not detected | -0.015 | -0.111 | 0.081 | 0.765 |
| [Clostridium] clariflavum | 0 | Not detected | -0.154 | -0.297 | -0.012 | 0.033 |
| [Clostridium] clostridioforme | 0 | Not detected | -0.013 | -0.144 | 0.117 | 0.844 |
| [Clostridium] dakarense | 0 | Not detected | -0.039 | -0.279 | 0.200 | 0.748 |
| [Clostridium] glycyrrhizinilyticum | 0 | Not detected | 0.029 | -0.055 | 0.113 | 0.498 |
| [Clostridium] hiranonis | 0 | Not detected | -0.004 | -0.224 | 0.215 | 0.971 |
| [Clostridium] hylemonae | 0 | Not detected | -0.101 | -0.273 | 0.071 | 0.250 |
| [Clostridium] innocuum | 0 | Not detected | -0.009 | -0.091 | 0.074 | 0.838 |
| [Clostridium] josui | 0 | Not detected | 0.105 | -0.088 | 0.298 | 0.289 |
| [Clostridium] lactatifermentans | 0 | Not detected | -0.042 | -0.150 | 0.065 | 0.438 |
| [Clostridium] leptum | 0 | Not detected | -0.033 | -0.174 | 0.107 | 0.641 |
| [Clostridium] methylpentosum | 0 | Not detected | -0.035 | -0.171 | 0.102 | 0.618 |
| [Clostridium] papyrosolvens | 0 | Not detected | -0.068 | -0.195 | 0.060 | 0.298 |
| [Clostridium] saccharogumia | 0 | Not detected | 0.001 | -0.123 | 0.125 | 0.985 |
| [Clostridium] saccharolyticum | 0 | Not detected | 0.002 | -0.113 | 0.116 | 0.976 |
| [Clostridium] scindens | 0 | Not detected | -0.049 | -0.151 | 0.054 | 0.351 |
| [Clostridium] spiroforme | 0 | Not detected | -0.032 | -0.255 | 0.190 | 0.776 |
| [Clostridium] sporosphaeroides | 0 | Not detected | -0.046 | -0.216 | 0.124 | 0.593 |
| [Clostridium] symbiosum | 0 | Not detected | -0.007 | -0.110 | 0.096 | 0.893 |
| [Clostridium] viride | 0 | Not detected | -0.118 | -0.277 | 0.040 | 0.144 |
| [Enterobacter] aerogenes | 0 | Not detected | 0.294 | -0.071 | 0.658 | 0.115 |
| [Eubacterium] brachy | 0 | Not detected | 0.035 | -0.194 | 0.264 | 0.764 |
| [Eubacterium] cellulosolvens | 0 | Not detected | 0.075 | -0.031 | 0.182 | 0.167 |
| [Eubacterium] contortum | 0 | Not detected | -0.046 | -0.122 | 0.029 | 0.231 |
| [Eubacterium] dolichum | 0 | Not detected | 0.012 | -0.068 | 0.092 | 0.765 |
| [Eubacterium] eligens | 0 | Not detected | 0.113 | -0.090 | 0.317 | 0.275 |
| [Eubacterium] fissicatena | 0 | Not detected | 0.023 | -0.079 | 0.125 | 0.656 |
| [Eubacterium] hallii | 0 | Not detected | -0.076 | -0.215 | 0.063 | 0.284 |
| [Eubacterium] nodatum | 0 | Not detected | -0.055 | -0.259 | 0.149 | 0.597 |
| [Eubacterium] rectale | 0 | Not detected | -0.021 | -0.216 | 0.175 | 0.835 |
| [Eubacterium] siraeum | 0 | Not detected | -0.160 | -0.459 | 0.139 | 0.295 |
| [Eubacterium] sulci | 0 | Not detected | -0.026 | -0.250 | 0.199 | 0.823 |
| [Eubacterium] yurii | 0 | Not detected | -0.105 | -0.230 | 0.021 | 0.103 |
| [Haemophilus] ducreyi | 0 | Not detected | 0.148 | -0.015 | 0.312 | 0.076 |
| [Haemophilus] parasuis | 26 | Not detected | 0.231 | 0.033 | 0.428 | 0.022 |
| [Ruminococcus] gnavus | 0 | Not detected | 0.102 | -0.055 | 0.258 | 0.205 |
| [Ruminococcus] torques | 0 | Not detected | -0.035 | -0.199 | 0.130 | 0.681 |
| bacterium F082 | 0 | Not detected | 0.094 | -0.030 | 0.218 | 0.137 |
| bacterium LF-3 | 0 | Not detected | 0.000 | -0.202 | 0.203 | 0.997 |
| bacterium MS4 | 0 | Not detected | -0.056 | -0.221 | 0.108 | 0.504 |

| bacterium OL-1 | 0 | Not detected | 0.074 | -0.211 | 0.359 | 0.610 |
| --- | --- | --- | --- | --- | --- | --- |
| bacterium P201 | 0 | Not detected | 0.105 | -0.092 | 0.302 | 0.297 |
| bacterium P3 | 1 | Not detected | 0.161 | -0.042 | 0.364 | 0.120 |

| **Supplementary Table 10.** Association of childhood economic hardship with functional modules. Analysis of Compositions of Microbiomes (ANCOM2) was used to identify functional modules related to childhood economic hardship . Regression coefficients were obtained by modeling the effect of childhood economic hardship on centered log ratio (CLR)-transformed species in multivariable linear regression adjusted for age, gender, BMI, field center, Hispanic/Latino background, U.S. nativity, Bristol stool type, current SEP, the alternative Healthy Eating Index 2010 score, cigarette smoking, alcohol use, depressive and anxiety symptoms, diabetes, hypertension, cardiovascular disease, dyslipidemia, and antibiotic use. | | | | | | | |
| --- | --- | --- | --- | --- | --- | --- | --- |
| **otu.names** | **pathname** | **W_stat** | **ANCOM** | **beta** | **l95ci** | **u95ci** | **qval** |
| PWY.6823 | molybdenum cofactor biosynthes | 496 | 0.7 | 0.373 | 0.130 | 0.617 | 0.003 |
| ADENOSYL | L-methionine salvage from L-ho | 0 | Not detected | 0.024 | -0.070 | 0.118 | 0.619 |
| ALANINE.V | L-alanine biosynthesis I | 0 | Not detected | 0.132 | -0.077 | 0.341 | 0.217 |
| ANAGLYCO | glycolysis III (from glucose) | 0 | Not detected | -0.054 | -0.138 | 0.030 | 0.205 |
| APYRASE.R | NA | 0 | Not detected | -0.045 | -0.134 | 0.045 | 0.333 |
| ARABCAT.P | L-arabinose degradation I | 0 | Not detected | 0.003 | -0.088 | 0.093 | 0.956 |
| ARGDEG.III | L-arginine degradation IV (argin | 0 | Not detected | 0.040 | -0.095 | 0.174 | 0.564 |
| ARGSYN.PW | L-arginine biosynthesis I (via L-o | 0 | Not detected | -0.069 | -0.200 | 0.061 | 0.300 |
| ARGSYNBS | L-arginine biosynthesis II (acetyl | 0 | Not detected | -0.061 | -0.191 | 0.069 | 0.357 |
| AST.PWY | L-arginine degradation II (AST p | 0 | Not detected | 0.111 | -0.143 | 0.365 | 0.391 |
| BETSYN.PW | glycine betaine biosynthesis I (G | 0 | Not detected | 0.097 | -0.135 | 0.329 | 0.414 |
| CALVIN.PW | Calvin-Benson-Bassham cycle | 0 | Not detected | -0.027 | -0.108 | 0.054 | 0.517 |
| CARBPSYN. | NA | 0 | Not detected | 0.079 | -0.098 | 0.255 | 0.381 |
| CARNMET.P | L-carnitine degradation I | 0 | Not detected | 0.015 | -0.243 | 0.274 | 0.907 |
| CENTFERM. | pyruvate fermentation to butanoa | 0 | Not detected | -0.064 | -0.180 | 0.051 | 0.274 |
| CHLOROPH | 3,8-divinyl-chlorophyllide <i>a< | 0 | Not detected | 0.197 | -0.056 | 0.449 | 0.127 |
| CHOLINE.B | choline degradation I | 0 | Not detected | 0.097 | -0.135 | 0.329 | 0.414 |
| COBALSYN. | superpathway of adenosylcobala | 0 | Not detected | 0.091 | -0.102 | 0.283 | 0.357 |
| CODH.PWY | reductive acetyl coenzyme A pat | 0 | Not detected | -0.085 | -0.234 | 0.064 | 0.263 |
| COLANSYN | colanic acid building blocks bios | 0 | Not detected | -0.146 | -0.310 | 0.019 | 0.082 |
| DAPLYSINE | L-lysine biosynthesis I | 0 | Not detected | 0.036 | -0.214 | 0.287 | 0.776 |
| DARABCAT | D-arabinose degradation I | 0 | Not detected | -0.088 | -0.358 | 0.182 | 0.522 |
| DENITRIFIC | nitrate reduction I (denitrification | 0 | Not detected | 0.102 | -0.129 | 0.332 | 0.386 |
| DETOX1.PW | superoxide radicals degradation | 0 | Not detected | -0.031 | -0.278 | 0.216 | 0.805 |
| DETOX1.PW | reactive oxygen species degradat | 0 | Not detected | 0.020 | -0.234 | 0.275 | 0.877 |
| DHGLUCON | glucose degradation (oxidative) | 0 | Not detected | 0.225 | -0.028 | 0.478 | 0.081 |
| DISSULFRE | dissimilatory sulfate reduction I ( | 0 | Not detected | -0.102 | -0.342 | 0.139 | 0.408 |
| DTDPRHAM | dTDP-L-rhamnose biosynthesis | 0 | Not detected | -0.057 | -0.145 | 0.031 | 0.202 |
| ECASYN.PW | enterobacterial common antigen | 0 | Not detected | 0.272 | 0.004 | 0.539 | 0.047 |
| ETOH.ACET | ethanol degradation I | 0 | Not detected | -0.008 | -0.290 | 0.274 | 0.954 |
| FAO.PWY | fatty acid &beta;-oxidation I | 0 | Not detected | 0.085 | -0.243 | 0.414 | 0.610 |
| FASYN.ELO | fatty acid elongation -- saturated | 0 | Not detected | -0.026 | -0.303 | 0.250 | 0.852 |
| FERMENTA | mixed acid fermentation | 0 | Not detected | -0.029 | -0.113 | 0.054 | 0.487 |
| FHLMULTI. | NA | 0 | Not detected | 0.025 | -0.231 | 0.280 | 0.851 |
| FUCCAT.PW | fucose degradation | 0 | Not detected | -0.088 | -0.358 | 0.182 | 0.522 |
| GALACTAR | D-galactarate degradation I | 0 | Not detected | 0.040 | -0.123 | 0.203 | 0.634 |
| GALACTCA | D-galactonate degradation | 0 | Not detected | -0.088 | -0.356 | 0.179 | 0.518 |
| GALACTUR | D-galacturonate degradation I | 0 | Not detected | -0.005 | -0.091 | 0.081 | 0.909 |

| GCVMULTI. | NA | 0 | Not detected | 0.034 | -0.169 | 0.238 | 0.742 |
| --- | --- | --- | --- | --- | --- | --- | --- |
| GDPRHAMS | GDP-D-rhamnose biosynthesis | 0 | Not detected | 0.082 | -0.190 | 0.354 | 0.555 |
| GLUCARDE | <i>D</i>-glucarate degradation I | 0 | Not detected | -0.025 | -0.192 | 0.142 | 0.770 |
| GLUCONEO | gluconeogenesis I | 0 | Not detected | -0.041 | -0.126 | 0.044 | 0.344 |
| GLUCOSE1P | glucose and glucose-1-phosphate | 0 | Not detected | -0.059 | -0.190 | 0.071 | 0.372 |
| GLUDEG.I.P | GABA shunt | 0 | Not detected | 0.187 | -0.047 | 0.420 | 0.117 |
| GLUTATHIO | glutathione biosynthesis | 0 | Not detected | 0.136 | -0.084 | 0.357 | 0.226 |
| GLUTORN.P | L-ornithine biosynthesis I | 0 | Not detected | 0.001 | -0.244 | 0.247 | 0.991 |
| GLYCLEAV. | glycine cleavage | 0 | Not detected | 0.034 | -0.169 | 0.238 | 0.742 |
| GLYCOCAT | glycogen degradation I | 0 | Not detected | -0.067 | -0.195 | 0.061 | 0.307 |
| GLYCOGEN | glycogen biosynthesis I (from AD | 0 | Not detected | -0.075 | -0.192 | 0.042 | 0.210 |
| GLYCOLAT | glycolate and glyoxylate degrada | 0 | Not detected | 0.112 | -0.141 | 0.365 | 0.386 |
| GLYCOLYSI | glycolysis I (from glucose 6-phos | 0 | Not detected | -0.053 | -0.138 | 0.031 | 0.217 |
| GLYCOLYSI | superpathway of glycolysis and t | 0 | Not detected | 0.219 | -0.012 | 0.449 | 0.064 |
| GLYOXDEG | glycolate and glyoxylate degrada | 0 | Not detected | 0.032 | -0.229 | 0.294 | 0.808 |
| GLYOXYLA | glyoxylate cycle | 0 | Not detected | -0.024 | -0.117 | 0.070 | 0.618 |
| HCAMHPDE | 3-phenylpropanoate and 3-(3-hyd | 326 | Not detected | 0.263 | 0.037 | 0.488 | 0.023 |
| HEME.BIOS | heme <i>b</i> biosynthesis I (ae | 0 | Not detected | 0.197 | -0.058 | 0.452 | 0.130 |
| HEMESYN2. | heme <i>b</i> biosynthesis II (a | 0 | Not detected | 0.206 | -0.056 | 0.468 | 0.124 |
| HISDEG.PW | L-histidine degradation I | 0 | Not detected | 0.035 | -0.185 | 0.254 | 0.757 |
| HISHP.PWY | L-histidine degradation VI | 0 | Not detected | 0.035 | -0.185 | 0.254 | 0.757 |
| HISTSYN.P | L-histidine biosynthesis | 0 | Not detected | -0.037 | -0.128 | 0.054 | 0.422 |
| HOMOSER. | L-methionine biosynthesis I | 0 | Not detected | 0.008 | -0.077 | 0.092 | 0.861 |
| HOMOSER.T | L-threonine biosynthesis | 0 | Not detected | 0.061 | -0.198 | 0.320 | 0.643 |
| HOMOSERS | L-homoserine biosynthesis | 0 | Not detected | -0.008 | -0.254 | 0.238 | 0.949 |
| HSERMETA | L-methionine biosynthesis III | 0 | Not detected | 0.025 | -0.063 | 0.113 | 0.576 |
| ILEUSYN.P | L-isoleucine biosynthesis I (from | 0 | Not detected | -0.079 | -0.188 | 0.029 | 0.154 |
| KDO.LIPAS | (Kdo)<sub>2</sub>-lipid A bios | 0 | Not detected | 0.178 | -0.045 | 0.402 | 0.118 |
| KDO.NAGLI | superpathway of (Kdo)<SUB>2< | 0 | Not detected | 0.166 | -0.089 | 0.421 | 0.203 |
| KDOSYN.PW | Kdo transfer to lipid IV<sub>A< | 0 | Not detected | 0.197 | -0.065 | 0.458 | 0.141 |
| KETOGLUC | ketogluconate metabolism | 0 | Not detected | 0.137 | -0.082 | 0.356 | 0.219 |
| LACTOSEC | lactose and galactose degradation | 0 | Not detected | -0.247 | -0.515 | 0.020 | 0.071 |
| LARABITOL | xylitol degradation | 0 | Not detected | -0.068 | -0.341 | 0.204 | 0.623 |
| LEUSYN.PW | L-leucine biosynthesis | 0 | Not detected | 0.007 | -0.147 | 0.161 | 0.928 |
| LIPA.CORES | lipid A-core biosynthesis (<i>E. | 0 | Not detected | 0.251 | -0.034 | 0.536 | 0.084 |
| LYSDEGII.P | L-lysine degradation III | 268 | Not detected | 0.237 | 0.041 | 0.433 | 0.018 |
| LYXMET.PW | L-lyxose degradation | 0 | Not detected | 0.016 | -0.062 | 0.095 | 0.684 |
| MALATE.AS | L-aspartate degradation II | 0 | Not detected | -0.070 | -0.350 | 0.209 | 0.623 |
| MANNCAT. | D-mannose degradation | 0 | Not detected | -0.023 | -0.252 | 0.206 | 0.842 |
| MANNIDEG | mannitol degradation I | 0 | Not detected | -0.025 | -0.272 | 0.222 | 0.844 |
| MENAQUIN | menaquinol-8 biosynthesis | 0 | Not detected | 0.031 | -0.219 | 0.281 | 0.807 |
| METH.ACET | methanogenesis from acetate | 0 | Not detected | -0.032 | -0.110 | 0.046 | 0.420 |
| NAGLIPASY | lipid IV<sub>A</sub> biosynthe | 0 | Not detected | 0.063 | -0.201 | 0.328 | 0.639 |
| NONMEVIP | methylerythritol phosphate pathw | 0 | Not detected | -0.115 | -0.212 | -0.018 | 0.020 |

| NONOXIPE | pentose phosphate pathway (non | 0 | Not detected | -0.006 | -0.176 | 0.164 | 0.944 |
| --- | --- | --- | --- | --- | --- | --- | --- |
| NPGLUCAT. | Entner-Doudoroff pathway II (no | 0 | Not detected | -0.092 | -0.306 | 0.122 | 0.398 |
| OANTIGEN. | <i>O</i>-antigen building block | 0 | Not detected | -0.055 | -0.163 | 0.054 | 0.326 |
| OXIDATIVE | pentose phosphate pathway (oxid | 0 | Not detected | -0.031 | -0.201 | 0.140 | 0.723 |
| P101.PWY | ectoine biosynthesis | 0 | Not detected | -0.009 | -0.256 | 0.237 | 0.942 |
| P105.PWY | TCA cycle IV (2-oxoglutarate de | 0 | Not detected | -0.039 | -0.127 | 0.050 | 0.395 |
| P108.PWY | pyruvate fermentation to propano | 0 | Not detected | -0.094 | -0.210 | 0.023 | 0.116 |
| P121.PWY | adenine and adenosine salvage I | 0 | Not detected | 0.018 | -0.216 | 0.251 | 0.883 |
| P122.PWY | heterolactic fermentation | 0 | Not detected | -0.022 | -0.099 | 0.055 | 0.574 |
| P124.PWY | Bifidobacterium shunt | 0 | Not detected | -0.045 | -0.121 | 0.032 | 0.255 |
| P161.PWY | acetylene degradation (anaerobic | 0 | Not detected | -0.023 | -0.094 | 0.047 | 0.519 |
| P162.PWY | L-glutamate degradation V (via h | 0 | Not detected | -0.064 | -0.180 | 0.051 | 0.274 |
| P163.PWY | L-lysine fermentation to acetate a | 0 | Not detected | -0.043 | -0.125 | 0.039 | 0.306 |
| P164.PWY | purine nucleobases degradation I | 0 | Not detected | -0.065 | -0.149 | 0.019 | 0.129 |
| P181.PWY | nicotine degradation I (pyridine p | 0 | Not detected | 0.230 | 0.008 | 0.451 | 0.043 |
| P185.PWY | formaldehyde assimilation III (di | 0 | Not detected | -0.027 | -0.108 | 0.054 | 0.512 |
| P2.PWY | citrate lyase activation | 0 | Not detected | 0.179 | -0.048 | 0.406 | 0.122 |
| P21.PWY | pentose phosphate pathway (part | 0 | Not detected | -0.006 | -0.176 | 0.164 | 0.944 |
| P23.PWY | reductive TCA cycle I | 0 | Not detected | -0.044 | -0.145 | 0.056 | 0.387 |
| P3.PWY | gallate degradation III (anaerobic | 0 | Not detected | -0.038 | -0.120 | 0.043 | 0.354 |
| P341.PWY | glycolysis V (Pyrococcus) | 0 | Not detected | -0.072 | -0.160 | 0.016 | 0.112 |
| P42.PWY | incomplete reductive TCA cycle | 0 | Not detected | -0.109 | -0.230 | 0.013 | 0.080 |
| P562.PWY | <I>myo</I>-inositol degradation | 0 | Not detected | -0.147 | -0.317 | 0.024 | 0.092 |
| PEPTIDOGL | peptidoglycan biosynthesis I (<I> | 0 | Not detected | 0.147 | -0.099 | 0.394 | 0.240 |
| PHOSLIPSY | superpathway of phospholipid bi | 0 | Not detected | 0.110 | -0.122 | 0.342 | 0.353 |
| PHOSPHON | 2-aminoethylphosphonate degrad | 0 | Not detected | 0.008 | -0.260 | 0.276 | 0.952 |
| PPGPPMET. | ppGpp biosynthesis | 0 | Not detected | -0.016 | -0.098 | 0.066 | 0.707 |
| PUTDEG.PW | putrescine degradation I | 0 | Not detected | 0.120 | -0.079 | 0.318 | 0.238 |
| PWY.1042 | glycolysis IV (plant cytosol) | 0 | Not detected | -0.057 | -0.140 | 0.027 | 0.181 |
| PWY.1186 | L-homomethionine biosynthesis | 0 | Not detected | -0.089 | -0.332 | 0.153 | 0.470 |
| PWY.1269 | CMP-3-deoxy-D-<I>manno</I>- | 0 | Not detected | 0.031 | -0.222 | 0.283 | 0.811 |
| PWY.1281 | sulfoacetaldehyde degradation I | 0 | Not detected | -0.029 | -0.129 | 0.071 | 0.564 |
| PWY.1341 | phenylacetate degradation II (ana | 0 | Not detected | -0.133 | -0.277 | 0.011 | 0.071 |
| PWY.1361 | benzoyl-CoA degradation I (aero | 0 | Not detected | 0.199 | -0.035 | 0.433 | 0.096 |
| PWY.1622 | formaldehyde assimilation I (seri | 0 | Not detected | -0.078 | -0.251 | 0.096 | 0.382 |
| PWY.1722 | formate assimilation into 5,10-m | 0 | Not detected | -0.085 | -0.234 | 0.064 | 0.263 |
| PWY.1861 | formaldehyde assimilation II (ass | 0 | Not detected | -0.114 | -0.213 | -0.015 | 0.024 |
| PWY.2 | putrescine degradation IV | 0 | Not detected | 0.120 | -0.079 | 0.318 | 0.238 |
| PWY.2161 | folate polyglutamylation | 0 | Not detected | 0.242 | -0.025 | 0.508 | 0.076 |
| PWY.2201 | folate transformations I | 0 | Not detected | -0.013 | -0.103 | 0.076 | 0.769 |
| PWY.2221 | Entner-Doudoroff pathway III (s | 0 | Not detected | -0.054 | -0.136 | 0.028 | 0.195 |
| PWY.2361 | 3-oxoadipate degradation | 0 | Not detected | 0.122 | -0.072 | 0.317 | 0.218 |
| PWY.2661 | trehalose biosynthesis V | 0 | Not detected | 0.115 | -0.164 | 0.395 | 0.418 |
| PWY.2722 | trehalose degradation IV | 0 | Not detected | -0.075 | -0.293 | 0.143 | 0.499 |

| PWY.2723 | trehalose degradation V | 0 | Not detected | -0.104 | -0.243 | 0.035 | 0.142 |
| --- | --- | --- | --- | --- | --- | --- | --- |
| PWY.2781 | <i>cis</i>-zeatin biosynthesis | 0 | Not detected | 0.022 | -0.211 | 0.254 | 0.855 |
| PWY.2941 | L-lysine biosynthesis II | 0 | Not detected | -0.008 | -0.254 | 0.238 | 0.949 |
| PWY.2942 | L-lysine biosynthesis III | 0 | Not detected | -0.008 | -0.254 | 0.238 | 0.949 |
| PWY.3 | putrescine degradation V | 0 | Not detected | 0.134 | -0.066 | 0.334 | 0.190 |
| PWY.3801 | sucrose degradation II (sucrose s | 0 | Not detected | -0.054 | -0.132 | 0.025 | 0.183 |
| PWY.3821 | D-galactose detoxification | 0 | Not detected | -0.087 | -0.219 | 0.044 | 0.193 |
| PWY.3841 | folate transformations II | 0 | Not detected | -0.011 | -0.099 | 0.077 | 0.813 |
| PWY.3861 | mannitol degradation II | 0 | Not detected | 0.055 | -0.191 | 0.300 | 0.662 |
| PWY.3881 | mannitol biosynthesis | 0 | Not detected | -0.058 | -0.304 | 0.187 | 0.643 |
| PWY.3941 | &beta;-alanine biosynthesis II | 0 | Not detected | 0.128 | -0.138 | 0.393 | 0.345 |
| PWY.4041 | &gamma;-glutamyl cycle | 0 | Not detected | 0.238 | -0.019 | 0.496 | 0.070 |
| PWY.4101 | D-sorbitol degradation I | 0 | Not detected | 0.098 | -0.149 | 0.345 | 0.435 |
| PWY.43 | putrescine biosynthesis II | 0 | Not detected | 0.040 | -0.095 | 0.174 | 0.564 |
| PWY.4321 | L-glutamate degradation IV | 0 | Not detected | 0.187 | -0.045 | 0.420 | 0.115 |
| PWY.4361 | <i>S</i>-methyl-5-thio-&alpha;- | 0 | Not detected | -0.046 | -0.299 | 0.207 | 0.722 |
| PWY.4381 | fatty acid biosynthesis initiation I | 0 | Not detected | -0.148 | -0.386 | 0.091 | 0.226 |
| PWY.4702 | phytate degradation I | 0 | Not detected | -0.012 | -0.264 | 0.239 | 0.923 |
| PWY.4983 | nitric oxide biosynthesis II (mam | 0 | Not detected | -0.069 | -0.199 | 0.062 | 0.302 |
| PWY.4984 | urea cycle | 0 | Not detected | 0.038 | -0.079 | 0.155 | 0.527 |
| PWY.5022 | 4-aminobutanoate degradation V | 0 | Not detected | -0.025 | -0.132 | 0.081 | 0.641 |
| PWY.5028 | L-histidine degradation II | 0 | Not detected | 0.043 | -0.178 | 0.263 | 0.704 |
| PWY.5030 | L-histidine degradation III | 0 | Not detected | -0.097 | -0.237 | 0.044 | 0.178 |
| PWY.5041 | <i>S</i>-adenosyl-L-methionine | 0 | Not detected | 0.112 | -0.148 | 0.373 | 0.398 |
| PWY.5049 | rosmarinic acid biosynthesis II | 0 | Not detected | 0.040 | -0.154 | 0.234 | 0.688 |
| PWY.5054 | D-sorbitol biosynthesis I | 0 | Not detected | -0.044 | -0.134 | 0.045 | 0.331 |
| PWY.5097 | L-lysine biosynthesis VI | 0 | Not detected | -0.020 | -0.267 | 0.226 | 0.871 |
| PWY.5101 | L-isoleucine biosynthesis II | 0 | Not detected | -0.151 | -0.396 | 0.094 | 0.226 |
| PWY.5103 | L-isoleucine biosynthesis III | 0 | Not detected | -0.079 | -0.188 | 0.029 | 0.154 |
| PWY.5104 | L-isoleucine biosynthesis IV | 0 | Not detected | 0.039 | -0.228 | 0.306 | 0.775 |
| PWY.5122 | geranyl diphosphate biosynthesis | 0 | Not detected | -0.059 | -0.283 | 0.166 | 0.609 |
| PWY.5123 | <i>trans, trans</i>-farnesyl diph | 0 | Not detected | 0.002 | -0.266 | 0.270 | 0.989 |
| PWY.5136 | fatty acid &beta;-oxidation II (pe | 0 | Not detected | 0.024 | -0.278 | 0.326 | 0.877 |
| PWY.5137 | fatty acid &beta;-oxidation III (u | 0 | Not detected | 0.093 | -0.143 | 0.329 | 0.440 |
| PWY.5138 | unsaturated, even numbered fatty | 0 | Not detected | 0.095 | -0.180 | 0.370 | 0.498 |
| PWY.5154 | L-arginine biosynthesis III (via < | 0 | Not detected | -0.061 | -0.190 | 0.069 | 0.360 |
| PWY.5162 | 2-hydroxypenta-2,4-dienoate deg | 0 | Not detected | 0.023 | -0.244 | 0.291 | 0.866 |
| PWY.5177 | glutaryl-CoA degradation | 0 | Not detected | -0.153 | -0.306 | -0.001 | 0.049 |
| PWY.5188 | tetrapyrrole biosynthesis I (from | 0 | Not detected | -0.009 | -0.131 | 0.112 | 0.878 |
| PWY.5189 | tetrapyrrole biosynthesis II (from | 0 | Not detected | 0.045 | -0.128 | 0.218 | 0.610 |
| PWY.5265 | peptidoglycan biosynthesis II (sta | 0 | Not detected | -0.066 | -0.218 | 0.085 | 0.393 |
| PWY.5269 | cardiolipin biosynthesis II | 0 | Not detected | 0.000 | -0.234 | 0.234 | 0.999 |
| PWY.5280 | L-lysine degradation IV | 258 | Not detected | 0.238 | 0.039 | 0.436 | 0.019 |
| PWY.5306 | superpathway of thiosulfate meta | 0 | Not detected | -0.102 | -0.342 | 0.139 | 0.408 |

| PWY.5316 | nicotine biosynthesis | 0 | Not detected | -0.134 | -0.429 | 0.162 | 0.377 |
| --- | --- | --- | --- | --- | --- | --- | --- |
| PWY.5344 | L-homocysteine biosynthesis | 0 | Not detected | -0.052 | -0.150 | 0.045 | 0.295 |
| PWY.5352 | thiosulfate disproportionation II ( | 0 | Not detected | -0.240 | -0.468 | -0.011 | 0.040 |
| PWY.5360 | superpathway of tetrathionate red | 0 | Not detected | -0.102 | -0.342 | 0.139 | 0.408 |
| PWY.5381 | pyridine nucleotide cycling (plan | 0 | Not detected | 0.243 | -0.004 | 0.489 | 0.054 |
| PWY.5384 | sucrose degradation IV (sucrose | 0 | Not detected | -0.041 | -0.120 | 0.038 | 0.307 |
| PWY.5386 | methylglyoxal degradation I | 0 | Not detected | 0.146 | -0.106 | 0.398 | 0.257 |
| PWY.5392 | reductive TCA cycle II | 0 | Not detected | -0.042 | -0.144 | 0.061 | 0.426 |
| PWY.5394 | betalamic acid biosynthesis | 0 | Not detected | 0.082 | -0.095 | 0.260 | 0.364 |
| PWY.5436 | L-threonine degradation IV | 0 | Not detected | 0.008 | -0.260 | 0.276 | 0.952 |
| PWY.5437 | L-threonine degradation I | 0 | Not detected | 0.079 | -0.183 | 0.341 | 0.555 |
| PWY.5441 | S-methyl-L-methionine cycle | 0 | Not detected | 0.188 | -0.001 | 0.377 | 0.051 |
| PWY.5453 | methylglyoxal degradation III | 0 | Not detected | -0.031 | -0.113 | 0.051 | 0.457 |
| PWY.5458 | methylglyoxal degradation V | 0 | Not detected | -0.059 | -0.303 | 0.185 | 0.637 |
| PWY.5459 | methylglyoxal degradation IV | 0 | Not detected | -0.054 | -0.299 | 0.191 | 0.665 |
| PWY.5480 | pyruvate fermentation to ethanol | 0 | Not detected | -0.008 | -0.290 | 0.274 | 0.954 |
| PWY.5482 | pyruvate fermentation to acetate | 0 | Not detected | -0.032 | -0.110 | 0.046 | 0.420 |
| PWY.5484 | glycolysis II (from fructose 6-pho | 0 | Not detected | -0.055 | -0.137 | 0.027 | 0.189 |
| PWY.5485 | pyruvate fermentation to acetate | 0 | Not detected | -0.032 | -0.110 | 0.046 | 0.420 |
| PWY.5486 | pyruvate fermentation to ethanol | 0 | Not detected | -0.109 | -0.335 | 0.117 | 0.345 |
| PWY.5497 | purine nucleobases degradation I | 0 | Not detected | -0.042 | -0.124 | 0.040 | 0.315 |
| PWY.5509 | adenosylcobalamin biosynthesis | 0 | Not detected | 0.091 | -0.102 | 0.283 | 0.357 |
| PWY.5512 | UDP-<i>N</i>-acetyl-D-galacto | 0 | Not detected | 0.002 | -0.317 | 0.320 | 0.992 |
| PWY.5514 | UDP-<i>N</i>-acetyl-D-galacto | 0 | Not detected | -0.046 | -0.138 | 0.046 | 0.325 |
| PWY.5523 | 5,6-dimethylbenzimidazole biosy | 0 | Not detected | 0.230 | 0.002 | 0.458 | 0.049 |
| PWY.5531 | 3,8-divinyl-chlorophyllide <i>a< | 0 | Not detected | 0.232 | -0.028 | 0.492 | 0.081 |
| PWY.5532 | nucleoside and nucleotide degrad | 0 | Not detected | 0.089 | -0.103 | 0.282 | 0.364 |
| PWY.5536 | acetate formation from acetyl-Co | 0 | Not detected | -0.197 | -0.369 | -0.024 | 0.025 |
| PWY.561 | superpathway of glyoxylate cycle | 0 | Not detected | -0.025 | -0.112 | 0.063 | 0.581 |
| PWY.5659 | GDP-mannose biosynthesis | 0 | Not detected | -0.047 | -0.129 | 0.036 | 0.265 |
| PWY.5661 | GDP-glucose biosynthesis | 0 | Not detected | -0.104 | -0.243 | 0.035 | 0.142 |
| PWY.5663 | erythro-tetrahydrobiopterin biosy | 0 | Not detected | -0.132 | -0.289 | 0.025 | 0.099 |
| PWY.5664 | <i>erythro</i>-tetrahydrobiopter | 0 | Not detected | -0.132 | -0.289 | 0.025 | 0.099 |
| PWY.5667 | CDP-diacylglycerol biosynthesis | 0 | Not detected | -0.066 | -0.269 | 0.138 | 0.527 |
| PWY.5668 | cardiolipin biosynthesis I | 0 | Not detected | 0.055 | -0.217 | 0.327 | 0.691 |
| PWY.5669 | phosphatidylserine and phosphat | 0 | Not detected | 0.110 | -0.122 | 0.342 | 0.353 |
| PWY.5676 | acetyl-CoA fermentation to butan | 0 | Not detected | -0.053 | -0.158 | 0.051 | 0.319 |
| PWY.5677 | succinate fermentation to butano | 0 | Not detected | -0.044 | -0.159 | 0.070 | 0.445 |
| PWY.5686 | UMP biosynthesis I | 0 | Not detected | -0.035 | -0.125 | 0.055 | 0.445 |
| PWY.5690 | TCA cycle II (plants and fungi) | 0 | Not detected | -0.041 | -0.144 | 0.061 | 0.432 |
| PWY.5692 | allantoin degradation to glyoxyla | 0 | Not detected | -0.125 | -0.345 | 0.096 | 0.268 |
| PWY.5698 | allantoin degradation to ureidogl | 0 | Not detected | -0.125 | -0.345 | 0.096 | 0.268 |
| PWY.5723 | Rubisco shunt | 0 | Not detected | -0.075 | -0.167 | 0.018 | 0.115 |
| PWY.5738 | GDP-6-deoxy-D-talose biosynthe | 0 | Not detected | 0.082 | -0.190 | 0.354 | 0.555 |

| PWY.5739 | GDP-D-perosamine biosynthesis | 0 | Not detected | 0.082 | -0.190 | 0.354 | 0.555 |
| --- | --- | --- | --- | --- | --- | --- | --- |
| PWY.5740 | GDP-L-colitose biosynthesis | 0 | Not detected | 0.082 | -0.190 | 0.354 | 0.555 |
| PWY.5741 | ethylmalonyl-CoA pathway | 0 | Not detected | 0.158 | -0.059 | 0.375 | 0.154 |
| PWY.5743 | 3-hydroxypropanoate cycle | 0 | Not detected | -0.033 | -0.152 | 0.085 | 0.581 |
| PWY.5744 | glyoxylate assimilation | 0 | Not detected | -0.034 | -0.153 | 0.084 | 0.570 |
| PWY.5747 | 2-methylcitrate cycle II | 0 | Not detected | 0.058 | -0.229 | 0.344 | 0.694 |
| PWY.5757 | fosfomycin biosynthesis | 0 | Not detected | -0.364 | -0.692 | -0.037 | 0.030 |
| PWY.5785 | di-<i>trans</i>,poly-<i>cis</i>-u | 0 | Not detected | 0.012 | -0.213 | 0.237 | 0.919 |
| PWY.5789 | 3-hydroxypropanoate/4-hydroxy | 0 | Not detected | -0.057 | -0.158 | 0.044 | 0.270 |
| PWY.5794 | malonate degradation I (biotin-in | 0 | Not detected | -0.032 | -0.271 | 0.208 | 0.795 |
| PWY.5796 | malonate decarboxylase activatio | 0 | Not detected | 0.162 | -0.031 | 0.355 | 0.100 |
| PWY.5837 | 2-carboxy-1,4-naphthoquinol bio | 0 | Not detected | 0.025 | -0.070 | 0.121 | 0.602 |
| PWY.5852 | demethylmenaquinol-8 biosynthe | 0 | Not detected | 0.202 | -0.006 | 0.410 | 0.057 |
| PWY.5870 | ubiquinol-8 biosynthesis (eukary | 0 | Not detected | 0.096 | -0.175 | 0.368 | 0.487 |
| PWY.5875 | staphyloxanthin biosynthesis | 0 | Not detected | -0.028 | -0.297 | 0.240 | 0.836 |
| PWY.5901 | 2,3-dihydroxybenzoate biosynthe | 0 | Not detected | 0.099 | -0.094 | 0.293 | 0.316 |
| PWY.5913 | partial TCA cycle (obligate autot | 0 | Not detected | -0.044 | -0.144 | 0.057 | 0.393 |
| PWY.5921 | glutaminyl-tRNA<sup>gln</sup | 0 | Not detected | 0.147 | -0.133 | 0.427 | 0.304 |
| PWY.5940 | streptomycin biosynthesis | 0 | Not detected | -0.108 | -0.245 | 0.028 | 0.121 |
| PWY.5941 | glycogen degradation II | 0 | Not detected | -0.104 | -0.243 | 0.035 | 0.142 |
| PWY.5971 | palmitate biosynthesis II (bacteri | 0 | Not detected | -0.060 | -0.236 | 0.117 | 0.508 |
| PWY.5981 | CDP-diacylglycerol biosynthesis | 0 | Not detected | -0.115 | -0.315 | 0.085 | 0.260 |
| PWY.5994 | palmitate biosynthesis I (animals | 0 | Not detected | -0.047 | -0.239 | 0.146 | 0.635 |
| PWY.6012 | acyl carrier protein metabolism | 0 | Not detected | 0.081 | -0.107 | 0.269 | 0.399 |
| PWY.6019 | pseudouridine degradation | 0 | Not detected | -0.207 | -0.427 | 0.012 | 0.065 |
| PWY.6053 | dimethylsulfoniopropanoate bios | 0 | Not detected | -0.089 | -0.332 | 0.153 | 0.470 |
| PWY.6060 | malonate degradation II (biotin-d | 0 | Not detected | -0.176 | -0.331 | -0.022 | 0.025 |
| PWY.6121 | 5-aminoimidazole ribonucleotide | 0 | Not detected | 0.093 | -0.011 | 0.198 | 0.080 |
| PWY.6122 | 5-aminoimidazole ribonucleotide | 0 | Not detected | 0.034 | -0.046 | 0.114 | 0.407 |
| PWY.6123 | inosine-5'-phosphate biosynthesi | 0 | Not detected | -0.060 | -0.217 | 0.097 | 0.451 |
| PWY.6124 | inosine-5'-phosphate biosynthesi | 0 | Not detected | -0.060 | -0.217 | 0.097 | 0.451 |
| PWY.6138 | CMP-<i>N</i>-acetylneuramina | 0 | Not detected | 0.006 | -0.202 | 0.215 | 0.952 |
| PWY.6140 | CMP-2-keto-3-deoxy-D-<i>glyc | 0 | Not detected | -0.072 | -0.362 | 0.217 | 0.625 |
| PWY.6142 | gluconeogenesis II (<i>Methano | 0 | Not detected | -0.070 | -0.159 | 0.018 | 0.117 |
| PWY.6143 | CMP-pseudaminate biosynthesis | 0 | Not detected | -0.223 | -0.432 | -0.013 | 0.037 |
| PWY.6146 | <i>Methanobacterium thermoaut | 0 | Not detected | 0.110 | -0.149 | 0.369 | 0.405 |
| PWY.6147 | 6-hydroxymethyl-dihydropterin d | 0 | Not detected | -0.081 | -0.214 | 0.052 | 0.234 |
| PWY.6148 | tetrahydromethanopterin biosynt | 0 | Not detected | 0.063 | -0.134 | 0.259 | 0.531 |
| PWY.6151 | <i>S</i>-adenosyl-L-methionine | 0 | Not detected | 0.086 | -0.185 | 0.357 | 0.533 |
| PWY.6153 | autoinducer AI-2 biosynthesis I | 0 | Not detected | 0.071 | -0.126 | 0.267 | 0.482 |
| PWY.6154 | autoinducer AI-2 biosynthesis II | 0 | Not detected | 0.071 | -0.126 | 0.267 | 0.482 |
| PWY.6160 | 3-dehydroquinate biosynthesis II | 0 | Not detected | -0.042 | -0.289 | 0.205 | 0.740 |
| PWY.6163 | chorismate biosynthesis from 3-d | 0 | Not detected | -0.014 | -0.207 | 0.180 | 0.890 |
| PWY.6164 | 3-dehydroquinate biosynthesis I | 0 | Not detected | 0.167 | -0.072 | 0.406 | 0.170 |

| PWY.6167 | flavin biosynthesis II (archaea) | 0 | Not detected | -0.001 | -0.085 | 0.084 | 0.984 |
| --- | --- | --- | --- | --- | --- | --- | --- |
| PWY.6168 | flavin biosynthesis III (fungi) | 0 | Not detected | -0.005 | -0.093 | 0.083 | 0.909 |
| PWY.6174 | mevalonate pathway II (archaea) | 0 | Not detected | 0.061 | -0.118 | 0.240 | 0.505 |
| PWY.621 | sucrose degradation III (sucrose i | 0 | Not detected | -0.043 | -0.126 | 0.041 | 0.315 |
| PWY.622 | starch biosynthesis | 0 | Not detected | -0.044 | -0.121 | 0.034 | 0.272 |
| PWY.6269 | superpathway of adenosylcobala | 0 | Not detected | 0.232 | 0.017 | 0.446 | 0.034 |
| PWY.6277 | superpathway of 5-aminoimidazo | 0 | Not detected | 0.034 | -0.046 | 0.114 | 0.407 |
| PWY.6317 | D-galactose degradation I (Leloir | 0 | Not detected | -0.038 | -0.121 | 0.045 | 0.366 |
| PWY.6318 | L-phenylalanine degradation IV ( | 0 | Not detected | -0.133 | -0.277 | 0.011 | 0.071 |
| PWY.6322 | phosphinothricin tripeptide biosy | 0 | Not detected | -0.330 | -0.624 | -0.037 | 0.028 |
| PWY.6328 | L-lysine degradation X | 258 | Not detected | 0.238 | 0.039 | 0.436 | 0.019 |
| PWY.6333 | acetaldehyde biosynthesis I | 0 | Not detected | -0.109 | -0.335 | 0.117 | 0.345 |
| PWY.6383 | mono-<i>trans</i>, poly-<i>cis< | 0 | Not detected | 0.024 | -0.213 | 0.261 | 0.840 |
| PWY.6386 | UDP-<i>N</i>-acetylmuramoyl- | 0 | Not detected | -0.058 | -0.134 | 0.017 | 0.127 |
| PWY.6387 | UDP-<i>N</i>-acetylmuramoyl- | 0 | Not detected | -0.048 | -0.119 | 0.023 | 0.186 |
| PWY.6397 | mycolyl-arabinogalactan-peptido | 0 | Not detected | -0.070 | -0.178 | 0.038 | 0.206 |
| PWY.6406 | salicylate biosynthesis I | 0 | Not detected | 0.099 | -0.094 | 0.293 | 0.316 |
| PWY.6416 | quinate degradation II | 0 | Not detected | -0.119 | -0.358 | 0.119 | 0.327 |
| PWY.6461 | peptidoglycan cross-bridge biosy | 0 | Not detected | -0.054 | -0.349 | 0.242 | 0.722 |
| PWY.6471 | peptidoglycan biosynthesis IV (E | 0 | Not detected | -0.070 | -0.221 | 0.082 | 0.367 |
| PWY.6473 | 4-aminobutanoate degradation IV | 0 | Not detected | 0.093 | -0.107 | 0.292 | 0.363 |
| PWY.6478 | GDP-D-<i>glycero</i>-&alpha;- | 0 | Not detected | -0.139 | -0.376 | 0.098 | 0.251 |
| PWY.6497 | D-galactarate degradation II | 0 | Not detected | 0.060 | -0.178 | 0.298 | 0.621 |
| PWY.6499 | D-glucarate degradation II | 0 | Not detected | 0.002 | -0.235 | 0.239 | 0.986 |
| PWY.6507 | 4-deoxy-L-<i>threo</i>-hex-4-e | 0 | Not detected | 0.000 | -0.085 | 0.086 | 0.991 |
| PWY.6517 | <i>N</i>-acetylglucosamine deg | 0 | Not detected | -0.010 | -0.111 | 0.091 | 0.844 |
| PWY.6518 | glycocholate metabolism (bacteri | 0 | Not detected | 0.201 | -0.068 | 0.470 | 0.144 |
| PWY.6519 | 8-amino-7-oxononanoate biosynt | 0 | Not detected | 0.157 | -0.061 | 0.375 | 0.158 |
| PWY.6527 | stachyose degradation | 0 | Not detected | -0.092 | -0.190 | 0.005 | 0.064 |
| PWY.6531 | mannitol cycle | 0 | Not detected | 0.068 | -0.168 | 0.304 | 0.571 |
| PWY.6535 | 4-aminobutanoate degradation I | 0 | Not detected | 0.187 | -0.045 | 0.420 | 0.115 |
| PWY.6536 | 4-aminobutanoate degradation II | 0 | Not detected | 0.183 | -0.018 | 0.383 | 0.075 |
| PWY.6537 | 4-aminobutanoate degradation II | 268 | Not detected | 0.237 | 0.041 | 0.433 | 0.018 |
| PWY.6545 | pyrimidine deoxyribonucleotides | 0 | Not detected | -0.059 | -0.164 | 0.047 | 0.277 |
| PWY.6549 | L-glutamine biosynthesis III | 0 | Not detected | -0.033 | -0.138 | 0.072 | 0.541 |
| PWY.6556 | pyrimidine ribonucleosides salva | 0 | Not detected | 0.154 | -0.036 | 0.344 | 0.112 |
| PWY.6559 | spermidine biosynthesis II | 0 | Not detected | -0.019 | -0.120 | 0.082 | 0.712 |
| PWY.6562 | norspermidine biosynthesis | 0 | Not detected | 0.040 | -0.193 | 0.272 | 0.738 |
| PWY.6572 | chondroitin sulfate degradation I | 0 | Not detected | 0.245 | -0.023 | 0.513 | 0.073 |
| PWY.6575 | juvenile hormone III biosynthesi | 0 | Not detected | -0.013 | -0.214 | 0.189 | 0.903 |
| PWY.6583 | pyruvate fermentation to butanol | 0 | Not detected | -0.102 | -0.212 | 0.007 | 0.067 |
| PWY.6587 | pyruvate fermentation to ethanol | 0 | Not detected | -0.008 | -0.290 | 0.274 | 0.954 |
| PWY.6596 | adenosine nucleotides degradatio | 0 | Not detected | 0.240 | -0.003 | 0.483 | 0.053 |
| PWY.66 | GDP-L-fucose biosynthesis I (fro | 0 | Not detected | 0.082 | -0.190 | 0.354 | 0.555 |

| PWY.6605 | adenine and adenosine salvage II | 0 | Not detected | 0.177 | -0.062 | 0.417 | 0.148 |
| --- | --- | --- | --- | --- | --- | --- | --- |
| PWY.6606 | guanosine nucleotides degradatio | 0 | Not detected | 0.129 | -0.106 | 0.364 | 0.282 |
| PWY.6607 | guanosine nucleotides degradatio | 0 | Not detected | 0.214 | -0.005 | 0.433 | 0.056 |
| PWY.6608 | guanosine nucleotides degradatio | 0 | Not detected | -0.007 | -0.251 | 0.236 | 0.952 |
| PWY.6610 | adenine salvage | 0 | Not detected | -0.050 | -0.290 | 0.189 | 0.680 |
| PWY.6613 | tetrahydrofolate salvage from 5,1 | 0 | Not detected | -0.081 | -0.212 | 0.050 | 0.226 |
| PWY.6614 | tetrahydrofolate biosynthesis | 0 | Not detected | 0.169 | -0.059 | 0.397 | 0.146 |
| PWY.6620 | guanine and guanosine salvage | 0 | Not detected | 0.063 | -0.185 | 0.311 | 0.618 |
| PWY.6626 | cytidine-5'-diphosphate-glycerol | 0 | Not detected | -0.097 | -0.318 | 0.123 | 0.388 |
| PWY.6637 | sulfolactate degradation II | 0 | Not detected | -0.029 | -0.129 | 0.071 | 0.564 |
| PWY.6654 | phosphopantothenate biosynthesi | 0 | Not detected | 0.130 | -0.064 | 0.325 | 0.189 |
| PWY.6672 | <i>cis</i>-genanyl-CoA degrada | 0 | Not detected | 0.078 | -0.172 | 0.327 | 0.541 |
| PWY.6682 | dehydrophos biosynthesis | 0 | Not detected | -0.337 | -0.632 | -0.041 | 0.026 |
| PWY.6690 | cinnamate and 3-hydroxycinnam | 326 | Not detected | 0.263 | 0.037 | 0.488 | 0.023 |
| PWY.6700 | queuosine biosynthesis | 0 | Not detected | -0.064 | -0.202 | 0.075 | 0.366 |
| PWY.6703 | preQ<sub>0</sub> biosynthesis | 0 | Not detected | -0.107 | -0.242 | 0.028 | 0.121 |
| PWY.6707 | gallate biosynthesis | 0 | Not detected | -0.119 | -0.358 | 0.119 | 0.327 |
| PWY.6708 | ubiquinol-8 biosynthesis (prokar | 0 | Not detected | 0.129 | -0.145 | 0.403 | 0.356 |
| PWY.6724 | starch degradation II | 0 | Not detected | -0.014 | -0.261 | 0.233 | 0.913 |
| PWY.6728 | methylaspartate cycle | 0 | Not detected | -0.044 | -0.144 | 0.057 | 0.392 |
| PWY.6731 | starch degradation III | 0 | Not detected | -0.099 | -0.239 | 0.041 | 0.166 |
| PWY.6737 | starch degradation V | 0 | Not detected | -0.100 | -0.240 | 0.040 | 0.160 |
| PWY.6748 | nitrate reduction VII (denitrificat | 0 | Not detected | 0.104 | -0.155 | 0.363 | 0.431 |
| PWY.6749 | CMP-legionaminate biosynthesis | 0 | Not detected | -0.005 | -0.228 | 0.219 | 0.967 |
| PWY.6771 | rhamnogalacturonan type I degra | 0 | Not detected | -0.028 | -0.212 | 0.155 | 0.764 |
| PWY.6772 | hydrogen production V | 0 | Not detected | 0.025 | -0.231 | 0.281 | 0.850 |
| PWY.6786 | detoxification of reactive carbony | 0 | Not detected | -0.033 | -0.114 | 0.049 | 0.432 |
| PWY.6788 | cellulose degradation II (fungi) | 0 | Not detected | 0.012 | -0.216 | 0.240 | 0.918 |
| PWY.6799 | fatty acid biosynthesis (plant mit | 0 | Not detected | -0.148 | -0.387 | 0.091 | 0.225 |
| PWY.6808 | dTDP-D-forosamine biosynthesis | 0 | Not detected | -0.056 | -0.143 | 0.031 | 0.206 |
| PWY.6839 | 2-aminoethylphosphonate biosyn | 0 | Not detected | -0.337 | -0.632 | -0.041 | 0.026 |
| PWY.6840 | homoglutathione biosynthesis | 0 | Not detected | 0.136 | -0.084 | 0.357 | 0.226 |
| PWY.6842 | glutathione-mediated detoxificati | 0 | Not detected | -0.077 | -0.289 | 0.136 | 0.480 |
| PWY.6854 | ethylene biosynthesis III (microb | 0 | Not detected | -0.107 | -0.367 | 0.153 | 0.421 |
| PWY.6855 | chitin degradation I (archaea) | 0 | Not detected | -0.021 | -0.251 | 0.210 | 0.861 |
| PWY.6859 | <i>all-trans</i>-farnesol biosynt | 0 | Not detected | 0.002 | -0.266 | 0.270 | 0.989 |
| PWY.6863 | pyruvate fermentation to hexanol | 0 | Not detected | -0.101 | -0.219 | 0.018 | 0.096 |
| PWY.6871 | 3-methylbutanol biosynthesis (en | 0 | Not detected | 0.007 | -0.147 | 0.161 | 0.928 |
| PWY.6883 | pyruvate fermentation to butanol | 0 | Not detected | -0.102 | -0.220 | 0.016 | 0.091 |
| PWY.6886 | 1-butanol autotrophic biosynthes | 0 | Not detected | -0.030 | -0.112 | 0.053 | 0.482 |
| PWY.6890 | 4-amino-2-methyl-5-diphosphom | 0 | Not detected | 0.013 | -0.090 | 0.116 | 0.803 |
| PWY.6891 | thiazole biosynthesis II (aerobic | 0 | Not detected | -0.014 | -0.108 | 0.080 | 0.772 |
| PWY.6892 | thiazole biosynthesis I (facultativ | 0 | Not detected | 0.001 | -0.078 | 0.080 | 0.981 |
| PWY.6893 | thiamine diphosphate biosynthes | 0 | Not detected | 0.087 | -0.115 | 0.289 | 0.397 |

| PWY.6894 | thiamine diphosphate biosynthes | 0 | Not detected | 0.112 | -0.110 | 0.335 | 0.322 |
| --- | --- | --- | --- | --- | --- | --- | --- |
| PWY.6896 | thiamine salvage I | 0 | Not detected | 0.179 | -0.043 | 0.400 | 0.114 |
| PWY.6897 | thiamine salvage II | 0 | Not detected | 0.112 | -0.110 | 0.335 | 0.322 |
| PWY.6901 | superpathway of glucose and xyl | 0 | Not detected | -0.030 | -0.113 | 0.052 | 0.475 |
| PWY.6902 | chitin degradation II (Vibrio) | 0 | Not detected | -0.016 | -0.245 | 0.213 | 0.891 |
| PWY.6906 | chitin derivatives degradation | 0 | Not detected | -0.011 | -0.096 | 0.073 | 0.792 |
| PWY.6938 | NADH repair | 0 | Not detected | 0.076 | -0.151 | 0.303 | 0.514 |
| PWY.6942 | dTDP-D-desosamine biosynthesi | 0 | Not detected | -0.053 | -0.140 | 0.035 | 0.237 |
| PWY.6944 | androstenedione degradation | 0 | Not detected | -0.115 | -0.355 | 0.125 | 0.346 |
| PWY.6949 | DIBOA-glucoside biosynthesis | 0 | Not detected | 0.158 | -0.023 | 0.340 | 0.088 |
| PWY.6953 | dTDP-3-acetamido-&alpha;-D-fu | 0 | Not detected | -0.052 | -0.140 | 0.035 | 0.241 |
| PWY.6961 | L-ascorbate degradation II (bacte | 0 | Not detected | 0.013 | -0.063 | 0.090 | 0.735 |
| PWY.6969 | TCA cycle V (2-oxoglutarate:ferr | 0 | Not detected | -0.036 | -0.125 | 0.053 | 0.431 |
| PWY.6973 | dTDP-D-olivose, dTDP-D-oliose | 0 | Not detected | -0.056 | -0.143 | 0.031 | 0.206 |
| PWY.6974 | dTDP-L-olivose biosynthesis | 0 | Not detected | -0.056 | -0.143 | 0.031 | 0.206 |
| PWY.6976 | dTDP-L-mycarose biosynthesis | 0 | Not detected | -0.052 | -0.139 | 0.034 | 0.234 |
| PWY.6981 | chitin biosynthesis | 0 | Not detected | -0.050 | -0.135 | 0.035 | 0.246 |
| PWY.6983 | <i>threo</i>-tetrahydrobiopterin | 0 | Not detected | -0.132 | -0.289 | 0.025 | 0.099 |
| PWY.6984 | lipoate salvage II | 0 | Not detected | 0.027 | -0.163 | 0.217 | 0.779 |
| PWY.6986 | alginate degradation | 0 | Not detected | -0.010 | -0.103 | 0.083 | 0.830 |
| PWY.6987 | lipoate biosynthesis and incorpor | 0 | Not detected | 0.055 | -0.176 | 0.287 | 0.640 |
| PWY.6992 | 1,5-anhydrofructose degradation | 0 | Not detected | -0.038 | -0.123 | 0.048 | 0.389 |
| PWY.7003 | glycerol degradation to butanol | 0 | Not detected | -0.030 | -0.112 | 0.053 | 0.481 |
| PWY.7007 | methyl ketone biosynthesis (engi | 0 | Not detected | 0.109 | -0.166 | 0.383 | 0.437 |
| PWY.7013 | (<i>S</i>)-propane-1,2-diol degr | 0 | Not detected | 0.019 | -0.238 | 0.276 | 0.886 |
| PWY.702 | L-methionine biosynthesis II (pla | 0 | Not detected | 0.112 | -0.148 | 0.373 | 0.398 |
| PWY.7031 | protein <i>N</i>-glycosylation ( | 0 | Not detected | -0.174 | -0.444 | 0.097 | 0.209 |
| PWY.7037 | protein <i>O</i>-glycosylation ( | 0 | Not detected | -0.172 | -0.443 | 0.099 | 0.214 |
| PWY.7077 | <i>N</i>-acetyl-D-galactosamin | 0 | Not detected | 0.024 | -0.219 | 0.267 | 0.846 |
| PWY.7085 | triethylamine degradation | 0 | Not detected | 0.008 | -0.260 | 0.276 | 0.952 |
| PWY.7090 | UDP-2,3-diacetamido-2,3-dideox | 0 | Not detected | -0.081 | -0.294 | 0.131 | 0.454 |
| PWY.7096 | triclosan resistance | 0 | Not detected | -0.026 | -0.303 | 0.250 | 0.852 |
| PWY.7102 | bisabolene biosynthesis (enginee | 0 | Not detected | 0.002 | -0.266 | 0.270 | 0.989 |
| PWY.7104 | dTDP-L-megosamine biosynthes | 0 | Not detected | -0.056 | -0.143 | 0.031 | 0.206 |
| PWY.7111 | pyruvate fermentation to isobutan | 0 | Not detected | -0.071 | -0.174 | 0.032 | 0.179 |
| PWY.7115 | C4 photosynthetic carbon assimil | 0 | Not detected | -0.070 | -0.350 | 0.209 | 0.623 |
| PWY.7117 | C4 photosynthetic carbon assimil | 0 | Not detected | -0.012 | -0.103 | 0.080 | 0.804 |
| PWY.7118 | chitin degradation to ethanol | 0 | Not detected | 0.053 | -0.228 | 0.334 | 0.713 |
| PWY.7124 | ethylene biosynthesis V (enginee | 0 | Not detected | -0.045 | -0.135 | 0.044 | 0.320 |
| PWY.7127 | CDP-D-mannitol biosynthesis | 0 | Not detected | -0.183 | -0.455 | 0.089 | 0.188 |
| PWY.7130 | L-glucose degradation | 0 | Not detected | 0.144 | -0.089 | 0.377 | 0.226 |
| PWY.7131 | CMP-legionaminate biosynthesis | 0 | Not detected | -0.006 | -0.238 | 0.225 | 0.958 |
| PWY.7141 | (3<i>S</i>)-linalool biosynthesis | 0 | Not detected | -0.059 | -0.283 | 0.166 | 0.609 |
| PWY.7153 | grixazone biosynthesis | 0 | Not detected | -0.008 | -0.254 | 0.238 | 0.949 |

| PWY.7159 | 3,8-divinyl-chlorophyllide <i>a< | 0 | Not detected | 0.197 | -0.056 | 0.449 | 0.127 |
| --- | --- | --- | --- | --- | --- | --- | --- |
| PWY.7165 | L-ascorbate biosynthesis VI (eng | 0 | Not detected | 0.156 | -0.114 | 0.426 | 0.257 |
| PWY.7174 | <i>S</i>-methyl-5-thio-&alpha;- | 0 | Not detected | -0.185 | -0.451 | 0.082 | 0.174 |
| PWY.7176 | UTP and CTP <i>de novo</i> bi | 0 | Not detected | -0.065 | -0.158 | 0.027 | 0.167 |
| PWY.7177 | UTP and CTP dephosphorylation | 0 | Not detected | -0.065 | -0.161 | 0.031 | 0.184 |
| PWY.7178 | ethylene glycol biosynthesis (eng | 0 | Not detected | 0.014 | -0.195 | 0.222 | 0.897 |
| PWY.7180 | 2'-deoxy-&alpha;-D-ribose 1-pho | 0 | Not detected | -0.020 | -0.278 | 0.239 | 0.881 |
| PWY.7182 | linalool biosynthesis I | 0 | Not detected | -0.059 | -0.283 | 0.166 | 0.609 |
| PWY.7184 | pyrimidine deoxyribonucleotides | 0 | Not detected | -0.059 | -0.164 | 0.047 | 0.275 |
| PWY.7185 | UTP and CTP dephosphorylation | 0 | Not detected | -0.065 | -0.161 | 0.031 | 0.184 |
| PWY.7187 | pyrimidine deoxyribonucleotides | 0 | Not detected | -0.056 | -0.157 | 0.045 | 0.278 |
| PWY.7193 | pyrimidine ribonucleosides salva | 0 | Not detected | 0.163 | -0.117 | 0.443 | 0.254 |
| PWY.7195 | pyrimidine ribonucleosides salva | 0 | Not detected | 0.154 | -0.036 | 0.344 | 0.112 |
| PWY.7197 | pyrimidine deoxyribonucleotide | 0 | Not detected | -0.061 | -0.165 | 0.042 | 0.246 |
| PWY.7198 | pyrimidine deoxyribonucleotides | 0 | Not detected | -0.063 | -0.170 | 0.045 | 0.251 |
| PWY.7204 | pyridoxal 5'-phosphate salvage II | 0 | Not detected | 0.197 | -0.047 | 0.442 | 0.113 |
| PWY.7205 | CMP phosphorylation | 0 | Not detected | -0.063 | -0.161 | 0.035 | 0.211 |
| PWY.7210 | pyrimidine deoxyribonucleotides | 0 | Not detected | -0.063 | -0.170 | 0.045 | 0.251 |
| PWY.7216 | (R)- and (S)-3-hydroxybutanoate | 0 | Not detected | -0.108 | -0.271 | 0.056 | 0.196 |
| PWY.7218 | photosynthetic 3-hydroxybutano | 0 | Not detected | -0.078 | -0.174 | 0.018 | 0.113 |
| PWY.7219 | adenosine ribonucleotides <i>de | 0 | Not detected | -0.068 | -0.174 | 0.037 | 0.203 |
| PWY.7221 | guanosine ribonucleotides <i>de | 0 | Not detected | -0.065 | -0.171 | 0.040 | 0.224 |
| PWY.7222 | guanosine deoxyribonucleotides | 0 | Not detected | -0.065 | -0.171 | 0.040 | 0.224 |
| PWY.7224 | purine deoxyribonucleosides salv | 0 | Not detected | -0.065 | -0.171 | 0.040 | 0.224 |
| PWY.7226 | guanosine deoxyribonucleotides | 0 | Not detected | -0.065 | -0.171 | 0.040 | 0.224 |
| PWY.7234 | inosine-5'-phosphate biosynthesi | 0 | Not detected | -0.076 | -0.220 | 0.068 | 0.301 |
| PWY.7237 | <i>myo</i>-, <i>chiro</i>- and | 0 | Not detected | -0.128 | -0.357 | 0.101 | 0.275 |
| PWY.7238 | sucrose biosynthesis II | 0 | Not detected | -0.059 | -0.140 | 0.022 | 0.153 |
| PWY.7241 | <I>myo</I>-inositol degradation | 0 | Not detected | -0.108 | -0.335 | 0.120 | 0.354 |
| PWY.7242 | D-fructuronate degradation | 0 | Not detected | -0.038 | -0.114 | 0.039 | 0.337 |
| PWY.7247 | &beta;-D-glucuronide and D-glu | 0 | Not detected | 0.000 | -0.091 | 0.091 | 0.995 |
| PWY.7248 | pectin degradation II | 0 | Not detected | 0.000 | -0.091 | 0.091 | 0.995 |
| PWY.7250 | [2Fe-2S] iron-sulfur cluster biosy | 0 | Not detected | 0.221 | -0.020 | 0.461 | 0.072 |
| PWY.7254 | TCA cycle VII (acetate-producer | 0 | Not detected | -0.039 | -0.131 | 0.054 | 0.413 |
| PWY.7255 | ergothioneine biosynthesis I (bac | 0 | Not detected | 0.136 | -0.084 | 0.357 | 0.226 |
| PWY.7268 | NAD/NADP-NADH/NADPH cy | 0 | Not detected | -0.075 | -0.274 | 0.124 | 0.460 |
| PWY.7269 | NAD/NADP-NADH/NADPH mi | 0 | Not detected | 0.084 | -0.122 | 0.289 | 0.425 |
| PWY.7279 | aerobic respiration II (cytochrom | 0 | Not detected | 0.088 | -0.117 | 0.294 | 0.401 |
| PWY.7282 | 4-amino-2-methyl-5-diphosphom | 0 | Not detected | 0.142 | -0.031 | 0.314 | 0.108 |
| PWY.7288 | fatty acid &beta;-oxidation (pero | 0 | Not detected | 0.079 | -0.179 | 0.337 | 0.550 |
| PWY.7290 | <i>Escherichia coli</i> serotype | 0 | Not detected | -0.008 | -0.289 | 0.274 | 0.957 |
| PWY.7294 | D-xylose degradation IV | 0 | Not detected | 0.073 | -0.199 | 0.344 | 0.599 |
| PWY.7295 | L-arabinose degradation IV | 0 | Not detected | 0.032 | -0.229 | 0.294 | 0.808 |
| PWY.7301 | dTDP-4-<i>O</i>-demethyl-&be | 0 | Not detected | -0.057 | -0.145 | 0.031 | 0.202 |

| PWY.7312 | dTDP-D-&beta;-fucofuranose bi | 0 | Not detected | -0.053 | -0.139 | 0.034 | 0.232 |
| --- | --- | --- | --- | --- | --- | --- | --- |
| PWY.7315 | dTDP-<i>N</i>-acetylthomosam | 0 | Not detected | -0.050 | -0.132 | 0.032 | 0.231 |
| PWY.7316 | dTDP-<i>N</i>-acetylviosamine | 0 | Not detected | -0.052 | -0.140 | 0.035 | 0.240 |
| PWY.7318 | dTDP-3-acetamido-3,6-dideoxy- | 0 | Not detected | -0.056 | -0.143 | 0.031 | 0.211 |
| PWY.7328 | superpathway of UDP-glucose-d | 0 | Not detected | -0.070 | -0.178 | 0.038 | 0.206 |
| PWY.7330 | UDP-<i>N</i>-acetyl-&beta;-L- | 0 | Not detected | -0.219 | -0.416 | -0.021 | 0.030 |
| PWY.7331 | UDP-<i>N</i>-acetyl-&beta;-L- | 0 | Not detected | -0.218 | -0.413 | -0.024 | 0.028 |
| PWY.7332 | superpathway of UDP-<I>N</i>- | 0 | Not detected | -0.222 | -0.431 | -0.013 | 0.038 |
| PWY.7335 | UDP-<i>N</i>-acetyl-&alpha;-D | 0 | Not detected | -0.145 | -0.282 | -0.009 | 0.037 |
| PWY.7336 | UDP-<i>N</i>-acetyl-&alpha;-D | 0 | Not detected | -0.068 | -0.288 | 0.153 | 0.548 |
| PWY.7342 | superpathway of nicotine biosynt | 0 | Not detected | -0.134 | -0.429 | 0.162 | 0.377 |
| PWY.7343 | UDP-&alpha;-D-glucose biosynt | 0 | Not detected | -0.100 | -0.196 | -0.003 | 0.043 |
| PWY.7344 | UDP-&alpha;-D-galactose biosy | 0 | Not detected | -0.146 | -0.310 | 0.019 | 0.082 |
| PWY.7346 | UDP-&alpha;-D-glucuronate bio | 0 | Not detected | -0.117 | -0.355 | 0.121 | 0.337 |
| PWY.7347 | sucrose biosynthesis III | 0 | Not detected | -0.055 | -0.153 | 0.044 | 0.277 |
| PWY.7377 | cob(II)yrinate <i>a,c</i>-diamid | 0 | Not detected | -0.008 | -0.119 | 0.103 | 0.893 |
| PWY.7378 | aminopropanol phosphate biosyn | 0 | Not detected | -0.113 | -0.364 | 0.139 | 0.381 |
| PWY.7383 | anaerobic energy metabolism (in | 0 | Not detected | -0.069 | -0.346 | 0.207 | 0.622 |
| PWY.7384 | anaerobic energy metabolism (in | 0 | Not detected | -0.086 | -0.193 | 0.021 | 0.114 |
| PWY.7385 | 1,3-propanediol biosynthesis (en | 0 | Not detected | -0.059 | -0.143 | 0.025 | 0.167 |
| PWY.7388 | octanoyl-[acyl-carrier protein] bi | 0 | Not detected | -0.147 | -0.385 | 0.092 | 0.230 |
| PWY.7391 | isoprene biosynthesis II (enginee | 0 | Not detected | 0.061 | -0.118 | 0.240 | 0.505 |
| PWY.7395 | D-galactosamine and <i>N</i>-a | 0 | Not detected | 0.024 | -0.219 | 0.267 | 0.846 |
| PWY.7396 | butanol and isobutanol biosynthe | 0 | Not detected | -0.082 | -0.207 | 0.044 | 0.203 |
| PWY.7398 | coumarins biosynthesis (engineer | 234 | Not detected | 0.209 | -0.021 | 0.439 | 0.075 |
| PWY.7399 | methylphosphonate degradation I | 0 | Not detected | 0.020 | -0.227 | 0.268 | 0.872 |
| PWY.7400 | L-arginine biosynthesis IV (archa | 0 | Not detected | -0.069 | -0.200 | 0.061 | 0.300 |
| PWY.7401 | crotonate fermentation (to acetate | 0 | Not detected | -0.153 | -0.305 | -0.001 | 0.049 |
| PWY.7410 | ipsdienol biosynthesis | 0 | Not detected | -0.059 | -0.283 | 0.166 | 0.609 |
| PWY.7413 | dTDP-6-deoxy-&alpha;-D-allose | 0 | Not detected | -0.056 | -0.143 | 0.031 | 0.206 |
| PWY.7414 | dTDP-&alpha;-D-mycaminose bi | 0 | Not detected | -0.056 | -0.143 | 0.031 | 0.211 |
| PWY.7419 | FR-900098 and FR-33289 antibi | 0 | Not detected | -0.337 | -0.632 | -0.041 | 0.026 |
| PWY.7425 | 2-chloroacrylate degradation I | 0 | Not detected | 0.088 | -0.132 | 0.307 | 0.434 |
| PWY.7431 | aromatic biogenic amine degrada | 302 | Not detected | 0.216 | 0.009 | 0.423 | 0.041 |
| PWY.7440 | dTDP-&beta;-L-4-<i>epi</i>-va | 0 | Not detected | -0.056 | -0.143 | 0.031 | 0.206 |
| PWY.7442 | drosopterin and aurodrosopterin | 0 | Not detected | -0.132 | -0.289 | 0.025 | 0.099 |
| PWY.7446 | sulfoquinovose degradation I | 0 | Not detected | -0.147 | -0.433 | 0.139 | 0.313 |
| PWY.7456 | &beta;-(1,4)-mannan degradation | 0 | Not detected | -0.043 | -0.187 | 0.102 | 0.563 |
| PWY.7459 | kojibiose degradation | 0 | Not detected | -0.027 | -0.258 | 0.204 | 0.821 |
| PWY.7467 | 2-acetamido-4-amino-2,4,6-tride | 0 | Not detected | -0.063 | -0.279 | 0.152 | 0.564 |
| PWY.7510 | rhizocticin A and B biosynthesis | 0 | Not detected | -0.337 | -0.632 | -0.041 | 0.026 |
| PWY.7524 | mevalonate pathway III (archaea) | 0 | Not detected | 0.061 | -0.118 | 0.240 | 0.505 |
| PWY.7530 | &beta;-D-galactosaminyl-(1&rar | 0 | Not detected | -0.005 | -0.287 | 0.276 | 0.971 |
| PWY.7539 | 6-hydroxymethyl-dihydropterin d | 0 | Not detected | -0.015 | -0.112 | 0.083 | 0.771 |

| PWY.7541 | 1,2-propanediol biosynthesis fro | 0 | Not detected | -0.039 | -0.322 | 0.244 | 0.788 |
| --- | --- | --- | --- | --- | --- | --- | --- |
| PWY.7552 | heme <i>b</i> biosynthesis III (f | 0 | Not detected | -0.203 | -0.459 | 0.053 | 0.121 |
| PWY.7559 | glutathione degradation (DUG pa | 0 | Not detected | 0.238 | -0.019 | 0.496 | 0.070 |
| PWY.7560 | methylerythritol phosphate pathw | 0 | Not detected | -0.073 | -0.199 | 0.053 | 0.257 |
| PWY.7562 | 3,6-anhydro-&alpha;-L-galactop | 0 | Not detected | -0.005 | -0.090 | 0.081 | 0.916 |
| PWY.7564 | bacimethrin and bacimethrin pyr | 0 | Not detected | -0.139 | -0.313 | 0.034 | 0.115 |
| PWY.7568 | L-galactose degradation | 0 | Not detected | -0.024 | -0.244 | 0.195 | 0.827 |
| PWY.7573 | GDP-mycosamine biosynthesis | 0 | Not detected | 0.082 | -0.190 | 0.354 | 0.555 |
| PWY.7581 | <i>N</i>-acetylneuraminate and | 0 | Not detected | 0.060 | -0.131 | 0.252 | 0.537 |
| PWY.7586 | &beta;-1,4-D-mannosyl-<i>N</i | 0 | Not detected | -0.083 | -0.313 | 0.147 | 0.478 |
| PWY.7588 | ursodeoxycholate biosynthesis (b | 0 | Not detected | 0.041 | -0.178 | 0.260 | 0.711 |
| PWY.7610 | GDP-6-deoxy-D-<i>altro</i>-he | 0 | Not detected | -0.128 | -0.332 | 0.075 | 0.217 |
| PWY.7616 | methanol oxidation to carbon dio | 0 | Not detected | 0.020 | -0.235 | 0.276 | 0.875 |
| PWY.7622 | UDP-&alpha;-D-galactofuranose | 0 | Not detected | -0.055 | -0.163 | 0.054 | 0.326 |
| PWY.7644 | heparin degradation | 0 | Not detected | -0.099 | -0.357 | 0.160 | 0.453 |
| PWY.7645 | hyaluronan degradation | 0 | Not detected | 0.206 | -0.027 | 0.440 | 0.084 |
| PWY.7646 | dermatan sulfate degradation I (b | 0 | Not detected | 0.205 | -0.027 | 0.436 | 0.084 |
| PWY.7651 | heparan sulfate degradation | 0 | Not detected | 0.071 | -0.263 | 0.404 | 0.678 |
| PWY.7657 | dTDP-&beta;-L-digitoxose biosy | 0 | Not detected | -0.056 | -0.143 | 0.031 | 0.206 |
| PWY.7659 | viridicatumtoxin biosynthesis | 0 | Not detected | -0.059 | -0.283 | 0.166 | 0.609 |
| PWY.7663 | gondoate biosynthesis (anaerobic | 0 | Not detected | -0.067 | -0.179 | 0.046 | 0.246 |
| PWY.7664 | oleate biosynthesis IV (anaerobic | 0 | Not detected | -0.068 | -0.182 | 0.045 | 0.240 |
| PWY.7674 | CMP-8-amino-3,8-dideoxy-D-<I | 0 | Not detected | -0.093 | -0.333 | 0.147 | 0.449 |
| PWY.7675 | Kdo transfer to lipid IV<sub>A< | 0 | Not detected | 0.164 | -0.064 | 0.392 | 0.159 |
| PWY.7684 | &alpha;-diglucosyldiacylglycerol | 0 | Not detected | -0.036 | -0.261 | 0.190 | 0.755 |
| PWY.7688 | dTDP-D-ravidosamine and dTDP | 0 | Not detected | -0.052 | -0.140 | 0.035 | 0.241 |
| PWY.7709 | (3<i>R</i>)-linalool biosynthesi | 0 | Not detected | -0.059 | -0.283 | 0.166 | 0.609 |
| PWY.7716 | penicillin G and penicillin V bios | 0 | Not detected | -0.133 | -0.277 | 0.011 | 0.071 |
| PWY.7719 | CMP-diacetamido-8-epilegionam | 0 | Not detected | -0.189 | -0.379 | 0.001 | 0.052 |
| PWY.7721 | methyl phomopsenoate biosynthe | 0 | Not detected | -0.059 | -0.289 | 0.170 | 0.612 |
| PWY.7736 | stellatic acid biosynthesis | 0 | Not detected | -0.058 | -0.325 | 0.208 | 0.668 |
| PWY.7754 | bile acids degradation | 0 | Not detected | -0.379 | -0.709 | -0.048 | 0.025 |
| PWY.7755 | iso-bile acids biosynthesis I | 0 | Not detected | 0.237 | -0.071 | 0.545 | 0.132 |
| PWY.7756 | iso-bile acids biosynthesis II | 0 | Not detected | -0.058 | -0.251 | 0.135 | 0.555 |
| PWY.7766 | heme <i>b</i> biosynthesis IV ( | 0 | Not detected | 0.195 | -0.057 | 0.447 | 0.130 |
| PWY.7769 | phosalacine biosynthesis | 0 | Not detected | -0.330 | -0.624 | -0.037 | 0.028 |
| PWY.7778 | 2-methylpropene degradation | 0 | Not detected | -0.109 | -0.272 | 0.054 | 0.192 |
| PWY.7779 | methyl <i>tert</i>-butyl ether de | 0 | Not detected | -0.109 | -0.272 | 0.054 | 0.192 |
| PWY.7790 | UMP biosynthesis II | 0 | Not detected | -0.042 | -0.149 | 0.066 | 0.446 |
| PWY.7791 | UMP biosynthesis III | 0 | Not detected | -0.042 | -0.149 | 0.066 | 0.446 |
| PWY.7793 | dimethyl sulfide biosynthesis fro | 17 | Not detected | -0.339 | -0.563 | -0.115 | 0.003 |
| PWY.7805 | (aminomethyl)phosphonate degra | 0 | Not detected | 0.105 | -0.140 | 0.351 | 0.401 |
| PWY.7807 | glyphosate degradation III | 0 | Not detected | 0.134 | -0.101 | 0.370 | 0.264 |
| PWY.7808 | tetracycline resistance | 0 | Not detected | -0.149 | -0.542 | 0.244 | 0.458 |

| PWY.7814 | dTDP-L-daunosamine biosynthe | 0 | Not detected | -0.057 | -0.145 | 0.031 | 0.202 |
| --- | --- | --- | --- | --- | --- | --- | --- |
| PWY.7815 | poly(ribitol phosphate) wall teich | 236 | Not detected | -0.170 | -0.294 | -0.046 | 0.007 |
| PWY.7816 | poly(ribitol phosphate) wall teich | 236 | Not detected | -0.170 | -0.294 | -0.046 | 0.007 |
| PWY.7817 | type I lipoteichoic acid biosynthe | 0 | Not detected | -0.088 | -0.199 | 0.024 | 0.125 |
| PWY.7818 | type IV lipoteichoic acid biosynt | 0 | Not detected | -0.183 | -0.383 | 0.018 | 0.074 |
| PWY.7819 | poly(3-O-&beta;-D-glucopyrano | 0 | Not detected | -0.146 | -0.282 | -0.009 | 0.037 |
| PWY.7820 | teichuronic acid biosynthesis (<i | 0 | Not detected | -0.109 | -0.347 | 0.128 | 0.368 |
| PWY.7822 | chitin degradation III (Serratia) | 0 | Not detected | -0.015 | -0.245 | 0.214 | 0.897 |
| PWY.7852 | 6-hydroxymethyl-dihydropterin d | 0 | Not detected | -0.091 | -0.230 | 0.048 | 0.200 |
| PWY.7854 | crotonyl-CoA/ethylmalonyl-CoA | 0 | Not detected | 0.041 | -0.220 | 0.303 | 0.756 |
| PWY.7856 | heme <i>a</i> biosynthesis | 0 | Not detected | 0.102 | -0.088 | 0.292 | 0.294 |
| PWY.7858 | (5Z)-dodecenoate biosynthesis II | 0 | Not detected | -0.089 | -0.198 | 0.019 | 0.108 |
| PWY.7863 | roseoflavin biosynthesis | 0 | Not detected | 0.158 | -0.040 | 0.357 | 0.118 |
| PWY.7864 | 8-methylmenaquinone biosynthe | 0 | Not detected | 0.040 | -0.240 | 0.321 | 0.778 |
| PWY.7873 | D-erythronate degradation II | 0 | Not detected | 0.056 | -0.153 | 0.264 | 0.599 |
| PWY.7874 | L-threonate degradation | 0 | Not detected | 0.180 | -0.055 | 0.414 | 0.134 |
| PWY.7883 | anhydromuropeptides recycling I | 0 | Not detected | 0.154 | -0.096 | 0.404 | 0.226 |
| PWY.7884 | lipoprotein posttranslational mod | 0 | Not detected | 0.294 | 0.012 | 0.577 | 0.042 |
| PWY.7892 | tRNA-uridine 2-thiolation (bacte | 0 | Not detected | 0.029 | -0.156 | 0.214 | 0.759 |
| PWY.7900 | glycogen biosynthesis III (from & | 0 | Not detected | -0.097 | -0.209 | 0.015 | 0.090 |
| PWY.7902 | glucosylglycerol biosynthesis | 0 | Not detected | -0.119 | -0.241 | 0.003 | 0.056 |
| PWY.7904 | tRNA-uridine 2-thiolation (therm | 0 | Not detected | -0.023 | -0.262 | 0.217 | 0.854 |
| PWY.7909 | formaldehyde oxidation VII (TH | 0 | Not detected | -0.085 | -0.234 | 0.064 | 0.263 |
| PWY.7928 | nickel cofactor biosynthesis | 0 | Not detected | -0.070 | -0.251 | 0.110 | 0.445 |
| PWY.7953 | UDP-<i>N</i>-acetylmuramoyl- | 0 | Not detected | -0.061 | -0.135 | 0.012 | 0.103 |
| PWY.7975 | adenosylcobalamin biosynthesis | 0 | Not detected | 0.091 | -0.102 | 0.283 | 0.357 |
| PWY.7977 | L-methionine biosynthesis IV (ar | 0 | Not detected | 0.007 | -0.085 | 0.099 | 0.874 |
| PWY.7978 | <i>N</i>-methylpyrrolidone deg | 0 | Not detected | 0.093 | -0.107 | 0.292 | 0.363 |
| PWY.7981 | &alpha;-dystroglycan glycosylati | 0 | Not detected | -0.191 | -0.462 | 0.080 | 0.167 |
| PWY.7991 | toxoflavin biosynthesis | 0 | Not detected | -0.005 | -0.095 | 0.085 | 0.915 |
| PWY.8004 | Entner-Doudoroff pathway I | 0 | Not detected | 0.017 | -0.066 | 0.100 | 0.694 |
| PWY.801 | homocysteine and cysteine interc | 0 | Not detected | -0.119 | -0.373 | 0.136 | 0.361 |
| PWY.8010 | L-cysteine biosynthesis IX (Trich | 0 | Not detected | -0.115 | -0.237 | 0.007 | 0.064 |
| PWY.8012 | mupirocin biosynthesis | 0 | Not detected | -0.148 | -0.387 | 0.091 | 0.225 |
| PWY.8013 | UDP-<i>N</i>-acetyl-D-galacto | 0 | Not detected | -0.055 | -0.153 | 0.044 | 0.277 |
| PWY.8015 | glycine degradation (Stickland re | 0 | Not detected | -0.059 | -0.140 | 0.022 | 0.155 |
| PWY.8040 | ansatrienin biosynthesis | 0 | Not detected | -0.002 | -0.271 | 0.267 | 0.990 |
| PWY.882 | L-ascorbate biosynthesis I (L-gal | 0 | Not detected | -0.021 | -0.253 | 0.211 | 0.858 |
| PWY.922 | mevalonate pathway I | 0 | Not detected | 0.061 | -0.118 | 0.240 | 0.505 |
| PWY.I9 | L-cysteine biosynthesis VI (from | 0 | Not detected | -0.042 | -0.302 | 0.218 | 0.750 |
| PWY0.1182 | trehalose degradation II (cytosoli | 0 | Not detected | 0.044 | -0.230 | 0.318 | 0.752 |
| PWY0.1221 | putrescine degradation II | 0 | Not detected | 0.120 | -0.112 | 0.352 | 0.312 |
| PWY0.1241 | ADP-L-<i>glycero</i>-&beta;-D | 0 | Not detected | -0.127 | -0.359 | 0.106 | 0.286 |
| PWY0.1261 | anhydromuropeptides recycling I | 0 | Not detected | -0.059 | -0.129 | 0.011 | 0.098 |

| PWY0.1264 | biotin-carboxyl carrier protein as | 0 | Not detected | -0.034 | -0.144 | 0.076 | 0.545 |
| --- | --- | --- | --- | --- | --- | --- | --- |
| PWY0.1275 | lipoate biosynthesis and incorpor | 0 | Not detected | -0.033 | -0.231 | 0.166 | 0.748 |
| PWY0.1296 | purine ribonucleosides degradati | 0 | Not detected | 0.001 | -0.213 | 0.215 | 0.993 |
| PWY0.1300 | 2-<I>O</I>-&alpha;-mannosyl-D | 0 | Not detected | 0.172 | -0.093 | 0.438 | 0.204 |
| PWY0.1306 | L-galactonate degradation | 0 | Not detected | 0.000 | -0.187 | 0.188 | 0.997 |
| PWY0.1312 | acetate and ATP formation from | 0 | Not detected | -0.032 | -0.110 | 0.046 | 0.420 |
| PWY0.1313 | acetate conversion to acetyl-CoA | 0 | Not detected | 0.078 | -0.172 | 0.327 | 0.541 |
| PWY0.1317 | L-lactaldehyde degradation (aero | 0 | Not detected | 0.189 | -0.040 | 0.418 | 0.107 |
| PWY0.1319 | CDP-diacylglycerol biosynthesis | 0 | Not detected | -0.045 | -0.243 | 0.153 | 0.653 |
| PWY0.1324 | <i>N</i>-acetylneuraminate and | 0 | Not detected | 0.037 | -0.127 | 0.201 | 0.661 |
| PWY0.1337 | oleate &beta;-oxidation | 0 | Not detected | 0.094 | -0.148 | 0.337 | 0.446 |
| PWY0.1338 | polymyxin resistance | 0 | Not detected | 0.155 | -0.102 | 0.411 | 0.237 |
| PWY0.1415 | superpathway of heme <i>b</i> | 0 | Not detected | 0.197 | -0.058 | 0.452 | 0.130 |
| PWY0.1433 | tetrahydromonapterin biosynthes | 0 | Not detected | -0.064 | -0.202 | 0.073 | 0.359 |
| PWY0.1465 | D-malate degradation | 0 | Not detected | -0.003 | -0.241 | 0.234 | 0.977 |
| PWY0.1466 | trehalose degradation VI (peripla | 0 | Not detected | 0.212 | -0.043 | 0.466 | 0.104 |
| PWY0.1471 | uracil degradation III | 0 | Not detected | 0.130 | -0.092 | 0.352 | 0.253 |
| PWY0.1477 | ethanolamine utilization | 0 | Not detected | -0.023 | -0.094 | 0.047 | 0.519 |
| PWY0.1479 | tRNA processing | 0 | Not detected | 0.231 | -0.036 | 0.499 | 0.090 |
| PWY0.1533 | methylphosphonate degradation I | 0 | Not detected | 0.023 | -0.220 | 0.266 | 0.853 |
| PWY0.1545 | cardiolipin biosynthesis III | 0 | Not detected | 0.026 | -0.225 | 0.278 | 0.838 |
| PWY0.1546 | muropeptide degradation | 0 | Not detected | 0.120 | -0.098 | 0.339 | 0.282 |
| PWY0.1565 | D-lactate to cytochrome <i>bo</ | 0 | Not detected | 0.088 | -0.132 | 0.307 | 0.434 |
| PWY0.1567 | NADH to cytochrome <i>bo</i> | 0 | Not detected | 0.088 | -0.117 | 0.294 | 0.401 |
| PWY0.1568 | NADH to cytochrome <i>bd</i> | 0 | Not detected | 0.088 | -0.117 | 0.294 | 0.401 |
| PWY0.1569 | autoinducer AI-2 degradation | 233 | Not detected | 0.212 | -0.006 | 0.429 | 0.056 |
| PWY0.1573 | nitrate reduction VIIIb (dissimila | 0 | Not detected | 0.088 | -0.117 | 0.294 | 0.401 |
| PWY0.1585 | formate to nitrite electron transfe | 0 | Not detected | 0.097 | -0.133 | 0.327 | 0.410 |
| PWY0.1586 | peptidoglycan maturation (<i>me | 0 | Not detected | 0.278 | -0.041 | 0.597 | 0.088 |
| PWY0.1590 | NADH to hydrogen peroxide ele | 0 | Not detected | 0.159 | -0.075 | 0.393 | 0.183 |
| PWY0.1591 | glycerol-3-phosphate to hydroge | 0 | Not detected | 0.159 | -0.075 | 0.393 | 0.183 |
| PWY0.1597 | prenylated FMNH<sub>2</sub> | 0 | Not detected | 0.144 | -0.045 | 0.334 | 0.136 |
| PWY0.166 | superpathway of pyrimidine deox | 0 | Not detected | -0.058 | -0.163 | 0.047 | 0.282 |
| PWY0.301 | L-ascorbate degradation I (bacter | 0 | Not detected | 0.013 | -0.063 | 0.090 | 0.735 |
| PWY0.321 | phenylacetate degradation I (aero | 0 | Not detected | -0.084 | -0.212 | 0.044 | 0.201 |
| PWY0.42 | 2-methylcitrate cycle I | 0 | Not detected | 0.118 | -0.174 | 0.409 | 0.429 |
| PWY0.43 | conversion of succinate to propa | 0 | Not detected | -0.064 | -0.239 | 0.110 | 0.470 |
| PWY0.44 | D-allose degradation | 0 | Not detected | -0.170 | -0.446 | 0.105 | 0.226 |
| PWY0.461 | L-lysine degradation I | 0 | Not detected | 0.120 | -0.117 | 0.357 | 0.320 |
| PWY0.501 | lipoate biosynthesis and incorpor | 0 | Not detected | 0.082 | -0.120 | 0.284 | 0.424 |
| PWY0.522 | lipoate salvage I | 0 | Not detected | 0.050 | -0.174 | 0.274 | 0.660 |
| PWY0.662 | PRPP biosynthesis | 144 | Not detected | -0.148 | -0.264 | -0.032 | 0.013 |
| PWY0.862 | (5Z)-dodecenoate biosynthesis I | 0 | Not detected | -0.086 | -0.195 | 0.022 | 0.120 |
| PWY1.3 | polyhydroxybutanoate biosynthe | 0 | Not detected | -0.036 | -0.237 | 0.165 | 0.725 |

| PWY3O.4107 | NAD salvage pathway V (PNC V | 0 | Not detected | 0.183 | -0.047 | 0.413 | 0.119 |
| --- | --- | --- | --- | --- | --- | --- | --- |
| PWY4FS.7 | phosphatidylglycerol biosynthesi | 0 | Not detected | 0.000 | -0.234 | 0.234 | 0.999 |
| PWY4FS.8 | phosphatidylglycerol biosynthesi | 0 | Not detected | 0.000 | -0.234 | 0.234 | 0.999 |
| PWY4LZ.257 | superpathway of fermentation (<i | 0 | Not detected | -0.024 | -0.094 | 0.047 | 0.513 |
| PWY66.161 | ethanol degradation III | 0 | Not detected | 0.086 | -0.164 | 0.335 | 0.501 |
| PWY66.162 | ethanol degradation IV | 0 | Not detected | 0.086 | -0.164 | 0.335 | 0.501 |
| PWY66.21 | ethanol degradation II | 0 | Not detected | 0.076 | -0.188 | 0.340 | 0.573 |
| PWY66.366 | flavin biosynthesis IV (mammali | 0 | Not detected | 0.189 | -0.047 | 0.424 | 0.116 |
| PWY66.391 | fatty acid &beta;-oxidation VI (p | 0 | Not detected | 0.011 | -0.291 | 0.313 | 0.942 |
| PWY66.398 | TCA cycle III (animals) | 0 | Not detected | -0.041 | -0.143 | 0.062 | 0.434 |
| PWY66.399 | gluconeogenesis III | 0 | Not detected | -0.039 | -0.128 | 0.049 | 0.384 |
| PWY66.422 | D-galactose degradation V (Lelo | 0 | Not detected | -0.038 | -0.121 | 0.045 | 0.366 |
| PWY66.426 | hydrogen sulfide biosynthesis II | 0 | Not detected | 0.170 | -0.053 | 0.393 | 0.136 |
| PYRIDNUCS | NAD salvage pathway I (PNC VI | 0 | Not detected | 0.191 | -0.041 | 0.423 | 0.107 |
| PYRIDNUCS | NAD <i>de novo</i> biosynthes | 0 | Not detected | -0.142 | -0.442 | 0.158 | 0.354 |
| PYRIDOXSY | pyridoxal 5'-phosphate biosynthe | 0 | Not detected | -0.014 | -0.312 | 0.283 | 0.925 |
| PYRUVDEH | NA | 0 | Not detected | -0.012 | -0.264 | 0.239 | 0.924 |
| PYRUVDEH | pyruvate decarboxylation to acet | 0 | Not detected | -0.012 | -0.264 | 0.239 | 0.924 |
| QUINATEDE | quinate degradation I | 0 | Not detected | -0.074 | -0.298 | 0.149 | 0.514 |
| REDCITCYC | TCA cycle VIII (Helicobacter) | 0 | Not detected | -0.044 | -0.144 | 0.057 | 0.395 |
| RHAMCAT.P | L-rhamnose degradation I | 0 | Not detected | 0.158 | -0.067 | 0.383 | 0.170 |
| RIBOSYN2.P | flavin biosynthesis I (bacteria an | 0 | Not detected | -0.005 | -0.093 | 0.083 | 0.914 |
| RUMP.PWY | formaldehyde oxidation I | 0 | Not detected | -0.038 | -0.122 | 0.047 | 0.381 |
| RXN.12588 | NA | 0 | Not detected | 0.147 | -0.108 | 0.403 | 0.260 |
| RXN.13163 | NA | 0 | Not detected | -0.213 | -0.509 | 0.083 | 0.159 |
| RXN.14047 | NA | 0 | Not detected | -0.030 | -0.138 | 0.077 | 0.580 |
| RXN.14201 | NA | 0 | Not detected | -0.032 | -0.096 | 0.033 | 0.335 |
| RXN.14249 | NA | 0 | Not detected | -0.113 | -0.364 | 0.139 | 0.381 |
| RXN.14314 | NA | 0 | Not detected | -0.095 | -0.337 | 0.146 | 0.439 |
| RXN.17127 | NA | 0 | Not detected | 0.050 | -0.174 | 0.274 | 0.660 |
| RXN.20025 | NA | 0 | Not detected | 0.179 | -0.022 | 0.380 | 0.081 |
| RXN0.1141 | NA | 0 | Not detected | 0.033 | -0.157 | 0.222 | 0.736 |
| RXN0.6511 | NA | 0 | Not detected | 0.226 | -0.018 | 0.470 | 0.070 |
| SERSYN.PW | L-serine biosynthesis I | 0 | Not detected | -0.098 | -0.200 | 0.004 | 0.061 |
| SUCROSEU | sucrose degradation VII (sucrose | 0 | Not detected | 0.098 | -0.149 | 0.345 | 0.435 |
| SUCSYN.PW | sucrose biosynthesis I (from phot | 0 | Not detected | -0.039 | -0.128 | 0.049 | 0.387 |
| SUCUTIL.P | sucrose degradation I (sucrose ph | 0 | Not detected | 0.034 | -0.204 | 0.272 | 0.780 |
| SULFMETII. | assimilatory sulfate reduction II | 0 | Not detected | 0.018 | -0.277 | 0.312 | 0.907 |
| TCA | TCA cycle I (prokaryotic) | 0 | Not detected | -0.035 | -0.128 | 0.059 | 0.466 |
| TEICHOICA | poly(glycerol phosphate) wall tei | 0 | Not detected | -0.149 | -0.280 | -0.018 | 0.026 |
| THRDLCTC | L-threonine degradation III (to m | 0 | Not detected | -0.113 | -0.364 | 0.139 | 0.381 |
| THREONINE | L-threonine degradation II | 0 | Not detected | -0.113 | -0.364 | 0.139 | 0.381 |
| TREDEGLO | trehalose degradation I (low osm | 0 | Not detected | -0.025 | -0.142 | 0.092 | 0.673 |
| TRESYN.PW | trehalose biosynthesis I | 0 | Not detected | 0.179 | -0.043 | 0.400 | 0.114 |

| TRIGLSYN.P | diacylglycerol and triacylglycero | 0 | Not detected | 0.215 | -0.059 | 0.490 | 0.124 |
| --- | --- | --- | --- | --- | --- | --- | --- |
| TRNA.CHAR | tRNA charging | 0 | Not detected | -0.038 | -0.098 | 0.022 | 0.212 |
| TRPSYN.PW | L-tryptophan biosynthesis | 0 | Not detected | 0.071 | -0.214 | 0.357 | 0.625 |
| TRYPSYN.R | NA | 0 | Not detected | 0.071 | -0.175 | 0.316 | 0.573 |
| UDPNACET | UDP-<i>N</i>-acetyl-D-glucosa | 0 | Not detected | -0.051 | -0.142 | 0.041 | 0.278 |
| UDPNAGSY | UDP-<i>N</i>-acetyl-D-glucosa | 0 | Not detected | -0.050 | -0.138 | 0.038 | 0.268 |
| VALSYN.PW | L-valine biosynthesis | 0 | Not detected | -0.071 | -0.174 | 0.032 | 0.179 |
| X1.1.1.39.RX | NA | 0 | Not detected | -0.070 | -0.350 | 0.209 | 0.623 |
| X1CMET2.P | <i>N</i><sup>10</sup>-formyl | 0 | Not detected | -0.011 | -0.099 | 0.077 | 0.813 |
| X3.HYDROX | 4-hydroxyphenylacetate degradat | 0 | Not detected | 0.116 | -0.125 | 0.357 | 0.346 |

| **Supplementary Table 11.** Association of childhood economic hardship with enzymatic reactions. Analysis of Compositions of Microbiomes (ANCOM2) was used to identify enzymatic reactions related to childhood economic hardship. Regression coefficients were obtained by modelling the effect of childhood economic hardship on centered log ratio (CLR)-transformed species in multivariable linear regression adjusted for age, gender, BMI, field center, Hispanic/Latino background, U.S. nativity, Bristol stool type, current SEP, the alternative Healthy Eating Index 2010 score, cigarette smoking, alcohol use, depressive and anxiety symptoms, diabetes, hypertension, cardiovascular disease, dyslipidemia, and antibiotic use. | | | | | | | |
| --- | --- | --- | --- | --- | --- | --- | --- |
| otu.names | V2 | W_stat | ANCOM | beta | l95ci | u95ci | qval |
| ENZRXN.17 | methylthioalkylmalate dehy | 822 | 0.7 | -0.239 | -0.419 | -0.060 | 0.009 |
| ENZRXN.17 | methylthioalkylmalate dehy | 822 | 0.7 | -0.239 | -0.419 | -0.060 | 0.009 |
| ENZRXN.17 | methylthioalkylmalate dehy | 822 | 0.7 | -0.239 | -0.419 | -0.060 | 0.009 |
| ENZRXN.17 | methylthioalkylmalate dehy | 822 | 0.7 | -0.239 | -0.419 | -0.060 | 0.009 |
| ENZRXN.17 | methylthioalkylmalate dehy | 822 | 0.7 | -0.239 | -0.419 | -0.060 | 0.009 |
| ENZRXN.25 | isopropylmalate dehydroge | 822 | 0.7 | -0.239 | -0.419 | -0.060 | 0.009 |
| ENZRXNI.14 | fumarase | 824 | 0.7 | -0.372 | -0.649 | -0.095 | 0.009 |
| ACETATEKI | acetate kinase | 0 | Not detected | 0.051 | -0.184 | 0.287 | 0.669 |
| ACS.ENZRX | acetyl-CoA synthetase (AM | 0 | Not detected | 0.049 | -0.232 | 0.330 | 0.731 |
| ACYLACPS | acyl-ACP synthetase | 0 | Not detected | 0.099 | -0.157 | 0.355 | 0.450 |
| ACYLCOAD | acyl-CoA dehydrogenase | 0 | Not detected | 0.082 | -0.174 | 0.338 | 0.529 |
| ACYLGPEA | 2-acylglycerophosphoethan | 0 | Not detected | 0.099 | -0.157 | 0.355 | 0.450 |
| ADENYL.KI | adenylate kinase | 0 | Not detected | -0.107 | -0.263 | 0.048 | 0.177 |
| ADENYLAT | adenylate cyclase | 0 | Not detected | 0.095 | -0.185 | 0.375 | 0.505 |
| ADOMET.D | S-adenosylmethionine:2-de | 0 | Not detected | -0.014 | -0.316 | 0.287 | 0.926 |
| AICARTRAN | AICAR transformylase | 0 | Not detected | -0.074 | -0.280 | 0.132 | 0.483 |
| ALDOSE1EP | galactose-1-epimerase | 16 | Not detected | 0.236 | 0.021 | 0.452 | 0.032 |
| ALPHA.AM | Î±-amylase | 0 | Not detected | 0.166 | -0.047 | 0.378 | 0.126 |
| ALPHA.AM | Î±-amylase | 0 | Not detected | 0.008 | -0.252 | 0.268 | 0.952 |
| ALTRO.OXI | tagaturonate reductase | 0 | Not detected | 0.131 | -0.095 | 0.357 | 0.257 |
| ALTRODEH | D-altronate dehydratase | 0 | Not detected | -0.056 | -0.204 | 0.092 | 0.457 |
| AMYLOMA | amylomaltase | 0 | Not detected | 0.050 | -0.218 | 0.318 | 0.713 |
| ARGS.ENZR | arginine&mdash;tRNA liga | 0 | Not detected | 0.146 | -0.138 | 0.430 | 0.313 |
| ARGSUCCIN | argininosuccinate lyase | 0 | Not detected | -0.121 | -0.285 | 0.044 | 0.150 |
| ARGSUCCT | arginine N-succinyltransfer | 0 | Not detected | 0.158 | -0.045 | 0.361 | 0.128 |
| BIOTINLIG. | biotin-[acetyl-CoA-carboxy | 0 | Not detected | 0.050 | -0.162 | 0.262 | 0.644 |
| CARDIOLIP | cardiolipin synthase | 0 | Not detected | 0.074 | -0.137 | 0.285 | 0.492 |
| CDPDIGLYP | CDP-diacylglycerol diphos | 0 | Not detected | 0.107 | -0.083 | 0.297 | 0.271 |
| CDPDIGLYS | CTP:2,3,4-saturated L-pho | 15 | Not detected | 0.177 | -0.009 | 0.362 | 0.063 |
| CHD.ENZRX | choline dehydrogenase | 0 | Not detected | 0.075 | -0.178 | 0.328 | 0.562 |
| CMPKI.ENZ | CMP kinase | 0 | Not detected | -0.131 | -0.393 | 0.130 | 0.325 |
| CPM.KDOSY | 3-deoxy-manno-octulosona | 0 | Not detected | -0.018 | -0.324 | 0.288 | 0.907 |
| CROBETRE | crotonobetainyl-CoA reduc | 0 | Not detected | 0.004 | -0.246 | 0.255 | 0.973 |
| CYCPHOSD | 2',3'-cyclic nucleotide 2'-ph | 0 | Not detected | 0.174 | -0.111 | 0.458 | 0.231 |
| CYSS.ENZR | cysteine&mdash;tRNA liga | 0 | Not detected | 0.072 | -0.230 | 0.373 | 0.642 |
| CYTIDINEK | cytidine kinase | 0 | Not detected | 0.136 | -0.162 | 0.434 | 0.370 |
| D.PPENTOM | deoxyribose 1,5-phosphom | 0 | Not detected | -0.083 | -0.413 | 0.246 | 0.620 |

| DALADALA | D-alanine&mdash;D-alanin | 0 | Not detected | 0.112 | -0.098 | 0.322 | 0.295 |
| --- | --- | --- | --- | --- | --- | --- | --- |
| DARABKIN. | D-ribulokinase | 0 | Not detected | -0.154 | -0.467 | 0.160 | 0.336 |
| DEHYDDEO | 2-dehydro-3-deoxygalacton | 0 | Not detected | 0.163 | -0.055 | 0.381 | 0.143 |
| DEHYDDEO | 2-dehydro-3-deoxy-6-phos | 0 | Not detected | 0.037 | -0.161 | 0.234 | 0.717 |
| DEOXYGLU | 2-dehydro-3-deoxyglucono | 0 | Not detected | 0.110 | -0.099 | 0.320 | 0.302 |
| DEOXYRIB | deoxyribose-phosphate ald | 0 | Not detected | 0.177 | -0.013 | 0.368 | 0.068 |
| DIENOYLCO | 2,4-dienoyl-CoA reductase | 0 | Not detected | 0.217 | -0.048 | 0.483 | 0.109 |
| DIHYDROF | dihydrofolate synthetase | 0 | Not detected | 0.191 | -0.033 | 0.415 | 0.095 |
| DIHYDROO | dihydroorotate dehydrogen | 0 | Not detected | -0.065 | -0.404 | 0.273 | 0.705 |
| DLACTDEH | D-lactate dehydrogenase (q | 0 | Not detected | 0.050 | -0.180 | 0.280 | 0.672 |
| DMK.ENZR | 1,4-dihydroxy-2-naphthoat | 0 | Not detected | 0.197 | -0.030 | 0.423 | 0.089 |
| DSERDEAM | D-serine ammonia-lyase | 0 | Not detected | -0.019 | -0.285 | 0.246 | 0.888 |
| DTDPDEHY | dTDP-4-dehydrorhamnose | 0 | Not detected | -0.106 | -0.270 | 0.058 | 0.207 |
| DTDPGLUC | dTDP-glucose 4,6-dehydra | 0 | Not detected | -0.152 | -0.318 | 0.015 | 0.074 |
| ENTC.ENZR | isochorismate synthase | 0 | Not detected | 0.083 | -0.118 | 0.285 | 0.418 |
| ENZRXN.10 | L-cysteine-S-conjugate thio | 0 | Not detected | -0.108 | -0.380 | 0.165 | 0.439 |
| ENZRXN.11 | imidazolone-5-propionate h | 0 | Not detected | -0.042 | -0.325 | 0.242 | 0.773 |
| ENZRXN.11 | ammonia-dependent carba | 0 | Not detected | 0.037 | -0.186 | 0.259 | 0.748 |
| ENZRXN.11 | cystathionine &gamma;-lya | 0 | Not detected | -0.202 | -0.513 | 0.110 | 0.205 |
| ENZRXN.12 | citrate synthase | 0 | Not detected | -0.116 | -0.497 | 0.265 | 0.552 |
| ENZRXN.12 | citrate hydro-lyase (cis-aco | 0 | Not detected | -0.215 | -0.572 | 0.142 | 0.239 |
| ENZRXN.12 | D-threo-isocitrate hydro-ly | 0 | Not detected | -0.215 | -0.572 | 0.142 | 0.239 |
| ENZRXN.12 | malate dehydrogenase | 0 | Not detected | -0.120 | -0.472 | 0.232 | 0.505 |
| ENZRXN.12 | 1-deoxy-D-xylulose 5-phos | 0 | Not detected | -0.134 | -0.330 | 0.061 | 0.178 |
| ENZRXN.12 | Pfl-activating enzyme | 0 | Not detected | -0.190 | -0.497 | 0.117 | 0.225 |
| ENZRXN.12 | phenylacetate-CoA ligase | 0 | Not detected | -0.184 | -0.372 | 0.004 | 0.056 |
| ENZRXN.12 | xylitol dehydrogenase | 0 | Not detected | -0.125 | -0.468 | 0.218 | 0.474 |
| ENZRXN.12 | UDP-N-acetylenolpyruvoyl | 0 | Not detected | -0.156 | -0.437 | 0.126 | 0.278 |
| ENZRXN.12 | UDP-N-acetylmuramoyl-L- | 0 | Not detected | -0.249 | -0.614 | 0.117 | 0.183 |
| ENZRXN.12 | 3,5-diaminohexanoate dehy | 0 | Not detected | -0.093 | -0.401 | 0.215 | 0.553 |
| ENZRXN.12 | 3-aminobutyryl-CoA deam | 0 | Not detected | -0.309 | -0.637 | 0.019 | 0.065 |
| ENZRXN.13 | thiosulfate reductase (cytoc | 0 | Not detected | -0.324 | -0.622 | -0.026 | 0.033 |
| ENZRXN.13 | Enzyme IIscr | 0 | Not detected | 0.049 | -0.279 | 0.377 | 0.771 |
| ENZRXN.13 | Enzyme IIscr | 0 | Not detected | -0.299 | -0.551 | -0.048 | 0.020 |
| ENZRXN.13 | fructokinase | 0 | Not detected | 0.010 | -0.203 | 0.224 | 0.924 |
| ENZRXN.13 | phosphoribosyl-formimino | 0 | Not detected | -0.156 | -0.358 | 0.046 | 0.131 |
| ENZRXN.13 | imidazoleglycerolphosphat | 0 | Not detected | -0.322 | -0.592 | -0.052 | 0.020 |
| ENZRXN.13 | sucrose phosphorylase | 0 | Not detected | -0.099 | -0.478 | 0.279 | 0.607 |
| ENZRXN.13 | phosphoserine aminotransf | 0 | Not detected | -0.162 | -0.335 | 0.012 | 0.069 |
| ENZRXN.13 | phosphoglucose isomerase | 0 | Not detected | -0.304 | -0.627 | 0.020 | 0.066 |
| ENZRXN.13 | 3-phosphoglycerate kinase | 0 | Not detected | -0.046 | -0.219 | 0.127 | 0.601 |
| ENZRXN.13 | myo-inosose-2 dehydratase | 0 | Not detected | -0.084 | -0.351 | 0.182 | 0.536 |
| ENZRXN.13 | glucose-6-phosphate isome | 0 | Not detected | -0.201 | -0.533 | 0.132 | 0.237 |
| ENZRXN.13 | fructokinase | 0 | Not detected | 0.008 | -0.268 | 0.284 | 0.953 |

| ENZRXN.13 | glucose-6-phosphate isome | 0 | Not detected | -0.100 | -0.259 | 0.059 | 0.219 |
| --- | --- | --- | --- | --- | --- | --- | --- |
| ENZRXN.13 | phosphotransacetylase | 0 | Not detected | -0.021 | -0.268 | 0.226 | 0.867 |
| ENZRXN.14 | UDP-N-acetylglucosamine | 0 | Not detected | -0.039 | -0.428 | 0.350 | 0.845 |
| ENZRXN.14 | UDP-galactose 4-epimeras | 0 | Not detected | -0.039 | -0.428 | 0.350 | 0.845 |
| ENZRXN.14 | cobalt precorrin-4 (C11)-m | 0 | Not detected | -0.073 | -0.248 | 0.102 | 0.414 |
| ENZRXN.14 | cobalt-precorrin-3 (C17)-m | 0 | Not detected | -0.170 | -0.477 | 0.136 | 0.277 |
| ENZRXN.14 | GDP-D-mannose pyrophos | 0 | Not detected | -0.214 | -0.492 | 0.065 | 0.133 |
| ENZRXN.14 | phosphomannose isomeras | 0 | Not detected | -0.214 | -0.492 | 0.065 | 0.133 |
| ENZRXN.14 | succinyl-CoA:CoA transfer | 0 | Not detected | -0.003 | -0.269 | 0.264 | 0.985 |
| ENZRXN.14 | 4-hydroxybutyrate coenzym | 0 | Not detected | -0.298 | -0.619 | 0.023 | 0.069 |
| ENZRXN.14 | crotonase | 0 | Not detected | -0.186 | -0.530 | 0.157 | 0.287 |
| ENZRXN.14 | butyryl-CoA dehydrogenas | 0 | Not detected | -0.221 | -0.480 | 0.037 | 0.093 |
| ENZRXN.14 | phosphate acetyltransferase | 0 | Not detected | -0.061 | -0.227 | 0.104 | 0.469 |
| ENZRXN.14 | acetate kinase | 0 | Not detected | -0.237 | -0.539 | 0.066 | 0.125 |
| ENZRXN.14 | GDP-Î±-D-mannose 4,6-de | 0 | Not detected | 0.079 | -0.233 | 0.391 | 0.621 |
| ENZRXN.14 | aconitase | 0 | Not detected | -0.060 | -0.257 | 0.137 | 0.553 |
| ENZRXN.14 | phosphoenolpyruvate phos | 599 | Not detected | -0.418 | -0.780 | -0.055 | 0.024 |
| ENZRXN.14 | naphthoyl-CoA synthase | 0 | Not detected | -0.047 | -0.226 | 0.131 | 0.603 |
| ENZRXN.14 | N-acetylhexosamine kinase | 0 | Not detected | -0.283 | -0.620 | 0.054 | 0.100 |
| ENZRXN.14 | glutamyl-tRNAGln synthet | 0 | Not detected | 0.140 | -0.207 | 0.486 | 0.429 |
| ENZRXN.15 | glutamyl-tRNAGlu synthet | 0 | Not detected | 0.140 | -0.207 | 0.486 | 0.429 |
| ENZRXN.15 | 1-acylglycerol-3-phosphate | 0 | Not detected | -0.094 | -0.351 | 0.163 | 0.474 |
| ENZRXN.15 | biotin sulfoxide reductase | 0 | Not detected | 0.096 | -0.169 | 0.361 | 0.477 |
| ENZRXN.15 | malonate decarboxylase ac | 0 | Not detected | -0.067 | -0.296 | 0.162 | 0.566 |
| ENZRXN.15 | acyl-carrier protein: acetate | 0 | Not detected | 0.024 | -0.318 | 0.367 | 0.890 |
| ENZRXN.15 | acetyl-[acyl-carrier protein] | 0 | Not detected | -0.077 | -0.407 | 0.252 | 0.645 |
| ENZRXN.15 | carboxybiotin decarboxylas | 0 | Not detected | -0.220 | -0.482 | 0.042 | 0.100 |
| ENZRXN.15 | N-succinylornithine carbam | 0 | Not detected | -0.035 | -0.220 | 0.151 | 0.715 |
| ENZRXN.15 | 5-monosubstituted-hydanto | 9 | Not detected | 0.143 | -0.178 | 0.464 | 0.383 |
| ENZRXN.15 | phosphoglycerate mutase | 0 | Not detected | -0.040 | -0.372 | 0.291 | 0.812 |
| ENZRXN.15 | 2-keto-3-deoxy-D-glycero- | 0 | Not detected | -0.181 | -0.571 | 0.209 | 0.362 |
| ENZRXN.15 | 2-keto-3-deoxy-D-glycero- | 0 | Not detected | -0.083 | -0.447 | 0.281 | 0.656 |
| ENZRXN.16 | Î²-D-galactosyl-(1->4)-L-rh | 0 | Not detected | -0.424 | -0.784 | -0.064 | 0.021 |
| ENZRXN.17 | UDP-N-acetylglucosamine | 0 | Not detected | -0.176 | -0.521 | 0.169 | 0.318 |
| ENZRXN.17 | UDP-galactopyranose muta | 0 | Not detected | -0.256 | -0.633 | 0.120 | 0.182 |
| ENZRXN.17 | 3-dehydroquinate dehydrat | 0 | Not detected | -0.180 | -0.470 | 0.111 | 0.226 |
| ENZRXN.17 | UDP-N-acetylglucosamine | 0 | Not detected | -0.043 | -0.257 | 0.171 | 0.693 |
| ENZRXN.17 | UDP-N-acetylmuramoyl--L | 0 | Not detected | -0.235 | -0.546 | 0.077 | 0.140 |
| ENZRXN.17 | UDP-N-acetylglucosamine | 0 | Not detected | -0.218 | -0.466 | 0.030 | 0.086 |
| ENZRXN.17 | UDP-N-acetylglucosamine | 0 | Not detected | -0.071 | -0.284 | 0.143 | 0.515 |
| ENZRXN.17 | undecaprenyl-pyrophospho | 0 | Not detected | -0.179 | -0.537 | 0.180 | 0.329 |
| ENZRXN.17 | asparagine synthase | 0 | Not detected | -0.077 | -0.437 | 0.283 | 0.676 |
| ENZRXN.17 | undecaprenyl-pyrophospho | 0 | Not detected | -0.179 | -0.537 | 0.180 | 0.329 |
| ENZRXN.17 | D-sedoheptulose 7-phosph | 0 | Not detected | -0.248 | -0.575 | 0.078 | 0.136 |

| ENZRXN.17 | D-glycero-Î±-D-manno-hep | 0 | Not detected | -0.045 | -0.408 | 0.318 | 0.809 |
| --- | --- | --- | --- | --- | --- | --- | --- |
| ENZRXN.17 | lipoate-ATP adenylate tran | 0 | Not detected | 0.030 | -0.162 | 0.222 | 0.756 |
| ENZRXN.17 | pimeloyl-[acp] methyl ester | 0 | Not detected | 0.081 | -0.117 | 0.279 | 0.423 |
| ENZRXN.17 | (2Z,6E)-farnesyl diphospha | 0 | Not detected | -0.303 | -0.653 | 0.046 | 0.089 |
| ENZRXN.18 | 3-(hydroxyamino)phenol m | 0 | Not detected | 0.071 | -0.279 | 0.421 | 0.691 |
| ENZRXN.18 | butanal dehydrogenase | 0 | Not detected | -0.123 | -0.298 | 0.052 | 0.167 |
| ENZRXN.18 | butanol dehydrogenase | 0 | Not detected | -0.123 | -0.298 | 0.052 | 0.167 |
| ENZRXN.18 | glycerol-2-phosphotransfer | 0 | Not detected | -0.114 | -0.401 | 0.173 | 0.435 |
| ENZRXN.18 | DsrC-trisulfide reductase | 0 | Not detected | -0.173 | -0.485 | 0.139 | 0.278 |
| ENZRXN.18 | dCMP kinase | 0 | Not detected | -0.131 | -0.393 | 0.130 | 0.325 |
| ENZRXN.18 | 3-hydroxy-trans-cinnamate | 0 | Not detected | 0.229 | 0.003 | 0.454 | 0.047 |
| ENZRXN.18 | 1-deoxy-D-xylulose 5-phos | 0 | Not detected | -0.228 | -0.576 | 0.120 | 0.199 |
| ENZRXN.18 | nitric oxide reductase | 0 | Not detected | -0.127 | -0.384 | 0.129 | 0.330 |
| ENZRXN.18 | rhamnogalacturonan endol | 0 | Not detected | -0.063 | -0.353 | 0.228 | 0.673 |
| ENZRXN.18 | unsaturated chondroitin 6-s | 0 | Not detected | 0.218 | -0.073 | 0.509 | 0.142 |
| ENZRXN.18 | legionaminate synthase | 0 | Not detected | -0.052 | -0.350 | 0.246 | 0.735 |
| ENZRXN.18 | rhamnogalacturonan exolya | 0 | Not detected | -0.178 | -0.475 | 0.119 | 0.240 |
| ENZRXN.18 | isoamylase | 0 | Not detected | -0.042 | -0.362 | 0.279 | 0.799 |
| ENZRXN.18 | formate dehydrogenase | 0 | Not detected | -0.018 | -0.319 | 0.282 | 0.905 |
| ENZRXN.19 | cellobiose phosphorylase | 0 | Not detected | -0.085 | -0.272 | 0.103 | 0.376 |
| ENZRXN.19 | GTP 3',8'-cyclase | 152 | Not detected | 0.340 | 0.118 | 0.563 | 0.003 |
| ENZRXN.19 | endo-Î±-N-acetylgalactosa | 0 | Not detected | -0.049 | -0.416 | 0.319 | 0.796 |
| ENZRXN.19 | trans-2-enoyl-CoA reductas | 0 | Not detected | 0.089 | -0.188 | 0.366 | 0.529 |
| ENZRXN.19 | crotonyl-CoA reductase | 0 | Not detected | 0.089 | -0.188 | 0.366 | 0.529 |
| ENZRXN.19 | trans-2-hexenoyl-CoA redu | 0 | Not detected | 0.089 | -0.188 | 0.366 | 0.529 |
| ENZRXN.19 | hexanal dehydrogenase | 0 | Not detected | -0.123 | -0.298 | 0.052 | 0.167 |
| ENZRXN.19 | hexanol dehydrogenase | 0 | Not detected | -0.123 | -0.298 | 0.052 | 0.167 |
| ENZRXN.19 | carboxy-adenylated-[ThiS s | 0 | Not detected | 0.113 | -0.142 | 0.368 | 0.387 |
| ENZRXN.19 | molybdopterin synthase sul | 0 | Not detected | 0.036 | -0.163 | 0.235 | 0.721 |
| ENZRXN.19 | N,N'-diacetylchitobiose ph | 0 | Not detected | -0.419 | -0.744 | -0.093 | 0.012 |
| ENZRXN.19 | 12Î±-hydroxy-3-oxochol-4, | 0 | Not detected | -0.417 | -0.738 | -0.096 | 0.011 |
| ENZRXN.19 | dTDP-glucose 4,6-dehydra | 0 | Not detected | -0.346 | -0.628 | -0.065 | 0.016 |
| ENZRXN.19 | glucose-1-phosphate thymi | 0 | Not detected | -0.083 | -0.249 | 0.083 | 0.328 |
| ENZRXN.19 | dTDP-3-amino-3,6-dideox | 0 | Not detected | -0.168 | -0.520 | 0.184 | 0.350 |
| ENZRXN.19 | 2-oxoglutarate decarboxyla | 0 | Not detected | -0.310 | -0.668 | 0.048 | 0.090 |
| ENZRXN.20 | deoxyribonuclease IV | 0 | Not detected | -0.171 | -0.399 | 0.057 | 0.142 |
| ENZRXN.20 | uracil-DNA glycosylase | 0 | Not detected | -0.110 | -0.260 | 0.040 | 0.151 |
| ENZRXN.20 | DNA glycosylase | 305 | Not detected | -0.267 | -0.581 | 0.047 | 0.096 |
| ENZRXN.20 | DNA-formamidopyrimidin | 0 | Not detected | 0.048 | -0.165 | 0.260 | 0.661 |
| ENZRXN.20 | adenine glycosylase | 0 | Not detected | 0.142 | -0.080 | 0.364 | 0.211 |
| ENZRXN.20 | glycerol-3-phosphate dehyd | 0 | Not detected | -0.312 | -0.628 | 0.004 | 0.053 |
| ENZRXN.20 | 3-isopropylmalate dehydrat | 0 | Not detected | -0.270 | -0.641 | 0.101 | 0.154 |
| ENZRXN.20 | (S)-NADPHX dehydratase | 0 | Not detected | 0.040 | -0.201 | 0.281 | 0.745 |
| ENZRXN.20 | NADPHX epimerase | 0 | Not detected | 0.040 | -0.201 | 0.281 | 0.745 |

| ENZRXN.20 | butanol dehydrogenase | 442 | Not detected | -0.155 | -0.330 | 0.019 | 0.082 |
| --- | --- | --- | --- | --- | --- | --- | --- |
| ENZRXN.20 | CoA-dependent propionald | 0 | Not detected | -0.116 | -0.387 | 0.155 | 0.402 |
| ENZRXN.20 | phosphate propanoyltransfe | 0 | Not detected | -0.157 | -0.436 | 0.122 | 0.271 |
| ENZRXN.20 | phosphate acetyltransferase | 0 | Not detected | -0.157 | -0.436 | 0.122 | 0.271 |
| ENZRXN.20 | propionate kinase | 0 | Not detected | 0.088 | -0.169 | 0.345 | 0.502 |
| ENZRXN.20 | thymine oxygenase | 0 | Not detected | 0.029 | -0.168 | 0.227 | 0.771 |
| ENZRXN.20 | hydroxybutyryl-coA dehyd | 0 | Not detected | -0.124 | -0.340 | 0.092 | 0.261 |
| ENZRXN.20 | biotin carboxylase | 0 | Not detected | -0.132 | -0.433 | 0.168 | 0.388 |
| ENZRXN.20 | N,N'-diacetylbacilliosamin | 0 | Not detected | -0.222 | -0.558 | 0.113 | 0.194 |
| ENZRXN.20 | carboxyspermidine dehydro | 0 | Not detected | -0.054 | -0.215 | 0.107 | 0.512 |
| ENZRXN.20 | N-carbamoylputrescine am | 0 | Not detected | -0.003 | -0.190 | 0.183 | 0.972 |
| ENZRXN.20 | 4,4'-diaponeurosporenal de | 0 | Not detected | -0.053 | -0.395 | 0.290 | 0.763 |
| ENZRXN.20 | superoxide dismutase | 0 | Not detected | -0.216 | -0.532 | 0.100 | 0.180 |
| ENZRXN.20 | UDP-N-acetyl-2-amino-2-d | 0 | Not detected | -0.452 | -0.807 | -0.097 | 0.013 |
| ENZRXN.20 | UDP-2,3-diacetamido-2,3- | 0 | Not detected | -0.397 | -0.756 | -0.038 | 0.030 |
| ENZRXN.20 | enoyl-[acyl-carrier-protein] | 0 | Not detected | -0.057 | -0.400 | 0.287 | 0.747 |
| ENZRXN.20 | enoyl-[acyl-carrier-protein] | 0 | Not detected | -0.057 | -0.400 | 0.287 | 0.747 |
| ENZRXN.20 | crotonyl-[acyl-carrier-prote | 0 | Not detected | -0.057 | -0.400 | 0.287 | 0.747 |
| ENZRXN.20 | D-fructose-6-phosphate cyt | 0 | Not detected | -0.104 | -0.442 | 0.233 | 0.545 |
| ENZRXN.21 | acetaldehyde dehydrogenas | 0 | Not detected | -0.177 | -0.465 | 0.111 | 0.228 |
| ENZRXN.21 | homoserine O-acetyltransfe | 451 | Not detected | -0.301 | -0.589 | -0.013 | 0.041 |
| ENZRXN.21 | methylthioribose-1-phosph | 0 | Not detected | -0.255 | -0.586 | 0.076 | 0.131 |
| ENZRXN.21 | oligosaccharide reducing-e | 0 | Not detected | -0.158 | -0.416 | 0.100 | 0.230 |
| ENZRXN.21 | Î²-L-arabinofuranosidase | 0 | Not detected | -0.069 | -0.417 | 0.279 | 0.698 |
| ENZRXN.21 | uridine kinase | 0 | Not detected | 0.136 | -0.162 | 0.434 | 0.370 |
| ENZRXN.21 | cytidine kinase | 0 | Not detected | 0.136 | -0.162 | 0.434 | 0.370 |
| ENZRXN.21 | urocanate reductase | 0 | Not detected | 0.030 | -0.331 | 0.392 | 0.869 |
| ENZRXN.21 | 3D-(3,5/4)-trihydroxycyclo | 0 | Not detected | -0.282 | -0.518 | -0.046 | 0.020 |
| ENZRXN.21 | myo-inositol 2-dehydrogen | 0 | Not detected | -0.228 | -0.505 | 0.049 | 0.107 |
| ENZRXN.21 | D-mannonate dehydrogena | 0 | Not detected | -0.095 | -0.282 | 0.093 | 0.323 |
| ENZRXN.21 | D-mannonate dehydratase | 0 | Not detected | -0.133 | -0.305 | 0.039 | 0.129 |
| ENZRXN.21 | 2-keto-3-deoxygluconate ki | 0 | Not detected | -0.070 | -0.235 | 0.096 | 0.409 |
| ENZRXN.21 | [sulfur carrier protein CysO | 0 | Not detected | -0.107 | -0.425 | 0.210 | 0.507 |
| ENZRXN.21 | N-acetyl-Î±-D-glucosaminy | 0 | Not detected | -0.061 | -0.412 | 0.290 | 0.733 |
| ENZRXN.21 | glucose-1-phosphate thymi | 0 | Not detected | -0.128 | -0.392 | 0.135 | 0.340 |
| ENZRXN.21 | dTDP-glucose 4,6-dehydra | 0 | Not detected | -0.209 | -0.477 | 0.059 | 0.126 |
| ENZRXN.21 | dTDP-4-amino-4,6-dideox | 0 | Not detected | -0.257 | -0.628 | 0.115 | 0.176 |
| ENZRXN.21 | UDP-glucose 6-dehydroge | 0 | Not detected | -0.163 | -0.485 | 0.159 | 0.321 |
| ENZRXN.21 | UDP-N-acetylglucosamine | 0 | Not detected | -0.202 | -0.503 | 0.098 | 0.187 |
| ENZRXN.21 | UDP-2-acetamido-2,6-dide | 0 | Not detected | -0.171 | -0.525 | 0.182 | 0.343 |
| ENZRXN.21 | UDP-N-acetyl-Î²-L-rhamno | 771 | 0.6 | -0.444 | -0.774 | -0.114 | 0.008 |
| ENZRXN.22 | UDP-N-acetylglucosamine | 476 | Not detected | -0.271 | -0.599 | 0.058 | 0.106 |
| ENZRXN.22 | Î²-L-arabinobiosidase | 0 | Not detected | -0.137 | -0.539 | 0.265 | 0.505 |
| ENZRXN.22 | butyryl-CoA dehydrogenas | 0 | Not detected | -0.042 | -0.222 | 0.138 | 0.648 |

| ENZRXN.22 | phosphoenolpyruvate carbo | 0 | Not detected | -0.004 | -0.251 | 0.243 | 0.976 |
| --- | --- | --- | --- | --- | --- | --- | --- |
| ENZRXN.22 | phosphoenolpyruvate carbo | 0 | Not detected | -0.004 | -0.251 | 0.243 | 0.976 |
| ENZRXN.22 | L-tyrosine 3-monooxygena | 14 | Not detected | 0.163 | -0.055 | 0.381 | 0.144 |
| ENZRXN.22 | 4-coumarate 3-monooxyge | 14 | Not detected | 0.163 | -0.055 | 0.381 | 0.144 |
| ENZRXN.22 | 4-tyrosol 3-monooxygenas | 14 | Not detected | 0.163 | -0.055 | 0.381 | 0.144 |
| ENZRXN.22 | succinyl-CoA:acetate CoA- | 786 | 0.6 | -0.555 | -0.912 | -0.198 | 0.002 |
| ENZRXN.22 | propionyl-CoA:succinate C | 786 | 0.6 | -0.555 | -0.912 | -0.198 | 0.002 |
| ENZRXN.22 | N-acetyl-Î²-neuraminate:H+ | 0 | Not detected | -0.013 | -0.239 | 0.214 | 0.913 |
| ENZRXN.22 | 1,4-Î²-mannanase | 0 | Not detected | -0.093 | -0.415 | 0.230 | 0.574 |
| ENZRXN.22 | mannobiose 2-epimerase | 0 | Not detected | -0.121 | -0.399 | 0.157 | 0.392 |
| ENZRXN.22 | cellobiose 2-epimerase | 0 | Not detected | -0.121 | -0.399 | 0.157 | 0.392 |
| ENZRXN.22 | cellobiose 2-epimerase | 0 | Not detected | -0.233 | -0.544 | 0.077 | 0.141 |
| ENZRXN.22 | UDP-acetamido-4-amino-6 | 0 | Not detected | -0.264 | -0.619 | 0.090 | 0.144 |
| ENZRXN.22 | 2-oxo-4-methylthiobutanoa | 0 | Not detected | -0.151 | -0.430 | 0.127 | 0.287 |
| ENZRXN.23 | CoA-dependent (R)-lactald | 0 | Not detected | -0.116 | -0.387 | 0.155 | 0.402 |
| ENZRXN.23 | CoA-dependent (S)-lactald | 0 | Not detected | -0.116 | -0.387 | 0.155 | 0.402 |
| ENZRXN.23 | (R)-lactaldehyde reductase | 0 | Not detected | 0.007 | -0.188 | 0.202 | 0.944 |
| ENZRXN.23 | (S)-lactaldehyde reductase | 0 | Not detected | 0.007 | -0.188 | 0.202 | 0.944 |
| ENZRXN.23 | (R)-2,3-dihydroxy-3-methy | 0 | Not detected | -0.124 | -0.287 | 0.040 | 0.140 |
| ENZRXN.23 | (R)-2,3-dihydroxy-3-methy | 0 | Not detected | -0.124 | -0.287 | 0.040 | 0.140 |
| ENZRXN.23 | (R)-2,3-dihydroxy-3-methy | 0 | Not detected | -0.216 | -0.526 | 0.093 | 0.171 |
| ENZRXN.23 | (R)-2,3-dihydroxy-3-methy | 0 | Not detected | -0.216 | -0.526 | 0.093 | 0.171 |
| ENZRXN.23 | Fe-coproporphyrin synthas | 0 | Not detected | -0.273 | -0.600 | 0.054 | 0.102 |
| ENZRXN.23 | bacimethrin diphosphokina | 0 | Not detected | -0.190 | -0.412 | 0.031 | 0.093 |
| ENZRXN.23 | L-galactose dehydrogenase | 0 | Not detected | -0.068 | -0.337 | 0.201 | 0.621 |
| ENZRXN.23 | L-galactonate 5-dehydroge | 0 | Not detected | -0.066 | -0.331 | 0.198 | 0.623 |
| ENZRXN.23 | N-acetylneuraminate lyase | 0 | Not detected | 0.031 | -0.212 | 0.275 | 0.801 |
| ENZRXN.23 | N-acetylglucosamine kinas | 0 | Not detected | -0.045 | -0.221 | 0.132 | 0.619 |
| ENZRXN.23 | 7-oxolithocholate reductase | 0 | Not detected | 0.027 | -0.257 | 0.311 | 0.852 |
| ENZRXN.23 | 7-oxolithocholate reductase | 0 | Not detected | 0.027 | -0.257 | 0.311 | 0.852 |
| ENZRXN.23 | GDP-6-deoxy-4-keto-D-ara | 0 | Not detected | -0.193 | -0.440 | 0.054 | 0.126 |
| ENZRXN.23 | UDP-N-acetylglucosamine | 0 | Not detected | -0.121 | -0.388 | 0.147 | 0.378 |
| ENZRXN.23 | UDP-N-acetylgalactosamin | 0 | Not detected | -0.121 | -0.388 | 0.147 | 0.378 |
| ENZRXN.23 | Kdo2-lipid A phosphotrans | 0 | Not detected | 0.031 | -0.235 | 0.296 | 0.821 |
| ENZRXN.23 | Kdo2-lipid A phosphoetha | 0 | Not detected | 0.112 | -0.137 | 0.362 | 0.378 |
| ENZRXN.23 | phosphomannomutase | 0 | Not detected | 0.027 | -0.296 | 0.351 | 0.869 |
| ENZRXN.23 | phosphoglucomutase | 0 | Not detected | 0.027 | -0.296 | 0.351 | 0.869 |
| ENZRXN.23 | heparin lyase | 0 | Not detected | 0.037 | -0.408 | 0.482 | 0.869 |
| ENZRXN.23 | heparan sulfate lyase | 0 | Not detected | 0.037 | -0.408 | 0.482 | 0.869 |
| ENZRXN.23 | heparin lyase | 0 | Not detected | -0.243 | -0.567 | 0.081 | 0.142 |
| ENZRXN.23 | unsaturated hyaluronan dis | 0 | Not detected | 0.218 | -0.073 | 0.509 | 0.142 |
| ENZRXN.23 | unsaturated chondroitin dis | 0 | Not detected | 0.218 | -0.073 | 0.509 | 0.142 |
| ENZRXN.23 | 4-deoxy-Î²-L-erythro-hex-4 | 0 | Not detected | 0.020 | -0.320 | 0.360 | 0.907 |
| ENZRXN.23 | 4-deoxy-Î²-D-gluc-4-enuro | 0 | Not detected | 0.020 | -0.320 | 0.360 | 0.907 |

| ENZRXN.23 | 4-deoxy-Î²-D-gluc-4-enuro | 0 | Not detected | 0.020 | -0.320 | 0.360 | 0.907 |
| --- | --- | --- | --- | --- | --- | --- | --- |
| ENZRXN.23 | 4-deoxy-Î²-L-erythro-hex-4 | 0 | Not detected | 0.020 | -0.320 | 0.360 | 0.907 |
| ENZRXN.23 | (5Z)-3-oxo-tetradec-5-enoy | 0 | Not detected | -0.102 | -0.274 | 0.071 | 0.249 |
| ENZRXN.23 | (5Z)-3-oxo-tetradec-5-enoy | 0 | Not detected | -0.217 | -0.509 | 0.074 | 0.145 |
| ENZRXN.23 | (2E,5Z)-tetradeca-2,5-dien | 0 | Not detected | -0.248 | -0.507 | 0.012 | 0.062 |
| ENZRXN.23 | (3R)-3-hydroxydecanoyl-[a | 0 | Not detected | -0.086 | -0.331 | 0.160 | 0.495 |
| ENZRXN.23 | (2E)-dec-2-enoyl-[acp] iso | 0 | Not detected | -0.086 | -0.331 | 0.160 | 0.495 |
| ENZRXN.23 | (5Z)-3-oxo-dodec-5-enoyl- | 0 | Not detected | -0.141 | -0.306 | 0.024 | 0.094 |
| ENZRXN.23 | (7Z)-3-oxo-hexadec-7-eno | 0 | Not detected | -0.102 | -0.274 | 0.071 | 0.249 |
| ENZRXN.23 | (9Z)-3-oxo-octadec-9-enoy | 0 | Not detected | -0.102 | -0.274 | 0.071 | 0.249 |
| ENZRXN.23 | (7Z)-3-oxo-hexadec-7-eno | 0 | Not detected | -0.217 | -0.509 | 0.074 | 0.145 |
| ENZRXN.23 | (9Z)-3-oxo-octadec-9-enoy | 0 | Not detected | -0.217 | -0.509 | 0.074 | 0.145 |
| ENZRXN.23 | (11Z)-3-oxo-icos-11-enoyl | 0 | Not detected | -0.217 | -0.509 | 0.074 | 0.145 |
| ENZRXN.23 | (11Z)-3-oxo-icos-11-enoyl | 0 | Not detected | -0.102 | -0.274 | 0.071 | 0.249 |
| ENZRXN.23 | (2E,7Z)-hexadeca-2,7-dien | 0 | Not detected | -0.248 | -0.507 | 0.012 | 0.062 |
| ENZRXN.23 | (2E,9Z)-octadeca-2,9-dieno | 0 | Not detected | -0.248 | -0.507 | 0.012 | 0.062 |
| ENZRXN.23 | (2E,11Z)-icosa-2,11-dieno | 0 | Not detected | -0.248 | -0.507 | 0.012 | 0.062 |
| ENZRXN.23 | lactose 2-epimerase | 0 | Not detected | -0.233 | -0.544 | 0.077 | 0.141 |
| ENZRXN.23 | 8-amino-3,8-dideoxy-mann | 0 | Not detected | -0.130 | -0.435 | 0.175 | 0.403 |
| ENZRXN.24 | scaffold protein for iron-su | 0 | Not detected | 0.002 | -0.288 | 0.291 | 0.991 |
| ENZRXN.24 | lactate racemase | 0 | Not detected | -0.108 | -0.381 | 0.165 | 0.440 |
| ENZRXN.24 | UDP-4-amino-4,6-dideoxy | 0 | Not detected | -0.008 | -0.393 | 0.377 | 0.967 |
| ENZRXN.24 | guanosine phosphorylase | 0 | Not detected | -0.007 | -0.344 | 0.329 | 0.966 |
| ENZRXN.24 | deoxycholate 3Î±-dehydrog | 0 | Not detected | -0.118 | -0.362 | 0.126 | 0.343 |
| ENZRXN.24 | deoxycholate 3Î±-dehydrog | 0 | Not detected | 0.206 | -0.139 | 0.551 | 0.242 |
| ENZRXN.24 | 3-oxochol-4,6-dien-24-oyl- | 0 | Not detected | -0.417 | -0.738 | -0.096 | 0.011 |
| ENZRXN.24 | lithocholate 3Î±-dehydroge | 0 | Not detected | 0.206 | -0.139 | 0.551 | 0.242 |
| ENZRXN.24 | chenodeoxycholate 3Î±-deh | 0 | Not detected | 0.206 | -0.139 | 0.551 | 0.242 |
| ENZRXN.24 | cholate 3Î±-dehydrogenase | 0 | Not detected | 0.206 | -0.139 | 0.551 | 0.242 |
| ENZRXN.24 | lithocholate 3Î±-dehydroge | 0 | Not detected | -0.118 | -0.362 | 0.126 | 0.343 |
| ENZRXN.24 | cholate 3Î±-dehydrogenase | 0 | Not detected | -0.118 | -0.362 | 0.126 | 0.343 |
| ENZRXN.24 | chenodeoxycholate 3Î±-deh | 0 | Not detected | -0.118 | -0.362 | 0.126 | 0.343 |
| ENZRXN.24 | Î²-1,2-mannosidase | 0 | Not detected | -0.219 | -0.512 | 0.074 | 0.143 |
| ENZRXN.24 | L-galacto-1,5-lactonase | 0 | Not detected | -0.022 | -0.280 | 0.236 | 0.865 |
| ENZRXN.24 | succinate:malate antiport | 0 | Not detected | 0.065 | -0.229 | 0.360 | 0.664 |
| ENZRXN.24 | succinate:L-aspartate antip | 0 | Not detected | 0.065 | -0.229 | 0.360 | 0.664 |
| ENZRXN.24 | malate:succinate antiporter | 0 | Not detected | -0.169 | -0.426 | 0.089 | 0.199 |
| ENZRXN.24 | L-aspartate:succinate antip | 0 | Not detected | -0.169 | -0.426 | 0.089 | 0.199 |
| ENZRXN.24 | dihydroorotate dehydrogen | 0 | Not detected | -0.065 | -0.404 | 0.273 | 0.705 |
| ENZRXN.24 | methionine &gamma;-lyase | 654 | Not detected | -0.470 | -0.761 | -0.179 | 0.002 |
| ENZRXN.24 | [HypE]-S-carboxamide deh | 0 | Not detected | 0.065 | -0.164 | 0.295 | 0.578 |
| ENZRXN.25 | Î±-D-ribose-1-(2-N-acetam | 0 | Not detected | 0.153 | -0.076 | 0.381 | 0.190 |
| ENZRXN.25 | carbon-phosphorous lyase | 0 | Not detected | -0.131 | -0.388 | 0.126 | 0.318 |
| ENZRXN.25 | Î±-D-ribose-1-[N-(phospho | 0 | Not detected | -0.131 | -0.373 | 0.110 | 0.287 |

| ENZRXN.25 | Î±-D-ribose-1-[N-(phospho | 0 | Not detected | -0.089 | -0.387 | 0.209 | 0.559 |
| --- | --- | --- | --- | --- | --- | --- | --- |
| ENZRXN.25 | adenine phosphoribosyltran | 0 | Not detected | -0.087 | -0.396 | 0.222 | 0.581 |
| ENZRXN.25 | tetracycline 11a-oxygenase | 0 | Not detected | -0.215 | -0.676 | 0.246 | 0.360 |
| ENZRXN.25 | tigecycline 11a-momooxyg | 0 | Not detected | -0.215 | -0.676 | 0.246 | 0.360 |
| ENZRXN.25 | UDP-N-acetylglucosamine | 0 | Not detected | 0.002 | -0.343 | 0.348 | 0.990 |
| ENZRXN.25 | ribitol-5-phosphate cytidyl | 0 | Not detected | -0.192 | -0.537 | 0.153 | 0.275 |
| ENZRXN.25 | ribitol-5-phosphate 1-dehy | 0 | Not detected | -0.379 | -0.705 | -0.052 | 0.023 |
| ENZRXN.25 | ribitol-5-phosphate 1-dehy | 0 | Not detected | -0.287 | -0.639 | 0.066 | 0.111 |
| ENZRXN.25 | UTP--glucose-1-phosphate | 378 | Not detected | -0.325 | -0.668 | 0.018 | 0.064 |
| ENZRXN.25 | undecaprenyl-phosphate 2- | 0 | Not detected | -0.007 | -0.323 | 0.309 | 0.965 |
| ENZRXN.25 | D-alanine--[D-alanyl carrie | 0 | Not detected | -0.033 | -0.371 | 0.304 | 0.846 |
| ENZRXN.25 | PG:teichoic acid D-alanyltr | 0 | Not detected | 0.020 | -0.288 | 0.328 | 0.901 |
| ENZRXN.25 | D-alanine carrier protein:P | 0 | Not detected | 0.023 | -0.279 | 0.324 | 0.882 |
| ENZRXN.25 | 1,2-diacylglycerol 3-Î±-glu | 0 | Not detected | -0.028 | -0.315 | 0.259 | 0.848 |
| ENZRXN.25 | tRNA uridine 4-sulfurtrans | 0 | Not detected | 0.113 | -0.142 | 0.368 | 0.387 |
| ENZRXN.25 | aminobutyraldehyde dehyd | 0 | Not detected | 0.091 | -0.122 | 0.305 | 0.401 |
| ENZRXN.25 | succinate-semialdehyde de | 0 | Not detected | 0.091 | -0.122 | 0.305 | 0.401 |
| ENZRXN.25 | 7,8-dihydro-8-oxoguanine | 0 | Not detected | 0.048 | -0.165 | 0.260 | 0.661 |
| ENZRXN.25 | [sulfur carrier protein TtuB | 0 | Not detected | -0.075 | -0.382 | 0.232 | 0.633 |
| ENZRXN.25 | trans-4-hydroxy-L-proline | 0 | Not detected | -0.125 | -0.338 | 0.089 | 0.252 |
| ENZRXN.25 | menaquinone C8-methyltra | 0 | Not detected | -0.001 | -0.355 | 0.353 | 0.994 |
| ENZRXN.25 | quinol--ferredoxin oxidore | 0 | Not detected | 0.215 | -0.135 | 0.565 | 0.229 |
| ENZRXN.25 | dissimilatory sulfite reducta | 0 | Not detected | 0.058 | -0.307 | 0.424 | 0.755 |
| ENZRXN.25 | L-threonate dehydrogenase | 0 | Not detected | 0.201 | -0.001 | 0.402 | 0.051 |
| ENZRXN.25 | 2-dehydro-D-threonate iso | 0 | Not detected | -0.005 | -0.198 | 0.188 | 0.963 |
| ENZRXN.25 | 2-dehydro-L-threonate isom | 0 | Not detected | -0.005 | -0.198 | 0.188 | 0.963 |
| ENZRXN.25 | 4-phospho-D-erythronate 3 | 0 | Not detected | -0.012 | -0.320 | 0.295 | 0.938 |
| ENZRXN.25 | UDP-N-acetylmuramate--L | 0 | Not detected | -0.007 | -0.357 | 0.344 | 0.971 |
| ENZRXN.25 | methenyltetrahydrofolate c | 0 | Not detected | -0.129 | -0.325 | 0.067 | 0.196 |
| ENZRXN.25 | methylenetetrahydrofolate | 0 | Not detected | -0.129 | -0.325 | 0.067 | 0.196 |
| ENZRXN.25 | deoxyribodipyrimidine pho | 0 | Not detected | 0.072 | -0.152 | 0.297 | 0.527 |
| ENZRXN.25 | deoxyribodipyrimidine pho | 0 | Not detected | 0.072 | -0.152 | 0.297 | 0.527 |
| ENZRXN.25 | pyridinium-3,5-biscarboxyl | 0 | Not detected | -0.101 | -0.332 | 0.130 | 0.393 |
| ENZRXN.26 | acetate kinase | 0 | Not detected | 0.056 | -0.135 | 0.247 | 0.564 |
| ENZRXN.26 | acetaldehyde dehydrogenas | 0 | Not detected | -0.145 | -0.422 | 0.133 | 0.307 |
| ENZRXN.26 | glyoxal reductase | 0 | Not detected | -0.085 | -0.386 | 0.215 | 0.578 |
| ENZRXN.26 | methylglyoxal reductase | 0 | Not detected | -0.085 | -0.386 | 0.215 | 0.578 |
| ENZRXN.26 | 3-oxochol-4-en-24-oyl-Co | 0 | Not detected | -0.417 | -0.738 | -0.096 | 0.011 |
| ENZRXN.26 | 12Î±-hydroxy-3-oxochol-4 | 0 | Not detected | -0.417 | -0.738 | -0.096 | 0.011 |
| ENZRXN.26 | 3-hydroxyhexadecanoyl-Co | 0 | Not detected | -0.014 | -0.196 | 0.167 | 0.876 |
| ENZRXN.26 | diaminohydroxyphosphorib | 0 | Not detected | -0.246 | -0.534 | 0.041 | 0.093 |
| ENZRXN.26 | 5-amino-6-(5-phosphoribos | 0 | Not detected | -0.246 | -0.534 | 0.041 | 0.093 |
| ENZRXN.26 | 1,2-Î±-L-arabinofuranosida | 0 | Not detected | -0.060 | -0.230 | 0.109 | 0.486 |
| ENZRXN.26 | 2-hydroxyethylphosphonat | 599 | Not detected | -0.418 | -0.780 | -0.055 | 0.024 |

| ENZRXN.26 | acetate kinase | 0 | Not detected | -0.122 | -0.315 | 0.071 | 0.216 |
| --- | --- | --- | --- | --- | --- | --- | --- |
| ENZRXN.26 | thiol-disulfide exchange | 0 | Not detected | 0.206 | -0.051 | 0.463 | 0.116 |
| ENZRXN.26 | phosphofructokinase | 624 | Not detected | -0.225 | -0.438 | -0.012 | 0.038 |
| ENZRXN.28 | fructose-bisphosphate aldol | 0 | Not detected | -0.173 | -0.342 | -0.004 | 0.045 |
| ENZRXN.29 | phospho-Î²-galactosidase | 645 | Not detected | -0.280 | -0.616 | 0.056 | 0.103 |
| ENZRXN.30 | 4-hydroxyphenylacetate 3- | 14 | Not detected | 0.163 | -0.055 | 0.381 | 0.144 |
| ENZRXN.33 | aspartokinase | 10 | Not detected | 0.194 | -0.138 | 0.525 | 0.253 |
| ENZRXN.44 | GDP-4-keto-6-deoxymann | 0 | Not detected | -0.234 | -0.572 | 0.104 | 0.175 |
| ENZRXN.58 | isoamylase | 0 | Not detected | 0.119 | -0.230 | 0.468 | 0.503 |
| ENZRXN.60 | trehalose-6-phosphate hydr | 0 | Not detected | -0.065 | -0.270 | 0.141 | 0.538 |
| ENZRXN.61 | 16S rRNA m2G1207 meth | 0 | Not detected | 0.108 | -0.102 | 0.319 | 0.314 |
| ENZRXN.62 | Î±-phosphoglucomutase | 0 | Not detected | -0.118 | -0.407 | 0.170 | 0.421 |
| ENZRXN.63 | UDP-glucose-4-epimerase | 0 | Not detected | -0.262 | -0.490 | -0.035 | 0.024 |
| ENZRXN.67 | N-succinyldiaminopimelate | 11 | Not detected | 0.275 | -0.037 | 0.588 | 0.084 |
| ENZRXN.68 | aspartate kinase | 0 | Not detected | -0.207 | -0.499 | 0.086 | 0.166 |
| ENZRXN.71 | 6-phosphogluconate dehyd | 0 | Not detected | -0.141 | -0.385 | 0.103 | 0.257 |
| ENZRXN.78 | phosphoribosylamidoimida | 0 | Not detected | -0.357 | -0.690 | -0.024 | 0.036 |
| ENZRXN.80 | nitric oxide dioxygenase | 0 | Not detected | 0.054 | -0.156 | 0.264 | 0.612 |
| ENZRXN.89 | UDP-N-acetylglucosamine | 766 | 0.6 | -0.234 | -0.433 | -0.035 | 0.022 |
| ENZRXN.90 | UTP-glucose-1-phosphate | 0 | Not detected | -0.118 | -0.336 | 0.099 | 0.287 |
| ENZRXN.93 | NAD-dependent glyceralde | 0 | Not detected | -0.273 | -0.587 | 0.041 | 0.089 |
| ENZRXN.95 | O-acetylhomoserine sulfhy | 0 | Not detected | -0.092 | -0.249 | 0.065 | 0.253 |
| ENZRXN0.1 | lipoyl-adenylate--protein li | 0 | Not detected | 0.030 | -0.162 | 0.222 | 0.756 |
| ENZRXN0.1 | 23S rRNA m5U1939 meth | 0 | Not detected | 0.163 | -0.055 | 0.382 | 0.143 |
| ENZRXN0.1 | GTPase | 255 | Not detected | 0.345 | 0.107 | 0.582 | 0.005 |
| ENZRXN0.1 | lipoate acetyltransferase | 0 | Not detected | -0.064 | -0.340 | 0.212 | 0.648 |
| ENZRXN0.1 | lipoate-apo-GcvH protein l | 0 | Not detected | 0.030 | -0.162 | 0.222 | 0.756 |
| ENZRXN0.1 | protein-(glutamine-N5) me | 0 | Not detected | 0.162 | -0.031 | 0.356 | 0.100 |
| ENZRXN0.1 | protein-glutamine N-methy | 0 | Not detected | 0.132 | -0.073 | 0.337 | 0.206 |
| ENZRXN0.1 | propanoate:CoA ligase (AM | 0 | Not detected | 0.049 | -0.232 | 0.330 | 0.731 |
| ENZRXN0.1 | tRNA-dihydrouridine synth | 0 | Not detected | 0.305 | 0.009 | 0.601 | 0.044 |
| ENZRXN0.1 | tRNA-dihydrouridine synth | 0 | Not detected | 0.161 | -0.061 | 0.382 | 0.155 |
| ENZRXN0.1 | tRNA-dihydrouridine16 sy | 0 | Not detected | 0.267 | 0.055 | 0.479 | 0.014 |
| ENZRXN0.1 | tRNA(m5U54)methyltransf | 0 | Not detected | 0.039 | -0.190 | 0.269 | 0.737 |
| ENZRXN0.1 | deoxyribodipyrimidine pho | 0 | Not detected | 0.072 | -0.152 | 0.297 | 0.527 |
| ENZRXN0.1 | O-6-methylguanine-DNA m | 0 | Not detected | 0.082 | -0.125 | 0.289 | 0.437 |
| ENZRXN0.1 | CCA tRNA nucleotidyltran | 0 | Not detected | 0.319 | 0.082 | 0.555 | 0.008 |
| ENZRXN0.1 | DNA adenine methyltransf | 0 | Not detected | 0.243 | 0.021 | 0.465 | 0.032 |
| ENZRXN0.1 | tRNA dimethylallyltransfer | 0 | Not detected | -0.032 | -0.300 | 0.236 | 0.816 |
| ENZRXN0.1 | tRNA preQ134 S-adenosyl | 0 | Not detected | -0.141 | -0.319 | 0.038 | 0.122 |
| ENZRXN0.1 | ribose 1,5-bisphosphate ph | 0 | Not detected | 0.157 | -0.023 | 0.337 | 0.088 |
| ENZRXN0.1 | TDP-N-acetylfucosamine:li | 0 | Not detected | 0.163 | -0.066 | 0.393 | 0.164 |
| ENZRXN0.1 | ferroxidase | 0 | Not detected | 0.088 | -0.146 | 0.322 | 0.463 |
| ENZRXN0.1 | inosine:H+ antiporter | 0 | Not detected | 0.228 | 0.008 | 0.449 | 0.043 |

| ENZRXN0.2 | carboxylesterase | 0 | Not detected | 0.081 | -0.117 | 0.279 | 0.423 |
| --- | --- | --- | --- | --- | --- | --- | --- |
| ENZRXN0.2 | hydroxylamine reductase | 0 | Not detected | 0.111 | -0.126 | 0.348 | 0.358 |
| ENZRXN0.2 | diadenosine tetraphosphata | 0 | Not detected | -0.043 | -0.252 | 0.166 | 0.688 |
| ENZRXN0.2 | DNA-cytosine methyltransf | 0 | Not detected | 0.123 | -0.091 | 0.337 | 0.261 |
| ENZRXN0.2 | phosphatidylglycerol&mda | 0 | Not detected | 0.202 | -0.068 | 0.473 | 0.142 |
| ENZRXN0.2 | enterochelin esterase | 0 | Not detected | 0.129 | -0.075 | 0.332 | 0.216 |
| ENZRXN0.2 | limit dextrin Î±-1,6-glucoh | 0 | Not detected | 0.240 | -0.022 | 0.503 | 0.073 |
| ENZRXN0.2 | long-chain fatty acid outer | 0 | Not detected | 0.110 | -0.102 | 0.321 | 0.310 |
| ENZRXN0.2 | ferric dihyroxybenzoylserin | 0 | Not detected | 0.009 | -0.219 | 0.236 | 0.941 |
| ENZRXN0.2 | UDP-4-amino-4-deoxy-L-a | 0 | Not detected | 0.124 | -0.080 | 0.329 | 0.234 |
| ENZRXN0.2 | guanosine:H+ antiporter | 0 | Not detected | 0.228 | 0.008 | 0.449 | 0.043 |
| ENZRXN0.2 | Î²-N-acetylglucosaminidase | 0 | Not detected | 0.185 | -0.038 | 0.408 | 0.104 |
| ENZRXN0.2 | guanine deaminase | 0 | Not detected | 0.061 | -0.176 | 0.298 | 0.614 |
| ENZRXN0.2 | crotonyl-CoA hydratase | 0 | Not detected | 0.193 | -0.035 | 0.421 | 0.097 |
| ENZRXN0.2 | L-ribulose-5-phosphate 4-e | 0 | Not detected | 0.123 | -0.074 | 0.321 | 0.222 |
| ENZRXN0.2 | dihydropteridine reductase | 0 | Not detected | 0.054 | -0.156 | 0.264 | 0.612 |
| ENZRXN0.2 | 23S rRNA pseudouridine7 | 0 | Not detected | 0.080 | -0.126 | 0.286 | 0.446 |
| ENZRXN0.2 | 23S rRNA pseudouridine2 | 0 | Not detected | -0.054 | -0.256 | 0.147 | 0.597 |
| ENZRXN0.2 | 23S rRNA pseudouridine2 | 0 | Not detected | 0.114 | -0.141 | 0.370 | 0.380 |
| ENZRXN0.2 | 23S rRNA pseudouridine2 | 0 | Not detected | -0.035 | -0.221 | 0.152 | 0.716 |
| ENZRXN0.2 | 23S rRNA pseudouridine9 | 0 | Not detected | -0.005 | -0.248 | 0.237 | 0.965 |
| ENZRXN0.2 | inositol-phosphate phospha | 0 | Not detected | -0.057 | -0.292 | 0.178 | 0.634 |
| ENZRXN0.2 | Î²-keto ester reductase | 0 | Not detected | 0.077 | -0.122 | 0.276 | 0.449 |
| ENZRXN0.2 | propionyl-CoA:succinate C | 0 | Not detected | -0.121 | -0.347 | 0.104 | 0.291 |
| ENZRXN0.2 | tRNAIle-lysidine synthetas | 0 | Not detected | 0.106 | -0.121 | 0.333 | 0.361 |
| ENZRXN0.2 | 23S rRNA pseudouridine1 | 0 | Not detected | 0.002 | -0.239 | 0.243 | 0.987 |
| ENZRXN0.2 | 16S rRNA pseudouridine5 | 0 | Not detected | 0.165 | -0.027 | 0.357 | 0.092 |
| ENZRXN0.2 | lipid IVA 4-amino-4-deoxy | 0 | Not detected | -0.005 | -0.228 | 0.218 | 0.962 |
| ENZRXN0.2 | 3-hydroxyacyl-CoA dehydr | 0 | Not detected | 0.070 | -0.182 | 0.322 | 0.587 |
| ENZRXN0.2 | dodecenoyl-CoA D-isomer | 0 | Not detected | 0.070 | -0.182 | 0.322 | 0.587 |
| ENZRXN0.2 | 3-hydroxybutyryl-CoA epi | 0 | Not detected | 0.070 | -0.182 | 0.322 | 0.587 |
| ENZRXN0.2 | acetyl-CoA C-acyltransfera | 0 | Not detected | 0.094 | -0.120 | 0.309 | 0.388 |
| ENZRXN0.2 | short chain acyl-CoA synth | 0 | Not detected | 0.033 | -0.242 | 0.309 | 0.813 |
| ENZRXN0.2 | enoyl-CoA hydratase | 0 | Not detected | 0.070 | -0.182 | 0.322 | 0.587 |
| ENZRXN0.2 | tyrosine kinase | 0 | Not detected | 0.005 | -0.234 | 0.244 | 0.967 |
| ENZRXN0.2 | tRNA-specific 2-thiouridyl | 0 | Not detected | -0.133 | -0.381 | 0.116 | 0.295 |
| ENZRXN0.2 | NADH oxidoreductase | 0 | Not detected | 0.121 | -0.070 | 0.312 | 0.214 |
| ENZRXN0.2 | 3-hydroxyadipyl-CoA dehy | 0 | Not detected | 0.132 | -0.070 | 0.333 | 0.201 |
| ENZRXN0.2 | D-allulose-6-phosphate 3-e | 0 | Not detected | -0.266 | -0.595 | 0.064 | 0.115 |
| ENZRXN0.2 | NADH cupric reductase | 0 | Not detected | 0.067 | -0.157 | 0.291 | 0.559 |
| ENZRXN0.2 | DNA ligase | 0 | Not detected | 0.080 | -0.167 | 0.327 | 0.527 |
| ENZRXN0.2 | DNA ligase | 0 | Not detected | 0.065 | -0.261 | 0.391 | 0.696 |
| ENZRXN0.2 | peptidyl-prolyl cis-trans iso | 0 | Not detected | 0.122 | -0.167 | 0.410 | 0.410 |
| ENZRXN0.2 | peptidyl-prolyl cis-trans iso | 0 | Not detected | 0.110 | -0.177 | 0.398 | 0.452 |

| ENZRXN0.2 | UDP-2,3-diacylglucosamin | 40 | Not detected | -0.087 | -0.280 | 0.107 | 0.380 |
| --- | --- | --- | --- | --- | --- | --- | --- |
| ENZRXN0.2 | pyruvate-flavodoxin oxidor | 0 | Not detected | -0.280 | -0.663 | 0.102 | 0.152 |
| ENZRXN0.2 | malate:quinone oxidoreduc | 0 | Not detected | 0.259 | 0.027 | 0.491 | 0.029 |
| ENZRXN0.2 | fructose-6-phosphate aldol | 0 | Not detected | 0.157 | -0.025 | 0.339 | 0.091 |
| ENZRXN0.2 | tRNA m7G46 methyltransf | 0 | Not detected | 0.142 | -0.128 | 0.411 | 0.303 |
| ENZRXN0.2 | 10-formyltetrahydrofolate: | 0 | Not detected | -0.090 | -0.381 | 0.201 | 0.545 |
| ENZRXN0.3 | RNase R | 0 | Not detected | 0.088 | -0.212 | 0.387 | 0.566 |
| ENZRXN0.3 | fructokinase | 0 | Not detected | 0.123 | -0.076 | 0.322 | 0.226 |
| ENZRXN0.3 | tRNA 2-selenouridine synt | 0 | Not detected | 0.057 | -0.151 | 0.265 | 0.593 |
| ENZRXN0.3 | endo-1,4-D-glucanase | 0 | Not detected | -0.011 | -0.221 | 0.200 | 0.921 |
| ENZRXN0.3 | UDP-N-acetylmuramate:L- | 0 | Not detected | -0.102 | -0.413 | 0.208 | 0.518 |
| ENZRXN0.3 | 23S rRNA m5U747 methyl | 0 | Not detected | 0.220 | -0.009 | 0.449 | 0.060 |
| ENZRXN0.3 | indoleglycerol phosphate al | 0 | Not detected | 0.147 | -0.051 | 0.345 | 0.146 |
| ENZRXN0.3 | L-threonine:H+ antiporter | 0 | Not detected | 0.079 | -0.174 | 0.333 | 0.540 |
| ENZRXN0.3 | L-homoserine:H+ antiporte | 0 | Not detected | 0.079 | -0.174 | 0.333 | 0.540 |
| ENZRXN0.3 | Fe2+:H+ symporter | 0 | Not detected | 0.157 | -0.049 | 0.363 | 0.135 |
| ENZRXN0.3 | N-acetylmuramyl-L-alanine | 0 | Not detected | 0.204 | -0.021 | 0.429 | 0.076 |
| ENZRXN0.3 | tRNA pseudouridine13 syn | 0 | Not detected | 0.138 | -0.066 | 0.343 | 0.186 |
| ENZRXN0.3 | tRNA pseudouridine55 syn | 0 | Not detected | -0.098 | -0.316 | 0.121 | 0.382 |
| ENZRXN0.3 | tRNA pseudouridine65 syn | 4 | Not detected | -0.057 | -0.245 | 0.132 | 0.555 |
| ENZRXN0.3 | methionine aminopeptidase | 0 | Not detected | -0.073 | -0.387 | 0.242 | 0.651 |
| ENZRXN0.3 | peptide deformylase | 0 | Not detected | 0.108 | -0.126 | 0.342 | 0.366 |
| ENZRXN0.3 | DNA polymerase | 0 | Not detected | 0.137 | -0.080 | 0.355 | 0.216 |
| ENZRXN0.3 | 16S rRNA m62A1518,m62 | 0 | Not detected | -0.024 | -0.268 | 0.219 | 0.846 |
| ENZRXN0.3 | undecaprenyl pyrophospha | 0 | Not detected | -0.054 | -0.291 | 0.182 | 0.653 |
| ENZRXN0.3 | 16S rRNA m5C967 methyl | 0 | Not detected | 0.266 | 0.015 | 0.517 | 0.038 |
| ENZRXN0.3 | leader peptidase | 0 | Not detected | 0.007 | -0.190 | 0.204 | 0.944 |
| ENZRXN0.3 | topoisomerase | 0 | Not detected | 0.018 | -0.286 | 0.321 | 0.909 |
| ENZRXN0.4 | topoisomerase | 0 | Not detected | 0.114 | -0.142 | 0.369 | 0.385 |
| ENZRXN0.4 | methylene-tetrahydrofolylp | 0 | Not detected | 0.191 | -0.033 | 0.415 | 0.095 |
| ENZRXN0.4 | 2,3-dihydroxybenzoate:oxy | 0 | Not detected | 0.088 | -0.146 | 0.322 | 0.463 |
| ENZRXN0.4 | cuprous oxidase | 0 | Not detected | 0.088 | -0.146 | 0.322 | 0.463 |
| ENZRXN0.4 | uridine nucleosidase | 0 | Not detected | 0.146 | -0.058 | 0.351 | 0.161 |
| ENZRXN0.4 | oligopeptidase | 0 | Not detected | 0.095 | -0.130 | 0.321 | 0.409 |
| ENZRXN0.4 | 23S rRNA 2'-O-ribose U25 | 0 | Not detected | 0.029 | -0.193 | 0.252 | 0.796 |
| ENZRXN0.4 | oligopeptidase | 0 | Not detected | 0.007 | -0.298 | 0.311 | 0.965 |
| ENZRXN0.4 | tail-specific protease | 131 | Not detected | 0.389 | 0.129 | 0.649 | 0.003 |
| ENZRXN0.4 | protease | 0 | Not detected | 0.033 | -0.246 | 0.313 | 0.815 |
| ENZRXN0.4 | alanyl aminopeptidase | 0 | Not detected | 0.239 | -0.044 | 0.522 | 0.098 |
| ENZRXN0.4 | alkaline phosphatase amino | 0 | Not detected | -0.044 | -0.248 | 0.159 | 0.671 |
| ENZRXN0.4 | peptidyl-dipeptidase | 0 | Not detected | 0.109 | -0.123 | 0.341 | 0.356 |
| ENZRXN0.4 | formate dehydrogenase | 0 | Not detected | -0.018 | -0.319 | 0.282 | 0.905 |
| ENZRXN0.4 | meso-diaminopimelic acid- | 0 | Not detected | 0.214 | -0.017 | 0.446 | 0.070 |
| ENZRXN0.4 | exoribonuclease II | 0 | Not detected | 0.149 | -0.113 | 0.412 | 0.265 |

| ENZRXN0.5 | methionine-S-sulfoxide red | 0 | Not detected | 0.096 | -0.169 | 0.361 | 0.477 |
| --- | --- | --- | --- | --- | --- | --- | --- |
| ENZRXN0.5 | 5'-nucleotidase | 70 | Not detected | 0.260 | 0.048 | 0.473 | 0.017 |
| ENZRXN0.5 | 3'-nucleotidase | 70 | Not detected | 0.260 | 0.048 | 0.473 | 0.017 |
| ENZRXN0.5 | pyrimidine 5'-nucleotidase | 0 | Not detected | 0.172 | -0.013 | 0.357 | 0.068 |
| ENZRXN0.5 | &gamma;-glutamylputresci | 0 | Not detected | 0.039 | -0.170 | 0.248 | 0.715 |
| ENZRXN0.5 | &gamma;-glutamyl-&gam | 0 | Not detected | 0.091 | -0.122 | 0.305 | 0.401 |
| ENZRXN0.5 | octanoul-ACP synthetase | 0 | Not detected | 0.099 | -0.157 | 0.355 | 0.450 |
| ENZRXN0.5 | 4-aminobutyrate aminotran | 17 | Not detected | 0.240 | 0.027 | 0.452 | 0.027 |
| ENZRXN0.5 | endochitinase | 0 | Not detected | 0.001 | -0.260 | 0.262 | 0.994 |
| ENZRXN0.5 | serine/threonine:Na+ symp | 0 | Not detected | -0.150 | -0.291 | -0.009 | 0.037 |
| ENZRXN0.5 | ribonuclease | 676 | 0.6 | -0.334 | -0.548 | -0.121 | 0.002 |
| ENZRXN0.5 | RNase | 0 | Not detected | -0.114 | -0.454 | 0.226 | 0.513 |
| ENZRXN0.5 | methylglyoxal reductase | 0 | Not detected | -0.087 | -0.237 | 0.063 | 0.257 |
| ENZRXN0.5 | methylglyoxal reductase | 0 | Not detected | 0.077 | -0.122 | 0.276 | 0.449 |
| ENZRXN0.5 | methylglyoxal reductase | 0 | Not detected | -0.205 | -0.524 | 0.115 | 0.210 |
| ENZRXN0.5 | Kdo2-lipid VIA phosphoet | 0 | Not detected | 0.112 | -0.137 | 0.362 | 0.378 |
| ENZRXN0.5 | anhydro-N-acetylmuramic | 0 | Not detected | 0.161 | -0.053 | 0.375 | 0.140 |
| ENZRXN0.5 | RNase | 0 | Not detected | -0.245 | -0.533 | 0.044 | 0.096 |
| ENZRXN0.6 | DNA polymerase | 0 | Not detected | 0.371 | 0.063 | 0.679 | 0.019 |
| ENZRXN0.6 | 3' to 5' proofreading exonu | 0 | Not detected | 0.154 | -0.047 | 0.355 | 0.134 |
| ENZRXN0.6 | DNA primase | 381 | Not detected | 0.344 | 0.073 | 0.615 | 0.013 |
| ENZRXN0.6 | DNA polymerase | 0 | Not detected | 0.189 | -0.069 | 0.448 | 0.152 |
| ENZRXN0.6 | DNA polymerase | 0 | Not detected | 0.189 | -0.069 | 0.448 | 0.152 |
| ENZRXN0.6 | peptidyl-prolyl cis-trans iso | 0 | Not detected | 0.001 | -0.219 | 0.222 | 0.990 |
| ENZRXN0.6 | cAMP phosphodiesterase | 0 | Not detected | 0.124 | -0.086 | 0.333 | 0.247 |
| ENZRXN0.6 | DNA polymerase | 0 | Not detected | 0.239 | -0.064 | 0.543 | 0.123 |
| ENZRXN0.6 | 3' to 5' proofreading exonu | 0 | Not detected | 0.239 | -0.064 | 0.543 | 0.123 |
| ENZRXN0.6 | 5' to 3' exonuclease | 0 | Not detected | 0.239 | -0.064 | 0.543 | 0.123 |
| ENZRXN0.6 | ADP-heptose:Kdo2-lipid I | 385 | Not detected | 0.332 | 0.111 | 0.553 | 0.003 |
| ENZRXN0.6 | ADP-heptose&mdash;LPS | 17 | Not detected | 0.320 | 0.090 | 0.550 | 0.007 |
| ENZRXN0.6 | isopentenyl-adenosine tRN | 0 | Not detected | 0.238 | -0.073 | 0.550 | 0.134 |
| ENZRXN0.6 | xanthine:H+ symporter Xa | 0 | Not detected | -0.078 | -0.256 | 0.100 | 0.392 |
| ENZRXN0.6 | 1-deoxy-D-xylulose 5-phos | 0 | Not detected | -0.050 | -0.208 | 0.107 | 0.531 |
| ENZRXN0.6 | octanoate-non-lipoylated a | 0 | Not detected | 0.030 | -0.162 | 0.222 | 0.756 |
| ENZRXN0.6 | 16S rRNA m5C1407 meth | 0 | Not detected | 0.071 | -0.142 | 0.284 | 0.515 |
| ENZRXN0.6 | Î²-D-glucoside glucohydrol | 0 | Not detected | 0.121 | -0.129 | 0.371 | 0.343 |
| ENZRXN0.6 | ADP-heptose:Kdo2-lipid A | 385 | Not detected | 0.332 | 0.111 | 0.553 | 0.003 |
| ENZRXN0.6 | transport of D-serine | 0 | Not detected | 0.186 | -0.086 | 0.458 | 0.181 |
| ENZRXN0.6 | methylglyoxal reductase | 0 | Not detected | -0.255 | -0.593 | 0.083 | 0.140 |
| ENZRXN0.6 | 5-methylaminomethyl-2-thi | 0 | Not detected | 0.113 | -0.124 | 0.350 | 0.351 |
| ENZRXN0.6 | 2-isopropylmalate synthase | 0 | Not detected | -0.017 | -0.214 | 0.181 | 0.869 |
| ENZRXN0.6 | 6-phosphogluconate phosp | 0 | Not detected | -0.058 | -0.251 | 0.135 | 0.554 |
| ENZRXN0.6 | sugar phosphatase | 0 | Not detected | -0.081 | -0.297 | 0.134 | 0.460 |
| ENZRXN0.6 | pyridoxal phosphatase | 0 | Not detected | 0.109 | -0.092 | 0.311 | 0.288 |

| ENZRXN0.6 | phosphosugar phosphatase | 0 | Not detected | 0.109 | -0.092 | 0.311 | 0.288 |
| --- | --- | --- | --- | --- | --- | --- | --- |
| ENZRXN0.6 | 23S rRNA m2G1835 meth | 0 | Not detected | 0.104 | -0.113 | 0.322 | 0.346 |
| ENZRXN0.6 | 23S rRNA m2G2445 meth | 0 | Not detected | 0.290 | 0.019 | 0.561 | 0.036 |
| ENZRXN0.6 | 3 methyl-adenine DNA gly | 0 | Not detected | -0.183 | -0.431 | 0.066 | 0.150 |
| ENZRXN0.6 | peptidyl-prolyl cis-trans iso | 0 | Not detected | 0.245 | -0.005 | 0.495 | 0.055 |
| ENZRXN0.6 | lysine 2,3-aminomutase | 0 | Not detected | 0.250 | 0.018 | 0.481 | 0.035 |
| ENZRXN0.6 | alanyl aminopeptidase | 0 | Not detected | 0.239 | -0.044 | 0.522 | 0.098 |
| ENZRXN0.6 | D-malate dehydrogenase (d | 0 | Not detected | -0.049 | -0.329 | 0.231 | 0.731 |
| ENZRXN0.6 | poly(A) polymerase | 0 | Not detected | 0.049 | -0.184 | 0.282 | 0.681 |
| ENZRXN0.6 | 2-oxobutanoate aminotrans | 0 | Not detected | 0.111 | -0.109 | 0.332 | 0.323 |
| ENZRXN0.6 | exodeoxyribonuclease I | 0 | Not detected | 0.228 | -0.014 | 0.469 | 0.065 |
| ENZRXN0.6 | transport of Î²-alanine | 0 | Not detected | 0.186 | -0.086 | 0.458 | 0.181 |
| ENZRXN0.6 | transport of L-alanine | 0 | Not detected | 0.186 | -0.086 | 0.458 | 0.181 |
| ENZRXN0.6 | transport of D-cycloserine | 0 | Not detected | 0.186 | -0.086 | 0.458 | 0.181 |
| ENZRXN0.6 | 3' &rarr; 5' exonuclease | 0 | Not detected | 0.072 | -0.115 | 0.260 | 0.450 |
| ENZRXN0.6 | Zn2+-exporting ATPase | 0 | Not detected | 0.208 | -0.065 | 0.480 | 0.136 |
| ENZRXN0.6 | S-ribosylhomocysteine lyas | 0 | Not detected | 0.056 | -0.160 | 0.271 | 0.613 |
| ENZRXN0.6 | 16S rRNA m2G966 methyl | 0 | Not detected | 0.185 | -0.009 | 0.379 | 0.062 |
| ENZRXN0.6 | D-gluconate transporter | 0 | Not detected | -0.017 | -0.241 | 0.207 | 0.880 |
| ENZRXN0.6 | high-affinity gluconate tran | 0 | Not detected | -0.112 | -0.399 | 0.174 | 0.443 |
| ENZRXN0.6 | 2-O-(6-phospho-Î±-manno | 0 | Not detected | 0.126 | -0.123 | 0.374 | 0.322 |
| ENZRXN0.6 | putrescine:H+ symporter | 0 | Not detected | -0.297 | -0.614 | 0.019 | 0.066 |
| ENZRXN0.6 | putrescine:L-ornithine anti | 0 | Not detected | -0.297 | -0.614 | 0.019 | 0.066 |
| ENZRXN0.6 | cadaverine:H+ symporter | 0 | Not detected | -0.123 | -0.413 | 0.167 | 0.406 |
| ENZRXN0.6 | serine/threonine protein kin | 0 | Not detected | 0.133 | -0.070 | 0.335 | 0.199 |
| ENZRXN0.6 | tRNA pseudouridine32 syn | 0 | Not detected | 0.080 | -0.126 | 0.286 | 0.446 |
| ENZRXN0.6 | transport of GlcNAc-1,6-an | 0 | Not detected | 0.238 | 0.022 | 0.454 | 0.031 |
| ENZRXN0.6 | 1,6-anhydro-N-acetylmura | 0 | Not detected | -0.019 | -0.225 | 0.187 | 0.854 |
| ENZRXN0.6 | glcNAc-1,6-anhMurNAcam | 0 | Not detected | 0.185 | -0.038 | 0.408 | 0.104 |
| ENZRXN0.6 | galactonate:H+ symporter | 0 | Not detected | -0.023 | -0.251 | 0.206 | 0.846 |
| ENZRXN0.6 | L-galactonate oxidoreducta | 0 | Not detected | 0.088 | -0.130 | 0.305 | 0.430 |
| ENZRXN0.6 | transport of L-aspartate | 0 | Not detected | 0.046 | -0.262 | 0.353 | 0.772 |
| ENZRXN0.6 | succinate:fumarate antiport | 0 | Not detected | 0.258 | 0.017 | 0.498 | 0.036 |
| ENZRXN0.6 | 16S rRNA m7G527 methyl | 0 | Not detected | 0.077 | -0.133 | 0.288 | 0.471 |
| ENZRXN0.6 | transport of L-lyxose | 16 | Not detected | 0.253 | 0.048 | 0.458 | 0.016 |
| ENZRXN0.6 | L-idarate epimerase | 0 | Not detected | -0.023 | -0.305 | 0.260 | 0.876 |
| ENZRXN0.6 | nitrate:nitrite antiporter | 0 | Not detected | -0.009 | -0.208 | 0.190 | 0.930 |
| ENZRXN0.6 | nitrate:nitrite antiporter | 0 | Not detected | 0.063 | -0.144 | 0.270 | 0.553 |
| ENZRXN0.6 | biotin transporter | 0 | Not detected | -0.011 | -0.215 | 0.194 | 0.919 |
| ENZRXN0.6 | arbutin 6-phosphate glucoh | 0 | Not detected | -0.180 | -0.470 | 0.110 | 0.225 |
| ENZRXN0.6 | arbutin 6-phosphate glucoh | 0 | Not detected | -0.092 | -0.234 | 0.049 | 0.201 |
| ENZRXN0.6 | arbutin-6-phosphate glucoh | 0 | Not detected | -0.186 | -0.374 | 0.001 | 0.051 |
| ENZRXN0.6 | salicin 6-phosphate glucoh | 0 | Not detected | -0.180 | -0.470 | 0.110 | 0.225 |
| ENZRXN0.6 | transport of N-acetyl-Î²-D-g | 0 | Not detected | 0.238 | 0.022 | 0.454 | 0.031 |

| ENZRXN0.6 | 23S rRNA m2A2503 meth | 0 | Not detected | -0.052 | -0.357 | 0.254 | 0.740 |
| --- | --- | --- | --- | --- | --- | --- | --- |
| ENZRXN0.6 | protein kinase | 0 | Not detected | 0.247 | 0.007 | 0.486 | 0.044 |
| ENZRXN0.6 | L-2-hydroxyglutarate dehy | 0 | Not detected | -0.025 | -0.241 | 0.190 | 0.818 |
| ENZRXN0.6 | undecaprenyl-pyrophospha | 0 | Not detected | 0.031 | -0.235 | 0.296 | 0.821 |
| ENZRXN0.6 | NAD(P)H:quinone oxidore | 0 | Not detected | 0.104 | -0.115 | 0.322 | 0.353 |
| ENZRXN0.6 | anthranilate phosphoribosy | 0 | Not detected | 0.078 | -0.154 | 0.310 | 0.511 |
| ENZRXN0.6 | nigerose hydrolase | 0 | Not detected | 0.135 | -0.118 | 0.387 | 0.296 |
| ENZRXN0.6 | peptidoglycan glycosyltran | 259 | Not detected | 0.296 | 0.046 | 0.546 | 0.020 |
| ENZRXN0.6 | peptidoglycan glycosyltran | 0 | Not detected | 0.043 | -0.175 | 0.262 | 0.697 |
| ENZRXN0.6 | murein transpeptidase | 0 | Not detected | 0.160 | -0.118 | 0.438 | 0.261 |
| ENZRXN0.6 | glycerol-2-phosphatase | 0 | Not detected | -0.057 | -0.292 | 0.178 | 0.634 |
| ENZRXN0.6 | L-glyceraldehyde 3-phosph | 0 | Not detected | -0.087 | -0.237 | 0.063 | 0.257 |
| ENZRXN0.6 | poly-Î²-1,6-N-acetyl-D-glu | 0 | Not detected | 0.117 | -0.120 | 0.353 | 0.334 |
| ENZRXN0.6 | tRNAMet cytidine acetyltra | 0 | Not detected | 0.216 | -0.027 | 0.459 | 0.082 |
| ENZRXN0.6 | ribosomal protein L11 met | 0 | Not detected | 0.031 | -0.212 | 0.273 | 0.803 |
| ENZRXN0.6 | glutamyl-Q tRNA(Asp) syn | 0 | Not detected | 0.141 | -0.059 | 0.341 | 0.167 |
| ENZRXN0.6 | ThiS adenylyltransferase | 0 | Not detected | 0.014 | -0.182 | 0.210 | 0.889 |
| ENZRXN0.6 | adenylate:persulfide transfe | 0 | Not detected | 0.014 | -0.182 | 0.210 | 0.889 |
| ENZRXN0.6 | 3-hydroxypropionaldehyde | 0 | Not detected | 0.091 | -0.122 | 0.305 | 0.401 |
| ENZRXN0.6 | lipid III flippase | 0 | Not detected | 0.267 | 0.037 | 0.497 | 0.023 |
| ENZRXN0.6 | glucose dehydrogenase | 0 | Not detected | 0.166 | -0.101 | 0.433 | 0.223 |
| ENZRXN0.6 | autoinducer-2 kinase | 83 | Not detected | 0.232 | -0.005 | 0.468 | 0.056 |
| ENZRXN0.6 | lipid II flippase | 0 | Not detected | 0.136 | -0.082 | 0.354 | 0.222 |
| ENZRXN0.6 | GTPase | 0 | Not detected | 0.002 | -0.249 | 0.252 | 0.990 |
| ENZRXN0.6 | transport of a dipeptide | 0 | Not detected | 0.102 | -0.135 | 0.340 | 0.399 |
| ENZRXN0.6 | acyl-[acp]:1-acyl-sn-glycer | 0 | Not detected | 0.043 | -0.160 | 0.247 | 0.676 |
| ENZRXN0.6 | CTP:phosphatidate cytidylt | 15 | Not detected | 0.177 | -0.009 | 0.362 | 0.063 |
| ENZRXN0.6 | acyl-acp:sn-glycerol-3-pho | 0 | Not detected | 0.208 | -0.083 | 0.499 | 0.161 |
| ENZRXN0.6 | 23S rRNA 2'-O-ribose C24 | 0 | Not detected | 0.031 | -0.192 | 0.254 | 0.785 |
| ENZRXN0.7 | fructoselysine transporter | 0 | Not detected | -0.365 | -0.669 | -0.061 | 0.019 |
| ENZRXN0.7 | psicoselysine transporter | 0 | Not detected | -0.365 | -0.669 | -0.061 | 0.019 |
| ENZRXN0.7 | L-ribulose-5-phosphate 4-e | 0 | Not detected | -0.042 | -0.192 | 0.107 | 0.578 |
| ENZRXN0.7 | L-ribulose-5-phosphate 3-e | 0 | Not detected | 0.233 | -0.039 | 0.504 | 0.094 |
| ENZRXN0.7 | transport of a dipeptide | 0 | Not detected | 0.098 | -0.127 | 0.322 | 0.394 |
| ENZRXN0.7 | non-chaperonin molecular | 0 | Not detected | 0.193 | -0.111 | 0.497 | 0.214 |
| ENZRXN0.7 | protein methylthiotransfera | 0 | Not detected | 0.047 | -0.255 | 0.350 | 0.759 |
| ENZRXN0.7 | dipeptide transport | 0 | Not detected | 0.063 | -0.162 | 0.289 | 0.582 |
| ENZRXN0.7 | (S)-ureidoglycine aminohy | 0 | Not detected | -0.202 | -0.469 | 0.065 | 0.139 |
| ENZRXN0.7 | chitobiose transport | 0 | Not detected | 0.040 | -0.181 | 0.261 | 0.726 |
| ENZRXN0.7 | transport of adenine | 0 | Not detected | 0.178 | -0.057 | 0.413 | 0.138 |
| ENZRXN0.7 | guanosine 3'-diphosphate 5 | 0 | Not detected | 0.072 | -0.231 | 0.375 | 0.643 |
| ENZRXN0.7 | GTPase | 0 | Not detected | -0.001 | -0.322 | 0.320 | 0.995 |
| ENZRXN0.7 | GTPase | 0 | Not detected | 0.171 | -0.082 | 0.425 | 0.186 |
| ENZRXN0.7 | carbamoyl--[HypE] ligase | 0 | Not detected | 0.175 | -0.095 | 0.445 | 0.205 |

| ENZRXN0.7 | 2-iminoacetate synthase | 0 | Not detected | 0.027 | -0.198 | 0.253 | 0.812 |
| --- | --- | --- | --- | --- | --- | --- | --- |
| ENZRXN0.7 | transport of malate | 0 | Not detected | 0.046 | -0.262 | 0.353 | 0.772 |
| ENZRXN0.7 | methionine-oxo-acid transa | 0 | Not detected | 0.009 | -0.202 | 0.220 | 0.934 |
| ENZRXN0.7 | p-aminobenzoyl glutamate: | 0 | Not detected | 0.107 | -0.100 | 0.314 | 0.312 |
| ENZRXN0.7 | uracil oxygenase | 0 | Not detected | 0.029 | -0.168 | 0.227 | 0.771 |
| ENZRXN0.7 | RNA helicase | 0 | Not detected | 0.184 | -0.020 | 0.389 | 0.078 |
| ENZRXN0.7 | exoribonuclease II | 0 | Not detected | 0.149 | -0.113 | 0.412 | 0.265 |
| ENZRXN0.7 | peptidoglycan DD transpep | 637 | Not detected | 0.409 | 0.133 | 0.686 | 0.004 |
| ENZRXN0.7 | L-aspartate oxidase | 0 | Not detected | -0.214 | -0.558 | 0.131 | 0.224 |
| ENZRXN0.7 | exoribonuclease II | 0 | Not detected | 0.149 | -0.113 | 0.412 | 0.265 |
| ENZRXN0.7 | adenosine nucleosidase | 0 | Not detected | 0.146 | -0.058 | 0.351 | 0.161 |
| ENZRXN0.7 | xanthosine nucleosidase | 0 | Not detected | 0.146 | -0.058 | 0.351 | 0.161 |
| ENZRXN0.7 | inosine nucleosidase | 0 | Not detected | 0.146 | -0.058 | 0.351 | 0.161 |
| ENZRXN0.7 | cytidine nucleosidase | 0 | Not detected | 0.146 | -0.058 | 0.351 | 0.161 |
| ENZRXN0.7 | dihydroorotate dehydrogen | 0 | Not detected | -0.065 | -0.404 | 0.273 | 0.705 |
| ENZRXN0.7 | transport of 3-hydroxy-tran | 0 | Not detected | 0.159 | -0.055 | 0.373 | 0.146 |
| ENZRXN0.7 | epoxyqueuosine reductase | 0 | Not detected | 0.221 | 0.002 | 0.440 | 0.048 |
| ENZRXN0.7 | putrescine:H+ symporter Pl | 0 | Not detected | 0.059 | -0.159 | 0.278 | 0.596 |
| ENZRXN0.7 | acyl-[acp]:1-acyl-sn-glycer | 0 | Not detected | 0.043 | -0.160 | 0.247 | 0.676 |
| ENZRXN0.7 | putative inorganic phospha | 0 | Not detected | 0.238 | -0.006 | 0.483 | 0.056 |
| ENZRXN0.7 | S-methyl-L-methionine tra | 0 | Not detected | -0.064 | -0.386 | 0.258 | 0.698 |
| ENZRXN0.7 | NADHX epimerase | 0 | Not detected | 0.040 | -0.201 | 0.281 | 0.745 |
| ENZRXN0.7 | (S)-NADHX dehydratase | 0 | Not detected | 0.040 | -0.201 | 0.281 | 0.745 |
| ENZRXN0.7 | transport of inosine | 0 | Not detected | -0.048 | -0.310 | 0.214 | 0.720 |
| ENZRXN0.7 | transport of deoxyinosine | 0 | Not detected | -0.048 | -0.310 | 0.214 | 0.720 |
| ENZRXN0.7 | 16S rRNA m2G1516 meth | 0 | Not detected | 0.140 | -0.070 | 0.350 | 0.191 |
| ENZRXN0.7 | Î±-D-ribose-1-methylphosp | 0 | Not detected | 0.153 | -0.076 | 0.381 | 0.190 |
| ENZRXN0.7 | carbon-phosphorous lyase | 0 | Not detected | -0.131 | -0.388 | 0.126 | 0.318 |
| ENZRXN0.7 | peptidoglycan DD-carboxy | 0 | Not detected | 0.112 | -0.107 | 0.331 | 0.317 |
| ENZRXN0.7 | peptidoglycan DD-endopep | 0 | Not detected | 0.112 | -0.107 | 0.331 | 0.317 |
| ENZRXN0.7 | ferric enterobactin esterase | 0 | Not detected | 0.129 | -0.075 | 0.332 | 0.216 |
| ENZRXN0.7 | (S)-lactate transporter | 0 | Not detected | -0.164 | -0.461 | 0.132 | 0.277 |
| ENZRXN0.7 | 23S rRNA m7G2069 meth | 0 | Not detected | 0.290 | 0.019 | 0.561 | 0.036 |
| ENZRXN0.7 | enterobactin exporter EntS | 226 | Not detected | 0.284 | 0.065 | 0.504 | 0.011 |
| ENZRXN0.7 | D-tartrate:succinate antipor | 0 | Not detected | -0.169 | -0.426 | 0.089 | 0.199 |
| ENZRXN0.7 | GTPase | 0 | Not detected | 0.204 | -0.076 | 0.484 | 0.154 |
| ENZRXN0.7 | fructose-6-phosphate:phosp | 0 | Not detected | 0.140 | -0.100 | 0.381 | 0.254 |
| ENZRXN0.7 | mannose-6-phosphate:phos | 0 | Not detected | 0.140 | -0.100 | 0.381 | 0.254 |
| ENZRXN0.7 | propionate transporter | 0 | Not detected | 0.007 | -0.299 | 0.313 | 0.963 |
| ENZRXN0.7 | metalloprotease | 0 | Not detected | 0.000 | -0.211 | 0.210 | 0.999 |
| ENZRXN0.7 | (R)-lactate transporter | 0 | Not detected | 0.173 | -0.088 | 0.433 | 0.194 |
| ENZRXN0.7 | glycolate transporter | 0 | Not detected | 0.173 | -0.088 | 0.433 | 0.194 |
| ENZRXN0.7 | (R)-lactate transporter | 0 | Not detected | -0.164 | -0.461 | 0.132 | 0.277 |
| ENZRXN0.7 | transport of a C4-dicarboxy | 0 | Not detected | 0.046 | -0.262 | 0.353 | 0.772 |

| ENZRXN0.7 | methyl Î²-D-glucoside 6-ph | 0 | Not detected | -0.092 | -0.234 | 0.049 | 0.201 |
| --- | --- | --- | --- | --- | --- | --- | --- |
| ENZRXN0.7 | melibiose:H+ symporter | 0 | Not detected | 0.002 | -0.213 | 0.217 | 0.985 |
| ENZRXN0.7 | D-galactarate transporter | 0 | Not detected | 0.021 | -0.188 | 0.230 | 0.844 |
| ENZRXN0.7 | D-glucarate transporter | 0 | Not detected | 0.021 | -0.188 | 0.230 | 0.844 |
| ENZRXN0.7 | D-glycerate transporter | 0 | Not detected | 0.021 | -0.188 | 0.230 | 0.844 |
| ENZRXN0.7 | D-galactarate transporter | 0 | Not detected | 0.080 | -0.136 | 0.295 | 0.470 |
| ENZRXN0.7 | D-glucarate transporter | 0 | Not detected | 0.080 | -0.136 | 0.295 | 0.470 |
| ENZRXN0.7 | D-glycerate transporter | 0 | Not detected | 0.080 | -0.136 | 0.295 | 0.470 |
| ENZRXN0.7 | propionyl-CoA synthetase | 0 | Not detected | 0.014 | -0.223 | 0.251 | 0.907 |
| ENZRXN0.7 | 23S rRNA m6A2030 meth | 0 | Not detected | 0.153 | -0.061 | 0.367 | 0.162 |
| ENZRXN0.7 | tRNA m2A37 methyltransf | 0 | Not detected | -0.052 | -0.357 | 0.254 | 0.740 |
| ENZRXN0.7 | inorganic triphosphatase | 0 | Not detected | 0.031 | -0.190 | 0.252 | 0.783 |
| ENZRXN0.7 | L-glyceraldehyde-3-phosph | 0 | Not detected | 0.140 | -0.100 | 0.381 | 0.254 |
| ENZRXN0.7 | 6-phospho-Î²-D-glucosyl-(1 | 0 | Not detected | -0.186 | -0.374 | 0.001 | 0.051 |
| ENZRXN0.7 | aldehyde reductase | 0 | Not detected | 0.007 | -0.188 | 0.202 | 0.944 |
| ENZRXN0.7 | aldehyde reductase | 0 | Not detected | 0.094 | -0.119 | 0.306 | 0.387 |
| ENZRXN0.7 | 2,5-diketo-D-gluconate red | 0 | Not detected | 0.077 | -0.122 | 0.276 | 0.449 |
| ENZRXN0.7 | cysteinylglycine dipeptidas | 0 | Not detected | 0.239 | -0.044 | 0.522 | 0.098 |
| ENZRXN0.7 | UDP-N-acetylmuramate:L- | 0 | Not detected | -0.102 | -0.413 | 0.208 | 0.518 |
| ENZRXN0.7 | transport of nitrite | 0 | Not detected | 0.063 | -0.144 | 0.270 | 0.553 |
| ENZRXN0.7 | 6-phospho-Î²-D-glucosyl-(1 | 0 | Not detected | -0.180 | -0.470 | 0.110 | 0.225 |
| ENZRXN0.7 | guanosine 2'3'-cyclic phosp | 0 | Not detected | 0.174 | -0.111 | 0.458 | 0.231 |
| ENZRXN0.7 | uridine 2'3'-cyclic phospho | 0 | Not detected | 0.174 | -0.111 | 0.458 | 0.231 |
| ENZRXN0.7 | cytidine 2'3'-cyclic phospho | 0 | Not detected | 0.174 | -0.111 | 0.458 | 0.231 |
| ENZRXN0.7 | adenosine 2'3'-cyclic phosp | 0 | Not detected | 0.174 | -0.111 | 0.458 | 0.231 |
| ENZRXN0.7 | cytidine 3'-phosphate phos | 70 | Not detected | 0.260 | 0.048 | 0.473 | 0.017 |
| ENZRXN0.7 | adenosine 3'-phosphate pho | 70 | Not detected | 0.260 | 0.048 | 0.473 | 0.017 |
| ENZRXN0.7 | dGMP 5'-phosphate phosph | 70 | Not detected | 0.260 | 0.048 | 0.473 | 0.017 |
| ENZRXN0.7 | arsenate:H+ symporter | 0 | Not detected | 0.150 | -0.094 | 0.394 | 0.228 |
| ENZRXN0.7 | succinate transporter | 0 | Not detected | 0.037 | -0.213 | 0.287 | 0.771 |
| ENZRXN0.7 | fumarate transporter | 0 | Not detected | 0.037 | -0.213 | 0.287 | 0.771 |
| ENZRXN0.7 | aspartate transport | 0 | Not detected | 0.037 | -0.213 | 0.287 | 0.771 |
| ENZRXN0.7 | amylomaltase | 0 | Not detected | 0.050 | -0.218 | 0.318 | 0.713 |
| ENZRXN0.7 | amylomaltase | 0 | Not detected | 0.050 | -0.218 | 0.318 | 0.713 |
| ENZRXN0.7 | maltopentaose glucosidase | 0 | Not detected | 0.147 | -0.091 | 0.385 | 0.225 |
| ENZRXN0.7 | maltohexaose glucosidase | 0 | Not detected | 0.147 | -0.091 | 0.385 | 0.225 |
| ENZRXN0.7 | maltoheptaose glucosidase | 0 | Not detected | 0.147 | -0.091 | 0.385 | 0.225 |
| ENZRXN0.7 | GTPase | 0 | Not detected | 0.092 | -0.111 | 0.296 | 0.372 |
| ENZRXN0.7 | Na+:H+ antiporter | 0 | Not detected | 0.126 | -0.074 | 0.325 | 0.218 |
| ENZRXN0.7 | K+:H+ antiporter | 0 | Not detected | 0.126 | -0.074 | 0.325 | 0.218 |
| ENZRXN0.7 | phosphoethanolamine trans | 0 | Not detected | 0.013 | -0.228 | 0.254 | 0.916 |
| ENZRXN0.7 | phosphoethanolamine trans | 0 | Not detected | 0.036 | -0.203 | 0.275 | 0.771 |
| ENZRXN0.7 | cytidine 3' nucleotidase | 0 | Not detected | 0.174 | -0.111 | 0.458 | 0.231 |
| ENZRXN0.7 | guanosine 3' nucleotidase | 0 | Not detected | 0.174 | -0.111 | 0.458 | 0.231 |

| ENZRXN0.7 | adenosine 3' nucleotidase | 0 | Not detected | 0.174 | -0.111 | 0.458 | 0.231 |
| --- | --- | --- | --- | --- | --- | --- | --- |
| ENZRXN0.7 | uridine 3' nucleotidase | 0 | Not detected | 0.174 | -0.111 | 0.458 | 0.231 |
| ENZRXN0.7 | 5'-deoxyribonucleotidase | 19 | Not detected | 0.351 | 0.093 | 0.610 | 0.008 |
| ENZRXN0.7 | 2-octaprenylphenol hydrox | 0 | Not detected | 0.120 | -0.091 | 0.330 | 0.265 |
| ENZRXN0.7 | DNA-AP site lyase | 305 | Not detected | -0.267 | -0.581 | 0.047 | 0.096 |
| ENZRXN0.7 | transport of D-glucose | 0 | Not detected | 0.054 | -0.172 | 0.280 | 0.640 |
| ENZRXN0.7 | AMP kinase (GTP) | 0 | Not detected | -0.107 | -0.263 | 0.048 | 0.177 |
| ENZRXN0.7 | protein-lysine deacetylase | 516 | Not detected | -0.268 | -0.510 | -0.026 | 0.030 |
| ENZRXN0.7 | transport of glucose | 0 | Not detected | 0.275 | 0.038 | 0.513 | 0.023 |
| ENZRXN0.7 | transport of maltose | 0 | Not detected | 0.275 | 0.038 | 0.513 | 0.023 |
| ENZRXN0.7 | guanine transport | 0 | Not detected | 0.162 | -0.087 | 0.412 | 0.203 |
| ENZRXN0.7 | hypoxanthine transport | 0 | Not detected | 0.162 | -0.087 | 0.412 | 0.203 |
| ENZRXN0.7 | 5-aminomethyl-2-thiouridi | 0 | Not detected | 0.113 | -0.124 | 0.350 | 0.351 |
| ENZRXN0.7 | phosphoethanolamine trans | 0 | Not detected | 0.173 | -0.054 | 0.399 | 0.136 |
| ENZRXN0.7 | 6-deoxy-6-sulfofructose kin | 0 | Not detected | 0.143 | -0.064 | 0.350 | 0.176 |
| ENZRXN0.7 | 6-deoxy-6-sulfofructose-1- | 0 | Not detected | -0.022 | -0.230 | 0.187 | 0.840 |
| ENZRXN0.7 | sulfoquinovose transporter | 0 | Not detected | 0.094 | -0.152 | 0.339 | 0.454 |
| ENZRXN0.7 | uracil:H+ symporter | 0 | Not detected | 0.107 | -0.196 | 0.411 | 0.488 |
| ENZRXN0.7 | transport of a tripeptide | 0 | Not detected | 0.102 | -0.135 | 0.340 | 0.399 |
| ENZRXN0.7 | transport of a tripeptide | 0 | Not detected | 0.098 | -0.127 | 0.322 | 0.394 |
| ENZRXN0.7 | L-threonine dehydrogenase | 0 | Not detected | -0.188 | -0.495 | 0.120 | 0.232 |
| ENZRXN0.7 | 11-deoxycorticosterone red | 0 | Not detected | 0.138 | -0.074 | 0.351 | 0.203 |
| ENZRXN0.7 | D-gluconate 5-dehydrogen | 0 | Not detected | 0.138 | -0.074 | 0.351 | 0.203 |
| ENZRXN0.7 | 2-keto-3-deoxy-D-gluconat | 0 | Not detected | 0.138 | -0.074 | 0.351 | 0.203 |
| ENZRXN0.7 | sodium dodecyl sulfatase | 0 | Not detected | 0.036 | -0.217 | 0.289 | 0.779 |
| ENZRXN0.7 | isobutyraldehyde reductase | 0 | Not detected | 0.007 | -0.188 | 0.202 | 0.944 |
| ENZRXN0.7 | isobutyraldehyde reductase | 0 | Not detected | 0.077 | -0.122 | 0.276 | 0.449 |
| ENZRXN0.7 | isobutyraldehyde reductase | 0 | Not detected | 0.110 | -0.080 | 0.300 | 0.256 |
| ENZRXN0.7 | NADP+-dependent aldehyd | 0 | Not detected | 0.110 | -0.080 | 0.300 | 0.256 |
| ENZRXN0.7 | isobutyraldehyde reductase | 0 | Not detected | -0.116 | -0.415 | 0.183 | 0.447 |
| ENZRXN0.7 | 1-hydroxy-2-methyl-2-(E)- | 740 | 0.6 | -0.214 | -0.363 | -0.065 | 0.005 |
| ENZRXN0.7 | 3' &rarr; 5' ssDNA exonucl | 0 | Not detected | -0.007 | -0.200 | 0.187 | 0.946 |
| ENZRXN0.7 | 3' &rarr; 5' RNA exonuclea | 0 | Not detected | -0.007 | -0.200 | 0.187 | 0.946 |
| ENZRXN0.7 | ATP pyrophosphatase | 0 | Not detected | 0.203 | -0.056 | 0.461 | 0.125 |
| ENZRXN0.7 | 3-isopropylmalate dehydro | 0 | Not detected | -0.049 | -0.329 | 0.231 | 0.731 |
| ENZRXN0.7 | L-tartrate dehydrogenase | 0 | Not detected | -0.049 | -0.329 | 0.231 | 0.731 |
| ENZRXN0.7 | glycerophosphoglycerol ac | 0 | Not detected | 0.072 | -0.139 | 0.283 | 0.504 |
| ENZRXN0.7 | NADH:menaquinone oxido | 0 | Not detected | 0.067 | -0.157 | 0.291 | 0.559 |
| ENZRXN0.7 | polyphenol oxidase | 0 | Not detected | 0.096 | -0.108 | 0.299 | 0.357 |
| ENZRXN0.7 | PII-UMP deuridylylation | 0 | Not detected | 0.146 | -0.122 | 0.414 | 0.287 |
| ENZRXN0.7 | D-ribulose 5-phosphate 1-d | 0 | Not detected | -0.177 | -0.437 | 0.083 | 0.182 |
| ENZRXN0.7 | flavin mononucleotide (FM | 0 | Not detected | -0.050 | -0.263 | 0.164 | 0.648 |
| ENZRXN0.7 | flavin adenine dinucleotide | 0 | Not detected | -0.050 | -0.263 | 0.164 | 0.648 |
| ENZRXN0.7 | nitroimidazole export | 0 | Not detected | 0.172 | -0.029 | 0.374 | 0.094 |

| ENZRXN0.7 | L,D-carboxypeptidase | 0 | Not detected | 0.135 | -0.099 | 0.369 | 0.258 |
| --- | --- | --- | --- | --- | --- | --- | --- |
| ENZRXN0.7 | L,D-transpeptidase | 0 | Not detected | 0.135 | -0.099 | 0.369 | 0.258 |
| ENZRXN0.7 | L,D transpeptidase | 0 | Not detected | 0.141 | -0.083 | 0.365 | 0.219 |
| ENZRXN0.7 | L,D-transpeptidase | 0 | Not detected | 0.135 | -0.099 | 0.369 | 0.258 |
| ENZRXN0.7 | 5' &rarr; 3' exonuclease | 0 | Not detected | 0.108 | -0.082 | 0.298 | 0.266 |
| ENZRXN0.7 | 3',5'-nucleoside bisphospha | 0 | Not detected | 0.108 | -0.082 | 0.298 | 0.266 |
| ENZRXN0.7 | ATPase | 0 | Not detected | -0.090 | -0.240 | 0.061 | 0.243 |
| ENZRXN0.7 | acyl-acp:sn-glycerol-3-pho | 0 | Not detected | 0.208 | -0.083 | 0.499 | 0.161 |
| ENZRXN0.7 | acyl-acp:sn-glycerol-3-pho | 0 | Not detected | 0.208 | -0.083 | 0.499 | 0.161 |
| ENZRXN0.7 | acyl-acp:sn-glycerol-3-pho | 0 | Not detected | 0.208 | -0.083 | 0.499 | 0.161 |
| ENZRXN0.7 | acyl-[acp]:1-acyl-sn-glycer | 0 | Not detected | 0.043 | -0.160 | 0.247 | 0.676 |
| ENZRXN0.7 | acyl-[acp]:1-acyl-sn-glycer | 0 | Not detected | 0.043 | -0.160 | 0.247 | 0.676 |
| ENZRXN0.7 | acyl-[acp]:1-acyl-sn-glycer | 0 | Not detected | 0.043 | -0.160 | 0.247 | 0.676 |
| ENZRXN0.7 | acyl-[acp]:1-acyl-sn-glycer | 0 | Not detected | 0.043 | -0.160 | 0.247 | 0.676 |
| ENZRXN0.7 | acyl-[acp]:1-acyl-sn-glycer | 0 | Not detected | 0.043 | -0.160 | 0.247 | 0.676 |
| ENZRXN0.8 | magnesium hydrogenphosp | 0 | Not detected | 0.150 | -0.094 | 0.394 | 0.228 |
| ENZRXN0.8 | calcium hydrogenphosphat | 0 | Not detected | 0.150 | -0.094 | 0.394 | 0.228 |
| ENZRXN0.8 | zinc hydrogenphosphate:H | 0 | Not detected | 0.150 | -0.094 | 0.394 | 0.228 |
| ENZRXN0.8 | tellurite:H+ symporter | 0 | Not detected | 0.150 | -0.094 | 0.394 | 0.228 |
| ENZRXN0.8 | L-methionine:H+ antiport | 0 | Not detected | 0.132 | -0.095 | 0.359 | 0.255 |
| ENZRXN0.8 | L-leucine:H+ antiport | 0 | Not detected | 0.132 | -0.095 | 0.359 | 0.255 |
| ENZRXN0.8 | L-isoleucine:H+ antiport | 0 | Not detected | 0.132 | -0.095 | 0.359 | 0.255 |
| ENZRXN0.8 | L-valine:H+ antiport | 0 | Not detected | 0.132 | -0.095 | 0.359 | 0.255 |
| ENZRXN0.8 | farnesyl diphosphate phosp | 0 | Not detected | -0.054 | -0.291 | 0.182 | 0.653 |
| ENZRXN0.8 | L-cystine:H+ symporter | 0 | Not detected | 0.158 | -0.069 | 0.385 | 0.173 |
| ENZRXN0.8 | D-cystine:H+ symporter | 0 | Not detected | 0.158 | -0.069 | 0.385 | 0.173 |
| ENZRXN0.8 | homocystine:H+ symporter | 0 | Not detected | 0.158 | -0.069 | 0.385 | 0.173 |
| ENZRXN0.8 | apolipoprotein N-acyltransf | 0 | Not detected | 0.100 | -0.122 | 0.323 | 0.378 |
| ENZRXN0.8 | peptidoglycan lytic exotran | 0 | Not detected | -0.015 | -0.231 | 0.200 | 0.889 |
| ENZRXN0.8 | peptidoglycan lytic exotran | 0 | Not detected | 0.129 | -0.077 | 0.334 | 0.220 |
| ENZRXN0.8 | peptidoglycan lytic exotran | 0 | Not detected | 0.156 | -0.079 | 0.390 | 0.194 |
| ENZRXN0.8 | peptidoglycan lytic endotra | 0 | Not detected | 0.156 | -0.079 | 0.390 | 0.194 |
| ENZRXN0.8 | peptidoglycan lytic endotra | 0 | Not detected | 0.129 | -0.077 | 0.334 | 0.220 |
| ENZRXN0.8 | peptidoglycan lytic exotran | 0 | Not detected | 0.160 | -0.062 | 0.382 | 0.157 |
| ENZRXN0.8 | peptidoglycan lytic endotra | 0 | Not detected | 0.160 | -0.062 | 0.382 | 0.157 |
| ENZRXN0.8 | peptidoglycan lytic endotra | 0 | Not detected | 0.067 | -0.183 | 0.318 | 0.598 |
| ENZRXN0.8 | peptidoglycan lytic exotran | 0 | Not detected | 0.067 | -0.183 | 0.318 | 0.598 |
| ENZRXN0.8 | peptidoglycan lytic exotran | 0 | Not detected | 0.238 | 0.005 | 0.471 | 0.045 |
| ENZRXN0.8 | GTPase, ribosome-stimulat | 0 | Not detected | -0.094 | -0.243 | 0.054 | 0.215 |
| ENZRXN0.8 | transport of 2-acyl-1-lysop | 0 | Not detected | 0.044 | -0.175 | 0.262 | 0.693 |
| ENZRXN0.8 | transport of lyso-phosphati | 0 | Not detected | 0.044 | -0.175 | 0.262 | 0.693 |
| ENZRXN0.8 | poly-3-hydroxybutyrate syn | 0 | Not detected | -0.051 | -0.250 | 0.148 | 0.616 |
| ENZRXN0.8 | biotin-ATP ligase | 0 | Not detected | 0.050 | -0.162 | 0.262 | 0.644 |
| ENZRXN0.8 | sulfoquinovosidase | 0 | Not detected | -0.188 | -0.546 | 0.169 | 0.302 |

| ENZRXN0.8 | sulfoquinovosidase | 0 | Not detected | -0.188 | -0.546 | 0.169 | 0.302 |
| --- | --- | --- | --- | --- | --- | --- | --- |
| ENZRXN0.8 | (S)-2-hydroxybutanoate de | 0 | Not detected | 0.193 | -0.056 | 0.443 | 0.130 |
| ENZRXN0.8 | (S)-2-hydroxybutanoate tra | 0 | Not detected | 0.173 | -0.088 | 0.433 | 0.194 |
| ENZRXN0.8 | (S)-2-hydroxybutanoate tra | 0 | Not detected | -0.164 | -0.461 | 0.132 | 0.277 |
| ENZRXN0.8 | melibionate:H+ symporter | 0 | Not detected | 0.002 | -0.213 | 0.217 | 0.985 |
| ENZRXN0.8 | choline dehydrogenase | 0 | Not detected | 0.075 | -0.178 | 0.328 | 0.562 |
| ENZRXN0.8 | betaine aldehyde dehydrog | 0 | Not detected | 0.075 | -0.178 | 0.328 | 0.562 |
| ENZRXN0.8 | short chain acyl-CoA synth | 0 | Not detected | 0.033 | -0.242 | 0.309 | 0.813 |
| ENZRXN0.8 | cis-vaccenoyl-CoA synthet | 0 | Not detected | 0.033 | -0.242 | 0.309 | 0.813 |
| ENZRXN0.8 | oleoyl-CoA synthetase (tra | 0 | Not detected | 0.033 | -0.242 | 0.309 | 0.813 |
| ENZRXN0.8 | inositol monophosphatase | 0 | Not detected | -0.057 | -0.292 | 0.178 | 0.634 |
| ENZRXN0.8 | fumarate:H+ symporter | 0 | Not detected | -0.169 | -0.426 | 0.089 | 0.199 |
| ENZRXN0.8 | fumarate:H+ symporter | 0 | Not detected | 0.258 | 0.017 | 0.498 | 0.036 |
| ENZRXN0.8 | acyl-CoA dehydrogenase | 0 | Not detected | 0.082 | -0.174 | 0.338 | 0.529 |
| ENZRXN0.8 | acyl-CoA dehydrogenase | 0 | Not detected | 0.082 | -0.174 | 0.338 | 0.529 |
| ENZRXN0.8 | acyl-CoA dehydrogenase | 0 | Not detected | 0.082 | -0.174 | 0.338 | 0.529 |
| ENZRXN0.8 | acyl-CoA dehydrogenase | 0 | Not detected | 0.082 | -0.174 | 0.338 | 0.529 |
| ENZRXN0.8 | acyl-CoA dehydrogenase | 0 | Not detected | 0.082 | -0.174 | 0.338 | 0.529 |
| ENZRXN0.8 | acyl-CoA dehydrogenase | 0 | Not detected | 0.082 | -0.174 | 0.338 | 0.529 |
| ENZRXN0.8 | acyl-CoA dehydrogenase | 0 | Not detected | 0.082 | -0.174 | 0.338 | 0.529 |
| ENZRXN0.8 | enoyl-CoA hydratase | 0 | Not detected | 0.070 | -0.182 | 0.322 | 0.587 |
| ENZRXN0.8 | enoyl-CoA hydratase | 0 | Not detected | 0.070 | -0.182 | 0.322 | 0.587 |
| ENZRXN0.8 | enoyl-CoA hydratase | 0 | Not detected | 0.070 | -0.182 | 0.322 | 0.587 |
| ENZRXN0.8 | enoyl-CoA hydratase | 0 | Not detected | 0.070 | -0.182 | 0.322 | 0.587 |
| ENZRXN0.8 | enoyl-CoA hydratase | 0 | Not detected | 0.070 | -0.182 | 0.322 | 0.587 |
| ENZRXN0.8 | enoyl-CoA hydratase | 0 | Not detected | 0.070 | -0.182 | 0.322 | 0.587 |
| ENZRXN0.8 | enoyl-CoA hydratase | 0 | Not detected | 0.070 | -0.182 | 0.322 | 0.587 |
| ENZRXN0.8 | 3-hydroxyacyl-CoA dehydr | 0 | Not detected | 0.070 | -0.182 | 0.322 | 0.587 |
| ENZRXN0.8 | 3-hydroxyacyl-CoA dehydr | 0 | Not detected | 0.070 | -0.182 | 0.322 | 0.587 |
| ENZRXN0.8 | 3-hydroxyacyl-CoA dehydr | 0 | Not detected | 0.070 | -0.182 | 0.322 | 0.587 |
| ENZRXN0.8 | 3-hydroxyacyl-CoA dehydr | 0 | Not detected | 0.070 | -0.182 | 0.322 | 0.587 |
| ENZRXN0.8 | 3-hydroxyacyl-CoA dehydr | 0 | Not detected | 0.070 | -0.182 | 0.322 | 0.587 |
| ENZRXN0.8 | 3-hydroxyacyl-CoA dehydr | 0 | Not detected | 0.070 | -0.182 | 0.322 | 0.587 |
| ENZRXN0.8 | 3-hydroxyacyl-CoA dehydr | 0 | Not detected | 0.070 | -0.182 | 0.322 | 0.587 |
| ENZRXN0.8 | acetyl-CoA C-acyltransfera | 0 | Not detected | 0.094 | -0.120 | 0.309 | 0.388 |
| ENZRXN0.8 | acetyl-CoA C-acyltransfera | 0 | Not detected | 0.094 | -0.120 | 0.309 | 0.388 |
| ENZRXN0.8 | acetyl-CoA C-acyltransfera | 0 | Not detected | 0.094 | -0.120 | 0.309 | 0.388 |
| ENZRXN0.8 | acetyl-CoA C-acyltransfera | 0 | Not detected | 0.094 | -0.120 | 0.309 | 0.388 |
| ENZRXN0.8 | acetyl-CoA C-acyltransfera | 0 | Not detected | 0.094 | -0.120 | 0.309 | 0.388 |
| ENZRXN0.8 | acetyl-CoA C-acyltransfera | 0 | Not detected | 0.094 | -0.120 | 0.309 | 0.388 |
| ENZRXN0.8 | acetyl-CoA C-acyltransfera | 0 | Not detected | 0.094 | -0.120 | 0.309 | 0.388 |
| ENZRXN0.8 | palmitoleoyl-CoA syntheta | 0 | Not detected | 0.033 | -0.242 | 0.309 | 0.813 |
| ENZRXN0.8 | fumarate:H+ symport | 0 | Not detected | 0.065 | -0.229 | 0.360 | 0.664 |
| ENZRXN0.8 | succinate:H+ symporter | 0 | Not detected | 0.258 | 0.017 | 0.498 | 0.036 |

| ENZRXN0.8 | methylphosphotriester-DN | 0 | Not detected | 0.082 | -0.125 | 0.289 | 0.437 |
| --- | --- | --- | --- | --- | --- | --- | --- |
| ENZRXN0.8 | O-4-methylthymine-DNA | 0 | Not detected | 0.082 | -0.125 | 0.289 | 0.437 |
| ENZRXN0.8 | ferredoxin-NADP+ reducta | 0 | Not detected | 0.126 | -0.059 | 0.311 | 0.181 |
| ENZRXN0.8 | 2,6-dimethoxyphenol:oxyg | 0 | Not detected | 0.088 | -0.146 | 0.322 | 0.463 |
| ENZRXN0.8 | 4,5-DOPA dioxygenase ext | 0 | Not detected | 0.058 | -0.128 | 0.243 | 0.541 |
| ENZRXN0.8 | ssDNA-specific exonucleas | 0 | Not detected | 0.327 | 0.056 | 0.598 | 0.018 |
| ENZRXN0.8 | nitrophenyl phosphatase | 19 | Not detected | 0.351 | 0.093 | 0.610 | 0.008 |
| ENZRXN0.8 | diguanylate cyclase | 0 | Not detected | 0.050 | -0.155 | 0.256 | 0.630 |
| ENZRXN0.8 | P1,P3-bis(5'-adenosyl) trip | 19 | Not detected | 0.351 | 0.093 | 0.610 | 0.008 |
| ENZRXN0.8 | CDP-glycerol hydrolase | 19 | Not detected | 0.351 | 0.093 | 0.610 | 0.008 |
| ENZRXN0.8 | tRNATyr pseudouridine35 | 0 | Not detected | -0.054 | -0.256 | 0.147 | 0.597 |
| ENZRXN0.8 | GTPase | 0 | Not detected | 0.063 | -0.187 | 0.313 | 0.620 |
| ENZRXN0.8 | hydroxyphenyllactate dehy | 0 | Not detected | 0.003 | -0.199 | 0.205 | 0.974 |
| ENZRXN0.8 | recombination-promoting n | 0 | Not detected | 0.135 | -0.134 | 0.404 | 0.325 |
| ENZRXN0.8 | S-sulfocysteine:H+ sympor | 0 | Not detected | 0.158 | -0.069 | 0.385 | 0.173 |
| ENZRXN0.8 | periplasmic nitrite reductas | 0 | Not detected | 0.086 | -0.169 | 0.341 | 0.511 |
| ENZRXN0.8 | signal peptide peptidase | 0 | Not detected | 0.204 | -0.045 | 0.453 | 0.109 |
| ENZRXN0.8 | lipoprotein signal peptide p | 0 | Not detected | 0.204 | -0.045 | 0.453 | 0.109 |
| ENZRXN0.8 | L-cysteine desulfidase | 0 | Not detected | 0.138 | -0.104 | 0.381 | 0.264 |
| ENZRXN0.8 | rhomboid protease | 0 | Not detected | 0.043 | -0.153 | 0.239 | 0.665 |
| ENZRXN0.8 | 3' to 5' ATP-dependent DN | 0 | Not detected | 0.152 | -0.139 | 0.444 | 0.305 |
| ENZRXN0.8 | 5' to 3' ATP-dependent DN | 0 | Not detected | 0.248 | 0.004 | 0.492 | 0.047 |
| ENZRXN0.8 | cytochrome c peroxidase | 0 | Not detected | 0.165 | -0.094 | 0.424 | 0.211 |
| ENZRXN0.8 | Î²-barrel assembly-enhancin | 0 | Not detected | 0.013 | -0.219 | 0.246 | 0.910 |
| ENZRXN0.8 | 2-acyl lysophospholipase | 0 | Not detected | 0.072 | -0.139 | 0.283 | 0.504 |
| ENZRXN0.8 | 1-acyl lysophospholipase | 0 | Not detected | 0.072 | -0.139 | 0.283 | 0.504 |
| ENZRXN0.8 | pyruvate:H+ symporter | 0 | Not detected | 0.149 | -0.153 | 0.451 | 0.334 |
| ENZRXN0.8 | Na+ : H+ antiporter | 0 | Not detected | 0.123 | -0.103 | 0.350 | 0.287 |
| ENZRXN0.8 | K+ : H+ antiporter | 0 | Not detected | 0.123 | -0.103 | 0.350 | 0.287 |
| ENZRXN0.8 | dequalinium:H+ antiporter | 0 | Not detected | 0.126 | -0.074 | 0.325 | 0.218 |
| ENZRXN0.8 | ethidium:H+ antiporter | 0 | Not detected | 0.126 | -0.074 | 0.325 | 0.218 |
| ENZRXN0.8 | glucosylglycerate phosphor | 0 | Not detected | 0.086 | -0.128 | 0.300 | 0.431 |
| ENZRXN0.8 | carbonylcyanide m-chlorop | 0 | Not detected | 0.052 | -0.169 | 0.273 | 0.646 |
| ENZRXN0.8 | bicozamycin:H+ antiporter | 0 | Not detected | 0.185 | -0.022 | 0.392 | 0.080 |
| ENZRXN0.8 | chloramphenicol:H+ antipo | 0 | Not detected | 0.083 | -0.147 | 0.313 | 0.479 |
| ENZRXN0.8 | fosfomycin:H+ antiporter | 0 | Not detected | 0.147 | -0.065 | 0.359 | 0.175 |
| ENZRXN0.8 | Fe2+ transporter | 0 | Not detected | 0.196 | -0.081 | 0.473 | 0.166 |
| ENZRXN0.8 | trimethylamine N-oxide red | 0 | Not detected | 0.144 | -0.097 | 0.386 | 0.241 |
| ENZRXN0.8 | UDP kinase | 0 | Not detected | -0.107 | -0.263 | 0.048 | 0.177 |
| ENZRXN0.8 | dTDP kinase | 0 | Not detected | -0.107 | -0.263 | 0.048 | 0.177 |
| ENZRXN0.8 | CDP kinase | 0 | Not detected | -0.107 | -0.263 | 0.048 | 0.177 |
| ENZRXN0.8 | dCDP kinase | 0 | Not detected | -0.107 | -0.263 | 0.048 | 0.177 |
| ENZRXN0.8 | dGDP kinase | 0 | Not detected | -0.107 | -0.263 | 0.048 | 0.177 |
| ENZRXN0.8 | GDP kinase | 0 | Not detected | -0.107 | -0.263 | 0.048 | 0.177 |

| ENZRXN0.8 | celulose phosphoethanolam | 228 | Not detected | 0.316 | 0.073 | 0.560 | 0.011 |
| --- | --- | --- | --- | --- | --- | --- | --- |
| ENZRXN0.8 | thymine:H+ symporter | 0 | Not detected | 0.107 | -0.196 | 0.411 | 0.488 |
| ENZRXN0.8 | xanthine:H+ symporter | 0 | Not detected | 0.107 | -0.196 | 0.411 | 0.488 |
| ENZRXN0.8 | c-di-GMP phosphodiestera | 0 | Not detected | 0.090 | -0.144 | 0.324 | 0.450 |
| ENZRXN0.8 | apolipoprotein N-acyltransf | 0 | Not detected | 0.100 | -0.122 | 0.323 | 0.378 |
| ENZRXN0.8 | hydroquinone:hydrogen pe | 0 | Not detected | 0.165 | -0.094 | 0.424 | 0.211 |
| ENZRXN0.8 | menadiol:hydrogen peroxid | 0 | Not detected | 0.165 | -0.094 | 0.424 | 0.211 |
| ENZRXN0.8 | kojibiose phosphorylase | 0 | Not detected | -0.047 | -0.284 | 0.190 | 0.697 |
| ENZRXN0.8 | endo-poly-Î²-1,6-D-glucosa | 0 | Not detected | 0.117 | -0.120 | 0.353 | 0.334 |
| ENZRXN0.8 | L-aspartate:fumarate antipo | 0 | Not detected | 0.065 | -0.229 | 0.360 | 0.664 |
| ENZRXN0.8 | thiol-disulfide exchange | 0 | Not detected | 0.206 | -0.051 | 0.463 | 0.116 |
| ENZRXN0.8 | ferric enterobactin esterase | 0 | Not detected | 0.129 | -0.075 | 0.332 | 0.216 |
| ENZRXN0.8 | thiol-disulfide exchange | 0 | Not detected | 0.206 | -0.051 | 0.463 | 0.116 |
| ENZRXN0.8 | 5-aminovalerate aminotran | 17 | Not detected | 0.240 | 0.027 | 0.452 | 0.027 |
| ENZRXN0.8 | protein-histidine auto-kinas | 0 | Not detected | -0.006 | -0.229 | 0.216 | 0.956 |
| ENZRXN0.8 | ATP-dependent 3' &rarr; 5 | 0 | Not detected | 0.144 | -0.135 | 0.423 | 0.311 |
| ENZRXN0.8 | 5-aminopentanoate:H+ sym | 0 | Not detected | 0.087 | -0.127 | 0.300 | 0.425 |
| ENZRXN0.8 | 5' &rarr; 3' helicase | 0 | Not detected | 0.140 | -0.113 | 0.392 | 0.278 |
| ENZRXN0.8 | apurinic/apyrimidinic endo | 0 | Not detected | 0.072 | -0.115 | 0.260 | 0.450 |
| ENZRXN0.8 | 3'-phosphodiesterase | 0 | Not detected | 0.072 | -0.115 | 0.260 | 0.450 |
| ENZRXN0.8 | 3'-phosphomonoesterase | 0 | Not detected | 0.072 | -0.115 | 0.260 | 0.450 |
| ENZRXN0.8 | uracil DNA glycosylase | 305 | Not detected | -0.267 | -0.581 | 0.047 | 0.096 |
| ENZRXN0.8 | thymine glycol DNA glyco | 305 | Not detected | -0.267 | -0.581 | 0.047 | 0.096 |
| ENZRXN0.8 | protein-histidine autokinas | 0 | Not detected | 0.057 | -0.167 | 0.281 | 0.618 |
| ENZRXN185 | 1,4-alpha-glucan branching | 0 | Not detected | -0.161 | -0.439 | 0.117 | 0.256 |
| ENZRXN185 | phosphoglucomutase | 0 | Not detected | -0.073 | -0.451 | 0.305 | 0.706 |
| ENZRXN1F. | glutamate-1-semialdehyde | 0 | Not detected | -0.281 | -0.638 | 0.075 | 0.122 |
| ENZRXN3O. | aconitate hydratase | 0 | Not detected | -0.041 | -0.552 | 0.469 | 0.874 |
| ENZRXN3O. | aconitate hydratase | 0 | Not detected | -0.041 | -0.552 | 0.469 | 0.874 |
| ENZRXN490 | enolase | 0 | Not detected | -0.117 | -0.440 | 0.207 | 0.481 |
| ENZRXN66. | pyruvate carboxylase | 0 | Not detected | 0.097 | -0.223 | 0.417 | 0.552 |
| ENZRXN66. | citrate dehydratase | 0 | Not detected | -0.043 | -0.329 | 0.243 | 0.768 |
| ENZRXN66. | aconitate hydratase | 0 | Not detected | -0.043 | -0.329 | 0.243 | 0.768 |
| ENZRXN8J2 | 1-deoxyxylulose-5-phosph | 0 | Not detected | -0.046 | -0.416 | 0.324 | 0.806 |
| ENZRXN8J2 | GTP cyclohydrolase | 0 | Not detected | -0.186 | -0.394 | 0.022 | 0.080 |
| ENZRXN8J2 | orotate phosphoribosyltran | 0 | Not detected | -0.060 | -0.233 | 0.113 | 0.495 |
| ENZRXN8J2 | orotidine 5'-phosphate deca | 314 | Not detected | -0.288 | -0.505 | -0.070 | 0.010 |
| ENZRXN8J2 | aspartate carbamoyltransfer | 0 | Not detected | -0.163 | -0.486 | 0.160 | 0.324 |
| ENZRXN8J2 | UDP-glucose 6-dehydroge | 0 | Not detected | -0.178 | -0.494 | 0.137 | 0.268 |
| ENZRXN8J2 | 5-dehydro-2-deoxy-D-gluc | 311 | Not detected | -0.362 | -0.665 | -0.059 | 0.020 |
| ENZRXN8J2 | methionine-glutamine amin | 0 | Not detected | -0.151 | -0.430 | 0.127 | 0.287 |
| ENZRXN8J2 | lipoyl synthase | 0 | Not detected | 0.020 | -0.268 | 0.308 | 0.892 |
| ENZRXN8J2 | 4-nitrobenzaldehyde reduct | 0 | Not detected | -0.085 | -0.386 | 0.215 | 0.578 |
| ENZRXN8J2 | CTP synthetase | 0 | Not detected | -0.110 | -0.266 | 0.046 | 0.167 |

| ENZRXN8J2 | UTP-glucose-1-phosphate | 0 | Not detected | -0.118 | -0.336 | 0.099 | 0.287 |
| --- | --- | --- | --- | --- | --- | --- | --- |
| ENZRXN8J2 | 1,5-Î±-L-arabinofuranosida | 0 | Not detected | -0.125 | -0.385 | 0.134 | 0.345 |
| ENZRXN8J2 | 1,3-Î±-L-arabinofuranosida | 0 | Not detected | -0.060 | -0.230 | 0.109 | 0.486 |
| ENZRXNIO2 | alcohol dehydrogenase | 0 | Not detected | -0.177 | -0.465 | 0.111 | 0.228 |
| ENZRXNME | oxepin-CoA hydrolase | 0 | Not detected | 0.193 | -0.035 | 0.421 | 0.097 |
| ENZRXNME | 3-oxo-5,6-dehydrosuberyl- | 0 | Not detected | 0.193 | -0.035 | 0.421 | 0.097 |
| ENZRXNQT | biotin carboxylase | 0 | Not detected | -0.107 | -0.291 | 0.077 | 0.253 |
| ENZRXNQT | H+-exporting diphosphatas | 0 | Not detected | -0.052 | -0.373 | 0.268 | 0.749 |
| ENZRXNQT | NADP+-dependent isocitra | 0 | Not detected | -0.190 | -0.475 | 0.096 | 0.193 |
| ENZRXNQT | tryptophan synthase | 0 | Not detected | -0.042 | -0.348 | 0.265 | 0.790 |
| ENZRXNQT | aconitase | 0 | Not detected | -0.226 | -0.548 | 0.096 | 0.169 |
| ENZRXNQT | 3,4 dihydroxy-2-butanone- | 0 | Not detected | -0.064 | -0.224 | 0.096 | 0.436 |
| ENZRXNQT | GTP cyclohydrolase | 0 | Not detected | -0.064 | -0.224 | 0.096 | 0.436 |
| ENZRXNT9. | aconitase | 0 | Not detected | -0.226 | -0.548 | 0.096 | 0.169 |
| FADSYN.EN | FMN adenylyltransferase | 0 | Not detected | 0.161 | -0.055 | 0.377 | 0.144 |
| FGAMSYN.E | phosphoribosylformylglyci | 0 | Not detected | 0.306 | 0.039 | 0.573 | 0.025 |
| FLAVONAD | flavodoxin-NADP+ reducta | 0 | Not detected | 0.126 | -0.059 | 0.311 | 0.181 |
| FOLYLPOLY | folylpoly-&gamma;-glutam | 0 | Not detected | 0.191 | -0.033 | 0.415 | 0.095 |
| FORMYLTH | 10-formyl-tetrahydrofolylp | 0 | Not detected | 0.191 | -0.033 | 0.415 | 0.095 |
| FPPSYN.EN | farnesyl diphosphate syntha | 0 | Not detected | -0.112 | -0.355 | 0.132 | 0.369 |
| FUCULOKIN | L-fuculokinase | 0 | Not detected | -0.154 | -0.467 | 0.160 | 0.336 |
| GALACTAR | galactarate dehydratase | 0 | Not detected | 0.027 | -0.257 | 0.312 | 0.851 |
| GALACTOK | galactokinase | 0 | Not detected | 0.202 | -0.029 | 0.433 | 0.087 |
| GALACTON | D-galactonate dehydratase | 0 | Not detected | -0.259 | -0.534 | 0.016 | 0.065 |
| GALACTUR | D-galacturonate isomerase | 0 | Not detected | -0.045 | -0.193 | 0.102 | 0.548 |
| GALPMUT.E | UDP-galactopyranose muta | 0 | Not detected | -0.101 | -0.266 | 0.065 | 0.233 |
| GARTRANS | phosphoribosylglycinamide | 0 | Not detected | -0.110 | -0.348 | 0.128 | 0.366 |
| GARTSIDE.E | acetate kinase | 0 | Not detected | -0.110 | -0.348 | 0.128 | 0.366 |
| GCVT.ENZR | aminomethyltransferase | 0 | Not detected | -0.004 | -0.221 | 0.214 | 0.975 |
| GDPPYPHO | GDP pyrophosphokinase | 0 | Not detected | 0.258 | -0.025 | 0.542 | 0.074 |
| GDPPYPHO | GDP diphosphokinase | 0 | Not detected | 0.072 | -0.231 | 0.375 | 0.643 |
| GKI.ENZRX | glycerate 2-kinase | 0 | Not detected | 0.105 | -0.161 | 0.371 | 0.439 |
| GLNS.ENZR | glutamine&mdash;tRNA li | 0 | Not detected | -0.086 | -0.234 | 0.061 | 0.253 |
| GLU6PDEH | NADP+-dependent glucose | 0 | Not detected | 0.129 | -0.085 | 0.343 | 0.239 |
| GLUCARDE | D-glucarate dehydratase | 0 | Not detected | -0.023 | -0.305 | 0.260 | 0.876 |
| GLUCUROIS | D-glucuronate isomerase | 0 | Not detected | -0.045 | -0.193 | 0.102 | 0.548 |
| GLURS.ENZ | glutamate&mdash;tRNA li | 0 | Not detected | -0.301 | -0.618 | 0.015 | 0.062 |
| GLUTCYSLI | glutamate&mdash;cysteine | 0 | Not detected | 0.112 | -0.123 | 0.347 | 0.351 |
| GLUTRACE. | glutamate racemase | 14 | Not detected | 0.240 | 0.046 | 0.434 | 0.015 |
| GLYCEROL. | acyl-CoA:sn-glycerol-3-ph | 0 | Not detected | 0.208 | -0.083 | 0.499 | 0.161 |
| GLYCOGEN | 1,4-Î±-glucan branching en | 0 | Not detected | -0.017 | -0.276 | 0.243 | 0.901 |
| GLYCOGEN | glycogen synthase | 0 | Not detected | 0.294 | 0.050 | 0.538 | 0.018 |
| GLYRIBON | phosphoribosylamine&mda | 0 | Not detected | 0.055 | -0.097 | 0.208 | 0.476 |
| GPPSYN.EN | geranyl diphosphate syntha | 0 | Not detected | -0.112 | -0.355 | 0.132 | 0.369 |

| GSADENYL | glutamine synthetase adeny | 0 | Not detected | 0.207 | -0.091 | 0.505 | 0.174 |
| --- | --- | --- | --- | --- | --- | --- | --- |
| GSDEADEN | glutamine synthetase deade | 0 | Not detected | 0.207 | -0.091 | 0.505 | 0.174 |
| GTPPYPHOS | GTP pyrophosphokinase | 0 | Not detected | 0.258 | -0.025 | 0.542 | 0.074 |
| HEMEOSYN | heme O synthase | 0 | Not detected | 0.083 | -0.117 | 0.283 | 0.414 |
| HEMN.ENZR | coproporphyrinogen III deh | 0 | Not detected | 0.170 | -0.094 | 0.434 | 0.208 |
| HISTCYCLO | phosphoribosyl-AMP cyclo | 0 | Not detected | -0.080 | -0.248 | 0.087 | 0.347 |
| HISTPRATP | phosphoribosyl-ATP pyrop | 0 | Not detected | -0.080 | -0.248 | 0.087 | 0.347 |
| HOMOCYSM | cobalamin-independent ho | 0 | Not detected | 0.066 | -0.223 | 0.356 | 0.655 |
| HOMOCYSM | methionine synthase | 0 | Not detected | -0.023 | -0.171 | 0.125 | 0.757 |
| IGPSYN.EN | indole-3-glycerol phosphat | 0 | Not detected | 0.141 | -0.097 | 0.379 | 0.246 |
| ILES.ENZRX | isoleucine&mdash;tRNA li | 0 | Not detected | 0.259 | -0.073 | 0.591 | 0.126 |
| IMPCYCLO | IMP cyclohydrolase | 0 | Not detected | -0.074 | -0.280 | 0.132 | 0.483 |
| KDOTRANS | 3-deoxy-D-manno-octuloso | 0 | Not detected | 0.158 | -0.083 | 0.399 | 0.198 |
| KDOTRANS | KDO transferase | 0 | Not detected | 0.158 | -0.083 | 0.399 | 0.198 |
| L.ASPARTA | L-aspartate oxidase | 0 | Not detected | -0.214 | -0.558 | 0.131 | 0.224 |
| L.LACTDEH | L-lactate dehydrogenase | 0 | Not detected | 0.193 | -0.056 | 0.443 | 0.130 |
| LAUROYLA | lauroyl acyltransferase | 0 | Not detected | 0.130 | -0.081 | 0.340 | 0.229 |
| LEUS.ENZR | leucine&mdash;tRNA ligas | 0 | Not detected | 0.367 | 0.038 | 0.697 | 0.029 |
| LSERINEDE | L-serine deaminase | 0 | Not detected | -0.004 | -0.334 | 0.325 | 0.980 |
| LSERINEDE | L-serine deaminase | 0 | Not detected | 0.054 | -0.180 | 0.288 | 0.653 |
| MALATESY | malate synthase A | 0 | Not detected | -0.028 | -0.269 | 0.214 | 0.823 |
| MALONYL. | [acyl-carrier-protein] S-mal | 0 | Not detected | -0.218 | -0.507 | 0.071 | 0.139 |
| MALSYNG.E | malate synthase G | 0 | Not detected | -0.030 | -0.279 | 0.219 | 0.813 |
| MALTODEX | maltodextrin glucosidase | 0 | Not detected | 0.147 | -0.091 | 0.385 | 0.225 |
| MANNKIN.E | mannokinase | 0 | Not detected | 0.123 | -0.076 | 0.322 | 0.226 |
| MANNOND | D-mannonate dehydratase | 0 | Not detected | -0.091 | -0.300 | 0.119 | 0.396 |
| MANNONO | D-mannonate oxidoreducta | 0 | Not detected | 0.016 | -0.254 | 0.286 | 0.905 |
| MANNPDEH | mannitol-1-phosphate 5-de | 0 | Not detected | -0.028 | -0.324 | 0.269 | 0.856 |
| MANNPISO | mannose-6-phosphate isom | 0 | Not detected | 0.143 | -0.051 | 0.336 | 0.148 |
| MHPHYDRO | 3-(3-hydroxyphenyl)propan | 0 | Not detected | 0.229 | 0.003 | 0.454 | 0.047 |
| MMUM.ENZ | homocysteine S-methyltran | 0 | Not detected | 0.192 | -0.038 | 0.423 | 0.102 |
| N.ACETYLG | N-acetylglutamylphosphate | 0 | Not detected | -0.033 | -0.313 | 0.246 | 0.816 |
| NACGLCTR | N-acetylglucosaminyl trans | 0 | Not detected | 0.113 | -0.108 | 0.333 | 0.317 |
| NACMURLA | N-acetylmuramoyl-L-alanin | 0 | Not detected | 0.109 | -0.123 | 0.341 | 0.358 |
| NADH.DHII. | NADH:ubiquinone oxidore | 0 | Not detected | 0.067 | -0.157 | 0.291 | 0.559 |
| NANK.ENZR | N-acetylmannosamine kina | 0 | Not detected | -0.040 | -0.249 | 0.170 | 0.711 |
| NICOTINAT | nicotinate phosphoribosyltr | 0 | Not detected | 0.094 | -0.109 | 0.297 | 0.363 |
| O.SUCCINY | o-succinylbenzoate synthas | 0 | Not detected | 0.171 | -0.046 | 0.388 | 0.123 |
| OCTPRNL.M | 2-octaprenyl-3-methyl-6-m | 0 | Not detected | 0.010 | -0.204 | 0.223 | 0.930 |
| OHMETHYL | hydroxymethylbilane synth | 0 | Not detected | -0.058 | -0.273 | 0.157 | 0.598 |
| PEPCARBO | phosphoenolpyruvate carbo | 0 | Not detected | -0.057 | -0.206 | 0.093 | 0.458 |
| PGLYCERO | phosphoglycerol transferas | 0 | Not detected | 0.019 | -0.239 | 0.278 | 0.884 |
| PGMI.ENZR | 2,3-bisphosphoglycerate-in | 0 | Not detected | -0.120 | -0.270 | 0.030 | 0.117 |
| PHOSGLUC | phosphoglucosamine mutas | 0 | Not detected | -0.031 | -0.325 | 0.263 | 0.835 |

| PHOSGLYC | phosphoglycerate kinase | 0 | Not detected | 0.020 | -0.262 | 0.302 | 0.889 |
| --- | --- | --- | --- | --- | --- | --- | --- |
| PHOSMANM | phosphomannomutase | 0 | Not detected | -0.099 | -0.430 | 0.231 | 0.556 |
| PHOSNACM | phospho-N-acetylmuramoy | 0 | Not detected | 0.073 | -0.223 | 0.369 | 0.629 |
| PHOSPHASE | phosphatidylserine synthas | 0 | Not detected | 0.099 | -0.158 | 0.356 | 0.451 |
| PHOSPHOG | phosphoglucomutase | 0 | Not detected | 0.022 | -0.253 | 0.296 | 0.878 |
| PPENTOMU | Î±-D-ribose 1,5-phosphom | 0 | Not detected | -0.083 | -0.413 | 0.246 | 0.620 |
| PRAISOM.E | phosphoribosylanthranilate | 0 | Not detected | 0.141 | -0.097 | 0.379 | 0.246 |
| PRIBFAICA | 1-(5-phosphoribosyl)-5-[(5 | 295 | Not detected | -0.279 | -0.550 | -0.008 | 0.044 |
| PROPKIN.E | propionate kinase | 0 | Not detected | 0.150 | -0.055 | 0.354 | 0.152 |
| PROPKIN2.E | propionate kinase | 0 | Not detected | 0.051 | -0.184 | 0.287 | 0.669 |
| PROTOHEM | ferrochelatase | 0 | Not detected | 0.056 | -0.165 | 0.276 | 0.620 |
| PRPPSYN.E | ribose-phosphate diphosph | 666 | Not detected | -0.195 | -0.357 | -0.033 | 0.019 |
| PSERPHOSP | phosphoserine phosphatase | 0 | Not detected | 0.087 | -0.135 | 0.308 | 0.443 |
| PSII.ENZRX | guanosine-3',5'-bis(diphosp | 0 | Not detected | 0.072 | -0.231 | 0.375 | 0.643 |
| RHAMNULO | rhamnulokinase | 0 | Not detected | 0.150 | -0.090 | 0.391 | 0.221 |
| RIBOFLAVI | riboflavin kinase | 0 | Not detected | 0.161 | -0.055 | 0.377 | 0.144 |
| RIBULP3EPI | ribulose-phosphate 3-epim | 0 | Not detected | -0.039 | -0.251 | 0.173 | 0.717 |
| SHIKIMATE | shikimate dehydrogenase | 0 | Not detected | 0.172 | -0.029 | 0.373 | 0.094 |
| SHIKIMATE | shikimate kinase | 0 | Not detected | -0.003 | -0.203 | 0.197 | 0.979 |
| SUCCGLUA | succinylglutamate semialde | 0 | Not detected | 0.021 | -0.210 | 0.252 | 0.856 |
| TDPFUCAC | dTDP-4-amino-4,6-dideox | 0 | Not detected | 0.171 | -0.014 | 0.357 | 0.071 |
| TETRAACY | tetraacyldisaccharide 4'-kin | 0 | Not detected | 0.078 | -0.126 | 0.283 | 0.453 |
| THI.P.KIN.E | thiamine monophosphate k | 0 | Not detected | 0.060 | -0.155 | 0.276 | 0.583 |
| THIC.ENZR | phosphomethylpyrimidine | 0 | Not detected | -0.020 | -0.170 | 0.131 | 0.797 |
| THRESYN.E | threonine synthase | 0 | Not detected | 0.085 | -0.183 | 0.353 | 0.535 |
| TRANS.ENZ | transport of arabinose | 0 | Not detected | -0.063 | -0.359 | 0.232 | 0.674 |
| TRANS.ENZ | (S)-lactate transporter | 0 | Not detected | 0.173 | -0.088 | 0.433 | 0.194 |
| TRANS.ENZ | glycolate transporter | 0 | Not detected | -0.164 | -0.461 | 0.132 | 0.277 |
| TRANS.ENZ | succinate:fumarate antiport | 0 | Not detected | 0.065 | -0.229 | 0.360 | 0.664 |
| TRANS.ENZ | fumarate:succinate antiport | 0 | Not detected | -0.169 | -0.426 | 0.089 | 0.199 |
| TRANS.ENZ | transport of adenosine | 0 | Not detected | -0.048 | -0.310 | 0.214 | 0.720 |
| TRANS.ENZ | transport of cytidine | 0 | Not detected | -0.048 | -0.310 | 0.214 | 0.720 |
| TRANS.ENZ | transport of deoxyadenosin | 0 | Not detected | -0.048 | -0.310 | 0.214 | 0.720 |
| TRANS.ENZ | transport of deoxycytidine | 0 | Not detected | -0.048 | -0.310 | 0.214 | 0.720 |
| TRANS.ENZ | transport of deoxyuridine | 0 | Not detected | -0.048 | -0.310 | 0.214 | 0.720 |
| TRANS.ENZ | transport of thymidine | 0 | Not detected | -0.048 | -0.310 | 0.214 | 0.720 |
| TRANS.ENZ | transport of uridine | 0 | Not detected | -0.048 | -0.310 | 0.214 | 0.720 |
| TRANS.ENZ | transport of rhamnose | 16 | Not detected | 0.253 | 0.048 | 0.458 | 0.016 |
| TRANS.ENZ | phosphate:H+ symporter | 0 | Not detected | 0.150 | -0.094 | 0.394 | 0.228 |
| TRANS.ENZ | cytosine transporter | 0 | Not detected | 0.034 | -0.216 | 0.285 | 0.787 |
| TRANS.ENZ | pantothenate:Na+ symporte | 0 | Not detected | 0.049 | -0.213 | 0.310 | 0.715 |
| TRANS.ENZ | proline:Na+ symporter | 0 | Not detected | 0.007 | -0.299 | 0.313 | 0.963 |
| TRANS.ENZ | transport of succinate | 0 | Not detected | 0.046 | -0.262 | 0.353 | 0.772 |
| TRANS.ENZ | transport of malate | 0 | Not detected | 0.046 | -0.262 | 0.353 | 0.772 |

| TRANS.ENZ | transport of fumarate | 0 | Not detected | 0.046 | -0.262 | 0.353 | 0.772 |
| --- | --- | --- | --- | --- | --- | --- | --- |
| TRANS.ENZ | transport of orotate | 0 | Not detected | 0.046 | -0.262 | 0.353 | 0.772 |
| TRANS.ENZ | L-glutamate:H+ symporter | 0 | Not detected | -0.111 | -0.349 | 0.126 | 0.359 |
| TRANS.ENZ | L-aspartate:H+ symporter | 0 | Not detected | -0.111 | -0.349 | 0.126 | 0.359 |
| TRANS.ENZ | transport of glutamate | 0 | Not detected | 0.087 | -0.167 | 0.342 | 0.502 |
| TRANS.ENZ | branched chain amino acid | 0 | Not detected | 0.061 | -0.163 | 0.284 | 0.594 |
| TRANS.ENZ | branched chain amino acid | 0 | Not detected | 0.061 | -0.163 | 0.284 | 0.594 |
| TRANS.ENZ | branched chain amino acid | 0 | Not detected | 0.061 | -0.163 | 0.284 | 0.594 |
| TRANS.ENZ | L-tartrate:succinate antipor | 0 | Not detected | 0.178 | -0.040 | 0.396 | 0.110 |
| TRANS.ENZ | Na+:H+ antiporter | 0 | Not detected | 0.093 | -0.175 | 0.360 | 0.498 |
| TRANS.ENZ | Mg2+ transporter | 0 | Not detected | 0.218 | -0.012 | 0.448 | 0.063 |
| TRANS.ENZ | Co2+ transporter | 0 | Not detected | 0.218 | -0.012 | 0.448 | 0.063 |
| TRANS.ENZ | Ni2+ transporter | 0 | Not detected | 0.218 | -0.012 | 0.448 | 0.063 |
| TRANS.ENZ | K+ : H+ antiporter KefB | 0 | Not detected | 0.096 | -0.155 | 0.346 | 0.454 |
| TRANS.ENZ | K+:H+ symporter Kup | 0 | Not detected | 0.085 | -0.171 | 0.341 | 0.515 |
| TRANS.ENZ | fucose:H+ symporter | 0 | Not detected | 0.088 | -0.131 | 0.307 | 0.431 |
| TRANS.ENZ | shikimate:H+ symporter | 0 | Not detected | 0.098 | -0.104 | 0.299 | 0.343 |
| TRANS.ENZ | fosmidomycin efflux pump | 0 | Not detected | -0.136 | -0.317 | 0.046 | 0.143 |
| TRANS.ENZ | transport of 3-(3-hydroxyp | 0 | Not detected | 0.159 | -0.055 | 0.373 | 0.146 |
| TRANS.ENZ | sugar exporter SetB | 0 | Not detected | 0.106 | -0.125 | 0.338 | 0.369 |
| TRANS.ENZ | transport of D-galactose | 0 | Not detected | 0.054 | -0.172 | 0.280 | 0.640 |
| TRANS.ENZ | Mn2+:H+ symporter | 0 | Not detected | 0.157 | -0.049 | 0.363 | 0.135 |
| TRANS.ENZ | L-threonine:H+ antiporter | 0 | Not detected | 0.137 | -0.097 | 0.371 | 0.253 |
| TRANS.ENZ | K+ transporter TrkH | 0 | Not detected | 0.031 | -0.227 | 0.289 | 0.814 |
| TRANS.ENZ | sn-glycerol 3-phosphate:ph | 0 | Not detected | -0.096 | -0.373 | 0.180 | 0.495 |
| TRANS.ENZ | transport of 2-oxoglutarate | 0 | Not detected | 0.045 | -0.166 | 0.256 | 0.674 |
| TRANS.ENZ | lactose:H+ symporter LacY | 0 | Not detected | 0.002 | -0.213 | 0.217 | 0.985 |
| TRANS.ENZ | N-acetylneuraminate:H+ sy | 0 | Not detected | -0.013 | -0.239 | 0.214 | 0.913 |
| TRANS.ENZ | transport of a nucleoside | 0 | Not detected | 0.078 | -0.267 | 0.423 | 0.659 |
| TRANS.ENZ | transport of D-xylose | 0 | Not detected | -0.165 | -0.411 | 0.082 | 0.191 |
| TRANS.ENZ | xanthosine:H+ symporter X | 0 | Not detected | -0.202 | -0.451 | 0.047 | 0.113 |
| TRANS.ENZ | glucose-6-phosphate:phosp | 0 | Not detected | 0.140 | -0.100 | 0.381 | 0.254 |
| TRANS.ENZ | D-glucuronate transporter | 0 | Not detected | 0.115 | -0.104 | 0.335 | 0.304 |
| TRANS.ENZ | D-galacturonate transporter | 0 | Not detected | 0.115 | -0.104 | 0.335 | 0.304 |
| TRANS.ENZ | L-arabinose exporter | 0 | Not detected | 0.012 | -0.184 | 0.207 | 0.907 |
| TRANS.ENZ | L-phenylalanine:H+ sympo | 0 | Not detected | -0.142 | -0.411 | 0.127 | 0.301 |
| TRANS.ENZ | L-tyrosine:H+ symporter | 0 | Not detected | -0.142 | -0.411 | 0.127 | 0.301 |
| TRANS.ENZ | L-tryptophan:H+ symporte | 0 | Not detected | -0.142 | -0.411 | 0.127 | 0.301 |
| TRANS.ENZ | 4-aminobutanoate:H+ symp | 0 | Not detected | 0.087 | -0.127 | 0.300 | 0.425 |
| TRANS.ENZ | lysine:H+ symporter | 0 | Not detected | 0.171 | -0.143 | 0.485 | 0.285 |
| TRANS.ENZ | phenylalanine:H+ symporte | 0 | Not detected | -0.103 | -0.392 | 0.186 | 0.485 |
| TRANS.ENZ | transport of D-alanine | 0 | Not detected | 0.186 | -0.086 | 0.458 | 0.181 |
| TRANS.ENZ | transport of glycine | 0 | Not detected | 0.186 | -0.086 | 0.458 | 0.181 |
| TRANS.ENZ | lysine:cadaverine antiporte | 0 | Not detected | -0.123 | -0.413 | 0.167 | 0.406 |

| TRANS.ENZ | serine:H+ symporter SdaC | 0 | Not detected | 0.050 | -0.222 | 0.321 | 0.719 |
| --- | --- | --- | --- | --- | --- | --- | --- |
| TRANS.ENZ | L-threonine:H+ symporter | 0 | Not detected | 0.243 | 0.027 | 0.460 | 0.028 |
| TRANS.ENZ | L-serine:H+ symporter | 0 | Not detected | 0.243 | 0.027 | 0.460 | 0.028 |
| TRANS.ENZ | tryptophan:H+ symporter | 702 | 0.6 | 0.422 | 0.173 | 0.671 | 0.001 |
| TRANS.ENZ | indole:H+ symporter | 702 | 0.6 | 0.422 | 0.173 | 0.671 | 0.001 |
| TRANS.ENZ | choline:H+ symporter | 0 | Not detected | 0.061 | -0.193 | 0.315 | 0.637 |
| TRE6PHYDR | trehalose-6-phosphate hydr | 0 | Not detected | -0.003 | -0.310 | 0.304 | 0.985 |
| TREHALAC | trehalase | 0 | Not detected | 0.250 | 0.010 | 0.491 | 0.042 |
| TREHALAPE | trehalase | 0 | Not detected | 0.045 | -0.172 | 0.261 | 0.686 |
| TREHALOSE | trehalose-6-phosphate synt | 0 | Not detected | 0.150 | -0.057 | 0.357 | 0.155 |
| TSA.REDUC | tartronate semialdehyde red | 0 | Not detected | 0.003 | -0.251 | 0.257 | 0.982 |
| UDP.NACM | UDP-N-acetylmuramoyl-L- | 0 | Not detected | 0.280 | 0.033 | 0.527 | 0.027 |
| UDP.NACM | D-alanyl-D-alanine-adding | 0 | Not detected | 0.199 | -0.042 | 0.441 | 0.105 |
| UDP.NACM | UDP-N-acetylmuramoyl-L- | 0 | Not detected | 0.171 | -0.088 | 0.430 | 0.195 |
| UDPACYLG | UDP-3-O-acyl-N-acetylglu | 0 | Not detected | 0.045 | -0.269 | 0.358 | 0.780 |
| UDPNACET | UDP-N-acetylglucosamine | 0 | Not detected | 0.094 | -0.192 | 0.380 | 0.519 |
| UDPNACET | UDP-N-acetylenolpyruvoyl | 0 | Not detected | 0.189 | -0.040 | 0.418 | 0.106 |
| UDPSUGAR | UDP-sugar hydrolase | 19 | Not detected | 0.351 | 0.093 | 0.610 | 0.008 |
| URITRANS. | [protein-PII] uridylyltransf | 0 | Not detected | 0.146 | -0.122 | 0.414 | 0.287 |
| URKI.ENZR | uridine kinase | 0 | Not detected | 0.136 | -0.162 | 0.434 | 0.370 |
| UROGENDE | uroporphyrinogen decarbox | 0 | Not detected | 0.208 | -0.093 | 0.509 | 0.177 |
| VALINE.PY | valine&mdash;pyruvate am | 0 | Not detected | 0.111 | -0.109 | 0.332 | 0.323 |
| VALS.ENZR | valine&mdash;tRNA ligase | 0 | Not detected | 0.026 | -0.317 | 0.369 | 0.881 |
| X1.ACYLGL | acyl-CoA:1-acyl-sn-glycero | 0 | Not detected | 0.043 | -0.160 | 0.247 | 0.676 |
| X2.OCTAPR | 2-octaprenyl-6-methoxy-1, | 0 | Not detected | -0.014 | -0.316 | 0.287 | 0.926 |
| X2.OCTPRN | 2-octaprenyl-6-methoxyphe | 0 | Not detected | 0.085 | -0.133 | 0.304 | 0.445 |
| X3.DEHYDR | 3-dehydroquinate synthase | 0 | Not detected | 0.182 | -0.085 | 0.450 | 0.182 |
| X3.NUCLEO | 3'-nucleotidase | 0 | Not detected | 0.174 | -0.111 | 0.458 | 0.231 |
| X3.P.SHIK.1 | 3-phosphoshikimate 1-carb | 0 | Not detected | 0.201 | -0.046 | 0.449 | 0.111 |
| X4OHBENZ | 4-hydroxybenzoate octapre | 0 | Not detected | 0.046 | -0.151 | 0.243 | 0.647 |
| X5.NUCLEO | 5'-ribonucleotidase | 19 | Not detected | 0.351 | 0.093 | 0.610 | 0.008 |
| X6PGLUCO | 6-phosphogluconolactonas | 0 | Not detected | 0.193 | 0.003 | 0.382 | 0.047 |

| **Supplementary Table 12**. Association of current economic hardship with species. Analysis of Compositions of Microbiomes (ANCOM2) was used to identify species related to current economic hardship. Regression coefficients were obtained by modelling the effect of current economic hardship on centered log ratio (CLR)-transformed species in multivariable linear regression adjusted for age, gender, BMI, field center, Hispanic/Latino background, U.S. nativity, Bristol stool type, parental education, the alternative Healthy Eating Index 2010 score, cigarette smoking, alcohol use, depressive and anxiety symptoms, diabetes, hypertension, cardiovascular disease, dyslipidemia, and antibiotic use. | | | | | | |
| --- | --- | --- | --- | --- | --- | --- |
| **species** | **W_stat** | **ANCOM** | **beta** | **l95ci** | **u95ci** | **qval** |
| Abiotrophia defectiva | 0 | Not detected | 0.184 | -0.030 | 0.399 | 0.093 |
| Acetanaerobacterium elongatum | 0 | Not detected | 0.009 | -0.142 | 0.161 | 0.902 |
| Acetitomaculum ruminis | 0 | Not detected | 0.001 | -0.098 | 0.100 | 0.986 |
| Acetivibrio cellulolyticus | 0 | Not detected | 0.054 | -0.105 | 0.213 | 0.505 |
| Acetivibrio ethanolgignens | 0 | Not detected | -0.003 | -0.092 | 0.085 | 0.940 |
| Acetobacter sp. 46_36 | 0 | Not detected | 0.016 | -0.326 | 0.359 | 0.925 |
| Acetobacter sp. CAG:267 | 0 | Not detected | -0.085 | -0.388 | 0.217 | 0.580 |
| Acholeplasma axanthum | 0 | Not detected | 0.076 | -0.070 | 0.221 | 0.309 |
| Acholeplasma equifetale | 0 | Not detected | 0.254 | 0.056 | 0.453 | 0.012 |
| Acidaminococcus fermentans | 0 | Not detected | -0.064 | -0.238 | 0.111 | 0.474 |
| Acidaminococcus intestini | 0 | Not detected | 0.037 | -0.279 | 0.353 | 0.818 |
| Acidaminococcus intestini CAG:325 | 0 | Not detected | -0.037 | -0.493 | 0.420 | 0.874 |
| Acidaminococcus sp. CAG:542 | 0 | Not detected | -0.090 | -0.329 | 0.149 | 0.461 |
| Acinetobacter baumannii | 0 | Not detected | 0.000 | -0.174 | 0.174 | 0.998 |
| Acinetobacter bereziniae | 0 | Not detected | -0.061 | -0.231 | 0.109 | 0.483 |
| Acinetobacter johnsonii | 0 | Not detected | -0.025 | -0.214 | 0.164 | 0.799 |
| Acinetobacter sp. CAG:196 | 0 | Not detected | 0.282 | -0.058 | 0.622 | 0.105 |
| Acinetobacter sp. CAG:196_36_41 | 0 | Not detected | 0.367 | 0.037 | 0.696 | 0.029 |
| Acinetobacter sp. N54.MGS-139 | 0 | Not detected | -0.046 | -0.257 | 0.164 | 0.666 |
| Acinetobacter venetianus | 0 | Not detected | 0.248 | 0.033 | 0.463 | 0.024 |
| Actinobaculum sp. oral taxon 183 | 0 | Not detected | -0.022 | -0.267 | 0.224 | 0.862 |
| Actinobaculum suis | 0 | Not detected | 0.027 | -0.152 | 0.206 | 0.768 |
| Actinomyces dentalis | 0 | Not detected | 0.113 | -0.141 | 0.368 | 0.382 |
| Actinomyces gerencseriae | 0 | Not detected | 0.043 | -0.181 | 0.267 | 0.709 |
| Actinomyces graevenitzii | 0 | Not detected | -0.040 | -0.217 | 0.136 | 0.654 |
| Actinomyces hongkongensis | 0 | Not detected | -0.042 | -0.268 | 0.184 | 0.717 |
| Actinomyces israelii | 0 | Not detected | 0.015 | -0.225 | 0.255 | 0.900 |
| Actinomyces johnsonii | 0 | Not detected | 0.056 | -0.182 | 0.294 | 0.645 |
| Actinomyces massiliensis | 0 | Not detected | 0.017 | -0.218 | 0.251 | 0.889 |
| Actinomyces meyeri | 0 | Not detected | 0.028 | -0.165 | 0.221 | 0.778 |
| Actinomyces odontolyticus | 0 | Not detected | 0.052 | -0.141 | 0.246 | 0.595 |
| Actinomyces oris | 0 | Not detected | -0.066 | -0.297 | 0.166 | 0.577 |
| Actinomyces sp. HMSC065F12 | 0 | Not detected | -0.018 | -0.151 | 0.115 | 0.791 |
| Actinomyces sp. HMSC06A08 | 0 | Not detected | -0.046 | -0.181 | 0.089 | 0.506 |
| Actinomyces sp. oral taxon 181 | 0 | Not detected | 0.000 | -0.238 | 0.237 | 0.998 |
| Actinomyces sp. oral taxon 448 | 0 | Not detected | -0.055 | -0.321 | 0.211 | 0.685 |

| Actinomyces sp. oral taxon 848 | 0 | Not detected | 0.213 | 0.038 | 0.388 | 0.018 |
| --- | --- | --- | --- | --- | --- | --- |
| Actinomyces sp. ph3 | 0 | Not detected | -0.093 | -0.318 | 0.133 | 0.420 |
| Actinomyces timonensis | 0 | Not detected | -0.012 | -0.160 | 0.137 | 0.876 |
| Actinomyces turicensis | 0 | Not detected | 0.217 | -0.009 | 0.443 | 0.060 |
| Adlercreutzia equolifaciens | 0 | Not detected | 0.082 | -0.217 | 0.382 | 0.590 |
| Aeromonas salmonicida | 0 | Not detected | -0.039 | -0.260 | 0.182 | 0.727 |
| Aggregatibacter aphrophilus | 0 | Not detected | -0.112 | -0.362 | 0.139 | 0.382 |
| Akkermansia glycaniphila | 0 | Not detected | 0.073 | -0.149 | 0.296 | 0.518 |
| Akkermansia muciniphila | 0 | Not detected | 0.025 | -0.307 | 0.357 | 0.883 |
| Akkermansia muciniphila CAG:154 | 0 | Not detected | -0.015 | -0.440 | 0.410 | 0.943 |
| Akkermansia sp. 54_46 | 0 | Not detected | 0.007 | -0.432 | 0.446 | 0.976 |
| Akkermansia sp. CAG:344 | 0 | Not detected | 0.255 | -0.182 | 0.691 | 0.253 |
| Akkermansia sp. KLE1605 | 0 | Not detected | 0.122 | -0.232 | 0.477 | 0.498 |
| Akkermansia sp. KLE1797 | 0 | Not detected | 0.107 | -0.247 | 0.462 | 0.553 |
| Akkermansia sp. KLE1798 | 0 | Not detected | 0.122 | -0.233 | 0.476 | 0.502 |
| Akkermansia sp. Phil8 | 0 | Not detected | 0.050 | -0.387 | 0.486 | 0.824 |
| Akkermansia sp. UNK.MGS-1 | 0 | Not detected | -0.029 | -0.464 | 0.405 | 0.894 |
| Alistipes finegoldii | 0 | Not detected | 0.075 | -0.108 | 0.259 | 0.419 |
| Alistipes ihumii | 0 | Not detected | 0.166 | -0.137 | 0.469 | 0.284 |
| Alistipes indistinctus | 0 | Not detected | 0.228 | -0.174 | 0.630 | 0.266 |
| Alistipes inops | 0 | Not detected | 0.079 | -0.243 | 0.401 | 0.632 |
| Alistipes obesi | 0 | Not detected | 0.160 | -0.040 | 0.360 | 0.117 |
| Alistipes putredinis | 0 | Not detected | 0.039 | -0.221 | 0.299 | 0.770 |
| Alistipes senegalensis | 0 | Not detected | 0.061 | -0.105 | 0.228 | 0.473 |
| Alistipes shahii | 0 | Not detected | 0.040 | -0.143 | 0.222 | 0.671 |
| Alistipes sp. CAG:435 | 0 | Not detected | -0.126 | -0.527 | 0.275 | 0.538 |
| Alistipes sp. CAG:514 | 0 | Not detected | 0.146 | -0.223 | 0.514 | 0.439 |
| Alistipes sp. Zagget8 | 0 | Not detected | 0.118 | -0.136 | 0.372 | 0.362 |
| Alistipes timonensis | 0 | Not detected | 0.210 | -0.054 | 0.475 | 0.120 |
| Alkaliflexus imshenetskii | 0 | Not detected | -0.026 | -0.195 | 0.142 | 0.759 |
| Allisonella histaminiformans | 0 | Not detected | -0.136 | -0.514 | 0.241 | 0.479 |
| Allobaculum stercoricanis | 0 | Not detected | -0.033 | -0.126 | 0.061 | 0.494 |
| Allofustis seminis | 0 | Not detected | 0.190 | -0.007 | 0.386 | 0.059 |
| Alloprevotella rava | 0 | Not detected | 0.066 | -0.098 | 0.230 | 0.429 |
| Alloprevotella tannerae | 0 | Not detected | 0.003 | -0.121 | 0.127 | 0.963 |
| Alloscardovia criceti | 0 | Not detected | -0.030 | -0.221 | 0.161 | 0.758 |
| Alloscardovia omnicolens | 0 | Not detected | -0.092 | -0.307 | 0.123 | 0.400 |
| Amphibacillus xylanus | 0 | Not detected | 0.201 | -0.003 | 0.405 | 0.054 |
| Anaerobium acetethylicum | 0 | Not detected | -0.027 | -0.121 | 0.068 | 0.577 |
| Anaerococcus hydrogenalis | 0 | Not detected | 0.041 | -0.102 | 0.184 | 0.575 |
| Anaerococcus lactolyticus | 0 | Not detected | 0.074 | -0.135 | 0.283 | 0.490 |
| Anaerococcus obesiensis | 0 | Not detected | 0.098 | -0.086 | 0.283 | 0.297 |
| Anaerocolumna aminovalerica | 0 | Not detected | -0.033 | -0.209 | 0.144 | 0.715 |
| Anaerocolumna xylanovorans | 0 | Not detected | -0.234 | -0.392 | -0.076 | 0.004 |

| Anaerofustis stercorihominis | 0 | Not detected | -0.006 | -0.268 | 0.257 | 0.967 |
| --- | --- | --- | --- | --- | --- | --- |
| Anaeroglobus geminatus | 0 | Not detected | -0.135 | -0.347 | 0.077 | 0.213 |
| Anaeromassilibacillus senegalensis | 0 | Not detected | 0.065 | -0.092 | 0.222 | 0.418 |
| Anaerophaga thermohalophila | 0 | Not detected | -0.004 | -0.129 | 0.120 | 0.945 |
| Anaerosalibacter massiliensis | 0 | Not detected | 0.151 | -0.066 | 0.369 | 0.173 |
| Anaerosporobacter mobilis | 0 | Not detected | -0.036 | -0.144 | 0.071 | 0.509 |
| Anaerosporomusa subterranea | 0 | Not detected | -0.091 | -0.233 | 0.052 | 0.214 |
| Anaerostipes caccae | 0 | Not detected | -0.032 | -0.136 | 0.072 | 0.547 |
| Anaerostipes hadrus | 0 | Not detected | -0.154 | -0.325 | 0.017 | 0.078 |
| Anaerostipes sp. CAG:276 | 0 | Not detected | 0.020 | -0.301 | 0.342 | 0.901 |
| Anaerotruncus colihominis | 0 | Not detected | 0.049 | -0.061 | 0.160 | 0.383 |
| Anaerotruncus sp. CAG:390 | 0 | Not detected | 0.090 | -0.216 | 0.396 | 0.563 |
| Anaerotruncus sp. CAG:528 | 0 | Not detected | 0.081 | -0.160 | 0.322 | 0.512 |
| Anaerotruncus sp. G3(2012) | 0 | Not detected | 0.039 | -0.047 | 0.124 | 0.374 |
| Anaerovorax odorimutans | 0 | Not detected | -0.063 | -0.217 | 0.091 | 0.424 |
| Angelakisella massiliensis | 0 | Not detected | 0.058 | -0.034 | 0.150 | 0.214 |
| Aphanizomenon flos-aquae | 0 | Not detected | -0.147 | -0.293 | -0.001 | 0.048 |
| Aquabacterium parvum | 0 | Not detected | 0.194 | 0.010 | 0.378 | 0.039 |
| Arcanobacterium haemolyticum | 0 | Not detected | -0.095 | -0.270 | 0.081 | 0.291 |
| Asaccharospora irregularis | 0 | Not detected | -0.013 | -0.223 | 0.198 | 0.907 |
| Atlantibacter hermannii | 0 | Not detected | 0.209 | -0.066 | 0.485 | 0.137 |
| Atopobium deltae | 0 | Not detected | 0.094 | -0.104 | 0.293 | 0.351 |
| Atopobium minutum | 0 | Not detected | 0.055 | -0.146 | 0.255 | 0.593 |
| Atopobium parvulum | 0 | Not detected | 0.093 | -0.128 | 0.315 | 0.409 |
| Atopobium rimae | 0 | Not detected | 0.118 | -0.061 | 0.297 | 0.196 |
| Atopobium sp. BS2 | 0 | Not detected | 0.089 | -0.130 | 0.309 | 0.425 |
| Atopobium sp. HMSC064B08 | 0 | Not detected | 0.113 | -0.040 | 0.265 | 0.149 |
| Atopobium sp. ICM42b | 0 | Not detected | 0.103 | -0.119 | 0.326 | 0.364 |
| Atopobium vaginae | 0 | Not detected | -0.017 | -0.220 | 0.186 | 0.868 |
| Atopostipes suicloacalis | 0 | Not detected | 0.115 | -0.079 | 0.309 | 0.245 |
| Avibacterium paragallinarum | 0 | Not detected | -0.023 | -0.198 | 0.153 | 0.801 |
| Azospirillum sp. 47_25 | 0 | Not detected | 0.037 | -0.371 | 0.446 | 0.858 |
| Azospirillum sp. CAG:260 | 0 | Not detected | 0.053 | -0.348 | 0.454 | 0.796 |
| Bacillus acidiproducens | 0 | Not detected | 0.099 | -0.119 | 0.317 | 0.374 |
| Bacillus andreraoultii | 0 | Not detected | 0.175 | -0.042 | 0.392 | 0.115 |
| Bacillus azotoformans | 0 | Not detected | -0.179 | -0.377 | 0.020 | 0.078 |
| Bacillus bataviensis | 0 | Not detected | -0.281 | -0.470 | -0.092 | 0.004 |
| Bacillus coahuilensis | 0 | Not detected | 0.079 | -0.147 | 0.306 | 0.492 |
| Bacillus rubiinfantis | 0 | Not detected | 0.009 | -0.185 | 0.202 | 0.931 |
| Bacillus sp. CAG:988 | 0 | Not detected | -0.035 | -0.219 | 0.148 | 0.708 |
| Bacillus thermotolerans | 0 | Not detected | 0.019 | -0.193 | 0.231 | 0.863 |
| Bacteroidales bacterium 45-6 | 0 | Not detected | -0.137 | -0.344 | 0.070 | 0.194 |
| Bacteroidales bacterium 55_9 | 0 | Not detected | 0.092 | -0.029 | 0.212 | 0.138 |
| Bacteroidales bacterium Bact_08 | 0 | Not detected | -0.047 | -0.209 | 0.115 | 0.571 |

| Bacteroidales bacterium Bact_18 | 0 | Not detected | -0.003 | -0.163 | 0.157 | 0.974 |
| --- | --- | --- | --- | --- | --- | --- |
| Bacteroidales bacterium Bact_23 | 0 | Not detected | 0.001 | -0.207 | 0.209 | 0.993 |
| Bacteroidales bacterium CF | 0 | Not detected | 0.098 | -0.074 | 0.271 | 0.265 |
| Bacteroidales bacterium H10 | 0 | Not detected | 0.129 | -0.043 | 0.300 | 0.142 |
| Bacteroidales bacterium K1 | 0 | Not detected | -0.144 | -0.335 | 0.047 | 0.139 |
| Bacteroidales bacterium K10 | 0 | Not detected | 0.100 | -0.101 | 0.301 | 0.330 |
| Bacteroidales bacterium KHT7 | 0 | Not detected | -0.100 | -0.314 | 0.114 | 0.358 |
| Bacteroides barnesiae | 0 | Not detected | 0.028 | -0.097 | 0.153 | 0.661 |
| Bacteroides caccae CAG:21 | 0 | Not detected | 0.043 | -0.207 | 0.292 | 0.737 |
| Bacteroides cellulosilyticus | 0 | Not detected | 0.072 | -0.141 | 0.285 | 0.509 |
| Bacteroides coprocola | 0 | Not detected | 0.129 | -0.040 | 0.299 | 0.134 |
| Bacteroides coprophilus | 0 | Not detected | 0.060 | -0.061 | 0.182 | 0.332 |
| Bacteroides coprosuis | 0 | Not detected | 0.106 | -0.069 | 0.281 | 0.237 |
| Bacteroides dorei CAG:222 | 0 | Not detected | 0.076 | -0.119 | 0.272 | 0.444 |
| Bacteroides faecichinchillae | 0 | Not detected | -0.012 | -0.141 | 0.117 | 0.858 |
| Bacteroides faecis CAG:32 | 0 | Not detected | 0.148 | -0.087 | 0.383 | 0.217 |
| Bacteroides finegoldii | 0 | Not detected | 0.101 | -0.122 | 0.324 | 0.374 |
| Bacteroides finegoldii CAG:203 | 0 | Not detected | 0.066 | -0.150 | 0.281 | 0.550 |
| Bacteroides fluxus | 0 | Not detected | 0.079 | -0.053 | 0.210 | 0.241 |
| Bacteroides fragilis | 0 | Not detected | -0.132 | -0.305 | 0.041 | 0.135 |
| Bacteroides fragilis CAG:47 | 0 | Not detected | -0.131 | -0.402 | 0.140 | 0.344 |
| Bacteroides graminisolvens | 0 | Not detected | -0.088 | -0.330 | 0.153 | 0.474 |
| Bacteroides neonati | 0 | Not detected | 0.010 | -0.089 | 0.108 | 0.847 |
| Bacteroides oleiciplenus | 0 | Not detected | 0.014 | -0.136 | 0.164 | 0.855 |
| Bacteroides ovatus | 0 | Not detected | -0.119 | -0.284 | 0.047 | 0.160 |
| Bacteroides ovatus CAG:22 | 0 | Not detected | -0.133 | -0.313 | 0.047 | 0.149 |
| Bacteroides paurosaccharolyticus | 0 | Not detected | -0.030 | -0.198 | 0.138 | 0.728 |
| Bacteroides pectinophilus CAG:437 | 0 | Not detected | -0.090 | -0.237 | 0.056 | 0.227 |
| Bacteroides plebeius | 0 | Not detected | 0.024 | -0.152 | 0.201 | 0.789 |
| Bacteroides propionicifaciens | 0 | Not detected | 0.089 | -0.102 | 0.280 | 0.362 |
| Bacteroides pyogenes | 0 | Not detected | -0.033 | -0.175 | 0.108 | 0.644 |
| Bacteroides reticulotermitis | 0 | Not detected | -0.080 | -0.231 | 0.071 | 0.299 |
| Bacteroides salanitronis | 0 | Not detected | 0.020 | -0.106 | 0.146 | 0.758 |
| Bacteroides salyersiae | 0 | Not detected | 0.046 | -0.123 | 0.215 | 0.596 |
| Bacteroides sp. 41_26 | 0 | Not detected | -0.089 | -0.282 | 0.104 | 0.365 |
| Bacteroides sp. 43_46 | 0 | Not detected | 0.004 | -0.166 | 0.174 | 0.960 |
| Bacteroides sp. CAG:1060 | 0 | Not detected | 0.019 | -0.255 | 0.292 | 0.894 |
| Bacteroides sp. CAG:1076 | 0 | Not detected | 0.146 | -0.054 | 0.347 | 0.154 |
| Bacteroides sp. CAG:530 | 0 | Not detected | -0.009 | -0.197 | 0.178 | 0.922 |
| Bacteroides sp. CAG:545 | 0 | Not detected | 0.021 | -0.352 | 0.393 | 0.913 |
| Bacteroides sp. CAG:714 | 0 | Not detected | 0.081 | -0.192 | 0.354 | 0.559 |
| Bacteroides sp. CAG:770 | 0 | Not detected | -0.020 | -0.233 | 0.193 | 0.857 |
| Bacteroides sp. CAG:98 | 0 | Not detected | -0.185 | -0.497 | 0.128 | 0.247 |
| Bacteroides sp. D2 | 0 | Not detected | -0.142 | -0.328 | 0.044 | 0.135 |

| Bacteroides sp. Ga6A1 | 0 | Not detected | -0.194 | -0.437 | 0.049 | 0.119 |
| --- | --- | --- | --- | --- | --- | --- |
| Bacteroides sp. Ga6A2 | 0 | Not detected | -0.204 | -0.443 | 0.035 | 0.095 |
| Bacteroides stercoris | 0 | Not detected | 0.097 | -0.127 | 0.321 | 0.396 |
| Bacteroides thetaiotaomicron | 0 | Not detected | -0.037 | -0.161 | 0.087 | 0.559 |
| Bacteroides thetaiotaomicron CAG:40 | 0 | Not detected | 0.007 | -0.187 | 0.200 | 0.947 |
| Bacteroides uniformis | 0 | Not detected | -0.007 | -0.188 | 0.175 | 0.944 |
| Bacteroides vulgatus | 0 | Not detected | 0.013 | -0.154 | 0.181 | 0.875 |
| Bacteroides vulgatus CAG:6 | 0 | Not detected | 0.036 | -0.161 | 0.234 | 0.719 |
| Bacteroidetes bacterium OLB8 | 0 | Not detected | -0.008 | -0.201 | 0.185 | 0.936 |
| Bacteroidetes oral taxon 274 | 0 | Not detected | 0.103 | -0.031 | 0.238 | 0.131 |
| Bariatricus massiliensis | 0 | Not detected | 0.015 | -0.073 | 0.103 | 0.741 |
| Barnesiella intestinihominis | 0 | Not detected | 0.085 | -0.130 | 0.300 | 0.440 |
| Barnesiella viscericola | 0 | Not detected | 0.032 | -0.074 | 0.138 | 0.554 |
| Bavariicoccus seileri | 0 | Not detected | -0.037 | -0.220 | 0.146 | 0.692 |
| Beduini massiliensis | 0 | Not detected | 0.203 | 0.074 | 0.332 | 0.002 |
| Bibersteinia trehalosi | 0 | Not detected | -0.012 | -0.229 | 0.205 | 0.911 |
| Bifidobacterium actinocoloniiforme | 0 | Not detected | 0.030 | -0.174 | 0.234 | 0.771 |
| Bifidobacterium adolescentis | 0 | Not detected | 0.003 | -0.360 | 0.366 | 0.986 |
| Bifidobacterium aesculapii | 0 | Not detected | 0.010 | -0.183 | 0.202 | 0.922 |
| Bifidobacterium angulatum | 0 | Not detected | -0.086 | -0.383 | 0.212 | 0.574 |
| Bifidobacterium animalis | 0 | Not detected | -0.027 | -0.268 | 0.213 | 0.825 |
| Bifidobacterium asteroides | 0 | Not detected | -0.048 | -0.263 | 0.166 | 0.660 |
| Bifidobacterium biavatii | 0 | Not detected | 0.021 | -0.081 | 0.123 | 0.685 |
| Bifidobacterium bifidum | 0 | Not detected | 0.062 | -0.276 | 0.401 | 0.719 |
| Bifidobacterium bohemicum | 0 | Not detected | 0.055 | -0.140 | 0.250 | 0.582 |
| Bifidobacterium bombi | 0 | Not detected | -0.014 | -0.206 | 0.178 | 0.886 |
| Bifidobacterium breve | 0 | Not detected | -0.066 | -0.395 | 0.262 | 0.693 |
| Bifidobacterium callitrichos | 0 | Not detected | -0.056 | -0.326 | 0.214 | 0.685 |
| Bifidobacterium choerinum | 0 | Not detected | 0.074 | -0.155 | 0.304 | 0.525 |
| Bifidobacterium coryneforme | 0 | Not detected | -0.001 | -0.206 | 0.204 | 0.992 |
| Bifidobacterium crudilactis | 0 | Not detected | -0.014 | -0.179 | 0.152 | 0.872 |
| Bifidobacterium cuniculi | 0 | Not detected | -0.003 | -0.224 | 0.217 | 0.976 |
| Bifidobacterium dentium | 0 | Not detected | 0.010 | -0.300 | 0.320 | 0.950 |
| Bifidobacterium gallicum | 0 | Not detected | 0.016 | -0.206 | 0.238 | 0.887 |
| Bifidobacterium gallinarum | 0 | Not detected | 0.036 | -0.243 | 0.315 | 0.798 |
| Bifidobacterium longum | 0 | Not detected | -0.155 | -0.489 | 0.179 | 0.364 |
| Bifidobacterium magnum | 0 | Not detected | 0.061 | -0.085 | 0.208 | 0.413 |
| Bifidobacterium minimum | 0 | Not detected | -0.003 | -0.286 | 0.280 | 0.985 |
| Bifidobacterium mongoliense | 0 | Not detected | -0.016 | -0.224 | 0.193 | 0.881 |
| Bifidobacterium pseudolongum | 0 | Not detected | -0.078 | -0.313 | 0.157 | 0.515 |
| Bifidobacterium psychraerophilum | 0 | Not detected | 0.016 | -0.138 | 0.169 | 0.841 |
| Bifidobacterium reuteri | 0 | Not detected | 0.015 | -0.228 | 0.258 | 0.904 |
| Bifidobacterium saguini | 0 | Not detected | -0.032 | -0.332 | 0.269 | 0.837 |
| Bifidobacterium scardovii | 0 | Not detected | -0.026 | -0.330 | 0.277 | 0.864 |

| Bifidobacterium stellenboschense | 0 | Not detected | 0.029 | -0.080 | 0.139 | 0.603 |
| --- | --- | --- | --- | --- | --- | --- |
| Bifidobacterium subtile | 0 | Not detected | 0.091 | -0.159 | 0.341 | 0.475 |
| Bifidobacterium thermophilum | 0 | Not detected | 0.018 | -0.132 | 0.168 | 0.813 |
| Bifidobacterium tsurumiense | 0 | Not detected | 0.047 | -0.174 | 0.268 | 0.677 |
| Bilophila wadsworthia | 0 | Not detected | -0.085 | -0.456 | 0.287 | 0.654 |
| Bittarella massiliensis | 0 | Not detected | 0.013 | -0.080 | 0.105 | 0.788 |
| Blautia hansenii | 0 | Not detected | -0.019 | -0.136 | 0.098 | 0.752 |
| Blautia hydrogenotrophica | 0 | Not detected | 0.040 | -0.055 | 0.135 | 0.407 |
| Blautia hydrogenotrophica CAG:147 | 0 | Not detected | -0.152 | -0.439 | 0.134 | 0.297 |
| Blautia obeum | 0 | Not detected | -0.078 | -0.181 | 0.025 | 0.136 |
| Blautia producta | 0 | Not detected | -0.025 | -0.161 | 0.110 | 0.713 |
| Blautia schinkii | 0 | Not detected | 0.005 | -0.071 | 0.081 | 0.902 |
| Blautia sp. CAG:237 | 0 | Not detected | 0.021 | -0.191 | 0.234 | 0.843 |
| Blautia sp. CAG:37 | 0 | Not detected | -0.134 | -0.334 | 0.066 | 0.190 |
| Blautia sp. CAG:37_48_57 | 0 | Not detected | -0.089 | -0.243 | 0.064 | 0.255 |
| Blautia sp. KLE 1732 | 0 | Not detected | 0.047 | -0.079 | 0.173 | 0.462 |
| Brenneria goodwinii | 0 | Not detected | 0.087 | -0.091 | 0.264 | 0.339 |
| Bulleidia extructa | 0 | Not detected | 0.060 | -0.153 | 0.272 | 0.583 |
| Burkholderiales bacterium GJ-E10 | 0 | Not detected | 0.000 | -0.191 | 0.192 | 0.996 |
| Buttiauxella ferragutiae | 0 | Not detected | 0.075 | -0.119 | 0.270 | 0.448 |
| Butyricicoccus desmolans | 0 | Not detected | -0.013 | -0.094 | 0.068 | 0.751 |
| Butyricicoccus pullicaecorum | 0 | Not detected | 0.042 | -0.062 | 0.147 | 0.429 |
| Butyricicoccus sp. K4410.MGS-46 | 0 | Not detected | 0.011 | -0.124 | 0.147 | 0.869 |
| Butyricimonas sp. Marseille-P2440 | 0 | Not detected | -0.040 | -0.247 | 0.166 | 0.702 |
| Butyricimonas synergistica | 0 | Not detected | 0.189 | -0.067 | 0.445 | 0.149 |
| Butyricimonas virosa | 0 | Not detected | 0.109 | -0.104 | 0.322 | 0.316 |
| Butyrivibrio crossotus | 0 | Not detected | -0.059 | -0.229 | 0.111 | 0.498 |
| Butyrivibrio crossotus CAG:259 | 0 | Not detected | -0.122 | -0.378 | 0.135 | 0.353 |
| Butyrivibrio fibrisolvens | 0 | Not detected | 0.048 | -0.126 | 0.222 | 0.589 |
| Butyrivibrio hungatei | 0 | Not detected | -0.009 | -0.155 | 0.138 | 0.909 |
| Butyrivibrio proteoclasticus | 0 | Not detected | -0.093 | -0.204 | 0.017 | 0.098 |
| Butyrivibrio sp. AD3002 | 0 | Not detected | -0.096 | -0.241 | 0.049 | 0.193 |
| Butyrivibrio sp. AE2005 | 0 | Not detected | -0.016 | -0.160 | 0.127 | 0.825 |
| Butyrivibrio sp. AE3003 | 0 | Not detected | -0.068 | -0.176 | 0.040 | 0.215 |
| Butyrivibrio sp. AE3004 | 0 | Not detected | -0.080 | -0.184 | 0.025 | 0.136 |
| Butyrivibrio sp. AE3009 | 0 | Not detected | -0.145 | -0.298 | 0.009 | 0.066 |
| Butyrivibrio sp. FCS006 | 0 | Not detected | -0.016 | -0.174 | 0.142 | 0.845 |
| Butyrivibrio sp. INlla14 | 0 | Not detected | -0.153 | -0.277 | -0.030 | 0.015 |
| Butyrivibrio sp. INlla16 | 0 | Not detected | -0.098 | -0.236 | 0.040 | 0.164 |
| Butyrivibrio sp. INlla21 | 0 | Not detected | -0.108 | -0.251 | 0.035 | 0.138 |
| Butyrivibrio sp. LB2008 | 0 | Not detected | -0.076 | -0.231 | 0.078 | 0.334 |
| Butyrivibrio sp. M55 | 0 | Not detected | -0.123 | -0.268 | 0.023 | 0.098 |
| Butyrivibrio sp. NC2002 | 0 | Not detected | -0.137 | -0.296 | 0.022 | 0.091 |
| Butyrivibrio sp. NC2007 | 0 | Not detected | -0.068 | -0.209 | 0.072 | 0.339 |

| Butyrivibrio sp. NC3005 | 0 | Not detected | -0.033 | -0.243 | 0.177 | 0.759 |
| --- | --- | --- | --- | --- | --- | --- |
| Butyrivibrio sp. VCD2006 | 0 | Not detected | -0.113 | -0.261 | 0.035 | 0.135 |
| Butyrivibrio sp. WCD2001 | 0 | Not detected | -0.047 | -0.195 | 0.102 | 0.541 |
| Butyrivibrio sp. WCD3002 | 0 | Not detected | -0.108 | -0.243 | 0.028 | 0.120 |
| Butyrivibrio sp. ob235 | 0 | Not detected | 0.071 | -0.096 | 0.238 | 0.405 |
| Campylobacter coli | 0 | Not detected | -0.031 | -0.157 | 0.096 | 0.637 |
| Campylobacter hominis | 0 | Not detected | -0.169 | -0.404 | 0.066 | 0.159 |
| Candidatus Azobacteroides pseudotrichony | 0 | Not detected | -0.136 | -0.280 | 0.008 | 0.064 |
| Candidatus Gastranaerophilales bacterium | 0 | Not detected | 0.029 | -0.203 | 0.261 | 0.806 |
| Candidatus Melainabacteria bacterium LEY | 0 | Not detected | -0.147 | -0.303 | 0.009 | 0.066 |
| Candidatus Melainabacteria bacterium ME | 0 | Not detected | -0.151 | -0.439 | 0.138 | 0.306 |
| Candidatus Saccharibacteria oral taxon TM | 0 | Not detected | -0.080 | -0.244 | 0.085 | 0.343 |
| Candidatus Soleaferrea massiliensis | 0 | Not detected | 0.096 | -0.039 | 0.232 | 0.165 |
| Candidatus Stoquefichus sp. SB1 | 0 | Not detected | 0.057 | -0.069 | 0.182 | 0.375 |
| Capnocytophaga granulosa | 0 | Not detected | 0.090 | -0.088 | 0.268 | 0.322 |
| Capnocytophaga sp. oral taxon 329 | 0 | Not detected | 0.096 | -0.163 | 0.356 | 0.467 |
| Capnocytophaga sp. oral taxon 863 | 0 | Not detected | -0.139 | -0.362 | 0.084 | 0.223 |
| Capnocytophaga sputigena | 0 | Not detected | -0.030 | -0.168 | 0.108 | 0.667 |
| Carnobacterium gallinarum | 0 | Not detected | 0.320 | 0.107 | 0.532 | 0.003 |
| Catabacter hongkongensis | 0 | Not detected | -0.013 | -0.196 | 0.171 | 0.892 |
| Catenibacterium mitsuokai | 0 | Not detected | -0.005 | -0.340 | 0.331 | 0.979 |
| Catenibacterium sp. CAG:290 | 0 | Not detected | 0.026 | -0.378 | 0.431 | 0.898 |
| Catonella morbi | 0 | Not detected | -0.008 | -0.123 | 0.107 | 0.893 |
| Cedecea neteri | 0 | Not detected | 0.046 | -0.199 | 0.291 | 0.712 |
| Cellulomonas carbonis | 0 | Not detected | -0.079 | -0.314 | 0.155 | 0.509 |
| Cellulomonas flavigena | 0 | Not detected | 0.125 | -0.108 | 0.359 | 0.293 |
| Cellulosilyticum lentocellum | 0 | Not detected | -0.099 | -0.256 | 0.059 | 0.219 |
| Cellulosilyticum ruminicola | 0 | Not detected | -0.030 | -0.195 | 0.135 | 0.718 |
| Chania multitudinisentens | 0 | Not detected | 0.079 | -0.092 | 0.249 | 0.366 |
| Chlamydia psittaci | 0 | Not detected | 0.073 | -0.116 | 0.262 | 0.448 |
| Christensenella sp. AF73-05CM02 | 0 | Not detected | 0.006 | -0.211 | 0.223 | 0.959 |
| Christensenellaceae bacterium Phil1 | 0 | Not detected | 0.075 | -0.103 | 0.253 | 0.408 |
| Christensenellaceae bacterium Phil10 | 0 | Not detected | -0.044 | -0.299 | 0.211 | 0.735 |
| Christensenellaceae bacterium Phil7 | 0 | Not detected | 0.209 | -0.045 | 0.463 | 0.108 |
| Chryseobacterium gallinarum | 0 | Not detected | -0.061 | -0.337 | 0.214 | 0.663 |
| Chryseobacterium greenlandense | 0 | Not detected | -0.171 | -0.431 | 0.088 | 0.196 |
| Citrobacter amalonaticus | 0 | Not detected | 0.281 | -0.032 | 0.594 | 0.079 |
| Citrobacter freundii | 0 | Not detected | 0.264 | -0.062 | 0.591 | 0.112 |
| Cloacibacillus evryensis | 0 | Not detected | 0.117 | -0.183 | 0.416 | 0.446 |
| Cloacibacillus porcorum | 0 | Not detected | 0.286 | 0.026 | 0.546 | 0.031 |
| Cloacibacterium normanense | 0 | Not detected | -0.153 | -0.400 | 0.094 | 0.226 |
| Clostridia bacterium UC5.1-1C12 | 0 | Not detected | -0.213 | -0.494 | 0.068 | 0.138 |
| Clostridia bacterium UC5.1-1D1 | 0 | Not detected | 0.114 | 0.015 | 0.213 | 0.025 |
| Clostridia bacterium UC5.1-1E11 | 0 | Not detected | 0.088 | -0.010 | 0.186 | 0.080 |

| Clostridiales bacterium 1_7_47FAA | 0 | Not detected | -0.107 | -0.213 | -0.001 | 0.048 |
| --- | --- | --- | --- | --- | --- | --- |
| Clostridiales bacterium 59_14 | 0 | Not detected | 0.125 | -0.044 | 0.294 | 0.148 |
| Clostridiales bacterium DTU035 | 0 | Not detected | 0.085 | -0.080 | 0.250 | 0.314 |
| Clostridiales bacterium DTU036 | 0 | Not detected | 0.070 | -0.090 | 0.229 | 0.393 |
| Clostridiales bacterium DTU089 | 0 | Not detected | 0.119 | -0.044 | 0.283 | 0.153 |
| Clostridiales bacterium Firm_03 | 0 | Not detected | 0.033 | -0.185 | 0.251 | 0.765 |
| Clostridiales bacterium Firm_16 | 0 | Not detected | 0.046 | -0.181 | 0.273 | 0.690 |
| Clostridiales bacterium NK3B98 | 0 | Not detected | 0.080 | -0.111 | 0.271 | 0.413 |
| Clostridiales bacterium S5-A14a | 0 | Not detected | -0.007 | -0.102 | 0.089 | 0.892 |
| Clostridiales bacterium SIT11 | 0 | Not detected | 0.136 | -0.052 | 0.324 | 0.158 |
| Clostridiales bacterium VE202-08 | 0 | Not detected | -0.045 | -0.155 | 0.065 | 0.424 |
| Clostridioides difficile | 0 | Not detected | -0.008 | -0.102 | 0.086 | 0.866 |
| Clostridioides mangenotii | 0 | Not detected | -0.142 | -0.317 | 0.034 | 0.114 |
| Clostridium baratii | 0 | Not detected | -0.113 | -0.330 | 0.104 | 0.307 |
| Clostridium beijerinckii | 0 | Not detected | 0.022 | -0.169 | 0.212 | 0.824 |
| Clostridium bolteae CAG:59 | 0 | Not detected | -0.057 | -0.238 | 0.123 | 0.536 |
| Clostridium botulinum | 0 | Not detected | -0.114 | -0.322 | 0.093 | 0.281 |
| Clostridium butyricum | 0 | Not detected | -0.069 | -0.281 | 0.144 | 0.526 |
| Clostridium cadaveris | 0 | Not detected | -0.092 | -0.274 | 0.089 | 0.318 |
| Clostridium celatum | 0 | Not detected | -0.062 | -0.379 | 0.256 | 0.703 |
| Clostridium cellulovorans | 0 | Not detected | 0.008 | -0.117 | 0.133 | 0.902 |
| Clostridium clostridioforme CAG:132 | 0 | Not detected | -0.070 | -0.315 | 0.174 | 0.574 |
| Clostridium clostridioforme CAG:511 | 0 | Not detected | -0.076 | -0.297 | 0.145 | 0.500 |
| Clostridium colicanis | 0 | Not detected | 0.046 | -0.112 | 0.205 | 0.568 |
| Clostridium disporicum | 0 | Not detected | 0.099 | -0.064 | 0.263 | 0.233 |
| Clostridium ihumii | 0 | Not detected | -0.010 | -0.189 | 0.169 | 0.914 |
| Clostridium intestinale | 0 | Not detected | 0.022 | -0.122 | 0.166 | 0.765 |
| Clostridium kluyveri | 0 | Not detected | 0.123 | -0.086 | 0.331 | 0.249 |
| Clostridium leptum CAG:27 | 0 | Not detected | 0.048 | -0.225 | 0.321 | 0.731 |
| Clostridium lundense | 0 | Not detected | 0.115 | -0.097 | 0.327 | 0.289 |
| Clostridium neonatale | 0 | Not detected | 0.032 | -0.153 | 0.217 | 0.735 |
| Clostridium nexile CAG:348 | 0 | Not detected | -0.158 | -0.338 | 0.021 | 0.084 |
| Clostridium paraputrificum | 0 | Not detected | -0.230 | -0.498 | 0.038 | 0.092 |
| Clostridium perfringens | 0 | Not detected | -0.029 | -0.360 | 0.302 | 0.866 |
| Clostridium phoceensis | 0 | Not detected | 0.021 | -0.125 | 0.166 | 0.781 |
| Clostridium polynesiense | 0 | Not detected | 0.058 | -0.151 | 0.266 | 0.586 |
| Clostridium saccharobutylicum | 0 | Not detected | 0.006 | -0.170 | 0.181 | 0.949 |
| Clostridium saccharoperbutylacetonicum | 0 | Not detected | -0.002 | -0.172 | 0.168 | 0.979 |
| Clostridium sartagoforme | 0 | Not detected | -0.097 | -0.314 | 0.120 | 0.379 |
| Clostridium sp. 44_14 | 0 | Not detected | 0.003 | -0.205 | 0.211 | 0.976 |
| Clostridium sp. ASF356 | 0 | Not detected | 0.093 | -0.119 | 0.304 | 0.390 |
| Clostridium sp. Ade.TY | 0 | Not detected | -0.045 | -0.227 | 0.136 | 0.626 |
| Clostridium sp. CAG:1193 | 0 | Not detected | -0.144 | -0.366 | 0.079 | 0.206 |
| Clostridium sp. CAG:122 | 0 | Not detected | 0.038 | -0.223 | 0.298 | 0.778 |

| Clostridium sp. CAG:12237_41 | 0 | Not detected | 0.036 | -0.172 | 0.244 | 0.737 |
| --- | --- | --- | --- | --- | --- | --- |
| Clostridium sp. CAG:127 | 0 | Not detected | 0.128 | -0.182 | 0.438 | 0.420 |
| Clostridium sp. CAG:138 | 0 | Not detected | -0.111 | -0.523 | 0.302 | 0.599 |
| Clostridium sp. CAG:167 | 0 | Not detected | 0.208 | -0.134 | 0.549 | 0.234 |
| Clostridium sp. CAG:226 | 0 | Not detected | 0.050 | -0.268 | 0.368 | 0.759 |
| Clostridium sp. CAG:230 | 0 | Not detected | -0.043 | -0.255 | 0.169 | 0.692 |
| Clostridium sp. CAG:242 | 0 | Not detected | 0.012 | -0.279 | 0.304 | 0.934 |
| Clostridium sp. CAG:245 | 0 | Not detected | -0.267 | -0.710 | 0.176 | 0.239 |
| Clostridium sp. CAG:245_30_32 | 0 | Not detected | -0.130 | -0.324 | 0.064 | 0.189 |
| Clostridium sp. CAG:264 | 0 | Not detected | -0.182 | -0.485 | 0.120 | 0.237 |
| Clostridium sp. CAG:277 | 0 | Not detected | -0.010 | -0.220 | 0.200 | 0.927 |
| Clostridium sp. CAG:288 | 0 | Not detected | -0.025 | -0.312 | 0.262 | 0.863 |
| Clostridium sp. CAG:299 | 0 | Not detected | -0.123 | -0.409 | 0.163 | 0.400 |
| Clostridium sp. CAG:307_30_263 | 0 | Not detected | -0.048 | -0.232 | 0.136 | 0.609 |
| Clostridium sp. CAG:349 | 0 | Not detected | 0.054 | -0.346 | 0.455 | 0.791 |
| Clostridium sp. CAG:352 | 0 | Not detected | -0.200 | -0.483 | 0.082 | 0.165 |
| Clostridium sp. CAG:413 | 0 | Not detected | 0.313 | -0.005 | 0.631 | 0.054 |
| Clostridium sp. CAG:448 | 0 | Not detected | 0.018 | -0.296 | 0.331 | 0.911 |
| Clostridium sp. CAG:451 | 0 | Not detected | 0.021 | -0.301 | 0.344 | 0.896 |
| Clostridium sp. CAG:465 | 0 | Not detected | -0.046 | -0.349 | 0.257 | 0.766 |
| Clostridium sp. CAG:505 | 0 | Not detected | -0.264 | -0.588 | 0.060 | 0.111 |
| Clostridium sp. CAG:568 | 0 | Not detected | 0.133 | -0.162 | 0.427 | 0.377 |
| Clostridium sp. CAG:58 | 0 | Not detected | 0.113 | -0.120 | 0.347 | 0.341 |
| Clostridium sp. CAG:590 | 0 | Not detected | 0.042 | -0.112 | 0.196 | 0.597 |
| Clostridium sp. CAG:594 | 0 | Not detected | -0.120 | -0.446 | 0.207 | 0.473 |
| Clostridium sp. CAG:628 | 0 | Not detected | -0.257 | -0.573 | 0.060 | 0.112 |
| Clostridium sp. CAG:62_40_43 | 0 | Not detected | 0.016 | -0.356 | 0.388 | 0.933 |
| Clostridium sp. CAG:632 | 0 | Not detected | -0.066 | -0.279 | 0.147 | 0.544 |
| Clostridium sp. CAG:678 | 0 | Not detected | 0.099 | -0.150 | 0.348 | 0.436 |
| Clostridium sp. CAG:762 | 0 | Not detected | 0.063 | -0.196 | 0.321 | 0.635 |
| Clostridium sp. CAG:768 | 0 | Not detected | -0.034 | -0.205 | 0.137 | 0.698 |
| Clostridium sp. CAG:780 | 0 | Not detected | -0.109 | -0.457 | 0.239 | 0.541 |
| Clostridium sp. CAG:793 | 0 | Not detected | -0.133 | -0.408 | 0.142 | 0.343 |
| Clostridium sp. CAG:81 | 0 | Not detected | -0.036 | -0.208 | 0.136 | 0.683 |
| Clostridium sp. CAG:91 | 0 | Not detected | -0.098 | -0.378 | 0.183 | 0.495 |
| Clostridium sp. CAG:964 | 0 | Not detected | -0.013 | -0.182 | 0.155 | 0.876 |
| Clostridium sp. CL-6 | 0 | Not detected | -0.061 | -0.209 | 0.087 | 0.421 |
| Clostridium sp. HGF2 | 0 | Not detected | 0.009 | -0.089 | 0.106 | 0.861 |
| Clostridium sp. K4410.MGS-306 | 0 | Not detected | 0.001 | -0.128 | 0.130 | 0.987 |
| Clostridium sp. KLE 1755 | 0 | Not detected | -0.033 | -0.175 | 0.110 | 0.652 |
| Clostridium sp. KNHs205 | 0 | Not detected | -0.036 | -0.119 | 0.048 | 0.400 |
| Clostridium sp. KNHs209 | 0 | Not detected | -0.062 | -0.141 | 0.018 | 0.129 |
| Clostridium sp. KNHs214 | 0 | Not detected | 0.064 | -0.084 | 0.211 | 0.399 |
| Clostridium sp. L2-50 | 0 | Not detected | -0.134 | -0.255 | -0.013 | 0.031 |

| Clostridium sp. M62/1 | 0 | Not detected | 0.069 | -0.015 | 0.153 | 0.110 |
| --- | --- | --- | --- | --- | --- | --- |
| Clostridium sp. ND2 | 0 | Not detected | -0.031 | -0.205 | 0.142 | 0.723 |
| Clostridium sp. SY8519 | 0 | Not detected | -0.040 | -0.130 | 0.051 | 0.389 |
| Clostridium sulfidigenes | 0 | Not detected | -0.062 | -0.207 | 0.083 | 0.402 |
| Collinsella aerofaciens | 0 | Not detected | 0.041 | -0.209 | 0.291 | 0.746 |
| Collinsella intestinalis | 0 | Not detected | 0.061 | -0.201 | 0.324 | 0.648 |
| Collinsella sp. CAG:166 | 0 | Not detected | 0.037 | -0.235 | 0.308 | 0.792 |
| Collinsella sp. MS5 | 0 | Not detected | 0.048 | -0.139 | 0.234 | 0.617 |
| Collinsella stercoris | 0 | Not detected | 0.102 | -0.136 | 0.341 | 0.401 |
| Collinsella tanakaei | 0 | Not detected | 0.040 | -0.136 | 0.217 | 0.654 |
| Comamonas kerstersii | 0 | Not detected | -0.094 | -0.351 | 0.163 | 0.475 |
| Coprobacillus sp. CAG:605 | 0 | Not detected | -0.171 | -0.467 | 0.124 | 0.256 |
| Coprobacillus sp. D6 | 0 | Not detected | -0.028 | -0.150 | 0.095 | 0.657 |
| Coprobacter secundus | 0 | Not detected | 0.032 | -0.082 | 0.146 | 0.582 |
| Coprococcus comes | 0 | Not detected | -0.045 | -0.147 | 0.057 | 0.391 |
| Coprococcus eutactus | 0 | Not detected | -0.050 | -0.186 | 0.085 | 0.465 |
| Coprococcus eutactus CAG:665 | 0 | Not detected | -0.184 | -0.478 | 0.111 | 0.221 |
| Coprococcus sp. 43_8 | 0 | Not detected | -0.042 | -0.239 | 0.156 | 0.680 |
| Coprococcus sp. CAG:131 | 0 | Not detected | -0.077 | -0.365 | 0.211 | 0.601 |
| Coprococcus sp. CAG:131_42_139 | 0 | Not detected | -0.007 | -0.296 | 0.281 | 0.960 |
| Coprococcus sp. HPP0048 | 0 | Not detected | -0.130 | -0.291 | 0.032 | 0.116 |
| Coriobacteriaceae bacterium 68-1-3 | 0 | Not detected | 0.034 | -0.145 | 0.213 | 0.713 |
| Coriobacteriaceae bacterium BV3Ac1 | 0 | Not detected | 0.057 | -0.165 | 0.279 | 0.614 |
| Coriobacteriales bacterium DNF00809 | 0 | Not detected | 0.155 | -0.035 | 0.346 | 0.111 |
| Coriobacterium glomerans | 0 | Not detected | 0.015 | -0.164 | 0.193 | 0.871 |
| Corynebacterium amycolatum | 0 | Not detected | -0.053 | -0.196 | 0.091 | 0.473 |
| Corynebacterium durum | 0 | Not detected | -0.038 | -0.249 | 0.173 | 0.725 |
| Corynebacterium glucuronolyticum | 0 | Not detected | 0.161 | -0.009 | 0.330 | 0.063 |
| Corynebacterium singulare | 0 | Not detected | 0.105 | -0.062 | 0.271 | 0.218 |
| Corynebacterium urealyticum | 0 | Not detected | 0.069 | -0.116 | 0.254 | 0.468 |
| Criibacterium bergeronii | 0 | Not detected | 0.055 | -0.082 | 0.191 | 0.432 |
| Cronobacter sakazakii | 0 | Not detected | 0.187 | -0.120 | 0.494 | 0.233 |
| Cryptobacterium sp. CAG:338 | 0 | Not detected | -0.241 | -0.557 | 0.076 | 0.136 |
| Culturomica massiliensis | 0 | Not detected | 0.083 | -0.045 | 0.211 | 0.203 |
| Cutibacterium acnes | 0 | Not detected | 0.183 | -0.019 | 0.385 | 0.076 |
| Defluviitalea phaphyphila | 0 | Not detected | 0.091 | -0.049 | 0.232 | 0.203 |
| Dehalobacter restrictus | 0 | Not detected | 0.022 | -0.121 | 0.164 | 0.768 |
| Dendrosporobacter quercicolus | 0 | Not detected | -0.001 | -0.145 | 0.142 | 0.985 |
| Denitrobacterium detoxificans | 0 | Not detected | 0.128 | -0.028 | 0.285 | 0.108 |
| Desnuesiella massiliensis | 0 | Not detected | -0.035 | -0.184 | 0.114 | 0.648 |
| Desulfovibrio alaskensis | 0 | Not detected | 0.012 | -0.140 | 0.163 | 0.881 |
| Desulfovibrio desulfuricans | 0 | Not detected | -0.081 | -0.351 | 0.188 | 0.555 |
| Desulfovibrio fairfieldensis | 0 | Not detected | 0.106 | -0.208 | 0.420 | 0.508 |
| Desulfovibrio piger | 0 | Not detected | -0.018 | -0.416 | 0.379 | 0.928 |

| Desulfovibrio vulgaris | 0 | Not detected | -0.070 | -0.241 | 0.102 | 0.426 |
| --- | --- | --- | --- | --- | --- | --- |
| Devosia insulae | 0 | Not detected | -0.038 | -0.159 | 0.083 | 0.540 |
| Dialister invisus | 0 | Not detected | 0.084 | -0.364 | 0.532 | 0.714 |
| Dialister invisus CAG:218 | 0 | Not detected | 0.002 | -0.454 | 0.458 | 0.995 |
| Dialister micraerophilus | 0 | Not detected | 0.093 | -0.160 | 0.347 | 0.470 |
| Dialister sp. CAG:357 | 0 | Not detected | 0.221 | -0.202 | 0.643 | 0.306 |
| Dialister sp. CAG:486 | 0 | Not detected | 0.041 | -0.359 | 0.442 | 0.840 |
| Dialister sp. CAG:588 | 0 | Not detected | 0.052 | -0.168 | 0.273 | 0.641 |
| Dialister succinatiphilus | 0 | Not detected | 0.022 | -0.233 | 0.277 | 0.863 |
| Dickeya dadantii | 0 | Not detected | 0.013 | -0.161 | 0.188 | 0.881 |
| Dielma fastidiosa | 0 | Not detected | -0.084 | -0.309 | 0.141 | 0.466 |
| Dorea formicigenerans | 0 | Not detected | -0.027 | -0.123 | 0.068 | 0.576 |
| Dorea longicatena | 0 | Not detected | -0.062 | -0.194 | 0.070 | 0.356 |
| Dorea sp. 42_8 | 0 | Not detected | -0.076 | -0.237 | 0.086 | 0.358 |
| Dorea sp. 5-2 | 0 | Not detected | -0.025 | -0.096 | 0.045 | 0.482 |
| Dorea sp. CAG:105 | 0 | Not detected | -0.016 | -0.220 | 0.188 | 0.878 |
| Draconibacterium orientale | 0 | Not detected | 0.162 | 0.026 | 0.298 | 0.020 |
| Drancourtella massiliensis | 0 | Not detected | 0.021 | -0.150 | 0.191 | 0.812 |
| Dubosiella newyorkensis | 0 | Not detected | -0.031 | -0.183 | 0.121 | 0.694 |
| Dysgonomonas gadei | 0 | Not detected | 0.038 | -0.090 | 0.166 | 0.561 |
| Dysgonomonas mossii | 0 | Not detected | 0.016 | -0.140 | 0.172 | 0.839 |
| Dysgonomonas sp. BGC7 | 0 | Not detected | -0.111 | -0.307 | 0.086 | 0.270 |
| Dysgonomonas sp. HGC4 | 0 | Not detected | -0.062 | -0.225 | 0.101 | 0.455 |
| Edwardsiella ictaluri | 0 | Not detected | 0.088 | -0.093 | 0.269 | 0.339 |
| Eggerthella lenta | 0 | Not detected | 0.106 | -0.133 | 0.345 | 0.385 |
| Eggerthella sp. 51_9 | 0 | Not detected | 0.022 | -0.230 | 0.275 | 0.864 |
| Eggerthella sp. CAG:209 | 0 | Not detected | -0.187 | -0.561 | 0.186 | 0.326 |
| Eggerthella sp. CAG:368 | 0 | Not detected | -0.159 | -0.415 | 0.097 | 0.224 |
| Eggerthella sp. YY7918 | 0 | Not detected | 0.053 | -0.135 | 0.240 | 0.582 |
| Eggerthellaceae bacterium AT8 | 0 | Not detected | 0.004 | -0.113 | 0.121 | 0.951 |
| Eggerthia catenaformis | 0 | Not detected | 0.139 | -0.085 | 0.363 | 0.226 |
| Eisenbergiella tayi | 0 | Not detected | 0.098 | -0.094 | 0.291 | 0.317 |
| Elizabethkingia anophelis | 0 | Not detected | -0.132 | -0.362 | 0.099 | 0.264 |
| Emergencia timonensis | 0 | Not detected | 0.048 | -0.043 | 0.138 | 0.302 |
| Empedobacter brevis | 0 | Not detected | 0.093 | -0.214 | 0.400 | 0.551 |
| Enhygromyxa salina | 0 | Not detected | 0.107 | -0.089 | 0.303 | 0.284 |
| Enorma massiliensis | 0 | Not detected | -0.005 | -0.133 | 0.124 | 0.942 |
| Enorma timonensis | 0 | Not detected | -0.040 | -0.189 | 0.108 | 0.593 |
| Enterobacter cloacae | 0 | Not detected | 0.224 | -0.103 | 0.550 | 0.179 |
| Enterobacter lignolyticus | 0 | Not detected | 0.258 | -0.033 | 0.549 | 0.082 |
| Enterobacter sp. 638 | 0 | Not detected | 0.169 | -0.098 | 0.437 | 0.215 |
| Enterobacteriaceae bacterium LSJC7 | 0 | Not detected | 0.146 | -0.050 | 0.342 | 0.145 |
| Enterococcus avium | 0 | Not detected | -0.070 | -0.261 | 0.122 | 0.477 |
| Enterococcus cecorum | 0 | Not detected | 0.212 | 0.035 | 0.388 | 0.019 |

| Enterococcus columbae | 0 | Not detected | 0.019 | -0.194 | 0.233 | 0.859 |
| --- | --- | --- | --- | --- | --- | --- |
| Enterococcus durans | 0 | Not detected | 0.135 | -0.113 | 0.383 | 0.286 |
| Enterococcus faecalis | 0 | Not detected | 0.116 | -0.075 | 0.306 | 0.234 |
| Enterococcus faecium | 0 | Not detected | 0.027 | -0.189 | 0.243 | 0.807 |
| Enterococcus gallinarum | 0 | Not detected | 0.238 | 0.041 | 0.436 | 0.018 |
| Enterococcus gilvus | 0 | Not detected | 0.027 | -0.159 | 0.212 | 0.778 |
| Enterococcus italicus | 0 | Not detected | -0.002 | -0.221 | 0.217 | 0.986 |
| Enterococcus massiliensis | 0 | Not detected | 0.178 | -0.031 | 0.388 | 0.096 |
| Enterococcus sp. HMSC064A12 | 0 | Not detected | 0.093 | -0.123 | 0.309 | 0.400 |
| Enterorhabdus caecimuris | 0 | Not detected | 0.220 | 0.006 | 0.435 | 0.044 |
| Enterorhabdus mucosicola | 0 | Not detected | 0.078 | -0.117 | 0.274 | 0.433 |
| Epulopiscium sp. 'N.t. morphotype B' | 0 | Not detected | -0.068 | -0.199 | 0.063 | 0.306 |
| Erwinia billingiae | 0 | Not detected | 0.122 | -0.055 | 0.298 | 0.176 |
| Erwinia oleae | 0 | Not detected | 0.096 | -0.139 | 0.331 | 0.425 |
| Erysipelatoclostridium ramosum | 0 | Not detected | -0.149 | -0.431 | 0.133 | 0.302 |
| Erysipelotrichaceae bacterium 21_3 | 0 | Not detected | -0.036 | -0.158 | 0.086 | 0.563 |
| Erysipelotrichaceae bacterium 5_2_54FAA | 0 | Not detected | 0.055 | -0.046 | 0.156 | 0.286 |
| Erysipelotrichaceae bacterium CAG:64 | 0 | Not detected | 0.001 | -0.207 | 0.210 | 0.990 |
| Erysipelotrichaceae bacterium I46 | 0 | Not detected | -0.010 | -0.126 | 0.107 | 0.873 |
| Escherichia coli | 0 | Not detected | 0.167 | -0.156 | 0.491 | 0.311 |
| Escherichia vulneris | 0 | Not detected | 0.212 | -0.048 | 0.472 | 0.111 |
| Ethanoligenens harbinense | 0 | Not detected | 0.063 | -0.134 | 0.259 | 0.532 |
| Eubacterium dolichum CAG:375 | 0 | Not detected | -0.130 | -0.440 | 0.181 | 0.414 |
| Eubacterium limosum | 0 | Not detected | 0.015 | -0.134 | 0.164 | 0.839 |
| Eubacterium plexicaudatum | 0 | Not detected | 0.036 | -0.047 | 0.120 | 0.395 |
| Eubacterium pyruvativorans | 0 | Not detected | -0.043 | -0.145 | 0.058 | 0.403 |
| Eubacterium ramulus | 0 | Not detected | -0.037 | -0.132 | 0.058 | 0.443 |
| Eubacterium sp. 14-2 | 0 | Not detected | 0.001 | -0.083 | 0.086 | 0.977 |
| Eubacterium sp. 3_1_31 | 0 | Not detected | 0.014 | -0.099 | 0.126 | 0.814 |
| Eubacterium sp. 45_250 | 0 | Not detected | 0.150 | -0.183 | 0.483 | 0.378 |
| Eubacterium sp. AB3007 | 0 | Not detected | -0.261 | -0.526 | 0.005 | 0.055 |
| Eubacterium sp. CAG:115 | 0 | Not detected | 0.063 | -0.382 | 0.507 | 0.782 |
| Eubacterium sp. CAG:146 | 0 | Not detected | -0.081 | -0.292 | 0.130 | 0.450 |
| Eubacterium sp. CAG:180 | 0 | Not detected | -0.004 | -0.427 | 0.419 | 0.986 |
| Eubacterium sp. CAG:192 | 0 | Not detected | -0.073 | -0.370 | 0.225 | 0.632 |
| Eubacterium sp. CAG:202 | 0 | Not detected | -0.093 | -0.398 | 0.211 | 0.549 |
| Eubacterium sp. CAG:248 | 0 | Not detected | 0.143 | -0.122 | 0.408 | 0.291 |
| Eubacterium sp. CAG:251 | 0 | Not detected | -0.019 | -0.379 | 0.342 | 0.919 |
| Eubacterium sp. CAG:252 | 0 | Not detected | 0.189 | -0.043 | 0.420 | 0.111 |
| Eubacterium sp. CAG:581 | 0 | Not detected | -0.117 | -0.420 | 0.186 | 0.449 |
| Eubacterium sp. CAG:603 | 0 | Not detected | -0.072 | -0.233 | 0.089 | 0.378 |
| Eubacterium sp. CAG:786 | 0 | Not detected | 0.062 | -0.274 | 0.398 | 0.717 |
| Eubacterium sp. CAG:841 | 0 | Not detected | 0.121 | -0.207 | 0.449 | 0.469 |
| Eubacterium sp. ER2 | 0 | Not detected | 0.015 | -0.068 | 0.097 | 0.726 |

| Eubacterium ventriosum | 0 | Not detected | -0.009 | -0.130 | 0.113 | 0.888 |
| --- | --- | --- | --- | --- | --- | --- |
| Eubacterium xylanophilum | 0 | Not detected | -0.153 | -0.320 | 0.014 | 0.074 |
| Facklamia hominis | 0 | Not detected | 0.214 | 0.016 | 0.412 | 0.035 |
| Facklamia ignava | 0 | Not detected | 0.077 | -0.118 | 0.272 | 0.438 |
| Facklamia languida | 0 | Not detected | 0.161 | -0.018 | 0.340 | 0.078 |
| Faecalibacterium prausnitzii | 0 | Not detected | 0.099 | -0.054 | 0.252 | 0.205 |
| Faecalibacterium sp. CAG:1138 | 0 | Not detected | 0.113 | -0.163 | 0.389 | 0.424 |
| Faecalibacterium sp. CAG:82-related_59_9 | 0 | Not detected | 0.190 | -0.037 | 0.418 | 0.102 |
| Faecalibaculum rodentium | 0 | Not detected | -0.142 | -0.353 | 0.069 | 0.188 |
| Faecalicoccus pleomorphus | 0 | Not detected | -0.002 | -0.124 | 0.121 | 0.975 |
| Faecalitalea cylindroides | 0 | Not detected | -0.021 | -0.102 | 0.061 | 0.620 |
| Fenollaria massiliensis | 0 | Not detected | 0.202 | -0.031 | 0.435 | 0.090 |
| Fermentimonas caenicola | 0 | Not detected | 0.006 | -0.190 | 0.202 | 0.948 |
| Fibrobacter sp. UWB12 | 0 | Not detected | 0.011 | -0.122 | 0.144 | 0.872 |
| Fibrobacter sp. UWCM | 0 | Not detected | -0.024 | -0.203 | 0.155 | 0.794 |
| Fibrobacter sp. UWOV1 | 0 | Not detected | 0.009 | -0.114 | 0.133 | 0.881 |
| Fibrobacterales bacterium Fibro_02 | 0 | Not detected | -0.011 | -0.140 | 0.118 | 0.867 |
| Fibrobacteria bacterium AD80 | 0 | Not detected | -0.037 | -0.168 | 0.094 | 0.579 |
| Filifactor alocis | 0 | Not detected | 0.080 | -0.129 | 0.289 | 0.454 |
| Finegoldia magna | 0 | Not detected | 0.039 | -0.176 | 0.255 | 0.722 |
| Firmicutes bacterium ASF500 | 0 | Not detected | 0.066 | -0.022 | 0.155 | 0.143 |
| Firmicutes bacterium CAG:102 | 0 | Not detected | 0.194 | -0.117 | 0.505 | 0.222 |
| Firmicutes bacterium CAG:137 | 0 | Not detected | 0.006 | -0.333 | 0.344 | 0.974 |
| Firmicutes bacterium CAG:137_57_8 | 0 | Not detected | 0.079 | -0.181 | 0.338 | 0.554 |
| Firmicutes bacterium CAG:145 | 0 | Not detected | -0.032 | -0.339 | 0.275 | 0.839 |
| Firmicutes bacterium CAG:176 | 0 | Not detected | -0.093 | -0.480 | 0.294 | 0.636 |
| Firmicutes bacterium CAG:176_59_8 | 0 | Not detected | 0.096 | -0.247 | 0.440 | 0.583 |
| Firmicutes bacterium CAG:227 | 0 | Not detected | -0.354 | -0.613 | -0.094 | 0.008 |
| Firmicutes bacterium CAG:238 | 0 | Not detected | -0.129 | -0.423 | 0.165 | 0.390 |
| Firmicutes bacterium CAG:24 | 0 | Not detected | 0.067 | -0.155 | 0.290 | 0.554 |
| Firmicutes bacterium CAG:240 | 0 | Not detected | -0.092 | -0.431 | 0.247 | 0.595 |
| Firmicutes bacterium CAG:270 | 0 | Not detected | -0.250 | -0.502 | 0.002 | 0.052 |
| Firmicutes bacterium CAG:272 | 0 | Not detected | -0.022 | -0.326 | 0.282 | 0.886 |
| Firmicutes bacterium CAG:272_52_7 | 0 | Not detected | -0.059 | -0.292 | 0.174 | 0.619 |
| Firmicutes bacterium CAG:308 | 0 | Not detected | -0.093 | -0.294 | 0.108 | 0.364 |
| Firmicutes bacterium CAG:313 | 0 | Not detected | 0.055 | -0.344 | 0.454 | 0.786 |
| Firmicutes bacterium CAG:41 | 0 | Not detected | 0.101 | -0.174 | 0.376 | 0.473 |
| Firmicutes bacterium CAG:466 | 0 | Not detected | -0.184 | -0.419 | 0.051 | 0.125 |
| Firmicutes bacterium CAG:475 | 0 | Not detected | 0.169 | -0.134 | 0.473 | 0.275 |
| Firmicutes bacterium CAG:536 | 0 | Not detected | 0.324 | 0.078 | 0.569 | 0.010 |
| Firmicutes bacterium CAG:552 | 0 | Not detected | -0.057 | -0.321 | 0.208 | 0.675 |
| Firmicutes bacterium CAG:555 | 0 | Not detected | -0.050 | -0.314 | 0.213 | 0.708 |
| Firmicutes bacterium CAG:65 | 0 | Not detected | -0.032 | -0.287 | 0.224 | 0.809 |
| Firmicutes bacterium CAG:791 | 0 | Not detected | -0.075 | -0.219 | 0.070 | 0.310 |

| Firmicutes bacterium CAG:83 | 0 | Not detected | 0.262 | -0.012 | 0.535 | 0.061 |
| --- | --- | --- | --- | --- | --- | --- |
| Firmicutes bacterium CAG:882 | 0 | Not detected | -0.063 | -0.250 | 0.125 | 0.512 |
| Firmicutes bacterium CAG:884 | 0 | Not detected | -0.133 | -0.392 | 0.126 | 0.314 |
| Firmicutes bacterium CAG:94 | 0 | Not detected | 0.066 | -0.230 | 0.361 | 0.664 |
| Firmicutes bacterium M10-2 | 0 | Not detected | -0.066 | -0.288 | 0.155 | 0.557 |
| Firmicutes bacterium ZOR0006 | 0 | Not detected | 0.206 | -0.024 | 0.436 | 0.079 |
| Flavonifractor plautii | 0 | Not detected | 0.004 | -0.190 | 0.199 | 0.966 |
| Fournierella massiliensis | 0 | Not detected | 0.026 | -0.068 | 0.119 | 0.591 |
| Franconibacter pulveris | 0 | Not detected | 0.144 | -0.099 | 0.387 | 0.246 |
| Fretibacterium fastidiosum | 0 | Not detected | 0.131 | -0.015 | 0.277 | 0.079 |
| Fusicatenibacter saccharivorans | 0 | Not detected | -0.075 | -0.187 | 0.037 | 0.189 |
| Fusobacterium mortiferum | 0 | Not detected | -0.025 | -0.352 | 0.302 | 0.880 |
| Fusobacterium necrophorum | 0 | Not detected | 0.076 | -0.138 | 0.290 | 0.489 |
| Fusobacterium nucleatum | 0 | Not detected | 0.064 | -0.231 | 0.360 | 0.670 |
| Fusobacterium periodonticum | 0 | Not detected | -0.088 | -0.333 | 0.157 | 0.481 |
| Gabonia massiliensis | 0 | Not detected | 0.066 | -0.116 | 0.249 | 0.475 |
| Gabonibacter massiliensis | 0 | Not detected | 0.142 | -0.075 | 0.359 | 0.200 |
| Gallibacterium anatis | 0 | Not detected | -0.140 | -0.295 | 0.015 | 0.077 |
| Gardnerella vaginalis | 0 | Not detected | 0.201 | -0.021 | 0.422 | 0.076 |
| Gemella haemolysans | 0 | Not detected | 0.024 | -0.185 | 0.233 | 0.822 |
| Gemella sanguinis | 0 | Not detected | -0.104 | -0.361 | 0.154 | 0.430 |
| Gemmata obscuriglobus | 0 | Not detected | -0.138 | -0.317 | 0.041 | 0.130 |
| Geofilum rubicundum | 0 | Not detected | -0.042 | -0.209 | 0.124 | 0.621 |
| Gilliamella apicola | 0 | Not detected | 0.113 | -0.063 | 0.288 | 0.209 |
| Globicatella sanguinis | 0 | Not detected | 0.005 | -0.190 | 0.201 | 0.959 |
| Gordonibacter pamelaeae | 0 | Not detected | -0.010 | -0.185 | 0.164 | 0.906 |
| Granulicatella adiacens | 0 | Not detected | -0.110 | -0.357 | 0.136 | 0.381 |
| Granulicatella elegans | 0 | Not detected | -0.006 | -0.208 | 0.196 | 0.953 |
| Haemophilus influenzae | 0 | Not detected | -0.164 | -0.449 | 0.121 | 0.260 |
| Haemophilus parainfluenzae | 0 | Not detected | -0.188 | -0.556 | 0.179 | 0.315 |
| Haemophilus sputorum | 0 | Not detected | -0.064 | -0.369 | 0.242 | 0.683 |
| Hafnia sp. HMSC23F03 | 0 | Not detected | -0.133 | -0.343 | 0.077 | 0.214 |
| Hathewaya proteolytica | 0 | Not detected | 0.007 | -0.191 | 0.206 | 0.945 |
| Heliobacterium sp. N15.MGS-306 | 0 | Not detected | 0.017 | -0.088 | 0.122 | 0.748 |
| Hespellia stercorisuis | 0 | Not detected | -0.057 | -0.152 | 0.038 | 0.237 |
| Holdemanella biformis | 0 | Not detected | -0.110 | -0.283 | 0.064 | 0.216 |
| Holdemania filiformis | 0 | Not detected | -0.030 | -0.136 | 0.075 | 0.574 |
| Holdemania massiliensis | 0 | Not detected | 0.139 | 0.000 | 0.278 | 0.050 |
| Holdemania sp. Marseille-P2844 | 0 | Not detected | -0.061 | -0.188 | 0.065 | 0.344 |
| Hungatella hathewayi | 0 | Not detected | -0.022 | -0.135 | 0.092 | 0.709 |
| Hydrogenoanaerobacterium saccharovorans | 0 | Not detected | 0.133 | -0.007 | 0.273 | 0.063 |
| Idiomarina zobellii | 0 | Not detected | -0.189 | -0.446 | 0.067 | 0.148 |
| Ileibacterium valens | 0 | Not detected | -0.005 | -0.232 | 0.221 | 0.963 |
| Intestinibacter bartlettii | 0 | Not detected | -0.080 | -0.296 | 0.137 | 0.471 |

| Intestinimonas butyriciproducens | 0 | Not detected | 0.073 | -0.063 | 0.209 | 0.295 |
| --- | --- | --- | --- | --- | --- | --- |
| Intestinimonas massiliensis | 0 | Not detected | 0.043 | -0.081 | 0.168 | 0.495 |
| Izhakiella sp. D4N98 | 0 | Not detected | 0.196 | 0.006 | 0.385 | 0.044 |
| Jeotgalibaca sp. PTS2502 | 0 | Not detected | -0.003 | -0.161 | 0.155 | 0.974 |
| Johnsonella ignava | 0 | Not detected | 0.064 | -0.046 | 0.174 | 0.254 |
| Kallipyga massiliensis | 0 | Not detected | 0.101 | -0.100 | 0.302 | 0.327 |
| Kandleria vitulina | 0 | Not detected | 0.120 | -0.194 | 0.435 | 0.454 |
| Kiloniella litopenaei | 0 | Not detected | -0.013 | -0.213 | 0.187 | 0.896 |
| Kingella kingae | 0 | Not detected | -0.087 | -0.278 | 0.104 | 0.372 |
| Klebsiella oxytoca | 0 | Not detected | 0.153 | -0.205 | 0.510 | 0.402 |
| Klebsiella pneumoniae | 0 | Not detected | 0.288 | -0.086 | 0.663 | 0.131 |
| Klebsiella sp. RIT-PI-d | 0 | Not detected | 0.217 | -0.042 | 0.476 | 0.101 |
| Kluyvera ascorbata | 0 | Not detected | 0.310 | -0.017 | 0.637 | 0.063 |
| Kluyvera cryocrescens | 0 | Not detected | 0.280 | -0.054 | 0.614 | 0.101 |
| Kluyvera intermedia | 0 | Not detected | 0.201 | -0.106 | 0.508 | 0.199 |
| Kocuria sp. ZOR0020 | 0 | Not detected | -0.145 | -0.312 | 0.022 | 0.089 |
| Kosakonia sacchari | 0 | Not detected | 0.176 | -0.101 | 0.452 | 0.213 |
| Lachnoanaerobaculum saburreum | 0 | Not detected | 0.056 | -0.057 | 0.169 | 0.330 |
| Lachnoanaerobaculum sp. OBRC5-5 | 0 | Not detected | 0.065 | -0.040 | 0.170 | 0.225 |
| Lachnobacterium bovis | 0 | Not detected | -0.007 | -0.108 | 0.095 | 0.900 |
| Lachnoclostridium phytofermentans | 0 | Not detected | -0.124 | -0.210 | -0.038 | 0.005 |
| Lachnospira multipara | 0 | Not detected | -0.067 | -0.222 | 0.088 | 0.396 |
| Lachnospiraceae bacterium 10-1 | 0 | Not detected | 0.018 | -0.055 | 0.091 | 0.624 |
| Lachnospiraceae bacterium 28-4 | 0 | Not detected | 0.010 | -0.063 | 0.083 | 0.784 |
| Lachnospiraceae bacterium 2_1_46FAA | 0 | Not detected | -0.082 | -0.197 | 0.032 | 0.160 |
| Lachnospiraceae bacterium 3-1 | 0 | Not detected | 0.032 | -0.057 | 0.121 | 0.479 |
| Lachnospiraceae bacterium 3-2 | 0 | Not detected | -0.041 | -0.127 | 0.046 | 0.358 |
| Lachnospiraceae bacterium A2 | 0 | Not detected | -0.026 | -0.115 | 0.064 | 0.574 |
| Lachnospiraceae bacterium A4 | 0 | Not detected | -0.008 | -0.085 | 0.069 | 0.838 |
| Lachnospiraceae bacterium AB2028 | 0 | Not detected | -0.085 | -0.296 | 0.126 | 0.430 |
| Lachnospiraceae bacterium AC2012 | 0 | Not detected | -0.012 | -0.110 | 0.085 | 0.801 |
| Lachnospiraceae bacterium AC2014 | 0 | Not detected | -0.060 | -0.205 | 0.084 | 0.414 |
| Lachnospiraceae bacterium AC2028 | 0 | Not detected | -0.026 | -0.159 | 0.107 | 0.702 |
| Lachnospiraceae bacterium AC2029 | 0 | Not detected | -0.099 | -0.202 | 0.003 | 0.057 |
| Lachnospiraceae bacterium AC2031 | 0 | Not detected | -0.006 | -0.145 | 0.134 | 0.937 |
| Lachnospiraceae bacterium AC3007 | 0 | Not detected | -0.082 | -0.246 | 0.083 | 0.331 |
| Lachnospiraceae bacterium AD3010 | 0 | Not detected | -0.076 | -0.271 | 0.120 | 0.450 |
| Lachnospiraceae bacterium C6A11 | 0 | Not detected | -0.030 | -0.165 | 0.104 | 0.659 |
| Lachnospiraceae bacterium COE1 | 0 | Not detected | 0.057 | -0.053 | 0.168 | 0.307 |
| Lachnospiraceae bacterium FD2005 | 0 | Not detected | 0.108 | -0.054 | 0.270 | 0.193 |
| Lachnospiraceae bacterium FE2018 | 0 | Not detected | -0.100 | -0.303 | 0.103 | 0.335 |
| Lachnospiraceae bacterium G41 | 0 | Not detected | -0.021 | -0.191 | 0.149 | 0.808 |
| Lachnospiraceae bacterium KHCPX20 | 0 | Not detected | -0.099 | -0.307 | 0.109 | 0.352 |
| Lachnospiraceae bacterium M18-1 | 0 | Not detected | -0.035 | -0.119 | 0.049 | 0.418 |

| Lachnospiraceae bacterium MC2017 | 0 | Not detected | -0.012 | -0.153 | 0.128 | 0.862 |
| --- | --- | --- | --- | --- | --- | --- |
| Lachnospiraceae bacterium NC2004 | 0 | Not detected | -0.109 | -0.289 | 0.071 | 0.237 |
| Lachnospiraceae bacterium NC2008 | 0 | Not detected | -0.100 | -0.274 | 0.075 | 0.263 |
| Lachnospiraceae bacterium ND2006 | 0 | Not detected | -0.060 | -0.232 | 0.113 | 0.499 |
| Lachnospiraceae bacterium NK3A20 | 0 | Not detected | -0.167 | -0.373 | 0.038 | 0.111 |
| Lachnospiraceae bacterium NK4A179 | 0 | Not detected | -0.060 | -0.237 | 0.117 | 0.506 |
| Lachnospiraceae bacterium NLAE-zl-G231 | 0 | Not detected | 0.132 | -0.082 | 0.347 | 0.226 |
| Lachnospiraceae bacterium P6B14 | 0 | Not detected | -0.148 | -0.293 | -0.002 | 0.047 |
| Lachnospiraceae bacterium TF01-11 | 0 | Not detected | 0.054 | -0.055 | 0.163 | 0.333 |
| Lachnospiraceae bacterium TWA4 | 0 | Not detected | -0.029 | -0.139 | 0.081 | 0.600 |
| Lachnospiraceae bacterium V9D3004 | 0 | Not detected | 0.022 | -0.142 | 0.186 | 0.790 |
| Lachnospiraceae bacterium YSB2008 | 0 | Not detected | 0.014 | -0.121 | 0.149 | 0.839 |
| Lachnospiraceae bacterium mt14 | 0 | Not detected | -0.077 | -0.235 | 0.082 | 0.342 |
| Lachnospiraceae bacterium oral taxon 500 | 0 | Not detected | 0.020 | -0.068 | 0.107 | 0.660 |
| Lactobacillus acidophilus | 0 | Not detected | 0.158 | 0.001 | 0.314 | 0.048 |
| Lactobacillus amylotrophicus | 0 | Not detected | 0.149 | -0.036 | 0.335 | 0.115 |
| Lactobacillus animalis | 0 | Not detected | 0.010 | -0.165 | 0.186 | 0.907 |
| Lactobacillus antri | 0 | Not detected | 0.083 | -0.136 | 0.301 | 0.458 |
| Lactobacillus aviarius | 0 | Not detected | 0.004 | -0.101 | 0.108 | 0.947 |
| Lactobacillus buchneri | 0 | Not detected | 0.077 | -0.070 | 0.224 | 0.307 |
| Lactobacillus casei | 0 | Not detected | -0.106 | -0.379 | 0.168 | 0.448 |
| Lactobacillus curvatus | 0 | Not detected | -0.024 | -0.229 | 0.181 | 0.821 |
| Lactobacillus delbrueckii | 0 | Not detected | 0.089 | -0.147 | 0.325 | 0.459 |
| Lactobacillus equi | 0 | Not detected | 0.028 | -0.176 | 0.231 | 0.790 |
| Lactobacillus fermentum | 0 | Not detected | 0.164 | -0.151 | 0.478 | 0.308 |
| Lactobacillus gasseri | 0 | Not detected | 0.224 | -0.096 | 0.544 | 0.170 |
| Lactobacillus helveticus | 0 | Not detected | 0.061 | -0.165 | 0.286 | 0.597 |
| Lactobacillus ingluviei | 0 | Not detected | 0.087 | -0.088 | 0.261 | 0.330 |
| Lactobacillus jensenii | 0 | Not detected | 0.104 | -0.085 | 0.292 | 0.282 |
| Lactobacillus kefiranofaciens | 0 | Not detected | 0.058 | -0.121 | 0.237 | 0.528 |
| Lactobacillus manihotivorans | 0 | Not detected | -0.229 | -0.413 | -0.046 | 0.015 |
| Lactobacillus mucosae | 0 | Not detected | 0.189 | -0.032 | 0.409 | 0.094 |
| Lactobacillus paracasei | 0 | Not detected | -0.001 | -0.285 | 0.283 | 0.995 |
| Lactobacillus paraplantarum | 0 | Not detected | 0.065 | -0.125 | 0.255 | 0.503 |
| Lactobacillus pasteurii | 0 | Not detected | 0.102 | -0.063 | 0.266 | 0.226 |
| Lactobacillus plantarum | 0 | Not detected | 0.058 | -0.178 | 0.294 | 0.631 |
| Lactobacillus reuteri | 0 | Not detected | 0.021 | -0.207 | 0.250 | 0.854 |
| Lactobacillus rhamnosus | 0 | Not detected | 0.082 | -0.176 | 0.339 | 0.535 |
| Lactobacillus ruminis | 0 | Not detected | 0.017 | -0.209 | 0.244 | 0.881 |
| Lactobacillus ruminis CAG:367 | 0 | Not detected | 0.096 | -0.134 | 0.326 | 0.414 |
| Lactobacillus sakei | 0 | Not detected | 0.124 | -0.080 | 0.327 | 0.235 |
| Lactobacillus salivarius | 0 | Not detected | 0.163 | -0.146 | 0.473 | 0.301 |
| Lactobacillus vaginalis | 0 | Not detected | 0.137 | -0.132 | 0.406 | 0.319 |
| Lactococcus garvieae | 0 | Not detected | -0.031 | -0.240 | 0.177 | 0.767 |

| Lactococcus lactis | 0 | Not detected | -0.183 | -0.507 | 0.141 | 0.268 |
| --- | --- | --- | --- | --- | --- | --- |
| Lactococcus piscium | 0 | Not detected | -0.178 | -0.389 | 0.034 | 0.100 |
| Lactonifactor longoviformis | 0 | Not detected | 0.007 | -0.067 | 0.080 | 0.861 |
| Lamprocystis purpurea | 0 | Not detected | 0.155 | -0.107 | 0.418 | 0.247 |
| Lascolabacillus massiliensis | 0 | Not detected | -0.010 | -0.172 | 0.152 | 0.903 |
| Lautropia mirabilis | 0 | Not detected | -0.181 | -0.389 | 0.028 | 0.090 |
| Lawsonia intracellularis | 0 | Not detected | 0.093 | -0.110 | 0.296 | 0.369 |
| Leclercia adecarboxylata | 0 | Not detected | 0.173 | -0.136 | 0.482 | 0.273 |
| Lelliottia amnigena | 0 | Not detected | 0.296 | -0.045 | 0.636 | 0.089 |
| Lentimicrobium saccharophilum | 0 | Not detected | -0.014 | -0.226 | 0.199 | 0.900 |
| Leuconostoc citreum | 0 | Not detected | -0.098 | -0.280 | 0.085 | 0.294 |
| Leuconostoc lactis | 0 | Not detected | -0.012 | -0.240 | 0.217 | 0.921 |
| Leuconostoc mesenteroides | 0 | Not detected | -0.077 | -0.315 | 0.161 | 0.526 |
| Levyella massiliensis | 0 | Not detected | 0.063 | -0.052 | 0.178 | 0.284 |
| Libanicoccus massiliensis | 0 | Not detected | 0.227 | 0.021 | 0.432 | 0.031 |
| Luteimonas sp. FCS-9 | 0 | Not detected | -0.011 | -0.158 | 0.137 | 0.889 |
| Lutispora thermophila | 0 | Not detected | -0.150 | -0.329 | 0.028 | 0.100 |
| Macellibacteroides sp. HH-ZS | 0 | Not detected | 0.063 | -0.089 | 0.215 | 0.417 |
| Macrococcus caseolyticus | 0 | Not detected | 0.210 | 0.010 | 0.411 | 0.040 |
| Mageeibacillus indolicus | 0 | Not detected | 0.108 | -0.052 | 0.269 | 0.185 |
| Mangrovibacter sp. MFB070 | 0 | Not detected | 0.187 | -0.054 | 0.428 | 0.129 |
| Mannheimia haemolytica | 0 | Not detected | -0.144 | -0.334 | 0.045 | 0.136 |
| Marvinbryantia formatexigens | 0 | Not detected | 0.023 | -0.081 | 0.128 | 0.664 |
| Massilioclostridium coli | 0 | Not detected | 0.014 | -0.097 | 0.126 | 0.800 |
| Massiliomicrobiota timonensis | 0 | Not detected | 0.024 | -0.185 | 0.232 | 0.825 |
| Mediterranea massiliensis | 0 | Not detected | -0.046 | -0.182 | 0.090 | 0.511 |
| Megamonas funiformis | 0 | Not detected | 0.145 | -0.251 | 0.542 | 0.473 |
| Megasphaera cerevisiae | 0 | Not detected | 0.147 | -0.135 | 0.430 | 0.307 |
| Megasphaera elsdenii | 0 | Not detected | 0.021 | -0.357 | 0.399 | 0.914 |
| Megasphaera genomosp. type_1 | 0 | Not detected | -0.023 | -0.104 | 0.058 | 0.582 |
| Megasphaera massiliensis | 0 | Not detected | 0.006 | -0.346 | 0.357 | 0.974 |
| Megasphaera micronuciformis | 0 | Not detected | -0.006 | -0.254 | 0.243 | 0.964 |
| Megasphaera sp. BV3C16-1 | 0 | Not detected | -0.041 | -0.262 | 0.180 | 0.718 |
| Megasphaera sp. UPII 135-E | 0 | Not detected | -0.069 | -0.156 | 0.018 | 0.121 |
| Merdibacter massiliensis | 0 | Not detected | 0.011 | -0.079 | 0.101 | 0.809 |
| Methanobrevibacter smithii | 0 | Not detected | -0.051 | -0.493 | 0.391 | 0.822 |
| Methanobrevibacter smithii CAG:186 | 0 | Not detected | -0.029 | -0.437 | 0.378 | 0.887 |
| Methanocorpusculaceae archaeon Phil4 | 0 | Not detected | 0.138 | -0.134 | 0.411 | 0.320 |
| Methanosarcina mazei | 0 | Not detected | 0.055 | -0.191 | 0.300 | 0.663 |
| Methylobacterium sp. ZNC0032 | 0 | Not detected | -0.138 | -0.308 | 0.032 | 0.113 |
| Methyloglobulus morosus | 0 | Not detected | 0.066 | -0.138 | 0.269 | 0.526 |
| Microcystis aeruginosa | 0 | Not detected | 0.139 | -0.072 | 0.351 | 0.197 |
| Mitsuokella multacida | 0 | Not detected | 0.233 | -0.147 | 0.612 | 0.230 |
| Mobilibacterium timonense | 0 | Not detected | 0.038 | -0.150 | 0.226 | 0.689 |

| Mobiluncus curtisii | 0 | Not detected | -0.044 | -0.132 | 0.044 | 0.327 |
| --- | --- | --- | --- | --- | --- | --- |
| Mobiluncus mulieris | 0 | Not detected | -0.079 | -0.176 | 0.018 | 0.112 |
| Mogibacterium timidum | 0 | Not detected | 0.231 | 0.000 | 0.461 | 0.050 |
| Moraxella porci | 0 | Not detected | -0.043 | -0.202 | 0.117 | 0.600 |
| Morganella morganii | 0 | Not detected | 0.057 | -0.178 | 0.292 | 0.634 |
| Morococcus cerebrosus | 0 | Not detected | -0.034 | -0.210 | 0.143 | 0.710 |
| Mucinivorans hirudinis | 0 | Not detected | 0.075 | -0.077 | 0.227 | 0.334 |
| Murdochiella sp. Marseille-P2341 | 0 | Not detected | 0.006 | -0.112 | 0.124 | 0.922 |
| Mycoplasma sp. CAG:877 | 0 | Not detected | -0.131 | -0.421 | 0.159 | 0.376 |
| Mycoplasma sp. CAG:956 | 0 | Not detected | -0.035 | -0.284 | 0.214 | 0.784 |
| Myroides injenensis | 0 | Not detected | -0.095 | -0.338 | 0.148 | 0.443 |
| Myroides odoratimimus | 0 | Not detected | -0.125 | -0.394 | 0.144 | 0.362 |
| Necropsobacter massiliensis | 0 | Not detected | -0.126 | -0.353 | 0.100 | 0.274 |
| Negativicoccus massiliensis | 0 | Not detected | -0.024 | -0.197 | 0.149 | 0.786 |
| Neglecta timonensis | 0 | Not detected | 0.023 | -0.119 | 0.164 | 0.751 |
| Neisseria cinerea | 0 | Not detected | 0.050 | -0.144 | 0.243 | 0.614 |
| Neisseria mucosa | 0 | Not detected | -0.025 | -0.222 | 0.171 | 0.801 |
| Nissabacter archeti | 0 | Not detected | 0.197 | -0.041 | 0.435 | 0.106 |
| Novibacillus thermophilus | 0 | Not detected | 0.172 | -0.031 | 0.375 | 0.098 |
| Obesumbacterium proteus | 0 | Not detected | 0.124 | -0.157 | 0.405 | 0.388 |
| Oceanospirillum linum | 0 | Not detected | 0.111 | -0.090 | 0.312 | 0.278 |
| Odoribacter laneus | 0 | Not detected | 0.175 | -0.034 | 0.385 | 0.101 |
| Odoribacter laneus CAG:561 | 0 | Not detected | 0.102 | -0.170 | 0.374 | 0.464 |
| Odoribacter sp. 43_10 | 0 | Not detected | 0.084 | -0.094 | 0.262 | 0.356 |
| Odoribacter sp. CAG:788 | 0 | Not detected | 0.113 | -0.140 | 0.365 | 0.383 |
| Odoribacter splanchnicus | 0 | Not detected | 0.030 | -0.099 | 0.159 | 0.651 |
| Odoribacter splanchnicus CAG:14 | 0 | Not detected | 0.091 | -0.107 | 0.289 | 0.366 |
| Oligella ureolytica | 0 | Not detected | 0.029 | -0.164 | 0.222 | 0.770 |
| Olsenella scatoligenes | 0 | Not detected | 0.070 | -0.143 | 0.282 | 0.521 |
| Olsenella sp. Marseille-P3197 | 0 | Not detected | 0.059 | -0.157 | 0.274 | 0.594 |
| Olsenella sp. oral taxon 809 | 0 | Not detected | 0.045 | -0.093 | 0.183 | 0.526 |
| Olsenella uli | 0 | Not detected | 0.097 | -0.113 | 0.307 | 0.363 |
| Olsenella umbonata | 0 | Not detected | 0.020 | -0.146 | 0.186 | 0.817 |
| Oribacterium parvum | 0 | Not detected | -0.058 | -0.133 | 0.017 | 0.133 |
| Oribacterium sinus | 0 | Not detected | -0.050 | -0.212 | 0.113 | 0.549 |
| Oribacterium sp. WCC10 | 0 | Not detected | -0.074 | -0.284 | 0.136 | 0.488 |
| Oribacterium sp. oral taxon 078 | 0 | Not detected | 0.058 | -0.065 | 0.181 | 0.352 |
| Oscillibacter ruminantium | 0 | Not detected | 0.143 | -0.026 | 0.311 | 0.097 |
| Oscillibacter sp. 1-3 | 0 | Not detected | 0.073 | -0.023 | 0.169 | 0.139 |
| Oscillibacter sp. 57_20 | 0 | Not detected | 0.196 | -0.118 | 0.511 | 0.222 |
| Oscillibacter sp. ER4 | 0 | Not detected | 0.112 | -0.075 | 0.299 | 0.240 |
| Oscillibacter sp. KLE 1745 | 0 | Not detected | 0.006 | -0.155 | 0.166 | 0.945 |
| Oscillibacter valericigenes | 0 | Not detected | 0.138 | -0.021 | 0.297 | 0.090 |
| Oscillospiraceae bacterium Zagget12 | 0 | Not detected | -0.091 | -0.267 | 0.085 | 0.312 |

| Oxalobacter formigenes | 0 | Not detected | -0.007 | -0.202 | 0.189 | 0.946 |
| --- | --- | --- | --- | --- | --- | --- |
| Paeniclostridium sordellii | 0 | Not detected | -0.064 | -0.288 | 0.159 | 0.573 |
| Paludibacter propionicigenes | 0 | Not detected | -0.015 | -0.163 | 0.134 | 0.847 |
| Pantoea agglomerans | 0 | Not detected | 0.119 | -0.074 | 0.313 | 0.227 |
| Pantoea ananatis | 0 | Not detected | 0.042 | -0.137 | 0.220 | 0.647 |
| Pantoea dispersa | 0 | Not detected | 0.101 | -0.073 | 0.275 | 0.256 |
| Pantoea rwandensis | 0 | Not detected | 0.136 | -0.056 | 0.327 | 0.167 |
| Pantoea sp. IMH | 0 | Not detected | 0.086 | -0.109 | 0.281 | 0.388 |
| Pantoea sp. PSNIH2 | 0 | Not detected | 0.219 | -0.081 | 0.518 | 0.153 |
| Parabacteroides distasonis | 0 | Not detected | 0.087 | -0.100 | 0.273 | 0.362 |
| Parabacteroides goldsteinii | 0 | Not detected | -0.105 | -0.304 | 0.093 | 0.300 |
| Parabacteroides gordonii | 0 | Not detected | -0.059 | -0.226 | 0.107 | 0.485 |
| Parabacteroides johnsonii | 0 | Not detected | 0.029 | -0.115 | 0.173 | 0.694 |
| Parabacteroides merdae CAG:48 | 0 | Not detected | 0.043 | -0.221 | 0.307 | 0.750 |
| Paraburkholderia sacchari | 0 | Not detected | 0.069 | -0.130 | 0.267 | 0.497 |
| Paraclostridium bifermentans | 0 | Not detected | -0.086 | -0.286 | 0.114 | 0.398 |
| Paraprevotella clara CAG:116 | 0 | Not detected | -0.032 | -0.344 | 0.281 | 0.843 |
| Paraprevotella xylaniphila | 0 | Not detected | -0.040 | -0.205 | 0.125 | 0.633 |
| Parascardovia denticolens | 0 | Not detected | 0.113 | -0.100 | 0.326 | 0.300 |
| Parasporobacterium paucivorans | 0 | Not detected | -0.053 | -0.177 | 0.072 | 0.409 |
| Parasutterella excrementihominis | 0 | Not detected | 0.120 | -0.191 | 0.431 | 0.450 |
| Parasutterella excrementihominis CAG:233 | 0 | Not detected | 0.073 | -0.375 | 0.521 | 0.749 |
| Parvimonas micra | 0 | Not detected | 0.084 | -0.156 | 0.324 | 0.493 |
| Pasteurella bettyae | 0 | Not detected | -0.103 | -0.291 | 0.085 | 0.284 |
| Pectobacterium carotovorum | 0 | Not detected | 0.125 | -0.054 | 0.304 | 0.170 |
| Pedobacter glucosidilyticus | 0 | Not detected | 0.031 | -0.153 | 0.215 | 0.745 |
| Pelosinus fermentans | 0 | Not detected | -0.054 | -0.224 | 0.115 | 0.530 |
| Peptoanaerobacter stomatis | 0 | Not detected | 0.120 | -0.049 | 0.289 | 0.164 |
| Peptococcus niger | 0 | Not detected | 0.040 | -0.130 | 0.209 | 0.644 |
| Peptoniphilus duerdenii | 0 | Not detected | 0.030 | -0.128 | 0.189 | 0.708 |
| Peptoniphilus grossensis | 0 | Not detected | 0.018 | -0.175 | 0.210 | 0.857 |
| Peptoniphilus harei | 0 | Not detected | -0.056 | -0.267 | 0.156 | 0.606 |
| Peptoniphilus lacrimalis | 0 | Not detected | 0.095 | -0.138 | 0.327 | 0.426 |
| Peptoniphilus obesi | 0 | Not detected | 0.104 | -0.107 | 0.314 | 0.335 |
| Peptoniphilus rhinitidis | 0 | Not detected | 0.171 | -0.030 | 0.373 | 0.096 |
| Peptoniphilus senegalensis | 0 | Not detected | 0.200 | -0.020 | 0.421 | 0.075 |
| Peptoniphilus sp. BV3C26 | 0 | Not detected | 0.034 | -0.201 | 0.269 | 0.776 |
| Peptoniphilus sp. ING2-D1G | 0 | Not detected | 0.054 | -0.143 | 0.251 | 0.588 |
| Peptoniphilus sp. oral taxon 375 | 0 | Not detected | -0.024 | -0.136 | 0.088 | 0.680 |
| Peptostreptococcaceae bacterium VA2 | 0 | Not detected | -0.073 | -0.327 | 0.181 | 0.572 |
| Peptostreptococcus anaerobius | 0 | Not detected | 0.008 | -0.080 | 0.096 | 0.853 |
| Peptostreptococcus anaerobius CAG:621 | 0 | Not detected | -0.020 | -0.202 | 0.162 | 0.830 |
| Peptostreptococcus stomatis | 0 | Not detected | -0.268 | -0.474 | -0.062 | 0.011 |
| Petrimonas mucosa | 0 | Not detected | 0.041 | -0.172 | 0.254 | 0.707 |

| Phaeodactylibacter xiamenensis | 0 | Not detected | -0.008 | -0.275 | 0.259 | 0.951 |
| --- | --- | --- | --- | --- | --- | --- |
| Phascolarctobacterium sp. CAG:207 | 0 | Not detected | -0.195 | -0.640 | 0.250 | 0.390 |
| Phascolarctobacterium sp. CAG:266 | 0 | Not detected | -0.239 | -0.523 | 0.045 | 0.099 |
| Phascolarctobacterium succinatutens | 0 | Not detected | 0.038 | -0.193 | 0.268 | 0.749 |
| Phocaeicola abscessus | 0 | Not detected | -0.003 | -0.168 | 0.163 | 0.976 |
| Phocea massiliensis | 0 | Not detected | -0.032 | -0.152 | 0.089 | 0.606 |
| Piscirickettsia salmonis | 0 | Not detected | 0.011 | -0.157 | 0.179 | 0.894 |
| Plautia stali symbiont | 0 | Not detected | 0.080 | -0.101 | 0.261 | 0.385 |
| Pluralibacter gergoviae | 0 | Not detected | 0.230 | -0.052 | 0.512 | 0.110 |
| Pontibacillus litoralis | 0 | Not detected | 0.035 | -0.240 | 0.310 | 0.805 |
| Porphyromonadaceae bacterium COT-184 | 0 | Not detected | 0.008 | -0.177 | 0.193 | 0.933 |
| Porphyromonadaceae bacterium KH3CP3R | 0 | Not detected | 0.041 | -0.135 | 0.218 | 0.647 |
| Porphyromonadaceae bacterium KH3R12 | 0 | Not detected | -0.008 | -0.173 | 0.157 | 0.927 |
| Porphyromonas asaccharolytica | 0 | Not detected | 0.103 | -0.116 | 0.321 | 0.358 |
| Porphyromonas bennonis | 0 | Not detected | 0.142 | -0.067 | 0.350 | 0.184 |
| Porphyromonas cangingivalis | 0 | Not detected | 0.025 | -0.164 | 0.214 | 0.796 |
| Porphyromonas canoris | 0 | Not detected | -0.015 | -0.208 | 0.178 | 0.882 |
| Porphyromonas catoniae | 0 | Not detected | -0.082 | -0.220 | 0.055 | 0.240 |
| Porphyromonas crevioricanis | 0 | Not detected | -0.014 | -0.169 | 0.140 | 0.855 |
| Porphyromonas endodontalis | 0 | Not detected | 0.029 | -0.181 | 0.240 | 0.784 |
| Porphyromonas gingivalis | 0 | Not detected | 0.010 | -0.168 | 0.189 | 0.908 |
| Porphyromonas gingivicanis | 0 | Not detected | 0.133 | -0.001 | 0.266 | 0.052 |
| Porphyromonas levii | 0 | Not detected | -0.111 | -0.372 | 0.150 | 0.406 |
| Porphyromonas macacae | 0 | Not detected | -0.014 | -0.202 | 0.174 | 0.886 |
| Porphyromonas somerae | 0 | Not detected | 0.008 | -0.103 | 0.120 | 0.884 |
| Porphyromonas sp. CAG:1061 | 0 | Not detected | 0.131 | -0.109 | 0.372 | 0.285 |
| Porphyromonas sp. COT-239 OH1446 | 0 | Not detected | -0.145 | -0.411 | 0.121 | 0.286 |
| Porphyromonas sp. COT-290 OH3588 | 0 | Not detected | 0.018 | -0.212 | 0.248 | 0.880 |
| Prevotella albensis | 0 | Not detected | -0.031 | -0.402 | 0.339 | 0.868 |
| Prevotella amnii | 0 | Not detected | -0.032 | -0.134 | 0.070 | 0.545 |
| Prevotella aurantiaca | 0 | Not detected | -0.062 | -0.298 | 0.173 | 0.604 |
| Prevotella baroniae | 0 | Not detected | 0.005 | -0.116 | 0.126 | 0.930 |
| Prevotella bergensis | 0 | Not detected | -0.017 | -0.203 | 0.168 | 0.855 |
| Prevotella bivia | 0 | Not detected | 0.024 | -0.106 | 0.154 | 0.716 |
| Prevotella bryantii | 0 | Not detected | -0.055 | -0.427 | 0.317 | 0.772 |
| Prevotella buccae | 0 | Not detected | -0.056 | -0.317 | 0.205 | 0.673 |
| Prevotella buccalis | 0 | Not detected | -0.026 | -0.140 | 0.088 | 0.659 |
| Prevotella copri | 0 | Not detected | -0.231 | -0.641 | 0.180 | 0.271 |
| Prevotella corporis | 0 | Not detected | 0.026 | -0.085 | 0.136 | 0.650 |
| Prevotella dentalis | 0 | Not detected | 0.042 | -0.246 | 0.330 | 0.775 |
| Prevotella dentasini | 0 | Not detected | -0.070 | -0.343 | 0.203 | 0.616 |
| Prevotella denticola | 0 | Not detected | 0.018 | -0.290 | 0.326 | 0.909 |
| Prevotella disiens | 0 | Not detected | 0.055 | -0.062 | 0.172 | 0.356 |
| Prevotella enoeca | 0 | Not detected | -0.063 | -0.332 | 0.206 | 0.647 |

| Prevotella fusca | 0 | Not detected | -0.122 | -0.440 | 0.195 | 0.451 |
| --- | --- | --- | --- | --- | --- | --- |
| Prevotella histicola | 0 | Not detected | 0.022 | -0.176 | 0.221 | 0.824 |
| Prevotella intermedia | 0 | Not detected | 0.000 | -0.180 | 0.181 | 0.999 |
| Prevotella loescheii | 0 | Not detected | -0.022 | -0.267 | 0.223 | 0.862 |
| Prevotella maculosa | 0 | Not detected | -0.098 | -0.317 | 0.121 | 0.381 |
| Prevotella marshii | 0 | Not detected | 0.030 | -0.087 | 0.147 | 0.615 |
| Prevotella melaninogenica | 0 | Not detected | -0.130 | -0.368 | 0.107 | 0.283 |
| Prevotella micans | 0 | Not detected | -0.189 | -0.435 | 0.057 | 0.132 |
| Prevotella multiformis | 0 | Not detected | -0.114 | -0.286 | 0.057 | 0.192 |
| Prevotella multisaccharivorax | 0 | Not detected | -0.077 | -0.441 | 0.286 | 0.677 |
| Prevotella nanceiensis | 0 | Not detected | 0.076 | -0.138 | 0.290 | 0.488 |
| Prevotella nigrescens | 0 | Not detected | -0.059 | -0.302 | 0.184 | 0.634 |
| Prevotella oralis | 0 | Not detected | -0.097 | -0.354 | 0.161 | 0.461 |
| Prevotella oris | 0 | Not detected | -0.018 | -0.317 | 0.282 | 0.908 |
| Prevotella oryzae | 0 | Not detected | -0.123 | -0.436 | 0.190 | 0.442 |
| Prevotella oulorum | 0 | Not detected | -0.154 | -0.480 | 0.173 | 0.357 |
| Prevotella pallens | 0 | Not detected | -0.030 | -0.163 | 0.102 | 0.653 |
| Prevotella paludivivens | 0 | Not detected | -0.071 | -0.267 | 0.124 | 0.476 |
| Prevotella ruminicola | 0 | Not detected | -0.089 | -0.384 | 0.206 | 0.554 |
| Prevotella saccharolytica | 0 | Not detected | -0.219 | -0.500 | 0.062 | 0.127 |
| Prevotella salivae | 0 | Not detected | 0.008 | -0.115 | 0.132 | 0.895 |
| Prevotella scopos | 0 | Not detected | -0.037 | -0.198 | 0.125 | 0.655 |
| Prevotella sp. 10(H) | 0 | Not detected | 0.013 | -0.155 | 0.182 | 0.876 |
| Prevotella sp. 109 | 0 | Not detected | 0.067 | -0.051 | 0.186 | 0.266 |
| Prevotella sp. AGR2160 | 0 | Not detected | -0.012 | -0.347 | 0.323 | 0.944 |
| Prevotella sp. BP1-145 | 0 | Not detected | -0.041 | -0.163 | 0.080 | 0.507 |
| Prevotella sp. BP1-148 | 0 | Not detected | 0.016 | -0.124 | 0.157 | 0.819 |
| Prevotella sp. CAG:1058 | 0 | Not detected | -0.031 | -0.302 | 0.241 | 0.826 |
| Prevotella sp. CAG:1092 | 0 | Not detected | -0.029 | -0.392 | 0.335 | 0.877 |
| Prevotella sp. CAG:1124 | 0 | Not detected | -0.059 | -0.339 | 0.222 | 0.682 |
| Prevotella sp. CAG:1185 | 0 | Not detected | -0.046 | -0.337 | 0.245 | 0.755 |
| Prevotella sp. CAG:1320 | 0 | Not detected | 0.225 | -0.013 | 0.464 | 0.064 |
| Prevotella sp. CAG:255 | 0 | Not detected | -0.033 | -0.229 | 0.163 | 0.742 |
| Prevotella sp. CAG:386 | 0 | Not detected | -0.117 | -0.541 | 0.308 | 0.589 |
| Prevotella sp. CAG:487 | 0 | Not detected | -0.007 | -0.208 | 0.195 | 0.947 |
| Prevotella sp. CAG:520 | 0 | Not detected | -0.037 | -0.382 | 0.308 | 0.834 |
| Prevotella sp. CAG:5226 | 0 | Not detected | 0.065 | -0.309 | 0.439 | 0.732 |
| Prevotella sp. CAG:604 | 0 | Not detected | -0.295 | -0.752 | 0.162 | 0.207 |
| Prevotella sp. CAG:732 | 0 | Not detected | 0.006 | -0.407 | 0.418 | 0.978 |
| Prevotella sp. CAG:891 | 0 | Not detected | -0.076 | -0.359 | 0.208 | 0.601 |
| Prevotella sp. FD3004 | 0 | Not detected | -0.066 | -0.292 | 0.159 | 0.565 |
| Prevotella sp. HUN102 | 0 | Not detected | -0.035 | -0.198 | 0.128 | 0.677 |
| Prevotella sp. MSX73 | 0 | Not detected | 0.012 | -0.246 | 0.270 | 0.928 |
| Prevotella sp. P4-76 | 0 | Not detected | -0.035 | -0.250 | 0.181 | 0.754 |

| Prevotella sp. P6B1 | 0 | Not detected | 0.011 | -0.135 | 0.157 | 0.881 |
| --- | --- | --- | --- | --- | --- | --- |
| Prevotella sp. P6B4 | 0 | Not detected | -0.004 | -0.148 | 0.141 | 0.960 |
| Prevotella sp. RM4 | 0 | Not detected | -0.156 | -0.341 | 0.030 | 0.100 |
| Prevotella sp. S7 MS 2 | 0 | Not detected | -0.022 | -0.143 | 0.100 | 0.728 |
| Prevotella sp. S7-1-8 | 0 | Not detected | -0.032 | -0.145 | 0.081 | 0.581 |
| Prevotella sp. kh1p2 | 0 | Not detected | -0.185 | -0.446 | 0.075 | 0.164 |
| Prevotella sp. khp1 | 0 | Not detected | -0.131 | -0.323 | 0.062 | 0.184 |
| Prevotella sp. ne3005 | 0 | Not detected | -0.066 | -0.231 | 0.099 | 0.431 |
| Prevotella sp. oral taxon 473 | 0 | Not detected | -0.109 | -0.368 | 0.150 | 0.409 |
| Prevotella stercorea | 0 | Not detected | 0.036 | -0.125 | 0.197 | 0.663 |
| Prevotella stercorea CAG:629 | 0 | Not detected | -0.036 | -0.401 | 0.330 | 0.849 |
| Prevotella timonensis | 0 | Not detected | 0.023 | -0.089 | 0.135 | 0.690 |
| Prevotellamassilia timonensis | 0 | Not detected | 0.040 | -0.150 | 0.229 | 0.683 |
| Propionibacterium acidifaciens | 0 | Not detected | 0.130 | -0.075 | 0.335 | 0.214 |
| Proteiniclasticum ruminis | 0 | Not detected | 0.064 | -0.107 | 0.234 | 0.465 |
| Proteiniphilum acetatigenes | 0 | Not detected | -0.108 | -0.287 | 0.071 | 0.239 |
| Proteiniphilum saccharofermentans | 0 | Not detected | -0.080 | -0.278 | 0.117 | 0.425 |
| Proteiniphilum sp. EBM-41 | 0 | Not detected | 0.128 | -0.118 | 0.375 | 0.307 |
| Proteobacteria bacterium CAG:139 | 0 | Not detected | 0.032 | -0.393 | 0.457 | 0.883 |
| Proteobacteria bacterium CAG:495 | 0 | Not detected | -0.002 | -0.348 | 0.343 | 0.990 |
| Providencia rettgeri | 0 | Not detected | 0.086 | -0.135 | 0.308 | 0.444 |
| Pseudobacteroides cellulosolvens | 0 | Not detected | 0.064 | -0.086 | 0.214 | 0.403 |
| Pseudobutyrivibrio ruminis | 0 | Not detected | -0.040 | -0.131 | 0.051 | 0.389 |
| Pseudobutyrivibrio sp. ACV-2 | 0 | Not detected | -0.009 | -0.183 | 0.166 | 0.920 |
| Pseudobutyrivibrio sp. JW11 | 0 | Not detected | -0.051 | -0.230 | 0.128 | 0.575 |
| Pseudobutyrivibrio sp. MD2005 | 0 | Not detected | -0.001 | -0.186 | 0.184 | 0.990 |
| Pseudobutyrivibrio sp. UC1225 | 0 | Not detected | 0.058 | -0.076 | 0.191 | 0.395 |
| Pseudobutyrivibrio sp. YE44 | 0 | Not detected | 0.009 | -0.162 | 0.180 | 0.920 |
| Pseudobutyrivibrio xylanivorans | 0 | Not detected | 0.057 | -0.121 | 0.235 | 0.532 |
| Pseudoflavonifractor capillosus | 0 | Not detected | 0.065 | -0.037 | 0.167 | 0.212 |
| Pseudopropionibacterium propionicum | 0 | Not detected | 0.055 | -0.148 | 0.258 | 0.595 |
| Pyramidobacter piscolens | 0 | Not detected | 0.049 | -0.234 | 0.333 | 0.732 |
| Rahnella aquatilis | 0 | Not detected | 0.052 | -0.122 | 0.227 | 0.558 |
| Ralstonia mannitolilytica | 0 | Not detected | 0.272 | 0.028 | 0.516 | 0.029 |
| Ralstonia pickettii | 0 | Not detected | 0.044 | -0.186 | 0.273 | 0.710 |
| Raoultella ornithinolytica | 0 | Not detected | 0.246 | -0.103 | 0.594 | 0.167 |
| Rappaport israeli | 0 | Not detected | 0.208 | 0.022 | 0.395 | 0.029 |
| Rhodococcus defluvii | 0 | Not detected | -0.074 | -0.246 | 0.097 | 0.394 |
| Rhodopseudomonas palustris | 0 | Not detected | 0.234 | 0.027 | 0.442 | 0.027 |
| Rhodospirillum sp. UNK.MGS-17 | 0 | Not detected | 0.043 | -0.362 | 0.448 | 0.837 |
| Riemerella columbina | 0 | Not detected | 0.002 | -0.111 | 0.114 | 0.978 |
| Rikenella microfusus | 0 | Not detected | 0.011 | -0.102 | 0.124 | 0.844 |
| Risungbinella massiliensis | 0 | Not detected | 0.042 | -0.102 | 0.185 | 0.571 |
| Robinsoniella peoriensis | 0 | Not detected | -0.055 | -0.129 | 0.020 | 0.153 |

| Rodentibacter pneumotropicus | 0 | Not detected | -0.079 | -0.237 | 0.078 | 0.324 |
| --- | --- | --- | --- | --- | --- | --- |
| Roseburia faecis | 0 | Not detected | -0.033 | -0.204 | 0.138 | 0.705 |
| Roseburia hominis | 0 | Not detected | -0.021 | -0.130 | 0.088 | 0.706 |
| Roseburia intestinalis | 0 | Not detected | -0.020 | -0.188 | 0.147 | 0.813 |
| Roseburia inulinivorans | 0 | Not detected | 0.123 | -0.012 | 0.258 | 0.075 |
| Roseburia sp. CAG:197_41_10 | 0 | Not detected | 0.013 | -0.123 | 0.149 | 0.850 |
| Roseburia sp. CAG:309 | 0 | Not detected | -0.120 | -0.390 | 0.149 | 0.381 |
| Roseburia sp. CAG:380 | 0 | Not detected | -0.024 | -0.285 | 0.237 | 0.855 |
| Rothia dentocariosa | 0 | Not detected | 0.003 | -0.254 | 0.260 | 0.982 |
| Rothia mucilaginosa | 0 | Not detected | -0.004 | -0.289 | 0.281 | 0.977 |
| Ruminiclostridium thermocellum | 0 | Not detected | -0.008 | -0.125 | 0.108 | 0.887 |
| Ruminococcaceae bacterium AB4001 | 0 | Not detected | 0.104 | -0.052 | 0.260 | 0.190 |
| Ruminococcaceae bacterium AE2021 | 0 | Not detected | 0.095 | -0.052 | 0.241 | 0.204 |
| Ruminococcaceae bacterium D16 | 0 | Not detected | 0.070 | -0.024 | 0.164 | 0.143 |
| Ruminococcaceae bacterium YRB3002 | 0 | Not detected | 0.109 | -0.075 | 0.292 | 0.245 |
| Ruminococcus albus | 0 | Not detected | 0.017 | -0.158 | 0.192 | 0.846 |
| Ruminococcus bicirculans | 0 | Not detected | -0.074 | -0.357 | 0.208 | 0.606 |
| Ruminococcus callidus | 0 | Not detected | -0.052 | -0.244 | 0.140 | 0.596 |
| Ruminococcus champanellensis | 0 | Not detected | 0.117 | -0.122 | 0.356 | 0.339 |
| Ruminococcus faecis | 0 | Not detected | -0.033 | -0.161 | 0.094 | 0.610 |
| Ruminococcus flavefaciens | 0 | Not detected | 0.045 | -0.127 | 0.218 | 0.606 |
| Ruminococcus gauvreauii | 0 | Not detected | -0.060 | -0.155 | 0.036 | 0.219 |
| Ruminococcus lactaris | 0 | Not detected | 0.019 | -0.126 | 0.164 | 0.802 |
| Ruminococcus obeum CAG:39 | 0 | Not detected | 0.071 | -0.098 | 0.239 | 0.410 |
| Ruminococcus sp. AT10 | 0 | Not detected | -0.017 | -0.108 | 0.073 | 0.707 |
| Ruminococcus sp. CAG:108-related_41_35 | 0 | Not detected | 0.066 | -0.230 | 0.362 | 0.661 |
| Ruminococcus sp. CAG:177 | 0 | Not detected | 0.194 | -0.265 | 0.654 | 0.407 |
| Ruminococcus sp. CAG:254 | 0 | Not detected | -0.146 | -0.555 | 0.263 | 0.484 |
| Ruminococcus sp. CAG:330 | 0 | Not detected | -0.219 | -0.603 | 0.164 | 0.263 |
| Ruminococcus sp. CAG:379 | 0 | Not detected | -0.125 | -0.398 | 0.147 | 0.367 |
| Ruminococcus sp. CAG:382 | 0 | Not detected | 0.092 | -0.283 | 0.467 | 0.629 |
| Ruminococcus sp. CAG:403 | 0 | Not detected | -0.165 | -0.466 | 0.135 | 0.281 |
| Ruminococcus sp. CAG:488 | 0 | Not detected | -0.004 | -0.270 | 0.262 | 0.976 |
| Ruminococcus sp. CAG:563 | 0 | Not detected | 0.048 | -0.237 | 0.332 | 0.743 |
| Ruminococcus sp. CAG:60 | 0 | Not detected | -0.067 | -0.238 | 0.103 | 0.439 |
| Ruminococcus sp. CAG:624 | 0 | Not detected | -0.034 | -0.300 | 0.232 | 0.803 |
| Ruminococcus sp. CAG:724 | 0 | Not detected | -0.061 | -0.377 | 0.256 | 0.707 |
| Ruminococcus sp. FC2018 | 0 | Not detected | 0.022 | -0.165 | 0.208 | 0.821 |
| Ruminococcus sp. HUN007 | 0 | Not detected | 0.082 | -0.076 | 0.240 | 0.307 |
| Ruminococcus sp. NK3A76 | 0 | Not detected | 0.095 | -0.093 | 0.282 | 0.322 |
| Ruminococcus sp. Phil15 | 0 | Not detected | -0.025 | -0.266 | 0.217 | 0.842 |
| Ruminococcus sp. Zagget7 | 0 | Not detected | 0.046 | -0.104 | 0.196 | 0.550 |
| Ruminococcus torques CAG:61 | 0 | Not detected | -0.047 | -0.333 | 0.240 | 0.751 |
| Ruthenibacterium lactatiformans | 0 | Not detected | 0.028 | -0.102 | 0.159 | 0.670 |

| Salmonella enterica | 0 | Not detected | 0.198 | -0.105 | 0.502 | 0.200 |
| --- | --- | --- | --- | --- | --- | --- |
| Sanguibacteroides justesenii | 0 | Not detected | -0.011 | -0.226 | 0.203 | 0.917 |
| Scardovia inopinata | 0 | Not detected | 0.092 | -0.082 | 0.266 | 0.299 |
| Scardovia wiggsiae | 0 | Not detected | -0.060 | -0.292 | 0.173 | 0.615 |
| Selenomonas bovis | 0 | Not detected | 0.159 | 0.000 | 0.319 | 0.050 |
| Selenomonas noxia | 0 | Not detected | -0.018 | -0.191 | 0.156 | 0.843 |
| Selenomonas ruminantium | 0 | Not detected | 0.000 | -0.223 | 0.224 | 0.998 |
| Selenomonas sputigena | 0 | Not detected | 0.018 | -0.163 | 0.198 | 0.849 |
| Sellimonas intestinalis | 0 | Not detected | 0.022 | -0.113 | 0.157 | 0.748 |
| Senegalimassilia anaerobia | 0 | Not detected | 0.037 | -0.204 | 0.277 | 0.765 |
| Serratia fonticola | 0 | Not detected | 0.088 | -0.082 | 0.258 | 0.310 |
| Serratia liquefaciens | 0 | Not detected | 0.051 | -0.127 | 0.229 | 0.577 |
| Serratia marcescens | 0 | Not detected | 0.139 | -0.055 | 0.332 | 0.160 |
| Serratia odorifera | 0 | Not detected | 0.062 | -0.125 | 0.249 | 0.517 |
| Serratia rubidaea | 0 | Not detected | 0.127 | -0.071 | 0.324 | 0.209 |
| Sharpea azabuensis | 0 | Not detected | 0.039 | -0.078 | 0.156 | 0.517 |
| Shigella dysenteriae | 0 | Not detected | 0.125 | -0.206 | 0.457 | 0.460 |
| Shigella flexneri | 0 | Not detected | 0.188 | -0.143 | 0.518 | 0.266 |
| Shimwellia blattae | 0 | Not detected | 0.172 | -0.082 | 0.425 | 0.185 |
| Shuttleworthia satelles | 0 | Not detected | -0.063 | -0.180 | 0.055 | 0.298 |
| Siccibacter turicensis | 0 | Not detected | 0.131 | -0.136 | 0.399 | 0.336 |
| Slackia exigua | 0 | Not detected | 0.078 | -0.107 | 0.263 | 0.406 |
| Slackia heliotrinireducens | 0 | Not detected | -0.012 | -0.168 | 0.144 | 0.881 |
| Slackia piriformis | 0 | Not detected | 0.234 | -0.058 | 0.527 | 0.117 |
| Solobacterium moorei | 0 | Not detected | 0.191 | 0.058 | 0.324 | 0.005 |
| Sphaerochaeta sp. Phil3 | 0 | Not detected | 0.064 | -0.163 | 0.291 | 0.583 |
| Sphingobacterium wenxiniae | 0 | Not detected | 0.141 | -0.072 | 0.355 | 0.195 |
| Spirochaetales bacterium Spiro_07 | 0 | Not detected | 0.136 | -0.018 | 0.290 | 0.083 |
| Sporobacter termitidis | 0 | Not detected | 0.144 | -0.036 | 0.324 | 0.117 |
| Sporomusa ovata | 0 | Not detected | -0.038 | -0.227 | 0.151 | 0.695 |
| Staphylococcus pseudintermedius | 0 | Not detected | 0.203 | 0.007 | 0.399 | 0.042 |
| Staphylococcus sp. CAG:324 | 0 | Not detected | -0.098 | -0.456 | 0.260 | 0.590 |
| Staphylococcus sp. DORA_6_22 | 0 | Not detected | -0.124 | -0.318 | 0.071 | 0.213 |
| Staphylococcus warneri | 0 | Not detected | 0.084 | -0.129 | 0.297 | 0.439 |
| Stomatobaculum longum | 0 | Not detected | 0.024 | -0.052 | 0.100 | 0.529 |
| Streptobacillus hongkongensis | 0 | Not detected | 0.014 | -0.180 | 0.208 | 0.887 |
| Streptobacillus notomytis | 0 | Not detected | 0.115 | -0.104 | 0.335 | 0.303 |
| Streptococcus agalactiae | 0 | Not detected | 0.053 | -0.162 | 0.268 | 0.631 |
| Streptococcus anginosus | 0 | Not detected | 0.131 | -0.123 | 0.386 | 0.313 |
| Streptococcus caballi | 0 | Not detected | 0.078 | -0.141 | 0.296 | 0.485 |
| Streptococcus cristatus | 0 | Not detected | -0.043 | -0.247 | 0.160 | 0.675 |
| Streptococcus dysgalactiae | 0 | Not detected | 0.182 | -0.030 | 0.394 | 0.093 |
| Streptococcus entericus | 0 | Not detected | 0.162 | -0.016 | 0.339 | 0.074 |
| Streptococcus gallolyticus | 0 | Not detected | -0.001 | -0.277 | 0.274 | 0.993 |

| Streptococcus gordonii | 0 | Not detected | 0.061 | -0.201 | 0.323 | 0.646 |
| --- | --- | --- | --- | --- | --- | --- |
| Streptococcus halotolerans | 0 | Not detected | -0.001 | -0.177 | 0.175 | 0.991 |
| Streptococcus henryi | 0 | Not detected | -0.007 | -0.218 | 0.205 | 0.952 |
| Streptococcus ictaluri | 0 | Not detected | -0.050 | -0.236 | 0.136 | 0.598 |
| Streptococcus infantis | 0 | Not detected | -0.031 | -0.240 | 0.178 | 0.771 |
| Streptococcus iniae | 0 | Not detected | -0.014 | -0.207 | 0.178 | 0.884 |
| Streptococcus intermedius | 0 | Not detected | -0.079 | -0.323 | 0.165 | 0.528 |
| Streptococcus massiliensis | 0 | Not detected | 0.140 | -0.056 | 0.336 | 0.163 |
| Streptococcus minor | 0 | Not detected | -0.010 | -0.157 | 0.136 | 0.890 |
| Streptococcus mitis | 0 | Not detected | 0.003 | -0.162 | 0.167 | 0.975 |
| Streptococcus mutans | 0 | Not detected | -0.036 | -0.333 | 0.261 | 0.813 |
| Streptococcus oralis | 0 | Not detected | 0.024 | -0.214 | 0.262 | 0.842 |
| Streptococcus orisratti | 0 | Not detected | 0.181 | -0.029 | 0.390 | 0.092 |
| Streptococcus ovis | 0 | Not detected | -0.010 | -0.199 | 0.179 | 0.919 |
| Streptococcus parasanguinis | 0 | Not detected | -0.062 | -0.322 | 0.197 | 0.638 |
| Streptococcus parauberis | 0 | Not detected | -0.089 | -0.281 | 0.104 | 0.367 |
| Streptococcus phocae | 0 | Not detected | 0.018 | -0.127 | 0.163 | 0.809 |
| Streptococcus plurextorum | 0 | Not detected | 0.004 | -0.099 | 0.107 | 0.934 |
| Streptococcus porci | 0 | Not detected | 0.007 | -0.118 | 0.132 | 0.912 |
| Streptococcus pseudoporcinus | 0 | Not detected | 0.105 | -0.104 | 0.314 | 0.324 |
| Streptococcus pyogenes | 0 | Not detected | 0.001 | -0.210 | 0.213 | 0.990 |
| Streptococcus salivarius | 0 | Not detected | -0.090 | -0.328 | 0.149 | 0.461 |
| Streptococcus sanguinis | 0 | Not detected | 0.043 | -0.207 | 0.294 | 0.735 |
| Streptococcus sobrinus | 0 | Not detected | -0.014 | -0.235 | 0.207 | 0.903 |
| Streptococcus sp. X13SY08 | 0 | Not detected | 0.008 | -0.145 | 0.160 | 0.922 |
| Streptococcus suis | 0 | Not detected | 0.068 | -0.047 | 0.183 | 0.244 |
| Streptococcus thermophilus | 0 | Not detected | -0.191 | -0.448 | 0.067 | 0.148 |
| Streptococcus uberis | 0 | Not detected | -0.025 | -0.208 | 0.159 | 0.793 |
| Streptococcus urinalis | 0 | Not detected | 0.002 | -0.211 | 0.214 | 0.989 |
| Streptomyces albulus | 0 | Not detected | -0.093 | -0.262 | 0.077 | 0.284 |
| Streptomyces cattleya | 0 | Not detected | 0.084 | -0.127 | 0.295 | 0.434 |
| Streptomyces kanamyceticus | 0 | Not detected | 0.046 | -0.075 | 0.167 | 0.453 |
| Subdoligranulum sp. CAG:314 | 0 | Not detected | 0.283 | -0.150 | 0.715 | 0.201 |
| Subdoligranulum variabile | 0 | Not detected | 0.048 | -0.043 | 0.139 | 0.304 |
| Succinatimonas sp. CAG:777 | 0 | Not detected | 0.052 | -0.300 | 0.403 | 0.774 |
| Succinispira mobilis | 0 | Not detected | -0.077 | -0.282 | 0.128 | 0.462 |
| Succinivibrio sp. Phil9 | 0 | Not detected | -0.016 | -0.353 | 0.320 | 0.925 |
| Sulfolobus acidocaldarius | 0 | Not detected | 0.112 | -0.147 | 0.370 | 0.398 |
| Sutterella parvirubra | 0 | Not detected | 0.096 | -0.145 | 0.337 | 0.436 |
| Sutterella sp. CAG:397 | 0 | Not detected | -0.074 | -0.551 | 0.402 | 0.760 |
| Sutterella sp. CAG:521 | 0 | Not detected | -0.085 | -0.360 | 0.191 | 0.547 |
| Sutterella wadsworthensis | 0 | Not detected | 0.167 | -0.241 | 0.575 | 0.423 |
| Sutterella wadsworthensis CAG:135 | 0 | Not detected | 0.029 | -0.468 | 0.526 | 0.908 |
| Sutterellaceae bacterium ND3 | 0 | Not detected | -0.010 | -0.313 | 0.293 | 0.947 |

| Synergistales bacterium Syner_02 | 0 | Not detected | 0.177 | -0.056 | 0.411 | 0.137 |
| --- | --- | --- | --- | --- | --- | --- |
| Synergistes sp. 3_1_syn1 | 0 | Not detected | 0.078 | -0.225 | 0.381 | 0.613 |
| Synergistes sp. Zagget9 | 0 | Not detected | 0.099 | -0.102 | 0.300 | 0.332 |
| Tannerella forsythia | 0 | Not detected | 0.009 | -0.145 | 0.162 | 0.914 |
| Tatumella morbirosei | 0 | Not detected | 0.152 | -0.027 | 0.331 | 0.096 |
| Tepidanaerobacter sp. EBM-49 | 0 | Not detected | 0.065 | -0.223 | 0.353 | 0.660 |
| Terrisporobacter glycolicus | 0 | Not detected | -0.202 | -0.435 | 0.031 | 0.089 |
| Thermophagus xiamenensis | 0 | Not detected | 0.005 | -0.201 | 0.210 | 0.964 |
| Thermosinus carboxydivorans | 0 | Not detected | -0.034 | -0.194 | 0.125 | 0.673 |
| Tidjanibacter massiliensis | 0 | Not detected | 0.036 | -0.285 | 0.357 | 0.827 |
| Tissierellia bacterium S7-1-4 | 0 | Not detected | -0.179 | -0.390 | 0.033 | 0.098 |
| Trabulsiella odontotermitis | 0 | Not detected | 0.241 | -0.041 | 0.522 | 0.094 |
| Traorella massiliensis | 0 | Not detected | 0.022 | -0.068 | 0.113 | 0.628 |
| Treponema denticola | 0 | Not detected | 0.042 | -0.114 | 0.198 | 0.594 |
| Treponema medium | 0 | Not detected | -0.055 | -0.323 | 0.213 | 0.689 |
| Treponema putidum | 0 | Not detected | -0.028 | -0.169 | 0.114 | 0.702 |
| Treponema socranskii | 0 | Not detected | -0.021 | -0.182 | 0.139 | 0.793 |
| Treponema succinifaciens | 0 | Not detected | 0.057 | -0.055 | 0.169 | 0.320 |
| Treponema vincentii | 0 | Not detected | -0.005 | -0.151 | 0.141 | 0.943 |
| Trichococcus flocculiformis | 0 | Not detected | -0.148 | -0.322 | 0.026 | 0.095 |
| Trueperella pyogenes | 0 | Not detected | 0.144 | -0.042 | 0.329 | 0.130 |
| Tumebacillus flagellatus | 0 | Not detected | 0.126 | -0.058 | 0.310 | 0.179 |
| Turicibacter sanguinis | 0 | Not detected | -0.230 | -0.557 | 0.097 | 0.169 |
| Turicibacter sp. H121 | 0 | Not detected | -0.085 | -0.224 | 0.054 | 0.233 |
| Tyzzerella nexilis | 0 | Not detected | -0.020 | -0.103 | 0.063 | 0.638 |
| Urmitella timonensis | 0 | Not detected | -0.096 | -0.234 | 0.042 | 0.173 |
| Vaginella massiliensis | 0 | Not detected | -0.155 | -0.426 | 0.117 | 0.264 |
| Varibaculum cambriense | 0 | Not detected | -0.061 | -0.181 | 0.059 | 0.321 |
| Varibaculum sp. Marseille-P2802 | 0 | Not detected | 0.093 | -0.091 | 0.277 | 0.320 |
| Veillonella magna | 0 | Not detected | -0.053 | -0.139 | 0.033 | 0.228 |
| Veillonella parvula | 0 | Not detected | -0.157 | -0.445 | 0.132 | 0.287 |
| Veillonella seminalis | 0 | Not detected | -0.034 | -0.150 | 0.083 | 0.570 |
| Veillonella sp. ACP1 | 0 | Not detected | -0.139 | -0.477 | 0.199 | 0.421 |
| Veillonella sp. CAG:933 | 0 | Not detected | -0.187 | -0.367 | -0.008 | 0.041 |
| Veillonella sp. oral taxon 780 | 0 | Not detected | -0.186 | -0.383 | 0.011 | 0.064 |
| Waddlia chondrophila | 0 | Not detected | 0.128 | -0.122 | 0.378 | 0.315 |
| Weissella cibaria | 0 | Not detected | -0.127 | -0.387 | 0.132 | 0.337 |
| Weissella confusa | 0 | Not detected | -0.271 | -0.560 | 0.019 | 0.067 |
| Xanthomonas sp. Mitacek01 | 0 | Not detected | -0.089 | -0.227 | 0.049 | 0.206 |
| Yersinia enterocolitica | 0 | Not detected | 0.122 | -0.123 | 0.367 | 0.329 |
| Yersinia pestis | 0 | Not detected | 0.127 | -0.186 | 0.440 | 0.427 |
| Yokenella regensburgei | 0 | Not detected | 0.246 | -0.032 | 0.524 | 0.083 |
| [Bacteroides] pectinophilus | 0 | Not detected | -0.023 | -0.119 | 0.074 | 0.646 |
| [Clostridium] aminophilum | 0 | Not detected | 0.001 | -0.087 | 0.089 | 0.981 |

| [Clostridium] asparagiforme | 0 | Not detected | 0.004 | -0.121 | 0.130 | 0.946 |
| --- | --- | --- | --- | --- | --- | --- |
| [Clostridium] bolteae | 0 | Not detected | -0.043 | -0.162 | 0.077 | 0.484 |
| [Clostridium] celerecrescens | 0 | Not detected | -0.071 | -0.152 | 0.011 | 0.090 |
| [Clostridium] cellobioparum | 0 | Not detected | 0.082 | -0.074 | 0.238 | 0.302 |
| [Clostridium] cellulolyticum | 0 | Not detected | 0.034 | -0.152 | 0.221 | 0.717 |
| [Clostridium] cellulosi | 0 | Not detected | -0.010 | -0.188 | 0.168 | 0.913 |
| [Clostridium] citroniae | 0 | Not detected | -0.065 | -0.162 | 0.032 | 0.192 |
| [Clostridium] clariflavum | 0 | Not detected | -0.056 | -0.200 | 0.088 | 0.445 |
| [Clostridium] clostridioforme | 0 | Not detected | -0.084 | -0.216 | 0.048 | 0.213 |
| [Clostridium] dakarense | 0 | Not detected | -0.149 | -0.392 | 0.095 | 0.232 |
| [Clostridium] glycyrrhizinilyticum | 0 | Not detected | -0.043 | -0.128 | 0.041 | 0.316 |
| [Clostridium] hiranonis | 0 | Not detected | 0.022 | -0.200 | 0.243 | 0.847 |
| [Clostridium] hylemonae | 0 | Not detected | -0.158 | -0.333 | 0.017 | 0.076 |
| [Clostridium] innocuum | 0 | Not detected | -0.042 | -0.126 | 0.042 | 0.329 |
| [Clostridium] josui | 0 | Not detected | 0.028 | -0.166 | 0.223 | 0.776 |
| [Clostridium] lactatifermentans | 0 | Not detected | -0.019 | -0.127 | 0.089 | 0.734 |
| [Clostridium] leptum | 0 | Not detected | 0.004 | -0.138 | 0.146 | 0.953 |
| [Clostridium] methylpentosum | 0 | Not detected | -0.007 | -0.145 | 0.130 | 0.916 |
| [Clostridium] papyrosolvens | 0 | Not detected | 0.025 | -0.103 | 0.153 | 0.699 |
| [Clostridium] saccharogumia | 0 | Not detected | -0.071 | -0.196 | 0.055 | 0.271 |
| [Clostridium] saccharolyticum | 0 | Not detected | 0.058 | -0.057 | 0.173 | 0.322 |
| [Clostridium] scindens | 0 | Not detected | -0.047 | -0.150 | 0.057 | 0.377 |
| [Clostridium] spiroforme | 0 | Not detected | 0.040 | -0.185 | 0.265 | 0.727 |
| [Clostridium] sporosphaeroides | 0 | Not detected | 0.094 | -0.078 | 0.265 | 0.285 |
| [Clostridium] symbiosum | 0 | Not detected | -0.017 | -0.121 | 0.087 | 0.743 |
| [Clostridium] viride | 0 | Not detected | 0.113 | -0.046 | 0.272 | 0.165 |
| [Enterobacter] aerogenes | 0 | Not detected | 0.249 | -0.119 | 0.617 | 0.184 |
| [Eubacterium] brachy | 0 | Not detected | 0.000 | -0.232 | 0.233 | 0.999 |
| [Eubacterium] cellulosolvens | 0 | Not detected | -0.032 | -0.140 | 0.076 | 0.565 |
| [Eubacterium] contortum | 0 | Not detected | -0.053 | -0.129 | 0.023 | 0.174 |
| [Eubacterium] dolichum | 0 | Not detected | -0.053 | -0.132 | 0.027 | 0.194 |
| [Eubacterium] eligens | 0 | Not detected | -0.053 | -0.259 | 0.152 | 0.611 |
| [Eubacterium] fissicatena | 0 | Not detected | -0.025 | -0.128 | 0.079 | 0.642 |
| [Eubacterium] hallii | 0 | Not detected | -0.044 | -0.183 | 0.095 | 0.538 |
| [Eubacterium] nodatum | 0 | Not detected | -0.086 | -0.294 | 0.122 | 0.418 |
| [Eubacterium] rectale | 0 | Not detected | 0.020 | -0.176 | 0.216 | 0.844 |
| [Eubacterium] siraeum | 0 | Not detected | 0.165 | -0.139 | 0.469 | 0.288 |
| [Eubacterium] sulci | 0 | Not detected | -0.161 | -0.387 | 0.064 | 0.162 |
| [Eubacterium] yurii | 0 | Not detected | 0.035 | -0.094 | 0.164 | 0.595 |
| [Haemophilus] ducreyi | 0 | Not detected | -0.027 | -0.193 | 0.140 | 0.753 |
| [Haemophilus] parasuis | 0 | Not detected | -0.120 | -0.321 | 0.081 | 0.241 |
| [Ruminococcus] gnavus | 0 | Not detected | -0.160 | -0.317 | -0.002 | 0.047 |
| [Ruminococcus] torques | 0 | Not detected | 0.028 | -0.139 | 0.196 | 0.739 |
| bacterium F082 | 0 | Not detected | -0.010 | -0.135 | 0.115 | 0.874 |

| bacterium LF-3 | 0 | Not detected | 0.024 | -0.182 | 0.229 | 0.822 |
| --- | --- | --- | --- | --- | --- | --- |
| bacterium MS4 | 0 | Not detected | 0.059 | -0.107 | 0.225 | 0.487 |
| bacterium OL-1 | 0 | Not detected | 0.116 | -0.171 | 0.404 | 0.429 |
| bacterium P201 | 0 | Not detected | -0.034 | -0.235 | 0.167 | 0.743 |
| bacterium P3 | 0 | Not detected | -0.069 | -0.276 | 0.138 | 0.512 |

| **Supplementary Table 13.** Association of current economic hardship with functional modules. Analysis of Compositions of Microbiomes (ANCOM2) was used to identify functional modules related to current economic hardship . Regression coefficients were obtained by modelling the effect of current economic hardship on centered log ratio (CLR)-transformed species in multivariable linear regression adjusted for age, gender, BMI, field center, Hispanic/Latino background, U.S. nativity, Bristol stool type, parental education, the alternative Healthy Eating Index 2010 score, cigarette smoking, alcohol use, depressive and anxiety symptoms, diabetes, hypertension, cardiovascular disease, dyslipidemia, and antibiotic use. | | | | | | | |
| --- | --- | --- | --- | --- | --- | --- | --- |
| **otu.names** | **pathname** | **W_stat** | **ANCOM** | **beta** | **l95ci** | **u95ci** | **qval** |
| ADENOSYL | L-methionine salvage from L-hom | 0 | Not detected | -0.051 | -0.147 | 0.045 | 0.295 |
| ALANINE.V | L-alanine biosynthesis I | 0 | Not detected | 0.044 | -0.169 | 0.257 | 0.685 |
| ANAGLYCO | glycolysis III (from glucose) | 0 | Not detected | -0.047 | -0.133 | 0.038 | 0.281 |
| APYRASE.R | NA | 0 | Not detected | -0.052 | -0.143 | 0.040 | 0.268 |
| ARABCAT.P | L-arabinose degradation I | 0 | Not detected | -0.058 | -0.149 | 0.033 | 0.211 |
| ARGDEG.III | L-arginine degradation IV (arginin | 0 | Not detected | -0.088 | -0.227 | 0.051 | 0.215 |
| ARGSYN.PW | L-arginine biosynthesis I (via L-or | 0 | Not detected | -0.073 | -0.208 | 0.062 | 0.292 |
| ARGSYNBS | L-arginine biosynthesis II (acetyl c | 0 | Not detected | -0.054 | -0.188 | 0.081 | 0.435 |
| AST.PWY | L-arginine degradation II (AST pa | 0 | Not detected | 0.128 | -0.130 | 0.386 | 0.332 |
| BETSYN.PW | glycine betaine biosynthesis I (Gra | 0 | Not detected | 0.150 | -0.086 | 0.386 | 0.212 |
| CALVIN.PW | Calvin-Benson-Bassham cycle | 0 | Not detected | -0.042 | -0.125 | 0.040 | 0.316 |
| CARBPSYN. | NA | 0 | Not detected | -0.050 | -0.233 | 0.132 | 0.590 |
| CARNMET.P | L-carnitine degradation I | 0 | Not detected | 0.224 | -0.042 | 0.489 | 0.099 |
| CENTFERM. | pyruvate fermentation to butanoate | 0 | Not detected | -0.016 | -0.135 | 0.102 | 0.790 |
| CHLOROPH | 3,8-divinyl-chlorophyllide <i>a</i | 0 | Not detected | 0.028 | -0.228 | 0.283 | 0.831 |
| CHOLINE.B | choline degradation I | 0 | Not detected | 0.150 | -0.086 | 0.386 | 0.212 |
| COBALSYN. | superpathway of adenosylcobalam | 0 | Not detected | 0.109 | -0.088 | 0.307 | 0.277 |
| CODH.PWY | reductive acetyl coenzyme A path | 0 | Not detected | -0.071 | -0.222 | 0.081 | 0.361 |
| COLANSYN | colanic acid building blocks biosy | 0 | Not detected | -0.028 | -0.193 | 0.138 | 0.744 |
| DAPLYSINE | L-lysine biosynthesis I | 0 | Not detected | 0.117 | -0.138 | 0.372 | 0.368 |
| DARABCAT | D-arabinose degradation I | 0 | Not detected | 0.186 | -0.086 | 0.459 | 0.180 |
| DENITRIFIC | nitrate reduction I (denitrification) | 0 | Not detected | 0.153 | -0.081 | 0.388 | 0.200 |
| DETOX1.PW | superoxide radicals degradation | 0 | Not detected | 0.024 | -0.226 | 0.273 | 0.852 |
| DETOX1.PW | reactive oxygen species degradatio | 0 | Not detected | 0.030 | -0.227 | 0.288 | 0.817 |
| DHGLUCON | glucose degradation (oxidative) | 0 | Not detected | 0.274 | 0.018 | 0.530 | 0.036 |
| DISSULFRE | dissimilatory sulfate reduction I (t | 0 | Not detected | -0.054 | -0.298 | 0.190 | 0.663 |
| DTDPRHAM | dTDP-L-rhamnose biosynthesis | 0 | Not detected | -0.065 | -0.154 | 0.025 | 0.158 |
| ECASYN.PW | enterobacterial common antigen bi | 0 | Not detected | 0.132 | -0.141 | 0.404 | 0.344 |
| ETOH.ACET | ethanol degradation I | 0 | Not detected | -0.119 | -0.403 | 0.165 | 0.412 |
| FAO.PWY | fatty acid &beta;-oxidation I | 0 | Not detected | 0.257 | -0.075 | 0.589 | 0.130 |
| FASYN.ELO | fatty acid elongation -- saturated | 0 | Not detected | -0.078 | -0.359 | 0.202 | 0.584 |
| FERMENTA | mixed acid fermentation | 0 | Not detected | -0.056 | -0.141 | 0.029 | 0.197 |
| FHLMULTI. | NA | 0 | Not detected | -0.015 | -0.275 | 0.244 | 0.907 |
| FUCCAT.PW | fucose degradation | 0 | Not detected | 0.186 | -0.086 | 0.459 | 0.180 |
| GALACTAR | D-galactarate degradation I | 0 | Not detected | -0.019 | -0.187 | 0.149 | 0.822 |
| GALACTCA | D-galactonate degradation | 0 | Not detected | 0.071 | -0.199 | 0.341 | 0.606 |
| GALACTUR | D-galacturonate degradation I | 0 | Not detected | -0.053 | -0.140 | 0.034 | 0.237 |

| GCVMULTI. | NA | 0 | Not detected | 0.052 | -0.156 | 0.261 | 0.622 |
| --- | --- | --- | --- | --- | --- | --- | --- |
| GDPRHAMS | GDP-D-rhamnose biosynthesis | 0 | Not detected | -0.220 | -0.494 | 0.053 | 0.115 |
| GLUCARDE | <i>D</i>-glucarate degradation I | 0 | Not detected | -0.044 | -0.216 | 0.128 | 0.616 |
| GLUCONEO | gluconeogenesis I | 0 | Not detected | -0.049 | -0.136 | 0.037 | 0.264 |
| GLUCOSE1P | glucose and glucose-1-phosphate | 0 | Not detected | -0.054 | -0.184 | 0.076 | 0.419 |
| GLUDEG.I.P | GABA shunt | 0 | Not detected | 0.209 | -0.028 | 0.446 | 0.085 |
| GLUTATHIO | glutathione biosynthesis | 0 | Not detected | 0.046 | -0.179 | 0.270 | 0.690 |
| GLUTORN.P | L-ornithine biosynthesis I | 0 | Not detected | 0.083 | -0.166 | 0.332 | 0.514 |
| GLYCLEAV. | glycine cleavage | 0 | Not detected | 0.052 | -0.156 | 0.261 | 0.622 |
| GLYCOCAT | glycogen degradation I | 0 | Not detected | -0.068 | -0.200 | 0.063 | 0.308 |
| GLYCOGEN | glycogen biosynthesis I (from AD | 0 | Not detected | -0.066 | -0.183 | 0.051 | 0.268 |
| GLYCOLAT | glycolate and glyoxylate degradati | 0 | Not detected | 0.197 | -0.060 | 0.454 | 0.133 |
| GLYCOLYSI | glycolysis I (from glucose 6-phosp | 0 | Not detected | -0.047 | -0.133 | 0.039 | 0.282 |
| GLYCOLYSI | superpathway of glycolysis and th | 0 | Not detected | 0.183 | -0.051 | 0.417 | 0.126 |
| GLYOXDEG | glycolate and glyoxylate degradati | 0 | Not detected | 0.104 | -0.161 | 0.369 | 0.443 |
| GLYOXYLA | glyoxylate cycle | 0 | Not detected | -0.066 | -0.161 | 0.029 | 0.173 |
| HCAMHPDE | 3-phenylpropanoate and 3-(3-hydr | 0 | Not detected | 0.239 | 0.010 | 0.468 | 0.041 |
| HEME.BIOS | heme <i>b</i> biosynthesis I (aer | 0 | Not detected | 0.000 | -0.259 | 0.260 | 0.997 |
| HEMESYN2. | heme <i>b</i> biosynthesis II (an | 0 | Not detected | -0.020 | -0.287 | 0.248 | 0.886 |
| HISDEG.PW | L-histidine degradation I | 0 | Not detected | 0.128 | -0.096 | 0.351 | 0.263 |
| HISHP.PWY | L-histidine degradation VI | 0 | Not detected | 0.128 | -0.096 | 0.351 | 0.263 |
| HISTSYN.P | L-histidine biosynthesis | 0 | Not detected | -0.049 | -0.141 | 0.043 | 0.295 |
| HOMOSER. | L-methionine biosynthesis I | 0 | Not detected | -0.049 | -0.135 | 0.037 | 0.265 |
| HOMOSER.T | L-threonine biosynthesis | 0 | Not detected | 0.191 | -0.070 | 0.452 | 0.152 |
| HOMOSERS | L-homoserine biosynthesis | 0 | Not detected | 0.154 | -0.096 | 0.404 | 0.226 |
| HSERMETA | L-methionine biosynthesis III | 0 | Not detected | -0.046 | -0.135 | 0.043 | 0.310 |
| ILEUSYN.P | L-isoleucine biosynthesis I (from t | 0 | Not detected | -0.065 | -0.175 | 0.045 | 0.248 |
| KDO.LIPAS | (Kdo)<sub>2</sub>-lipid A biosy | 0 | Not detected | 0.015 | -0.212 | 0.242 | 0.894 |
| KDO.NAGLI | superpathway of (Kdo)<SUB>2</ | 0 | Not detected | -0.045 | -0.304 | 0.214 | 0.734 |
| KDOSYN.PW | Kdo transfer to lipid IV<sub>A</s | 0 | Not detected | 0.039 | -0.228 | 0.306 | 0.776 |
| KETOGLUC | ketogluconate metabolism | 0 | Not detected | 0.246 | 0.023 | 0.469 | 0.031 |
| LACTOSEC | lactose and galactose degradation | 0 | Not detected | -0.004 | -0.274 | 0.266 | 0.978 |
| LARABITOL | xylitol degradation | 0 | Not detected | 0.156 | -0.119 | 0.430 | 0.266 |
| LEUSYN.PW | L-leucine biosynthesis | 0 | Not detected | 0.054 | -0.100 | 0.209 | 0.492 |
| LIPA.CORES | lipid A-core biosynthesis (<i>E. c | 0 | Not detected | -0.023 | -0.312 | 0.266 | 0.876 |
| LYSDEGII.P | L-lysine degradation III | 0 | Not detected | 0.152 | -0.047 | 0.351 | 0.136 |
| LYXMET.PW | L-lyxose degradation | 0 | Not detected | -0.049 | -0.128 | 0.031 | 0.229 |
| MALATE.AS | L-aspartate degradation II | 0 | Not detected | 0.236 | -0.048 | 0.519 | 0.103 |
| MANNCAT. | D-mannose degradation | 0 | Not detected | 0.139 | -0.093 | 0.371 | 0.242 |
| MANNIDEG | mannitol degradation I | 0 | Not detected | 0.082 | -0.168 | 0.331 | 0.523 |
| MENAQUIN | menaquinol-8 biosynthesis | 0 | Not detected | 0.047 | -0.206 | 0.301 | 0.715 |
| METH.ACET | methanogenesis from acetate | 0 | Not detected | -0.039 | -0.118 | 0.040 | 0.337 |
| NAGLIPASY | lipid IV<sub>A</sub> biosynthes | 0 | Not detected | 0.153 | -0.113 | 0.420 | 0.260 |
| NONMEVIP | methylerythritol phosphate pathwa | 0 | Not detected | -0.087 | -0.186 | 0.011 | 0.081 |

| NONOXIPE | pentose phosphate pathway (non-o | 0 | Not detected | -0.029 | -0.200 | 0.142 | 0.740 |
| --- | --- | --- | --- | --- | --- | --- | --- |
| NPGLUCAT. | Entner-Doudoroff pathway II (non | 0 | Not detected | 0.049 | -0.169 | 0.267 | 0.660 |
| OANTIGEN. | <i>O</i>-antigen building blocks | 0 | Not detected | -0.057 | -0.167 | 0.054 | 0.316 |
| OXIDATIVE | pentose phosphate pathway (oxida | 0 | Not detected | -0.038 | -0.211 | 0.135 | 0.664 |
| P101.PWY | ectoine biosynthesis | 0 | Not detected | 0.153 | -0.097 | 0.403 | 0.231 |
| P105.PWY | TCA cycle IV (2-oxoglutarate dec | 0 | Not detected | -0.071 | -0.161 | 0.019 | 0.125 |
| P108.PWY | pyruvate fermentation to propanoa | 0 | Not detected | 0.006 | -0.112 | 0.124 | 0.921 |
| P121.PWY | adenine and adenosine salvage I | 0 | Not detected | 0.108 | -0.129 | 0.345 | 0.370 |
| P122.PWY | heterolactic fermentation | 0 | Not detected | -0.044 | -0.123 | 0.034 | 0.270 |
| P124.PWY | Bifidobacterium shunt | 0 | Not detected | -0.045 | -0.123 | 0.033 | 0.263 |
| P161.PWY | acetylene degradation (anaerobic) | 0 | Not detected | -0.035 | -0.107 | 0.038 | 0.346 |
| P162.PWY | L-glutamate degradation V (via hy | 0 | Not detected | -0.016 | -0.135 | 0.102 | 0.790 |
| P163.PWY | L-lysine fermentation to acetate an | 0 | Not detected | -0.025 | -0.108 | 0.059 | 0.565 |
| P164.PWY | purine nucleobases degradation I ( | 0 | Not detected | -0.027 | -0.112 | 0.058 | 0.527 |
| P181.PWY | nicotine degradation I (pyridine pa | 0 | Not detected | 0.187 | -0.038 | 0.413 | 0.104 |
| P185.PWY | formaldehyde assimilation III (dih | 0 | Not detected | -0.039 | -0.121 | 0.043 | 0.352 |
| P2.PWY | citrate lyase activation | 0 | Not detected | 0.026 | -0.205 | 0.258 | 0.824 |
| P21.PWY | pentose phosphate pathway (partia | 0 | Not detected | -0.029 | -0.200 | 0.142 | 0.740 |
| P23.PWY | reductive TCA cycle I | 0 | Not detected | -0.078 | -0.180 | 0.025 | 0.137 |
| P3.PWY | gallate degradation III (anaerobic) | 0 | Not detected | -0.027 | -0.110 | 0.056 | 0.529 |
| P341.PWY | glycolysis V (Pyrococcus) | 0 | Not detected | -0.053 | -0.143 | 0.036 | 0.245 |
| P42.PWY | incomplete reductive TCA cycle | 0 | Not detected | -0.030 | -0.152 | 0.091 | 0.628 |
| P562.PWY | <I>myo</I>-inositol degradation I | 0 | Not detected | 0.069 | -0.104 | 0.241 | 0.435 |
| PANTO.PWY | phosphopantothenate biosynthesis | 0 | Not detected | 0.137 | -0.051 | 0.326 | 0.154 |
| PEPTIDOGL | peptidoglycan biosynthesis I (<I> | 0 | Not detected | 0.160 | -0.089 | 0.410 | 0.208 |
| PHOSLIPSY | superpathway of phospholipid bio | 0 | Not detected | -0.125 | -0.361 | 0.111 | 0.300 |
| PHOSPHON | 2-aminoethylphosphonate degrada | 0 | Not detected | -0.099 | -0.369 | 0.171 | 0.473 |
| PPGPPMET. | ppGpp biosynthesis | 0 | Not detected | -0.034 | -0.116 | 0.047 | 0.410 |
| PUTDEG.PW | putrescine degradation I | 0 | Not detected | 0.228 | 0.026 | 0.429 | 0.027 |
| PWY.1042 | glycolysis IV (plant cytosol) | 0 | Not detected | -0.043 | -0.128 | 0.042 | 0.322 |
| PWY.1186 | L-homomethionine biosynthesis | 0 | Not detected | -0.042 | -0.288 | 0.203 | 0.735 |
| PWY.1269 | CMP-3-deoxy-D-<I>manno</I>-o | 0 | Not detected | 0.095 | -0.161 | 0.350 | 0.467 |
| PWY.1281 | sulfoacetaldehyde degradation I | 0 | Not detected | -0.060 | -0.162 | 0.042 | 0.249 |
| PWY.1341 | phenylacetate degradation II (anae | 0 | Not detected | 0.011 | -0.135 | 0.158 | 0.879 |
| PWY.1361 | benzoyl-CoA degradation I (aerob | 0 | Not detected | 0.305 | 0.067 | 0.543 | 0.012 |
| PWY.1622 | formaldehyde assimilation I (serin | 0 | Not detected | 0.107 | -0.072 | 0.286 | 0.241 |
| PWY.1722 | formate assimilation into 5,10-met | 0 | Not detected | -0.071 | -0.222 | 0.081 | 0.361 |
| PWY.1861 | formaldehyde assimilation II (assi | 0 | Not detected | -0.032 | -0.133 | 0.069 | 0.538 |
| PWY.2 | putrescine degradation IV | 0 | Not detected | 0.228 | 0.026 | 0.429 | 0.027 |
| PWY.2161 | folate polyglutamylation | 0 | Not detected | 0.012 | -0.259 | 0.282 | 0.931 |
| PWY.2201 | folate transformations I | 0 | Not detected | -0.047 | -0.139 | 0.044 | 0.313 |
| PWY.2221 | Entner-Doudoroff pathway III (se | 0 | Not detected | -0.052 | -0.135 | 0.031 | 0.217 |
| PWY.2361 | 3-oxoadipate degradation | 0 | Not detected | 0.194 | -0.004 | 0.393 | 0.055 |
| PWY.2661 | trehalose biosynthesis V | 0 | Not detected | -0.032 | -0.316 | 0.251 | 0.823 |

| PWY.2722 | trehalose degradation IV | 0 | Not detected | 0.095 | -0.125 | 0.316 | 0.398 |
| --- | --- | --- | --- | --- | --- | --- | --- |
| PWY.2723 | trehalose degradation V | 0 | Not detected | -0.105 | -0.247 | 0.036 | 0.145 |
| PWY.2781 | <i>cis</i>-zeatin biosynthesis | 0 | Not detected | 0.006 | -0.231 | 0.243 | 0.961 |
| PWY.2941 | L-lysine biosynthesis II | 0 | Not detected | 0.154 | -0.096 | 0.404 | 0.226 |
| PWY.2942 | L-lysine biosynthesis III | 0 | Not detected | 0.154 | -0.096 | 0.404 | 0.226 |
| PWY.3 | putrescine degradation V | 0 | Not detected | 0.238 | 0.035 | 0.441 | 0.022 |
| PWY.3801 | sucrose degradation II (sucrose sy | 0 | Not detected | -0.053 | -0.133 | 0.027 | 0.196 |
| PWY.3821 | D-galactose detoxification | 0 | Not detected | -0.034 | -0.166 | 0.099 | 0.621 |
| PWY.3841 | folate transformations II | 0 | Not detected | -0.045 | -0.135 | 0.045 | 0.325 |
| PWY.3861 | mannitol degradation II | 0 | Not detected | 0.183 | -0.065 | 0.432 | 0.148 |
| PWY.3881 | mannitol biosynthesis | 0 | Not detected | 0.112 | -0.137 | 0.361 | 0.378 |
| PWY.3941 | &beta;-alanine biosynthesis II | 0 | Not detected | 0.422 | 0.154 | 0.689 | 0.002 |
| PWY.4041 | &gamma;-glutamyl cycle | 0 | Not detected | 0.072 | -0.190 | 0.334 | 0.589 |
| PWY.4101 | D-sorbitol degradation I | 0 | Not detected | 0.209 | -0.042 | 0.460 | 0.103 |
| PWY.43 | putrescine biosynthesis II | 0 | Not detected | -0.088 | -0.227 | 0.051 | 0.215 |
| PWY.4321 | L-glutamate degradation IV | 0 | Not detected | 0.222 | -0.014 | 0.458 | 0.066 |
| PWY.4361 | <i>S</i>-methyl-5-thio-&alpha;-D | 0 | Not detected | 0.081 | -0.175 | 0.337 | 0.538 |
| PWY.4381 | fatty acid biosynthesis initiation I | 0 | Not detected | -0.017 | -0.258 | 0.223 | 0.887 |
| PWY.4702 | phytate degradation I | 0 | Not detected | 0.079 | -0.176 | 0.333 | 0.545 |
| PWY.4983 | nitric oxide biosynthesis II (mamm | 0 | Not detected | -0.073 | -0.208 | 0.062 | 0.291 |
| PWY.4984 | urea cycle | 0 | Not detected | -0.093 | -0.212 | 0.025 | 0.124 |
| PWY.5022 | 4-aminobutanoate degradation V | 0 | Not detected | -0.022 | -0.132 | 0.088 | 0.695 |
| PWY.5028 | L-histidine degradation II | 0 | Not detected | 0.139 | -0.085 | 0.363 | 0.225 |
| PWY.5030 | L-histidine degradation III | 0 | Not detected | -0.062 | -0.205 | 0.081 | 0.395 |
| PWY.5041 | <i>S</i>-adenosyl-L-methionine c | 0 | Not detected | -0.042 | -0.305 | 0.222 | 0.757 |
| PWY.5049 | rosmarinic acid biosynthesis II | 0 | Not detected | -0.043 | -0.239 | 0.154 | 0.672 |
| PWY.5054 | D-sorbitol biosynthesis I | 0 | Not detected | -0.055 | -0.146 | 0.037 | 0.241 |
| PWY.5097 | L-lysine biosynthesis VI | 0 | Not detected | 0.151 | -0.099 | 0.402 | 0.237 |
| PWY.5101 | L-isoleucine biosynthesis II | 0 | Not detected | -0.141 | -0.387 | 0.105 | 0.262 |
| PWY.5103 | L-isoleucine biosynthesis III | 0 | Not detected | -0.065 | -0.175 | 0.045 | 0.248 |
| PWY.5104 | L-isoleucine biosynthesis IV | 0 | Not detected | 0.245 | -0.022 | 0.513 | 0.073 |
| PWY.5122 | geranyl diphosphate biosynthesis | 0 | Not detected | 0.032 | -0.194 | 0.259 | 0.780 |
| PWY.5123 | <i>trans, trans</i>-farnesyl diphos | 0 | Not detected | -0.003 | -0.274 | 0.268 | 0.984 |
| PWY.5136 | fatty acid &beta;-oxidation II (per | 0 | Not detected | 0.232 | -0.073 | 0.537 | 0.136 |
| PWY.5137 | fatty acid &beta;-oxidation III (un | 0 | Not detected | 0.198 | -0.040 | 0.437 | 0.104 |
| PWY.5138 | unsaturated, even numbered fatty a | 0 | Not detected | 0.233 | -0.045 | 0.511 | 0.101 |
| PWY.5154 | L-arginine biosynthesis III (via <i | 0 | Not detected | -0.054 | -0.188 | 0.081 | 0.434 |
| PWY.5162 | 2-hydroxypenta-2,4-dienoate degr | 0 | Not detected | 0.035 | -0.234 | 0.305 | 0.797 |
| PWY.5177 | glutaryl-CoA degradation | 0 | Not detected | 0.010 | -0.145 | 0.164 | 0.900 |
| PWY.5188 | tetrapyrrole biosynthesis I (from g | 0 | Not detected | -0.012 | -0.133 | 0.109 | 0.846 |
| PWY.5189 | tetrapyrrole biosynthesis II (from g | 0 | Not detected | -0.006 | -0.182 | 0.170 | 0.949 |
| PWY.5265 | peptidoglycan biosynthesis II (stap | 0 | Not detected | -0.090 | -0.243 | 0.062 | 0.247 |
| PWY.5269 | cardiolipin biosynthesis II | 0 | Not detected | 0.150 | -0.086 | 0.387 | 0.213 |
| PWY.5280 | L-lysine degradation IV | 0 | Not detected | 0.172 | -0.031 | 0.375 | 0.097 |

| PWY.5306 | superpathway of thiosulfate metab | 0 | Not detected | -0.054 | -0.298 | 0.190 | 0.663 |
| --- | --- | --- | --- | --- | --- | --- | --- |
| PWY.5316 | nicotine biosynthesis | 0 | Not detected | 0.126 | -0.172 | 0.425 | 0.407 |
| PWY.5344 | L-homocysteine biosynthesis | 0 | Not detected | -0.060 | -0.160 | 0.039 | 0.234 |
| PWY.5352 | thiosulfate disproportionation II (c | 0 | Not detected | -0.055 | -0.287 | 0.176 | 0.641 |
| PWY.5360 | superpathway of tetrathionate redu | 0 | Not detected | -0.054 | -0.298 | 0.190 | 0.663 |
| PWY.5381 | pyridine nucleotide cycling (plants | 0 | Not detected | -0.084 | -0.334 | 0.166 | 0.511 |
| PWY.5384 | sucrose degradation IV (sucrose p | 0 | Not detected | -0.051 | -0.132 | 0.029 | 0.211 |
| PWY.5386 | methylglyoxal degradation I | 0 | Not detected | 0.079 | -0.178 | 0.335 | 0.548 |
| PWY.5392 | reductive TCA cycle II | 0 | Not detected | -0.080 | -0.184 | 0.025 | 0.137 |
| PWY.5394 | betalamic acid biosynthesis | 0 | Not detected | 0.059 | -0.122 | 0.240 | 0.523 |
| PWY.5436 | L-threonine degradation IV | 0 | Not detected | -0.099 | -0.369 | 0.171 | 0.473 |
| PWY.5437 | L-threonine degradation I | 0 | Not detected | -0.111 | -0.377 | 0.155 | 0.414 |
| PWY.5441 | S-methyl-L-methionine cycle | 0 | Not detected | -0.037 | -0.231 | 0.157 | 0.708 |
| PWY.5453 | methylglyoxal degradation III | 0 | Not detected | 0.004 | -0.079 | 0.087 | 0.919 |
| PWY.5458 | methylglyoxal degradation V | 0 | Not detected | -0.192 | -0.440 | 0.056 | 0.129 |
| PWY.5459 | methylglyoxal degradation IV | 0 | Not detected | -0.202 | -0.450 | 0.047 | 0.112 |
| PWY.5480 | pyruvate fermentation to ethanol I | 0 | Not detected | -0.119 | -0.403 | 0.165 | 0.412 |
| PWY.5482 | pyruvate fermentation to acetate II | 0 | Not detected | -0.039 | -0.118 | 0.040 | 0.337 |
| PWY.5484 | glycolysis II (from fructose 6-phos | 0 | Not detected | -0.042 | -0.125 | 0.042 | 0.327 |
| PWY.5485 | pyruvate fermentation to acetate I | 0 | Not detected | -0.039 | -0.118 | 0.040 | 0.337 |
| PWY.5486 | pyruvate fermentation to ethanol I | 0 | Not detected | -0.202 | -0.428 | 0.025 | 0.081 |
| PWY.5497 | purine nucleobases degradation II | 0 | Not detected | -0.037 | -0.121 | 0.047 | 0.382 |
| PWY.5509 | adenosylcobalamin biosynthesis fr | 0 | Not detected | 0.109 | -0.088 | 0.307 | 0.277 |
| PWY.5512 | UDP-<i>N</i>-acetyl-D-galactosa | 0 | Not detected | 0.054 | -0.268 | 0.376 | 0.743 |
| PWY.5514 | UDP-<i>N</i>-acetyl-D-galactosa | 0 | Not detected | -0.053 | -0.147 | 0.041 | 0.267 |
| PWY.5523 | 5,6-dimethylbenzimidazole biosyn | 0 | Not detected | 0.123 | -0.109 | 0.356 | 0.299 |
| PWY.5531 | 3,8-divinyl-chlorophyllide <i>a</i | 0 | Not detected | 0.000 | -0.265 | 0.264 | 0.999 |
| PWY.5532 | nucleoside and nucleotide degrada | 0 | Not detected | 0.118 | -0.076 | 0.312 | 0.235 |
| PWY.5536 | acetate formation from acetyl-CoA | 0 | Not detected | -0.030 | -0.203 | 0.143 | 0.732 |
| PWY.561 | superpathway of glyoxylate cycle | 0 | Not detected | -0.051 | -0.140 | 0.039 | 0.265 |
| PWY.5659 | GDP-mannose biosynthesis | 0 | Not detected | -0.048 | -0.132 | 0.036 | 0.261 |
| PWY.5661 | GDP-glucose biosynthesis | 0 | Not detected | -0.105 | -0.247 | 0.036 | 0.145 |
| PWY.5663 | erythro-tetrahydrobiopterin biosyn | 0 | Not detected | -0.083 | -0.241 | 0.075 | 0.305 |
| PWY.5664 | <i>erythro</i>-tetrahydrobiopterin | 0 | Not detected | -0.083 | -0.241 | 0.075 | 0.305 |
| PWY.5667 | CDP-diacylglycerol biosynthesis I | 0 | Not detected | -0.066 | -0.273 | 0.141 | 0.531 |
| PWY.5668 | cardiolipin biosynthesis I | 0 | Not detected | 0.208 | -0.067 | 0.483 | 0.138 |
| PWY.5669 | phosphatidylserine and phosphatid | 0 | Not detected | -0.125 | -0.361 | 0.111 | 0.300 |
| PWY.5676 | acetyl-CoA fermentation to butano | 0 | Not detected | -0.004 | -0.112 | 0.104 | 0.941 |
| PWY.5677 | succinate fermentation to butanoat | 0 | Not detected | -0.031 | -0.146 | 0.084 | 0.597 |
| PWY.5686 | UMP biosynthesis I | 0 | Not detected | -0.040 | -0.131 | 0.052 | 0.399 |
| PWY.5690 | TCA cycle II (plants and fungi) | 0 | Not detected | -0.077 | -0.181 | 0.028 | 0.151 |
| PWY.5692 | allantoin degradation to glyoxylate | 0 | Not detected | -0.098 | -0.322 | 0.127 | 0.393 |
| PWY.5698 | allantoin degradation to ureidogly | 0 | Not detected | -0.098 | -0.322 | 0.127 | 0.393 |
| PWY.5723 | Rubisco shunt | 0 | Not detected | -0.050 | -0.144 | 0.043 | 0.292 |

| PWY.5738 | GDP-6-deoxy-D-talose biosynthes | 0 | Not detected | -0.220 | -0.494 | 0.053 | 0.115 |
| --- | --- | --- | --- | --- | --- | --- | --- |
| PWY.5739 | GDP-D-perosamine biosynthesis | 0 | Not detected | -0.220 | -0.494 | 0.053 | 0.115 |
| PWY.5740 | GDP-L-colitose biosynthesis | 0 | Not detected | -0.220 | -0.494 | 0.053 | 0.115 |
| PWY.5741 | ethylmalonyl-CoA pathway | 0 | Not detected | 0.169 | -0.050 | 0.389 | 0.130 |
| PWY.5743 | 3-hydroxypropanoate cycle | 0 | Not detected | -0.106 | -0.226 | 0.013 | 0.082 |
| PWY.5744 | glyoxylate assimilation | 0 | Not detected | -0.105 | -0.225 | 0.015 | 0.086 |
| PWY.5747 | 2-methylcitrate cycle II | 0 | Not detected | 0.416 | 0.129 | 0.702 | 0.005 |
| PWY.5757 | fosfomycin biosynthesis | 0 | Not detected | -0.275 | -0.607 | 0.058 | 0.106 |
| PWY.5785 | di-<i>trans</i>,poly-<i>cis</i>-un | 0 | Not detected | -0.001 | -0.230 | 0.228 | 0.990 |
| PWY.5789 | 3-hydroxypropanoate/4-hydroxybu | 0 | Not detected | -0.031 | -0.134 | 0.071 | 0.549 |
| PWY.5794 | malonate degradation I (biotin-ind | 0 | Not detected | -0.039 | -0.281 | 0.203 | 0.751 |
| PWY.5796 | malonate decarboxylase activation | 0 | Not detected | -0.014 | -0.211 | 0.183 | 0.889 |
| PWY.5837 | 2-carboxy-1,4-naphthoquinol bios | 0 | Not detected | -0.080 | -0.176 | 0.016 | 0.103 |
| PWY.5852 | demethylmenaquinol-8 biosynthes | 0 | Not detected | 0.017 | -0.195 | 0.229 | 0.877 |
| PWY.5870 | ubiquinol-8 biosynthesis (eukaryo | 0 | Not detected | 0.060 | -0.216 | 0.336 | 0.670 |
| PWY.5875 | staphyloxanthin biosynthesis | 0 | Not detected | -0.063 | -0.336 | 0.209 | 0.648 |
| PWY.5901 | 2,3-dihydroxybenzoate biosynthes | 0 | Not detected | 0.147 | -0.051 | 0.345 | 0.146 |
| PWY.5913 | partial TCA cycle (obligate autotro | 0 | Not detected | -0.075 | -0.177 | 0.027 | 0.150 |
| PWY.5921 | glutaminyl-tRNA<sup>gln</sup> | 0 | Not detected | 0.039 | -0.243 | 0.321 | 0.785 |
| PWY.5940 | streptomycin biosynthesis | 0 | Not detected | -0.124 | -0.263 | 0.016 | 0.083 |
| PWY.5941 | glycogen degradation II | 0 | Not detected | -0.105 | -0.247 | 0.036 | 0.145 |
| PWY.5971 | palmitate biosynthesis II (bacteria | 0 | Not detected | -0.155 | -0.334 | 0.024 | 0.090 |
| PWY.5981 | CDP-diacylglycerol biosynthesis I | 0 | Not detected | -0.139 | -0.343 | 0.065 | 0.181 |
| PWY.5994 | palmitate biosynthesis I (animals a | 0 | Not detected | -0.154 | -0.347 | 0.039 | 0.119 |
| PWY.6012 | acyl carrier protein metabolism | 0 | Not detected | 0.023 | -0.169 | 0.215 | 0.816 |
| PWY.6019 | pseudouridine degradation | 0 | Not detected | 0.020 | -0.204 | 0.243 | 0.862 |
| PWY.6053 | dimethylsulfoniopropanoate biosy | 0 | Not detected | -0.042 | -0.288 | 0.203 | 0.735 |
| PWY.6060 | malonate degradation II (biotin-de | 0 | Not detected | 0.000 | -0.155 | 0.156 | 0.996 |
| PWY.6121 | 5-aminoimidazole ribonucleotide | 0 | Not detected | 0.064 | -0.041 | 0.168 | 0.232 |
| PWY.6122 | 5-aminoimidazole ribonucleotide | 0 | Not detected | 0.041 | -0.040 | 0.121 | 0.321 |
| PWY.6123 | inosine-5'-phosphate biosynthesis | 0 | Not detected | 0.112 | -0.047 | 0.270 | 0.168 |
| PWY.6124 | inosine-5'-phosphate biosynthesis | 0 | Not detected | 0.112 | -0.047 | 0.270 | 0.168 |
| PWY.6138 | CMP-<i>N</i>-acetylneuraminate | 0 | Not detected | 0.092 | -0.121 | 0.304 | 0.398 |
| PWY.6140 | CMP-2-keto-3-deoxy-D-<i>glycer | 0 | Not detected | -0.057 | -0.350 | 0.235 | 0.700 |
| PWY.6142 | gluconeogenesis II (<i>Methanob | 0 | Not detected | -0.054 | -0.144 | 0.036 | 0.237 |
| PWY.6143 | CMP-pseudaminate biosynthesis | 0 | Not detected | -0.127 | -0.339 | 0.085 | 0.239 |
| PWY.6146 | <i>Methanobacterium thermoauto | 0 | Not detected | -0.092 | -0.353 | 0.169 | 0.489 |
| PWY.6147 | 6-hydroxymethyl-dihydropterin di | 0 | Not detected | -0.036 | -0.170 | 0.097 | 0.594 |
| PWY.6148 | tetrahydromethanopterin biosynth | 271 | Not detected | 0.327 | 0.128 | 0.526 | 0.001 |
| PWY.6151 | <i>S</i>-adenosyl-L-methionine c | 0 | Not detected | 0.015 | -0.259 | 0.290 | 0.913 |
| PWY.6153 | autoinducer AI-2 biosynthesis I | 0 | Not detected | 0.092 | -0.108 | 0.293 | 0.367 |
| PWY.6154 | autoinducer AI-2 biosynthesis II ( | 0 | Not detected | 0.092 | -0.108 | 0.293 | 0.367 |
| PWY.6160 | 3-dehydroquinate biosynthesis II ( | 0 | Not detected | 0.166 | -0.085 | 0.417 | 0.195 |
| PWY.6163 | chorismate biosynthesis from 3-de | 0 | Not detected | -0.285 | -0.480 | -0.090 | 0.004 |

| PWY.6164 | 3-dehydroquinate biosynthesis I | 0 | Not detected | 0.126 | -0.117 | 0.369 | 0.310 |
| --- | --- | --- | --- | --- | --- | --- | --- |
| PWY.6167 | flavin biosynthesis II (archaea) | 0 | Not detected | -0.039 | -0.125 | 0.047 | 0.371 |
| PWY.6168 | flavin biosynthesis III (fungi) | 0 | Not detected | -0.042 | -0.131 | 0.048 | 0.364 |
| PWY.6174 | mevalonate pathway II (archaea) | 0 | Not detected | 0.022 | -0.159 | 0.203 | 0.815 |
| PWY.621 | sucrose degradation III (sucrose in | 0 | Not detected | -0.047 | -0.132 | 0.038 | 0.278 |
| PWY.622 | starch biosynthesis | 0 | Not detected | -0.054 | -0.133 | 0.026 | 0.185 |
| PWY.6269 | superpathway of adenosylcobalam | 0 | Not detected | 0.079 | -0.139 | 0.297 | 0.478 |
| PWY.6277 | superpathway of 5-aminoimidazol | 0 | Not detected | 0.041 | -0.040 | 0.121 | 0.321 |
| PWY.6317 | D-galactose degradation I (Leloir | 0 | Not detected | -0.051 | -0.136 | 0.033 | 0.234 |
| PWY.6318 | L-phenylalanine degradation IV ( | 0 | Not detected | 0.011 | -0.135 | 0.158 | 0.879 |
| PWY.6322 | phosphinothricin tripeptide biosyn | 0 | Not detected | -0.247 | -0.545 | 0.051 | 0.105 |
| PWY.6328 | L-lysine degradation X | 0 | Not detected | 0.172 | -0.031 | 0.375 | 0.097 |
| PWY.6333 | acetaldehyde biosynthesis I | 0 | Not detected | -0.202 | -0.428 | 0.025 | 0.081 |
| PWY.6383 | mono-<i>trans</i>, poly-<i>cis</i | 0 | Not detected | -0.006 | -0.246 | 0.234 | 0.964 |
| PWY.6386 | UDP-<i>N</i>-acetylmuramoyl-p | 0 | Not detected | -0.027 | -0.103 | 0.049 | 0.484 |
| PWY.6387 | UDP-<i>N</i>-acetylmuramoyl-p | 0 | Not detected | -0.022 | -0.094 | 0.050 | 0.542 |
| PWY.6397 | mycolyl-arabinogalactan-peptidog | 0 | Not detected | -0.054 | -0.164 | 0.056 | 0.333 |
| PWY.6406 | salicylate biosynthesis I | 0 | Not detected | 0.147 | -0.051 | 0.345 | 0.146 |
| PWY.6416 | quinate degradation II | 0 | Not detected | -0.243 | -0.483 | -0.002 | 0.049 |
| PWY.6461 | peptidoglycan cross-bridge biosyn | 0 | Not detected | -0.101 | -0.400 | 0.199 | 0.510 |
| PWY.6471 | peptidoglycan biosynthesis IV (En | 0 | Not detected | -0.086 | -0.239 | 0.067 | 0.271 |
| PWY.6473 | 4-aminobutanoate degradation IV | 0 | Not detected | 0.243 | 0.040 | 0.445 | 0.019 |
| PWY.6478 | GDP-D-<i>glycero</i>-&alpha;-D | 0 | Not detected | 0.075 | -0.165 | 0.315 | 0.540 |
| PWY.6497 | D-galactarate degradation II | 0 | Not detected | -0.087 | -0.329 | 0.155 | 0.481 |
| PWY.6499 | D-glucarate degradation II | 0 | Not detected | -0.184 | -0.424 | 0.055 | 0.131 |
| PWY.6507 | 4-deoxy-L-<i>threo</i>-hex-4-en | 0 | Not detected | -0.049 | -0.136 | 0.038 | 0.269 |
| PWY.6517 | <i>N</i>-acetylglucosamine degra | 0 | Not detected | -0.072 | -0.174 | 0.030 | 0.169 |
| PWY.6518 | glycocholate metabolism (bacteria | 0 | Not detected | -0.207 | -0.480 | 0.065 | 0.137 |
| PWY.6519 | 8-amino-7-oxononanoate biosynth | 0 | Not detected | 0.057 | -0.164 | 0.278 | 0.615 |
| PWY.6527 | stachyose degradation | 0 | Not detected | -0.019 | -0.118 | 0.081 | 0.713 |
| PWY.6531 | mannitol cycle | 0 | Not detected | 0.175 | -0.063 | 0.413 | 0.149 |
| PWY.6535 | 4-aminobutanoate degradation I | 0 | Not detected | 0.222 | -0.014 | 0.458 | 0.066 |
| PWY.6536 | 4-aminobutanoate degradation III | 0 | Not detected | 0.190 | -0.014 | 0.394 | 0.068 |
| PWY.6537 | 4-aminobutanoate degradation II | 0 | Not detected | 0.152 | -0.047 | 0.351 | 0.136 |
| PWY.6545 | pyrimidine deoxyribonucleotides < | 0 | Not detected | -0.077 | -0.183 | 0.029 | 0.155 |
| PWY.6549 | L-glutamine biosynthesis III | 0 | Not detected | -0.077 | -0.184 | 0.030 | 0.158 |
| PWY.6556 | pyrimidine ribonucleosides salvag | 0 | Not detected | -0.018 | -0.212 | 0.176 | 0.857 |
| PWY.6559 | spermidine biosynthesis II | 0 | Not detected | -0.049 | -0.151 | 0.054 | 0.352 |
| PWY.6562 | norspermidine biosynthesis | 0 | Not detected | 0.097 | -0.138 | 0.333 | 0.417 |
| PWY.6572 | chondroitin sulfate degradation I ( | 0 | Not detected | -0.097 | -0.367 | 0.174 | 0.484 |
| PWY.6575 | juvenile hormone III biosynthesis | 0 | Not detected | -0.061 | -0.266 | 0.144 | 0.560 |
| PWY.6583 | pyruvate fermentation to butanol I | 0 | Not detected | -0.047 | -0.159 | 0.065 | 0.410 |
| PWY.6587 | pyruvate fermentation to ethanol I | 0 | Not detected | -0.119 | -0.403 | 0.165 | 0.412 |
| PWY.6596 | adenosine nucleotides degradation | 0 | Not detected | 0.090 | -0.158 | 0.338 | 0.477 |

| PWY.66 | GDP-L-fucose biosynthesis I (from | 0 | Not detected | -0.220 | -0.494 | 0.053 | 0.115 |
| --- | --- | --- | --- | --- | --- | --- | --- |
| PWY.6605 | adenine and adenosine salvage II | 0 | Not detected | 0.164 | -0.079 | 0.408 | 0.186 |
| PWY.6606 | guanosine nucleotides degradation | 0 | Not detected | 0.259 | 0.021 | 0.497 | 0.033 |
| PWY.6607 | guanosine nucleotides degradation | 0 | Not detected | 0.101 | -0.123 | 0.324 | 0.377 |
| PWY.6608 | guanosine nucleotides degradation | 0 | Not detected | 0.187 | -0.059 | 0.432 | 0.136 |
| PWY.6610 | adenine salvage | 0 | Not detected | 0.077 | -0.167 | 0.320 | 0.537 |
| PWY.6613 | tetrahydrofolate salvage from 5,10 | 0 | Not detected | -0.043 | -0.177 | 0.090 | 0.522 |
| PWY.6614 | tetrahydrofolate biosynthesis | 0 | Not detected | 0.009 | -0.224 | 0.241 | 0.943 |
| PWY.6620 | guanine and guanosine salvage | 0 | Not detected | 0.020 | -0.230 | 0.270 | 0.877 |
| PWY.6626 | cytidine-5'-diphosphate-glycerol b | 0 | Not detected | -0.125 | -0.348 | 0.098 | 0.272 |
| PWY.6637 | sulfolactate degradation II | 0 | Not detected | -0.060 | -0.162 | 0.042 | 0.249 |
| PWY.6654 | phosphopantothenate biosynthesis | 0 | Not detected | 0.177 | -0.020 | 0.375 | 0.079 |
| PWY.6672 | <i>cis</i>-genanyl-CoA degradati | 0 | Not detected | 0.389 | 0.138 | 0.640 | 0.002 |
| PWY.6682 | dehydrophos biosynthesis | 0 | Not detected | -0.264 | -0.563 | 0.036 | 0.085 |
| PWY.6690 | cinnamate and 3-hydroxycinnamat | 0 | Not detected | 0.239 | 0.010 | 0.468 | 0.041 |
| PWY.6700 | queuosine biosynthesis | 0 | Not detected | 0.065 | -0.076 | 0.205 | 0.368 |
| PWY.6703 | preQ<sub>0</sub> biosynthesis | 0 | Not detected | -0.050 | -0.186 | 0.086 | 0.472 |
| PWY.6707 | gallate biosynthesis | 0 | Not detected | -0.243 | -0.483 | -0.002 | 0.049 |
| PWY.6708 | ubiquinol-8 biosynthesis (prokary | 0 | Not detected | 0.121 | -0.157 | 0.400 | 0.394 |
| PWY.6724 | starch degradation II | 0 | Not detected | -0.081 | -0.331 | 0.169 | 0.524 |
| PWY.6728 | methylaspartate cycle | 0 | Not detected | -0.075 | -0.177 | 0.027 | 0.151 |
| PWY.6731 | starch degradation III | 0 | Not detected | -0.116 | -0.259 | 0.026 | 0.111 |
| PWY.6737 | starch degradation V | 0 | Not detected | -0.116 | -0.259 | 0.026 | 0.110 |
| PWY.6748 | nitrate reduction VII (denitrificatio | 0 | Not detected | 0.066 | -0.196 | 0.328 | 0.620 |
| PWY.6749 | CMP-legionaminate biosynthesis I | 0 | Not detected | 0.233 | 0.008 | 0.458 | 0.042 |
| PWY.6771 | rhamnogalacturonan type I degrad | 0 | Not detected | -0.032 | -0.220 | 0.156 | 0.738 |
| PWY.6772 | hydrogen production V | 0 | Not detected | -0.015 | -0.274 | 0.244 | 0.907 |
| PWY.6786 | detoxification of reactive carbonyl | 0 | Not detected | 0.003 | -0.080 | 0.086 | 0.936 |
| PWY.6788 | cellulose degradation II (fungi) | 27 | Not detected | -0.119 | -0.351 | 0.113 | 0.314 |
| PWY.6799 | fatty acid biosynthesis (plant mito | 0 | Not detected | -0.014 | -0.254 | 0.226 | 0.910 |
| PWY.6808 | dTDP-D-forosamine biosynthesis | 0 | Not detected | -0.074 | -0.163 | 0.015 | 0.102 |
| PWY.6823 | molybdenum cofactor biosynthesis | 0 | Not detected | 0.198 | -0.050 | 0.445 | 0.117 |
| PWY.6839 | 2-aminoethylphosphonate biosynt | 0 | Not detected | -0.264 | -0.563 | 0.036 | 0.085 |
| PWY.6840 | homoglutathione biosynthesis | 0 | Not detected | 0.046 | -0.179 | 0.270 | 0.690 |
| PWY.6842 | glutathione-mediated detoxificatio | 0 | Not detected | -0.049 | -0.262 | 0.165 | 0.654 |
| PWY.6854 | ethylene biosynthesis III (microbe | 0 | Not detected | 0.037 | -0.226 | 0.301 | 0.781 |
| PWY.6855 | chitin degradation I (archaea) | 0 | Not detected | 0.032 | -0.201 | 0.266 | 0.787 |
| PWY.6859 | <i>all-trans</i>-farnesol biosynth | 0 | Not detected | -0.003 | -0.274 | 0.268 | 0.984 |
| PWY.6863 | pyruvate fermentation to hexanol ( | 0 | Not detected | -0.063 | -0.183 | 0.058 | 0.307 |
| PWY.6871 | 3-methylbutanol biosynthesis (eng | 0 | Not detected | 0.054 | -0.100 | 0.209 | 0.492 |
| PWY.6883 | pyruvate fermentation to butanol I | 0 | Not detected | -0.063 | -0.183 | 0.058 | 0.308 |
| PWY.6886 | 1-butanol autotrophic biosynthesis | 0 | Not detected | -0.050 | -0.134 | 0.034 | 0.241 |
| PWY.6890 | 4-amino-2-methyl-5-diphosphome | 0 | Not detected | -0.046 | -0.150 | 0.058 | 0.390 |
| PWY.6891 | thiazole biosynthesis II (aerobic ba | 0 | Not detected | -0.046 | -0.141 | 0.049 | 0.343 |

| PWY.6892 | thiazole biosynthesis I (facultative | 0 | Not detected | -0.028 | -0.107 | 0.051 | 0.490 |
| --- | --- | --- | --- | --- | --- | --- | --- |
| PWY.6893 | thiamine diphosphate biosynthesis | 0 | Not detected | 0.003 | -0.202 | 0.208 | 0.977 |
| PWY.6894 | thiamine diphosphate biosynthesis | 0 | Not detected | -0.001 | -0.226 | 0.224 | 0.992 |
| PWY.6896 | thiamine salvage I | 0 | Not detected | 0.054 | -0.171 | 0.280 | 0.638 |
| PWY.6897 | thiamine salvage II | 0 | Not detected | -0.001 | -0.226 | 0.224 | 0.992 |
| PWY.6901 | superpathway of glucose and xylo | 0 | Not detected | -0.048 | -0.132 | 0.035 | 0.258 |
| PWY.6902 | chitin degradation II (Vibrio) | 0 | Not detected | 0.035 | -0.197 | 0.268 | 0.765 |
| PWY.6906 | chitin derivatives degradation | 0 | Not detected | -0.055 | -0.141 | 0.030 | 0.206 |
| PWY.6938 | NADH repair | 0 | Not detected | 0.118 | -0.113 | 0.350 | 0.316 |
| PWY.6942 | dTDP-D-desosamine biosynthesis | 0 | Not detected | -0.069 | -0.158 | 0.020 | 0.128 |
| PWY.6944 | androstenedione degradation | 0 | Not detected | 0.088 | -0.155 | 0.330 | 0.480 |
| PWY.6949 | DIBOA-glucoside biosynthesis | 0 | Not detected | 0.088 | -0.097 | 0.273 | 0.349 |
| PWY.6953 | dTDP-3-acetamido-&alpha;-D-fuc | 0 | Not detected | -0.079 | -0.168 | 0.010 | 0.082 |
| PWY.6961 | L-ascorbate degradation II (bacteri | 0 | Not detected | -0.047 | -0.124 | 0.030 | 0.233 |
| PWY.6969 | TCA cycle V (2-oxoglutarate:ferre | 0 | Not detected | -0.066 | -0.157 | 0.024 | 0.153 |
| PWY.6973 | dTDP-D-olivose, dTDP-D-oliose | 0 | Not detected | -0.074 | -0.163 | 0.015 | 0.102 |
| PWY.6974 | dTDP-L-olivose biosynthesis | 0 | Not detected | -0.074 | -0.163 | 0.015 | 0.102 |
| PWY.6976 | dTDP-L-mycarose biosynthesis | 0 | Not detected | -0.072 | -0.160 | 0.016 | 0.107 |
| PWY.6981 | chitin biosynthesis | 0 | Not detected | -0.058 | -0.145 | 0.028 | 0.186 |
| PWY.6983 | <i>threo</i>-tetrahydrobiopterin b | 0 | Not detected | -0.083 | -0.241 | 0.075 | 0.305 |
| PWY.6984 | lipoate salvage II | 0 | Not detected | 0.102 | -0.092 | 0.295 | 0.303 |
| PWY.6986 | alginate degradation | 0 | Not detected | -0.054 | -0.147 | 0.040 | 0.260 |
| PWY.6987 | lipoate biosynthesis and incorpora | 0 | Not detected | 0.006 | -0.230 | 0.242 | 0.961 |
| PWY.6992 | 1,5-anhydrofructose degradation | 0 | Not detected | -0.049 | -0.137 | 0.038 | 0.270 |
| PWY.7003 | glycerol degradation to butanol | 0 | Not detected | -0.048 | -0.132 | 0.036 | 0.260 |
| PWY.7007 | methyl ketone biosynthesis (engin | 0 | Not detected | 0.232 | -0.046 | 0.510 | 0.102 |
| PWY.7013 | (<i>S</i>)-propane-1,2-diol degra | 0 | Not detected | -0.145 | -0.405 | 0.114 | 0.273 |
| PWY.702 | L-methionine biosynthesis II (plan | 0 | Not detected | -0.042 | -0.305 | 0.222 | 0.757 |
| PWY.7031 | protein <i>N</i>-glycosylation (b | 0 | Not detected | 0.014 | -0.258 | 0.286 | 0.919 |
| PWY.7037 | protein <i>O</i>-glycosylation (N | 0 | Not detected | 0.017 | -0.256 | 0.290 | 0.903 |
| PWY.7077 | <i>N</i>-acetyl-D-galactosamine | 0 | Not detected | 0.131 | -0.116 | 0.378 | 0.299 |
| PWY.7085 | triethylamine degradation | 0 | Not detected | -0.099 | -0.369 | 0.171 | 0.473 |
| PWY.7090 | UDP-2,3-diacetamido-2,3-dideoxy | 0 | Not detected | -0.174 | -0.389 | 0.042 | 0.115 |
| PWY.7096 | triclosan resistance | 0 | Not detected | -0.078 | -0.359 | 0.202 | 0.584 |
| PWY.7102 | bisabolene biosynthesis (engineere | 0 | Not detected | -0.003 | -0.274 | 0.268 | 0.984 |
| PWY.7104 | dTDP-L-megosamine biosynthesis | 0 | Not detected | -0.074 | -0.163 | 0.015 | 0.102 |
| PWY.7111 | pyruvate fermentation to isobutano | 0 | Not detected | -0.070 | -0.175 | 0.035 | 0.190 |
| PWY.7115 | C4 photosynthetic carbon assimila | 0 | Not detected | 0.236 | -0.048 | 0.519 | 0.103 |
| PWY.7117 | C4 photosynthetic carbon assimila | 0 | Not detected | -0.053 | -0.146 | 0.040 | 0.266 |
| PWY.7118 | chitin degradation to ethanol | 0 | Not detected | 0.201 | -0.083 | 0.484 | 0.165 |
| PWY.7124 | ethylene biosynthesis V (engineere | 0 | Not detected | -0.067 | -0.158 | 0.024 | 0.148 |
| PWY.7127 | CDP-D-mannitol biosynthesis | 0 | Not detected | -0.112 | -0.387 | 0.163 | 0.425 |
| PWY.7130 | L-glucose degradation | 0 | Not detected | 0.109 | -0.128 | 0.346 | 0.368 |
| PWY.7131 | CMP-legionaminate biosynthesis I | 0 | Not detected | -0.024 | -0.261 | 0.212 | 0.840 |

| PWY.7141 | (3<i>S</i>)-linalool biosynthesis | 0 | Not detected | 0.032 | -0.194 | 0.259 | 0.780 |
| --- | --- | --- | --- | --- | --- | --- | --- |
| PWY.7153 | grixazone biosynthesis | 0 | Not detected | 0.154 | -0.096 | 0.404 | 0.226 |
| PWY.7159 | 3,8-divinyl-chlorophyllide <i>a</i | 0 | Not detected | 0.028 | -0.228 | 0.283 | 0.831 |
| PWY.7165 | L-ascorbate biosynthesis VI (engin | 0 | Not detected | 0.329 | 0.055 | 0.602 | 0.019 |
| PWY.7174 | <i>S</i>-methyl-5-thio-&alpha;-D | 0 | Not detected | 0.145 | -0.125 | 0.415 | 0.292 |
| PWY.7176 | UTP and CTP <i>de novo</i> bio | 0 | Not detected | -0.045 | -0.139 | 0.049 | 0.347 |
| PWY.7177 | UTP and CTP dephosphorylation | 0 | Not detected | -0.043 | -0.141 | 0.055 | 0.390 |
| PWY.7180 | 2'-deoxy-&alpha;-D-ribose 1-phos | 0 | Not detected | 0.021 | -0.239 | 0.282 | 0.874 |
| PWY.7182 | linalool biosynthesis I | 0 | Not detected | 0.032 | -0.194 | 0.259 | 0.780 |
| PWY.7184 | pyrimidine deoxyribonucleotides < | 0 | Not detected | -0.077 | -0.183 | 0.029 | 0.154 |
| PWY.7185 | UTP and CTP dephosphorylation | 0 | Not detected | -0.043 | -0.141 | 0.055 | 0.390 |
| PWY.7187 | pyrimidine deoxyribonucleotides < | 0 | Not detected | -0.071 | -0.173 | 0.031 | 0.173 |
| PWY.7193 | pyrimidine ribonucleosides salvag | 0 | Not detected | -0.007 | -0.289 | 0.276 | 0.964 |
| PWY.7195 | pyrimidine ribonucleosides salvag | 0 | Not detected | -0.018 | -0.212 | 0.176 | 0.857 |
| PWY.7197 | pyrimidine deoxyribonucleotide p | 0 | Not detected | -0.073 | -0.177 | 0.031 | 0.169 |
| PWY.7198 | pyrimidine deoxyribonucleotides < | 0 | Not detected | -0.079 | -0.188 | 0.029 | 0.150 |
| PWY.7204 | pyridoxal 5'-phosphate salvage II ( | 0 | Not detected | 0.092 | -0.156 | 0.340 | 0.467 |
| PWY.7205 | CMP phosphorylation | 0 | Not detected | -0.067 | -0.166 | 0.032 | 0.183 |
| PWY.7210 | pyrimidine deoxyribonucleotides b | 0 | Not detected | -0.079 | -0.188 | 0.029 | 0.150 |
| PWY.7216 | (R)- and (S)-3-hydroxybutanoate b | 0 | Not detected | -0.025 | -0.192 | 0.142 | 0.769 |
| PWY.7218 | photosynthetic 3-hydroxybutanoat | 0 | Not detected | -0.055 | -0.152 | 0.042 | 0.266 |
| PWY.7219 | adenosine ribonucleotides <i>de n | 0 | Not detected | -0.077 | -0.183 | 0.029 | 0.155 |
| PWY.7221 | guanosine ribonucleotides <i>de n | 0 | Not detected | -0.079 | -0.185 | 0.027 | 0.144 |
| PWY.7222 | guanosine deoxyribonucleotides < | 0 | Not detected | -0.079 | -0.185 | 0.027 | 0.144 |
| PWY.7224 | purine deoxyribonucleosides salva | 0 | Not detected | -0.079 | -0.185 | 0.027 | 0.144 |
| PWY.7226 | guanosine deoxyribonucleotides < | 0 | Not detected | -0.079 | -0.185 | 0.027 | 0.144 |
| PWY.7234 | inosine-5'-phosphate biosynthesis | 0 | Not detected | 0.081 | -0.064 | 0.227 | 0.275 |
| PWY.7237 | <i>myo</i>-, <i>chiro</i>- and <i | 0 | Not detected | -0.091 | -0.323 | 0.141 | 0.444 |
| PWY.7238 | sucrose biosynthesis II | 0 | Not detected | -0.057 | -0.140 | 0.026 | 0.178 |
| PWY.7241 | <I>myo</I>-inositol degradation I | 0 | Not detected | -0.097 | -0.328 | 0.133 | 0.408 |
| PWY.7242 | D-fructuronate degradation | 0 | Not detected | -0.049 | -0.127 | 0.029 | 0.217 |
| PWY.7247 | &beta;-D-glucuronide and D-gluc | 0 | Not detected | -0.048 | -0.140 | 0.044 | 0.310 |
| PWY.7248 | pectin degradation II | 0 | Not detected | -0.048 | -0.140 | 0.044 | 0.310 |
| PWY.7250 | [2Fe-2S] iron-sulfur cluster biosyn | 0 | Not detected | 0.260 | 0.019 | 0.502 | 0.035 |
| PWY.7254 | TCA cycle VII (acetate-producers) | 0 | Not detected | -0.072 | -0.166 | 0.023 | 0.137 |
| PWY.7255 | ergothioneine biosynthesis I (bacte | 0 | Not detected | 0.046 | -0.179 | 0.270 | 0.690 |
| PWY.7268 | NAD/NADP-NADH/NADPH cyto | 0 | Not detected | -0.043 | -0.245 | 0.158 | 0.675 |
| PWY.7269 | NAD/NADP-NADH/NADPH mit | 0 | Not detected | 0.023 | -0.186 | 0.232 | 0.827 |
| PWY.7279 | aerobic respiration II (cytochrome | 0 | Not detected | 0.027 | -0.182 | 0.236 | 0.798 |
| PWY.7282 | 4-amino-2-methyl-5-diphosphome | 0 | Not detected | 0.089 | -0.087 | 0.264 | 0.322 |
| PWY.7288 | fatty acid &beta;-oxidation (perox | 0 | Not detected | 0.115 | -0.147 | 0.376 | 0.390 |
| PWY.7290 | <i>Escherichia coli</i> serotype O | 0 | Not detected | -0.261 | -0.545 | 0.022 | 0.071 |
| PWY.7294 | D-xylose degradation IV | 0 | Not detected | 0.146 | -0.129 | 0.421 | 0.299 |
| PWY.7295 | L-arabinose degradation IV | 0 | Not detected | 0.104 | -0.161 | 0.369 | 0.443 |

| PWY.7301 | dTDP-4-<i>O</i>-demethyl-&bet | 0 | Not detected | -0.065 | -0.154 | 0.025 | 0.158 |
| --- | --- | --- | --- | --- | --- | --- | --- |
| PWY.7312 | dTDP-D-&beta;-fucofuranose bios | 0 | Not detected | -0.071 | -0.159 | 0.016 | 0.111 |
| PWY.7315 | dTDP-<i>N</i>-acetylthomosami | 0 | Not detected | -0.070 | -0.154 | 0.013 | 0.100 |
| PWY.7316 | dTDP-<i>N</i>-acetylviosamine | 0 | Not detected | -0.069 | -0.157 | 0.020 | 0.130 |
| PWY.7318 | dTDP-3-acetamido-3,6-dideoxy-& | 0 | Not detected | -0.074 | -0.163 | 0.014 | 0.101 |
| PWY.7328 | superpathway of UDP-glucose-der | 0 | Not detected | -0.054 | -0.164 | 0.055 | 0.332 |
| PWY.7330 | UDP-<i>N</i>-acetyl-&beta;-L-fu | 0 | Not detected | -0.117 | -0.317 | 0.084 | 0.255 |
| PWY.7331 | UDP-<i>N</i>-acetyl-&beta;-L-q | 0 | Not detected | -0.076 | -0.274 | 0.123 | 0.454 |
| PWY.7332 | superpathway of UDP-<I>N</i>-a | 0 | Not detected | -0.127 | -0.339 | 0.085 | 0.239 |
| PWY.7335 | UDP-<i>N</i>-acetyl-&alpha;-D- | 0 | Not detected | -0.091 | -0.229 | 0.047 | 0.195 |
| PWY.7336 | UDP-<i>N</i>-acetyl-&alpha;-D- | 0 | Not detected | -0.203 | -0.428 | 0.022 | 0.077 |
| PWY.7342 | superpathway of nicotine biosynth | 0 | Not detected | 0.126 | -0.172 | 0.425 | 0.407 |
| PWY.7343 | UDP-&alpha;-D-glucose biosynth | 0 | Not detected | -0.061 | -0.158 | 0.036 | 0.220 |
| PWY.7344 | UDP-&alpha;-D-galactose biosynt | 0 | Not detected | -0.028 | -0.193 | 0.138 | 0.744 |
| PWY.7346 | UDP-&alpha;-D-glucuronate bios | 0 | Not detected | -0.005 | -0.245 | 0.235 | 0.967 |
| PWY.7347 | sucrose biosynthesis III | 0 | Not detected | -0.062 | -0.162 | 0.039 | 0.230 |
| PWY.7377 | cob(II)yrinate <i>a,c</i>-diamide | 0 | Not detected | -0.007 | -0.119 | 0.105 | 0.901 |
| PWY.7378 | aminopropanol phosphate biosynt | 0 | Not detected | 0.273 | 0.018 | 0.527 | 0.036 |
| PWY.7383 | anaerobic energy metabolism (inv | 0 | Not detected | 0.101 | -0.180 | 0.383 | 0.481 |
| PWY.7384 | anaerobic energy metabolism (inv | 0 | Not detected | -0.002 | -0.110 | 0.106 | 0.976 |
| PWY.7385 | 1,3-propanediol biosynthesis (engi | 0 | Not detected | -0.044 | -0.129 | 0.042 | 0.319 |
| PWY.7388 | octanoyl-[acyl-carrier protein] bio | 0 | Not detected | -0.022 | -0.263 | 0.218 | 0.858 |
| PWY.7391 | isoprene biosynthesis II (engineere | 0 | Not detected | 0.022 | -0.159 | 0.203 | 0.815 |
| PWY.7395 | D-galactosamine and <i>N</i>-ac | 0 | Not detected | 0.131 | -0.116 | 0.378 | 0.299 |
| PWY.7396 | butanol and isobutanol biosynthes | 0 | Not detected | -0.081 | -0.207 | 0.046 | 0.214 |
| PWY.7398 | coumarins biosynthesis (engineere | 0 | Not detected | 0.022 | -0.211 | 0.255 | 0.851 |
| PWY.7399 | methylphosphonate degradation II | 0 | Not detected | 0.152 | -0.098 | 0.402 | 0.234 |
| PWY.7400 | L-arginine biosynthesis IV (archae | 0 | Not detected | -0.073 | -0.208 | 0.062 | 0.292 |
| PWY.7401 | crotonate fermentation (to acetate | 0 | Not detected | 0.010 | -0.144 | 0.165 | 0.895 |
| PWY.7410 | ipsdienol biosynthesis | 0 | Not detected | 0.032 | -0.194 | 0.259 | 0.780 |
| PWY.7413 | dTDP-6-deoxy-&alpha;-D-allose b | 0 | Not detected | -0.074 | -0.163 | 0.015 | 0.102 |
| PWY.7414 | dTDP-&alpha;-D-mycaminose bio | 0 | Not detected | -0.074 | -0.163 | 0.014 | 0.101 |
| PWY.7419 | FR-900098 and FR-33289 antibio | 0 | Not detected | -0.264 | -0.563 | 0.036 | 0.085 |
| PWY.7425 | 2-chloroacrylate degradation I | 0 | Not detected | 0.080 | -0.145 | 0.305 | 0.485 |
| PWY.7431 | aromatic biogenic amine degradati | 0 | Not detected | 0.079 | -0.132 | 0.290 | 0.463 |
| PWY.7440 | dTDP-&beta;-L-4-<i>epi</i>-van | 0 | Not detected | -0.074 | -0.163 | 0.015 | 0.102 |
| PWY.7442 | drosopterin and aurodrosopterin b | 0 | Not detected | -0.083 | -0.241 | 0.075 | 0.305 |
| PWY.7446 | sulfoquinovose degradation I | 0 | Not detected | -0.073 | -0.361 | 0.215 | 0.620 |
| PWY.7456 | &beta;-(1,4)-mannan degradation | 0 | Not detected | -0.023 | -0.169 | 0.122 | 0.752 |
| PWY.7459 | kojibiose degradation | 0 | Not detected | 0.164 | -0.070 | 0.397 | 0.171 |
| PWY.7467 | 2-acetamido-4-amino-2,4,6-trideo | 0 | Not detected | 0.073 | -0.148 | 0.295 | 0.518 |
| PWY.7510 | rhizocticin A and B biosynthesis | 0 | Not detected | -0.264 | -0.563 | 0.036 | 0.085 |
| PWY.7524 | mevalonate pathway III (archaea) | 0 | Not detected | 0.022 | -0.159 | 0.203 | 0.815 |
| PWY.7530 | &beta;-D-galactosaminyl-(1&rarr; | 0 | Not detected | -0.263 | -0.547 | 0.020 | 0.069 |

| PWY.7539 | 6-hydroxymethyl-dihydropterin di | 0 | Not detected | -0.049 | -0.148 | 0.051 | 0.339 |
| --- | --- | --- | --- | --- | --- | --- | --- |
| PWY.7541 | 1,2-propanediol biosynthesis from | 0 | Not detected | 0.049 | -0.238 | 0.336 | 0.738 |
| PWY.7552 | heme <i>b</i> biosynthesis III (fr | 0 | Not detected | 0.187 | -0.071 | 0.445 | 0.156 |
| PWY.7559 | glutathione degradation (DUG pat | 0 | Not detected | 0.072 | -0.190 | 0.334 | 0.589 |
| PWY.7560 | methylerythritol phosphate pathwa | 0 | Not detected | -0.055 | -0.184 | 0.074 | 0.402 |
| PWY.7562 | 3,6-anhydro-&alpha;-L-galactopyr | 0 | Not detected | -0.048 | -0.134 | 0.038 | 0.274 |
| PWY.7564 | bacimethrin and bacimethrin pyro | 0 | Not detected | -0.129 | -0.305 | 0.047 | 0.151 |
| PWY.7568 | L-galactose degradation | 0 | Not detected | 0.021 | -0.200 | 0.241 | 0.855 |
| PWY.7573 | GDP-mycosamine biosynthesis | 0 | Not detected | -0.220 | -0.494 | 0.053 | 0.115 |
| PWY.7581 | <i>N</i>-acetylneuraminate and < | 0 | Not detected | -0.073 | -0.265 | 0.120 | 0.461 |
| PWY.7586 | &beta;-1,4-D-mannosyl-<i>N</i> | 0 | Not detected | -0.026 | -0.258 | 0.207 | 0.827 |
| PWY.7588 | ursodeoxycholate biosynthesis (ba | 0 | Not detected | -0.186 | -0.409 | 0.037 | 0.103 |
| PWY.7610 | GDP-6-deoxy-D-<i>altro</i>-hep | 0 | Not detected | -0.055 | -0.261 | 0.152 | 0.602 |
| PWY.7616 | methanol oxidation to carbon diox | 0 | Not detected | -0.019 | -0.278 | 0.240 | 0.885 |
| PWY.7622 | UDP-&alpha;-D-galactofuranose b | 0 | Not detected | -0.057 | -0.167 | 0.054 | 0.316 |
| PWY.7644 | heparin degradation | 0 | Not detected | 0.010 | -0.254 | 0.273 | 0.944 |
| PWY.7645 | hyaluronan degradation | 0 | Not detected | -0.058 | -0.294 | 0.177 | 0.628 |
| PWY.7646 | dermatan sulfate degradation I (ba | 0 | Not detected | -0.090 | -0.323 | 0.144 | 0.451 |
| PWY.7651 | heparan sulfate degradation | 0 | Not detected | 0.269 | -0.067 | 0.605 | 0.117 |
| PWY.7657 | dTDP-&beta;-L-digitoxose biosyn | 0 | Not detected | -0.074 | -0.163 | 0.015 | 0.102 |
| PWY.7659 | viridicatumtoxin biosynthesis | 0 | Not detected | 0.032 | -0.194 | 0.259 | 0.780 |
| PWY.7663 | gondoate biosynthesis (anaerobic) | 0 | Not detected | -0.073 | -0.187 | 0.041 | 0.209 |
| PWY.7664 | oleate biosynthesis IV (anaerobic) | 0 | Not detected | -0.074 | -0.189 | 0.041 | 0.205 |
| PWY.7674 | CMP-8-amino-3,8-dideoxy-D-<I> | 0 | Not detected | -0.196 | -0.438 | 0.046 | 0.112 |
| PWY.7675 | Kdo transfer to lipid IV<sub>A</s | 0 | Not detected | 0.022 | -0.210 | 0.254 | 0.853 |
| PWY.7684 | &alpha;-diglucosyldiacylglycerol | 0 | Not detected | -0.064 | -0.293 | 0.165 | 0.585 |
| PWY.7688 | dTDP-D-ravidosamine and dTDP- | 0 | Not detected | -0.079 | -0.168 | 0.010 | 0.082 |
| PWY.7709 | (3<i>R</i>)-linalool biosynthesis | 0 | Not detected | 0.032 | -0.194 | 0.259 | 0.780 |
| PWY.7716 | penicillin G and penicillin V biosy | 0 | Not detected | 0.011 | -0.135 | 0.158 | 0.879 |
| PWY.7719 | CMP-diacetamido-8-epilegionami | 0 | Not detected | -0.195 | -0.387 | -0.002 | 0.048 |
| PWY.7721 | methyl phomopsenoate biosynthes | 0 | Not detected | 0.029 | -0.202 | 0.261 | 0.805 |
| PWY.7736 | stellatic acid biosynthesis | 0 | Not detected | 0.031 | -0.238 | 0.300 | 0.822 |
| PWY.7754 | bile acids degradation | 0 | Not detected | -0.216 | -0.550 | 0.119 | 0.207 |
| PWY.7755 | iso-bile acids biosynthesis I | 0 | Not detected | -0.236 | -0.549 | 0.076 | 0.139 |
| PWY.7756 | iso-bile acids biosynthesis II | 0 | Not detected | 0.059 | -0.140 | 0.258 | 0.562 |
| PWY.7766 | heme <i>b</i> biosynthesis IV (G | 0 | Not detected | 0.029 | -0.227 | 0.284 | 0.825 |
| PWY.7769 | phosalacine biosynthesis | 0 | Not detected | -0.247 | -0.545 | 0.051 | 0.105 |
| PWY.7778 | 2-methylpropene degradation | 0 | Not detected | -0.024 | -0.191 | 0.143 | 0.775 |
| PWY.7779 | methyl <i>tert</i>-butyl ether deg | 0 | Not detected | -0.024 | -0.191 | 0.143 | 0.775 |
| PWY.7790 | UMP biosynthesis II | 0 | Not detected | -0.046 | -0.155 | 0.063 | 0.408 |
| PWY.7791 | UMP biosynthesis III | 0 | Not detected | -0.046 | -0.155 | 0.063 | 0.408 |
| PWY.7793 | dimethyl sulfide biosynthesis from | 0 | Not detected | -0.090 | -0.319 | 0.139 | 0.442 |
| PWY.7805 | (aminomethyl)phosphonate degrad | 0 | Not detected | 0.158 | -0.092 | 0.407 | 0.217 |
| PWY.7807 | glyphosate degradation III | 0 | Not detected | 0.077 | -0.162 | 0.317 | 0.527 |

| PWY.7808 | tetracycline resistance | 0 | Not detected | 0.120 | -0.279 | 0.519 | 0.556 |
| --- | --- | --- | --- | --- | --- | --- | --- |
| PWY.7814 | dTDP-L-daunosamine biosynthesi | 0 | Not detected | -0.065 | -0.154 | 0.025 | 0.158 |
| PWY.7815 | poly(ribitol phosphate) wall teicho | 0 | Not detected | -0.108 | -0.233 | 0.018 | 0.093 |
| PWY.7816 | poly(ribitol phosphate) wall teicho | 0 | Not detected | -0.108 | -0.233 | 0.018 | 0.093 |
| PWY.7817 | type I lipoteichoic acid biosynthes | 0 | Not detected | 0.007 | -0.104 | 0.118 | 0.903 |
| PWY.7818 | type IV lipoteichoic acid biosynth | 0 | Not detected | -0.084 | -0.287 | 0.119 | 0.419 |
| PWY.7819 | poly(3-O-&beta;-D-glucopyranosy | 0 | Not detected | -0.091 | -0.229 | 0.047 | 0.196 |
| PWY.7820 | teichuronic acid biosynthesis (<i> | 0 | Not detected | 0.000 | -0.238 | 0.238 | 1.000 |
| PWY.7822 | chitin degradation III (Serratia) | 0 | Not detected | 0.037 | -0.196 | 0.269 | 0.757 |
| PWY.7852 | 6-hydroxymethyl-dihydropterin di | 0 | Not detected | -0.058 | -0.198 | 0.082 | 0.416 |
| PWY.7854 | crotonyl-CoA/ethylmalonyl-CoA/h | 0 | Not detected | 0.091 | -0.174 | 0.356 | 0.501 |
| PWY.7856 | heme <i>a</i> biosynthesis | 0 | Not detected | -0.062 | -0.255 | 0.131 | 0.530 |
| PWY.7858 | (5Z)-dodecenoate biosynthesis II | 0 | Not detected | -0.067 | -0.178 | 0.044 | 0.236 |
| PWY.7863 | roseoflavin biosynthesis | 0 | Not detected | 0.094 | -0.109 | 0.296 | 0.365 |
| PWY.7864 | 8-methylmenaquinone biosynthesi | 0 | Not detected | 0.061 | -0.225 | 0.346 | 0.677 |
| PWY.7873 | D-erythronate degradation II | 0 | Not detected | 0.128 | -0.083 | 0.338 | 0.235 |
| PWY.7874 | L-threonate degradation | 0 | Not detected | 0.223 | -0.015 | 0.461 | 0.066 |
| PWY.7883 | anhydromuropeptides recycling II | 0 | Not detected | 0.142 | -0.112 | 0.396 | 0.273 |
| PWY.7884 | lipoprotein posttranslational modif | 0 | Not detected | 0.105 | -0.182 | 0.392 | 0.475 |
| PWY.7892 | tRNA-uridine 2-thiolation (bacteri | 0 | Not detected | 0.147 | -0.039 | 0.332 | 0.121 |
| PWY.7900 | glycogen biosynthesis III (from & | 0 | Not detected | -0.098 | -0.210 | 0.014 | 0.085 |
| PWY.7902 | glucosylglycerol biosynthesis | 0 | Not detected | -0.099 | -0.223 | 0.025 | 0.117 |
| PWY.7904 | tRNA-uridine 2-thiolation (thermo | 0 | Not detected | 0.074 | -0.169 | 0.318 | 0.550 |
| PWY.7909 | formaldehyde oxidation VII (THF | 0 | Not detected | -0.071 | -0.222 | 0.081 | 0.361 |
| PWY.7928 | nickel cofactor biosynthesis | 0 | Not detected | -0.061 | -0.242 | 0.121 | 0.512 |
| PWY.7953 | UDP-<i>N</i>-acetylmuramoyl-p | 0 | Not detected | -0.026 | -0.100 | 0.049 | 0.495 |
| PWY.7975 | adenosylcobalamin biosynthesis fr | 0 | Not detected | 0.109 | -0.088 | 0.307 | 0.277 |
| PWY.7977 | L-methionine biosynthesis IV (arc | 0 | Not detected | -0.049 | -0.143 | 0.045 | 0.309 |
| PWY.7978 | <i>N</i>-methylpyrrolidone degra | 0 | Not detected | 0.243 | 0.040 | 0.445 | 0.019 |
| PWY.7981 | &alpha;-dystroglycan glycosylatio | 0 | Not detected | 0.023 | -0.251 | 0.297 | 0.870 |
| PWY.7991 | toxoflavin biosynthesis | 0 | Not detected | -0.040 | -0.131 | 0.052 | 0.394 |
| PWY.8004 | Entner-Doudoroff pathway I | 0 | Not detected | -0.039 | -0.123 | 0.046 | 0.369 |
| PWY.801 | homocysteine and cysteine interco | 0 | Not detected | -0.164 | -0.422 | 0.093 | 0.211 |
| PWY.8010 | L-cysteine biosynthesis IX (Tricho | 0 | Not detected | -0.033 | -0.157 | 0.091 | 0.598 |
| PWY.8012 | mupirocin biosynthesis | 0 | Not detected | -0.014 | -0.254 | 0.226 | 0.910 |
| PWY.8013 | UDP-<i>N</i>-acetyl-D-galactosa | 0 | Not detected | -0.062 | -0.162 | 0.039 | 0.230 |
| PWY.8015 | glycine degradation (Stickland rea | 0 | Not detected | -0.037 | -0.119 | 0.045 | 0.379 |
| PWY.8040 | ansatrienin biosynthesis | 0 | Not detected | -0.161 | -0.432 | 0.110 | 0.246 |
| PWY.882 | L-ascorbate biosynthesis I (L-gala | 0 | Not detected | 0.037 | -0.197 | 0.272 | 0.755 |
| PWY.922 | mevalonate pathway I | 0 | Not detected | 0.022 | -0.159 | 0.203 | 0.815 |
| PWY.I9 | L-cysteine biosynthesis VI (from L | 0 | Not detected | -0.089 | -0.352 | 0.174 | 0.506 |
| PWY0.1182 | trehalose degradation II (cytosolic | 0 | Not detected | 0.039 | -0.239 | 0.317 | 0.784 |
| PWY0.1221 | putrescine degradation II | 0 | Not detected | 0.235 | -0.001 | 0.471 | 0.051 |
| PWY0.1241 | ADP-L-<i>glycero</i>-&beta;-D- | 0 | Not detected | 0.141 | -0.093 | 0.375 | 0.238 |

| PWY0.1261 | anhydromuropeptides recycling I | 0 | Not detected | -0.006 | -0.076 | 0.065 | 0.874 |
| --- | --- | --- | --- | --- | --- | --- | --- |
| PWY0.1264 | biotin-carboxyl carrier protein ass | 0 | Not detected | -0.097 | -0.207 | 0.014 | 0.087 |
| PWY0.1275 | lipoate biosynthesis and incorpora | 0 | Not detected | 0.034 | -0.169 | 0.237 | 0.743 |
| PWY0.1296 | purine ribonucleosides degradatio | 0 | Not detected | 0.051 | -0.166 | 0.267 | 0.646 |
| PWY0.1300 | 2-<I>O</I>-&alpha;-mannosyl-D- | 0 | Not detected | 0.157 | -0.113 | 0.427 | 0.255 |
| PWY0.1306 | L-galactonate degradation | 0 | Not detected | 0.004 | -0.188 | 0.196 | 0.966 |
| PWY0.1312 | acetate and ATP formation from a | 0 | Not detected | -0.039 | -0.118 | 0.040 | 0.337 |
| PWY0.1313 | acetate conversion to acetyl-CoA | 0 | Not detected | 0.389 | 0.138 | 0.640 | 0.002 |
| PWY0.1317 | L-lactaldehyde degradation (aerob | 0 | Not detected | 0.144 | -0.089 | 0.377 | 0.227 |
| PWY0.1319 | CDP-diacylglycerol biosynthesis I | 0 | Not detected | -0.092 | -0.293 | 0.110 | 0.371 |
| PWY0.1324 | <i>N</i>-acetylneuraminate and < | 0 | Not detected | -0.079 | -0.243 | 0.084 | 0.342 |
| PWY0.1337 | oleate &beta;-oxidation | 0 | Not detected | 0.202 | -0.043 | 0.447 | 0.107 |
| PWY0.1338 | polymyxin resistance | 0 | Not detected | 0.138 | -0.122 | 0.398 | 0.298 |
| PWY0.1415 | superpathway of heme <i>b</i> bi | 0 | Not detected | 0.000 | -0.259 | 0.260 | 0.997 |
| PWY0.1433 | tetrahydromonapterin biosynthesis | 0 | Not detected | -0.060 | -0.198 | 0.078 | 0.397 |
| PWY0.1465 | D-malate degradation | 0 | Not detected | -0.005 | -0.246 | 0.235 | 0.965 |
| PWY0.1466 | trehalose degradation VI (periplas | 0 | Not detected | 0.011 | -0.248 | 0.271 | 0.932 |
| PWY0.1471 | uracil degradation III | 0 | Not detected | 0.172 | -0.055 | 0.398 | 0.137 |
| PWY0.1477 | ethanolamine utilization | 0 | Not detected | -0.035 | -0.107 | 0.038 | 0.346 |
| PWY0.1479 | tRNA processing | 0 | Not detected | 0.107 | -0.165 | 0.378 | 0.441 |
| PWY0.1533 | methylphosphonate degradation I | 0 | Not detected | 0.078 | -0.168 | 0.323 | 0.536 |
| PWY0.1545 | cardiolipin biosynthesis III | 0 | Not detected | 0.204 | -0.052 | 0.459 | 0.118 |
| PWY0.1546 | muropeptide degradation | 0 | Not detected | 0.044 | -0.180 | 0.268 | 0.700 |
| PWY0.1565 | D-lactate to cytochrome <i>bo</i> | 0 | Not detected | 0.080 | -0.145 | 0.305 | 0.485 |
| PWY0.1567 | NADH to cytochrome <i>bo</i> o | 0 | Not detected | 0.027 | -0.182 | 0.236 | 0.798 |
| PWY0.1568 | NADH to cytochrome <i>bd</i> o | 0 | Not detected | 0.027 | -0.182 | 0.236 | 0.798 |
| PWY0.1569 | autoinducer AI-2 degradation | 0 | Not detected | 0.170 | -0.050 | 0.390 | 0.129 |
| PWY0.1573 | nitrate reduction VIIIb (dissimilat | 0 | Not detected | 0.027 | -0.182 | 0.236 | 0.798 |
| PWY0.1585 | formate to nitrite electron transfer | 0 | Not detected | 0.016 | -0.219 | 0.252 | 0.893 |
| PWY0.1586 | peptidoglycan maturation (<i>mes | 0 | Not detected | 0.181 | -0.142 | 0.504 | 0.272 |
| PWY0.1590 | NADH to hydrogen peroxide elect | 0 | Not detected | 0.190 | -0.050 | 0.430 | 0.121 |
| PWY0.1591 | glycerol-3-phosphate to hydrogen | 0 | Not detected | 0.190 | -0.050 | 0.430 | 0.121 |
| PWY0.1597 | prenylated FMNH<sub>2</sub> b | 0 | Not detected | 0.049 | -0.145 | 0.242 | 0.620 |
| PWY0.166 | superpathway of pyrimidine deoxy | 0 | Not detected | -0.075 | -0.181 | 0.031 | 0.166 |
| PWY0.301 | L-ascorbate degradation I (bacteria | 0 | Not detected | -0.047 | -0.124 | 0.030 | 0.233 |
| PWY0.321 | phenylacetate degradation I (aerob | 0 | Not detected | 0.066 | -0.064 | 0.195 | 0.319 |
| PWY0.42 | 2-methylcitrate cycle I | 0 | Not detected | 0.488 | 0.197 | 0.779 | 0.001 |
| PWY0.43 | conversion of succinate to propano | 0 | Not detected | -0.008 | -0.183 | 0.167 | 0.929 |
| PWY0.44 | D-allose degradation | 0 | Not detected | -0.199 | -0.478 | 0.081 | 0.164 |
| PWY0.461 | L-lysine degradation I | 0 | Not detected | 0.079 | -0.163 | 0.320 | 0.524 |
| PWY0.501 | lipoate biosynthesis and incorpora | 0 | Not detected | 0.019 | -0.187 | 0.225 | 0.856 |
| PWY0.522 | lipoate salvage I | 0 | Not detected | 0.109 | -0.119 | 0.337 | 0.350 |
| PWY0.662 | PRPP biosynthesis | 0 | Not detected | 0.009 | -0.109 | 0.126 | 0.887 |
| PWY0.862 | (5Z)-dodecenoate biosynthesis I | 0 | Not detected | -0.069 | -0.180 | 0.042 | 0.224 |

| PWY1.3 | polyhydroxybutanoate biosynthesi | 0 | Not detected | 0.033 | -0.170 | 0.236 | 0.749 |
| --- | --- | --- | --- | --- | --- | --- | --- |
| PWY3O.4107 | NAD salvage pathway V (PNC V | 0 | Not detected | 0.059 | -0.175 | 0.293 | 0.623 |
| PWY4FS.7 | phosphatidylglycerol biosynthesis | 0 | Not detected | 0.150 | -0.086 | 0.387 | 0.213 |
| PWY4FS.8 | phosphatidylglycerol biosynthesis | 0 | Not detected | 0.150 | -0.086 | 0.387 | 0.213 |
| PWY4LZ.257 | superpathway of fermentation (<i> | 0 | Not detected | -0.035 | -0.107 | 0.037 | 0.344 |
| PWY66.161 | ethanol degradation III | 0 | Not detected | 0.392 | 0.141 | 0.642 | 0.002 |
| PWY66.162 | ethanol degradation IV | 0 | Not detected | 0.392 | 0.141 | 0.642 | 0.002 |
| PWY66.21 | ethanol degradation II | 0 | Not detected | 0.212 | -0.054 | 0.478 | 0.119 |
| PWY66.366 | flavin biosynthesis IV (mammalia | 0 | Not detected | 0.113 | -0.127 | 0.354 | 0.355 |
| PWY66.391 | fatty acid &beta;-oxidation VI (pe | 0 | Not detected | 0.233 | -0.071 | 0.538 | 0.134 |
| PWY66.398 | TCA cycle III (animals) | 0 | Not detected | -0.077 | -0.181 | 0.028 | 0.150 |
| PWY66.399 | gluconeogenesis III | 0 | Not detected | -0.051 | -0.141 | 0.039 | 0.266 |
| PWY66.422 | D-galactose degradation V (Leloir | 0 | Not detected | -0.051 | -0.136 | 0.033 | 0.234 |
| PWY66.426 | hydrogen sulfide biosynthesis II ( | 0 | Not detected | 0.021 | -0.208 | 0.250 | 0.858 |
| PYRIDNUCS | NAD salvage pathway I (PNC VI | 0 | Not detected | 0.050 | -0.186 | 0.286 | 0.677 |
| PYRIDNUCS | NAD <i>de novo</i> biosynthesis | 0 | Not detected | 0.136 | -0.167 | 0.440 | 0.379 |
| PYRIDOXSY | pyridoxal 5'-phosphate biosynthes | 0 | Not detected | -0.100 | -0.400 | 0.200 | 0.515 |
| PYRUVDEH | NA | 0 | Not detected | -0.050 | -0.305 | 0.205 | 0.700 |
| PYRUVDEH | pyruvate decarboxylation to acetyl | 0 | Not detected | -0.050 | -0.305 | 0.205 | 0.700 |
| QUINATEDE | quinate degradation I | 0 | Not detected | -0.203 | -0.428 | 0.023 | 0.079 |
| REDCITCYC | TCA cycle VIII (Helicobacter) | 0 | Not detected | -0.077 | -0.179 | 0.025 | 0.141 |
| RHAMCAT.P | L-rhamnose degradation I | 8 | Not detected | 0.272 | 0.044 | 0.500 | 0.019 |
| RIBOSYN2.P | flavin biosynthesis I (bacteria and | 0 | Not detected | -0.040 | -0.130 | 0.050 | 0.382 |
| RUMP.PWY | formaldehyde oxidation I | 0 | Not detected | -0.050 | -0.136 | 0.036 | 0.255 |
| RXN.12588 | NA | 0 | Not detected | 0.120 | -0.138 | 0.378 | 0.363 |
| RXN.13163 | NA | 0 | Not detected | 0.044 | -0.256 | 0.344 | 0.774 |
| RXN.14047 | NA | 0 | Not detected | -0.085 | -0.196 | 0.025 | 0.131 |
| RXN.14201 | NA | 0 | Not detected | -0.022 | -0.087 | 0.044 | 0.514 |
| RXN.14249 | NA | 0 | Not detected | 0.273 | 0.018 | 0.527 | 0.036 |
| RXN.14314 | NA | 0 | Not detected | -0.202 | -0.446 | 0.042 | 0.105 |
| RXN.17127 | NA | 0 | Not detected | 0.109 | -0.119 | 0.337 | 0.350 |
| RXN.20025 | NA | 0 | Not detected | 0.034 | -0.170 | 0.238 | 0.741 |
| RXN0.1141 | NA | 0 | Not detected | 0.104 | -0.089 | 0.297 | 0.292 |
| RXN0.6511 | NA | 0 | Not detected | 0.235 | -0.012 | 0.483 | 0.062 |
| SERSYN.PW | L-serine biosynthesis I | 0 | Not detected | -0.007 | -0.111 | 0.097 | 0.894 |
| SUCROSEU | sucrose degradation VII (sucrose 3 | 0 | Not detected | 0.209 | -0.042 | 0.460 | 0.103 |
| SUCSYN.PW | sucrose biosynthesis I (from photo | 0 | Not detected | -0.051 | -0.141 | 0.040 | 0.272 |
| SUCUTIL.P | sucrose degradation I (sucrose pho | 0 | Not detected | 0.058 | -0.184 | 0.301 | 0.639 |
| SULFMETII. | assimilatory sulfate reduction II | 0 | Not detected | 0.162 | -0.136 | 0.460 | 0.287 |
| TCA | TCA cycle I (prokaryotic) | 0 | Not detected | -0.071 | -0.166 | 0.024 | 0.144 |
| TEICHOICA | poly(glycerol phosphate) wall teic | 0 | Not detected | -0.129 | -0.262 | 0.003 | 0.056 |
| THRDLCTC | L-threonine degradation III (to me | 0 | Not detected | 0.273 | 0.018 | 0.527 | 0.036 |
| THREONINE | L-threonine degradation II | 0 | Not detected | 0.273 | 0.018 | 0.527 | 0.036 |
| TREDEGLO | trehalose degradation I (low osmo | 0 | Not detected | -0.065 | -0.181 | 0.051 | 0.272 |

| TRESYN.PW | trehalose biosynthesis I | 0 | Not detected | 0.088 | -0.137 | 0.313 | 0.445 |
| --- | --- | --- | --- | --- | --- | --- | --- |
| TRIGLSYN.P | diacylglycerol and triacylglycerol | 0 | Not detected | -0.002 | -0.280 | 0.275 | 0.988 |
| TRNA.CHAR | tRNA charging | 0 | Not detected | -0.025 | -0.086 | 0.036 | 0.421 |
| TRPSYN.PW | L-tryptophan biosynthesis | 0 | Not detected | 0.165 | -0.123 | 0.454 | 0.261 |
| TRYPSYN.R | NA | 0 | Not detected | 0.032 | -0.216 | 0.280 | 0.798 |
| UDPNACET | UDP-<i>N</i>-acetyl-D-glucosam | 0 | Not detected | -0.057 | -0.150 | 0.037 | 0.235 |
| UDPNAGSY | UDP-<i>N</i>-acetyl-D-glucosam | 0 | Not detected | -0.050 | -0.140 | 0.040 | 0.276 |
| VALSYN.PW | L-valine biosynthesis | 0 | Not detected | -0.070 | -0.175 | 0.035 | 0.190 |
| X1.1.1.39.RX | NA | 0 | Not detected | 0.236 | -0.048 | 0.519 | 0.103 |
| X1CMET2.P | <i>N</i><sup>10</sup>-formyl-t | 0 | Not detected | -0.045 | -0.135 | 0.045 | 0.325 |
| X3.HYDROX | 4-hydroxyphenylacetate degradati | 0 | Not detected | 0.126 | -0.117 | 0.369 | 0.310 |

| **Supplementary Table 14.** Association of current economic hardship with enzymatic reactions. Analysis of Compositions of Microbiomes (ANCOM2) was used to identify enzymatic reactions related to current economic hardship. Regression coefficients were obtained by modelling the effect of current economic hardship on centered log ratio (CLR)-transformed species in multivariable linear regression adjusted for age, gender, BMI, field center, Hispanic/Latino background, U.S. nativity, Bristol stool type, parental education, the alternative Healthy Eating Index 2010 score, cigarette smoking, alcohol use, depressive and anxiety symptoms, diabetes, hypertension, cardiovascular disease, dyslipidemia, and antibiotic use. | | | | | | | |
| --- | --- | --- | --- | --- | --- | --- | --- |
| **otu.names** | **V2** | **W_stat** | **ANCOM** | **beta** | **l95ci** | **u95ci** | **qval** |
| ACETATEKI | acetate kinase | 0 | Not detected | 0.054 | -0.184 | 0.292 | 0.658 |
| ACS.ENZRX | acetyl-CoA synthetase (AMP-f | 0 | Not detected | 0.458 | 0.176 | 0.740 | 0.002 |
| ACYLACPS | acyl-ACP synthetase | 0 | Not detected | 0.077 | -0.183 | 0.337 | 0.559 |
| ACYLCOAD | acyl-CoA dehydrogenase | 0 | Not detected | 0.144 | -0.116 | 0.404 | 0.278 |
| ACYLGPEA | 2-acylglycerophosphoethanola | 0 | Not detected | 0.077 | -0.183 | 0.337 | 0.559 |
| ADENYL.KI | adenylate kinase | 0 | Not detected | -0.122 | -0.280 | 0.036 | 0.131 |
| ADENYLAT | adenylate cyclase | 0 | Not detected | 0.084 | -0.200 | 0.368 | 0.562 |
| ADOMET.D | S-adenosylmethionine:2-demet | 0 | Not detected | 0.029 | -0.277 | 0.334 | 0.855 |
| AICARTRAN | AICAR transformylase | 0 | Not detected | 0.154 | -0.055 | 0.363 | 0.150 |
| ALDOSE1EP | galactose-1-epimerase | 0 | Not detected | 0.147 | -0.072 | 0.366 | 0.190 |
| ALPHA.AM | Î±-amylase | 0 | Not detected | 0.031 | -0.183 | 0.246 | 0.775 |
| ALPHA.AM | Î±-amylase | 0 | Not detected | 0.223 | -0.040 | 0.486 | 0.097 |
| ALTRO.OXI | tagaturonate reductase | 0 | Not detected | -0.019 | -0.249 | 0.210 | 0.868 |
| ALTRODEH | D-altronate dehydratase | 0 | Not detected | -0.106 | -0.257 | 0.045 | 0.168 |
| AMYLOMA | amylomaltase | 0 | Not detected | 0.216 | -0.054 | 0.487 | 0.117 |
| ARGS.ENZR | arginine&mdash;tRNA ligase | 0 | Not detected | 0.097 | -0.192 | 0.385 | 0.511 |
| ARGSUCCIN | argininosuccinate lyase | 0 | Not detected | -0.120 | -0.290 | 0.050 | 0.167 |
| ARGSUCCT | arginine N-succinyltransferase | 0 | Not detected | 0.136 | -0.068 | 0.341 | 0.192 |
| BIOTINLIG. | biotin-[acetyl-CoA-carboxylas | 0 | Not detected | -0.056 | -0.272 | 0.160 | 0.613 |
| CARDIOLIP | cardiolipin synthase | 0 | Not detected | 0.137 | -0.076 | 0.350 | 0.209 |
| CDPDIGLYP | CDP-diacylglycerol diphospha | 0 | Not detected | -0.036 | -0.228 | 0.156 | 0.711 |
| CDPDIGLYS | CTP:2,3,4-saturated L-phospha | 0 | Not detected | 0.198 | 0.008 | 0.388 | 0.042 |
| CHD.ENZRX | choline dehydrogenase | 0 | Not detected | 0.133 | -0.123 | 0.390 | 0.308 |
| CMPKI.ENZ | CMP kinase | 0 | Not detected | 0.299 | 0.036 | 0.561 | 0.026 |
| CPM.KDOSY | 3-deoxy-manno-octulosonate c | 0 | Not detected | 0.066 | -0.243 | 0.375 | 0.675 |
| CROBETRE | crotonobetainyl-CoA reductase | 0 | Not detected | 0.219 | -0.038 | 0.476 | 0.095 |
| CYCPHOSD | 2',3'-cyclic nucleotide 2'-phosp | 0 | Not detected | 0.106 | -0.182 | 0.395 | 0.470 |
| CYSS.ENZR | cysteine&mdash;tRNA ligase | 0 | Not detected | -0.047 | -0.351 | 0.257 | 0.762 |
| CYTIDINEK | cytidine kinase | 0 | Not detected | -0.049 | -0.349 | 0.251 | 0.749 |
| D.PPENTOM | deoxyribose 1,5-phosphomutas | 0 | Not detected | -0.006 | -0.337 | 0.326 | 0.972 |
| DALADALA | D-alanine&mdash;D-alanine li | 0 | Not detected | 0.069 | -0.143 | 0.282 | 0.523 |
| DARABKIN. | D-ribulokinase | 0 | Not detected | 0.210 | -0.107 | 0.526 | 0.194 |
| DEHYDDEO | 2-dehydro-3-deoxygalactonoki | 0 | Not detected | 0.129 | -0.092 | 0.350 | 0.254 |
| DEHYDDEO | 2-dehydro-3-deoxy-6-phospho | 0 | Not detected | -0.050 | -0.251 | 0.151 | 0.625 |
| DEOXYGLU | 2-dehydro-3-deoxygluconokin | 0 | Not detected | 0.173 | -0.039 | 0.386 | 0.111 |
| DEOXYRIB | deoxyribose-phosphate aldolas | 0 | Not detected | 0.063 | -0.131 | 0.258 | 0.522 |
| DIENOYLCO | 2,4-dienoyl-CoA reductase | 0 | Not detected | 0.180 | -0.088 | 0.449 | 0.189 |

| DIHYDROF | dihydrofolate synthetase | 0 | Not detected | -0.021 | -0.248 | 0.206 | 0.859 |
| --- | --- | --- | --- | --- | --- | --- | --- |
| DIHYDROO | dihydroorotate dehydrogenase | 0 | Not detected | 0.179 | -0.162 | 0.520 | 0.304 |
| DLACTDEH | D-lactate dehydrogenase (quio | 0 | Not detected | 0.061 | -0.174 | 0.297 | 0.610 |
| DMK.ENZR | 1,4-dihydroxy-2-naphthoate oc | 0 | Not detected | -0.013 | -0.244 | 0.218 | 0.913 |
| DSERDEAM | D-serine ammonia-lyase | 0 | Not detected | -0.135 | -0.404 | 0.134 | 0.324 |
| DTDPDEHY | dTDP-4-dehydrorhamnose 3,5- | 0 | Not detected | -0.098 | -0.266 | 0.070 | 0.253 |
| DTDPGLUC | dTDP-glucose 4,6-dehydratase | 0 | Not detected | -0.094 | -0.262 | 0.074 | 0.275 |
| ENTC.ENZR | isochorismate synthase | 0 | Not detected | 0.161 | -0.045 | 0.367 | 0.126 |
| ENZRXN.10 | L-cysteine-S-conjugate thiol-ly | 0 | Not detected | -0.067 | -0.342 | 0.207 | 0.630 |
| ENZRXN.11 | imidazolone-5-propionate hydr | 0 | Not detected | 0.079 | -0.209 | 0.367 | 0.589 |
| ENZRXN.11 | ammonia-dependent carbamoyl | 0 | Not detected | -0.087 | -0.317 | 0.143 | 0.457 |
| ENZRXN.11 | cystathionine &gamma;-lyase | 0 | Not detected | -0.274 | -0.590 | 0.042 | 0.089 |
| ENZRXN.12 | citrate synthase | 0 | Not detected | -0.102 | -0.486 | 0.281 | 0.601 |
| ENZRXN.12 | citrate hydro-lyase (cis-aconita | 0 | Not detected | -0.055 | -0.420 | 0.310 | 0.768 |
| ENZRXN.12 | D-threo-isocitrate hydro-lyase | 0 | Not detected | -0.055 | -0.420 | 0.310 | 0.768 |
| ENZRXN.12 | malate dehydrogenase | 0 | Not detected | 0.264 | -0.093 | 0.621 | 0.148 |
| ENZRXN.12 | 1-deoxy-D-xylulose 5-phospha | 0 | Not detected | -0.047 | -0.249 | 0.155 | 0.647 |
| ENZRXN.12 | Pfl-activating enzyme | 0 | Not detected | -0.240 | -0.552 | 0.072 | 0.132 |
| ENZRXN.12 | phenylacetate-CoA ligase | 0 | Not detected | -0.032 | -0.224 | 0.159 | 0.740 |
| ENZRXN.12 | xylitol dehydrogenase | 0 | Not detected | 0.158 | -0.187 | 0.504 | 0.370 |
| ENZRXN.12 | UDP-N-acetylenolpyruvoylglu | 0 | Not detected | -0.008 | -0.293 | 0.278 | 0.959 |
| ENZRXN.12 | UDP-N-acetylmuramoyl-L-ala | 0 | Not detected | 0.111 | -0.258 | 0.479 | 0.556 |
| ENZRXN.12 | 3,5-diaminohexanoate dehydro | 0 | Not detected | 0.089 | -0.222 | 0.401 | 0.574 |
| ENZRXN.12 | 3-aminobutyryl-CoA deaminas | 0 | Not detected | -0.128 | -0.460 | 0.204 | 0.451 |
| ENZRXN.13 | thiosulfate reductase (cytochro | 0 | Not detected | -0.087 | -0.389 | 0.215 | 0.572 |
| ENZRXN.13 | Enzyme IIscr | 0 | Not detected | -0.242 | -0.574 | 0.089 | 0.152 |
| ENZRXN.13 | Enzyme IIscr | 0 | Not detected | -0.083 | -0.339 | 0.173 | 0.526 |
| ENZRXN.13 | fructokinase | 0 | Not detected | 0.018 | -0.201 | 0.237 | 0.873 |
| ENZRXN.13 | phosphoribosyl-formimino-5-a | 0 | Not detected | -0.120 | -0.324 | 0.084 | 0.249 |
| ENZRXN.13 | imidazoleglycerolphosphate de | 0 | Not detected | -0.191 | -0.466 | 0.084 | 0.174 |
| ENZRXN.13 | sucrose phosphorylase | 0 | Not detected | -0.106 | -0.489 | 0.276 | 0.586 |
| ENZRXN.13 | phosphoserine aminotransferas | 0 | Not detected | -0.072 | -0.249 | 0.106 | 0.430 |
| ENZRXN.13 | phosphoglucose isomerase | 0 | Not detected | 0.078 | -0.251 | 0.407 | 0.641 |
| ENZRXN.13 | 3-phosphoglycerate kinase | 0 | Not detected | -0.125 | -0.305 | 0.055 | 0.174 |
| ENZRXN.13 | myo-inosose-2 dehydratase | 0 | Not detected | 0.107 | -0.163 | 0.377 | 0.437 |
| ENZRXN.13 | glucose-6-phosphate isomerase | 0 | Not detected | -0.206 | -0.543 | 0.130 | 0.230 |
| ENZRXN.13 | fructokinase | 0 | Not detected | 0.085 | -0.195 | 0.366 | 0.552 |
| ENZRXN.13 | glucose-6-phosphate isomerase | 0 | Not detected | -0.106 | -0.269 | 0.056 | 0.200 |
| ENZRXN.13 | phosphotransacetylase | 0 | Not detected | -0.039 | -0.289 | 0.211 | 0.760 |
| ENZRXN.14 | UDP-N-acetylglucosamine 4-e | 0 | Not detected | 0.044 | -0.350 | 0.438 | 0.828 |
| ENZRXN.14 | UDP-galactose 4-epimerase | 0 | Not detected | 0.044 | -0.350 | 0.438 | 0.828 |
| ENZRXN.14 | cobalt precorrin-4 (C11)-methy | 0 | Not detected | -0.067 | -0.243 | 0.110 | 0.460 |
| ENZRXN.14 | cobalt-precorrin-3 (C17)-meth | 0 | Not detected | -0.282 | -0.592 | 0.028 | 0.075 |
| ENZRXN.14 | GDP-D-mannose pyrophospho | 0 | Not detected | 0.114 | -0.168 | 0.396 | 0.430 |

| ENZRXN.14 | phosphomannose isomerase | 0 | Not detected | 0.114 | -0.168 | 0.396 | 0.430 |
| --- | --- | --- | --- | --- | --- | --- | --- |
| ENZRXN.14 | succinyl-CoA:CoA transferase | 0 | Not detected | -0.146 | -0.416 | 0.124 | 0.288 |
| ENZRXN.14 | 4-hydroxybutyrate coenzyme A | 0 | Not detected | -0.220 | -0.547 | 0.106 | 0.187 |
| ENZRXN.14 | crotonase | 0 | Not detected | 0.092 | -0.256 | 0.440 | 0.606 |
| ENZRXN.14 | butyryl-CoA dehydrogenase | 0 | Not detected | -0.196 | -0.462 | 0.070 | 0.149 |
| ENZRXN.14 | phosphate acetyltransferase | 0 | Not detected | -0.110 | -0.278 | 0.059 | 0.203 |
| ENZRXN.14 | acetate kinase | 0 | Not detected | -0.264 | -0.568 | 0.040 | 0.089 |
| ENZRXN.14 | GDP-Î±-D-mannose 4,6-dehyd | 0 | Not detected | -0.199 | -0.514 | 0.116 | 0.216 |
| ENZRXN.14 | aconitase | 0 | Not detected | -0.036 | -0.240 | 0.167 | 0.726 |
| ENZRXN.14 | phosphoenolpyruvate phospho | 0 | Not detected | -0.303 | -0.672 | 0.065 | 0.107 |
| ENZRXN.14 | naphthoyl-CoA synthase | 0 | Not detected | -0.174 | -0.359 | 0.010 | 0.064 |
| ENZRXN.14 | N-acetylhexosamine kinase | 0 | Not detected | -0.280 | -0.623 | 0.062 | 0.109 |
| ENZRXN.14 | glutamyl-tRNAGln synthetase | 0 | Not detected | 0.034 | -0.315 | 0.383 | 0.849 |
| ENZRXN.15 | glutamyl-tRNAGlu synthetase | 0 | Not detected | 0.034 | -0.315 | 0.383 | 0.849 |
| ENZRXN.15 | 1-acylglycerol-3-phosphate acy | 0 | Not detected | -0.059 | -0.321 | 0.202 | 0.656 |
| ENZRXN.15 | biotin sulfoxide reductase | 0 | Not detected | 0.194 | -0.073 | 0.462 | 0.155 |
| ENZRXN.15 | malonate decarboxylase acetyl- | 0 | Not detected | -0.037 | -0.269 | 0.196 | 0.758 |
| ENZRXN.15 | acyl-carrier protein: acetate lig | 0 | Not detected | -0.190 | -0.536 | 0.156 | 0.283 |
| ENZRXN.15 | acetyl-[acyl-carrier protein]:ma | 0 | Not detected | -0.073 | -0.407 | 0.262 | 0.671 |
| ENZRXN.15 | carboxybiotin decarboxylase | 0 | Not detected | -0.069 | -0.338 | 0.199 | 0.613 |
| ENZRXN.15 | N-succinylornithine carbamoyl | 0 | Not detected | -0.136 | -0.327 | 0.055 | 0.163 |
| ENZRXN.15 | 5-monosubstituted-hydantoin r | 0 | Not detected | -0.045 | -0.371 | 0.282 | 0.788 |
| ENZRXN.15 | phosphoglycerate mutase | 0 | Not detected | -0.167 | -0.500 | 0.167 | 0.327 |
| ENZRXN.15 | 2-keto-3-deoxy-D-glycero-D-g | 0 | Not detected | -0.204 | -0.600 | 0.191 | 0.311 |
| ENZRXN.15 | 2-keto-3-deoxy-D-glycero-D-g | 0 | Not detected | -0.091 | -0.459 | 0.276 | 0.626 |
| ENZRXN.16 | Î²-D-galactosyl-(1->4)-L-rham | 0 | Not detected | -0.306 | -0.672 | 0.060 | 0.102 |
| ENZRXN.17 | UDP-N-acetylglucosamine eno | 0 | Not detected | -0.008 | -0.358 | 0.341 | 0.963 |
| ENZRXN.17 | UDP-galactopyranose mutase | 0 | Not detected | -0.116 | -0.496 | 0.265 | 0.552 |
| ENZRXN.17 | 3-dehydroquinate dehydratase | 0 | Not detected | -0.321 | -0.614 | -0.029 | 0.032 |
| ENZRXN.17 | UDP-N-acetylglucosamine--N- | 0 | Not detected | -0.176 | -0.392 | 0.040 | 0.110 |
| ENZRXN.17 | UDP-N-acetylmuramoyl--L-ala | 0 | Not detected | -0.123 | -0.438 | 0.192 | 0.444 |
| ENZRXN.17 | UDP-N-acetylglucosamine 1-c | 0 | Not detected | -0.273 | -0.521 | -0.025 | 0.032 |
| ENZRXN.17 | UDP-N-acetylglucosamine 1-c | 0 | Not detected | 0.006 | -0.211 | 0.223 | 0.956 |
| ENZRXN.17 | undecaprenyl-pyrophosphoryl- | 0 | Not detected | -0.034 | -0.396 | 0.329 | 0.854 |
| ENZRXN.17 | asparagine synthase | 0 | Not detected | -0.152 | -0.516 | 0.213 | 0.414 |
| ENZRXN.17 | undecaprenyl-pyrophosphoryl- | 0 | Not detected | -0.034 | -0.396 | 0.329 | 0.854 |
| ENZRXN.17 | D-sedoheptulose 7-phosphate i | 0 | Not detected | 0.086 | -0.244 | 0.417 | 0.608 |
| ENZRXN.17 | D-glycero-Î±-D-manno-heptos | 0 | Not detected | -0.196 | -0.563 | 0.170 | 0.294 |
| ENZRXN.17 | methylthioalkylmalate dehydro | 0 | Not detected | -0.100 | -0.281 | 0.082 | 0.283 |
| ENZRXN.17 | methylthioalkylmalate dehydro | 0 | Not detected | -0.100 | -0.281 | 0.082 | 0.283 |
| ENZRXN.17 | methylthioalkylmalate dehydro | 0 | Not detected | -0.100 | -0.281 | 0.082 | 0.283 |
| ENZRXN.17 | methylthioalkylmalate dehydro | 0 | Not detected | -0.100 | -0.281 | 0.082 | 0.283 |
| ENZRXN.17 | methylthioalkylmalate dehydro | 0 | Not detected | -0.100 | -0.281 | 0.082 | 0.283 |
| ENZRXN.17 | lipoate-ATP adenylate transfer | 0 | Not detected | 0.048 | -0.148 | 0.245 | 0.629 |

| ENZRXN.17 | pimeloyl-[acp] methyl ester est | 0 | Not detected | 0.038 | -0.162 | 0.238 | 0.707 |
| --- | --- | --- | --- | --- | --- | --- | --- |
| ENZRXN.17 | (2Z,6E)-farnesyl diphosphate m | 0 | Not detected | -0.033 | -0.387 | 0.321 | 0.854 |
| ENZRXN.18 | 3-(hydroxyamino)phenol muta | 0 | Not detected | 0.245 | -0.108 | 0.599 | 0.174 |
| ENZRXN.18 | butanal dehydrogenase | 0 | Not detected | -0.123 | -0.301 | 0.054 | 0.174 |
| ENZRXN.18 | butanol dehydrogenase | 0 | Not detected | -0.123 | -0.301 | 0.054 | 0.174 |
| ENZRXN.18 | glycerol-2-phosphotransferase | 0 | Not detected | -0.129 | -0.420 | 0.162 | 0.384 |
| ENZRXN.18 | DsrC-trisulfide reductase | 0 | Not detected | -0.091 | -0.409 | 0.226 | 0.572 |
| ENZRXN.18 | dCMP kinase | 0 | Not detected | 0.299 | 0.036 | 0.561 | 0.026 |
| ENZRXN.18 | 3-hydroxy-trans-cinnamate hyd | 0 | Not detected | 0.238 | 0.009 | 0.467 | 0.042 |
| ENZRXN.18 | 1-deoxy-D-xylulose 5-phospha | 0 | Not detected | -0.582 | -0.933 | -0.231 | 0.001 |
| ENZRXN.18 | nitric oxide reductase | 0 | Not detected | -0.119 | -0.379 | 0.140 | 0.367 |
| ENZRXN.18 | rhamnogalacturonan endolyase | 0 | Not detected | -0.009 | -0.306 | 0.287 | 0.951 |
| ENZRXN.18 | unsaturated chondroitin 6-sulfa | 0 | Not detected | -0.150 | -0.443 | 0.143 | 0.315 |
| ENZRXN.18 | legionaminate synthase | 0 | Not detected | -0.061 | -0.365 | 0.243 | 0.695 |
| ENZRXN.18 | rhamnogalacturonan exolyase | 0 | Not detected | -0.132 | -0.435 | 0.171 | 0.393 |
| ENZRXN.18 | isoamylase | 0 | Not detected | -0.121 | -0.445 | 0.203 | 0.465 |
| ENZRXN.18 | formate dehydrogenase | 0 | Not detected | -0.066 | -0.370 | 0.238 | 0.672 |
| ENZRXN.19 | cellobiose phosphorylase | 0 | Not detected | -0.107 | -0.299 | 0.085 | 0.275 |
| ENZRXN.19 | GTP 3',8'-cyclase | 0 | Not detected | 0.029 | -0.197 | 0.255 | 0.802 |
| ENZRXN.19 | endo-Î±-N-acetylgalactosamini | 0 | Not detected | -0.149 | -0.523 | 0.224 | 0.434 |
| ENZRXN.19 | trans-2-enoyl-CoA reductase ( | 0 | Not detected | -0.183 | -0.466 | 0.099 | 0.203 |
| ENZRXN.19 | crotonyl-CoA reductase | 0 | Not detected | -0.183 | -0.466 | 0.099 | 0.203 |
| ENZRXN.19 | trans-2-hexenoyl-CoA reductas | 0 | Not detected | -0.183 | -0.466 | 0.099 | 0.203 |
| ENZRXN.19 | hexanal dehydrogenase | 0 | Not detected | -0.123 | -0.301 | 0.054 | 0.174 |
| ENZRXN.19 | hexanol dehydrogenase | 0 | Not detected | -0.123 | -0.301 | 0.054 | 0.174 |
| ENZRXN.19 | carboxy-adenylated-[ThiS sulf | 0 | Not detected | -0.050 | -0.309 | 0.209 | 0.706 |
| ENZRXN.19 | molybdopterin synthase sulfurt | 0 | Not detected | 0.064 | -0.138 | 0.265 | 0.535 |
| ENZRXN.19 | N,N'-diacetylchitobiose phosph | 0 | Not detected | -0.166 | -0.497 | 0.165 | 0.326 |
| ENZRXN.19 | 12Î±-hydroxy-3-oxochol-4,6-d | 0 | Not detected | -0.236 | -0.561 | 0.089 | 0.154 |
| ENZRXN.19 | dTDP-glucose 4,6-dehydratase | 0 | Not detected | -0.169 | -0.455 | 0.116 | 0.245 |
| ENZRXN.19 | glucose-1-phosphate thymidyly | 0 | Not detected | -0.149 | -0.318 | 0.020 | 0.085 |
| ENZRXN.19 | dTDP-3-amino-3,6-dideoxy-Î± | 0 | Not detected | -0.252 | -0.607 | 0.103 | 0.165 |
| ENZRXN.19 | 2-oxoglutarate decarboxylase | 0 | Not detected | -0.272 | -0.633 | 0.090 | 0.141 |
| ENZRXN.20 | deoxyribonuclease IV | 0 | Not detected | -0.004 | -0.235 | 0.227 | 0.974 |
| ENZRXN.20 | uracil-DNA glycosylase | 0 | Not detected | -0.071 | -0.224 | 0.083 | 0.367 |
| ENZRXN.20 | DNA glycosylase | 0 | Not detected | -0.128 | -0.443 | 0.188 | 0.427 |
| ENZRXN.20 | DNA-formamidopyrimidine gl | 0 | Not detected | 0.068 | -0.148 | 0.283 | 0.539 |
| ENZRXN.20 | adenine glycosylase | 0 | Not detected | 0.043 | -0.183 | 0.270 | 0.708 |
| ENZRXN.20 | glycerol-3-phosphate dehydrog | 0 | Not detected | -0.175 | -0.498 | 0.147 | 0.287 |
| ENZRXN.20 | 3-isopropylmalate dehydratase | 0 | Not detected | 0.034 | -0.342 | 0.410 | 0.858 |
| ENZRXN.20 | (S)-NADPHX dehydratase | 0 | Not detected | 0.110 | -0.136 | 0.355 | 0.382 |
| ENZRXN.20 | NADPHX epimerase | 0 | Not detected | 0.110 | -0.136 | 0.355 | 0.382 |
| ENZRXN.20 | butanol dehydrogenase | 0 | Not detected | -0.086 | -0.264 | 0.092 | 0.343 |
| ENZRXN.20 | CoA-dependent propionaldehy | 0 | Not detected | -0.008 | -0.282 | 0.267 | 0.957 |

| ENZRXN.20 | phosphate propanoyltransferas | 0 | Not detected | -0.373 | -0.659 | -0.087 | 0.011 |
| --- | --- | --- | --- | --- | --- | --- | --- |
| ENZRXN.20 | phosphate acetyltransferase | 0 | Not detected | -0.373 | -0.659 | -0.087 | 0.011 |
| ENZRXN.20 | propionate kinase | 0 | Not detected | 0.037 | -0.223 | 0.297 | 0.780 |
| ENZRXN.20 | thymine oxygenase | 0 | Not detected | 0.159 | -0.041 | 0.360 | 0.120 |
| ENZRXN.20 | hydroxybutyryl-coA dehydroge | 0 | Not detected | -0.082 | -0.303 | 0.140 | 0.471 |
| ENZRXN.20 | biotin carboxylase | 0 | Not detected | -0.117 | -0.420 | 0.187 | 0.451 |
| ENZRXN.20 | N,N'-diacetylbacilliosaminyl-1 | 0 | Not detected | -0.021 | -0.360 | 0.317 | 0.901 |
| ENZRXN.20 | carboxyspermidine dehydrogen | 0 | Not detected | -0.096 | -0.260 | 0.069 | 0.254 |
| ENZRXN.20 | N-carbamoylputrescine amidoh | 0 | Not detected | -0.131 | -0.323 | 0.061 | 0.182 |
| ENZRXN.20 | 4,4'-diaponeurosporenal dehyd | 0 | Not detected | -0.110 | -0.457 | 0.237 | 0.536 |
| ENZRXN.20 | superoxide dismutase | 0 | Not detected | -0.101 | -0.421 | 0.218 | 0.535 |
| ENZRXN.20 | UDP-N-acetyl-2-amino-2-deox | 0 | Not detected | 0.180 | -0.180 | 0.540 | 0.327 |
| ENZRXN.20 | UDP-2,3-diacetamido-2,3-dide | 0 | Not detected | -0.068 | -0.431 | 0.295 | 0.714 |
| ENZRXN.20 | enoyl-[acyl-carrier-protein] red | 0 | Not detected | -0.089 | -0.438 | 0.260 | 0.616 |
| ENZRXN.20 | enoyl-[acyl-carrier-protein] red | 0 | Not detected | -0.089 | -0.438 | 0.260 | 0.616 |
| ENZRXN.20 | crotonyl-[acyl-carrier-protein] | 0 | Not detected | -0.089 | -0.438 | 0.260 | 0.616 |
| ENZRXN.20 | D-fructose-6-phosphate cytidyl | 0 | Not detected | -0.146 | -0.486 | 0.195 | 0.402 |
| ENZRXN.21 | acetaldehyde dehydrogenase | 0 | Not detected | -0.242 | -0.531 | 0.047 | 0.101 |
| ENZRXN.21 | homoserine O-acetyltransferase | 0 | Not detected | -0.215 | -0.508 | 0.079 | 0.152 |
| ENZRXN.21 | methylthioribose-1-phosphate i | 0 | Not detected | 0.186 | -0.149 | 0.522 | 0.277 |
| ENZRXN.21 | oligosaccharide reducing-end x | 0 | Not detected | -0.159 | -0.421 | 0.103 | 0.235 |
| ENZRXN.21 | Î²-L-arabinofuranosidase | 0 | Not detected | -0.341 | -0.695 | 0.013 | 0.059 |
| ENZRXN.21 | uridine kinase | 0 | Not detected | -0.049 | -0.349 | 0.251 | 0.749 |
| ENZRXN.21 | cytidine kinase | 0 | Not detected | -0.049 | -0.349 | 0.251 | 0.749 |
| ENZRXN.21 | urocanate reductase | 0 | Not detected | 0.054 | -0.312 | 0.420 | 0.772 |
| ENZRXN.21 | 3D-(3,5/4)-trihydroxycyclohex | 0 | Not detected | 0.037 | -0.202 | 0.277 | 0.760 |
| ENZRXN.21 | myo-inositol 2-dehydrogenase | 0 | Not detected | -0.185 | -0.466 | 0.097 | 0.199 |
| ENZRXN.21 | D-mannonate dehydrogenase | 0 | Not detected | -0.086 | -0.276 | 0.105 | 0.378 |
| ENZRXN.21 | D-mannonate dehydratase | 0 | Not detected | -0.147 | -0.323 | 0.029 | 0.102 |
| ENZRXN.21 | 2-keto-3-deoxygluconate kinas | 0 | Not detected | -0.107 | -0.275 | 0.061 | 0.213 |
| ENZRXN.21 | [sulfur carrier protein CysO]--s | 0 | Not detected | 0.147 | -0.174 | 0.468 | 0.369 |
| ENZRXN.21 | N-acetyl-Î±-D-glucosaminyl-di | 0 | Not detected | -0.328 | -0.681 | 0.025 | 0.069 |
| ENZRXN.21 | glucose-1-phosphate thymidyly | 0 | Not detected | -0.053 | -0.321 | 0.215 | 0.700 |
| ENZRXN.21 | dTDP-glucose 4,6-dehydratase | 0 | Not detected | -0.089 | -0.359 | 0.182 | 0.522 |
| ENZRXN.21 | dTDP-4-amino-4,6-dideoxy-D- | 0 | Not detected | 0.011 | -0.365 | 0.387 | 0.953 |
| ENZRXN.21 | UDP-glucose 6-dehydrogenase | 0 | Not detected | -0.040 | -0.366 | 0.285 | 0.809 |
| ENZRXN.21 | UDP-N-acetylglucosamine 4,6 | 0 | Not detected | 0.046 | -0.260 | 0.351 | 0.768 |
| ENZRXN.21 | UDP-2-acetamido-2,6-dideoxy | 0 | Not detected | 0.146 | -0.212 | 0.504 | 0.423 |
| ENZRXN.21 | UDP-N-acetyl-Î²-L-rhamnosam | 0 | Not detected | -0.008 | -0.343 | 0.327 | 0.962 |
| ENZRXN.22 | UDP-N-acetylglucosamine 4,6 | 0 | Not detected | -0.263 | -0.595 | 0.069 | 0.121 |
| ENZRXN.22 | Î²-L-arabinobiosidase | 0 | Not detected | -0.202 | -0.609 | 0.205 | 0.331 |
| ENZRXN.22 | butyryl-CoA dehydrogenase | 0 | Not detected | -0.075 | -0.259 | 0.108 | 0.420 |
| ENZRXN.22 | phosphoenolpyruvate carboxyk | 0 | Not detected | -0.131 | -0.382 | 0.120 | 0.307 |
| ENZRXN.22 | phosphoenolpyruvate carboxyk | 0 | Not detected | -0.131 | -0.382 | 0.120 | 0.307 |

| ENZRXN.22 | L-tyrosine 3-monooxygenase | 0 | Not detected | -0.001 | -0.222 | 0.220 | 0.995 |
| --- | --- | --- | --- | --- | --- | --- | --- |
| ENZRXN.22 | 4-coumarate 3-monooxygenase | 0 | Not detected | -0.001 | -0.222 | 0.220 | 0.995 |
| ENZRXN.22 | 4-tyrosol 3-monooxygenase | 0 | Not detected | -0.001 | -0.222 | 0.220 | 0.995 |
| ENZRXN.22 | succinyl-CoA:acetate CoA-tran | 0 | Not detected | 0.065 | -0.299 | 0.429 | 0.728 |
| ENZRXN.22 | propionyl-CoA:succinate CoA | 0 | Not detected | 0.065 | -0.299 | 0.429 | 0.728 |
| ENZRXN.22 | N-acetyl-Î²-neuraminate:H+ sy | 0 | Not detected | -0.091 | -0.322 | 0.139 | 0.437 |
| ENZRXN.22 | 1,4-Î²-mannanase | 0 | Not detected | -0.014 | -0.341 | 0.313 | 0.934 |
| ENZRXN.22 | mannobiose 2-epimerase | 0 | Not detected | 0.018 | -0.268 | 0.304 | 0.903 |
| ENZRXN.22 | cellobiose 2-epimerase | 0 | Not detected | 0.018 | -0.268 | 0.304 | 0.903 |
| ENZRXN.22 | cellobiose 2-epimerase | 0 | Not detected | -0.247 | -0.560 | 0.066 | 0.123 |
| ENZRXN.22 | UDP-acetamido-4-amino-6-de | 0 | Not detected | -0.117 | -0.477 | 0.242 | 0.522 |
| ENZRXN.22 | 2-oxo-4-methylthiobutanoate-g | 0 | Not detected | -0.112 | -0.395 | 0.172 | 0.441 |
| ENZRXN.23 | CoA-dependent (R)-lactaldehy | 0 | Not detected | -0.008 | -0.282 | 0.267 | 0.957 |
| ENZRXN.23 | CoA-dependent (S)-lactaldehy | 0 | Not detected | -0.008 | -0.282 | 0.267 | 0.957 |
| ENZRXN.23 | (R)-lactaldehyde reductase | 0 | Not detected | 0.156 | -0.042 | 0.353 | 0.122 |
| ENZRXN.23 | (S)-lactaldehyde reductase | 0 | Not detected | 0.156 | -0.042 | 0.353 | 0.122 |
| ENZRXN.23 | (R)-2,3-dihydroxy-3-methylbut | 0 | Not detected | -0.105 | -0.272 | 0.062 | 0.217 |
| ENZRXN.23 | (R)-2,3-dihydroxy-3-methylpe | 0 | Not detected | -0.105 | -0.272 | 0.062 | 0.217 |
| ENZRXN.23 | (R)-2,3-dihydroxy-3-methylbut | 0 | Not detected | -0.181 | -0.492 | 0.131 | 0.256 |
| ENZRXN.23 | (R)-2,3-dihydroxy-3-methylpe | 0 | Not detected | -0.181 | -0.492 | 0.131 | 0.256 |
| ENZRXN.23 | Fe-coproporphyrin synthase | 0 | Not detected | 0.236 | -0.093 | 0.564 | 0.161 |
| ENZRXN.23 | bacimethrin diphosphokinase | 0 | Not detected | -0.186 | -0.411 | 0.039 | 0.106 |
| ENZRXN.23 | L-galactose dehydrogenase | 0 | Not detected | 0.003 | -0.269 | 0.275 | 0.982 |
| ENZRXN.23 | L-galactonate 5-dehydrogenase | 0 | Not detected | -0.083 | -0.356 | 0.189 | 0.548 |
| ENZRXN.23 | N-acetylneuraminate lyase | 0 | Not detected | -0.099 | -0.345 | 0.147 | 0.431 |
| ENZRXN.23 | N-acetylglucosamine kinase | 0 | Not detected | -0.129 | -0.312 | 0.053 | 0.166 |
| ENZRXN.23 | 7-oxolithocholate reductase | 0 | Not detected | -0.238 | -0.527 | 0.051 | 0.107 |
| ENZRXN.23 | 7-oxolithocholate reductase | 0 | Not detected | -0.238 | -0.527 | 0.051 | 0.107 |
| ENZRXN.23 | GDP-6-deoxy-4-keto-D-arabin | 0 | Not detected | -0.108 | -0.360 | 0.143 | 0.399 |
| ENZRXN.23 | UDP-N-acetylglucosamine 6-d | 0 | Not detected | -0.268 | -0.542 | 0.005 | 0.054 |
| ENZRXN.23 | UDP-N-acetylgalactosamine 6- | 0 | Not detected | -0.268 | -0.542 | 0.005 | 0.054 |
| ENZRXN.23 | Kdo2-lipid A phosphotransfera | 0 | Not detected | -0.032 | -0.300 | 0.236 | 0.813 |
| ENZRXN.23 | Kdo2-lipid A phosphoethanola | 0 | Not detected | 0.182 | -0.071 | 0.435 | 0.158 |
| ENZRXN.23 | phosphomannomutase | 0 | Not detected | 0.097 | -0.233 | 0.427 | 0.563 |
| ENZRXN.23 | phosphoglucomutase | 0 | Not detected | 0.097 | -0.233 | 0.427 | 0.563 |
| ENZRXN.23 | heparin lyase | 0 | Not detected | 0.207 | -0.243 | 0.656 | 0.367 |
| ENZRXN.23 | heparan sulfate lyase | 0 | Not detected | 0.207 | -0.243 | 0.656 | 0.367 |
| ENZRXN.23 | heparin lyase | 0 | Not detected | -0.108 | -0.440 | 0.225 | 0.526 |
| ENZRXN.23 | unsaturated hyaluronan disacch | 0 | Not detected | -0.150 | -0.443 | 0.143 | 0.315 |
| ENZRXN.23 | unsaturated chondroitin disacc | 0 | Not detected | -0.150 | -0.443 | 0.143 | 0.315 |
| ENZRXN.23 | 4-deoxy-Î²-L-erythro-hex-4-en | 0 | Not detected | 0.038 | -0.305 | 0.381 | 0.829 |
| ENZRXN.23 | 4-deoxy-Î²-D-gluc-4-enuronosy | 0 | Not detected | 0.038 | -0.305 | 0.381 | 0.829 |
| ENZRXN.23 | 4-deoxy-Î²-D-gluc-4-enuronosy | 0 | Not detected | 0.038 | -0.305 | 0.381 | 0.829 |
| ENZRXN.23 | 4-deoxy-Î²-L-erythro-hex-4-en | 0 | Not detected | 0.038 | -0.305 | 0.381 | 0.829 |

| ENZRXN.23 | (5Z)-3-oxo-tetradec-5-enoyl-[a | 0 | Not detected | -0.130 | -0.305 | 0.045 | 0.146 |
| --- | --- | --- | --- | --- | --- | --- | --- |
| ENZRXN.23 | (5Z)-3-oxo-tetradec-5-enoyl-[a | 0 | Not detected | -0.052 | -0.349 | 0.244 | 0.729 |
| ENZRXN.23 | (2E,5Z)-tetradeca-2,5-dienoyl- | 0 | Not detected | 0.012 | -0.254 | 0.279 | 0.928 |
| ENZRXN.23 | (3R)-3-hydroxydecanoyl-[acp] | 0 | Not detected | -0.205 | -0.453 | 0.042 | 0.105 |
| ENZRXN.23 | (2E)-dec-2-enoyl-[acp] isomer | 0 | Not detected | -0.205 | -0.453 | 0.042 | 0.105 |
| ENZRXN.23 | (5Z)-3-oxo-dodec-5-enoyl-[acp | 0 | Not detected | -0.102 | -0.271 | 0.067 | 0.239 |
| ENZRXN.23 | (7Z)-3-oxo-hexadec-7-enoyl-[a | 0 | Not detected | -0.130 | -0.305 | 0.045 | 0.146 |
| ENZRXN.23 | (9Z)-3-oxo-octadec-9-enoyl-[a | 0 | Not detected | -0.130 | -0.305 | 0.045 | 0.146 |
| ENZRXN.23 | (7Z)-3-oxo-hexadec-7-enoyl-[a | 0 | Not detected | -0.052 | -0.349 | 0.244 | 0.729 |
| ENZRXN.23 | (9Z)-3-oxo-octadec-9-enoyl-[a | 0 | Not detected | -0.052 | -0.349 | 0.244 | 0.729 |
| ENZRXN.23 | (11Z)-3-oxo-icos-11-enoyl-[ac | 0 | Not detected | -0.052 | -0.349 | 0.244 | 0.729 |
| ENZRXN.23 | (11Z)-3-oxo-icos-11-enoyl-[ac | 0 | Not detected | -0.130 | -0.305 | 0.045 | 0.146 |
| ENZRXN.23 | (2E,7Z)-hexadeca-2,7-dienoyl- | 0 | Not detected | 0.012 | -0.254 | 0.279 | 0.928 |
| ENZRXN.23 | (2E,9Z)-octadeca-2,9-dienoyl-[ | 0 | Not detected | 0.012 | -0.254 | 0.279 | 0.928 |
| ENZRXN.23 | (2E,11Z)-icosa-2,11-dienoyl-[a | 0 | Not detected | 0.012 | -0.254 | 0.279 | 0.928 |
| ENZRXN.23 | lactose 2-epimerase | 0 | Not detected | -0.247 | -0.560 | 0.066 | 0.123 |
| ENZRXN.23 | 8-amino-3,8-dideoxy-manno-o | 0 | Not detected | -0.269 | -0.576 | 0.037 | 0.085 |
| ENZRXN.24 | scaffold protein for iron-sulfur | 117 | Not detected | 0.316 | 0.023 | 0.608 | 0.034 |
| ENZRXN.24 | lactate racemase | 0 | Not detected | -0.042 | -0.318 | 0.234 | 0.766 |
| ENZRXN.24 | UDP-4-amino-4,6-dideoxy-N-a | 0 | Not detected | -0.317 | -0.705 | 0.071 | 0.109 |
| ENZRXN.24 | guanosine phosphorylase | 0 | Not detected | -0.052 | -0.392 | 0.288 | 0.764 |
| ENZRXN.24 | deoxycholate 3Î±-dehydrogena | 0 | Not detected | 0.006 | -0.245 | 0.257 | 0.960 |
| ENZRXN.24 | deoxycholate 3Î±-dehydrogena | 0 | Not detected | -0.251 | -0.600 | 0.098 | 0.160 |
| ENZRXN.24 | 3-oxochol-4,6-dien-24-oyl-Co | 0 | Not detected | -0.236 | -0.561 | 0.089 | 0.154 |
| ENZRXN.24 | lithocholate 3Î±-dehydrogenas | 0 | Not detected | -0.251 | -0.600 | 0.098 | 0.160 |
| ENZRXN.24 | chenodeoxycholate 3Î±-dehydr | 0 | Not detected | -0.251 | -0.600 | 0.098 | 0.160 |
| ENZRXN.24 | cholate 3Î±-dehydrogenase | 0 | Not detected | -0.251 | -0.600 | 0.098 | 0.160 |
| ENZRXN.24 | lithocholate 3Î±-dehydrogenas | 0 | Not detected | 0.006 | -0.245 | 0.257 | 0.960 |
| ENZRXN.24 | cholate 3Î±-dehydrogenase | 0 | Not detected | 0.006 | -0.245 | 0.257 | 0.960 |
| ENZRXN.24 | chenodeoxycholate 3Î±-dehydr | 0 | Not detected | 0.006 | -0.245 | 0.257 | 0.960 |
| ENZRXN.24 | Î²-1,2-mannosidase | 0 | Not detected | -0.154 | -0.452 | 0.144 | 0.312 |
| ENZRXN.24 | L-galacto-1,5-lactonase | 0 | Not detected | -0.164 | -0.426 | 0.099 | 0.221 |
| ENZRXN.24 | succinate:malate antiport | 0 | Not detected | 0.095 | -0.203 | 0.393 | 0.532 |
| ENZRXN.24 | succinate:L-aspartate antiport | 0 | Not detected | 0.095 | -0.203 | 0.393 | 0.532 |
| ENZRXN.24 | malate:succinate antiporter | 0 | Not detected | 0.120 | -0.140 | 0.380 | 0.365 |
| ENZRXN.24 | L-aspartate:succinate antiporte | 0 | Not detected | 0.120 | -0.140 | 0.380 | 0.365 |
| ENZRXN.24 | dihydroorotate dehydrogenase | 0 | Not detected | 0.179 | -0.162 | 0.520 | 0.304 |
| ENZRXN.24 | methionine &gamma;-lyase | 0 | Not detected | -0.128 | -0.425 | 0.169 | 0.399 |
| ENZRXN.24 | [HypE]-S-carboxamide dehydr | 0 | Not detected | -0.053 | -0.286 | 0.180 | 0.658 |
| ENZRXN.25 | Î±-D-ribose-1-(2-N-acetamido | 0 | Not detected | 0.011 | -0.220 | 0.242 | 0.926 |
| ENZRXN.25 | carbon-phosphorous lyase | 0 | Not detected | 0.027 | -0.233 | 0.286 | 0.841 |
| ENZRXN.25 | Î±-D-ribose-1-[N-(phosphonom | 0 | Not detected | -0.187 | -0.432 | 0.058 | 0.135 |
| ENZRXN.25 | Î±-D-ribose-1-[N-(phosphonom | 0 | Not detected | -0.099 | -0.399 | 0.201 | 0.518 |
| ENZRXN.25 | adenine phosphoribosyltransfer | 0 | Not detected | 0.078 | -0.236 | 0.392 | 0.626 |

| ENZRXN.25 | tetracycline 11a-oxygenase | 0 | Not detected | 0.115 | -0.353 | 0.583 | 0.631 |
| --- | --- | --- | --- | --- | --- | --- | --- |
| ENZRXN.25 | tigecycline 11a-momooxygena | 0 | Not detected | 0.115 | -0.353 | 0.583 | 0.631 |
| ENZRXN.25 | UDP-N-acetylglucosamine 2-e | 0 | Not detected | -0.458 | -0.807 | -0.110 | 0.010 |
| ENZRXN.25 | ribitol-5-phosphate cytidylyltra | 0 | Not detected | -0.048 | -0.396 | 0.301 | 0.788 |
| ENZRXN.25 | ribitol-5-phosphate 1-dehydrog | 0 | Not detected | -0.211 | -0.544 | 0.121 | 0.213 |
| ENZRXN.25 | ribitol-5-phosphate 1-dehydrog | 0 | Not detected | -0.112 | -0.469 | 0.244 | 0.537 |
| ENZRXN.25 | UTP--glucose-1-phosphate uri | 0 | Not detected | -0.131 | -0.477 | 0.215 | 0.457 |
| ENZRXN.25 | undecaprenyl-phosphate 2-acet | 0 | Not detected | 0.263 | -0.057 | 0.584 | 0.107 |
| ENZRXN.25 | D-alanine--[D-alanyl carrier pr | 0 | Not detected | -0.244 | -0.584 | 0.095 | 0.159 |
| ENZRXN.25 | PG:teichoic acid D-alanyltrans | 0 | Not detected | -0.067 | -0.377 | 0.244 | 0.673 |
| ENZRXN.25 | D-alanine carrier protein:PG D | 0 | Not detected | -0.209 | -0.513 | 0.095 | 0.178 |
| ENZRXN.25 | 1,2-diacylglycerol 3-Î±-glucosy | 0 | Not detected | -0.063 | -0.354 | 0.228 | 0.671 |
| ENZRXN.25 | isopropylmalate dehydrogenase | 0 | Not detected | -0.100 | -0.281 | 0.082 | 0.283 |
| ENZRXN.25 | tRNA uridine 4-sulfurtransfera | 0 | Not detected | -0.050 | -0.309 | 0.209 | 0.706 |
| ENZRXN.25 | aminobutyraldehyde dehydroge | 0 | Not detected | 0.234 | 0.018 | 0.451 | 0.034 |
| ENZRXN.25 | succinate-semialdehyde dehydr | 0 | Not detected | 0.234 | 0.018 | 0.451 | 0.034 |
| ENZRXN.25 | 7,8-dihydro-8-oxoguanine DN | 0 | Not detected | 0.068 | -0.148 | 0.283 | 0.539 |
| ENZRXN.25 | [sulfur carrier protein TtuB] ad | 0 | Not detected | 0.005 | -0.308 | 0.318 | 0.974 |
| ENZRXN.25 | trans-4-hydroxy-L-proline deh | 0 | Not detected | -0.101 | -0.318 | 0.117 | 0.366 |
| ENZRXN.25 | menaquinone C8-methyltransfe | 0 | Not detected | 0.042 | -0.318 | 0.401 | 0.820 |
| ENZRXN.25 | quinol--ferredoxin oxidoreduct | 0 | Not detected | 0.313 | -0.041 | 0.667 | 0.084 |
| ENZRXN.25 | dissimilatory sulfite reductase | 0 | Not detected | 0.038 | -0.332 | 0.408 | 0.842 |
| ENZRXN.25 | L-threonate dehydrogenase | 0 | Not detected | 0.288 | 0.082 | 0.494 | 0.006 |
| ENZRXN.25 | 2-dehydro-D-threonate isomera | 0 | Not detected | 0.131 | -0.064 | 0.325 | 0.188 |
| ENZRXN.25 | 2-dehydro-L-threonate isomera | 0 | Not detected | 0.131 | -0.064 | 0.325 | 0.188 |
| ENZRXN.25 | 4-phospho-D-erythronate 3-de | 0 | Not detected | -0.175 | -0.487 | 0.137 | 0.271 |
| ENZRXN.25 | UDP-N-acetylmuramate--L-ala | 0 | Not detected | 0.036 | -0.320 | 0.392 | 0.843 |
| ENZRXN.25 | methenyltetrahydrofolate cyclo | 0 | Not detected | -0.115 | -0.314 | 0.085 | 0.260 |
| ENZRXN.25 | methylenetetrahydrofolate dehy | 0 | Not detected | -0.115 | -0.314 | 0.085 | 0.260 |
| ENZRXN.25 | deoxyribodipyrimidine photoly | 0 | Not detected | -0.055 | -0.285 | 0.175 | 0.638 |
| ENZRXN.25 | deoxyribodipyrimidine photoly | 0 | Not detected | -0.055 | -0.285 | 0.175 | 0.638 |
| ENZRXN.25 | pyridinium-3,5-biscarboxylic a | 0 | Not detected | -0.118 | -0.351 | 0.115 | 0.321 |
| ENZRXN.26 | acetate kinase | 0 | Not detected | 0.195 | 0.000 | 0.390 | 0.050 |
| ENZRXN.26 | acetaldehyde dehydrogenase (a | 0 | Not detected | -0.218 | -0.497 | 0.062 | 0.127 |
| ENZRXN.26 | glyoxal reductase | 0 | Not detected | -0.283 | -0.588 | 0.022 | 0.070 |
| ENZRXN.26 | methylglyoxal reductase | 0 | Not detected | -0.283 | -0.588 | 0.022 | 0.070 |
| ENZRXN.26 | 3-oxochol-4-en-24-oyl-CoA 4- | 0 | Not detected | -0.236 | -0.561 | 0.089 | 0.154 |
| ENZRXN.26 | 12Î±-hydroxy-3-oxochol-4-en- | 0 | Not detected | -0.236 | -0.561 | 0.089 | 0.154 |
| ENZRXN.26 | 3-hydroxyhexadecanoyl-CoA:g | 0 | Not detected | -0.150 | -0.338 | 0.038 | 0.117 |
| ENZRXN.26 | diaminohydroxyphosphoribosy | 0 | Not detected | 0.208 | -0.081 | 0.497 | 0.159 |
| ENZRXN.26 | 5-amino-6-(5-phosphoribosyla | 0 | Not detected | 0.208 | -0.081 | 0.497 | 0.159 |
| ENZRXN.26 | 1,2-Î±-L-arabinofuranosidase | 0 | Not detected | -0.098 | -0.271 | 0.075 | 0.268 |
| ENZRXN.26 | 2-hydroxyethylphosphonate cy | 0 | Not detected | -0.303 | -0.672 | 0.065 | 0.107 |
| ENZRXN.26 | acetate kinase | 0 | Not detected | -0.132 | -0.328 | 0.063 | 0.186 |

| ENZRXN.26 | thiol-disulfide exchange | 0 | Not detected | 0.106 | -0.154 | 0.366 | 0.425 |
| --- | --- | --- | --- | --- | --- | --- | --- |
| ENZRXN.26 | phosphofructokinase | 0 | Not detected | 0.014 | -0.203 | 0.230 | 0.900 |
| ENZRXN.28 | fructose-bisphosphate aldolase | 0 | Not detected | -0.087 | -0.259 | 0.085 | 0.323 |
| ENZRXN.29 | phospho-Î²-galactosidase | 0 | Not detected | -0.040 | -0.378 | 0.299 | 0.819 |
| ENZRXN.30 | 4-hydroxyphenylacetate 3-mon | 0 | Not detected | -0.001 | -0.222 | 0.220 | 0.995 |
| ENZRXN.33 | aspartokinase | 0 | Not detected | 0.253 | -0.085 | 0.591 | 0.143 |
| ENZRXN.44 | GDP-4-keto-6-deoxymannose- | 0 | Not detected | -0.260 | -0.600 | 0.080 | 0.134 |
| ENZRXN.58 | isoamylase | 0 | Not detected | -0.018 | -0.373 | 0.336 | 0.919 |
| ENZRXN.60 | trehalose-6-phosphate hydrolas | 0 | Not detected | -0.184 | -0.390 | 0.023 | 0.082 |
| ENZRXN.61 | 16S rRNA m2G1207 methyltra | 0 | Not detected | 0.051 | -0.163 | 0.265 | 0.641 |
| ENZRXN.62 | Î±-phosphoglucomutase | 0 | Not detected | -0.144 | -0.438 | 0.150 | 0.337 |
| ENZRXN.63 | UDP-glucose-4-epimerase | 0 | Not detected | -0.140 | -0.369 | 0.090 | 0.233 |
| ENZRXN.67 | N-succinyldiaminopimelate-am | 0 | Not detected | -0.148 | -0.467 | 0.170 | 0.361 |
| ENZRXN.68 | aspartate kinase | 0 | Not detected | -0.056 | -0.351 | 0.240 | 0.712 |
| ENZRXN.71 | 6-phosphogluconate dehydroge | 0 | Not detected | -0.154 | -0.403 | 0.095 | 0.226 |
| ENZRXN.78 | phosphoribosylamidoimidazole | 0 | Not detected | -0.450 | -0.786 | -0.115 | 0.009 |
| ENZRXN.80 | nitric oxide dioxygenase | 0 | Not detected | 0.159 | -0.053 | 0.371 | 0.142 |
| ENZRXN.89 | UDP-N-acetylglucosamine 2-e | 0 | Not detected | -0.105 | -0.310 | 0.099 | 0.314 |
| ENZRXN.90 | UTP-glucose-1-phosphate urid | 0 | Not detected | -0.065 | -0.286 | 0.156 | 0.564 |
| ENZRXN.93 | NAD-dependent glyceraldehyd | 0 | Not detected | 0.171 | -0.149 | 0.491 | 0.295 |
| ENZRXN.95 | O-acetylhomoserine sulfhydryl | 0 | Not detected | -0.096 | -0.257 | 0.064 | 0.239 |
| ENZRXN0.1 | lipoyl-adenylate--protein ligase | 0 | Not detected | 0.048 | -0.148 | 0.245 | 0.629 |
| ENZRXN0.1 | 23S rRNA m5U1939 methyltra | 0 | Not detected | -0.050 | -0.273 | 0.173 | 0.661 |
| ENZRXN0.1 | GTPase | 0 | Not detected | -0.061 | -0.302 | 0.179 | 0.616 |
| ENZRXN0.1 | lipoate acetyltransferase | 0 | Not detected | -0.097 | -0.377 | 0.182 | 0.496 |
| ENZRXN0.1 | lipoate-apo-GcvH protein ligas | 0 | Not detected | 0.048 | -0.148 | 0.245 | 0.629 |
| ENZRXN0.1 | protein-(glutamine-N5) methyl | 0 | Not detected | 0.019 | -0.179 | 0.217 | 0.850 |
| ENZRXN0.1 | protein-glutamine N-methyltra | 0 | Not detected | 0.164 | -0.045 | 0.373 | 0.124 |
| ENZRXN0.1 | propanoate:CoA ligase (AMP-f | 0 | Not detected | 0.458 | 0.176 | 0.740 | 0.002 |
| ENZRXN0.1 | tRNA-dihydrouridine synthase | 0 | Not detected | 0.130 | -0.171 | 0.430 | 0.398 |
| ENZRXN0.1 | tRNA-dihydrouridine synthase | 0 | Not detected | 0.005 | -0.220 | 0.231 | 0.963 |
| ENZRXN0.1 | tRNA-dihydrouridine16 syntha | 0 | Not detected | 0.028 | -0.188 | 0.244 | 0.801 |
| ENZRXN0.1 | tRNA(m5U54)methyltransferas | 0 | Not detected | 0.057 | -0.175 | 0.290 | 0.630 |
| ENZRXN0.1 | deoxyribodipyrimidine photoly | 0 | Not detected | -0.055 | -0.285 | 0.175 | 0.638 |
| ENZRXN0.1 | O-6-methylguanine-DNA meth | 0 | Not detected | -0.036 | -0.246 | 0.174 | 0.738 |
| ENZRXN0.1 | CCA tRNA nucleotidyltransfer | 0 | Not detected | 0.202 | -0.040 | 0.444 | 0.102 |
| ENZRXN0.1 | DNA adenine methyltransferas | 0 | Not detected | 0.122 | -0.104 | 0.349 | 0.290 |
| ENZRXN0.1 | tRNA dimethylallyltransferase | 0 | Not detected | -0.023 | -0.295 | 0.250 | 0.870 |
| ENZRXN0.1 | tRNA preQ134 S-adenosylmet | 0 | Not detected | 0.014 | -0.169 | 0.197 | 0.879 |
| ENZRXN0.1 | ribose 1,5-bisphosphate phosp | 0 | Not detected | -0.035 | -0.218 | 0.148 | 0.707 |
| ENZRXN0.1 | TDP-N-acetylfucosamine:lipid | 0 | Not detected | 0.211 | -0.022 | 0.444 | 0.077 |
| ENZRXN0.1 | ferroxidase | 0 | Not detected | 0.132 | -0.105 | 0.369 | 0.276 |
| ENZRXN0.1 | inosine:H+ antiporter | 0 | Not detected | -0.036 | -0.260 | 0.189 | 0.755 |
| ENZRXN0.2 | carboxylesterase | 0 | Not detected | 0.038 | -0.162 | 0.238 | 0.707 |

| ENZRXN0.2 | hydroxylamine reductase | 0 | Not detected | 0.186 | -0.053 | 0.426 | 0.128 |
| --- | --- | --- | --- | --- | --- | --- | --- |
| ENZRXN0.2 | diadenosine tetraphosphatase | 0 | Not detected | 0.089 | -0.124 | 0.302 | 0.415 |
| ENZRXN0.2 | DNA-cytosine methyltransfera | 0 | Not detected | 0.178 | -0.040 | 0.395 | 0.110 |
| ENZRXN0.2 | phosphatidylglycerol&mdash;p | 0 | Not detected | 0.178 | -0.097 | 0.453 | 0.206 |
| ENZRXN0.2 | enterochelin esterase | 0 | Not detected | 0.079 | -0.128 | 0.286 | 0.456 |
| ENZRXN0.2 | limit dextrin Î±-1,6-glucohydro | 0 | Not detected | -0.063 | -0.330 | 0.205 | 0.646 |
| ENZRXN0.2 | long-chain fatty acid outer mem | 0 | Not detected | 0.013 | -0.202 | 0.227 | 0.908 |
| ENZRXN0.2 | ferric dihyroxybenzoylserine o | 0 | Not detected | 0.003 | -0.227 | 0.233 | 0.981 |
| ENZRXN0.2 | UDP-4-amino-4-deoxy-L-arabi | 0 | Not detected | 0.156 | -0.051 | 0.364 | 0.140 |
| ENZRXN0.2 | guanosine:H+ antiporter | 0 | Not detected | -0.036 | -0.260 | 0.189 | 0.755 |
| ENZRXN0.2 | Î²-N-acetylglucosaminidase | 0 | Not detected | -0.056 | -0.284 | 0.173 | 0.631 |
| ENZRXN0.2 | guanine deaminase | 0 | Not detected | 0.157 | -0.082 | 0.396 | 0.198 |
| ENZRXN0.2 | crotonyl-CoA hydratase | 0 | Not detected | 0.209 | -0.022 | 0.440 | 0.076 |
| ENZRXN0.2 | L-ribulose-5-phosphate 4-epim | 0 | Not detected | 0.063 | -0.138 | 0.264 | 0.540 |
| ENZRXN0.2 | dihydropteridine reductase | 0 | Not detected | 0.159 | -0.053 | 0.371 | 0.142 |
| ENZRXN0.2 | 23S rRNA pseudouridine746 s | 0 | Not detected | 0.084 | -0.126 | 0.293 | 0.433 |
| ENZRXN0.2 | 23S rRNA pseudouridine2604 | 0 | Not detected | -0.127 | -0.331 | 0.076 | 0.221 |
| ENZRXN0.2 | 23S rRNA pseudouridine2457 | 0 | Not detected | 0.268 | 0.011 | 0.525 | 0.041 |
| ENZRXN0.2 | 23S rRNA pseudouridine2605 | 0 | Not detected | 0.021 | -0.167 | 0.209 | 0.826 |
| ENZRXN0.2 | 23S rRNA pseudouridine955/2 | 0 | Not detected | 0.234 | -0.013 | 0.480 | 0.063 |
| ENZRXN0.2 | inositol-phosphate phosphatase | 0 | Not detected | 0.052 | -0.185 | 0.290 | 0.666 |
| ENZRXN0.2 | Î²-keto ester reductase | 0 | Not detected | 0.215 | 0.013 | 0.417 | 0.037 |
| ENZRXN0.2 | propionyl-CoA:succinate CoA | 0 | Not detected | -0.057 | -0.285 | 0.172 | 0.627 |
| ENZRXN0.2 | tRNAIle-lysidine synthetase | 0 | Not detected | 0.121 | -0.110 | 0.351 | 0.305 |
| ENZRXN0.2 | 23S rRNA pseudouridine1911/ | 0 | Not detected | 0.041 | -0.203 | 0.286 | 0.741 |
| ENZRXN0.2 | 16S rRNA pseudouridine516 s | 0 | Not detected | 0.039 | -0.156 | 0.235 | 0.692 |
| ENZRXN0.2 | lipid IVA 4-amino-4-deoxy-L- | 0 | Not detected | 0.103 | -0.123 | 0.328 | 0.372 |
| ENZRXN0.2 | 3-hydroxyacyl-CoA dehydroge | 0 | Not detected | 0.210 | -0.044 | 0.465 | 0.105 |
| ENZRXN0.2 | dodecenoyl-CoA D-isomerase | 0 | Not detected | 0.210 | -0.044 | 0.465 | 0.105 |
| ENZRXN0.2 | 3-hydroxybutyryl-CoA epimera | 0 | Not detected | 0.210 | -0.044 | 0.465 | 0.105 |
| ENZRXN0.2 | acetyl-CoA C-acyltransferase | 0 | Not detected | 0.157 | -0.059 | 0.373 | 0.155 |
| ENZRXN0.2 | short chain acyl-CoA synthetas | 0 | Not detected | 0.077 | -0.201 | 0.355 | 0.588 |
| ENZRXN0.2 | enoyl-CoA hydratase | 0 | Not detected | 0.210 | -0.044 | 0.465 | 0.105 |
| ENZRXN0.2 | tyrosine kinase | 0 | Not detected | -0.008 | -0.250 | 0.234 | 0.947 |
| ENZRXN0.2 | tRNA-specific 2-thiouridylase | 0 | Not detected | -0.009 | -0.260 | 0.241 | 0.941 |
| ENZRXN0.2 | NADH oxidoreductase | 0 | Not detected | 0.103 | -0.092 | 0.298 | 0.300 |
| ENZRXN0.2 | 3-hydroxyadipyl-CoA dehydro | 0 | Not detected | 0.199 | -0.006 | 0.404 | 0.057 |
| ENZRXN0.2 | D-allulose-6-phosphate 3-epim | 0 | Not detected | -0.303 | -0.636 | 0.031 | 0.076 |
| ENZRXN0.2 | NADH cupric reductase | 0 | Not detected | 0.009 | -0.218 | 0.236 | 0.938 |
| ENZRXN0.2 | DNA ligase | 0 | Not detected | 0.011 | -0.240 | 0.261 | 0.934 |
| ENZRXN0.2 | DNA ligase | 0 | Not detected | 0.215 | -0.115 | 0.544 | 0.202 |
| ENZRXN0.2 | peptidyl-prolyl cis-trans isomer | 0 | Not detected | 0.143 | -0.149 | 0.435 | 0.337 |
| ENZRXN0.2 | peptidyl-prolyl cis-trans isomer | 0 | Not detected | 0.133 | -0.158 | 0.425 | 0.370 |
| ENZRXN0.2 | UDP-2,3-diacylglucosamine di | 0 | Not detected | -0.025 | -0.223 | 0.174 | 0.808 |

| ENZRXN0.2 | pyruvate-flavodoxin oxidoredu | 0 | Not detected | 0.129 | -0.258 | 0.517 | 0.512 |
| --- | --- | --- | --- | --- | --- | --- | --- |
| ENZRXN0.2 | malate:quinone oxidoreductase | 0 | Not detected | 0.057 | -0.178 | 0.291 | 0.637 |
| ENZRXN0.2 | fructose-6-phosphate aldolase | 0 | Not detected | -0.014 | -0.199 | 0.171 | 0.885 |
| ENZRXN0.2 | tRNA m7G46 methyltransferas | 0 | Not detected | 0.237 | -0.036 | 0.511 | 0.089 |
| ENZRXN0.2 | 10-formyltetrahydrofolate:L-m | 0 | Not detected | 0.031 | -0.263 | 0.325 | 0.838 |
| ENZRXN0.3 | RNase R | 0 | Not detected | 0.103 | -0.200 | 0.406 | 0.507 |
| ENZRXN0.3 | fructokinase | 0 | Not detected | 0.149 | -0.053 | 0.350 | 0.148 |
| ENZRXN0.3 | tRNA 2-selenouridine synthase | 0 | Not detected | 0.233 | 0.024 | 0.442 | 0.029 |
| ENZRXN0.3 | endo-1,4-D-glucanase | 0 | Not detected | 0.014 | -0.200 | 0.227 | 0.900 |
| ENZRXN0.3 | UDP-N-acetylmuramate:L-alan | 0 | Not detected | 0.105 | -0.208 | 0.417 | 0.512 |
| ENZRXN0.3 | 23S rRNA m5U747 methyltran | 0 | Not detected | 0.086 | -0.146 | 0.318 | 0.468 |
| ENZRXN0.3 | indoleglycerol phosphate aldol | 0 | Not detected | 0.075 | -0.126 | 0.277 | 0.463 |
| ENZRXN0.3 | L-threonine:H+ antiporter | 0 | Not detected | 0.019 | -0.236 | 0.275 | 0.881 |
| ENZRXN0.3 | L-homoserine:H+ antiporter | 0 | Not detected | 0.019 | -0.236 | 0.275 | 0.881 |
| ENZRXN0.3 | Fe2+:H+ symporter | 0 | Not detected | 0.029 | -0.180 | 0.238 | 0.786 |
| ENZRXN0.3 | N-acetylmuramyl-L-alanine am | 0 | Not detected | 0.231 | 0.003 | 0.459 | 0.048 |
| ENZRXN0.3 | tRNA pseudouridine13 synthas | 0 | Not detected | 0.151 | -0.057 | 0.358 | 0.154 |
| ENZRXN0.3 | tRNA pseudouridine55 synthas | 0 | Not detected | -0.069 | -0.291 | 0.153 | 0.541 |
| ENZRXN0.3 | tRNA pseudouridine65 synthas | 0 | Not detected | 0.037 | -0.155 | 0.229 | 0.708 |
| ENZRXN0.3 | methionine aminopeptidase | 0 | Not detected | 0.178 | -0.140 | 0.495 | 0.273 |
| ENZRXN0.3 | peptide deformylase | 0 | Not detected | -0.078 | -0.314 | 0.158 | 0.518 |
| ENZRXN0.3 | DNA polymerase | 0 | Not detected | 0.006 | -0.214 | 0.226 | 0.954 |
| ENZRXN0.3 | 16S rRNA m62A1518,m62A1 | 0 | Not detected | 0.015 | -0.232 | 0.262 | 0.905 |
| ENZRXN0.3 | undecaprenyl pyrophosphate p | 0 | Not detected | -0.116 | -0.355 | 0.123 | 0.342 |
| ENZRXN0.3 | 16S rRNA m5C967 methyltran | 0 | Not detected | 0.004 | -0.252 | 0.259 | 0.977 |
| ENZRXN0.3 | leader peptidase | 0 | Not detected | -0.024 | -0.223 | 0.176 | 0.816 |
| ENZRXN0.3 | topoisomerase | 0 | Not detected | 0.078 | -0.230 | 0.386 | 0.618 |
| ENZRXN0.4 | topoisomerase | 0 | Not detected | 0.045 | -0.213 | 0.304 | 0.732 |
| ENZRXN0.4 | methylene-tetrahydrofolylpolyg | 0 | Not detected | -0.021 | -0.248 | 0.206 | 0.859 |
| ENZRXN0.4 | 2,3-dihydroxybenzoate:oxygen | 0 | Not detected | 0.132 | -0.105 | 0.369 | 0.276 |
| ENZRXN0.4 | cuprous oxidase | 0 | Not detected | 0.132 | -0.105 | 0.369 | 0.276 |
| ENZRXN0.4 | uridine nucleosidase | 0 | Not detected | -0.059 | -0.268 | 0.149 | 0.578 |
| ENZRXN0.4 | oligopeptidase | 0 | Not detected | 0.130 | -0.099 | 0.359 | 0.265 |
| ENZRXN0.4 | 23S rRNA 2'-O-ribose U2552 | 0 | Not detected | -0.082 | -0.309 | 0.144 | 0.476 |
| ENZRXN0.4 | oligopeptidase | 0 | Not detected | -0.111 | -0.419 | 0.197 | 0.479 |
| ENZRXN0.4 | tail-specific protease | 0 | Not detected | 0.208 | -0.057 | 0.474 | 0.124 |
| ENZRXN0.4 | protease | 0 | Not detected | 0.205 | -0.078 | 0.488 | 0.156 |
| ENZRXN0.4 | alanyl aminopeptidase | 0 | Not detected | 0.044 | -0.244 | 0.332 | 0.766 |
| ENZRXN0.4 | alkaline phosphatase aminopep | 0 | Not detected | 0.119 | -0.087 | 0.325 | 0.258 |
| ENZRXN0.4 | peptidyl-dipeptidase | 0 | Not detected | 0.132 | -0.102 | 0.366 | 0.270 |
| ENZRXN0.4 | formate dehydrogenase | 0 | Not detected | -0.066 | -0.370 | 0.238 | 0.672 |
| ENZRXN0.4 | meso-diaminopimelic acid-D-a | 0 | Not detected | 0.116 | -0.119 | 0.352 | 0.333 |
| ENZRXN0.4 | D-alanyl-D-alanine carboxypep | 0 | Not detected | 0.010 | -0.187 | 0.207 | 0.923 |
| ENZRXN0.4 | exoribonuclease II | 0 | Not detected | -0.005 | -0.272 | 0.261 | 0.969 |

| ENZRXN0.4 | undecaprenyl-phosphate 4-deo | 0 | Not detected | 0.013 | -0.185 | 0.211 | 0.900 |
| --- | --- | --- | --- | --- | --- | --- | --- |
| ENZRXN0.5 | methionine-S-sulfoxide reducta | 0 | Not detected | 0.194 | -0.073 | 0.462 | 0.155 |
| ENZRXN0.5 | RNase D exonuclease | 0 | Not detected | 0.085 | -0.114 | 0.284 | 0.402 |
| ENZRXN0.5 | 5'-nucleotidase | 0 | Not detected | 0.043 | -0.173 | 0.259 | 0.699 |
| ENZRXN0.5 | 3'-nucleotidase | 0 | Not detected | 0.043 | -0.173 | 0.259 | 0.699 |
| ENZRXN0.5 | pyrimidine 5'-nucleotidase | 0 | Not detected | 0.015 | -0.173 | 0.203 | 0.877 |
| ENZRXN0.5 | &gamma;-glutamylputrescine o | 0 | Not detected | 0.183 | -0.029 | 0.396 | 0.091 |
| ENZRXN0.5 | &gamma;-glutamyl-&gamma;- | 0 | Not detected | 0.234 | 0.018 | 0.451 | 0.034 |
| ENZRXN0.5 | octanoul-ACP synthetase | 0 | Not detected | 0.077 | -0.183 | 0.337 | 0.559 |
| ENZRXN0.5 | 4-aminobutyrate aminotransfer | 0 | Not detected | 0.151 | -0.066 | 0.368 | 0.174 |
| ENZRXN0.5 | endochitinase | 0 | Not detected | 0.044 | -0.220 | 0.308 | 0.745 |
| ENZRXN0.5 | serine/threonine:Na+ symporte | 0 | Not detected | -0.093 | -0.238 | 0.052 | 0.208 |
| ENZRXN0.5 | ribonuclease | 0 | Not detected | -0.087 | -0.306 | 0.131 | 0.432 |
| ENZRXN0.5 | RNase | 0 | Not detected | 0.089 | -0.253 | 0.432 | 0.609 |
| ENZRXN0.5 | methylglyoxal reductase | 0 | Not detected | -0.051 | -0.204 | 0.103 | 0.519 |
| ENZRXN0.5 | methylglyoxal reductase | 0 | Not detected | 0.215 | 0.013 | 0.417 | 0.037 |
| ENZRXN0.5 | methylglyoxal reductase | 0 | Not detected | -0.026 | -0.350 | 0.297 | 0.875 |
| ENZRXN0.5 | Kdo2-lipid VIA phosphoethan | 0 | Not detected | 0.182 | -0.071 | 0.435 | 0.158 |
| ENZRXN0.5 | anhydro-N-acetylmuramic acid | 0 | Not detected | 0.018 | -0.199 | 0.235 | 0.871 |
| ENZRXN0.5 | RNase | 0 | Not detected | 0.114 | -0.178 | 0.407 | 0.444 |
| ENZRXN0.6 | DNA polymerase | 0 | Not detected | 0.123 | -0.190 | 0.436 | 0.442 |
| ENZRXN0.6 | 3' to 5' proofreading exonuclea | 0 | Not detected | -0.069 | -0.273 | 0.136 | 0.511 |
| ENZRXN0.6 | DNA primase | 0 | Not detected | 0.118 | -0.156 | 0.393 | 0.399 |
| ENZRXN0.6 | DNA polymerase | 0 | Not detected | 0.181 | -0.080 | 0.442 | 0.175 |
| ENZRXN0.6 | DNA polymerase | 0 | Not detected | 0.181 | -0.080 | 0.442 | 0.175 |
| ENZRXN0.6 | peptidyl-prolyl cis-trans isomer | 0 | Not detected | 0.158 | -0.064 | 0.381 | 0.163 |
| ENZRXN0.6 | cAMP phosphodiesterase | 0 | Not detected | -0.224 | -0.436 | -0.011 | 0.039 |
| ENZRXN0.6 | DNA polymerase | 0 | Not detected | -0.041 | -0.348 | 0.265 | 0.792 |
| ENZRXN0.6 | 3' to 5' proofreading exonuclea | 0 | Not detected | -0.041 | -0.348 | 0.265 | 0.792 |
| ENZRXN0.6 | 5' to 3' exonuclease | 0 | Not detected | -0.041 | -0.348 | 0.265 | 0.792 |
| ENZRXN0.6 | ADP-heptose:Kdo2-lipid IVA- | 0 | Not detected | -0.009 | -0.233 | 0.215 | 0.937 |
| ENZRXN0.6 | ADP-heptose&mdash;LPS hep | 192 | Not detected | -0.214 | -0.447 | 0.020 | 0.073 |
| ENZRXN0.6 | isopentenyl-adenosine tRNA m | 0 | Not detected | 0.125 | -0.190 | 0.440 | 0.437 |
| ENZRXN0.6 | xanthine:H+ symporter XanP | 0 | Not detected | -0.068 | -0.250 | 0.115 | 0.467 |
| ENZRXN0.6 | 1-deoxy-D-xylulose 5-phospha | 0 | Not detected | -0.088 | -0.248 | 0.073 | 0.284 |
| ENZRXN0.6 | octanoate-non-lipoylated apo d | 0 | Not detected | 0.048 | -0.148 | 0.245 | 0.629 |
| ENZRXN0.6 | 16S rRNA m5C1407 methyltra | 0 | Not detected | 0.110 | -0.105 | 0.325 | 0.316 |
| ENZRXN0.6 | Î²-D-glucoside glucohydrolase | 0 | Not detected | 0.083 | -0.171 | 0.337 | 0.523 |
| ENZRXN0.6 | ADP-heptose:Kdo2-lipid A he | 0 | Not detected | -0.009 | -0.233 | 0.215 | 0.937 |
| ENZRXN0.6 | transport of D-serine | 0 | Not detected | -0.135 | -0.410 | 0.140 | 0.337 |
| ENZRXN0.6 | methylglyoxal reductase | 0 | Not detected | -0.104 | -0.444 | 0.236 | 0.547 |
| ENZRXN0.6 | 5-methylaminomethyl-2-thiour | 0 | Not detected | 0.134 | -0.106 | 0.375 | 0.273 |
| ENZRXN0.6 | 2-isopropylmalate synthase | 0 | Not detected | 0.020 | -0.179 | 0.219 | 0.841 |
| ENZRXN0.6 | 6-phosphogluconate phosphata | 0 | Not detected | 0.018 | -0.177 | 0.213 | 0.858 |

| ENZRXN0.6 | guanosine-5'-phosphate phosph | 0 | Not detected | 0.247 | 0.055 | 0.439 | 0.012 |
| --- | --- | --- | --- | --- | --- | --- | --- |
| ENZRXN0.6 | inosine-5'-phosphate phosphoh | 0 | Not detected | 0.247 | 0.055 | 0.439 | 0.012 |
| ENZRXN0.6 | sugar phosphatase | 0 | Not detected | 0.051 | -0.168 | 0.270 | 0.650 |
| ENZRXN0.6 | FMN phosphatase | 0 | Not detected | 0.080 | -0.112 | 0.271 | 0.415 |
| ENZRXN0.6 | pyridoxal phosphatase | 0 | Not detected | 0.011 | -0.193 | 0.215 | 0.918 |
| ENZRXN0.6 | phosphosugar phosphatase | 0 | Not detected | 0.011 | -0.193 | 0.215 | 0.918 |
| ENZRXN0.6 | 23S rRNA m2G1835 methyltra | 0 | Not detected | 0.034 | -0.185 | 0.254 | 0.761 |
| ENZRXN0.6 | 23S rRNA m2G2445 methyltra | 0 | Not detected | -0.127 | -0.403 | 0.149 | 0.367 |
| ENZRXN0.6 | 3 methyl-adenine DNA glycosy | 0 | Not detected | -0.110 | -0.361 | 0.141 | 0.390 |
| ENZRXN0.6 | peptidyl-prolyl cis-trans isomer | 0 | Not detected | 0.040 | -0.214 | 0.295 | 0.755 |
| ENZRXN0.6 | lysine 2,3-aminomutase | 0 | Not detected | -0.014 | -0.249 | 0.221 | 0.905 |
| ENZRXN0.6 | alanyl aminopeptidase | 0 | Not detected | 0.044 | -0.244 | 0.332 | 0.766 |
| ENZRXN0.6 | D-malate dehydrogenase (deca | 0 | Not detected | -0.033 | -0.315 | 0.249 | 0.818 |
| ENZRXN0.6 | poly(A) polymerase | 0 | Not detected | 0.109 | -0.127 | 0.345 | 0.367 |
| ENZRXN0.6 | 2-oxobutanoate aminotransfera | 0 | Not detected | 0.032 | -0.192 | 0.256 | 0.777 |
| ENZRXN0.6 | exodeoxyribonuclease I | 0 | Not detected | 0.155 | -0.089 | 0.399 | 0.214 |
| ENZRXN0.6 | transport of Î²-alanine | 0 | Not detected | -0.135 | -0.410 | 0.140 | 0.337 |
| ENZRXN0.6 | transport of L-alanine | 0 | Not detected | -0.135 | -0.410 | 0.140 | 0.337 |
| ENZRXN0.6 | transport of D-cycloserine | 0 | Not detected | -0.135 | -0.410 | 0.140 | 0.337 |
| ENZRXN0.6 | 3' &rarr; 5' exonuclease | 0 | Not detected | 0.010 | -0.181 | 0.201 | 0.917 |
| ENZRXN0.6 | Zn2+-exporting ATPase | 0 | Not detected | 0.313 | 0.037 | 0.588 | 0.026 |
| ENZRXN0.6 | S-ribosylhomocysteine lyase | 0 | Not detected | 0.053 | -0.167 | 0.272 | 0.638 |
| ENZRXN0.6 | 16S rRNA m2G966 methyltran | 0 | Not detected | 0.079 | -0.118 | 0.277 | 0.431 |
| ENZRXN0.6 | D-gluconate transporter | 0 | Not detected | 0.056 | -0.172 | 0.284 | 0.631 |
| ENZRXN0.6 | high-affinity gluconate transpo | 0 | Not detected | 0.162 | -0.127 | 0.451 | 0.272 |
| ENZRXN0.6 | 2-O-(6-phospho-Î±-mannosyl)- | 0 | Not detected | 0.025 | -0.229 | 0.280 | 0.845 |
| ENZRXN0.6 | putrescine:H+ symporter | 0 | Not detected | 0.089 | -0.232 | 0.409 | 0.588 |
| ENZRXN0.6 | putrescine:L-ornithine antiport | 0 | Not detected | 0.089 | -0.232 | 0.409 | 0.588 |
| ENZRXN0.6 | cadaverine:H+ symporter | 0 | Not detected | -0.022 | -0.316 | 0.271 | 0.881 |
| ENZRXN0.6 | serine/threonine protein kinase | 0 | Not detected | 0.162 | -0.043 | 0.367 | 0.122 |
| ENZRXN0.6 | tRNA pseudouridine32 synthas | 0 | Not detected | 0.084 | -0.126 | 0.293 | 0.433 |
| ENZRXN0.6 | transport of GlcNAc-1,6-anhM | 0 | Not detected | 0.124 | -0.095 | 0.343 | 0.268 |
| ENZRXN0.6 | 1,6-anhydro-N-acetylmuramoy | 0 | Not detected | 0.047 | -0.160 | 0.254 | 0.656 |
| ENZRXN0.6 | glcNAc-1,6-anhMurNAcamini | 0 | Not detected | -0.056 | -0.284 | 0.173 | 0.631 |
| ENZRXN0.6 | galactonate:H+ symporter | 0 | Not detected | 0.052 | -0.180 | 0.284 | 0.661 |
| ENZRXN0.6 | L-galactonate oxidoreductase | 0 | Not detected | 0.008 | -0.212 | 0.229 | 0.940 |
| ENZRXN0.6 | transport of L-aspartate | 0 | Not detected | 0.099 | -0.212 | 0.410 | 0.532 |
| ENZRXN0.6 | succinate:fumarate antiporter | 0 | Not detected | 0.013 | -0.232 | 0.258 | 0.916 |
| ENZRXN0.6 | 16S rRNA m7G527 methyltran | 0 | Not detected | -0.048 | -0.261 | 0.165 | 0.658 |
| ENZRXN0.6 | transport of L-lyxose | 0 | Not detected | 0.017 | -0.194 | 0.227 | 0.876 |
| ENZRXN0.6 | L-idarate epimerase | 0 | Not detected | -0.268 | -0.553 | 0.017 | 0.066 |
| ENZRXN0.6 | nitrate:nitrite antiporter | 0 | Not detected | 0.197 | -0.006 | 0.399 | 0.058 |
| ENZRXN0.6 | nitrate:nitrite antiporter | 0 | Not detected | -0.032 | -0.242 | 0.178 | 0.765 |
| ENZRXN0.6 | biotin transporter | 0 | Not detected | 0.110 | -0.098 | 0.318 | 0.301 |

| ENZRXN0.6 | arbutin 6-phosphate glucohydr | 0 | Not detected | 0.305 | 0.013 | 0.598 | 0.041 |
| --- | --- | --- | --- | --- | --- | --- | --- |
| ENZRXN0.6 | arbutin 6-phosphate glucohydr | 0 | Not detected | -0.068 | -0.211 | 0.074 | 0.348 |
| ENZRXN0.6 | arbutin-6-phosphate glucohydr | 0 | Not detected | -0.009 | -0.197 | 0.179 | 0.924 |
| ENZRXN0.6 | salicin 6-phosphate glucohydro | 0 | Not detected | 0.305 | 0.013 | 0.598 | 0.041 |
| ENZRXN0.6 | transport of N-acetyl-Î²-D-gluc | 0 | Not detected | 0.124 | -0.095 | 0.343 | 0.268 |
| ENZRXN0.6 | 23S rRNA m2A2503 methyltra | 0 | Not detected | -0.031 | -0.341 | 0.278 | 0.842 |
| ENZRXN0.6 | transport of a tripeptide | 0 | Not detected | 0.012 | -0.194 | 0.217 | 0.910 |
| ENZRXN0.6 | protein kinase | 0 | Not detected | 0.244 | 0.002 | 0.486 | 0.049 |
| ENZRXN0.6 | L-2-hydroxyglutarate dehydrog | 0 | Not detected | -0.026 | -0.247 | 0.194 | 0.816 |
| ENZRXN0.6 | undecaprenyl-pyrophosphate p | 0 | Not detected | -0.032 | -0.300 | 0.236 | 0.813 |
| ENZRXN0.6 | NAD(P)H:quinone oxidoreduc | 0 | Not detected | -0.012 | -0.234 | 0.209 | 0.913 |
| ENZRXN0.6 | anthranilate phosphoribosyl tra | 0 | Not detected | 0.202 | -0.034 | 0.437 | 0.093 |
| ENZRXN0.6 | nigerose hydrolase | 0 | Not detected | -0.135 | -0.391 | 0.121 | 0.301 |
| ENZRXN0.6 | peptidoglycan glycosyltransfer | 0 | Not detected | 0.140 | -0.113 | 0.394 | 0.279 |
| ENZRXN0.6 | peptidoglycan glycosyltransfer | 0 | Not detected | 0.065 | -0.156 | 0.286 | 0.562 |
| ENZRXN0.6 | murein transpeptidase | 0 | Not detected | 0.080 | -0.201 | 0.361 | 0.578 |
| ENZRXN0.6 | glycerol-2-phosphatase | 0 | Not detected | 0.052 | -0.185 | 0.290 | 0.666 |
| ENZRXN0.6 | 3'(2'),5'-bisphosphate nucleotid | 0 | Not detected | 0.139 | -0.056 | 0.333 | 0.162 |
| ENZRXN0.6 | L-glyceraldehyde 3-phosphate | 0 | Not detected | -0.051 | -0.204 | 0.103 | 0.519 |
| ENZRXN0.6 | poly-Î²-1,6-N-acetyl-D-glucosa | 0 | Not detected | 0.104 | -0.135 | 0.344 | 0.395 |
| ENZRXN0.6 | tRNAMet cytidine acetyltransf | 0 | Not detected | 0.291 | 0.046 | 0.537 | 0.020 |
| ENZRXN0.6 | ribosomal protein L11 methyltr | 0 | Not detected | 0.060 | -0.187 | 0.307 | 0.634 |
| ENZRXN0.6 | glutamyl-Q tRNA(Asp) synthet | 0 | Not detected | 0.169 | -0.035 | 0.372 | 0.105 |
| ENZRXN0.6 | ThiS adenylyltransferase | 0 | Not detected | -0.052 | -0.252 | 0.147 | 0.609 |
| ENZRXN0.6 | adenylate:persulfide transferase | 0 | Not detected | -0.052 | -0.252 | 0.147 | 0.609 |
| ENZRXN0.6 | 3-hydroxypropionaldehyde deh | 0 | Not detected | 0.234 | 0.018 | 0.451 | 0.034 |
| ENZRXN0.6 | lipid III flippase | 0 | Not detected | 0.149 | -0.087 | 0.384 | 0.217 |
| ENZRXN0.6 | glucose dehydrogenase | 0 | Not detected | 0.292 | 0.022 | 0.561 | 0.034 |
| ENZRXN0.6 | autoinducer-2 kinase | 0 | Not detected | 0.194 | -0.045 | 0.433 | 0.112 |
| ENZRXN0.6 | lipid II flippase | 0 | Not detected | 0.045 | -0.177 | 0.267 | 0.691 |
| ENZRXN0.6 | GTPase | 0 | Not detected | 0.014 | -0.239 | 0.267 | 0.915 |
| ENZRXN0.6 | transport of a dipeptide | 0 | Not detected | 0.181 | -0.058 | 0.420 | 0.138 |
| ENZRXN0.6 | acyl-[acp]:1-acyl-sn-glycerol-3 | 0 | Not detected | -0.096 | -0.302 | 0.110 | 0.359 |
| ENZRXN0.6 | CTP:phosphatidate cytidyltrans | 0 | Not detected | 0.198 | 0.008 | 0.388 | 0.042 |
| ENZRXN0.6 | acyl-acp:sn-glycerol-3-phospha | 0 | Not detected | 0.029 | -0.265 | 0.323 | 0.846 |
| ENZRXN0.6 | 23S rRNA 2'-O-ribose C2498 | 0 | Not detected | 0.001 | -0.227 | 0.228 | 0.995 |
| ENZRXN0.7 | fructoselysine transporter | 0 | Not detected | -0.112 | -0.422 | 0.197 | 0.477 |
| ENZRXN0.7 | psicoselysine transporter | 0 | Not detected | -0.112 | -0.422 | 0.197 | 0.477 |
| ENZRXN0.7 | L-ribulose-5-phosphate 4-epim | 0 | Not detected | -0.105 | -0.257 | 0.047 | 0.175 |
| ENZRXN0.7 | L-ribulose-5-phosphate 3-epim | 0 | Not detected | 0.176 | -0.100 | 0.452 | 0.212 |
| ENZRXN0.7 | transport of a dipeptide | 0 | Not detected | 0.020 | -0.206 | 0.247 | 0.861 |
| ENZRXN0.7 | non-chaperonin molecular chap | 0 | Not detected | 0.247 | -0.061 | 0.555 | 0.116 |
| ENZRXN0.7 | protein methylthiotransferase | 0 | Not detected | 0.172 | -0.134 | 0.478 | 0.270 |
| ENZRXN0.7 | dipeptide transport | 0 | Not detected | 0.151 | -0.076 | 0.379 | 0.193 |

| ENZRXN0.7 | (S)-ureidoglycine aminohydrol | 0 | Not detected | -0.149 | -0.420 | 0.122 | 0.281 |
| --- | --- | --- | --- | --- | --- | --- | --- |
| ENZRXN0.7 | chitobiose transport | 0 | Not detected | 0.268 | 0.045 | 0.490 | 0.018 |
| ENZRXN0.7 | transport of adenine | 0 | Not detected | 0.057 | -0.182 | 0.297 | 0.641 |
| ENZRXN0.7 | guanosine 3'-diphosphate 5'-tri | 0 | Not detected | -0.007 | -0.315 | 0.300 | 0.963 |
| ENZRXN0.7 | GTPase | 0 | Not detected | 0.175 | -0.148 | 0.498 | 0.288 |
| ENZRXN0.7 | GTPase | 0 | Not detected | 0.152 | -0.105 | 0.408 | 0.247 |
| ENZRXN0.7 | carbamoyl--[HypE] ligase | 0 | Not detected | -0.027 | -0.302 | 0.247 | 0.845 |
| ENZRXN0.7 | 2-iminoacetate synthase | 0 | Not detected | 0.179 | -0.049 | 0.407 | 0.124 |
| ENZRXN0.7 | transport of malate | 0 | Not detected | 0.099 | -0.212 | 0.410 | 0.532 |
| ENZRXN0.7 | methionine-oxo-acid transamin | 0 | Not detected | 0.025 | -0.190 | 0.241 | 0.819 |
| ENZRXN0.7 | p-aminobenzoyl glutamate:H+ | 0 | Not detected | 0.125 | -0.087 | 0.336 | 0.248 |
| ENZRXN0.7 | uracil oxygenase | 0 | Not detected | 0.159 | -0.041 | 0.360 | 0.120 |
| ENZRXN0.7 | RNA helicase | 0 | Not detected | 0.091 | -0.116 | 0.298 | 0.390 |
| ENZRXN0.7 | exoribonuclease II | 0 | Not detected | -0.005 | -0.272 | 0.261 | 0.969 |
| ENZRXN0.7 | RNase D exonuclease | 0 | Not detected | 0.085 | -0.114 | 0.284 | 0.402 |
| ENZRXN0.7 | RNase D exonuclease | 0 | Not detected | 0.085 | -0.114 | 0.284 | 0.402 |
| ENZRXN0.7 | peptidoglycan DD transpeptida | 0 | Not detected | 0.083 | -0.197 | 0.364 | 0.560 |
| ENZRXN0.7 | L-aspartate oxidase | 0 | Not detected | 0.120 | -0.227 | 0.468 | 0.497 |
| ENZRXN0.7 | exoribonuclease II | 0 | Not detected | -0.005 | -0.272 | 0.261 | 0.969 |
| ENZRXN0.7 | adenosine nucleosidase | 0 | Not detected | -0.059 | -0.268 | 0.149 | 0.578 |
| ENZRXN0.7 | xanthosine nucleosidase | 0 | Not detected | -0.059 | -0.268 | 0.149 | 0.578 |
| ENZRXN0.7 | inosine nucleosidase | 0 | Not detected | -0.059 | -0.268 | 0.149 | 0.578 |
| ENZRXN0.7 | cytidine nucleosidase | 0 | Not detected | -0.059 | -0.268 | 0.149 | 0.578 |
| ENZRXN0.7 | dihydroorotate dehydrogenase | 0 | Not detected | 0.179 | -0.162 | 0.520 | 0.304 |
| ENZRXN0.7 | transport of 3-hydroxy-trans-ci | 0 | Not detected | 0.290 | 0.073 | 0.507 | 0.009 |
| ENZRXN0.7 | epoxyqueuosine reductase | 0 | Not detected | 0.178 | -0.045 | 0.401 | 0.117 |
| ENZRXN0.7 | putrescine:H+ symporter PlaP | 0 | Not detected | 0.131 | -0.091 | 0.352 | 0.249 |
| ENZRXN0.7 | acyl-[acp]:1-acyl-sn-glycerol-3 | 0 | Not detected | -0.096 | -0.302 | 0.110 | 0.359 |
| ENZRXN0.7 | putative inorganic phosphate e | 0 | Not detected | 0.053 | -0.196 | 0.302 | 0.676 |
| ENZRXN0.7 | S-methyl-L-methionine transpo | 0 | Not detected | 0.130 | -0.196 | 0.457 | 0.434 |
| ENZRXN0.7 | NADHX epimerase | 0 | Not detected | 0.110 | -0.136 | 0.355 | 0.382 |
| ENZRXN0.7 | (S)-NADHX dehydratase | 0 | Not detected | 0.110 | -0.136 | 0.355 | 0.382 |
| ENZRXN0.7 | transport of inosine | 0 | Not detected | 0.153 | -0.112 | 0.418 | 0.258 |
| ENZRXN0.7 | transport of deoxyinosine | 0 | Not detected | 0.153 | -0.112 | 0.418 | 0.258 |
| ENZRXN0.7 | 16S rRNA m2G1516 methyltra | 0 | Not detected | 0.124 | -0.088 | 0.336 | 0.253 |
| ENZRXN0.7 | Î±-D-ribose-1-methylphosphon | 0 | Not detected | 0.011 | -0.220 | 0.242 | 0.926 |
| ENZRXN0.7 | carbon-phosphorous lyase | 0 | Not detected | 0.027 | -0.233 | 0.286 | 0.841 |
| ENZRXN0.7 | peptidoglycan DD-carboxypep | 0 | Not detected | 0.088 | -0.133 | 0.309 | 0.436 |
| ENZRXN0.7 | peptidoglycan DD-endopeptida | 0 | Not detected | 0.088 | -0.133 | 0.309 | 0.436 |
| ENZRXN0.7 | ferric enterobactin esterase | 0 | Not detected | 0.079 | -0.128 | 0.286 | 0.456 |
| ENZRXN0.7 | (S)-lactate transporter | 0 | Not detected | 0.044 | -0.256 | 0.344 | 0.774 |
| ENZRXN0.7 | 23S rRNA m7G2069 methyltra | 0 | Not detected | -0.127 | -0.403 | 0.149 | 0.367 |
| ENZRXN0.7 | enterobactin exporter EntS | 0 | Not detected | 0.037 | -0.186 | 0.260 | 0.746 |
| ENZRXN0.7 | D-tartrate:succinate antiporter | 0 | Not detected | 0.120 | -0.140 | 0.380 | 0.365 |

| ENZRXN0.7 | GTPase | 0 | Not detected | 0.247 | -0.036 | 0.530 | 0.088 |
| --- | --- | --- | --- | --- | --- | --- | --- |
| ENZRXN0.7 | fructose-6-phosphate:phosphat | 0 | Not detected | 0.205 | -0.039 | 0.450 | 0.100 |
| ENZRXN0.7 | mannose-6-phosphate:phospha | 0 | Not detected | 0.205 | -0.039 | 0.450 | 0.100 |
| ENZRXN0.7 | propionate transporter | 0 | Not detected | 0.165 | -0.144 | 0.474 | 0.295 |
| ENZRXN0.7 | metalloprotease | 0 | Not detected | 0.098 | -0.116 | 0.312 | 0.370 |
| ENZRXN0.7 | (R)-lactate transporter | 0 | Not detected | 0.078 | -0.185 | 0.342 | 0.560 |
| ENZRXN0.7 | glycolate transporter | 0 | Not detected | 0.078 | -0.185 | 0.342 | 0.560 |
| ENZRXN0.7 | (R)-lactate transporter | 0 | Not detected | 0.044 | -0.256 | 0.344 | 0.774 |
| ENZRXN0.7 | transport of a C4-dicarboxylate | 0 | Not detected | 0.099 | -0.212 | 0.410 | 0.532 |
| ENZRXN0.7 | methyl Î²-D-glucoside 6-phosp | 0 | Not detected | -0.068 | -0.211 | 0.074 | 0.348 |
| ENZRXN0.7 | melibiose:H+ symporter | 0 | Not detected | 0.015 | -0.202 | 0.232 | 0.890 |
| ENZRXN0.7 | D-galactarate transporter | 0 | Not detected | 0.097 | -0.113 | 0.308 | 0.365 |
| ENZRXN0.7 | D-glucarate transporter | 0 | Not detected | 0.097 | -0.113 | 0.308 | 0.365 |
| ENZRXN0.7 | D-glycerate transporter | 0 | Not detected | 0.097 | -0.113 | 0.308 | 0.365 |
| ENZRXN0.7 | D-galactarate transporter | 0 | Not detected | 0.085 | -0.133 | 0.303 | 0.446 |
| ENZRXN0.7 | D-glucarate transporter | 0 | Not detected | 0.085 | -0.133 | 0.303 | 0.446 |
| ENZRXN0.7 | D-glycerate transporter | 0 | Not detected | 0.085 | -0.133 | 0.303 | 0.446 |
| ENZRXN0.7 | propionyl-CoA synthetase | 0 | Not detected | 0.063 | -0.178 | 0.303 | 0.609 |
| ENZRXN0.7 | 23S rRNA m6A2030 methyltra | 0 | Not detected | 0.132 | -0.086 | 0.350 | 0.237 |
| ENZRXN0.7 | EF-P-Lys34 hydroxylase | 0 | Not detected | 0.094 | -0.086 | 0.273 | 0.306 |
| ENZRXN0.7 | tRNA m2A37 methyltransferas | 0 | Not detected | -0.031 | -0.341 | 0.278 | 0.842 |
| ENZRXN0.7 | inorganic triphosphatase | 0 | Not detected | -0.002 | -0.226 | 0.222 | 0.988 |
| ENZRXN0.7 | L-glyceraldehyde-3-phosphate: | 0 | Not detected | 0.205 | -0.039 | 0.450 | 0.100 |
| ENZRXN0.7 | 6-phospho-Î²-D-glucosyl-(1,4)- | 0 | Not detected | -0.009 | -0.197 | 0.179 | 0.924 |
| ENZRXN0.7 | aldehyde reductase | 0 | Not detected | 0.156 | -0.042 | 0.353 | 0.122 |
| ENZRXN0.7 | aldehyde reductase | 0 | Not detected | -0.001 | -0.216 | 0.213 | 0.990 |
| ENZRXN0.7 | 2,5-diketo-D-gluconate reducta | 0 | Not detected | 0.215 | 0.013 | 0.417 | 0.037 |
| ENZRXN0.7 | cysteinylglycine dipeptidase | 0 | Not detected | 0.044 | -0.244 | 0.332 | 0.766 |
| ENZRXN0.7 | UDP-N-acetylmuramate:L-alan | 0 | Not detected | 0.105 | -0.208 | 0.417 | 0.512 |
| ENZRXN0.7 | transport of nitrite | 0 | Not detected | -0.032 | -0.242 | 0.178 | 0.765 |
| ENZRXN0.7 | 6-phospho-Î²-D-glucosyl-(1-4) | 0 | Not detected | 0.305 | 0.013 | 0.598 | 0.041 |
| ENZRXN0.7 | guanosine 2'3'-cyclic phosphod | 0 | Not detected | 0.106 | -0.182 | 0.395 | 0.470 |
| ENZRXN0.7 | uridine 2'3'-cyclic phosphodies | 0 | Not detected | 0.106 | -0.182 | 0.395 | 0.470 |
| ENZRXN0.7 | cytidine 2'3'-cyclic phosphodie | 0 | Not detected | 0.106 | -0.182 | 0.395 | 0.470 |
| ENZRXN0.7 | adenosine 2'3'-cyclic phosphod | 0 | Not detected | 0.106 | -0.182 | 0.395 | 0.470 |
| ENZRXN0.7 | cytidine 3'-phosphate phospho | 0 | Not detected | 0.043 | -0.173 | 0.259 | 0.699 |
| ENZRXN0.7 | adenosine 3'-phosphate phosph | 0 | Not detected | 0.043 | -0.173 | 0.259 | 0.699 |
| ENZRXN0.7 | dGMP 5'-phosphate phosphohy | 0 | Not detected | 0.043 | -0.173 | 0.259 | 0.699 |
| ENZRXN0.7 | arsenate:H+ symporter | 0 | Not detected | 0.216 | -0.032 | 0.463 | 0.088 |
| ENZRXN0.7 | succinate transporter | 0 | Not detected | 0.154 | -0.099 | 0.407 | 0.232 |
| ENZRXN0.7 | fumarate transporter | 0 | Not detected | 0.154 | -0.099 | 0.407 | 0.232 |
| ENZRXN0.7 | aspartate transport | 0 | Not detected | 0.154 | -0.099 | 0.407 | 0.232 |
| ENZRXN0.7 | amylomaltase | 0 | Not detected | 0.216 | -0.054 | 0.487 | 0.117 |
| ENZRXN0.7 | amylomaltase | 0 | Not detected | 0.216 | -0.054 | 0.487 | 0.117 |

| ENZRXN0.7 | maltopentaose glucosidase | 0 | Not detected | 0.195 | -0.046 | 0.436 | 0.113 |
| --- | --- | --- | --- | --- | --- | --- | --- |
| ENZRXN0.7 | maltohexaose glucosidase | 0 | Not detected | 0.195 | -0.046 | 0.436 | 0.113 |
| ENZRXN0.7 | maltoheptaose glucosidase | 0 | Not detected | 0.195 | -0.046 | 0.436 | 0.113 |
| ENZRXN0.7 | GTPase | 0 | Not detected | 0.082 | -0.124 | 0.288 | 0.434 |
| ENZRXN0.7 | Na+:H+ antiporter | 0 | Not detected | 0.060 | -0.145 | 0.264 | 0.568 |
| ENZRXN0.7 | K+:H+ antiporter | 0 | Not detected | 0.060 | -0.145 | 0.264 | 0.568 |
| ENZRXN0.7 | phosphoethanolamine transfera | 0 | Not detected | 0.222 | -0.024 | 0.468 | 0.077 |
| ENZRXN0.7 | phosphoethanolamine transfera | 0 | Not detected | 0.012 | -0.231 | 0.255 | 0.924 |
| ENZRXN0.7 | cytidine 3' nucleotidase | 0 | Not detected | 0.106 | -0.182 | 0.395 | 0.470 |
| ENZRXN0.7 | guanosine 3' nucleotidase | 0 | Not detected | 0.106 | -0.182 | 0.395 | 0.470 |
| ENZRXN0.7 | adenosine 3' nucleotidase | 0 | Not detected | 0.106 | -0.182 | 0.395 | 0.470 |
| ENZRXN0.7 | uridine 3' nucleotidase | 0 | Not detected | 0.106 | -0.182 | 0.395 | 0.470 |
| ENZRXN0.7 | 5'-deoxyribonucleotidase | 0 | Not detected | 0.038 | -0.225 | 0.301 | 0.778 |
| ENZRXN0.7 | 2-octaprenylphenol hydroxylas | 0 | Not detected | 0.235 | 0.020 | 0.450 | 0.033 |
| ENZRXN0.7 | DNA-AP site lyase | 0 | Not detected | -0.128 | -0.443 | 0.188 | 0.427 |
| ENZRXN0.7 | transport of D-glucose | 0 | Not detected | 0.095 | -0.134 | 0.325 | 0.416 |
| ENZRXN0.7 | AMP kinase (GTP) | 0 | Not detected | -0.122 | -0.280 | 0.036 | 0.131 |
| ENZRXN0.7 | protein-lysine deacetylase | 0 | Not detected | 0.063 | -0.180 | 0.306 | 0.612 |
| ENZRXN0.7 | transport of a dipeptide | 0 | Not detected | 0.012 | -0.194 | 0.217 | 0.910 |
| ENZRXN0.7 | transport of glucose | 0 | Not detected | -0.049 | -0.289 | 0.191 | 0.691 |
| ENZRXN0.7 | transport of maltose | 0 | Not detected | -0.049 | -0.289 | 0.191 | 0.691 |
| ENZRXN0.7 | guanine transport | 0 | Not detected | 0.056 | -0.197 | 0.309 | 0.663 |
| ENZRXN0.7 | hypoxanthine transport | 0 | Not detected | 0.056 | -0.197 | 0.309 | 0.663 |
| ENZRXN0.7 | 5-aminomethyl-2-thiouridine s | 0 | Not detected | 0.134 | -0.106 | 0.375 | 0.273 |
| ENZRXN0.7 | phosphoethanolamine transfera | 0 | Not detected | 0.031 | -0.199 | 0.261 | 0.791 |
| ENZRXN0.7 | 6-deoxy-6-sulfofructose kinase | 0 | Not detected | 0.062 | -0.149 | 0.273 | 0.564 |
| ENZRXN0.7 | 6-deoxy-6-sulfofructose-1-pho | 0 | Not detected | 0.111 | -0.102 | 0.323 | 0.307 |
| ENZRXN0.7 | sulfoquinovose transporter | 0 | Not detected | 0.083 | -0.166 | 0.332 | 0.515 |
| ENZRXN0.7 | uracil:H+ symporter | 0 | Not detected | 0.088 | -0.219 | 0.395 | 0.574 |
| ENZRXN0.7 | transport of a tripeptide | 0 | Not detected | 0.181 | -0.058 | 0.420 | 0.138 |
| ENZRXN0.7 | transport of a tripeptide | 0 | Not detected | 0.020 | -0.206 | 0.247 | 0.861 |
| ENZRXN0.7 | L-threonine dehydrogenase | 0 | Not detected | 0.319 | 0.009 | 0.630 | 0.044 |
| ENZRXN0.7 | 11-deoxycorticosterone reducta | 0 | Not detected | 0.044 | -0.169 | 0.258 | 0.684 |
| ENZRXN0.7 | D-gluconate 5-dehydrogenase | 0 | Not detected | 0.044 | -0.169 | 0.258 | 0.684 |
| ENZRXN0.7 | 2-keto-3-deoxy-D-gluconate de | 0 | Not detected | 0.044 | -0.169 | 0.258 | 0.684 |
| ENZRXN0.7 | sodium dodecyl sulfatase | 0 | Not detected | 0.165 | -0.091 | 0.420 | 0.206 |
| ENZRXN0.7 | isobutyraldehyde reductase | 0 | Not detected | 0.156 | -0.042 | 0.353 | 0.122 |
| ENZRXN0.7 | isobutyraldehyde reductase | 0 | Not detected | 0.215 | 0.013 | 0.417 | 0.037 |
| ENZRXN0.7 | isobutyraldehyde reductase | 0 | Not detected | 0.097 | -0.096 | 0.290 | 0.326 |
| ENZRXN0.7 | NADP+-dependent aldehyde re | 0 | Not detected | 0.097 | -0.096 | 0.290 | 0.326 |
| ENZRXN0.7 | isobutyraldehyde reductase | 0 | Not detected | 0.147 | -0.154 | 0.448 | 0.339 |
| ENZRXN0.7 | 1-hydroxy-2-methyl-2-(E)-bute | 0 | Not detected | -0.110 | -0.261 | 0.040 | 0.151 |
| ENZRXN0.7 | 3' &rarr; 5' ssDNA exonuclease | 0 | Not detected | -0.049 | -0.248 | 0.149 | 0.627 |
| ENZRXN0.7 | 3' &rarr; 5' RNA exonuclease | 0 | Not detected | -0.049 | -0.248 | 0.149 | 0.627 |

| ENZRXN0.7 | ATP pyrophosphatase | 0 | Not detected | 0.095 | -0.167 | 0.358 | 0.477 |
| --- | --- | --- | --- | --- | --- | --- | --- |
| ENZRXN0.7 | 3-isopropylmalate dehydrogena | 0 | Not detected | -0.033 | -0.315 | 0.249 | 0.818 |
| ENZRXN0.7 | L-tartrate dehydrogenase | 0 | Not detected | -0.033 | -0.315 | 0.249 | 0.818 |
| ENZRXN0.7 | glycerophosphoglycerol acyltra | 0 | Not detected | -0.003 | -0.216 | 0.211 | 0.980 |
| ENZRXN0.7 | NADH:menaquinone oxidored | 0 | Not detected | 0.009 | -0.218 | 0.236 | 0.938 |
| ENZRXN0.7 | polyphenol oxidase | 0 | Not detected | -0.036 | -0.242 | 0.170 | 0.735 |
| ENZRXN0.7 | PII-UMP deuridylylation | 0 | Not detected | 0.063 | -0.209 | 0.335 | 0.652 |
| ENZRXN0.7 | D-ribulose 5-phosphate 1-dehy | 0 | Not detected | 0.039 | -0.224 | 0.302 | 0.772 |
| ENZRXN0.7 | Kemp eliminase | 0 | Not detected | 0.093 | -0.092 | 0.277 | 0.324 |
| ENZRXN0.7 | flavin mononucleotide (FMN) | 0 | Not detected | 0.003 | -0.213 | 0.219 | 0.979 |
| ENZRXN0.7 | flavin adenine dinucleotide (FA | 0 | Not detected | 0.003 | -0.213 | 0.219 | 0.979 |
| ENZRXN0.7 | hydroxyacylglutathione hydrol | 0 | Not detected | -0.015 | -0.210 | 0.181 | 0.884 |
| ENZRXN0.7 | nitroimidazole export | 0 | Not detected | 0.124 | -0.081 | 0.328 | 0.236 |
| ENZRXN0.7 | L,D-carboxypeptidase | 0 | Not detected | 0.085 | -0.151 | 0.322 | 0.479 |
| ENZRXN0.7 | L,D-transpeptidase | 0 | Not detected | 0.085 | -0.151 | 0.322 | 0.479 |
| ENZRXN0.7 | L,D transpeptidase | 0 | Not detected | 0.057 | -0.171 | 0.285 | 0.624 |
| ENZRXN0.7 | L,D-transpeptidase | 0 | Not detected | 0.085 | -0.151 | 0.322 | 0.479 |
| ENZRXN0.7 | 5' &rarr; 3' exonuclease | 0 | Not detected | 0.047 | -0.146 | 0.239 | 0.636 |
| ENZRXN0.7 | 3',5'-nucleoside bisphosphate p | 0 | Not detected | 0.047 | -0.146 | 0.239 | 0.636 |
| ENZRXN0.7 | ATPase | 0 | Not detected | -0.094 | -0.248 | 0.059 | 0.229 |
| ENZRXN0.7 | acyl-acp:sn-glycerol-3-phospha | 0 | Not detected | 0.029 | -0.265 | 0.323 | 0.846 |
| ENZRXN0.7 | acyl-acp:sn-glycerol-3-phospha | 0 | Not detected | 0.029 | -0.265 | 0.323 | 0.846 |
| ENZRXN0.7 | acyl-acp:sn-glycerol-3-phospha | 0 | Not detected | 0.029 | -0.265 | 0.323 | 0.846 |
| ENZRXN0.7 | acyl-[acp]:1-acyl-sn-glycerol-3 | 0 | Not detected | -0.096 | -0.302 | 0.110 | 0.359 |
| ENZRXN0.7 | acyl-[acp]:1-acyl-sn-glycerol-3 | 0 | Not detected | -0.096 | -0.302 | 0.110 | 0.359 |
| ENZRXN0.7 | acyl-[acp]:1-acyl-sn-glycerol-3 | 0 | Not detected | -0.096 | -0.302 | 0.110 | 0.359 |
| ENZRXN0.7 | acyl-[acp]:1-acyl-sn-glycerol-3 | 0 | Not detected | -0.096 | -0.302 | 0.110 | 0.359 |
| ENZRXN0.7 | acyl-[acp]:1-acyl-sn-glycerol-3 | 0 | Not detected | -0.096 | -0.302 | 0.110 | 0.359 |
| ENZRXN0.8 | magnesium hydrogenphosphate | 0 | Not detected | 0.216 | -0.032 | 0.463 | 0.088 |
| ENZRXN0.8 | calcium hydrogenphosphate:H | 0 | Not detected | 0.216 | -0.032 | 0.463 | 0.088 |
| ENZRXN0.8 | zinc hydrogenphosphate:H+ sy | 0 | Not detected | 0.216 | -0.032 | 0.463 | 0.088 |
| ENZRXN0.8 | tellurite:H+ symporter | 0 | Not detected | 0.216 | -0.032 | 0.463 | 0.088 |
| ENZRXN0.8 | L-methionine:H+ antiport | 0 | Not detected | 0.073 | -0.156 | 0.302 | 0.534 |
| ENZRXN0.8 | L-leucine:H+ antiport | 0 | Not detected | 0.073 | -0.156 | 0.302 | 0.534 |
| ENZRXN0.8 | L-isoleucine:H+ antiport | 0 | Not detected | 0.073 | -0.156 | 0.302 | 0.534 |
| ENZRXN0.8 | L-valine:H+ antiport | 0 | Not detected | 0.073 | -0.156 | 0.302 | 0.534 |
| ENZRXN0.8 | farnesyl diphosphate phosphata | 0 | Not detected | -0.116 | -0.355 | 0.123 | 0.342 |
| ENZRXN0.8 | L-cystine:H+ symporter | 0 | Not detected | 0.144 | -0.087 | 0.376 | 0.221 |
| ENZRXN0.8 | D-cystine:H+ symporter | 0 | Not detected | 0.144 | -0.087 | 0.376 | 0.221 |
| ENZRXN0.8 | homocystine:H+ symporter | 0 | Not detected | 0.144 | -0.087 | 0.376 | 0.221 |
| ENZRXN0.8 | apolipoprotein N-acyltransfera | 0 | Not detected | 0.080 | -0.147 | 0.308 | 0.490 |
| ENZRXN0.8 | peptidoglycan lytic exotransgly | 0 | Not detected | 0.045 | -0.174 | 0.264 | 0.688 |
| ENZRXN0.8 | peptidoglycan lytic exotransgly | 0 | Not detected | 0.043 | -0.166 | 0.253 | 0.686 |
| ENZRXN0.8 | peptidoglycan lytic exotransgly | 0 | Not detected | -0.008 | -0.248 | 0.232 | 0.947 |

| ENZRXN0.8 | peptidoglycan lytic endotransg | 0 | Not detected | -0.008 | -0.248 | 0.232 | 0.947 |
| --- | --- | --- | --- | --- | --- | --- | --- |
| ENZRXN0.8 | peptidoglycan lytic endotransg | 0 | Not detected | 0.043 | -0.166 | 0.253 | 0.686 |
| ENZRXN0.8 | peptidoglycan lytic exotransgly | 0 | Not detected | -0.030 | -0.254 | 0.194 | 0.792 |
| ENZRXN0.8 | peptidoglycan lytic endotransg | 0 | Not detected | -0.030 | -0.254 | 0.194 | 0.792 |
| ENZRXN0.8 | peptidoglycan lytic endotransg | 0 | Not detected | 0.128 | -0.125 | 0.380 | 0.322 |
| ENZRXN0.8 | peptidoglycan lytic exotransgly | 0 | Not detected | 0.128 | -0.125 | 0.380 | 0.322 |
| ENZRXN0.8 | peptidoglycan lytic exotransgly | 0 | Not detected | 0.130 | -0.107 | 0.366 | 0.282 |
| ENZRXN0.8 | GTPase, ribosome-stimulated | 0 | Not detected | -0.079 | -0.231 | 0.072 | 0.306 |
| ENZRXN0.8 | transport of 2-acyl-1-lysophosp | 0 | Not detected | 0.106 | -0.115 | 0.327 | 0.346 |
| ENZRXN0.8 | transport of lyso-phosphatidylg | 0 | Not detected | 0.106 | -0.115 | 0.327 | 0.346 |
| ENZRXN0.8 | poly-3-hydroxybutyrate syntha | 0 | Not detected | 0.055 | -0.147 | 0.256 | 0.595 |
| ENZRXN0.8 | biotin-ATP ligase | 0 | Not detected | -0.056 | -0.272 | 0.160 | 0.613 |
| ENZRXN0.8 | sulfoquinovosidase | 0 | Not detected | -0.117 | -0.476 | 0.242 | 0.524 |
| ENZRXN0.8 | sulfoquinovosidase | 0 | Not detected | -0.117 | -0.476 | 0.242 | 0.524 |
| ENZRXN0.8 | (S)-2-hydroxybutanoate dehyd | 0 | Not detected | 0.139 | -0.114 | 0.393 | 0.282 |
| ENZRXN0.8 | (S)-2-hydroxybutanoate transp | 0 | Not detected | 0.078 | -0.185 | 0.342 | 0.560 |
| ENZRXN0.8 | (S)-2-hydroxybutanoate transp | 0 | Not detected | 0.044 | -0.256 | 0.344 | 0.774 |
| ENZRXN0.8 | melibionate:H+ symporter | 0 | Not detected | 0.015 | -0.202 | 0.232 | 0.890 |
| ENZRXN0.8 | choline dehydrogenase | 0 | Not detected | 0.133 | -0.123 | 0.390 | 0.308 |
| ENZRXN0.8 | betaine aldehyde dehydrogenas | 0 | Not detected | 0.133 | -0.123 | 0.390 | 0.308 |
| ENZRXN0.8 | short chain acyl-CoA synthetas | 0 | Not detected | 0.077 | -0.201 | 0.355 | 0.588 |
| ENZRXN0.8 | cis-vaccenoyl-CoA synthetase | 0 | Not detected | 0.077 | -0.201 | 0.355 | 0.588 |
| ENZRXN0.8 | oleoyl-CoA synthetase (transpo | 0 | Not detected | 0.077 | -0.201 | 0.355 | 0.588 |
| ENZRXN0.8 | inositol monophosphatase | 0 | Not detected | 0.052 | -0.185 | 0.290 | 0.666 |
| ENZRXN0.8 | fumarate:H+ symporter | 0 | Not detected | 0.120 | -0.140 | 0.380 | 0.365 |
| ENZRXN0.8 | fumarate:H+ symporter | 0 | Not detected | 0.013 | -0.232 | 0.258 | 0.916 |
| ENZRXN0.8 | acyl-CoA dehydrogenase | 0 | Not detected | 0.144 | -0.116 | 0.404 | 0.278 |
| ENZRXN0.8 | acyl-CoA dehydrogenase | 0 | Not detected | 0.144 | -0.116 | 0.404 | 0.278 |
| ENZRXN0.8 | acyl-CoA dehydrogenase | 0 | Not detected | 0.144 | -0.116 | 0.404 | 0.278 |
| ENZRXN0.8 | acyl-CoA dehydrogenase | 0 | Not detected | 0.144 | -0.116 | 0.404 | 0.278 |
| ENZRXN0.8 | acyl-CoA dehydrogenase | 0 | Not detected | 0.144 | -0.116 | 0.404 | 0.278 |
| ENZRXN0.8 | acyl-CoA dehydrogenase | 0 | Not detected | 0.144 | -0.116 | 0.404 | 0.278 |
| ENZRXN0.8 | acyl-CoA dehydrogenase | 0 | Not detected | 0.144 | -0.116 | 0.404 | 0.278 |
| ENZRXN0.8 | enoyl-CoA hydratase | 0 | Not detected | 0.210 | -0.044 | 0.465 | 0.105 |
| ENZRXN0.8 | enoyl-CoA hydratase | 0 | Not detected | 0.210 | -0.044 | 0.465 | 0.105 |
| ENZRXN0.8 | enoyl-CoA hydratase | 0 | Not detected | 0.210 | -0.044 | 0.465 | 0.105 |
| ENZRXN0.8 | enoyl-CoA hydratase | 0 | Not detected | 0.210 | -0.044 | 0.465 | 0.105 |
| ENZRXN0.8 | enoyl-CoA hydratase | 0 | Not detected | 0.210 | -0.044 | 0.465 | 0.105 |
| ENZRXN0.8 | enoyl-CoA hydratase | 0 | Not detected | 0.210 | -0.044 | 0.465 | 0.105 |
| ENZRXN0.8 | enoyl-CoA hydratase | 0 | Not detected | 0.210 | -0.044 | 0.465 | 0.105 |
| ENZRXN0.8 | 3-hydroxyacyl-CoA dehydroge | 0 | Not detected | 0.210 | -0.044 | 0.465 | 0.105 |
| ENZRXN0.8 | 3-hydroxyacyl-CoA dehydroge | 0 | Not detected | 0.210 | -0.044 | 0.465 | 0.105 |
| ENZRXN0.8 | 3-hydroxyacyl-CoA dehydroge | 0 | Not detected | 0.210 | -0.044 | 0.465 | 0.105 |
| ENZRXN0.8 | 3-hydroxyacyl-CoA dehydroge | 0 | Not detected | 0.210 | -0.044 | 0.465 | 0.105 |

| ENZRXN0.8 | 3-hydroxyacyl-CoA dehydroge | 0 | Not detected | 0.210 | -0.044 | 0.465 | 0.105 |
| --- | --- | --- | --- | --- | --- | --- | --- |
| ENZRXN0.8 | 3-hydroxyacyl-CoA dehydroge | 0 | Not detected | 0.210 | -0.044 | 0.465 | 0.105 |
| ENZRXN0.8 | 3-hydroxyacyl-CoA dehydroge | 0 | Not detected | 0.210 | -0.044 | 0.465 | 0.105 |
| ENZRXN0.8 | acetyl-CoA C-acyltransferase | 0 | Not detected | 0.157 | -0.059 | 0.373 | 0.155 |
| ENZRXN0.8 | acetyl-CoA C-acyltransferase | 0 | Not detected | 0.157 | -0.059 | 0.373 | 0.155 |
| ENZRXN0.8 | acetyl-CoA C-acyltransferase | 0 | Not detected | 0.157 | -0.059 | 0.373 | 0.155 |
| ENZRXN0.8 | acetyl-CoA C-acyltransferase | 0 | Not detected | 0.157 | -0.059 | 0.373 | 0.155 |
| ENZRXN0.8 | acetyl-CoA C-acyltransferase | 0 | Not detected | 0.157 | -0.059 | 0.373 | 0.155 |
| ENZRXN0.8 | acetyl-CoA C-acyltransferase | 0 | Not detected | 0.157 | -0.059 | 0.373 | 0.155 |
| ENZRXN0.8 | acetyl-CoA C-acyltransferase | 0 | Not detected | 0.157 | -0.059 | 0.373 | 0.155 |
| ENZRXN0.8 | palmitoleoyl-CoA synthetase (t | 0 | Not detected | 0.077 | -0.201 | 0.355 | 0.588 |
| ENZRXN0.8 | fumarate:H+ symport | 0 | Not detected | 0.095 | -0.203 | 0.393 | 0.532 |
| ENZRXN0.8 | succinate:H+ symporter | 0 | Not detected | 0.013 | -0.232 | 0.258 | 0.916 |
| ENZRXN0.8 | methylphosphotriester-DNA-pr | 0 | Not detected | -0.036 | -0.246 | 0.174 | 0.738 |
| ENZRXN0.8 | O-4-methylthymine-DNA meth | 0 | Not detected | -0.036 | -0.246 | 0.174 | 0.738 |
| ENZRXN0.8 | ferredoxin-NADP+ reductase | 0 | Not detected | 0.068 | -0.121 | 0.257 | 0.481 |
| ENZRXN0.8 | 2,6-dimethoxyphenol:oxygen o | 0 | Not detected | 0.132 | -0.105 | 0.369 | 0.276 |
| ENZRXN0.8 | 4,5-DOPA dioxygenase extradi | 0 | Not detected | 0.043 | -0.145 | 0.231 | 0.655 |
| ENZRXN0.8 | ssDNA-specific exonuclease | 0 | Not detected | 0.150 | -0.125 | 0.425 | 0.285 |
| ENZRXN0.8 | nitrophenyl phosphatase | 0 | Not detected | 0.038 | -0.225 | 0.301 | 0.778 |
| ENZRXN0.8 | diguanylate cyclase | 0 | Not detected | 0.104 | -0.104 | 0.313 | 0.327 |
| ENZRXN0.8 | P1,P3-bis(5'-adenosyl) triphosp | 0 | Not detected | 0.038 | -0.225 | 0.301 | 0.778 |
| ENZRXN0.8 | CDP-glycerol hydrolase | 0 | Not detected | 0.038 | -0.225 | 0.301 | 0.778 |
| ENZRXN0.8 | tRNATyr pseudouridine35 syn | 0 | Not detected | -0.127 | -0.331 | 0.076 | 0.221 |
| ENZRXN0.8 | GTPase | 0 | Not detected | 0.010 | -0.244 | 0.263 | 0.941 |
| ENZRXN0.8 | hydroxyphenyllactate dehydrog | 0 | Not detected | -0.089 | -0.292 | 0.115 | 0.393 |
| ENZRXN0.8 | pyridoxine 4-dehydrogenase | 0 | Not detected | 0.093 | -0.092 | 0.277 | 0.324 |
| ENZRXN0.8 | recombination-promoting nucl | 0 | Not detected | 0.181 | -0.092 | 0.453 | 0.194 |
| ENZRXN0.8 | S-sulfocysteine:H+ symporter | 0 | Not detected | 0.144 | -0.087 | 0.376 | 0.221 |
| ENZRXN0.8 | periplasmic nitrite reductase | 0 | Not detected | -0.014 | -0.275 | 0.247 | 0.916 |
| ENZRXN0.8 | signal peptide peptidase | 0 | Not detected | -0.099 | -0.353 | 0.154 | 0.442 |
| ENZRXN0.8 | lipoprotein signal peptide pepti | 0 | Not detected | -0.099 | -0.353 | 0.154 | 0.442 |
| ENZRXN0.8 | L-cysteine desulfidase | 0 | Not detected | -0.026 | -0.275 | 0.223 | 0.839 |
| ENZRXN0.8 | rhomboid protease | 0 | Not detected | 0.108 | -0.091 | 0.307 | 0.289 |
| ENZRXN0.8 | 3' to 5' ATP-dependent DNA h | 0 | Not detected | 0.055 | -0.240 | 0.350 | 0.716 |
| ENZRXN0.8 | 5' to 3' ATP-dependent DNA h | 0 | Not detected | 0.124 | -0.122 | 0.370 | 0.324 |
| ENZRXN0.8 | cytochrome c peroxidase | 0 | Not detected | 0.190 | -0.075 | 0.455 | 0.161 |
| ENZRXN0.8 | Î²-barrel assembly-enhancing p | 0 | Not detected | 0.078 | -0.160 | 0.315 | 0.521 |
| ENZRXN0.8 | D-alanyl-D-alanine carboxypep | 0 | Not detected | 0.010 | -0.187 | 0.207 | 0.923 |
| ENZRXN0.8 | D-alanyl-D-alanine carboxypep | 0 | Not detected | 0.010 | -0.187 | 0.207 | 0.923 |
| ENZRXN0.8 | Î²-lactamase | 0 | Not detected | 0.010 | -0.187 | 0.207 | 0.923 |
| ENZRXN0.8 | 2-acyl lysophospholipase | 0 | Not detected | -0.003 | -0.216 | 0.211 | 0.980 |
| ENZRXN0.8 | 1-acyl lysophospholipase | 0 | Not detected | -0.003 | -0.216 | 0.211 | 0.980 |
| ENZRXN0.8 | pyruvate:H+ symporter | 0 | Not detected | 0.218 | -0.087 | 0.523 | 0.161 |

| ENZRXN0.8 | Na+ : H+ antiporter | 0 | Not detected | 0.145 | -0.084 | 0.375 | 0.216 |
| --- | --- | --- | --- | --- | --- | --- | --- |
| ENZRXN0.8 | K+ : H+ antiporter | 0 | Not detected | 0.145 | -0.084 | 0.375 | 0.216 |
| ENZRXN0.8 | dequalinium:H+ antiporter | 0 | Not detected | 0.060 | -0.145 | 0.264 | 0.568 |
| ENZRXN0.8 | ethidium:H+ antiporter | 0 | Not detected | 0.060 | -0.145 | 0.264 | 0.568 |
| ENZRXN0.8 | glucosylglycerate phosphorylas | 0 | Not detected | 0.227 | 0.009 | 0.444 | 0.041 |
| ENZRXN0.8 | carbonylcyanide m-chlorophen | 0 | Not detected | -0.015 | -0.239 | 0.209 | 0.898 |
| ENZRXN0.8 | bicozamycin:H+ antiporter | 0 | Not detected | 0.070 | -0.141 | 0.281 | 0.516 |
| ENZRXN0.8 | chloramphenicol:H+ antiporter | 0 | Not detected | 0.072 | -0.162 | 0.306 | 0.547 |
| ENZRXN0.8 | fosfomycin:H+ antiporter | 0 | Not detected | 0.137 | -0.079 | 0.354 | 0.214 |
| ENZRXN0.8 | Fe2+ transporter | 0 | Not detected | 0.221 | -0.058 | 0.501 | 0.121 |
| ENZRXN0.8 | norfloxacin:Na+ antiport | 0 | Not detected | -0.020 | -0.221 | 0.181 | 0.844 |
| ENZRXN0.8 | tetraphenylphosphonium:Na+ a | 0 | Not detected | -0.020 | -0.221 | 0.181 | 0.844 |
| ENZRXN0.8 | deoxycholate:Na+ antiport | 0 | Not detected | -0.020 | -0.221 | 0.181 | 0.844 |
| ENZRXN0.8 | trimethylamine N-oxide reduct | 0 | Not detected | 0.042 | -0.205 | 0.288 | 0.739 |
| ENZRXN0.8 | trimethylamine N-oxide reduct | 0 | Not detected | 0.108 | -0.131 | 0.347 | 0.375 |
| ENZRXN0.8 | UDP kinase | 0 | Not detected | -0.122 | -0.280 | 0.036 | 0.131 |
| ENZRXN0.8 | dTDP kinase | 0 | Not detected | -0.122 | -0.280 | 0.036 | 0.131 |
| ENZRXN0.8 | CDP kinase | 0 | Not detected | -0.122 | -0.280 | 0.036 | 0.131 |
| ENZRXN0.8 | dCDP kinase | 0 | Not detected | -0.122 | -0.280 | 0.036 | 0.131 |
| ENZRXN0.8 | dGDP kinase | 0 | Not detected | -0.122 | -0.280 | 0.036 | 0.131 |
| ENZRXN0.8 | GDP kinase | 0 | Not detected | -0.122 | -0.280 | 0.036 | 0.131 |
| ENZRXN0.8 | celulose phosphoethanolamine | 0 | Not detected | 0.138 | -0.110 | 0.386 | 0.275 |
| ENZRXN0.8 | thymine:H+ symporter | 0 | Not detected | 0.088 | -0.219 | 0.395 | 0.574 |
| ENZRXN0.8 | xanthine:H+ symporter | 0 | Not detected | 0.088 | -0.219 | 0.395 | 0.574 |
| ENZRXN0.8 | c-di-GMP phosphodiesterase | 0 | Not detected | 0.090 | -0.148 | 0.328 | 0.457 |
| ENZRXN0.8 | apolipoprotein N-acyltransfera | 0 | Not detected | 0.080 | -0.147 | 0.308 | 0.490 |
| ENZRXN0.8 | hydroquinone:hydrogen peroxi | 0 | Not detected | 0.190 | -0.075 | 0.455 | 0.161 |
| ENZRXN0.8 | menadiol:hydrogen peroxide o | 0 | Not detected | 0.190 | -0.075 | 0.455 | 0.161 |
| ENZRXN0.8 | kojibiose phosphorylase | 0 | Not detected | 0.184 | -0.055 | 0.423 | 0.132 |
| ENZRXN0.8 | endo-poly-Î²-1,6-D-glucosamin | 0 | Not detected | 0.104 | -0.135 | 0.344 | 0.395 |
| ENZRXN0.8 | L-aspartate:fumarate antiport | 0 | Not detected | 0.095 | -0.203 | 0.393 | 0.532 |
| ENZRXN0.8 | thiol-disulfide exchange | 0 | Not detected | 0.106 | -0.154 | 0.366 | 0.425 |
| ENZRXN0.8 | ferric enterobactin esterase | 0 | Not detected | 0.079 | -0.128 | 0.286 | 0.456 |
| ENZRXN0.8 | thiol-disulfide exchange | 0 | Not detected | 0.106 | -0.154 | 0.366 | 0.425 |
| ENZRXN0.8 | 5-aminovalerate aminotransfer | 0 | Not detected | 0.151 | -0.066 | 0.368 | 0.174 |
| ENZRXN0.8 | protein-histidine auto-kinase | 0 | Not detected | 0.031 | -0.194 | 0.256 | 0.787 |
| ENZRXN0.8 | ATP-dependent 3' &rarr; 5" he | 0 | Not detected | -0.039 | -0.321 | 0.243 | 0.784 |
| ENZRXN0.8 | 5-aminopentanoate:H+ sympor | 0 | Not detected | 0.072 | -0.145 | 0.288 | 0.516 |
| ENZRXN0.8 | 5' &rarr; 3' helicase | 0 | Not detected | 0.112 | -0.144 | 0.369 | 0.391 |
| ENZRXN0.8 | apurinic/apyrimidinic endonuc | 0 | Not detected | 0.010 | -0.181 | 0.201 | 0.917 |
| ENZRXN0.8 | 3'-phosphodiesterase | 0 | Not detected | 0.010 | -0.181 | 0.201 | 0.917 |
| ENZRXN0.8 | 3'-phosphomonoesterase | 0 | Not detected | 0.010 | -0.181 | 0.201 | 0.917 |
| ENZRXN0.8 | uracil DNA glycosylase | 0 | Not detected | -0.128 | -0.443 | 0.188 | 0.427 |
| ENZRXN0.8 | thymine glycol DNA glycosyla | 0 | Not detected | -0.128 | -0.443 | 0.188 | 0.427 |

| ENZRXN0.8 | protein-histidine autokinase | 0 | Not detected | 0.121 | -0.107 | 0.348 | 0.299 |
| --- | --- | --- | --- | --- | --- | --- | --- |
| ENZRXN185 | 1,4-alpha-glucan branching enz | 0 | Not detected | -0.228 | -0.507 | 0.051 | 0.110 |
| ENZRXN185 | phosphoglucomutase | 0 | Not detected | -0.129 | -0.511 | 0.253 | 0.508 |
| ENZRXN1F. | glutamate-1-semialdehyde ami | 0 | Not detected | -0.092 | -0.453 | 0.270 | 0.620 |
| ENZRXN3O. | aconitate hydratase | 0 | Not detected | -0.246 | -0.762 | 0.271 | 0.351 |
| ENZRXN3O. | aconitate hydratase | 0 | Not detected | -0.246 | -0.762 | 0.271 | 0.351 |
| ENZRXN490 | enolase | 0 | Not detected | -0.223 | -0.551 | 0.106 | 0.184 |
| ENZRXN66. | pyruvate carboxylase | 0 | Not detected | -0.152 | -0.476 | 0.171 | 0.356 |
| ENZRXN66. | citrate dehydratase | 0 | Not detected | 0.132 | -0.158 | 0.423 | 0.372 |
| ENZRXN66. | aconitate hydratase | 0 | Not detected | 0.132 | -0.158 | 0.423 | 0.372 |
| ENZRXN8J2 | 1-deoxyxylulose-5-phosphate s | 0 | Not detected | -0.139 | -0.512 | 0.235 | 0.467 |
| ENZRXN8J2 | GTP cyclohydrolase | 0 | Not detected | -0.131 | -0.342 | 0.080 | 0.223 |
| ENZRXN8J2 | orotate phosphoribosyltransfer | 0 | Not detected | -0.099 | -0.276 | 0.077 | 0.271 |
| ENZRXN8J2 | orotidine 5'-phosphate decarbo | 0 | Not detected | 0.010 | -0.209 | 0.230 | 0.926 |
| ENZRXN8J2 | aspartate carbamoyltransferase | 0 | Not detected | -0.107 | -0.434 | 0.219 | 0.519 |
| ENZRXN8J2 | UDP-glucose 6-dehydrogenase | 0 | Not detected | -0.016 | -0.336 | 0.305 | 0.924 |
| ENZRXN8J2 | 5-dehydro-2-deoxy-D-gluconat | 0 | Not detected | 0.085 | -0.222 | 0.391 | 0.589 |
| ENZRXN8J2 | methionine-glutamine aminotra | 0 | Not detected | -0.112 | -0.395 | 0.172 | 0.441 |
| ENZRXN8J2 | lipoyl synthase | 0 | Not detected | -0.022 | -0.317 | 0.272 | 0.883 |
| ENZRXN8J2 | 4-nitrobenzaldehyde reductase | 0 | Not detected | -0.283 | -0.588 | 0.022 | 0.070 |
| ENZRXN8J2 | CTP synthetase | 0 | Not detected | -0.085 | -0.245 | 0.074 | 0.294 |
| ENZRXN8J2 | UTP-glucose-1-phosphate urid | 0 | Not detected | -0.065 | -0.286 | 0.156 | 0.564 |
| ENZRXN8J2 | 1,5-Î±-L-arabinofuranosidase | 0 | Not detected | -0.140 | -0.405 | 0.125 | 0.302 |
| ENZRXN8J2 | 1,3-Î±-L-arabinofuranosidase | 0 | Not detected | -0.098 | -0.271 | 0.075 | 0.268 |
| ENZRXNI.14 | fumarase | 0 | Not detected | -0.126 | -0.405 | 0.154 | 0.378 |
| ENZRXNIO2 | alcohol dehydrogenase | 0 | Not detected | -0.242 | -0.531 | 0.047 | 0.101 |
| ENZRXNME | oxepin-CoA hydrolase | 0 | Not detected | 0.209 | -0.022 | 0.440 | 0.076 |
| ENZRXNME | 3-oxo-5,6-dehydrosuberyl-CoA | 0 | Not detected | 0.209 | -0.022 | 0.440 | 0.076 |
| ENZRXNQT | biotin carboxylase | 0 | Not detected | -0.183 | -0.370 | 0.003 | 0.055 |
| ENZRXNQT | H+-exporting diphosphatase | 0 | Not detected | -0.082 | -0.406 | 0.243 | 0.622 |
| ENZRXNQT | NADP+-dependent isocitrate d | 0 | Not detected | -0.268 | -0.559 | 0.024 | 0.072 |
| ENZRXNQT | tryptophan synthase | 0 | Not detected | -0.074 | -0.383 | 0.234 | 0.638 |
| ENZRXNQT | aconitase | 0 | Not detected | 0.027 | -0.297 | 0.352 | 0.869 |
| ENZRXNQT | 3,4 dihydroxy-2-butanone-4-ph | 0 | Not detected | -0.096 | -0.259 | 0.067 | 0.250 |
| ENZRXNQT | GTP cyclohydrolase | 0 | Not detected | -0.096 | -0.259 | 0.067 | 0.250 |
| ENZRXNT9. | aconitase | 0 | Not detected | 0.027 | -0.297 | 0.352 | 0.869 |
| FADSYN.EN | FMN adenylyltransferase | 0 | Not detected | 0.092 | -0.129 | 0.312 | 0.416 |
| FGAMSYN.E | phosphoribosylformylglycinam | 0 | Not detected | 0.201 | -0.067 | 0.468 | 0.142 |
| FLAVONAD | flavodoxin-NADP+ reductase | 0 | Not detected | 0.068 | -0.121 | 0.257 | 0.481 |
| FOLYLPOLY | folylpoly-&gamma;-glutamate | 0 | Not detected | -0.021 | -0.248 | 0.206 | 0.859 |
| FORMYLTH | 10-formyl-tetrahydrofolylpolyg | 0 | Not detected | -0.021 | -0.248 | 0.206 | 0.859 |
| FPPSYN.EN | farnesyl diphosphate synthase | 0 | Not detected | 0.008 | -0.238 | 0.255 | 0.948 |
| FUCULOKIN | L-fuculokinase | 0 | Not detected | 0.210 | -0.107 | 0.526 | 0.194 |
| GALACTAR | galactarate dehydratase | 0 | Not detected | -0.159 | -0.447 | 0.129 | 0.278 |

| GALACTOK | galactokinase | 0 | Not detected | -0.166 | -0.402 | 0.069 | 0.167 |
| --- | --- | --- | --- | --- | --- | --- | --- |
| GALACTON | D-galactonate dehydratase | 0 | Not detected | 0.049 | -0.229 | 0.327 | 0.729 |
| GALACTUR | D-galacturonate isomerase | 0 | Not detected | -0.090 | -0.241 | 0.060 | 0.240 |
| GALPMUT.E | UDP-galactopyranose mutase | 0 | Not detected | -0.105 | -0.274 | 0.063 | 0.222 |
| GARTRANS | phosphoribosylglycinamide for | 0 | Not detected | -0.068 | -0.308 | 0.173 | 0.581 |
| GARTSIDE.E | acetate kinase | 0 | Not detected | -0.068 | -0.308 | 0.173 | 0.581 |
| GCVT.ENZR | aminomethyltransferase | 0 | Not detected | 0.029 | -0.192 | 0.251 | 0.796 |
| GDPPYPHO | GDP pyrophosphokinase | 0 | Not detected | 0.133 | -0.154 | 0.420 | 0.363 |
| GDPPYPHO | GDP diphosphokinase | 0 | Not detected | -0.007 | -0.315 | 0.300 | 0.963 |
| GKI.ENZRX | glycerate 2-kinase | 0 | Not detected | 0.048 | -0.221 | 0.317 | 0.727 |
| GLNS.ENZR | glutamine&mdash;tRNA ligase | 0 | Not detected | -0.088 | -0.239 | 0.063 | 0.252 |
| GLU6PDEH | NADP+-dependent glucose-6- | 0 | Not detected | 0.088 | -0.129 | 0.305 | 0.426 |
| GLUCARDE | D-glucarate dehydratase | 0 | Not detected | -0.268 | -0.553 | 0.017 | 0.066 |
| GLUCUROIS | D-glucuronate isomerase | 0 | Not detected | -0.090 | -0.241 | 0.060 | 0.240 |
| GLURS.ENZ | glutamate&mdash;tRNA ligase | 0 | Not detected | 0.115 | -0.205 | 0.436 | 0.482 |
| GLUTCYSLI | glutamate&mdash;cysteine liga | 0 | Not detected | 0.016 | -0.223 | 0.254 | 0.896 |
| GLUTRACE. | glutamate racemase | 0 | Not detected | 0.078 | -0.121 | 0.277 | 0.443 |
| GLYCEROL. | acyl-CoA:sn-glycerol-3-phosph | 0 | Not detected | 0.029 | -0.265 | 0.323 | 0.846 |
| GLYCOGEN | 1,4-Î±-glucan branching enzym | 0 | Not detected | 0.046 | -0.218 | 0.311 | 0.732 |
| GLYCOGEN | glycogen synthase | 0 | Not detected | 0.059 | -0.189 | 0.307 | 0.642 |
| GLYRIBON | phosphoribosylamine&mdash; | 0 | Not detected | -0.036 | -0.191 | 0.119 | 0.647 |
| GPPSYN.EN | geranyl diphosphate synthase | 0 | Not detected | 0.008 | -0.238 | 0.255 | 0.948 |
| GSADENYL | glutamine synthetase adenylylt | 0 | Not detected | 0.142 | -0.161 | 0.446 | 0.358 |
| GSDEADEN | glutamine synthetase deadenyla | 0 | Not detected | 0.142 | -0.161 | 0.446 | 0.358 |
| GTPPYPHOS | GTP pyrophosphokinase | 0 | Not detected | 0.133 | -0.154 | 0.420 | 0.363 |
| HEMEOSYN | heme O synthase | 0 | Not detected | -0.098 | -0.301 | 0.105 | 0.344 |
| HEMN.ENZR | coproporphyrinogen III dehydr | 0 | Not detected | -0.134 | -0.402 | 0.135 | 0.330 |
| HISTCYCLO | phosphoribosyl-AMP cyclohyd | 0 | Not detected | -0.149 | -0.322 | 0.024 | 0.093 |
| HISTPRATP | phosphoribosyl-ATP pyrophos | 0 | Not detected | -0.149 | -0.322 | 0.024 | 0.093 |
| HOMOCYSM | cobalamin-independent homoc | 0 | Not detected | -0.051 | -0.344 | 0.243 | 0.735 |
| HOMOCYSM | methionine synthase | 0 | Not detected | -0.094 | -0.245 | 0.057 | 0.222 |
| IGPSYN.EN | indole-3-glycerol phosphate sy | 0 | Not detected | 0.136 | -0.103 | 0.376 | 0.266 |
| ILES.ENZRX | isoleucine&mdash;tRNA ligase | 0 | Not detected | 0.081 | -0.255 | 0.418 | 0.636 |
| IMPCYCLO | IMP cyclohydrolase | 0 | Not detected | 0.154 | -0.055 | 0.363 | 0.150 |
| KDOTRANS | 3-deoxy-D-manno-octulosonic | 0 | Not detected | 0.007 | -0.239 | 0.253 | 0.954 |
| KDOTRANS | KDO transferase | 0 | Not detected | 0.007 | -0.239 | 0.253 | 0.954 |
| L.ASPARTA | L-aspartate oxidase | 0 | Not detected | 0.120 | -0.227 | 0.468 | 0.497 |
| L.LACTDEH | L-lactate dehydrogenase | 0 | Not detected | 0.139 | -0.114 | 0.393 | 0.282 |
| LAUROYLA | lauroyl acyltransferase | 0 | Not detected | 0.026 | -0.188 | 0.240 | 0.810 |
| LEUS.ENZR | leucine&mdash;tRNA ligase | 0 | Not detected | 0.207 | -0.126 | 0.541 | 0.224 |
| LSERINEDE | L-serine deaminase | 345 | Not detected | 0.434 | 0.102 | 0.766 | 0.011 |
| LSERINEDE | L-serine deaminase | 0 | Not detected | 0.013 | -0.226 | 0.252 | 0.917 |
| MALATESY | malate synthase A | 0 | Not detected | 0.108 | -0.137 | 0.352 | 0.388 |
| MALONYL. | [acyl-carrier-protein] S-malony | 0 | Not detected | -0.059 | -0.350 | 0.232 | 0.690 |

| MALSYNG.E | malate synthase G | 0 | Not detected | 0.060 | -0.192 | 0.313 | 0.640 |
| --- | --- | --- | --- | --- | --- | --- | --- |
| MALTODEX | maltodextrin glucosidase | 0 | Not detected | 0.195 | -0.046 | 0.436 | 0.113 |
| MANNKIN.E | mannokinase | 0 | Not detected | 0.149 | -0.053 | 0.350 | 0.148 |
| MANNOND | D-mannonate dehydratase | 0 | Not detected | -0.029 | -0.243 | 0.184 | 0.788 |
| MANNONO | D-mannonate oxidoreductase | 0 | Not detected | -0.017 | -0.290 | 0.257 | 0.905 |
| MANNPDEH | mannitol-1-phosphate 5-dehyd | 0 | Not detected | 0.030 | -0.270 | 0.330 | 0.843 |
| MANNPISO | mannose-6-phosphate isomeras | 0 | Not detected | 0.117 | -0.080 | 0.313 | 0.245 |
| MHPHYDRO | 3-(3-hydroxyphenyl)propanoat | 0 | Not detected | 0.238 | 0.009 | 0.467 | 0.042 |
| MMUM.ENZ | homocysteine S-methyltransfer | 0 | Not detected | -0.066 | -0.303 | 0.172 | 0.588 |
| N.ACETYLG | N-acetylglutamylphosphate red | 0 | Not detected | 0.077 | -0.206 | 0.360 | 0.595 |
| NACGLCTR | N-acetylglucosaminyl transfera | 0 | Not detected | 0.123 | -0.102 | 0.347 | 0.285 |
| NACMURLA | N-acetylmuramoyl-L-alanine a | 0 | Not detected | 0.057 | -0.179 | 0.293 | 0.634 |
| NADH.DHII. | NADH:ubiquinone oxidoreduc | 0 | Not detected | 0.009 | -0.218 | 0.236 | 0.938 |
| NANK.ENZR | N-acetylmannosamine kinase | 0 | Not detected | 0.032 | -0.181 | 0.246 | 0.767 |
| NICOTINAT | nicotinate phosphoribosyltrans | 0 | Not detected | 0.077 | -0.130 | 0.285 | 0.464 |
| O.SUCCINY | o-succinylbenzoate synthase | 0 | Not detected | -0.034 | -0.255 | 0.187 | 0.764 |
| OCTPRNL.M | 2-octaprenyl-3-methyl-6-metho | 0 | Not detected | -0.052 | -0.270 | 0.166 | 0.642 |
| OHMETHYL | hydroxymethylbilane synthase | 0 | Not detected | -0.091 | -0.310 | 0.129 | 0.419 |
| PEPCARBO | phosphoenolpyruvate carboxyk | 0 | Not detected | -0.095 | -0.248 | 0.057 | 0.221 |
| PGLYCERO | phosphoglycerol transferase | 0 | Not detected | 0.006 | -0.256 | 0.267 | 0.966 |
| PGMI.ENZR | 2,3-bisphosphoglycerate-indep | 0 | Not detected | -0.096 | -0.248 | 0.056 | 0.217 |
| PHOSGLUC | phosphoglucosamine mutase | 0 | Not detected | 0.317 | 0.021 | 0.612 | 0.036 |
| PHOSGLYC | phosphoglycerate kinase | 0 | Not detected | 0.160 | -0.125 | 0.445 | 0.271 |
| PHOSMANM | phosphomannomutase | 0 | Not detected | -0.147 | -0.482 | 0.187 | 0.388 |
| PHOSNACM | phospho-N-acetylmuramoyl-pe | 0 | Not detected | 0.148 | -0.151 | 0.446 | 0.332 |
| PHOSPHASE | phosphatidylserine synthase | 0 | Not detected | -0.180 | -0.441 | 0.081 | 0.176 |
| PHOSPHOG | phosphoglucomutase | 0 | Not detected | 0.195 | -0.083 | 0.472 | 0.169 |
| PPENTOMU | Î±-D-ribose 1,5-phosphomutas | 0 | Not detected | -0.006 | -0.337 | 0.326 | 0.972 |
| PRAISOM.E | phosphoribosylanthranilate iso | 0 | Not detected | 0.136 | -0.103 | 0.376 | 0.266 |
| PRIBFAICA | 1-(5-phosphoribosyl)-5-[(5-ph | 0 | Not detected | 0.206 | -0.067 | 0.480 | 0.140 |
| PROPKIN.E | propionate kinase | 0 | Not detected | 0.027 | -0.182 | 0.236 | 0.799 |
| PROPKIN2.E | propionate kinase | 0 | Not detected | 0.054 | -0.184 | 0.292 | 0.658 |
| PROTOHEM | ferrochelatase | 0 | Not detected | -0.179 | -0.402 | 0.045 | 0.117 |
| PRPPSYN.E | ribose-phosphate diphosphokin | 0 | Not detected | -0.025 | -0.190 | 0.139 | 0.762 |
| PSERPHOSP | phosphoserine phosphatase | 0 | Not detected | 0.035 | -0.189 | 0.259 | 0.758 |
| PSII.ENZRX | guanosine-3',5'-bis(diphosphat | 0 | Not detected | -0.007 | -0.315 | 0.300 | 0.963 |
| RHAMNULO | rhamnulokinase | 193 | Not detected | 0.297 | 0.054 | 0.540 | 0.017 |
| RIBOFLAVI | riboflavin kinase | 0 | Not detected | 0.092 | -0.129 | 0.312 | 0.416 |
| RIBOPHOSP | 5-amino-6-(5-phospho-D-ribity | 0 | Not detected | 0.080 | -0.112 | 0.271 | 0.415 |
| RIBULP3EPI | ribulose-phosphate 3-epimeras | 0 | Not detected | -0.085 | -0.298 | 0.129 | 0.438 |
| SARCOX.EN | N-methyl-L-tryptophan oxidas | 0 | Not detected | 0.067 | -0.131 | 0.265 | 0.507 |
| SHIKIMATE | shikimate dehydrogenase | 0 | Not detected | -0.043 | -0.247 | 0.161 | 0.677 |
| SHIKIMATE | shikimate kinase | 0 | Not detected | -0.015 | -0.217 | 0.187 | 0.886 |
| SUCCGLUA | succinylglutamate semialdehyd | 0 | Not detected | 0.092 | -0.144 | 0.328 | 0.443 |

| TDPFUCAC | dTDP-4-amino-4,6-dideoxy-D- | 0 | Not detected | 0.050 | -0.138 | 0.239 | 0.602 |
| --- | --- | --- | --- | --- | --- | --- | --- |
| TETRAACY | tetraacyldisaccharide 4'-kinase | 0 | Not detected | 0.102 | -0.104 | 0.309 | 0.333 |
| THI.P.KIN.E | thiamine monophosphate kinas | 0 | Not detected | -0.034 | -0.252 | 0.183 | 0.759 |
| THIC.ENZR | phosphomethylpyrimidine synt | 0 | Not detected | -0.092 | -0.244 | 0.061 | 0.238 |
| THRESYN.E | threonine synthase | 0 | Not detected | 0.170 | -0.100 | 0.441 | 0.217 |
| TRANS.ENZ | transport of arabinose | 0 | Not detected | 0.172 | -0.126 | 0.470 | 0.259 |
| TRANS.ENZ | (S)-lactate transporter | 0 | Not detected | 0.078 | -0.185 | 0.342 | 0.560 |
| TRANS.ENZ | glycolate transporter | 0 | Not detected | 0.044 | -0.256 | 0.344 | 0.774 |
| TRANS.ENZ | succinate:fumarate antiport | 0 | Not detected | 0.095 | -0.203 | 0.393 | 0.532 |
| TRANS.ENZ | fumarate:succinate antiporter | 0 | Not detected | 0.120 | -0.140 | 0.380 | 0.365 |
| TRANS.ENZ | transport of adenosine | 0 | Not detected | 0.153 | -0.112 | 0.418 | 0.258 |
| TRANS.ENZ | transport of cytidine | 0 | Not detected | 0.153 | -0.112 | 0.418 | 0.258 |
| TRANS.ENZ | transport of deoxyadenosine | 0 | Not detected | 0.153 | -0.112 | 0.418 | 0.258 |
| TRANS.ENZ | transport of deoxycytidine | 0 | Not detected | 0.153 | -0.112 | 0.418 | 0.258 |
| TRANS.ENZ | transport of deoxyuridine | 0 | Not detected | 0.153 | -0.112 | 0.418 | 0.258 |
| TRANS.ENZ | transport of thymidine | 0 | Not detected | 0.153 | -0.112 | 0.418 | 0.258 |
| TRANS.ENZ | transport of uridine | 0 | Not detected | 0.153 | -0.112 | 0.418 | 0.258 |
| TRANS.ENZ | transport of rhamnose | 0 | Not detected | 0.017 | -0.194 | 0.227 | 0.876 |
| TRANS.ENZ | phosphate:H+ symporter | 0 | Not detected | 0.216 | -0.032 | 0.463 | 0.088 |
| TRANS.ENZ | cytosine transporter | 0 | Not detected | 0.038 | -0.216 | 0.293 | 0.769 |
| TRANS.ENZ | pantothenate:Na+ symporter | 0 | Not detected | 0.085 | -0.178 | 0.349 | 0.525 |
| TRANS.ENZ | proline:Na+ symporter | 0 | Not detected | 0.165 | -0.144 | 0.474 | 0.295 |
| TRANS.ENZ | transport of succinate | 0 | Not detected | 0.099 | -0.212 | 0.410 | 0.532 |
| TRANS.ENZ | transport of malate | 0 | Not detected | 0.099 | -0.212 | 0.410 | 0.532 |
| TRANS.ENZ | transport of fumarate | 0 | Not detected | 0.099 | -0.212 | 0.410 | 0.532 |
| TRANS.ENZ | transport of orotate | 0 | Not detected | 0.099 | -0.212 | 0.410 | 0.532 |
| TRANS.ENZ | L-glutamate:H+ symporter | 0 | Not detected | -0.017 | -0.257 | 0.223 | 0.888 |
| TRANS.ENZ | L-aspartate:H+ symporter | 0 | Not detected | -0.017 | -0.257 | 0.223 | 0.888 |
| TRANS.ENZ | transport of glutamate | 0 | Not detected | 0.000 | -0.259 | 0.259 | 1.000 |
| TRANS.ENZ | branched chain amino acid tran | 0 | Not detected | 0.156 | -0.069 | 0.382 | 0.174 |
| TRANS.ENZ | branched chain amino acid tran | 0 | Not detected | 0.156 | -0.069 | 0.382 | 0.174 |
| TRANS.ENZ | branched chain amino acid tran | 0 | Not detected | 0.156 | -0.069 | 0.382 | 0.174 |
| TRANS.ENZ | L-tartrate:succinate antiporter | 0 | Not detected | 0.074 | -0.148 | 0.296 | 0.515 |
| TRANS.ENZ | Na+:H+ antiporter | 0 | Not detected | 0.029 | -0.240 | 0.298 | 0.833 |
| TRANS.ENZ | Mg2+ transporter | 0 | Not detected | 0.022 | -0.211 | 0.255 | 0.853 |
| TRANS.ENZ | Co2+ transporter | 0 | Not detected | 0.022 | -0.211 | 0.255 | 0.853 |
| TRANS.ENZ | Ni2+ transporter | 0 | Not detected | 0.022 | -0.211 | 0.255 | 0.853 |
| TRANS.ENZ | K+ : H+ antiporter KefB | 0 | Not detected | 0.054 | -0.200 | 0.308 | 0.675 |
| TRANS.ENZ | K+:H+ symporter Kup | 0 | Not detected | 0.212 | -0.048 | 0.471 | 0.110 |
| TRANS.ENZ | fucose:H+ symporter | 0 | Not detected | 0.112 | -0.109 | 0.333 | 0.321 |
| TRANS.ENZ | shikimate:H+ symporter | 0 | Not detected | 0.133 | -0.073 | 0.339 | 0.206 |
| TRANS.ENZ | fosmidomycin efflux pump | 0 | Not detected | -0.106 | -0.289 | 0.078 | 0.259 |
| TRANS.ENZ | transport of 3-(3-hydroxypheny | 0 | Not detected | 0.290 | 0.073 | 0.507 | 0.009 |
| TRANS.ENZ | sugar exporter SetB | 0 | Not detected | -0.029 | -0.263 | 0.205 | 0.808 |

| TRANS.ENZ | transport of D-galactose | 0 | Not detected | 0.095 | -0.134 | 0.325 | 0.416 |
| --- | --- | --- | --- | --- | --- | --- | --- |
| TRANS.ENZ | Mn2+:H+ symporter | 0 | Not detected | 0.029 | -0.180 | 0.238 | 0.786 |
| TRANS.ENZ | L-threonine:H+ antiporter | 0 | Not detected | 0.006 | -0.233 | 0.245 | 0.960 |
| TRANS.ENZ | K+ transporter TrkH | 0 | Not detected | 0.103 | -0.159 | 0.365 | 0.442 |
| TRANS.ENZ | sn-glycerol 3-phosphate:phosp | 0 | Not detected | 0.063 | -0.216 | 0.342 | 0.658 |
| TRANS.ENZ | transport of 2-oxoglutarate | 0 | Not detected | 0.160 | -0.054 | 0.375 | 0.144 |
| TRANS.ENZ | lactose:H+ symporter LacY | 0 | Not detected | 0.015 | -0.202 | 0.232 | 0.890 |
| TRANS.ENZ | N-acetylneuraminate:H+ symp | 0 | Not detected | -0.091 | -0.322 | 0.139 | 0.437 |
| TRANS.ENZ | transport of a nucleoside | 0 | Not detected | 0.333 | -0.012 | 0.679 | 0.059 |
| TRANS.ENZ | transport of D-xylose | 0 | Not detected | -0.012 | -0.260 | 0.236 | 0.923 |
| TRANS.ENZ | xanthosine:H+ symporter XapB | 0 | Not detected | -0.120 | -0.374 | 0.133 | 0.353 |
| TRANS.ENZ | glucose-6-phosphate:phosphate | 0 | Not detected | 0.205 | -0.039 | 0.450 | 0.100 |
| TRANS.ENZ | D-glucuronate transporter | 0 | Not detected | 0.023 | -0.201 | 0.247 | 0.840 |
| TRANS.ENZ | D-galacturonate transporter | 0 | Not detected | 0.023 | -0.201 | 0.247 | 0.840 |
| TRANS.ENZ | L-arabinose exporter | 0 | Not detected | -0.010 | -0.207 | 0.187 | 0.921 |
| TRANS.ENZ | L-phenylalanine:H+ symporter | 0 | Not detected | 0.073 | -0.199 | 0.345 | 0.599 |
| TRANS.ENZ | L-tyrosine:H+ symporter | 0 | Not detected | 0.073 | -0.199 | 0.345 | 0.599 |
| TRANS.ENZ | L-tryptophan:H+ symporter | 0 | Not detected | 0.073 | -0.199 | 0.345 | 0.599 |
| TRANS.ENZ | 4-aminobutanoate:H+ symport | 0 | Not detected | 0.072 | -0.145 | 0.288 | 0.516 |
| TRANS.ENZ | lysine:H+ symporter | 0 | Not detected | 0.240 | -0.078 | 0.557 | 0.139 |
| TRANS.ENZ | phenylalanine:H+ symporter P | 0 | Not detected | 0.325 | 0.034 | 0.616 | 0.029 |
| TRANS.ENZ | transport of D-alanine | 0 | Not detected | -0.135 | -0.410 | 0.140 | 0.337 |
| TRANS.ENZ | transport of glycine | 0 | Not detected | -0.135 | -0.410 | 0.140 | 0.337 |
| TRANS.ENZ | lysine:cadaverine antiporter | 0 | Not detected | -0.022 | -0.316 | 0.271 | 0.881 |
| TRANS.ENZ | serine:H+ symporter SdaC | 0 | Not detected | 0.116 | -0.159 | 0.392 | 0.408 |
| TRANS.ENZ | L-threonine:H+ symporter | 0 | Not detected | 0.090 | -0.132 | 0.311 | 0.427 |
| TRANS.ENZ | L-serine:H+ symporter | 0 | Not detected | 0.090 | -0.132 | 0.311 | 0.427 |
| TRANS.ENZ | tryptophan:H+ symporter | 0 | Not detected | 0.096 | -0.157 | 0.349 | 0.457 |
| TRANS.ENZ | indole:H+ symporter | 0 | Not detected | 0.096 | -0.157 | 0.349 | 0.457 |
| TRANS.ENZ | choline:H+ symporter | 0 | Not detected | 0.083 | -0.174 | 0.341 | 0.527 |
| TRE6PHYDR | trehalose-6-phosphate hydrolas | 0 | Not detected | 0.012 | -0.298 | 0.323 | 0.938 |
| TREHALAC | trehalase | 0 | Not detected | -0.026 | -0.272 | 0.220 | 0.834 |
| TREHALAPE | trehalase | 0 | Not detected | 0.030 | -0.188 | 0.248 | 0.788 |
| TREHALOSE | trehalose-6-phosphate synthase | 0 | Not detected | 0.078 | -0.132 | 0.289 | 0.466 |
| TSA.REDUC | tartronate semialdehyde reduct | 0 | Not detected | -0.029 | -0.286 | 0.228 | 0.827 |
| UDP.NACM | UDP-N-acetylmuramoyl-L-ala | 0 | Not detected | 0.172 | -0.079 | 0.423 | 0.180 |
| UDP.NACM | D-alanyl-D-alanine-adding enz | 0 | Not detected | 0.194 | -0.050 | 0.438 | 0.119 |
| UDP.NACM | UDP-N-acetylmuramoyl-L-ala | 0 | Not detected | -0.039 | -0.303 | 0.225 | 0.770 |
| UDPACYLG | UDP-3-O-acyl-N-acetylglucosa | 0 | Not detected | 0.206 | -0.108 | 0.521 | 0.199 |
| UDPMANAC | UDP-N-acetyl-D-mannosamin | 0 | Not detected | 0.046 | -0.148 | 0.240 | 0.644 |
| UDPNACET | UDP-N-acetylglucosamine 1-c | 0 | Not detected | -0.089 | -0.379 | 0.200 | 0.545 |
| UDPNACET | UDP-N-acetylenolpyruvoylglu | 0 | Not detected | 0.010 | -0.222 | 0.242 | 0.932 |
| UDPSUGAR | UDP-sugar hydrolase | 0 | Not detected | 0.038 | -0.225 | 0.301 | 0.778 |
| URITRANS. | [protein-PII] uridylyltransferas | 0 | Not detected | 0.063 | -0.209 | 0.335 | 0.652 |

| URKI.ENZR | uridine kinase | 0 | Not detected | -0.049 | -0.349 | 0.251 | 0.749 |
| --- | --- | --- | --- | --- | --- | --- | --- |
| UROGENDE | uroporphyrinogen decarboxyla | 0 | Not detected | 0.007 | -0.298 | 0.312 | 0.964 |
| VALINE.PY | valine&mdash;pyruvate aminot | 0 | Not detected | 0.032 | -0.192 | 0.256 | 0.777 |
| VALS.ENZR | valine&mdash;tRNA ligase | 0 | Not detected | 0.141 | -0.204 | 0.485 | 0.424 |
| X1.ACYLGL | acyl-CoA:1-acyl-sn-glycerol-3- | 0 | Not detected | -0.096 | -0.302 | 0.110 | 0.359 |
| X2.DEHYDR | 2-dehydropantoate 2-reductase | 0 | Not detected | 0.131 | -0.066 | 0.327 | 0.194 |
| X2.OCTAPR | 2-octaprenyl-6-methoxy-1,4-be | 0 | Not detected | 0.029 | -0.277 | 0.334 | 0.855 |
| X2.OCTPRN | 2-octaprenyl-6-methoxyphenol | 0 | Not detected | 0.033 | -0.191 | 0.256 | 0.775 |
| X3.DEHYDR | 3-dehydroquinate synthase | 0 | Not detected | 0.123 | -0.149 | 0.395 | 0.375 |
| X3.NUCLEO | 3'-nucleotidase | 0 | Not detected | 0.106 | -0.182 | 0.395 | 0.470 |
| X3.P.SHIK.1 | 3-phosphoshikimate 1-carboxy | 0 | Not detected | -0.113 | -0.364 | 0.137 | 0.374 |
| X4OHBENZ | 4-hydroxybenzoate octaprenylt | 0 | Not detected | 0.032 | -0.171 | 0.235 | 0.757 |
| X5.NUCLEO | 5'-ribonucleotidase | 0 | Not detected | 0.038 | -0.225 | 0.301 | 0.778 |
| X6PGLUCO | 6-phosphogluconolactonase | 0 | Not detected | 0.120 | -0.073 | 0.313 | 0.224 |

| **Supplementary Table 15.** Association of current education (higher SEP vs. Lower SEP) with species in overall participants (n=2273). Analysis of Compositions of Microbiomes (ANCOM2) was used to identify species related to current education. Regression coefficients were obtained by modeling the effect of current education on centered log ratio (CLR)-transformed species in multivariable linear regression adjusted for age, gender, BMI, field center, Hispanic/Latino background, U.S. nativity, Bristol stool type, parental education, the alternative Healthy Eating Index 2010 score, cigarette smoking, alcohol use, depressive and anxiety symptoms, diabetes, hypertension, cardiovascular disease, dyslipidemia, and antibiotic use. | | | | | | | |
| --- | --- | --- | --- | --- | --- | --- | --- |
| **species** | **model** | **W_stat** | **ANCOM** | **beta** | **l95ci** | **u95ci** | **qval** |
| Bacteroides finegoldii | cov3 | 1021 | 0.8 | -0.274 | -0.442 | -0.106 | 0.001 |
| Bacteroides finegoldii CA | cov3 | 910 | 0.7 | -0.255 | -0.413 | -0.098 | 0.001 |
| Bacteroides salyersiae | cov3 | 981 | 0.8 | -0.236 | -0.362 | -0.109 | 0.000 |
| Bacteroides sp. CAG:98 | cov3 | 1119 | 0.9 | -0.385 | -0.617 | -0.154 | 0.001 |
| Bacteroides stercoris | cov3 | 834 | 0.7 | -0.247 | -0.412 | -0.083 | 0.003 |
| Bifidobacterium angulatum | cov3 | 984 | 0.8 | 0.342 | 0.117 | 0.568 | 0.003 |
| Bifidobacterium dentium | cov3 | 884 | 0.7 | 0.324 | 0.076 | 0.572 | 0.011 |
| Clostridium clostridioform | cov3 | 862 | 0.7 | -0.280 | -0.465 | -0.095 | 0.003 |
| Clostridium clostridioform | cov3 | 964 | 0.8 | -0.321 | -0.491 | -0.152 | 0.000 |
| Empedobacter brevis | cov3 | 833 | 0.7 | -0.323 | -0.581 | -0.066 | 0.014 |
| Porphyromonas sp. COT-2 | cov3 | 865 | 0.7 | -0.297 | -0.506 | -0.088 | 0.005 |
| [Bacteroides] pectinophilu | cov3 | 0 | Not detected | -0.010 | -0.083 | 0.062 | 0.784 |
| [Clostridium] aminophilu | cov3 | 0 | Not detected | 0.044 | -0.021 | 0.110 | 0.185 |
| [Clostridium] asparagiform | cov3 | 0 | Not detected | -0.089 | -0.184 | 0.006 | 0.067 |
| [Clostridium] bolteae | cov3 | 0 | Not detected | -0.089 | -0.177 | -0.001 | 0.048 |
| [Clostridium] celerecresce | cov3 | 0 | Not detected | -0.040 | -0.102 | 0.021 | 0.198 |
| [Clostridium] cellobioparu | cov3 | 0 | Not detected | -0.002 | -0.142 | 0.138 | 0.980 |
| [Clostridium] cellulolyticu | cov3 | 0 | Not detected | 0.029 | -0.129 | 0.186 | 0.721 |
| [Clostridium] cellulosi | cov3 | 0 | Not detected | 0.150 | -0.008 | 0.307 | 0.062 |
| [Clostridium] citroniae | cov3 | 0 | Not detected | -0.077 | -0.147 | -0.007 | 0.032 |
| [Clostridium] clariflavum | cov3 | 0 | Not detected | 0.075 | -0.062 | 0.212 | 0.281 |
| [Clostridium] clostridiofor | cov3 | 89 | Not detected | -0.152 | -0.245 | -0.058 | 0.002 |
| [Clostridium] dakarense | cov3 | 0 | Not detected | 0.336 | 0.124 | 0.547 | 0.002 |
| [Clostridium] glycyrrhizin | cov3 | 0 | Not detected | -0.045 | -0.106 | 0.017 | 0.154 |
| [Clostridium] hiranonis | cov3 | 0 | Not detected | 0.110 | -0.080 | 0.300 | 0.255 |
| [Clostridium] hylemonae | cov3 | 0 | Not detected | -0.147 | -0.280 | -0.013 | 0.031 |
| [Clostridium] innocuum | cov3 | 0 | Not detected | 0.020 | -0.046 | 0.086 | 0.559 |
| [Clostridium] josui | cov3 | 0 | Not detected | 0.016 | -0.145 | 0.177 | 0.844 |
| [Clostridium] lactatiferme | cov3 | 0 | Not detected | -0.055 | -0.137 | 0.026 | 0.184 |
| [Clostridium] leptum | cov3 | 0 | Not detected | -0.021 | -0.126 | 0.085 | 0.703 |
| [Clostridium] methylpento | cov3 | 0 | Not detected | -0.051 | -0.157 | 0.055 | 0.349 |
| [Clostridium] papyrosolve | cov3 | 0 | Not detected | -0.025 | -0.147 | 0.097 | 0.691 |
| [Clostridium] saccharogum | cov3 | 0 | Not detected | -0.009 | -0.104 | 0.085 | 0.844 |
| [Clostridium] saccharolyti | cov3 | 0 | Not detected | -0.039 | -0.126 | 0.047 | 0.370 |
| [Clostridium] scindens | cov3 | 0 | Not detected | -0.044 | -0.120 | 0.031 | 0.251 |
| [Clostridium] spiroforme | cov3 | 0 | Not detected | -0.098 | -0.275 | 0.078 | 0.274 |
| [Clostridium] sporosphaer | cov3 | 0 | Not detected | 0.046 | -0.094 | 0.187 | 0.519 |

| [Clostridium] straminisolv | cov3 | 0 | Not detected | 0.095 | -0.019 | 0.210 | 0.103 |
| --- | --- | --- | --- | --- | --- | --- | --- |
| [Clostridium] symbiosum | cov3 | 0 | Not detected | -0.072 | -0.148 | 0.005 | 0.067 |
| [Clostridium] viride | cov3 | 0 | Not detected | 0.022 | -0.112 | 0.156 | 0.749 |
| [Enterobacter] aerogenes | cov3 | 0 | Not detected | 0.098 | -0.198 | 0.393 | 0.517 |
| [Eubacterium] brachy | cov3 | 0 | Not detected | 0.014 | -0.189 | 0.218 | 0.891 |
| [Eubacterium] cellulosolve | cov3 | 0 | Not detected | -0.025 | -0.109 | 0.059 | 0.557 |
| [Eubacterium] contortum | cov3 | 0 | Not detected | -0.024 | -0.082 | 0.035 | 0.426 |
| [Eubacterium] dolichum | cov3 | 0 | Not detected | -0.001 | -0.060 | 0.059 | 0.978 |
| [Eubacterium] eligens | cov3 | 0 | Not detected | 0.023 | -0.129 | 0.175 | 0.765 |
| [Eubacterium] fissicatena | cov3 | 0 | Not detected | -0.021 | -0.100 | 0.059 | 0.608 |
| [Eubacterium] hallii | cov3 | 0 | Not detected | 0.014 | -0.088 | 0.116 | 0.792 |
| [Eubacterium] nodatum | cov3 | 0 | Not detected | 0.037 | -0.149 | 0.223 | 0.698 |
| [Eubacterium] rectale | cov3 | 0 | Not detected | 0.101 | -0.046 | 0.249 | 0.179 |
| [Eubacterium] siraeum | cov3 | 0 | Not detected | 0.138 | -0.089 | 0.365 | 0.235 |
| [Eubacterium] sulci | cov3 | 0 | Not detected | -0.110 | -0.311 | 0.091 | 0.283 |
| [Eubacterium] yurii | cov3 | 0 | Not detected | 0.037 | -0.080 | 0.155 | 0.536 |
| [Haemophilus] ducreyi | cov3 | 0 | Not detected | 0.006 | -0.139 | 0.152 | 0.930 |
| [Haemophilus] parasuis | cov3 | 0 | Not detected | 0.105 | -0.070 | 0.279 | 0.240 |
| [Ruminococcus] gnavus | cov3 | 0 | Not detected | -0.061 | -0.180 | 0.057 | 0.312 |
| [Ruminococcus] torques | cov3 | 0 | Not detected | -0.123 | -0.249 | 0.002 | 0.055 |
| Abiotrophia defectiva | cov3 | 0 | Not detected | 0.001 | -0.183 | 0.184 | 0.995 |
| Acetanaerobacterium elon | cov3 | 0 | Not detected | 0.142 | 0.014 | 0.269 | 0.029 |
| Acetitomaculum ruminis | cov3 | 0 | Not detected | 0.052 | -0.022 | 0.126 | 0.171 |
| Acetivibrio cellulolyticus | cov3 | 0 | Not detected | 0.060 | -0.089 | 0.210 | 0.430 |
| Acetivibrio ethanolgignens | cov3 | 0 | Not detected | -0.017 | -0.080 | 0.046 | 0.606 |
| Acetobacter sp. 46_36 | cov3 | 0 | Not detected | 0.015 | -0.273 | 0.304 | 0.917 |
| Acetobacter sp. CAG:267 | cov3 | 0 | Not detected | -0.259 | -0.521 | 0.003 | 0.053 |
| Acholeplasma axanthum | cov3 | 0 | Not detected | 0.067 | -0.062 | 0.196 | 0.308 |
| Acholeplasma equifetale | cov3 | 0 | Not detected | -0.029 | -0.204 | 0.147 | 0.750 |
| Acidaminococcus ferment | cov3 | 0 | Not detected | 0.034 | -0.092 | 0.160 | 0.598 |
| Acidaminococcus intestini | cov3 | 0 | Not detected | -0.064 | -0.294 | 0.166 | 0.585 |
| Acidaminococcus intestini | cov3 | 0 | Not detected | -0.267 | -0.628 | 0.094 | 0.148 |
| Acinetobacter baumannii | cov3 | 0 | Not detected | 0.039 | -0.112 | 0.191 | 0.611 |
| Acinetobacter bereziniae | cov3 | 0 | Not detected | 0.065 | -0.084 | 0.215 | 0.391 |
| Acinetobacter johnsonii | cov3 | 0 | Not detected | 0.117 | -0.047 | 0.281 | 0.162 |
| Acinetobacter sp. CAG:19 | cov3 | 0 | Not detected | -0.072 | -0.377 | 0.234 | 0.645 |
| Acinetobacter sp. CAG:19 | cov3 | 0 | Not detected | -0.048 | -0.344 | 0.249 | 0.753 |
| Acinetobacter sp. N54.MG | cov3 | 0 | Not detected | -0.068 | -0.228 | 0.093 | 0.411 |
| Acinetobacter venetianus | cov3 | 0 | Not detected | 0.112 | -0.068 | 0.292 | 0.224 |
| Actinobaculum sp. oral tax | cov3 | 0 | Not detected | -0.161 | -0.374 | 0.052 | 0.139 |
| Actinobaculum suis | cov3 | 0 | Not detected | 0.002 | -0.152 | 0.155 | 0.984 |
| Actinomyces dentalis | cov3 | 0 | Not detected | 0.158 | -0.062 | 0.378 | 0.159 |
| Actinomyces gerencseriae | cov3 | 0 | Not detected | 0.037 | -0.162 | 0.236 | 0.716 |
| Actinomyces graevenitzii | cov3 | 0 | Not detected | -0.013 | -0.156 | 0.131 | 0.862 |

| Actinomyces hongkongens | cov3 | 0 | Not detected | 0.016 | -0.182 | 0.214 | 0.877 |
| --- | --- | --- | --- | --- | --- | --- | --- |
| Actinomyces israelii | cov3 | 0 | Not detected | 0.139 | -0.070 | 0.347 | 0.192 |
| Actinomyces johnsonii | cov3 | 0 | Not detected | -0.071 | -0.273 | 0.131 | 0.494 |
| Actinomyces massiliensis | cov3 | 0 | Not detected | 0.034 | -0.172 | 0.239 | 0.748 |
| Actinomyces meyeri | cov3 | 0 | Not detected | 0.017 | -0.157 | 0.190 | 0.851 |
| Actinomyces odontolyticu | cov3 | 0 | Not detected | 0.014 | -0.138 | 0.167 | 0.855 |
| Actinomyces oris | cov3 | 0 | Not detected | 0.099 | -0.084 | 0.282 | 0.289 |
| Actinomyces sp. HMSC06 | cov3 | 0 | Not detected | -0.038 | -0.157 | 0.082 | 0.538 |
| Actinomyces sp. HMSC06 | cov3 | 0 | Not detected | -0.081 | -0.203 | 0.041 | 0.191 |
| Actinomyces sp. oral taxon | cov3 | 0 | Not detected | 0.099 | -0.096 | 0.294 | 0.320 |
| Actinomyces sp. oral taxon | cov3 | 0 | Not detected | 0.160 | -0.073 | 0.392 | 0.179 |
| Actinomyces sp. oral taxon | cov3 | 0 | Not detected | 0.042 | -0.111 | 0.195 | 0.592 |
| Actinomyces sp. ph3 | cov3 | 0 | Not detected | 0.016 | -0.172 | 0.205 | 0.865 |
| Actinomyces timonensis | cov3 | 0 | Not detected | 0.047 | -0.094 | 0.187 | 0.514 |
| Actinomyces turicensis | cov3 | 0 | Not detected | 0.046 | -0.157 | 0.250 | 0.656 |
| Adlercreutzia equolifacien | cov3 | 0 | Not detected | 0.055 | -0.185 | 0.294 | 0.654 |
| Aeromonas salmonicida | cov3 | 0 | Not detected | -0.074 | -0.268 | 0.120 | 0.455 |
| Aggregatibacter aphrophil | cov3 | 0 | Not detected | 0.068 | -0.149 | 0.285 | 0.538 |
| Akkermansia glycaniphila | cov3 | 0 | Not detected | -0.048 | -0.247 | 0.151 | 0.635 |
| Akkermansia muciniphila | cov3 | 0 | Not detected | -0.002 | -0.259 | 0.256 | 0.991 |
| Akkermansia muciniphila | cov3 | 0 | Not detected | 0.004 | -0.332 | 0.340 | 0.981 |
| Akkermansia sp. 54_46 | cov3 | 0 | Not detected | -0.050 | -0.399 | 0.300 | 0.780 |
| Akkermansia sp. CAG:344 | cov3 | 0 | Not detected | -0.084 | -0.436 | 0.267 | 0.639 |
| Akkermansia sp. KLE1605 | cov3 | 0 | Not detected | -0.178 | -0.479 | 0.122 | 0.245 |
| Akkermansia sp. KLE1797 | cov3 | 0 | Not detected | -0.172 | -0.473 | 0.129 | 0.264 |
| Akkermansia sp. KLE1798 | cov3 | 0 | Not detected | -0.166 | -0.467 | 0.135 | 0.279 |
| Akkermansia sp. Phil8 | cov3 | 0 | Not detected | 0.028 | -0.318 | 0.374 | 0.876 |
| Akkermansia sp. UNK.MG | cov3 | 0 | Not detected | -0.035 | -0.381 | 0.312 | 0.844 |
| Alistipes finegoldii | cov3 | 0 | Not detected | -0.132 | -0.270 | 0.007 | 0.062 |
| Alistipes ihumii | cov3 | 0 | Not detected | -0.037 | -0.272 | 0.197 | 0.755 |
| Alistipes indistinctus | cov3 | 0 | Not detected | -0.069 | -0.391 | 0.253 | 0.675 |
| Alistipes inops | cov3 | 0 | Not detected | 0.019 | -0.232 | 0.271 | 0.879 |
| Alistipes obesi | cov3 | 0 | Not detected | -0.155 | -0.306 | -0.005 | 0.044 |
| Alistipes putredinis | cov3 | 0 | Not detected | -0.157 | -0.352 | 0.039 | 0.116 |
| Alistipes senegalensis | cov3 | 0 | Not detected | -0.028 | -0.152 | 0.096 | 0.657 |
| Alistipes shahii | cov3 | 0 | Not detected | -0.052 | -0.189 | 0.084 | 0.452 |
| Alistipes sp. CAG:435 | cov3 | 0 | Not detected | 0.133 | -0.186 | 0.451 | 0.414 |
| Alistipes sp. CAG:514 | cov3 | 0 | Not detected | 0.226 | -0.065 | 0.516 | 0.128 |
| Alistipes sp. Zagget8 | cov3 | 0 | Not detected | -0.056 | -0.258 | 0.146 | 0.588 |
| Alistipes timonensis | cov3 | 0 | Not detected | -0.053 | -0.264 | 0.157 | 0.620 |
| Alkaliflexus imshenetskii | cov3 | 0 | Not detected | -0.005 | -0.157 | 0.146 | 0.947 |
| Allisonella histaminiforma | cov3 | 0 | Not detected | -0.095 | -0.400 | 0.210 | 0.543 |
| Allobaculum stercoricanis | cov3 | 0 | Not detected | 0.028 | -0.045 | 0.101 | 0.455 |
| Allofustis seminis | cov3 | 0 | Not detected | 0.148 | -0.025 | 0.320 | 0.093 |

| Alloprevotella rava | cov3 | 0 | Not detected | 0.008 | -0.126 | 0.142 | 0.906 |
| --- | --- | --- | --- | --- | --- | --- | --- |
| Alloprevotella tannerae | cov3 | 0 | Not detected | -0.061 | -0.156 | 0.035 | 0.214 |
| Alloscardovia criceti | cov3 | 0 | Not detected | -0.057 | -0.226 | 0.113 | 0.514 |
| Alloscardovia omnicolens | cov3 | 0 | Not detected | 0.012 | -0.168 | 0.192 | 0.897 |
| Amphibacillus xylanus | cov3 | 0 | Not detected | 0.003 | -0.175 | 0.182 | 0.969 |
| Anaerobium acetethylicum | cov3 | 0 | Not detected | 0.012 | -0.061 | 0.084 | 0.756 |
| Anaerococcus hydrogenali | cov3 | 0 | Not detected | -0.058 | -0.190 | 0.075 | 0.393 |
| Anaerococcus lactolyticus | cov3 | 0 | Not detected | -0.105 | -0.290 | 0.079 | 0.264 |
| Anaerococcus obesiensis | cov3 | 0 | Not detected | -0.117 | -0.271 | 0.037 | 0.136 |
| Anaerocolumna aminovale | cov3 | 0 | Not detected | -0.029 | -0.183 | 0.126 | 0.714 |
| Anaerocolumna xylanovor | cov3 | 0 | Not detected | -0.111 | -0.252 | 0.029 | 0.119 |
| Anaerofustis stercorihomin | cov3 | 0 | Not detected | -0.070 | -0.297 | 0.157 | 0.544 |
| Anaeroglobus geminatus | cov3 | 0 | Not detected | 0.010 | -0.174 | 0.194 | 0.918 |
| Anaeromassilibacillus sene | cov3 | 0 | Not detected | 0.041 | -0.087 | 0.168 | 0.532 |
| Anaerophaga thermohalop | cov3 | 0 | Not detected | -0.022 | -0.139 | 0.096 | 0.719 |
| Anaerosalibacter massilien | cov3 | 0 | Not detected | 0.090 | -0.097 | 0.277 | 0.345 |
| Anaerosporobacter mobilis | cov3 | 0 | Not detected | -0.027 | -0.109 | 0.056 | 0.527 |
| Anaerosporomusa subterra | cov3 | 0 | Not detected | 0.003 | -0.128 | 0.134 | 0.961 |
| Anaerostipes caccae | cov3 | 0 | Not detected | -0.029 | -0.104 | 0.047 | 0.453 |
| Anaerostipes hadrus | cov3 | 0 | Not detected | -0.104 | -0.233 | 0.025 | 0.114 |
| Anaerostipes sp. CAG:276 | cov3 | 0 | Not detected | -0.314 | -0.578 | -0.049 | 0.020 |
| Anaerotruncus colihomini | cov3 | 0 | Not detected | -0.036 | -0.119 | 0.047 | 0.399 |
| Anaerotruncus sp. CAG:39 | cov3 | 630 | Not detected | 0.254 | 0.019 | 0.489 | 0.035 |
| Anaerotruncus sp. CAG:52 | cov3 | 0 | Not detected | 0.083 | -0.106 | 0.273 | 0.390 |
| Anaerotruncus sp. G3(201 | cov3 | 0 | Not detected | -0.033 | -0.097 | 0.030 | 0.307 |
| Anaerovorax odorimutans | cov3 | 0 | Not detected | 0.044 | -0.103 | 0.191 | 0.555 |
| Angelakisella massiliensis | cov3 | 0 | Not detected | -0.021 | -0.092 | 0.050 | 0.556 |
| Aphanizomenon flos-aqua | cov3 | 0 | Not detected | 0.078 | -0.056 | 0.212 | 0.253 |
| Aquabacterium parvum | cov3 | 0 | Not detected | 0.145 | -0.014 | 0.305 | 0.073 |
| Arcanobacterium haemoly | cov3 | 0 | Not detected | -0.155 | -0.315 | 0.004 | 0.056 |
| Asaccharospora irregularis | cov3 | 0 | Not detected | 0.135 | -0.042 | 0.313 | 0.136 |
| Atlantibacter hermannii | cov3 | 0 | Not detected | 0.081 | -0.151 | 0.312 | 0.494 |
| Atopobium deltae | cov3 | 0 | Not detected | 0.079 | -0.094 | 0.252 | 0.373 |
| Atopobium minutum | cov3 | 0 | Not detected | 0.000 | -0.175 | 0.176 | 0.996 |
| Atopobium parvulum | cov3 | 0 | Not detected | 0.064 | -0.130 | 0.258 | 0.518 |
| Atopobium rimae | cov3 | 0 | Not detected | -0.012 | -0.175 | 0.150 | 0.883 |
| Atopobium sp. BS2 | cov3 | 0 | Not detected | -0.014 | -0.203 | 0.175 | 0.886 |
| Atopobium sp. HMSC064 | cov3 | 0 | Not detected | 0.021 | -0.120 | 0.161 | 0.774 |
| Atopobium sp. ICM42b | cov3 | 0 | Not detected | 0.027 | -0.164 | 0.218 | 0.781 |
| Atopobium vaginae | cov3 | 0 | Not detected | 0.124 | -0.056 | 0.304 | 0.177 |
| Atopostipes suicloacalis | cov3 | 0 | Not detected | 0.172 | 0.002 | 0.342 | 0.048 |
| Avibacterium paragallinar | cov3 | 0 | Not detected | 0.046 | -0.111 | 0.203 | 0.568 |
| Azospirillum sp. 47_25 | cov3 | 0 | Not detected | -0.131 | -0.464 | 0.201 | 0.439 |
| Azospirillum sp. CAG:260 | cov3 | 0 | Not detected | -0.098 | -0.425 | 0.229 | 0.558 |

| Bacillus acidiproducens | cov3 | 0 | Not detected | -0.001 | -0.192 | 0.189 | 0.988 |
| --- | --- | --- | --- | --- | --- | --- | --- |
| Bacillus andreraoultii | cov3 | 0 | Not detected | -0.009 | -0.197 | 0.180 | 0.927 |
| Bacillus azotoformans | cov3 | 0 | Not detected | 0.018 | -0.153 | 0.189 | 0.839 |
| Bacillus bataviensis | cov3 | 0 | Not detected | -0.047 | -0.212 | 0.118 | 0.579 |
| Bacillus coahuilensis | cov3 | 0 | Not detected | 0.027 | -0.165 | 0.219 | 0.780 |
| Bacillus rubiinfantis | cov3 | 0 | Not detected | -0.071 | -0.240 | 0.097 | 0.407 |
| Bacillus sp. CAG:988 | cov3 | 0 | Not detected | -0.013 | -0.175 | 0.148 | 0.872 |
| Bacillus thermotolerans | cov3 | 0 | Not detected | 0.006 | -0.178 | 0.189 | 0.953 |
| bacterium F082 | cov3 | 0 | Not detected | 0.036 | -0.065 | 0.138 | 0.483 |
| bacterium LF-3 | cov3 | 0 | Not detected | 0.003 | -0.151 | 0.157 | 0.971 |
| bacterium MS4 | cov3 | 0 | Not detected | 0.083 | -0.048 | 0.214 | 0.215 |
| bacterium OL-1 | cov3 | 0 | Not detected | -0.011 | -0.257 | 0.234 | 0.927 |
| bacterium P201 | cov3 | 0 | Not detected | 0.174 | 0.017 | 0.332 | 0.030 |
| bacterium P3 | cov3 | 0 | Not detected | 0.220 | 0.037 | 0.403 | 0.019 |
| Bacteroidales bacterium 4 | cov3 | 0 | Not detected | -0.166 | -0.347 | 0.015 | 0.073 |
| Bacteroidales bacterium 5 | cov3 | 0 | Not detected | -0.049 | -0.141 | 0.042 | 0.292 |
| Bacteroidales bacterium B | cov3 | 0 | Not detected | -0.093 | -0.237 | 0.051 | 0.206 |
| Bacteroidales bacterium B | cov3 | 0 | Not detected | -0.040 | -0.187 | 0.108 | 0.599 |
| Bacteroidales bacterium B | cov3 | 0 | Not detected | -0.086 | -0.276 | 0.104 | 0.376 |
| Bacteroidales bacterium C | cov3 | 0 | Not detected | 0.116 | -0.043 | 0.275 | 0.153 |
| Bacteroidales bacterium H | cov3 | 0 | Not detected | 0.010 | -0.131 | 0.150 | 0.893 |
| Bacteroidales bacterium K | cov3 | 0 | Not detected | -0.002 | -0.163 | 0.159 | 0.980 |
| Bacteroidales bacterium K | cov3 | 0 | Not detected | -0.180 | -0.339 | -0.021 | 0.027 |
| Bacteroidales bacterium K | cov3 | 0 | Not detected | 0.013 | -0.173 | 0.199 | 0.888 |
| Bacteroides barnesiae | cov3 | 0 | Not detected | -0.103 | -0.193 | -0.012 | 0.027 |
| Bacteroides caccae CAG:2 | cov3 | 0 | Not detected | -0.119 | -0.307 | 0.069 | 0.217 |
| Bacteroides cellulosilyticu | cov3 | 0 | Not detected | -0.077 | -0.239 | 0.085 | 0.349 |
| Bacteroides coprocola | cov3 | 4 | Not detected | -0.179 | -0.305 | -0.053 | 0.005 |
| Bacteroides coprophilus | cov3 | 0 | Not detected | -0.106 | -0.195 | -0.016 | 0.021 |
| Bacteroides coprosuis | cov3 | 0 | Not detected | 0.074 | -0.063 | 0.212 | 0.289 |
| Bacteroides dorei CAG:22 | cov3 | 0 | Not detected | -0.113 | -0.262 | 0.036 | 0.138 |
| Bacteroides faecichinchilla | cov3 | 0 | Not detected | -0.124 | -0.216 | -0.032 | 0.008 |
| Bacteroides faecis CAG:32 | cov3 | 0 | Not detected | -0.171 | -0.354 | 0.012 | 0.067 |
| Bacteroides fluxus | cov3 | 0 | Not detected | -0.083 | -0.178 | 0.012 | 0.088 |
| Bacteroides fragilis | cov3 | 0 | Not detected | -0.091 | -0.220 | 0.037 | 0.164 |
| Bacteroides fragilis CAG:4 | cov3 | 0 | Not detected | 0.019 | -0.188 | 0.226 | 0.858 |
| Bacteroides graminisolven | cov3 | 0 | Not detected | -0.100 | -0.300 | 0.100 | 0.328 |
| Bacteroides neonati | cov3 | 82 | Not detected | -0.119 | -0.194 | -0.044 | 0.002 |
| Bacteroides oleiciplenus | cov3 | 0 | Not detected | -0.135 | -0.243 | -0.026 | 0.015 |
| Bacteroides ovatus | cov3 | 0 | Not detected | -0.106 | -0.231 | 0.019 | 0.095 |
| Bacteroides ovatus CAG:2 | cov3 | 0 | Not detected | -0.135 | -0.271 | 0.001 | 0.053 |
| Bacteroides paurosaccharo | cov3 | 0 | Not detected | -0.130 | -0.259 | -0.001 | 0.049 |
| Bacteroides pectinophilus | cov3 | 0 | Not detected | 0.071 | -0.050 | 0.193 | 0.249 |
| Bacteroides plebeius | cov3 | 0 | Not detected | -0.162 | -0.294 | -0.029 | 0.017 |

| Bacteroides propionicifaci | cov3 | 0 | Not detected | -0.104 | -0.277 | 0.068 | 0.236 |
| --- | --- | --- | --- | --- | --- | --- | --- |
| Bacteroides pyogenes | cov3 | 4 | Not detected | -0.137 | -0.244 | -0.030 | 0.012 |
| Bacteroides reticulotermiti | cov3 | 0 | Not detected | -0.097 | -0.211 | 0.016 | 0.094 |
| Bacteroides salanitronis | cov3 | 0 | Not detected | -0.106 | -0.199 | -0.013 | 0.026 |
| Bacteroides sp. 41_26 | cov3 | 0 | Not detected | -0.088 | -0.236 | 0.059 | 0.241 |
| Bacteroides sp. 43_46 | cov3 | 0 | Not detected | -0.081 | -0.209 | 0.048 | 0.219 |
| Bacteroides sp. CAG:1060 | cov3 | 0 | Not detected | 0.045 | -0.187 | 0.278 | 0.703 |
| Bacteroides sp. CAG:1076 | cov3 | 0 | Not detected | -0.081 | -0.233 | 0.071 | 0.297 |
| Bacteroides sp. CAG:530 | cov3 | 24 | Not detected | 0.139 | 0.002 | 0.276 | 0.047 |
| Bacteroides sp. CAG:545 | cov3 | 0 | Not detected | 0.057 | -0.244 | 0.358 | 0.709 |
| Bacteroides sp. CAG:714 | cov3 | 0 | Not detected | -0.176 | -0.389 | 0.036 | 0.104 |
| Bacteroides sp. CAG:770 | cov3 | 0 | Not detected | 0.140 | -0.025 | 0.306 | 0.096 |
| Bacteroides sp. D2 | cov3 | 0 | Not detected | -0.150 | -0.293 | -0.008 | 0.039 |
| Bacteroides sp. Ga6A1 | cov3 | 0 | Not detected | 0.104 | -0.104 | 0.313 | 0.328 |
| Bacteroides sp. Ga6A2 | cov3 | 0 | Not detected | 0.049 | -0.158 | 0.257 | 0.641 |
| Bacteroides thetaiotaomicr | cov3 | 0 | Not detected | -0.060 | -0.153 | 0.034 | 0.211 |
| Bacteroides thetaiotaomicr | cov3 | 0 | Not detected | -0.090 | -0.238 | 0.058 | 0.232 |
| Bacteroides uniformis | cov3 | 0 | Not detected | -0.109 | -0.243 | 0.025 | 0.113 |
| Bacteroides vulgatus | cov3 | 0 | Not detected | -0.087 | -0.211 | 0.037 | 0.171 |
| Bacteroides vulgatus CAG | cov3 | 0 | Not detected | -0.115 | -0.265 | 0.035 | 0.133 |
| Bacteroidetes bacterium O | cov3 | 0 | Not detected | -0.065 | -0.239 | 0.110 | 0.467 |
| Bacteroidetes oral taxon 2 | cov3 | 0 | Not detected | 0.034 | -0.090 | 0.159 | 0.588 |
| Bariatricus massiliensis | cov3 | 0 | Not detected | -0.045 | -0.110 | 0.021 | 0.182 |
| Barnesiella intestinihomin | cov3 | 0 | Not detected | -0.032 | -0.196 | 0.132 | 0.702 |
| Barnesiella viscericola | cov3 | 0 | Not detected | -0.099 | -0.177 | -0.021 | 0.013 |
| Bavariicoccus seileri | cov3 | 0 | Not detected | 0.036 | -0.121 | 0.194 | 0.651 |
| Beduini massiliensis | cov3 | 0 | Not detected | 0.012 | -0.091 | 0.115 | 0.817 |
| Bibersteinia trehalosi | cov3 | 0 | Not detected | 0.104 | -0.082 | 0.290 | 0.274 |
| Bifidobacterium actinocol | cov3 | 0 | Not detected | 0.212 | 0.033 | 0.391 | 0.020 |
| Bifidobacterium adolescen | cov3 | 0 | Not detected | 0.160 | -0.114 | 0.434 | 0.253 |
| Bifidobacterium aesculapii | cov3 | 0 | Not detected | 0.137 | -0.007 | 0.280 | 0.062 |
| Bifidobacterium animalis | cov3 | 0 | Not detected | -0.068 | -0.261 | 0.126 | 0.494 |
| Bifidobacterium asteroides | cov3 | 0 | Not detected | 0.238 | 0.051 | 0.425 | 0.013 |
| Bifidobacterium biavatii | cov3 | 429 | Not detected | 0.093 | 0.020 | 0.165 | 0.012 |
| Bifidobacterium bifidum | cov3 | 0 | Not detected | 0.265 | 0.003 | 0.526 | 0.048 |
| Bifidobacterium bohemicu | cov3 | 108 | Not detected | 0.301 | 0.130 | 0.471 | 0.001 |
| Bifidobacterium bombi | cov3 | 53 | Not detected | 0.299 | 0.130 | 0.468 | 0.001 |
| Bifidobacterium breve | cov3 | 8 | Not detected | 0.274 | 0.019 | 0.529 | 0.035 |
| Bifidobacterium callitricho | cov3 | 495 | Not detected | 0.238 | 0.012 | 0.464 | 0.039 |
| Bifidobacterium choerinu | cov3 | 0 | Not detected | 0.205 | 0.007 | 0.403 | 0.042 |
| Bifidobacterium corynefor | cov3 | 46 | Not detected | 0.271 | 0.091 | 0.451 | 0.003 |
| Bifidobacterium crudilacti | cov3 | 0 | Not detected | 0.163 | 0.013 | 0.313 | 0.033 |
| Bifidobacterium cuniculi | cov3 | 0 | Not detected | 0.231 | 0.040 | 0.423 | 0.018 |
| Bifidobacterium gallicum | cov3 | 38 | Not detected | 0.223 | 0.030 | 0.416 | 0.024 |

| Bifidobacterium gallinaru | cov3 | 0 | Not detected | 0.212 | -0.018 | 0.442 | 0.071 |
| --- | --- | --- | --- | --- | --- | --- | --- |
| Bifidobacterium longum | cov3 | 0 | Not detected | 0.156 | -0.096 | 0.409 | 0.225 |
| Bifidobacterium magnum | cov3 | 0 | Not detected | 0.096 | -0.025 | 0.218 | 0.121 |
| Bifidobacterium minimum | cov3 | 0 | Not detected | 0.241 | 0.005 | 0.477 | 0.046 |
| Bifidobacterium mongolie | cov3 | 24 | Not detected | 0.267 | 0.084 | 0.449 | 0.004 |
| Bifidobacterium pseudolo | cov3 | 0 | Not detected | 0.193 | -0.012 | 0.398 | 0.065 |
| Bifidobacterium psychraer | cov3 | 0 | Not detected | 0.158 | 0.019 | 0.297 | 0.026 |
| Bifidobacterium reuteri | cov3 | 89 | Not detected | 0.157 | -0.025 | 0.340 | 0.091 |
| Bifidobacterium saguini | cov3 | 0 | Not detected | 0.253 | 0.008 | 0.498 | 0.043 |
| Bifidobacterium scardovii | cov3 | 800 | 0.6 | 0.307 | 0.064 | 0.550 | 0.013 |
| Bifidobacterium stellenbos | cov3 | 265 | Not detected | 0.086 | 0.007 | 0.166 | 0.033 |
| Bifidobacterium subtile | cov3 | 670 | Not detected | 0.316 | 0.100 | 0.531 | 0.004 |
| Bifidobacterium thermoph | cov3 | 0 | Not detected | 0.099 | -0.020 | 0.218 | 0.102 |
| Bifidobacterium tsurumien | cov3 | 0 | Not detected | 0.224 | 0.030 | 0.417 | 0.024 |
| Bilophila wadsworthia | cov3 | 0 | Not detected | -0.032 | -0.325 | 0.262 | 0.832 |
| Bittarella massiliensis | cov3 | 0 | Not detected | -0.025 | -0.095 | 0.045 | 0.484 |
| Blautia hansenii | cov3 | 0 | Not detected | -0.090 | -0.172 | -0.008 | 0.031 |
| Blautia hydrogenotrophica | cov3 | 0 | Not detected | -0.054 | -0.129 | 0.020 | 0.150 |
| Blautia hydrogenotrophica | cov3 | 0 | Not detected | -0.097 | -0.329 | 0.135 | 0.414 |
| Blautia obeum | cov3 | 0 | Not detected | -0.029 | -0.106 | 0.048 | 0.457 |
| Blautia producta | cov3 | 213 | Not detected | -0.157 | -0.262 | -0.052 | 0.003 |
| Blautia schinkii | cov3 | 0 | Not detected | -0.018 | -0.074 | 0.039 | 0.539 |
| Blautia sp. CAG:237 | cov3 | 0 | Not detected | -0.050 | -0.212 | 0.111 | 0.542 |
| Blautia sp. CAG:37 | cov3 | 0 | Not detected | -0.026 | -0.178 | 0.127 | 0.741 |
| Blautia sp. CAG:37_48_5 | cov3 | 0 | Not detected | -0.043 | -0.159 | 0.073 | 0.466 |
| Blautia sp. KLE 1732 | cov3 | 0 | Not detected | 0.023 | -0.071 | 0.117 | 0.637 |
| Brenneria goodwinii | cov3 | 0 | Not detected | 0.040 | -0.107 | 0.187 | 0.595 |
| Bulleidia extructa | cov3 | 0 | Not detected | 0.045 | -0.138 | 0.228 | 0.629 |
| Burkholderiales bacterium | cov3 | 0 | Not detected | -0.058 | -0.227 | 0.111 | 0.501 |
| Buttiauxella ferragutiae | cov3 | 0 | Not detected | 0.033 | -0.132 | 0.199 | 0.693 |
| Butyricicoccus desmolans | cov3 | 0 | Not detected | -0.057 | -0.119 | 0.004 | 0.069 |
| Butyricicoccus pullicaecor | cov3 | 0 | Not detected | 0.047 | -0.032 | 0.125 | 0.244 |
| Butyricicoccus sp. K4410. | cov3 | 0 | Not detected | -0.035 | -0.141 | 0.071 | 0.512 |
| Butyricimonas sp. Marseil | cov3 | 0 | Not detected | -0.058 | -0.215 | 0.098 | 0.464 |
| Butyricimonas synergistica | cov3 | 0 | Not detected | 0.069 | -0.121 | 0.259 | 0.476 |
| Butyricimonas virosa | cov3 | 0 | Not detected | 0.040 | -0.119 | 0.199 | 0.619 |
| Butyrivibrio crossotus | cov3 | 0 | Not detected | -0.017 | -0.146 | 0.112 | 0.797 |
| Butyrivibrio crossotus CA | cov3 | 0 | Not detected | 0.027 | -0.169 | 0.224 | 0.784 |
| Butyrivibrio fibrisolvens | cov3 | 0 | Not detected | 0.026 | -0.137 | 0.188 | 0.757 |
| Butyrivibrio hungatei | cov3 | 1 | Not detected | -0.132 | -0.262 | -0.002 | 0.047 |
| Butyrivibrio proteoclasticu | cov3 | 0 | Not detected | -0.020 | -0.111 | 0.071 | 0.661 |
| Butyrivibrio sp. AD3002 | cov3 | 0 | Not detected | 0.045 | -0.065 | 0.156 | 0.421 |
| Butyrivibrio sp. AE2005 | cov3 | 0 | Not detected | 0.018 | -0.105 | 0.141 | 0.778 |
| Butyrivibrio sp. AE3003 | cov3 | 0 | Not detected | 0.004 | -0.086 | 0.095 | 0.924 |

| Butyrivibrio sp. AE3004 | cov3 | 0 | Not detected | -0.050 | -0.137 | 0.038 | 0.265 |
| --- | --- | --- | --- | --- | --- | --- | --- |
| Butyrivibrio sp. AE3009 | cov3 | 0 | Not detected | 0.050 | -0.093 | 0.192 | 0.496 |
| Butyrivibrio sp. FCS006 | cov3 | 0 | Not detected | -0.031 | -0.176 | 0.114 | 0.673 |
| Butyrivibrio sp. INlla14 | cov3 | 0 | Not detected | -0.013 | -0.130 | 0.104 | 0.828 |
| Butyrivibrio sp. INlla16 | cov3 | 0 | Not detected | -0.084 | -0.210 | 0.043 | 0.196 |
| Butyrivibrio sp. INlla21 | cov3 | 0 | Not detected | -0.078 | -0.204 | 0.048 | 0.227 |
| Butyrivibrio sp. LB2008 | cov3 | 0 | Not detected | 0.083 | -0.059 | 0.225 | 0.254 |
| Butyrivibrio sp. M55 | cov3 | 0 | Not detected | -0.062 | -0.197 | 0.073 | 0.367 |
| Butyrivibrio sp. NC2002 | cov3 | 0 | Not detected | 0.011 | -0.131 | 0.153 | 0.878 |
| Butyrivibrio sp. NC2007 | cov3 | 0 | Not detected | -0.015 | -0.145 | 0.115 | 0.819 |
| Butyrivibrio sp. NC3005 | cov3 | 0 | Not detected | -0.021 | -0.204 | 0.163 | 0.823 |
| Butyrivibrio sp. ob235 | cov3 | 0 | Not detected | 0.047 | -0.108 | 0.202 | 0.549 |
| Butyrivibrio sp. VCD2006 | cov3 | 0 | Not detected | -0.024 | -0.156 | 0.109 | 0.725 |
| Butyrivibrio sp. WCD200 | cov3 | 0 | Not detected | -0.072 | -0.203 | 0.059 | 0.284 |
| Butyrivibrio sp. WCD300 | cov3 | 0 | Not detected | -0.096 | -0.222 | 0.029 | 0.133 |
| Campylobacter coli | cov3 | 0 | Not detected | -0.097 | -0.196 | 0.002 | 0.055 |
| Campylobacter hominis | cov3 | 0 | Not detected | -0.065 | -0.269 | 0.139 | 0.531 |
| Candidatus Azobacteroide | cov3 | 0 | Not detected | -0.025 | -0.156 | 0.106 | 0.713 |
| Candidatus Gastranaeroph | cov3 | 0 | Not detected | -0.125 | -0.299 | 0.049 | 0.160 |
| Candidatus Melainabacteri | cov3 | 0 | Not detected | -0.086 | -0.209 | 0.037 | 0.173 |
| Candidatus Melainabacteri | cov3 | 0 | Not detected | 0.003 | -0.243 | 0.248 | 0.984 |
| Candidatus Saccharibacter | cov3 | 0 | Not detected | 0.122 | -0.030 | 0.274 | 0.117 |
| Candidatus Soleaferrea ma | cov3 | 0 | Not detected | 0.105 | -0.004 | 0.213 | 0.059 |
| Candidatus Stoquefichus s | cov3 | 0 | Not detected | -0.046 | -0.145 | 0.053 | 0.365 |
| Capnocytophaga granulosa | cov3 | 0 | Not detected | 0.033 | -0.125 | 0.192 | 0.679 |
| Capnocytophaga sp. oral t | cov3 | 0 | Not detected | -0.025 | -0.246 | 0.197 | 0.828 |
| Capnocytophaga sp. oral t | cov3 | 0 | Not detected | -0.128 | -0.321 | 0.064 | 0.192 |
| Capnocytophaga sputigena | cov3 | 36 | Not detected | -0.159 | -0.284 | -0.033 | 0.013 |
| Carnobacterium gallinarum | cov3 | 0 | Not detected | 0.079 | -0.105 | 0.264 | 0.398 |
| Catabacter hongkongensis | cov3 | 0 | Not detected | 0.068 | -0.081 | 0.217 | 0.372 |
| Catenibacterium mitsuokai | cov3 | 0 | Not detected | 0.108 | -0.142 | 0.359 | 0.397 |
| Catenibacterium sp. CAG: | cov3 | 0 | Not detected | 0.097 | -0.213 | 0.406 | 0.540 |
| Catonella morbi | cov3 | 0 | Not detected | -0.032 | -0.119 | 0.054 | 0.460 |
| Cedecea neteri | cov3 | 0 | Not detected | 0.065 | -0.141 | 0.271 | 0.536 |
| Cellulomonas carbonis | cov3 | 0 | Not detected | 0.020 | -0.158 | 0.199 | 0.823 |
| Cellulomonas flavigena | cov3 | 0 | Not detected | 0.050 | -0.146 | 0.247 | 0.617 |
| Cellulosilyticum lentocellu | cov3 | 0 | Not detected | 0.080 | -0.063 | 0.223 | 0.273 |
| Cellulosilyticum ruminicol | cov3 | 0 | Not detected | -0.039 | -0.192 | 0.114 | 0.619 |
| Chlamydia psittaci | cov3 | 0 | Not detected | 0.136 | -0.030 | 0.301 | 0.108 |
| Christensenella sp. AF73- | cov3 | 0 | Not detected | -0.003 | -0.195 | 0.189 | 0.975 |
| Christensenellaceae bacter | cov3 | 0 | Not detected | 0.117 | -0.017 | 0.251 | 0.088 |
| Christensenellaceae bacter | cov3 | 0 | Not detected | 0.097 | -0.101 | 0.294 | 0.338 |
| Christensenellaceae bacter | cov3 | 0 | Not detected | 0.121 | -0.081 | 0.324 | 0.240 |
| Chryseobacterium gallinar | cov3 | 0 | Not detected | -0.143 | -0.374 | 0.089 | 0.226 |

| Chryseobacterium greenla | cov3 | 0 | Not detected | -0.254 | -0.473 | -0.035 | 0.023 |
| --- | --- | --- | --- | --- | --- | --- | --- |
| Citrobacter amalonaticus | cov3 | 0 | Not detected | 0.071 | -0.190 | 0.331 | 0.596 |
| Citrobacter freundii | cov3 | 0 | Not detected | 0.055 | -0.215 | 0.324 | 0.692 |
| Cloacibacillus evryensis | cov3 | 0 | Not detected | 0.057 | -0.193 | 0.308 | 0.653 |
| Cloacibacillus porcorum | cov3 | 0 | Not detected | -0.018 | -0.229 | 0.192 | 0.865 |
| Cloacibacterium normanen | cov3 | 0 | Not detected | -0.111 | -0.321 | 0.099 | 0.299 |
| Clostridia bacterium UC5. | cov3 | 0 | Not detected | -0.142 | -0.378 | 0.094 | 0.239 |
| Clostridia bacterium UC5. | cov3 | 0 | Not detected | -0.017 | -0.091 | 0.058 | 0.661 |
| Clostridia bacterium UC5. | cov3 | 0 | Not detected | 0.001 | -0.070 | 0.073 | 0.975 |
| Clostridiales bacterium 1_ | cov3 | 0 | Not detected | -0.038 | -0.120 | 0.044 | 0.368 |
| Clostridiales bacterium 59 | cov3 | 0 | Not detected | 0.072 | -0.051 | 0.195 | 0.253 |
| Clostridiales bacterium DT | cov3 | 0 | Not detected | 0.049 | -0.105 | 0.204 | 0.532 |
| Clostridiales bacterium DT | cov3 | 0 | Not detected | -0.036 | -0.180 | 0.107 | 0.622 |
| Clostridiales bacterium DT | cov3 | 0 | Not detected | -0.019 | -0.150 | 0.112 | 0.776 |
| Clostridiales bacterium Fir | cov3 | 0 | Not detected | 0.010 | -0.169 | 0.188 | 0.917 |
| Clostridiales bacterium Fir | cov3 | 0 | Not detected | -0.055 | -0.256 | 0.145 | 0.588 |
| Clostridiales bacterium NK | cov3 | 0 | Not detected | 0.062 | -0.103 | 0.227 | 0.461 |
| Clostridiales bacterium S5 | cov3 | 0 | Not detected | 0.009 | -0.065 | 0.083 | 0.809 |
| Clostridiales bacterium SI | cov3 | 0 | Not detected | 0.167 | -0.001 | 0.335 | 0.052 |
| Clostridiales bacterium VE | cov3 | 0 | Not detected | -0.025 | -0.110 | 0.059 | 0.560 |
| Clostridioides difficile | cov3 | 0 | Not detected | -0.066 | -0.135 | 0.003 | 0.063 |
| Clostridioides mangenotii | cov3 | 0 | Not detected | 0.125 | -0.036 | 0.287 | 0.129 |
| Clostridium baratii | cov3 | 0 | Not detected | 0.157 | -0.031 | 0.345 | 0.101 |
| Clostridium beijerinckii | cov3 | 0 | Not detected | 0.130 | -0.042 | 0.301 | 0.139 |
| Clostridium bolteae CAG: | cov3 | 621 | Not detected | -0.206 | -0.338 | -0.073 | 0.002 |
| Clostridium botulinum | cov3 | 0 | Not detected | 0.193 | 0.009 | 0.376 | 0.040 |
| Clostridium butyricum | cov3 | 0 | Not detected | 0.068 | -0.120 | 0.257 | 0.477 |
| Clostridium cadaveris | cov3 | 0 | Not detected | -0.082 | -0.236 | 0.072 | 0.297 |
| Clostridium celatum | cov3 | 0 | Not detected | 0.246 | -0.023 | 0.515 | 0.073 |
| Clostridium cellulovorans | cov3 | 0 | Not detected | -0.034 | -0.141 | 0.073 | 0.530 |
| Clostridium colicanis | cov3 | 0 | Not detected | 0.026 | -0.119 | 0.171 | 0.725 |
| Clostridium disporicum | cov3 | 0 | Not detected | 0.138 | 0.004 | 0.271 | 0.043 |
| Clostridium ihumii | cov3 | 73 | Not detected | 0.294 | 0.135 | 0.453 | 0.000 |
| Clostridium intestinale | cov3 | 0 | Not detected | 0.073 | -0.058 | 0.204 | 0.272 |
| Clostridium kluyveri | cov3 | 0 | Not detected | 0.059 | -0.123 | 0.241 | 0.524 |
| Clostridium leptum CAG:2 | cov3 | 0 | Not detected | -0.018 | -0.224 | 0.189 | 0.868 |
| Clostridium lundense | cov3 | 0 | Not detected | 0.073 | -0.108 | 0.253 | 0.430 |
| Clostridium neonatale | cov3 | 0 | Not detected | 0.134 | -0.025 | 0.293 | 0.098 |
| Clostridium nexile CAG:3 | cov3 | 0 | Not detected | -0.097 | -0.232 | 0.037 | 0.157 |
| Clostridium paraputrificum | cov3 | 0 | Not detected | -0.079 | -0.303 | 0.146 | 0.492 |
| Clostridium perfringens | cov3 | 0 | Not detected | 0.218 | -0.060 | 0.495 | 0.124 |
| Clostridium phoceensis | cov3 | 0 | Not detected | -0.131 | -0.238 | -0.023 | 0.017 |
| Clostridium polynesiense | cov3 | 0 | Not detected | 0.115 | -0.063 | 0.294 | 0.204 |
| Clostridium saccharobutyl | cov3 | 0 | Not detected | 0.066 | -0.095 | 0.226 | 0.424 |

| Clostridium saccharoperbu | cov3 | 0 | Not detected | 0.157 | 0.002 | 0.313 | 0.048 |
| --- | --- | --- | --- | --- | --- | --- | --- |
| Clostridium sartagoforme | cov3 | 0 | Not detected | 0.139 | -0.052 | 0.330 | 0.154 |
| Clostridium sp. 44_14 | cov3 | 0 | Not detected | 0.004 | -0.158 | 0.166 | 0.957 |
| Clostridium sp. Ade.TY | cov3 | 0 | Not detected | 0.116 | -0.047 | 0.280 | 0.164 |
| Clostridium sp. ASF356 | cov3 | 1 | Not detected | -0.128 | -0.314 | 0.057 | 0.175 |
| Clostridium sp. CAG:1193 | cov3 | 0 | Not detected | 0.220 | 0.021 | 0.418 | 0.030 |
| Clostridium sp. CAG:122 | cov3 | 0 | Not detected | -0.090 | -0.301 | 0.121 | 0.405 |
| Clostridium sp. CAG:1223 | cov3 | 0 | Not detected | -0.063 | -0.223 | 0.096 | 0.438 |
| Clostridium sp. CAG:127 | cov3 | 0 | Not detected | 0.077 | -0.163 | 0.317 | 0.529 |
| Clostridium sp. CAG:138 | cov3 | 0 | Not detected | 0.176 | -0.141 | 0.493 | 0.277 |
| Clostridium sp. CAG:167 | cov3 | 0 | Not detected | 0.076 | -0.203 | 0.355 | 0.592 |
| Clostridium sp. CAG:226 | cov3 | 0 | Not detected | 0.244 | 0.002 | 0.487 | 0.048 |
| Clostridium sp. CAG:230 | cov3 | 0 | Not detected | 0.053 | -0.118 | 0.225 | 0.540 |
| Clostridium sp. CAG:242 | cov3 | 0 | Not detected | -0.050 | -0.293 | 0.193 | 0.687 |
| Clostridium sp. CAG:245 | cov3 | 0 | Not detected | 0.086 | -0.272 | 0.444 | 0.639 |
| Clostridium sp. CAG:245_ | cov3 | 0 | Not detected | -0.042 | -0.190 | 0.106 | 0.576 |
| Clostridium sp. CAG:264 | cov3 | 0 | Not detected | 0.033 | -0.202 | 0.269 | 0.781 |
| Clostridium sp. CAG:277 | cov3 | 0 | Not detected | -0.033 | -0.199 | 0.134 | 0.703 |
| Clostridium sp. CAG:288 | cov3 | 0 | Not detected | 0.041 | -0.209 | 0.291 | 0.746 |
| Clostridium sp. CAG:299 | cov3 | 0 | Not detected | -0.148 | -0.363 | 0.067 | 0.178 |
| Clostridium sp. CAG:307_ | cov3 | 0 | Not detected | -0.021 | -0.160 | 0.118 | 0.764 |
| Clostridium sp. CAG:349 | cov3 | 0 | Not detected | -0.039 | -0.368 | 0.289 | 0.815 |
| Clostridium sp. CAG:352 | cov3 | 0 | Not detected | 0.122 | -0.083 | 0.327 | 0.242 |
| Clostridium sp. CAG:413 | cov3 | 0 | Not detected | 0.203 | -0.041 | 0.446 | 0.103 |
| Clostridium sp. CAG:448 | cov3 | 0 | Not detected | 0.096 | -0.170 | 0.363 | 0.479 |
| Clostridium sp. CAG:451 | cov3 | 0 | Not detected | 0.218 | -0.052 | 0.487 | 0.114 |
| Clostridium sp. CAG:465 | cov3 | 0 | Not detected | -0.012 | -0.266 | 0.243 | 0.929 |
| Clostridium sp. CAG:505 | cov3 | 0 | Not detected | -0.072 | -0.323 | 0.180 | 0.577 |
| Clostridium sp. CAG:568 | cov3 | 0 | Not detected | -0.118 | -0.383 | 0.146 | 0.380 |
| Clostridium sp. CAG:58 | cov3 | 0 | Not detected | -0.074 | -0.251 | 0.103 | 0.413 |
| Clostridium sp. CAG:590 | cov3 | 0 | Not detected | 0.052 | -0.078 | 0.181 | 0.432 |
| Clostridium sp. CAG:594 | cov3 | 0 | Not detected | 0.286 | -0.004 | 0.576 | 0.053 |
| Clostridium sp. CAG:62_4 | cov3 | 0 | Not detected | 0.063 | -0.221 | 0.348 | 0.663 |
| Clostridium sp. CAG:628 | cov3 | 0 | Not detected | 0.162 | -0.113 | 0.437 | 0.249 |
| Clostridium sp. CAG:632 | cov3 | 0 | Not detected | 0.141 | -0.028 | 0.310 | 0.103 |
| Clostridium sp. CAG:678 | cov3 | 0 | Not detected | 0.032 | -0.167 | 0.231 | 0.755 |
| Clostridium sp. CAG:762 | cov3 | 0 | Not detected | 0.161 | -0.069 | 0.391 | 0.170 |
| Clostridium sp. CAG:768 | cov3 | 0 | Not detected | -0.018 | -0.169 | 0.134 | 0.821 |
| Clostridium sp. CAG:780 | cov3 | 0 | Not detected | 0.106 | -0.185 | 0.397 | 0.475 |
| Clostridium sp. CAG:793 | cov3 | 0 | Not detected | 0.141 | -0.094 | 0.377 | 0.240 |
| Clostridium sp. CAG:81 | cov3 | 0 | Not detected | 0.017 | -0.112 | 0.147 | 0.794 |
| Clostridium sp. CAG:91 | cov3 | 0 | Not detected | -0.028 | -0.237 | 0.181 | 0.791 |
| Clostridium sp. CAG:964 | cov3 | 0 | Not detected | 0.017 | -0.114 | 0.148 | 0.795 |
| Clostridium sp. CL-6 | cov3 | 0 | Not detected | 0.091 | -0.047 | 0.229 | 0.197 |

| Clostridium sp. HGF2 | cov3 | 0 | Not detected | -0.007 | -0.080 | 0.066 | 0.847 |
| --- | --- | --- | --- | --- | --- | --- | --- |
| Clostridium sp. K4410.M | cov3 | 0 | Not detected | -0.057 | -0.151 | 0.036 | 0.229 |
| Clostridium sp. KLE 1755 | cov3 | 0 | Not detected | -0.113 | -0.226 | 0.001 | 0.051 |
| Clostridium sp. KNHs205 | cov3 | 1 | Not detected | -0.063 | -0.126 | 0.001 | 0.052 |
| Clostridium sp. KNHs209 | cov3 | 0 | Not detected | -0.040 | -0.102 | 0.022 | 0.210 |
| Clostridium sp. KNHs214 | cov3 | 285 | Not detected | 0.134 | 0.028 | 0.241 | 0.013 |
| Clostridium sp. L2-50 | cov3 | 0 | Not detected | -0.021 | -0.115 | 0.073 | 0.664 |
| Clostridium sp. LF2 | cov3 | 0 | Not detected | 0.116 | -0.012 | 0.244 | 0.075 |
| Clostridium sp. M62/1 | cov3 | 0 | Not detected | -0.021 | -0.082 | 0.040 | 0.503 |
| Clostridium sp. ND2 | cov3 | 0 | Not detected | 0.058 | -0.096 | 0.211 | 0.462 |
| Clostridium sp. SY8519 | cov3 | 0 | Not detected | 0.022 | -0.047 | 0.090 | 0.534 |
| Clostridium sulfidigenes | cov3 | 0 | Not detected | 0.110 | -0.016 | 0.235 | 0.087 |
| Collinsella aerofaciens | cov3 | 0 | Not detected | 0.201 | 0.017 | 0.386 | 0.033 |
| Collinsella intestinalis | cov3 | 0 | Not detected | -0.019 | -0.229 | 0.192 | 0.863 |
| Collinsella sp. CAG:166 | cov3 | 435 | Not detected | 0.224 | 0.019 | 0.430 | 0.032 |
| Collinsella sp. MS5 | cov3 | 333 | Not detected | 0.180 | 0.039 | 0.321 | 0.013 |
| Collinsella stercoris | cov3 | 0 | Not detected | 0.020 | -0.168 | 0.209 | 0.832 |
| Collinsella tanakaei | cov3 | 0 | Not detected | -0.066 | -0.203 | 0.071 | 0.342 |
| Comamonas kerstersii | cov3 | 0 | Not detected | 0.130 | -0.090 | 0.351 | 0.247 |
| Coprobacillus sp. CAG:60 | cov3 | 0 | Not detected | 0.185 | -0.072 | 0.442 | 0.159 |
| Coprobacillus sp. D6 | cov3 | 0 | Not detected | -0.050 | -0.143 | 0.043 | 0.292 |
| Coprobacter secundus | cov3 | 0 | Not detected | -0.073 | -0.158 | 0.013 | 0.095 |
| Coprococcus comes | cov3 | 0 | Not detected | 0.045 | -0.030 | 0.120 | 0.241 |
| Coprococcus eutactus | cov3 | 0 | Not detected | 0.050 | -0.057 | 0.156 | 0.360 |
| Coprococcus eutactus CA | cov3 | 0 | Not detected | 0.185 | -0.051 | 0.421 | 0.124 |
| Coprococcus sp. 43_8 | cov3 | 0 | Not detected | 0.120 | -0.028 | 0.269 | 0.112 |
| Coprococcus sp. CAG:131 | cov3 | 0 | Not detected | 0.085 | -0.142 | 0.311 | 0.463 |
| Coprococcus sp. CAG:131 | cov3 | 0 | Not detected | 0.158 | -0.070 | 0.386 | 0.174 |
| Coprococcus sp. HPP0048 | cov3 | 0 | Not detected | -0.021 | -0.138 | 0.095 | 0.718 |
| Coriobacteriaceae bacteriu | cov3 | 0 | Not detected | -0.036 | -0.185 | 0.113 | 0.633 |
| Coriobacteriaceae bacteriu | cov3 | 0 | Not detected | 0.072 | -0.122 | 0.267 | 0.466 |
| Coriobacteriales bacterium | cov3 | 0 | Not detected | 0.062 | -0.110 | 0.234 | 0.480 |
| Coriobacterium glomerans | cov3 | 0 | Not detected | 0.150 | -0.009 | 0.309 | 0.065 |
| Corynebacterium amycolat | cov3 | 0 | Not detected | -0.021 | -0.150 | 0.108 | 0.747 |
| Corynebacterium durum | cov3 | 0 | Not detected | -0.090 | -0.282 | 0.101 | 0.355 |
| Corynebacterium glucuron | cov3 | 0 | Not detected | 0.052 | -0.099 | 0.203 | 0.499 |
| Corynebacterium singulare | cov3 | 0 | Not detected | 0.045 | -0.097 | 0.186 | 0.537 |
| Corynebacterium urealytic | cov3 | 0 | Not detected | 0.017 | -0.140 | 0.174 | 0.833 |
| Criibacterium bergeronii | cov3 | 0 | Not detected | 0.013 | -0.093 | 0.119 | 0.807 |
| Crocosphaera watsonii | cov3 | 0 | Not detected | 0.109 | -0.039 | 0.258 | 0.149 |
| Cronobacter sakazakii | cov3 | 0 | Not detected | 0.084 | -0.173 | 0.342 | 0.520 |
| Cryptobacterium sp. CAG: | cov3 | 0 | Not detected | -0.316 | -0.584 | -0.048 | 0.021 |
| Culturomica massiliensis | cov3 | 520 | Not detected | -0.163 | -0.260 | -0.067 | 0.001 |
| Cutibacterium acnes | cov3 | 0 | Not detected | 0.085 | -0.092 | 0.262 | 0.345 |

| Defluviitalea phaphyphila | cov3 | 0 | Not detected | -0.092 | -0.220 | 0.036 | 0.159 |
| --- | --- | --- | --- | --- | --- | --- | --- |
| Dehalobacter restrictus | cov3 | 0 | Not detected | 0.059 | -0.074 | 0.192 | 0.384 |
| Dendrosporobacter quercic | cov3 | 0 | Not detected | -0.046 | -0.176 | 0.085 | 0.494 |
| Denitrobacterium detoxific | cov3 | 0 | Not detected | 0.015 | -0.117 | 0.148 | 0.819 |
| Desnuesiella massiliensis | cov3 | 0 | Not detected | 0.057 | -0.080 | 0.194 | 0.415 |
| Desulfovibrio desulfurican | cov3 | 0 | Not detected | 0.202 | -0.035 | 0.439 | 0.096 |
| Desulfovibrio fairfieldensi | cov3 | 0 | Not detected | -0.126 | -0.392 | 0.141 | 0.356 |
| Desulfovibrio piger | cov3 | 0 | Not detected | -0.184 | -0.515 | 0.146 | 0.275 |
| Desulfovibrio vulgaris | cov3 | 0 | Not detected | 0.086 | -0.069 | 0.242 | 0.278 |
| Devosia insulae | cov3 | 0 | Not detected | 0.029 | -0.080 | 0.138 | 0.606 |
| Dialister invisus | cov3 | 0 | Not detected | 0.019 | -0.335 | 0.372 | 0.916 |
| Dialister invisus CAG:218 | cov3 | 0 | Not detected | -0.037 | -0.398 | 0.324 | 0.840 |
| Dialister micraerophilus | cov3 | 0 | Not detected | 0.096 | -0.122 | 0.314 | 0.390 |
| Dialister sp. CAG:357 | cov3 | 0 | Not detected | -0.115 | -0.459 | 0.229 | 0.512 |
| Dialister sp. CAG:486 | cov3 | 0 | Not detected | 0.253 | -0.059 | 0.565 | 0.112 |
| Dialister sp. CAG:588 | cov3 | 0 | Not detected | 0.002 | -0.187 | 0.190 | 0.987 |
| Dialister succinatiphilus | cov3 | 0 | Not detected | 0.022 | -0.175 | 0.219 | 0.828 |
| Dickeya dadantii | cov3 | 0 | Not detected | 0.081 | -0.069 | 0.231 | 0.290 |
| Dielma fastidiosa | cov3 | 0 | Not detected | -0.127 | -0.300 | 0.046 | 0.151 |
| Dorea formicigenerans | cov3 | 0 | Not detected | 0.002 | -0.067 | 0.071 | 0.950 |
| Dorea longicatena | cov3 | 0 | Not detected | 0.041 | -0.057 | 0.140 | 0.410 |
| Dorea sp. 42_8 | cov3 | 0 | Not detected | 0.043 | -0.072 | 0.158 | 0.463 |
| Dorea sp. 5-2 | cov3 | 0 | Not detected | -0.027 | -0.080 | 0.025 | 0.301 |
| Dorea sp. CAG:105 | cov3 | 0 | Not detected | 0.141 | -0.011 | 0.292 | 0.069 |
| Draconibacterium oriental | cov3 | 0 | Not detected | 0.035 | -0.091 | 0.161 | 0.588 |
| Drancourtella massiliensis | cov3 | 1 | Not detected | 0.031 | -0.095 | 0.158 | 0.629 |
| Dubosiella newyorkensis | cov3 | 0 | Not detected | 0.119 | -0.019 | 0.257 | 0.092 |
| Dysgonomonas gadei | cov3 | 0 | Not detected | -0.064 | -0.181 | 0.054 | 0.287 |
| Dysgonomonas mossii | cov3 | 0 | Not detected | -0.065 | -0.205 | 0.076 | 0.367 |
| Dysgonomonas sp. BGC7 | cov3 | 0 | Not detected | -0.112 | -0.283 | 0.058 | 0.198 |
| Dysgonomonas sp. HGC4 | cov3 | 0 | Not detected | 0.037 | -0.111 | 0.185 | 0.622 |
| Edwardsiella ictaluri | cov3 | 0 | Not detected | -0.014 | -0.167 | 0.140 | 0.862 |
| Eggerthella lenta | cov3 | 0 | Not detected | -0.102 | -0.286 | 0.082 | 0.279 |
| Eggerthella sp. 51_9 | cov3 | 0 | Not detected | 0.098 | -0.106 | 0.303 | 0.346 |
| Eggerthella sp. CAG:209 | cov3 | 0 | Not detected | 0.137 | -0.177 | 0.451 | 0.394 |
| Eggerthella sp. CAG:368 | cov3 | 0 | Not detected | 0.089 | -0.137 | 0.315 | 0.439 |
| Eggerthella sp. YY7918 | cov3 | 0 | Not detected | -0.109 | -0.261 | 0.043 | 0.161 |
| Eggerthellaceae bacterium | cov3 | 0 | Not detected | 0.025 | -0.069 | 0.118 | 0.605 |
| Eggerthia catenaformis | cov3 | 0 | Not detected | 0.069 | -0.123 | 0.261 | 0.483 |
| Eisenbergiella tayi | cov3 | 0 | Not detected | -0.024 | -0.174 | 0.127 | 0.757 |
| Elizabethkingia anophelis | cov3 | 0 | Not detected | -0.094 | -0.294 | 0.106 | 0.355 |
| Emergencia timonensis | cov3 | 0 | Not detected | -0.001 | -0.070 | 0.068 | 0.981 |
| Enhygromyxa salina | cov3 | 0 | Not detected | -0.013 | -0.181 | 0.155 | 0.882 |
| Enorma massiliensis | cov3 | 0 | Not detected | -0.014 | -0.112 | 0.084 | 0.781 |

| Enorma timonensis | cov3 | 0 | Not detected | 0.056 | -0.070 | 0.181 | 0.384 |
| --- | --- | --- | --- | --- | --- | --- | --- |
| Enterobacter cloacae | cov3 | 0 | Not detected | -0.008 | -0.278 | 0.262 | 0.951 |
| Enterobacter lignolyticus | cov3 | 0 | Not detected | 0.075 | -0.170 | 0.321 | 0.548 |
| Enterobacter sp. 638 | cov3 | 0 | Not detected | 0.047 | -0.179 | 0.274 | 0.682 |
| Enterobacteriaceae bacteri | cov3 | 0 | Not detected | 0.004 | -0.160 | 0.168 | 0.965 |
| Enterococcus avium | cov3 | 0 | Not detected | -0.018 | -0.181 | 0.144 | 0.826 |
| Enterococcus cecorum | cov3 | 0 | Not detected | -0.079 | -0.219 | 0.062 | 0.273 |
| Enterococcus columbae | cov3 | 0 | Not detected | -0.034 | -0.215 | 0.147 | 0.712 |
| Enterococcus durans | cov3 | 0 | Not detected | 0.191 | -0.016 | 0.399 | 0.071 |
| Enterococcus faecalis | cov3 | 0 | Not detected | -0.042 | -0.199 | 0.115 | 0.599 |
| Enterococcus faecium | cov3 | 0 | Not detected | 0.129 | -0.034 | 0.293 | 0.120 |
| Enterococcus gallinarum | cov3 | 0 | Not detected | 0.002 | -0.160 | 0.163 | 0.985 |
| Enterococcus gilvus | cov3 | 0 | Not detected | 0.058 | -0.095 | 0.212 | 0.457 |
| Enterococcus italicus | cov3 | 0 | Not detected | 0.077 | -0.110 | 0.265 | 0.419 |
| Enterococcus massiliensis | cov3 | 0 | Not detected | 0.132 | -0.050 | 0.315 | 0.155 |
| Enterococcus sp. HMSC06 | cov3 | 0 | Not detected | -0.017 | -0.206 | 0.171 | 0.855 |
| Enterorhabdus caecimuris | cov3 | 0 | Not detected | -0.014 | -0.202 | 0.174 | 0.887 |
| Enterorhabdus mucosicola | cov3 | 0 | Not detected | 0.033 | -0.129 | 0.195 | 0.691 |
| Epulopiscium sp. 'N.t. mor | cov3 | 0 | Not detected | -0.021 | -0.144 | 0.102 | 0.738 |
| Erwinia billingiae | cov3 | 0 | Not detected | 0.048 | -0.101 | 0.197 | 0.525 |
| Erwinia oleae | cov3 | 0 | Not detected | 0.001 | -0.190 | 0.193 | 0.989 |
| Erysipelatoclostridium ram | cov3 | 0 | Not detected | -0.244 | -0.469 | -0.020 | 0.033 |
| Erysipelotrichaceae bacter | cov3 | 0 | Not detected | -0.038 | -0.132 | 0.056 | 0.430 |
| Erysipelotrichaceae bacter | cov3 | 0 | Not detected | -0.017 | -0.093 | 0.058 | 0.653 |
| Erysipelotrichaceae bacter | cov3 | 0 | Not detected | -0.025 | -0.193 | 0.143 | 0.770 |
| Erysipelotrichaceae bacter | cov3 | 0 | Not detected | -0.022 | -0.110 | 0.065 | 0.616 |
| Escherichia coli | cov3 | 0 | Not detected | 0.105 | -0.140 | 0.350 | 0.400 |
| Escherichia vulneris | cov3 | 0 | Not detected | 0.025 | -0.192 | 0.242 | 0.819 |
| Ethanoligenens harbinense | cov3 | 0 | Not detected | 0.073 | -0.093 | 0.239 | 0.388 |
| Eubacterium dolichum CA | cov3 | 0 | Not detected | 0.033 | -0.218 | 0.284 | 0.796 |
| Eubacterium limosum | cov3 | 0 | Not detected | 0.005 | -0.106 | 0.117 | 0.926 |
| Eubacterium plexicaudatu | cov3 | 0 | Not detected | -0.032 | -0.093 | 0.029 | 0.307 |
| Eubacterium pyruvativora | cov3 | 0 | Not detected | -0.011 | -0.092 | 0.069 | 0.781 |
| Eubacterium ramulus | cov3 | 0 | Not detected | -0.023 | -0.094 | 0.048 | 0.521 |
| Eubacterium sp. 14-2 | cov3 | 0 | Not detected | -0.042 | -0.106 | 0.022 | 0.196 |
| Eubacterium sp. 3_1_31 | cov3 | 0 | Not detected | -0.054 | -0.141 | 0.033 | 0.224 |
| Eubacterium sp. 45_250 | cov3 | 0 | Not detected | 0.175 | -0.073 | 0.422 | 0.166 |
| Eubacterium sp. AB3007 | cov3 | 0 | Not detected | 0.221 | -0.010 | 0.451 | 0.061 |
| Eubacterium sp. CAG:115 | cov3 | 0 | Not detected | 0.168 | -0.198 | 0.533 | 0.368 |
| Eubacterium sp. CAG:146 | cov3 | 0 | Not detected | 0.032 | -0.124 | 0.188 | 0.689 |
| Eubacterium sp. CAG:180 | cov3 | 0 | Not detected | 0.139 | -0.172 | 0.451 | 0.381 |
| Eubacterium sp. CAG:192 | cov3 | 0 | Not detected | -0.019 | -0.252 | 0.213 | 0.870 |
| Eubacterium sp. CAG:202 | cov3 | 0 | Not detected | 0.031 | -0.202 | 0.265 | 0.792 |
| Eubacterium sp. CAG:248 | cov3 | 0 | Not detected | -0.040 | -0.236 | 0.155 | 0.686 |

| Eubacterium sp. CAG:251 | cov3 | 0 | Not detected | 0.099 | -0.189 | 0.386 | 0.501 |
| --- | --- | --- | --- | --- | --- | --- | --- |
| Eubacterium sp. CAG:252 | cov3 | 0 | Not detected | 0.018 | -0.158 | 0.195 | 0.841 |
| Eubacterium sp. CAG:581 | cov3 | 0 | Not detected | -0.029 | -0.279 | 0.221 | 0.821 |
| Eubacterium sp. CAG:603 | cov3 | 0 | Not detected | 0.050 | -0.077 | 0.177 | 0.437 |
| Eubacterium sp. CAG:786 | cov3 | 0 | Not detected | 0.067 | -0.220 | 0.355 | 0.645 |
| Eubacterium sp. CAG:841 | cov3 | 0 | Not detected | 0.118 | -0.154 | 0.391 | 0.393 |
| Eubacterium sp. ER2 | cov3 | 0 | Not detected | -0.042 | -0.103 | 0.018 | 0.170 |
| Eubacterium ventriosum | cov3 | 0 | Not detected | -0.032 | -0.122 | 0.058 | 0.487 |
| Eubacterium xylanophilum | cov3 | 0 | Not detected | -0.034 | -0.178 | 0.110 | 0.644 |
| Facklamia hominis | cov3 | 0 | Not detected | 0.143 | -0.032 | 0.318 | 0.110 |
| Facklamia ignava | cov3 | 0 | Not detected | 0.116 | -0.055 | 0.288 | 0.184 |
| Facklamia languida | cov3 | 0 | Not detected | -0.107 | -0.269 | 0.055 | 0.197 |
| Faecalibacterium prausnitz | cov3 | 0 | Not detected | 0.036 | -0.078 | 0.151 | 0.534 |
| Faecalibacterium sp. CAG | cov3 | 0 | Not detected | 0.007 | -0.233 | 0.246 | 0.956 |
| Faecalibacterium sp. CAG | cov3 | 0 | Not detected | 0.143 | -0.029 | 0.315 | 0.104 |
| Faecalibaculum rodentium | cov3 | 0 | Not detected | 0.222 | 0.037 | 0.408 | 0.019 |
| Faecalicoccus pleomorphu | cov3 | 0 | Not detected | 0.012 | -0.086 | 0.110 | 0.813 |
| Faecalitalea cylindroides | cov3 | 0 | Not detected | -0.021 | -0.080 | 0.039 | 0.499 |
| Fenollaria massiliensis | cov3 | 0 | Not detected | 0.187 | -0.020 | 0.393 | 0.077 |
| Fermentimonas caenicola | cov3 | 0 | Not detected | 0.015 | -0.161 | 0.191 | 0.870 |
| Fibrobacter sp. UWB12 | cov3 | 0 | Not detected | -0.070 | -0.170 | 0.031 | 0.174 |
| Fibrobacter sp. UWCM | cov3 | 0 | Not detected | 0.052 | -0.084 | 0.188 | 0.452 |
| Fibrobacter sp. UWOV1 | cov3 | 0 | Not detected | -0.009 | -0.109 | 0.092 | 0.863 |
| Fibrobacterales bacterium | cov3 | 0 | Not detected | -0.045 | -0.156 | 0.065 | 0.419 |
| Fibrobacteria bacterium A | cov3 | 0 | Not detected | -0.036 | -0.147 | 0.075 | 0.526 |
| Filifactor alocis | cov3 | 35 | Not detected | -0.204 | -0.388 | -0.021 | 0.029 |
| Finegoldia magna | cov3 | 0 | Not detected | -0.053 | -0.244 | 0.138 | 0.586 |
| Firmicutes bacterium ASF | cov3 | 0 | Not detected | 0.014 | -0.052 | 0.080 | 0.670 |
| Firmicutes bacterium CAG | cov3 | 0 | Not detected | -0.141 | -0.381 | 0.099 | 0.249 |
| Firmicutes bacterium CAG | cov3 | 0 | Not detected | 0.174 | -0.110 | 0.458 | 0.230 |
| Firmicutes bacterium CAG | cov3 | 0 | Not detected | 0.109 | -0.106 | 0.324 | 0.319 |
| Firmicutes bacterium CAG | cov3 | 0 | Not detected | 0.060 | -0.197 | 0.316 | 0.648 |
| Firmicutes bacterium CAG | cov3 | 0 | Not detected | 0.225 | -0.079 | 0.528 | 0.148 |
| Firmicutes bacterium CAG | cov3 | 0 | Not detected | 0.205 | -0.075 | 0.485 | 0.151 |
| Firmicutes bacterium CAG | cov3 | 0 | Not detected | 0.112 | -0.087 | 0.312 | 0.269 |
| Firmicutes bacterium CAG | cov3 | 0 | Not detected | 0.115 | -0.124 | 0.353 | 0.346 |
| Firmicutes bacterium CAG | cov3 | 0 | Not detected | 0.044 | -0.123 | 0.212 | 0.604 |
| Firmicutes bacterium CAG | cov3 | 0 | Not detected | 0.235 | -0.035 | 0.504 | 0.088 |
| Firmicutes bacterium CAG | cov3 | 0 | Not detected | -0.081 | -0.279 | 0.117 | 0.422 |
| Firmicutes bacterium CAG | cov3 | 0 | Not detected | 0.117 | -0.137 | 0.371 | 0.367 |
| Firmicutes bacterium CAG | cov3 | 0 | Not detected | 0.117 | -0.072 | 0.306 | 0.225 |
| Firmicutes bacterium CAG | cov3 | 0 | Not detected | 0.056 | -0.104 | 0.216 | 0.491 |
| Firmicutes bacterium CAG | cov3 | 0 | Not detected | 0.053 | -0.280 | 0.386 | 0.754 |
| Firmicutes bacterium CAG | cov3 | 0 | Not detected | -0.071 | -0.286 | 0.144 | 0.517 |

| Firmicutes bacterium CAG | cov3 | 0 | Not detected | -0.043 | -0.219 | 0.132 | 0.629 |
| --- | --- | --- | --- | --- | --- | --- | --- |
| Firmicutes bacterium CAG | cov3 | 0 | Not detected | 0.085 | -0.154 | 0.324 | 0.486 |
| Firmicutes bacterium CAG | cov3 | 14 | Not detected | 0.256 | 0.067 | 0.446 | 0.008 |
| Firmicutes bacterium CAG | cov3 | 0 | Not detected | -0.058 | -0.288 | 0.172 | 0.620 |
| Firmicutes bacterium CAG | cov3 | 0 | Not detected | 0.099 | -0.125 | 0.323 | 0.385 |
| Firmicutes bacterium CAG | cov3 | 0 | Not detected | 0.098 | -0.091 | 0.288 | 0.308 |
| Firmicutes bacterium CAG | cov3 | 753 | 0.6 | 0.207 | 0.085 | 0.330 | 0.001 |
| Firmicutes bacterium CAG | cov3 | 0 | Not detected | 0.041 | -0.166 | 0.248 | 0.698 |
| Firmicutes bacterium CAG | cov3 | 0 | Not detected | -0.025 | -0.174 | 0.124 | 0.744 |
| Firmicutes bacterium CAG | cov3 | 0 | Not detected | 0.054 | -0.173 | 0.281 | 0.642 |
| Firmicutes bacterium CAG | cov3 | 0 | Not detected | 0.131 | -0.104 | 0.365 | 0.275 |
| Firmicutes bacterium M10 | cov3 | 0 | Not detected | 0.035 | -0.153 | 0.224 | 0.715 |
| Firmicutes bacterium ZOR | cov3 | 0 | Not detected | 0.029 | -0.170 | 0.227 | 0.778 |
| Flavonifractor plautii | cov3 | 0 | Not detected | -0.162 | -0.307 | -0.017 | 0.029 |
| Fournierella massiliensis | cov3 | 0 | Not detected | 0.003 | -0.068 | 0.074 | 0.936 |
| Franconibacter pulveris | cov3 | 0 | Not detected | -0.036 | -0.240 | 0.167 | 0.726 |
| Fretibacterium fastidiosum | cov3 | 0 | Not detected | 0.044 | -0.090 | 0.177 | 0.523 |
| Fusicatenibacter sacchariv | cov3 | 0 | Not detected | -0.033 | -0.116 | 0.051 | 0.441 |
| Fusobacterium mortiferum | cov3 | 0 | Not detected | 0.019 | -0.241 | 0.279 | 0.884 |
| Fusobacterium necrophoru | cov3 | 0 | Not detected | -0.005 | -0.181 | 0.171 | 0.957 |
| Fusobacterium nucleatum | cov3 | 0 | Not detected | 0.064 | -0.187 | 0.314 | 0.618 |
| Fusobacterium periodontic | cov3 | 0 | Not detected | -0.080 | -0.297 | 0.136 | 0.467 |
| Gabonia massiliensis | cov3 | 0 | Not detected | -0.146 | -0.283 | -0.009 | 0.037 |
| Gabonibacter massiliensis | cov3 | 0 | Not detected | -0.164 | -0.328 | 0.001 | 0.052 |
| Gallibacterium anatis | cov3 | 0 | Not detected | -0.025 | -0.153 | 0.103 | 0.705 |
| Gardnerella vaginalis | cov3 | 0 | Not detected | 0.093 | -0.101 | 0.287 | 0.347 |
| Gemella haemolysans | cov3 | 0 | Not detected | 0.037 | -0.147 | 0.220 | 0.696 |
| Gemella sanguinis | cov3 | 0 | Not detected | -0.213 | -0.436 | 0.010 | 0.062 |
| Gemmata obscuriglobus | cov3 | 0 | Not detected | -0.047 | -0.204 | 0.111 | 0.562 |
| Geofilum rubicundum | cov3 | 0 | Not detected | -0.127 | -0.278 | 0.023 | 0.098 |
| Gilliamella apicola | cov3 | 0 | Not detected | 0.078 | -0.072 | 0.228 | 0.308 |
| Globicatella sanguinis | cov3 | 0 | Not detected | 0.127 | -0.046 | 0.300 | 0.150 |
| Gordonibacter pamelaeae | cov3 | 0 | Not detected | -0.083 | -0.211 | 0.045 | 0.203 |
| Granulicatella adiacens | cov3 | 0 | Not detected | -0.048 | -0.263 | 0.168 | 0.665 |
| Granulicatella elegans | cov3 | 0 | Not detected | 0.137 | -0.044 | 0.317 | 0.139 |
| Haemophilus influenzae | cov3 | 0 | Not detected | 0.176 | -0.066 | 0.418 | 0.154 |
| Haemophilus parainfluenz | cov3 | 0 | Not detected | 0.117 | -0.175 | 0.409 | 0.432 |
| Haemophilus sputorum | cov3 | 0 | Not detected | 0.041 | -0.209 | 0.292 | 0.747 |
| Hafnia sp. HMSC23F03 | cov3 | 0 | Not detected | 0.118 | -0.066 | 0.301 | 0.208 |
| Hathewaya proteolytica | cov3 | 0 | Not detected | 0.202 | 0.026 | 0.378 | 0.025 |
| Heliobacterium sp. N15.M | cov3 | 0 | Not detected | -0.013 | -0.089 | 0.063 | 0.732 |
| Hespellia stercorisuis | cov3 | 0 | Not detected | 0.008 | -0.063 | 0.078 | 0.832 |
| Holdemanella biformis | cov3 | 0 | Not detected | 0.125 | -0.006 | 0.255 | 0.061 |
| Holdemania filiformis | cov3 | 0 | Not detected | -0.006 | -0.086 | 0.074 | 0.877 |

| Holdemania massiliensis | cov3 | 0 | Not detected | -0.027 | -0.133 | 0.079 | 0.614 |
| --- | --- | --- | --- | --- | --- | --- | --- |
| Holdemania sp. Marseille- | cov3 | 0 | Not detected | -0.063 | -0.158 | 0.031 | 0.189 |
| Hungatella hathewayi | cov3 | 0 | Not detected | -0.018 | -0.104 | 0.067 | 0.673 |
| Hydrogenoanaerobacteriu | cov3 | 0 | Not detected | 0.102 | -0.013 | 0.217 | 0.081 |
| Idiomarina zobellii | cov3 | 0 | Not detected | 0.144 | -0.073 | 0.360 | 0.193 |
| Ileibacterium valens | cov3 | 0 | Not detected | 0.180 | -0.013 | 0.373 | 0.067 |
| Intestinibacter bartlettii | cov3 | 0 | Not detected | 0.083 | -0.084 | 0.250 | 0.330 |
| Intestinimonas butyricipro | cov3 | 0 | Not detected | 0.010 | -0.092 | 0.113 | 0.844 |
| Intestinimonas massiliensi | cov3 | 0 | Not detected | -0.044 | -0.137 | 0.048 | 0.349 |
| Izhakiella sp. D4N98 | cov3 | 0 | Not detected | 0.084 | -0.076 | 0.244 | 0.304 |
| Jeotgalibaca sp. PTS2502 | cov3 | 0 | Not detected | 0.116 | -0.023 | 0.256 | 0.103 |
| Johnsonella ignava | cov3 | 0 | Not detected | 0.064 | -0.020 | 0.149 | 0.133 |
| Kallipyga massiliensis | cov3 | 0 | Not detected | -0.087 | -0.261 | 0.087 | 0.326 |
| Kandleria vitulina | cov3 | 0 | Not detected | 0.175 | -0.074 | 0.424 | 0.169 |
| Kiloniella litopenaei | cov3 | 0 | Not detected | 0.051 | -0.118 | 0.221 | 0.552 |
| Kingella kingae | cov3 | 0 | Not detected | 0.050 | -0.112 | 0.212 | 0.544 |
| Klebsiella oxytoca | cov3 | 0 | Not detected | 0.047 | -0.241 | 0.336 | 0.748 |
| Klebsiella pneumoniae | cov3 | 0 | Not detected | 0.082 | -0.211 | 0.374 | 0.585 |
| Klebsiella sp. RIT-PI-d | cov3 | 0 | Not detected | 0.054 | -0.163 | 0.271 | 0.624 |
| Kluyvera ascorbata | cov3 | 0 | Not detected | 0.076 | -0.192 | 0.344 | 0.578 |
| Kluyvera cryocrescens | cov3 | 0 | Not detected | 0.007 | -0.266 | 0.280 | 0.960 |
| Kluyvera intermedia | cov3 | 0 | Not detected | 0.033 | -0.221 | 0.287 | 0.801 |
| Kocuria sp. ZOR0020 | cov3 | 0 | Not detected | -0.088 | -0.233 | 0.057 | 0.234 |
| Kosakonia sacchari | cov3 | 0 | Not detected | 0.042 | -0.189 | 0.273 | 0.720 |
| Lachnoanaerobaculum sab | cov3 | 1 | Not detected | -0.086 | -0.175 | 0.003 | 0.060 |
| Lachnoanaerobaculum sp. | cov3 | 0 | Not detected | 0.029 | -0.053 | 0.111 | 0.495 |
| Lachnobacterium bovis | cov3 | 0 | Not detected | -0.041 | -0.124 | 0.042 | 0.331 |
| Lachnoclostridium phytofe | cov3 | 1 | Not detected | -0.042 | -0.107 | 0.023 | 0.201 |
| Lachnospira multipara | cov3 | 0 | Not detected | -0.127 | -0.245 | -0.008 | 0.036 |
| Lachnospiraceae bacterium | cov3 | 0 | Not detected | 0.008 | -0.045 | 0.061 | 0.765 |
| Lachnospiraceae bacterium | cov3 | 0 | Not detected | -0.066 | -0.148 | 0.016 | 0.117 |
| Lachnospiraceae bacterium | cov3 | 0 | Not detected | -0.008 | -0.063 | 0.048 | 0.791 |
| Lachnospiraceae bacterium | cov3 | 0 | Not detected | -0.017 | -0.081 | 0.047 | 0.610 |
| Lachnospiraceae bacterium | cov3 | 0 | Not detected | -0.028 | -0.090 | 0.034 | 0.374 |
| Lachnospiraceae bacterium | cov3 | 0 | Not detected | -0.025 | -0.092 | 0.041 | 0.457 |
| Lachnospiraceae bacterium | cov3 | 0 | Not detected | -0.008 | -0.066 | 0.051 | 0.802 |
| Lachnospiraceae bacterium | cov3 | 0 | Not detected | 0.109 | -0.073 | 0.292 | 0.241 |
| Lachnospiraceae bacterium | cov3 | 0 | Not detected | -0.026 | -0.102 | 0.049 | 0.491 |
| Lachnospiraceae bacterium | cov3 | 0 | Not detected | 0.029 | -0.089 | 0.147 | 0.627 |
| Lachnospiraceae bacterium | cov3 | 0 | Not detected | 0.043 | -0.084 | 0.171 | 0.506 |
| Lachnospiraceae bacterium | cov3 | 0 | Not detected | -0.005 | -0.087 | 0.078 | 0.911 |
| Lachnospiraceae bacterium | cov3 | 0 | Not detected | -0.036 | -0.154 | 0.083 | 0.555 |
| Lachnospiraceae bacterium | cov3 | 0 | Not detected | 0.041 | -0.112 | 0.193 | 0.601 |
| Lachnospiraceae bacterium | cov3 | 0 | Not detected | 0.155 | -0.021 | 0.330 | 0.084 |

| Lachnospiraceae bacterium | cov3 | 0 | Not detected | 0.016 | -0.100 | 0.132 | 0.788 |
| --- | --- | --- | --- | --- | --- | --- | --- |
| Lachnospiraceae bacterium | cov3 | 15 | Not detected | 0.072 | -0.012 | 0.155 | 0.092 |
| Lachnospiraceae bacterium | cov3 | 0 | Not detected | -0.053 | -0.203 | 0.097 | 0.487 |
| Lachnospiraceae bacterium | cov3 | 0 | Not detected | 0.083 | -0.096 | 0.262 | 0.365 |
| Lachnospiraceae bacterium | cov3 | 0 | Not detected | -0.056 | -0.210 | 0.098 | 0.476 |
| Lachnospiraceae bacterium | cov3 | 0 | Not detected | 0.017 | -0.156 | 0.189 | 0.848 |
| Lachnospiraceae bacterium | cov3 | 0 | Not detected | -0.002 | -0.064 | 0.060 | 0.943 |
| Lachnospiraceae bacterium | cov3 | 0 | Not detected | -0.036 | -0.161 | 0.089 | 0.576 |
| Lachnospiraceae bacterium | cov3 | 0 | Not detected | 0.064 | -0.084 | 0.213 | 0.396 |
| Lachnospiraceae bacterium | cov3 | 0 | Not detected | 0.041 | -0.125 | 0.207 | 0.629 |
| Lachnospiraceae bacterium | cov3 | 0 | Not detected | 0.046 | -0.108 | 0.199 | 0.559 |
| Lachnospiraceae bacterium | cov3 | 0 | Not detected | 0.018 | -0.139 | 0.176 | 0.819 |
| Lachnospiraceae bacterium | cov3 | 0 | Not detected | 0.037 | -0.140 | 0.215 | 0.680 |
| Lachnospiraceae bacterium | cov3 | 0 | Not detected | 0.071 | -0.088 | 0.231 | 0.381 |
| Lachnospiraceae bacterium | cov3 | 0 | Not detected | -0.046 | -0.213 | 0.122 | 0.593 |
| Lachnospiraceae bacterium | cov3 | 0 | Not detected | 0.001 | -0.066 | 0.067 | 0.981 |
| Lachnospiraceae bacterium | cov3 | 0 | Not detected | 0.002 | -0.126 | 0.129 | 0.977 |
| Lachnospiraceae bacterium | cov3 | 0 | Not detected | -0.042 | -0.127 | 0.042 | 0.329 |
| Lachnospiraceae bacterium | cov3 | 0 | Not detected | 0.000 | -0.088 | 0.088 | 0.993 |
| Lachnospiraceae bacterium | cov3 | 0 | Not detected | 0.115 | -0.039 | 0.270 | 0.143 |
| Lachnospiraceae bacterium | cov3 | 0 | Not detected | -0.030 | -0.146 | 0.085 | 0.607 |
| Lactobacillus acidophilus | cov3 | 0 | Not detected | -0.042 | -0.172 | 0.088 | 0.529 |
| Lactobacillus amylotrophi | cov3 | 0 | Not detected | -0.083 | -0.246 | 0.080 | 0.316 |
| Lactobacillus animalis | cov3 | 0 | Not detected | 0.044 | -0.119 | 0.207 | 0.597 |
| Lactobacillus antri | cov3 | 0 | Not detected | 0.233 | 0.053 | 0.413 | 0.011 |
| Lactobacillus aviarius | cov3 | 0 | Not detected | -0.003 | -0.093 | 0.086 | 0.942 |
| Lactobacillus casei | cov3 | 0 | Not detected | -0.100 | -0.327 | 0.128 | 0.390 |
| Lactobacillus curvatus | cov3 | 0 | Not detected | 0.058 | -0.120 | 0.235 | 0.524 |
| Lactobacillus delbrueckii | cov3 | 0 | Not detected | -0.043 | -0.246 | 0.159 | 0.674 |
| Lactobacillus equi | cov3 | 0 | Not detected | 0.084 | -0.093 | 0.261 | 0.353 |
| Lactobacillus fermentum | cov3 | 0 | Not detected | 0.079 | -0.178 | 0.337 | 0.546 |
| Lactobacillus gasseri | cov3 | 0 | Not detected | 0.282 | 0.021 | 0.543 | 0.034 |
| Lactobacillus helveticus | cov3 | 0 | Not detected | 0.150 | -0.042 | 0.342 | 0.127 |
| Lactobacillus ingluviei | cov3 | 0 | Not detected | 0.059 | -0.093 | 0.211 | 0.449 |
| Lactobacillus jensenii | cov3 | 0 | Not detected | 0.063 | -0.101 | 0.227 | 0.455 |
| Lactobacillus kefiranofacie | cov3 | 0 | Not detected | 0.073 | -0.076 | 0.222 | 0.337 |
| Lactobacillus manihotivor | cov3 | 0 | Not detected | 0.030 | -0.136 | 0.196 | 0.723 |
| Lactobacillus mucosae | cov3 | 0 | Not detected | -0.012 | -0.185 | 0.160 | 0.887 |
| Lactobacillus paracasei | cov3 | 0 | Not detected | -0.030 | -0.264 | 0.204 | 0.800 |
| Lactobacillus pasteurii | cov3 | 0 | Not detected | 0.025 | -0.116 | 0.166 | 0.724 |
| Lactobacillus plantarum | cov3 | 0 | Not detected | 0.054 | -0.142 | 0.250 | 0.592 |
| Lactobacillus reuteri | cov3 | 0 | Not detected | 0.072 | -0.127 | 0.271 | 0.480 |
| Lactobacillus rhamnosus | cov3 | 0 | Not detected | -0.045 | -0.256 | 0.166 | 0.677 |
| Lactobacillus ruminis | cov3 | 0 | Not detected | 0.062 | -0.110 | 0.234 | 0.479 |

| Lactobacillus ruminis CAG | cov3 | 0 | Not detected | 0.089 | -0.082 | 0.261 | 0.308 |
| --- | --- | --- | --- | --- | --- | --- | --- |
| Lactobacillus sakei | cov3 | 0 | Not detected | 0.112 | -0.064 | 0.287 | 0.214 |
| Lactobacillus salivarius | cov3 | 0 | Not detected | 0.171 | -0.079 | 0.422 | 0.180 |
| Lactobacillus vaginalis | cov3 | 0 | Not detected | 0.152 | -0.069 | 0.373 | 0.178 |
| Lactococcus garvieae | cov3 | 0 | Not detected | 0.021 | -0.157 | 0.198 | 0.819 |
| Lactococcus lactis | cov3 | 0 | Not detected | -0.067 | -0.338 | 0.203 | 0.626 |
| Lactococcus piscium | cov3 | 0 | Not detected | -0.153 | -0.336 | 0.031 | 0.103 |
| Lactonifactor longoviform | cov3 | 0 | Not detected | -0.003 | -0.058 | 0.052 | 0.914 |
| Lamprocystis purpurea | cov3 | 0 | Not detected | 0.056 | -0.164 | 0.275 | 0.620 |
| Lascolabacillus massiliens | cov3 | 0 | Not detected | 0.030 | -0.118 | 0.178 | 0.693 |
| Lautropia mirabilis | cov3 | 0 | Not detected | 0.191 | 0.008 | 0.374 | 0.041 |
| Lawsonia intracellularis | cov3 | 0 | Not detected | 0.004 | -0.174 | 0.183 | 0.961 |
| Leclercia adecarboxylata | cov3 | 0 | Not detected | 0.084 | -0.175 | 0.342 | 0.526 |
| Lelliottia amnigena | cov3 | 0 | Not detected | 0.061 | -0.219 | 0.340 | 0.670 |
| Lentimicrobium saccharop | cov3 | 0 | Not detected | -0.067 | -0.260 | 0.126 | 0.496 |
| Leuconostoc citreum | cov3 | 0 | Not detected | -0.076 | -0.238 | 0.087 | 0.361 |
| Leuconostoc lactis | cov3 | 0 | Not detected | -0.222 | -0.417 | -0.027 | 0.025 |
| Leuconostoc mesenteroide | cov3 | 0 | Not detected | -0.150 | -0.358 | 0.057 | 0.157 |
| Levyella massiliensis | cov3 | 0 | Not detected | -0.004 | -0.097 | 0.088 | 0.930 |
| Libanicoccus massiliensis | cov3 | 0 | Not detected | -0.030 | -0.194 | 0.134 | 0.720 |
| Luteimonas sp. FCS-9 | cov3 | 0 | Not detected | -0.013 | -0.151 | 0.124 | 0.848 |
| Lutispora thermophila | cov3 | 0 | Not detected | 0.061 | -0.099 | 0.221 | 0.455 |
| Macellibacteroides sp. HH | cov3 | 0 | Not detected | -0.101 | -0.226 | 0.023 | 0.111 |
| Macrococcus caseolyticus | cov3 | 0 | Not detected | -0.106 | -0.282 | 0.070 | 0.238 |
| Mageeibacillus indolicus | cov3 | 213 | Not detected | 0.202 | 0.074 | 0.331 | 0.002 |
| Mangrovibacter sp. MFB0 | cov3 | 0 | Not detected | 0.039 | -0.165 | 0.243 | 0.706 |
| Mannheimia haemolytica | cov3 | 0 | Not detected | 0.077 | -0.092 | 0.247 | 0.372 |
| Marvinbryantia formatexig | cov3 | 0 | Not detected | -0.003 | -0.085 | 0.079 | 0.942 |
| Massilioclostridium coli | cov3 | 0 | Not detected | -0.041 | -0.121 | 0.040 | 0.322 |
| Massiliomicrobiota timone | cov3 | 0 | Not detected | 0.086 | -0.098 | 0.270 | 0.360 |
| Mediterranea massiliensis | cov3 | 0 | Not detected | -0.001 | -0.105 | 0.104 | 0.991 |
| Megamonas funiformis | cov3 | 0 | Not detected | 0.082 | -0.238 | 0.401 | 0.616 |
| Megasphaera cerevisiae | cov3 | 0 | Not detected | -0.027 | -0.263 | 0.210 | 0.824 |
| Megasphaera elsdenii | cov3 | 0 | Not detected | 0.130 | -0.174 | 0.433 | 0.403 |
| Megasphaera genomosp. t | cov3 | 0 | Not detected | 0.030 | -0.030 | 0.089 | 0.326 |
| Megasphaera massiliensis | cov3 | 0 | Not detected | 0.163 | -0.118 | 0.445 | 0.255 |
| Megasphaera micronucifor | cov3 | 0 | Not detected | 0.204 | -0.011 | 0.419 | 0.063 |
| Megasphaera sp. BV3C16 | cov3 | 0 | Not detected | 0.135 | -0.046 | 0.317 | 0.144 |
| Megasphaera sp. UPII 135 | cov3 | 0 | Not detected | 0.010 | -0.051 | 0.071 | 0.751 |
| Merdibacter massiliensis | cov3 | 5 | Not detected | -0.060 | -0.128 | 0.008 | 0.085 |
| Methanobrevibacter smithi | cov3 | 0 | Not detected | 0.261 | -0.103 | 0.624 | 0.160 |
| Methanobrevibacter smithi | cov3 | 0 | Not detected | 0.238 | -0.101 | 0.578 | 0.169 |
| Methanocorpusculaceae ar | cov3 | 0 | Not detected | 0.155 | -0.082 | 0.393 | 0.201 |
| Methanosarcina mazei | cov3 | 0 | Not detected | 0.153 | -0.054 | 0.361 | 0.148 |

| Methylobacterium sp. ZN | cov3 | 33 | Not detected | -0.147 | -0.292 | -0.002 | 0.048 |
| --- | --- | --- | --- | --- | --- | --- | --- |
| Methyloglobulus morosus | cov3 | 0 | Not detected | 0.100 | -0.073 | 0.273 | 0.259 |
| Microcystis aeruginosa | cov3 | 0 | Not detected | 0.001 | -0.178 | 0.180 | 0.991 |
| Mitsuokella multacida | cov3 | 0 | Not detected | 0.130 | -0.180 | 0.439 | 0.411 |
| Mobilibacterium timonens | cov3 | 0 | Not detected | -0.065 | -0.235 | 0.105 | 0.452 |
| Mobiluncus curtisii | cov3 | 0 | Not detected | -0.027 | -0.099 | 0.045 | 0.466 |
| Mobiluncus mulieris | cov3 | 0 | Not detected | -0.020 | -0.102 | 0.061 | 0.622 |
| Mogibacterium timidum | cov3 | 0 | Not detected | -0.078 | -0.279 | 0.122 | 0.445 |
| Moraxella porci | cov3 | 0 | Not detected | 0.012 | -0.131 | 0.155 | 0.871 |
| Morganella morganii | cov3 | 0 | Not detected | 0.040 | -0.153 | 0.233 | 0.686 |
| Morococcus cerebrosus | cov3 | 0 | Not detected | 0.042 | -0.117 | 0.201 | 0.603 |
| Mucinivorans hirudinis | cov3 | 0 | Not detected | 0.012 | -0.129 | 0.153 | 0.869 |
| Murdochiella sp. Marseille | cov3 | 0 | Not detected | -0.017 | -0.110 | 0.077 | 0.727 |
| Mycoplasma sp. CAG:877 | cov3 | 0 | Not detected | 0.277 | 0.027 | 0.526 | 0.030 |
| Mycoplasma sp. CAG:956 | cov3 | 0 | Not detected | 0.137 | -0.082 | 0.356 | 0.220 |
| Myroides injenensis | cov3 | 0 | Not detected | -0.134 | -0.348 | 0.080 | 0.220 |
| Myroides odoratimimus | cov3 | 0 | Not detected | -0.214 | -0.438 | 0.009 | 0.060 |
| Necropsobacter massiliens | cov3 | 0 | Not detected | -0.009 | -0.206 | 0.189 | 0.932 |
| Negativicoccus massiliens | cov3 | 0 | Not detected | -0.016 | -0.172 | 0.139 | 0.836 |
| Neglecta timonensis | cov3 | 0 | Not detected | -0.068 | -0.174 | 0.038 | 0.210 |
| Neisseria cinerea | cov3 | 0 | Not detected | -0.072 | -0.241 | 0.097 | 0.403 |
| Neisseria mucosa | cov3 | 0 | Not detected | -0.105 | -0.281 | 0.071 | 0.243 |
| Nissabacter archeti | cov3 | 0 | Not detected | 0.064 | -0.135 | 0.263 | 0.527 |
| Novibacillus thermophilus | cov3 | 0 | Not detected | -0.027 | -0.206 | 0.152 | 0.766 |
| Obesumbacterium proteus | cov3 | 0 | Not detected | 0.195 | -0.043 | 0.432 | 0.109 |
| Oceanospirillum linum | cov3 | 0 | Not detected | 0.026 | -0.143 | 0.195 | 0.762 |
| Odoribacter laneus | cov3 | 773 | 0.6 | -0.241 | -0.398 | -0.084 | 0.003 |
| Odoribacter laneus CAG:5 | cov3 | 530 | Not detected | -0.270 | -0.482 | -0.058 | 0.013 |
| Odoribacter sp. 43_10 | cov3 | 0 | Not detected | -0.056 | -0.188 | 0.075 | 0.399 |
| Odoribacter sp. CAG:788 | cov3 | 224 | Not detected | -0.175 | -0.383 | 0.033 | 0.100 |
| Odoribacter splanchnicus | cov3 | 0 | Not detected | -0.103 | -0.201 | -0.006 | 0.038 |
| Odoribacter splanchnicus | cov3 | 0 | Not detected | -0.020 | -0.164 | 0.124 | 0.786 |
| Oligella ureolytica | cov3 | 0 | Not detected | 0.120 | -0.050 | 0.290 | 0.166 |
| Olsenella scatoligenes | cov3 | 0 | Not detected | 0.047 | -0.139 | 0.233 | 0.618 |
| Olsenella sp. Marseille-P3 | cov3 | 0 | Not detected | 0.165 | -0.018 | 0.347 | 0.077 |
| Olsenella sp. oral taxon 80 | cov3 | 0 | Not detected | 0.063 | -0.067 | 0.193 | 0.340 |
| Olsenella uli | cov3 | 0 | Not detected | -0.008 | -0.193 | 0.178 | 0.937 |
| Olsenella umbonata | cov3 | 0 | Not detected | 0.043 | -0.108 | 0.193 | 0.580 |
| Oribacterium parvum | cov3 | 0 | Not detected | 0.026 | -0.033 | 0.085 | 0.386 |
| Oribacterium sinus | cov3 | 0 | Not detected | -0.019 | -0.159 | 0.122 | 0.797 |
| Oribacterium sp. oral taxo | cov3 | 0 | Not detected | 0.038 | -0.058 | 0.134 | 0.437 |
| Oribacterium sp. WCC10 | cov3 | 0 | Not detected | 0.102 | -0.085 | 0.290 | 0.284 |
| Oscillibacter ruminantium | cov3 | 0 | Not detected | 0.035 | -0.098 | 0.167 | 0.609 |
| Oscillibacter sp. 1-3 | cov3 | 0 | Not detected | 0.025 | -0.048 | 0.099 | 0.497 |

| Oscillibacter sp. 57_20 | cov3 | 0 | Not detected | -0.003 | -0.235 | 0.228 | 0.977 |
| --- | --- | --- | --- | --- | --- | --- | --- |
| Oscillibacter sp. ER4 | cov3 | 0 | Not detected | 0.121 | -0.017 | 0.259 | 0.086 |
| Oscillibacter sp. KLE 174 | cov3 | 0 | Not detected | -0.055 | -0.174 | 0.064 | 0.362 |
| Oscillibacter valericigenes | cov3 | 0 | Not detected | 0.032 | -0.087 | 0.151 | 0.595 |
| Oscillospiraceae bacterium | cov3 | 0 | Not detected | 0.060 | -0.084 | 0.204 | 0.415 |
| Oxalobacter formigenes | cov3 | 0 | Not detected | 0.029 | -0.118 | 0.176 | 0.702 |
| Paeniclostridium sordellii | cov3 | 0 | Not detected | 0.216 | 0.015 | 0.416 | 0.035 |
| Paludibacter propionicigen | cov3 | 0 | Not detected | -0.038 | -0.174 | 0.099 | 0.590 |
| Pantoea agglomerans | cov3 | 0 | Not detected | -0.010 | -0.177 | 0.157 | 0.903 |
| Pantoea ananatis | cov3 | 0 | Not detected | 0.116 | -0.036 | 0.268 | 0.134 |
| Pantoea dispersa | cov3 | 0 | Not detected | 0.062 | -0.083 | 0.207 | 0.402 |
| Pantoea rwandensis | cov3 | 0 | Not detected | 0.036 | -0.124 | 0.195 | 0.659 |
| Pantoea septica | cov3 | 0 | Not detected | 0.062 | -0.078 | 0.202 | 0.387 |
| Pantoea sp. IMH | cov3 | 0 | Not detected | 0.025 | -0.137 | 0.187 | 0.761 |
| Pantoea sp. PSNIH2 | cov3 | 0 | Not detected | 0.075 | -0.176 | 0.326 | 0.557 |
| Parabacteroides distasonis | cov3 | 0 | Not detected | -0.061 | -0.195 | 0.073 | 0.373 |
| Parabacteroides goldsteini | cov3 | 0 | Not detected | -0.030 | -0.178 | 0.119 | 0.694 |
| Parabacteroides gordonii | cov3 | 0 | Not detected | -0.098 | -0.220 | 0.024 | 0.115 |
| Parabacteroides johnsonii | cov3 | 0 | Not detected | -0.131 | -0.239 | -0.024 | 0.017 |
| Parabacteroides merdae C | cov3 | 0 | Not detected | -0.212 | -0.409 | -0.015 | 0.035 |
| Paraburkholderia sacchari | cov3 | 0 | Not detected | -0.121 | -0.284 | 0.041 | 0.144 |
| Paraclostridium biferment | cov3 | 0 | Not detected | 0.181 | 0.001 | 0.361 | 0.048 |
| Paraprevotella clara CAG: | cov3 | 0 | Not detected | -0.154 | -0.391 | 0.083 | 0.204 |
| Paraprevotella xylaniphila | cov3 | 0 | Not detected | 0.024 | -0.103 | 0.151 | 0.708 |
| Parascardovia denticolens | cov3 | 195 | Not detected | 0.301 | 0.113 | 0.488 | 0.002 |
| Parasporobacterium pauci | cov3 | 0 | Not detected | -0.043 | -0.154 | 0.067 | 0.440 |
| Parasutterella excrementih | cov3 | 0 | Not detected | -0.149 | -0.385 | 0.087 | 0.216 |
| Parasutterella excrementih | cov3 | 0 | Not detected | -0.155 | -0.516 | 0.205 | 0.398 |
| Parvimonas micra | cov3 | 3 | Not detected | -0.205 | -0.415 | 0.004 | 0.055 |
| Pasteurella bettyae | cov3 | 0 | Not detected | -0.016 | -0.184 | 0.151 | 0.850 |
| Pectobacterium carotovoru | cov3 | 0 | Not detected | 0.022 | -0.130 | 0.175 | 0.775 |
| Pedobacter glucosidilyticu | cov3 | 0 | Not detected | 0.049 | -0.109 | 0.207 | 0.542 |
| Pelosinus fermentans | cov3 | 0 | Not detected | -0.049 | -0.202 | 0.103 | 0.525 |
| Peptoanaerobacter stomati | cov3 | 0 | Not detected | 0.006 | -0.153 | 0.164 | 0.944 |
| Peptococcus niger | cov3 | 0 | Not detected | -0.005 | -0.158 | 0.148 | 0.946 |
| Peptoniphilus duerdenii | cov3 | 0 | Not detected | 0.034 | -0.095 | 0.162 | 0.607 |
| Peptoniphilus grossensis | cov3 | 0 | Not detected | -0.057 | -0.228 | 0.113 | 0.510 |
| Peptoniphilus harei | cov3 | 0 | Not detected | 0.030 | -0.162 | 0.222 | 0.757 |
| Peptoniphilus lacrimalis | cov3 | 0 | Not detected | 0.066 | -0.139 | 0.271 | 0.528 |
| Peptoniphilus obesi | cov3 | 0 | Not detected | -0.090 | -0.268 | 0.087 | 0.319 |
| Peptoniphilus rhinitidis | cov3 | 0 | Not detected | 0.092 | -0.085 | 0.270 | 0.306 |
| Peptoniphilus senegalensis | cov3 | 0 | Not detected | 0.150 | -0.041 | 0.341 | 0.124 |
| Peptoniphilus sp. BV3C26 | cov3 | 0 | Not detected | 0.126 | -0.079 | 0.331 | 0.228 |
| Peptoniphilus sp. ING2-D | cov3 | 0 | Not detected | -0.037 | -0.212 | 0.138 | 0.681 |

| Peptoniphilus sp. oral taxo | cov3 | 0 | Not detected | 0.022 | -0.064 | 0.109 | 0.612 |
| --- | --- | --- | --- | --- | --- | --- | --- |
| Peptostreptococcaceae bac | cov3 | 0 | Not detected | 0.235 | 0.020 | 0.450 | 0.033 |
| Peptostreptococcus anaero | cov3 | 0 | Not detected | -0.001 | -0.067 | 0.065 | 0.975 |
| Peptostreptococcus anaero | cov3 | 0 | Not detected | -0.001 | -0.152 | 0.151 | 0.993 |
| Peptostreptococcus stomat | cov3 | 0 | Not detected | 0.117 | -0.068 | 0.301 | 0.215 |
| Petrimonas mucosa | cov3 | 426 | Not detected | 0.207 | 0.034 | 0.380 | 0.019 |
| Phaeodactylibacter xiamen | cov3 | 0 | Not detected | -0.282 | -0.509 | -0.055 | 0.015 |
| Phascolarctobacterium sp. | cov3 | 0 | Not detected | -0.139 | -0.482 | 0.203 | 0.425 |
| Phascolarctobacterium sp. | cov3 | 0 | Not detected | -0.143 | -0.386 | 0.101 | 0.251 |
| Phascolarctobacterium suc | cov3 | 0 | Not detected | -0.023 | -0.198 | 0.152 | 0.797 |
| Phocaeicola abscessus | cov3 | 0 | Not detected | -0.049 | -0.192 | 0.094 | 0.498 |
| Phocea massiliensis | cov3 | 0 | Not detected | -0.044 | -0.135 | 0.047 | 0.339 |
| Piscirickettsia salmonis | cov3 | 0 | Not detected | -0.032 | -0.178 | 0.114 | 0.665 |
| Plautia stali symbiont | cov3 | 0 | Not detected | -0.024 | -0.176 | 0.128 | 0.757 |
| Pluralibacter gergoviae | cov3 | 0 | Not detected | 0.008 | -0.229 | 0.246 | 0.945 |
| Pontibacillus litoralis | cov3 | 0 | Not detected | -0.096 | -0.313 | 0.122 | 0.390 |
| Porphyromonadaceae bact | cov3 | 0 | Not detected | -0.048 | -0.206 | 0.110 | 0.553 |
| Porphyromonadaceae bact | cov3 | 0 | Not detected | -0.015 | -0.171 | 0.142 | 0.854 |
| Porphyromonadaceae bact | cov3 | 0 | Not detected | -0.011 | -0.161 | 0.140 | 0.890 |
| Porphyromonas asaccharol | cov3 | 0 | Not detected | -0.164 | -0.361 | 0.032 | 0.101 |
| Porphyromonas bennonis | cov3 | 0 | Not detected | -0.120 | -0.305 | 0.065 | 0.202 |
| Porphyromonas cangingiv | cov3 | 0 | Not detected | 0.042 | -0.129 | 0.212 | 0.634 |
| Porphyromonas canoris | cov3 | 0 | Not detected | -0.060 | -0.231 | 0.112 | 0.496 |
| Porphyromonas catoniae | cov3 | 0 | Not detected | -0.037 | -0.169 | 0.094 | 0.578 |
| Porphyromonas creviorica | cov3 | 0 | Not detected | -0.065 | -0.183 | 0.054 | 0.284 |
| Porphyromonas endodonta | cov3 | 0 | Not detected | 0.060 | -0.129 | 0.250 | 0.532 |
| Porphyromonas gingivalis | cov3 | 0 | Not detected | 0.066 | -0.089 | 0.220 | 0.405 |
| Porphyromonas gingivican | cov3 | 0 | Not detected | -0.007 | -0.130 | 0.117 | 0.916 |
| Porphyromonas levii | cov3 | 0 | Not detected | 0.056 | -0.170 | 0.282 | 0.629 |
| Porphyromonas macacae | cov3 | 0 | Not detected | 0.047 | -0.121 | 0.214 | 0.585 |
| Porphyromonas somerae | cov3 | 0 | Not detected | -0.100 | -0.184 | -0.016 | 0.019 |
| Porphyromonas sp. CAG:1 | cov3 | 0 | Not detected | -0.206 | -0.410 | -0.002 | 0.048 |
| Porphyromonas sp. COT-2 | cov3 | 0 | Not detected | 0.008 | -0.194 | 0.210 | 0.939 |
| Prevotella albensis | cov3 | 0 | Not detected | 0.185 | -0.107 | 0.477 | 0.214 |
| Prevotella amnii | cov3 | 128 | Not detected | -0.127 | -0.205 | -0.049 | 0.001 |
| Prevotella aurantiaca | cov3 | 0 | Not detected | 0.072 | -0.115 | 0.259 | 0.452 |
| Prevotella baroniae | cov3 | 0 | Not detected | -0.020 | -0.112 | 0.072 | 0.672 |
| Prevotella bergensis | cov3 | 0 | Not detected | -0.007 | -0.150 | 0.136 | 0.925 |
| Prevotella bivia | cov3 | 0 | Not detected | -0.002 | -0.100 | 0.097 | 0.976 |
| Prevotella bryantii | cov3 | 0 | Not detected | 0.250 | -0.041 | 0.542 | 0.093 |
| Prevotella buccae | cov3 | 0 | Not detected | 0.116 | -0.107 | 0.339 | 0.308 |
| Prevotella buccalis | cov3 | 0 | Not detected | -0.007 | -0.092 | 0.077 | 0.863 |
| Prevotella copri | cov3 | 0 | Not detected | 0.313 | 0.003 | 0.622 | 0.048 |
| Prevotella corporis | cov3 | 312 | Not detected | -0.143 | -0.226 | -0.060 | 0.001 |

| Prevotella dentalis | cov3 | 0 | Not detected | 0.199 | -0.029 | 0.428 | 0.087 |
| --- | --- | --- | --- | --- | --- | --- | --- |
| Prevotella dentasini | cov3 | 0 | Not detected | 0.151 | -0.076 | 0.379 | 0.193 |
| Prevotella denticola | cov3 | 0 | Not detected | 0.172 | -0.082 | 0.427 | 0.184 |
| Prevotella disiens | cov3 | 0 | Not detected | -0.072 | -0.162 | 0.017 | 0.113 |
| Prevotella enoeca | cov3 | 0 | Not detected | 0.105 | -0.115 | 0.325 | 0.351 |
| Prevotella fusca | cov3 | 0 | Not detected | 0.161 | -0.092 | 0.413 | 0.213 |
| Prevotella histicola | cov3 | 0 | Not detected | 0.005 | -0.152 | 0.162 | 0.950 |
| Prevotella intermedia | cov3 | 0 | Not detected | 0.084 | -0.071 | 0.238 | 0.287 |
| Prevotella loescheii | cov3 | 0 | Not detected | 0.176 | -0.034 | 0.386 | 0.101 |
| Prevotella maculosa | cov3 | 0 | Not detected | -0.016 | -0.198 | 0.166 | 0.860 |
| Prevotella marshii | cov3 | 0 | Not detected | -0.026 | -0.115 | 0.063 | 0.563 |
| Prevotella melaninogenica | cov3 | 0 | Not detected | 0.149 | -0.029 | 0.327 | 0.101 |
| Prevotella micans | cov3 | 46 | Not detected | 0.234 | 0.023 | 0.445 | 0.029 |
| Prevotella multiformis | cov3 | 0 | Not detected | -0.005 | -0.137 | 0.127 | 0.938 |
| Prevotella multisaccharivo | cov3 | 0 | Not detected | 0.134 | -0.151 | 0.420 | 0.357 |
| Prevotella nanceiensis | cov3 | 0 | Not detected | -0.008 | -0.180 | 0.164 | 0.928 |
| Prevotella nigrescens | cov3 | 0 | Not detected | 0.164 | -0.043 | 0.370 | 0.120 |
| Prevotella oralis | cov3 | 0 | Not detected | 0.207 | -0.012 | 0.427 | 0.064 |
| Prevotella oris | cov3 | 0 | Not detected | 0.097 | -0.147 | 0.342 | 0.434 |
| Prevotella oryzae | cov3 | 0 | Not detected | 0.194 | -0.062 | 0.451 | 0.137 |
| Prevotella oulorum | cov3 | 0 | Not detected | 0.200 | -0.063 | 0.464 | 0.137 |
| Prevotella pallens | cov3 | 0 | Not detected | -0.032 | -0.130 | 0.066 | 0.521 |
| Prevotella paludivivens | cov3 | 0 | Not detected | 0.146 | -0.029 | 0.321 | 0.102 |
| Prevotella ruminicola | cov3 | 0 | Not detected | 0.210 | -0.036 | 0.455 | 0.094 |
| Prevotella saccharolytica | cov3 | 0 | Not detected | 0.127 | -0.109 | 0.363 | 0.291 |
| Prevotella salivae | cov3 | 0 | Not detected | -0.056 | -0.147 | 0.036 | 0.233 |
| Prevotella scopos | cov3 | 0 | Not detected | 0.036 | -0.088 | 0.159 | 0.573 |
| Prevotella sp. 10(H) | cov3 | 0 | Not detected | 0.072 | -0.080 | 0.224 | 0.355 |
| Prevotella sp. 109 | cov3 | 0 | Not detected | -0.063 | -0.149 | 0.024 | 0.158 |
| Prevotella sp. AGR2160 | cov3 | 0 | Not detected | 0.296 | 0.019 | 0.572 | 0.036 |
| Prevotella sp. BP1-148 | cov3 | 0 | Not detected | 0.123 | -0.005 | 0.251 | 0.059 |
| Prevotella sp. CAG:1058 | cov3 | 0 | Not detected | -0.149 | -0.373 | 0.074 | 0.190 |
| Prevotella sp. CAG:1092 | cov3 | 0 | Not detected | 0.320 | 0.030 | 0.610 | 0.031 |
| Prevotella sp. CAG:1124 | cov3 | 0 | Not detected | -0.057 | -0.291 | 0.177 | 0.632 |
| Prevotella sp. CAG:1185 | cov3 | 0 | Not detected | -0.117 | -0.359 | 0.126 | 0.346 |
| Prevotella sp. CAG:1320 | cov3 | 0 | Not detected | -0.049 | -0.237 | 0.139 | 0.612 |
| Prevotella sp. CAG:255 | cov3 | 0 | Not detected | -0.014 | -0.161 | 0.133 | 0.853 |
| Prevotella sp. CAG:386 | cov3 | 0 | Not detected | 0.294 | -0.024 | 0.612 | 0.070 |
| Prevotella sp. CAG:487 | cov3 | 0 | Not detected | -0.080 | -0.232 | 0.073 | 0.306 |
| Prevotella sp. CAG:520 | cov3 | 0 | Not detected | 0.153 | -0.112 | 0.417 | 0.258 |
| Prevotella sp. CAG:5226 | cov3 | 0 | Not detected | 0.118 | -0.170 | 0.406 | 0.421 |
| Prevotella sp. CAG:604 | cov3 | 0 | Not detected | 0.291 | -0.053 | 0.636 | 0.098 |
| Prevotella sp. CAG:732 | cov3 | 0 | Not detected | 0.268 | -0.044 | 0.579 | 0.092 |
| Prevotella sp. CAG:891 | cov3 | 0 | Not detected | 0.134 | -0.094 | 0.362 | 0.248 |

| Prevotella sp. FD3004 | cov3 | 0 | Not detected | 0.244 | 0.048 | 0.440 | 0.015 |
| --- | --- | --- | --- | --- | --- | --- | --- |
| Prevotella sp. HUN102 | cov3 | 0 | Not detected | 0.056 | -0.070 | 0.181 | 0.385 |
| Prevotella sp. kh1p2 | cov3 | 0 | Not detected | 0.243 | 0.020 | 0.466 | 0.033 |
| Prevotella sp. khp1 | cov3 | 0 | Not detected | 0.154 | -0.017 | 0.324 | 0.077 |
| Prevotella sp. MSX73 | cov3 | 0 | Not detected | 0.114 | -0.108 | 0.337 | 0.314 |
| Prevotella sp. ne3005 | cov3 | 0 | Not detected | 0.105 | -0.045 | 0.255 | 0.170 |
| Prevotella sp. oral taxon 4 | cov3 | 0 | Not detected | -0.056 | -0.279 | 0.168 | 0.625 |
| Prevotella sp. P4-76 | cov3 | 0 | Not detected | 0.139 | -0.025 | 0.304 | 0.097 |
| Prevotella sp. P6B1 | cov3 | 0 | Not detected | 0.190 | 0.054 | 0.326 | 0.006 |
| Prevotella sp. P6B4 | cov3 | 0 | Not detected | 0.185 | 0.053 | 0.317 | 0.006 |
| Prevotella sp. RM4 | cov3 | 0 | Not detected | 0.137 | -0.029 | 0.303 | 0.107 |
| Prevotella sp. S7-1-8 | cov3 | 0 | Not detected | -0.060 | -0.142 | 0.022 | 0.151 |
| Prevotella sp. S7 MS 2 | cov3 | 0 | Not detected | -0.036 | -0.125 | 0.052 | 0.421 |
| Prevotella stercorea | cov3 | 0 | Not detected | 0.012 | -0.110 | 0.134 | 0.850 |
| Prevotella stercorea CAG: | cov3 | 0 | Not detected | 0.099 | -0.176 | 0.374 | 0.479 |
| Prevotella timonensis | cov3 | 0 | Not detected | -0.049 | -0.135 | 0.037 | 0.266 |
| Prevotellamassilia timonen | cov3 | 0 | Not detected | 0.081 | -0.062 | 0.224 | 0.266 |
| Propionibacterium acidifac | cov3 | 0 | Not detected | 0.161 | -0.023 | 0.345 | 0.087 |
| Proteiniclasticum ruminis | cov3 | 0 | Not detected | -0.062 | -0.215 | 0.090 | 0.423 |
| Proteiniphilum acetatigene | cov3 | 0 | Not detected | 0.025 | -0.135 | 0.184 | 0.763 |
| Proteiniphilum saccharofe | cov3 | 0 | Not detected | -0.041 | -0.216 | 0.135 | 0.649 |
| Proteiniphilum sp. EBM-4 | cov3 | 0 | Not detected | -0.180 | -0.399 | 0.038 | 0.106 |
| Proteobacteria bacterium C | cov3 | 0 | Not detected | -0.131 | -0.477 | 0.214 | 0.457 |
| Proteobacteria bacterium C | cov3 | 0 | Not detected | -0.173 | -0.455 | 0.110 | 0.231 |
| Providencia rettgeri | cov3 | 0 | Not detected | 0.056 | -0.134 | 0.246 | 0.563 |
| Pseudobacteroides cellulos | cov3 | 0 | Not detected | -0.047 | -0.187 | 0.093 | 0.514 |
| Pseudobutyrivibrio rumini | cov3 | 0 | Not detected | -0.020 | -0.093 | 0.052 | 0.585 |
| Pseudobutyrivibrio sp. AC | cov3 | 0 | Not detected | 0.036 | -0.125 | 0.197 | 0.661 |
| Pseudobutyrivibrio sp. JW | cov3 | 0 | Not detected | -0.077 | -0.235 | 0.081 | 0.338 |
| Pseudobutyrivibrio sp. MD | cov3 | 0 | Not detected | 0.102 | -0.067 | 0.271 | 0.237 |
| Pseudobutyrivibrio sp. UC | cov3 | 0 | Not detected | 0.066 | -0.041 | 0.173 | 0.227 |
| Pseudobutyrivibrio sp. YE | cov3 | 0 | Not detected | 0.009 | -0.148 | 0.166 | 0.911 |
| Pseudobutyrivibrio xylani | cov3 | 0 | Not detected | 0.051 | -0.113 | 0.214 | 0.544 |
| Pseudoflavonifractor capil | cov3 | 0 | Not detected | -0.008 | -0.084 | 0.067 | 0.834 |
| Pseudopropionibacterium | cov3 | 0 | Not detected | 0.076 | -0.108 | 0.259 | 0.419 |
| Pyramidobacter piscolens | cov3 | 0 | Not detected | -0.038 | -0.275 | 0.200 | 0.756 |
| Rahnella aquatilis | cov3 | 0 | Not detected | 0.053 | -0.099 | 0.204 | 0.495 |
| Ralstonia mannitolilytica | cov3 | 0 | Not detected | 0.164 | -0.045 | 0.373 | 0.124 |
| Ralstonia pickettii | cov3 | 0 | Not detected | 0.030 | -0.168 | 0.228 | 0.766 |
| Raoultella ornithinolytica | cov3 | 0 | Not detected | 0.036 | -0.252 | 0.324 | 0.804 |
| Rappaport israeli | cov3 | 0 | Not detected | 0.038 | -0.123 | 0.198 | 0.645 |
| Rhodococcus defluvii | cov3 | 0 | Not detected | -0.043 | -0.197 | 0.110 | 0.581 |
| Rhodopseudomonas palust | cov3 | 0 | Not detected | -0.024 | -0.198 | 0.151 | 0.791 |
| Rhodospirillum sp. UNK. | cov3 | 0 | Not detected | -0.156 | -0.486 | 0.173 | 0.353 |

| Riemerella columbina | cov3 | 521 | Not detected | -0.149 | -0.233 | -0.066 | 0.000 |
| --- | --- | --- | --- | --- | --- | --- | --- |
| Rikenella microfusus | cov3 | 157 | Not detected | -0.133 | -0.218 | -0.049 | 0.002 |
| Risungbinella massiliensis | cov3 | 0 | Not detected | -0.070 | -0.208 | 0.067 | 0.317 |
| Robinsoniella peoriensis | cov3 | 0 | Not detected | -0.048 | -0.106 | 0.011 | 0.111 |
| Rodentibacter pneumotrop | cov3 | 0 | Not detected | 0.001 | -0.143 | 0.145 | 0.990 |
| Roseburia faecis | cov3 | 0 | Not detected | -0.076 | -0.205 | 0.054 | 0.252 |
| Roseburia hominis | cov3 | 0 | Not detected | 0.067 | -0.016 | 0.150 | 0.116 |
| Roseburia intestinalis | cov3 | 0 | Not detected | -0.069 | -0.194 | 0.056 | 0.280 |
| Roseburia inulinivorans | cov3 | 0 | Not detected | -0.006 | -0.108 | 0.096 | 0.907 |
| Roseburia sp. CAG:197_4 | cov3 | 0 | Not detected | -0.011 | -0.115 | 0.093 | 0.834 |
| Roseburia sp. CAG:309 | cov3 | 0 | Not detected | -0.112 | -0.337 | 0.113 | 0.329 |
| Roseburia sp. CAG:380 | cov3 | 0 | Not detected | -0.168 | -0.376 | 0.041 | 0.115 |
| Rothia dentocariosa | cov3 | 0 | Not detected | -0.099 | -0.322 | 0.123 | 0.382 |
| Rothia mucilaginosa | cov3 | 0 | Not detected | -0.008 | -0.241 | 0.225 | 0.948 |
| Ruminiclostridium thermo | cov3 | 0 | Not detected | 0.078 | -0.032 | 0.188 | 0.163 |
| Ruminococcaceae bacteriu | cov3 | 0 | Not detected | 0.081 | -0.061 | 0.223 | 0.265 |
| Ruminococcaceae bacteriu | cov3 | 0 | Not detected | 0.217 | 0.078 | 0.356 | 0.002 |
| Ruminococcaceae bacteriu | cov3 | 0 | Not detected | 0.011 | -0.059 | 0.080 | 0.765 |
| Ruminococcaceae bacteriu | cov3 | 0 | Not detected | 0.025 | -0.143 | 0.193 | 0.767 |
| Ruminococcus albus | cov3 | 0 | Not detected | 0.122 | -0.019 | 0.263 | 0.089 |
| Ruminococcus bicirculans | cov3 | 0 | Not detected | 0.102 | -0.110 | 0.314 | 0.345 |
| Ruminococcus callidus | cov3 | 0 | Not detected | 0.069 | -0.076 | 0.214 | 0.350 |
| Ruminococcus champanell | cov3 | 0 | Not detected | 0.076 | -0.105 | 0.257 | 0.410 |
| Ruminococcus faecis | cov3 | 0 | Not detected | 0.052 | -0.042 | 0.147 | 0.277 |
| Ruminococcus flavefacien | cov3 | 0 | Not detected | 0.089 | -0.041 | 0.219 | 0.181 |
| Ruminococcus gauvreauii | cov3 | 0 | Not detected | -0.039 | -0.115 | 0.037 | 0.319 |
| Ruminococcus lactaris | cov3 | 0 | Not detected | 0.037 | -0.071 | 0.146 | 0.498 |
| Ruminococcus obeum CA | cov3 | 0 | Not detected | -0.002 | -0.126 | 0.122 | 0.977 |
| Ruminococcus sp. AT10 | cov3 | 0 | Not detected | -0.065 | -0.134 | 0.004 | 0.065 |
| Ruminococcus sp. CAG:1 | cov3 | 0 | Not detected | 0.011 | -0.213 | 0.234 | 0.927 |
| Ruminococcus sp. CAG:1 | cov3 | 0 | Not detected | 0.325 | -0.025 | 0.675 | 0.069 |
| Ruminococcus sp. CAG:2 | cov3 | 0 | Not detected | -0.073 | -0.393 | 0.247 | 0.653 |
| Ruminococcus sp. CAG:3 | cov3 | 0 | Not detected | 0.191 | -0.130 | 0.511 | 0.244 |
| Ruminococcus sp. CAG:3 | cov3 | 0 | Not detected | -0.035 | -0.274 | 0.203 | 0.771 |
| Ruminococcus sp. CAG:3 | cov3 | 0 | Not detected | 0.206 | -0.099 | 0.510 | 0.186 |
| Ruminococcus sp. CAG:4 | cov3 | 0 | Not detected | 0.142 | -0.110 | 0.394 | 0.270 |
| Ruminococcus sp. CAG:4 | cov3 | 771 | 0.6 | 0.265 | 0.051 | 0.479 | 0.015 |
| Ruminococcus sp. CAG:5 | cov3 | 564 | Not detected | 0.287 | 0.053 | 0.521 | 0.016 |
| Ruminococcus sp. CAG:6 | cov3 | 0 | Not detected | 0.036 | -0.097 | 0.169 | 0.598 |
| Ruminococcus sp. CAG:6 | cov3 | 0 | Not detected | 0.091 | -0.144 | 0.325 | 0.448 |
| Ruminococcus sp. CAG:7 | cov3 | 0 | Not detected | 0.152 | -0.109 | 0.414 | 0.254 |
| Ruminococcus sp. FC2018 | cov3 | 0 | Not detected | 0.123 | -0.028 | 0.274 | 0.111 |
| Ruminococcus sp. HUN00 | cov3 | 0 | Not detected | 0.100 | -0.026 | 0.226 | 0.120 |
| Ruminococcus sp. NK3A7 | cov3 | 0 | Not detected | 0.134 | -0.021 | 0.288 | 0.089 |

| Ruminococcus sp. Phil15 | cov3 | 0 | Not detected | -0.001 | -0.203 | 0.202 | 0.994 |
| --- | --- | --- | --- | --- | --- | --- | --- |
| Ruminococcus sp. Zagget7 | cov3 | 0 | Not detected | -0.005 | -0.119 | 0.110 | 0.938 |
| Ruminococcus torques CA | cov3 | 0 | Not detected | -0.244 | -0.457 | -0.031 | 0.025 |
| Ruthenibacterium lactatifo | cov3 | 0 | Not detected | -0.075 | -0.177 | 0.027 | 0.151 |
| Salmonella enterica | cov3 | 0 | Not detected | 0.054 | -0.200 | 0.308 | 0.677 |
| Sanguibacteroides justesen | cov3 | 0 | Not detected | -0.109 | -0.268 | 0.049 | 0.178 |
| Scardovia inopinata | cov3 | 0 | Not detected | 0.197 | 0.044 | 0.350 | 0.012 |
| Scardovia wiggsiae | cov3 | 20 | Not detected | 0.214 | 0.014 | 0.414 | 0.037 |
| Selenomonas bovis | cov3 | 0 | Not detected | -0.045 | -0.178 | 0.088 | 0.507 |
| Selenomonas noxia | cov3 | 0 | Not detected | 0.147 | -0.005 | 0.299 | 0.058 |
| Selenomonas ruminantium | cov3 | 0 | Not detected | 0.141 | -0.044 | 0.327 | 0.135 |
| Selenomonas sputigena | cov3 | 0 | Not detected | 0.078 | -0.082 | 0.238 | 0.339 |
| Sellimonas intestinalis | cov3 | 0 | Not detected | 0.038 | -0.063 | 0.139 | 0.466 |
| Senegalimassilia anaerobia | cov3 | 0 | Not detected | 0.064 | -0.117 | 0.246 | 0.487 |
| Serratia fonticola | cov3 | 0 | Not detected | -0.013 | -0.159 | 0.133 | 0.860 |
| Serratia liquefaciens | cov3 | 0 | Not detected | 0.081 | -0.073 | 0.236 | 0.304 |
| Serratia marcescens | cov3 | 0 | Not detected | 0.057 | -0.111 | 0.225 | 0.505 |
| Serratia odorifera | cov3 | 0 | Not detected | 0.070 | -0.089 | 0.229 | 0.387 |
| Serratia rubidaea | cov3 | 0 | Not detected | 0.008 | -0.157 | 0.172 | 0.929 |
| Sharpea azabuensis | cov3 | 0 | Not detected | 0.018 | -0.069 | 0.105 | 0.687 |
| Shigella dysenteriae | cov3 | 0 | Not detected | 0.102 | -0.152 | 0.357 | 0.431 |
| Shigella flexneri | cov3 | 0 | Not detected | 0.113 | -0.141 | 0.366 | 0.384 |
| Shimwellia blattae | cov3 | 0 | Not detected | 0.073 | -0.139 | 0.286 | 0.499 |
| Shuttleworthia satelles | cov3 | 0 | Not detected | -0.009 | -0.107 | 0.089 | 0.856 |
| Siccibacter turicensis | cov3 | 0 | Not detected | 0.056 | -0.170 | 0.281 | 0.628 |
| Slackia exigua | cov3 | 0 | Not detected | -0.013 | -0.180 | 0.154 | 0.878 |
| Slackia heliotrinireducens | cov3 | 0 | Not detected | 0.032 | -0.116 | 0.180 | 0.673 |
| Slackia piriformis | cov3 | 0 | Not detected | 0.241 | -0.005 | 0.487 | 0.055 |
| Solobacterium moorei | cov3 | 0 | Not detected | 0.015 | -0.085 | 0.115 | 0.769 |
| Sphaerochaeta sp. Phil3 | cov3 | 0 | Not detected | 0.116 | -0.062 | 0.294 | 0.200 |
| Sphingobacterium wenxini | cov3 | 0 | Not detected | -0.114 | -0.297 | 0.068 | 0.220 |
| Spirochaetales bacterium S | cov3 | 10 | Not detected | -0.135 | -0.282 | 0.012 | 0.073 |
| Sporobacter termitidis | cov3 | 0 | Not detected | 0.115 | -0.031 | 0.260 | 0.122 |
| Sporomusa ovata | cov3 | 0 | Not detected | -0.033 | -0.200 | 0.134 | 0.698 |
| Staphylococcus pseudinter | cov3 | 0 | Not detected | 0.184 | 0.012 | 0.356 | 0.036 |
| Staphylococcus sp. CAG:3 | cov3 | 0 | Not detected | 0.170 | -0.135 | 0.475 | 0.276 |
| Staphylococcus sp. DORA | cov3 | 0 | Not detected | 0.057 | -0.112 | 0.225 | 0.510 |
| Staphylococcus warneri | cov3 | 0 | Not detected | 0.015 | -0.169 | 0.199 | 0.872 |
| Stomatobaculum longum | cov3 | 0 | Not detected | 0.012 | -0.044 | 0.067 | 0.677 |
| Streptobacillus hongkonge | cov3 | 0 | Not detected | 0.023 | -0.140 | 0.186 | 0.780 |
| Streptobacillus notomytis | cov3 | 0 | Not detected | -0.030 | -0.214 | 0.153 | 0.745 |
| Streptococcus agalactiae | cov3 | 0 | Not detected | 0.124 | -0.063 | 0.312 | 0.194 |
| Streptococcus anginosus | cov3 | 0 | Not detected | 0.210 | 0.003 | 0.417 | 0.047 |
| Streptococcus caballi | cov3 | 0 | Not detected | 0.078 | -0.114 | 0.271 | 0.426 |

| Streptococcus cristatus | cov3 | 0 | Not detected | 0.115 | -0.052 | 0.281 | 0.177 |
| --- | --- | --- | --- | --- | --- | --- | --- |
| Streptococcus dysgalactiae | cov3 | 0 | Not detected | 0.021 | -0.169 | 0.210 | 0.832 |
| Streptococcus entericus | cov3 | 0 | Not detected | 0.019 | -0.135 | 0.173 | 0.807 |
| Streptococcus gallolyticus | cov3 | 0 | Not detected | 0.143 | -0.087 | 0.373 | 0.223 |
| Streptococcus gordonii | cov3 | 0 | Not detected | 0.140 | -0.081 | 0.361 | 0.215 |
| Streptococcus halotolerans | cov3 | 0 | Not detected | 0.021 | -0.136 | 0.178 | 0.793 |
| Streptococcus henryi | cov3 | 0 | Not detected | 0.094 | -0.092 | 0.280 | 0.320 |
| Streptococcus ictaluri | cov3 | 0 | Not detected | 0.025 | -0.139 | 0.189 | 0.767 |
| Streptococcus infantis | cov3 | 0 | Not detected | 0.076 | -0.090 | 0.241 | 0.370 |
| Streptococcus iniae | cov3 | 0 | Not detected | 0.005 | -0.164 | 0.173 | 0.955 |
| Streptococcus intermedius | cov3 | 0 | Not detected | 0.122 | -0.091 | 0.335 | 0.260 |
| Streptococcus massiliensis | cov3 | 0 | Not detected | -0.067 | -0.241 | 0.106 | 0.445 |
| Streptococcus minor | cov3 | 0 | Not detected | 0.025 | -0.106 | 0.155 | 0.711 |
| Streptococcus mitis | cov3 | 0 | Not detected | -0.012 | -0.138 | 0.114 | 0.852 |
| Streptococcus mutans | cov3 | 0 | Not detected | 0.149 | -0.103 | 0.401 | 0.246 |
| Streptococcus oralis | cov3 | 0 | Not detected | 0.007 | -0.187 | 0.201 | 0.944 |
| Streptococcus orisratti | cov3 | 0 | Not detected | 0.081 | -0.103 | 0.265 | 0.391 |
| Streptococcus ovis | cov3 | 0 | Not detected | 0.091 | -0.078 | 0.261 | 0.291 |
| Streptococcus parasanguin | cov3 | 0 | Not detected | 0.119 | -0.080 | 0.318 | 0.241 |
| Streptococcus parauberis | cov3 | 0 | Not detected | 0.044 | -0.128 | 0.215 | 0.618 |
| Streptococcus phocae | cov3 | 0 | Not detected | -0.031 | -0.161 | 0.099 | 0.641 |
| Streptococcus plurextorum | cov3 | 0 | Not detected | -0.034 | -0.120 | 0.051 | 0.434 |
| Streptococcus porci | cov3 | 0 | Not detected | -0.045 | -0.148 | 0.057 | 0.389 |
| Streptococcus pseudoporci | cov3 | 0 | Not detected | 0.122 | -0.062 | 0.306 | 0.194 |
| Streptococcus pyogenes | cov3 | 0 | Not detected | 0.155 | -0.033 | 0.343 | 0.107 |
| Streptococcus ratti | cov3 | 0 | Not detected | -0.010 | -0.143 | 0.124 | 0.887 |
| Streptococcus salivarius | cov3 | 0 | Not detected | 0.079 | -0.104 | 0.262 | 0.399 |
| Streptococcus sanguinis | cov3 | 0 | Not detected | 0.175 | -0.040 | 0.390 | 0.111 |
| Streptococcus sobrinus | cov3 | 0 | Not detected | 0.156 | -0.041 | 0.353 | 0.121 |
| Streptococcus sp. X13SY0 | cov3 | 0 | Not detected | 0.040 | -0.095 | 0.175 | 0.559 |
| Streptococcus suis | cov3 | 0 | Not detected | 0.023 | -0.065 | 0.111 | 0.608 |
| Streptococcus thermophilu | cov3 | 0 | Not detected | -0.018 | -0.210 | 0.174 | 0.853 |
| Streptococcus uberis | cov3 | 0 | Not detected | 0.081 | -0.084 | 0.245 | 0.337 |
| Streptococcus urinalis | cov3 | 0 | Not detected | 0.029 | -0.159 | 0.216 | 0.763 |
| Streptomyces albulus | cov3 | 0 | Not detected | -0.056 | -0.210 | 0.097 | 0.471 |
| Streptomyces cattleya | cov3 | 0 | Not detected | 0.052 | -0.122 | 0.226 | 0.557 |
| Streptomyces kanamycetic | cov3 | 0 | Not detected | 0.005 | -0.110 | 0.121 | 0.929 |
| Subdoligranulum sp. CAG | cov3 | 0 | Not detected | -0.043 | -0.401 | 0.315 | 0.815 |
| Subdoligranulum variabile | cov3 | 0 | Not detected | 0.027 | -0.040 | 0.095 | 0.428 |
| Succinatimonas sp. CAG:7 | cov3 | 0 | Not detected | 0.049 | -0.246 | 0.344 | 0.746 |
| Succinispira mobilis | cov3 | 0 | Not detected | -0.072 | -0.247 | 0.102 | 0.418 |
| Succinivibrio sp. Phil9 | cov3 | 0 | Not detected | 0.098 | -0.183 | 0.379 | 0.494 |
| Sulfolobus acidocaldarius | cov3 | 0 | Not detected | -0.205 | -0.427 | 0.016 | 0.069 |
| Sutterella parvirubra | cov3 | 0 | Not detected | -0.068 | -0.259 | 0.122 | 0.483 |

| Sutterella sp. CAG:397 | cov3 | 0 | Not detected | 0.042 | -0.338 | 0.421 | 0.830 |
| --- | --- | --- | --- | --- | --- | --- | --- |
| Sutterella sp. CAG:521 | cov3 | 0 | Not detected | -0.156 | -0.394 | 0.082 | 0.199 |
| Sutterella wadsworthensis | cov3 | 0 | Not detected | 0.243 | -0.066 | 0.553 | 0.123 |
| Sutterella wadsworthensis | cov3 | 0 | Not detected | 0.210 | -0.176 | 0.596 | 0.286 |
| Sutterellaceae bacterium N | cov3 | 0 | Not detected | 0.092 | -0.141 | 0.326 | 0.439 |
| Synergistales bacterium Sy | cov3 | 0 | Not detected | 0.082 | -0.121 | 0.284 | 0.429 |
| Synergistes sp. 3_1_syn1 | cov3 | 0 | Not detected | 0.051 | -0.202 | 0.304 | 0.692 |
| Synergistes sp. Zagget9 | cov3 | 0 | Not detected | -0.179 | -0.338 | -0.021 | 0.027 |
| Tannerella forsythia | cov3 | 0 | Not detected | -0.085 | -0.203 | 0.033 | 0.158 |
| Tatumella morbirosei | cov3 | 0 | Not detected | 0.064 | -0.089 | 0.216 | 0.413 |
| Tepidanaerobacter sp. EB | cov3 | 0 | Not detected | -0.027 | -0.273 | 0.218 | 0.828 |
| Terrisporobacter glycolicu | cov3 | 0 | Not detected | 0.172 | -0.038 | 0.382 | 0.109 |
| Thermophagus xiamenensi | cov3 | 0 | Not detected | -0.097 | -0.285 | 0.091 | 0.312 |
| Thermosinus carboxydivor | cov3 | 0 | Not detected | 0.010 | -0.133 | 0.154 | 0.889 |
| Tidjanibacter massiliensis | cov3 | 0 | Not detected | 0.023 | -0.222 | 0.268 | 0.855 |
| Tissierellia bacterium S7-1 | cov3 | 0 | Not detected | -0.191 | -0.384 | 0.002 | 0.052 |
| Trabulsiella odontotermiti | cov3 | 0 | Not detected | -0.003 | -0.239 | 0.232 | 0.978 |
| Traorella massiliensis | cov3 | 0 | Not detected | -0.025 | -0.091 | 0.042 | 0.470 |
| Treponema denticola | cov3 | 0 | Not detected | 0.118 | -0.020 | 0.255 | 0.095 |
| Treponema medium | cov3 | 0 | Not detected | -0.189 | -0.416 | 0.038 | 0.102 |
| Treponema putidum | cov3 | 0 | Not detected | 0.102 | -0.020 | 0.223 | 0.100 |
| Treponema socranskii | cov3 | 0 | Not detected | 0.021 | -0.118 | 0.161 | 0.763 |
| Treponema succinifaciens | cov3 | 0 | Not detected | 0.025 | -0.058 | 0.108 | 0.553 |
| Treponema vincentii | cov3 | 0 | Not detected | 0.024 | -0.103 | 0.151 | 0.714 |
| Trichococcus flocculiform | cov3 | 0 | Not detected | -0.100 | -0.253 | 0.053 | 0.202 |
| Trueperella pyogenes | cov3 | 0 | Not detected | -0.088 | -0.242 | 0.067 | 0.266 |
| Tumebacillus flagellatus | cov3 | 0 | Not detected | 0.151 | -0.003 | 0.304 | 0.054 |
| Turicibacter sanguinis | cov3 | 0 | Not detected | 0.151 | -0.120 | 0.422 | 0.276 |
| Turicibacter sp. H121 | cov3 | 0 | Not detected | 0.028 | -0.078 | 0.134 | 0.605 |
| Tyzzerella nexilis | cov3 | 0 | Not detected | -0.067 | -0.129 | -0.004 | 0.037 |
| Urmitella timonensis | cov3 | 0 | Not detected | -0.020 | -0.138 | 0.097 | 0.737 |
| Vaginella massiliensis | cov3 | 0 | Not detected | -0.238 | -0.468 | -0.008 | 0.042 |
| Varibaculum cambriense | cov3 | 0 | Not detected | -0.024 | -0.120 | 0.073 | 0.630 |
| Varibaculum sp. Marseille | cov3 | 0 | Not detected | 0.040 | -0.112 | 0.191 | 0.607 |
| Veillonella magna | cov3 | 0 | Not detected | -0.034 | -0.103 | 0.034 | 0.327 |
| Veillonella parvula | cov3 | 0 | Not detected | 0.094 | -0.134 | 0.322 | 0.418 |
| Veillonella seminalis | cov3 | 0 | Not detected | -0.007 | -0.087 | 0.074 | 0.872 |
| Veillonella sp. ACP1 | cov3 | 0 | Not detected | -0.025 | -0.300 | 0.250 | 0.858 |
| Veillonella sp. CAG:933 | cov3 | 0 | Not detected | -0.084 | -0.219 | 0.050 | 0.220 |
| Veillonella sp. oral taxon 7 | cov3 | 0 | Not detected | -0.050 | -0.224 | 0.123 | 0.569 |
| Waddlia chondrophila | cov3 | 0 | Not detected | -0.189 | -0.404 | 0.026 | 0.085 |
| Weissella cibaria | cov3 | 0 | Not detected | -0.036 | -0.250 | 0.179 | 0.745 |
| Weissella confusa | cov3 | 0 | Not detected | -0.138 | -0.381 | 0.104 | 0.263 |
| Xanthomonas sp. Mitacek | cov3 | 0 | Not detected | 0.041 | -0.086 | 0.168 | 0.529 |

| Yersinia enterocolitica | cov3 | 0 | Not detected | 0.061 | -0.142 | 0.265 | 0.555 |
| --- | --- | --- | --- | --- | --- | --- | --- |
| Yersinia pestis | cov3 | 0 | Not detected | 0.116 | -0.142 | 0.374 | 0.378 |
| Yokenella regensburgei | cov3 | 0 | Not detected | -0.004 | -0.236 | 0.229 | 0.976 |

| **Supplementary Table 16**. Association of current education (High school or more vs. less than High school) with functional modules in overall participants (n=2273). Analysis of Compositions of Microbiomes (ANCOM2) was used to identify functional modules related to current education. Regression coefficients were obtained by modelling the effect of current education on centered log ratio (CLR)-transformed species in multivariable linear regression adjusted for age, gender, BMI, field center, Hispanic/Latino background,  U.S. nativity, Bristol stool type, parental education, the alternative Healthy Eating Index 2010 score, cigarette smoking, alcohol use,  depressive and anxiety symptoms, diabetes, hypertension, cardiovascular disease, dyslipidemia, and antibiotic use. | | | | | | | |
| --- | --- | --- | --- | --- | --- | --- | --- |
| **otu.names** | **pathname** | **W_stat** | **ANCOM** | **beta** | **l95ci** | **u95ci** | **qval** |
| PWY.7644 | heparin degradation | 519 | 0.7 | -0.257 | -0.467 | -0.048 | 0.016 |
| ADENOSYLHOM | L-methionine salvage from L | 0 | Not detected | -0.041 | -0.114 | 0.033 | 0.277 |
| ALANINE.VALIN | L-alanine biosynthesis I | 0 | Not detected | 0.122 | -0.055 | 0.299 | 0.177 |
| ANAGLYCOLYSI | glycolysis III (from glucose) | 0 | Not detected | -0.039 | -0.105 | 0.026 | 0.239 |
| APYRASE.RXN | NA | 0 | Not detected | -0.031 | -0.100 | 0.038 | 0.378 |
| ARABCAT.PWY | L-arabinose degradation I | 0 | Not detected | -0.026 | -0.098 | 0.046 | 0.481 |
| ARGDEG.III.PWY | L-arginine degradation IV (a | 0 | Not detected | -0.058 | -0.161 | 0.045 | 0.269 |
| ARGSYN.PWY | L-arginine biosynthesis I (vi | 0 | Not detected | -0.055 | -0.167 | 0.056 | 0.330 |
| ARGSYNBSUB.P | L-arginine biosynthesis II (a | 0 | Not detected | -0.053 | -0.162 | 0.056 | 0.341 |
| AST.PWY | L-arginine degradation II (A | 0 | Not detected | -0.005 | -0.214 | 0.205 | 0.966 |
| BETSYN.PWY | glycine betaine biosynthesis | 0 | Not detected | 0.197 | 0.005 | 0.389 | 0.044 |
| CALVIN.PWY | Calvin-Benson-Bassham cyc | 0 | Not detected | -0.039 | -0.103 | 0.025 | 0.236 |
| CARBPSYN.RXN | NA | 0 | Not detected | 0.040 | -0.097 | 0.176 | 0.570 |
| CARNMET.PWY | L-carnitine degradation I | 0 | Not detected | 0.052 | -0.166 | 0.269 | 0.642 |
| CENTFERM.PWY | pyruvate fermentation to but | 0 | Not detected | -0.028 | -0.114 | 0.058 | 0.526 |
| CHLOROPHYLL.S | 3,8-divinyl-chlorophyllide < | 0 | Not detected | 0.146 | -0.064 | 0.356 | 0.173 |
| CHOLINE.BETAI | choline degradation I | 0 | Not detected | 0.197 | 0.005 | 0.389 | 0.044 |
| COBALSYN.PWY | superpathway of adenosylco | 0 | Not detected | 0.049 | -0.114 | 0.213 | 0.554 |
| CODH.PWY | reductive acetyl coenzyme A | 0 | Not detected | -0.125 | -0.231 | -0.019 | 0.021 |
| COLANSYN.PWY | colanic acid building blocks | 0 | Not detected | -0.077 | -0.205 | 0.051 | 0.239 |
| DAPLYSINESYN. | L-lysine biosynthesis I | 0 | Not detected | -0.115 | -0.325 | 0.095 | 0.284 |
| DARABCATK12.P | D-arabinose degradation I | 0 | Not detected | 0.021 | -0.209 | 0.251 | 0.859 |
| DENITRIFICATIO | nitrate reduction I (denitrific | 0 | Not detected | 0.071 | -0.119 | 0.260 | 0.465 |
| DETOX1.PWY | superoxide radicals degradat | 0 | Not detected | 0.088 | -0.123 | 0.299 | 0.413 |
| DETOX1.PWY.1 | reactive oxygen species degr | 0 | Not detected | 0.103 | -0.113 | 0.318 | 0.352 |
| DHGLUCONATE. | glucose degradation (oxidati | 0 | Not detected | 0.158 | -0.054 | 0.370 | 0.145 |
| DISSULFRED.PW | dissimilatory sulfate reductio | 0 | Not detected | 0.094 | -0.117 | 0.304 | 0.384 |
| DTDPRHAMSYN. | dTDP-L-rhamnose biosynthe | 0 | Not detected | -0.040 | -0.109 | 0.029 | 0.256 |
| ECASYN.PWY | enterobacterial common anti | 0 | Not detected | 0.191 | -0.030 | 0.412 | 0.091 |
| ETOH.ACETYLCO | ethanol degradation I | 0 | Not detected | 0.175 | -0.060 | 0.410 | 0.145 |
| FAO.PWY | fatty acid &beta;-oxidation I | 0 | Not detected | 0.219 | -0.048 | 0.486 | 0.108 |
| FASYN.ELONG.P | fatty acid elongation -- satur | 0 | Not detected | -0.099 | -0.333 | 0.136 | 0.409 |
| FERMENTATION. | mixed acid fermentation | 0 | Not detected | -0.030 | -0.096 | 0.035 | 0.369 |
| FHLMULTI.RXN | NA | 0 | Not detected | -0.056 | -0.266 | 0.154 | 0.601 |
| FUCCAT.PWY | fucose degradation | 0 | Not detected | 0.021 | -0.209 | 0.251 | 0.859 |
| GALACTARDEG. | D-galactarate degradation I | 0 | Not detected | -0.075 | -0.207 | 0.057 | 0.266 |
| GALACTCAT.PW | D-galactonate degradation | 0 | Not detected | 0.108 | -0.113 | 0.328 | 0.339 |

| GALACTUROCAT | D-galacturonate degradation | 0 | Not detected | -0.041 | -0.109 | 0.026 | 0.232 |
| --- | --- | --- | --- | --- | --- | --- | --- |
| GCVMULTI.RXN | NA | 0 | Not detected | 0.016 | -0.156 | 0.189 | 0.851 |
| GDPRHAMSYN.P | GDP-D-rhamnose biosynthe | 0 | Not detected | -0.074 | -0.304 | 0.155 | 0.527 |
| GLUCARDEG.PW | <i>D</i>-glucarate degradat | 0 | Not detected | -0.099 | -0.236 | 0.038 | 0.156 |
| GLUCONEO.PWY | gluconeogenesis I | 0 | Not detected | -0.039 | -0.105 | 0.027 | 0.248 |
| GLUCOSE1PMET | glucose and glucose-1-phosp | 0 | Not detected | 0.010 | -0.092 | 0.113 | 0.841 |
| GLUDEG.I.PWY | GABA shunt | 0 | Not detected | 0.000 | -0.195 | 0.196 | 0.996 |
| GLUTATHIONES | glutathione biosynthesis | 0 | Not detected | 0.044 | -0.138 | 0.226 | 0.635 |
| GLUTORN.PWY | L-ornithine biosynthesis I | 0 | Not detected | 0.089 | -0.116 | 0.293 | 0.396 |
| GLYCLEAV.PWY | glycine cleavage | 0 | Not detected | 0.016 | -0.156 | 0.189 | 0.851 |
| GLYCOCAT.PWY | glycogen degradation I | 0 | Not detected | 0.019 | -0.080 | 0.118 | 0.708 |
| GLYCOGENSYNT | glycogen biosynthesis I (fro | 0 | Not detected | 0.031 | -0.055 | 0.117 | 0.482 |
| GLYCOLATEMET | glycolate and glyoxylate deg | 0 | Not detected | 0.049 | -0.161 | 0.259 | 0.648 |
| GLYCOLYSIS | glycolysis I (from glucose 6- | 0 | Not detected | -0.040 | -0.106 | 0.026 | 0.233 |
| GLYCOLYSIS.E.D | superpathway of glycolysis a | 0 | Not detected | 0.041 | -0.147 | 0.229 | 0.671 |
| GLYOXDEG.PWY | glycolate and glyoxylate deg | 0 | Not detected | 0.087 | -0.129 | 0.303 | 0.431 |
| GLYOXYLATE.B | glyoxylate cycle | 0 | Not detected | -0.015 | -0.088 | 0.059 | 0.698 |
| HCAMHPDEG.PW | 3-phenylpropanoate and 3-(3 | 0 | Not detected | 0.055 | -0.132 | 0.243 | 0.564 |
| HEME.BIOSYNTH | heme <i>b</i> biosynthesis | 0 | Not detected | 0.173 | -0.038 | 0.384 | 0.108 |
| HEMESYN2.PWY | heme <i>b</i> biosynthesis | 0 | Not detected | 0.285 | 0.072 | 0.498 | 0.009 |
| HISDEG.PWY | L-histidine degradation I | 0 | Not detected | 0.021 | -0.175 | 0.216 | 0.836 |
| HISHP.PWY | L-histidine degradation VI | 0 | Not detected | 0.021 | -0.175 | 0.216 | 0.836 |
| HISTSYN.PWY | L-histidine biosynthesis | 0 | Not detected | -0.048 | -0.118 | 0.022 | 0.182 |
| HOMOSER.METS | L-methionine biosynthesis I | 0 | Not detected | -0.030 | -0.097 | 0.038 | 0.386 |
| HOMOSER.THRE | L-threonine biosynthesis | 0 | Not detected | 0.042 | -0.176 | 0.260 | 0.707 |
| HOMOSERSYN.P | L-homoserine biosynthesis | 0 | Not detected | -0.066 | -0.274 | 0.141 | 0.533 |
| HSERMETANA.P | L-methionine biosynthesis II | 0 | Not detected | -0.037 | -0.106 | 0.032 | 0.298 |
| ILEUSYN.PWY | L-isoleucine biosynthesis I ( | 0 | Not detected | -0.029 | -0.111 | 0.053 | 0.487 |
| KDO.LIPASYN.P | (Kdo)<sub>2</sub>-lipid A | 0 | Not detected | 0.168 | -0.018 | 0.354 | 0.076 |
| KDO.NAGLIPASY | superpathway of (Kdo)<SUB | 0 | Not detected | 0.155 | -0.054 | 0.364 | 0.147 |
| KDOSYN.PWY | Kdo transfer to lipid IV<sub | 0 | Not detected | 0.124 | -0.094 | 0.342 | 0.265 |
| KETOGLUCONM | ketogluconate metabolism | 0 | Not detected | 0.074 | -0.106 | 0.254 | 0.421 |
| LACTOSECAT.PW | lactose and galactose degrad | 0 | Not detected | 0.162 | -0.055 | 0.380 | 0.144 |
| LARABITOLUTIL | xylitol degradation | 0 | Not detected | 0.214 | -0.019 | 0.447 | 0.072 |
| LEUSYN.PWY | L-leucine biosynthesis | 0 | Not detected | -0.104 | -0.215 | 0.007 | 0.065 |
| LIPA.CORESYN.P | lipid A-core biosynthesis (<i | 0 | Not detected | 0.136 | -0.097 | 0.369 | 0.254 |
| LYSDEGII.PWY | L-lysine degradation III | 0 | Not detected | -0.061 | -0.229 | 0.106 | 0.474 |
| LYXMET.PWY | L-lyxose degradation | 0 | Not detected | -0.021 | -0.085 | 0.044 | 0.526 |
| MALATE.ASPART | L-aspartate degradation II | 0 | Not detected | -0.130 | -0.369 | 0.109 | 0.285 |
| MANNCAT.PWY | D-mannose degradation | 0 | Not detected | 0.054 | -0.145 | 0.252 | 0.597 |
| MANNIDEG.PWY | mannitol degradation I | 0 | Not detected | 0.079 | -0.132 | 0.289 | 0.463 |
| MENAQUINONES | menaquinol-8 biosynthesis | 0 | Not detected | -0.028 | -0.242 | 0.185 | 0.795 |
| METH.ACETATE. | methanogenesis from acetate | 0 | Not detected | -0.031 | -0.093 | 0.030 | 0.316 |
| NAGLIPASYN.PW | lipid IV<sub>A</sub> biosy | 0 | Not detected | 0.004 | -0.215 | 0.224 | 0.968 |

| NONMEVIPP.PW | methylerythritol phosphate p | 0 | Not detected | -0.037 | -0.109 | 0.035 | 0.309 |
| --- | --- | --- | --- | --- | --- | --- | --- |
| NONOXIPENT.PW | pentose phosphate pathway ( | 0 | Not detected | 0.029 | -0.114 | 0.172 | 0.689 |
| NPGLUCAT.PWY | Entner-Doudoroff pathway I | 0 | Not detected | 0.071 | -0.105 | 0.247 | 0.429 |
| OANTIGEN.PWY | <i>O</i>-antigen building b | 0 | Not detected | -0.088 | -0.168 | -0.008 | 0.032 |
| OXIDATIVEPENT | pentose phosphate pathway ( | 0 | Not detected | -0.051 | -0.187 | 0.085 | 0.465 |
| P101.PWY | ectoine biosynthesis | 0 | Not detected | -0.068 | -0.276 | 0.140 | 0.522 |
| P105.PWY | TCA cycle IV (2-oxoglutarat | 0 | Not detected | -0.020 | -0.090 | 0.050 | 0.576 |
| P108.PWY | pyruvate fermentation to pro | 0 | Not detected | -0.126 | -0.212 | -0.041 | 0.004 |
| P121.PWY | adenine and adenosine salva | 0 | Not detected | 0.000 | -0.210 | 0.209 | 0.997 |
| P122.PWY | heterolactic fermentation | 0 | Not detected | -0.035 | -0.097 | 0.027 | 0.269 |
| P124.PWY | Bifidobacterium shunt | 0 | Not detected | -0.036 | -0.097 | 0.024 | 0.243 |
| P161.PWY | acetylene degradation (anaer | 0 | Not detected | -0.031 | -0.088 | 0.026 | 0.286 |
| P162.PWY | L-glutamate degradation V ( | 0 | Not detected | -0.028 | -0.114 | 0.058 | 0.526 |
| P163.PWY | L-lysine fermentation to acet | 0 | Not detected | -0.031 | -0.095 | 0.033 | 0.339 |
| P164.PWY | purine nucleobases degradat | 0 | Not detected | -0.069 | -0.132 | -0.006 | 0.033 |
| P181.PWY | nicotine degradation I (pyrid | 0 | Not detected | 0.029 | -0.158 | 0.216 | 0.761 |
| P185.PWY | formaldehyde assimilation II | 0 | Not detected | -0.039 | -0.103 | 0.025 | 0.232 |
| P2.PWY | citrate lyase activation | 0 | Not detected | 0.155 | -0.033 | 0.343 | 0.106 |
| P21.PWY | pentose phosphate pathway ( | 0 | Not detected | 0.029 | -0.114 | 0.172 | 0.689 |
| P23.PWY | reductive TCA cycle I | 0 | Not detected | -0.029 | -0.106 | 0.049 | 0.467 |
| P3.PWY | gallate degradation III (anaer | 0 | Not detected | -0.031 | -0.094 | 0.033 | 0.346 |
| P341.PWY | glycolysis V (Pyrococcus) | 0 | Not detected | -0.037 | -0.105 | 0.031 | 0.284 |
| P42.PWY | incomplete reductive TCA c | 0 | Not detected | -0.062 | -0.158 | 0.035 | 0.208 |
| P562.PWY | <I>myo</I>-inositol degrada | 0 | Not detected | 0.027 | -0.108 | 0.163 | 0.692 |
| PEPTIDOGLYCAN | peptidoglycan biosynthesis I | 0 | Not detected | 0.058 | -0.143 | 0.260 | 0.570 |
| PHOSLIPSYN2.PW | superpathway of phospholip | 0 | Not detected | -0.016 | -0.211 | 0.179 | 0.873 |
| PHOSPHONOTAS | 2-aminoethylphosphonate de | 0 | Not detected | 0.199 | -0.026 | 0.423 | 0.083 |
| PPGPPMET.PWY | ppGpp biosynthesis | 0 | Not detected | -0.041 | -0.099 | 0.017 | 0.163 |
| PUTDEG.PWY | putrescine degradation I | 0 | Not detected | 0.098 | -0.067 | 0.264 | 0.245 |
| PWY.1042 | glycolysis IV (plant cytosol) | 0 | Not detected | -0.043 | -0.108 | 0.023 | 0.200 |
| PWY.1186 | L-homomethionine biosynth | 0 | Not detected | 0.145 | -0.057 | 0.347 | 0.158 |
| PWY.1269 | CMP-3-deoxy-D-<I>manno | 0 | Not detected | 0.074 | -0.143 | 0.290 | 0.505 |
| PWY.1281 | sulfoacetaldehyde degradatio | 0 | Not detected | -0.043 | -0.118 | 0.033 | 0.269 |
| PWY.1341 | phenylacetate degradation II | 0 | Not detected | -0.117 | -0.225 | -0.008 | 0.036 |
| PWY.1361 | benzoyl-CoA degradation I ( | 0 | Not detected | 0.037 | -0.159 | 0.233 | 0.711 |
| PWY.1622 | formaldehyde assimilation I | 0 | Not detected | 0.013 | -0.124 | 0.151 | 0.849 |
| PWY.1722 | formate assimilation into 5,1 | 0 | Not detected | -0.125 | -0.231 | -0.019 | 0.021 |
| PWY.181 | photorespiration | 0 | Not detected | 0.045 | -0.110 | 0.199 | 0.569 |
| PWY.1861 | formaldehyde assimilation II | 0 | Not detected | -0.037 | -0.113 | 0.040 | 0.348 |
| PWY.2 | putrescine degradation IV | 0 | Not detected | 0.098 | -0.067 | 0.264 | 0.245 |
| PWY.2161 | folate polyglutamylation | 0 | Not detected | -0.001 | -0.220 | 0.217 | 0.991 |
| PWY.2201 | folate transformations I | 0 | Not detected | -0.055 | -0.124 | 0.014 | 0.119 |
| PWY.2221 | Entner-Doudoroff pathway I | 0 | Not detected | -0.047 | -0.111 | 0.017 | 0.148 |
| PWY.2361 | 3-oxoadipate degradation | 0 | Not detected | -0.020 | -0.181 | 0.141 | 0.804 |

| PWY.2661 | trehalose biosynthesis V | 0 | Not detected | 0.171 | -0.065 | 0.407 | 0.156 |
| --- | --- | --- | --- | --- | --- | --- | --- |
| PWY.2722 | trehalose degradation IV | 0 | Not detected | 0.103 | -0.081 | 0.286 | 0.272 |
| PWY.2723 | trehalose degradation V | 0 | Not detected | 0.019 | -0.092 | 0.129 | 0.738 |
| PWY.2781 | <i>cis</i>-zeatin biosynthes | 0 | Not detected | -0.058 | -0.257 | 0.140 | 0.563 |
| PWY.2941 | L-lysine biosynthesis II | 0 | Not detected | -0.066 | -0.274 | 0.141 | 0.533 |
| PWY.2942 | L-lysine biosynthesis III | 0 | Not detected | -0.066 | -0.274 | 0.141 | 0.533 |
| PWY.3 | putrescine degradation V | 0 | Not detected | 0.090 | -0.077 | 0.256 | 0.293 |
| PWY.3801 | sucrose degradation II (sucro | 0 | Not detected | -0.028 | -0.090 | 0.034 | 0.378 |
| PWY.3821 | D-galactose detoxification | 0 | Not detected | -0.057 | -0.162 | 0.048 | 0.287 |
| PWY.3841 | folate transformations II | 0 | Not detected | -0.054 | -0.122 | 0.014 | 0.120 |
| PWY.3861 | mannitol degradation II | 0 | Not detected | 0.061 | -0.148 | 0.270 | 0.567 |
| PWY.3881 | mannitol biosynthesis | 0 | Not detected | 0.051 | -0.157 | 0.260 | 0.631 |
| PWY.3941 | &beta;-alanine biosynthesis | 0 | Not detected | -0.040 | -0.262 | 0.181 | 0.721 |
| PWY.4041 | &gamma;-glutamyl cycle | 0 | Not detected | 0.002 | -0.212 | 0.217 | 0.983 |
| PWY.4101 | D-sorbitol degradation I | 0 | Not detected | 0.138 | -0.071 | 0.348 | 0.195 |
| PWY.43 | putrescine biosynthesis II | 0 | Not detected | -0.058 | -0.161 | 0.045 | 0.269 |
| PWY.4321 | L-glutamate degradation IV | 0 | Not detected | 0.010 | -0.185 | 0.205 | 0.917 |
| PWY.4361 | <i>S</i>-methyl-5-thio-&al | 0 | Not detected | 0.008 | -0.209 | 0.225 | 0.942 |
| PWY.4381 | fatty acid biosynthesis initiat | 0 | Not detected | 0.177 | -0.025 | 0.380 | 0.086 |
| PWY.4702 | phytate degradation I | 0 | Not detected | 0.108 | -0.102 | 0.318 | 0.313 |
| PWY.4983 | nitric oxide biosynthesis II ( | 0 | Not detected | -0.055 | -0.167 | 0.056 | 0.331 |
| PWY.4984 | urea cycle | 0 | Not detected | 0.021 | -0.070 | 0.113 | 0.646 |
| PWY.5022 | 4-aminobutanoate degradatio | 0 | Not detected | -0.038 | -0.118 | 0.042 | 0.347 |
| PWY.5028 | L-histidine degradation II | 0 | Not detected | 0.022 | -0.175 | 0.218 | 0.829 |
| PWY.5030 | L-histidine degradation III | 0 | Not detected | -0.110 | -0.210 | -0.010 | 0.031 |
| PWY.5041 | <i>S</i>-adenosyl-L-methio | 0 | Not detected | 0.078 | -0.142 | 0.299 | 0.487 |
| PWY.5049 | rosmarinic acid biosynthesis | 0 | Not detected | 0.002 | -0.158 | 0.162 | 0.982 |
| PWY.5054 | D-sorbitol biosynthesis I | 0 | Not detected | -0.035 | -0.104 | 0.035 | 0.329 |
| PWY.5097 | L-lysine biosynthesis VI | 0 | Not detected | -0.055 | -0.263 | 0.152 | 0.601 |
| PWY.5101 | L-isoleucine biosynthesis II | 0 | Not detected | -0.115 | -0.323 | 0.093 | 0.280 |
| PWY.5103 | L-isoleucine biosynthesis III | 0 | Not detected | -0.029 | -0.111 | 0.053 | 0.487 |
| PWY.5104 | L-isoleucine biosynthesis IV | 0 | Not detected | 0.022 | -0.199 | 0.244 | 0.844 |
| PWY.5122 | geranyl diphosphate biosynt | 0 | Not detected | 0.063 | -0.128 | 0.253 | 0.518 |
| PWY.5123 | <i>trans, trans</i>-farnesyl | 0 | Not detected | 0.122 | -0.102 | 0.346 | 0.286 |
| PWY.5136 | fatty acid &beta;-oxidation I | 0 | Not detected | 0.209 | -0.037 | 0.455 | 0.096 |
| PWY.5137 | fatty acid &beta;-oxidation I | 0 | Not detected | 0.112 | -0.079 | 0.304 | 0.251 |
| PWY.5138 | unsaturated, even numbered | 0 | Not detected | 0.137 | -0.083 | 0.356 | 0.223 |
| PWY.5154 | L-arginine biosynthesis III ( | 0 | Not detected | -0.053 | -0.162 | 0.056 | 0.342 |
| PWY.5162 | 2-hydroxypenta-2,4-dienoate | 0 | Not detected | 0.200 | -0.025 | 0.425 | 0.081 |
| PWY.5177 | glutaryl-CoA degradation | 0 | Not detected | -0.051 | -0.169 | 0.067 | 0.395 |
| PWY.5188 | tetrapyrrole biosynthesis I (f | 0 | Not detected | 0.020 | -0.080 | 0.120 | 0.695 |
| PWY.5189 | tetrapyrrole biosynthesis II ( | 0 | Not detected | 0.084 | -0.065 | 0.232 | 0.271 |
| PWY.5265 | peptidoglycan biosynthesis I | 0 | Not detected | -0.119 | -0.238 | 0.001 | 0.051 |
| PWY.5269 | cardiolipin biosynthesis II | 0 | Not detected | 0.079 | -0.116 | 0.274 | 0.430 |

| PWY.5280 | L-lysine degradation IV | 0 | Not detected | -0.062 | -0.234 | 0.110 | 0.482 |
| --- | --- | --- | --- | --- | --- | --- | --- |
| PWY.5306 | superpathway of thiosulfate | 0 | Not detected | 0.094 | -0.117 | 0.304 | 0.384 |
| PWY.5316 | nicotine biosynthesis | 0 | Not detected | 0.016 | -0.234 | 0.266 | 0.900 |
| PWY.5344 | L-homocysteine biosynthesis | 0 | Not detected | -0.045 | -0.119 | 0.028 | 0.228 |
| PWY.5352 | thiosulfate disproportionatio | 0 | Not detected | 0.038 | -0.162 | 0.238 | 0.707 |
| PWY.5360 | superpathway of tetrathionat | 0 | Not detected | 0.094 | -0.117 | 0.304 | 0.384 |
| PWY.5381 | pyridine nucleotide cycling ( | 0 | Not detected | 0.024 | -0.182 | 0.230 | 0.817 |
| PWY.5384 | sucrose degradation IV (sucr | 0 | Not detected | -0.029 | -0.091 | 0.033 | 0.357 |
| PWY.5386 | methylglyoxal degradation I | 0 | Not detected | 0.153 | -0.055 | 0.360 | 0.149 |
| PWY.5392 | reductive TCA cycle II | 0 | Not detected | -0.028 | -0.107 | 0.051 | 0.492 |
| PWY.5394 | betalamic acid biosynthesis | 0 | Not detected | 0.041 | -0.111 | 0.193 | 0.595 |
| PWY.5436 | L-threonine degradation IV | 0 | Not detected | 0.199 | -0.026 | 0.423 | 0.083 |
| PWY.5437 | L-threonine degradation I | 0 | Not detected | 0.164 | -0.057 | 0.386 | 0.147 |
| PWY.5453 | methylglyoxal degradation II | 0 | Not detected | -0.054 | -0.118 | 0.009 | 0.094 |
| PWY.5458 | methylglyoxal degradation V | 0 | Not detected | -0.075 | -0.277 | 0.126 | 0.463 |
| PWY.5459 | methylglyoxal degradation I | 0 | Not detected | -0.075 | -0.277 | 0.126 | 0.463 |
| PWY.5480 | pyruvate fermentation to eth | 0 | Not detected | 0.175 | -0.060 | 0.410 | 0.145 |
| PWY.5482 | pyruvate fermentation to ace | 0 | Not detected | -0.031 | -0.093 | 0.030 | 0.316 |
| PWY.5484 | glycolysis II (from fructose 6 | 0 | Not detected | -0.042 | -0.106 | 0.022 | 0.202 |
| PWY.5485 | pyruvate fermentation to ace | 0 | Not detected | -0.031 | -0.093 | 0.030 | 0.316 |
| PWY.5486 | pyruvate fermentation to eth | 0 | Not detected | -0.112 | -0.306 | 0.082 | 0.257 |
| PWY.5497 | purine nucleobases degradat | 0 | Not detected | -0.054 | -0.117 | 0.009 | 0.093 |
| PWY.5509 | adenosylcobalamin biosynth | 0 | Not detected | 0.049 | -0.114 | 0.213 | 0.554 |
| PWY.5512 | UDP-<i>N</i>-acetyl-D-gal | 0 | Not detected | -0.220 | -0.483 | 0.042 | 0.100 |
| PWY.5514 | UDP-<i>N</i>-acetyl-D-gal | 0 | Not detected | -0.039 | -0.110 | 0.031 | 0.277 |
| PWY.5523 | 5,6-dimethylbenzimidazole b | 0 | Not detected | -0.030 | -0.219 | 0.159 | 0.757 |
| PWY.5531 | 3,8-divinyl-chlorophyllide < | 0 | Not detected | 0.275 | 0.063 | 0.487 | 0.011 |
| PWY.5532 | nucleoside and nucleotide de | 0 | Not detected | 0.035 | -0.127 | 0.197 | 0.674 |
| PWY.5536 | acetate formation from acety | 0 | Not detected | -0.102 | -0.243 | 0.039 | 0.158 |
| PWY.561 | superpathway of glyoxylate | 0 | Not detected | -0.045 | -0.113 | 0.024 | 0.200 |
| PWY.5659 | GDP-mannose biosynthesis | 0 | Not detected | -0.028 | -0.093 | 0.036 | 0.386 |
| PWY.5661 | GDP-glucose biosynthesis | 0 | Not detected | 0.019 | -0.092 | 0.129 | 0.738 |
| PWY.5663 | erythro-tetrahydrobiopterin b | 0 | Not detected | 0.005 | -0.116 | 0.125 | 0.942 |
| PWY.5664 | <i>erythro</i>-tetrahydrobio | 0 | Not detected | 0.005 | -0.116 | 0.125 | 0.942 |
| PWY.5667 | CDP-diacylglycerol biosynth | 0 | Not detected | -0.006 | -0.178 | 0.167 | 0.947 |
| PWY.5668 | cardiolipin biosynthesis I | 0 | Not detected | 0.121 | -0.103 | 0.346 | 0.290 |
| PWY.5669 | phosphatidylserine and phos | 0 | Not detected | -0.016 | -0.211 | 0.179 | 0.873 |
| PWY.5676 | acetyl-CoA fermentation to b | 0 | Not detected | -0.022 | -0.101 | 0.057 | 0.590 |
| PWY.5677 | succinate fermentation to bu | 0 | Not detected | -0.055 | -0.138 | 0.028 | 0.194 |
| PWY.5686 | UMP biosynthesis I | 0 | Not detected | -0.014 | -0.083 | 0.056 | 0.701 |
| PWY.5690 | TCA cycle II (plants and fun | 0 | Not detected | -0.027 | -0.106 | 0.052 | 0.500 |
| PWY.5692 | allantoin degradation to glyo | 0 | Not detected | 0.096 | -0.093 | 0.285 | 0.320 |
| PWY.5698 | allantoin degradation to urei | 0 | Not detected | 0.096 | -0.093 | 0.285 | 0.320 |
| PWY.5723 | Rubisco shunt | 0 | Not detected | -0.050 | -0.120 | 0.020 | 0.159 |

| PWY.5738 | GDP-6-deoxy-D-talose bios | 0 | Not detected | -0.074 | -0.304 | 0.155 | 0.527 |
| --- | --- | --- | --- | --- | --- | --- | --- |
| PWY.5739 | GDP-D-perosamine biosynth | 0 | Not detected | -0.074 | -0.304 | 0.155 | 0.527 |
| PWY.5740 | GDP-L-colitose biosynthesis | 0 | Not detected | -0.074 | -0.304 | 0.155 | 0.527 |
| PWY.5741 | ethylmalonyl-CoA pathway | 0 | Not detected | 0.018 | -0.163 | 0.199 | 0.846 |
| PWY.5743 | 3-hydroxypropanoate cycle | 0 | Not detected | -0.085 | -0.175 | 0.004 | 0.061 |
| PWY.5744 | glyoxylate assimilation | 0 | Not detected | -0.086 | -0.176 | 0.003 | 0.058 |
| PWY.5747 | 2-methylcitrate cycle II | 0 | Not detected | -0.026 | -0.265 | 0.213 | 0.831 |
| PWY.5757 | fosfomycin biosynthesis | 0 | Not detected | -0.091 | -0.347 | 0.165 | 0.488 |
| PWY.5785 | di-<i>trans</i>,poly-<i>cis< | 0 | Not detected | 0.090 | -0.104 | 0.283 | 0.364 |
| PWY.5789 | 3-hydroxypropanoate/4-hydr | 0 | Not detected | -0.044 | -0.121 | 0.034 | 0.271 |
| PWY.5794 | malonate degradation I (biot | 0 | Not detected | -0.029 | -0.233 | 0.174 | 0.777 |
| PWY.5796 | malonate decarboxylase acti | 0 | Not detected | 0.150 | -0.011 | 0.311 | 0.068 |
| PWY.5837 | 2-carboxy-1,4-naphthoquino | 0 | Not detected | 0.012 | -0.062 | 0.085 | 0.750 |
| PWY.5852 | demethylmenaquinol-8 biosy | 0 | Not detected | 0.033 | -0.143 | 0.210 | 0.710 |
| PWY.5870 | ubiquinol-8 biosynthesis (eu | 0 | Not detected | 0.104 | -0.118 | 0.326 | 0.359 |
| PWY.5875 | staphyloxanthin biosynthesis | 0 | Not detected | 0.040 | -0.189 | 0.270 | 0.730 |
| PWY.5901 | 2,3-dihydroxybenzoate biosy | 0 | Not detected | -0.035 | -0.201 | 0.130 | 0.678 |
| PWY.5913 | partial TCA cycle (obligate a | 0 | Not detected | -0.028 | -0.106 | 0.049 | 0.475 |
| PWY.5921 | glutaminyl-tRNA<sup>gln< | 0 | Not detected | -0.043 | -0.278 | 0.192 | 0.721 |
| PWY.5940 | streptomycin biosynthesis | 0 | Not detected | 0.007 | -0.101 | 0.115 | 0.904 |
| PWY.5941 | glycogen degradation II | 0 | Not detected | 0.019 | -0.092 | 0.129 | 0.738 |
| PWY.5971 | palmitate biosynthesis II (ba | 0 | Not detected | -0.096 | -0.238 | 0.045 | 0.183 |
| PWY.5981 | CDP-diacylglycerol biosynth | 0 | Not detected | 0.100 | -0.069 | 0.269 | 0.248 |
| PWY.5994 | palmitate biosynthesis I (ani | 0 | Not detected | -0.126 | -0.285 | 0.033 | 0.120 |
| PWY.6012 | acyl carrier protein metaboli | 0 | Not detected | 0.011 | -0.145 | 0.167 | 0.890 |
| PWY.6019 | pseudouridine degradation | 0 | Not detected | 0.075 | -0.113 | 0.263 | 0.435 |
| PWY.6053 | dimethylsulfoniopropanoate | 0 | Not detected | 0.145 | -0.057 | 0.347 | 0.158 |
| PWY.6060 | malonate degradation II (bio | 0 | Not detected | -0.143 | -0.265 | -0.022 | 0.021 |
| PWY.6121 | 5-aminoimidazole ribonucle | 0 | Not detected | -0.008 | -0.087 | 0.072 | 0.846 |
| PWY.6122 | 5-aminoimidazole ribonucle | 0 | Not detected | -0.028 | -0.091 | 0.035 | 0.391 |
| PWY.6123 | inosine-5'-phosphate biosynt | 0 | Not detected | 0.082 | -0.046 | 0.210 | 0.209 |
| PWY.6124 | inosine-5'-phosphate biosynt | 0 | Not detected | 0.082 | -0.046 | 0.210 | 0.209 |
| PWY.6138 | CMP-<i>N</i>-acetylneura | 0 | Not detected | 0.150 | -0.026 | 0.325 | 0.094 |
| PWY.6140 | CMP-2-keto-3-deoxy-D-<i> | 0 | Not detected | -0.295 | -0.535 | -0.056 | 0.016 |
| PWY.6142 | gluconeogenesis II (<i>Meth | 0 | Not detected | -0.039 | -0.107 | 0.029 | 0.262 |
| PWY.6143 | CMP-pseudaminate biosynth | 0 | Not detected | -0.160 | -0.322 | 0.003 | 0.054 |
| PWY.6146 | <i>Methanobacterium therm | 0 | Not detected | -0.015 | -0.224 | 0.195 | 0.890 |
| PWY.6147 | 6-hydroxymethyl-dihydropte | 0 | Not detected | 0.002 | -0.100 | 0.103 | 0.973 |
| PWY.6148 | tetrahydromethanopterin bio | 0 | Not detected | 0.081 | -0.091 | 0.253 | 0.357 |
| PWY.6151 | <i>S</i>-adenosyl-L-methio | 0 | Not detected | 0.099 | -0.130 | 0.327 | 0.398 |
| PWY.6153 | autoinducer AI-2 biosynthes | 0 | Not detected | 0.096 | -0.074 | 0.267 | 0.269 |
| PWY.6154 | autoinducer AI-2 biosynthes | 0 | Not detected | 0.096 | -0.074 | 0.267 | 0.269 |
| PWY.6160 | 3-dehydroquinate biosynthes | 0 | Not detected | -0.083 | -0.290 | 0.125 | 0.435 |
| PWY.6163 | chorismate biosynthesis from | 0 | Not detected | 0.125 | -0.032 | 0.281 | 0.118 |

| PWY.6164 | 3-dehydroquinate biosynthes | 0 | Not detected | 0.032 | -0.172 | 0.236 | 0.759 |
| --- | --- | --- | --- | --- | --- | --- | --- |
| PWY.6167 | flavin biosynthesis II (archae | 0 | Not detected | -0.043 | -0.108 | 0.023 | 0.206 |
| PWY.6168 | flavin biosynthesis III (fungi | 0 | Not detected | -0.045 | -0.113 | 0.024 | 0.200 |
| PWY.6174 | mevalonate pathway II (arch | 0 | Not detected | 0.101 | -0.050 | 0.251 | 0.190 |
| PWY.621 | sucrose degradation III (sucr | 0 | Not detected | -0.031 | -0.097 | 0.034 | 0.346 |
| PWY.622 | starch biosynthesis | 0 | Not detected | -0.028 | -0.089 | 0.034 | 0.378 |
| PWY.6269 | superpathway of adenosylco | 0 | Not detected | 0.106 | -0.076 | 0.287 | 0.253 |
| PWY.6277 | superpathway of 5-aminoimi | 0 | Not detected | -0.028 | -0.091 | 0.035 | 0.391 |
| PWY.6317 | D-galactose degradation I (L | 0 | Not detected | -0.042 | -0.108 | 0.024 | 0.210 |
| PWY.6318 | L-phenylalanine degradation | 0 | Not detected | -0.117 | -0.225 | -0.008 | 0.036 |
| PWY.6322 | phosphinothricin tripeptide b | 0 | Not detected | -0.070 | -0.302 | 0.162 | 0.553 |
| PWY.6328 | L-lysine degradation X | 0 | Not detected | -0.062 | -0.234 | 0.110 | 0.482 |
| PWY.6333 | acetaldehyde biosynthesis I | 0 | Not detected | -0.112 | -0.306 | 0.082 | 0.257 |
| PWY.6383 | mono-<i>trans</i>, poly-<i> | 0 | Not detected | 0.136 | -0.067 | 0.338 | 0.189 |
| PWY.6386 | UDP-<i>N</i>-acetylmuram | 0 | Not detected | 0.044 | -0.016 | 0.105 | 0.154 |
| PWY.6387 | UDP-<i>N</i>-acetylmuram | 0 | Not detected | 0.045 | -0.012 | 0.102 | 0.123 |
| PWY.6397 | mycolyl-arabinogalactan-pep | 0 | Not detected | -0.096 | -0.175 | -0.016 | 0.019 |
| PWY.6406 | salicylate biosynthesis I | 0 | Not detected | -0.035 | -0.201 | 0.130 | 0.678 |
| PWY.6416 | quinate degradation II | 0 | Not detected | 0.112 | -0.083 | 0.307 | 0.260 |
| PWY.6461 | peptidoglycan cross-bridge b | 0 | Not detected | 0.071 | -0.168 | 0.311 | 0.559 |
| PWY.6471 | peptidoglycan biosynthesis I | 0 | Not detected | -0.117 | -0.236 | 0.002 | 0.053 |
| PWY.6473 | 4-aminobutanoate degradatio | 0 | Not detected | 0.106 | -0.059 | 0.272 | 0.209 |
| PWY.6478 | GDP-D-<i>glycero</i>-&al | 0 | Not detected | 0.046 | -0.151 | 0.242 | 0.649 |
| PWY.6497 | D-galactarate degradation II | 0 | Not detected | 0.005 | -0.202 | 0.213 | 0.960 |
| PWY.6499 | D-glucarate degradation II | 0 | Not detected | -0.098 | -0.303 | 0.106 | 0.347 |
| PWY.6507 | 4-deoxy-L-<i>threo</i>-hex | 0 | Not detected | -0.045 | -0.112 | 0.022 | 0.191 |
| PWY.6517 | <i>N</i>-acetylglucosamine | 0 | Not detected | -0.074 | -0.154 | 0.006 | 0.068 |
| PWY.6518 | glycocholate metabolism (ba | 0 | Not detected | -0.064 | -0.292 | 0.163 | 0.581 |
| PWY.6519 | 8-amino-7-oxononanoate bio | 0 | Not detected | 0.086 | -0.093 | 0.266 | 0.347 |
| PWY.6527 | stachyose degradation | 0 | Not detected | -0.028 | -0.105 | 0.050 | 0.483 |
| PWY.6531 | mannitol cycle | 0 | Not detected | 0.146 | -0.049 | 0.340 | 0.142 |
| PWY.6535 | 4-aminobutanoate degradatio | 0 | Not detected | 0.010 | -0.185 | 0.205 | 0.917 |
| PWY.6536 | 4-aminobutanoate degradatio | 0 | Not detected | -0.072 | -0.242 | 0.099 | 0.410 |
| PWY.6537 | 4-aminobutanoate degradatio | 0 | Not detected | -0.061 | -0.228 | 0.107 | 0.477 |
| PWY.6545 | pyrimidine deoxyribonucleo | 0 | Not detected | -0.069 | -0.149 | 0.011 | 0.093 |
| PWY.6549 | L-glutamine biosynthesis III | 0 | Not detected | -0.028 | -0.108 | 0.053 | 0.496 |
| PWY.6556 | pyrimidine ribonucleosides s | 0 | Not detected | 0.010 | -0.153 | 0.173 | 0.906 |
| PWY.6559 | spermidine biosynthesis II | 0 | Not detected | -0.036 | -0.112 | 0.040 | 0.348 |
| PWY.6562 | norspermidine biosynthesis | 0 | Not detected | -0.049 | -0.249 | 0.151 | 0.631 |
| PWY.6572 | chondroitin sulfate degradati | 0 | Not detected | -0.090 | -0.305 | 0.125 | 0.412 |
| PWY.6575 | juvenile hormone III biosynt | 0 | Not detected | 0.004 | -0.173 | 0.182 | 0.961 |
| PWY.6583 | pyruvate fermentation to but | 0 | Not detected | -0.017 | -0.099 | 0.066 | 0.694 |
| PWY.6587 | pyruvate fermentation to eth | 0 | Not detected | 0.175 | -0.060 | 0.410 | 0.145 |
| PWY.6596 | adenosine nucleotides degra | 0 | Not detected | 0.018 | -0.186 | 0.221 | 0.865 |

| PWY.66 | GDP-L-fucose biosynthesis I | 0 | Not detected | -0.074 | -0.304 | 0.155 | 0.527 |
| --- | --- | --- | --- | --- | --- | --- | --- |
| PWY.6605 | adenine and adenosine salva | 0 | Not detected | -0.056 | -0.266 | 0.154 | 0.599 |
| PWY.6606 | guanosine nucleotides degra | 0 | Not detected | 0.000 | -0.200 | 0.201 | 0.997 |
| PWY.6607 | guanosine nucleotides degra | 0 | Not detected | 0.014 | -0.171 | 0.200 | 0.879 |
| PWY.6608 | guanosine nucleotides degra | 0 | Not detected | -0.074 | -0.286 | 0.138 | 0.495 |
| PWY.6610 | adenine salvage | 0 | Not detected | -0.060 | -0.274 | 0.154 | 0.582 |
| PWY.6613 | tetrahydrofolate salvage from | 0 | Not detected | -0.122 | -0.218 | -0.025 | 0.013 |
| PWY.6614 | tetrahydrofolate biosynthesis | 0 | Not detected | -0.011 | -0.205 | 0.182 | 0.908 |
| PWY.6620 | guanine and guanosine salva | 0 | Not detected | -0.118 | -0.337 | 0.102 | 0.293 |
| PWY.6626 | cytidine-5'-diphosphate-glyc | 0 | Not detected | 0.137 | -0.058 | 0.331 | 0.168 |
| PWY.6637 | sulfolactate degradation II | 0 | Not detected | -0.043 | -0.118 | 0.033 | 0.269 |
| PWY.6654 | phosphopantothenate biosyn | 0 | Not detected | 0.055 | -0.108 | 0.218 | 0.508 |
| PWY.6672 | <i>cis</i>-genanyl-CoA deg | 0 | Not detected | 0.007 | -0.202 | 0.217 | 0.945 |
| PWY.6682 | dehydrophos biosynthesis | 0 | Not detected | -0.093 | -0.325 | 0.140 | 0.435 |
| PWY.6690 | cinnamate and 3-hydroxycin | 0 | Not detected | 0.055 | -0.132 | 0.243 | 0.564 |
| PWY.6700 | queuosine biosynthesis | 0 | Not detected | -0.033 | -0.141 | 0.074 | 0.543 |
| PWY.6703 | preQ<sub>0</sub> biosynth | 0 | Not detected | 0.022 | -0.082 | 0.126 | 0.677 |
| PWY.6707 | gallate biosynthesis | 0 | Not detected | 0.112 | -0.083 | 0.307 | 0.260 |
| PWY.6708 | ubiquinol-8 biosynthesis (pr | 0 | Not detected | 0.095 | -0.128 | 0.318 | 0.405 |
| PWY.6717 | (1,4)-&beta;-D-xylan degrad | 0 | Not detected | 0.062 | -0.115 | 0.238 | 0.493 |
| PWY.6724 | starch degradation II | 0 | Not detected | 0.196 | -0.017 | 0.409 | 0.072 |
| PWY.6728 | methylaspartate cycle | 0 | Not detected | -0.028 | -0.106 | 0.049 | 0.475 |
| PWY.6731 | starch degradation III | 0 | Not detected | 0.012 | -0.099 | 0.124 | 0.833 |
| PWY.6737 | starch degradation V | 0 | Not detected | 0.012 | -0.100 | 0.123 | 0.834 |
| PWY.6748 | nitrate reduction VII (denitri | 0 | Not detected | 0.011 | -0.205 | 0.227 | 0.920 |
| PWY.6749 | CMP-legionaminate biosynt | 0 | Not detected | 0.020 | -0.166 | 0.207 | 0.832 |
| PWY.6771 | rhamnogalacturonan type I d | 0 | Not detected | -0.105 | -0.248 | 0.038 | 0.150 |
| PWY.6772 | hydrogen production V | 0 | Not detected | -0.056 | -0.266 | 0.154 | 0.603 |
| PWY.6784 | cellulose and hemicellulose | 0 | Not detected | 0.062 | -0.116 | 0.240 | 0.494 |
| PWY.6786 | detoxification of reactive car | 0 | Not detected | -0.054 | -0.117 | 0.010 | 0.098 |
| PWY.6788 | cellulose degradation II (fun | 0 | Not detected | 0.120 | -0.074 | 0.314 | 0.225 |
| PWY.6799 | fatty acid biosynthesis (plant | 0 | Not detected | 0.183 | -0.020 | 0.386 | 0.078 |
| PWY.6808 | dTDP-D-forosamine biosynt | 0 | Not detected | -0.035 | -0.103 | 0.033 | 0.319 |
| PWY.6823 | molybdenum cofactor biosyn | 0 | Not detected | 0.165 | -0.043 | 0.374 | 0.120 |
| PWY.6839 | 2-aminoethylphosphonate bi | 0 | Not detected | -0.093 | -0.325 | 0.140 | 0.435 |
| PWY.6840 | homoglutathione biosynthesi | 0 | Not detected | 0.044 | -0.138 | 0.226 | 0.635 |
| PWY.6842 | glutathione-mediated detoxif | 0 | Not detected | 0.048 | -0.134 | 0.229 | 0.608 |
| PWY.6854 | ethylene biosynthesis III (mi | 0 | Not detected | 0.098 | -0.122 | 0.319 | 0.383 |
| PWY.6855 | chitin degradation I (archaea | 0 | Not detected | 0.094 | -0.100 | 0.288 | 0.344 |
| PWY.6859 | <i>all-trans</i>-farnesol bio | 0 | Not detected | 0.122 | -0.102 | 0.346 | 0.286 |
| PWY.6863 | pyruvate fermentation to hex | 0 | Not detected | -0.014 | -0.101 | 0.074 | 0.761 |
| PWY.6871 | 3-methylbutanol biosynthesi | 0 | Not detected | -0.104 | -0.215 | 0.007 | 0.065 |
| PWY.6883 | pyruvate fermentation to but | 0 | Not detected | -0.013 | -0.101 | 0.075 | 0.771 |
| PWY.6886 | 1-butanol autotrophic biosyn | 0 | Not detected | -0.048 | -0.112 | 0.015 | 0.137 |

| PWY.6890 | 4-amino-2-methyl-5-diphosp | 0 | Not detected | -0.014 | -0.096 | 0.068 | 0.732 |
| --- | --- | --- | --- | --- | --- | --- | --- |
| PWY.6891 | thiazole biosynthesis II (aero | 0 | Not detected | -0.067 | -0.138 | 0.003 | 0.062 |
| PWY.6892 | thiazole biosynthesis I (facul | 0 | Not detected | -0.056 | -0.116 | 0.003 | 0.063 |
| PWY.6893 | thiamine diphosphate biosyn | 0 | Not detected | 0.104 | -0.064 | 0.273 | 0.224 |
| PWY.6894 | thiamine diphosphate biosyn | 0 | Not detected | 0.104 | -0.079 | 0.288 | 0.265 |
| PWY.6896 | thiamine salvage I | 0 | Not detected | 0.162 | -0.022 | 0.346 | 0.085 |
| PWY.6897 | thiamine salvage II | 0 | Not detected | 0.104 | -0.079 | 0.288 | 0.265 |
| PWY.6901 | superpathway of glucose and | 0 | Not detected | -0.049 | -0.112 | 0.015 | 0.134 |
| PWY.6902 | chitin degradation II (Vibrio | 0 | Not detected | 0.076 | -0.117 | 0.268 | 0.441 |
| PWY.6906 | chitin derivatives degradatio | 0 | Not detected | -0.053 | -0.120 | 0.014 | 0.121 |
| PWY.6938 | NADH repair | 0 | Not detected | 0.108 | -0.081 | 0.298 | 0.263 |
| PWY.6942 | dTDP-D-desosamine biosyn | 0 | Not detected | -0.034 | -0.102 | 0.034 | 0.327 |
| PWY.6944 | androstenedione degradation | 0 | Not detected | 0.007 | -0.198 | 0.213 | 0.943 |
| PWY.6949 | DIBOA-glucoside biosynthe | 0 | Not detected | 0.025 | -0.128 | 0.179 | 0.746 |
| PWY.6952 | glycerophosphodiester degra | 0 | Not detected | 0.101 | -0.052 | 0.254 | 0.195 |
| PWY.6953 | dTDP-3-acetamido-&alpha;- | 0 | Not detected | -0.037 | -0.106 | 0.031 | 0.287 |
| PWY.6961 | L-ascorbate degradation II (b | 0 | Not detected | -0.016 | -0.079 | 0.046 | 0.607 |
| PWY.6969 | TCA cycle V (2-oxoglutarat | 0 | Not detected | -0.019 | -0.089 | 0.051 | 0.598 |
| PWY.6973 | dTDP-D-olivose, dTDP-D-o | 0 | Not detected | -0.035 | -0.103 | 0.033 | 0.319 |
| PWY.6974 | dTDP-L-olivose biosynthesi | 0 | Not detected | -0.035 | -0.103 | 0.033 | 0.319 |
| PWY.6976 | dTDP-L-mycarose biosynthe | 0 | Not detected | -0.034 | -0.102 | 0.033 | 0.322 |
| PWY.6981 | chitin biosynthesis | 0 | Not detected | -0.032 | -0.098 | 0.034 | 0.337 |
| PWY.6983 | <i>threo</i>-tetrahydrobiop | 0 | Not detected | 0.005 | -0.116 | 0.125 | 0.942 |
| PWY.6984 | lipoate salvage II | 0 | Not detected | 0.078 | -0.080 | 0.235 | 0.333 |
| PWY.6986 | alginate degradation | 0 | Not detected | -0.046 | -0.118 | 0.026 | 0.209 |
| PWY.6987 | lipoate biosynthesis and inco | 0 | Not detected | -0.219 | -0.406 | -0.032 | 0.022 |
| PWY.6992 | 1,5-anhydrofructose degrada | 0 | Not detected | -0.033 | -0.100 | 0.034 | 0.336 |
| PWY.7003 | glycerol degradation to buta | 0 | Not detected | -0.049 | -0.112 | 0.015 | 0.135 |
| PWY.7007 | methyl ketone biosynthesis ( | 0 | Not detected | 0.130 | -0.090 | 0.350 | 0.246 |
| PWY.7013 | (<i>S</i>)-propane-1,2-diol | 0 | Not detected | 0.205 | -0.009 | 0.419 | 0.060 |
| PWY.702 | L-methionine biosynthesis II | 0 | Not detected | 0.078 | -0.142 | 0.299 | 0.487 |
| PWY.7031 | protein <i>N</i>-glycosylati | 0 | Not detected | -0.197 | -0.416 | 0.022 | 0.078 |
| PWY.7037 | protein <i>O</i>-glycosylati | 0 | Not detected | -0.192 | -0.412 | 0.027 | 0.086 |
| PWY.7077 | <i>N</i>-acetyl-D-galactosa | 0 | Not detected | 0.160 | -0.047 | 0.366 | 0.130 |
| PWY.7085 | triethylamine degradation | 0 | Not detected | 0.199 | -0.026 | 0.423 | 0.083 |
| PWY.7090 | UDP-2,3-diacetamido-2,3-di | 0 | Not detected | -0.167 | -0.323 | -0.011 | 0.036 |
| PWY.7096 | triclosan resistance | 0 | Not detected | -0.099 | -0.333 | 0.136 | 0.409 |
| PWY.7102 | bisabolene biosynthesis (eng | 0 | Not detected | 0.122 | -0.102 | 0.346 | 0.286 |
| PWY.7104 | dTDP-L-megosamine biosyn | 0 | Not detected | -0.035 | -0.103 | 0.033 | 0.319 |
| PWY.7111 | pyruvate fermentation to iso | 0 | Not detected | -0.026 | -0.106 | 0.053 | 0.512 |
| PWY.7115 | C4 photosynthetic carbon as | 0 | Not detected | -0.130 | -0.369 | 0.109 | 0.285 |
| PWY.7117 | C4 photosynthetic carbon as | 0 | Not detected | -0.045 | -0.115 | 0.025 | 0.209 |
| PWY.7118 | chitin degradation to ethanol | 0 | Not detected | -0.007 | -0.242 | 0.228 | 0.953 |
| PWY.7124 | ethylene biosynthesis V (eng | 0 | Not detected | -0.033 | -0.102 | 0.036 | 0.345 |

| PWY.7127 | CDP-D-mannitol biosynthes | 0 | Not detected | 0.061 | -0.172 | 0.294 | 0.609 |
| --- | --- | --- | --- | --- | --- | --- | --- |
| PWY.7130 | L-glucose degradation | 0 | Not detected | 0.161 | -0.030 | 0.352 | 0.099 |
| PWY.7131 | CMP-legionaminate biosynt | 0 | Not detected | -0.156 | -0.364 | 0.051 | 0.140 |
| PWY.7141 | (3<i>S</i>)-linalool biosynt | 0 | Not detected | 0.063 | -0.128 | 0.253 | 0.518 |
| PWY.7153 | grixazone biosynthesis | 0 | Not detected | -0.066 | -0.274 | 0.141 | 0.533 |
| PWY.7159 | 3,8-divinyl-chlorophyllide < | 0 | Not detected | 0.146 | -0.064 | 0.356 | 0.173 |
| PWY.7165 | L-ascorbate biosynthesis VI | 0 | Not detected | 0.116 | -0.108 | 0.341 | 0.310 |
| PWY.7174 | <i>S</i>-methyl-5-thio-&al | 0 | Not detected | -0.061 | -0.291 | 0.169 | 0.602 |
| PWY.7176 | UTP and CTP <i>de novo</ | 0 | Not detected | -0.027 | -0.098 | 0.043 | 0.450 |
| PWY.7177 | UTP and CTP dephosphoryl | 0 | Not detected | -0.023 | -0.096 | 0.049 | 0.525 |
| PWY.7180 | 2'-deoxy-&alpha;-D-ribose 1 | 0 | Not detected | 0.094 | -0.113 | 0.301 | 0.375 |
| PWY.7182 | linalool biosynthesis I | 0 | Not detected | 0.063 | -0.128 | 0.253 | 0.518 |
| PWY.7184 | pyrimidine deoxyribonucleo | 0 | Not detected | -0.069 | -0.148 | 0.011 | 0.093 |
| PWY.7185 | UTP and CTP dephosphoryl | 0 | Not detected | -0.023 | -0.096 | 0.049 | 0.525 |
| PWY.7187 | pyrimidine deoxyribonucleo | 0 | Not detected | -0.065 | -0.142 | 0.012 | 0.100 |
| PWY.7193 | pyrimidine ribonucleosides s | 0 | Not detected | 0.103 | -0.129 | 0.335 | 0.385 |
| PWY.7195 | pyrimidine ribonucleosides s | 0 | Not detected | 0.010 | -0.153 | 0.173 | 0.906 |
| PWY.7197 | pyrimidine deoxyribonucleo | 0 | Not detected | -0.067 | -0.145 | 0.011 | 0.094 |
| PWY.7198 | pyrimidine deoxyribonucleo | 0 | Not detected | -0.072 | -0.153 | 0.010 | 0.085 |
| PWY.7204 | pyridoxal 5'-phosphate salva | 0 | Not detected | 0.091 | -0.111 | 0.293 | 0.377 |
| PWY.7205 | CMP phosphorylation | 0 | Not detected | -0.060 | -0.136 | 0.015 | 0.116 |
| PWY.7210 | pyrimidine deoxyribonucleo | 0 | Not detected | -0.072 | -0.153 | 0.010 | 0.085 |
| PWY.7216 | (R)- and (S)-3-hydroxybutan | 0 | Not detected | -0.040 | -0.166 | 0.087 | 0.542 |
| PWY.7218 | photosynthetic 3-hydroxybut | 0 | Not detected | -0.050 | -0.122 | 0.023 | 0.178 |
| PWY.7219 | adenosine ribonucleotides <i | 0 | Not detected | -0.068 | -0.148 | 0.013 | 0.099 |
| PWY.7221 | guanosine ribonucleotides <i | 0 | Not detected | -0.069 | -0.150 | 0.012 | 0.094 |
| PWY.7222 | guanosine deoxyribonucleoti | 0 | Not detected | -0.069 | -0.150 | 0.012 | 0.094 |
| PWY.7224 | purine deoxyribonucleosides | 0 | Not detected | -0.069 | -0.150 | 0.012 | 0.094 |
| PWY.7226 | guanosine deoxyribonucleoti | 0 | Not detected | -0.069 | -0.150 | 0.012 | 0.094 |
| PWY.7234 | inosine-5'-phosphate biosynt | 0 | Not detected | 0.063 | -0.055 | 0.182 | 0.293 |
| PWY.7237 | <i>myo</i>-, <i>chiro</i>- | 0 | Not detected | -0.054 | -0.250 | 0.143 | 0.592 |
| PWY.7238 | sucrose biosynthesis II | 0 | Not detected | -0.027 | -0.090 | 0.037 | 0.410 |
| PWY.7241 | <I>myo</I>-inositol degrada | 0 | Not detected | -0.028 | -0.225 | 0.169 | 0.781 |
| PWY.7242 | D-fructuronate degradation | 0 | Not detected | -0.067 | -0.127 | -0.006 | 0.031 |
| PWY.7247 | &beta;-D-glucuronide and D | 0 | Not detected | -0.036 | -0.106 | 0.034 | 0.310 |
| PWY.7248 | pectin degradation II | 0 | Not detected | -0.036 | -0.106 | 0.034 | 0.309 |
| PWY.7250 | [2Fe-2S] iron-sulfur cluster | 0 | Not detected | 0.112 | -0.086 | 0.311 | 0.268 |
| PWY.7254 | TCA cycle VII (acetate-prod | 0 | Not detected | -0.028 | -0.101 | 0.044 | 0.447 |
| PWY.7255 | ergothioneine biosynthesis I | 0 | Not detected | 0.044 | -0.138 | 0.226 | 0.635 |
| PWY.7268 | NAD/NADP-NADH/NADP | 0 | Not detected | 0.018 | -0.146 | 0.182 | 0.833 |
| PWY.7269 | NAD/NADP-NADH/NADP | 0 | Not detected | 0.117 | -0.060 | 0.295 | 0.195 |
| PWY.7279 | aerobic respiration II (cytoch | 0 | Not detected | 0.118 | -0.059 | 0.295 | 0.192 |
| PWY.7282 | 4-amino-2-methyl-5-diphosp | 0 | Not detected | -0.036 | -0.185 | 0.113 | 0.634 |
| PWY.7288 | fatty acid &beta;-oxidation ( | 0 | Not detected | 0.196 | -0.020 | 0.411 | 0.075 |

| PWY.7290 | <i>Escherichia coli</i> sero | 0 | Not detected | -0.215 | -0.451 | 0.021 | 0.075 |
| --- | --- | --- | --- | --- | --- | --- | --- |
| PWY.7294 | D-xylose degradation IV | 0 | Not detected | 0.222 | -0.006 | 0.449 | 0.056 |
| PWY.7295 | L-arabinose degradation IV | 0 | Not detected | 0.087 | -0.129 | 0.303 | 0.431 |
| PWY.7301 | dTDP-4-<i>O</i>-demethyl | 0 | Not detected | -0.040 | -0.109 | 0.029 | 0.256 |
| PWY.7312 | dTDP-D-&beta;-fucofuranos | 0 | Not detected | -0.034 | -0.102 | 0.033 | 0.318 |
| PWY.7315 | dTDP-<i>N</i>-acetylthom | 0 | Not detected | -0.032 | -0.097 | 0.033 | 0.337 |
| PWY.7316 | dTDP-<i>N</i>-acetylviosa | 0 | Not detected | -0.034 | -0.102 | 0.034 | 0.329 |
| PWY.7318 | dTDP-3-acetamido-3,6-dide | 0 | Not detected | -0.035 | -0.103 | 0.033 | 0.317 |
| PWY.7328 | superpathway of UDP-gluco | 0 | Not detected | -0.096 | -0.175 | -0.016 | 0.019 |
| PWY.7330 | UDP-<i>N</i>-acetyl-&beta | 0 | Not detected | -0.092 | -0.245 | 0.060 | 0.235 |
| PWY.7331 | UDP-<i>N</i>-acetyl-&beta | 0 | Not detected | -0.166 | -0.318 | -0.013 | 0.033 |
| PWY.7332 | superpathway of UDP-<I>N | 0 | Not detected | -0.162 | -0.324 | 0.000 | 0.051 |
| PWY.7335 | UDP-<i>N</i>-acetyl-&alph | 0 | Not detected | -0.016 | -0.129 | 0.097 | 0.778 |
| PWY.7336 | UDP-<i>N</i>-acetyl-&alph | 0 | Not detected | -0.156 | -0.320 | 0.008 | 0.062 |
| PWY.7342 | superpathway of nicotine bio | 0 | Not detected | 0.016 | -0.234 | 0.266 | 0.900 |
| PWY.7343 | UDP-&alpha;-D-glucose bio | 0 | Not detected | 0.010 | -0.064 | 0.084 | 0.786 |
| PWY.7344 | UDP-&alpha;-D-galactose b | 0 | Not detected | -0.077 | -0.205 | 0.051 | 0.239 |
| PWY.7346 | UDP-&alpha;-D-glucuronate | 0 | Not detected | -0.120 | -0.301 | 0.061 | 0.195 |
| PWY.7347 | sucrose biosynthesis III | 0 | Not detected | -0.039 | -0.113 | 0.035 | 0.304 |
| PWY.7377 | cob(II)yrinate <i>a,c</i>-dia | 0 | Not detected | -0.028 | -0.116 | 0.060 | 0.535 |
| PWY.7378 | aminopropanol phosphate bi | 0 | Not detected | 0.053 | -0.163 | 0.269 | 0.632 |
| PWY.7383 | anaerobic energy metabolism | 0 | Not detected | -0.057 | -0.291 | 0.177 | 0.633 |
| PWY.7384 | anaerobic energy metabolism | 0 | Not detected | -0.112 | -0.191 | -0.034 | 0.005 |
| PWY.7385 | 1,3-propanediol biosynthesis | 0 | Not detected | -0.034 | -0.100 | 0.032 | 0.309 |
| PWY.7388 | octanoyl-[acyl-carrier protei | 0 | Not detected | 0.162 | -0.041 | 0.365 | 0.117 |
| PWY.7391 | isoprene biosynthesis II (eng | 0 | Not detected | 0.101 | -0.050 | 0.251 | 0.190 |
| PWY.7395 | D-galactosamine and <i>N< | 0 | Not detected | 0.160 | -0.047 | 0.366 | 0.130 |
| PWY.7396 | butanol and isobutanol biosy | 0 | Not detected | -0.023 | -0.114 | 0.068 | 0.618 |
| PWY.7399 | methylphosphonate degradat | 0 | Not detected | 0.201 | -0.009 | 0.411 | 0.061 |
| PWY.7400 | L-arginine biosynthesis IV ( | 0 | Not detected | -0.055 | -0.167 | 0.056 | 0.330 |
| PWY.7401 | crotonate fermentation (to ac | 0 | Not detected | -0.051 | -0.169 | 0.067 | 0.396 |
| PWY.7409 | phospholipid remodeling (ph | 0 | Not detected | 0.101 | -0.052 | 0.254 | 0.195 |
| PWY.7410 | ipsdienol biosynthesis | 0 | Not detected | 0.063 | -0.128 | 0.253 | 0.518 |
| PWY.7413 | dTDP-6-deoxy-&alpha;-D-a | 0 | Not detected | -0.035 | -0.103 | 0.033 | 0.319 |
| PWY.7414 | dTDP-&alpha;-D-mycamino | 0 | Not detected | -0.035 | -0.103 | 0.033 | 0.317 |
| PWY.7419 | FR-900098 and FR-33289 a | 0 | Not detected | -0.093 | -0.325 | 0.140 | 0.435 |
| PWY.7425 | 2-chloroacrylate degradation | 0 | Not detected | 0.106 | -0.077 | 0.289 | 0.255 |
| PWY.7431 | aromatic biogenic amine deg | 0 | Not detected | 0.035 | -0.136 | 0.206 | 0.687 |
| PWY.7440 | dTDP-&beta;-L-4-<i>epi</i | 0 | Not detected | -0.035 | -0.103 | 0.033 | 0.319 |
| PWY.7442 | drosopterin and aurodrosopt | 0 | Not detected | 0.005 | -0.116 | 0.125 | 0.942 |
| PWY.7446 | sulfoquinovose degradation | 0 | Not detected | -0.024 | -0.257 | 0.210 | 0.842 |
| PWY.7456 | &beta;-(1,4)-mannan degrad | 0 | Not detected | -0.092 | -0.201 | 0.016 | 0.096 |
| PWY.7459 | kojibiose degradation | 0 | Not detected | 0.188 | -0.002 | 0.379 | 0.053 |
| PWY.7467 | 2-acetamido-4-amino-2,4,6-t | 0 | Not detected | -0.116 | -0.290 | 0.058 | 0.191 |

| PWY.7510 | rhizocticin A and B biosynth | 0 | Not detected | -0.093 | -0.325 | 0.140 | 0.435 |
| --- | --- | --- | --- | --- | --- | --- | --- |
| PWY.7524 | mevalonate pathway III (arc | 0 | Not detected | 0.101 | -0.050 | 0.251 | 0.190 |
| PWY.7530 | &beta;-D-galactosaminyl-(1 | 0 | Not detected | -0.213 | -0.450 | 0.023 | 0.077 |
| PWY.7539 | 6-hydroxymethyl-dihydropte | 0 | Not detected | -0.051 | -0.125 | 0.024 | 0.181 |
| PWY.7541 | 1,2-propanediol biosynthesis | 0 | Not detected | 0.088 | -0.148 | 0.323 | 0.465 |
| PWY.7552 | heme <i>b</i> biosynthesis | 0 | Not detected | 0.148 | -0.071 | 0.367 | 0.185 |
| PWY.7559 | glutathione degradation (DU | 0 | Not detected | 0.002 | -0.212 | 0.217 | 0.983 |
| PWY.7560 | methylerythritol phosphate p | 0 | Not detected | -0.026 | -0.117 | 0.064 | 0.569 |
| PWY.7562 | 3,6-anhydro-&alpha;-L-gala | 0 | Not detected | -0.043 | -0.110 | 0.024 | 0.213 |
| PWY.7564 | bacimethrin and bacimethrin | 0 | Not detected | -0.138 | -0.270 | -0.005 | 0.042 |
| PWY.7568 | L-galactose degradation | 0 | Not detected | -0.154 | -0.326 | 0.019 | 0.080 |
| PWY.7573 | GDP-mycosamine biosynthe | 0 | Not detected | -0.074 | -0.304 | 0.155 | 0.527 |
| PWY.7581 | <i>N</i>-acetylneuraminate | 237 | Not detected | -0.208 | -0.358 | -0.059 | 0.006 |
| PWY.7586 | &beta;-1,4-D-mannosyl-<i> | 0 | Not detected | 0.041 | -0.149 | 0.231 | 0.675 |
| PWY.7588 | ursodeoxycholate biosynthes | 0 | Not detected | 0.035 | -0.157 | 0.228 | 0.718 |
| PWY.7610 | GDP-6-deoxy-D-<i>altro</i | 0 | Not detected | -0.165 | -0.320 | -0.009 | 0.038 |
| PWY.7616 | methanol oxidation to carbo | 0 | Not detected | -0.057 | -0.267 | 0.153 | 0.593 |
| PWY.7622 | UDP-&alpha;-D-galactofura | 0 | Not detected | -0.088 | -0.168 | -0.008 | 0.032 |
| PWY.7645 | hyaluronan degradation | 0 | Not detected | -0.066 | -0.256 | 0.125 | 0.498 |
| PWY.7646 | dermatan sulfate degradation | 0 | Not detected | -0.083 | -0.272 | 0.106 | 0.389 |
| PWY.7651 | heparan sulfate degradation | 0 | Not detected | -0.198 | -0.468 | 0.071 | 0.149 |
| PWY.7657 | dTDP-&beta;-L-digitoxose b | 0 | Not detected | -0.035 | -0.103 | 0.033 | 0.319 |
| PWY.7659 | viridicatumtoxin biosynthesi | 0 | Not detected | 0.063 | -0.128 | 0.253 | 0.518 |
| PWY.7663 | gondoate biosynthesis (anaer | 0 | Not detected | -0.065 | -0.149 | 0.020 | 0.133 |
| PWY.7664 | oleate biosynthesis IV (anaer | 0 | Not detected | -0.063 | -0.148 | 0.022 | 0.147 |
| PWY.7674 | CMP-8-amino-3,8-dideoxy- | 0 | Not detected | -0.132 | -0.332 | 0.069 | 0.198 |
| PWY.7675 | Kdo transfer to lipid IV<sub | 0 | Not detected | 0.136 | -0.057 | 0.329 | 0.167 |
| PWY.7684 | &alpha;-diglucosyldiacylgly | 0 | Not detected | 0.053 | -0.143 | 0.249 | 0.595 |
| PWY.7688 | dTDP-D-ravidosamine and d | 0 | Not detected | -0.037 | -0.106 | 0.031 | 0.287 |
| PWY.7709 | (3<i>R</i>)-linalool biosynt | 0 | Not detected | 0.063 | -0.128 | 0.253 | 0.518 |
| PWY.7716 | penicillin G and penicillin V | 0 | Not detected | -0.117 | -0.225 | -0.008 | 0.036 |
| PWY.7719 | CMP-diacetamido-8-epilegio | 0 | Not detected | -0.139 | -0.281 | 0.003 | 0.056 |
| PWY.7721 | methyl phomopsenoate biosy | 0 | Not detected | 0.062 | -0.133 | 0.256 | 0.534 |
| PWY.7736 | stellatic acid biosynthesis | 0 | Not detected | 0.071 | -0.150 | 0.292 | 0.529 |
| PWY.7754 | bile acids degradation | 0 | Not detected | 0.219 | -0.056 | 0.494 | 0.119 |
| PWY.7755 | iso-bile acids biosynthesis I | 0 | Not detected | -0.063 | -0.320 | 0.193 | 0.629 |
| PWY.7756 | iso-bile acids biosynthesis II | 0 | Not detected | 0.021 | -0.139 | 0.182 | 0.794 |
| PWY.7766 | heme <i>b</i> biosynthesis | 0 | Not detected | 0.147 | -0.063 | 0.357 | 0.171 |
| PWY.7769 | phosalacine biosynthesis | 0 | Not detected | -0.070 | -0.302 | 0.162 | 0.553 |
| PWY.7778 | 2-methylpropene degradatio | 0 | Not detected | -0.039 | -0.166 | 0.088 | 0.546 |
| PWY.7779 | methyl <i>tert</i>-butyl eth | 0 | Not detected | -0.039 | -0.166 | 0.088 | 0.546 |
| PWY.7790 | UMP biosynthesis II | 0 | Not detected | -0.017 | -0.096 | 0.062 | 0.669 |
| PWY.7791 | UMP biosynthesis III | 0 | Not detected | -0.017 | -0.096 | 0.062 | 0.669 |
| PWY.7793 | dimethyl sulfide biosynthesi | 0 | Not detected | -0.058 | -0.257 | 0.141 | 0.568 |

| PWY.7805 | (aminomethyl)phosphonate | 0 | Not detected | 0.043 | -0.166 | 0.253 | 0.685 |
| --- | --- | --- | --- | --- | --- | --- | --- |
| PWY.7807 | glyphosate degradation III | 0 | Not detected | -0.109 | -0.310 | 0.092 | 0.289 |
| PWY.7808 | tetracycline resistance | 0 | Not detected | -0.233 | -0.552 | 0.085 | 0.152 |
| PWY.7814 | dTDP-L-daunosamine biosy | 0 | Not detected | -0.040 | -0.109 | 0.029 | 0.256 |
| PWY.7815 | poly(ribitol phosphate) wall | 0 | Not detected | 0.025 | -0.073 | 0.123 | 0.616 |
| PWY.7816 | poly(ribitol phosphate) wall | 0 | Not detected | 0.029 | -0.070 | 0.128 | 0.570 |
| PWY.7817 | type I lipoteichoic acid biosy | 0 | Not detected | 0.055 | -0.037 | 0.146 | 0.242 |
| PWY.7818 | type IV lipoteichoic acid bio | 0 | Not detected | 0.170 | 0.005 | 0.336 | 0.043 |
| PWY.7819 | poly(3-O-&beta;-D-glucopyr | 0 | Not detected | -0.016 | -0.129 | 0.097 | 0.777 |
| PWY.7820 | teichuronic acid biosynthesis | 0 | Not detected | -0.181 | -0.359 | -0.004 | 0.046 |
| PWY.7822 | chitin degradation III (Serrat | 0 | Not detected | 0.075 | -0.117 | 0.268 | 0.443 |
| PWY.7852 | 6-hydroxymethyl-dihydropte | 0 | Not detected | 0.011 | -0.095 | 0.118 | 0.834 |
| PWY.7854 | crotonyl-CoA/ethylmalonyl- | 0 | Not detected | 0.096 | -0.120 | 0.313 | 0.384 |
| PWY.7856 | heme <i>a</i> biosynthesis | 0 | Not detected | 0.084 | -0.078 | 0.246 | 0.307 |
| PWY.7858 | (5Z)-dodecenoate biosynthes | 0 | Not detected | -0.044 | -0.127 | 0.039 | 0.297 |
| PWY.7863 | roseoflavin biosynthesis | 0 | Not detected | 0.031 | -0.138 | 0.200 | 0.722 |
| PWY.7864 | 8-methylmenaquinone biosy | 0 | Not detected | -0.046 | -0.287 | 0.196 | 0.710 |
| PWY.7873 | D-erythronate degradation II | 0 | Not detected | 0.143 | -0.032 | 0.318 | 0.110 |
| PWY.7874 | L-threonate degradation | 0 | Not detected | 0.073 | -0.123 | 0.270 | 0.465 |
| PWY.7883 | anhydromuropeptides recycl | 0 | Not detected | -0.051 | -0.257 | 0.155 | 0.628 |
| PWY.7884 | lipoprotein posttranslational | 0 | Not detected | 0.165 | -0.069 | 0.399 | 0.168 |
| PWY.7892 | tRNA-uridine 2-thiolation (b | 0 | Not detected | 0.023 | -0.125 | 0.171 | 0.762 |
| PWY.7900 | glycogen biosynthesis III (fr | 0 | Not detected | -0.003 | -0.090 | 0.083 | 0.937 |
| PWY.7902 | glucosylglycerol biosynthesi | 0 | Not detected | -0.006 | -0.103 | 0.091 | 0.905 |
| PWY.7904 | tRNA-uridine 2-thiolation (t | 0 | Not detected | -0.193 | -0.386 | -0.001 | 0.049 |
| PWY.7909 | formaldehyde oxidation VII | 0 | Not detected | -0.125 | -0.231 | -0.019 | 0.021 |
| PWY.7928 | nickel cofactor biosynthesis | 0 | Not detected | -0.072 | -0.215 | 0.072 | 0.327 |
| PWY.7953 | UDP-<i>N</i>-acetylmuram | 0 | Not detected | 0.040 | -0.019 | 0.099 | 0.186 |
| PWY.7975 | adenosylcobalamin biosynth | 0 | Not detected | 0.049 | -0.114 | 0.213 | 0.554 |
| PWY.7977 | L-methionine biosynthesis I | 0 | Not detected | -0.035 | -0.107 | 0.038 | 0.346 |
| PWY.7978 | <i>N</i>-methylpyrrolidone | 0 | Not detected | 0.106 | -0.059 | 0.272 | 0.209 |
| PWY.7981 | &alpha;-dystroglycan glycos | 0 | Not detected | -0.028 | -0.258 | 0.202 | 0.810 |
| PWY.7991 | toxoflavin biosynthesis | 0 | Not detected | -0.046 | -0.115 | 0.024 | 0.197 |
| PWY.8004 | Entner-Doudoroff pathway I | 0 | Not detected | -0.040 | -0.104 | 0.025 | 0.232 |
| PWY.801 | homocysteine and cysteine i | 0 | Not detected | 0.128 | -0.094 | 0.349 | 0.258 |
| PWY.8010 | L-cysteine biosynthesis IX ( | 0 | Not detected | -0.003 | -0.094 | 0.088 | 0.956 |
| PWY.8012 | mupirocin biosynthesis | 0 | Not detected | 0.179 | -0.024 | 0.382 | 0.085 |
| PWY.8013 | UDP-<i>N</i>-acetyl-D-gal | 0 | Not detected | -0.039 | -0.113 | 0.035 | 0.304 |
| PWY.8015 | glycine degradation (Stickla | 0 | Not detected | -0.039 | -0.105 | 0.028 | 0.256 |
| PWY.8040 | ansatrienin biosynthesis | 0 | Not detected | 0.031 | -0.193 | 0.256 | 0.784 |
| PWY.882 | L-ascorbate biosynthesis I (L | 0 | Not detected | 0.061 | -0.129 | 0.250 | 0.529 |
| PWY.922 | mevalonate pathway I | 0 | Not detected | 0.101 | -0.050 | 0.251 | 0.190 |
| PWY.I9 | L-cysteine biosynthesis VI (f | 0 | Not detected | 0.127 | -0.097 | 0.351 | 0.266 |
| PWY0.1182 | trehalose degradation II (cyt | 0 | Not detected | 0.188 | -0.041 | 0.417 | 0.108 |

| PWY0.1221 | putrescine degradation II | 0 | Not detected | 0.186 | -0.006 | 0.378 | 0.058 |
| --- | --- | --- | --- | --- | --- | --- | --- |
| PWY0.1241 | ADP-L-<i>glycero</i>-&be | 0 | Not detected | 0.037 | -0.157 | 0.232 | 0.709 |
| PWY0.1261 | anhydromuropeptides recycl | 0 | Not detected | 0.029 | -0.026 | 0.084 | 0.305 |
| PWY0.1264 | biotin-carboxyl carrier protei | 0 | Not detected | -0.100 | -0.183 | -0.016 | 0.020 |
| PWY0.1275 | lipoate biosynthesis and inco | 0 | Not detected | -0.147 | -0.310 | 0.016 | 0.078 |
| PWY0.1296 | purine ribonucleosides degra | 0 | Not detected | -0.027 | -0.208 | 0.154 | 0.768 |
| PWY0.1300 | 2-<I>O</I>-&alpha;-manno | 0 | Not detected | 0.005 | -0.218 | 0.227 | 0.967 |
| PWY0.1306 | L-galactonate degradation | 0 | Not detected | -0.082 | -0.227 | 0.064 | 0.271 |
| PWY0.1312 | acetate and ATP formation f | 0 | Not detected | -0.031 | -0.093 | 0.030 | 0.316 |
| PWY0.1313 | acetate conversion to acetyl- | 0 | Not detected | 0.007 | -0.202 | 0.217 | 0.945 |
| PWY0.1317 | L-lactaldehyde degradation ( | 0 | Not detected | 0.127 | -0.065 | 0.319 | 0.195 |
| PWY0.1319 | CDP-diacylglycerol biosynth | 0 | Not detected | 0.017 | -0.149 | 0.182 | 0.844 |
| PWY0.1324 | <i>N</i>-acetylneuraminate | 274 | Not detected | -0.193 | -0.324 | -0.061 | 0.004 |
| PWY0.1337 | oleate &beta;-oxidation | 0 | Not detected | 0.090 | -0.106 | 0.287 | 0.367 |
| PWY0.1338 | polymyxin resistance | 0 | Not detected | 0.100 | -0.112 | 0.312 | 0.354 |
| PWY0.1415 | superpathway of heme <i>b< | 0 | Not detected | 0.173 | -0.038 | 0.384 | 0.108 |
| PWY0.1433 | tetrahydromonapterin biosyn | 0 | Not detected | 0.008 | -0.098 | 0.114 | 0.887 |
| PWY0.1465 | D-malate degradation | 0 | Not detected | -0.147 | -0.345 | 0.051 | 0.146 |
| PWY0.1466 | trehalose degradation VI (pe | 0 | Not detected | 0.209 | -0.002 | 0.421 | 0.052 |
| PWY0.1471 | uracil degradation III | 0 | Not detected | 0.064 | -0.124 | 0.252 | 0.504 |
| PWY0.1477 | ethanolamine utilization | 0 | Not detected | -0.031 | -0.088 | 0.026 | 0.286 |
| PWY0.1479 | tRNA processing | 0 | Not detected | 0.076 | -0.144 | 0.297 | 0.498 |
| PWY0.1533 | methylphosphonate degradat | 0 | Not detected | 0.184 | -0.022 | 0.390 | 0.079 |
| PWY0.1545 | cardiolipin biosynthesis III | 0 | Not detected | 0.084 | -0.126 | 0.294 | 0.431 |
| PWY0.1546 | muropeptide degradation | 0 | Not detected | 0.142 | -0.039 | 0.323 | 0.124 |
| PWY0.1565 | D-lactate to cytochrome <i> | 0 | Not detected | 0.106 | -0.077 | 0.289 | 0.255 |
| PWY0.1567 | NADH to cytochrome <i>bo | 0 | Not detected | 0.118 | -0.059 | 0.295 | 0.192 |
| PWY0.1568 | NADH to cytochrome <i>bd | 0 | Not detected | 0.118 | -0.059 | 0.295 | 0.192 |
| PWY0.1569 | autoinducer AI-2 degradatio | 0 | Not detected | 0.163 | -0.021 | 0.346 | 0.082 |
| PWY0.1573 | nitrate reduction VIIIb (dissi | 0 | Not detected | 0.118 | -0.059 | 0.295 | 0.192 |
| PWY0.1585 | formate to nitrite electron tra | 0 | Not detected | 0.058 | -0.137 | 0.253 | 0.560 |
| PWY0.1586 | peptidoglycan maturation (< | 0 | Not detected | 0.165 | -0.095 | 0.426 | 0.214 |
| PWY0.1590 | NADH to hydrogen peroxide | 0 | Not detected | 0.007 | -0.193 | 0.206 | 0.948 |
| PWY0.1591 | glycerol-3-phosphate to hydr | 0 | Not detected | 0.007 | -0.193 | 0.206 | 0.948 |
| PWY0.1597 | prenylated FMNH<sub>2</s | 0 | Not detected | -0.001 | -0.157 | 0.156 | 0.994 |
| PWY0.166 | superpathway of pyrimidine | 0 | Not detected | -0.069 | -0.149 | 0.010 | 0.088 |
| PWY0.301 | L-ascorbate degradation I (b | 0 | Not detected | -0.016 | -0.079 | 0.046 | 0.607 |
| PWY0.321 | phenylacetate degradation I ( | 0 | Not detected | -0.105 | -0.200 | -0.010 | 0.031 |
| PWY0.42 | 2-methylcitrate cycle I | 0 | Not detected | -0.014 | -0.257 | 0.229 | 0.911 |
| PWY0.43 | conversion of succinate to pr | 0 | Not detected | -0.118 | -0.249 | 0.013 | 0.078 |
| PWY0.44 | D-allose degradation | 0 | Not detected | 0.056 | -0.177 | 0.289 | 0.636 |
| PWY0.461 | L-lysine degradation I | 0 | Not detected | 0.000 | -0.201 | 0.201 | 0.999 |
| PWY0.501 | lipoate biosynthesis and inco | 0 | Not detected | -0.199 | -0.363 | -0.035 | 0.017 |
| PWY0.522 | lipoate salvage I | 0 | Not detected | 0.088 | -0.094 | 0.270 | 0.343 |

| PWY0.662 | PRPP biosynthesis | 0 | Not detected | -0.045 | -0.134 | 0.045 | 0.328 |
| --- | --- | --- | --- | --- | --- | --- | --- |
| PWY0.862 | (5Z)-dodecenoate biosynthes | 0 | Not detected | -0.047 | -0.130 | 0.036 | 0.265 |
| PWY1.3 | polyhydroxybutanoate biosy | 0 | Not detected | 0.086 | -0.079 | 0.252 | 0.307 |
| PWY3O.4107 | NAD salvage pathway V (PN | 0 | Not detected | 0.082 | -0.111 | 0.274 | 0.406 |
| PWY4FS.7 | phosphatidylglycerol biosyn | 0 | Not detected | 0.079 | -0.116 | 0.274 | 0.430 |
| PWY4FS.8 | phosphatidylglycerol biosyn | 0 | Not detected | 0.079 | -0.116 | 0.274 | 0.430 |
| PWY4LZ.257 | superpathway of fermentatio | 0 | Not detected | -0.031 | -0.089 | 0.026 | 0.284 |
| PWY66.161 | ethanol degradation III | 0 | Not detected | 0.010 | -0.200 | 0.220 | 0.927 |
| PWY66.162 | ethanol degradation IV | 0 | Not detected | 0.010 | -0.200 | 0.220 | 0.927 |
| PWY66.21 | ethanol degradation II | 0 | Not detected | 0.011 | -0.211 | 0.233 | 0.925 |
| PWY66.366 | flavin biosynthesis IV (mam | 0 | Not detected | 0.033 | -0.164 | 0.229 | 0.744 |
| PWY66.391 | fatty acid &beta;-oxidation V | 0 | Not detected | 0.216 | -0.030 | 0.461 | 0.085 |
| PWY66.398 | TCA cycle III (animals) | 0 | Not detected | -0.027 | -0.106 | 0.052 | 0.501 |
| PWY66.399 | gluconeogenesis III | 0 | Not detected | -0.041 | -0.109 | 0.027 | 0.242 |
| PWY66.422 | D-galactose degradation V ( | 0 | Not detected | -0.042 | -0.108 | 0.024 | 0.210 |
| PWY66.426 | hydrogen sulfide biosynthesi | 0 | Not detected | 0.097 | -0.094 | 0.287 | 0.321 |
| PYRIDNUCSAL.P | NAD salvage pathway I (PN | 0 | Not detected | 0.067 | -0.129 | 0.262 | 0.504 |
| PYRIDNUCSYN.P | NAD <i>de novo</i> biosyn | 0 | Not detected | 0.049 | -0.203 | 0.301 | 0.705 |
| PYRIDOXSYN.PW | pyridoxal 5'-phosphate biosy | 0 | Not detected | 0.004 | -0.247 | 0.254 | 0.978 |
| PYRUVDEH.RXN | NA | 0 | Not detected | 0.182 | -0.027 | 0.390 | 0.088 |
| PYRUVDEHYD.P | pyruvate decarboxylation to | 0 | Not detected | 0.182 | -0.027 | 0.390 | 0.088 |
| QUINATEDEG.PW | quinate degradation I | 0 | Not detected | 0.104 | -0.081 | 0.289 | 0.269 |
| REDCITCYC | TCA cycle VIII (Helicobacte | 0 | Not detected | -0.029 | -0.106 | 0.049 | 0.471 |
| RHAMCAT.PWY | L-rhamnose degradation I | 0 | Not detected | 0.142 | -0.047 | 0.330 | 0.141 |
| RIBOSYN2.PWY | flavin biosynthesis I (bacteri | 0 | Not detected | -0.044 | -0.112 | 0.024 | 0.204 |
| RUMP.PWY | formaldehyde oxidation I | 0 | Not detected | -0.031 | -0.097 | 0.035 | 0.354 |
| RXN.12588 | NA | 0 | Not detected | 0.204 | -0.016 | 0.424 | 0.069 |
| RXN.13163 | NA | 0 | Not detected | -0.195 | -0.445 | 0.054 | 0.125 |
| RXN.14047 | NA | 0 | Not detected | -0.029 | -0.112 | 0.055 | 0.502 |
| RXN.14201 | NA | 0 | Not detected | -0.028 | -0.081 | 0.025 | 0.301 |
| RXN.14249 | NA | 0 | Not detected | 0.053 | -0.163 | 0.269 | 0.632 |
| RXN.14314 | NA | 0 | Not detected | -0.039 | -0.252 | 0.173 | 0.717 |
| RXN.17127 | NA | 0 | Not detected | 0.088 | -0.094 | 0.270 | 0.343 |
| RXN.20025 | NA | 0 | Not detected | 0.049 | -0.121 | 0.218 | 0.573 |
| RXN0.1141 | NA | 0 | Not detected | 0.077 | -0.080 | 0.234 | 0.338 |
| RXN0.6511 | NA | 0 | Not detected | 0.030 | -0.171 | 0.231 | 0.768 |
| SERSYN.PWY | L-serine biosynthesis I | 0 | Not detected | 0.003 | -0.076 | 0.082 | 0.942 |
| SUCROSEUTIL2.P | sucrose degradation VII (suc | 0 | Not detected | 0.138 | -0.071 | 0.348 | 0.195 |
| SUCSYN.PWY | sucrose biosynthesis I (from | 0 | Not detected | -0.039 | -0.107 | 0.030 | 0.269 |
| SUCUTIL.PWY | sucrose degradation I (sucro | 0 | Not detected | 0.142 | -0.062 | 0.346 | 0.172 |
| SULFMETII.PWY | assimilatory sulfate reductio | 0 | Not detected | 0.154 | -0.091 | 0.400 | 0.218 |
| TCA | TCA cycle I (prokaryotic) | 0 | Not detected | -0.024 | -0.098 | 0.049 | 0.515 |
| TEICHOICACID.P | poly(glycerol phosphate) wa | 0 | Not detected | 0.025 | -0.078 | 0.128 | 0.633 |
| THRDLCTCAT.PW | L-threonine degradation III ( | 0 | Not detected | 0.053 | -0.163 | 0.269 | 0.632 |

| THREONINE.DEG | L-threonine degradation II | 0 | Not detected | 0.053 | -0.163 | 0.269 | 0.632 |
| --- | --- | --- | --- | --- | --- | --- | --- |
| TREDEGLOW.PW | trehalose degradation I (low | 0 | Not detected | -0.080 | -0.165 | 0.006 | 0.067 |
| TRESYN.PWY | trehalose biosynthesis I | 0 | Not detected | 0.103 | -0.081 | 0.287 | 0.271 |
| TRIGLSYN.PWY | diacylglycerol and triacylgly | 0 | Not detected | -0.014 | -0.244 | 0.215 | 0.902 |
| TRNA.CHARGING | tRNA charging | 0 | Not detected | -0.021 | -0.071 | 0.029 | 0.414 |
| TRPSYN.PWY | L-tryptophan biosynthesis | 0 | Not detected | 0.141 | -0.093 | 0.376 | 0.238 |
| TRYPSYN.RXN | NA | 0 | Not detected | 0.101 | -0.106 | 0.309 | 0.338 |
| UDPNACETYLGA | UDP-<i>N</i>-acetyl-D-glu | 0 | Not detected | -0.034 | -0.104 | 0.036 | 0.343 |
| UDPNAGSYN.PW | UDP-<i>N</i>-acetyl-D-glu | 0 | Not detected | -0.033 | -0.101 | 0.035 | 0.339 |
| VALSYN.PWY | L-valine biosynthesis | 0 | Not detected | -0.026 | -0.106 | 0.053 | 0.512 |
| X1.1.1.39.RXN | NA | 0 | Not detected | -0.130 | -0.369 | 0.109 | 0.285 |
| X1CMET2.PWY | <i>N</i><sup>10</sup>-for | 0 | Not detected | -0.054 | -0.122 | 0.014 | 0.120 |
| X3.HYDROXYPH | 4-hydroxyphenylacetate degr | 0 | Not detected | -0.005 | -0.203 | 0.192 | 0.959 |

| **Supplementary Table 17.** Association of current education with enzymatic reactions in overall participants (n=2273). Analysis of Compositions of Microbiomes (ANCOM2) was used to identify enzymatic reactions related to current education. Regression coefficients were obtained by modeling the effect of current education on centered log ratio (CLR)-transformed species in multivariable linear regression adjusted for age, gender, BMI, field center, Hispanic/Latino background, U.S. nativity, Bristol stool type, parental education, the alternative Healthy Eating Index 2010 score, cigarette smoking, alcohol use, depressive and anxiety symptoms, diabetes, hypertension, cardiovascular disease, dyslipidemia, and antibiotic use. | | | | | | | |
| --- | --- | --- | --- | --- | --- | --- | --- |
| **otu.names** | **V2** | **W_stat** | **ANCOM** | **beta** | **l95ci** | **u95ci** | **qval** |
| ACETATEKI | acetate kinase | 0 | Not detected | 0.164 | -0.033 | 0.361 | 0.102 |
| ACS.ENZRX | acetyl-CoA synthetase (AMP-formin | 0 | Not detected | -0.024 | -0.255 | 0.208 | 0.841 |
| ACYLACPS | acyl-ACP synthetase | 0 | Not detected | 0.093 | -0.116 | 0.302 | 0.381 |
| ACYLCOAD | acyl-CoA dehydrogenase | 0 | Not detected | 0.140 | -0.075 | 0.355 | 0.203 |
| ACYLGPEA | 2-acylglycerophosphoethanolamine | 0 | Not detected | 0.093 | -0.116 | 0.302 | 0.381 |
| ADENYL.KI | adenylate kinase | 0 | Not detected | -0.099 | -0.218 | 0.020 | 0.104 |
| ADENYLAT | adenylate cyclase | 0 | Not detected | 0.206 | -0.023 | 0.436 | 0.078 |
| ADOMET.D | S-adenosylmethionine:2-demethylqu | 0 | Not detected | -0.056 | -0.307 | 0.195 | 0.660 |
| AICARTRAN | AICAR transformylase | 0 | Not detected | 0.054 | -0.118 | 0.226 | 0.541 |
| ALDOSE1EP | galactose-1-epimerase | 0 | Not detected | 0.098 | -0.082 | 0.279 | 0.286 |
| ALPHA.AM | a-amylase | 0 | Not detected | 0.116 | -0.061 | 0.292 | 0.199 |
| ALPHA.AM | a-amylase | 0 | Not detected | 0.163 | -0.044 | 0.371 | 0.123 |
| ALTRO.OXI | tagaturonate reductase | 0 | Not detected | 0.087 | -0.098 | 0.271 | 0.357 |
| ALTRODEH | D-altronate dehydratase | 0 | Not detected | -0.069 | -0.183 | 0.045 | 0.235 |
| AMYLOMA | amylomaltase | 0 | Not detected | 0.089 | -0.133 | 0.310 | 0.432 |
| ARGS.ENZR | arginine&mdash;tRNA ligase | 0 | Not detected | 0.007 | -0.227 | 0.242 | 0.952 |
| ARGSUCCIN | argininosuccinate lyase | 0 | Not detected | -0.088 | -0.226 | 0.051 | 0.216 |
| ARGSUCCT | arginine N-succinyltransferase | 0 | Not detected | -0.029 | -0.193 | 0.135 | 0.732 |
| BIOTINLIG. | biotin-[acetyl-CoA-carboxylase] liga | 0 | Not detected | 0.178 | 0.004 | 0.352 | 0.046 |
| CARDIOLIP | cardiolipin synthase | 0 | Not detected | 0.116 | -0.057 | 0.289 | 0.189 |
| CDPDIGLYP | CDP-diacylglycerol diphosphatase | 0 | Not detected | 0.076 | -0.079 | 0.232 | 0.335 |
| CDPDIGLYS | CTP:2,3,4-saturated L-phosphatidate | 0 | Not detected | 0.118 | -0.036 | 0.271 | 0.133 |
| CHD.ENZRX | choline dehydrogenase | 0 | Not detected | 0.200 | -0.005 | 0.404 | 0.056 |
| CMPKI.ENZ | CMP kinase | 0 | Not detected | 0.163 | -0.055 | 0.381 | 0.143 |
| CPM.KDOSY | 3-deoxy-manno-octulosonate cytidyl | 0 | Not detected | 0.060 | -0.195 | 0.314 | 0.646 |
| CROBETRE | crotonobetainyl-CoA reductase | 0 | Not detected | -0.002 | -0.214 | 0.210 | 0.988 |
| CYCPHOSD | 2',3'-cyclic nucleotide 2'-phosphodie | 0 | Not detected | 0.029 | -0.201 | 0.259 | 0.806 |
| CYSS.ENZR | cysteine&mdash;tRNA ligase | 0 | Not detected | -0.057 | -0.304 | 0.190 | 0.650 |
| CYTIDINEK | cytidine kinase | 0 | Not detected | 0.077 | -0.168 | 0.323 | 0.537 |
| D.PPENTOM | deoxyribose 1,5-phosphomutase | 0 | Not detected | -0.028 | -0.297 | 0.240 | 0.835 |
| DALADALA | D-alanine&mdash;D-alanine ligase | 0 | Not detected | 0.164 | -0.005 | 0.334 | 0.057 |
| DARABKIN. | D-ribulokinase | 0 | Not detected | 0.000 | -0.260 | 0.260 | 0.999 |
| DEHYDDEO | 2-dehydro-3-deoxygalactonokinase | 0 | Not detected | 0.098 | -0.081 | 0.278 | 0.283 |
| DEOXYGLU | 2-dehydro-3-deoxygluconokinase | 0 | Not detected | 0.049 | -0.123 | 0.221 | 0.576 |
| DEOXYRIB | deoxyribose-phosphate aldolase | 0 | Not detected | 0.104 | -0.054 | 0.263 | 0.197 |
| DIENOYLCO | 2,4-dienoyl-CoA reductase | 0 | Not detected | 0.147 | -0.067 | 0.361 | 0.178 |
| DIHYDROF | dihydrofolate synthetase | 0 | Not detected | -0.030 | -0.217 | 0.157 | 0.755 |

| DIHYDROO | dihydroorotate dehydrogenase | 0 | Not detected | -0.037 | -0.314 | 0.240 | 0.795 |
| --- | --- | --- | --- | --- | --- | --- | --- |
| DLACTDEH | D-lactate dehydrogenase (quionone) | 0 | Not detected | 0.092 | -0.098 | 0.283 | 0.341 |
| DMK.ENZR | 1,4-dihydroxy-2-naphthoate octapre | 0 | Not detected | 0.012 | -0.176 | 0.201 | 0.897 |
| DSERDEAM | D-serine ammonia-lyase | 0 | Not detected | 0.003 | -0.218 | 0.224 | 0.978 |
| DTDPDEHY | dTDP-4-dehydrorhamnose 3,5-epim | 0 | Not detected | -0.067 | -0.194 | 0.060 | 0.302 |
| DTDPGLUC | dTDP-glucose 4,6-dehydratase | 0 | Not detected | -0.049 | -0.174 | 0.077 | 0.447 |
| ENTC.ENZR | isochorismate synthase | 0 | Not detected | -0.080 | -0.251 | 0.091 | 0.358 |
| ENZRXN.10 | L-cysteine-S-conjugate thiol-lyase (d | 0 | Not detected | 0.047 | -0.177 | 0.272 | 0.678 |
| ENZRXN.11 | imidazolone-5-propionate hydrolase | 0 | Not detected | -0.020 | -0.262 | 0.221 | 0.869 |
| ENZRXN.11 | ammonia-dependent carbamoyl-phos | 0 | Not detected | 0.015 | -0.155 | 0.186 | 0.859 |
| ENZRXN.11 | cystathionine &gamma;-lyase | 0 | Not detected | 0.146 | -0.118 | 0.410 | 0.279 |
| ENZRXN.12 | citrate synthase | 0 | Not detected | 0.027 | -0.278 | 0.333 | 0.861 |
| ENZRXN.12 | citrate hydro-lyase (cis-aconitate-for | 0 | Not detected | 0.085 | -0.199 | 0.370 | 0.556 |
| ENZRXN.12 | D-threo-isocitrate hydro-lyase (cis-a | 0 | Not detected | 0.085 | -0.199 | 0.370 | 0.556 |
| ENZRXN.12 | malate dehydrogenase | 0 | Not detected | -0.166 | -0.457 | 0.125 | 0.263 |
| ENZRXN.12 | 1-deoxy-D-xylulose 5-phosphate red | 0 | Not detected | -0.044 | -0.190 | 0.101 | 0.550 |
| ENZRXN.12 | Pfl-activating enzyme | 0 | Not detected | -0.253 | -0.505 | -0.001 | 0.049 |
| ENZRXN.12 | phenylacetate-CoA ligase | 0 | Not detected | -0.145 | -0.286 | -0.004 | 0.044 |
| ENZRXN.12 | xylitol dehydrogenase | 0 | Not detected | 0.250 | -0.034 | 0.534 | 0.084 |
| ENZRXN.12 | UDP-N-acetylenolpyruvoylglucosam | 0 | Not detected | 0.051 | -0.179 | 0.281 | 0.663 |
| ENZRXN.12 | UDP-N-acetylmuramoyl-L-alanyl-D | 0 | Not detected | -0.118 | -0.413 | 0.177 | 0.434 |
| ENZRXN.12 | 3,5-diaminohexanoate dehydrogenas | 0 | Not detected | -0.100 | -0.346 | 0.146 | 0.424 |
| ENZRXN.12 | 3-aminobutyryl-CoA deaminase | 0 | Not detected | -0.152 | -0.420 | 0.116 | 0.267 |
| ENZRXN.13 | thiosulfate reductase (cytochrome) | 0 | Not detected | 0.038 | -0.213 | 0.289 | 0.766 |
| ENZRXN.13 | Enzyme IIscr | 0 | Not detected | -0.174 | -0.448 | 0.099 | 0.211 |
| ENZRXN.13 | Enzyme IIscr | 0 | Not detected | 0.183 | -0.031 | 0.397 | 0.094 |
| ENZRXN.13 | fructokinase | 0 | Not detected | 0.052 | -0.123 | 0.226 | 0.563 |
| ENZRXN.13 | phosphoribosyl-formimino-5-aminoi | 0 | Not detected | 0.015 | -0.137 | 0.167 | 0.847 |
| ENZRXN.13 | imidazoleglycerolphosphate dehydra | 0 | Not detected | -0.190 | -0.409 | 0.030 | 0.090 |
| ENZRXN.13 | sucrose phosphorylase | 0 | Not detected | 0.215 | -0.090 | 0.520 | 0.168 |
| ENZRXN.13 | phosphoserine aminotransferase | 0 | Not detected | -0.027 | -0.157 | 0.104 | 0.687 |
| ENZRXN.13 | phosphoglucose isomerase | 0 | Not detected | -0.086 | -0.354 | 0.183 | 0.532 |
| ENZRXN.13 | 3-phosphoglycerate kinase | 0 | Not detected | -0.082 | -0.217 | 0.052 | 0.231 |
| ENZRXN.13 | myo-inosose-2 dehydratase | 0 | Not detected | 0.064 | -0.155 | 0.283 | 0.566 |
| ENZRXN.13 | glucose-6-phosphate isomerase | 0 | Not detected | 0.083 | -0.189 | 0.355 | 0.550 |
| ENZRXN.13 | fructokinase | 0 | Not detected | -0.148 | -0.378 | 0.082 | 0.207 |
| ENZRXN.13 | glucose-6-phosphate isomerase | 0 | Not detected | -0.064 | -0.183 | 0.056 | 0.298 |
| ENZRXN.13 | phosphotransacetylase | 0 | Not detected | 0.092 | -0.107 | 0.292 | 0.364 |
| ENZRXN.14 | UDP-N-acetylglucosamine 4-epimer | 0 | Not detected | -0.254 | -0.569 | 0.060 | 0.113 |
| ENZRXN.14 | UDP-galactose 4-epimerase | 0 | Not detected | -0.254 | -0.569 | 0.060 | 0.113 |
| ENZRXN.14 | cobalt precorrin-4 (C11)-methyltrans | 0 | Not detected | -0.065 | -0.203 | 0.073 | 0.357 |
| ENZRXN.14 | cobalt-precorrin-3 (C17)-methyltran | 0 | Not detected | -0.060 | -0.322 | 0.201 | 0.650 |
| ENZRXN.14 | GDP-D-mannose pyrophosphorylase | 0 | Not detected | -0.073 | -0.308 | 0.163 | 0.545 |
| ENZRXN.14 | phosphomannose isomerase | 0 | Not detected | -0.073 | -0.308 | 0.163 | 0.545 |

| ENZRXN.14 | succinyl-CoA:CoA transferase | 0 | Not detected | -0.187 | -0.400 | 0.026 | 0.086 |
| --- | --- | --- | --- | --- | --- | --- | --- |
| ENZRXN.14 | 4-hydroxybutyrate coenzyme A trans | 0 | Not detected | -0.391 | -0.651 | -0.131 | 0.003 |
| ENZRXN.14 | crotonase | 0 | Not detected | -0.086 | -0.363 | 0.192 | 0.546 |
| ENZRXN.14 | butyryl-CoA dehydrogenase | 0 | Not detected | -0.058 | -0.262 | 0.146 | 0.579 |
| ENZRXN.14 | phosphate acetyltransferase | 0 | Not detected | -0.080 | -0.203 | 0.042 | 0.199 |
| ENZRXN.14 | acetate kinase | 0 | Not detected | -0.102 | -0.347 | 0.142 | 0.413 |
| ENZRXN.14 | GDP-a-D-mannose 4,6-dehydratase | 0 | Not detected | -0.087 | -0.350 | 0.176 | 0.518 |
| ENZRXN.14 | aconitase | 0 | Not detected | -0.116 | -0.266 | 0.033 | 0.128 |
| ENZRXN.14 | phosphoenolpyruvate phosphomutas | 0 | Not detected | -0.115 | -0.395 | 0.165 | 0.420 |
| ENZRXN.14 | naphthoyl-CoA synthase | 0 | Not detected | -0.036 | -0.171 | 0.099 | 0.598 |
| ENZRXN.14 | N-acetylhexosamine kinase | 0 | Not detected | 0.005 | -0.272 | 0.283 | 0.971 |
| ENZRXN.14 | glutamyl-tRNAGln synthetase | 0 | Not detected | -0.062 | -0.344 | 0.220 | 0.667 |
| ENZRXN.15 | glutamyl-tRNAGlu synthetase | 0 | Not detected | -0.062 | -0.344 | 0.220 | 0.667 |
| ENZRXN.15 | acyl phosphate:glycerol-3-phosphate | 0 | Not detected | 0.066 | -0.131 | 0.262 | 0.514 |
| ENZRXN.15 | 1-acylglycerol-3-phosphate acyltrans | 0 | Not detected | -0.039 | -0.252 | 0.174 | 0.719 |
| ENZRXN.15 | biotin sulfoxide reductase | 0 | Not detected | 0.112 | -0.107 | 0.332 | 0.315 |
| ENZRXN.15 | malonate decarboxylase acetyl-[acp] | 0 | Not detected | 0.116 | -0.077 | 0.310 | 0.237 |
| ENZRXN.15 | acyl-carrier protein: acetate ligase | 0 | Not detected | -0.156 | -0.440 | 0.127 | 0.280 |
| ENZRXN.15 | acetyl-[acyl-carrier protein]:malonat | 0 | Not detected | -0.094 | -0.366 | 0.177 | 0.495 |
| ENZRXN.15 | carboxybiotin decarboxylase | 0 | Not detected | -0.307 | -0.522 | -0.092 | 0.005 |
| ENZRXN.15 | N-succinylornithine carbamoyltransf | 0 | Not detected | -0.076 | -0.216 | 0.065 | 0.292 |
| ENZRXN.15 | 5-monosubstituted-hydantoin racem | 0 | Not detected | 0.126 | -0.139 | 0.392 | 0.351 |
| ENZRXN.15 | phosphoglycerate mutase | 0 | Not detected | -0.264 | -0.538 | 0.010 | 0.059 |
| ENZRXN.15 | 2-keto-3-deoxy-D-glycero-D-galacto | 0 | Not detected | -0.431 | -0.745 | -0.117 | 0.007 |
| ENZRXN.15 | 2-keto-3-deoxy-D-glycero-D-galacto | 0 | Not detected | -0.286 | -0.580 | 0.007 | 0.056 |
| ENZRXN.16 | ß-D-galactosyl-(1->4)-L-rhamnose p | 0 | Not detected | -0.144 | -0.441 | 0.153 | 0.342 |
| ENZRXN.17 | UDP-N-acetylglucosamine enolpyru | 0 | Not detected | 0.240 | -0.048 | 0.527 | 0.102 |
| ENZRXN.17 | UDP-galactopyranose mutase | 0 | Not detected | 0.253 | -0.051 | 0.557 | 0.102 |
| ENZRXN.17 | 3-dehydroquinate dehydratase | 0 | Not detected | 0.099 | -0.135 | 0.333 | 0.406 |
| ENZRXN.17 | UDP-N-acetylglucosamine--N-acety | 0 | Not detected | -0.190 | -0.357 | -0.023 | 0.026 |
| ENZRXN.17 | UDP-N-acetylmuramoyl--L-alanine | 0 | Not detected | 0.159 | -0.095 | 0.413 | 0.220 |
| ENZRXN.17 | UDP-N-acetylglucosamine 1-carbox | 0 | Not detected | -0.100 | -0.294 | 0.095 | 0.317 |
| ENZRXN.17 | UDP-N-acetylglucosamine 1-carbox | 0 | Not detected | 0.035 | -0.128 | 0.198 | 0.674 |
| ENZRXN.17 | undecaprenyl-pyrophosphoryl-MurN | 0 | Not detected | 0.055 | -0.237 | 0.346 | 0.714 |
| ENZRXN.17 | asparagine synthase | 0 | Not detected | 0.014 | -0.274 | 0.303 | 0.923 |
| ENZRXN.17 | undecaprenyl-pyrophosphoryl-MurN | 0 | Not detected | 0.055 | -0.237 | 0.346 | 0.714 |
| ENZRXN.17 | D-sedoheptulose 7-phosphate isome | 0 | Not detected | -0.048 | -0.312 | 0.215 | 0.719 |
| ENZRXN.17 | D-glycero-a-D-manno-heptose-7-ph | 0 | Not detected | 0.058 | -0.240 | 0.355 | 0.703 |
| ENZRXN.17 | methylthioalkylmalate dehydrogenas | 0 | Not detected | -0.025 | -0.160 | 0.110 | 0.714 |
| ENZRXN.17 | methylthioalkylmalate dehydrogenas | 0 | Not detected | -0.025 | -0.160 | 0.110 | 0.714 |
| ENZRXN.17 | methylthioalkylmalate dehydrogenas | 0 | Not detected | -0.025 | -0.160 | 0.110 | 0.714 |
| ENZRXN.17 | methylthioalkylmalate dehydrogenas | 0 | Not detected | -0.025 | -0.160 | 0.110 | 0.714 |
| ENZRXN.17 | methylthioalkylmalate dehydrogenas | 0 | Not detected | -0.025 | -0.160 | 0.110 | 0.714 |
| ENZRXN.17 | lipoate-ATP adenylate transferase | 0 | Not detected | 0.030 | -0.128 | 0.187 | 0.710 |

| ENZRXN.17 | pimeloyl-[acp] methyl ester esterase | 0 | Not detected | 0.192 | 0.032 | 0.353 | 0.019 |
| --- | --- | --- | --- | --- | --- | --- | --- |
| ENZRXN.17 | (2Z,6E)-farnesyl diphosphate mono- | 0 | Not detected | 0.152 | -0.134 | 0.438 | 0.299 |
| ENZRXN.18 | 3-(hydroxyamino)phenol mutase | 0 | Not detected | -0.123 | -0.415 | 0.169 | 0.410 |
| ENZRXN.18 | butanal dehydrogenase | 0 | Not detected | -0.050 | -0.180 | 0.079 | 0.443 |
| ENZRXN.18 | butanol dehydrogenase | 0 | Not detected | -0.050 | -0.180 | 0.079 | 0.443 |
| ENZRXN.18 | glycerol-2-phosphotransferase | 0 | Not detected | 0.156 | -0.086 | 0.399 | 0.207 |
| ENZRXN.18 | DsrC-trisulfide reductase | 0 | Not detected | 0.083 | -0.181 | 0.346 | 0.539 |
| ENZRXN.18 | dCMP kinase | 0 | Not detected | 0.163 | -0.055 | 0.381 | 0.143 |
| ENZRXN.18 | 3-hydroxy-trans-cinnamate hydroxyl | 0 | Not detected | 0.075 | -0.112 | 0.262 | 0.431 |
| ENZRXN.18 | 1-deoxy-D-xylulose 5-phosphate red | 0 | Not detected | -0.160 | -0.445 | 0.125 | 0.271 |
| ENZRXN.18 | rhamnogalacturonan endolyase | 0 | Not detected | -0.285 | -0.506 | -0.064 | 0.011 |
| ENZRXN.18 | unsaturated chondroitin 6-sulfate dis | 0 | Not detected | -0.099 | -0.329 | 0.130 | 0.397 |
| ENZRXN.18 | legionaminate synthase | 0 | Not detected | -0.213 | -0.470 | 0.044 | 0.105 |
| ENZRXN.18 | rhamnogalacturonan exolyase | 0 | Not detected | -0.168 | -0.411 | 0.076 | 0.177 |
| ENZRXN.18 | isoamylase | 0 | Not detected | 0.226 | -0.039 | 0.492 | 0.095 |
| ENZRXN.18 | formate dehydrogenase | 0 | Not detected | -0.106 | -0.348 | 0.136 | 0.391 |
| ENZRXN.19 | cellobiose phosphorylase | 0 | Not detected | -0.070 | -0.213 | 0.072 | 0.334 |
| ENZRXN.19 | GTP 3',8'-cyclase | 0 | Not detected | 0.068 | -0.116 | 0.252 | 0.471 |
| ENZRXN.19 | endo-a-N-acetylgalactosaminidase | 0 | Not detected | -0.097 | -0.394 | 0.200 | 0.522 |
| ENZRXN.19 | trans-2-enoyl-CoA reductase (NAD+ | 0 | Not detected | -0.090 | -0.322 | 0.141 | 0.444 |
| ENZRXN.19 | crotonyl-CoA reductase | 0 | Not detected | -0.090 | -0.322 | 0.141 | 0.444 |
| ENZRXN.19 | trans-2-hexenoyl-CoA reductase | 0 | Not detected | -0.090 | -0.322 | 0.141 | 0.444 |
| ENZRXN.19 | hexanal dehydrogenase | 0 | Not detected | -0.050 | -0.180 | 0.079 | 0.443 |
| ENZRXN.19 | hexanol dehydrogenase | 0 | Not detected | -0.050 | -0.180 | 0.079 | 0.443 |
| ENZRXN.19 | carboxy-adenylated-[ThiS sulfur-car | 0 | Not detected | -0.042 | -0.245 | 0.162 | 0.688 |
| ENZRXN.19 | molybdopterin synthase sulfurtransfe | 0 | Not detected | 0.211 | 0.046 | 0.375 | 0.012 |
| ENZRXN.19 | N,N'-diacetylchitobiose phosphoryla | 0 | Not detected | 0.001 | -0.272 | 0.275 | 0.991 |
| ENZRXN.19 | 12a-hydroxy-3-oxochol-4,6-dien-24- | 0 | Not detected | 0.197 | -0.071 | 0.466 | 0.150 |
| ENZRXN.19 | dTDP-glucose 4,6-dehydratase | 0 | Not detected | -0.051 | -0.266 | 0.163 | 0.640 |
| ENZRXN.19 | glucose-1-phosphate thymidylyltrans | 0 | Not detected | -0.040 | -0.167 | 0.087 | 0.537 |
| ENZRXN.19 | dTDP-3-amino-3,6-dideoxy-a-D-gal | 0 | Not detected | -0.111 | -0.400 | 0.177 | 0.450 |
| ENZRXN.19 | 2-oxoglutarate decarboxylase | 0 | Not detected | 0.029 | -0.267 | 0.324 | 0.849 |
| ENZRXN.20 | deoxyribonuclease IV | 0 | Not detected | -0.171 | -0.344 | 0.002 | 0.053 |
| ENZRXN.20 | uracil-DNA glycosylase | 0 | Not detected | -0.101 | -0.214 | 0.012 | 0.081 |
| ENZRXN.20 | DNA glycosylase | 0 | Not detected | 0.084 | -0.177 | 0.344 | 0.529 |
| ENZRXN.20 | DNA-formamidopyrimidine glycosy | 0 | Not detected | -0.015 | -0.189 | 0.160 | 0.868 |
| ENZRXN.20 | adenine glycosylase | 0 | Not detected | -0.002 | -0.188 | 0.185 | 0.987 |
| ENZRXN.20 | glycerol-3-phosphate dehydrogenase | 0 | Not detected | -0.159 | -0.419 | 0.101 | 0.232 |
| ENZRXN.20 | 3-isopropylmalate dehydratase | 0 | Not detected | -0.238 | -0.540 | 0.064 | 0.122 |
| ENZRXN.20 | (S)-NADPHX dehydratase | 0 | Not detected | 0.108 | -0.091 | 0.307 | 0.288 |
| ENZRXN.20 | NADPHX epimerase | 0 | Not detected | 0.108 | -0.091 | 0.307 | 0.288 |
| ENZRXN.20 | butanol dehydrogenase | 0 | Not detected | -0.071 | -0.202 | 0.060 | 0.288 |
| ENZRXN.20 | CoA-dependent propionaldehyde de | 0 | Not detected | 0.029 | -0.198 | 0.256 | 0.803 |
| ENZRXN.20 | phosphate propanoyltransferase | 0 | Not detected | -0.071 | -0.312 | 0.169 | 0.560 |

| ENZRXN.20 | phosphate acetyltransferase | 0 | Not detected | -0.071 | -0.312 | 0.169 | 0.560 |
| --- | --- | --- | --- | --- | --- | --- | --- |
| ENZRXN.20 | propionate kinase | 0 | Not detected | 0.071 | -0.144 | 0.287 | 0.516 |
| ENZRXN.20 | thymine oxygenase | 0 | Not detected | -0.034 | -0.198 | 0.131 | 0.687 |
| ENZRXN.20 | hydroxybutyryl-coA dehydrogenase | 0 | Not detected | -0.074 | -0.241 | 0.094 | 0.388 |
| ENZRXN.20 | biotin carboxylase | 0 | Not detected | -0.095 | -0.350 | 0.160 | 0.467 |
| ENZRXN.20 | N,N'-diacetylbacilliosaminyl-1-phos | 0 | Not detected | -0.235 | -0.501 | 0.031 | 0.083 |
| ENZRXN.20 | carboxyspermidine dehydrogenase | 0 | Not detected | -0.068 | -0.189 | 0.053 | 0.271 |
| ENZRXN.20 | N-carbamoylputrescine amidohydrol | 0 | Not detected | -0.086 | -0.228 | 0.056 | 0.235 |
| ENZRXN.20 | 4,4'-diaponeurosporenal dehydrogen | 0 | Not detected | 0.022 | -0.261 | 0.304 | 0.881 |
| ENZRXN.20 | superoxide dismutase | 0 | Not detected | 0.005 | -0.256 | 0.267 | 0.967 |
| ENZRXN.20 | UDP-N-acetyl-2-amino-2-deoxygluc | 0 | Not detected | -0.315 | -0.607 | -0.023 | 0.035 |
| ENZRXN.20 | UDP-2,3-diacetamido-2,3-dideoxygl | 0 | Not detected | 0.001 | -0.289 | 0.292 | 0.992 |
| ENZRXN.20 | enoyl-[acyl-carrier-protein] reductas | 0 | Not detected | -0.132 | -0.416 | 0.153 | 0.365 |
| ENZRXN.20 | enoyl-[acyl-carrier-protein] reductas | 0 | Not detected | -0.132 | -0.416 | 0.153 | 0.365 |
| ENZRXN.20 | crotonyl-[acyl-carrier-protein] reduc | 0 | Not detected | -0.132 | -0.416 | 0.153 | 0.365 |
| ENZRXN.20 | endo-1,4-ß-xylanase | 0 | Not detected | 0.087 | -0.130 | 0.304 | 0.433 |
| ENZRXN.20 | D-fructose-6-phosphate cytidylyltran | 0 | Not detected | 0.082 | -0.198 | 0.361 | 0.566 |
| ENZRXN.21 | acetaldehyde dehydrogenase | 0 | Not detected | -0.107 | -0.345 | 0.130 | 0.377 |
| ENZRXN.21 | homoserine O-acetyltransferase | 0 | Not detected | -0.142 | -0.377 | 0.093 | 0.235 |
| ENZRXN.21 | methylthioribose-1-phosphate isome | 0 | Not detected | -0.025 | -0.301 | 0.251 | 0.860 |
| ENZRXN.21 | oligosaccharide reducing-end xylana | 0 | Not detected | 0.028 | -0.171 | 0.226 | 0.784 |
| ENZRXN.21 | ß-L-arabinofuranosidase | 0 | Not detected | 0.132 | -0.151 | 0.415 | 0.360 |
| ENZRXN.21 | uridine kinase | 0 | Not detected | 0.077 | -0.168 | 0.323 | 0.537 |
| ENZRXN.21 | cytidine kinase | 0 | Not detected | 0.077 | -0.168 | 0.323 | 0.537 |
| ENZRXN.21 | urocanate reductase | 0 | Not detected | 0.066 | -0.231 | 0.364 | 0.662 |
| ENZRXN.21 | 3D-(3,5/4)-trihydroxycyclohexane-1 | 0 | Not detected | -0.055 | -0.243 | 0.132 | 0.563 |
| ENZRXN.21 | myo-inositol 2-dehydrogenase | 0 | Not detected | -0.048 | -0.281 | 0.185 | 0.686 |
| ENZRXN.21 | D-mannonate dehydrogenase | 0 | Not detected | -0.098 | -0.236 | 0.040 | 0.164 |
| ENZRXN.21 | D-mannonate dehydratase | 0 | Not detected | -0.027 | -0.159 | 0.104 | 0.682 |
| ENZRXN.21 | 2-keto-3-deoxygluconate kinase | 0 | Not detected | -0.081 | -0.206 | 0.044 | 0.204 |
| ENZRXN.21 | [sulfur carrier protein CysO]--sulfur | 0 | Not detected | 0.038 | -0.231 | 0.306 | 0.784 |
| ENZRXN.21 | N-acetyl-a-D-glucosaminyl-diphosp | 0 | Not detected | -0.269 | -0.554 | 0.016 | 0.065 |
| ENZRXN.21 | glucose-1-phosphate thymidylyltrans | 0 | Not detected | -0.213 | -0.427 | 0.001 | 0.051 |
| ENZRXN.21 | dTDP-glucose 4,6-dehydratase | 0 | Not detected | -0.094 | -0.300 | 0.111 | 0.368 |
| ENZRXN.21 | dTDP-4-amino-4,6-dideoxy-D-gluco | 0 | Not detected | -0.110 | -0.411 | 0.192 | 0.476 |
| ENZRXN.21 | UDP-glucose 6-dehydrogenase | 0 | Not detected | -0.165 | -0.438 | 0.108 | 0.235 |
| ENZRXN.21 | UDP-N-acetylglucosamine 4,6-dehy | 0 | Not detected | -0.110 | -0.350 | 0.131 | 0.371 |
| ENZRXN.21 | UDP-2-acetamido-2,6-dideoxy-ß-L-t | 0 | Not detected | 0.201 | -0.085 | 0.486 | 0.168 |
| ENZRXN.21 | UDP-N-acetyl-ß-L-rhamnosamine 2- | 0 | Not detected | -0.257 | -0.512 | -0.001 | 0.049 |
| ENZRXN.22 | UDP-N-acetylglucosamine 4,6-dehy | 0 | Not detected | -0.144 | -0.404 | 0.117 | 0.279 |
| ENZRXN.22 | ß-L-arabinobiosidase | 0 | Not detected | 0.097 | -0.226 | 0.420 | 0.556 |
| ENZRXN.22 | butyryl-CoA dehydrogenase | 0 | Not detected | -0.049 | -0.182 | 0.084 | 0.469 |
| ENZRXN.22 | succinyl-CoA:acetate CoA-transfera | 0 | Not detected | 0.078 | -0.215 | 0.371 | 0.603 |
| ENZRXN.22 | propionyl-CoA:succinate CoA transf | 0 | Not detected | 0.078 | -0.215 | 0.371 | 0.603 |

| ENZRXN.22 | N-acetyl-ß-neuraminate:H+ symport | 0 | Not detected | 0.222 | 0.034 | 0.410 | 0.021 |
| --- | --- | --- | --- | --- | --- | --- | --- |
| ENZRXN.22 | 1,4-ß-mannanase | 0 | Not detected | -0.156 | -0.408 | 0.095 | 0.223 |
| ENZRXN.22 | mannobiose 2-epimerase | 0 | Not detected | -0.147 | -0.364 | 0.069 | 0.182 |
| ENZRXN.22 | cellobiose 2-epimerase | 0 | Not detected | -0.147 | -0.364 | 0.069 | 0.182 |
| ENZRXN.22 | cellobiose 2-epimerase | 0 | Not detected | 0.001 | -0.249 | 0.252 | 0.992 |
| ENZRXN.22 | UDP-acetamido-4-amino-6-deoxyga | 0 | Not detected | -0.306 | -0.596 | -0.016 | 0.039 |
| ENZRXN.22 | 2-oxo-4-methylthiobutanoate-glutam | 0 | Not detected | -0.010 | -0.235 | 0.215 | 0.930 |
| ENZRXN.23 | CoA-dependent (R)-lactaldehyde de | 0 | Not detected | 0.029 | -0.198 | 0.256 | 0.803 |
| ENZRXN.23 | CoA-dependent (S)-lactaldehyde deh | 0 | Not detected | 0.029 | -0.198 | 0.256 | 0.803 |
| ENZRXN.23 | (R)-lactaldehyde reductase | 0 | Not detected | 0.103 | -0.057 | 0.262 | 0.208 |
| ENZRXN.23 | (S)-lactaldehyde reductase | 0 | Not detected | 0.103 | -0.057 | 0.262 | 0.208 |
| ENZRXN.23 | (R)-2,3-dihydroxy-3-methylbutanoat | 0 | Not detected | -0.053 | -0.176 | 0.071 | 0.404 |
| ENZRXN.23 | (R)-2,3-dihydroxy-3-methylpentanoa | 0 | Not detected | -0.053 | -0.176 | 0.071 | 0.404 |
| ENZRXN.23 | (R)-2,3-dihydroxy-3-methylbutanoat | 0 | Not detected | -0.137 | -0.392 | 0.118 | 0.292 |
| ENZRXN.23 | (R)-2,3-dihydroxy-3-methylpentanoa | 0 | Not detected | -0.137 | -0.392 | 0.118 | 0.292 |
| ENZRXN.23 | Fe-coproporphyrin synthase | 0 | Not detected | 0.162 | -0.107 | 0.432 | 0.237 |
| ENZRXN.23 | bacimethrin diphosphokinase | 0 | Not detected | -0.173 | -0.342 | -0.004 | 0.045 |
| ENZRXN.23 | L-galactose dehydrogenase | 0 | Not detected | -0.188 | -0.397 | 0.021 | 0.078 |
| ENZRXN.23 | L-galactonate 5-dehydrogenase | 0 | Not detected | -0.163 | -0.369 | 0.043 | 0.122 |
| ENZRXN.23 | N-acetylneuraminate lyase | 0 | Not detected | -0.258 | -0.449 | -0.068 | 0.008 |
| ENZRXN.23 | N-acetylglucosamine kinase | 0 | Not detected | -0.105 | -0.241 | 0.031 | 0.131 |
| ENZRXN.23 | 7-oxolithocholate reductase | 0 | Not detected | 0.016 | -0.224 | 0.256 | 0.896 |
| ENZRXN.23 | 7-oxolithocholate reductase | 0 | Not detected | 0.016 | -0.224 | 0.256 | 0.896 |
| ENZRXN.23 | GDP-6-deoxy-4-keto-D-arabino-hep | 0 | Not detected | -0.195 | -0.384 | -0.007 | 0.042 |
| ENZRXN.23 | UDP-N-acetylglucosamine 6-dehydr | 0 | Not detected | -0.180 | -0.378 | 0.018 | 0.074 |
| ENZRXN.23 | UDP-N-acetylgalactosamine 6-dehy | 0 | Not detected | -0.180 | -0.378 | 0.018 | 0.074 |
| ENZRXN.23 | Kdo2-lipid A phosphotransferase | 0 | Not detected | -0.054 | -0.279 | 0.170 | 0.635 |
| ENZRXN.23 | Kdo2-lipid A phosphoethanolamine | 0 | Not detected | 0.141 | -0.065 | 0.346 | 0.180 |
| ENZRXN.23 | phosphomannomutase | 0 | Not detected | -0.081 | -0.360 | 0.198 | 0.567 |
| ENZRXN.23 | phosphoglucomutase | 0 | Not detected | -0.081 | -0.360 | 0.198 | 0.567 |
| ENZRXN.23 | heparin lyase | 0 | Not detected | -0.365 | -0.722 | -0.008 | 0.045 |
| ENZRXN.23 | heparan sulfate lyase | 0 | Not detected | -0.365 | -0.722 | -0.008 | 0.045 |
| ENZRXN.23 | heparin lyase | 0 | Not detected | -0.305 | -0.573 | -0.036 | 0.026 |
| ENZRXN.23 | unsaturated hyaluronan disaccharide | 0 | Not detected | -0.099 | -0.329 | 0.130 | 0.397 |
| ENZRXN.23 | unsaturated chondroitin disaccharide | 0 | Not detected | -0.099 | -0.329 | 0.130 | 0.397 |
| ENZRXN.23 | 4-deoxy-ß-L-erythro-hex-4-enopyran | 0 | Not detected | 0.129 | -0.154 | 0.411 | 0.373 |
| ENZRXN.23 | 4-deoxy-ß-D-gluc-4-enuronosyl-(1,4 | 0 | Not detected | 0.129 | -0.154 | 0.411 | 0.373 |
| ENZRXN.23 | 4-deoxy-ß-D-gluc-4-enuronosyl-(1,4 | 0 | Not detected | 0.129 | -0.154 | 0.411 | 0.373 |
| ENZRXN.23 | 4-deoxy-ß-L-erythro-hex-4-enopyran | 0 | Not detected | 0.129 | -0.154 | 0.411 | 0.373 |
| ENZRXN.23 | (5Z)-3-oxo-tetradec-5-enoyl-[acyl-ca | 0 | Not detected | -0.107 | -0.236 | 0.021 | 0.101 |
| ENZRXN.23 | (5Z)-3-oxo-tetradec-5-enoyl-[acyl-ca | 0 | Not detected | 0.031 | -0.203 | 0.265 | 0.793 |
| ENZRXN.23 | (2E,5Z)-tetradeca-2,5-dienoyl-[acp] | 0 | Not detected | -0.046 | -0.249 | 0.158 | 0.660 |
| ENZRXN.23 | (3R)-3-hydroxydecanoyl-[acp] dehy | 0 | Not detected | -0.148 | -0.346 | 0.049 | 0.142 |
| ENZRXN.23 | (2E)-dec-2-enoyl-[acp] isomerase | 0 | Not detected | -0.148 | -0.346 | 0.049 | 0.142 |

| ENZRXN.23 | (5Z)-3-oxo-dodec-5-enoyl-[acp] syn | 0 | Not detected | -0.065 | -0.190 | 0.060 | 0.306 |
| --- | --- | --- | --- | --- | --- | --- | --- |
| ENZRXN.23 | (7Z)-3-oxo-hexadec-7-enoyl-[acp] s | 0 | Not detected | -0.107 | -0.236 | 0.021 | 0.101 |
| ENZRXN.23 | (9Z)-3-oxo-octadec-9-enoyl-[acp] sy | 0 | Not detected | -0.107 | -0.236 | 0.021 | 0.101 |
| ENZRXN.23 | (7Z)-3-oxo-hexadec-7-enoyl-[acp] re | 0 | Not detected | 0.031 | -0.203 | 0.265 | 0.793 |
| ENZRXN.23 | (9Z)-3-oxo-octadec-9-enoyl-[acp] re | 0 | Not detected | 0.031 | -0.203 | 0.265 | 0.793 |
| ENZRXN.23 | (11Z)-3-oxo-icos-11-enoyl-[acp] red | 0 | Not detected | 0.031 | -0.203 | 0.265 | 0.793 |
| ENZRXN.23 | (11Z)-3-oxo-icos-11-enoyl-[acp] syn | 0 | Not detected | -0.107 | -0.236 | 0.021 | 0.101 |
| ENZRXN.23 | (2E,7Z)-hexadeca-2,7-dienoyl-[acp] | 0 | Not detected | -0.046 | -0.249 | 0.158 | 0.660 |
| ENZRXN.23 | (2E,9Z)-octadeca-2,9-dienoyl-[acp] | 0 | Not detected | -0.046 | -0.249 | 0.158 | 0.660 |
| ENZRXN.23 | (2E,11Z)-icosa-2,11-dienoyl-[acp] r | 0 | Not detected | -0.046 | -0.249 | 0.158 | 0.660 |
| ENZRXN.23 | lactose 2-epimerase | 0 | Not detected | 0.001 | -0.249 | 0.252 | 0.992 |
| ENZRXN.23 | 8-amino-3,8-dideoxy-manno-octulos | 0 | Not detected | -0.177 | -0.423 | 0.068 | 0.157 |
| ENZRXN.24 | scaffold protein for iron-sulfur cluste | 0 | Not detected | -0.297 | -0.541 | -0.052 | 0.017 |
| ENZRXN.24 | lactate racemase | 0 | Not detected | -0.061 | -0.283 | 0.161 | 0.589 |
| ENZRXN.24 | UDP-4-amino-4,6-dideoxy-N-acetyl | 0 | Not detected | -0.177 | -0.478 | 0.124 | 0.249 |
| ENZRXN.24 | guanosine phosphorylase | 0 | Not detected | -0.233 | -0.515 | 0.048 | 0.105 |
| ENZRXN.24 | deoxycholate 3a-dehydrogenase | 0 | Not detected | 0.035 | -0.160 | 0.229 | 0.726 |
| ENZRXN.24 | deoxycholate 3a-dehydrogenase | 0 | Not detected | -0.093 | -0.375 | 0.190 | 0.521 |
| ENZRXN.24 | 3-oxochol-4,6-dien-24-oyl-CoA 6-re | 0 | Not detected | 0.197 | -0.071 | 0.466 | 0.150 |
| ENZRXN.24 | lithocholate 3a-dehydrogenase | 0 | Not detected | -0.093 | -0.375 | 0.190 | 0.521 |
| ENZRXN.24 | chenodeoxycholate 3a-dehydrogenas | 0 | Not detected | -0.093 | -0.375 | 0.190 | 0.521 |
| ENZRXN.24 | cholate 3a-dehydrogenase | 0 | Not detected | -0.093 | -0.375 | 0.190 | 0.521 |
| ENZRXN.24 | lithocholate 3a-dehydrogenase | 0 | Not detected | 0.035 | -0.160 | 0.229 | 0.726 |
| ENZRXN.24 | cholate 3a-dehydrogenase | 0 | Not detected | 0.035 | -0.160 | 0.229 | 0.726 |
| ENZRXN.24 | chenodeoxycholate 3a-dehydrogenas | 0 | Not detected | 0.035 | -0.160 | 0.229 | 0.726 |
| ENZRXN.24 | ß-1,2-mannosidase | 0 | Not detected | -0.280 | -0.517 | -0.042 | 0.021 |
| ENZRXN.24 | L-galacto-1,5-lactonase | 0 | Not detected | -0.217 | -0.416 | -0.017 | 0.034 |
| ENZRXN.24 | succinate:malate antiport | 0 | Not detected | 0.001 | -0.238 | 0.240 | 0.996 |
| ENZRXN.24 | succinate:L-aspartate antiport | 0 | Not detected | 0.001 | -0.238 | 0.240 | 0.996 |
| ENZRXN.24 | malate:succinate antiporter | 0 | Not detected | 0.043 | -0.162 | 0.248 | 0.681 |
| ENZRXN.24 | L-aspartate:succinate antiporter | 0 | Not detected | 0.043 | -0.162 | 0.248 | 0.681 |
| ENZRXN.24 | dihydroorotate dehydrogenase | 0 | Not detected | -0.037 | -0.314 | 0.240 | 0.795 |
| ENZRXN.24 | methionine &gamma;-lyase | 0 | Not detected | -0.079 | -0.328 | 0.169 | 0.532 |
| ENZRXN.24 | [HypE]-S-carboxamide dehydratase | 0 | Not detected | 0.022 | -0.169 | 0.214 | 0.820 |
| ENZRXN.25 | a-D-ribose-1-(2-N-acetamidomethyl | 0 | Not detected | 0.165 | -0.022 | 0.351 | 0.083 |
| ENZRXN.25 | carbon-phosphorous lyase | 0 | Not detected | 0.171 | -0.044 | 0.386 | 0.119 |
| ENZRXN.25 | a-D-ribose-1-[N-(phosphonomethyl) | 0 | Not detected | -0.074 | -0.281 | 0.133 | 0.483 |
| ENZRXN.25 | a-D-ribose-1-[N-(phosphonomethyl) | 0 | Not detected | -0.182 | -0.427 | 0.063 | 0.145 |
| ENZRXN.25 | adenine phosphoribosyltransferase | 0 | Not detected | -0.089 | -0.354 | 0.177 | 0.513 |
| ENZRXN.25 | tetracycline 11a-oxygenase | 0 | Not detected | -0.267 | -0.634 | 0.100 | 0.154 |
| ENZRXN.25 | tigecycline 11a-momooxygenase | 0 | Not detected | -0.267 | -0.634 | 0.100 | 0.154 |
| ENZRXN.25 | UDP-N-acetylglucosamine 2-epimer | 0 | Not detected | -0.027 | -0.309 | 0.256 | 0.854 |
| ENZRXN.25 | ribitol-5-phosphate cytidylyltransfer | 0 | Not detected | -0.060 | -0.342 | 0.222 | 0.676 |
| ENZRXN.25 | ribitol-5-phosphate 1-dehydrogenase | 0 | Not detected | 0.080 | -0.200 | 0.360 | 0.576 |

| ENZRXN.25 | ribitol-5-phosphate 1-dehydrogenase | 0 | Not detected | 0.146 | -0.146 | 0.438 | 0.327 |
| --- | --- | --- | --- | --- | --- | --- | --- |
| ENZRXN.25 | UTP--glucose-1-phosphate uridylylt | 0 | Not detected | -0.075 | -0.342 | 0.193 | 0.583 |
| ENZRXN.25 | undecaprenyl-phosphate 2-acetamid | 0 | Not detected | -0.088 | -0.347 | 0.171 | 0.506 |
| ENZRXN.25 | D-alanine--[D-alanyl carrier protein] | 0 | Not detected | 0.026 | -0.246 | 0.299 | 0.849 |
| ENZRXN.25 | PG:teichoic acid D-alanyltransferase | 0 | Not detected | 0.125 | -0.124 | 0.375 | 0.325 |
| ENZRXN.25 | D-alanine carrier protein:PG D-alany | 0 | Not detected | 0.057 | -0.192 | 0.307 | 0.652 |
| ENZRXN.25 | 1,2-diacylglycerol 3-a-glucosyltransf | 0 | Not detected | 0.069 | -0.169 | 0.306 | 0.570 |
| ENZRXN.25 | isopropylmalate dehydrogenase | 0 | Not detected | -0.025 | -0.160 | 0.110 | 0.714 |
| ENZRXN.25 | tRNA uridine 4-sulfurtransferase | 0 | Not detected | -0.042 | -0.245 | 0.162 | 0.688 |
| ENZRXN.25 | aminobutyraldehyde dehydrogenase | 0 | Not detected | 0.115 | -0.061 | 0.290 | 0.201 |
| ENZRXN.25 | succinate-semialdehyde dehydrogen | 0 | Not detected | 0.115 | -0.061 | 0.290 | 0.201 |
| ENZRXN.25 | 7,8-dihydro-8-oxoguanine DNA glyc | 0 | Not detected | -0.015 | -0.189 | 0.160 | 0.868 |
| ENZRXN.25 | [sulfur carrier protein TtuB] adenyly | 0 | Not detected | -0.264 | -0.508 | -0.020 | 0.034 |
| ENZRXN.25 | trans-4-hydroxy-L-proline dehydrata | 0 | Not detected | -0.118 | -0.275 | 0.040 | 0.142 |
| ENZRXN.25 | menaquinone C8-methyltransferase | 0 | Not detected | -0.054 | -0.349 | 0.241 | 0.719 |
| ENZRXN.25 | quinol--ferredoxin oxidoreductase | 0 | Not detected | -0.031 | -0.323 | 0.261 | 0.836 |
| ENZRXN.25 | dissimilatory sulfite reductase | 0 | Not detected | 0.134 | -0.166 | 0.434 | 0.382 |
| ENZRXN.25 | L-threonate dehydrogenase | 0 | Not detected | -0.049 | -0.218 | 0.120 | 0.570 |
| ENZRXN.25 | 2-dehydro-D-threonate isomerase | 0 | Not detected | 0.048 | -0.110 | 0.207 | 0.550 |
| ENZRXN.25 | 2-dehydro-L-threonate isomerase | 0 | Not detected | 0.048 | -0.110 | 0.207 | 0.550 |
| ENZRXN.25 | 4-phospho-D-erythronate 3-dehydro | 0 | Not detected | -0.126 | -0.385 | 0.133 | 0.339 |
| ENZRXN.25 | UDP-N-acetylmuramate--L-alanyl-g | 0 | Not detected | -0.223 | -0.519 | 0.073 | 0.139 |
| ENZRXN.25 | methenyltetrahydrofolate cyclohydro | 0 | Not detected | -0.152 | -0.293 | -0.012 | 0.033 |
| ENZRXN.25 | methylenetetrahydrofolate dehydrog | 0 | Not detected | -0.152 | -0.293 | -0.012 | 0.033 |
| ENZRXN.25 | deoxyribodipyrimidine photolyase | 0 | Not detected | 0.110 | -0.078 | 0.298 | 0.250 |
| ENZRXN.25 | deoxyribodipyrimidine photolyase | 0 | Not detected | 0.110 | -0.078 | 0.298 | 0.250 |
| ENZRXN.25 | pyridinium-3,5-biscarboxylic acid m | 0 | Not detected | -0.102 | -0.282 | 0.079 | 0.269 |
| ENZRXN.26 | acetate kinase | 0 | Not detected | 0.066 | -0.095 | 0.227 | 0.421 |
| ENZRXN.26 | acetaldehyde dehydrogenase (acetyla | 0 | Not detected | 0.165 | -0.066 | 0.396 | 0.163 |
| ENZRXN.26 | glyoxal reductase | 0 | Not detected | -0.109 | -0.351 | 0.134 | 0.380 |
| ENZRXN.26 | methylglyoxal reductase | 0 | Not detected | -0.109 | -0.351 | 0.134 | 0.380 |
| ENZRXN.26 | 3-oxochol-4-en-24-oyl-CoA 4-reduc | 0 | Not detected | 0.197 | -0.071 | 0.466 | 0.150 |
| ENZRXN.26 | 12a-hydroxy-3-oxochol-4-en-24-oyl | 0 | Not detected | 0.197 | -0.071 | 0.466 | 0.150 |
| ENZRXN.26 | 3-hydroxyhexadecanoyl-CoA:glycin | 0 | Not detected | -0.058 | -0.197 | 0.082 | 0.417 |
| ENZRXN.26 | diaminohydroxyphosphoribosylamin | 0 | Not detected | 0.027 | -0.219 | 0.273 | 0.830 |
| ENZRXN.26 | 5-amino-6-(5-phosphoribosylamino) | 0 | Not detected | 0.027 | -0.219 | 0.273 | 0.830 |
| ENZRXN.26 | 1,2-a-L-arabinofuranosidase | 0 | Not detected | -0.054 | -0.183 | 0.075 | 0.410 |
| ENZRXN.26 | 2-hydroxyethylphosphonate cytidyly | 0 | Not detected | -0.115 | -0.395 | 0.165 | 0.420 |
| ENZRXN.26 | acetate kinase | 0 | Not detected | -0.070 | -0.218 | 0.078 | 0.353 |
| ENZRXN.26 | thiol-disulfide exchange | 0 | Not detected | 0.232 | 0.025 | 0.438 | 0.028 |
| ENZRXN.26 | phosphofructokinase | 0 | Not detected | -0.008 | -0.170 | 0.154 | 0.920 |
| ENZRXN.28 | fructose-bisphosphate aldolase | 0 | Not detected | -0.063 | -0.191 | 0.066 | 0.338 |
| ENZRXN.29 | phospho-ß-galactosidase | 0 | Not detected | 0.153 | -0.115 | 0.421 | 0.263 |
| ENZRXN.33 | aspartokinase | 0 | Not detected | -0.170 | -0.444 | 0.104 | 0.225 |

| ENZRXN.44 | GDP-4-keto-6-deoxymannose-3,5-ep | 0 | Not detected | -0.097 | -0.368 | 0.173 | 0.481 |
| --- | --- | --- | --- | --- | --- | --- | --- |
| ENZRXN.58 | isoamylase | 0 | Not detected | 0.186 | -0.102 | 0.473 | 0.206 |
| ENZRXN.60 | trehalose-6-phosphate hydrolase | 0 | Not detected | -0.153 | -0.299 | -0.008 | 0.039 |
| ENZRXN.61 | 16S rRNA m2G1207 methyltransfer | 0 | Not detected | -0.075 | -0.251 | 0.102 | 0.406 |
| ENZRXN.62 | a-phosphoglucomutase | 0 | Not detected | -0.139 | -0.365 | 0.088 | 0.230 |
| ENZRXN.63 | UDP-glucose-4-epimerase | 0 | Not detected | 0.025 | -0.159 | 0.209 | 0.793 |
| ENZRXN.67 | N-succinyldiaminopimelate-aminotr | 0 | Not detected | -0.174 | -0.434 | 0.086 | 0.190 |
| ENZRXN.68 | aspartate kinase | 0 | Not detected | -0.065 | -0.306 | 0.176 | 0.596 |
| ENZRXN.71 | 6-phosphogluconate dehydrogenase | 0 | Not detected | -0.131 | -0.322 | 0.060 | 0.178 |
| ENZRXN.78 | phosphoribosylamidoimidazole-succ | 0 | Not detected | -0.024 | -0.301 | 0.252 | 0.862 |
| ENZRXN.80 | nitric oxide dioxygenase | 56 | Not detected | -0.100 | -0.271 | 0.072 | 0.256 |
| ENZRXN.89 | UDP-N-acetylglucosamine 2-epimer | 0 | Not detected | -0.028 | -0.188 | 0.132 | 0.728 |
| ENZRXN.90 | UTP-glucose-1-phosphate uridylyltr | 0 | Not detected | -0.046 | -0.228 | 0.136 | 0.619 |
| ENZRXN.93 | NAD-dependent glyceraldehyde-3-p | 0 | Not detected | -0.155 | -0.421 | 0.112 | 0.257 |
| ENZRXN.95 | O-acetylhomoserine sulfhydrylase | 0 | Not detected | -0.071 | -0.190 | 0.047 | 0.237 |
| ENZRXN0.1 | lipoyl-adenylate--protein ligase | 0 | Not detected | 0.030 | -0.128 | 0.187 | 0.710 |
| ENZRXN0.1 | 23S rRNA m5U1939 methyltransfer | 0 | Not detected | 0.087 | -0.095 | 0.269 | 0.348 |
| ENZRXN0.1 | GTPase | 0 | Not detected | 0.022 | -0.173 | 0.216 | 0.828 |
| ENZRXN0.1 | lipoate acetyltransferase | 0 | Not detected | 0.191 | -0.033 | 0.416 | 0.095 |
| ENZRXN0.1 | lipoate-apo-GcvH protein ligase | 0 | Not detected | 0.030 | -0.128 | 0.187 | 0.710 |
| ENZRXN0.1 | protein-(glutamine-N5) methyltransf | 0 | Not detected | 0.153 | -0.012 | 0.318 | 0.070 |
| ENZRXN0.1 | protein-glutamine N-methyltransfera | 0 | Not detected | -0.084 | -0.258 | 0.090 | 0.343 |
| ENZRXN0.1 | propanoate:CoA ligase (AMP-formi | 0 | Not detected | -0.024 | -0.255 | 0.208 | 0.841 |
| ENZRXN0.1 | tRNA-dihydrouridine synthase | 0 | Not detected | 0.038 | -0.209 | 0.285 | 0.765 |
| ENZRXN0.1 | tRNA-dihydrouridine synthase | 0 | Not detected | -0.080 | -0.265 | 0.104 | 0.394 |
| ENZRXN0.1 | tRNA-dihydrouridine16 synthase | 0 | Not detected | 0.225 | 0.050 | 0.400 | 0.012 |
| ENZRXN0.1 | tRNA(m5U54)methyltransferase | 0 | Not detected | 0.197 | -0.001 | 0.394 | 0.051 |
| ENZRXN0.1 | deoxyribodipyrimidine photolyase | 0 | Not detected | 0.110 | -0.078 | 0.298 | 0.250 |
| ENZRXN0.1 | O-6-methylguanine-DNA methyltran | 0 | Not detected | 0.132 | -0.037 | 0.301 | 0.125 |
| ENZRXN0.1 | CCA tRNA nucleotidyltransferase | 0 | Not detected | -0.071 | -0.269 | 0.128 | 0.485 |
| ENZRXN0.1 | DNA adenine methyltransferase | 0 | Not detected | -0.114 | -0.299 | 0.071 | 0.227 |
| ENZRXN0.1 | tRNA dimethylallyltransferase | 0 | Not detected | -0.100 | -0.323 | 0.123 | 0.379 |
| ENZRXN0.1 | tRNA preQ134 S-adenosylmethionin | 0 | Not detected | -0.084 | -0.226 | 0.057 | 0.243 |
| ENZRXN0.1 | ribose 1,5-bisphosphate phosphokin | 0 | Not detected | 0.115 | -0.036 | 0.266 | 0.135 |
| ENZRXN0.1 | TDP-N-acetylfucosamine:lipid II N- | 0 | Not detected | 0.185 | 0.001 | 0.369 | 0.048 |
| ENZRXN0.1 | ferroxidase | 0 | Not detected | 0.175 | -0.016 | 0.366 | 0.072 |
| ENZRXN0.1 | inosine:H+ antiporter | 0 | Not detected | 0.034 | -0.149 | 0.217 | 0.713 |
| ENZRXN0.2 | carboxylesterase | 0 | Not detected | 0.192 | 0.032 | 0.353 | 0.019 |
| ENZRXN0.2 | hydroxylamine reductase | 0 | Not detected | 0.179 | -0.014 | 0.372 | 0.069 |
| ENZRXN0.2 | diadenosine tetraphosphatase | 0 | Not detected | -0.007 | -0.182 | 0.169 | 0.942 |
| ENZRXN0.2 | DNA-cytosine methyltransferase | 0 | Not detected | 0.142 | -0.036 | 0.320 | 0.117 |
| ENZRXN0.2 | phosphatidylglycerol&mdash;prolip | 0 | Not detected | 0.083 | -0.141 | 0.307 | 0.470 |
| ENZRXN0.2 | enterochelin esterase | 0 | Not detected | 0.031 | -0.140 | 0.202 | 0.720 |
| ENZRXN0.2 | limit dextrin a-1,6-glucohydrolase | 0 | Not detected | 0.091 | -0.131 | 0.314 | 0.422 |

| ENZRXN0.2 | long-chain fatty acid outer membran | 0 | Not detected | 0.220 | 0.052 | 0.388 | 0.010 |
| --- | --- | --- | --- | --- | --- | --- | --- |
| ENZRXN0.2 | ferric dihyroxybenzoylserine outer m | 0 | Not detected | 0.009 | -0.175 | 0.193 | 0.926 |
| ENZRXN0.2 | UDP-4-amino-4-deoxy-L-arabinose | 0 | Not detected | 0.108 | -0.062 | 0.279 | 0.214 |
| ENZRXN0.2 | guanosine:H+ antiporter | 0 | Not detected | 0.034 | -0.149 | 0.217 | 0.713 |
| ENZRXN0.2 | ß-N-acetylglucosaminidase | 0 | Not detected | 0.028 | -0.157 | 0.213 | 0.767 |
| ENZRXN0.2 | guanine deaminase | 0 | Not detected | 0.032 | -0.169 | 0.233 | 0.755 |
| ENZRXN0.2 | crotonyl-CoA hydratase | 0 | Not detected | 0.010 | -0.180 | 0.199 | 0.920 |
| ENZRXN0.2 | L-ribulose-5-phosphate 4-epimerase | 0 | Not detected | 0.100 | -0.069 | 0.269 | 0.247 |
| ENZRXN0.2 | dihydropteridine reductase | 56 | Not detected | -0.100 | -0.271 | 0.072 | 0.256 |
| ENZRXN0.2 | 23S rRNA pseudouridine746 syntha | 0 | Not detected | -0.127 | -0.300 | 0.046 | 0.150 |
| ENZRXN0.2 | 23S rRNA pseudouridine2604 synth | 0 | Not detected | -0.104 | -0.258 | 0.051 | 0.188 |
| ENZRXN0.2 | 23S rRNA pseudouridine2457 synth | 0 | Not detected | 0.148 | -0.061 | 0.358 | 0.166 |
| ENZRXN0.2 | 23S rRNA pseudouridine955/2504/2 | 0 | Not detected | 0.027 | -0.177 | 0.230 | 0.797 |
| ENZRXN0.2 | inositol-phosphate phosphatase | 0 | Not detected | 0.086 | -0.113 | 0.284 | 0.398 |
| ENZRXN0.2 | ß-keto ester reductase | 0 | Not detected | -0.003 | -0.165 | 0.158 | 0.970 |
| ENZRXN0.2 | propionyl-CoA:succinate CoA transf | 0 | Not detected | -0.165 | -0.333 | 0.004 | 0.055 |
| ENZRXN0.2 | tRNAIle-lysidine synthetase | 0 | Not detected | 0.046 | -0.142 | 0.234 | 0.629 |
| ENZRXN0.2 | 23S rRNA pseudouridine1911/1915/ | 0 | Not detected | 0.061 | -0.144 | 0.266 | 0.557 |
| ENZRXN0.2 | 16S rRNA pseudouridine516 syntha | 0 | Not detected | -0.006 | -0.166 | 0.154 | 0.943 |
| ENZRXN0.2 | lipid IVA 4-amino-4-deoxy-L-arabin | 0 | Not detected | -0.011 | -0.195 | 0.173 | 0.906 |
| ENZRXN0.2 | 3-hydroxyacyl-CoA dehydrogenase | 0 | Not detected | 0.105 | -0.096 | 0.306 | 0.304 |
| ENZRXN0.2 | dodecenoyl-CoA D-isomerase | 0 | Not detected | 0.105 | -0.096 | 0.306 | 0.304 |
| ENZRXN0.2 | 3-hydroxybutyryl-CoA epimerase | 0 | Not detected | 0.105 | -0.096 | 0.306 | 0.304 |
| ENZRXN0.2 | acetyl-CoA C-acyltransferase | 0 | Not detected | 0.031 | -0.148 | 0.210 | 0.735 |
| ENZRXN0.2 | short chain acyl-CoA synthetase | 0 | Not detected | 0.206 | -0.018 | 0.430 | 0.072 |
| ENZRXN0.2 | enoyl-CoA hydratase | 0 | Not detected | 0.105 | -0.096 | 0.306 | 0.304 |
| ENZRXN0.2 | tyrosine kinase | 0 | Not detected | 0.117 | -0.075 | 0.309 | 0.234 |
| ENZRXN0.2 | tRNA-specific 2-thiouridylase | 0 | Not detected | 0.010 | -0.187 | 0.207 | 0.921 |
| ENZRXN0.2 | NADH oxidoreductase | 0 | Not detected | 0.180 | 0.023 | 0.338 | 0.025 |
| ENZRXN0.2 | 3-hydroxyadipyl-CoA dehydrogenas | 0 | Not detected | -0.045 | -0.215 | 0.126 | 0.606 |
| ENZRXN0.2 | D-allulose-6-phosphate 3-epimerase | 0 | Not detected | 0.022 | -0.249 | 0.293 | 0.874 |
| ENZRXN0.2 | cardiolipin synthase | 0 | Not detected | 0.236 | 0.071 | 0.402 | 0.005 |
| ENZRXN0.2 | NADH cupric reductase | 0 | Not detected | 0.109 | -0.082 | 0.299 | 0.263 |
| ENZRXN0.2 | DNA ligase | 0 | Not detected | 0.087 | -0.119 | 0.294 | 0.406 |
| ENZRXN0.2 | DNA ligase | 0 | Not detected | -0.016 | -0.286 | 0.254 | 0.908 |
| ENZRXN0.2 | peptidyl-prolyl cis-trans isomerase | 0 | Not detected | -0.053 | -0.299 | 0.193 | 0.673 |
| ENZRXN0.2 | peptidyl-prolyl cis-trans isomerase | 0 | Not detected | 0.088 | -0.159 | 0.334 | 0.486 |
| ENZRXN0.2 | UDP-2,3-diacylglucosamine diphosp | 0 | Not detected | 0.006 | -0.152 | 0.165 | 0.936 |
| ENZRXN0.2 | pyruvate-flavodoxin oxidoreductase | 0 | Not detected | 0.199 | -0.112 | 0.510 | 0.209 |
| ENZRXN0.2 | malate:quinone oxidoreductase | 0 | Not detected | 0.095 | -0.100 | 0.290 | 0.340 |
| ENZRXN0.2 | fructose-6-phosphate aldolase | 0 | Not detected | 0.075 | -0.078 | 0.227 | 0.336 |
| ENZRXN0.2 | tRNA m7G46 methyltransferase | 0 | Not detected | 0.179 | -0.046 | 0.405 | 0.119 |
| ENZRXN0.2 | 10-formyltetrahydrofolate:L-methion | 0 | Not detected | -0.174 | -0.415 | 0.068 | 0.159 |
| ENZRXN0.2 | tRNA (Gm18) 2'-O-methyltransferas | 0 | Not detected | 0.036 | -0.118 | 0.190 | 0.644 |

| ENZRXN0.3 | RNase R | 0 | Not detected | 0.057 | -0.188 | 0.303 | 0.648 |
| --- | --- | --- | --- | --- | --- | --- | --- |
| ENZRXN0.3 | fructokinase | 0 | Not detected | 0.057 | -0.104 | 0.218 | 0.489 |
| ENZRXN0.3 | tRNA 2-selenouridine synthase | 0 | Not detected | 0.114 | -0.058 | 0.287 | 0.195 |
| ENZRXN0.3 | endo-1,4-D-glucanase | 0 | Not detected | 0.072 | -0.102 | 0.246 | 0.416 |
| ENZRXN0.3 | UDP-N-acetylmuramate:L-alanyl-& | 0 | Not detected | -0.077 | -0.329 | 0.174 | 0.547 |
| ENZRXN0.3 | 23S rRNA m5U747 methyltransferas | 0 | Not detected | -0.046 | -0.237 | 0.144 | 0.633 |
| ENZRXN0.3 | indoleglycerol phosphate aldolase | 0 | Not detected | 0.007 | -0.158 | 0.173 | 0.929 |
| ENZRXN0.3 | L-threonine:H+ antiporter | 0 | Not detected | 0.028 | -0.181 | 0.237 | 0.793 |
| ENZRXN0.3 | L-homoserine:H+ antiporter | 0 | Not detected | 0.028 | -0.181 | 0.237 | 0.793 |
| ENZRXN0.3 | Fe2+:H+ symporter | 0 | Not detected | -0.004 | -0.171 | 0.163 | 0.963 |
| ENZRXN0.3 | N-acetylmuramyl-L-alanine amidase | 0 | Not detected | -0.106 | -0.289 | 0.077 | 0.255 |
| ENZRXN0.3 | 2-O-a-mannosyl-D-glycerate specific | 0 | Not detected | 0.004 | -0.180 | 0.189 | 0.963 |
| ENZRXN0.3 | tRNA pseudouridine13 synthase | 0 | Not detected | 0.123 | -0.048 | 0.295 | 0.159 |
| ENZRXN0.3 | tRNA pseudouridine55 synthase | 0 | Not detected | -0.073 | -0.254 | 0.107 | 0.426 |
| ENZRXN0.3 | tRNA pseudouridine65 synthase | 0 | Not detected | -0.028 | -0.191 | 0.134 | 0.731 |
| ENZRXN0.3 | methionine aminopeptidase | 0 | Not detected | 0.056 | -0.205 | 0.318 | 0.674 |
| ENZRXN0.3 | peptide deformylase | 0 | Not detected | -0.038 | -0.236 | 0.161 | 0.711 |
| ENZRXN0.3 | DNA polymerase | 0 | Not detected | 0.137 | -0.045 | 0.319 | 0.141 |
| ENZRXN0.3 | 16S rRNA m62A1518,m62A1519 di | 0 | Not detected | 0.102 | -0.102 | 0.305 | 0.328 |
| ENZRXN0.3 | undecaprenyl pyrophosphate phosph | 0 | Not detected | -0.029 | -0.232 | 0.175 | 0.780 |
| ENZRXN0.3 | 16S rRNA m5C967 methyltransferas | 0 | Not detected | 0.027 | -0.183 | 0.237 | 0.801 |
| ENZRXN0.3 | leader peptidase | 0 | Not detected | 0.105 | -0.055 | 0.265 | 0.197 |
| ENZRXN0.3 | topoisomerase | 0 | Not detected | 0.112 | -0.140 | 0.364 | 0.384 |
| ENZRXN0.4 | topoisomerase | 0 | Not detected | -0.070 | -0.277 | 0.136 | 0.504 |
| ENZRXN0.4 | methylene-tetrahydrofolylpolyglutam | 0 | Not detected | -0.030 | -0.217 | 0.157 | 0.755 |
| ENZRXN0.4 | 2,3-dihydroxybenzoate:oxygen oxid | 0 | Not detected | 0.175 | -0.016 | 0.366 | 0.072 |
| ENZRXN0.4 | cuprous oxidase | 0 | Not detected | 0.175 | -0.016 | 0.366 | 0.072 |
| ENZRXN0.4 | uridine nucleosidase | 0 | Not detected | -0.020 | -0.192 | 0.152 | 0.824 |
| ENZRXN0.4 | oligopeptidase | 0 | Not detected | 0.099 | -0.086 | 0.284 | 0.293 |
| ENZRXN0.4 | 23S rRNA 2'-O-ribose U2552 methy | 0 | Not detected | 0.074 | -0.113 | 0.261 | 0.437 |
| ENZRXN0.4 | oligopeptidase | 0 | Not detected | 0.049 | -0.202 | 0.299 | 0.703 |
| ENZRXN0.4 | tail-specific protease | 0 | Not detected | -0.137 | -0.353 | 0.078 | 0.212 |
| ENZRXN0.4 | protease | 0 | Not detected | 0.078 | -0.150 | 0.306 | 0.502 |
| ENZRXN0.4 | alanyl aminopeptidase | 0 | Not detected | -0.023 | -0.255 | 0.209 | 0.848 |
| ENZRXN0.4 | alkaline phosphatase aminopeptidase | 0 | Not detected | 0.191 | 0.024 | 0.357 | 0.025 |
| ENZRXN0.4 | peptidyl-dipeptidase | 0 | Not detected | 0.196 | 0.009 | 0.382 | 0.040 |
| ENZRXN0.4 | formate dehydrogenase | 0 | Not detected | -0.106 | -0.348 | 0.136 | 0.391 |
| ENZRXN0.4 | meso-diaminopimelic acid-D-alanyl | 0 | Not detected | 0.075 | -0.113 | 0.263 | 0.434 |
| ENZRXN0.4 | D-alanyl-D-alanine carboxypeptidas | 0 | Not detected | 0.119 | -0.047 | 0.284 | 0.160 |
| ENZRXN0.4 | exoribonuclease II | 0 | Not detected | 0.003 | -0.213 | 0.220 | 0.976 |
| ENZRXN0.5 | methionine-S-sulfoxide reductase | 0 | Not detected | 0.112 | -0.107 | 0.332 | 0.315 |
| ENZRXN0.5 | 5'-nucleotidase | 0 | Not detected | 0.077 | -0.098 | 0.252 | 0.388 |
| ENZRXN0.5 | 3'-nucleotidase | 0 | Not detected | 0.077 | -0.098 | 0.252 | 0.388 |
| ENZRXN0.5 | pyrimidine 5'-nucleotidase | 0 | Not detected | 0.036 | -0.118 | 0.191 | 0.647 |

| ENZRXN0.5 | &gamma;-glutamylputrescine oxidas | 0 | Not detected | 0.160 | -0.014 | 0.334 | 0.072 |
| --- | --- | --- | --- | --- | --- | --- | --- |
| ENZRXN0.5 | &gamma;-glutamyl-&gamma;-amino | 0 | Not detected | 0.115 | -0.061 | 0.290 | 0.201 |
| ENZRXN0.5 | octanoul-ACP synthetase | 0 | Not detected | 0.093 | -0.116 | 0.302 | 0.381 |
| ENZRXN0.5 | 4-aminobutyrate aminotransferase | 0 | Not detected | -0.088 | -0.268 | 0.092 | 0.337 |
| ENZRXN0.5 | endochitinase | 0 | Not detected | 0.051 | -0.163 | 0.264 | 0.642 |
| ENZRXN0.5 | serine/threonine:Na+ symporter | 0 | Not detected | -0.028 | -0.140 | 0.084 | 0.628 |
| ENZRXN0.5 | ribonuclease | 0 | Not detected | 0.021 | -0.137 | 0.178 | 0.796 |
| ENZRXN0.5 | RNase | 0 | Not detected | -0.043 | -0.326 | 0.239 | 0.764 |
| ENZRXN0.5 | methylglyoxal reductase | 0 | Not detected | -0.090 | -0.204 | 0.023 | 0.119 |
| ENZRXN0.5 | methylglyoxal reductase | 0 | Not detected | -0.003 | -0.165 | 0.158 | 0.970 |
| ENZRXN0.5 | methylglyoxal reductase | 0 | Not detected | -0.088 | -0.353 | 0.176 | 0.513 |
| ENZRXN0.5 | Kdo2-lipid VIA phosphoethanolami | 0 | Not detected | 0.141 | -0.065 | 0.346 | 0.180 |
| ENZRXN0.5 | anhydro-N-acetylmuramic acid kinas | 0 | Not detected | -0.003 | -0.181 | 0.175 | 0.975 |
| ENZRXN0.5 | RNase | 0 | Not detected | 0.080 | -0.157 | 0.318 | 0.508 |
| ENZRXN0.6 | DNA polymerase | 0 | Not detected | 0.062 | -0.190 | 0.314 | 0.630 |
| ENZRXN0.6 | 3' to 5' proofreading exonuclease | 0 | Not detected | 0.032 | -0.136 | 0.200 | 0.711 |
| ENZRXN0.6 | DNA primase | 219 | Not detected | -0.237 | -0.459 | -0.015 | 0.037 |
| ENZRXN0.6 | DNA polymerase | 0 | Not detected | 0.156 | -0.057 | 0.369 | 0.152 |
| ENZRXN0.6 | DNA polymerase | 0 | Not detected | 0.156 | -0.057 | 0.369 | 0.152 |
| ENZRXN0.6 | peptidyl-prolyl cis-trans isomerase | 0 | Not detected | -0.036 | -0.220 | 0.147 | 0.698 |
| ENZRXN0.6 | cAMP phosphodiesterase | 0 | Not detected | -0.032 | -0.207 | 0.143 | 0.719 |
| ENZRXN0.6 | DNA polymerase | 0 | Not detected | 0.058 | -0.194 | 0.310 | 0.653 |
| ENZRXN0.6 | 3' to 5' proofreading exonuclease | 0 | Not detected | 0.058 | -0.194 | 0.310 | 0.653 |
| ENZRXN0.6 | 5' to 3' exonuclease | 0 | Not detected | 0.058 | -0.194 | 0.310 | 0.653 |
| ENZRXN0.6 | ADP-heptose:Kdo2-lipid IVA-hepto | 0 | Not detected | 0.131 | -0.054 | 0.316 | 0.166 |
| ENZRXN0.6 | ADP-heptose&mdash;LPS heptosylt | 0 | Not detected | -0.009 | -0.201 | 0.184 | 0.930 |
| ENZRXN0.6 | isopentenyl-adenosine tRNA methyl | 0 | Not detected | -0.086 | -0.336 | 0.165 | 0.502 |
| ENZRXN0.6 | xanthine:H+ symporter XanP | 0 | Not detected | -0.178 | -0.321 | -0.036 | 0.014 |
| ENZRXN0.6 | 1-deoxy-D-xylulose 5-phosphate:thi | 0 | Not detected | -0.092 | -0.210 | 0.027 | 0.130 |
| ENZRXN0.6 | octanoate-non-lipoylated apo domai | 0 | Not detected | 0.030 | -0.128 | 0.187 | 0.710 |
| ENZRXN0.6 | 16S rRNA m5C1407 methyltransfer | 0 | Not detected | 0.033 | -0.143 | 0.208 | 0.716 |
| ENZRXN0.6 | ß-D-glucoside glucohydrolase | 0 | Not detected | 0.101 | -0.105 | 0.307 | 0.337 |
| ENZRXN0.6 | ADP-heptose:Kdo2-lipid A heptosyl | 0 | Not detected | 0.131 | -0.054 | 0.316 | 0.166 |
| ENZRXN0.6 | transport of D-serine | 0 | Not detected | 0.066 | -0.155 | 0.288 | 0.558 |
| ENZRXN0.6 | methylglyoxal reductase | 0 | Not detected | -0.049 | -0.324 | 0.227 | 0.730 |
| ENZRXN0.6 | 5-methylaminomethyl-2-thiouridine- | 0 | Not detected | 0.022 | -0.176 | 0.220 | 0.828 |
| ENZRXN0.6 | 2-isopropylmalate synthase | 0 | Not detected | -0.133 | -0.275 | 0.010 | 0.069 |
| ENZRXN0.6 | 6-phosphogluconate phosphatase | 0 | Not detected | -0.018 | -0.174 | 0.139 | 0.825 |
| ENZRXN0.6 | sugar phosphatase | 0 | Not detected | 0.140 | -0.038 | 0.319 | 0.124 |
| ENZRXN0.6 | pyridoxal phosphatase | 0 | Not detected | 0.015 | -0.150 | 0.179 | 0.862 |
| ENZRXN0.6 | phosphosugar phosphatase | 0 | Not detected | 0.015 | -0.150 | 0.179 | 0.862 |
| ENZRXN0.6 | 23S rRNA m2G1835 methyltransfer | 0 | Not detected | -0.010 | -0.187 | 0.167 | 0.913 |
| ENZRXN0.6 | 23S rRNA m2G2445 methyltransfer | 0 | Not detected | 0.205 | -0.021 | 0.430 | 0.075 |
| ENZRXN0.6 | 3 methyl-adenine DNA glycosylase | 0 | Not detected | -0.204 | -0.394 | -0.014 | 0.036 |

| ENZRXN0.6 | peptidyl-prolyl cis-trans isomerase | 0 | Not detected | 0.091 | -0.121 | 0.303 | 0.399 |
| --- | --- | --- | --- | --- | --- | --- | --- |
| ENZRXN0.6 | lysine 2,3-aminomutase | 0 | Not detected | 0.009 | -0.182 | 0.201 | 0.923 |
| ENZRXN0.6 | alanyl aminopeptidase | 0 | Not detected | -0.023 | -0.255 | 0.209 | 0.848 |
| ENZRXN0.6 | D-malate dehydrogenase (decarboxy | 0 | Not detected | -0.196 | -0.425 | 0.032 | 0.092 |
| ENZRXN0.6 | poly(A) polymerase | 0 | Not detected | -0.065 | -0.261 | 0.130 | 0.514 |
| ENZRXN0.6 | 2-oxobutanoate aminotransferase | 0 | Not detected | 0.115 | -0.068 | 0.299 | 0.218 |
| ENZRXN0.6 | exodeoxyribonuclease I | 0 | Not detected | -0.040 | -0.238 | 0.158 | 0.693 |
| ENZRXN0.6 | transport of ß-alanine | 0 | Not detected | 0.066 | -0.155 | 0.288 | 0.558 |
| ENZRXN0.6 | transport of L-alanine | 0 | Not detected | 0.066 | -0.155 | 0.288 | 0.558 |
| ENZRXN0.6 | transport of D-cycloserine | 0 | Not detected | 0.066 | -0.155 | 0.288 | 0.558 |
| ENZRXN0.6 | 3' &rarr; 5' exonuclease | 0 | Not detected | 0.055 | -0.105 | 0.216 | 0.500 |
| ENZRXN0.6 | Zn2+-exporting ATPase | 0 | Not detected | 0.022 | -0.201 | 0.245 | 0.847 |
| ENZRXN0.6 | S-ribosylhomocysteine lyase | 0 | Not detected | 0.074 | -0.111 | 0.259 | 0.433 |
| ENZRXN0.6 | 16S rRNA m2G966 methyltransferas | 0 | Not detected | 0.067 | -0.090 | 0.225 | 0.403 |
| ENZRXN0.6 | D-gluconate transporter | 0 | Not detected | 0.076 | -0.110 | 0.263 | 0.422 |
| ENZRXN0.6 | high-affinity gluconate transporter | 0 | Not detected | 0.254 | 0.024 | 0.484 | 0.031 |
| ENZRXN0.6 | 2-O-(6-phospho-a-mannosyl)-D-glyc | 0 | Not detected | -0.190 | -0.395 | 0.015 | 0.070 |
| ENZRXN0.6 | putrescine:H+ symporter | 0 | Not detected | -0.120 | -0.387 | 0.148 | 0.381 |
| ENZRXN0.6 | putrescine:L-ornithine antiporter | 0 | Not detected | -0.120 | -0.387 | 0.148 | 0.381 |
| ENZRXN0.6 | cadaverine:H+ symporter | 0 | Not detected | 0.282 | 0.041 | 0.524 | 0.022 |
| ENZRXN0.6 | serine/threonine protein kinase | 0 | Not detected | 0.193 | 0.021 | 0.364 | 0.028 |
| ENZRXN0.6 | tRNA pseudouridine32 synthase | 0 | Not detected | -0.127 | -0.300 | 0.046 | 0.150 |
| ENZRXN0.6 | transport of GlcNAc-1,6-anhMurNA | 0 | Not detected | 0.123 | -0.059 | 0.304 | 0.186 |
| ENZRXN0.6 | 1,6-anhydro-N-acetylmuramoyl-L-al | 0 | Not detected | 0.205 | 0.028 | 0.383 | 0.024 |
| ENZRXN0.6 | glcNAc-1,6-anhMurNAcaminidase | 0 | Not detected | 0.028 | -0.157 | 0.213 | 0.767 |
| ENZRXN0.6 | galactonate:H+ symporter | 0 | Not detected | 0.098 | -0.093 | 0.289 | 0.314 |
| ENZRXN0.6 | L-galactonate oxidoreductase | 0 | Not detected | 0.107 | -0.071 | 0.285 | 0.239 |
| ENZRXN0.6 | transport of L-aspartate | 0 | Not detected | -0.118 | -0.368 | 0.132 | 0.356 |
| ENZRXN0.6 | succinate:fumarate antiporter | 0 | Not detected | 0.156 | -0.044 | 0.357 | 0.127 |
| ENZRXN0.6 | 16S rRNA m7G527 methyltransferas | 0 | Not detected | 0.021 | -0.152 | 0.193 | 0.815 |
| ENZRXN0.6 | transport of L-lyxose | 0 | Not detected | -0.018 | -0.193 | 0.156 | 0.836 |
| ENZRXN0.6 | L-idarate epimerase | 0 | Not detected | -0.154 | -0.394 | 0.086 | 0.209 |
| ENZRXN0.6 | nitrate:nitrite antiporter | 0 | Not detected | 0.054 | -0.111 | 0.220 | 0.521 |
| ENZRXN0.6 | nitrate:nitrite antiporter | 0 | Not detected | 0.058 | -0.109 | 0.225 | 0.495 |
| ENZRXN0.6 | biotin transporter | 0 | Not detected | -0.009 | -0.177 | 0.160 | 0.920 |
| ENZRXN0.6 | arbutin 6-phosphate glucohydrolase | 0 | Not detected | 0.078 | -0.166 | 0.322 | 0.532 |
| ENZRXN0.6 | arbutin 6-phosphate glucohydrolase | 0 | Not detected | -0.040 | -0.148 | 0.068 | 0.464 |
| ENZRXN0.6 | arbutin-6-phosphate glucohydrolase | 0 | Not detected | -0.049 | -0.192 | 0.093 | 0.498 |
| ENZRXN0.6 | salicin 6-phosphate glucohydrolase | 0 | Not detected | 0.078 | -0.166 | 0.322 | 0.532 |
| ENZRXN0.6 | transport of N-acetyl-ß-D-glucosami | 0 | Not detected | 0.123 | -0.059 | 0.304 | 0.186 |
| ENZRXN0.6 | 23S rRNA m2A2503 methyltransfer | 0 | Not detected | 0.031 | -0.225 | 0.287 | 0.814 |
| ENZRXN0.6 | transport of a tripeptide | 0 | Not detected | 0.140 | -0.026 | 0.306 | 0.099 |
| ENZRXN0.6 | protein kinase | 0 | Not detected | 0.112 | -0.080 | 0.304 | 0.254 |
| ENZRXN0.6 | L-2-hydroxyglutarate dehydrogenase | 0 | Not detected | 0.015 | -0.164 | 0.194 | 0.871 |

| ENZRXN0.6 | undecaprenyl-pyrophosphate pyroph | 0 | Not detected | -0.054 | -0.279 | 0.170 | 0.635 |
| --- | --- | --- | --- | --- | --- | --- | --- |
| ENZRXN0.6 | NAD(P)H:quinone oxidoreductase | 0 | Not detected | 0.045 | -0.137 | 0.227 | 0.628 |
| ENZRXN0.6 | anthranilate phosphoribosyl transfera | 0 | Not detected | 0.111 | -0.082 | 0.304 | 0.260 |
| ENZRXN0.6 | nigerose hydrolase | 0 | Not detected | 0.139 | -0.065 | 0.342 | 0.182 |
| ENZRXN0.6 | peptidoglycan glycosyltransferase | 0 | Not detected | 0.195 | -0.012 | 0.402 | 0.065 |
| ENZRXN0.6 | peptidoglycan glycosyltransferase | 0 | Not detected | 0.044 | -0.140 | 0.228 | 0.639 |
| ENZRXN0.6 | murein transpeptidase | 0 | Not detected | -0.041 | -0.269 | 0.187 | 0.725 |
| ENZRXN0.6 | glycerol-2-phosphatase | 0 | Not detected | 0.086 | -0.113 | 0.284 | 0.398 |
| ENZRXN0.6 | L-glyceraldehyde 3-phosphate reduc | 0 | Not detected | -0.090 | -0.204 | 0.023 | 0.119 |
| ENZRXN0.6 | poly-ß-1,6-N-acetyl-D-glucosamine | 0 | Not detected | 0.155 | -0.036 | 0.345 | 0.112 |
| ENZRXN0.6 | tRNAMet cytidine acetyltransferase | 0 | Not detected | 0.083 | -0.116 | 0.283 | 0.415 |
| ENZRXN0.6 | ribosomal protein L11 methyltransfe | 0 | Not detected | -0.035 | -0.238 | 0.167 | 0.733 |
| ENZRXN0.6 | glutamyl-Q tRNA(Asp) synthetase | 0 | Not detected | 0.000 | -0.167 | 0.166 | 0.996 |
| ENZRXN0.6 | ThiS adenylyltransferase | 0 | Not detected | 0.129 | -0.032 | 0.289 | 0.117 |
| ENZRXN0.6 | adenylate:persulfide transferase | 0 | Not detected | 0.129 | -0.032 | 0.289 | 0.117 |
| ENZRXN0.6 | 3-hydroxypropionaldehyde dehydrog | 0 | Not detected | 0.115 | -0.061 | 0.290 | 0.201 |
| ENZRXN0.6 | lipid III flippase | 0 | Not detected | 0.071 | -0.124 | 0.265 | 0.477 |
| ENZRXN0.6 | glucose dehydrogenase | 0 | Not detected | 0.132 | -0.089 | 0.354 | 0.243 |
| ENZRXN0.6 | autoinducer-2 kinase | 0 | Not detected | 0.182 | -0.015 | 0.379 | 0.071 |
| ENZRXN0.6 | lipid II flippase | 0 | Not detected | 0.040 | -0.142 | 0.221 | 0.670 |
| ENZRXN0.6 | GTPase | 0 | Not detected | 0.062 | -0.143 | 0.267 | 0.556 |
| ENZRXN0.6 | transport of a dipeptide | 0 | Not detected | 0.031 | -0.164 | 0.226 | 0.757 |
| ENZRXN0.6 | acyl-[acp]:1-acyl-sn-glycerol-3-phos | 0 | Not detected | 0.010 | -0.160 | 0.180 | 0.909 |
| ENZRXN0.6 | CTP:phosphatidate cytidyltransferas | 0 | Not detected | 0.118 | -0.036 | 0.271 | 0.133 |
| ENZRXN0.6 | acyl-acp:sn-glycerol-3-phosphate 1- | 0 | Not detected | -0.051 | -0.292 | 0.189 | 0.677 |
| ENZRXN0.6 | 23S rRNA 2'-O-ribose C2498 methy | 0 | Not detected | 0.088 | -0.100 | 0.276 | 0.358 |
| ENZRXN0.7 | fructoselysine transporter | 0 | Not detected | 0.012 | -0.244 | 0.267 | 0.929 |
| ENZRXN0.7 | psicoselysine transporter | 0 | Not detected | 0.012 | -0.244 | 0.267 | 0.929 |
| ENZRXN0.7 | L-ribulose-5-phosphate 4-epimerase | 0 | Not detected | -0.053 | -0.170 | 0.063 | 0.370 |
| ENZRXN0.7 | L-ribulose-5-phosphate 3-epimerase | 0 | Not detected | -0.032 | -0.259 | 0.195 | 0.782 |
| ENZRXN0.7 | transport of a dipeptide | 0 | Not detected | 0.045 | -0.141 | 0.230 | 0.635 |
| ENZRXN0.7 | non-chaperonin molecular chaperon | 0 | Not detected | 0.057 | -0.190 | 0.303 | 0.653 |
| ENZRXN0.7 | protein methylthiotransferase | 0 | Not detected | 0.064 | -0.184 | 0.312 | 0.614 |
| ENZRXN0.7 | dipeptide transport | 0 | Not detected | 0.007 | -0.177 | 0.192 | 0.939 |
| ENZRXN0.7 | (S)-ureidoglycine aminohydrolase | 0 | Not detected | 0.088 | -0.133 | 0.309 | 0.436 |
| ENZRXN0.7 | chitobiose transport | 0 | Not detected | 0.090 | -0.088 | 0.268 | 0.322 |
| ENZRXN0.7 | transport of adenine | 0 | Not detected | -0.009 | -0.211 | 0.193 | 0.929 |
| ENZRXN0.7 | guanosine 3'-diphosphate 5'-triphosp | 0 | Not detected | -0.051 | -0.300 | 0.199 | 0.692 |
| ENZRXN0.7 | GTPase | 0 | Not detected | 0.096 | -0.168 | 0.360 | 0.478 |
| ENZRXN0.7 | GTPase | 0 | Not detected | 0.202 | -0.007 | 0.412 | 0.059 |
| ENZRXN0.7 | carbamoyl--[HypE] ligase | 0 | Not detected | 0.127 | -0.096 | 0.350 | 0.265 |
| ENZRXN0.7 | 2-iminoacetate synthase | 0 | Not detected | 0.037 | -0.145 | 0.218 | 0.690 |
| ENZRXN0.7 | transport of malate | 0 | Not detected | -0.118 | -0.368 | 0.132 | 0.356 |
| ENZRXN0.7 | methionine-oxo-acid transaminase | 0 | Not detected | 0.155 | -0.021 | 0.331 | 0.084 |

| ENZRXN0.7 | p-aminobenzoyl glutamate:H+ symp | 0 | Not detected | -0.064 | -0.233 | 0.105 | 0.457 |
| --- | --- | --- | --- | --- | --- | --- | --- |
| ENZRXN0.7 | uracil oxygenase | 0 | Not detected | -0.034 | -0.198 | 0.131 | 0.687 |
| ENZRXN0.7 | RNA helicase | 0 | Not detected | 0.055 | -0.117 | 0.226 | 0.533 |
| ENZRXN0.7 | exoribonuclease II | 0 | Not detected | 0.003 | -0.213 | 0.220 | 0.976 |
| ENZRXN0.7 | peptidoglycan DD transpeptidase | 0 | Not detected | 0.136 | -0.086 | 0.358 | 0.229 |
| ENZRXN0.7 | L-aspartate oxidase | 0 | Not detected | -0.001 | -0.285 | 0.282 | 0.993 |
| ENZRXN0.7 | exoribonuclease II | 0 | Not detected | 0.003 | -0.213 | 0.220 | 0.976 |
| ENZRXN0.7 | adenosine nucleosidase | 0 | Not detected | -0.020 | -0.192 | 0.152 | 0.824 |
| ENZRXN0.7 | xanthosine nucleosidase | 0 | Not detected | -0.020 | -0.192 | 0.152 | 0.824 |
| ENZRXN0.7 | inosine nucleosidase | 0 | Not detected | -0.020 | -0.192 | 0.152 | 0.824 |
| ENZRXN0.7 | cytidine nucleosidase | 0 | Not detected | -0.020 | -0.192 | 0.152 | 0.824 |
| ENZRXN0.7 | dihydroorotate dehydrogenase (MQ) | 0 | Not detected | -0.037 | -0.314 | 0.240 | 0.795 |
| ENZRXN0.7 | transport of 3-hydroxy-trans-cinnam | 0 | Not detected | -0.054 | -0.229 | 0.122 | 0.548 |
| ENZRXN0.7 | epoxyqueuosine reductase | 0 | Not detected | -0.021 | -0.204 | 0.161 | 0.819 |
| ENZRXN0.7 | putrescine:H+ symporter PlaP | 0 | Not detected | 0.119 | -0.058 | 0.295 | 0.188 |
| ENZRXN0.7 | acyl-[acp]:1-acyl-sn-glycerol-3-phos | 0 | Not detected | 0.010 | -0.160 | 0.180 | 0.909 |
| ENZRXN0.7 | putative inorganic phosphate export | 0 | Not detected | 0.060 | -0.144 | 0.263 | 0.566 |
| ENZRXN0.7 | S-methyl-L-methionine transporter | 0 | Not detected | 0.091 | -0.179 | 0.361 | 0.511 |
| ENZRXN0.7 | NADHX epimerase | 0 | Not detected | 0.108 | -0.091 | 0.307 | 0.288 |
| ENZRXN0.7 | (S)-NADHX dehydratase | 0 | Not detected | 0.108 | -0.091 | 0.307 | 0.288 |
| ENZRXN0.7 | transport of inosine | 0 | Not detected | 0.313 | 0.101 | 0.524 | 0.004 |
| ENZRXN0.7 | transport of deoxyinosine | 0 | Not detected | 0.313 | 0.101 | 0.524 | 0.004 |
| ENZRXN0.7 | 16S rRNA m2G1516 methyltransfer | 0 | Not detected | -0.033 | -0.207 | 0.141 | 0.713 |
| ENZRXN0.7 | a-D-ribose-1-methylphosphonate-5-t | 0 | Not detected | 0.165 | -0.022 | 0.351 | 0.083 |
| ENZRXN0.7 | carbon-phosphorous lyase | 0 | Not detected | 0.171 | -0.044 | 0.386 | 0.119 |
| ENZRXN0.7 | peptidoglycan DD-carboxypeptidase | 0 | Not detected | -0.060 | -0.235 | 0.116 | 0.505 |
| ENZRXN0.7 | peptidoglycan DD-endopeptidase | 0 | Not detected | -0.060 | -0.235 | 0.116 | 0.505 |
| ENZRXN0.7 | ferric enterobactin esterase | 0 | Not detected | 0.031 | -0.140 | 0.202 | 0.720 |
| ENZRXN0.7 | (S)-lactate transporter | 0 | Not detected | 0.045 | -0.199 | 0.288 | 0.719 |
| ENZRXN0.7 | 23S rRNA m7G2069 methyltransfer | 0 | Not detected | 0.205 | -0.021 | 0.430 | 0.075 |
| ENZRXN0.7 | enterobactin exporter EntS | 0 | Not detected | -0.004 | -0.187 | 0.178 | 0.965 |
| ENZRXN0.7 | D-tartrate:succinate antiporter | 0 | Not detected | 0.043 | -0.162 | 0.248 | 0.681 |
| ENZRXN0.7 | GTPase | 0 | Not detected | -0.095 | -0.334 | 0.143 | 0.433 |
| ENZRXN0.7 | fructose-6-phosphate:phosphate anti | 0 | Not detected | 0.102 | -0.093 | 0.297 | 0.304 |
| ENZRXN0.7 | mannose-6-phosphate:phosphate ant | 0 | Not detected | 0.102 | -0.093 | 0.297 | 0.304 |
| ENZRXN0.7 | propionate transporter | 0 | Not detected | 0.004 | -0.246 | 0.253 | 0.976 |
| ENZRXN0.7 | metalloprotease | 0 | Not detected | 0.121 | -0.054 | 0.297 | 0.176 |
| ENZRXN0.7 | (R)-lactate transporter | 0 | Not detected | 0.123 | -0.090 | 0.335 | 0.257 |
| ENZRXN0.7 | glycolate transporter | 0 | Not detected | 0.123 | -0.090 | 0.335 | 0.257 |
| ENZRXN0.7 | (R)-lactate transporter | 0 | Not detected | 0.045 | -0.199 | 0.288 | 0.719 |
| ENZRXN0.7 | transport of a C4-dicarboxylate | 0 | Not detected | -0.118 | -0.368 | 0.132 | 0.356 |
| ENZRXN0.7 | methyl ß-D-glucoside 6-phosphate g | 0 | Not detected | -0.040 | -0.148 | 0.068 | 0.464 |
| ENZRXN0.7 | D-galactarate transporter | 0 | Not detected | 0.083 | -0.097 | 0.262 | 0.366 |
| ENZRXN0.7 | D-glucarate transporter | 0 | Not detected | 0.083 | -0.097 | 0.262 | 0.366 |

| ENZRXN0.7 | D-glycerate transporter | 0 | Not detected | 0.083 | -0.097 | 0.262 | 0.366 |
| --- | --- | --- | --- | --- | --- | --- | --- |
| ENZRXN0.7 | propionyl-CoA synthetase | 0 | Not detected | 0.001 | -0.195 | 0.198 | 0.989 |
| ENZRXN0.7 | 23S rRNA m6A2030 methyltransfer | 0 | Not detected | 0.014 | -0.163 | 0.191 | 0.875 |
| ENZRXN0.7 | tRNA m2A37 methyltransferase | 0 | Not detected | 0.031 | -0.225 | 0.287 | 0.814 |
| ENZRXN0.7 | inorganic triphosphatase | 0 | Not detected | 0.202 | 0.019 | 0.385 | 0.030 |
| ENZRXN0.7 | L-glyceraldehyde-3-phosphate:phosp | 0 | Not detected | 0.102 | -0.093 | 0.297 | 0.304 |
| ENZRXN0.7 | 6-phospho-ß-D-glucosyl-(1,4)-D-glu | 0 | Not detected | -0.049 | -0.192 | 0.093 | 0.498 |
| ENZRXN0.7 | aldehyde reductase | 0 | Not detected | 0.103 | -0.057 | 0.262 | 0.208 |
| ENZRXN0.7 | aldehyde reductase | 0 | Not detected | -0.027 | -0.203 | 0.149 | 0.761 |
| ENZRXN0.7 | 2,5-diketo-D-gluconate reductase | 0 | Not detected | -0.003 | -0.165 | 0.158 | 0.970 |
| ENZRXN0.7 | cysteinylglycine dipeptidase | 0 | Not detected | -0.023 | -0.255 | 0.209 | 0.848 |
| ENZRXN0.7 | UDP-N-acetylmuramate:L-alanyl-& | 0 | Not detected | -0.077 | -0.329 | 0.174 | 0.547 |
| ENZRXN0.7 | transport of nitrite | 0 | Not detected | 0.058 | -0.109 | 0.225 | 0.495 |
| ENZRXN0.7 | 6-phospho-ß-D-glucosyl-(1-4)-D-glu | 0 | Not detected | 0.078 | -0.166 | 0.322 | 0.532 |
| ENZRXN0.7 | guanosine 2'3'-cyclic phosphodiester | 0 | Not detected | 0.029 | -0.201 | 0.259 | 0.806 |
| ENZRXN0.7 | uridine 2'3'-cyclic phosphodiesterase | 0 | Not detected | 0.029 | -0.201 | 0.259 | 0.806 |
| ENZRXN0.7 | cytidine 2'3'-cyclic phosphodiesteras | 0 | Not detected | 0.029 | -0.201 | 0.259 | 0.806 |
| ENZRXN0.7 | adenosine 2'3'-cyclic phosphodiester | 0 | Not detected | 0.029 | -0.201 | 0.259 | 0.806 |
| ENZRXN0.7 | cytidine 3'-phosphate phosphohydro | 0 | Not detected | 0.077 | -0.098 | 0.252 | 0.388 |
| ENZRXN0.7 | adenosine 3'-phosphate phosphohydr | 0 | Not detected | 0.077 | -0.098 | 0.252 | 0.388 |
| ENZRXN0.7 | L-1-glycerophophorylethanolamine | 0 | Not detected | 0.101 | -0.057 | 0.258 | 0.212 |
| ENZRXN0.7 | dGMP 5'-phosphate phosphohydrola | 0 | Not detected | 0.077 | -0.098 | 0.252 | 0.388 |
| ENZRXN0.7 | arsenate:H+ symporter | 0 | Not detected | 0.059 | -0.144 | 0.261 | 0.570 |
| ENZRXN0.7 | glycerophosphoserine phosphodieste | 0 | Not detected | 0.101 | -0.057 | 0.258 | 0.212 |
| ENZRXN0.7 | glycerophosphoglycerol phosphodie | 0 | Not detected | 0.101 | -0.057 | 0.258 | 0.212 |
| ENZRXN0.7 | succinate transporter | 0 | Not detected | 0.183 | -0.023 | 0.388 | 0.082 |
| ENZRXN0.7 | fumarate transporter | 0 | Not detected | 0.183 | -0.023 | 0.388 | 0.082 |
| ENZRXN0.7 | aspartate transport | 0 | Not detected | 0.183 | -0.023 | 0.388 | 0.082 |
| ENZRXN0.7 | amylomaltase | 0 | Not detected | 0.089 | -0.133 | 0.310 | 0.432 |
| ENZRXN0.7 | amylomaltase | 0 | Not detected | 0.089 | -0.133 | 0.310 | 0.432 |
| ENZRXN0.7 | maltopentaose glucosidase | 0 | Not detected | 0.074 | -0.121 | 0.269 | 0.457 |
| ENZRXN0.7 | maltohexaose glucosidase | 0 | Not detected | 0.074 | -0.121 | 0.269 | 0.457 |
| ENZRXN0.7 | maltoheptaose glucosidase | 0 | Not detected | 0.074 | -0.121 | 0.269 | 0.457 |
| ENZRXN0.7 | GTPase | 0 | Not detected | 0.126 | -0.046 | 0.297 | 0.151 |
| ENZRXN0.7 | Na+:H+ antiporter | 0 | Not detected | -0.003 | -0.170 | 0.164 | 0.971 |
| ENZRXN0.7 | K+:H+ antiporter | 0 | Not detected | -0.003 | -0.170 | 0.164 | 0.971 |
| ENZRXN0.7 | phosphoethanolamine transferase | 0 | Not detected | 0.109 | -0.086 | 0.304 | 0.275 |
| ENZRXN0.7 | phosphoethanolamine transferase | 0 | Not detected | 0.073 | -0.122 | 0.268 | 0.462 |
| ENZRXN0.7 | cytidine 3' nucleotidase | 0 | Not detected | 0.029 | -0.201 | 0.259 | 0.806 |
| ENZRXN0.7 | guanosine 3' nucleotidase | 0 | Not detected | 0.029 | -0.201 | 0.259 | 0.806 |
| ENZRXN0.7 | adenosine 3' nucleotidase | 0 | Not detected | 0.029 | -0.201 | 0.259 | 0.806 |
| ENZRXN0.7 | uridine 3' nucleotidase | 0 | Not detected | 0.029 | -0.201 | 0.259 | 0.806 |
| ENZRXN0.7 | 5'-deoxyribonucleotidase | 0 | Not detected | -0.012 | -0.229 | 0.205 | 0.915 |
| ENZRXN0.7 | 2-octaprenylphenol hydroxylase | 0 | Not detected | 0.111 | -0.066 | 0.288 | 0.220 |

| ENZRXN0.7 | DNA-AP site lyase | 0 | Not detected | 0.084 | -0.177 | 0.344 | 0.529 |
| --- | --- | --- | --- | --- | --- | --- | --- |
| ENZRXN0.7 | transport of D-glucose | 0 | Not detected | 0.048 | -0.136 | 0.231 | 0.612 |
| ENZRXN0.7 | AMP kinase (GTP) | 0 | Not detected | -0.099 | -0.218 | 0.020 | 0.104 |
| ENZRXN0.7 | protein-lysine deacetylase | 0 | Not detected | 0.218 | 0.021 | 0.415 | 0.030 |
| ENZRXN0.7 | transport of a dipeptide | 0 | Not detected | 0.140 | -0.026 | 0.306 | 0.099 |
| ENZRXN0.7 | transport of glucose | 0 | Not detected | 0.061 | -0.129 | 0.252 | 0.529 |
| ENZRXN0.7 | transport of maltose | 0 | Not detected | 0.061 | -0.129 | 0.252 | 0.529 |
| ENZRXN0.7 | guanine transport | 0 | Not detected | -0.019 | -0.225 | 0.187 | 0.856 |
| ENZRXN0.7 | hypoxanthine transport | 0 | Not detected | -0.019 | -0.225 | 0.187 | 0.856 |
| ENZRXN0.7 | 5-aminomethyl-2-thiouridine syntha | 0 | Not detected | 0.022 | -0.176 | 0.220 | 0.828 |
| ENZRXN0.7 | phosphoethanolamine transferase | 0 | Not detected | 0.078 | -0.107 | 0.263 | 0.408 |
| ENZRXN0.7 | 6-deoxy-6-sulfofructose kinase | 0 | Not detected | 0.132 | -0.041 | 0.306 | 0.135 |
| ENZRXN0.7 | 6-deoxy-6-sulfofructose-1-phosphate | 0 | Not detected | 0.221 | 0.047 | 0.395 | 0.013 |
| ENZRXN0.7 | sulfoquinovose transporter | 0 | Not detected | 0.031 | -0.170 | 0.231 | 0.764 |
| ENZRXN0.7 | uracil:H+ symporter | 0 | Not detected | 0.091 | -0.161 | 0.343 | 0.479 |
| ENZRXN0.7 | transport of a tripeptide | 0 | Not detected | 0.031 | -0.164 | 0.226 | 0.757 |
| ENZRXN0.7 | transport of a tripeptide | 0 | Not detected | 0.045 | -0.141 | 0.230 | 0.635 |
| ENZRXN0.7 | L-threonine dehydrogenase | 0 | Not detected | 0.037 | -0.219 | 0.293 | 0.776 |
| ENZRXN0.7 | 11-deoxycorticosterone reductase | 0 | Not detected | 0.051 | -0.129 | 0.230 | 0.580 |
| ENZRXN0.7 | D-gluconate 5-dehydrogenase | 0 | Not detected | 0.051 | -0.129 | 0.230 | 0.580 |
| ENZRXN0.7 | 2-keto-3-deoxy-D-gluconate dehydr | 0 | Not detected | 0.051 | -0.129 | 0.230 | 0.580 |
| ENZRXN0.7 | sodium dodecyl sulfatase | 0 | Not detected | 0.073 | -0.131 | 0.278 | 0.483 |
| ENZRXN0.7 | isobutyraldehyde reductase | 0 | Not detected | 0.103 | -0.057 | 0.262 | 0.208 |
| ENZRXN0.7 | isobutyraldehyde reductase | 0 | Not detected | -0.003 | -0.165 | 0.158 | 0.970 |
| ENZRXN0.7 | isobutyraldehyde reductase | 0 | Not detected | -0.151 | -0.396 | 0.095 | 0.230 |
| ENZRXN0.7 | 1-hydroxy-2-methyl-2-(E)-butenyl 4 | 0 | Not detected | -0.071 | -0.184 | 0.042 | 0.219 |
| ENZRXN0.7 | 3' &rarr; 5' ssDNA exonuclease | 0 | Not detected | 0.070 | -0.092 | 0.232 | 0.397 |
| ENZRXN0.7 | 3' &rarr; 5' RNA exonuclease | 0 | Not detected | 0.070 | -0.092 | 0.232 | 0.397 |
| ENZRXN0.7 | ATP pyrophosphatase | 0 | Not detected | 0.024 | -0.191 | 0.238 | 0.829 |
| ENZRXN0.7 | 3-isopropylmalate dehydrogenase (d | 0 | Not detected | -0.196 | -0.425 | 0.032 | 0.092 |
| ENZRXN0.7 | L-tartrate dehydrogenase | 0 | Not detected | -0.196 | -0.425 | 0.032 | 0.092 |
| ENZRXN0.7 | glycerophosphoglycerol acyltransfer | 0 | Not detected | 0.139 | -0.036 | 0.314 | 0.120 |
| ENZRXN0.7 | NADH:menaquinone oxidoreductase | 0 | Not detected | 0.109 | -0.082 | 0.299 | 0.263 |
| ENZRXN0.7 | polyphenol oxidase | 0 | Not detected | 0.050 | -0.119 | 0.219 | 0.559 |
| ENZRXN0.7 | PII-UMP deuridylylation | 0 | Not detected | 0.115 | -0.104 | 0.334 | 0.303 |
| ENZRXN0.7 | D-ribulose 5-phosphate 1-dehydroge | 0 | Not detected | 0.208 | -0.003 | 0.420 | 0.054 |
| ENZRXN0.7 | flavin mononucleotide (FMN) expor | 0 | Not detected | -0.033 | -0.205 | 0.140 | 0.712 |
| ENZRXN0.7 | flavin adenine dinucleotide (FAD) e | 0 | Not detected | -0.033 | -0.205 | 0.140 | 0.712 |
| ENZRXN0.7 | L,D-carboxypeptidase | 0 | Not detected | 0.127 | -0.063 | 0.318 | 0.191 |
| ENZRXN0.7 | L,D-transpeptidase | 0 | Not detected | 0.127 | -0.063 | 0.318 | 0.191 |
| ENZRXN0.7 | L,D transpeptidase | 0 | Not detected | 0.053 | -0.125 | 0.232 | 0.558 |
| ENZRXN0.7 | L,D-transpeptidase | 0 | Not detected | 0.127 | -0.063 | 0.318 | 0.191 |
| ENZRXN0.7 | 5' &rarr; 3' exonuclease | 0 | Not detected | 0.039 | -0.124 | 0.203 | 0.638 |
| ENZRXN0.7 | 3',5'-nucleoside bisphosphate phosph | 0 | Not detected | 0.039 | -0.124 | 0.203 | 0.638 |

| ENZRXN0.7 | ATPase | 0 | Not detected | -0.057 | -0.171 | 0.057 | 0.326 |
| --- | --- | --- | --- | --- | --- | --- | --- |
| ENZRXN0.7 | acyl-acp:sn-glycerol-3-phosphate 1- | 0 | Not detected | -0.051 | -0.292 | 0.189 | 0.677 |
| ENZRXN0.7 | acyl-acp:sn-glycerol-3-phosphate 1- | 0 | Not detected | -0.051 | -0.292 | 0.189 | 0.677 |
| ENZRXN0.7 | acyl-acp:sn-glycerol-3-phosphate 1- | 0 | Not detected | -0.051 | -0.292 | 0.189 | 0.677 |
| ENZRXN0.7 | acyl-[acp]:1-acyl-sn-glycerol-3-phos | 0 | Not detected | 0.010 | -0.160 | 0.180 | 0.909 |
| ENZRXN0.7 | acyl-[acp]:1-acyl-sn-glycerol-3-phos | 0 | Not detected | 0.010 | -0.160 | 0.180 | 0.909 |
| ENZRXN0.7 | acyl-[acp]:1-acyl-sn-glycerol-3-phos | 0 | Not detected | 0.010 | -0.160 | 0.180 | 0.909 |
| ENZRXN0.7 | acyl-[acp]:1-acyl-sn-glycerol-3-phos | 0 | Not detected | 0.010 | -0.160 | 0.180 | 0.909 |
| ENZRXN0.7 | acyl-[acp]:1-acyl-sn-glycerol-3-phos | 0 | Not detected | 0.010 | -0.160 | 0.180 | 0.909 |
| ENZRXN0.8 | magnesium hydrogenphosphate:H+ s | 0 | Not detected | 0.059 | -0.144 | 0.261 | 0.570 |
| ENZRXN0.8 | calcium hydrogenphosphate:H+ sym | 0 | Not detected | 0.059 | -0.144 | 0.261 | 0.570 |
| ENZRXN0.8 | zinc hydrogenphosphate:H+ symport | 0 | Not detected | 0.059 | -0.144 | 0.261 | 0.570 |
| ENZRXN0.8 | tellurite:H+ symporter | 0 | Not detected | 0.059 | -0.144 | 0.261 | 0.570 |
| ENZRXN0.8 | L-methionine:H+ antiport | 0 | Not detected | 0.081 | -0.101 | 0.263 | 0.384 |
| ENZRXN0.8 | L-leucine:H+ antiport | 0 | Not detected | 0.081 | -0.101 | 0.263 | 0.384 |
| ENZRXN0.8 | L-isoleucine:H+ antiport | 0 | Not detected | 0.081 | -0.101 | 0.263 | 0.384 |
| ENZRXN0.8 | L-valine:H+ antiport | 0 | Not detected | 0.081 | -0.101 | 0.263 | 0.384 |
| ENZRXN0.8 | farnesyl diphosphate phosphatase | 0 | Not detected | -0.029 | -0.232 | 0.175 | 0.780 |
| ENZRXN0.8 | L-cystine:H+ symporter | 0 | Not detected | 0.172 | -0.016 | 0.360 | 0.074 |
| ENZRXN0.8 | D-cystine:H+ symporter | 0 | Not detected | 0.172 | -0.016 | 0.360 | 0.074 |
| ENZRXN0.8 | homocystine:H+ symporter | 0 | Not detected | 0.172 | -0.016 | 0.360 | 0.074 |
| ENZRXN0.8 | apolipoprotein N-acyltransferase | 38 | Not detected | 0.219 | 0.034 | 0.403 | 0.020 |
| ENZRXN0.8 | peptidoglycan lytic exotransglycosyl | 0 | Not detected | 0.099 | -0.073 | 0.271 | 0.261 |
| ENZRXN0.8 | peptidoglycan lytic exotransglycosyl | 0 | Not detected | 0.018 | -0.150 | 0.187 | 0.830 |
| ENZRXN0.8 | peptidoglycan lytic exotransglycosyl | 0 | Not detected | 0.084 | -0.116 | 0.285 | 0.409 |
| ENZRXN0.8 | peptidoglycan lytic endotransglycosy | 0 | Not detected | 0.084 | -0.116 | 0.285 | 0.409 |
| ENZRXN0.8 | peptidoglycan lytic endotransglycosy | 0 | Not detected | 0.018 | -0.150 | 0.187 | 0.830 |
| ENZRXN0.8 | peptidoglycan lytic exotransglycosyl | 0 | Not detected | 0.086 | -0.093 | 0.264 | 0.346 |
| ENZRXN0.8 | peptidoglycan lytic endotransglycosy | 0 | Not detected | 0.086 | -0.093 | 0.264 | 0.346 |
| ENZRXN0.8 | peptidoglycan lytic endotransglycosy | 0 | Not detected | 0.109 | -0.097 | 0.314 | 0.301 |
| ENZRXN0.8 | peptidoglycan lytic exotransglycosyl | 0 | Not detected | 0.109 | -0.097 | 0.314 | 0.301 |
| ENZRXN0.8 | peptidoglycan lytic exotransglycosyl | 0 | Not detected | -0.011 | -0.203 | 0.180 | 0.909 |
| ENZRXN0.8 | GTPase, ribosome-stimulated | 0 | Not detected | -0.066 | -0.179 | 0.047 | 0.253 |
| ENZRXN0.8 | transport of 2-acyl-1-lysophosphatid | 0 | Not detected | 0.175 | -0.003 | 0.353 | 0.054 |
| ENZRXN0.8 | transport of lyso-phosphatidylglycer | 0 | Not detected | 0.175 | -0.003 | 0.353 | 0.054 |
| ENZRXN0.8 | poly-3-hydroxybutyrate synthase | 0 | Not detected | 0.107 | -0.056 | 0.270 | 0.198 |
| ENZRXN0.8 | biotin-ATP ligase | 0 | Not detected | 0.178 | 0.004 | 0.352 | 0.046 |
| ENZRXN0.8 | sulfoquinovosidase | 0 | Not detected | -0.110 | -0.398 | 0.178 | 0.455 |
| ENZRXN0.8 | sulfoquinovosidase | 0 | Not detected | -0.110 | -0.398 | 0.178 | 0.455 |
| ENZRXN0.8 | (S)-2-hydroxybutanoate dehydrogen | 0 | Not detected | 0.123 | -0.082 | 0.328 | 0.241 |
| ENZRXN0.8 | (S)-2-hydroxybutanoate transporter | 0 | Not detected | 0.123 | -0.090 | 0.335 | 0.257 |
| ENZRXN0.8 | (S)-2-hydroxybutanoate transporter | 0 | Not detected | 0.045 | -0.199 | 0.288 | 0.719 |
| ENZRXN0.8 | choline dehydrogenase | 0 | Not detected | 0.200 | -0.005 | 0.404 | 0.056 |
| ENZRXN0.8 | betaine aldehyde dehydrogenase | 0 | Not detected | 0.200 | -0.005 | 0.404 | 0.056 |

| ENZRXN0.8 | short chain acyl-CoA synthetase (tra | 0 | Not detected | 0.206 | -0.018 | 0.430 | 0.072 |
| --- | --- | --- | --- | --- | --- | --- | --- |
| ENZRXN0.8 | cis-vaccenoyl-CoA synthetase (trans | 0 | Not detected | 0.206 | -0.018 | 0.430 | 0.072 |
| ENZRXN0.8 | oleoyl-CoA synthetase (transporting | 0 | Not detected | 0.206 | -0.018 | 0.430 | 0.072 |
| ENZRXN0.8 | inositol monophosphatase | 0 | Not detected | 0.086 | -0.113 | 0.284 | 0.398 |
| ENZRXN0.8 | fumarate:H+ symporter | 0 | Not detected | 0.043 | -0.162 | 0.248 | 0.681 |
| ENZRXN0.8 | fumarate:H+ symporter | 0 | Not detected | 0.156 | -0.044 | 0.357 | 0.127 |
| ENZRXN0.8 | acyl-CoA dehydrogenase | 0 | Not detected | 0.140 | -0.075 | 0.355 | 0.203 |
| ENZRXN0.8 | acyl-CoA dehydrogenase | 0 | Not detected | 0.140 | -0.075 | 0.355 | 0.203 |
| ENZRXN0.8 | acyl-CoA dehydrogenase | 0 | Not detected | 0.140 | -0.075 | 0.355 | 0.203 |
| ENZRXN0.8 | acyl-CoA dehydrogenase | 0 | Not detected | 0.140 | -0.075 | 0.355 | 0.203 |
| ENZRXN0.8 | acyl-CoA dehydrogenase | 0 | Not detected | 0.140 | -0.075 | 0.355 | 0.203 |
| ENZRXN0.8 | acyl-CoA dehydrogenase | 0 | Not detected | 0.140 | -0.075 | 0.355 | 0.203 |
| ENZRXN0.8 | acyl-CoA dehydrogenase | 0 | Not detected | 0.140 | -0.075 | 0.355 | 0.203 |
| ENZRXN0.8 | enoyl-CoA hydratase | 0 | Not detected | 0.105 | -0.096 | 0.306 | 0.304 |
| ENZRXN0.8 | enoyl-CoA hydratase | 0 | Not detected | 0.105 | -0.096 | 0.306 | 0.304 |
| ENZRXN0.8 | enoyl-CoA hydratase | 0 | Not detected | 0.105 | -0.096 | 0.306 | 0.304 |
| ENZRXN0.8 | enoyl-CoA hydratase | 0 | Not detected | 0.105 | -0.096 | 0.306 | 0.304 |
| ENZRXN0.8 | enoyl-CoA hydratase | 0 | Not detected | 0.105 | -0.096 | 0.306 | 0.304 |
| ENZRXN0.8 | enoyl-CoA hydratase | 0 | Not detected | 0.105 | -0.096 | 0.306 | 0.304 |
| ENZRXN0.8 | enoyl-CoA hydratase | 0 | Not detected | 0.105 | -0.096 | 0.306 | 0.304 |
| ENZRXN0.8 | 3-hydroxyacyl-CoA dehydrogenase | 0 | Not detected | 0.105 | -0.096 | 0.306 | 0.304 |
| ENZRXN0.8 | 3-hydroxyacyl-CoA dehydrogenase | 0 | Not detected | 0.105 | -0.096 | 0.306 | 0.304 |
| ENZRXN0.8 | 3-hydroxyacyl-CoA dehydrogenase | 0 | Not detected | 0.105 | -0.096 | 0.306 | 0.304 |
| ENZRXN0.8 | 3-hydroxyacyl-CoA dehydrogenase | 0 | Not detected | 0.105 | -0.096 | 0.306 | 0.304 |
| ENZRXN0.8 | 3-hydroxyacyl-CoA dehydrogenase | 0 | Not detected | 0.105 | -0.096 | 0.306 | 0.304 |
| ENZRXN0.8 | 3-hydroxyacyl-CoA dehydrogenase | 0 | Not detected | 0.105 | -0.096 | 0.306 | 0.304 |
| ENZRXN0.8 | 3-hydroxyacyl-CoA dehydrogenase | 0 | Not detected | 0.105 | -0.096 | 0.306 | 0.304 |
| ENZRXN0.8 | acetyl-CoA C-acyltransferase | 0 | Not detected | 0.031 | -0.148 | 0.210 | 0.735 |
| ENZRXN0.8 | acetyl-CoA C-acyltransferase | 0 | Not detected | 0.031 | -0.148 | 0.210 | 0.735 |
| ENZRXN0.8 | acetyl-CoA C-acyltransferase | 0 | Not detected | 0.031 | -0.148 | 0.210 | 0.735 |
| ENZRXN0.8 | acetyl-CoA C-acyltransferase | 0 | Not detected | 0.031 | -0.148 | 0.210 | 0.735 |
| ENZRXN0.8 | acetyl-CoA C-acyltransferase | 0 | Not detected | 0.031 | -0.148 | 0.210 | 0.735 |
| ENZRXN0.8 | acetyl-CoA C-acyltransferase | 0 | Not detected | 0.031 | -0.148 | 0.210 | 0.735 |
| ENZRXN0.8 | acetyl-CoA C-acyltransferase | 0 | Not detected | 0.031 | -0.148 | 0.210 | 0.735 |
| ENZRXN0.8 | palmitoleoyl-CoA synthetase (transp | 0 | Not detected | 0.206 | -0.018 | 0.430 | 0.072 |
| ENZRXN0.8 | fumarate:H+ symport | 0 | Not detected | 0.001 | -0.238 | 0.240 | 0.996 |
| ENZRXN0.8 | succinate:H+ symporter | 0 | Not detected | 0.156 | -0.044 | 0.357 | 0.127 |
| ENZRXN0.8 | methylphosphotriester-DNA-protein | 0 | Not detected | 0.132 | -0.037 | 0.301 | 0.125 |
| ENZRXN0.8 | O-4-methylthymine-DNA methyltran | 0 | Not detected | 0.132 | -0.037 | 0.301 | 0.125 |
| ENZRXN0.8 | ferredoxin-NADP+ reductase | 0 | Not detected | 0.138 | -0.019 | 0.295 | 0.086 |
| ENZRXN0.8 | 2,6-dimethoxyphenol:oxygen oxidor | 0 | Not detected | 0.175 | -0.016 | 0.366 | 0.072 |
| ENZRXN0.8 | 4,5-DOPA dioxygenase extradiol | 0 | Not detected | 0.019 | -0.138 | 0.176 | 0.809 |
| ENZRXN0.8 | ssDNA-specific exonuclease | 0 | Not detected | 0.002 | -0.219 | 0.224 | 0.983 |
| ENZRXN0.8 | nitrophenyl phosphatase | 0 | Not detected | -0.012 | -0.229 | 0.205 | 0.915 |

| ENZRXN0.8 | diguanylate cyclase | 0 | Not detected | 0.184 | 0.015 | 0.354 | 0.033 |
| --- | --- | --- | --- | --- | --- | --- | --- |
| ENZRXN0.8 | P1,P3-bis(5'-adenosyl) triphosphate | 0 | Not detected | -0.012 | -0.229 | 0.205 | 0.915 |
| ENZRXN0.8 | CDP-glycerol hydrolase | 0 | Not detected | -0.012 | -0.229 | 0.205 | 0.915 |
| ENZRXN0.8 | tRNATyr pseudouridine35 synthase | 0 | Not detected | -0.104 | -0.258 | 0.051 | 0.188 |
| ENZRXN0.8 | GTPase | 0 | Not detected | 0.028 | -0.180 | 0.236 | 0.794 |
| ENZRXN0.8 | hydroxyphenyllactate dehydrogenase | 0 | Not detected | -0.029 | -0.194 | 0.135 | 0.728 |
| ENZRXN0.8 | recombination-promoting nuclease | 0 | Not detected | 0.201 | -0.015 | 0.418 | 0.068 |
| ENZRXN0.8 | S-sulfocysteine:H+ symporter | 0 | Not detected | 0.172 | -0.016 | 0.360 | 0.074 |
| ENZRXN0.8 | periplasmic nitrite reductase | 0 | Not detected | 0.037 | -0.175 | 0.249 | 0.732 |
| ENZRXN0.8 | phosphatidylglycerol synthase | 0 | Not detected | 0.236 | 0.071 | 0.402 | 0.005 |
| ENZRXN0.8 | signal peptide peptidase | 0 | Not detected | 0.072 | -0.136 | 0.280 | 0.498 |
| ENZRXN0.8 | lipoprotein signal peptide peptidase | 0 | Not detected | 0.072 | -0.136 | 0.280 | 0.498 |
| ENZRXN0.8 | L-cysteine desulfidase | 0 | Not detected | 0.029 | -0.173 | 0.232 | 0.777 |
| ENZRXN0.8 | rhomboid protease | 0 | Not detected | 0.104 | -0.059 | 0.267 | 0.212 |
| ENZRXN0.8 | 3' to 5' ATP-dependent DNA helicas | 0 | Not detected | 0.137 | -0.102 | 0.377 | 0.261 |
| ENZRXN0.8 | 5' to 3' ATP-dependent DNA helicas | 0 | Not detected | 0.133 | -0.071 | 0.338 | 0.202 |
| ENZRXN0.8 | cytochrome c peroxidase | 0 | Not detected | -0.026 | -0.243 | 0.192 | 0.818 |
| ENZRXN0.8 | ß-barrel assembly-enhancing proteas | 0 | Not detected | 0.050 | -0.140 | 0.240 | 0.609 |
| ENZRXN0.8 | D-alanyl-D-alanine carboxypeptidas | 0 | Not detected | 0.119 | -0.047 | 0.284 | 0.160 |
| ENZRXN0.8 | D-alanyl-D-alanine carboxypeptidas | 0 | Not detected | 0.119 | -0.047 | 0.284 | 0.160 |
| ENZRXN0.8 | ß-lactamase | 0 | Not detected | 0.119 | -0.047 | 0.284 | 0.160 |
| ENZRXN0.8 | 2-acyl lysophospholipase | 0 | Not detected | 0.139 | -0.036 | 0.314 | 0.120 |
| ENZRXN0.8 | 1-acyl lysophospholipase | 0 | Not detected | 0.139 | -0.036 | 0.314 | 0.120 |
| ENZRXN0.8 | pyruvate:H+ symporter | 0 | Not detected | 0.163 | -0.082 | 0.408 | 0.193 |
| ENZRXN0.8 | Na+ : H+ antiporter | 0 | Not detected | -0.031 | -0.216 | 0.154 | 0.743 |
| ENZRXN0.8 | K+ : H+ antiporter | 0 | Not detected | -0.031 | -0.216 | 0.154 | 0.743 |
| ENZRXN0.8 | dequalinium:H+ antiporter | 0 | Not detected | -0.003 | -0.170 | 0.164 | 0.971 |
| ENZRXN0.8 | ethidium:H+ antiporter | 0 | Not detected | -0.003 | -0.170 | 0.164 | 0.971 |
| ENZRXN0.8 | glucosylglycerate phosphorylase | 0 | Not detected | 0.079 | -0.100 | 0.258 | 0.388 |
| ENZRXN0.8 | carbonylcyanide m-chlorophenylhyd | 0 | Not detected | 0.104 | -0.078 | 0.285 | 0.263 |
| ENZRXN0.8 | bicozamycin:H+ antiporter | 0 | Not detected | -0.079 | -0.249 | 0.090 | 0.359 |
| ENZRXN0.8 | chloramphenicol:H+ antiporter | 0 | Not detected | 0.085 | -0.104 | 0.274 | 0.377 |
| ENZRXN0.8 | fosfomycin:H+ antiporter | 0 | Not detected | 0.026 | -0.154 | 0.207 | 0.776 |
| ENZRXN0.8 | Fe2+ transporter | 0 | Not detected | 0.160 | -0.064 | 0.384 | 0.162 |
| ENZRXN0.8 | trimethylamine N-oxide reductase | 0 | Not detected | 0.036 | -0.165 | 0.237 | 0.727 |
| ENZRXN0.8 | trimethylamine N-oxide reductase | 0 | Not detected | 0.036 | -0.160 | 0.231 | 0.722 |
| ENZRXN0.8 | UDP kinase | 0 | Not detected | -0.099 | -0.218 | 0.020 | 0.104 |
| ENZRXN0.8 | dTDP kinase | 0 | Not detected | -0.099 | -0.218 | 0.020 | 0.104 |
| ENZRXN0.8 | CDP kinase | 0 | Not detected | -0.099 | -0.218 | 0.020 | 0.104 |
| ENZRXN0.8 | dCDP kinase | 0 | Not detected | -0.099 | -0.218 | 0.020 | 0.104 |
| ENZRXN0.8 | dGDP kinase | 0 | Not detected | -0.099 | -0.218 | 0.020 | 0.104 |
| ENZRXN0.8 | GDP kinase | 0 | Not detected | -0.099 | -0.218 | 0.020 | 0.104 |
| ENZRXN0.8 | celulose phosphoethanolamine trans | 0 | Not detected | 0.165 | -0.035 | 0.366 | 0.106 |
| ENZRXN0.8 | thymine:H+ symporter | 0 | Not detected | 0.091 | -0.161 | 0.343 | 0.479 |

| ENZRXN0.8 | xanthine:H+ symporter | 0 | Not detected | 0.091 | -0.161 | 0.343 | 0.479 |
| --- | --- | --- | --- | --- | --- | --- | --- |
| ENZRXN0.8 | c-di-GMP phosphodiesterase | 0 | Not detected | 0.182 | -0.010 | 0.375 | 0.063 |
| ENZRXN0.8 | apolipoprotein N-acyltransferase | 38 | Not detected | 0.219 | 0.034 | 0.403 | 0.020 |
| ENZRXN0.8 | hydroquinone:hydrogen peroxide ox | 0 | Not detected | -0.026 | -0.243 | 0.192 | 0.818 |
| ENZRXN0.8 | menadiol:hydrogen peroxide oxidore | 0 | Not detected | -0.026 | -0.243 | 0.192 | 0.818 |
| ENZRXN0.8 | kojibiose phosphorylase | 0 | Not detected | 0.153 | -0.041 | 0.346 | 0.122 |
| ENZRXN0.8 | endo-poly-ß-1,6-D-glucosamine hyd | 0 | Not detected | 0.155 | -0.036 | 0.345 | 0.112 |
| ENZRXN0.8 | L-aspartate:fumarate antiport | 0 | Not detected | 0.001 | -0.238 | 0.240 | 0.996 |
| ENZRXN0.8 | thiol-disulfide exchange | 0 | Not detected | 0.232 | 0.025 | 0.438 | 0.028 |
| ENZRXN0.8 | ferric enterobactin esterase | 0 | Not detected | 0.031 | -0.140 | 0.202 | 0.720 |
| ENZRXN0.8 | thiol-disulfide exchange | 0 | Not detected | 0.232 | 0.025 | 0.438 | 0.028 |
| ENZRXN0.8 | 5-aminovalerate aminotransferase | 0 | Not detected | -0.088 | -0.268 | 0.092 | 0.337 |
| ENZRXN0.8 | protein-histidine auto-kinase | 0 | Not detected | 0.128 | -0.053 | 0.309 | 0.166 |
| ENZRXN0.8 | ATP-dependent 3' &rarr; 5" helicase | 0 | Not detected | 0.040 | -0.192 | 0.271 | 0.736 |
| ENZRXN0.8 | 5-aminopentanoate:H+ symporter | 0 | Not detected | 0.027 | -0.150 | 0.205 | 0.762 |
| ENZRXN0.8 | 5' &rarr; 3' helicase | 0 | Not detected | 0.153 | -0.052 | 0.357 | 0.144 |
| ENZRXN0.8 | apurinic/apyrimidinic endonuclease | 0 | Not detected | 0.055 | -0.105 | 0.216 | 0.500 |
| ENZRXN0.8 | 3'-phosphodiesterase | 0 | Not detected | 0.055 | -0.105 | 0.216 | 0.500 |
| ENZRXN0.8 | 3'-phosphomonoesterase | 0 | Not detected | 0.055 | -0.105 | 0.216 | 0.500 |
| ENZRXN0.8 | uracil DNA glycosylase | 0 | Not detected | 0.084 | -0.177 | 0.344 | 0.529 |
| ENZRXN0.8 | thymine glycol DNA glycosylase | 0 | Not detected | 0.084 | -0.177 | 0.344 | 0.529 |
| ENZRXN0.8 | protein-histidine autokinase | 0 | Not detected | 0.030 | -0.157 | 0.217 | 0.754 |
| ENZRXN185 | 1,4-alpha-glucan branching enzyme | 0 | Not detected | -0.026 | -0.228 | 0.175 | 0.798 |
| ENZRXN185 | phosphoglucomutase | 0 | Not detected | 0.269 | -0.037 | 0.575 | 0.085 |
| ENZRXN1F. | glutamate-1-semialdehyde aminotran | 0 | Not detected | -0.034 | -0.329 | 0.262 | 0.823 |
| ENZRXN3O. | aconitate hydratase | 0 | Not detected | 0.293 | -0.114 | 0.700 | 0.159 |
| ENZRXN3O. | aconitate hydratase | 0 | Not detected | 0.293 | -0.114 | 0.700 | 0.159 |
| ENZRXN490 | enolase | 0 | Not detected | -0.062 | -0.327 | 0.202 | 0.645 |
| ENZRXN66. | pyruvate carboxylase | 0 | Not detected | -0.029 | -0.282 | 0.224 | 0.822 |
| ENZRXN66. | citrate dehydratase | 0 | Not detected | -0.130 | -0.364 | 0.104 | 0.277 |
| ENZRXN66. | aconitate hydratase | 0 | Not detected | -0.130 | -0.364 | 0.104 | 0.277 |
| ENZRXN8J2 | 1-deoxyxylulose-5-phosphate syntha | 0 | Not detected | -0.004 | -0.307 | 0.299 | 0.980 |
| ENZRXN8J2 | GTP cyclohydrolase | 0 | Not detected | -0.018 | -0.176 | 0.140 | 0.824 |
| ENZRXN8J2 | orotate phosphoribosyltransferase | 0 | Not detected | -0.058 | -0.186 | 0.070 | 0.375 |
| ENZRXN8J2 | orotidine 5'-phosphate decarboxylas | 0 | Not detected | 0.010 | -0.159 | 0.180 | 0.905 |
| ENZRXN8J2 | aspartate carbamoyltransferase | 0 | Not detected | -0.003 | -0.275 | 0.269 | 0.981 |
| ENZRXN8J2 | UDP-glucose 6-dehydrogenase | 0 | Not detected | -0.122 | -0.364 | 0.120 | 0.325 |
| ENZRXN8J2 | 5-dehydro-2-deoxy-D-gluconate kin | 0 | Not detected | -0.064 | -0.315 | 0.188 | 0.620 |
| ENZRXN8J2 | methionine-glutamine aminotransfer | 0 | Not detected | -0.010 | -0.235 | 0.215 | 0.930 |
| ENZRXN8J2 | lipoyl synthase | 0 | Not detected | -0.270 | -0.497 | -0.042 | 0.020 |
| ENZRXN8J2 | 4-nitrobenzaldehyde reductase | 0 | Not detected | -0.109 | -0.351 | 0.134 | 0.380 |
| ENZRXN8J2 | CTP synthetase | 0 | Not detected | -0.050 | -0.167 | 0.068 | 0.407 |
| ENZRXN8J2 | UTP-glucose-1-phosphate uridylyltr | 0 | Not detected | -0.046 | -0.228 | 0.136 | 0.619 |
| ENZRXN8J2 | 1,5-a-L-arabinofuranosidase | 0 | Not detected | -0.033 | -0.234 | 0.169 | 0.752 |

| ENZRXN8J2 | 1,3-a-L-arabinofuranosidase | 0 | Not detected | -0.054 | -0.183 | 0.075 | 0.410 |
| --- | --- | --- | --- | --- | --- | --- | --- |
| ENZRXNI.14 | fumarase | 0 | Not detected | -0.136 | -0.362 | 0.090 | 0.238 |
| ENZRXNIO2 | alcohol dehydrogenase | 0 | Not detected | -0.107 | -0.345 | 0.130 | 0.377 |
| ENZRXNME | oxepin-CoA hydrolase | 0 | Not detected | 0.010 | -0.180 | 0.199 | 0.920 |
| ENZRXNME | 3-oxo-5,6-dehydrosuberyl-CoA semi | 0 | Not detected | 0.010 | -0.180 | 0.199 | 0.920 |
| ENZRXNQT | phosphomethylpyrimidine synthase | 0 | Not detected | 0.135 | -0.120 | 0.390 | 0.298 |
| ENZRXNQT | biotin carboxylase | 0 | Not detected | -0.134 | -0.271 | 0.003 | 0.056 |
| ENZRXNQT | H+-exporting diphosphatase | 0 | Not detected | -0.026 | -0.292 | 0.241 | 0.850 |
| ENZRXNQT | NADP+-dependent isocitrate dehydr | 0 | Not detected | -0.054 | -0.287 | 0.178 | 0.646 |
| ENZRXNQT | tryptophan synthase | 0 | Not detected | 0.124 | -0.129 | 0.378 | 0.336 |
| ENZRXNQT | aconitase | 0 | Not detected | 0.097 | -0.167 | 0.360 | 0.473 |
| ENZRXNQT | 3,4 dihydroxy-2-butanone-4-phosph | 0 | Not detected | -0.078 | -0.198 | 0.043 | 0.206 |
| ENZRXNQT | GTP cyclohydrolase | 0 | Not detected | -0.078 | -0.198 | 0.043 | 0.206 |
| ENZRXNT9. | aconitase | 0 | Not detected | 0.097 | -0.167 | 0.360 | 0.473 |
| FADSYN.EN | FMN adenylyltransferase | 0 | Not detected | 0.003 | -0.179 | 0.184 | 0.978 |
| FGAMSYN.E | phosphoribosylformylglycinamide sy | 0 | Not detected | 0.117 | -0.093 | 0.327 | 0.276 |
| FLAVONAD | flavodoxin-NADP+ reductase | 0 | Not detected | 0.138 | -0.019 | 0.295 | 0.086 |
| FOLYLPOLY | folylpoly-&gamma;-glutamate synth | 0 | Not detected | -0.030 | -0.217 | 0.157 | 0.755 |
| FORMYLTH | 10-formyl-tetrahydrofolylpolyglutam | 0 | Not detected | -0.030 | -0.217 | 0.157 | 0.755 |
| FPPSYN.EN | farnesyl diphosphate synthase | 0 | Not detected | 0.056 | -0.145 | 0.257 | 0.586 |
| FUCULOKIN | L-fuculokinase | 0 | Not detected | 0.000 | -0.260 | 0.260 | 0.999 |
| GALACTAR | galactarate dehydratase | 0 | Not detected | -0.014 | -0.256 | 0.227 | 0.908 |
| GALACTOK | galactokinase | 0 | Not detected | 0.157 | -0.034 | 0.348 | 0.108 |
| GALACTON | D-galactonate dehydratase | 0 | Not detected | 0.005 | -0.219 | 0.229 | 0.965 |
| GALACTUR | D-galacturonate isomerase | 0 | Not detected | -0.062 | -0.175 | 0.050 | 0.277 |
| GALPMUT.E | UDP-galactopyranose mutase | 0 | Not detected | -0.121 | -0.244 | 0.001 | 0.052 |
| GARTRANS | phosphoribosylglycinamide formyltr | 0 | Not detected | -0.102 | -0.295 | 0.091 | 0.299 |
| GARTSIDE.E | acetate kinase | 0 | Not detected | -0.102 | -0.295 | 0.091 | 0.299 |
| GCVT.ENZR | aminomethyltransferase | 0 | Not detected | -0.017 | -0.197 | 0.163 | 0.855 |
| GDPPYPHO | GDP pyrophosphokinase | 0 | Not detected | -0.100 | -0.332 | 0.132 | 0.398 |
| GDPPYPHO | GDP diphosphokinase | 0 | Not detected | -0.051 | -0.300 | 0.199 | 0.692 |
| GKI.ENZRX | glycerate 2-kinase | 0 | Not detected | 0.056 | -0.162 | 0.274 | 0.614 |
| GLNS.ENZR | glutamine&mdash;tRNA ligase | 0 | Not detected | -0.063 | -0.175 | 0.050 | 0.274 |
| GLU6PDEH | NADP+-dependent glucose-6-phosp | 0 | Not detected | 0.051 | -0.121 | 0.222 | 0.562 |
| GLUCARDE | D-glucarate dehydratase | 0 | Not detected | -0.154 | -0.394 | 0.086 | 0.209 |
| GLUCUROIS | D-glucuronate isomerase | 0 | Not detected | -0.062 | -0.175 | 0.050 | 0.277 |
| GLURS.ENZ | glutamate&mdash;tRNA ligase | 0 | Not detected | 0.083 | -0.178 | 0.344 | 0.533 |
| GLUTCYSLI | glutamate&mdash;cysteine ligase | 0 | Not detected | 0.029 | -0.161 | 0.218 | 0.766 |
| GLUTRACE. | glutamate racemase | 0 | Not detected | 0.033 | -0.130 | 0.196 | 0.691 |
| GLYCEROL. | acyl-CoA:sn-glycerol-3-phosphate 1 | 0 | Not detected | -0.051 | -0.292 | 0.189 | 0.677 |
| GLYCOGEN | 1,4-a-glucan branching enzyme | 0 | Not detected | 0.032 | -0.171 | 0.235 | 0.756 |
| GLYCOGEN | glycogen synthase | 0 | Not detected | 0.195 | -0.006 | 0.397 | 0.058 |
| GLYCPDIES | glycerophosphodiester phosphodiest | 0 | Not detected | 0.101 | -0.057 | 0.258 | 0.212 |
| GLYRIBON | phosphoribosylamine&mdash;glycin | 0 | Not detected | -0.047 | -0.172 | 0.078 | 0.458 |

| GPH.ENZRX | phosphoglycolate phosphatase | 0 | Not detected | 0.031 | -0.129 | 0.190 | 0.706 |
| --- | --- | --- | --- | --- | --- | --- | --- |
| GPPSYN.EN | geranyl diphosphate synthase | 0 | Not detected | 0.056 | -0.145 | 0.257 | 0.586 |
| GSADENYL | glutamine synthetase adenylyltransfe | 0 | Not detected | -0.106 | -0.354 | 0.142 | 0.402 |
| GSDEADEN | glutamine synthetase deadenylase | 0 | Not detected | -0.106 | -0.354 | 0.142 | 0.402 |
| GTPPYPHOS | GTP pyrophosphokinase | 0 | Not detected | -0.100 | -0.332 | 0.132 | 0.398 |
| HEMEOSYN | heme O synthase | 0 | Not detected | 0.070 | -0.099 | 0.240 | 0.417 |
| HEMN.ENZR | coproporphyrinogen III dehydrogena | 0 | Not detected | 0.225 | 0.006 | 0.444 | 0.044 |
| HISTCYCLO | phosphoribosyl-AMP cyclohydrolas | 0 | Not detected | -0.082 | -0.209 | 0.045 | 0.207 |
| HISTPRATP | phosphoribosyl-ATP pyrophosphata | 0 | Not detected | -0.082 | -0.209 | 0.045 | 0.207 |
| HOMOCYSM | cobalamin-independent homocystein | 0 | Not detected | 0.074 | -0.168 | 0.316 | 0.548 |
| HOMOCYSM | methionine synthase | 0 | Not detected | -0.068 | -0.182 | 0.046 | 0.243 |
| IGPSYN.EN | indole-3-glycerol phosphate synthas | 0 | Not detected | 0.112 | -0.082 | 0.306 | 0.257 |
| ILES.ENZRX | isoleucine&mdash;tRNA ligase | 0 | Not detected | 0.053 | -0.221 | 0.328 | 0.703 |
| IMPCYCLO | IMP cyclohydrolase | 0 | Not detected | 0.054 | -0.118 | 0.226 | 0.541 |
| KDOTRANS | 3-deoxy-D-manno-octulosonic acid t | 0 | Not detected | 0.101 | -0.101 | 0.304 | 0.328 |
| KDOTRANS | KDO transferase | 0 | Not detected | 0.101 | -0.101 | 0.304 | 0.328 |
| L.ASPARTA | L-aspartate oxidase | 0 | Not detected | -0.001 | -0.285 | 0.282 | 0.993 |
| L.LACTDEH | L-lactate dehydrogenase | 0 | Not detected | 0.123 | -0.082 | 0.328 | 0.241 |
| LAUROYLA | lauroyl acyltransferase | 0 | Not detected | 0.098 | -0.075 | 0.270 | 0.267 |
| LEUS.ENZR | leucine&mdash;tRNA ligase | 0 | Not detected | 0.037 | -0.237 | 0.311 | 0.791 |
| LSERINEDE | L-serine deaminase | 0 | Not detected | -0.172 | -0.446 | 0.103 | 0.220 |
| LSERINEDE | L-serine deaminase | 0 | Not detected | -0.008 | -0.203 | 0.187 | 0.935 |
| MALATESY | malate synthase A | 0 | Not detected | 0.068 | -0.131 | 0.267 | 0.502 |
| MALONYL. | [acyl-carrier-protein] S-malonyltrans | 0 | Not detected | 0.196 | -0.044 | 0.435 | 0.109 |
| MALSYNG.E | malate synthase G | 0 | Not detected | 0.032 | -0.169 | 0.233 | 0.756 |
| MALTODEX | maltodextrin glucosidase | 0 | Not detected | 0.074 | -0.121 | 0.269 | 0.457 |
| MANNKIN.E | mannokinase | 0 | Not detected | 0.057 | -0.104 | 0.218 | 0.489 |
| MANNOND | D-mannonate dehydratase | 0 | Not detected | -0.170 | -0.322 | -0.019 | 0.028 |
| MANNONO | D-mannonate oxidoreductase | 0 | Not detected | 0.194 | -0.028 | 0.416 | 0.087 |
| MANNPDEH | mannitol-1-phosphate 5-dehydrogen | 0 | Not detected | 0.071 | -0.176 | 0.318 | 0.572 |
| MHPHYDRO | 3-(3-hydroxyphenyl)propanoate hyd | 0 | Not detected | 0.075 | -0.112 | 0.262 | 0.431 |
| N.ACETYLG | N-acetylglutamylphosphate reductas | 0 | Not detected | 0.089 | -0.138 | 0.317 | 0.442 |
| NACGLCTR | N-acetylglucosaminyl transferase | 0 | Not detected | 0.056 | -0.128 | 0.239 | 0.552 |
| NACMURLA | N-acetylmuramoyl-L-alanine amidas | 0 | Not detected | 0.085 | -0.106 | 0.275 | 0.385 |
| NADH.DHII. | NADH:ubiquinone oxidoreductase | 0 | Not detected | 0.109 | -0.082 | 0.299 | 0.263 |
| NANK.ENZR | N-acetylmannosamine kinase | 0 | Not detected | 0.113 | -0.060 | 0.286 | 0.202 |
| NICOTINAT | nicotinate phosphoribosyltransferase | 0 | Not detected | 0.069 | -0.103 | 0.240 | 0.433 |
| O.SUCCINY | o-succinylbenzoate synthase | 0 | Not detected | 0.055 | -0.126 | 0.236 | 0.551 |
| OCTPRNL.M | 2-octaprenyl-3-methyl-6-methoxy-1, | 0 | Not detected | -0.020 | -0.197 | 0.158 | 0.827 |
| OHMETHYL | hydroxymethylbilane synthase | 0 | Not detected | 0.080 | -0.103 | 0.262 | 0.393 |
| PEPCARBO | phosphoenolpyruvate carboxykinase | 0 | Not detected | -0.071 | -0.185 | 0.043 | 0.222 |
| PGLYCERO | phosphoglycerol transferase | 0 | Not detected | 0.160 | -0.051 | 0.370 | 0.138 |
| PGMI.ENZR | 2,3-bisphosphoglycerate-independen | 0 | Not detected | -0.078 | -0.190 | 0.035 | 0.177 |
| PHOSGLUC | phosphoglucosamine mutase | 0 | Not detected | 0.139 | -0.104 | 0.382 | 0.263 |

| PHOSGLYC | phosphoglycerate kinase | 0 | Not detected | -0.049 | -0.281 | 0.184 | 0.683 |
| --- | --- | --- | --- | --- | --- | --- | --- |
| PHOSMANM | phosphomannomutase | 0 | Not detected | -0.003 | -0.273 | 0.267 | 0.984 |
| PHOSNACM | phospho-N-acetylmuramoyl-pentape | 0 | Not detected | 0.051 | -0.191 | 0.292 | 0.680 |
| PHOSPHASE | phosphatidylserine synthase | 0 | Not detected | -0.045 | -0.256 | 0.166 | 0.677 |
| PHOSPHOG | phosphoglucomutase | 0 | Not detected | -0.097 | -0.327 | 0.133 | 0.411 |
| PPENTOMU | a-D-ribose 1,5-phosphomutase | 0 | Not detected | -0.028 | -0.297 | 0.240 | 0.835 |
| PRAISOM.E | phosphoribosylanthranilate isomeras | 0 | Not detected | 0.112 | -0.082 | 0.306 | 0.257 |
| PRIBFAICA | 1-(5-phosphoribosyl)-5-[(5-phospho | 0 | Not detected | 0.116 | -0.116 | 0.347 | 0.328 |
| PROPKIN.E | propionate kinase | 0 | Not detected | 0.111 | -0.060 | 0.283 | 0.204 |
| PROPKIN2.E | propionate kinase | 0 | Not detected | 0.164 | -0.033 | 0.361 | 0.102 |
| PROTOHEM | ferrochelatase | 0 | Not detected | 0.062 | -0.125 | 0.249 | 0.517 |
| PRPPSYN.E | ribose-phosphate diphosphokinase | 0 | Not detected | -0.073 | -0.197 | 0.050 | 0.245 |
| PSERPHOSP | phosphoserine phosphatase | 0 | Not detected | 0.025 | -0.160 | 0.210 | 0.793 |
| PSII.ENZRX | guanosine-3',5'-bis(diphosphate) 3'-d | 0 | Not detected | -0.051 | -0.300 | 0.199 | 0.692 |
| RHAMNULO | rhamnulokinase | 0 | Not detected | 0.143 | -0.055 | 0.341 | 0.158 |
| RIBOFLAVI | riboflavin kinase | 0 | Not detected | 0.003 | -0.179 | 0.184 | 0.978 |
| RIBULP3EPI | ribulose-phosphate 3-epimerase | 0 | Not detected | 0.017 | -0.159 | 0.192 | 0.853 |
| SHIKIMATE | shikimate dehydrogenase | 0 | Not detected | -0.045 | -0.213 | 0.123 | 0.599 |
| SHIKIMATE | shikimate kinase | 0 | Not detected | 0.020 | -0.147 | 0.187 | 0.816 |
| SUCCGLUA | succinylglutamate semialdehyde deh | 0 | Not detected | -0.022 | -0.214 | 0.171 | 0.827 |
| TDPFUCAC | dTDP-4-amino-4,6-dideoxy-D-galac | 0 | Not detected | -0.027 | -0.183 | 0.130 | 0.737 |
| TETRAACY | tetraacyldisaccharide 4'-kinase | 0 | Not detected | 0.021 | -0.145 | 0.187 | 0.805 |
| THI.P.KIN.E | thiamine monophosphate kinase | 0 | Not detected | 0.098 | -0.079 | 0.276 | 0.278 |
| THIC.ENZR | phosphomethylpyrimidine synthase | 0 | Not detected | -0.035 | -0.154 | 0.084 | 0.566 |
| THRESYN.E | threonine synthase | 0 | Not detected | -0.113 | -0.332 | 0.106 | 0.312 |
| TRANS.ENZ | transport of arabinose | 0 | Not detected | -0.001 | -0.238 | 0.237 | 0.995 |
| TRANS.ENZ | (S)-lactate transporter | 0 | Not detected | 0.123 | -0.090 | 0.335 | 0.257 |
| TRANS.ENZ | glycolate transporter | 0 | Not detected | 0.045 | -0.199 | 0.288 | 0.719 |
| TRANS.ENZ | succinate:fumarate antiport | 0 | Not detected | 0.001 | -0.238 | 0.240 | 0.996 |
| TRANS.ENZ | fumarate:succinate antiporter | 0 | Not detected | 0.043 | -0.162 | 0.248 | 0.681 |
| TRANS.ENZ | transport of adenosine | 0 | Not detected | 0.313 | 0.101 | 0.524 | 0.004 |
| TRANS.ENZ | transport of cytidine | 0 | Not detected | 0.313 | 0.101 | 0.524 | 0.004 |
| TRANS.ENZ | transport of deoxyadenosine | 0 | Not detected | 0.313 | 0.101 | 0.524 | 0.004 |
| TRANS.ENZ | transport of deoxycytidine | 0 | Not detected | 0.313 | 0.101 | 0.524 | 0.004 |
| TRANS.ENZ | transport of deoxyuridine | 0 | Not detected | 0.313 | 0.101 | 0.524 | 0.004 |
| TRANS.ENZ | transport of thymidine | 0 | Not detected | 0.313 | 0.101 | 0.524 | 0.004 |
| TRANS.ENZ | transport of uridine | 0 | Not detected | 0.313 | 0.101 | 0.524 | 0.004 |
| TRANS.ENZ | transport of rhamnose | 0 | Not detected | -0.018 | -0.193 | 0.156 | 0.836 |
| TRANS.ENZ | phosphate:H+ symporter | 0 | Not detected | 0.059 | -0.144 | 0.261 | 0.570 |
| TRANS.ENZ | cytosine transporter | 0 | Not detected | 0.131 | -0.083 | 0.346 | 0.230 |
| TRANS.ENZ | pantothenate:Na+ symporter | 0 | Not detected | 0.148 | -0.071 | 0.367 | 0.184 |
| TRANS.ENZ | proline:Na+ symporter | 0 | Not detected | 0.004 | -0.246 | 0.253 | 0.976 |
| TRANS.ENZ | transport of succinate | 0 | Not detected | -0.118 | -0.368 | 0.132 | 0.356 |
| TRANS.ENZ | transport of malate | 0 | Not detected | -0.118 | -0.368 | 0.132 | 0.356 |

| TRANS.ENZ | transport of fumarate | 0 | Not detected | -0.118 | -0.368 | 0.132 | 0.356 |
| --- | --- | --- | --- | --- | --- | --- | --- |
| TRANS.ENZ | transport of orotate | 0 | Not detected | -0.118 | -0.368 | 0.132 | 0.356 |
| TRANS.ENZ | L-glutamate:H+ symporter | 0 | Not detected | 0.078 | -0.111 | 0.268 | 0.417 |
| TRANS.ENZ | L-aspartate:H+ symporter | 0 | Not detected | 0.078 | -0.111 | 0.268 | 0.417 |
| TRANS.ENZ | transport of glutamate | 0 | Not detected | 0.040 | -0.171 | 0.251 | 0.711 |
| TRANS.ENZ | branched chain amino acid transport | 0 | Not detected | -0.015 | -0.197 | 0.167 | 0.872 |
| TRANS.ENZ | branched chain amino acid transport | 0 | Not detected | -0.015 | -0.197 | 0.167 | 0.872 |
| TRANS.ENZ | branched chain amino acid transport | 0 | Not detected | -0.015 | -0.197 | 0.167 | 0.872 |
| TRANS.ENZ | L-tartrate:succinate antiporter | 0 | Not detected | 0.046 | -0.132 | 0.224 | 0.614 |
| TRANS.ENZ | Na+:H+ antiporter | 0 | Not detected | 0.052 | -0.170 | 0.275 | 0.646 |
| TRANS.ENZ | Mg2+ transporter | 0 | Not detected | -0.098 | -0.289 | 0.093 | 0.315 |
| TRANS.ENZ | Co2+ transporter | 0 | Not detected | -0.098 | -0.289 | 0.093 | 0.315 |
| TRANS.ENZ | Ni2+ transporter | 0 | Not detected | -0.098 | -0.289 | 0.093 | 0.315 |
| TRANS.ENZ | K+ : H+ antiporter KefB | 0 | Not detected | 0.145 | -0.061 | 0.350 | 0.167 |
| TRANS.ENZ | K+:H+ symporter Kup | 0 | Not detected | 0.058 | -0.149 | 0.264 | 0.586 |
| TRANS.ENZ | fucose:H+ symporter | 0 | Not detected | 0.034 | -0.140 | 0.207 | 0.704 |
| TRANS.ENZ | shikimate:H+ symporter | 0 | Not detected | 0.130 | -0.036 | 0.296 | 0.124 |
| TRANS.ENZ | fosmidomycin efflux pump | 0 | Not detected | -0.081 | -0.221 | 0.058 | 0.252 |
| TRANS.ENZ | transport of 3-(3-hydroxyphenyl)pro | 0 | Not detected | -0.054 | -0.229 | 0.122 | 0.548 |
| TRANS.ENZ | sugar exporter SetA | 87 | Not detected | -0.111 | -0.284 | 0.063 | 0.212 |
| TRANS.ENZ | sugar exporter SetB | 0 | Not detected | 0.048 | -0.142 | 0.238 | 0.621 |
| TRANS.ENZ | transport of D-galactose | 0 | Not detected | 0.048 | -0.136 | 0.231 | 0.612 |
| TRANS.ENZ | Mn2+:H+ symporter | 0 | Not detected | -0.004 | -0.171 | 0.163 | 0.963 |
| TRANS.ENZ | L-threonine:H+ antiporter | 0 | Not detected | 0.019 | -0.176 | 0.213 | 0.849 |
| TRANS.ENZ | K+ transporter TrkH | 0 | Not detected | -0.003 | -0.214 | 0.208 | 0.977 |
| TRANS.ENZ | sn-glycerol 3-phosphate:phosphate a | 0 | Not detected | -0.232 | -0.459 | -0.005 | 0.045 |
| TRANS.ENZ | transport of 2-oxoglutarate | 0 | Not detected | 0.056 | -0.123 | 0.234 | 0.542 |
| TRANS.ENZ | N-acetylneuraminate:H+ symporter | 0 | Not detected | 0.222 | 0.034 | 0.410 | 0.021 |
| TRANS.ENZ | transport of a nucleoside | 0 | Not detected | 0.134 | -0.144 | 0.413 | 0.345 |
| TRANS.ENZ | transport of D-xylose | 0 | Not detected | -0.122 | -0.305 | 0.062 | 0.193 |
| TRANS.ENZ | xanthosine:H+ symporter XapB | 0 | Not detected | -0.205 | -0.397 | -0.013 | 0.037 |
| TRANS.ENZ | glucose-6-phosphate:phosphate antip | 0 | Not detected | 0.102 | -0.093 | 0.297 | 0.304 |
| TRANS.ENZ | D-glucuronate transporter | 0 | Not detected | 0.180 | -0.004 | 0.363 | 0.055 |
| TRANS.ENZ | D-galacturonate transporter | 0 | Not detected | 0.180 | -0.004 | 0.363 | 0.055 |
| TRANS.ENZ | L-arabinose exporter | 0 | Not detected | 0.007 | -0.153 | 0.168 | 0.928 |
| TRANS.ENZ | L-phenylalanine:H+ symporter | 0 | Not detected | 0.209 | -0.011 | 0.429 | 0.063 |
| TRANS.ENZ | L-tyrosine:H+ symporter | 0 | Not detected | 0.209 | -0.011 | 0.429 | 0.063 |
| TRANS.ENZ | L-tryptophan:H+ symporter | 0 | Not detected | 0.209 | -0.011 | 0.429 | 0.063 |
| TRANS.ENZ | 4-aminobutanoate:H+ symporter | 0 | Not detected | 0.027 | -0.150 | 0.205 | 0.762 |
| TRANS.ENZ | lysine:H+ symporter | 0 | Not detected | 0.306 | 0.046 | 0.566 | 0.021 |
| TRANS.ENZ | phenylalanine:H+ symporter PheP | 0 | Not detected | 0.104 | -0.130 | 0.338 | 0.383 |
| TRANS.ENZ | transport of D-alanine | 0 | Not detected | 0.066 | -0.155 | 0.288 | 0.558 |
| TRANS.ENZ | transport of glycine | 0 | Not detected | 0.066 | -0.155 | 0.288 | 0.558 |
| TRANS.ENZ | lysine:cadaverine antiporter | 0 | Not detected | 0.282 | 0.041 | 0.524 | 0.022 |

| TRANS.ENZ | serine:H+ symporter SdaC | 0 | Not detected | 0.048 | -0.176 | 0.272 | 0.676 |
| --- | --- | --- | --- | --- | --- | --- | --- |
| TRANS.ENZ | L-threonine:H+ symporter | 0 | Not detected | 0.054 | -0.128 | 0.236 | 0.560 |
| TRANS.ENZ | L-serine:H+ symporter | 0 | Not detected | 0.054 | -0.128 | 0.236 | 0.560 |
| TRANS.ENZ | tryptophan:H+ symporter | 0 | Not detected | 0.010 | -0.194 | 0.215 | 0.922 |
| TRANS.ENZ | indole:H+ symporter | 0 | Not detected | 0.010 | -0.194 | 0.215 | 0.922 |
| TRANS.ENZ | choline:H+ symporter | 0 | Not detected | 0.001 | -0.209 | 0.210 | 0.996 |
| TRE6PHYDR | trehalose-6-phosphate hydrolase | 0 | Not detected | -0.127 | -0.379 | 0.125 | 0.323 |
| TREHALAC | trehalase | 0 | Not detected | 0.211 | 0.013 | 0.409 | 0.037 |
| TREHALAPE | trehalase | 0 | Not detected | 0.054 | -0.124 | 0.233 | 0.549 |
| TREHALOSE | trehalose-6-phosphate synthase | 0 | Not detected | 0.100 | -0.071 | 0.271 | 0.253 |
| TSA.REDUC | tartronate semialdehyde reductase | 0 | Not detected | -0.159 | -0.366 | 0.048 | 0.132 |
| UDP.NACM | UDP-N-acetylmuramoyl-L-alanine& | 0 | Not detected | -0.039 | -0.244 | 0.167 | 0.713 |
| UDP.NACM | D-alanyl-D-alanine-adding enzyme | 0 | Not detected | 0.113 | -0.088 | 0.314 | 0.270 |
| UDP.NACM | UDP-N-acetylmuramoyl-L-alanyl-D | 0 | Not detected | 0.065 | -0.148 | 0.278 | 0.549 |
| UDPACYLG | UDP-3-O-acyl-N-acetylglucosamine | 0 | Not detected | 0.075 | -0.182 | 0.331 | 0.568 |
| UDPNACET | UDP-N-acetylglucosamine 1-carbox | 0 | Not detected | 0.178 | -0.050 | 0.407 | 0.127 |
| UDPNACET | UDP-N-acetylenolpyruvoylglucosam | 0 | Not detected | 0.136 | -0.052 | 0.323 | 0.157 |
| UDPSUGAR | UDP-sugar hydrolase | 0 | Not detected | -0.012 | -0.229 | 0.205 | 0.915 |
| URITRANS. | [protein-PII] uridylyltransferase | 0 | Not detected | 0.115 | -0.104 | 0.334 | 0.303 |
| URKI.ENZR | uridine kinase | 0 | Not detected | 0.077 | -0.168 | 0.323 | 0.537 |
| UROGENDE | uroporphyrinogen decarboxylase | 0 | Not detected | 0.158 | -0.088 | 0.404 | 0.207 |
| VALINE.PY | valine&mdash;pyruvate aminotransf | 0 | Not detected | 0.115 | -0.068 | 0.299 | 0.218 |
| VALS.ENZR | valine&mdash;tRNA ligase | 0 | Not detected | 0.169 | -0.111 | 0.449 | 0.238 |
| X1.ACYLGL | acyl-CoA:1-acyl-sn-glycerol-3-phos | 0 | Not detected | 0.010 | -0.160 | 0.180 | 0.909 |
| X2.OCTAPR | 2-octaprenyl-6-methoxy-1,4-benzoq | 0 | Not detected | -0.056 | -0.307 | 0.195 | 0.660 |
| X2.OCTPRN | 2-octaprenyl-6-methoxyphenol hydr | 0 | Not detected | 0.138 | -0.043 | 0.318 | 0.136 |
| X3.DEHYDR | 3-dehydroquinate synthase | 0 | Not detected | 0.011 | -0.213 | 0.235 | 0.925 |
| X3.NUCLEO | 3'-nucleotidase | 0 | Not detected | 0.029 | -0.201 | 0.259 | 0.806 |
| X3.P.SHIK.1 | 3-phosphoshikimate 1-carboxyvinylt | 0 | Not detected | 0.112 | -0.094 | 0.319 | 0.287 |
| X4OHBENZ | 4-hydroxybenzoate octaprenyltransfe | 0 | Not detected | 0.076 | -0.088 | 0.241 | 0.363 |
| X5.NUCLEO | 5'-ribonucleotidase | 0 | Not detected | -0.012 | -0.229 | 0.205 | 0.915 |

| **Supplementary Table 18**. Association of childhood SEP with species in foreign-born participants (n=1872). Analysis of Compositions of Microbiomes (ANCOM2) was used to identify species related to Lower childhood SEP. Regression coefficients were obtained by modelling the effect of Lower childhood SEP on centered log ratio (CLR)-transformed species in multivariable linear regression adjusted for age, gender, BMI, field center, Hispanic/Latino background, U.S. nativity, Bristol stool type, current SEP, the alternative Healthy Eating Index 2010 score, cigarette smoking, alcohol use, depressive and anxiety symptoms, diabetes, hypertension, cardiovascular disease, dyslipidemia, and antibiotic use. | | | | | | |
| --- | --- | --- | --- | --- | --- | --- |
| **species** | **W_stat** | **ANCOM** | **beta** | **l95ci** | **u95ci** | **qval** |
| Anaerotruncus sp. CAG:528 | 883 | 0.7 | 0.298 | 0.099 | 0.496 | 0.003 |
| Bacteroides finegoldii CAG:203 | 871 | 0.7 | -0.250 | -0.418 | -0.083 | 0.003 |
| Bacteroides sp. CAG:98 | 1168 | 0.9 | -0.525 | -0.771 | -0.279 | 0.000 |
| Blautia hydrogenotrophica CAG:147 | 1150 | 0.9 | -0.612 | -0.864 | -0.360 | 0.000 |
| Clostridia bacterium UC5.1-1C12 | 920 | 0.7 | -0.464 | -0.718 | -0.209 | 0.000 |
| Clostridium clostridioforme CAG:132 | 944 | 0.7 | -0.287 | -0.485 | -0.088 | 0.005 |
| Clostridium clostridioforme CAG:511 | 969 | 0.8 | -0.307 | -0.488 | -0.126 | 0.001 |
| Clostridium sp. CAG:12237_41 | 916 | 0.7 | 0.301 | 0.129 | 0.473 | 0.001 |
| Clostridium sp. CAG:62_40_43 | 830 | 0.7 | 0.382 | 0.078 | 0.687 | 0.014 |
| Coprobacillus sp. CAG:605 | 910 | 0.7 | 0.470 | 0.191 | 0.750 | 0.001 |
| Coprococcus sp. HPP0048 | 1072 | 0.9 | -0.297 | -0.421 | -0.172 | 0.000 |
| Dialister sp. CAG:357 | 979 | 0.8 | 0.445 | 0.074 | 0.816 | 0.019 |
| Dialister sp. CAG:486 | 833 | 0.7 | 0.382 | 0.040 | 0.724 | 0.029 |
| Dielma fastidiosa | 918 | 0.7 | -0.321 | -0.506 | -0.137 | 0.001 |
| Eggerthella sp. CAG:209 | 951 | 0.8 | 0.449 | 0.110 | 0.788 | 0.010 |
| Empedobacter brevis | 851 | 0.7 | -0.398 | -0.670 | -0.126 | 0.004 |
| Eubacterium sp. CAG:180 | 983 | 0.8 | 0.454 | 0.120 | 0.788 | 0.008 |
| Eubacterium sp. CAG:248 | 1044 | 0.8 | 0.372 | 0.163 | 0.581 | 0.000 |
| Eubacterium sp. CAG:252 | 991 | 0.8 | 0.357 | 0.172 | 0.543 | 0.000 |
| Eubacterium sp. CAG:603 | 945 | 0.7 | 0.289 | 0.151 | 0.427 | 0.000 |
| Lactobacillus ruminis | 858 | 0.7 | 0.268 | 0.083 | 0.453 | 0.005 |
| Roseburia faecis | 932 | 0.7 | 0.250 | 0.114 | 0.386 | 0.000 |
| Roseburia sp. CAG:309 | 1041 | 0.8 | 0.483 | 0.245 | 0.721 | 0.000 |
| Roseburia sp. CAG:380 | 1082 | 0.9 | 0.470 | 0.244 | 0.695 | 0.000 |
| Ruminococcus sp. CAG:254 | 903 | 0.7 | 0.443 | 0.100 | 0.785 | 0.011 |
| Ruminococcus torques CAG:61 | 925 | 0.7 | -0.349 | -0.581 | -0.117 | 0.003 |
| Sharpea azabuensis | 842 | 0.7 | 0.197 | 0.104 | 0.290 | 0.000 |
| [Clostridium] spiroforme | 1133 | 0.9 | -0.401 | -0.589 | -0.214 | 0.000 |
| Abiotrophia defectiva | 2 | Not detected | -0.042 | -0.241 | 0.156 | 0.677 |
| Acetanaerobacterium elongatum | 41 | Not detected | 0.070 | -0.066 | 0.206 | 0.314 |
| Acetitomaculum ruminis | 21 | Not detected | 0.006 | -0.071 | 0.083 | 0.886 |
| Acetivibrio cellulolyticus | 8 | Not detected | 0.029 | -0.131 | 0.189 | 0.724 |
| Acetivibrio ethanolgignens | 15 | Not detected | -0.037 | -0.105 | 0.030 | 0.274 |
| Acetobacter sp. 46_36 | 0 | Not detected | -0.068 | -0.377 | 0.242 | 0.669 |
| Acetobacter sp. CAG:267 | 0 | Not detected | 0.019 | -0.263 | 0.302 | 0.895 |
| Acholeplasma axanthum | 5 | Not detected | 0.052 | -0.089 | 0.192 | 0.471 |
| Acholeplasma equifetale | 10 | Not detected | -0.072 | -0.261 | 0.118 | 0.459 |

| Acidaminococcus fermentans | 1 | Not detected | 0.023 | -0.110 | 0.156 | 0.734 |
| --- | --- | --- | --- | --- | --- | --- |
| Acidaminococcus intestini | 0 | Not detected | -0.115 | -0.352 | 0.122 | 0.342 |
| Acidaminococcus intestini CAG:325 | 0 | Not detected | -0.208 | -0.588 | 0.172 | 0.283 |
| Acinetobacter baumannii | 4 | Not detected | 0.128 | -0.038 | 0.294 | 0.132 |
| Acinetobacter bereziniae | 5 | Not detected | 0.041 | -0.123 | 0.205 | 0.625 |
| Acinetobacter johnsonii | 5 | Not detected | -0.010 | -0.190 | 0.170 | 0.914 |
| Acinetobacter sp. CAG:196 | 0 | Not detected | -0.149 | -0.481 | 0.183 | 0.379 |
| Acinetobacter sp. CAG:196_36_41 | 0 | Not detected | -0.105 | -0.429 | 0.218 | 0.523 |
| Acinetobacter sp. N54.MGS-139 | 0 | Not detected | -0.060 | -0.233 | 0.113 | 0.498 |
| Acinetobacter venetianus | 4 | Not detected | 0.041 | -0.157 | 0.239 | 0.683 |
| Actinobaculum sp. oral taxon 183 | 52 | Not detected | 0.226 | -0.004 | 0.457 | 0.054 |
| Actinobaculum suis | 3 | Not detected | -0.033 | -0.201 | 0.135 | 0.697 |
| Actinomyces dentalis | 0 | Not detected | -0.055 | -0.292 | 0.182 | 0.647 |
| Actinomyces gerencseriae | 4 | Not detected | 0.004 | -0.212 | 0.220 | 0.972 |
| Actinomyces graevenitzii | 0 | Not detected | 0.028 | -0.123 | 0.179 | 0.715 |
| Actinomyces hongkongensis | 13 | Not detected | -0.229 | -0.442 | -0.017 | 0.034 |
| Actinomyces israelii | 1 | Not detected | -0.002 | -0.226 | 0.222 | 0.986 |
| Actinomyces johnsonii | 4 | Not detected | -0.070 | -0.286 | 0.147 | 0.528 |
| Actinomyces massiliensis | 1 | Not detected | -0.023 | -0.245 | 0.198 | 0.837 |
| Actinomyces meyeri | 11 | Not detected | -0.088 | -0.274 | 0.099 | 0.356 |
| Actinomyces odontolyticus | 0 | Not detected | -0.061 | -0.224 | 0.102 | 0.463 |
| Actinomyces oris | 0 | Not detected | -0.097 | -0.295 | 0.101 | 0.338 |
| Actinomyces sp. HMSC065F12 | 22 | Not detected | -0.035 | -0.160 | 0.091 | 0.588 |
| Actinomyces sp. HMSC06A08 | 24 | Not detected | 0.065 | -0.064 | 0.194 | 0.323 |
| Actinomyces sp. oral taxon 181 | 0 | Not detected | -0.095 | -0.306 | 0.115 | 0.373 |
| Actinomyces sp. oral taxon 448 | 0 | Not detected | -0.043 | -0.293 | 0.208 | 0.739 |
| Actinomyces sp. oral taxon 848 | 22 | Not detected | 0.082 | -0.086 | 0.249 | 0.340 |
| Actinomyces sp. ph3 | 1 | Not detected | -0.112 | -0.315 | 0.092 | 0.282 |
| Actinomyces timonensis | 5 | Not detected | -0.014 | -0.162 | 0.133 | 0.849 |
| Actinomyces turicensis | 2 | Not detected | -0.047 | -0.262 | 0.168 | 0.668 |
| Adlercreutzia equolifaciens | 0 | Not detected | -0.083 | -0.341 | 0.175 | 0.527 |
| Aeromonas salmonicida | 2 | Not detected | -0.069 | -0.279 | 0.141 | 0.520 |
| Aggregatibacter aphrophilus | 4 | Not detected | 0.121 | -0.115 | 0.358 | 0.314 |
| Akkermansia glycaniphila | 11 | Not detected | -0.196 | -0.410 | 0.017 | 0.072 |
| Akkermansia muciniphila | 0 | Not detected | -0.229 | -0.505 | 0.046 | 0.103 |
| Akkermansia muciniphila CAG:154 | 0 | Not detected | -0.188 | -0.545 | 0.170 | 0.304 |
| Akkermansia sp. 54_46 | 0 | Not detected | -0.252 | -0.627 | 0.122 | 0.187 |
| Akkermansia sp. CAG:344 | 0 | Not detected | -0.133 | -0.509 | 0.244 | 0.489 |
| Akkermansia sp. KLE1605 | 0 | Not detected | -0.205 | -0.526 | 0.116 | 0.211 |
| Akkermansia sp. KLE1797 | 0 | Not detected | -0.213 | -0.535 | 0.109 | 0.195 |
| Akkermansia sp. KLE1798 | 0 | Not detected | -0.208 | -0.530 | 0.113 | 0.204 |
| Akkermansia sp. Phil8 | 0 | Not detected | -0.241 | -0.611 | 0.128 | 0.201 |
| Akkermansia sp. UNK.MGS-1 | 0 | Not detected | -0.270 | -0.640 | 0.099 | 0.152 |
| Alistipes finegoldii | 2 | Not detected | -0.017 | -0.163 | 0.129 | 0.820 |

| Alistipes ihumii | 0 | Not detected | -0.027 | -0.276 | 0.223 | 0.835 |
| --- | --- | --- | --- | --- | --- | --- |
| Alistipes indistinctus | 0 | Not detected | 0.202 | -0.143 | 0.548 | 0.251 |
| Alistipes inops | 0 | Not detected | -0.146 | -0.419 | 0.127 | 0.294 |
| Alistipes obesi | 0 | Not detected | -0.003 | -0.164 | 0.157 | 0.967 |
| Alistipes putredinis | 464 | Not detected | -0.257 | -0.465 | -0.049 | 0.016 |
| Alistipes senegalensis | 3 | Not detected | 0.055 | -0.076 | 0.186 | 0.407 |
| Alistipes shahii | 0 | Not detected | -0.036 | -0.183 | 0.111 | 0.635 |
| Alistipes sp. CAG:435 | 0 | Not detected | 0.219 | -0.135 | 0.574 | 0.226 |
| Alistipes sp. CAG:514 | 3 | Not detected | 0.386 | 0.070 | 0.702 | 0.017 |
| Alistipes sp. Zagget8 | 3 | Not detected | 0.133 | -0.080 | 0.346 | 0.222 |
| Alistipes timonensis | 2 | Not detected | 0.071 | -0.150 | 0.291 | 0.528 |
| Alkaliflexus imshenetskii | 159 | Not detected | -0.206 | -0.370 | -0.042 | 0.014 |
| Allisonella histaminiformans | 2 | Not detected | 0.377 | 0.050 | 0.704 | 0.024 |
| Allobaculum stercoricanis | 21 | Not detected | 0.041 | -0.036 | 0.119 | 0.296 |
| Allofustis seminis | 5 | Not detected | 0.024 | -0.161 | 0.209 | 0.800 |
| Alloprevotella rava | 4 | Not detected | 0.043 | -0.101 | 0.187 | 0.559 |
| Alloprevotella tannerae | 14 | Not detected | -0.086 | -0.184 | 0.011 | 0.084 |
| Alloscardovia criceti | 5 | Not detected | 0.018 | -0.163 | 0.198 | 0.848 |
| Alloscardovia omnicolens | 15 | Not detected | 0.184 | -0.011 | 0.378 | 0.064 |
| Amphibacillus xylanus | 10 | Not detected | -0.105 | -0.297 | 0.088 | 0.287 |
| Anaerobium acetethylicum | 14 | Not detected | -0.006 | -0.085 | 0.072 | 0.872 |
| Anaerococcus hydrogenalis | 49 | Not detected | -0.140 | -0.276 | -0.003 | 0.046 |
| Anaerococcus lactolyticus | 27 | Not detected | -0.142 | -0.339 | 0.055 | 0.157 |
| Anaerococcus obesiensis | 529 | Not detected | -0.216 | -0.381 | -0.052 | 0.010 |
| Anaerocolumna aminovalerica | 7 | Not detected | 0.035 | -0.129 | 0.200 | 0.674 |
| Anaerocolumna xylanovorans | 9 | Not detected | -0.062 | -0.212 | 0.089 | 0.422 |
| Anaerofustis stercorihominis | 2 | Not detected | 0.003 | -0.238 | 0.244 | 0.982 |
| Anaeroglobus geminatus | 11 | Not detected | -0.101 | -0.296 | 0.093 | 0.307 |
| Anaerolineales bacterium Chloro_04 | 4 | Not detected | 0.098 | -0.080 | 0.276 | 0.282 |
| Anaeromassilibacillus senegalensis | 24 | Not detected | 0.046 | -0.088 | 0.180 | 0.502 |
| Anaerophaga thermohalophila | 8 | Not detected | -0.008 | -0.135 | 0.118 | 0.899 |
| Anaerosalibacter massiliensis | 14 | Not detected | -0.217 | -0.417 | -0.016 | 0.034 |
| Anaerosporobacter mobilis | 12 | Not detected | -0.068 | -0.154 | 0.017 | 0.119 |
| Anaerosporomusa subterranea | 5 | Not detected | 0.026 | -0.112 | 0.165 | 0.709 |
| Anaerostipes caccae | 212 | Not detected | -0.093 | -0.173 | -0.013 | 0.022 |
| Anaerostipes hadrus | 0 | Not detected | -0.037 | -0.172 | 0.098 | 0.591 |
| Anaerostipes sp. CAG:276 | 753 | 0.6 | -0.332 | -0.612 | -0.052 | 0.020 |
| Anaerotruncus colihominis | 23 | Not detected | -0.067 | -0.157 | 0.023 | 0.144 |
| Anaerotruncus sp. CAG:390 | 4 | Not detected | 0.205 | -0.041 | 0.450 | 0.103 |
| Anaerotruncus sp. G3(2012) | 14 | Not detected | -0.006 | -0.072 | 0.061 | 0.868 |
| Anaerovorax odorimutans | 5 | Not detected | 0.052 | -0.107 | 0.211 | 0.519 |
| Angelakisella massiliensis | 10 | Not detected | -0.008 | -0.084 | 0.068 | 0.841 |
| Aphanizomenon flos-aquae | 27 | Not detected | 0.117 | -0.026 | 0.261 | 0.110 |
| Aquabacterium parvum | 4 | Not detected | 0.012 | -0.162 | 0.185 | 0.896 |

| Arcanobacterium haemolyticum | 45 | Not detected | 0.160 | -0.011 | 0.331 | 0.067 |
| --- | --- | --- | --- | --- | --- | --- |
| Asaccharospora irregularis | 4 | Not detected | 0.100 | -0.092 | 0.292 | 0.307 |
| Atlantibacter hermannii | 0 | Not detected | -0.007 | -0.257 | 0.243 | 0.957 |
| Atopobium deltae | 8 | Not detected | -0.060 | -0.245 | 0.126 | 0.530 |
| Atopobium minutum | 10 | Not detected | -0.108 | -0.296 | 0.079 | 0.258 |
| Atopobium parvulum | 9 | Not detected | -0.138 | -0.345 | 0.070 | 0.194 |
| Atopobium rimae | 5 | Not detected | 0.022 | -0.150 | 0.194 | 0.801 |
| Atopobium sp. BS2 | 9 | Not detected | -0.171 | -0.373 | 0.032 | 0.099 |
| Atopobium sp. HMSC064B08 | 10 | Not detected | -0.081 | -0.227 | 0.066 | 0.283 |
| Atopobium sp. ICM42b | 17 | Not detected | -0.168 | -0.373 | 0.036 | 0.107 |
| Atopobium vaginae | 4 | Not detected | 0.048 | -0.144 | 0.240 | 0.622 |
| Atopostipes suicloacalis | 4 | Not detected | 0.115 | -0.068 | 0.297 | 0.217 |
| Avibacterium paragallinarum | 4 | Not detected | 0.103 | -0.068 | 0.275 | 0.239 |
| Azospirillum sp. 47_25 | 0 | Not detected | -0.065 | -0.425 | 0.295 | 0.723 |
| Azospirillum sp. CAG:260 | 0 | Not detected | -0.095 | -0.451 | 0.261 | 0.601 |
| Bacillus acidiproducens | 31 | Not detected | -0.194 | -0.398 | 0.009 | 0.062 |
| Bacillus andreraoultii | 304 | Not detected | -0.218 | -0.421 | -0.016 | 0.035 |
| Bacillus azotoformans | 108 | Not detected | -0.229 | -0.412 | -0.045 | 0.015 |
| Bacillus bataviensis | 33 | Not detected | -0.200 | -0.377 | -0.024 | 0.026 |
| Bacillus coahuilensis | 0 | Not detected | -0.021 | -0.229 | 0.188 | 0.846 |
| Bacillus rubiinfantis | 18 | Not detected | -0.123 | -0.304 | 0.058 | 0.183 |
| Bacillus sp. CAG:988 | 9 | Not detected | -0.042 | -0.212 | 0.128 | 0.630 |
| Bacillus thermotolerans | 166 | Not detected | -0.202 | -0.399 | -0.005 | 0.044 |
| Bacteroidales bacterium 45-6 | 4 | Not detected | 0.017 | -0.178 | 0.212 | 0.864 |
| Bacteroidales bacterium 55_9 | 5 | Not detected | -0.034 | -0.129 | 0.061 | 0.481 |
| Bacteroidales bacterium Bact_08 | 5 | Not detected | 0.052 | -0.102 | 0.207 | 0.507 |
| Bacteroidales bacterium Bact_18 | 5 | Not detected | 0.036 | -0.123 | 0.195 | 0.655 |
| Bacteroidales bacterium Bact_23 | 10 | Not detected | -0.249 | -0.451 | -0.046 | 0.016 |
| Bacteroidales bacterium CF | 6 | Not detected | 0.005 | -0.169 | 0.179 | 0.957 |
| Bacteroidales bacterium H10 | 10 | Not detected | -0.080 | -0.230 | 0.070 | 0.294 |
| Bacteroidales bacterium K1 | 5 | Not detected | 0.061 | -0.117 | 0.239 | 0.501 |
| Bacteroidales bacterium K10 | 5 | Not detected | -0.126 | -0.298 | 0.045 | 0.150 |
| Bacteroidales bacterium KHT7 | 4 | Not detected | 0.088 | -0.115 | 0.291 | 0.395 |
| Bacteroides barnesiae | 15 | Not detected | -0.089 | -0.183 | 0.005 | 0.063 |
| Bacteroides caccae CAG:21 | 37 | Not detected | -0.232 | -0.432 | -0.032 | 0.023 |
| Bacteroides cellulosilyticus | 0 | Not detected | -0.026 | -0.201 | 0.149 | 0.771 |
| Bacteroides coprocola | 603 | Not detected | -0.191 | -0.324 | -0.057 | 0.005 |
| Bacteroides coprophilus | 7 | Not detected | -0.080 | -0.174 | 0.014 | 0.095 |
| Bacteroides coprosuis | 3 | Not detected | 0.057 | -0.094 | 0.208 | 0.456 |
| Bacteroides dorei CAG:222 | 20 | Not detected | -0.183 | -0.339 | -0.027 | 0.022 |
| Bacteroides faecichinchillae | 728 | 0.6 | -0.173 | -0.268 | -0.078 | 0.000 |
| Bacteroides faecis CAG:32 | 7 | Not detected | -0.228 | -0.425 | -0.032 | 0.022 |
| Bacteroides finegoldii | 282 | Not detected | -0.222 | -0.400 | -0.044 | 0.015 |
| Bacteroides fluxus | 528 | Not detected | -0.157 | -0.258 | -0.056 | 0.002 |

| Bacteroides fragilis | 19 | Not detected | -0.163 | -0.300 | -0.025 | 0.020 |
| --- | --- | --- | --- | --- | --- | --- |
| Bacteroides fragilis CAG:47 | 0 | Not detected | -0.129 | -0.352 | 0.094 | 0.256 |
| Bacteroides graminisolvens | 0 | Not detected | -0.171 | -0.387 | 0.044 | 0.119 |
| Bacteroides neonati | 22 | Not detected | -0.082 | -0.155 | -0.008 | 0.030 |
| Bacteroides oleiciplenus | 318 | Not detected | -0.156 | -0.270 | -0.042 | 0.007 |
| Bacteroides ovatus | 46 | Not detected | -0.159 | -0.291 | -0.026 | 0.019 |
| Bacteroides ovatus CAG:22 | 21 | Not detected | -0.170 | -0.315 | -0.026 | 0.021 |
| Bacteroides paurosaccharolyticus | 744 | 0.6 | -0.219 | -0.356 | -0.082 | 0.002 |
| Bacteroides pectinophilus CAG:437 | 266 | Not detected | 0.167 | 0.036 | 0.298 | 0.012 |
| Bacteroides plebeius | 13 | Not detected | -0.152 | -0.294 | -0.011 | 0.035 |
| Bacteroides propionicifaciens | 10 | Not detected | -0.121 | -0.307 | 0.065 | 0.202 |
| Bacteroides pyogenes | 356 | Not detected | -0.169 | -0.282 | -0.056 | 0.003 |
| Bacteroides reticulotermitis | 108 | Not detected | -0.155 | -0.274 | -0.037 | 0.010 |
| Bacteroides salanitronis | 7 | Not detected | -0.063 | -0.158 | 0.032 | 0.194 |
| Bacteroides salyersiae | 1 | Not detected | -0.111 | -0.246 | 0.025 | 0.110 |
| Bacteroides sp. 41_26 | 4 | Not detected | -0.152 | -0.310 | 0.006 | 0.059 |
| Bacteroides sp. 43_46 | 656 | Not detected | -0.197 | -0.333 | -0.062 | 0.004 |
| Bacteroides sp. CAG:1060 | 1 | Not detected | 0.087 | -0.165 | 0.340 | 0.498 |
| Bacteroides sp. CAG:1076 | 8 | Not detected | -0.134 | -0.291 | 0.023 | 0.094 |
| Bacteroides sp. CAG:530 | 4 | Not detected | 0.134 | -0.014 | 0.282 | 0.076 |
| Bacteroides sp. CAG:545 | 4 | Not detected | 0.384 | 0.061 | 0.707 | 0.020 |
| Bacteroides sp. CAG:714 | 0 | Not detected | -0.064 | -0.295 | 0.167 | 0.587 |
| Bacteroides sp. CAG:770 | 0 | Not detected | 0.092 | -0.085 | 0.270 | 0.308 |
| Bacteroides sp. D2 | 10 | Not detected | -0.169 | -0.321 | -0.017 | 0.030 |
| Bacteroides sp. Ga6A1 | 0 | Not detected | 0.089 | -0.133 | 0.311 | 0.434 |
| Bacteroides sp. Ga6A2 | 0 | Not detected | -0.023 | -0.245 | 0.199 | 0.839 |
| Bacteroides stercoris | 401 | Not detected | -0.219 | -0.392 | -0.047 | 0.013 |
| Bacteroides thetaiotaomicron | 0 | Not detected | -0.052 | -0.150 | 0.046 | 0.297 |
| Bacteroides thetaiotaomicron CAG:40 | 0 | Not detected | -0.071 | -0.227 | 0.086 | 0.377 |
| Bacteroides uniformis | 4 | Not detected | -0.117 | -0.258 | 0.024 | 0.104 |
| Bacteroides vulgatus | 42 | Not detected | -0.158 | -0.289 | -0.028 | 0.017 |
| Bacteroides vulgatus CAG:6 | 13 | Not detected | -0.172 | -0.328 | -0.016 | 0.031 |
| Bacteroidetes bacterium OLB8 | 10 | Not detected | -0.155 | -0.341 | 0.032 | 0.104 |
| Bacteroidetes oral taxon 274 | 30 | Not detected | -0.133 | -0.265 | 0.000 | 0.051 |
| Bariatricus massiliensis | 11 | Not detected | -0.001 | -0.072 | 0.070 | 0.977 |
| Barnesiella intestinihominis | 0 | Not detected | -0.129 | -0.304 | 0.046 | 0.149 |
| Barnesiella viscericola | 95 | Not detected | -0.100 | -0.180 | -0.020 | 0.014 |
| Bavariicoccus seileri | 11 | Not detected | -0.178 | -0.349 | -0.007 | 0.041 |
| Beduini massiliensis | 8 | Not detected | 0.002 | -0.104 | 0.109 | 0.966 |
| Bibersteinia trehalosi | 0 | Not detected | -0.032 | -0.231 | 0.167 | 0.752 |
| Bifidobacterium actinocoloniiforme | 5 | Not detected | -0.020 | -0.212 | 0.172 | 0.839 |
| Bifidobacterium adolescentis | 0 | Not detected | 0.161 | -0.133 | 0.455 | 0.283 |
| Bifidobacterium aesculapii | 4 | Not detected | 0.053 | -0.100 | 0.206 | 0.496 |
| Bifidobacterium angulatum | 4 | Not detected | 0.207 | -0.036 | 0.450 | 0.096 |

| Bifidobacterium animalis | 0 | Not detected | 0.016 | -0.196 | 0.228 | 0.882 |
| --- | --- | --- | --- | --- | --- | --- |
| Bifidobacterium asteroides | 5 | Not detected | -0.036 | -0.237 | 0.166 | 0.727 |
| Bifidobacterium biavatii | 9 | Not detected | 0.017 | -0.059 | 0.093 | 0.661 |
| Bifidobacterium bifidum | 0 | Not detected | 0.064 | -0.216 | 0.344 | 0.655 |
| Bifidobacterium bohemicum | 4 | Not detected | 0.075 | -0.107 | 0.257 | 0.418 |
| Bifidobacterium bombi | 5 | Not detected | -0.069 | -0.250 | 0.112 | 0.454 |
| Bifidobacterium breve | 0 | Not detected | 0.044 | -0.228 | 0.317 | 0.750 |
| Bifidobacterium callitrichos | 3 | Not detected | 0.126 | -0.119 | 0.371 | 0.314 |
| Bifidobacterium choerinum | 4 | Not detected | 0.060 | -0.153 | 0.273 | 0.584 |
| Bifidobacterium coryneforme | 3 | Not detected | -0.070 | -0.263 | 0.123 | 0.476 |
| Bifidobacterium crudilactis | 5 | Not detected | 0.012 | -0.150 | 0.173 | 0.889 |
| Bifidobacterium cuniculi | 5 | Not detected | -0.026 | -0.232 | 0.179 | 0.801 |
| Bifidobacterium dentium | 0 | Not detected | 0.074 | -0.191 | 0.339 | 0.583 |
| Bifidobacterium gallicum | 5 | Not detected | 0.003 | -0.203 | 0.210 | 0.975 |
| Bifidobacterium gallinarum | 0 | Not detected | 0.010 | -0.238 | 0.258 | 0.936 |
| Bifidobacterium longum | 0 | Not detected | 0.022 | -0.251 | 0.296 | 0.873 |
| Bifidobacterium magnum | 63 | Not detected | 0.107 | -0.022 | 0.235 | 0.104 |
| Bifidobacterium minimum | 0 | Not detected | -0.011 | -0.266 | 0.243 | 0.930 |
| Bifidobacterium mongoliense | 5 | Not detected | 0.038 | -0.158 | 0.235 | 0.702 |
| Bifidobacterium pseudolongum | 0 | Not detected | -0.089 | -0.311 | 0.133 | 0.431 |
| Bifidobacterium psychraerophilum | 5 | Not detected | -0.017 | -0.164 | 0.130 | 0.818 |
| Bifidobacterium reuteri | 0 | Not detected | 0.063 | -0.135 | 0.261 | 0.531 |
| Bifidobacterium saguini | 0 | Not detected | 0.020 | -0.246 | 0.287 | 0.881 |
| Bifidobacterium scardovii | 0 | Not detected | 0.073 | -0.189 | 0.336 | 0.585 |
| Bifidobacterium stellenboschense | 9 | Not detected | -0.006 | -0.092 | 0.080 | 0.899 |
| Bifidobacterium subtile | 2 | Not detected | -0.040 | -0.272 | 0.192 | 0.735 |
| Bifidobacterium thermophilum | 8 | Not detected | 0.091 | -0.035 | 0.216 | 0.158 |
| Bifidobacterium tsurumiense | 4 | Not detected | 0.059 | -0.149 | 0.267 | 0.579 |
| Bilophila wadsworthia | 0 | Not detected | 0.114 | -0.199 | 0.427 | 0.475 |
| Bittarella massiliensis | 18 | Not detected | -0.035 | -0.109 | 0.040 | 0.362 |
| Blautia hansenii | 381 | Not detected | -0.126 | -0.212 | -0.039 | 0.004 |
| Blautia hydrogenotrophica | 764 | 0.6 | -0.167 | -0.245 | -0.089 | 0.000 |
| Blautia obeum | 13 | Not detected | -0.039 | -0.121 | 0.043 | 0.351 |
| Blautia producta | 212 | Not detected | -0.139 | -0.252 | -0.027 | 0.015 |
| Blautia schinkii | 17 | Not detected | -0.020 | -0.081 | 0.040 | 0.508 |
| Blautia sp. CAG:237 | 3 | Not detected | 0.100 | -0.075 | 0.274 | 0.262 |
| Blautia sp. CAG:37 | 4 | Not detected | 0.117 | -0.043 | 0.276 | 0.152 |
| Blautia sp. CAG:37_48_57 | 4 | Not detected | 0.078 | -0.046 | 0.203 | 0.218 |
| Blautia sp. KLE 1732 | 4 | Not detected | 0.003 | -0.099 | 0.104 | 0.961 |
| Brenneria goodwinii | 4 | Not detected | 0.001 | -0.160 | 0.162 | 0.989 |
| Bulleidia extructa | 3 | Not detected | -0.031 | -0.228 | 0.165 | 0.755 |
| Burkholderiales bacterium GJ-E10 | 89 | Not detected | 0.287 | 0.105 | 0.469 | 0.002 |
| Buttiauxella ferragutiae | 4 | Not detected | 0.000 | -0.181 | 0.180 | 0.996 |
| Butyricicoccus desmolans | 16 | Not detected | -0.023 | -0.088 | 0.042 | 0.493 |

| Butyricicoccus pullicaecorum | 19 | Not detected | -0.063 | -0.146 | 0.021 | 0.141 |
| --- | --- | --- | --- | --- | --- | --- |
| Butyricicoccus sp. K4410.MGS-46 | 4 | Not detected | 0.034 | -0.076 | 0.143 | 0.544 |
| Butyricimonas sp. Marseille-P2440 | 12 | Not detected | -0.183 | -0.347 | -0.018 | 0.030 |
| Butyricimonas synergistica | 0 | Not detected | -0.123 | -0.327 | 0.081 | 0.236 |
| Butyricimonas virosa | 0 | Not detected | -0.091 | -0.259 | 0.077 | 0.290 |
| Butyrivibrio crossotus | 765 | 0.6 | 0.213 | 0.070 | 0.356 | 0.004 |
| Butyrivibrio crossotus CAG:259 | 250 | Not detected | 0.248 | 0.033 | 0.462 | 0.024 |
| Butyrivibrio fibrisolvens | 5 | Not detected | 0.043 | -0.132 | 0.218 | 0.628 |
| Butyrivibrio hungatei | 83 | Not detected | 0.095 | -0.042 | 0.233 | 0.174 |
| Butyrivibrio proteoclasticus | 24 | Not detected | 0.011 | -0.085 | 0.106 | 0.826 |
| Butyrivibrio sp. AD3002 | 12 | Not detected | -0.069 | -0.188 | 0.051 | 0.260 |
| Butyrivibrio sp. AE2005 | 9 | Not detected | -0.004 | -0.134 | 0.127 | 0.955 |
| Butyrivibrio sp. AE3003 | 36 | Not detected | 0.017 | -0.077 | 0.112 | 0.723 |
| Butyrivibrio sp. AE3004 | 21 | Not detected | -0.006 | -0.099 | 0.086 | 0.896 |
| Butyrivibrio sp. AE3009 | 21 | Not detected | 0.090 | -0.063 | 0.243 | 0.249 |
| Butyrivibrio sp. FCS006 | 125 | Not detected | 0.167 | 0.012 | 0.322 | 0.035 |
| Butyrivibrio sp. INlla14 | 7 | Not detected | 0.040 | -0.088 | 0.168 | 0.537 |
| Butyrivibrio sp. INlla16 | 22 | Not detected | 0.028 | -0.108 | 0.163 | 0.689 |
| Butyrivibrio sp. INlla21 | 34 | Not detected | 0.083 | -0.050 | 0.216 | 0.222 |
| Butyrivibrio sp. LB2008 | 47 | Not detected | 0.129 | -0.024 | 0.282 | 0.098 |
| Butyrivibrio sp. M55 | 14 | Not detected | 0.001 | -0.141 | 0.143 | 0.991 |
| Butyrivibrio sp. NC2002 | 12 | Not detected | -0.060 | -0.211 | 0.092 | 0.439 |
| Butyrivibrio sp. NC2007 | 15 | Not detected | 0.032 | -0.105 | 0.170 | 0.645 |
| Butyrivibrio sp. NC3005 | 37 | Not detected | 0.197 | 0.000 | 0.394 | 0.050 |
| Butyrivibrio sp. VCD2006 | 124 | Not detected | 0.098 | -0.044 | 0.240 | 0.178 |
| Butyrivibrio sp. WCD2001 | 28 | Not detected | -0.011 | -0.152 | 0.129 | 0.876 |
| Butyrivibrio sp. WCD3002 | 21 | Not detected | -0.014 | -0.147 | 0.119 | 0.833 |
| Butyrivibrio sp. ob235 | 17 | Not detected | 0.055 | -0.115 | 0.225 | 0.527 |
| Campylobacter coli | 11 | Not detected | -0.056 | -0.162 | 0.049 | 0.296 |
| Campylobacter hominis | 413 | Not detected | -0.218 | -0.432 | -0.004 | 0.046 |
| Candidatus Azobacteroides pseudotrichonympha | 8 | Not detected | -0.016 | -0.155 | 0.124 | 0.825 |
| Candidatus Gastranaerophilales bacterium MH_3 | 11 | Not detected | -0.160 | -0.346 | 0.026 | 0.093 |
| Candidatus Melainabacteria bacterium LEY3_CP | 6 | Not detected | 0.008 | -0.124 | 0.141 | 0.901 |
| Candidatus Melainabacteria bacterium MEL.A1 | 0 | Not detected | 0.146 | -0.124 | 0.417 | 0.290 |
| Candidatus Saccharibacteria oral taxon TM7x | 5 | Not detected | 0.050 | -0.113 | 0.213 | 0.551 |
| Candidatus Soleaferrea massiliensis | 10 | Not detected | -0.011 | -0.125 | 0.103 | 0.849 |
| Candidatus Stoquefichus sp. SB1 | 5 | Not detected | -0.027 | -0.135 | 0.081 | 0.621 |
| Capnocytophaga granulosa | 5 | Not detected | 0.018 | -0.152 | 0.188 | 0.833 |
| Capnocytophaga sp. oral taxon 329 | 0 | Not detected | -0.039 | -0.281 | 0.204 | 0.754 |
| Capnocytophaga sp. oral taxon 863 | 707 | Not detected | -0.385 | -0.594 | -0.177 | 0.000 |
| Capnocytophaga sputigena | 13 | Not detected | -0.079 | -0.212 | 0.055 | 0.251 |
| Carnobacterium gallinarum | 11 | Not detected | -0.208 | -0.408 | -0.007 | 0.042 |
| Catabacter hongkongensis | 4 | Not detected | -0.027 | -0.181 | 0.127 | 0.733 |
| Catenibacterium mitsuokai | 3 | Not detected | 0.261 | -0.007 | 0.528 | 0.056 |

| Catenibacterium sp. CAG:290 | 0 | Not detected | 0.320 | -0.011 | 0.651 | 0.058 |
| --- | --- | --- | --- | --- | --- | --- |
| Catonella morbi | 12 | Not detected | -0.033 | -0.125 | 0.060 | 0.486 |
| Cedecea neteri | 4 | Not detected | 0.075 | -0.149 | 0.299 | 0.510 |
| Cellulomonas carbonis | 3 | Not detected | 0.181 | -0.010 | 0.372 | 0.063 |
| Cellulomonas flavigena | 7 | Not detected | -0.136 | -0.349 | 0.077 | 0.210 |
| Cellulosilyticum lentocellum | 56 | Not detected | -0.169 | -0.321 | -0.017 | 0.030 |
| Cellulosilyticum ruminicola | 9 | Not detected | 0.004 | -0.160 | 0.169 | 0.960 |
| Chlamydia psittaci | 5 | Not detected | -0.057 | -0.237 | 0.122 | 0.531 |
| Christensenella sp. AF73-05CM02 | 4 | Not detected | -0.042 | -0.249 | 0.165 | 0.692 |
| Christensenellaceae bacterium Phil1 | 3 | Not detected | 0.083 | -0.063 | 0.228 | 0.266 |
| Christensenellaceae bacterium Phil10 | 5 | Not detected | 0.192 | -0.016 | 0.399 | 0.071 |
| Christensenellaceae bacterium Phil7 | 3 | Not detected | 0.185 | -0.032 | 0.402 | 0.094 |
| Chryseobacterium gallinarum | 0 | Not detected | -0.026 | -0.270 | 0.218 | 0.832 |
| Chryseobacterium greenlandense | 19 | Not detected | -0.260 | -0.490 | -0.030 | 0.027 |
| Citrobacter amalonaticus | 0 | Not detected | -0.057 | -0.338 | 0.225 | 0.693 |
| Citrobacter freundii | 0 | Not detected | -0.065 | -0.352 | 0.222 | 0.658 |
| Cloacibacillus evryensis | 0 | Not detected | 0.013 | -0.262 | 0.287 | 0.928 |
| Cloacibacillus porcorum | 0 | Not detected | -0.137 | -0.367 | 0.093 | 0.243 |
| Cloacibacterium normanense | 156 | Not detected | -0.341 | -0.567 | -0.116 | 0.003 |
| Clostridia bacterium UC5.1-1D1 | 22 | Not detected | -0.070 | -0.150 | 0.011 | 0.089 |
| Clostridia bacterium UC5.1-1E11 | 11 | Not detected | 0.023 | -0.053 | 0.099 | 0.548 |
| Clostridiales bacterium 1_7_47FAA | 670 | Not detected | -0.149 | -0.238 | -0.061 | 0.001 |
| Clostridiales bacterium 59_14 | 11 | Not detected | 0.127 | -0.002 | 0.255 | 0.053 |
| Clostridiales bacterium DTU035 | 5 | Not detected | 0.060 | -0.107 | 0.227 | 0.482 |
| Clostridiales bacterium DTU036 | 5 | Not detected | 0.032 | -0.124 | 0.188 | 0.688 |
| Clostridiales bacterium DTU089 | 5 | Not detected | 0.071 | -0.066 | 0.207 | 0.311 |
| Clostridiales bacterium Firm_03 | 0 | Not detected | 0.004 | -0.185 | 0.194 | 0.963 |
| Clostridiales bacterium Firm_16 | 2 | Not detected | -0.003 | -0.217 | 0.211 | 0.978 |
| Clostridiales bacterium NK3B98 | 73 | Not detected | 0.130 | -0.046 | 0.307 | 0.149 |
| Clostridiales bacterium S5-A14a | 13 | Not detected | 0.015 | -0.063 | 0.093 | 0.707 |
| Clostridiales bacterium SIT11 | 5 | Not detected | -0.018 | -0.197 | 0.161 | 0.843 |
| Clostridiales bacterium VE202-08 | 13 | Not detected | -0.041 | -0.132 | 0.049 | 0.374 |
| Clostridioides difficile | 775 | 0.6 | -0.166 | -0.240 | -0.091 | 0.000 |
| Clostridioides mangenotii | 5 | Not detected | -0.051 | -0.225 | 0.124 | 0.571 |
| Clostridium baratii | 7 | Not detected | -0.079 | -0.285 | 0.127 | 0.454 |
| Clostridium beijerinckii | 5 | Not detected | 0.074 | -0.116 | 0.264 | 0.445 |
| Clostridium bolteae CAG:59 | 492 | Not detected | -0.183 | -0.326 | -0.040 | 0.012 |
| Clostridium botulinum | 4 | Not detected | 0.126 | -0.074 | 0.327 | 0.218 |
| Clostridium butyricum | 4 | Not detected | 0.061 | -0.145 | 0.266 | 0.562 |
| Clostridium cadaveris | 99 | Not detected | 0.101 | -0.064 | 0.265 | 0.231 |
| Clostridium carboxidivorans | 5 | Not detected | 0.066 | -0.065 | 0.197 | 0.325 |
| Clostridium celatum | 0 | Not detected | 0.046 | -0.249 | 0.340 | 0.762 |
| Clostridium cellulovorans | 37 | Not detected | 0.046 | -0.066 | 0.158 | 0.424 |
| Clostridium colicanis | 5 | Not detected | 0.024 | -0.136 | 0.185 | 0.765 |

| Clostridium disporicum | 0 | Not detected | -0.031 | -0.173 | 0.110 | 0.665 |
| --- | --- | --- | --- | --- | --- | --- |
| Clostridium ihumii | 10 | Not detected | -0.045 | -0.217 | 0.127 | 0.605 |
| Clostridium intestinale | 10 | Not detected | -0.040 | -0.185 | 0.105 | 0.586 |
| Clostridium kluyveri | 24 | Not detected | -0.182 | -0.376 | 0.011 | 0.065 |
| Clostridium leptum CAG:27 | 0 | Not detected | -0.002 | -0.224 | 0.219 | 0.985 |
| Clostridium lundense | 7 | Not detected | -0.178 | -0.370 | 0.015 | 0.070 |
| Clostridium magnum | 7 | Not detected | -0.022 | -0.164 | 0.120 | 0.762 |
| Clostridium neonatale | 4 | Not detected | 0.068 | -0.103 | 0.240 | 0.437 |
| Clostridium nexile CAG:348 | 6 | Not detected | -0.109 | -0.250 | 0.032 | 0.131 |
| Clostridium paraputrificum | 0 | Not detected | -0.023 | -0.267 | 0.220 | 0.851 |
| Clostridium perfringens | 0 | Not detected | 0.050 | -0.249 | 0.349 | 0.744 |
| Clostridium phoceensis | 2 | Not detected | -0.040 | -0.155 | 0.075 | 0.498 |
| Clostridium polynesiense | 5 | Not detected | -0.064 | -0.251 | 0.124 | 0.507 |
| Clostridium saccharobutylicum | 5 | Not detected | 0.041 | -0.136 | 0.219 | 0.649 |
| Clostridium saccharoperbutylacetonicum | 5 | Not detected | 0.033 | -0.140 | 0.206 | 0.708 |
| Clostridium sartagoforme | 2 | Not detected | -0.019 | -0.230 | 0.192 | 0.862 |
| Clostridium sp. 44_14 | 234 | Not detected | 0.162 | -0.011 | 0.336 | 0.067 |
| Clostridium sp. ASF356 | 14 | Not detected | -0.146 | -0.344 | 0.053 | 0.150 |
| Clostridium sp. Ade.TY | 9 | Not detected | -0.070 | -0.252 | 0.111 | 0.448 |
| Clostridium sp. CAG:1193 | 4 | Not detected | 0.135 | -0.084 | 0.355 | 0.227 |
| Clostridium sp. CAG:122 | 304 | Not detected | 0.313 | 0.087 | 0.539 | 0.007 |
| Clostridium sp. CAG:127 | 746 | 0.6 | 0.325 | 0.070 | 0.581 | 0.013 |
| Clostridium sp. CAG:138 | 0 | Not detected | 0.255 | -0.087 | 0.596 | 0.144 |
| Clostridium sp. CAG:167 | 3 | Not detected | 0.352 | 0.055 | 0.649 | 0.020 |
| Clostridium sp. CAG:226 | 2 | Not detected | 0.142 | -0.119 | 0.403 | 0.287 |
| Clostridium sp. CAG:230 | 4 | Not detected | 0.164 | -0.017 | 0.345 | 0.076 |
| Clostridium sp. CAG:242 | 0 | Not detected | -0.035 | -0.295 | 0.226 | 0.794 |
| Clostridium sp. CAG:245 | 3 | Not detected | 0.414 | 0.023 | 0.804 | 0.038 |
| Clostridium sp. CAG:245_30_32 | 4 | Not detected | 0.110 | -0.049 | 0.269 | 0.175 |
| Clostridium sp. CAG:264 | 819 | 0.6 | 0.361 | 0.105 | 0.617 | 0.006 |
| Clostridium sp. CAG:277 | 401 | Not detected | 0.231 | 0.051 | 0.411 | 0.012 |
| Clostridium sp. CAG:288 | 3 | Not detected | 0.246 | -0.030 | 0.523 | 0.081 |
| Clostridium sp. CAG:299 | 0 | Not detected | -0.164 | -0.392 | 0.065 | 0.160 |
| Clostridium sp. CAG:307_30_263 | 30 | Not detected | 0.150 | 0.000 | 0.300 | 0.050 |
| Clostridium sp. CAG:349 | 0 | Not detected | -0.026 | -0.389 | 0.336 | 0.886 |
| Clostridium sp. CAG:352 | 2 | Not detected | 0.085 | -0.137 | 0.307 | 0.452 |
| Clostridium sp. CAG:413 | 2 | Not detected | 0.241 | -0.019 | 0.502 | 0.070 |
| Clostridium sp. CAG:448 | 4 | Not detected | 0.290 | 0.001 | 0.578 | 0.049 |
| Clostridium sp. CAG:451 | 3 | Not detected | 0.110 | -0.184 | 0.404 | 0.464 |
| Clostridium sp. CAG:465 | 0 | Not detected | 0.118 | -0.163 | 0.399 | 0.409 |
| Clostridium sp. CAG:505 | 0 | Not detected | -0.182 | -0.447 | 0.083 | 0.179 |
| Clostridium sp. CAG:568 | 0 | Not detected | 0.207 | -0.085 | 0.499 | 0.165 |
| Clostridium sp. CAG:58 | 4 | Not detected | -0.160 | -0.349 | 0.030 | 0.098 |
| Clostridium sp. CAG:590 | 251 | Not detected | 0.193 | 0.054 | 0.331 | 0.007 |

| Clostridium sp. CAG:594 | 4 | Not detected | 0.377 | 0.056 | 0.698 | 0.022 |
| --- | --- | --- | --- | --- | --- | --- |
| Clostridium sp. CAG:628 | 795 | 0.6 | 0.478 | 0.178 | 0.778 | 0.002 |
| Clostridium sp. CAG:632 | 436 | Not detected | 0.169 | -0.013 | 0.351 | 0.070 |
| Clostridium sp. CAG:678 | 0 | Not detected | -0.030 | -0.241 | 0.181 | 0.781 |
| Clostridium sp. CAG:762 | 4 | Not detected | 0.255 | 0.002 | 0.509 | 0.049 |
| Clostridium sp. CAG:768 | 7 | Not detected | 0.171 | 0.003 | 0.340 | 0.047 |
| Clostridium sp. CAG:780 | 0 | Not detected | 0.040 | -0.273 | 0.354 | 0.801 |
| Clostridium sp. CAG:793 | 4 | Not detected | 0.184 | -0.070 | 0.438 | 0.157 |
| Clostridium sp. CAG:81 | 17 | Not detected | 0.132 | -0.003 | 0.266 | 0.055 |
| Clostridium sp. CAG:91 | 3 | Not detected | 0.187 | -0.036 | 0.410 | 0.101 |
| Clostridium sp. CAG:964 | 4 | Not detected | -0.029 | -0.163 | 0.104 | 0.667 |
| Clostridium sp. CL-6 | 8 | Not detected | 0.094 | -0.060 | 0.247 | 0.231 |
| Clostridium sp. HGF2 | 78 | Not detected | -0.075 | -0.154 | 0.004 | 0.061 |
| Clostridium sp. K4410.MGS-306 | 18 | Not detected | -0.093 | -0.194 | 0.008 | 0.070 |
| Clostridium sp. KLE 1755 | 306 | Not detected | -0.146 | -0.268 | -0.023 | 0.020 |
| Clostridium sp. KNHs205 | 35 | Not detected | 0.019 | -0.049 | 0.088 | 0.582 |
| Clostridium sp. KNHs209 | 77 | Not detected | 0.053 | -0.013 | 0.120 | 0.114 |
| Clostridium sp. KNHs214 | 24 | Not detected | 0.076 | -0.037 | 0.189 | 0.186 |
| Clostridium sp. L2-50 | 6 | Not detected | 0.078 | -0.025 | 0.182 | 0.138 |
| Clostridium sp. LF2 | 5 | Not detected | 0.016 | -0.127 | 0.159 | 0.823 |
| Clostridium sp. M62/1 | 15 | Not detected | -0.011 | -0.076 | 0.055 | 0.749 |
| Clostridium sp. ND2 | 7 | Not detected | -0.042 | -0.213 | 0.129 | 0.629 |
| Clostridium sp. SY8519 | 20 | Not detected | 0.031 | -0.041 | 0.103 | 0.399 |
| Clostridium sulfidigenes | 10 | Not detected | -0.015 | -0.148 | 0.118 | 0.824 |
| Collinsella aerofaciens | 0 | Not detected | 0.098 | -0.097 | 0.293 | 0.326 |
| Collinsella intestinalis | 3 | Not detected | 0.158 | -0.061 | 0.376 | 0.157 |
| Collinsella sp. CAG:166 | 0 | Not detected | 0.102 | -0.117 | 0.320 | 0.361 |
| Collinsella sp. MS5 | 6 | Not detected | 0.147 | 0.000 | 0.294 | 0.051 |
| Collinsella stercoris | 4 | Not detected | 0.160 | -0.037 | 0.357 | 0.111 |
| Collinsella tanakaei | 3 | Not detected | 0.011 | -0.129 | 0.152 | 0.873 |
| Comamonas kerstersii | 0 | Not detected | 0.054 | -0.192 | 0.299 | 0.668 |
| Coprobacillus sp. D6 | 21 | Not detected | -0.085 | -0.182 | 0.012 | 0.085 |
| Coprobacter secundus | 5 | Not detected | -0.014 | -0.102 | 0.074 | 0.750 |
| Coprococcus comes | 6 | Not detected | 0.026 | -0.053 | 0.105 | 0.523 |
| Coprococcus eutactus | 9 | Not detected | 0.108 | -0.010 | 0.226 | 0.073 |
| Coprococcus eutactus CAG:665 | 3 | Not detected | 0.242 | -0.014 | 0.498 | 0.065 |
| Coprococcus sp. 43_8 | 0 | Not detected | 0.050 | -0.103 | 0.204 | 0.521 |
| Coprococcus sp. CAG:131 | 3 | Not detected | 0.239 | -0.009 | 0.487 | 0.059 |
| Coprococcus sp. CAG:131_42_139 | 3 | Not detected | 0.277 | 0.029 | 0.525 | 0.029 |
| Coriobacteriaceae bacterium 68-1-3 | 5 | Not detected | 0.059 | -0.098 | 0.216 | 0.459 |
| Coriobacteriaceae bacterium BV3Ac1 | 2 | Not detected | -0.038 | -0.245 | 0.168 | 0.718 |
| Coriobacteriales bacterium DNF00809 | 17 | Not detected | 0.092 | -0.091 | 0.275 | 0.323 |
| Coriobacterium glomerans | 5 | Not detected | -0.051 | -0.223 | 0.120 | 0.559 |
| Corynebacterium amycolatum | 13 | Not detected | 0.063 | -0.071 | 0.196 | 0.359 |

| Corynebacterium durum | 4 | Not detected | 0.021 | -0.186 | 0.228 | 0.842 |
| --- | --- | --- | --- | --- | --- | --- |
| Corynebacterium glucuronolyticum | 12 | Not detected | 0.084 | -0.076 | 0.244 | 0.303 |
| Corynebacterium singulare | 5 | Not detected | 0.015 | -0.142 | 0.171 | 0.853 |
| Corynebacterium urealyticum | 4 | Not detected | 0.057 | -0.116 | 0.230 | 0.518 |
| Criibacterium bergeronii | 4 | Not detected | 0.039 | -0.076 | 0.154 | 0.508 |
| Cronobacter sakazakii | 0 | Not detected | -0.039 | -0.315 | 0.237 | 0.781 |
| Cryptobacterium sp. CAG:338 | 0 | Not detected | 0.154 | -0.144 | 0.453 | 0.310 |
| Culturomica massiliensis | 3 | Not detected | -0.037 | -0.138 | 0.064 | 0.470 |
| Cutibacterium acnes | 302 | Not detected | -0.226 | -0.416 | -0.035 | 0.020 |
| Defluviitalea phaphyphila | 11 | Not detected | -0.031 | -0.170 | 0.108 | 0.658 |
| Dehalobacter restrictus | 5 | Not detected | 0.078 | -0.067 | 0.223 | 0.292 |
| Dendrosporobacter quercicolus | 5 | Not detected | 0.074 | -0.065 | 0.213 | 0.298 |
| Denitrobacterium detoxificans | 90 | Not detected | 0.083 | -0.056 | 0.223 | 0.242 |
| Desnuesiella massiliensis | 5 | Not detected | 0.026 | -0.127 | 0.180 | 0.737 |
| Desulfovibrio desulfuricans | 3 | Not detected | 0.066 | -0.187 | 0.319 | 0.610 |
| Desulfovibrio fairfieldensis | 0 | Not detected | 0.082 | -0.211 | 0.376 | 0.582 |
| Desulfovibrio piger | 0 | Not detected | -0.053 | -0.413 | 0.306 | 0.771 |
| Desulfovibrio vulgaris | 5 | Not detected | -0.018 | -0.185 | 0.149 | 0.833 |
| Devosia insulae | 8 | Not detected | -0.003 | -0.123 | 0.117 | 0.962 |
| Dialister invisus | 0 | Not detected | 0.012 | -0.360 | 0.384 | 0.950 |
| Dialister invisus CAG:218 | 0 | Not detected | -0.050 | -0.430 | 0.330 | 0.798 |
| Dialister micraerophilus | 4 | Not detected | 0.142 | -0.090 | 0.374 | 0.230 |
| Dialister sp. CAG:588 | 25 | Not detected | -0.200 | -0.403 | 0.003 | 0.054 |
| Dialister succinatiphilus | 1 | Not detected | 0.134 | -0.073 | 0.342 | 0.204 |
| Dickeya dadantii | 5 | Not detected | -0.016 | -0.180 | 0.148 | 0.849 |
| Dorea formicigenerans | 231 | Not detected | 0.094 | 0.021 | 0.166 | 0.012 |
| Dorea longicatena | 8 | Not detected | 0.084 | -0.020 | 0.188 | 0.115 |
| Dorea sp. 42_8 | 3 | Not detected | 0.019 | -0.103 | 0.142 | 0.755 |
| Dorea sp. 5-2 | 18 | Not detected | -0.004 | -0.060 | 0.052 | 0.882 |
| Dorea sp. CAG:105 | 0 | Not detected | 0.049 | -0.113 | 0.211 | 0.556 |
| Draconibacterium orientale | 10 | Not detected | -0.035 | -0.170 | 0.101 | 0.615 |
| Drancourtella massiliensis | 6 | Not detected | -0.101 | -0.234 | 0.031 | 0.135 |
| Dubosiella newyorkensis | 10 | Not detected | -0.004 | -0.153 | 0.144 | 0.953 |
| Dysgonomonas gadei | 10 | Not detected | -0.062 | -0.187 | 0.062 | 0.328 |
| Dysgonomonas mossii | 32 | Not detected | -0.135 | -0.284 | 0.014 | 0.075 |
| Dysgonomonas sp. BGC7 | 10 | Not detected | -0.126 | -0.309 | 0.057 | 0.177 |
| Dysgonomonas sp. HGC4 | 19 | Not detected | -0.096 | -0.254 | 0.062 | 0.236 |
| Edwardsiella ictaluri | 4 | Not detected | 0.049 | -0.118 | 0.217 | 0.564 |
| Eggerthella lenta | 0 | Not detected | -0.056 | -0.251 | 0.139 | 0.572 |
| Eggerthella sp. 51_9 | 659 | Not detected | 0.263 | 0.045 | 0.481 | 0.018 |
| Eggerthella sp. CAG:368 | 0 | Not detected | 0.116 | -0.136 | 0.368 | 0.367 |
| Eggerthella sp. YY7918 | 10 | Not detected | -0.163 | -0.326 | 0.001 | 0.051 |
| Eggerthellaceae bacterium AT8 | 21 | Not detected | 0.067 | -0.032 | 0.166 | 0.183 |
| Eggerthia catenaformis | 4 | Not detected | 0.093 | -0.113 | 0.299 | 0.375 |

| Eisenbergiella tayi | 107 | Not detected | -0.177 | -0.336 | -0.019 | 0.029 |
| --- | --- | --- | --- | --- | --- | --- |
| Elizabethkingia anophelis | 15 | Not detected | -0.275 | -0.490 | -0.060 | 0.012 |
| Emergencia timonensis | 208 | Not detected | -0.091 | -0.166 | -0.016 | 0.017 |
| Enhygromyxa salina | 4 | Not detected | -0.012 | -0.196 | 0.171 | 0.897 |
| Enorma massiliensis | 18 | Not detected | 0.093 | -0.013 | 0.200 | 0.086 |
| Enorma timonensis | 254 | Not detected | 0.182 | 0.053 | 0.311 | 0.006 |
| Enterobacter cloacae | 0 | Not detected | 0.042 | -0.247 | 0.331 | 0.775 |
| Enterobacter lignolyticus | 0 | Not detected | -0.102 | -0.367 | 0.162 | 0.449 |
| Enterobacter sp. 638 | 0 | Not detected | -0.055 | -0.301 | 0.192 | 0.664 |
| Enterobacteriaceae bacterium LSJC7 | 4 | Not detected | -0.029 | -0.209 | 0.151 | 0.750 |
| Enterococcus avium | 5 | Not detected | 0.003 | -0.169 | 0.175 | 0.970 |
| Enterococcus cecorum | 0 | Not detected | -0.087 | -0.236 | 0.061 | 0.248 |
| Enterococcus columbae | 0 | Not detected | -0.117 | -0.311 | 0.077 | 0.236 |
| Enterococcus durans | 4 | Not detected | -0.228 | -0.456 | -0.001 | 0.049 |
| Enterococcus faecalis | 0 | Not detected | -0.047 | -0.217 | 0.122 | 0.586 |
| Enterococcus faecium | 3 | Not detected | 0.044 | -0.134 | 0.222 | 0.629 |
| Enterococcus gallinarum | 1 | Not detected | -0.188 | -0.361 | -0.014 | 0.034 |
| Enterococcus gilvus | 128 | Not detected | 0.128 | -0.037 | 0.293 | 0.128 |
| Enterococcus italicus | 17 | Not detected | -0.200 | -0.405 | 0.005 | 0.056 |
| Enterococcus massiliensis | 11 | Not detected | -0.202 | -0.399 | -0.004 | 0.046 |
| Enterococcus sp. HMSC064A12 | 7 | Not detected | -0.173 | -0.372 | 0.027 | 0.091 |
| Enterorhabdus caecimuris | 8 | Not detected | -0.095 | -0.297 | 0.107 | 0.357 |
| Enterorhabdus mucosicola | 4 | Not detected | 0.034 | -0.138 | 0.206 | 0.699 |
| Epulopiscium sp. 'N.t. morphotype B' | 6 | Not detected | 0.056 | -0.075 | 0.188 | 0.400 |
| Erwinia billingiae | 4 | Not detected | 0.021 | -0.142 | 0.184 | 0.798 |
| Erwinia oleae | 4 | Not detected | 0.104 | -0.102 | 0.310 | 0.322 |
| Erysipelatoclostridium ramosum | 0 | Not detected | -0.163 | -0.402 | 0.076 | 0.182 |
| Erysipelotrichaceae bacterium 21_3 | 723 | 0.6 | -0.181 | -0.283 | -0.080 | 0.000 |
| Erysipelotrichaceae bacterium 5_2_54FAA | 19 | Not detected | -0.044 | -0.123 | 0.034 | 0.269 |
| Erysipelotrichaceae bacterium CAG:64 | 14 | Not detected | -0.226 | -0.406 | -0.045 | 0.014 |
| Erysipelotrichaceae bacterium I46 | 675 | Not detected | -0.158 | -0.252 | -0.064 | 0.001 |
| Escherichia coli | 0 | Not detected | -0.110 | -0.371 | 0.152 | 0.411 |
| Escherichia vulneris | 0 | Not detected | -0.031 | -0.266 | 0.205 | 0.799 |
| Ethanoligenens harbinense | 213 | Not detected | 0.121 | -0.056 | 0.298 | 0.180 |
| Eubacterium dolichum CAG:375 | 736 | 0.6 | -0.358 | -0.624 | -0.091 | 0.009 |
| Eubacterium limosum | 2 | Not detected | -0.063 | -0.182 | 0.056 | 0.298 |
| Eubacterium plexicaudatum | 33 | Not detected | 0.030 | -0.037 | 0.096 | 0.379 |
| Eubacterium pyruvativorans | 96 | Not detected | 0.067 | -0.015 | 0.148 | 0.111 |
| Eubacterium ramulus | 13 | Not detected | 0.009 | -0.067 | 0.084 | 0.826 |
| Eubacterium sp. 14-2 | 38 | Not detected | -0.041 | -0.110 | 0.028 | 0.242 |
| Eubacterium sp. 3_1_31 | 30 | Not detected | -0.090 | -0.181 | 0.000 | 0.049 |
| Eubacterium sp. 45_250 | 0 | Not detected | 0.035 | -0.231 | 0.302 | 0.795 |
| Eubacterium sp. AB3007 | 8 | Not detected | -0.255 | -0.501 | -0.008 | 0.043 |
| Eubacterium sp. CAG:115 | 0 | Not detected | 0.185 | -0.211 | 0.581 | 0.360 |

| Eubacterium sp. CAG:146 | 4 | Not detected | 0.109 | -0.054 | 0.271 | 0.191 |
| --- | --- | --- | --- | --- | --- | --- |
| Eubacterium sp. CAG:192 | 155 | Not detected | 0.369 | 0.124 | 0.614 | 0.003 |
| Eubacterium sp. CAG:202 | 4 | Not detected | 0.286 | 0.034 | 0.539 | 0.026 |
| Eubacterium sp. CAG:251 | 3 | Not detected | 0.314 | 0.011 | 0.617 | 0.042 |
| Eubacterium sp. CAG:581 | 4 | Not detected | 0.164 | -0.104 | 0.431 | 0.230 |
| Eubacterium sp. CAG:786 | 0 | Not detected | 0.080 | -0.232 | 0.392 | 0.616 |
| Eubacterium sp. CAG:841 | 2 | Not detected | 0.280 | -0.012 | 0.573 | 0.060 |
| Eubacterium sp. ER2 | 13 | Not detected | -0.016 | -0.080 | 0.049 | 0.633 |
| Eubacterium ventriosum | 10 | Not detected | -0.006 | -0.100 | 0.089 | 0.909 |
| Eubacterium xylanophilum | 258 | Not detected | 0.170 | 0.019 | 0.321 | 0.028 |
| Facklamia hominis | 5 | Not detected | 0.033 | -0.154 | 0.220 | 0.728 |
| Facklamia ignava | 5 | Not detected | 0.034 | -0.151 | 0.219 | 0.716 |
| Facklamia languida | 5 | Not detected | 0.009 | -0.166 | 0.184 | 0.917 |
| Faecalibacterium prausnitzii | 104 | Not detected | 0.126 | 0.006 | 0.247 | 0.040 |
| Faecalibacterium sp. CAG:1138 | 4 | Not detected | 0.219 | -0.046 | 0.483 | 0.105 |
| Faecalibacterium sp. CAG:82-related_59_9 | 0 | Not detected | 0.066 | -0.117 | 0.250 | 0.480 |
| Faecalibaculum rodentium | 7 | Not detected | 0.027 | -0.170 | 0.224 | 0.790 |
| Faecalicoccus pleomorphus | 62 | Not detected | 0.082 | -0.018 | 0.181 | 0.110 |
| Faecalitalea cylindroides | 13 | Not detected | -0.007 | -0.071 | 0.058 | 0.841 |
| Fenollaria massiliensis | 7 | Not detected | -0.066 | -0.286 | 0.155 | 0.559 |
| Fermentimonas caenicola | 306 | Not detected | -0.267 | -0.453 | -0.081 | 0.005 |
| Fibrobacter sp. UWB12 | 4 | Not detected | 0.032 | -0.075 | 0.140 | 0.554 |
| Fibrobacter sp. UWCM | 10 | Not detected | 0.160 | 0.013 | 0.307 | 0.033 |
| Fibrobacter sp. UWOV1 | 7 | Not detected | 0.009 | -0.095 | 0.112 | 0.868 |
| Fibrobacterales bacterium Fibro_02 | 104 | Not detected | 0.078 | -0.034 | 0.191 | 0.173 |
| Fibrobacteria bacterium AD80 | 134 | Not detected | 0.076 | -0.036 | 0.188 | 0.186 |
| Filifactor alocis | 3 | Not detected | -0.077 | -0.275 | 0.122 | 0.449 |
| Finegoldia magna | 26 | Not detected | -0.161 | -0.361 | 0.038 | 0.114 |
| Firmicutes bacterium ASF500 | 13 | Not detected | 0.018 | -0.052 | 0.088 | 0.614 |
| Firmicutes bacterium CAG:102 | 0 | Not detected | 0.100 | -0.155 | 0.356 | 0.441 |
| Firmicutes bacterium CAG:137 | 0 | Not detected | -0.130 | -0.433 | 0.173 | 0.401 |
| Firmicutes bacterium CAG:137_57_8 | 0 | Not detected | -0.024 | -0.248 | 0.200 | 0.833 |
| Firmicutes bacterium CAG:145 | 62 | Not detected | -0.254 | -0.523 | 0.015 | 0.064 |
| Firmicutes bacterium CAG:176 | 0 | Not detected | 0.324 | -0.010 | 0.658 | 0.057 |
| Firmicutes bacterium CAG:176_59_8 | 0 | Not detected | 0.250 | -0.049 | 0.548 | 0.101 |
| Firmicutes bacterium CAG:227 | 4 | Not detected | -0.173 | -0.390 | 0.043 | 0.116 |
| Firmicutes bacterium CAG:238 | 0 | Not detected | 0.063 | -0.192 | 0.318 | 0.630 |
| Firmicutes bacterium CAG:24 | 3 | Not detected | 0.094 | -0.084 | 0.272 | 0.299 |
| Firmicutes bacterium CAG:240 | 0 | Not detected | 0.075 | -0.215 | 0.365 | 0.612 |
| Firmicutes bacterium CAG:270 | 0 | Not detected | -0.060 | -0.272 | 0.152 | 0.579 |
| Firmicutes bacterium CAG:272 | 3 | Not detected | 0.218 | -0.054 | 0.489 | 0.116 |
| Firmicutes bacterium CAG:272_52_7 | 0 | Not detected | 0.042 | -0.158 | 0.241 | 0.684 |
| Firmicutes bacterium CAG:308 | 8 | Not detected | -0.100 | -0.273 | 0.074 | 0.260 |
| Firmicutes bacterium CAG:313 | 0 | Not detected | 0.092 | -0.274 | 0.458 | 0.623 |

| Firmicutes bacterium CAG:41 | 465 | Not detected | 0.259 | 0.030 | 0.488 | 0.027 |
| --- | --- | --- | --- | --- | --- | --- |
| Firmicutes bacterium CAG:466 | 0 | Not detected | -0.093 | -0.278 | 0.091 | 0.321 |
| Firmicutes bacterium CAG:475 | 0 | Not detected | -0.031 | -0.297 | 0.236 | 0.822 |
| Firmicutes bacterium CAG:536 | 724 | 0.6 | 0.328 | 0.130 | 0.526 | 0.001 |
| Firmicutes bacterium CAG:552 | 4 | Not detected | 0.300 | 0.047 | 0.554 | 0.020 |
| Firmicutes bacterium CAG:555 | 0 | Not detected | -0.002 | -0.242 | 0.237 | 0.987 |
| Firmicutes bacterium CAG:65 | 590 | Not detected | 0.266 | 0.063 | 0.469 | 0.010 |
| Firmicutes bacterium CAG:791 | 21 | Not detected | 0.161 | 0.032 | 0.290 | 0.014 |
| Firmicutes bacterium CAG:83 | 534 | Not detected | 0.270 | 0.055 | 0.484 | 0.014 |
| Firmicutes bacterium CAG:882 | 4 | Not detected | 0.106 | -0.052 | 0.265 | 0.189 |
| Firmicutes bacterium CAG:884 | 4 | Not detected | 0.214 | -0.035 | 0.463 | 0.092 |
| Firmicutes bacterium CAG:94 | 0 | Not detected | -0.111 | -0.359 | 0.136 | 0.377 |
| Firmicutes bacterium M10-2 | 620 | Not detected | 0.272 | 0.072 | 0.473 | 0.008 |
| Firmicutes bacterium ZOR0006 | 0 | Not detected | 0.029 | -0.185 | 0.243 | 0.790 |
| Flavonifractor plautii | 13 | Not detected | -0.179 | -0.336 | -0.022 | 0.025 |
| Fournierella massiliensis | 9 | Not detected | 0.017 | -0.059 | 0.094 | 0.658 |
| Franconibacter pulveris | 0 | Not detected | 0.003 | -0.218 | 0.225 | 0.976 |
| Fretibacterium fastidiosum | 8 | Not detected | -0.072 | -0.219 | 0.075 | 0.335 |
| Fusicatenibacter saccharivorans | 19 | Not detected | 0.049 | -0.041 | 0.139 | 0.287 |
| Fusobacterium mortiferum | 0 | Not detected | -0.137 | -0.417 | 0.143 | 0.338 |
| Fusobacterium necrophorum | 394 | Not detected | -0.215 | -0.403 | -0.026 | 0.026 |
| Fusobacterium nucleatum | 0 | Not detected | -0.036 | -0.304 | 0.232 | 0.793 |
| Fusobacterium periodonticum | 0 | Not detected | -0.018 | -0.250 | 0.214 | 0.879 |
| Gabonia massiliensis | 0 | Not detected | -0.046 | -0.191 | 0.099 | 0.537 |
| Gabonibacter massiliensis | 5 | Not detected | -0.147 | -0.322 | 0.027 | 0.098 |
| Gallibacterium anatis | 5 | Not detected | 0.083 | -0.050 | 0.216 | 0.220 |
| Gardnerella vaginalis | 19 | Not detected | -0.181 | -0.390 | 0.028 | 0.090 |
| Gemella haemolysans | 10 | Not detected | -0.120 | -0.315 | 0.075 | 0.229 |
| Gemella sanguinis | 0 | Not detected | -0.059 | -0.297 | 0.180 | 0.629 |
| Gemmata obscuriglobus | 92 | Not detected | 0.143 | -0.026 | 0.312 | 0.097 |
| Geofilum rubicundum | 73 | Not detected | -0.140 | -0.300 | 0.020 | 0.086 |
| Gilliamella apicola | 4 | Not detected | 0.092 | -0.071 | 0.255 | 0.270 |
| Globicatella sanguinis | 4 | Not detected | 0.132 | -0.055 | 0.319 | 0.167 |
| Gordonibacter pamelaeae | 4 | Not detected | 0.072 | -0.065 | 0.210 | 0.303 |
| Granulicatella adiacens | 0 | Not detected | -0.016 | -0.247 | 0.215 | 0.891 |
| Granulicatella elegans | 3 | Not detected | -0.064 | -0.258 | 0.129 | 0.514 |
| Haemophilus influenzae | 165 | Not detected | 0.323 | 0.061 | 0.584 | 0.016 |
| Haemophilus parainfluenzae | 564 | Not detected | 0.347 | 0.033 | 0.662 | 0.030 |
| Haemophilus sputorum | 3 | Not detected | 0.139 | -0.129 | 0.407 | 0.308 |
| Hafnia sp. HMSC23F03 | 1 | Not detected | 0.042 | -0.158 | 0.242 | 0.683 |
| Hathewaya proteolytica | 8 | Not detected | -0.027 | -0.216 | 0.162 | 0.780 |
| Heliobacterium sp. N15.MGS-306 | 11 | Not detected | 0.016 | -0.066 | 0.099 | 0.695 |
| Hespellia stercorisuis | 46 | Not detected | -0.064 | -0.138 | 0.010 | 0.092 |
| Holdemanella biformis | 3 | Not detected | 0.081 | -0.060 | 0.221 | 0.261 |

| Holdemania filiformis | 26 | Not detected | -0.054 | -0.139 | 0.031 | 0.210 |
| --- | --- | --- | --- | --- | --- | --- |
| Holdemania massiliensis | 4 | Not detected | -0.030 | -0.147 | 0.086 | 0.614 |
| Holdemania sp. Marseille-P2844 | 6 | Not detected | -0.018 | -0.118 | 0.083 | 0.732 |
| Hungatella hathewayi | 244 | Not detected | -0.108 | -0.200 | -0.016 | 0.021 |
| Hydrogenoanaerobacterium saccharovorans | 14 | Not detected | 0.050 | -0.071 | 0.171 | 0.420 |
| Idiomarina zobellii | 4 | Not detected | 0.239 | 0.008 | 0.469 | 0.043 |
| Ileibacterium valens | 5 | Not detected | 0.096 | -0.114 | 0.306 | 0.368 |
| Intestinibacter bartlettii | 0 | Not detected | -0.115 | -0.296 | 0.066 | 0.215 |
| Intestinimonas butyriciproducens | 9 | Not detected | 0.070 | -0.038 | 0.178 | 0.206 |
| Intestinimonas massiliensis | 6 | Not detected | 0.042 | -0.057 | 0.141 | 0.405 |
| Izhakiella sp. D4N98 | 2 | Not detected | -0.070 | -0.246 | 0.105 | 0.433 |
| Jeotgalibaca sp. PTS2502 | 11 | Not detected | -0.037 | -0.191 | 0.116 | 0.633 |
| Johnsonella ignava | 45 | Not detected | 0.078 | -0.012 | 0.168 | 0.090 |
| Kallipyga massiliensis | 236 | Not detected | -0.266 | -0.453 | -0.079 | 0.005 |
| Kandleria vitulina | 236 | Not detected | 0.309 | 0.046 | 0.573 | 0.022 |
| Kiloniella litopenaei | 4 | Not detected | 0.079 | -0.110 | 0.268 | 0.410 |
| Kingella kingae | 4 | Not detected | 0.061 | -0.117 | 0.240 | 0.502 |
| Klebsiella oxytoca | 0 | Not detected | -0.065 | -0.375 | 0.246 | 0.684 |
| Klebsiella pneumoniae | 0 | Not detected | 0.027 | -0.289 | 0.344 | 0.866 |
| Klebsiella sp. RIT-PI-d | 0 | Not detected | -0.084 | -0.320 | 0.152 | 0.484 |
| Kluyvera ascorbata | 0 | Not detected | 0.173 | -0.115 | 0.462 | 0.240 |
| Kluyvera cryocrescens | 0 | Not detected | 0.143 | -0.153 | 0.438 | 0.345 |
| Kluyvera intermedia | 0 | Not detected | 0.032 | -0.239 | 0.304 | 0.815 |
| Kocuria sp. ZOR0020 | 230 | Not detected | -0.227 | -0.381 | -0.074 | 0.004 |
| Kosakonia sacchari | 0 | Not detected | -0.048 | -0.298 | 0.203 | 0.710 |
| Lachnoanaerobaculum saburreum | 19 | Not detected | 0.066 | -0.028 | 0.159 | 0.168 |
| Lachnoanaerobaculum sp. OBRC5-5 | 8 | Not detected | 0.011 | -0.077 | 0.099 | 0.811 |
| Lachnobacterium bovis | 14 | Not detected | 0.025 | -0.064 | 0.114 | 0.577 |
| Lachnoclostridium phytofermentans | 33 | Not detected | 0.025 | -0.044 | 0.094 | 0.472 |
| Lachnospira multipara | 393 | Not detected | 0.144 | 0.021 | 0.268 | 0.022 |
| Lachnospiraceae bacterium 10-1 | 47 | Not detected | 0.044 | -0.013 | 0.101 | 0.130 |
| Lachnospiraceae bacterium 28-4 | 25 | Not detected | 0.021 | -0.037 | 0.079 | 0.476 |
| Lachnospiraceae bacterium 2_1_46FAA | 685 | Not detected | -0.152 | -0.239 | -0.065 | 0.001 |
| Lachnospiraceae bacterium 3-1 | 13 | Not detected | 0.000 | -0.070 | 0.069 | 0.990 |
| Lachnospiraceae bacterium 3-2 | 50 | Not detected | -0.058 | -0.124 | 0.008 | 0.086 |
| Lachnospiraceae bacterium A2 | 14 | Not detected | 0.016 | -0.056 | 0.087 | 0.671 |
| Lachnospiraceae bacterium A4 | 18 | Not detected | -0.019 | -0.083 | 0.044 | 0.547 |
| Lachnospiraceae bacterium AB2028 | 154 | Not detected | 0.165 | -0.032 | 0.362 | 0.102 |
| Lachnospiraceae bacterium AC2012 | 117 | Not detected | 0.077 | -0.002 | 0.155 | 0.055 |
| Lachnospiraceae bacterium AC2014 | 7 | Not detected | 0.094 | -0.028 | 0.216 | 0.131 |
| Lachnospiraceae bacterium AC2028 | 70 | Not detected | 0.174 | 0.033 | 0.314 | 0.015 |
| Lachnospiraceae bacterium AC2029 | 118 | Not detected | 0.055 | -0.030 | 0.139 | 0.204 |
| Lachnospiraceae bacterium AC2031 | 121 | Not detected | 0.094 | -0.029 | 0.217 | 0.135 |
| Lachnospiraceae bacterium AC3007 | 16 | Not detected | 0.103 | -0.063 | 0.269 | 0.222 |

| Lachnospiraceae bacterium AD3010 | 5 | Not detected | 0.089 | -0.101 | 0.279 | 0.357 |
| --- | --- | --- | --- | --- | --- | --- |
| Lachnospiraceae bacterium C6A11 | 258 | Not detected | 0.124 | 0.005 | 0.244 | 0.041 |
| Lachnospiraceae bacterium COE1 | 7 | Not detected | 0.036 | -0.054 | 0.125 | 0.437 |
| Lachnospiraceae bacterium FD2005 | 119 | Not detected | 0.159 | -0.002 | 0.320 | 0.053 |
| Lachnospiraceae bacterium FE2018 | 11 | Not detected | -0.102 | -0.292 | 0.088 | 0.294 |
| Lachnospiraceae bacterium G41 | 129 | Not detected | 0.210 | 0.041 | 0.380 | 0.015 |
| Lachnospiraceae bacterium KHCPX20 | 22 | Not detected | 0.108 | -0.078 | 0.294 | 0.255 |
| Lachnospiraceae bacterium M18-1 | 13 | Not detected | -0.015 | -0.082 | 0.053 | 0.670 |
| Lachnospiraceae bacterium MC2017 | 11 | Not detected | -0.068 | -0.204 | 0.068 | 0.328 |
| Lachnospiraceae bacterium NC2004 | 5 | Not detected | 0.070 | -0.108 | 0.249 | 0.441 |
| Lachnospiraceae bacterium NC2008 | 25 | Not detected | 0.071 | -0.092 | 0.234 | 0.392 |
| Lachnospiraceae bacterium ND2006 | 113 | Not detected | 0.141 | -0.031 | 0.312 | 0.108 |
| Lachnospiraceae bacterium NK3A20 | 9 | Not detected | -0.088 | -0.276 | 0.101 | 0.362 |
| Lachnospiraceae bacterium NK4A179 | 80 | Not detected | 0.155 | -0.018 | 0.328 | 0.079 |
| Lachnospiraceae bacterium NLAE-zl-G231 | 17 | Not detected | -0.198 | -0.375 | -0.020 | 0.030 |
| Lachnospiraceae bacterium P6B14 | 78 | Not detected | 0.101 | -0.035 | 0.236 | 0.145 |
| Lachnospiraceae bacterium TF01-11 | 139 | Not detected | 0.101 | 0.010 | 0.192 | 0.029 |
| Lachnospiraceae bacterium TWA4 | 116 | Not detected | 0.035 | -0.057 | 0.128 | 0.455 |
| Lachnospiraceae bacterium V9D3004 | 81 | Not detected | 0.181 | 0.012 | 0.350 | 0.036 |
| Lachnospiraceae bacterium YSB2008 | 180 | Not detected | 0.127 | 0.005 | 0.248 | 0.041 |
| Lachnospiraceae bacterium mt14 | 5 | Not detected | 0.032 | -0.127 | 0.192 | 0.693 |
| Lachnospiraceae bacterium oral taxon 500 | 44 | Not detected | 0.061 | -0.010 | 0.132 | 0.090 |
| Lactobacillus acidophilus | 5 | Not detected | 0.037 | -0.098 | 0.171 | 0.595 |
| Lactobacillus amylotrophicus | 11 | Not detected | -0.081 | -0.257 | 0.096 | 0.372 |
| Lactobacillus animalis | 2 | Not detected | 0.074 | -0.102 | 0.249 | 0.410 |
| Lactobacillus antri | 7 | Not detected | -0.130 | -0.325 | 0.065 | 0.192 |
| Lactobacillus aviarius | 21 | Not detected | -0.007 | -0.101 | 0.086 | 0.876 |
| Lactobacillus casei | 16 | Not detected | -0.195 | -0.444 | 0.054 | 0.124 |
| Lactobacillus curvatus | 7 | Not detected | -0.166 | -0.360 | 0.028 | 0.093 |
| Lactobacillus delbrueckii | 1 | Not detected | -0.148 | -0.368 | 0.071 | 0.185 |
| Lactobacillus equi | 8 | Not detected | -0.136 | -0.326 | 0.054 | 0.160 |
| Lactobacillus fermentum | 0 | Not detected | -0.119 | -0.399 | 0.160 | 0.402 |
| Lactobacillus gasseri | 0 | Not detected | -0.162 | -0.443 | 0.120 | 0.260 |
| Lactobacillus helveticus | 8 | Not detected | -0.094 | -0.299 | 0.112 | 0.371 |
| Lactobacillus ingluviei | 5 | Not detected | -0.034 | -0.196 | 0.127 | 0.677 |
| Lactobacillus jensenii | 443 | Not detected | -0.205 | -0.380 | -0.030 | 0.021 |
| Lactobacillus kefiranofaciens | 8 | Not detected | -0.050 | -0.212 | 0.112 | 0.544 |
| Lactobacillus manihotivorans | 21 | Not detected | 0.143 | -0.038 | 0.323 | 0.121 |
| Lactobacillus mucosae | 1 | Not detected | 0.007 | -0.179 | 0.194 | 0.937 |
| Lactobacillus paracasei | 1 | Not detected | -0.186 | -0.441 | 0.069 | 0.153 |
| Lactobacillus pasteurii | 24 | Not detected | 0.100 | -0.047 | 0.247 | 0.184 |
| Lactobacillus plantarum | 0 | Not detected | -0.043 | -0.256 | 0.171 | 0.695 |
| Lactobacillus rhamnosus | 5 | Not detected | -0.189 | -0.419 | 0.041 | 0.108 |
| Lactobacillus ruminis CAG:367 | 4 | Not detected | 0.108 | -0.074 | 0.291 | 0.245 |

| Lactobacillus sakei | 0 | Not detected | -0.068 | -0.258 | 0.123 | 0.486 |
| --- | --- | --- | --- | --- | --- | --- |
| Lactobacillus salivarius | 0 | Not detected | 0.029 | -0.243 | 0.300 | 0.836 |
| Lactobacillus vaginalis | 0 | Not detected | -0.157 | -0.396 | 0.082 | 0.198 |
| Lactococcus garvieae | 7 | Not detected | -0.156 | -0.352 | 0.041 | 0.120 |
| Lactococcus lactis | 251 | Not detected | -0.319 | -0.616 | -0.023 | 0.035 |
| Lactococcus piscium | 8 | Not detected | -0.130 | -0.326 | 0.066 | 0.194 |
| Lactonifactor longoviformis | 25 | Not detected | -0.034 | -0.094 | 0.026 | 0.271 |
| Lamprocystis purpurea | 0 | Not detected | -0.069 | -0.306 | 0.169 | 0.571 |
| Lascolabacillus massiliensis | 11 | Not detected | -0.082 | -0.241 | 0.078 | 0.314 |
| Lautropia mirabilis | 1 | Not detected | -0.016 | -0.216 | 0.183 | 0.873 |
| Lawsonia intracellularis | 5 | Not detected | -0.036 | -0.227 | 0.155 | 0.713 |
| Leclercia adecarboxylata | 0 | Not detected | 0.071 | -0.208 | 0.350 | 0.617 |
| Lelliottia amnigena | 0 | Not detected | 0.002 | -0.303 | 0.307 | 0.989 |
| Lentimicrobium saccharophilum | 8 | Not detected | -0.221 | -0.427 | -0.015 | 0.035 |
| Leuconostoc citreum | 2 | Not detected | -0.018 | -0.195 | 0.159 | 0.841 |
| Leuconostoc lactis | 1 | Not detected | 0.024 | -0.186 | 0.234 | 0.824 |
| Leuconostoc mesenteroides | 0 | Not detected | -0.003 | -0.228 | 0.222 | 0.979 |
| Levyella massiliensis | 7 | Not detected | 0.001 | -0.096 | 0.098 | 0.989 |
| Libanicoccus massiliensis | 4 | Not detected | 0.144 | -0.030 | 0.319 | 0.105 |
| Luteimonas sp. FCS-9 | 7 | Not detected | 0.038 | -0.110 | 0.187 | 0.612 |
| Lutispora thermophila | 6 | Not detected | 0.031 | -0.140 | 0.202 | 0.719 |
| Macellibacteroides sp. HH-ZS | 177 | Not detected | -0.156 | -0.288 | -0.024 | 0.020 |
| Macrococcus caseolyticus | 36 | Not detected | -0.238 | -0.429 | -0.047 | 0.015 |
| Mageeibacillus indolicus | 9 | Not detected | -0.058 | -0.195 | 0.080 | 0.412 |
| Mangrovibacter sp. MFB070 | 0 | Not detected | -0.007 | -0.229 | 0.215 | 0.948 |
| Mannheimia haemolytica | 4 | Not detected | 0.150 | -0.036 | 0.336 | 0.115 |
| Marvinbryantia formatexigens | 22 | Not detected | -0.056 | -0.142 | 0.030 | 0.200 |
| Massilioclostridium coli | 8 | Not detected | 0.002 | -0.085 | 0.088 | 0.972 |
| Massiliomicrobiota timonensis | 4 | Not detected | 0.100 | -0.096 | 0.296 | 0.318 |
| Mediterranea massiliensis | 0 | Not detected | 0.005 | -0.107 | 0.116 | 0.936 |
| Megamonas funiformis | 0 | Not detected | -0.122 | -0.458 | 0.215 | 0.480 |
| Megasphaera cerevisiae | 3 | Not detected | 0.040 | -0.214 | 0.293 | 0.758 |
| Megasphaera elsdenii | 0 | Not detected | -0.161 | -0.492 | 0.169 | 0.339 |
| Megasphaera genomosp. type_1 | 79 | Not detected | 0.060 | -0.004 | 0.124 | 0.067 |
| Megasphaera massiliensis | 0 | Not detected | -0.210 | -0.504 | 0.084 | 0.162 |
| Megasphaera micronuciformis | 1 | Not detected | -0.151 | -0.380 | 0.078 | 0.196 |
| Megasphaera sp. BV3C16-1 | 4 | Not detected | 0.126 | -0.068 | 0.319 | 0.205 |
| Megasphaera sp. UPII 135-E | 30 | Not detected | 0.022 | -0.043 | 0.087 | 0.503 |
| Merdibacter massiliensis | 11 | Not detected | -0.002 | -0.075 | 0.071 | 0.957 |
| Methanobrevibacter smithii | 0 | Not detected | -0.030 | -0.426 | 0.365 | 0.881 |
| Methanobrevibacter smithii CAG:186 | 0 | Not detected | -0.019 | -0.389 | 0.351 | 0.920 |
| Methanocorpusculaceae archaeon Phil4 | 4 | Not detected | 0.284 | 0.021 | 0.546 | 0.034 |
| Methanosarcina mazei | 0 | Not detected | -0.042 | -0.269 | 0.184 | 0.713 |
| Methylobacterium sp. ZNC0032 | 128 | Not detected | -0.210 | -0.367 | -0.054 | 0.009 |

| Methyloglobulus morosus | 4 | Not detected | 0.009 | -0.181 | 0.198 | 0.930 |
| --- | --- | --- | --- | --- | --- | --- |
| Microcystis aeruginosa | 4 | Not detected | 0.071 | -0.125 | 0.267 | 0.479 |
| Mitsuokella multacida | 0 | Not detected | 0.078 | -0.264 | 0.420 | 0.654 |
| Mobilibacterium timonense | 4 | Not detected | 0.035 | -0.148 | 0.218 | 0.710 |
| Mobiluncus curtisii | 16 | Not detected | -0.009 | -0.083 | 0.065 | 0.811 |
| Mobiluncus mulieris | 31 | Not detected | -0.004 | -0.087 | 0.080 | 0.930 |
| Mogibacterium timidum | 4 | Not detected | 0.051 | -0.163 | 0.266 | 0.640 |
| Moraxella porci | 5 | Not detected | 0.046 | -0.112 | 0.203 | 0.570 |
| Morganella morganii | 0 | Not detected | -0.106 | -0.315 | 0.103 | 0.321 |
| Morococcus cerebrosus | 5 | Not detected | 0.076 | -0.098 | 0.250 | 0.390 |
| Mucinivorans hirudinis | 17 | Not detected | -0.120 | -0.271 | 0.030 | 0.118 |
| Murdochiella sp. Marseille-P2341 | 8 | Not detected | -0.013 | -0.113 | 0.087 | 0.800 |
| Mycoplasma sp. CAG:877 | 4 | Not detected | 0.262 | -0.009 | 0.533 | 0.059 |
| Mycoplasma sp. CAG:956 | 4 | Not detected | 0.167 | -0.074 | 0.408 | 0.175 |
| Myroides injenensis | 419 | Not detected | -0.322 | -0.550 | -0.094 | 0.006 |
| Myroides odoratimimus | 19 | Not detected | -0.257 | -0.491 | -0.023 | 0.032 |
| Necropsobacter massiliensis | 4 | Not detected | 0.066 | -0.150 | 0.283 | 0.550 |
| Negativicoccus massiliensis | 5 | Not detected | 0.095 | -0.071 | 0.261 | 0.262 |
| Neglecta timonensis | 16 | Not detected | -0.082 | -0.195 | 0.031 | 0.155 |
| Neisseria cinerea | 12 | Not detected | -0.110 | -0.293 | 0.074 | 0.242 |
| Neisseria mucosa | 4 | Not detected | 0.126 | -0.066 | 0.318 | 0.198 |
| Nissabacter archeti | 4 | Not detected | 0.076 | -0.142 | 0.293 | 0.496 |
| Novibacillus thermophilus | 11 | Not detected | -0.071 | -0.264 | 0.121 | 0.469 |
| Obesumbacterium proteus | 0 | Not detected | -0.036 | -0.290 | 0.219 | 0.783 |
| Oceanospirillum linum | 4 | Not detected | 0.053 | -0.131 | 0.237 | 0.572 |
| Odoribacter laneus | 440 | Not detected | -0.191 | -0.359 | -0.024 | 0.025 |
| Odoribacter laneus CAG:561 | 5 | Not detected | -0.191 | -0.418 | 0.036 | 0.098 |
| Odoribacter sp. 43_10 | 2 | Not detected | 0.030 | -0.107 | 0.166 | 0.672 |
| Odoribacter sp. CAG:788 | 1 | Not detected | 0.008 | -0.218 | 0.233 | 0.947 |
| Odoribacter splanchnicus | 3 | Not detected | -0.027 | -0.128 | 0.074 | 0.602 |
| Odoribacter splanchnicus CAG:14 | 2 | Not detected | 0.053 | -0.098 | 0.204 | 0.492 |
| Oligella ureolytica | 4 | Not detected | 0.085 | -0.098 | 0.268 | 0.363 |
| Olsenella scatoligenes | 342 | Not detected | 0.313 | 0.110 | 0.517 | 0.003 |
| Olsenella sp. Marseille-P3197 | 4 | Not detected | 0.104 | -0.093 | 0.300 | 0.301 |
| Olsenella sp. oral taxon 809 | 10 | Not detected | 0.113 | -0.024 | 0.249 | 0.107 |
| Olsenella uli | 46 | Not detected | 0.168 | -0.030 | 0.366 | 0.097 |
| Olsenella umbonata | 5 | Not detected | 0.064 | -0.097 | 0.225 | 0.437 |
| Oribacterium parvum | 45 | Not detected | 0.047 | -0.017 | 0.110 | 0.153 |
| Oribacterium sinus | 10 | Not detected | -0.011 | -0.159 | 0.136 | 0.881 |
| Oribacterium sp. WCC10 | 4 | Not detected | 0.157 | -0.046 | 0.360 | 0.130 |
| Oribacterium sp. oral taxon 078 | 28 | Not detected | 0.052 | -0.048 | 0.153 | 0.307 |
| Oscillibacter ruminantium | 6 | Not detected | 0.038 | -0.100 | 0.177 | 0.587 |
| Oscillibacter sp. 1-3 | 10 | Not detected | 0.006 | -0.070 | 0.082 | 0.880 |
| Oscillibacter sp. 57_20 | 3 | Not detected | 0.255 | 0.009 | 0.501 | 0.042 |

| Oscillibacter sp. ER4 | 7 | Not detected | 0.139 | -0.007 | 0.285 | 0.062 |
| --- | --- | --- | --- | --- | --- | --- |
| Oscillibacter sp. KLE 1745 | 1 | Not detected | -0.040 | -0.166 | 0.086 | 0.533 |
| Oscillibacter valericigenes | 9 | Not detected | 0.067 | -0.061 | 0.195 | 0.307 |
| Oscillospiraceae bacterium Zagget12 | 2 | Not detected | 0.000 | -0.152 | 0.153 | 0.996 |
| Oxalobacter formigenes | 3 | Not detected | 0.073 | -0.086 | 0.233 | 0.367 |
| Paeniclostridium sordellii | 2 | Not detected | 0.003 | -0.213 | 0.219 | 0.977 |
| Paludibacter propionicigenes | 10 | Not detected | -0.015 | -0.160 | 0.130 | 0.836 |
| Pantoea agglomerans | 3 | Not detected | -0.024 | -0.205 | 0.158 | 0.797 |
| Pantoea ananatis | 5 | Not detected | -0.024 | -0.190 | 0.141 | 0.774 |
| Pantoea dispersa | 5 | Not detected | -0.006 | -0.165 | 0.153 | 0.942 |
| Pantoea rwandensis | 5 | Not detected | -0.038 | -0.212 | 0.136 | 0.669 |
| Pantoea septica | 5 | Not detected | 0.017 | -0.136 | 0.170 | 0.828 |
| Pantoea sp. IMH | 4 | Not detected | 0.053 | -0.125 | 0.232 | 0.558 |
| Pantoea sp. PSNIH2 | 0 | Not detected | 0.018 | -0.252 | 0.287 | 0.896 |
| Parabacteroides distasonis | 0 | Not detected | -0.107 | -0.249 | 0.036 | 0.142 |
| Parabacteroides goldsteinii | 0 | Not detected | 0.015 | -0.145 | 0.174 | 0.858 |
| Parabacteroides gordonii | 18 | Not detected | -0.143 | -0.270 | -0.017 | 0.027 |
| Parabacteroides johnsonii | 0 | Not detected | -0.086 | -0.199 | 0.028 | 0.138 |
| Parabacteroides merdae CAG:48 | 0 | Not detected | -0.054 | -0.261 | 0.154 | 0.612 |
| Paraburkholderia sacchari | 29 | Not detected | -0.192 | -0.367 | -0.017 | 0.032 |
| Paraclostridium bifermentans | 5 | Not detected | 0.088 | -0.106 | 0.283 | 0.373 |
| Paraprevotella clara CAG:116 | 0 | Not detected | -0.019 | -0.277 | 0.238 | 0.882 |
| Paraprevotella xylaniphila | 2 | Not detected | 0.058 | -0.081 | 0.196 | 0.414 |
| Parascardovia denticolens | 7 | Not detected | -0.083 | -0.285 | 0.120 | 0.425 |
| Parasporobacterium paucivorans | 78 | Not detected | 0.040 | -0.075 | 0.154 | 0.500 |
| Parasutterella excrementihominis | 0 | Not detected | -0.032 | -0.283 | 0.219 | 0.803 |
| Parasutterella excrementihominis CAG:233 | 0 | Not detected | -0.071 | -0.456 | 0.314 | 0.716 |
| Parvimonas micra | 329 | Not detected | -0.270 | -0.493 | -0.048 | 0.018 |
| Pasteurella bettyae | 53 | Not detected | 0.194 | 0.012 | 0.375 | 0.037 |
| Pectobacterium carotovorum | 4 | Not detected | -0.001 | -0.168 | 0.167 | 0.995 |
| Pedobacter glucosidilyticus | 4 | Not detected | 0.039 | -0.135 | 0.213 | 0.659 |
| Pelosinus fermentans | 5 | Not detected | 0.116 | -0.047 | 0.279 | 0.163 |
| Peptoanaerobacter stomatis | 8 | Not detected | 0.111 | -0.059 | 0.281 | 0.201 |
| Peptococcus niger | 31 | Not detected | -0.123 | -0.282 | 0.035 | 0.128 |
| Peptoniphilus duerdenii | 30 | Not detected | -0.113 | -0.248 | 0.022 | 0.102 |
| Peptoniphilus grossensis | 645 | Not detected | -0.272 | -0.448 | -0.096 | 0.002 |
| Peptoniphilus harei | 170 | Not detected | -0.156 | -0.358 | 0.047 | 0.132 |
| Peptoniphilus lacrimalis | 25 | Not detected | -0.104 | -0.321 | 0.113 | 0.348 |
| Peptoniphilus obesi | 198 | Not detected | -0.162 | -0.351 | 0.026 | 0.092 |
| Peptoniphilus rhinitidis | 4 | Not detected | 0.007 | -0.182 | 0.196 | 0.943 |
| Peptoniphilus senegalensis | 5 | Not detected | -0.020 | -0.222 | 0.183 | 0.849 |
| Peptoniphilus sp. BV3C26 | 11 | Not detected | -0.107 | -0.327 | 0.113 | 0.341 |
| Peptoniphilus sp. ING2-D1G | 16 | Not detected | -0.139 | -0.327 | 0.048 | 0.146 |
| Peptoniphilus sp. oral taxon 375 | 13 | Not detected | -0.010 | -0.101 | 0.081 | 0.831 |

| Peptostreptococcaceae bacterium VA2 | 4 | Not detected | 0.134 | -0.097 | 0.366 | 0.255 |
| --- | --- | --- | --- | --- | --- | --- |
| Peptostreptococcus anaerobius | 12 | Not detected | -0.004 | -0.073 | 0.066 | 0.920 |
| Peptostreptococcus anaerobius CAG:621 | 9 | Not detected | -0.058 | -0.218 | 0.103 | 0.481 |
| Peptostreptococcus stomatis | 122 | Not detected | -0.214 | -0.412 | -0.017 | 0.034 |
| Petrimonas mucosa | 4 | Not detected | 0.050 | -0.137 | 0.238 | 0.600 |
| Phaeodactylibacter xiamenensis | 12 | Not detected | -0.256 | -0.500 | -0.013 | 0.039 |
| Phascolarctobacterium sp. CAG:207 | 0 | Not detected | -0.205 | -0.571 | 0.161 | 0.272 |
| Phascolarctobacterium sp. CAG:266 | 0 | Not detected | -0.065 | -0.323 | 0.194 | 0.623 |
| Phascolarctobacterium succinatutens | 3 | Not detected | 0.150 | -0.038 | 0.338 | 0.117 |
| Phocaeicola abscessus | 112 | Not detected | -0.177 | -0.331 | -0.023 | 0.025 |
| Phocea massiliensis | 5 | Not detected | -0.006 | -0.104 | 0.092 | 0.906 |
| Piscirickettsia salmonis | 5 | Not detected | -0.023 | -0.187 | 0.140 | 0.782 |
| Plautia stali symbiont | 4 | Not detected | 0.030 | -0.136 | 0.197 | 0.719 |
| Pluralibacter gergoviae | 0 | Not detected | -0.041 | -0.299 | 0.216 | 0.752 |
| Pontibacillus litoralis | 420 | Not detected | -0.222 | -0.450 | 0.006 | 0.057 |
| Porphyromonadaceae bacterium COT-184 OH45 | 12 | Not detected | -0.115 | -0.284 | 0.054 | 0.184 |
| Porphyromonadaceae bacterium KH3CP3RA | 289 | Not detected | -0.225 | -0.394 | -0.056 | 0.009 |
| Porphyromonadaceae bacterium KH3R12 | 7 | Not detected | -0.007 | -0.167 | 0.153 | 0.930 |
| Porphyromonas asaccharolytica | 285 | Not detected | -0.213 | -0.420 | -0.005 | 0.045 |
| Porphyromonas bennonis | 22 | Not detected | -0.148 | -0.343 | 0.047 | 0.137 |
| Porphyromonas cangingivalis | 5 | Not detected | 0.047 | -0.137 | 0.231 | 0.617 |
| Porphyromonas canoris | 6 | Not detected | -0.017 | -0.200 | 0.165 | 0.851 |
| Porphyromonas catoniae | 8 | Not detected | -0.035 | -0.175 | 0.105 | 0.625 |
| Porphyromonas crevioricanis | 70 | Not detected | 0.105 | -0.017 | 0.227 | 0.092 |
| Porphyromonas endodontalis | 2 | Not detected | -0.089 | -0.294 | 0.115 | 0.393 |
| Porphyromonas gingivalis | 3 | Not detected | -0.005 | -0.173 | 0.164 | 0.957 |
| Porphyromonas gingivicanis | 17 | Not detected | -0.096 | -0.226 | 0.035 | 0.151 |
| Porphyromonas levii | 0 | Not detected | -0.095 | -0.334 | 0.144 | 0.437 |
| Porphyromonas macacae | 5 | Not detected | 0.008 | -0.172 | 0.188 | 0.934 |
| Porphyromonas somerae | 4 | Not detected | 0.011 | -0.075 | 0.097 | 0.804 |
| Porphyromonas sp. CAG:1061 | 9 | Not detected | -0.158 | -0.378 | 0.061 | 0.158 |
| Porphyromonas sp. COT-239 OH1446 | 3 | Not detected | 0.055 | -0.165 | 0.276 | 0.624 |
| Porphyromonas sp. COT-290 OH3588 | 0 | Not detected | 0.064 | -0.159 | 0.287 | 0.575 |
| Prevotella albensis | 3 | Not detected | 0.380 | 0.067 | 0.693 | 0.018 |
| Prevotella amnii | 4 | Not detected | 0.004 | -0.077 | 0.085 | 0.930 |
| Prevotella aurantiaca | 0 | Not detected | 0.090 | -0.111 | 0.292 | 0.378 |
| Prevotella baroniae | 4 | Not detected | -0.010 | -0.105 | 0.085 | 0.833 |
| Prevotella bergensis | 4 | Not detected | 0.099 | -0.055 | 0.253 | 0.207 |
| Prevotella bivia | 4 | Not detected | 0.060 | -0.046 | 0.167 | 0.267 |
| Prevotella bryantii | 0 | Not detected | 0.190 | -0.122 | 0.503 | 0.233 |
| Prevotella buccae | 4 | Not detected | 0.269 | 0.032 | 0.506 | 0.026 |
| Prevotella buccalis | 4 | Not detected | 0.027 | -0.060 | 0.114 | 0.549 |
| Prevotella copri | 0 | Not detected | 0.218 | -0.116 | 0.551 | 0.202 |
| Prevotella corporis | 15 | Not detected | -0.067 | -0.150 | 0.015 | 0.108 |

| Prevotella dentalis | 4 | Not detected | 0.315 | 0.071 | 0.560 | 0.012 |
| --- | --- | --- | --- | --- | --- | --- |
| Prevotella dentasini | 34 | Not detected | 0.265 | 0.017 | 0.512 | 0.036 |
| Prevotella denticola | 0 | Not detected | 0.145 | -0.126 | 0.417 | 0.294 |
| Prevotella disiens | 1 | Not detected | -0.010 | -0.103 | 0.084 | 0.841 |
| Prevotella enoeca | 3 | Not detected | 0.188 | -0.047 | 0.424 | 0.117 |
| Prevotella fusca | 4 | Not detected | 0.249 | -0.019 | 0.517 | 0.068 |
| Prevotella histicola | 0 | Not detected | -0.037 | -0.200 | 0.127 | 0.662 |
| Prevotella intermedia | 0 | Not detected | 0.042 | -0.125 | 0.209 | 0.622 |
| Prevotella loescheii | 4 | Not detected | 0.155 | -0.072 | 0.382 | 0.181 |
| Prevotella maculosa | 0 | Not detected | 0.035 | -0.158 | 0.227 | 0.725 |
| Prevotella marshii | 9 | Not detected | -0.059 | -0.150 | 0.031 | 0.200 |
| Prevotella melaninogenica | 2 | Not detected | 0.134 | -0.061 | 0.329 | 0.178 |
| Prevotella micans | 4 | Not detected | 0.154 | -0.071 | 0.380 | 0.180 |
| Prevotella multiformis | 5 | Not detected | 0.121 | -0.018 | 0.261 | 0.087 |
| Prevotella multisaccharivorax | 0 | Not detected | 0.274 | -0.034 | 0.582 | 0.082 |
| Prevotella nanceiensis | 0 | Not detected | -0.074 | -0.251 | 0.104 | 0.415 |
| Prevotella nigrescens | 4 | Not detected | 0.223 | -0.001 | 0.447 | 0.051 |
| Prevotella oralis | 4 | Not detected | 0.149 | -0.087 | 0.385 | 0.217 |
| Prevotella oris | 0 | Not detected | 0.037 | -0.226 | 0.300 | 0.783 |
| Prevotella oryzae | 4 | Not detected | 0.334 | 0.060 | 0.609 | 0.017 |
| Prevotella oulorum | 0 | Not detected | 0.259 | -0.025 | 0.544 | 0.074 |
| Prevotella pallens | 1 | Not detected | 0.007 | -0.095 | 0.109 | 0.889 |
| Prevotella paludivivens | 4 | Not detected | 0.142 | -0.048 | 0.332 | 0.144 |
| Prevotella ruminicola | 4 | Not detected | 0.225 | -0.038 | 0.488 | 0.094 |
| Prevotella saccharolytica | 0 | Not detected | 0.149 | -0.107 | 0.404 | 0.254 |
| Prevotella salivae | 1 | Not detected | -0.003 | -0.098 | 0.092 | 0.954 |
| Prevotella scopos | 0 | Not detected | -0.017 | -0.148 | 0.114 | 0.803 |
| Prevotella sp. 10(H) | 17 | Not detected | 0.103 | -0.061 | 0.266 | 0.219 |
| Prevotella sp. 109 | 3 | Not detected | -0.034 | -0.126 | 0.058 | 0.471 |
| Prevotella sp. AGR2160 | 0 | Not detected | 0.285 | -0.009 | 0.579 | 0.057 |
| Prevotella sp. BP1-148 | 5 | Not detected | 0.053 | -0.086 | 0.192 | 0.453 |
| Prevotella sp. CAG:1058 | 5 | Not detected | -0.208 | -0.455 | 0.039 | 0.100 |
| Prevotella sp. CAG:1092 | 0 | Not detected | 0.208 | -0.105 | 0.520 | 0.193 |
| Prevotella sp. CAG:1124 | 5 | Not detected | -0.263 | -0.513 | -0.013 | 0.039 |
| Prevotella sp. CAG:1185 | 0 | Not detected | 0.148 | -0.112 | 0.408 | 0.265 |
| Prevotella sp. CAG:1320 | 5 | Not detected | -0.157 | -0.355 | 0.040 | 0.119 |
| Prevotella sp. CAG:255 | 0 | Not detected | 0.055 | -0.105 | 0.216 | 0.500 |
| Prevotella sp. CAG:386 | 0 | Not detected | 0.248 | -0.099 | 0.596 | 0.161 |
| Prevotella sp. CAG:487 | 2 | Not detected | 0.008 | -0.156 | 0.172 | 0.923 |
| Prevotella sp. CAG:520 | 0 | Not detected | 0.228 | -0.053 | 0.509 | 0.111 |
| Prevotella sp. CAG:5226 | 793 | 0.6 | 0.372 | 0.060 | 0.683 | 0.019 |
| Prevotella sp. CAG:604 | 0 | Not detected | 0.326 | -0.045 | 0.698 | 0.085 |
| Prevotella sp. CAG:732 | 0 | Not detected | 0.370 | 0.038 | 0.702 | 0.029 |
| Prevotella sp. CAG:891 | 0 | Not detected | 0.141 | -0.095 | 0.377 | 0.241 |

| Prevotella sp. FD3004 | 6 | Not detected | 0.249 | 0.037 | 0.460 | 0.021 |
| --- | --- | --- | --- | --- | --- | --- |
| Prevotella sp. HUN102 | 4 | Not detected | 0.039 | -0.099 | 0.177 | 0.583 |
| Prevotella sp. MSX73 | 1 | Not detected | 0.165 | -0.074 | 0.405 | 0.177 |
| Prevotella sp. P4-76 | 3 | Not detected | 0.146 | -0.029 | 0.321 | 0.103 |
| Prevotella sp. P6B1 | 67 | Not detected | 0.207 | 0.061 | 0.354 | 0.006 |
| Prevotella sp. P6B4 | 8 | Not detected | 0.147 | 0.004 | 0.291 | 0.044 |
| Prevotella sp. RM4 | 4 | Not detected | 0.132 | -0.048 | 0.313 | 0.151 |
| Prevotella sp. S7 MS 2 | 3 | Not detected | 0.010 | -0.079 | 0.100 | 0.818 |
| Prevotella sp. S7-1-8 | 4 | Not detected | 0.022 | -0.061 | 0.106 | 0.602 |
| Prevotella sp. kh1p2 | 3 | Not detected | 0.190 | -0.049 | 0.429 | 0.120 |
| Prevotella sp. khp1 | 4 | Not detected | 0.159 | -0.026 | 0.344 | 0.093 |
| Prevotella sp. ne3005 | 5 | Not detected | 0.099 | -0.065 | 0.263 | 0.236 |
| Prevotella sp. oral taxon 473 | 4 | Not detected | 0.168 | -0.073 | 0.410 | 0.171 |
| Prevotella sp. tf2-5 | 9 | Not detected | 0.156 | -0.006 | 0.318 | 0.059 |
| Prevotella stercorea | 1 | Not detected | 0.049 | -0.082 | 0.180 | 0.461 |
| Prevotella stercorea CAG:629 | 0 | Not detected | 0.269 | -0.024 | 0.563 | 0.073 |
| Prevotella timonensis | 3 | Not detected | 0.003 | -0.087 | 0.094 | 0.940 |
| Prevotellamassilia timonensis | 247 | Not detected | 0.187 | 0.034 | 0.341 | 0.017 |
| Propionibacterium acidifaciens | 1 | Not detected | -0.060 | -0.256 | 0.136 | 0.550 |
| Proteiniclasticum ruminis | 18 | Not detected | -0.154 | -0.321 | 0.013 | 0.070 |
| Proteiniphilum acetatigenes | 166 | Not detected | -0.165 | -0.339 | 0.008 | 0.061 |
| Proteiniphilum saccharofermentans | 14 | Not detected | -0.070 | -0.260 | 0.120 | 0.468 |
| Proteiniphilum sp. EBM-41 | 0 | Not detected | -0.163 | -0.395 | 0.068 | 0.167 |
| Proteobacteria bacterium CAG:139 | 0 | Not detected | -0.094 | -0.465 | 0.276 | 0.617 |
| Proteobacteria bacterium CAG:495 | 0 | Not detected | -0.045 | -0.363 | 0.273 | 0.782 |
| Providencia rettgeri | 1 | Not detected | 0.040 | -0.165 | 0.245 | 0.703 |
| Pseudobacteroides cellulosolvens | 9 | Not detected | 0.047 | -0.103 | 0.198 | 0.539 |
| Pseudobutyrivibrio ruminis | 52 | Not detected | 0.064 | -0.012 | 0.140 | 0.100 |
| Pseudobutyrivibrio sp. ACV-2 | 318 | Not detected | 0.270 | 0.095 | 0.444 | 0.002 |
| Pseudobutyrivibrio sp. JW11 | 50 | Not detected | 0.123 | -0.047 | 0.292 | 0.155 |
| Pseudobutyrivibrio sp. MD2005 | 7 | Not detected | 0.003 | -0.179 | 0.185 | 0.976 |
| Pseudobutyrivibrio sp. UC1225 | 375 | Not detected | 0.138 | 0.025 | 0.252 | 0.017 |
| Pseudobutyrivibrio sp. YE44 | 10 | Not detected | 0.045 | -0.126 | 0.215 | 0.608 |
| Pseudobutyrivibrio xylanivorans | 21 | Not detected | 0.134 | -0.040 | 0.307 | 0.132 |
| Pseudoflavonifractor capillosus | 11 | Not detected | -0.012 | -0.093 | 0.069 | 0.771 |
| Pseudopropionibacterium propionicum | 8 | Not detected | -0.116 | -0.314 | 0.083 | 0.253 |
| Pyramidobacter piscolens | 3 | Not detected | 0.147 | -0.108 | 0.402 | 0.260 |
| Rahnella aquatilis | 4 | Not detected | 0.026 | -0.139 | 0.192 | 0.754 |
| Ralstonia mannitolilytica | 1 | Not detected | -0.171 | -0.396 | 0.055 | 0.138 |
| Ralstonia pickettii | 5 | Not detected | 0.029 | -0.184 | 0.242 | 0.788 |
| Raoultella ornithinolytica | 0 | Not detected | 0.138 | -0.173 | 0.448 | 0.385 |
| Rappaport israeli | 4 | Not detected | 0.016 | -0.159 | 0.192 | 0.856 |
| Rhodococcus defluvii | 17 | Not detected | 0.084 | -0.080 | 0.248 | 0.317 |
| Rhodopseudomonas palustris | 4 | Not detected | 0.051 | -0.140 | 0.243 | 0.599 |

| Rhodospirillum sp. UNK.MGS-17 | 0 | Not detected | -0.041 | -0.399 | 0.317 | 0.822 |
| --- | --- | --- | --- | --- | --- | --- |
| Riemerella columbina | 15 | Not detected | -0.075 | -0.158 | 0.007 | 0.074 |
| Rikenella microfusus | 16 | Not detected | -0.069 | -0.153 | 0.015 | 0.105 |
| Risungbinella massiliensis | 11 | Not detected | 0.009 | -0.139 | 0.157 | 0.905 |
| Robinsoniella peoriensis | 94 | Not detected | 0.054 | -0.009 | 0.117 | 0.092 |
| Rodentibacter pneumotropicus | 4 | Not detected | 0.091 | -0.067 | 0.249 | 0.259 |
| Roseburia hominis | 6 | Not detected | 0.028 | -0.060 | 0.116 | 0.537 |
| Roseburia intestinalis | 141 | Not detected | 0.143 | 0.010 | 0.277 | 0.036 |
| Roseburia inulinivorans | 695 | Not detected | 0.177 | 0.068 | 0.285 | 0.001 |
| Roseburia sp. CAG:197_41_10 | 353 | Not detected | 0.160 | 0.050 | 0.270 | 0.004 |
| Rothia dentocariosa | 4 | Not detected | 0.082 | -0.158 | 0.321 | 0.503 |
| Rothia mucilaginosa | 0 | Not detected | 0.074 | -0.176 | 0.324 | 0.562 |
| Ruminococcaceae bacterium AB4001 | 5 | Not detected | 0.030 | -0.125 | 0.185 | 0.705 |
| Ruminococcaceae bacterium AE2021 | 7 | Not detected | 0.035 | -0.116 | 0.187 | 0.648 |
| Ruminococcaceae bacterium D16 | 12 | Not detected | -0.001 | -0.075 | 0.074 | 0.986 |
| Ruminococcaceae bacterium YRB3002 | 5 | Not detected | 0.065 | -0.117 | 0.248 | 0.483 |
| Ruminococcus albus | 11 | Not detected | 0.083 | -0.065 | 0.231 | 0.271 |
| Ruminococcus bicirculans | 0 | Not detected | 0.091 | -0.134 | 0.317 | 0.428 |
| Ruminococcus callidus | 4 | Not detected | 0.140 | -0.014 | 0.294 | 0.075 |
| Ruminococcus champanellensis | 5 | Not detected | 0.201 | 0.009 | 0.393 | 0.040 |
| Ruminococcus faecis | 5 | Not detected | 0.007 | -0.093 | 0.108 | 0.888 |
| Ruminococcus flavefaciens | 43 | Not detected | 0.125 | -0.012 | 0.262 | 0.075 |
| Ruminococcus gauvreauii | 36 | Not detected | 0.047 | -0.034 | 0.128 | 0.259 |
| Ruminococcus lactaris | 7 | Not detected | -0.053 | -0.168 | 0.062 | 0.367 |
| Ruminococcus obeum CAG:39 | 6 | Not detected | -0.053 | -0.184 | 0.078 | 0.427 |
| Ruminococcus sp. AT10 | 502 | Not detected | -0.130 | -0.205 | -0.056 | 0.001 |
| Ruminococcus sp. CAG:108-related_41_35 | 0 | Not detected | -0.046 | -0.287 | 0.196 | 0.711 |
| Ruminococcus sp. CAG:177 | 0 | Not detected | 0.379 | 0.005 | 0.754 | 0.047 |
| Ruminococcus sp. CAG:330 | 3 | Not detected | 0.338 | -0.003 | 0.678 | 0.052 |
| Ruminococcus sp. CAG:379 | 450 | Not detected | 0.291 | 0.033 | 0.549 | 0.027 |
| Ruminococcus sp. CAG:382 | 2 | Not detected | 0.256 | -0.079 | 0.591 | 0.134 |
| Ruminococcus sp. CAG:403 | 3 | Not detected | 0.229 | -0.039 | 0.498 | 0.094 |
| Ruminococcus sp. CAG:488 | 4 | Not detected | 0.180 | -0.050 | 0.410 | 0.125 |
| Ruminococcus sp. CAG:563 | 578 | Not detected | 0.403 | 0.148 | 0.658 | 0.002 |
| Ruminococcus sp. CAG:60 | 4 | Not detected | 0.090 | -0.052 | 0.233 | 0.213 |
| Ruminococcus sp. CAG:624 | 1 | Not detected | 0.099 | -0.152 | 0.350 | 0.438 |
| Ruminococcus sp. CAG:724 | 0 | Not detected | 0.154 | -0.128 | 0.437 | 0.285 |
| Ruminococcus sp. FC2018 | 7 | Not detected | 0.045 | -0.116 | 0.205 | 0.587 |
| Ruminococcus sp. HUN007 | 15 | Not detected | 0.040 | -0.092 | 0.172 | 0.554 |
| Ruminococcus sp. NK3A76 | 8 | Not detected | 0.084 | -0.078 | 0.247 | 0.309 |
| Ruminococcus sp. Phil15 | 0 | Not detected | -0.030 | -0.247 | 0.187 | 0.788 |
| Ruminococcus sp. Zagget7 | 1 | Not detected | -0.087 | -0.211 | 0.036 | 0.166 |
| Ruthenibacterium lactatiformans | 334 | Not detected | -0.140 | -0.247 | -0.033 | 0.011 |
| Salmonella enterica | 0 | Not detected | -0.055 | -0.325 | 0.215 | 0.690 |

| Sanguibacteroides justesenii | 596 | Not detected | -0.234 | -0.404 | -0.064 | 0.007 |
| --- | --- | --- | --- | --- | --- | --- |
| Scardovia inopinata | 11 | Not detected | -0.098 | -0.262 | 0.065 | 0.239 |
| Scardovia wiggsiae | 1 | Not detected | -0.042 | -0.258 | 0.173 | 0.702 |
| Selenomonas bovis | 4 | Not detected | 0.011 | -0.128 | 0.150 | 0.874 |
| Selenomonas noxia | 5 | Not detected | 0.021 | -0.145 | 0.186 | 0.806 |
| Selenomonas ruminantium | 4 | Not detected | 0.167 | -0.035 | 0.370 | 0.106 |
| Selenomonas sputigena | 4 | Not detected | 0.065 | -0.107 | 0.238 | 0.458 |
| Sellimonas intestinalis | 5 | Not detected | -0.052 | -0.157 | 0.054 | 0.339 |
| Senegalimassilia anaerobia | 103 | Not detected | 0.212 | 0.017 | 0.407 | 0.033 |
| Serratia fonticola | 4 | Not detected | 0.080 | -0.078 | 0.239 | 0.321 |
| Serratia liquefaciens | 4 | Not detected | 0.015 | -0.154 | 0.184 | 0.858 |
| Serratia marcescens | 4 | Not detected | 0.004 | -0.180 | 0.188 | 0.968 |
| Serratia odorifera | 4 | Not detected | 0.043 | -0.130 | 0.216 | 0.626 |
| Serratia rubidaea | 5 | Not detected | -0.065 | -0.244 | 0.114 | 0.474 |
| Shigella dysenteriae | 0 | Not detected | -0.080 | -0.352 | 0.192 | 0.563 |
| Shigella flexneri | 0 | Not detected | -0.088 | -0.360 | 0.184 | 0.527 |
| Shimwellia blattae | 0 | Not detected | -0.138 | -0.369 | 0.093 | 0.241 |
| Shuttleworthia satelles | 38 | Not detected | 0.075 | -0.027 | 0.178 | 0.148 |
| Siccibacter turicensis | 0 | Not detected | -0.001 | -0.246 | 0.244 | 0.993 |
| Slackia exigua | 5 | Not detected | 0.064 | -0.114 | 0.243 | 0.482 |
| Slackia heliotrinireducens | 15 | Not detected | 0.125 | -0.034 | 0.284 | 0.123 |
| Slackia piriformis | 0 | Not detected | -0.012 | -0.275 | 0.251 | 0.927 |
| Solobacterium moorei | 1 | Not detected | -0.009 | -0.116 | 0.099 | 0.870 |
| Sphaerochaeta sp. Phil3 | 5 | Not detected | 0.127 | -0.060 | 0.315 | 0.184 |
| Sphingobacterium wenxiniae | 476 | Not detected | -0.286 | -0.477 | -0.095 | 0.003 |
| Spirochaetales bacterium Spiro_07 | 73 | Not detected | 0.177 | 0.018 | 0.335 | 0.029 |
| Sporobacter termitidis | 6 | Not detected | 0.073 | -0.079 | 0.225 | 0.348 |
| Sporomusa ovata | 4 | Not detected | 0.089 | -0.089 | 0.267 | 0.327 |
| Staphylococcus pseudintermedius | 4 | Not detected | 0.094 | -0.091 | 0.278 | 0.319 |
| Staphylococcus sp. CAG:324 | 0 | Not detected | 0.027 | -0.309 | 0.362 | 0.876 |
| Staphylococcus sp. DORA_6_22 | 9 | Not detected | -0.124 | -0.304 | 0.055 | 0.175 |
| Staphylococcus warneri | 4 | Not detected | 0.013 | -0.186 | 0.212 | 0.896 |
| Stomatobaculum longum | 139 | Not detected | 0.063 | 0.003 | 0.122 | 0.040 |
| Streptobacillus hongkongensis | 5 | Not detected | -0.041 | -0.218 | 0.136 | 0.647 |
| Streptobacillus notomytis | 0 | Not detected | -0.167 | -0.364 | 0.031 | 0.099 |
| Streptococcus agalactiae | 2 | Not detected | 0.080 | -0.120 | 0.279 | 0.433 |
| Streptococcus anginosus | 21 | Not detected | -0.084 | -0.310 | 0.142 | 0.468 |
| Streptococcus caballi | 7 | Not detected | -0.106 | -0.314 | 0.102 | 0.320 |
| Streptococcus cristatus | 0 | Not detected | 0.012 | -0.166 | 0.190 | 0.894 |
| Streptococcus dysgalactiae | 2 | Not detected | -0.043 | -0.247 | 0.161 | 0.680 |
| Streptococcus entericus | 15 | Not detected | -0.190 | -0.353 | -0.026 | 0.023 |
| Streptococcus gallolyticus | 0 | Not detected | -0.036 | -0.283 | 0.210 | 0.773 |
| Streptococcus gordonii | 4 | Not detected | 0.186 | -0.050 | 0.422 | 0.123 |
| Streptococcus halotolerans | 11 | Not detected | -0.041 | -0.209 | 0.128 | 0.635 |

| Streptococcus henryi | 8 | Not detected | -0.116 | -0.317 | 0.084 | 0.257 |
| --- | --- | --- | --- | --- | --- | --- |
| Streptococcus ictaluri | 4 | Not detected | -0.029 | -0.207 | 0.148 | 0.747 |
| Streptococcus infantis | 0 | Not detected | 0.043 | -0.133 | 0.219 | 0.634 |
| Streptococcus iniae | 11 | Not detected | -0.092 | -0.273 | 0.089 | 0.320 |
| Streptococcus intermedius | 1 | Not detected | -0.092 | -0.321 | 0.137 | 0.432 |
| Streptococcus massiliensis | 10 | Not detected | -0.066 | -0.252 | 0.121 | 0.490 |
| Streptococcus minor | 11 | Not detected | -0.010 | -0.151 | 0.131 | 0.892 |
| Streptococcus mitis | 1 | Not detected | -0.104 | -0.237 | 0.029 | 0.126 |
| Streptococcus mutans | 0 | Not detected | 0.111 | -0.162 | 0.384 | 0.425 |
| Streptococcus oralis | 4 | Not detected | 0.107 | -0.103 | 0.316 | 0.318 |
| Streptococcus orisratti | 7 | Not detected | -0.134 | -0.332 | 0.063 | 0.182 |
| Streptococcus ovis | 5 | Not detected | -0.040 | -0.222 | 0.142 | 0.666 |
| Streptococcus parasanguinis | 3 | Not detected | 0.112 | -0.099 | 0.323 | 0.299 |
| Streptococcus parauberis | 8 | Not detected | -0.134 | -0.319 | 0.052 | 0.159 |
| Streptococcus phocae | 11 | Not detected | -0.052 | -0.193 | 0.089 | 0.469 |
| Streptococcus plurextorum | 13 | Not detected | -0.027 | -0.114 | 0.061 | 0.549 |
| Streptococcus porci | 11 | Not detected | -0.037 | -0.143 | 0.069 | 0.491 |
| Streptococcus pseudoporcinus | 8 | Not detected | -0.089 | -0.288 | 0.110 | 0.381 |
| Streptococcus pyogenes | 2 | Not detected | -0.037 | -0.239 | 0.166 | 0.722 |
| Streptococcus salivarius | 16 | Not detected | 0.212 | 0.016 | 0.408 | 0.034 |
| Streptococcus sanguinis | 4 | Not detected | 0.105 | -0.124 | 0.335 | 0.369 |
| Streptococcus sobrinus | 275 | Not detected | -0.191 | -0.405 | 0.024 | 0.082 |
| Streptococcus sp. X13SY08 | 5 | Not detected | -0.002 | -0.147 | 0.144 | 0.982 |
| Streptococcus suis | 15 | Not detected | -0.065 | -0.155 | 0.025 | 0.156 |
| Streptococcus thermophilus | 0 | Not detected | -0.086 | -0.293 | 0.122 | 0.419 |
| Streptococcus uberis | 9 | Not detected | -0.147 | -0.324 | 0.029 | 0.102 |
| Streptococcus urinalis | 2 | Not detected | -0.016 | -0.217 | 0.185 | 0.876 |
| Streptomyces albulus | 5 | Not detected | 0.049 | -0.115 | 0.213 | 0.560 |
| Streptomyces cattleya | 5 | Not detected | -0.009 | -0.196 | 0.179 | 0.929 |
| Streptomyces kanamyceticus | 5 | Not detected | 0.061 | -0.064 | 0.186 | 0.342 |
| Subdoligranulum sp. CAG:314 | 0 | Not detected | 0.094 | -0.292 | 0.480 | 0.633 |
| Subdoligranulum variabile | 12 | Not detected | -0.001 | -0.073 | 0.071 | 0.977 |
| Succinatimonas sp. CAG:777 | 0 | Not detected | 0.222 | -0.101 | 0.546 | 0.178 |
| Succinispira mobilis | 5 | Not detected | 0.001 | -0.187 | 0.189 | 0.993 |
| Succinivibrio sp. Phil9 | 0 | Not detected | 0.167 | -0.140 | 0.475 | 0.287 |
| Sulfolobus acidocaldarius | 235 | Not detected | -0.310 | -0.545 | -0.076 | 0.010 |
| Sutterella parvirubra | 622 | Not detected | 0.354 | 0.149 | 0.560 | 0.001 |
| Sutterella sp. CAG:397 | 0 | Not detected | 0.358 | -0.058 | 0.774 | 0.092 |
| Sutterella sp. CAG:521 | 0 | Not detected | 0.012 | -0.257 | 0.280 | 0.931 |
| Sutterella wadsworthensis | 764 | 0.6 | 0.421 | 0.093 | 0.750 | 0.012 |
| Sutterella wadsworthensis CAG:135 | 0 | Not detected | 0.382 | -0.032 | 0.796 | 0.071 |
| Sutterellaceae bacterium ND3 | 0 | Not detected | 0.000 | -0.252 | 0.251 | 0.999 |
| Synergistales bacterium Syner_02 | 1 | Not detected | -0.007 | -0.224 | 0.210 | 0.950 |
| Synergistes sp. 3_1_syn1 | 0 | Not detected | 0.019 | -0.257 | 0.296 | 0.891 |

| Synergistes sp. Zagget9 | 5 | Not detected | -0.125 | -0.297 | 0.046 | 0.152 |
| --- | --- | --- | --- | --- | --- | --- |
| Tannerella forsythia | 2 | Not detected | 0.002 | -0.126 | 0.129 | 0.979 |
| Tatumella morbirosei | 5 | Not detected | -0.009 | -0.174 | 0.157 | 0.917 |
| Tepidanaerobacter sp. EBM-49 | 0 | Not detected | 0.028 | -0.236 | 0.292 | 0.834 |
| Terrisporobacter glycolicus | 4 | Not detected | 0.074 | -0.153 | 0.300 | 0.524 |
| Thermophagus xiamenensis | 1 | Not detected | -0.123 | -0.324 | 0.077 | 0.227 |
| Thermosinus carboxydivorans | 5 | Not detected | 0.071 | -0.081 | 0.223 | 0.359 |
| Tidjanibacter massiliensis | 0 | Not detected | -0.151 | -0.416 | 0.113 | 0.263 |
| Tissierellia bacterium S7-1-4 | 131 | Not detected | -0.190 | -0.392 | 0.012 | 0.065 |
| Trabulsiella odontotermitis | 0 | Not detected | -0.023 | -0.277 | 0.232 | 0.862 |
| Traorella massiliensis | 14 | Not detected | 0.019 | -0.053 | 0.091 | 0.603 |
| Treponema denticola | 102 | Not detected | 0.090 | -0.056 | 0.237 | 0.227 |
| Treponema medium | 42 | Not detected | -0.251 | -0.497 | -0.006 | 0.045 |
| Treponema putidum | 102 | Not detected | 0.092 | -0.038 | 0.221 | 0.165 |
| Treponema socranskii | 25 | Not detected | 0.023 | -0.125 | 0.171 | 0.762 |
| Treponema succinifaciens | 8 | Not detected | 0.000 | -0.083 | 0.084 | 0.991 |
| Treponema vincentii | 28 | Not detected | 0.038 | -0.096 | 0.172 | 0.579 |
| Trichococcus flocculiformis | 74 | Not detected | 0.141 | -0.022 | 0.303 | 0.090 |
| Trueperella pyogenes | 4 | Not detected | 0.052 | -0.112 | 0.217 | 0.534 |
| Tumebacillus flagellatus | 5 | Not detected | -0.072 | -0.238 | 0.095 | 0.398 |
| Turicibacter sanguinis | 0 | Not detected | -0.038 | -0.332 | 0.256 | 0.800 |
| Turicibacter sp. H121 | 3 | Not detected | -0.035 | -0.150 | 0.079 | 0.543 |
| Tyzzerella nexilis | 197 | Not detected | -0.087 | -0.154 | -0.021 | 0.010 |
| Urmitella timonensis | 11 | Not detected | 0.012 | -0.110 | 0.134 | 0.848 |
| Vaginella massiliensis | 0 | Not detected | -0.163 | -0.406 | 0.080 | 0.189 |
| Varibaculum cambriense | 7 | Not detected | 0.012 | -0.087 | 0.112 | 0.809 |
| Varibaculum sp. Marseille-P2802 | 38 | Not detected | 0.101 | -0.059 | 0.261 | 0.215 |
| Veillonella magna | 18 | Not detected | 0.020 | -0.055 | 0.095 | 0.602 |
| Veillonella parvula | 0 | Not detected | 0.158 | -0.083 | 0.400 | 0.199 |
| Veillonella seminalis | 59 | Not detected | 0.088 | 0.007 | 0.169 | 0.033 |
| Veillonella sp. ACP1 | 0 | Not detected | 0.059 | -0.234 | 0.352 | 0.694 |
| Veillonella sp. CAG:933 | 3 | Not detected | 0.035 | -0.108 | 0.178 | 0.631 |
| Veillonella sp. oral taxon 780 | 5 | Not detected | -0.040 | -0.224 | 0.144 | 0.671 |
| Waddlia chondrophila | 507 | Not detected | -0.349 | -0.576 | -0.121 | 0.003 |
| Weissella cibaria | 4 | Not detected | 0.244 | 0.008 | 0.480 | 0.043 |
| Weissella confusa | 4 | Not detected | 0.202 | -0.066 | 0.469 | 0.139 |
| Xanthomonas sp. Mitacek01 | 7 | Not detected | 0.009 | -0.128 | 0.147 | 0.897 |
| Yersinia enterocolitica | 4 | Not detected | -0.072 | -0.294 | 0.150 | 0.525 |
| Yersinia pestis | 0 | Not detected | -0.104 | -0.382 | 0.174 | 0.463 |
| Yokenella regensburgei | 0 | Not detected | 0.017 | -0.234 | 0.269 | 0.893 |
| [Bacteroides] pectinophilus | 58 | Not detected | 0.074 | -0.003 | 0.151 | 0.060 |
| [Clostridium] aminophilum | 56 | Not detected | 0.062 | -0.007 | 0.132 | 0.079 |
| [Clostridium] asparagiforme | 8 | Not detected | -0.066 | -0.168 | 0.036 | 0.202 |
| [Clostridium] bolteae | 361 | Not detected | -0.136 | -0.230 | -0.042 | 0.005 |

| [Clostridium] celerecrescens | 18 | Not detected | -0.024 | -0.088 | 0.041 | 0.476 |
| --- | --- | --- | --- | --- | --- | --- |
| [Clostridium] cellobioparum | 11 | Not detected | -0.025 | -0.176 | 0.127 | 0.749 |
| [Clostridium] cellulolyticum | 10 | Not detected | -0.094 | -0.263 | 0.074 | 0.273 |
| [Clostridium] cellulosi | 9 | Not detected | -0.030 | -0.198 | 0.137 | 0.724 |
| [Clostridium] citroniae | 406 | Not detected | -0.113 | -0.190 | -0.036 | 0.004 |
| [Clostridium] clariflavum | 41 | Not detected | 0.133 | -0.015 | 0.280 | 0.078 |
| [Clostridium] clostridioforme | 528 | Not detected | -0.142 | -0.240 | -0.044 | 0.005 |
| [Clostridium] dakarense | 4 | Not detected | 0.061 | -0.167 | 0.288 | 0.601 |
| [Clostridium] glycyrrhizinilyticum | 61 | Not detected | -0.058 | -0.124 | 0.008 | 0.086 |
| [Clostridium] hiranonis | 178 | Not detected | -0.238 | -0.440 | -0.036 | 0.021 |
| [Clostridium] hylemonae | 94 | Not detected | 0.094 | -0.050 | 0.238 | 0.203 |
| [Clostridium] innocuum | 211 | Not detected | -0.092 | -0.163 | -0.022 | 0.011 |
| [Clostridium] josui | 8 | Not detected | -0.128 | -0.300 | 0.044 | 0.145 |
| [Clostridium] lactatifermentans | 184 | Not detected | -0.100 | -0.187 | -0.014 | 0.023 |
| [Clostridium] leptum | 8 | Not detected | -0.055 | -0.166 | 0.056 | 0.331 |
| [Clostridium] methylpentosum | 37 | Not detected | 0.085 | -0.029 | 0.199 | 0.144 |
| [Clostridium] papyrosolvens | 5 | Not detected | 0.014 | -0.117 | 0.146 | 0.831 |
| [Clostridium] saccharogumia | 5 | Not detected | -0.022 | -0.124 | 0.080 | 0.672 |
| [Clostridium] saccharolyticum | 108 | Not detected | 0.099 | 0.007 | 0.191 | 0.036 |
| [Clostridium] scindens | 11 | Not detected | -0.041 | -0.122 | 0.040 | 0.318 |
| [Clostridium] sporosphaeroides | 107 | Not detected | 0.069 | -0.080 | 0.218 | 0.364 |
| [Clostridium] straminisolvens | 10 | Not detected | 0.055 | -0.070 | 0.179 | 0.389 |
| [Clostridium] symbiosum | 20 | Not detected | -0.059 | -0.141 | 0.024 | 0.163 |
| [Clostridium] viride | 7 | Not detected | 0.023 | -0.118 | 0.164 | 0.751 |
| [Enterobacter] aerogenes | 0 | Not detected | 0.106 | -0.214 | 0.426 | 0.517 |
| [Eubacterium] brachy | 2 | Not detected | -0.062 | -0.282 | 0.157 | 0.578 |
| [Eubacterium] cellulosolvens | 147 | Not detected | 0.114 | 0.021 | 0.207 | 0.016 |
| [Eubacterium] contortum | 25 | Not detected | -0.048 | -0.110 | 0.014 | 0.130 |
| [Eubacterium] dolichum | 27 | Not detected | -0.051 | -0.114 | 0.013 | 0.117 |
| [Eubacterium] eligens | 805 | 0.6 | 0.232 | 0.071 | 0.393 | 0.005 |
| [Eubacterium] fissicatena | 59 | Not detected | -0.099 | -0.184 | -0.013 | 0.024 |
| [Eubacterium] hallii | 4 | Not detected | 0.041 | -0.066 | 0.149 | 0.452 |
| [Eubacterium] nodatum | 5 | Not detected | -0.061 | -0.260 | 0.137 | 0.545 |
| [Eubacterium] rectale | 1 | Not detected | 0.042 | -0.114 | 0.197 | 0.600 |
| [Eubacterium] siraeum | 0 | Not detected | -0.016 | -0.264 | 0.231 | 0.896 |
| [Eubacterium] sulci | 5 | Not detected | -0.072 | -0.287 | 0.143 | 0.512 |
| [Eubacterium] yurii | 7 | Not detected | 0.014 | -0.115 | 0.142 | 0.834 |
| [Haemophilus] ducreyi | 5 | Not detected | 0.033 | -0.125 | 0.191 | 0.685 |
| [Haemophilus] parasuis | 25 | Not detected | 0.199 | 0.009 | 0.390 | 0.040 |
| [Ruminococcus] gnavus | 755 | 0.6 | -0.197 | -0.321 | -0.073 | 0.002 |
| [Ruminococcus] torques | 786 | 0.6 | -0.213 | -0.348 | -0.079 | 0.002 |
| bacterium F082 | 5 | Not detected | 0.031 | -0.078 | 0.140 | 0.575 |
| bacterium LF-3 | 4 | Not detected | 0.122 | -0.041 | 0.286 | 0.143 |
| bacterium MS4 | 12 | Not detected | 0.029 | -0.109 | 0.166 | 0.684 |

| bacterium OL-1 | 4 | Not detected | 0.246 | -0.023 | 0.514 | 0.073 |
| --- | --- | --- | --- | --- | --- | --- |
| bacterium P201 | 4 | Not detected | 0.179 | 0.012 | 0.346 | 0.035 |
| bacterium P3 | 6 | Not detected | 0.237 | 0.040 | 0.434 | 0.018 |

| **Supplementary Table 19**. Association of current SEP with species in foreign-born participants (n=1872). Analysis of Compositions of Microbiomes (ANCOM2) was used to identify species related to current SEP. Regression coefficients were obtained by modelling the effect of current SEP on centered log ratio (CLR)-transformed species in multivariable linear regression adjusted for age, gender, BMI, field center, Hispanic/Latino background, U.S. nativity, Bristol stool type, parental education, the alternative Healthy Eating Index 2010 score, cigarette smoking, alcohol use, depressive and anxiety symptoms, diabetes, hypertension, cardiovascular disease, dyslipidemia, and antibiotic use. | | | | | | |
| --- | --- | --- | --- | --- | --- | --- |
| **species** | **W_stat** | **ANCOM** | **beta** | **l95ci** | **u95ci** | **qval** |
| Bacteroides sp. CAG:98 | 1168 | 0.9 | -0.499 | -0.754 | -0.243 | 0.000 |
| Bifidobacterium dentium | 834 | 0.7 | 0.419 | 0.151 | 0.687 | 0.002 |
| Cryptobacterium sp. CAG:338 | 872 | 0.7 | -0.375 | -0.670 | -0.079 | 0.013 |
| Petrimonas mucosa | 889 | 0.7 | 0.297 | 0.105 | 0.489 | 0.002 |
| Ruminococcus sp. CAG:488 | 842 | 0.7 | 0.323 | 0.087 | 0.559 | 0.007 |
| Ruminococcus torques CAG:61 | 871 | 0.7 | -0.339 | -0.575 | -0.103 | 0.005 |
| Abiotrophia defectiva | 0 | Not detected | -0.015 | -0.213 | 0.184 | 0.885 |
| Acetanaerobacterium elongatum | 0 | Not detected | 0.126 | -0.010 | 0.262 | 0.070 |
| Acetitomaculum ruminis | 0 | Not detected | 0.055 | -0.022 | 0.133 | 0.163 |
| Acetivibrio cellulolyticus | 0 | Not detected | 0.051 | -0.107 | 0.210 | 0.524 |
| Acetivibrio ethanolgignens | 0 | Not detected | -0.003 | -0.072 | 0.066 | 0.933 |
| Acetobacter sp. 46_36 | 0 | Not detected | -0.021 | -0.329 | 0.286 | 0.892 |
| Acetobacter sp. CAG:267 | 0 | Not detected | -0.325 | -0.603 | -0.046 | 0.022 |
| Acholeplasma axanthum | 0 | Not detected | 0.108 | -0.031 | 0.246 | 0.127 |
| Acholeplasma equifetale | 0 | Not detected | 0.101 | -0.087 | 0.288 | 0.292 |
| Acidaminococcus fermentans | 0 | Not detected | -0.053 | -0.190 | 0.083 | 0.446 |
| Acidaminococcus intestini | 0 | Not detected | -0.152 | -0.396 | 0.092 | 0.222 |
| Acidaminococcus intestini CAG:325 | 0 | Not detected | -0.339 | -0.726 | 0.049 | 0.087 |
| Acinetobacter baumannii | 0 | Not detected | -0.048 | -0.214 | 0.118 | 0.569 |
| Acinetobacter bereziniae | 0 | Not detected | 0.053 | -0.110 | 0.216 | 0.525 |
| Acinetobacter johnsonii | 0 | Not detected | 0.001 | -0.178 | 0.181 | 0.987 |
| Acinetobacter sp. CAG:196 | 0 | Not detected | -0.104 | -0.445 | 0.238 | 0.552 |
| Acinetobacter sp. CAG:196_36_41 | 0 | Not detected | -0.102 | -0.434 | 0.230 | 0.546 |
| Acinetobacter sp. N54.MGS-139 | 0 | Not detected | -0.044 | -0.222 | 0.134 | 0.626 |
| Acinetobacter venetianus | 0 | Not detected | 0.039 | -0.158 | 0.237 | 0.697 |
| Actinobaculum sp. oral taxon 183 | 0 | Not detected | -0.051 | -0.281 | 0.180 | 0.667 |
| Actinobaculum suis | 0 | Not detected | -0.006 | -0.170 | 0.159 | 0.945 |
| Actinomyces dentalis | 2 | Not detected | 0.336 | 0.101 | 0.570 | 0.005 |
| Actinomyces gerencseriae | 0 | Not detected | 0.168 | -0.047 | 0.383 | 0.125 |
| Actinomyces graevenitzii | 0 | Not detected | 0.020 | -0.134 | 0.175 | 0.798 |
| Actinomyces hongkongensis | 0 | Not detected | -0.012 | -0.224 | 0.199 | 0.909 |
| Actinomyces israelii | 0 | Not detected | 0.191 | -0.031 | 0.414 | 0.092 |
| Actinomyces johnsonii | 0 | Not detected | 0.018 | -0.198 | 0.234 | 0.868 |
| Actinomyces massiliensis | 0 | Not detected | 0.072 | -0.149 | 0.292 | 0.525 |
| Actinomyces meyeri | 0 | Not detected | -0.007 | -0.192 | 0.178 | 0.942 |
| Actinomyces odontolyticus | 0 | Not detected | 0.093 | -0.074 | 0.261 | 0.275 |
| Actinomyces oris | 0 | Not detected | 0.092 | -0.106 | 0.290 | 0.361 |
| Actinomyces sp. HMSC065F12 | 0 | Not detected | -0.040 | -0.166 | 0.086 | 0.534 |

| Actinomyces sp. HMSC06A08 | 0 | Not detected | -0.051 | -0.180 | 0.079 | 0.444 |
| --- | --- | --- | --- | --- | --- | --- |
| Actinomyces sp. oral taxon 181 | 0 | Not detected | 0.061 | -0.149 | 0.271 | 0.569 |
| Actinomyces sp. oral taxon 448 | 0 | Not detected | 0.133 | -0.117 | 0.383 | 0.297 |
| Actinomyces sp. oral taxon 848 | 0 | Not detected | 0.122 | -0.045 | 0.290 | 0.151 |
| Actinomyces sp. ph3 | 0 | Not detected | -0.026 | -0.229 | 0.178 | 0.806 |
| Actinomyces timonensis | 0 | Not detected | -0.068 | -0.215 | 0.079 | 0.368 |
| Actinomyces turicensis | 0 | Not detected | 0.067 | -0.147 | 0.282 | 0.539 |
| Adlercreutzia equolifaciens | 0 | Not detected | -0.021 | -0.282 | 0.241 | 0.876 |
| Aeromonas salmonicida | 0 | Not detected | 0.005 | -0.204 | 0.215 | 0.960 |
| Aggregatibacter aphrophilus | 0 | Not detected | -0.027 | -0.263 | 0.208 | 0.820 |
| Akkermansia glycaniphila | 0 | Not detected | -0.042 | -0.254 | 0.169 | 0.695 |
| Akkermansia muciniphila | 0 | Not detected | 0.028 | -0.254 | 0.311 | 0.843 |
| Akkermansia muciniphila CAG:154 | 0 | Not detected | 0.092 | -0.271 | 0.455 | 0.620 |
| Akkermansia sp. 54_46 | 0 | Not detected | 0.083 | -0.296 | 0.462 | 0.670 |
| Akkermansia sp. CAG:344 | 0 | Not detected | 0.078 | -0.306 | 0.462 | 0.690 |
| Akkermansia sp. KLE1605 | 0 | Not detected | -0.230 | -0.552 | 0.091 | 0.160 |
| Akkermansia sp. KLE1797 | 0 | Not detected | -0.222 | -0.544 | 0.100 | 0.177 |
| Akkermansia sp. KLE1798 | 0 | Not detected | -0.217 | -0.539 | 0.105 | 0.187 |
| Akkermansia sp. Phil8 | 0 | Not detected | 0.099 | -0.276 | 0.474 | 0.605 |
| Akkermansia sp. UNK.MGS-1 | 0 | Not detected | 0.065 | -0.311 | 0.440 | 0.736 |
| Alistipes finegoldii | 0 | Not detected | -0.011 | -0.162 | 0.140 | 0.885 |
| Alistipes ihumii | 0 | Not detected | -0.061 | -0.315 | 0.193 | 0.637 |
| Alistipes indistinctus | 0 | Not detected | -0.061 | -0.412 | 0.290 | 0.733 |
| Alistipes inops | 0 | Not detected | -0.020 | -0.296 | 0.255 | 0.887 |
| Alistipes obesi | 0 | Not detected | -0.172 | -0.337 | -0.006 | 0.043 |
| Alistipes putredinis | 0 | Not detected | -0.157 | -0.373 | 0.058 | 0.153 |
| Alistipes senegalensis | 0 | Not detected | 0.019 | -0.115 | 0.153 | 0.781 |
| Alistipes shahii | 0 | Not detected | 0.008 | -0.143 | 0.160 | 0.914 |
| Alistipes sp. CAG:435 | 0 | Not detected | 0.221 | -0.142 | 0.584 | 0.232 |
| Alistipes sp. CAG:514 | 0 | Not detected | 0.300 | -0.024 | 0.623 | 0.070 |
| Alistipes sp. Zagget8 | 0 | Not detected | -0.011 | -0.230 | 0.208 | 0.919 |
| Alistipes timonensis | 0 | Not detected | 0.024 | -0.204 | 0.251 | 0.838 |
| Alkaliflexus imshenetskii | 0 | Not detected | -0.070 | -0.232 | 0.092 | 0.396 |
| Allisonella histaminiformans | 0 | Not detected | 0.219 | -0.111 | 0.548 | 0.194 |
| Allobaculum stercoricanis | 0 | Not detected | 0.011 | -0.068 | 0.091 | 0.778 |
| Allofustis seminis | 0 | Not detected | 0.147 | -0.037 | 0.332 | 0.117 |
| Alloprevotella rava | 0 | Not detected | 0.025 | -0.119 | 0.170 | 0.732 |
| Alloprevotella tannerae | 0 | Not detected | 0.010 | -0.091 | 0.111 | 0.841 |
| Alloscardovia criceti | 0 | Not detected | 0.104 | -0.075 | 0.283 | 0.256 |
| Alloscardovia omnicolens | 0 | Not detected | 0.062 | -0.132 | 0.255 | 0.533 |
| Amphibacillus xylanus | 0 | Not detected | 0.087 | -0.104 | 0.278 | 0.374 |
| Anaerobium acetethylicum | 0 | Not detected | 0.041 | -0.037 | 0.120 | 0.302 |
| Anaerococcus hydrogenalis | 0 | Not detected | -0.064 | -0.199 | 0.070 | 0.350 |
| Anaerococcus lactolyticus | 0 | Not detected | -0.120 | -0.317 | 0.077 | 0.233 |

| Anaerococcus obesiensis | 0 | Not detected | -0.149 | -0.315 | 0.018 | 0.081 |
| --- | --- | --- | --- | --- | --- | --- |
| Anaerocolumna aminovalerica | 0 | Not detected | -0.087 | -0.251 | 0.076 | 0.296 |
| Anaerocolumna xylanovorans | 0 | Not detected | -0.062 | -0.211 | 0.087 | 0.417 |
| Anaerofustis stercorihominis | 0 | Not detected | -0.080 | -0.322 | 0.162 | 0.519 |
| Anaeroglobus geminatus | 0 | Not detected | -0.098 | -0.290 | 0.095 | 0.322 |
| Anaeromassilibacillus senegalensis | 0 | Not detected | 0.021 | -0.115 | 0.156 | 0.767 |
| Anaerophaga thermohalophila | 0 | Not detected | 0.035 | -0.089 | 0.160 | 0.577 |
| Anaerosalibacter massiliensis | 0 | Not detected | 0.092 | -0.107 | 0.290 | 0.367 |
| Anaerosporobacter mobilis | 0 | Not detected | 0.028 | -0.060 | 0.117 | 0.532 |
| Anaerosporomusa subterranea | 0 | Not detected | -0.045 | -0.181 | 0.090 | 0.513 |
| Anaerostipes caccae | 0 | Not detected | 0.017 | -0.064 | 0.098 | 0.689 |
| Anaerostipes hadrus | 0 | Not detected | -0.052 | -0.190 | 0.085 | 0.457 |
| Anaerostipes sp. CAG:276 | 0 | Not detected | -0.122 | -0.402 | 0.158 | 0.392 |
| Anaerotruncus colihominis | 0 | Not detected | -0.062 | -0.154 | 0.031 | 0.190 |
| Anaerotruncus sp. CAG:390 | 0 | Not detected | 0.114 | -0.137 | 0.365 | 0.375 |
| Anaerotruncus sp. CAG:528 | 0 | Not detected | 0.087 | -0.115 | 0.289 | 0.401 |
| Anaerotruncus sp. G3(2012) | 0 | Not detected | -0.044 | -0.113 | 0.025 | 0.211 |
| Anaerovorax odorimutans | 0 | Not detected | 0.015 | -0.142 | 0.171 | 0.854 |
| Angelakisella massiliensis | 0 | Not detected | -0.011 | -0.090 | 0.068 | 0.782 |
| Aphanizomenon flos-aquae | 0 | Not detected | 0.093 | -0.047 | 0.233 | 0.194 |
| Aquabacterium parvum | 0 | Not detected | 0.117 | -0.056 | 0.289 | 0.185 |
| Arcanobacterium haemolyticum | 0 | Not detected | -0.158 | -0.328 | 0.011 | 0.067 |
| Asaccharospora irregularis | 0 | Not detected | 0.098 | -0.096 | 0.291 | 0.322 |
| Atlantibacter hermannii | 0 | Not detected | 0.019 | -0.234 | 0.272 | 0.882 |
| Atopobium deltae | 0 | Not detected | 0.100 | -0.084 | 0.285 | 0.287 |
| Atopobium minutum | 0 | Not detected | -0.035 | -0.222 | 0.152 | 0.713 |
| Atopobium parvulum | 0 | Not detected | 0.123 | -0.085 | 0.331 | 0.247 |
| Atopobium rimae | 0 | Not detected | 0.141 | -0.030 | 0.312 | 0.107 |
| Atopobium sp. BS2 | 0 | Not detected | 0.079 | -0.125 | 0.283 | 0.448 |
| Atopobium sp. HMSC064B08 | 0 | Not detected | 0.140 | -0.006 | 0.287 | 0.061 |
| Atopobium sp. ICM42b | 0 | Not detected | 0.153 | -0.053 | 0.359 | 0.145 |
| Atopobium vaginae | 0 | Not detected | 0.148 | -0.044 | 0.340 | 0.132 |
| Atopostipes suicloacalis | 0 | Not detected | 0.185 | 0.004 | 0.367 | 0.045 |
| Avibacterium paragallinarum | 2 | Not detected | -0.143 | -0.314 | 0.027 | 0.099 |
| Azospirillum sp. 47_25 | 0 | Not detected | -0.009 | -0.377 | 0.358 | 0.960 |
| Azospirillum sp. CAG:260 | 0 | Not detected | -0.023 | -0.385 | 0.340 | 0.902 |
| Bacillus acidiproducens | 0 | Not detected | 0.207 | 0.006 | 0.408 | 0.044 |
| Bacillus andreraoultii | 0 | Not detected | 0.208 | 0.008 | 0.409 | 0.042 |
| Bacillus azotoformans | 0 | Not detected | 0.003 | -0.179 | 0.186 | 0.971 |
| Bacillus bataviensis | 0 | Not detected | 0.002 | -0.173 | 0.177 | 0.983 |
| Bacillus coahuilensis | 0 | Not detected | -0.042 | -0.252 | 0.168 | 0.692 |
| Bacillus rubiinfantis | 0 | Not detected | 0.162 | -0.017 | 0.340 | 0.076 |
| Bacillus sp. CAG:988 | 0 | Not detected | 0.098 | -0.070 | 0.267 | 0.254 |
| Bacillus thermotolerans | 0 | Not detected | 0.173 | -0.020 | 0.367 | 0.080 |

| Bacteroidales bacterium 45-6 | 0 | Not detected | -0.022 | -0.217 | 0.172 | 0.821 |
| --- | --- | --- | --- | --- | --- | --- |
| Bacteroidales bacterium 55_9 | 0 | Not detected | -0.042 | -0.139 | 0.056 | 0.404 |
| Bacteroidales bacterium Bact_08 | 0 | Not detected | -0.130 | -0.284 | 0.024 | 0.098 |
| Bacteroidales bacterium Bact_18 | 0 | Not detected | 0.073 | -0.086 | 0.231 | 0.370 |
| Bacteroidales bacterium Bact_23 | 0 | Not detected | 0.026 | -0.176 | 0.228 | 0.803 |
| Bacteroidales bacterium CF | 0 | Not detected | 0.108 | -0.066 | 0.282 | 0.223 |
| Bacteroidales bacterium H10 | 1 | Not detected | 0.034 | -0.116 | 0.185 | 0.656 |
| Bacteroidales bacterium K1 | 0 | Not detected | -0.032 | -0.208 | 0.143 | 0.718 |
| Bacteroidales bacterium K10 | 0 | Not detected | -0.117 | -0.292 | 0.058 | 0.192 |
| Bacteroidales bacterium KHT7 | 0 | Not detected | 0.080 | -0.122 | 0.281 | 0.438 |
| Bacteroides barnesiae | 0 | Not detected | -0.079 | -0.176 | 0.017 | 0.108 |
| Bacteroides caccae CAG:21 | 0 | Not detected | -0.082 | -0.289 | 0.124 | 0.435 |
| Bacteroides cellulosilyticus | 0 | Not detected | -0.057 | -0.237 | 0.122 | 0.530 |
| Bacteroides coprocola | 396 | Not detected | -0.198 | -0.335 | -0.061 | 0.005 |
| Bacteroides coprophilus | 0 | Not detected | -0.037 | -0.133 | 0.060 | 0.459 |
| Bacteroides coprosuis | 1 | Not detected | 0.142 | -0.012 | 0.296 | 0.071 |
| Bacteroides dorei CAG:222 | 0 | Not detected | -0.098 | -0.261 | 0.066 | 0.241 |
| Bacteroides faecichinchillae | 0 | Not detected | -0.085 | -0.183 | 0.014 | 0.092 |
| Bacteroides faecis CAG:32 | 0 | Not detected | -0.053 | -0.255 | 0.149 | 0.607 |
| Bacteroides finegoldii | 0 | Not detected | -0.244 | -0.429 | -0.058 | 0.010 |
| Bacteroides finegoldii CAG:203 | 0 | Not detected | -0.213 | -0.387 | -0.038 | 0.017 |
| Bacteroides fluxus | 0 | Not detected | -0.026 | -0.129 | 0.078 | 0.628 |
| Bacteroides fragilis | 0 | Not detected | -0.103 | -0.244 | 0.038 | 0.153 |
| Bacteroides fragilis CAG:47 | 0 | Not detected | 0.003 | -0.226 | 0.231 | 0.981 |
| Bacteroides graminisolvens | 0 | Not detected | -0.001 | -0.218 | 0.215 | 0.991 |
| Bacteroides neonati | 0 | Not detected | -0.066 | -0.142 | 0.010 | 0.089 |
| Bacteroides oleiciplenus | 0 | Not detected | -0.117 | -0.235 | 0.002 | 0.054 |
| Bacteroides ovatus | 0 | Not detected | -0.078 | -0.215 | 0.060 | 0.268 |
| Bacteroides ovatus CAG:22 | 0 | Not detected | -0.114 | -0.264 | 0.036 | 0.136 |
| Bacteroides paurosaccharolyticus | 0 | Not detected | -0.153 | -0.294 | -0.012 | 0.034 |
| Bacteroides pectinophilus CAG:437 | 0 | Not detected | 0.005 | -0.131 | 0.142 | 0.938 |
| Bacteroides plebeius | 0 | Not detected | -0.166 | -0.311 | -0.021 | 0.025 |
| Bacteroides propionicifaciens | 0 | Not detected | 0.002 | -0.185 | 0.189 | 0.980 |
| Bacteroides pyogenes | 0 | Not detected | -0.148 | -0.265 | -0.030 | 0.014 |
| Bacteroides reticulotermitis | 0 | Not detected | -0.097 | -0.221 | 0.026 | 0.122 |
| Bacteroides salanitronis | 0 | Not detected | -0.073 | -0.171 | 0.025 | 0.146 |
| Bacteroides salyersiae | 0 | Not detected | -0.170 | -0.310 | -0.030 | 0.018 |
| Bacteroides sp. 41_26 | 0 | Not detected | -0.128 | -0.288 | 0.033 | 0.119 |
| Bacteroides sp. 43_46 | 0 | Not detected | -0.065 | -0.205 | 0.075 | 0.364 |
| Bacteroides sp. CAG:1060 | 0 | Not detected | 0.059 | -0.196 | 0.313 | 0.650 |
| Bacteroides sp. CAG:1076 | 0 | Not detected | -0.059 | -0.221 | 0.103 | 0.475 |
| Bacteroides sp. CAG:530 | 804 | 0.6 | 0.210 | 0.057 | 0.364 | 0.007 |
| Bacteroides sp. CAG:545 | 0 | Not detected | 0.205 | -0.128 | 0.537 | 0.228 |
| Bacteroides sp. CAG:714 | 0 | Not detected | -0.095 | -0.329 | 0.138 | 0.424 |

| Bacteroides sp. CAG:770 | 0 | Not detected | 0.051 | -0.132 | 0.235 | 0.583 |
| --- | --- | --- | --- | --- | --- | --- |
| Bacteroides sp. D2 | 0 | Not detected | -0.128 | -0.286 | 0.029 | 0.111 |
| Bacteroides sp. Ga6A1 | 0 | Not detected | -0.001 | -0.224 | 0.222 | 0.995 |
| Bacteroides sp. Ga6A2 | 0 | Not detected | -0.025 | -0.248 | 0.198 | 0.825 |
| Bacteroides stercoris | 0 | Not detected | -0.223 | -0.401 | -0.044 | 0.015 |
| Bacteroides thetaiotaomicron | 0 | Not detected | -0.027 | -0.128 | 0.074 | 0.598 |
| Bacteroides thetaiotaomicron CAG:40 | 0 | Not detected | -0.052 | -0.212 | 0.108 | 0.524 |
| Bacteroides uniformis | 0 | Not detected | -0.107 | -0.252 | 0.038 | 0.149 |
| Bacteroides vulgatus | 0 | Not detected | -0.080 | -0.216 | 0.056 | 0.248 |
| Bacteroides vulgatus CAG:6 | 0 | Not detected | -0.113 | -0.276 | 0.049 | 0.172 |
| Bacteroidetes bacterium OLB8 | 0 | Not detected | 0.055 | -0.131 | 0.241 | 0.562 |
| Bacteroidetes oral taxon 274 | 0 | Not detected | 0.092 | -0.038 | 0.221 | 0.166 |
| Bariatricus massiliensis | 0 | Not detected | -0.008 | -0.081 | 0.065 | 0.823 |
| Barnesiella intestinihominis | 0 | Not detected | -0.157 | -0.337 | 0.022 | 0.086 |
| Barnesiella viscericola | 0 | Not detected | -0.040 | -0.122 | 0.042 | 0.337 |
| Bavariicoccus seileri | 0 | Not detected | 0.035 | -0.135 | 0.205 | 0.685 |
| Beduini massiliensis | 0 | Not detected | 0.078 | -0.033 | 0.189 | 0.166 |
| Bibersteinia trehalosi | 0 | Not detected | 0.050 | -0.150 | 0.250 | 0.625 |
| Bifidobacterium actinocoloniiforme | 0 | Not detected | 0.167 | -0.023 | 0.358 | 0.086 |
| Bifidobacterium adolescentis | 0 | Not detected | 0.154 | -0.149 | 0.456 | 0.320 |
| Bifidobacterium aesculapii | 0 | Not detected | 0.124 | -0.031 | 0.280 | 0.118 |
| Bifidobacterium angulatum | 596 | Not detected | 0.316 | 0.069 | 0.563 | 0.012 |
| Bifidobacterium animalis | 0 | Not detected | -0.040 | -0.256 | 0.175 | 0.712 |
| Bifidobacterium asteroides | 0 | Not detected | 0.203 | 0.004 | 0.403 | 0.046 |
| Bifidobacterium biavatii | 0 | Not detected | 0.063 | -0.015 | 0.142 | 0.115 |
| Bifidobacterium bifidum | 0 | Not detected | 0.118 | -0.169 | 0.405 | 0.419 |
| Bifidobacterium bohemicum | 0 | Not detected | 0.188 | 0.008 | 0.369 | 0.041 |
| Bifidobacterium bombi | 0 | Not detected | 0.248 | 0.069 | 0.427 | 0.007 |
| Bifidobacterium breve | 1 | Not detected | 0.310 | 0.036 | 0.584 | 0.027 |
| Bifidobacterium callitrichos | 1 | Not detected | 0.198 | -0.046 | 0.441 | 0.112 |
| Bifidobacterium choerinum | 0 | Not detected | 0.129 | -0.083 | 0.341 | 0.233 |
| Bifidobacterium coryneforme | 0 | Not detected | 0.227 | 0.036 | 0.418 | 0.020 |
| Bifidobacterium crudilactis | 0 | Not detected | 0.133 | -0.026 | 0.292 | 0.101 |
| Bifidobacterium cuniculi | 0 | Not detected | 0.228 | 0.023 | 0.433 | 0.029 |
| Bifidobacterium gallicum | 0 | Not detected | 0.173 | -0.033 | 0.379 | 0.099 |
| Bifidobacterium gallinarum | 1 | Not detected | 0.305 | 0.060 | 0.551 | 0.015 |
| Bifidobacterium longum | 0 | Not detected | 0.257 | -0.020 | 0.535 | 0.069 |
| Bifidobacterium magnum | 0 | Not detected | 0.083 | -0.048 | 0.214 | 0.212 |
| Bifidobacterium minimum | 0 | Not detected | 0.200 | -0.056 | 0.456 | 0.126 |
| Bifidobacterium mongoliense | 1 | Not detected | 0.318 | 0.124 | 0.513 | 0.001 |
| Bifidobacterium pseudolongum | 0 | Not detected | 0.172 | -0.048 | 0.392 | 0.125 |
| Bifidobacterium psychraerophilum | 0 | Not detected | 0.070 | -0.075 | 0.215 | 0.346 |
| Bifidobacterium reuteri | 1 | Not detected | 0.168 | -0.033 | 0.369 | 0.101 |
| Bifidobacterium saguini | 1 | Not detected | 0.261 | -0.005 | 0.526 | 0.054 |

| Bifidobacterium scardovii | 1 | Not detected | 0.287 | 0.026 | 0.549 | 0.032 |
| --- | --- | --- | --- | --- | --- | --- |
| Bifidobacterium stellenboschense | 1 | Not detected | 0.085 | -0.003 | 0.174 | 0.059 |
| Bifidobacterium subtile | 0 | Not detected | 0.243 | 0.012 | 0.475 | 0.039 |
| Bifidobacterium thermophilum | 0 | Not detected | 0.126 | -0.002 | 0.255 | 0.054 |
| Bifidobacterium tsurumiense | 0 | Not detected | 0.151 | -0.055 | 0.358 | 0.152 |
| Bilophila wadsworthia | 0 | Not detected | 0.126 | -0.189 | 0.441 | 0.434 |
| Bittarella massiliensis | 0 | Not detected | -0.016 | -0.094 | 0.062 | 0.685 |
| Blautia hansenii | 0 | Not detected | -0.041 | -0.127 | 0.046 | 0.358 |
| Blautia hydrogenotrophica | 0 | Not detected | -0.067 | -0.148 | 0.014 | 0.104 |
| Blautia hydrogenotrophica CAG:147 | 0 | Not detected | -0.027 | -0.281 | 0.226 | 0.833 |
| Blautia obeum | 0 | Not detected | -0.024 | -0.107 | 0.060 | 0.578 |
| Blautia producta | 0 | Not detected | -0.151 | -0.265 | -0.036 | 0.010 |
| Blautia schinkii | 0 | Not detected | -0.016 | -0.079 | 0.046 | 0.613 |
| Blautia sp. CAG:237 | 0 | Not detected | -0.080 | -0.259 | 0.100 | 0.384 |
| Blautia sp. CAG:37 | 0 | Not detected | -0.089 | -0.254 | 0.077 | 0.293 |
| Blautia sp. CAG:37_48_57 | 0 | Not detected | -0.098 | -0.226 | 0.030 | 0.132 |
| Blautia sp. KLE 1732 | 0 | Not detected | 0.030 | -0.072 | 0.132 | 0.565 |
| Brenneria goodwinii | 0 | Not detected | -0.051 | -0.212 | 0.111 | 0.538 |
| Bulleidia extructa | 0 | Not detected | 0.160 | -0.034 | 0.354 | 0.106 |
| Burkholderiales bacterium GJ-E10 | 0 | Not detected | -0.129 | -0.310 | 0.051 | 0.160 |
| Buttiauxella ferragutiae | 0 | Not detected | -0.024 | -0.205 | 0.157 | 0.798 |
| Butyricicoccus desmolans | 0 | Not detected | -0.059 | -0.127 | 0.010 | 0.092 |
| Butyricicoccus pullicaecorum | 0 | Not detected | 0.053 | -0.034 | 0.141 | 0.232 |
| Butyricicoccus sp. K4410.MGS-46 | 0 | Not detected | -0.050 | -0.163 | 0.063 | 0.384 |
| Butyricimonas sp. Marseille-P2440 | 0 | Not detected | -0.075 | -0.242 | 0.093 | 0.382 |
| Butyricimonas synergistica | 0 | Not detected | 0.065 | -0.143 | 0.272 | 0.541 |
| Butyricimonas virosa | 0 | Not detected | -0.008 | -0.181 | 0.165 | 0.931 |
| Butyrivibrio crossotus | 0 | Not detected | 0.008 | -0.142 | 0.157 | 0.919 |
| Butyrivibrio crossotus CAG:259 | 0 | Not detected | 0.033 | -0.191 | 0.256 | 0.775 |
| Butyrivibrio fibrisolvens | 0 | Not detected | 0.029 | -0.147 | 0.204 | 0.747 |
| Butyrivibrio hungatei | 0 | Not detected | -0.076 | -0.212 | 0.061 | 0.276 |
| Butyrivibrio proteoclasticus | 0 | Not detected | -0.048 | -0.144 | 0.048 | 0.326 |
| Butyrivibrio sp. AD3002 | 0 | Not detected | 0.038 | -0.084 | 0.161 | 0.540 |
| Butyrivibrio sp. AE2005 | 0 | Not detected | 0.040 | -0.091 | 0.172 | 0.547 |
| Butyrivibrio sp. AE3003 | 0 | Not detected | -0.014 | -0.108 | 0.080 | 0.770 |
| Butyrivibrio sp. AE3004 | 0 | Not detected | -0.061 | -0.155 | 0.033 | 0.205 |
| Butyrivibrio sp. AE3009 | 0 | Not detected | 0.103 | -0.047 | 0.254 | 0.179 |
| Butyrivibrio sp. FCS006 | 0 | Not detected | -0.011 | -0.166 | 0.143 | 0.884 |
| Butyrivibrio sp. INlla14 | 0 | Not detected | -0.006 | -0.133 | 0.121 | 0.925 |
| Butyrivibrio sp. INlla16 | 0 | Not detected | -0.021 | -0.154 | 0.113 | 0.763 |
| Butyrivibrio sp. INlla21 | 0 | Not detected | -0.071 | -0.204 | 0.062 | 0.294 |
| Butyrivibrio sp. LB2008 | 0 | Not detected | 0.100 | -0.051 | 0.250 | 0.194 |
| Butyrivibrio sp. M55 | 0 | Not detected | 0.019 | -0.123 | 0.160 | 0.794 |
| Butyrivibrio sp. NC2002 | 0 | Not detected | -0.050 | -0.200 | 0.100 | 0.514 |

| Butyrivibrio sp. NC2007 | 0 | Not detected | -0.001 | -0.139 | 0.137 | 0.985 |
| --- | --- | --- | --- | --- | --- | --- |
| Butyrivibrio sp. NC3005 | 0 | Not detected | 0.022 | -0.174 | 0.218 | 0.826 |
| Butyrivibrio sp. VCD2006 | 0 | Not detected | 0.028 | -0.113 | 0.170 | 0.698 |
| Butyrivibrio sp. WCD2001 | 0 | Not detected | -0.029 | -0.169 | 0.112 | 0.691 |
| Butyrivibrio sp. WCD3002 | 0 | Not detected | -0.036 | -0.168 | 0.096 | 0.591 |
| Butyrivibrio sp. ob235 | 0 | Not detected | 0.087 | -0.082 | 0.255 | 0.315 |
| Campylobacter coli | 0 | Not detected | -0.119 | -0.229 | -0.009 | 0.034 |
| Campylobacter hominis | 0 | Not detected | -0.110 | -0.329 | 0.108 | 0.321 |
| Candidatus Azobacteroides pseudotricho | 0 | Not detected | -0.006 | -0.142 | 0.130 | 0.931 |
| Candidatus Gastranaerophilales bacteriu | 0 | Not detected | -0.131 | -0.323 | 0.062 | 0.184 |
| Candidatus Melainabacteria bacterium L | 0 | Not detected | -0.010 | -0.146 | 0.126 | 0.885 |
| Candidatus Melainabacteria bacterium M | 0 | Not detected | 0.075 | -0.203 | 0.352 | 0.598 |
| Candidatus Saccharibacteria oral taxon T | 0 | Not detected | 0.047 | -0.114 | 0.208 | 0.565 |
| Candidatus Soleaferrea massiliensis | 0 | Not detected | 0.078 | -0.038 | 0.195 | 0.187 |
| Candidatus Stoquefichus sp. SB1 | 0 | Not detected | -0.059 | -0.169 | 0.050 | 0.289 |
| Capnocytophaga granulosa | 0 | Not detected | 0.090 | -0.079 | 0.260 | 0.296 |
| Capnocytophaga sp. oral taxon 329 | 0 | Not detected | -0.062 | -0.305 | 0.181 | 0.618 |
| Capnocytophaga sp. oral taxon 863 | 0 | Not detected | -0.154 | -0.360 | 0.052 | 0.144 |
| Capnocytophaga sputigena | 0 | Not detected | -0.029 | -0.161 | 0.103 | 0.663 |
| Carnobacterium gallinarum | 0 | Not detected | 0.057 | -0.143 | 0.257 | 0.575 |
| Catabacter hongkongensis | 0 | Not detected | -0.066 | -0.222 | 0.089 | 0.404 |
| Catenibacterium mitsuokai | 0 | Not detected | 0.125 | -0.152 | 0.402 | 0.376 |
| Catenibacterium sp. CAG:290 | 0 | Not detected | 0.146 | -0.194 | 0.487 | 0.400 |
| Catonella morbi | 0 | Not detected | -0.087 | -0.181 | 0.007 | 0.070 |
| Cedecea neteri | 0 | Not detected | 0.039 | -0.187 | 0.264 | 0.738 |
| Cellulomonas carbonis | 0 | Not detected | 0.126 | -0.071 | 0.324 | 0.211 |
| Cellulomonas flavigena | 0 | Not detected | 0.072 | -0.140 | 0.285 | 0.506 |
| Cellulosilyticum lentocellum | 0 | Not detected | 0.098 | -0.053 | 0.250 | 0.203 |
| Cellulosilyticum ruminicola | 0 | Not detected | -0.046 | -0.209 | 0.117 | 0.581 |
| Chlamydia psittaci | 0 | Not detected | 0.110 | -0.069 | 0.288 | 0.230 |
| Christensenella sp. AF73-05CM02 | 0 | Not detected | -0.010 | -0.215 | 0.196 | 0.927 |
| Christensenellaceae bacterium Phil1 | 1 | Not detected | 0.165 | 0.014 | 0.316 | 0.033 |
| Christensenellaceae bacterium Phil10 | 0 | Not detected | -0.036 | -0.249 | 0.178 | 0.742 |
| Christensenellaceae bacterium Phil7 | 0 | Not detected | 0.144 | -0.077 | 0.366 | 0.202 |
| Chryseobacterium gallinarum | 0 | Not detected | -0.136 | -0.385 | 0.112 | 0.282 |
| Chryseobacterium greenlandense | 0 | Not detected | -0.139 | -0.371 | 0.093 | 0.240 |
| Citrobacter amalonaticus | 0 | Not detected | 0.019 | -0.267 | 0.304 | 0.899 |
| Citrobacter freundii | 0 | Not detected | -0.035 | -0.328 | 0.258 | 0.813 |
| Cloacibacillus evryensis | 0 | Not detected | 0.180 | -0.096 | 0.456 | 0.202 |
| Cloacibacillus porcorum | 0 | Not detected | 0.040 | -0.192 | 0.273 | 0.734 |
| Cloacibacterium normanense | 0 | Not detected | -0.100 | -0.326 | 0.126 | 0.386 |
| Clostridia bacterium UC5.1-1C12 | 0 | Not detected | -0.206 | -0.461 | 0.048 | 0.113 |
| Clostridia bacterium UC5.1-1D1 | 0 | Not detected | -0.012 | -0.095 | 0.071 | 0.783 |
| Clostridia bacterium UC5.1-1E11 | 0 | Not detected | 0.008 | -0.072 | 0.087 | 0.850 |

| Clostridiales bacterium 1_7_47FAA | 0 | Not detected | 0.003 | -0.089 | 0.094 | 0.952 |
| --- | --- | --- | --- | --- | --- | --- |
| Clostridiales bacterium 59_14 | 0 | Not detected | 0.011 | -0.122 | 0.143 | 0.874 |
| Clostridiales bacterium DTU035 | 0 | Not detected | 0.048 | -0.117 | 0.212 | 0.569 |
| Clostridiales bacterium DTU036 | 0 | Not detected | -0.053 | -0.207 | 0.101 | 0.500 |
| Clostridiales bacterium DTU089 | 0 | Not detected | -0.041 | -0.181 | 0.099 | 0.564 |
| Clostridiales bacterium Firm_03 | 0 | Not detected | -0.007 | -0.196 | 0.182 | 0.940 |
| Clostridiales bacterium Firm_16 | 0 | Not detected | -0.063 | -0.277 | 0.150 | 0.561 |
| Clostridiales bacterium NK3B98 | 0 | Not detected | 0.046 | -0.130 | 0.221 | 0.610 |
| Clostridiales bacterium S5-A14a | 0 | Not detected | 0.022 | -0.058 | 0.101 | 0.589 |
| Clostridiales bacterium SIT11 | 0 | Not detected | 0.169 | -0.008 | 0.346 | 0.061 |
| Clostridiales bacterium VE202-08 | 0 | Not detected | -0.041 | -0.134 | 0.052 | 0.391 |
| Clostridioides difficile | 0 | Not detected | -0.072 | -0.148 | 0.005 | 0.066 |
| Clostridioides mangenotii | 0 | Not detected | 0.026 | -0.146 | 0.199 | 0.764 |
| Clostridium baratii | 0 | Not detected | 0.085 | -0.121 | 0.291 | 0.418 |
| Clostridium beijerinckii | 0 | Not detected | 0.111 | -0.078 | 0.299 | 0.251 |
| Clostridium bolteae CAG:59 | 0 | Not detected | -0.174 | -0.320 | -0.029 | 0.019 |
| Clostridium botulinum | 0 | Not detected | 0.236 | 0.036 | 0.436 | 0.021 |
| Clostridium butyricum | 0 | Not detected | 0.100 | -0.104 | 0.304 | 0.338 |
| Clostridium cadaveris | 0 | Not detected | -0.042 | -0.206 | 0.123 | 0.620 |
| Clostridium celatum | 0 | Not detected | 0.281 | -0.015 | 0.577 | 0.063 |
| Clostridium cellulovorans | 0 | Not detected | -0.019 | -0.132 | 0.095 | 0.748 |
| Clostridium clostridioforme CAG:132 | 0 | Not detected | -0.228 | -0.430 | -0.025 | 0.028 |
| Clostridium clostridioforme CAG:511 | 0 | Not detected | -0.259 | -0.445 | -0.074 | 0.006 |
| Clostridium colicanis | 0 | Not detected | -0.016 | -0.174 | 0.142 | 0.841 |
| Clostridium disporicum | 0 | Not detected | 0.153 | 0.009 | 0.297 | 0.037 |
| Clostridium ihumii | 0 | Not detected | 0.245 | 0.073 | 0.418 | 0.005 |
| Clostridium intestinale | 0 | Not detected | 0.030 | -0.113 | 0.173 | 0.679 |
| Clostridium kluyveri | 0 | Not detected | 0.173 | -0.021 | 0.366 | 0.080 |
| Clostridium leptum CAG:27 | 0 | Not detected | -0.042 | -0.271 | 0.186 | 0.716 |
| Clostridium lundense | 0 | Not detected | 0.094 | -0.099 | 0.286 | 0.341 |
| Clostridium magnum | 0 | Not detected | 0.092 | -0.049 | 0.232 | 0.201 |
| Clostridium neonatale | 0 | Not detected | 0.105 | -0.067 | 0.278 | 0.232 |
| Clostridium nexile CAG:348 | 0 | Not detected | -0.054 | -0.198 | 0.089 | 0.459 |
| Clostridium paraputrificum | 0 | Not detected | -0.064 | -0.309 | 0.181 | 0.608 |
| Clostridium perfringens | 0 | Not detected | 0.095 | -0.205 | 0.395 | 0.534 |
| Clostridium phoceensis | 166 | Not detected | -0.169 | -0.287 | -0.051 | 0.005 |
| Clostridium polynesiense | 1 | Not detected | 0.189 | 0.000 | 0.378 | 0.051 |
| Clostridium saccharobutylicum | 0 | Not detected | 0.098 | -0.078 | 0.275 | 0.276 |
| Clostridium saccharoperbutylacetonicum | 0 | Not detected | 0.133 | -0.038 | 0.305 | 0.127 |
| Clostridium sartagoforme | 0 | Not detected | 0.167 | -0.043 | 0.377 | 0.119 |
| Clostridium sp. 44_14 | 0 | Not detected | -0.076 | -0.253 | 0.101 | 0.399 |
| Clostridium sp. ASF356 | 0 | Not detected | -0.094 | -0.292 | 0.104 | 0.354 |
| Clostridium sp. Ade.TY | 0 | Not detected | 0.061 | -0.119 | 0.242 | 0.506 |
| Clostridium sp. CAG:1193 | 0 | Not detected | 0.324 | 0.106 | 0.542 | 0.004 |

| Clostridium sp. CAG:122 | 0 | Not detected | 0.005 | -0.227 | 0.236 | 0.968 |
| --- | --- | --- | --- | --- | --- | --- |
| Clostridium sp. CAG:12237_41 | 0 | Not detected | 0.019 | -0.159 | 0.196 | 0.835 |
| Clostridium sp. CAG:127 | 0 | Not detected | 0.221 | -0.042 | 0.484 | 0.100 |
| Clostridium sp. CAG:138 | 0 | Not detected | 0.338 | -0.013 | 0.689 | 0.060 |
| Clostridium sp. CAG:167 | 0 | Not detected | -0.121 | -0.420 | 0.178 | 0.427 |
| Clostridium sp. CAG:226 | 0 | Not detected | 0.253 | -0.014 | 0.521 | 0.064 |
| Clostridium sp. CAG:230 | 0 | Not detected | 0.024 | -0.160 | 0.207 | 0.799 |
| Clostridium sp. CAG:242 | 0 | Not detected | 0.034 | -0.228 | 0.297 | 0.797 |
| Clostridium sp. CAG:245 | 0 | Not detected | 0.027 | -0.371 | 0.425 | 0.895 |
| Clostridium sp. CAG:245_30_32 | 0 | Not detected | 0.018 | -0.145 | 0.182 | 0.827 |
| Clostridium sp. CAG:264 | 0 | Not detected | 0.036 | -0.225 | 0.296 | 0.788 |
| Clostridium sp. CAG:277 | 0 | Not detected | -0.114 | -0.297 | 0.069 | 0.221 |
| Clostridium sp. CAG:288 | 0 | Not detected | 0.194 | -0.086 | 0.475 | 0.175 |
| Clostridium sp. CAG:299 | 0 | Not detected | -0.215 | -0.450 | 0.019 | 0.072 |
| Clostridium sp. CAG:307_30_263 | 0 | Not detected | 0.020 | -0.134 | 0.173 | 0.804 |
| Clostridium sp. CAG:349 | 0 | Not detected | -0.007 | -0.377 | 0.363 | 0.969 |
| Clostridium sp. CAG:352 | 0 | Not detected | -0.018 | -0.248 | 0.213 | 0.882 |
| Clostridium sp. CAG:413 | 0 | Not detected | 0.194 | -0.073 | 0.461 | 0.155 |
| Clostridium sp. CAG:448 | 0 | Not detected | 0.183 | -0.110 | 0.476 | 0.221 |
| Clostridium sp. CAG:451 | 0 | Not detected | 0.227 | -0.070 | 0.524 | 0.134 |
| Clostridium sp. CAG:465 | 0 | Not detected | 0.109 | -0.169 | 0.387 | 0.442 |
| Clostridium sp. CAG:505 | 0 | Not detected | -0.196 | -0.467 | 0.075 | 0.156 |
| Clostridium sp. CAG:568 | 0 | Not detected | -0.169 | -0.459 | 0.121 | 0.253 |
| Clostridium sp. CAG:58 | 0 | Not detected | -0.159 | -0.354 | 0.037 | 0.112 |
| Clostridium sp. CAG:590 | 0 | Not detected | 0.065 | -0.077 | 0.207 | 0.369 |
| Clostridium sp. CAG:594 | 0 | Not detected | 0.163 | -0.162 | 0.487 | 0.326 |
| Clostridium sp. CAG:628 | 0 | Not detected | 0.320 | 0.020 | 0.621 | 0.037 |
| Clostridium sp. CAG:62_40_43 | 0 | Not detected | -0.033 | -0.344 | 0.278 | 0.836 |
| Clostridium sp. CAG:632 | 1 | Not detected | 0.192 | 0.005 | 0.380 | 0.044 |
| Clostridium sp. CAG:678 | 0 | Not detected | 0.052 | -0.162 | 0.266 | 0.635 |
| Clostridium sp. CAG:762 | 0 | Not detected | 0.263 | 0.007 | 0.519 | 0.045 |
| Clostridium sp. CAG:768 | 0 | Not detected | -0.037 | -0.206 | 0.132 | 0.667 |
| Clostridium sp. CAG:780 | 0 | Not detected | 0.110 | -0.209 | 0.429 | 0.500 |
| Clostridium sp. CAG:793 | 0 | Not detected | -0.016 | -0.272 | 0.239 | 0.900 |
| Clostridium sp. CAG:81 | 0 | Not detected | -0.007 | -0.145 | 0.132 | 0.927 |
| Clostridium sp. CAG:91 | 0 | Not detected | -0.069 | -0.297 | 0.159 | 0.553 |
| Clostridium sp. CAG:964 | 0 | Not detected | 0.001 | -0.134 | 0.137 | 0.985 |
| Clostridium sp. CL-6 | 0 | Not detected | 0.088 | -0.063 | 0.239 | 0.256 |
| Clostridium sp. HGF2 | 0 | Not detected | 0.007 | -0.074 | 0.087 | 0.872 |
| Clostridium sp. K4410.MGS-306 | 0 | Not detected | -0.018 | -0.123 | 0.087 | 0.734 |
| Clostridium sp. KLE 1755 | 0 | Not detected | -0.108 | -0.234 | 0.018 | 0.094 |
| Clostridium sp. KNHs205 | 0 | Not detected | -0.061 | -0.132 | 0.010 | 0.093 |
| Clostridium sp. KNHs209 | 0 | Not detected | -0.046 | -0.114 | 0.022 | 0.183 |
| Clostridium sp. KNHs214 | 1 | Not detected | 0.101 | -0.017 | 0.218 | 0.094 |

| Clostridium sp. L2-50 | 0 | Not detected | 0.008 | -0.098 | 0.113 | 0.885 |
| --- | --- | --- | --- | --- | --- | --- |
| Clostridium sp. LF2 | 0 | Not detected | 0.001 | -0.139 | 0.142 | 0.985 |
| Clostridium sp. M62/1 | 0 | Not detected | 0.000 | -0.068 | 0.067 | 0.991 |
| Clostridium sp. ND2 | 0 | Not detected | 0.014 | -0.155 | 0.183 | 0.871 |
| Clostridium sp. SY8519 | 0 | Not detected | 0.027 | -0.048 | 0.102 | 0.479 |
| Clostridium sulfidigenes | 0 | Not detected | 0.170 | 0.036 | 0.305 | 0.013 |
| Collinsella aerofaciens | 0 | Not detected | 0.089 | -0.113 | 0.292 | 0.388 |
| Collinsella intestinalis | 0 | Not detected | -0.060 | -0.284 | 0.164 | 0.602 |
| Collinsella sp. CAG:166 | 0 | Not detected | 0.088 | -0.137 | 0.313 | 0.443 |
| Collinsella sp. MS5 | 0 | Not detected | 0.112 | -0.041 | 0.264 | 0.152 |
| Collinsella stercoris | 0 | Not detected | -0.013 | -0.213 | 0.186 | 0.895 |
| Collinsella tanakaei | 0 | Not detected | -0.104 | -0.249 | 0.042 | 0.162 |
| Comamonas kerstersii | 0 | Not detected | 0.035 | -0.211 | 0.282 | 0.778 |
| Coprobacillus sp. CAG:605 | 0 | Not detected | 0.179 | -0.103 | 0.460 | 0.213 |
| Coprobacillus sp. D6 | 0 | Not detected | -0.014 | -0.114 | 0.085 | 0.778 |
| Coprobacter secundus | 0 | Not detected | -0.081 | -0.171 | 0.009 | 0.078 |
| Coprococcus comes | 0 | Not detected | 0.030 | -0.052 | 0.111 | 0.474 |
| Coprococcus eutactus | 0 | Not detected | 0.028 | -0.091 | 0.147 | 0.642 |
| Coprococcus eutactus CAG:665 | 0 | Not detected | 0.100 | -0.161 | 0.362 | 0.453 |
| Coprococcus sp. 43_8 | 0 | Not detected | 0.077 | -0.081 | 0.235 | 0.337 |
| Coprococcus sp. CAG:131 | 0 | Not detected | 0.052 | -0.203 | 0.307 | 0.688 |
| Coprococcus sp. CAG:131_42_139 | 0 | Not detected | 0.164 | -0.091 | 0.418 | 0.207 |
| Coprococcus sp. HPP0048 | 0 | Not detected | 0.008 | -0.117 | 0.133 | 0.899 |
| Coriobacteriaceae bacterium 68-1-3 | 0 | Not detected | -0.034 | -0.192 | 0.124 | 0.673 |
| Coriobacteriaceae bacterium BV3Ac1 | 0 | Not detected | 0.089 | -0.117 | 0.295 | 0.398 |
| Coriobacteriales bacterium DNF00809 | 0 | Not detected | 0.012 | -0.170 | 0.195 | 0.895 |
| Coriobacterium glomerans | 0 | Not detected | 0.141 | -0.030 | 0.312 | 0.105 |
| Corynebacterium amycolatum | 0 | Not detected | -0.023 | -0.159 | 0.112 | 0.734 |
| Corynebacterium durum | 0 | Not detected | -0.053 | -0.260 | 0.154 | 0.619 |
| Corynebacterium glucuronolyticum | 0 | Not detected | 0.036 | -0.123 | 0.196 | 0.655 |
| Corynebacterium singulare | 0 | Not detected | 0.035 | -0.121 | 0.191 | 0.660 |
| Corynebacterium urealyticum | 0 | Not detected | 0.020 | -0.152 | 0.191 | 0.823 |
| Criibacterium bergeronii | 0 | Not detected | 0.030 | -0.088 | 0.147 | 0.621 |
| Cronobacter sakazakii | 0 | Not detected | 0.009 | -0.271 | 0.290 | 0.949 |
| Culturomica massiliensis | 0 | Not detected | -0.092 | -0.197 | 0.012 | 0.083 |
| Cutibacterium acnes | 0 | Not detected | -0.028 | -0.215 | 0.160 | 0.773 |
| Defluviitalea phaphyphila | 0 | Not detected | -0.062 | -0.198 | 0.074 | 0.371 |
| Dehalobacter restrictus | 0 | Not detected | 0.085 | -0.058 | 0.227 | 0.244 |
| Dendrosporobacter quercicolus | 0 | Not detected | -0.069 | -0.205 | 0.067 | 0.317 |
| Denitrobacterium detoxificans | 0 | Not detected | -0.057 | -0.198 | 0.083 | 0.422 |
| Desnuesiella massiliensis | 0 | Not detected | 0.062 | -0.089 | 0.214 | 0.420 |
| Desulfovibrio desulfuricans | 0 | Not detected | 0.223 | -0.033 | 0.479 | 0.088 |
| Desulfovibrio fairfieldensis | 0 | Not detected | -0.021 | -0.314 | 0.272 | 0.887 |
| Desulfovibrio piger | 0 | Not detected | -0.211 | -0.575 | 0.154 | 0.258 |

| Desulfovibrio vulgaris | 0 | Not detected | 0.128 | -0.037 | 0.293 | 0.129 |
| --- | --- | --- | --- | --- | --- | --- |
| Devosia insulae | 0 | Not detected | 0.037 | -0.079 | 0.154 | 0.531 |
| Dialister invisus | 0 | Not detected | 0.043 | -0.332 | 0.417 | 0.823 |
| Dialister invisus CAG:218 | 0 | Not detected | -0.004 | -0.387 | 0.380 | 0.984 |
| Dialister micraerophilus | 0 | Not detected | 0.062 | -0.171 | 0.295 | 0.602 |
| Dialister sp. CAG:357 | 0 | Not detected | -0.022 | -0.401 | 0.357 | 0.910 |
| Dialister sp. CAG:486 | 0 | Not detected | 0.221 | -0.129 | 0.572 | 0.216 |
| Dialister sp. CAG:588 | 0 | Not detected | -0.042 | -0.246 | 0.162 | 0.686 |
| Dialister succinatiphilus | 0 | Not detected | 0.121 | -0.090 | 0.331 | 0.262 |
| Dickeya dadantii | 0 | Not detected | -0.025 | -0.190 | 0.140 | 0.765 |
| Dielma fastidiosa | 551 | Not detected | -0.255 | -0.444 | -0.065 | 0.008 |
| Dorea formicigenerans | 0 | Not detected | -0.003 | -0.077 | 0.072 | 0.943 |
| Dorea longicatena | 0 | Not detected | 0.079 | -0.029 | 0.188 | 0.153 |
| Dorea sp. 42_8 | 0 | Not detected | 0.060 | -0.066 | 0.186 | 0.353 |
| Dorea sp. 5-2 | 0 | Not detected | -0.020 | -0.077 | 0.037 | 0.500 |
| Dorea sp. CAG:105 | 0 | Not detected | 0.007 | -0.160 | 0.174 | 0.933 |
| Draconibacterium orientale | 0 | Not detected | 0.066 | -0.068 | 0.200 | 0.336 |
| Drancourtella massiliensis | 0 | Not detected | -0.060 | -0.194 | 0.073 | 0.374 |
| Dubosiella newyorkensis | 0 | Not detected | 0.148 | -0.001 | 0.296 | 0.051 |
| Dysgonomonas gadei | 0 | Not detected | -0.067 | -0.189 | 0.056 | 0.285 |
| Dysgonomonas mossii | 0 | Not detected | -0.035 | -0.180 | 0.110 | 0.636 |
| Dysgonomonas sp. BGC7 | 0 | Not detected | 0.044 | -0.137 | 0.225 | 0.634 |
| Dysgonomonas sp. HGC4 | 0 | Not detected | -0.010 | -0.166 | 0.146 | 0.899 |
| Edwardsiella ictaluri | 0 | Not detected | -0.090 | -0.259 | 0.078 | 0.294 |
| Eggerthella lenta | 0 | Not detected | -0.142 | -0.341 | 0.058 | 0.164 |
| Eggerthella sp. 51_9 | 0 | Not detected | 0.099 | -0.124 | 0.322 | 0.385 |
| Eggerthella sp. CAG:209 | 0 | Not detected | 0.204 | -0.140 | 0.547 | 0.245 |
| Eggerthella sp. CAG:368 | 0 | Not detected | 0.126 | -0.127 | 0.378 | 0.329 |
| Eggerthella sp. YY7918 | 0 | Not detected | -0.033 | -0.199 | 0.133 | 0.698 |
| Eggerthellaceae bacterium AT8 | 0 | Not detected | -0.006 | -0.105 | 0.094 | 0.908 |
| Eggerthia catenaformis | 0 | Not detected | 0.034 | -0.171 | 0.239 | 0.744 |
| Eisenbergiella tayi | 0 | Not detected | -0.094 | -0.258 | 0.069 | 0.259 |
| Elizabethkingia anophelis | 0 | Not detected | -0.071 | -0.286 | 0.143 | 0.514 |
| Emergencia timonensis | 0 | Not detected | -0.006 | -0.083 | 0.070 | 0.870 |
| Empedobacter brevis | 0 | Not detected | -0.205 | -0.479 | 0.068 | 0.141 |
| Enhygromyxa salina | 0 | Not detected | -0.068 | -0.253 | 0.117 | 0.471 |
| Enorma massiliensis | 0 | Not detected | 0.007 | -0.099 | 0.113 | 0.896 |
| Enorma timonensis | 0 | Not detected | -0.016 | -0.147 | 0.115 | 0.812 |
| Enterobacter cloacae | 0 | Not detected | -0.114 | -0.407 | 0.180 | 0.449 |
| Enterobacter lignolyticus | 0 | Not detected | 0.078 | -0.189 | 0.346 | 0.566 |
| Enterobacter sp. 638 | 0 | Not detected | -0.036 | -0.284 | 0.212 | 0.777 |
| Enterobacteriaceae bacterium LSJC7 | 0 | Not detected | -0.046 | -0.227 | 0.135 | 0.616 |
| Enterococcus avium | 0 | Not detected | -0.010 | -0.183 | 0.163 | 0.909 |
| Enterococcus cecorum | 0 | Not detected | 0.067 | -0.084 | 0.219 | 0.382 |

| Enterococcus columbae | 0 | Not detected | -0.036 | -0.231 | 0.159 | 0.715 |
| --- | --- | --- | --- | --- | --- | --- |
| Enterococcus durans | 1 | Not detected | 0.238 | 0.008 | 0.468 | 0.042 |
| Enterococcus faecalis | 0 | Not detected | -0.028 | -0.201 | 0.144 | 0.748 |
| Enterococcus faecium | 1 | Not detected | 0.148 | -0.037 | 0.332 | 0.118 |
| Enterococcus gallinarum | 0 | Not detected | -0.043 | -0.220 | 0.135 | 0.637 |
| Enterococcus gilvus | 0 | Not detected | 0.024 | -0.143 | 0.190 | 0.779 |
| Enterococcus italicus | 0 | Not detected | 0.086 | -0.119 | 0.291 | 0.410 |
| Enterococcus massiliensis | 0 | Not detected | 0.183 | -0.015 | 0.380 | 0.070 |
| Enterococcus sp. HMSC064A12 | 0 | Not detected | -0.013 | -0.213 | 0.187 | 0.898 |
| Enterorhabdus caecimuris | 0 | Not detected | -0.019 | -0.220 | 0.183 | 0.857 |
| Enterorhabdus mucosicola | 0 | Not detected | -0.063 | -0.235 | 0.108 | 0.470 |
| Erwinia billingiae | 0 | Not detected | -0.018 | -0.182 | 0.146 | 0.832 |
| Erwinia oleae | 0 | Not detected | -0.037 | -0.243 | 0.168 | 0.722 |
| Erysipelatoclostridium ramosum | 0 | Not detected | -0.134 | -0.377 | 0.109 | 0.281 |
| Erysipelotrichaceae bacterium 21_3 | 0 | Not detected | 0.011 | -0.093 | 0.115 | 0.837 |
| Erysipelotrichaceae bacterium 5_2_54FA | 0 | Not detected | 0.001 | -0.081 | 0.084 | 0.973 |
| Erysipelotrichaceae bacterium CAG:64 | 0 | Not detected | 0.065 | -0.117 | 0.248 | 0.482 |
| Erysipelotrichaceae bacterium I46 | 0 | Not detected | -0.010 | -0.106 | 0.085 | 0.833 |
| Escherichia coli | 0 | Not detected | 0.089 | -0.180 | 0.358 | 0.516 |
| Escherichia vulneris | 0 | Not detected | 0.008 | -0.229 | 0.245 | 0.949 |
| Ethanoligenens harbinense | 0 | Not detected | 0.106 | -0.071 | 0.284 | 0.240 |
| Eubacterium dolichum CAG:375 | 0 | Not detected | 0.080 | -0.188 | 0.349 | 0.559 |
| Eubacterium limosum | 0 | Not detected | 0.016 | -0.103 | 0.135 | 0.793 |
| Eubacterium plexicaudatum | 0 | Not detected | -0.018 | -0.087 | 0.051 | 0.607 |
| Eubacterium pyruvativorans | 0 | Not detected | 0.021 | -0.064 | 0.107 | 0.623 |
| Eubacterium ramulus | 0 | Not detected | -0.046 | -0.123 | 0.032 | 0.248 |
| Eubacterium sp. 14-2 | 0 | Not detected | -0.041 | -0.112 | 0.030 | 0.260 |
| Eubacterium sp. 3_1_31 | 0 | Not detected | -0.036 | -0.130 | 0.058 | 0.451 |
| Eubacterium sp. 45_250 | 0 | Not detected | 0.087 | -0.185 | 0.359 | 0.532 |
| Eubacterium sp. AB3007 | 0 | Not detected | 0.221 | -0.026 | 0.467 | 0.079 |
| Eubacterium sp. CAG:115 | 0 | Not detected | 0.053 | -0.348 | 0.455 | 0.794 |
| Eubacterium sp. CAG:146 | 0 | Not detected | 0.086 | -0.081 | 0.252 | 0.313 |
| Eubacterium sp. CAG:180 | 0 | Not detected | 0.044 | -0.302 | 0.389 | 0.804 |
| Eubacterium sp. CAG:192 | 0 | Not detected | -0.091 | -0.337 | 0.156 | 0.472 |
| Eubacterium sp. CAG:202 | 0 | Not detected | 0.062 | -0.200 | 0.325 | 0.642 |
| Eubacterium sp. CAG:248 | 0 | Not detected | -0.073 | -0.287 | 0.142 | 0.507 |
| Eubacterium sp. CAG:251 | 0 | Not detected | 0.093 | -0.217 | 0.403 | 0.558 |
| Eubacterium sp. CAG:252 | 0 | Not detected | -0.061 | -0.252 | 0.131 | 0.535 |
| Eubacterium sp. CAG:581 | 0 | Not detected | -0.036 | -0.309 | 0.238 | 0.799 |
| Eubacterium sp. CAG:603 | 0 | Not detected | 0.052 | -0.087 | 0.192 | 0.463 |
| Eubacterium sp. CAG:786 | 0 | Not detected | 0.110 | -0.203 | 0.423 | 0.490 |
| Eubacterium sp. CAG:841 | 0 | Not detected | 0.050 | -0.249 | 0.349 | 0.742 |
| Eubacterium sp. ER2 | 0 | Not detected | -0.021 | -0.087 | 0.046 | 0.544 |
| Eubacterium ventriosum | 0 | Not detected | -0.032 | -0.131 | 0.067 | 0.526 |

| Eubacterium xylanophilum | 0 | Not detected | -0.029 | -0.180 | 0.122 | 0.703 |
| --- | --- | --- | --- | --- | --- | --- |
| Facklamia hominis | 0 | Not detected | 0.165 | -0.021 | 0.350 | 0.082 |
| Facklamia ignava | 0 | Not detected | 0.069 | -0.114 | 0.253 | 0.459 |
| Facklamia languida | 0 | Not detected | -0.075 | -0.247 | 0.097 | 0.393 |
| Faecalibacterium prausnitzii | 0 | Not detected | 0.007 | -0.116 | 0.131 | 0.905 |
| Faecalibacterium sp. CAG:1138 | 0 | Not detected | -0.047 | -0.314 | 0.221 | 0.733 |
| Faecalibacterium sp. CAG:82-related_59 | 0 | Not detected | 0.053 | -0.133 | 0.239 | 0.577 |
| Faecalibaculum rodentium | 42 | Not detected | 0.268 | 0.071 | 0.466 | 0.008 |
| Faecalicoccus pleomorphus | 0 | Not detected | 0.013 | -0.087 | 0.114 | 0.795 |
| Faecalitalea cylindroides | 0 | Not detected | -0.006 | -0.072 | 0.061 | 0.867 |
| Fenollaria massiliensis | 0 | Not detected | 0.065 | -0.157 | 0.287 | 0.566 |
| Fermentimonas caenicola | 0 | Not detected | 0.024 | -0.164 | 0.212 | 0.803 |
| Fibrobacter sp. UWB12 | 0 | Not detected | 0.028 | -0.083 | 0.139 | 0.622 |
| Fibrobacter sp. UWCM | 0 | Not detected | 0.086 | -0.066 | 0.237 | 0.269 |
| Fibrobacter sp. UWOV1 | 0 | Not detected | -0.027 | -0.133 | 0.078 | 0.613 |
| Fibrobacterales bacterium Fibro_02 | 0 | Not detected | -0.060 | -0.175 | 0.056 | 0.311 |
| Fibrobacteria bacterium AD80 | 0 | Not detected | -0.011 | -0.124 | 0.102 | 0.851 |
| Filifactor alocis | 401 | Not detected | -0.302 | -0.498 | -0.105 | 0.003 |
| Finegoldia magna | 0 | Not detected | -0.138 | -0.336 | 0.061 | 0.174 |
| Firmicutes bacterium ASF500 | 0 | Not detected | 0.019 | -0.054 | 0.091 | 0.615 |
| Firmicutes bacterium CAG:102 | 0 | Not detected | -0.214 | -0.477 | 0.049 | 0.110 |
| Firmicutes bacterium CAG:137 | 0 | Not detected | 0.019 | -0.288 | 0.327 | 0.901 |
| Firmicutes bacterium CAG:137_57_8 | 0 | Not detected | -0.026 | -0.254 | 0.202 | 0.821 |
| Firmicutes bacterium CAG:145 | 0 | Not detected | 0.092 | -0.180 | 0.364 | 0.508 |
| Firmicutes bacterium CAG:176 | 0 | Not detected | 0.238 | -0.106 | 0.582 | 0.175 |
| Firmicutes bacterium CAG:176_59_8 | 550 | Not detected | 0.319 | 0.014 | 0.624 | 0.040 |
| Firmicutes bacterium CAG:227 | 0 | Not detected | 0.048 | -0.174 | 0.269 | 0.674 |
| Firmicutes bacterium CAG:238 | 0 | Not detected | 0.151 | -0.109 | 0.412 | 0.255 |
| Firmicutes bacterium CAG:24 | 0 | Not detected | 0.030 | -0.153 | 0.213 | 0.749 |
| Firmicutes bacterium CAG:240 | 0 | Not detected | 0.165 | -0.129 | 0.459 | 0.271 |
| Firmicutes bacterium CAG:270 | 0 | Not detected | -0.086 | -0.302 | 0.131 | 0.437 |
| Firmicutes bacterium CAG:272 | 0 | Not detected | 0.026 | -0.250 | 0.301 | 0.855 |
| Firmicutes bacterium CAG:272_52_7 | 0 | Not detected | 0.079 | -0.125 | 0.284 | 0.446 |
| Firmicutes bacterium CAG:308 | 0 | Not detected | 0.033 | -0.148 | 0.215 | 0.719 |
| Firmicutes bacterium CAG:313 | 0 | Not detected | 0.273 | -0.098 | 0.644 | 0.149 |
| Firmicutes bacterium CAG:41 | 0 | Not detected | -0.110 | -0.343 | 0.124 | 0.358 |
| Firmicutes bacterium CAG:466 | 0 | Not detected | -0.090 | -0.281 | 0.101 | 0.356 |
| Firmicutes bacterium CAG:475 | 0 | Not detected | 0.147 | -0.125 | 0.419 | 0.289 |
| Firmicutes bacterium CAG:536 | 1 | Not detected | 0.257 | 0.055 | 0.460 | 0.013 |
| Firmicutes bacterium CAG:552 | 0 | Not detected | -0.069 | -0.325 | 0.186 | 0.594 |
| Firmicutes bacterium CAG:555 | 0 | Not detected | 0.083 | -0.159 | 0.325 | 0.501 |
| Firmicutes bacterium CAG:65 | 0 | Not detected | 0.104 | -0.106 | 0.314 | 0.331 |
| Firmicutes bacterium CAG:791 | 443 | Not detected | 0.177 | 0.043 | 0.310 | 0.010 |
| Firmicutes bacterium CAG:83 | 0 | Not detected | -0.025 | -0.247 | 0.197 | 0.825 |

| Firmicutes bacterium CAG:882 | 0 | Not detected | -0.027 | -0.189 | 0.136 | 0.748 |
| --- | --- | --- | --- | --- | --- | --- |
| Firmicutes bacterium CAG:884 | 0 | Not detected | 0.062 | -0.187 | 0.311 | 0.626 |
| Firmicutes bacterium CAG:94 | 0 | Not detected | 0.002 | -0.249 | 0.252 | 0.991 |
| Firmicutes bacterium M10-2 | 0 | Not detected | 0.103 | -0.098 | 0.303 | 0.315 |
| Firmicutes bacterium ZOR0006 | 0 | Not detected | 0.203 | -0.012 | 0.417 | 0.064 |
| Flavonifractor plautii | 0 | Not detected | -0.143 | -0.305 | 0.018 | 0.082 |
| Fournierella massiliensis | 0 | Not detected | -0.002 | -0.081 | 0.077 | 0.953 |
| Franconibacter pulveris | 0 | Not detected | -0.046 | -0.269 | 0.176 | 0.683 |
| Fretibacterium fastidiosum | 0 | Not detected | 0.076 | -0.070 | 0.222 | 0.305 |
| Fusicatenibacter saccharivorans | 0 | Not detected | -0.065 | -0.157 | 0.027 | 0.168 |
| Fusobacterium mortiferum | 0 | Not detected | -0.041 | -0.323 | 0.241 | 0.775 |
| Fusobacterium necrophorum | 0 | Not detected | 0.028 | -0.160 | 0.216 | 0.769 |
| Fusobacterium nucleatum | 0 | Not detected | -0.152 | -0.423 | 0.119 | 0.271 |
| Fusobacterium periodonticum | 0 | Not detected | -0.189 | -0.422 | 0.043 | 0.111 |
| Gabonia massiliensis | 0 | Not detected | -0.163 | -0.311 | -0.014 | 0.032 |
| Gabonibacter massiliensis | 0 | Not detected | -0.101 | -0.277 | 0.075 | 0.260 |
| Gallibacterium anatis | 0 | Not detected | -0.008 | -0.144 | 0.128 | 0.910 |
| Gardnerella vaginalis | 1 | Not detected | 0.304 | 0.094 | 0.515 | 0.005 |
| Gemella haemolysans | 0 | Not detected | 0.077 | -0.117 | 0.270 | 0.437 |
| Gemella sanguinis | 0 | Not detected | -0.179 | -0.417 | 0.060 | 0.142 |
| Gemmata obscuriglobus | 0 | Not detected | 0.002 | -0.166 | 0.170 | 0.983 |
| Geofilum rubicundum | 0 | Not detected | -0.148 | -0.306 | 0.010 | 0.067 |
| Gilliamella apicola | 0 | Not detected | 0.010 | -0.152 | 0.171 | 0.907 |
| Globicatella sanguinis | 0 | Not detected | 0.050 | -0.136 | 0.236 | 0.599 |
| Gordonibacter pamelaeae | 0 | Not detected | -0.142 | -0.281 | -0.004 | 0.044 |
| Granulicatella adiacens | 0 | Not detected | 0.008 | -0.223 | 0.238 | 0.949 |
| Granulicatella elegans | 0 | Not detected | 0.134 | -0.059 | 0.327 | 0.173 |
| Haemophilus influenzae | 0 | Not detected | -0.072 | -0.335 | 0.190 | 0.590 |
| Haemophilus parainfluenzae | 0 | Not detected | -0.089 | -0.408 | 0.231 | 0.587 |
| Haemophilus sputorum | 0 | Not detected | -0.263 | -0.534 | 0.008 | 0.057 |
| Hafnia sp. HMSC23F03 | 0 | Not detected | -0.042 | -0.240 | 0.155 | 0.675 |
| Hathewaya proteolytica | 0 | Not detected | 0.173 | -0.016 | 0.362 | 0.073 |
| Heliobacterium sp. N15.MGS-306 | 0 | Not detected | 0.010 | -0.074 | 0.095 | 0.808 |
| Hespellia stercorisuis | 0 | Not detected | 0.048 | -0.027 | 0.124 | 0.212 |
| Holdemanella biformis | 744 | 0.6 | 0.193 | 0.047 | 0.339 | 0.009 |
| Holdemania filiformis | 0 | Not detected | -0.044 | -0.131 | 0.044 | 0.329 |
| Holdemania massiliensis | 0 | Not detected | -0.052 | -0.172 | 0.068 | 0.394 |
| Holdemania sp. Marseille-P2844 | 0 | Not detected | -0.104 | -0.207 | -0.001 | 0.048 |
| Hungatella hathewayi | 0 | Not detected | 0.014 | -0.081 | 0.109 | 0.776 |
| Hydrogenoanaerobacterium saccharovor | 0 | Not detected | 0.023 | -0.098 | 0.143 | 0.715 |
| Idiomarina zobellii | 0 | Not detected | 0.200 | -0.031 | 0.431 | 0.090 |
| Ileibacterium valens | 1 | Not detected | 0.290 | 0.081 | 0.498 | 0.006 |
| Intestinibacter bartlettii | 0 | Not detected | 0.055 | -0.130 | 0.240 | 0.558 |
| Intestinimonas butyriciproducens | 0 | Not detected | -0.002 | -0.113 | 0.109 | 0.977 |

| Intestinimonas massiliensis | 0 | Not detected | -0.046 | -0.149 | 0.056 | 0.377 |
| --- | --- | --- | --- | --- | --- | --- |
| Izhakiella sp. D4N98 | 0 | Not detected | 0.049 | -0.127 | 0.226 | 0.584 |
| Jeotgalibaca sp. PTS2502 | 0 | Not detected | 0.165 | 0.013 | 0.318 | 0.034 |
| Johnsonella ignava | 0 | Not detected | 0.030 | -0.064 | 0.123 | 0.532 |
| Kallipyga massiliensis | 0 | Not detected | -0.038 | -0.224 | 0.147 | 0.687 |
| Kandleria vitulina | 0 | Not detected | 0.257 | -0.012 | 0.526 | 0.061 |
| Kiloniella litopenaei | 0 | Not detected | -0.029 | -0.216 | 0.158 | 0.761 |
| Kingella kingae | 0 | Not detected | -0.056 | -0.231 | 0.120 | 0.534 |
| Klebsiella oxytoca | 0 | Not detected | -0.013 | -0.331 | 0.305 | 0.935 |
| Klebsiella pneumoniae | 0 | Not detected | 0.005 | -0.321 | 0.331 | 0.974 |
| Klebsiella sp. RIT-PI-d | 0 | Not detected | 0.038 | -0.199 | 0.276 | 0.753 |
| Kluyvera ascorbata | 0 | Not detected | 0.022 | -0.271 | 0.315 | 0.882 |
| Kluyvera cryocrescens | 0 | Not detected | -0.001 | -0.302 | 0.301 | 0.996 |
| Kluyvera intermedia | 0 | Not detected | -0.022 | -0.298 | 0.253 | 0.874 |
| Kocuria sp. ZOR0020 | 0 | Not detected | -0.022 | -0.176 | 0.131 | 0.778 |
| Kosakonia sacchari | 0 | Not detected | 0.064 | -0.188 | 0.316 | 0.621 |
| Lachnoanaerobaculum saburreum | 0 | Not detected | -0.109 | -0.203 | -0.015 | 0.023 |
| Lachnoanaerobaculum sp. OBRC5-5 | 0 | Not detected | 0.041 | -0.051 | 0.132 | 0.383 |
| Lachnobacterium bovis | 0 | Not detected | 0.007 | -0.085 | 0.098 | 0.887 |
| Lachnoclostridium phytofermentans | 0 | Not detected | -0.051 | -0.121 | 0.019 | 0.155 |
| Lachnospira multipara | 0 | Not detected | -0.116 | -0.241 | 0.010 | 0.071 |
| Lachnospiraceae bacterium 10-1 | 0 | Not detected | 0.018 | -0.042 | 0.077 | 0.561 |
| Lachnospiraceae bacterium 28-4 | 0 | Not detected | -0.019 | -0.079 | 0.042 | 0.543 |
| Lachnospiraceae bacterium 2_1_46FAA | 0 | Not detected | -0.050 | -0.139 | 0.039 | 0.267 |
| Lachnospiraceae bacterium 3-1 | 0 | Not detected | -0.002 | -0.072 | 0.069 | 0.966 |
| Lachnospiraceae bacterium 3-2 | 0 | Not detected | -0.014 | -0.082 | 0.054 | 0.692 |
| Lachnospiraceae bacterium A2 | 0 | Not detected | -0.029 | -0.103 | 0.046 | 0.452 |
| Lachnospiraceae bacterium A4 | 0 | Not detected | -0.021 | -0.087 | 0.044 | 0.524 |
| Lachnospiraceae bacterium AB2028 | 0 | Not detected | 0.090 | -0.110 | 0.290 | 0.378 |
| Lachnospiraceae bacterium AC2012 | 0 | Not detected | 0.031 | -0.051 | 0.113 | 0.457 |
| Lachnospiraceae bacterium AC2014 | 0 | Not detected | 0.067 | -0.057 | 0.192 | 0.290 |
| Lachnospiraceae bacterium AC2028 | 0 | Not detected | -0.020 | -0.158 | 0.118 | 0.778 |
| Lachnospiraceae bacterium AC2029 | 0 | Not detected | 0.009 | -0.079 | 0.096 | 0.846 |
| Lachnospiraceae bacterium AC2031 | 0 | Not detected | 0.067 | -0.059 | 0.193 | 0.298 |
| Lachnospiraceae bacterium AC3007 | 0 | Not detected | 0.049 | -0.115 | 0.212 | 0.560 |
| Lachnospiraceae bacterium AD3010 | 0 | Not detected | 0.140 | -0.048 | 0.329 | 0.145 |
| Lachnospiraceae bacterium C6A11 | 0 | Not detected | -0.011 | -0.132 | 0.109 | 0.856 |
| Lachnospiraceae bacterium COE1 | 0 | Not detected | 0.050 | -0.043 | 0.143 | 0.294 |
| Lachnospiraceae bacterium FD2005 | 0 | Not detected | 0.048 | -0.111 | 0.208 | 0.553 |
| Lachnospiraceae bacterium FE2018 | 0 | Not detected | 0.029 | -0.161 | 0.218 | 0.767 |
| Lachnospiraceae bacterium G41 | 0 | Not detected | -0.009 | -0.177 | 0.159 | 0.919 |
| Lachnospiraceae bacterium KHCPX20 | 0 | Not detected | 0.013 | -0.175 | 0.201 | 0.893 |
| Lachnospiraceae bacterium M18-1 | 0 | Not detected | 0.028 | -0.041 | 0.098 | 0.426 |
| Lachnospiraceae bacterium NC2004 | 0 | Not detected | 0.075 | -0.102 | 0.251 | 0.408 |

| Lachnospiraceae bacterium NC2008 | 0 | Not detected | 0.000 | -0.163 | 0.162 | 0.998 |
| --- | --- | --- | --- | --- | --- | --- |
| Lachnospiraceae bacterium ND2006 | 0 | Not detected | -0.063 | -0.233 | 0.107 | 0.468 |
| Lachnospiraceae bacterium NK3A20 | 0 | Not detected | 0.050 | -0.139 | 0.240 | 0.603 |
| Lachnospiraceae bacterium NK4A179 | 0 | Not detected | 0.065 | -0.106 | 0.235 | 0.458 |
| Lachnospiraceae bacterium NLAE-zl-G2 | 0 | Not detected | -0.144 | -0.327 | 0.040 | 0.125 |
| Lachnospiraceae bacterium P6B14 | 0 | Not detected | -0.028 | -0.163 | 0.108 | 0.689 |
| Lachnospiraceae bacterium TF01-11 | 0 | Not detected | 0.004 | -0.090 | 0.097 | 0.938 |
| Lachnospiraceae bacterium TWA4 | 0 | Not detected | -0.029 | -0.124 | 0.065 | 0.544 |
| Lachnospiraceae bacterium V9D3004 | 0 | Not detected | 0.160 | -0.009 | 0.328 | 0.064 |
| Lachnospiraceae bacterium YSB2008 | 0 | Not detected | 0.022 | -0.098 | 0.142 | 0.715 |
| Lachnospiraceae bacterium mt14 | 0 | Not detected | 0.084 | -0.074 | 0.242 | 0.295 |
| Lachnospiraceae bacterium oral taxon 50 | 0 | Not detected | 0.000 | -0.074 | 0.073 | 0.998 |
| Lactobacillus acidophilus | 0 | Not detected | 0.009 | -0.128 | 0.146 | 0.896 |
| Lactobacillus amylotrophicus | 0 | Not detected | -0.013 | -0.189 | 0.162 | 0.882 |
| Lactobacillus animalis | 0 | Not detected | -0.068 | -0.241 | 0.105 | 0.441 |
| Lactobacillus antri | 0 | Not detected | 0.181 | -0.012 | 0.375 | 0.067 |
| Lactobacillus aviarius | 0 | Not detected | 0.018 | -0.078 | 0.114 | 0.714 |
| Lactobacillus casei | 0 | Not detected | -0.081 | -0.330 | 0.169 | 0.526 |
| Lactobacillus curvatus | 0 | Not detected | 0.095 | -0.098 | 0.288 | 0.336 |
| Lactobacillus delbrueckii | 0 | Not detected | -0.091 | -0.308 | 0.126 | 0.412 |
| Lactobacillus equi | 0 | Not detected | 0.056 | -0.132 | 0.245 | 0.560 |
| Lactobacillus fermentum | 0 | Not detected | 0.079 | -0.202 | 0.361 | 0.581 |
| Lactobacillus gasseri | 0 | Not detected | 0.298 | 0.016 | 0.581 | 0.038 |
| Lactobacillus helveticus | 0 | Not detected | 0.138 | -0.068 | 0.344 | 0.190 |
| Lactobacillus ingluviei | 0 | Not detected | 0.049 | -0.114 | 0.211 | 0.557 |
| Lactobacillus jensenii | 0 | Not detected | 0.043 | -0.130 | 0.215 | 0.627 |
| Lactobacillus kefiranofaciens | 0 | Not detected | 0.061 | -0.099 | 0.221 | 0.455 |
| Lactobacillus manihotivorans | 0 | Not detected | 0.023 | -0.156 | 0.202 | 0.799 |
| Lactobacillus mucosae | 0 | Not detected | -0.112 | -0.304 | 0.079 | 0.249 |
| Lactobacillus paracasei | 0 | Not detected | -0.015 | -0.271 | 0.241 | 0.911 |
| Lactobacillus pasteurii | 0 | Not detected | 0.030 | -0.119 | 0.178 | 0.696 |
| Lactobacillus plantarum | 0 | Not detected | -0.041 | -0.256 | 0.174 | 0.708 |
| Lactobacillus rhamnosus | 0 | Not detected | -0.017 | -0.247 | 0.213 | 0.884 |
| Lactobacillus ruminis | 0 | Not detected | -0.041 | -0.231 | 0.149 | 0.670 |
| Lactobacillus ruminis CAG:367 | 0 | Not detected | -0.042 | -0.230 | 0.147 | 0.664 |
| Lactobacillus sakei | 0 | Not detected | 0.066 | -0.124 | 0.256 | 0.495 |
| Lactobacillus salivarius | 0 | Not detected | 0.129 | -0.142 | 0.400 | 0.352 |
| Lactobacillus vaginalis | 0 | Not detected | 0.024 | -0.216 | 0.264 | 0.846 |
| Lactococcus garvieae | 0 | Not detected | 0.075 | -0.119 | 0.269 | 0.448 |
| Lactococcus lactis | 0 | Not detected | -0.082 | -0.383 | 0.218 | 0.592 |
| Lactococcus piscium | 0 | Not detected | -0.134 | -0.328 | 0.061 | 0.179 |
| Lactonifactor longoviformis | 0 | Not detected | -0.019 | -0.081 | 0.044 | 0.561 |
| Lamprocystis purpurea | 0 | Not detected | 0.060 | -0.179 | 0.299 | 0.622 |
| Lascolabacillus massiliensis | 0 | Not detected | 0.117 | -0.041 | 0.275 | 0.146 |

| Lautropia mirabilis | 0 | Not detected | 0.061 | -0.138 | 0.260 | 0.546 |
| --- | --- | --- | --- | --- | --- | --- |
| Lawsonia intracellularis | 0 | Not detected | 0.061 | -0.129 | 0.252 | 0.528 |
| Leclercia adecarboxylata | 0 | Not detected | -0.049 | -0.331 | 0.233 | 0.733 |
| Lelliottia amnigena | 0 | Not detected | 0.018 | -0.289 | 0.325 | 0.910 |
| Lentimicrobium saccharophilum | 0 | Not detected | -0.022 | -0.227 | 0.184 | 0.837 |
| Leuconostoc citreum | 0 | Not detected | -0.016 | -0.191 | 0.160 | 0.862 |
| Leuconostoc lactis | 0 | Not detected | -0.091 | -0.302 | 0.121 | 0.402 |
| Leuconostoc mesenteroides | 0 | Not detected | -0.020 | -0.246 | 0.205 | 0.859 |
| Levyella massiliensis | 0 | Not detected | 0.014 | -0.085 | 0.113 | 0.785 |
| Libanicoccus massiliensis | 0 | Not detected | -0.021 | -0.199 | 0.157 | 0.820 |
| Luteimonas sp. FCS-9 | 0 | Not detected | 0.028 | -0.117 | 0.173 | 0.704 |
| Lutispora thermophila | 0 | Not detected | 0.096 | -0.073 | 0.266 | 0.265 |
| Macellibacteroides sp. HH-ZS | 0 | Not detected | -0.107 | -0.241 | 0.028 | 0.121 |
| Macrococcus caseolyticus | 0 | Not detected | 0.016 | -0.174 | 0.206 | 0.867 |
| Mageeibacillus indolicus | 1 | Not detected | 0.181 | 0.042 | 0.320 | 0.011 |
| Mangrovibacter sp. MFB070 | 0 | Not detected | -0.020 | -0.244 | 0.204 | 0.859 |
| Mannheimia haemolytica | 0 | Not detected | 0.012 | -0.172 | 0.196 | 0.897 |
| Marvinbryantia formatexigens | 0 | Not detected | -0.026 | -0.115 | 0.063 | 0.573 |
| Massilioclostridium coli | 0 | Not detected | -0.042 | -0.131 | 0.047 | 0.355 |
| Massiliomicrobiota timonensis | 0 | Not detected | 0.054 | -0.143 | 0.251 | 0.591 |
| Mediterranea massiliensis | 0 | Not detected | 0.019 | -0.095 | 0.134 | 0.744 |
| Megamonas funiformis | 0 | Not detected | -0.054 | -0.400 | 0.292 | 0.759 |
| Megasphaera cerevisiae | 0 | Not detected | -0.021 | -0.274 | 0.232 | 0.872 |
| Megasphaera elsdenii | 0 | Not detected | 0.095 | -0.237 | 0.426 | 0.575 |
| Megasphaera genomosp. type_1 | 0 | Not detected | 0.032 | -0.034 | 0.098 | 0.344 |
| Megasphaera massiliensis | 0 | Not detected | 0.204 | -0.090 | 0.497 | 0.175 |
| Megasphaera micronuciformis | 0 | Not detected | 0.179 | -0.051 | 0.409 | 0.127 |
| Megasphaera sp. BV3C16-1 | 0 | Not detected | 0.112 | -0.082 | 0.305 | 0.258 |
| Megasphaera sp. UPII 135-E | 0 | Not detected | 0.024 | -0.042 | 0.090 | 0.479 |
| Merdibacter massiliensis | 0 | Not detected | -0.013 | -0.089 | 0.063 | 0.741 |
| Methanobrevibacter smithii | 0 | Not detected | 0.283 | -0.121 | 0.686 | 0.169 |
| Methanobrevibacter smithii CAG:186 | 0 | Not detected | 0.222 | -0.154 | 0.598 | 0.247 |
| Methanocorpusculaceae archaeon Phil4 | 0 | Not detected | 0.221 | -0.041 | 0.484 | 0.099 |
| Methanosarcina mazei | 0 | Not detected | 0.026 | -0.201 | 0.253 | 0.823 |
| Methylobacterium sp. ZNC0032 | 0 | Not detected | -0.093 | -0.250 | 0.064 | 0.248 |
| Methyloglobulus morosus | 0 | Not detected | 0.067 | -0.122 | 0.257 | 0.487 |
| Microcystis aeruginosa | 0 | Not detected | -0.004 | -0.200 | 0.192 | 0.967 |
| Mitsuokella multacida | 0 | Not detected | 0.164 | -0.179 | 0.507 | 0.349 |
| Mobilibacterium timonense | 0 | Not detected | -0.065 | -0.249 | 0.118 | 0.484 |
| Mobiluncus curtisii | 0 | Not detected | -0.015 | -0.091 | 0.061 | 0.697 |
| Mobiluncus mulieris | 0 | Not detected | -0.027 | -0.113 | 0.059 | 0.539 |
| Mogibacterium timidum | 0 | Not detected | -0.005 | -0.220 | 0.209 | 0.960 |
| Moraxella porci | 0 | Not detected | 0.022 | -0.135 | 0.179 | 0.781 |
| Morganella morganii | 0 | Not detected | -0.034 | -0.242 | 0.174 | 0.751 |

| Morococcus cerebrosus | 0 | Not detected | -0.005 | -0.178 | 0.169 | 0.959 |
| --- | --- | --- | --- | --- | --- | --- |
| Mucinivorans hirudinis | 0 | Not detected | 0.063 | -0.085 | 0.211 | 0.405 |
| Murdochiella sp. Marseille-P2341 | 0 | Not detected | 0.000 | -0.103 | 0.103 | 1.000 |
| Mycoplasma sp. CAG:877 | 0 | Not detected | 0.264 | -0.009 | 0.537 | 0.058 |
| Mycoplasma sp. CAG:956 | 0 | Not detected | 0.205 | -0.038 | 0.447 | 0.098 |
| Myroides injenensis | 0 | Not detected | -0.068 | -0.297 | 0.161 | 0.559 |
| Myroides odoratimimus | 0 | Not detected | -0.106 | -0.344 | 0.132 | 0.383 |
| Necropsobacter massiliensis | 0 | Not detected | -0.159 | -0.375 | 0.056 | 0.148 |
| Negativicoccus massiliensis | 0 | Not detected | -0.067 | -0.232 | 0.099 | 0.429 |
| Neglecta timonensis | 0 | Not detected | -0.017 | -0.133 | 0.099 | 0.773 |
| Neisseria cinerea | 0 | Not detected | -0.119 | -0.302 | 0.065 | 0.205 |
| Neisseria mucosa | 0 | Not detected | -0.194 | -0.386 | -0.002 | 0.047 |
| Nissabacter archeti | 0 | Not detected | -0.006 | -0.225 | 0.214 | 0.960 |
| Novibacillus thermophilus | 0 | Not detected | 0.070 | -0.121 | 0.262 | 0.471 |
| Obesumbacterium proteus | 0 | Not detected | 0.007 | -0.250 | 0.264 | 0.958 |
| Oceanospirillum linum | 0 | Not detected | 0.043 | -0.142 | 0.227 | 0.649 |
| Odoribacter laneus | 0 | Not detected | -0.146 | -0.317 | 0.025 | 0.094 |
| Odoribacter laneus CAG:561 | 0 | Not detected | -0.148 | -0.381 | 0.084 | 0.211 |
| Odoribacter sp. 43_10 | 0 | Not detected | -0.070 | -0.210 | 0.070 | 0.326 |
| Odoribacter sp. CAG:788 | 0 | Not detected | -0.055 | -0.283 | 0.173 | 0.635 |
| Odoribacter splanchnicus | 0 | Not detected | -0.063 | -0.167 | 0.042 | 0.240 |
| Odoribacter splanchnicus CAG:14 | 0 | Not detected | -0.013 | -0.167 | 0.141 | 0.865 |
| Oligella ureolytica | 0 | Not detected | 0.137 | -0.045 | 0.319 | 0.141 |
| Olsenella scatoligenes | 0 | Not detected | -0.096 | -0.300 | 0.108 | 0.357 |
| Olsenella sp. Marseille-P3197 | 0 | Not detected | 0.248 | 0.052 | 0.444 | 0.013 |
| Olsenella sp. oral taxon 809 | 0 | Not detected | -0.005 | -0.140 | 0.131 | 0.944 |
| Olsenella uli | 0 | Not detected | 0.098 | -0.099 | 0.296 | 0.330 |
| Olsenella umbonata | 0 | Not detected | -0.004 | -0.163 | 0.155 | 0.958 |
| Oribacterium parvum | 0 | Not detected | 0.014 | -0.052 | 0.079 | 0.688 |
| Oribacterium sinus | 0 | Not detected | 0.007 | -0.143 | 0.157 | 0.928 |
| Oribacterium sp. WCC10 | 0 | Not detected | 0.062 | -0.141 | 0.266 | 0.550 |
| Oribacterium sp. oral taxon 078 | 0 | Not detected | 0.007 | -0.095 | 0.109 | 0.888 |
| Oscillibacter ruminantium | 0 | Not detected | 0.018 | -0.125 | 0.161 | 0.806 |
| Oscillibacter sp. 1-3 | 0 | Not detected | 0.042 | -0.037 | 0.122 | 0.298 |
| Oscillibacter sp. 57_20 | 0 | Not detected | -0.042 | -0.295 | 0.211 | 0.745 |
| Oscillibacter sp. ER4 | 0 | Not detected | 0.100 | -0.051 | 0.250 | 0.193 |
| Oscillibacter sp. KLE 1745 | 0 | Not detected | -0.089 | -0.220 | 0.042 | 0.183 |
| Oscillibacter valericigenes | 0 | Not detected | 0.018 | -0.114 | 0.150 | 0.792 |
| Oscillospiraceae bacterium Zagget12 | 0 | Not detected | 0.001 | -0.155 | 0.156 | 0.992 |
| Oxalobacter formigenes | 0 | Not detected | 0.048 | -0.116 | 0.213 | 0.564 |
| Paeniclostridium sordellii | 0 | Not detected | 0.097 | -0.118 | 0.312 | 0.376 |
| Paludibacter propionicigenes | 3 | Not detected | -0.139 | -0.281 | 0.003 | 0.055 |
| Pantoea agglomerans | 0 | Not detected | -0.052 | -0.234 | 0.130 | 0.577 |
| Pantoea ananatis | 0 | Not detected | 0.006 | -0.161 | 0.173 | 0.946 |

| Pantoea dispersa | 0 | Not detected | 0.022 | -0.138 | 0.181 | 0.791 |
| --- | --- | --- | --- | --- | --- | --- |
| Pantoea rwandensis | 0 | Not detected | -0.004 | -0.178 | 0.170 | 0.964 |
| Pantoea septica | 0 | Not detected | 0.013 | -0.141 | 0.167 | 0.871 |
| Pantoea sp. IMH | 0 | Not detected | -0.065 | -0.245 | 0.115 | 0.481 |
| Pantoea sp. PSNIH2 | 0 | Not detected | -0.076 | -0.350 | 0.198 | 0.586 |
| Parabacteroides distasonis | 0 | Not detected | -0.145 | -0.293 | 0.002 | 0.054 |
| Parabacteroides goldsteinii | 0 | Not detected | 0.029 | -0.135 | 0.192 | 0.732 |
| Parabacteroides gordonii | 0 | Not detected | -0.095 | -0.225 | 0.035 | 0.153 |
| Parabacteroides johnsonii | 0 | Not detected | -0.146 | -0.264 | -0.029 | 0.014 |
| Parabacteroides merdae CAG:48 | 0 | Not detected | -0.178 | -0.392 | 0.036 | 0.104 |
| Paraburkholderia sacchari | 0 | Not detected | -0.110 | -0.288 | 0.068 | 0.227 |
| Paraclostridium bifermentans | 0 | Not detected | 0.130 | -0.062 | 0.321 | 0.185 |
| Paraprevotella clara CAG:116 | 0 | Not detected | -0.202 | -0.467 | 0.063 | 0.135 |
| Paraprevotella xylaniphila | 0 | Not detected | 0.001 | -0.141 | 0.144 | 0.985 |
| Parascardovia denticolens | 0 | Not detected | 0.252 | 0.049 | 0.456 | 0.015 |
| Parasporobacterium paucivorans | 0 | Not detected | -0.037 | -0.152 | 0.078 | 0.526 |
| Parasutterella excrementihominis | 0 | Not detected | -0.020 | -0.277 | 0.237 | 0.878 |
| Parasutterella excrementihominis CAG:2 | 0 | Not detected | 0.088 | -0.305 | 0.481 | 0.662 |
| Parvimonas micra | 0 | Not detected | -0.052 | -0.277 | 0.172 | 0.648 |
| Pasteurella bettyae | 2 | Not detected | -0.128 | -0.309 | 0.053 | 0.165 |
| Pectobacterium carotovorum | 0 | Not detected | -0.045 | -0.213 | 0.123 | 0.599 |
| Pedobacter glucosidilyticus | 0 | Not detected | -0.031 | -0.205 | 0.143 | 0.728 |
| Pelosinus fermentans | 2 | Not detected | -0.138 | -0.298 | 0.022 | 0.091 |
| Peptoanaerobacter stomatis | 0 | Not detected | -0.093 | -0.262 | 0.076 | 0.283 |
| Peptococcus niger | 0 | Not detected | -0.024 | -0.184 | 0.136 | 0.767 |
| Peptoniphilus duerdenii | 0 | Not detected | 0.001 | -0.136 | 0.137 | 0.991 |
| Peptoniphilus grossensis | 0 | Not detected | -0.121 | -0.297 | 0.056 | 0.181 |
| Peptoniphilus harei | 2 | Not detected | -0.113 | -0.315 | 0.088 | 0.270 |
| Peptoniphilus lacrimalis | 0 | Not detected | 0.026 | -0.192 | 0.244 | 0.813 |
| Peptoniphilus obesi | 0 | Not detected | -0.156 | -0.344 | 0.033 | 0.105 |
| Peptoniphilus rhinitidis | 0 | Not detected | 0.040 | -0.148 | 0.227 | 0.679 |
| Peptoniphilus senegalensis | 0 | Not detected | 0.014 | -0.188 | 0.217 | 0.889 |
| Peptoniphilus sp. BV3C26 | 0 | Not detected | 0.100 | -0.121 | 0.320 | 0.376 |
| Peptoniphilus sp. ING2-D1G | 0 | Not detected | -0.069 | -0.256 | 0.118 | 0.470 |
| Peptoniphilus sp. oral taxon 375 | 0 | Not detected | 0.009 | -0.083 | 0.100 | 0.853 |
| Peptostreptococcaceae bacterium VA2 | 0 | Not detected | 0.168 | -0.063 | 0.399 | 0.153 |
| Peptostreptococcus anaerobius | 0 | Not detected | -0.023 | -0.095 | 0.049 | 0.526 |
| Peptostreptococcus anaerobius CAG:621 | 0 | Not detected | -0.083 | -0.243 | 0.078 | 0.313 |
| Peptostreptococcus stomatis | 0 | Not detected | 0.062 | -0.136 | 0.260 | 0.541 |
| Phaeodactylibacter xiamenensis | 0 | Not detected | -0.189 | -0.433 | 0.055 | 0.130 |
| Phascolarctobacterium sp. CAG:207 | 0 | Not detected | -0.357 | -0.732 | 0.018 | 0.062 |
| Phascolarctobacterium sp. CAG:266 | 0 | Not detected | -0.159 | -0.420 | 0.103 | 0.234 |
| Phascolarctobacterium succinatutens | 0 | Not detected | 0.061 | -0.133 | 0.254 | 0.540 |
| Phocaeicola abscessus | 0 | Not detected | -0.038 | -0.193 | 0.117 | 0.631 |

| Phocea massiliensis | 0 | Not detected | -0.085 | -0.184 | 0.015 | 0.095 |
| --- | --- | --- | --- | --- | --- | --- |
| Piscirickettsia salmonis | 0 | Not detected | -0.051 | -0.213 | 0.111 | 0.537 |
| Plautia stali symbiont | 0 | Not detected | -0.039 | -0.205 | 0.127 | 0.646 |
| Pluralibacter gergoviae | 0 | Not detected | -0.057 | -0.316 | 0.203 | 0.669 |
| Pontibacillus litoralis | 0 | Not detected | -0.196 | -0.430 | 0.038 | 0.101 |
| Porphyromonadaceae bacterium COT-18 | 0 | Not detected | -0.033 | -0.202 | 0.137 | 0.705 |
| Porphyromonadaceae bacterium KH3CP | 0 | Not detected | -0.046 | -0.214 | 0.122 | 0.590 |
| Porphyromonadaceae bacterium KH3R1 | 0 | Not detected | -0.082 | -0.240 | 0.076 | 0.309 |
| Porphyromonas asaccharolytica | 98 | Not detected | -0.211 | -0.420 | -0.002 | 0.048 |
| Porphyromonas bennonis | 0 | Not detected | -0.201 | -0.396 | -0.006 | 0.044 |
| Porphyromonas cangingivalis | 0 | Not detected | 0.059 | -0.126 | 0.243 | 0.535 |
| Porphyromonas canoris | 0 | Not detected | -0.056 | -0.239 | 0.127 | 0.548 |
| Porphyromonas catoniae | 0 | Not detected | -0.051 | -0.190 | 0.087 | 0.465 |
| Porphyromonas crevioricanis | 0 | Not detected | 0.025 | -0.102 | 0.152 | 0.699 |
| Porphyromonas endodontalis | 0 | Not detected | 0.048 | -0.157 | 0.253 | 0.647 |
| Porphyromonas gingivalis | 0 | Not detected | 0.033 | -0.137 | 0.203 | 0.703 |
| Porphyromonas gingivicanis | 0 | Not detected | -0.022 | -0.150 | 0.107 | 0.742 |
| Porphyromonas levii | 0 | Not detected | 0.006 | -0.234 | 0.246 | 0.959 |
| Porphyromonas macacae | 0 | Not detected | 0.051 | -0.127 | 0.229 | 0.575 |
| Porphyromonas somerae | 0 | Not detected | -0.076 | -0.164 | 0.012 | 0.089 |
| Porphyromonas sp. CAG:1061 | 0 | Not detected | -0.099 | -0.317 | 0.119 | 0.374 |
| Porphyromonas sp. COT-239 OH1446 | 0 | Not detected | -0.241 | -0.466 | -0.016 | 0.036 |
| Porphyromonas sp. COT-290 OH3588 | 0 | Not detected | 0.073 | -0.153 | 0.300 | 0.525 |
| Prevotella albensis | 0 | Not detected | 0.163 | -0.156 | 0.483 | 0.316 |
| Prevotella amnii | 2 | Not detected | -0.077 | -0.161 | 0.006 | 0.071 |
| Prevotella aurantiaca | 0 | Not detected | 0.100 | -0.109 | 0.308 | 0.349 |
| Prevotella baroniae | 0 | Not detected | 0.024 | -0.074 | 0.122 | 0.630 |
| Prevotella bergensis | 0 | Not detected | 0.028 | -0.131 | 0.187 | 0.729 |
| Prevotella bivia | 0 | Not detected | 0.042 | -0.067 | 0.151 | 0.447 |
| Prevotella bryantii | 0 | Not detected | 0.241 | -0.079 | 0.562 | 0.140 |
| Prevotella buccae | 0 | Not detected | 0.088 | -0.152 | 0.328 | 0.473 |
| Prevotella buccalis | 1 | Not detected | 0.064 | -0.025 | 0.154 | 0.159 |
| Prevotella copri | 0 | Not detected | 0.285 | -0.059 | 0.629 | 0.104 |
| Prevotella corporis | 0 | Not detected | -0.072 | -0.157 | 0.013 | 0.099 |
| Prevotella dentalis | 0 | Not detected | 0.162 | -0.084 | 0.409 | 0.197 |
| Prevotella dentasini | 0 | Not detected | 0.221 | -0.030 | 0.471 | 0.084 |
| Prevotella denticola | 389 | Not detected | 0.302 | 0.027 | 0.578 | 0.032 |
| Prevotella disiens | 0 | Not detected | -0.022 | -0.119 | 0.075 | 0.655 |
| Prevotella enoeca | 0 | Not detected | 0.119 | -0.122 | 0.360 | 0.334 |
| Prevotella fusca | 0 | Not detected | 0.211 | -0.063 | 0.485 | 0.131 |
| Prevotella histicola | 0 | Not detected | 0.021 | -0.149 | 0.190 | 0.811 |
| Prevotella intermedia | 0 | Not detected | 0.104 | -0.068 | 0.276 | 0.237 |
| Prevotella loescheii | 0 | Not detected | 0.208 | -0.020 | 0.436 | 0.074 |
| Prevotella maculosa | 0 | Not detected | 0.012 | -0.186 | 0.210 | 0.903 |

| Prevotella marshii | 0 | Not detected | -0.009 | -0.102 | 0.085 | 0.856 |
| --- | --- | --- | --- | --- | --- | --- |
| Prevotella melaninogenica | 0 | Not detected | 0.165 | -0.035 | 0.365 | 0.105 |
| Prevotella micans | 0 | Not detected | 0.207 | -0.021 | 0.434 | 0.075 |
| Prevotella multiformis | 0 | Not detected | 0.022 | -0.121 | 0.165 | 0.759 |
| Prevotella multisaccharivorax | 0 | Not detected | 0.239 | -0.075 | 0.553 | 0.136 |
| Prevotella nanceiensis | 0 | Not detected | -0.024 | -0.206 | 0.159 | 0.800 |
| Prevotella nigrescens | 0 | Not detected | 0.178 | -0.048 | 0.403 | 0.124 |
| Prevotella oralis | 0 | Not detected | 0.132 | -0.105 | 0.369 | 0.275 |
| Prevotella oris | 0 | Not detected | 0.194 | -0.072 | 0.460 | 0.153 |
| Prevotella oryzae | 0 | Not detected | 0.223 | -0.056 | 0.501 | 0.117 |
| Prevotella oulorum | 0 | Not detected | 0.160 | -0.130 | 0.450 | 0.280 |
| Prevotella pallens | 0 | Not detected | -0.038 | -0.143 | 0.066 | 0.474 |
| Prevotella paludivivens | 0 | Not detected | 0.125 | -0.064 | 0.315 | 0.195 |
| Prevotella ruminicola | 0 | Not detected | 0.218 | -0.049 | 0.485 | 0.109 |
| Prevotella saccharolytica | 0 | Not detected | 0.193 | -0.064 | 0.451 | 0.142 |
| Prevotella salivae | 0 | Not detected | -0.023 | -0.121 | 0.074 | 0.641 |
| Prevotella scopos | 0 | Not detected | 0.056 | -0.079 | 0.191 | 0.415 |
| Prevotella sp. 10(H) | 0 | Not detected | 0.123 | -0.039 | 0.284 | 0.137 |
| Prevotella sp. 109 | 0 | Not detected | 0.003 | -0.090 | 0.097 | 0.942 |
| Prevotella sp. AGR2160 | 0 | Not detected | 0.246 | -0.052 | 0.545 | 0.106 |
| Prevotella sp. BP1-148 | 0 | Not detected | 0.143 | 0.003 | 0.282 | 0.045 |
| Prevotella sp. CAG:1058 | 0 | Not detected | -0.146 | -0.392 | 0.100 | 0.245 |
| Prevotella sp. CAG:1092 | 0 | Not detected | 0.334 | 0.015 | 0.653 | 0.041 |
| Prevotella sp. CAG:1124 | 0 | Not detected | -0.013 | -0.265 | 0.239 | 0.919 |
| Prevotella sp. CAG:1185 | 0 | Not detected | -0.188 | -0.449 | 0.073 | 0.158 |
| Prevotella sp. CAG:1320 | 0 | Not detected | -0.024 | -0.228 | 0.179 | 0.814 |
| Prevotella sp. CAG:255 | 0 | Not detected | -0.027 | -0.187 | 0.133 | 0.744 |
| Prevotella sp. CAG:386 | 0 | Not detected | 0.345 | -0.013 | 0.703 | 0.059 |
| Prevotella sp. CAG:487 | 0 | Not detected | -0.031 | -0.198 | 0.136 | 0.714 |
| Prevotella sp. CAG:520 | 0 | Not detected | 0.179 | -0.114 | 0.472 | 0.232 |
| Prevotella sp. CAG:5226 | 0 | Not detected | 0.245 | -0.075 | 0.565 | 0.133 |
| Prevotella sp. CAG:604 | 0 | Not detected | 0.196 | -0.189 | 0.580 | 0.319 |
| Prevotella sp. CAG:732 | 0 | Not detected | 0.288 | -0.053 | 0.630 | 0.098 |
| Prevotella sp. CAG:891 | 0 | Not detected | 0.086 | -0.158 | 0.329 | 0.491 |
| Prevotella sp. FD3004 | 34 | Not detected | 0.330 | 0.118 | 0.543 | 0.002 |
| Prevotella sp. HUN102 | 0 | Not detected | 0.094 | -0.046 | 0.233 | 0.190 |
| Prevotella sp. MSX73 | 0 | Not detected | 0.072 | -0.169 | 0.312 | 0.560 |
| Prevotella sp. P4-76 | 0 | Not detected | 0.161 | -0.020 | 0.342 | 0.081 |
| Prevotella sp. P6B1 | 0 | Not detected | 0.211 | 0.065 | 0.357 | 0.005 |
| Prevotella sp. P6B4 | 9 | Not detected | 0.273 | 0.131 | 0.415 | 0.000 |
| Prevotella sp. RM4 | 0 | Not detected | 0.175 | -0.004 | 0.354 | 0.056 |
| Prevotella sp. S7 MS 2 | 0 | Not detected | 0.013 | -0.079 | 0.105 | 0.778 |
| Prevotella sp. S7-1-8 | 0 | Not detected | 0.036 | -0.051 | 0.122 | 0.420 |
| Prevotella sp. kh1p2 | 0 | Not detected | 0.226 | -0.014 | 0.466 | 0.065 |

| Prevotella sp. khp1 | 0 | Not detected | 0.172 | -0.012 | 0.356 | 0.068 |
| --- | --- | --- | --- | --- | --- | --- |
| Prevotella sp. ne3005 | 0 | Not detected | 0.147 | -0.015 | 0.309 | 0.076 |
| Prevotella sp. oral taxon 473 | 0 | Not detected | 0.071 | -0.170 | 0.312 | 0.564 |
| Prevotella sp. tf2-5 | 0 | Not detected | 0.213 | 0.051 | 0.375 | 0.010 |
| Prevotella stercorea | 0 | Not detected | 0.053 | -0.082 | 0.187 | 0.444 |
| Prevotella stercorea CAG:629 | 0 | Not detected | 0.198 | -0.106 | 0.502 | 0.202 |
| Prevotella timonensis | 0 | Not detected | -0.005 | -0.099 | 0.088 | 0.915 |
| Prevotellamassilia timonensis | 0 | Not detected | 0.146 | -0.013 | 0.305 | 0.072 |
| Propionibacterium acidifaciens | 553 | Not detected | 0.328 | 0.133 | 0.524 | 0.001 |
| Proteiniclasticum ruminis | 0 | Not detected | 0.044 | -0.121 | 0.210 | 0.599 |
| Proteiniphilum acetatigenes | 0 | Not detected | -0.025 | -0.198 | 0.147 | 0.772 |
| Proteiniphilum saccharofermentans | 0 | Not detected | -0.083 | -0.273 | 0.106 | 0.388 |
| Proteiniphilum sp. EBM-41 | 0 | Not detected | -0.022 | -0.256 | 0.211 | 0.851 |
| Proteobacteria bacterium CAG:139 | 0 | Not detected | 0.007 | -0.368 | 0.382 | 0.971 |
| Proteobacteria bacterium CAG:495 | 0 | Not detected | 0.043 | -0.277 | 0.363 | 0.793 |
| Providencia rettgeri | 0 | Not detected | -0.080 | -0.287 | 0.127 | 0.449 |
| Pseudobacteroides cellulosolvens | 3 | Not detected | -0.133 | -0.281 | 0.016 | 0.080 |
| Pseudobutyrivibrio ruminis | 0 | Not detected | 0.023 | -0.056 | 0.101 | 0.572 |
| Pseudobutyrivibrio sp. ACV-2 | 0 | Not detected | -0.014 | -0.187 | 0.160 | 0.877 |
| Pseudobutyrivibrio sp. JW11 | 0 | Not detected | -0.048 | -0.216 | 0.121 | 0.580 |
| Pseudobutyrivibrio sp. MD2005 | 0 | Not detected | 0.107 | -0.074 | 0.289 | 0.246 |
| Pseudobutyrivibrio sp. UC1225 | 0 | Not detected | 0.042 | -0.075 | 0.159 | 0.481 |
| Pseudobutyrivibrio sp. YE44 | 0 | Not detected | 0.072 | -0.097 | 0.241 | 0.403 |
| Pseudobutyrivibrio xylanivorans | 0 | Not detected | 0.051 | -0.122 | 0.224 | 0.562 |
| Pseudoflavonifractor capillosus | 0 | Not detected | -0.003 | -0.088 | 0.081 | 0.938 |
| Pseudopropionibacterium propionicum | 0 | Not detected | -0.005 | -0.202 | 0.193 | 0.964 |
| Pyramidobacter piscolens | 0 | Not detected | -0.124 | -0.380 | 0.131 | 0.340 |
| Rahnella aquatilis | 0 | Not detected | -0.043 | -0.209 | 0.122 | 0.607 |
| Ralstonia mannitolilytica | 0 | Not detected | 0.074 | -0.152 | 0.301 | 0.521 |
| Ralstonia pickettii | 0 | Not detected | -0.025 | -0.237 | 0.187 | 0.818 |
| Raoultella ornithinolytica | 0 | Not detected | -0.042 | -0.358 | 0.274 | 0.795 |
| Rappaport israeli | 0 | Not detected | -0.044 | -0.221 | 0.134 | 0.630 |
| Rhodococcus defluvii | 0 | Not detected | 0.022 | -0.140 | 0.185 | 0.787 |
| Rhodopseudomonas palustris | 0 | Not detected | -0.082 | -0.274 | 0.111 | 0.406 |
| Rhodospirillum sp. UNK.MGS-17 | 0 | Not detected | -0.062 | -0.427 | 0.304 | 0.741 |
| Riemerella columbina | 0 | Not detected | -0.054 | -0.139 | 0.031 | 0.217 |
| Rikenella microfusus | 0 | Not detected | -0.041 | -0.128 | 0.045 | 0.352 |
| Risungbinella massiliensis | 0 | Not detected | -0.087 | -0.233 | 0.058 | 0.239 |
| Robinsoniella peoriensis | 0 | Not detected | -0.040 | -0.105 | 0.024 | 0.219 |
| Rodentibacter pneumotropicus | 30 | Not detected | -0.152 | -0.308 | 0.005 | 0.057 |
| Roseburia faecis | 0 | Not detected | -0.012 | -0.153 | 0.129 | 0.868 |
| Roseburia hominis | 1 | Not detected | 0.071 | -0.020 | 0.161 | 0.129 |
| Roseburia intestinalis | 0 | Not detected | -0.054 | -0.191 | 0.084 | 0.445 |
| Roseburia inulinivorans | 0 | Not detected | -0.017 | -0.128 | 0.094 | 0.764 |

| Roseburia sp. CAG:197_41_10 | 0 | Not detected | -0.034 | -0.147 | 0.080 | 0.561 |
| --- | --- | --- | --- | --- | --- | --- |
| Roseburia sp. CAG:309 | 0 | Not detected | -0.232 | -0.476 | 0.012 | 0.063 |
| Roseburia sp. CAG:380 | 0 | Not detected | -0.035 | -0.266 | 0.197 | 0.768 |
| Rothia dentocariosa | 0 | Not detected | -0.101 | -0.342 | 0.140 | 0.411 |
| Rothia mucilaginosa | 0 | Not detected | -0.051 | -0.303 | 0.202 | 0.695 |
| Ruminiclostridium thermocellum | 0 | Not detected | 0.017 | -0.099 | 0.133 | 0.777 |
| Ruminococcaceae bacterium AB4001 | 0 | Not detected | 0.146 | -0.009 | 0.301 | 0.065 |
| Ruminococcaceae bacterium AE2021 | 7 | Not detected | 0.254 | 0.105 | 0.404 | 0.001 |
| Ruminococcaceae bacterium D16 | 0 | Not detected | 0.051 | -0.026 | 0.129 | 0.194 |
| Ruminococcaceae bacterium YRB3002 | 0 | Not detected | 0.019 | -0.163 | 0.201 | 0.836 |
| Ruminococcus albus | 0 | Not detected | 0.028 | -0.123 | 0.180 | 0.714 |
| Ruminococcus bicirculans | 0 | Not detected | 0.032 | -0.200 | 0.264 | 0.786 |
| Ruminococcus callidus | 0 | Not detected | 0.037 | -0.122 | 0.196 | 0.646 |
| Ruminococcus champanellensis | 0 | Not detected | 0.045 | -0.152 | 0.241 | 0.656 |
| Ruminococcus faecis | 0 | Not detected | 0.075 | -0.029 | 0.178 | 0.158 |
| Ruminococcus flavefaciens | 0 | Not detected | 0.011 | -0.130 | 0.151 | 0.883 |
| Ruminococcus gauvreauii | 0 | Not detected | -0.018 | -0.101 | 0.064 | 0.668 |
| Ruminococcus lactaris | 0 | Not detected | 0.078 | -0.041 | 0.196 | 0.198 |
| Ruminococcus obeum CAG:39 | 0 | Not detected | -0.050 | -0.186 | 0.087 | 0.476 |
| Ruminococcus sp. AT10 | 1 | Not detected | -0.111 | -0.188 | -0.034 | 0.005 |
| Ruminococcus sp. CAG:108-related_41_ | 0 | Not detected | -0.021 | -0.269 | 0.227 | 0.868 |
| Ruminococcus sp. CAG:177 | 0 | Not detected | 0.112 | -0.275 | 0.499 | 0.571 |
| Ruminococcus sp. CAG:254 | 0 | Not detected | -0.315 | -0.667 | 0.038 | 0.080 |
| Ruminococcus sp. CAG:330 | 0 | Not detected | 0.033 | -0.310 | 0.377 | 0.849 |
| Ruminococcus sp. CAG:379 | 0 | Not detected | -0.023 | -0.281 | 0.236 | 0.864 |
| Ruminococcus sp. CAG:382 | 0 | Not detected | 0.280 | -0.063 | 0.624 | 0.110 |
| Ruminococcus sp. CAG:403 | 0 | Not detected | -0.021 | -0.293 | 0.251 | 0.882 |
| Ruminococcus sp. CAG:563 | 0 | Not detected | 0.200 | -0.057 | 0.458 | 0.127 |
| Ruminococcus sp. CAG:60 | 0 | Not detected | 0.024 | -0.123 | 0.171 | 0.748 |
| Ruminococcus sp. CAG:624 | 0 | Not detected | 0.089 | -0.163 | 0.341 | 0.488 |
| Ruminococcus sp. CAG:724 | 0 | Not detected | 0.208 | -0.078 | 0.494 | 0.155 |
| Ruminococcus sp. FC2018 | 0 | Not detected | 0.044 | -0.120 | 0.207 | 0.600 |
| Ruminococcus sp. HUN007 | 0 | Not detected | 0.009 | -0.126 | 0.143 | 0.898 |
| Ruminococcus sp. NK3A76 | 0 | Not detected | 0.028 | -0.137 | 0.192 | 0.743 |
| Ruminococcus sp. Phil15 | 0 | Not detected | -0.064 | -0.281 | 0.152 | 0.559 |
| Ruminococcus sp. Zagget7 | 0 | Not detected | -0.014 | -0.143 | 0.115 | 0.831 |
| Ruthenibacterium lactatiformans | 0 | Not detected | -0.072 | -0.182 | 0.039 | 0.204 |
| Salmonella enterica | 0 | Not detected | 0.009 | -0.266 | 0.285 | 0.948 |
| Sanguibacteroides justesenii | 0 | Not detected | -0.092 | -0.268 | 0.083 | 0.302 |
| Scardovia inopinata | 0 | Not detected | 0.070 | -0.093 | 0.233 | 0.398 |
| Scardovia wiggsiae | 1 | Not detected | 0.231 | 0.017 | 0.445 | 0.034 |
| Selenomonas bovis | 0 | Not detected | -0.049 | -0.191 | 0.093 | 0.502 |
| Selenomonas noxia | 0 | Not detected | 0.072 | -0.092 | 0.236 | 0.389 |
| Selenomonas ruminantium | 0 | Not detected | 0.081 | -0.123 | 0.285 | 0.437 |

| Selenomonas sputigena | 0 | Not detected | 0.008 | -0.164 | 0.179 | 0.930 |
| --- | --- | --- | --- | --- | --- | --- |
| Sellimonas intestinalis | 0 | Not detected | -0.035 | -0.142 | 0.071 | 0.517 |
| Senegalimassilia anaerobia | 0 | Not detected | 0.057 | -0.145 | 0.260 | 0.579 |
| Serratia fonticola | 0 | Not detected | -0.059 | -0.217 | 0.099 | 0.465 |
| Serratia liquefaciens | 0 | Not detected | 0.094 | -0.075 | 0.263 | 0.276 |
| Serratia marcescens | 0 | Not detected | -0.015 | -0.199 | 0.168 | 0.869 |
| Serratia odorifera | 0 | Not detected | -0.011 | -0.184 | 0.162 | 0.899 |
| Serratia rubidaea | 0 | Not detected | -0.032 | -0.211 | 0.148 | 0.729 |
| Sharpea azabuensis | 0 | Not detected | 0.024 | -0.073 | 0.121 | 0.630 |
| Shigella dysenteriae | 0 | Not detected | 0.091 | -0.189 | 0.371 | 0.525 |
| Shigella flexneri | 0 | Not detected | 0.106 | -0.174 | 0.385 | 0.459 |
| Shimwellia blattae | 0 | Not detected | 0.066 | -0.167 | 0.299 | 0.576 |
| Shuttleworthia satelles | 0 | Not detected | 0.012 | -0.093 | 0.117 | 0.822 |
| Siccibacter turicensis | 0 | Not detected | 0.007 | -0.240 | 0.254 | 0.957 |
| Slackia exigua | 0 | Not detected | 0.023 | -0.154 | 0.200 | 0.797 |
| Slackia heliotrinireducens | 0 | Not detected | -0.069 | -0.226 | 0.089 | 0.392 |
| Slackia piriformis | 1 | Not detected | 0.258 | -0.006 | 0.523 | 0.056 |
| Solobacterium moorei | 0 | Not detected | 0.059 | -0.052 | 0.169 | 0.298 |
| Sphaerochaeta sp. Phil3 | 0 | Not detected | 0.054 | -0.139 | 0.248 | 0.580 |
| Sphingobacterium wenxiniae | 0 | Not detected | 0.002 | -0.188 | 0.192 | 0.984 |
| Spirochaetales bacterium Spiro_07 | 0 | Not detected | -0.095 | -0.252 | 0.061 | 0.233 |
| Sporobacter termitidis | 0 | Not detected | 0.083 | -0.072 | 0.239 | 0.293 |
| Sporomusa ovata | 0 | Not detected | -0.147 | -0.323 | 0.030 | 0.103 |
| Staphylococcus pseudintermedius | 0 | Not detected | 0.203 | 0.019 | 0.387 | 0.031 |
| Staphylococcus sp. CAG:324 | 0 | Not detected | 0.077 | -0.259 | 0.414 | 0.652 |
| Staphylococcus sp. DORA_6_22 | 0 | Not detected | 0.044 | -0.136 | 0.224 | 0.630 |
| Staphylococcus warneri | 0 | Not detected | -0.003 | -0.202 | 0.196 | 0.978 |
| Stomatobaculum longum | 0 | Not detected | 0.001 | -0.060 | 0.063 | 0.964 |
| Streptobacillus hongkongensis | 0 | Not detected | 0.005 | -0.172 | 0.182 | 0.954 |
| Streptobacillus notomytis | 0 | Not detected | -0.026 | -0.226 | 0.174 | 0.797 |
| Streptococcus agalactiae | 0 | Not detected | 0.175 | -0.024 | 0.374 | 0.086 |
| Streptococcus anginosus | 0 | Not detected | 0.253 | 0.024 | 0.482 | 0.031 |
| Streptococcus caballi | 0 | Not detected | 0.042 | -0.168 | 0.251 | 0.696 |
| Streptococcus cristatus | 0 | Not detected | 0.120 | -0.062 | 0.303 | 0.196 |
| Streptococcus dysgalactiae | 0 | Not detected | 0.146 | -0.058 | 0.350 | 0.160 |
| Streptococcus entericus | 0 | Not detected | 0.099 | -0.066 | 0.264 | 0.239 |
| Streptococcus gallolyticus | 0 | Not detected | 0.078 | -0.175 | 0.330 | 0.546 |
| Streptococcus gordonii | 0 | Not detected | 0.020 | -0.220 | 0.259 | 0.873 |
| Streptococcus halotolerans | 0 | Not detected | -0.016 | -0.185 | 0.153 | 0.849 |
| Streptococcus henryi | 0 | Not detected | 0.055 | -0.147 | 0.257 | 0.593 |
| Streptococcus ictaluri | 0 | Not detected | -0.054 | -0.231 | 0.123 | 0.550 |
| Streptococcus infantis | 0 | Not detected | 0.026 | -0.154 | 0.206 | 0.777 |
| Streptococcus iniae | 0 | Not detected | -0.013 | -0.194 | 0.167 | 0.884 |
| Streptococcus intermedius | 0 | Not detected | 0.193 | -0.038 | 0.425 | 0.101 |

| Streptococcus massiliensis | 0 | Not detected | 0.014 | -0.172 | 0.200 | 0.881 |
| --- | --- | --- | --- | --- | --- | --- |
| Streptococcus minor | 0 | Not detected | -0.010 | -0.151 | 0.131 | 0.889 |
| Streptococcus mitis | 0 | Not detected | 0.018 | -0.120 | 0.155 | 0.802 |
| Streptococcus mutans | 0 | Not detected | 0.138 | -0.136 | 0.413 | 0.324 |
| Streptococcus oralis | 0 | Not detected | 0.015 | -0.197 | 0.227 | 0.891 |
| Streptococcus orisratti | 0 | Not detected | 0.261 | 0.062 | 0.460 | 0.010 |
| Streptococcus ovis | 0 | Not detected | 0.020 | -0.162 | 0.201 | 0.831 |
| Streptococcus parasanguinis | 0 | Not detected | 0.074 | -0.144 | 0.292 | 0.504 |
| Streptococcus parauberis | 0 | Not detected | -0.026 | -0.212 | 0.159 | 0.780 |
| Streptococcus phocae | 0 | Not detected | -0.024 | -0.164 | 0.116 | 0.740 |
| Streptococcus plurextorum | 0 | Not detected | -0.025 | -0.117 | 0.068 | 0.602 |
| Streptococcus porci | 0 | Not detected | 0.027 | -0.082 | 0.135 | 0.631 |
| Streptococcus pseudoporcinus | 0 | Not detected | 0.106 | -0.092 | 0.305 | 0.294 |
| Streptococcus pyogenes | 0 | Not detected | 0.082 | -0.120 | 0.285 | 0.427 |
| Streptococcus salivarius | 0 | Not detected | 0.021 | -0.181 | 0.223 | 0.839 |
| Streptococcus sanguinis | 0 | Not detected | 0.105 | -0.126 | 0.337 | 0.374 |
| Streptococcus sobrinus | 0 | Not detected | 0.187 | -0.027 | 0.401 | 0.087 |
| Streptococcus sp. X13SY08 | 0 | Not detected | 0.046 | -0.099 | 0.191 | 0.535 |
| Streptococcus suis | 0 | Not detected | 0.079 | -0.015 | 0.174 | 0.101 |
| Streptococcus thermophilus | 0 | Not detected | -0.079 | -0.291 | 0.133 | 0.466 |
| Streptococcus uberis | 0 | Not detected | 0.060 | -0.116 | 0.237 | 0.502 |
| Streptococcus urinalis | 0 | Not detected | 0.011 | -0.191 | 0.212 | 0.917 |
| Streptomyces albulus | 0 | Not detected | 0.023 | -0.140 | 0.187 | 0.779 |
| Streptomyces cattleya | 0 | Not detected | 0.100 | -0.088 | 0.287 | 0.298 |
| Streptomyces kanamyceticus | 0 | Not detected | 0.095 | -0.028 | 0.218 | 0.129 |
| Subdoligranulum sp. CAG:314 | 0 | Not detected | 0.349 | -0.042 | 0.741 | 0.081 |
| Subdoligranulum variabile | 0 | Not detected | 0.024 | -0.050 | 0.099 | 0.526 |
| Succinatimonas sp. CAG:777 | 0 | Not detected | 0.111 | -0.215 | 0.437 | 0.504 |
| Succinispira mobilis | 2 | Not detected | -0.243 | -0.430 | -0.056 | 0.011 |
| Succinivibrio sp. Phil9 | 0 | Not detected | 0.186 | -0.125 | 0.497 | 0.241 |
| Sulfolobus acidocaldarius | 0 | Not detected | -0.145 | -0.380 | 0.091 | 0.229 |
| Sutterella parvirubra | 0 | Not detected | -0.155 | -0.361 | 0.050 | 0.139 |
| Sutterella sp. CAG:397 | 0 | Not detected | 0.132 | -0.293 | 0.557 | 0.542 |
| Sutterella sp. CAG:521 | 0 | Not detected | -0.173 | -0.442 | 0.097 | 0.209 |
| Sutterella wadsworthensis | 0 | Not detected | -0.071 | -0.409 | 0.267 | 0.682 |
| Sutterella wadsworthensis CAG:135 | 0 | Not detected | -0.121 | -0.544 | 0.301 | 0.574 |
| Sutterellaceae bacterium ND3 | 0 | Not detected | 0.101 | -0.156 | 0.357 | 0.443 |
| Synergistales bacterium Syner_02 | 0 | Not detected | 0.037 | -0.180 | 0.255 | 0.737 |
| Synergistes sp. 3_1_syn1 | 0 | Not detected | 0.141 | -0.137 | 0.419 | 0.321 |
| Synergistes sp. Zagget9 | 0 | Not detected | -0.116 | -0.291 | 0.059 | 0.194 |
| Tannerella forsythia | 0 | Not detected | -0.054 | -0.184 | 0.076 | 0.414 |
| Tatumella morbirosei | 0 | Not detected | 0.047 | -0.120 | 0.213 | 0.584 |
| Tepidanaerobacter sp. EBM-49 | 0 | Not detected | -0.087 | -0.352 | 0.178 | 0.521 |
| Terrisporobacter glycolicus | 0 | Not detected | 0.091 | -0.135 | 0.316 | 0.430 |

| Thermophagus xiamenensis | 0 | Not detected | 0.021 | -0.179 | 0.221 | 0.839 |
| --- | --- | --- | --- | --- | --- | --- |
| Thermosinus carboxydivorans | 1 | Not detected | -0.123 | -0.273 | 0.027 | 0.107 |
| Tidjanibacter massiliensis | 0 | Not detected | 0.039 | -0.229 | 0.307 | 0.775 |
| Tissierellia bacterium S7-1-4 | 517 | Not detected | -0.295 | -0.498 | -0.092 | 0.004 |
| Trabulsiella odontotermitis | 0 | Not detected | -0.034 | -0.291 | 0.223 | 0.794 |
| Traorella massiliensis | 0 | Not detected | -0.023 | -0.097 | 0.051 | 0.547 |
| Treponema denticola | 0 | Not detected | 0.101 | -0.046 | 0.247 | 0.178 |
| Treponema medium | 0 | Not detected | -0.301 | -0.547 | -0.055 | 0.017 |
| Treponema putidum | 0 | Not detected | 0.061 | -0.068 | 0.189 | 0.355 |
| Treponema socranskii | 0 | Not detected | -0.019 | -0.166 | 0.129 | 0.805 |
| Treponema succinifaciens | 1 | Not detected | 0.068 | -0.020 | 0.156 | 0.130 |
| Treponema vincentii | 0 | Not detected | -0.014 | -0.147 | 0.119 | 0.836 |
| Trichococcus flocculiformis | 0 | Not detected | -0.119 | -0.281 | 0.042 | 0.148 |
| Trueperella pyogenes | 0 | Not detected | -0.047 | -0.212 | 0.118 | 0.576 |
| Tumebacillus flagellatus | 0 | Not detected | 0.120 | -0.047 | 0.287 | 0.160 |
| Turicibacter sanguinis | 0 | Not detected | 0.000 | -0.297 | 0.297 | 0.999 |
| Turicibacter sp. H121 | 1 | Not detected | 0.103 | -0.013 | 0.219 | 0.083 |
| Tyzzerella nexilis | 0 | Not detected | -0.044 | -0.111 | 0.024 | 0.207 |
| Urmitella timonensis | 0 | Not detected | -0.042 | -0.166 | 0.081 | 0.499 |
| Vaginella massiliensis | 0 | Not detected | -0.104 | -0.350 | 0.142 | 0.409 |
| Varibaculum cambriense | 0 | Not detected | -0.041 | -0.143 | 0.061 | 0.435 |
| Varibaculum sp. Marseille-P2802 | 0 | Not detected | -0.037 | -0.198 | 0.124 | 0.652 |
| Veillonella magna | 0 | Not detected | -0.031 | -0.107 | 0.046 | 0.429 |
| Veillonella parvula | 0 | Not detected | 0.102 | -0.146 | 0.351 | 0.420 |
| Veillonella seminalis | 0 | Not detected | -0.023 | -0.106 | 0.060 | 0.586 |
| Veillonella sp. ACP1 | 0 | Not detected | 0.019 | -0.282 | 0.320 | 0.902 |
| Veillonella sp. CAG:933 | 0 | Not detected | -0.042 | -0.184 | 0.100 | 0.564 |
| Veillonella sp. oral taxon 780 | 0 | Not detected | -0.126 | -0.309 | 0.056 | 0.176 |
| Waddlia chondrophila | 0 | Not detected | -0.142 | -0.370 | 0.087 | 0.224 |
| Weissella cibaria | 0 | Not detected | 0.036 | -0.200 | 0.272 | 0.764 |
| Weissella confusa | 0 | Not detected | -0.044 | -0.312 | 0.223 | 0.745 |
| Xanthomonas sp. Mitacek01 | 0 | Not detected | 0.061 | -0.075 | 0.196 | 0.380 |
| Yersinia enterocolitica | 0 | Not detected | -0.018 | -0.240 | 0.204 | 0.875 |
| Yersinia pestis | 0 | Not detected | 0.072 | -0.210 | 0.353 | 0.618 |
| Yokenella regensburgei | 0 | Not detected | -0.021 | -0.274 | 0.233 | 0.872 |
| [Bacteroides] pectinophilus | 0 | Not detected | 0.017 | -0.063 | 0.097 | 0.671 |
| [Clostridium] aminophilum | 0 | Not detected | 0.019 | -0.052 | 0.091 | 0.601 |
| [Clostridium] asparagiforme | 99 | Not detected | -0.153 | -0.257 | -0.049 | 0.004 |
| [Clostridium] bolteae | 0 | Not detected | -0.076 | -0.171 | 0.020 | 0.121 |
| [Clostridium] celerecrescens | 0 | Not detected | -0.070 | -0.136 | -0.004 | 0.038 |
| [Clostridium] cellobioparum | 0 | Not detected | 0.087 | -0.062 | 0.237 | 0.253 |
| [Clostridium] cellulolyticum | 0 | Not detected | 0.127 | -0.040 | 0.294 | 0.136 |
| [Clostridium] cellulosi | 0 | Not detected | 0.107 | -0.059 | 0.274 | 0.205 |
| [Clostridium] citroniae | 0 | Not detected | -0.083 | -0.162 | -0.004 | 0.040 |

| [Clostridium] clariflavum | 0 | Not detected | 0.056 | -0.090 | 0.202 | 0.452 |
| --- | --- | --- | --- | --- | --- | --- |
| [Clostridium] clostridioforme | 0 | Not detected | -0.127 | -0.229 | -0.026 | 0.014 |
| [Clostridium] dakarense | 0 | Not detected | 0.141 | -0.087 | 0.368 | 0.226 |
| [Clostridium] glycyrrhizinilyticum | 0 | Not detected | -0.039 | -0.107 | 0.028 | 0.249 |
| [Clostridium] hiranonis | 0 | Not detected | 0.171 | -0.032 | 0.373 | 0.098 |
| [Clostridium] hylemonae | 0 | Not detected | -0.159 | -0.306 | -0.012 | 0.035 |
| [Clostridium] innocuum | 5 | Not detected | 0.053 | -0.019 | 0.125 | 0.149 |
| [Clostridium] josui | 0 | Not detected | 0.052 | -0.121 | 0.224 | 0.556 |
| [Clostridium] lactatifermentans | 0 | Not detected | -0.046 | -0.135 | 0.043 | 0.312 |
| [Clostridium] leptum | 0 | Not detected | -0.006 | -0.119 | 0.108 | 0.923 |
| [Clostridium] methylpentosum | 0 | Not detected | -0.078 | -0.196 | 0.041 | 0.197 |
| [Clostridium] papyrosolvens | 0 | Not detected | 0.038 | -0.091 | 0.168 | 0.564 |
| [Clostridium] saccharogumia | 0 | Not detected | -0.039 | -0.143 | 0.066 | 0.469 |
| [Clostridium] saccharolyticum | 0 | Not detected | -0.033 | -0.128 | 0.063 | 0.503 |
| [Clostridium] scindens | 0 | Not detected | 0.014 | -0.068 | 0.097 | 0.736 |
| [Clostridium] spiroforme | 0 | Not detected | -0.062 | -0.251 | 0.127 | 0.521 |
| [Clostridium] sporosphaeroides | 0 | Not detected | -0.004 | -0.155 | 0.147 | 0.959 |
| [Clostridium] straminisolvens | 0 | Not detected | 0.084 | -0.039 | 0.208 | 0.182 |
| [Clostridium] symbiosum | 0 | Not detected | -0.081 | -0.165 | 0.003 | 0.060 |
| [Clostridium] viride | 0 | Not detected | 0.045 | -0.098 | 0.188 | 0.537 |
| [Enterobacter] aerogenes | 0 | Not detected | 0.107 | -0.217 | 0.431 | 0.517 |
| [Eubacterium] brachy | 0 | Not detected | 0.068 | -0.152 | 0.288 | 0.546 |
| [Eubacterium] cellulosolvens | 0 | Not detected | -0.048 | -0.143 | 0.047 | 0.324 |
| [Eubacterium] contortum | 0 | Not detected | -0.002 | -0.066 | 0.062 | 0.950 |
| [Eubacterium] dolichum | 0 | Not detected | 0.030 | -0.036 | 0.095 | 0.374 |
| [Eubacterium] eligens | 0 | Not detected | -0.052 | -0.217 | 0.113 | 0.538 |
| [Eubacterium] fissicatena | 0 | Not detected | -0.007 | -0.094 | 0.079 | 0.865 |
| [Eubacterium] hallii | 0 | Not detected | 0.019 | -0.092 | 0.130 | 0.734 |
| [Eubacterium] nodatum | 0 | Not detected | 0.011 | -0.187 | 0.209 | 0.913 |
| [Eubacterium] rectale | 0 | Not detected | 0.137 | -0.025 | 0.298 | 0.098 |
| [Eubacterium] siraeum | 0 | Not detected | 0.108 | -0.145 | 0.361 | 0.402 |
| [Eubacterium] sulci | 521 | Not detected | -0.197 | -0.411 | 0.018 | 0.072 |
| [Eubacterium] yurii | 0 | Not detected | 0.084 | -0.042 | 0.210 | 0.192 |
| [Haemophilus] ducreyi | 0 | Not detected | -0.164 | -0.322 | -0.007 | 0.041 |
| [Haemophilus] parasuis | 0 | Not detected | -0.125 | -0.314 | 0.064 | 0.195 |
| [Ruminococcus] gnavus | 0 | Not detected | -0.039 | -0.165 | 0.087 | 0.547 |
| [Ruminococcus] torques | 0 | Not detected | -0.167 | -0.305 | -0.029 | 0.017 |
| bacterium F082 | 0 | Not detected | 0.011 | -0.099 | 0.120 | 0.849 |
| bacterium LF-3 | 0 | Not detected | -0.032 | -0.200 | 0.137 | 0.712 |
| bacterium MS4 | 0 | Not detected | 0.056 | -0.083 | 0.195 | 0.432 |
| bacterium OL-1 | 0 | Not detected | -0.139 | -0.408 | 0.130 | 0.310 |
| bacterium P201 | 0 | Not detected | 0.162 | -0.009 | 0.333 | 0.063 |
| bacterium P3 | 0 | Not detected | 0.254 | 0.057 | 0.451 | 0.012 |

| **Supplementary Table 20**. Association of childhood SEP with species in US-born participants (n=302). Analysis of Compositions of Microbiomes (ANCOM2) was used to identify species related to Lower childhood SEP. Regression coefficients were obtained by modelling the effect of Lower childhood SEP on centered log ratio (CLR)-transformed species in multivariable linear regression adjusted for age, gender, BMI, field center, Hispanic/Latino background, U.S. nativity, Bristol stool type, current SEP, the alternative Healthy Eating Index 2010 score, cigarette smoking, alcohol use, depressive and anxiety symptoms, diabetes, hypertension, cardiovascular disease, dyslipidemia, and antibiotic use. | | | | | | |
| --- | --- | --- | --- | --- | --- | --- |
| **species** | **W_stat** | **ANCOM** | **beta** | **l95ci** | **u95ci** | **qval** |
| Abiotrophia defectiva | 0 | Not detected | -0.148 | -0.532 | 0.236 | 0.451 |
| Acetanaerobacterium elongatum | 0 | Not detected | -0.212 | -0.517 | 0.094 | 0.175 |
| Acetitomaculum ruminis | 0 | Not detected | -0.018 | -0.239 | 0.203 | 0.874 |
| Acetivibrio cellulolyticus | 0 | Not detected | -0.111 | -0.420 | 0.199 | 0.485 |
| Acetivibrio ethanolgignens | 0 | Not detected | -0.087 | -0.256 | 0.083 | 0.317 |
| Acetobacter sp. 46_36 | 0 | Not detected | -0.471 | -1.205 | 0.263 | 0.210 |
| Acetobacter sp. CAG:267 | 0 | Not detected | -0.145 | -0.786 | 0.495 | 0.657 |
| Acholeplasma equifetale | 0 | Not detected | 0.339 | -0.032 | 0.710 | 0.074 |
| Acidaminococcus fermentans | 0 | Not detected | -0.005 | -0.373 | 0.362 | 0.978 |
| Acidaminococcus intestini | 0 | Not detected | 0.057 | -0.655 | 0.769 | 0.875 |
| Acidaminococcus intestini CAG:325 | 0 | Not detected | 0.396 | -0.588 | 1.381 | 0.431 |
| Acinetobacter sp. CAG:196 | 0 | Not detected | 0.115 | -0.582 | 0.812 | 0.747 |
| Acinetobacter sp. CAG:196_36_41 | 0 | Not detected | 0.049 | -0.615 | 0.712 | 0.886 |
| Acinetobacter sp. N54.MGS-139 | 0 | Not detected | 0.085 | -0.326 | 0.497 | 0.686 |
| Acinetobacter venetianus | 0 | Not detected | -0.243 | -0.593 | 0.107 | 0.174 |
| Actinobaculum sp. oral taxon 183 | 0 | Not detected | 0.250 | -0.237 | 0.738 | 0.315 |
| Actinomyces dentalis | 0 | Not detected | 0.184 | -0.314 | 0.683 | 0.469 |
| Actinomyces gerencseriae | 0 | Not detected | -0.076 | -0.506 | 0.354 | 0.729 |
| Actinomyces graevenitzii | 0 | Not detected | 0.133 | -0.260 | 0.526 | 0.507 |
| Actinomyces hongkongensis | 0 | Not detected | 0.109 | -0.341 | 0.560 | 0.635 |
| Actinomyces israelii | 0 | Not detected | 0.214 | -0.260 | 0.688 | 0.376 |
| Actinomyces johnsonii | 0 | Not detected | -0.059 | -0.521 | 0.403 | 0.803 |
| Actinomyces massiliensis | 0 | Not detected | 0.175 | -0.266 | 0.616 | 0.437 |
| Actinomyces meyeri | 0 | Not detected | -0.152 | -0.524 | 0.221 | 0.426 |
| Actinomyces odontolyticus | 0 | Not detected | -0.086 | -0.472 | 0.300 | 0.664 |
| Actinomyces oris | 0 | Not detected | 0.158 | -0.261 | 0.576 | 0.461 |
| Actinomyces sp. HMSC065F12 | 0 | Not detected | -0.015 | -0.310 | 0.280 | 0.921 |
| Actinomyces sp. HMSC06A08 | 0 | Not detected | 0.029 | -0.260 | 0.317 | 0.845 |
| Actinomyces sp. oral taxon 181 | 0 | Not detected | -0.316 | -0.771 | 0.139 | 0.175 |
| Actinomyces sp. oral taxon 448 | 0 | Not detected | 0.187 | -0.346 | 0.720 | 0.492 |
| Actinomyces sp. oral taxon 848 | 0 | Not detected | 0.133 | -0.189 | 0.455 | 0.420 |
| Actinomyces sp. ph3 | 0 | Not detected | -0.302 | -0.755 | 0.151 | 0.193 |
| Actinomyces timonensis | 0 | Not detected | -0.105 | -0.445 | 0.234 | 0.544 |
| Actinomyces turicensis | 0 | Not detected | -0.360 | -0.825 | 0.106 | 0.131 |
| Adlercreutzia equolifaciens | 0 | Not detected | 0.133 | -0.463 | 0.728 | 0.662 |
| Aeromonas salmonicida | 0 | Not detected | -0.185 | -0.591 | 0.222 | 0.374 |
| Aggregatibacter aphrophilus | 0 | Not detected | 0.184 | -0.237 | 0.606 | 0.392 |

| Akkermansia glycaniphila | 0 | Not detected | 0.158 | -0.282 | 0.597 | 0.483 |
| --- | --- | --- | --- | --- | --- | --- |
| Akkermansia muciniphila | 0 | Not detected | 0.171 | -0.485 | 0.828 | 0.609 |
| Akkermansia muciniphila CAG:154 | 0 | Not detected | 0.394 | -0.465 | 1.253 | 0.369 |
| Akkermansia sp. 54_46 | 0 | Not detected | 0.426 | -0.450 | 1.301 | 0.341 |
| Akkermansia sp. CAG:344 | 0 | Not detected | -0.270 | -1.141 | 0.600 | 0.543 |
| Akkermansia sp. KLE1605 | 0 | Not detected | -0.200 | -0.945 | 0.546 | 0.600 |
| Akkermansia sp. KLE1797 | 0 | Not detected | -0.191 | -0.937 | 0.554 | 0.616 |
| Akkermansia sp. KLE1798 | 0 | Not detected | -0.211 | -0.957 | 0.536 | 0.581 |
| Akkermansia sp. Phil8 | 0 | Not detected | 0.359 | -0.517 | 1.235 | 0.423 |
| Akkermansia sp. UNK.MGS-1 | 0 | Not detected | 0.470 | -0.395 | 1.334 | 0.288 |
| Alistipes finegoldii | 0 | Not detected | -0.188 | -0.548 | 0.172 | 0.306 |
| Alistipes ihumii | 0 | Not detected | 0.155 | -0.487 | 0.796 | 0.637 |
| Alistipes indistinctus | 0 | Not detected | 0.337 | -0.518 | 1.193 | 0.440 |
| Alistipes inops | 0 | Not detected | 0.043 | -0.575 | 0.661 | 0.891 |
| Alistipes obesi | 0 | Not detected | 0.145 | -0.221 | 0.511 | 0.439 |
| Alistipes putredinis | 0 | Not detected | -0.047 | -0.552 | 0.457 | 0.855 |
| Alistipes senegalensis | 0 | Not detected | -0.009 | -0.336 | 0.318 | 0.957 |
| Alistipes shahii | 0 | Not detected | 0.079 | -0.259 | 0.417 | 0.646 |
| Alistipes sp. CAG:435 | 0 | Not detected | 0.137 | -0.526 | 0.800 | 0.685 |
| Alistipes sp. CAG:514 | 0 | Not detected | 0.518 | -0.141 | 1.177 | 0.124 |
| Alistipes sp. Zagget8 | 0 | Not detected | 0.024 | -0.495 | 0.542 | 0.929 |
| Alistipes timonensis | 0 | Not detected | 0.091 | -0.484 | 0.666 | 0.756 |
| Alkaliflexus imshenetskii | 2 | Not detected | -0.337 | -0.659 | -0.015 | 0.041 |
| Allisonella histaminiformans | 0 | Not detected | -0.163 | -0.913 | 0.587 | 0.671 |
| Allobaculum stercoricanis | 0 | Not detected | -0.017 | -0.218 | 0.183 | 0.865 |
| Allofustis seminis | 0 | Not detected | -0.105 | -0.484 | 0.274 | 0.589 |
| Alloprevotella rava | 0 | Not detected | -0.240 | -0.561 | 0.082 | 0.145 |
| Alloprevotella tannerae | 0 | Not detected | -0.117 | -0.374 | 0.139 | 0.371 |
| Alloscardovia criceti | 0 | Not detected | 0.166 | -0.210 | 0.542 | 0.388 |
| Alloscardovia omnicolens | 0 | Not detected | 0.085 | -0.348 | 0.518 | 0.702 |
| Amphibacillus xylanus | 0 | Not detected | 0.251 | -0.131 | 0.632 | 0.199 |
| Anaerobium acetethylicum | 0 | Not detected | -0.129 | -0.317 | 0.059 | 0.179 |
| Anaerococcus hydrogenalis | 0 | Not detected | 0.038 | -0.297 | 0.374 | 0.822 |
| Anaerococcus lactolyticus | 0 | Not detected | 0.114 | -0.300 | 0.528 | 0.590 |
| Anaerococcus obesiensis | 0 | Not detected | -0.255 | -0.629 | 0.119 | 0.182 |
| Anaerococcus tetradius | 0 | Not detected | -0.256 | -0.591 | 0.080 | 0.137 |
| Anaerocolumna aminovalerica | 0 | Not detected | -0.056 | -0.413 | 0.302 | 0.760 |
| Anaerocolumna xylanovorans | 0 | Not detected | -0.088 | -0.414 | 0.238 | 0.597 |
| Anaerofustis stercorihominis | 0 | Not detected | -0.213 | -0.764 | 0.338 | 0.449 |
| Anaeroglobus geminatus | 0 | Not detected | -0.346 | -0.820 | 0.129 | 0.155 |
| Anaeromassilibacillus senegalensis | 0 | Not detected | 0.004 | -0.331 | 0.339 | 0.980 |
| Anaerophaga thermohalophila | 0 | Not detected | -0.092 | -0.339 | 0.155 | 0.465 |
| Anaerosalibacter massiliensis | 0 | Not detected | 0.165 | -0.254 | 0.585 | 0.440 |
| Anaerosporobacter mobilis | 0 | Not detected | -0.069 | -0.304 | 0.166 | 0.566 |

| Anaerosporomusa subterranea | 0 | Not detected | 0.018 | -0.265 | 0.300 | 0.902 |
| --- | --- | --- | --- | --- | --- | --- |
| Anaerostipes caccae | 0 | Not detected | -0.049 | -0.270 | 0.171 | 0.662 |
| Anaerostipes hadrus | 0 | Not detected | -0.026 | -0.384 | 0.332 | 0.887 |
| Anaerostipes sp. CAG:276 | 0 | Not detected | 0.098 | -0.625 | 0.822 | 0.790 |
| Anaerotruncus colihominis | 0 | Not detected | -0.139 | -0.354 | 0.075 | 0.205 |
| Anaerotruncus sp. CAG:390 | 0 | Not detected | -0.034 | -0.694 | 0.625 | 0.919 |
| Anaerotruncus sp. CAG:528 | 0 | Not detected | -0.056 | -0.591 | 0.478 | 0.836 |
| Anaerotruncus sp. G3(2012) | 0 | Not detected | -0.012 | -0.192 | 0.168 | 0.894 |
| Anaerovorax odorimutans | 0 | Not detected | 0.163 | -0.138 | 0.465 | 0.290 |
| Angelakisella massiliensis | 0 | Not detected | -0.095 | -0.287 | 0.096 | 0.330 |
| Aphanizomenon flos-aquae | 0 | Not detected | 0.151 | -0.115 | 0.417 | 0.268 |
| Arcanobacterium haemolyticum | 0 | Not detected | -0.224 | -0.576 | 0.129 | 0.215 |
| Asaccharospora irregularis | 0 | Not detected | 0.224 | -0.196 | 0.645 | 0.297 |
| Atlantibacter hermannii | 0 | Not detected | 0.008 | -0.448 | 0.465 | 0.971 |
| Atopobium deltae | 0 | Not detected | -0.359 | -0.756 | 0.038 | 0.077 |
| Atopobium minutum | 0 | Not detected | -0.450 | -0.846 | -0.055 | 0.026 |
| Atopobium parvulum | 0 | Not detected | 0.122 | -0.360 | 0.604 | 0.620 |
| Atopobium rimae | 0 | Not detected | -0.165 | -0.546 | 0.216 | 0.398 |
| Atopobium sp. BS2 | 0 | Not detected | 0.092 | -0.360 | 0.544 | 0.690 |
| Atopobium sp. HMSC064B08 | 0 | Not detected | 0.017 | -0.319 | 0.352 | 0.922 |
| Atopobium sp. ICM42b | 0 | Not detected | 0.179 | -0.279 | 0.636 | 0.445 |
| Atopobium vaginae | 0 | Not detected | -0.045 | -0.494 | 0.405 | 0.845 |
| Atopostipes suicloacalis | 0 | Not detected | -0.041 | -0.406 | 0.324 | 0.825 |
| Azospirillum sp. 47_25 | 0 | Not detected | -0.443 | -1.160 | 0.273 | 0.227 |
| Azospirillum sp. CAG:260 | 0 | Not detected | -0.371 | -1.054 | 0.312 | 0.288 |
| Bacillus acidiproducens | 0 | Not detected | 0.150 | -0.282 | 0.583 | 0.496 |
| Bacillus andreraoultii | 0 | Not detected | 0.042 | -0.374 | 0.458 | 0.844 |
| Bacillus azotoformans | 0 | Not detected | 0.066 | -0.312 | 0.444 | 0.732 |
| Bacillus bataviensis | 0 | Not detected | -0.063 | -0.443 | 0.317 | 0.745 |
| Bacillus coahuilensis | 0 | Not detected | -0.078 | -0.440 | 0.284 | 0.673 |
| Bacillus rubiinfantis | 0 | Not detected | 0.007 | -0.352 | 0.365 | 0.970 |
| Bacillus sp. CAG:988 | 0 | Not detected | 0.141 | -0.227 | 0.508 | 0.453 |
| Bacillus thermotolerans | 0 | Not detected | 0.075 | -0.324 | 0.475 | 0.712 |
| Bacteroidales bacterium 45-6 | 0 | Not detected | 0.251 | -0.108 | 0.611 | 0.172 |
| Bacteroidales bacterium 55_9 | 0 | Not detected | -0.195 | -0.444 | 0.055 | 0.128 |
| Bacteroidales bacterium Bact_08 | 0 | Not detected | -0.229 | -0.561 | 0.102 | 0.177 |
| Bacteroidales bacterium Bact_18 | 0 | Not detected | 0.059 | -0.249 | 0.368 | 0.706 |
| Bacteroidales bacterium Bact_23 | 0 | Not detected | 0.222 | -0.210 | 0.655 | 0.315 |
| Bacteroidales bacterium CF | 0 | Not detected | -0.060 | -0.377 | 0.258 | 0.713 |
| Bacteroidales bacterium H10 | 0 | Not detected | -0.150 | -0.497 | 0.198 | 0.400 |
| Bacteroidales bacterium K1 | 0 | Not detected | -0.206 | -0.540 | 0.127 | 0.226 |
| Bacteroidales bacterium K10 | 0 | Not detected | -0.013 | -0.378 | 0.352 | 0.945 |
| Bacteroides barnesiae | 0 | Not detected | 0.046 | -0.178 | 0.269 | 0.690 |
| Bacteroides caccae CAG:21 | 0 | Not detected | -0.195 | -0.673 | 0.283 | 0.424 |

| Bacteroides cellulosilyticus | 0 | Not detected | -0.284 | -0.673 | 0.105 | 0.154 |
| --- | --- | --- | --- | --- | --- | --- |
| Bacteroides coprocola | 0 | Not detected | -0.124 | -0.430 | 0.182 | 0.427 |
| Bacteroides coprophilus | 0 | Not detected | 0.037 | -0.184 | 0.258 | 0.743 |
| Bacteroides coprosuis | 0 | Not detected | 0.050 | -0.260 | 0.360 | 0.753 |
| Bacteroides dorei CAG:222 | 0 | Not detected | 0.262 | -0.120 | 0.644 | 0.179 |
| Bacteroides faecichinchillae | 0 | Not detected | -0.072 | -0.294 | 0.151 | 0.529 |
| Bacteroides faecis CAG:32 | 0 | Not detected | -0.171 | -0.603 | 0.260 | 0.437 |
| Bacteroides finegoldii | 0 | Not detected | -0.214 | -0.663 | 0.234 | 0.350 |
| Bacteroides finegoldii CAG:203 | 0 | Not detected | -0.175 | -0.593 | 0.243 | 0.413 |
| Bacteroides fluxus | 0 | Not detected | -0.086 | -0.319 | 0.147 | 0.470 |
| Bacteroides fragilis | 0 | Not detected | -0.326 | -0.630 | -0.023 | 0.036 |
| Bacteroides fragilis CAG:47 | 0 | Not detected | -0.583 | -1.103 | -0.064 | 0.029 |
| Bacteroides graminisolvens | 0 | Not detected | -0.058 | -0.508 | 0.391 | 0.799 |
| Bacteroides neonati | 0 | Not detected | -0.063 | -0.261 | 0.136 | 0.539 |
| Bacteroides oleiciplenus | 0 | Not detected | -0.140 | -0.402 | 0.121 | 0.295 |
| Bacteroides ovatus | 0 | Not detected | 0.082 | -0.236 | 0.400 | 0.614 |
| Bacteroides ovatus CAG:22 | 0 | Not detected | 0.138 | -0.211 | 0.487 | 0.438 |
| Bacteroides paurosaccharolyticus | 0 | Not detected | 0.011 | -0.270 | 0.293 | 0.937 |
| Bacteroides pectinophilus CAG:437 | 0 | Not detected | 0.008 | -0.284 | 0.300 | 0.959 |
| Bacteroides plebeius | 0 | Not detected | -0.210 | -0.527 | 0.107 | 0.195 |
| Bacteroides propionicifaciens | 0 | Not detected | 0.315 | -0.032 | 0.662 | 0.077 |
| Bacteroides pyogenes | 0 | Not detected | 0.126 | -0.111 | 0.362 | 0.298 |
| Bacteroides reticulotermitis | 0 | Not detected | -0.127 | -0.411 | 0.158 | 0.384 |
| Bacteroides salanitronis | 0 | Not detected | -0.074 | -0.307 | 0.159 | 0.534 |
| Bacteroides salyersiae | 0 | Not detected | 0.056 | -0.236 | 0.347 | 0.708 |
| Bacteroides sp. 41_26 | 0 | Not detected | 0.039 | -0.347 | 0.425 | 0.844 |
| Bacteroides sp. 43_46 | 0 | Not detected | -0.174 | -0.514 | 0.165 | 0.315 |
| Bacteroides sp. CAG:1060 | 0 | Not detected | -0.354 | -0.875 | 0.167 | 0.184 |
| Bacteroides sp. CAG:1076 | 0 | Not detected | -0.339 | -0.773 | 0.095 | 0.127 |
| Bacteroides sp. CAG:530 | 0 | Not detected | -0.029 | -0.326 | 0.267 | 0.846 |
| Bacteroides sp. CAG:545 | 0 | Not detected | 0.038 | -0.654 | 0.731 | 0.913 |
| Bacteroides sp. CAG:714 | 0 | Not detected | 0.162 | -0.358 | 0.682 | 0.543 |
| Bacteroides sp. CAG:770 | 0 | Not detected | -0.227 | -0.594 | 0.139 | 0.226 |
| Bacteroides sp. CAG:98 | 0 | Not detected | -0.002 | -0.589 | 0.584 | 0.994 |
| Bacteroides sp. D2 | 0 | Not detected | 0.075 | -0.282 | 0.431 | 0.682 |
| Bacteroides sp. Ga6A1 | 0 | Not detected | -0.185 | -0.688 | 0.317 | 0.470 |
| Bacteroides sp. Ga6A2 | 0 | Not detected | -0.009 | -0.495 | 0.477 | 0.970 |
| Bacteroides stercoris | 0 | Not detected | 0.130 | -0.312 | 0.573 | 0.564 |
| Bacteroides thetaiotaomicron | 0 | Not detected | 0.064 | -0.183 | 0.310 | 0.613 |
| Bacteroides thetaiotaomicron CAG:40 | 0 | Not detected | 0.192 | -0.206 | 0.590 | 0.346 |
| Bacteroides uniformis | 0 | Not detected | 0.092 | -0.270 | 0.455 | 0.618 |
| Bacteroides vulgatus | 0 | Not detected | 0.195 | -0.129 | 0.518 | 0.239 |
| Bacteroides vulgatus CAG:6 | 0 | Not detected | 0.257 | -0.136 | 0.650 | 0.201 |
| Bacteroidetes bacterium OLB8 | 0 | Not detected | 0.252 | -0.134 | 0.638 | 0.202 |

| Bacteroidetes oral taxon 274 | 0 | Not detected | -0.023 | -0.297 | 0.250 | 0.868 |
| --- | --- | --- | --- | --- | --- | --- |
| Bariatricus massiliensis | 0 | Not detected | -0.083 | -0.264 | 0.097 | 0.365 |
| Barnesiella intestinihominis | 0 | Not detected | 0.180 | -0.236 | 0.596 | 0.398 |
| Barnesiella viscericola | 0 | Not detected | 0.017 | -0.177 | 0.211 | 0.864 |
| Bavariicoccus seileri | 0 | Not detected | 0.003 | -0.330 | 0.336 | 0.987 |
| Beduini massiliensis | 0 | Not detected | -0.204 | -0.463 | 0.055 | 0.123 |
| Bibersteinia trehalosi | 0 | Not detected | 0.194 | -0.238 | 0.627 | 0.380 |
| Bifidobacterium actinocoloniiforme | 0 | Not detected | -0.048 | -0.458 | 0.362 | 0.819 |
| Bifidobacterium adolescentis | 0 | Not detected | 0.282 | -0.428 | 0.992 | 0.437 |
| Bifidobacterium aesculapii | 0 | Not detected | 0.097 | -0.312 | 0.506 | 0.644 |
| Bifidobacterium angulatum | 0 | Not detected | 0.135 | -0.454 | 0.725 | 0.653 |
| Bifidobacterium animalis | 0 | Not detected | 0.084 | -0.351 | 0.518 | 0.706 |
| Bifidobacterium asteroides | 0 | Not detected | 0.085 | -0.349 | 0.520 | 0.700 |
| Bifidobacterium biavatii | 0 | Not detected | -0.051 | -0.243 | 0.140 | 0.600 |
| Bifidobacterium bifidum | 0 | Not detected | -0.101 | -0.794 | 0.591 | 0.774 |
| Bifidobacterium bohemicum | 0 | Not detected | 0.030 | -0.364 | 0.424 | 0.882 |
| Bifidobacterium bombi | 0 | Not detected | 0.091 | -0.296 | 0.478 | 0.646 |
| Bifidobacterium breve | 0 | Not detected | 0.559 | -0.104 | 1.222 | 0.100 |
| Bifidobacterium callitrichos | 0 | Not detected | 0.310 | -0.224 | 0.844 | 0.256 |
| Bifidobacterium choerinum | 0 | Not detected | 0.174 | -0.271 | 0.618 | 0.445 |
| Bifidobacterium coryneforme | 0 | Not detected | 0.028 | -0.383 | 0.440 | 0.892 |
| Bifidobacterium crudilactis | 0 | Not detected | -0.052 | -0.386 | 0.283 | 0.762 |
| Bifidobacterium cuniculi | 0 | Not detected | 0.114 | -0.323 | 0.552 | 0.609 |
| Bifidobacterium dentium | 0 | Not detected | -0.043 | -0.662 | 0.575 | 0.891 |
| Bifidobacterium gallicum | 0 | Not detected | -0.251 | -0.698 | 0.195 | 0.271 |
| Bifidobacterium gallinarum | 0 | Not detected | 0.164 | -0.412 | 0.739 | 0.578 |
| Bifidobacterium longum | 0 | Not detected | 0.429 | -0.227 | 1.084 | 0.201 |
| Bifidobacterium magnum | 0 | Not detected | 0.004 | -0.304 | 0.312 | 0.981 |
| Bifidobacterium minimum | 0 | Not detected | 0.149 | -0.426 | 0.723 | 0.612 |
| Bifidobacterium mongoliense | 0 | Not detected | 0.017 | -0.388 | 0.421 | 0.935 |
| Bifidobacterium pseudolongum | 0 | Not detected | 0.065 | -0.401 | 0.531 | 0.785 |
| Bifidobacterium psychraerophilum | 0 | Not detected | -0.028 | -0.340 | 0.283 | 0.858 |
| Bifidobacterium reuteri | 0 | Not detected | 0.316 | -0.163 | 0.796 | 0.197 |
| Bifidobacterium saguini | 0 | Not detected | 0.420 | -0.175 | 1.015 | 0.168 |
| Bifidobacterium scardovii | 0 | Not detected | 0.369 | -0.239 | 0.976 | 0.235 |
| Bifidobacterium stellenboschense | 0 | Not detected | -0.026 | -0.233 | 0.181 | 0.805 |
| Bifidobacterium subtile | 0 | Not detected | 0.166 | -0.355 | 0.687 | 0.533 |
| Bifidobacterium thermophilum | 0 | Not detected | 0.001 | -0.333 | 0.335 | 0.995 |
| Bifidobacterium tsurumiense | 0 | Not detected | 0.093 | -0.360 | 0.546 | 0.687 |
| Bilophila wadsworthia | 0 | Not detected | 0.124 | -0.666 | 0.914 | 0.759 |
| Bittarella massiliensis | 0 | Not detected | -0.029 | -0.206 | 0.147 | 0.746 |
| Blautia hansenii | 0 | Not detected | 0.100 | -0.138 | 0.338 | 0.411 |
| Blautia hydrogenotrophica | 0 | Not detected | -0.028 | -0.229 | 0.173 | 0.786 |
| Blautia hydrogenotrophica CAG:147 | 0 | Not detected | -0.203 | -0.741 | 0.336 | 0.462 |

| Blautia obeum | 0 | Not detected | 0.001 | -0.190 | 0.191 | 0.995 |
| --- | --- | --- | --- | --- | --- | --- |
| Blautia producta | 0 | Not detected | 0.130 | -0.138 | 0.398 | 0.341 |
| Blautia schinkii | 0 | Not detected | -0.075 | -0.215 | 0.066 | 0.299 |
| Blautia sp. CAG:237 | 0 | Not detected | 0.274 | -0.131 | 0.679 | 0.185 |
| Blautia sp. CAG:37 | 0 | Not detected | 0.047 | -0.361 | 0.455 | 0.822 |
| Blautia sp. CAG:37_48_57 | 0 | Not detected | 0.061 | -0.233 | 0.355 | 0.685 |
| Blautia sp. KLE 1732 | 0 | Not detected | -0.118 | -0.369 | 0.132 | 0.355 |
| Bulleidia extructa | 0 | Not detected | 0.276 | -0.156 | 0.708 | 0.212 |
| Burkholderiales bacterium GJ-E10 | 0 | Not detected | 0.106 | -0.283 | 0.496 | 0.592 |
| Butyricicoccus desmolans | 0 | Not detected | -0.100 | -0.264 | 0.064 | 0.233 |
| Butyricicoccus pullicaecorum | 0 | Not detected | -0.163 | -0.379 | 0.052 | 0.139 |
| Butyricicoccus sp. K4410.MGS-46 | 0 | Not detected | 0.124 | -0.177 | 0.426 | 0.420 |
| Butyricimonas sp. Marseille-P2440 | 0 | Not detected | -0.051 | -0.486 | 0.384 | 0.819 |
| Butyricimonas synergistica | 0 | Not detected | 0.350 | -0.142 | 0.842 | 0.164 |
| Butyricimonas virosa | 0 | Not detected | 0.262 | -0.171 | 0.695 | 0.237 |
| Butyrivibrio crossotus | 0 | Not detected | 0.020 | -0.249 | 0.289 | 0.886 |
| Butyrivibrio crossotus CAG:259 | 0 | Not detected | 0.002 | -0.428 | 0.432 | 0.993 |
| Butyrivibrio fibrisolvens | 0 | Not detected | 0.026 | -0.289 | 0.340 | 0.873 |
| Butyrivibrio hungatei | 0 | Not detected | 0.041 | -0.293 | 0.374 | 0.811 |
| Butyrivibrio proteoclasticus | 0 | Not detected | -0.096 | -0.324 | 0.132 | 0.411 |
| Butyrivibrio sp. AD3002 | 0 | Not detected | 0.107 | -0.160 | 0.373 | 0.434 |
| Butyrivibrio sp. AE2005 | 0 | Not detected | -0.066 | -0.364 | 0.233 | 0.667 |
| Butyrivibrio sp. AE3003 | 0 | Not detected | -0.091 | -0.346 | 0.165 | 0.488 |
| Butyrivibrio sp. AE3004 | 0 | Not detected | -0.193 | -0.394 | 0.008 | 0.061 |
| Butyrivibrio sp. AE3009 | 0 | Not detected | 0.035 | -0.264 | 0.333 | 0.820 |
| Butyrivibrio sp. FCS006 | 0 | Not detected | 0.057 | -0.268 | 0.383 | 0.729 |
| Butyrivibrio sp. INlla16 | 0 | Not detected | 0.230 | -0.059 | 0.520 | 0.120 |
| Butyrivibrio sp. INlla21 | 0 | Not detected | 0.079 | -0.228 | 0.386 | 0.615 |
| Butyrivibrio sp. LB2008 | 0 | Not detected | 0.045 | -0.266 | 0.355 | 0.777 |
| Butyrivibrio sp. M55 | 0 | Not detected | 0.060 | -0.265 | 0.385 | 0.719 |
| Butyrivibrio sp. NC2002 | 0 | Not detected | -0.085 | -0.415 | 0.246 | 0.615 |
| Butyrivibrio sp. NC2007 | 0 | Not detected | 0.084 | -0.225 | 0.392 | 0.595 |
| Butyrivibrio sp. NC3005 | 0 | Not detected | 0.437 | 0.003 | 0.871 | 0.049 |
| Butyrivibrio sp. VCD2006 | 0 | Not detected | 0.121 | -0.184 | 0.425 | 0.438 |
| Butyrivibrio sp. WCD2001 | 0 | Not detected | 0.340 | 0.029 | 0.652 | 0.033 |
| Butyrivibrio sp. WCD3002 | 0 | Not detected | -0.083 | -0.375 | 0.208 | 0.576 |
| Butyrivibrio sp. ob235 | 0 | Not detected | 0.076 | -0.213 | 0.365 | 0.607 |
| Campylobacter coli | 0 | Not detected | 0.023 | -0.243 | 0.289 | 0.866 |
| Campylobacter hominis | 0 | Not detected | -0.214 | -0.679 | 0.250 | 0.367 |
| Candidatus Azobacteroides pseudotrichony | 0 | Not detected | 0.059 | -0.217 | 0.335 | 0.676 |
| Candidatus Gastranaerophilales bacterium | 0 | Not detected | -0.067 | -0.505 | 0.371 | 0.764 |
| Candidatus Melainabacteria bacterium LE | 0 | Not detected | 0.046 | -0.240 | 0.333 | 0.751 |
| Candidatus Melainabacteria bacterium ME | 0 | Not detected | 0.159 | -0.338 | 0.655 | 0.531 |
| Candidatus Saccharibacteria oral taxon TM | 0 | Not detected | 0.259 | -0.090 | 0.608 | 0.147 |

| Candidatus Soleaferrea massiliensis | 0 | Not detected | -0.143 | -0.421 | 0.134 | 0.312 |
| --- | --- | --- | --- | --- | --- | --- |
| Candidatus Stoquefichus sp. SB1 | 0 | Not detected | -0.052 | -0.319 | 0.216 | 0.705 |
| Capnocytophaga granulosa | 0 | Not detected | 0.006 | -0.326 | 0.339 | 0.971 |
| Capnocytophaga sp. oral taxon 329 | 0 | Not detected | 0.541 | 0.064 | 1.018 | 0.027 |
| Capnocytophaga sp. oral taxon 863 | 0 | Not detected | 0.008 | -0.422 | 0.439 | 0.969 |
| Capnocytophaga sputigena | 0 | Not detected | 0.103 | -0.172 | 0.378 | 0.464 |
| Carnobacterium gallinarum | 0 | Not detected | -0.052 | -0.463 | 0.359 | 0.805 |
| Catabacter hongkongensis | 0 | Not detected | -0.244 | -0.670 | 0.183 | 0.264 |
| Catenibacterium mitsuokai | 0 | Not detected | 0.287 | -0.375 | 0.949 | 0.396 |
| Catenibacterium sp. CAG:290 | 0 | Not detected | 0.398 | -0.407 | 1.204 | 0.333 |
| Catonella morbi | 0 | Not detected | -0.006 | -0.229 | 0.218 | 0.959 |
| Cedecea neteri | 0 | Not detected | 0.012 | -0.381 | 0.405 | 0.953 |
| Cellulomonas carbonis | 0 | Not detected | 0.217 | -0.255 | 0.688 | 0.369 |
| Cellulomonas flavigena | 0 | Not detected | -0.285 | -0.743 | 0.174 | 0.225 |
| Cellulosilyticum lentocellum | 0 | Not detected | -0.029 | -0.362 | 0.304 | 0.864 |
| Cellulosilyticum ruminicola | 0 | Not detected | 0.062 | -0.275 | 0.399 | 0.719 |
| Chlamydia psittaci | 0 | Not detected | 0.026 | -0.293 | 0.345 | 0.873 |
| Christensenella sp. AF73-05CM02 | 0 | Not detected | 0.088 | -0.333 | 0.510 | 0.682 |
| Christensenellaceae bacterium Phil1 | 0 | Not detected | -0.167 | -0.484 | 0.151 | 0.304 |
| Christensenellaceae bacterium Phil10 | 0 | Not detected | 0.001 | -0.521 | 0.522 | 0.998 |
| Christensenellaceae bacterium Phil7 | 0 | Not detected | -0.120 | -0.638 | 0.397 | 0.648 |
| Chryseobacterium gallinarum | 0 | Not detected | 0.000 | -0.602 | 0.602 | 1.000 |
| Chryseobacterium greenlandense | 0 | Not detected | 0.554 | 0.019 | 1.089 | 0.044 |
| Citrobacter amalonaticus | 0 | Not detected | -0.053 | -0.634 | 0.528 | 0.858 |
| Citrobacter freundii | 0 | Not detected | -0.084 | -0.724 | 0.556 | 0.798 |
| Cloacibacillus porcorum | 0 | Not detected | -0.047 | -0.538 | 0.445 | 0.852 |
| Cloacibacterium normanense | 0 | Not detected | 0.329 | -0.125 | 0.784 | 0.156 |
| Clostridia bacterium UC5.1-1C12 | 0 | Not detected | -0.212 | -0.762 | 0.338 | 0.451 |
| Clostridia bacterium UC5.1-1D1 | 0 | Not detected | -0.128 | -0.317 | 0.062 | 0.188 |
| Clostridia bacterium UC5.1-1E11 | 0 | Not detected | -0.039 | -0.243 | 0.164 | 0.704 |
| Clostridiales bacterium 1_7_47FAA | 0 | Not detected | -0.109 | -0.321 | 0.102 | 0.312 |
| Clostridiales bacterium 59_14 | 0 | Not detected | 0.149 | -0.199 | 0.496 | 0.403 |
| Clostridiales bacterium DTU035 | 0 | Not detected | 0.101 | -0.220 | 0.423 | 0.537 |
| Clostridiales bacterium DTU036 | 0 | Not detected | 0.039 | -0.252 | 0.330 | 0.794 |
| Clostridiales bacterium DTU089 | 0 | Not detected | -0.199 | -0.558 | 0.160 | 0.278 |
| Clostridiales bacterium Firm_03 | 0 | Not detected | -0.396 | -0.872 | 0.081 | 0.105 |
| Clostridiales bacterium Firm_16 | 0 | Not detected | -0.202 | -0.691 | 0.287 | 0.420 |
| Clostridiales bacterium NK3B98 | 23 | Not detected | -0.451 | -0.830 | -0.072 | 0.020 |
| Clostridiales bacterium S5-A14a | 0 | Not detected | 0.030 | -0.170 | 0.230 | 0.768 |
| Clostridiales bacterium SIT11 | 0 | Not detected | -0.143 | -0.515 | 0.230 | 0.453 |
| Clostridiales bacterium VE202-08 | 0 | Not detected | 0.015 | -0.191 | 0.221 | 0.889 |
| Clostridioides difficile | 0 | Not detected | 0.000 | -0.174 | 0.174 | 0.998 |
| Clostridioides mangenotii | 0 | Not detected | 0.002 | -0.331 | 0.334 | 0.992 |
| Clostridium baratii | 0 | Not detected | 0.531 | 0.174 | 0.887 | 0.004 |

| Clostridium beijerinckii | 0 | Not detected | 0.347 | 0.038 | 0.656 | 0.028 |
| --- | --- | --- | --- | --- | --- | --- |
| Clostridium bolteae CAG:59 | 0 | Not detected | -0.223 | -0.561 | 0.116 | 0.199 |
| Clostridium botulinum | 0 | Not detected | 0.168 | -0.200 | 0.537 | 0.371 |
| Clostridium cadaveris | 0 | Not detected | -0.023 | -0.432 | 0.386 | 0.912 |
| Clostridium celatum | 0 | Not detected | 0.535 | -0.067 | 1.136 | 0.083 |
| Clostridium cellulovorans | 0 | Not detected | -0.001 | -0.284 | 0.281 | 0.992 |
| Clostridium clostridioforme CAG:132 | 0 | Not detected | -0.267 | -0.749 | 0.214 | 0.278 |
| Clostridium clostridioforme CAG:511 | 0 | Not detected | -0.276 | -0.717 | 0.165 | 0.221 |
| Clostridium disporicum | 0 | Not detected | 0.250 | -0.105 | 0.606 | 0.169 |
| Clostridium ihumii | 0 | Not detected | 0.063 | -0.244 | 0.371 | 0.687 |
| Clostridium kluyveri | 0 | Not detected | 0.302 | -0.127 | 0.732 | 0.169 |
| Clostridium leptum CAG:27 | 0 | Not detected | -0.476 | -1.017 | 0.065 | 0.086 |
| Clostridium lundense | 0 | Not detected | 0.149 | -0.283 | 0.581 | 0.499 |
| Clostridium nexile CAG:348 | 0 | Not detected | -0.153 | -0.524 | 0.218 | 0.419 |
| Clostridium paraputrificum | 720 | 0.6 | 0.716 | 0.253 | 1.180 | 0.003 |
| Clostridium perfringens | 0 | Not detected | 0.631 | 0.048 | 1.214 | 0.035 |
| Clostridium phoceensis | 0 | Not detected | -0.022 | -0.304 | 0.260 | 0.880 |
| Clostridium polynesiense | 0 | Not detected | -0.022 | -0.471 | 0.427 | 0.923 |
| Clostridium sartagoforme | 0 | Not detected | 0.407 | 0.036 | 0.779 | 0.033 |
| Clostridium sp. 44_14 | 0 | Not detected | -0.019 | -0.409 | 0.371 | 0.923 |
| Clostridium sp. ASF356 | 0 | Not detected | -0.050 | -0.482 | 0.383 | 0.822 |
| Clostridium sp. CAG:122 | 0 | Not detected | 0.213 | -0.318 | 0.743 | 0.433 |
| Clostridium sp. CAG:12237_41 | 0 | Not detected | 0.124 | -0.296 | 0.545 | 0.562 |
| Clostridium sp. CAG:127 | 0 | Not detected | -0.345 | -0.972 | 0.282 | 0.282 |
| Clostridium sp. CAG:138 | 0 | Not detected | 0.096 | -0.723 | 0.915 | 0.818 |
| Clostridium sp. CAG:167 | 0 | Not detected | 0.127 | -0.540 | 0.793 | 0.709 |
| Clostridium sp. CAG:226 | 0 | Not detected | -0.527 | -1.120 | 0.066 | 0.083 |
| Clostridium sp. CAG:230 | 0 | Not detected | 0.233 | -0.221 | 0.686 | 0.316 |
| Clostridium sp. CAG:242 | 0 | Not detected | -0.202 | -0.778 | 0.374 | 0.493 |
| Clostridium sp. CAG:245 | 0 | Not detected | 0.468 | -0.381 | 1.318 | 0.281 |
| Clostridium sp. CAG:245_30_32 | 0 | Not detected | 0.170 | -0.227 | 0.566 | 0.402 |
| Clostridium sp. CAG:264 | 0 | Not detected | 0.108 | -0.451 | 0.668 | 0.704 |
| Clostridium sp. CAG:277 | 0 | Not detected | -0.078 | -0.458 | 0.302 | 0.687 |
| Clostridium sp. CAG:299 | 0 | Not detected | 0.351 | -0.190 | 0.891 | 0.205 |
| Clostridium sp. CAG:307_30_263 | 0 | Not detected | 0.075 | -0.299 | 0.448 | 0.696 |
| Clostridium sp. CAG:349 | 0 | Not detected | 0.151 | -0.455 | 0.757 | 0.626 |
| Clostridium sp. CAG:352 | 0 | Not detected | 0.065 | -0.452 | 0.582 | 0.806 |
| Clostridium sp. CAG:413 | 0 | Not detected | 0.091 | -0.515 | 0.697 | 0.770 |
| Clostridium sp. CAG:448 | 0 | Not detected | -0.014 | -0.536 | 0.509 | 0.959 |
| Clostridium sp. CAG:451 | 0 | Not detected | -0.284 | -0.908 | 0.340 | 0.373 |
| Clostridium sp. CAG:465 | 0 | Not detected | 0.309 | -0.248 | 0.866 | 0.278 |
| Clostridium sp. CAG:505 | 0 | Not detected | -0.086 | -0.737 | 0.565 | 0.796 |
| Clostridium sp. CAG:568 | 0 | Not detected | 0.002 | -0.482 | 0.486 | 0.994 |
| Clostridium sp. CAG:58 | 0 | Not detected | -0.375 | -0.827 | 0.077 | 0.106 |

| Clostridium sp. CAG:590 | 0 | Not detected | -0.106 | -0.446 | 0.233 | 0.541 |
| --- | --- | --- | --- | --- | --- | --- |
| Clostridium sp. CAG:594 | 0 | Not detected | 0.173 | -0.410 | 0.756 | 0.561 |
| Clostridium sp. CAG:628 | 0 | Not detected | 0.142 | -0.394 | 0.678 | 0.604 |
| Clostridium sp. CAG:62_40_43 | 0 | Not detected | 0.197 | -0.554 | 0.949 | 0.607 |
| Clostridium sp. CAG:632 | 0 | Not detected | 0.206 | -0.233 | 0.646 | 0.358 |
| Clostridium sp. CAG:678 | 0 | Not detected | 0.400 | -0.111 | 0.910 | 0.126 |
| Clostridium sp. CAG:768 | 0 | Not detected | 0.235 | -0.057 | 0.526 | 0.116 |
| Clostridium sp. CAG:780 | 0 | Not detected | 0.520 | -0.120 | 1.159 | 0.112 |
| Clostridium sp. CAG:793 | 0 | Not detected | 0.268 | -0.274 | 0.810 | 0.333 |
| Clostridium sp. CAG:81 | 0 | Not detected | 0.033 | -0.335 | 0.402 | 0.860 |
| Clostridium sp. CAG:91 | 0 | Not detected | 0.339 | -0.232 | 0.911 | 0.246 |
| Clostridium sp. CAG:964 | 0 | Not detected | 0.093 | -0.293 | 0.480 | 0.636 |
| Clostridium sp. HGF2 | 0 | Not detected | -0.042 | -0.233 | 0.149 | 0.666 |
| Clostridium sp. K4410.MGS-306 | 0 | Not detected | 0.158 | -0.080 | 0.396 | 0.195 |
| Clostridium sp. KLE 1755 | 0 | Not detected | -0.005 | -0.300 | 0.291 | 0.976 |
| Clostridium sp. KNHs205 | 0 | Not detected | -0.073 | -0.219 | 0.073 | 0.328 |
| Clostridium sp. KNHs209 | 0 | Not detected | -0.037 | -0.187 | 0.114 | 0.632 |
| Clostridium sp. KNHs214 | 0 | Not detected | -0.273 | -0.555 | 0.009 | 0.059 |
| Clostridium sp. L2-50 | 0 | Not detected | -0.036 | -0.228 | 0.156 | 0.710 |
| Clostridium sp. M62/1 | 0 | Not detected | -0.051 | -0.209 | 0.108 | 0.531 |
| Clostridium sp. SY8519 | 0 | Not detected | 0.032 | -0.156 | 0.219 | 0.741 |
| Clostridium sulfidigenes | 0 | Not detected | -0.209 | -0.512 | 0.094 | 0.178 |
| Collinsella aerofaciens | 0 | Not detected | 0.100 | -0.437 | 0.637 | 0.716 |
| Collinsella intestinalis | 0 | Not detected | 0.067 | -0.568 | 0.702 | 0.837 |
| Collinsella sp. CAG:166 | 0 | Not detected | 0.139 | -0.457 | 0.735 | 0.648 |
| Collinsella sp. MS5 | 0 | Not detected | -0.140 | -0.545 | 0.264 | 0.497 |
| Collinsella stercoris | 0 | Not detected | -0.179 | -0.701 | 0.343 | 0.502 |
| Collinsella tanakaei | 0 | Not detected | 0.011 | -0.399 | 0.420 | 0.959 |
| Comamonas kerstersii | 0 | Not detected | 0.041 | -0.296 | 0.378 | 0.813 |
| Coprobacillus sp. CAG:605 | 0 | Not detected | 0.185 | -0.342 | 0.711 | 0.493 |
| Coprobacillus sp. D6 | 0 | Not detected | -0.016 | -0.284 | 0.252 | 0.907 |
| Coprobacter secundus | 0 | Not detected | -0.079 | -0.287 | 0.128 | 0.456 |
| Coprococcus comes | 0 | Not detected | 0.039 | -0.162 | 0.240 | 0.706 |
| Coprococcus eutactus | 0 | Not detected | 0.216 | -0.019 | 0.450 | 0.072 |
| Coprococcus eutactus CAG:665 | 0 | Not detected | 0.540 | -0.003 | 1.083 | 0.052 |
| Coprococcus sp. 43_8 | 0 | Not detected | 0.106 | -0.326 | 0.539 | 0.630 |
| Coprococcus sp. CAG:131 | 0 | Not detected | 0.619 | 0.076 | 1.162 | 0.026 |
| Coprococcus sp. CAG:131_42_139 | 591 | Not detected | 0.681 | 0.118 | 1.243 | 0.018 |
| Coprococcus sp. HPP0048 | 0 | Not detected | -0.090 | -0.414 | 0.234 | 0.587 |
| Coriobacteriaceae bacterium 68-1-3 | 0 | Not detected | 0.297 | -0.088 | 0.682 | 0.132 |
| Coriobacteriaceae bacterium BV3Ac1 | 0 | Not detected | -0.008 | -0.503 | 0.487 | 0.976 |
| Coriobacteriales bacterium DNF00809 | 0 | Not detected | -0.328 | -0.749 | 0.093 | 0.128 |
| Coriobacterium glomerans | 0 | Not detected | -0.160 | -0.513 | 0.193 | 0.375 |
| Corynebacterium amycolatum | 0 | Not detected | -0.023 | -0.345 | 0.299 | 0.889 |

| Corynebacterium durum | 0 | Not detected | 0.236 | -0.176 | 0.649 | 0.262 |
| --- | --- | --- | --- | --- | --- | --- |
| Corynebacterium glucuronolyticum | 0 | Not detected | 0.034 | -0.341 | 0.410 | 0.858 |
| Criibacterium bergeronii | 0 | Not detected | -0.174 | -0.429 | 0.082 | 0.183 |
| Cronobacter sakazakii | 0 | Not detected | 0.066 | -0.504 | 0.637 | 0.820 |
| Culturomica massiliensis | 0 | Not detected | 0.014 | -0.230 | 0.258 | 0.912 |
| Cutibacterium acnes | 0 | Not detected | 0.329 | -0.060 | 0.718 | 0.098 |
| Defluviitalea phaphyphila | 0 | Not detected | 0.286 | 0.012 | 0.561 | 0.042 |
| Dendrosporobacter quercicolus | 0 | Not detected | 0.056 | -0.229 | 0.341 | 0.701 |
| Denitrobacterium detoxificans | 0 | Not detected | -0.101 | -0.448 | 0.245 | 0.566 |
| Desulfovibrio desulfuricans | 0 | Not detected | -0.136 | -0.709 | 0.438 | 0.643 |
| Desulfovibrio fairfieldensis | 0 | Not detected | 0.256 | -0.318 | 0.830 | 0.383 |
| Desulfovibrio piger | 0 | Not detected | -0.318 | -1.122 | 0.486 | 0.439 |
| Desulfovibrio vulgaris | 0 | Not detected | 0.185 | -0.145 | 0.515 | 0.272 |
| Dialister invisus | 0 | Not detected | -0.421 | -1.415 | 0.572 | 0.407 |
| Dialister invisus CAG:218 | 0 | Not detected | -0.547 | -1.566 | 0.471 | 0.293 |
| Dialister micraerophilus | 0 | Not detected | -0.294 | -0.824 | 0.237 | 0.279 |
| Dialister sp. CAG:357 | 0 | Not detected | -0.380 | -1.204 | 0.445 | 0.368 |
| Dialister sp. CAG:486 | 0 | Not detected | -0.075 | -0.737 | 0.586 | 0.823 |
| Dialister sp. CAG:588 | 0 | Not detected | -0.129 | -0.574 | 0.316 | 0.570 |
| Dialister succinatiphilus | 0 | Not detected | -0.174 | -0.683 | 0.334 | 0.503 |
| Dielma fastidiosa | 0 | Not detected | -0.234 | -0.701 | 0.233 | 0.328 |
| Dorea formicigenerans | 0 | Not detected | 0.145 | -0.054 | 0.344 | 0.155 |
| Dorea longicatena | 0 | Not detected | -0.023 | -0.299 | 0.252 | 0.869 |
| Dorea sp. 42_8 | 0 | Not detected | 0.216 | -0.093 | 0.526 | 0.172 |
| Dorea sp. 5-2 | 0 | Not detected | 0.018 | -0.123 | 0.158 | 0.806 |
| Dorea sp. CAG:105 | 0 | Not detected | -0.006 | -0.408 | 0.397 | 0.978 |
| Draconibacterium orientale | 1 | Not detected | -0.153 | -0.423 | 0.117 | 0.266 |
| Drancourtella massiliensis | 0 | Not detected | -0.218 | -0.603 | 0.167 | 0.269 |
| Dubosiella newyorkensis | 0 | Not detected | -0.113 | -0.408 | 0.182 | 0.455 |
| Dysgonomonas gadei | 0 | Not detected | 0.128 | -0.116 | 0.372 | 0.304 |
| Dysgonomonas mossii | 0 | Not detected | -0.165 | -0.477 | 0.147 | 0.302 |
| Dysgonomonas sp. BGC7 | 0 | Not detected | -0.478 | -0.876 | -0.080 | 0.019 |
| Dysgonomonas sp. HGC4 | 0 | Not detected | -0.168 | -0.492 | 0.157 | 0.312 |
| Eggerthella lenta | 0 | Not detected | -0.191 | -0.667 | 0.285 | 0.433 |
| Eggerthella sp. 51_9 | 0 | Not detected | 0.204 | -0.292 | 0.700 | 0.420 |
| Eggerthella sp. CAG:209 | 0 | Not detected | 0.623 | -0.067 | 1.314 | 0.078 |
| Eggerthella sp. YY7918 | 0 | Not detected | -0.175 | -0.557 | 0.206 | 0.369 |
| Eggerthellaceae bacterium AT8 | 0 | Not detected | -0.018 | -0.259 | 0.223 | 0.883 |
| Eggerthia catenaformis | 0 | Not detected | 0.071 | -0.368 | 0.511 | 0.751 |
| Eisenbergiella tayi | 0 | Not detected | -0.066 | -0.492 | 0.359 | 0.760 |
| Elizabethkingia anophelis | 0 | Not detected | 0.331 | -0.100 | 0.762 | 0.133 |
| Emergencia timonensis | 0 | Not detected | -0.097 | -0.280 | 0.085 | 0.297 |
| Empedobacter brevis | 0 | Not detected | 0.333 | -0.298 | 0.963 | 0.302 |
| Enorma massiliensis | 0 | Not detected | -0.074 | -0.308 | 0.160 | 0.535 |

| Enorma timonensis | 0 | Not detected | -0.056 | -0.396 | 0.285 | 0.749 |
| --- | --- | --- | --- | --- | --- | --- |
| Enterobacter cloacae | 0 | Not detected | 0.279 | -0.354 | 0.911 | 0.389 |
| Enterobacter lignolyticus | 0 | Not detected | 0.098 | -0.412 | 0.609 | 0.706 |
| Enterobacter sp. 638 | 0 | Not detected | -0.013 | -0.465 | 0.439 | 0.954 |
| Enterococcus avium | 0 | Not detected | -0.001 | -0.414 | 0.413 | 0.996 |
| Enterococcus cecorum | 0 | Not detected | 0.084 | -0.285 | 0.453 | 0.655 |
| Enterococcus columbae | 0 | Not detected | 0.243 | -0.182 | 0.669 | 0.264 |
| Enterococcus durans | 0 | Not detected | 0.045 | -0.386 | 0.475 | 0.838 |
| Enterococcus faecalis | 0 | Not detected | 0.180 | -0.202 | 0.563 | 0.357 |
| Enterococcus faecium | 0 | Not detected | 0.221 | -0.175 | 0.617 | 0.276 |
| Enterococcus gallinarum | 0 | Not detected | -0.216 | -0.606 | 0.173 | 0.277 |
| Enterococcus gilvus | 0 | Not detected | -0.001 | -0.372 | 0.369 | 0.995 |
| Enterococcus italicus | 0 | Not detected | -0.214 | -0.606 | 0.178 | 0.285 |
| Enterococcus massiliensis | 0 | Not detected | -0.166 | -0.552 | 0.220 | 0.399 |
| Enterococcus sp. HMSC064A12 | 0 | Not detected | 0.028 | -0.386 | 0.441 | 0.896 |
| Enterorhabdus caecimuris | 0 | Not detected | -0.052 | -0.505 | 0.402 | 0.823 |
| Enterorhabdus mucosicola | 0 | Not detected | -0.229 | -0.639 | 0.181 | 0.275 |
| Epulopiscium sp. 'N.t. morphotype B' | 0 | Not detected | -0.030 | -0.293 | 0.232 | 0.823 |
| Erwinia oleae | 0 | Not detected | -0.341 | -0.758 | 0.075 | 0.109 |
| Erysipelatoclostridium ramosum | 0 | Not detected | 0.188 | -0.389 | 0.766 | 0.524 |
| Erysipelotrichaceae bacterium 21_3 | 0 | Not detected | -0.147 | -0.366 | 0.071 | 0.188 |
| Erysipelotrichaceae bacterium 5_2_54FAA | 0 | Not detected | -0.195 | -0.426 | 0.037 | 0.100 |
| Erysipelotrichaceae bacterium CAG:64 | 0 | Not detected | -0.166 | -0.559 | 0.228 | 0.410 |
| Erysipelotrichaceae bacterium I46 | 0 | Not detected | -0.143 | -0.369 | 0.083 | 0.216 |
| Escherichia coli | 0 | Not detected | -0.366 | -1.004 | 0.273 | 0.263 |
| Escherichia vulneris | 0 | Not detected | 0.057 | -0.363 | 0.476 | 0.791 |
| Ethanoligenens harbinense | 0 | Not detected | -0.122 | -0.526 | 0.282 | 0.555 |
| Eubacterium dolichum CAG:375 | 0 | Not detected | 0.269 | -0.384 | 0.921 | 0.420 |
| Eubacterium limosum | 0 | Not detected | -0.299 | -0.616 | 0.018 | 0.066 |
| Eubacterium plexicaudatum | 0 | Not detected | 0.052 | -0.100 | 0.203 | 0.507 |
| Eubacterium pyruvativorans | 0 | Not detected | -0.024 | -0.256 | 0.208 | 0.839 |
| Eubacterium ramulus | 0 | Not detected | -0.036 | -0.217 | 0.144 | 0.693 |
| Eubacterium sp. 14-2 | 0 | Not detected | 0.042 | -0.112 | 0.197 | 0.592 |
| Eubacterium sp. 3_1_31 | 0 | Not detected | -0.128 | -0.389 | 0.133 | 0.337 |
| Eubacterium sp. 45_250 | 0 | Not detected | 0.091 | -0.565 | 0.747 | 0.785 |
| Eubacterium sp. AB3007 | 0 | Not detected | 0.509 | -0.016 | 1.033 | 0.059 |
| Eubacterium sp. CAG:115 | 0 | Not detected | 0.041 | -0.823 | 0.904 | 0.927 |
| Eubacterium sp. CAG:146 | 0 | Not detected | -0.084 | -0.529 | 0.361 | 0.712 |
| Eubacterium sp. CAG:180 | 0 | Not detected | -0.118 | -0.909 | 0.674 | 0.771 |
| Eubacterium sp. CAG:192 | 0 | Not detected | -0.170 | -0.795 | 0.455 | 0.595 |
| Eubacterium sp. CAG:202 | 0 | Not detected | 0.069 | -0.496 | 0.634 | 0.811 |
| Eubacterium sp. CAG:248 | 0 | Not detected | 0.072 | -0.419 | 0.563 | 0.774 |
| Eubacterium sp. CAG:251 | 0 | Not detected | -0.188 | -0.986 | 0.611 | 0.646 |
| Eubacterium sp. CAG:252 | 0 | Not detected | 0.000 | -0.482 | 0.482 | 1.000 |

| Eubacterium sp. CAG:581 | 0 | Not detected | -0.264 | -0.798 | 0.270 | 0.333 |
| --- | --- | --- | --- | --- | --- | --- |
| Eubacterium sp. CAG:603 | 0 | Not detected | -0.043 | -0.379 | 0.294 | 0.804 |
| Eubacterium sp. CAG:786 | 0 | Not detected | -0.129 | -0.748 | 0.490 | 0.684 |
| Eubacterium sp. CAG:841 | 0 | Not detected | -0.466 | -1.098 | 0.167 | 0.150 |
| Eubacterium sp. ER2 | 0 | Not detected | -0.123 | -0.293 | 0.047 | 0.157 |
| Eubacterium ventriosum | 0 | Not detected | 0.099 | -0.159 | 0.358 | 0.451 |
| Eubacterium xylanophilum | 0 | Not detected | 0.141 | -0.189 | 0.471 | 0.403 |
| Facklamia hominis | 0 | Not detected | -0.050 | -0.442 | 0.342 | 0.803 |
| Facklamia ignava | 0 | Not detected | -0.101 | -0.469 | 0.267 | 0.592 |
| Facklamia languida | 0 | Not detected | 0.131 | -0.211 | 0.473 | 0.453 |
| Faecalibacterium prausnitzii | 0 | Not detected | 0.000 | -0.347 | 0.348 | 0.998 |
| Faecalibacterium sp. CAG:1138 | 0 | Not detected | -0.289 | -0.722 | 0.143 | 0.191 |
| Faecalibacterium sp. CAG:82-related_59_ | 0 | Not detected | -0.015 | -0.535 | 0.504 | 0.955 |
| Faecalibaculum rodentium | 0 | Not detected | -0.027 | -0.446 | 0.393 | 0.901 |
| Faecalicoccus pleomorphus | 0 | Not detected | -0.011 | -0.287 | 0.266 | 0.940 |
| Faecalitalea cylindroides | 0 | Not detected | 0.057 | -0.108 | 0.222 | 0.497 |
| Fenollaria massiliensis | 0 | Not detected | -0.440 | -0.910 | 0.029 | 0.067 |
| Fermentimonas caenicola | 0 | Not detected | 0.028 | -0.384 | 0.440 | 0.895 |
| Fibrobacter sp. UWB12 | 0 | Not detected | -0.014 | -0.264 | 0.237 | 0.915 |
| Fibrobacter sp. UWCM | 0 | Not detected | 0.041 | -0.293 | 0.375 | 0.811 |
| Fibrobacter sp. UWOV1 | 0 | Not detected | -0.079 | -0.367 | 0.210 | 0.594 |
| Fibrobacterales bacterium Fibro_02 | 0 | Not detected | 0.033 | -0.260 | 0.326 | 0.824 |
| Fibrobacteria bacterium AD80 | 0 | Not detected | -0.041 | -0.348 | 0.266 | 0.793 |
| Filifactor alocis | 0 | Not detected | 0.001 | -0.431 | 0.432 | 0.998 |
| Finegoldia magna | 0 | Not detected | -0.397 | -0.843 | 0.049 | 0.082 |
| Firmicutes bacterium ASF500 | 0 | Not detected | -0.080 | -0.258 | 0.097 | 0.375 |
| Firmicutes bacterium CAG:102 | 0 | Not detected | 0.152 | -0.522 | 0.826 | 0.659 |
| Firmicutes bacterium CAG:137 | 0 | Not detected | -0.049 | -0.763 | 0.665 | 0.893 |
| Firmicutes bacterium CAG:137_57_8 | 0 | Not detected | -0.462 | -1.072 | 0.147 | 0.138 |
| Firmicutes bacterium CAG:145 | 0 | Not detected | 0.128 | -0.563 | 0.820 | 0.716 |
| Firmicutes bacterium CAG:176 | 0 | Not detected | 0.001 | -0.732 | 0.735 | 0.997 |
| Firmicutes bacterium CAG:176_59_8 | 0 | Not detected | -0.064 | -0.741 | 0.613 | 0.853 |
| Firmicutes bacterium CAG:227 | 0 | Not detected | 0.439 | -0.076 | 0.953 | 0.096 |
| Firmicutes bacterium CAG:238 | 0 | Not detected | 0.709 | 0.148 | 1.271 | 0.014 |
| Firmicutes bacterium CAG:24 | 0 | Not detected | 0.100 | -0.371 | 0.572 | 0.677 |
| Firmicutes bacterium CAG:240 | 0 | Not detected | 0.073 | -0.580 | 0.727 | 0.827 |
| Firmicutes bacterium CAG:270 | 0 | Not detected | -0.093 | -0.602 | 0.415 | 0.719 |
| Firmicutes bacterium CAG:272 | 0 | Not detected | -0.385 | -0.936 | 0.165 | 0.171 |
| Firmicutes bacterium CAG:272_52_7 | 0 | Not detected | -0.105 | -0.556 | 0.347 | 0.650 |
| Firmicutes bacterium CAG:308 | 0 | Not detected | 0.036 | -0.315 | 0.387 | 0.841 |
| Firmicutes bacterium CAG:313 | 0 | Not detected | -0.185 | -0.872 | 0.502 | 0.598 |
| Firmicutes bacterium CAG:41 | 0 | Not detected | 0.491 | -0.083 | 1.064 | 0.095 |
| Firmicutes bacterium CAG:466 | 0 | Not detected | -0.194 | -0.662 | 0.275 | 0.418 |
| Firmicutes bacterium CAG:475 | 0 | Not detected | -0.206 | -0.629 | 0.217 | 0.340 |

| Firmicutes bacterium CAG:536 | 0 | Not detected | 0.245 | -0.272 | 0.761 | 0.354 |
| --- | --- | --- | --- | --- | --- | --- |
| Firmicutes bacterium CAG:555 | 0 | Not detected | 0.184 | -0.359 | 0.728 | 0.507 |
| Firmicutes bacterium CAG:65 | 0 | Not detected | 0.155 | -0.359 | 0.669 | 0.555 |
| Firmicutes bacterium CAG:791 | 0 | Not detected | 0.073 | -0.210 | 0.356 | 0.615 |
| Firmicutes bacterium CAG:83 | 0 | Not detected | 0.258 | -0.366 | 0.882 | 0.418 |
| Firmicutes bacterium CAG:882 | 0 | Not detected | 0.183 | -0.180 | 0.546 | 0.324 |
| Firmicutes bacterium CAG:884 | 0 | Not detected | 0.014 | -0.427 | 0.455 | 0.950 |
| Firmicutes bacterium CAG:94 | 0 | Not detected | -0.042 | -0.681 | 0.596 | 0.897 |
| Firmicutes bacterium M10-2 | 0 | Not detected | 0.031 | -0.403 | 0.464 | 0.890 |
| Firmicutes bacterium ZOR0006 | 0 | Not detected | 0.127 | -0.327 | 0.581 | 0.585 |
| Flavonifractor plautii | 0 | Not detected | -0.296 | -0.655 | 0.062 | 0.106 |
| Fournierella massiliensis | 0 | Not detected | 0.008 | -0.182 | 0.199 | 0.931 |
| Franconibacter pulveris | 0 | Not detected | -0.002 | -0.383 | 0.380 | 0.993 |
| Fusicatenibacter saccharivorans | 0 | Not detected | 0.071 | -0.141 | 0.283 | 0.513 |
| Fusobacterium mortiferum | 0 | Not detected | -0.069 | -0.685 | 0.547 | 0.827 |
| Fusobacterium necrophorum | 0 | Not detected | -0.204 | -0.617 | 0.208 | 0.332 |
| Fusobacterium nucleatum | 0 | Not detected | 0.191 | -0.345 | 0.727 | 0.485 |
| Fusobacterium periodonticum | 0 | Not detected | 0.474 | 0.012 | 0.936 | 0.046 |
| Gabonia massiliensis | 0 | Not detected | -0.128 | -0.494 | 0.239 | 0.496 |
| Gabonibacter massiliensis | 0 | Not detected | 0.303 | -0.097 | 0.702 | 0.138 |
| Gallibacterium anatis | 0 | Not detected | 0.020 | -0.311 | 0.350 | 0.908 |
| Gardnerella vaginalis | 0 | Not detected | -0.066 | -0.509 | 0.378 | 0.772 |
| Gemella haemolysans | 0 | Not detected | 0.205 | -0.224 | 0.635 | 0.350 |
| Gemella sanguinis | 0 | Not detected | 0.481 | -0.041 | 1.003 | 0.072 |
| Gemmata obscuriglobus | 0 | Not detected | -0.136 | -0.488 | 0.215 | 0.449 |
| Geofilum rubicundum | 0 | Not detected | -0.163 | -0.499 | 0.173 | 0.343 |
| Globicatella sanguinis | 0 | Not detected | -0.042 | -0.408 | 0.324 | 0.822 |
| Gordonibacter pamelaeae | 0 | Not detected | -0.006 | -0.325 | 0.312 | 0.969 |
| Granulicatella adiacens | 0 | Not detected | 0.509 | 0.014 | 1.004 | 0.045 |
| Granulicatella elegans | 0 | Not detected | -0.179 | -0.591 | 0.234 | 0.396 |
| Haemophilus influenzae | 0 | Not detected | 0.132 | -0.376 | 0.639 | 0.612 |
| Haemophilus parainfluenzae | 0 | Not detected | 0.246 | -0.468 | 0.959 | 0.500 |
| Haemophilus sputorum | 0 | Not detected | 0.482 | -0.071 | 1.035 | 0.089 |
| Hathewaya proteolytica | 0 | Not detected | -0.093 | -0.477 | 0.292 | 0.637 |
| Heliobacterium sp. N15.MGS-306 | 0 | Not detected | 0.086 | -0.125 | 0.298 | 0.424 |
| Hespellia stercorisuis | 0 | Not detected | -0.026 | -0.224 | 0.173 | 0.801 |
| Holdemanella biformis | 0 | Not detected | -0.004 | -0.338 | 0.331 | 0.984 |
| Holdemania filiformis | 0 | Not detected | -0.107 | -0.316 | 0.103 | 0.319 |
| Holdemania massiliensis | 0 | Not detected | -0.057 | -0.301 | 0.187 | 0.645 |
| Holdemania sp. Marseille-P2844 | 0 | Not detected | -0.148 | -0.397 | 0.100 | 0.242 |
| Hungatella hathewayi | 0 | Not detected | -0.162 | -0.372 | 0.047 | 0.130 |
| Hydrogenoanaerobacterium saccharovoran | 0 | Not detected | -0.084 | -0.369 | 0.200 | 0.562 |
| Idiomarina zobellii | 0 | Not detected | 0.217 | -0.293 | 0.727 | 0.405 |
| Ileibacterium valens | 0 | Not detected | -0.108 | -0.537 | 0.321 | 0.621 |

| Intestinibacter bartlettii | 0 | Not detected | 0.279 | -0.113 | 0.671 | 0.164 |
| --- | --- | --- | --- | --- | --- | --- |
| Intestinimonas butyriciproducens | 0 | Not detected | -0.058 | -0.360 | 0.244 | 0.706 |
| Intestinimonas massiliensis | 0 | Not detected | -0.007 | -0.261 | 0.248 | 0.959 |
| Jeotgalibaca sp. PTS2502 | 0 | Not detected | -0.084 | -0.350 | 0.181 | 0.534 |
| Johnsonella ignava | 0 | Not detected | -0.026 | -0.251 | 0.199 | 0.821 |
| Kallipyga massiliensis | 0 | Not detected | -0.223 | -0.603 | 0.157 | 0.251 |
| Kandleria vitulina | 0 | Not detected | 0.039 | -0.632 | 0.710 | 0.910 |
| Klebsiella oxytoca | 0 | Not detected | -0.092 | -0.771 | 0.587 | 0.791 |
| Klebsiella pneumoniae | 0 | Not detected | -0.135 | -0.806 | 0.537 | 0.695 |
| Klebsiella sp. RIT-PI-d | 0 | Not detected | -0.084 | -0.511 | 0.343 | 0.701 |
| Kluyvera ascorbata | 0 | Not detected | -0.130 | -0.698 | 0.438 | 0.654 |
| Kluyvera cryocrescens | 0 | Not detected | -0.233 | -0.831 | 0.365 | 0.446 |
| Kluyvera intermedia | 0 | Not detected | 0.012 | -0.596 | 0.621 | 0.968 |
| Kocuria sp. ZOR0020 | 0 | Not detected | -0.034 | -0.374 | 0.306 | 0.844 |
| Kosakonia sacchari | 0 | Not detected | -0.121 | -0.600 | 0.357 | 0.620 |
| Lachnoanaerobaculum saburreum | 0 | Not detected | 0.045 | -0.194 | 0.283 | 0.713 |
| Lachnoanaerobaculum sp. OBRC5-5 | 0 | Not detected | -0.031 | -0.249 | 0.187 | 0.782 |
| Lachnobacterium bovis | 0 | Not detected | -0.038 | -0.248 | 0.172 | 0.725 |
| Lachnoclostridium phytofermentans | 0 | Not detected | -0.088 | -0.241 | 0.064 | 0.258 |
| Lachnospira multipara | 0 | Not detected | 0.106 | -0.202 | 0.413 | 0.502 |
| Lachnospiraceae bacterium 10-1 | 0 | Not detected | -0.036 | -0.169 | 0.096 | 0.590 |
| Lachnospiraceae bacterium 28-4 | 0 | Not detected | 0.012 | -0.157 | 0.181 | 0.892 |
| Lachnospiraceae bacterium 2_1_46FAA | 0 | Not detected | -0.059 | -0.288 | 0.171 | 0.617 |
| Lachnospiraceae bacterium 3-1 | 0 | Not detected | -0.023 | -0.194 | 0.148 | 0.796 |
| Lachnospiraceae bacterium 3-2 | 0 | Not detected | 0.005 | -0.171 | 0.180 | 0.960 |
| Lachnospiraceae bacterium A2 | 0 | Not detected | -0.035 | -0.202 | 0.132 | 0.681 |
| Lachnospiraceae bacterium A4 | 0 | Not detected | -0.026 | -0.173 | 0.121 | 0.730 |
| Lachnospiraceae bacterium AB2028 | 0 | Not detected | -0.006 | -0.411 | 0.398 | 0.975 |
| Lachnospiraceae bacterium AC2012 | 0 | Not detected | -0.092 | -0.310 | 0.127 | 0.412 |
| Lachnospiraceae bacterium AC2014 | 0 | Not detected | -0.115 | -0.442 | 0.211 | 0.489 |
| Lachnospiraceae bacterium AC2029 | 0 | Not detected | -0.164 | -0.402 | 0.074 | 0.177 |
| Lachnospiraceae bacterium AC2031 | 0 | Not detected | -0.050 | -0.353 | 0.252 | 0.744 |
| Lachnospiraceae bacterium AC3007 | 0 | Not detected | -0.043 | -0.352 | 0.266 | 0.785 |
| Lachnospiraceae bacterium AD3010 | 0 | Not detected | -0.129 | -0.495 | 0.237 | 0.490 |
| Lachnospiraceae bacterium C6A11 | 0 | Not detected | 0.045 | -0.254 | 0.344 | 0.767 |
| Lachnospiraceae bacterium COE1 | 0 | Not detected | 0.127 | -0.085 | 0.338 | 0.242 |
| Lachnospiraceae bacterium FD2005 | 0 | Not detected | -0.060 | -0.406 | 0.285 | 0.732 |
| Lachnospiraceae bacterium FE2018 | 0 | Not detected | -0.169 | -0.575 | 0.237 | 0.416 |
| Lachnospiraceae bacterium G41 | 0 | Not detected | 0.031 | -0.229 | 0.292 | 0.815 |
| Lachnospiraceae bacterium KHCPX20 | 0 | Not detected | -0.153 | -0.575 | 0.270 | 0.479 |
| Lachnospiraceae bacterium M18-1 | 0 | Not detected | -0.028 | -0.189 | 0.133 | 0.736 |
| Lachnospiraceae bacterium NC2004 | 0 | Not detected | -0.124 | -0.479 | 0.231 | 0.494 |
| Lachnospiraceae bacterium NC2008 | 0 | Not detected | 0.148 | -0.214 | 0.511 | 0.423 |
| Lachnospiraceae bacterium ND2006 | 0 | Not detected | 0.170 | -0.159 | 0.500 | 0.313 |

| Lachnospiraceae bacterium NK3A20 | 0 | Not detected | -0.034 | -0.461 | 0.393 | 0.876 |
| --- | --- | --- | --- | --- | --- | --- |
| Lachnospiraceae bacterium NK4A179 | 0 | Not detected | 0.000 | -0.338 | 0.338 | 1.000 |
| Lachnospiraceae bacterium NLAE-zl-G231 | 0 | Not detected | -0.137 | -0.593 | 0.319 | 0.557 |
| Lachnospiraceae bacterium P6B14 | 0 | Not detected | -0.022 | -0.331 | 0.286 | 0.887 |
| Lachnospiraceae bacterium TF01-11 | 0 | Not detected | 0.146 | -0.071 | 0.364 | 0.189 |
| Lachnospiraceae bacterium TWA4 | 0 | Not detected | -0.074 | -0.299 | 0.151 | 0.519 |
| Lachnospiraceae bacterium YSB2008 | 0 | Not detected | 0.013 | -0.270 | 0.296 | 0.928 |
| Lachnospiraceae bacterium mt14 | 0 | Not detected | -0.038 | -0.334 | 0.257 | 0.800 |
| Lachnospiraceae bacterium oral taxon 500 | 0 | Not detected | -0.094 | -0.273 | 0.086 | 0.307 |
| Lactobacillus acidophilus | 0 | Not detected | 0.159 | -0.215 | 0.534 | 0.404 |
| Lactobacillus amylotrophicus | 0 | Not detected | -0.044 | -0.382 | 0.295 | 0.800 |
| Lactobacillus animalis | 0 | Not detected | -0.317 | -0.686 | 0.052 | 0.094 |
| Lactobacillus aviarius | 0 | Not detected | -0.047 | -0.291 | 0.197 | 0.707 |
| Lactobacillus casei | 0 | Not detected | -0.017 | -0.518 | 0.484 | 0.947 |
| Lactobacillus curvatus | 0 | Not detected | 0.122 | -0.243 | 0.487 | 0.513 |
| Lactobacillus delbrueckii | 0 | Not detected | -0.353 | -0.814 | 0.108 | 0.134 |
| Lactobacillus equi | 0 | Not detected | -0.107 | -0.498 | 0.284 | 0.594 |
| Lactobacillus fermentum | 0 | Not detected | 0.141 | -0.465 | 0.746 | 0.649 |
| Lactobacillus gasseri | 0 | Not detected | -0.184 | -0.793 | 0.426 | 0.555 |
| Lactobacillus helveticus | 0 | Not detected | 0.076 | -0.342 | 0.494 | 0.723 |
| Lactobacillus ingluviei | 0 | Not detected | 0.136 | -0.245 | 0.517 | 0.484 |
| Lactobacillus jensenii | 0 | Not detected | -0.250 | -0.644 | 0.145 | 0.216 |
| Lactobacillus manihotivorans | 0 | Not detected | 0.016 | -0.331 | 0.363 | 0.928 |
| Lactobacillus mucosae | 0 | Not detected | 0.146 | -0.258 | 0.549 | 0.480 |
| Lactobacillus paracasei | 0 | Not detected | 0.125 | -0.389 | 0.639 | 0.634 |
| Lactobacillus paraplantarum | 0 | Not detected | 0.227 | -0.134 | 0.588 | 0.219 |
| Lactobacillus pasteurii | 0 | Not detected | 0.118 | -0.254 | 0.489 | 0.535 |
| Lactobacillus plantarum | 0 | Not detected | 0.144 | -0.298 | 0.586 | 0.524 |
| Lactobacillus reuteri | 0 | Not detected | 0.056 | -0.466 | 0.578 | 0.834 |
| Lactobacillus rhamnosus | 0 | Not detected | -0.175 | -0.679 | 0.328 | 0.496 |
| Lactobacillus ruminis | 0 | Not detected | -0.276 | -0.714 | 0.161 | 0.217 |
| Lactobacillus ruminis CAG:367 | 0 | Not detected | -0.332 | -0.780 | 0.116 | 0.147 |
| Lactobacillus salivarius | 0 | Not detected | 0.001 | -0.538 | 0.540 | 0.997 |
| Lactobacillus vaginalis | 0 | Not detected | 0.129 | -0.354 | 0.611 | 0.602 |
| Lactococcus garvieae | 0 | Not detected | 0.327 | -0.020 | 0.673 | 0.066 |
| Lactococcus lactis | 0 | Not detected | 0.227 | -0.388 | 0.842 | 0.470 |
| Lactococcus piscium | 0 | Not detected | -0.117 | -0.535 | 0.301 | 0.583 |
| Lactonifactor longoviformis | 0 | Not detected | -0.060 | -0.202 | 0.082 | 0.410 |
| Lamprocystis purpurea | 0 | Not detected | 0.087 | -0.339 | 0.513 | 0.690 |
| Lascolabacillus massiliensis | 0 | Not detected | -0.243 | -0.565 | 0.079 | 0.140 |
| Lautropia mirabilis | 376 | Not detected | 0.683 | 0.329 | 1.036 | 0.000 |
| Lawsonia intracellularis | 0 | Not detected | -0.159 | -0.571 | 0.254 | 0.452 |
| Leclercia adecarboxylata | 0 | Not detected | 0.019 | -0.541 | 0.579 | 0.947 |
| Lelliottia amnigena | 0 | Not detected | 0.019 | -0.635 | 0.673 | 0.954 |

| Lentimicrobium saccharophilum | 0 | Not detected | 0.347 | -0.090 | 0.784 | 0.121 |
| --- | --- | --- | --- | --- | --- | --- |
| Leuconostoc lactis | 0 | Not detected | -0.181 | -0.578 | 0.216 | 0.372 |
| Leuconostoc mesenteroides | 0 | Not detected | 0.221 | -0.188 | 0.630 | 0.291 |
| Levyella massiliensis | 0 | Not detected | 0.198 | -0.047 | 0.444 | 0.115 |
| Libanicoccus massiliensis | 0 | Not detected | -0.002 | -0.432 | 0.427 | 0.992 |
| Luteimonas sp. FCS-9 | 0 | Not detected | -0.163 | -0.450 | 0.124 | 0.268 |
| Lutispora thermophila | 0 | Not detected | -0.055 | -0.417 | 0.307 | 0.767 |
| Macellibacteroides sp. HH-ZS | 0 | Not detected | -0.125 | -0.403 | 0.153 | 0.380 |
| Macrococcus caseolyticus | 0 | Not detected | 0.031 | -0.324 | 0.385 | 0.866 |
| Mageeibacillus indolicus | 0 | Not detected | -0.355 | -0.664 | -0.046 | 0.025 |
| Mangrovibacter sp. MFB070 | 0 | Not detected | -0.058 | -0.438 | 0.321 | 0.763 |
| Mannheimia haemolytica | 0 | Not detected | 0.170 | -0.129 | 0.470 | 0.267 |
| Mariniphaga anaerophila | 0 | Not detected | -0.003 | -0.329 | 0.322 | 0.984 |
| Marvinbryantia formatexigens | 0 | Not detected | 0.051 | -0.160 | 0.261 | 0.638 |
| Massilioclostridium coli | 0 | Not detected | -0.068 | -0.274 | 0.138 | 0.520 |
| Massiliomicrobiota timonensis | 0 | Not detected | -0.041 | -0.440 | 0.358 | 0.840 |
| Mediterranea massiliensis | 0 | Not detected | 0.049 | -0.214 | 0.311 | 0.716 |
| Megamonas funiformis | 0 | Not detected | 0.466 | -0.323 | 1.254 | 0.248 |
| Megasphaera cerevisiae | 0 | Not detected | 0.140 | -0.430 | 0.710 | 0.631 |
| Megasphaera elsdenii | 0 | Not detected | 0.062 | -0.646 | 0.770 | 0.865 |
| Megasphaera genomosp. type_1 | 0 | Not detected | -0.014 | -0.176 | 0.148 | 0.863 |
| Megasphaera massiliensis | 0 | Not detected | 0.334 | -0.487 | 1.156 | 0.426 |
| Megasphaera micronuciformis | 0 | Not detected | -0.035 | -0.537 | 0.467 | 0.890 |
| Megasphaera sp. BV3C16-1 | 0 | Not detected | 0.074 | -0.369 | 0.518 | 0.743 |
| Megasphaera sp. UPII 135-E | 0 | Not detected | -0.009 | -0.181 | 0.162 | 0.915 |
| Merdibacter massiliensis | 0 | Not detected | -0.078 | -0.259 | 0.103 | 0.400 |
| Methanobrevibacter smithii | 0 | Not detected | -0.487 | -1.320 | 0.347 | 0.254 |
| Methanobrevibacter smithii CAG:186 | 0 | Not detected | -0.455 | -1.215 | 0.304 | 0.241 |
| Methanocorpusculaceae archaeon Phil4 | 0 | Not detected | -0.281 | -0.720 | 0.158 | 0.210 |
| Methanosarcina mazei | 0 | Not detected | -0.031 | -0.447 | 0.385 | 0.885 |
| Methylobacterium sp. ZNC0032 | 0 | Not detected | -0.011 | -0.355 | 0.333 | 0.951 |
| Mitsuokella multacida | 0 | Not detected | 0.317 | -0.367 | 1.001 | 0.365 |
| Mobilibacterium timonense | 0 | Not detected | 0.004 | -0.345 | 0.353 | 0.984 |
| Mobiluncus curtisii | 0 | Not detected | 0.006 | -0.196 | 0.207 | 0.956 |
| Mobiluncus mulieris | 0 | Not detected | 0.004 | -0.244 | 0.252 | 0.977 |
| Mogibacterium timidum | 0 | Not detected | 0.082 | -0.358 | 0.522 | 0.717 |
| Morganella morganii | 0 | Not detected | 0.045 | -0.304 | 0.393 | 0.802 |
| Mucinivorans hirudinis | 0 | Not detected | -0.069 | -0.375 | 0.238 | 0.661 |
| Murdochiella sp. Marseille-P2341 | 0 | Not detected | -0.148 | -0.375 | 0.079 | 0.201 |
| Mycoplasma sp. CAG:877 | 0 | Not detected | 0.234 | -0.262 | 0.730 | 0.357 |
| Mycoplasma sp. CAG:956 | 0 | Not detected | 0.268 | -0.137 | 0.672 | 0.196 |
| Myroides injenensis | 0 | Not detected | 0.384 | -0.097 | 0.866 | 0.119 |
| Myroides odoratimimus | 0 | Not detected | 0.620 | 0.067 | 1.172 | 0.029 |
| Necropsobacter massiliensis | 0 | Not detected | 0.181 | -0.192 | 0.555 | 0.342 |

| Negativicoccus massiliensis | 0 | Not detected | -0.089 | -0.448 | 0.271 | 0.629 |
| --- | --- | --- | --- | --- | --- | --- |
| Neglecta timonensis | 0 | Not detected | -0.011 | -0.314 | 0.293 | 0.944 |
| Neisseria cinerea | 0 | Not detected | 0.098 | -0.281 | 0.478 | 0.611 |
| Novibacillus thermophilus | 0 | Not detected | 0.296 | -0.085 | 0.678 | 0.129 |
| Obesumbacterium proteus | 0 | Not detected | 0.109 | -0.394 | 0.611 | 0.672 |
| Odoribacter laneus | 0 | Not detected | -0.012 | -0.434 | 0.411 | 0.957 |
| Odoribacter laneus CAG:561 | 0 | Not detected | 0.195 | -0.328 | 0.719 | 0.465 |
| Odoribacter sp. 43_10 | 0 | Not detected | 0.070 | -0.308 | 0.449 | 0.716 |
| Odoribacter sp. CAG:788 | 0 | Not detected | -0.298 | -0.766 | 0.170 | 0.214 |
| Odoribacter splanchnicus | 0 | Not detected | -0.048 | -0.313 | 0.217 | 0.725 |
| Odoribacter splanchnicus CAG:14 | 0 | Not detected | 0.203 | -0.224 | 0.630 | 0.352 |
| Oligella ureolytica | 0 | Not detected | -0.099 | -0.475 | 0.276 | 0.604 |
| Olsenella sp. Marseille-P3197 | 0 | Not detected | -0.048 | -0.473 | 0.376 | 0.823 |
| Olsenella sp. oral taxon 809 | 0 | Not detected | -0.247 | -0.539 | 0.045 | 0.099 |
| Olsenella uli | 0 | Not detected | -0.214 | -0.631 | 0.202 | 0.314 |
| Olsenella umbonata | 1 | Not detected | -0.329 | -0.654 | -0.004 | 0.048 |
| Oribacterium parvum | 0 | Not detected | -0.025 | -0.172 | 0.122 | 0.737 |
| Oribacterium sinus | 0 | Not detected | 0.421 | 0.059 | 0.782 | 0.023 |
| Oribacterium sp. WCC10 | 0 | Not detected | -0.054 | -0.465 | 0.357 | 0.796 |
| Oribacterium sp. oral taxon 078 | 0 | Not detected | -0.003 | -0.263 | 0.256 | 0.980 |
| Oscillibacter ruminantium | 0 | Not detected | -0.172 | -0.512 | 0.169 | 0.324 |
| Oscillibacter sp. 1-3 | 0 | Not detected | -0.011 | -0.211 | 0.189 | 0.912 |
| Oscillibacter sp. 57_20 | 0 | Not detected | 0.225 | -0.428 | 0.879 | 0.500 |
| Oscillibacter sp. ER4 | 0 | Not detected | 0.279 | -0.111 | 0.669 | 0.162 |
| Oscillibacter sp. KLE 1745 | 0 | Not detected | -0.362 | -0.651 | -0.072 | 0.015 |
| Oscillibacter valericigenes | 0 | Not detected | -0.073 | -0.354 | 0.209 | 0.614 |
| Oscillospiraceae bacterium Zagget12 | 0 | Not detected | -0.105 | -0.480 | 0.271 | 0.585 |
| Oxalobacter formigenes | 0 | Not detected | 0.089 | -0.263 | 0.442 | 0.619 |
| Paeniclostridium sordellii | 0 | Not detected | 0.110 | -0.322 | 0.542 | 0.618 |
| Paludibacter propionicigenes | 0 | Not detected | 0.106 | -0.197 | 0.409 | 0.494 |
| Paludibacter sp. 47-17 | 0 | Not detected | -0.136 | -0.478 | 0.206 | 0.435 |
| Pantoea sp. PSNIH2 | 0 | Not detected | -0.116 | -0.669 | 0.437 | 0.682 |
| Parabacteroides distasonis | 0 | Not detected | -0.266 | -0.616 | 0.084 | 0.138 |
| Parabacteroides goldsteinii | 0 | Not detected | -0.164 | -0.526 | 0.199 | 0.377 |
| Parabacteroides gordonii | 0 | Not detected | -0.132 | -0.438 | 0.173 | 0.397 |
| Parabacteroides johnsonii | 0 | Not detected | -0.141 | -0.428 | 0.145 | 0.334 |
| Parabacteroides merdae CAG:48 | 0 | Not detected | -0.222 | -0.761 | 0.317 | 0.421 |
| Paraburkholderia kururiensis | 0 | Not detected | 0.101 | -0.129 | 0.331 | 0.390 |
| Paraburkholderia sacchari | 0 | Not detected | 0.031 | -0.347 | 0.410 | 0.871 |
| Paraclostridium bifermentans | 0 | Not detected | -0.023 | -0.409 | 0.363 | 0.908 |
| Paraprevotella clara CAG:116 | 0 | Not detected | 0.579 | -0.016 | 1.174 | 0.058 |
| Paraprevotella xylaniphila | 0 | Not detected | 0.371 | 0.054 | 0.688 | 0.023 |
| Parascardovia denticolens | 0 | Not detected | -0.134 | -0.546 | 0.278 | 0.525 |
| Parasporobacterium paucivorans | 0 | Not detected | -0.310 | -0.599 | -0.021 | 0.036 |

| Parasutterella excrementihominis | 0 | Not detected | 0.097 | -0.552 | 0.745 | 0.770 |
| --- | --- | --- | --- | --- | --- | --- |
| Parasutterella excrementihominis CAG:23 | 0 | Not detected | 0.189 | -0.715 | 1.092 | 0.683 |
| Parvimonas micra | 0 | Not detected | 0.294 | -0.142 | 0.730 | 0.187 |
| Pelosinus fermentans | 0 | Not detected | 0.118 | -0.203 | 0.439 | 0.472 |
| Peptoanaerobacter stomatis | 0 | Not detected | -0.017 | -0.341 | 0.306 | 0.916 |
| Peptococcus niger | 0 | Not detected | -0.040 | -0.384 | 0.303 | 0.818 |
| Peptoniphilus duerdenii | 0 | Not detected | -0.129 | -0.438 | 0.179 | 0.411 |
| Peptoniphilus grossensis | 0 | Not detected | -0.389 | -0.803 | 0.024 | 0.066 |
| Peptoniphilus harei | 0 | Not detected | -0.170 | -0.604 | 0.264 | 0.442 |
| Peptoniphilus lacrimalis | 0 | Not detected | -0.433 | -0.903 | 0.037 | 0.072 |
| Peptoniphilus obesi | 0 | Not detected | -0.069 | -0.494 | 0.355 | 0.748 |
| Peptoniphilus rhinitidis | 0 | Not detected | -0.298 | -0.690 | 0.094 | 0.137 |
| Peptoniphilus senegalensis | 0 | Not detected | -0.236 | -0.669 | 0.197 | 0.286 |
| Peptoniphilus sp. BV3C26 | 0 | Not detected | -0.300 | -0.751 | 0.151 | 0.194 |
| Peptoniphilus sp. ING2-D1G | 0 | Not detected | -0.064 | -0.474 | 0.346 | 0.759 |
| Peptoniphilus sp. oral taxon 375 | 0 | Not detected | 0.058 | -0.168 | 0.283 | 0.616 |
| Peptostreptococcaceae bacterium VA2 | 0 | Not detected | 0.199 | -0.292 | 0.691 | 0.427 |
| Peptostreptococcus anaerobius | 0 | Not detected | -0.166 | -0.357 | 0.026 | 0.091 |
| Peptostreptococcus anaerobius CAG:621 | 0 | Not detected | -0.162 | -0.546 | 0.222 | 0.409 |
| Peptostreptococcus stomatis | 0 | Not detected | 0.231 | -0.161 | 0.624 | 0.249 |
| Petrimonas mucosa | 0 | Not detected | 0.275 | -0.125 | 0.675 | 0.180 |
| Phaeodactylibacter xiamenensis | 0 | Not detected | 0.078 | -0.463 | 0.618 | 0.779 |
| Phascolarctobacterium sp. CAG:207 | 0 | Not detected | 0.300 | -0.591 | 1.191 | 0.510 |
| Phascolarctobacterium sp. CAG:266 | 0 | Not detected | 0.145 | -0.352 | 0.642 | 0.568 |
| Phascolarctobacterium succinatutens | 0 | Not detected | 0.161 | -0.294 | 0.617 | 0.488 |
| Phocaeicola abscessus | 0 | Not detected | 0.051 | -0.247 | 0.348 | 0.739 |
| Phocea massiliensis | 0 | Not detected | 0.038 | -0.182 | 0.258 | 0.735 |
| Pluralibacter gergoviae | 0 | Not detected | -0.031 | -0.501 | 0.438 | 0.897 |
| Pontibacillus litoralis | 0 | Not detected | -0.573 | -1.137 | -0.009 | 0.048 |
| Porphyromonadaceae bacterium COT-184 | 0 | Not detected | 0.095 | -0.270 | 0.461 | 0.610 |
| Porphyromonadaceae bacterium KH3CP3R | 0 | Not detected | -0.042 | -0.347 | 0.264 | 0.790 |
| Porphyromonadaceae bacterium KH3R12 | 0 | Not detected | -0.083 | -0.421 | 0.255 | 0.631 |
| Porphyromonas asaccharolytica | 0 | Not detected | -0.195 | -0.676 | 0.287 | 0.429 |
| Porphyromonas bennonis | 1 | Not detected | -0.347 | -0.792 | 0.097 | 0.127 |
| Porphyromonas cangingivalis | 2 | Not detected | -0.370 | -0.717 | -0.023 | 0.037 |
| Porphyromonas canoris | 0 | Not detected | -0.107 | -0.483 | 0.270 | 0.578 |
| Porphyromonas catoniae | 0 | Not detected | -0.012 | -0.296 | 0.272 | 0.934 |
| Porphyromonas crevioricanis | 0 | Not detected | -0.048 | -0.367 | 0.270 | 0.766 |
| Porphyromonas endodontalis | 0 | Not detected | -0.236 | -0.620 | 0.149 | 0.231 |
| Porphyromonas gingivalis | 0 | Not detected | -0.363 | -0.679 | -0.046 | 0.025 |
| Porphyromonas gingivicanis | 0 | Not detected | -0.117 | -0.398 | 0.165 | 0.418 |
| Porphyromonas levii | 0 | Not detected | -0.022 | -0.574 | 0.530 | 0.937 |
| Porphyromonas macacae | 0 | Not detected | -0.276 | -0.627 | 0.074 | 0.124 |
| Porphyromonas somerae | 0 | Not detected | -0.045 | -0.260 | 0.170 | 0.684 |

| Porphyromonas sp. CAG:1061 | 0 | Not detected | -0.033 | -0.506 | 0.440 | 0.890 |
| --- | --- | --- | --- | --- | --- | --- |
| Porphyromonas sp. COT-239 OH1446 | 0 | Not detected | -0.118 | -0.666 | 0.430 | 0.673 |
| Porphyromonas sp. COT-290 OH3588 | 0 | Not detected | -0.234 | -0.592 | 0.124 | 0.201 |
| Prevotella albensis | 0 | Not detected | 0.265 | -0.429 | 0.959 | 0.455 |
| Prevotella amnii | 0 | Not detected | 0.013 | -0.207 | 0.233 | 0.906 |
| Prevotella aurantiaca | 0 | Not detected | 0.140 | -0.310 | 0.589 | 0.543 |
| Prevotella baroniae | 0 | Not detected | -0.064 | -0.316 | 0.188 | 0.618 |
| Prevotella bergensis | 0 | Not detected | 0.198 | -0.169 | 0.565 | 0.292 |
| Prevotella bivia | 0 | Not detected | 0.044 | -0.193 | 0.282 | 0.714 |
| Prevotella bryantii | 0 | Not detected | 0.085 | -0.607 | 0.777 | 0.810 |
| Prevotella buccae | 0 | Not detected | 0.119 | -0.396 | 0.633 | 0.652 |
| Prevotella buccalis | 0 | Not detected | -0.031 | -0.267 | 0.205 | 0.799 |
| Prevotella copri | 0 | Not detected | 0.338 | -0.433 | 1.109 | 0.391 |
| Prevotella corporis | 0 | Not detected | -0.076 | -0.324 | 0.172 | 0.549 |
| Prevotella dentalis | 0 | Not detected | 0.097 | -0.491 | 0.684 | 0.747 |
| Prevotella dentasini | 0 | Not detected | -0.019 | -0.517 | 0.480 | 0.942 |
| Prevotella denticola | 0 | Not detected | 0.296 | -0.291 | 0.883 | 0.324 |
| Prevotella disiens | 0 | Not detected | -0.038 | -0.274 | 0.199 | 0.755 |
| Prevotella enoeca | 0 | Not detected | 0.062 | -0.473 | 0.597 | 0.821 |
| Prevotella fusca | 0 | Not detected | 0.099 | -0.551 | 0.750 | 0.765 |
| Prevotella histicola | 0 | Not detected | 0.188 | -0.244 | 0.620 | 0.394 |
| Prevotella intermedia | 0 | Not detected | -0.049 | -0.434 | 0.335 | 0.802 |
| Prevotella loescheii | 0 | Not detected | 0.163 | -0.283 | 0.609 | 0.474 |
| Prevotella maculosa | 0 | Not detected | 0.222 | -0.244 | 0.689 | 0.351 |
| Prevotella marshii | 0 | Not detected | -0.071 | -0.345 | 0.204 | 0.614 |
| Prevotella melaninogenica | 0 | Not detected | 0.301 | -0.134 | 0.736 | 0.176 |
| Prevotella micans | 0 | Not detected | 0.141 | -0.366 | 0.648 | 0.586 |
| Prevotella multiformis | 0 | Not detected | 0.258 | -0.098 | 0.614 | 0.156 |
| Prevotella multisaccharivorax | 0 | Not detected | 0.565 | -0.088 | 1.218 | 0.091 |
| Prevotella nanceiensis | 0 | Not detected | 0.211 | -0.283 | 0.705 | 0.402 |
| Prevotella nigrescens | 0 | Not detected | -0.046 | -0.509 | 0.417 | 0.847 |
| Prevotella oralis | 0 | Not detected | 0.152 | -0.345 | 0.649 | 0.549 |
| Prevotella oris | 0 | Not detected | 0.366 | -0.219 | 0.950 | 0.221 |
| Prevotella oryzae | 0 | Not detected | 0.174 | -0.424 | 0.773 | 0.569 |
| Prevotella oulorum | 0 | Not detected | 0.167 | -0.448 | 0.783 | 0.594 |
| Prevotella pallens | 0 | Not detected | 0.104 | -0.188 | 0.395 | 0.487 |
| Prevotella paludivivens | 0 | Not detected | 0.152 | -0.190 | 0.494 | 0.385 |
| Prevotella ruminicola | 0 | Not detected | 0.036 | -0.543 | 0.614 | 0.904 |
| Prevotella saccharolytica | 0 | Not detected | -0.021 | -0.563 | 0.522 | 0.941 |
| Prevotella salivae | 0 | Not detected | 0.103 | -0.150 | 0.355 | 0.426 |
| Prevotella scopos | 0 | Not detected | 0.172 | -0.170 | 0.513 | 0.325 |
| Prevotella sp. 10(H) | 0 | Not detected | -0.148 | -0.461 | 0.165 | 0.355 |
| Prevotella sp. 109 | 0 | Not detected | -0.158 | -0.399 | 0.082 | 0.198 |
| Prevotella sp. AGR2160 | 0 | Not detected | 0.013 | -0.675 | 0.701 | 0.970 |

| Prevotella sp. CAG:1058 | 0 | Not detected | 0.304 | -0.212 | 0.819 | 0.249 |
| --- | --- | --- | --- | --- | --- | --- |
| Prevotella sp. CAG:1092 | 0 | Not detected | 0.693 | 0.039 | 1.347 | 0.039 |
| Prevotella sp. CAG:1124 | 0 | Not detected | 0.159 | -0.379 | 0.697 | 0.563 |
| Prevotella sp. CAG:1185 | 0 | Not detected | -0.117 | -0.717 | 0.483 | 0.703 |
| Prevotella sp. CAG:1320 | 0 | Not detected | 0.444 | -0.067 | 0.955 | 0.090 |
| Prevotella sp. CAG:255 | 0 | Not detected | -0.183 | -0.591 | 0.226 | 0.382 |
| Prevotella sp. CAG:386 | 0 | Not detected | 0.285 | -0.463 | 1.033 | 0.456 |
| Prevotella sp. CAG:487 | 0 | Not detected | 0.295 | -0.075 | 0.665 | 0.119 |
| Prevotella sp. CAG:520 | 0 | Not detected | -0.053 | -0.718 | 0.612 | 0.875 |
| Prevotella sp. CAG:5226 | 0 | Not detected | 0.437 | -0.222 | 1.095 | 0.195 |
| Prevotella sp. CAG:604 | 0 | Not detected | 0.086 | -0.748 | 0.919 | 0.840 |
| Prevotella sp. CAG:732 | 0 | Not detected | 0.288 | -0.477 | 1.054 | 0.461 |
| Prevotella sp. CAG:891 | 0 | Not detected | 0.420 | -0.174 | 1.015 | 0.167 |
| Prevotella sp. FD3004 | 0 | Not detected | 0.102 | -0.307 | 0.511 | 0.626 |
| Prevotella sp. HUN102 | 0 | Not detected | 0.162 | -0.150 | 0.474 | 0.309 |
| Prevotella sp. MSX73 | 0 | Not detected | 0.228 | -0.254 | 0.711 | 0.355 |
| Prevotella sp. P4-76 | 0 | Not detected | 0.172 | -0.226 | 0.570 | 0.397 |
| Prevotella sp. RM4 | 0 | Not detected | 0.106 | -0.232 | 0.445 | 0.539 |
| Prevotella sp. S7 MS 2 | 0 | Not detected | 0.049 | -0.212 | 0.309 | 0.714 |
| Prevotella sp. S7-1-8 | 0 | Not detected | 0.054 | -0.180 | 0.287 | 0.653 |
| Prevotella sp. kh1p2 | 0 | Not detected | 0.030 | -0.483 | 0.544 | 0.908 |
| Prevotella sp. khp1 | 0 | Not detected | 0.124 | -0.210 | 0.457 | 0.468 |
| Prevotella sp. ne3005 | 0 | Not detected | -0.014 | -0.300 | 0.272 | 0.922 |
| Prevotella sp. oral taxon 473 | 0 | Not detected | -0.187 | -0.710 | 0.336 | 0.485 |
| Prevotella stercorea | 0 | Not detected | 0.127 | -0.177 | 0.430 | 0.414 |
| Prevotella stercorea CAG:629 | 0 | Not detected | 0.099 | -0.584 | 0.783 | 0.776 |
| Prevotella timonensis | 0 | Not detected | -0.018 | -0.254 | 0.217 | 0.879 |
| Prevotellamassilia timonensis | 0 | Not detected | -0.012 | -0.364 | 0.339 | 0.946 |
| Propionibacterium acidifaciens | 0 | Not detected | -0.229 | -0.629 | 0.171 | 0.263 |
| Proteiniclasticum ruminis | 0 | Not detected | 0.032 | -0.257 | 0.321 | 0.828 |
| Proteiniphilum acetatigenes | 0 | Not detected | 0.045 | -0.264 | 0.355 | 0.774 |
| Proteiniphilum saccharofermentans | 0 | Not detected | -0.197 | -0.548 | 0.154 | 0.272 |
| Proteiniphilum sp. EBM-41 | 0 | Not detected | 0.481 | -0.032 | 0.995 | 0.067 |
| Proteobacteria bacterium CAG:139 | 0 | Not detected | 0.326 | -0.515 | 1.167 | 0.448 |
| Proteobacteria bacterium CAG:495 | 0 | Not detected | -0.173 | -0.849 | 0.503 | 0.617 |
| Providencia rettgeri | 0 | Not detected | 0.047 | -0.322 | 0.415 | 0.805 |
| Pseudobacteroides cellulosolvens | 0 | Not detected | 0.077 | -0.216 | 0.369 | 0.607 |
| Pseudobutyrivibrio ruminis | 0 | Not detected | -0.053 | -0.243 | 0.137 | 0.587 |
| Pseudobutyrivibrio sp. ACV-2 | 0 | Not detected | 0.275 | -0.062 | 0.612 | 0.110 |
| Pseudobutyrivibrio sp. JW11 | 0 | Not detected | 0.014 | -0.330 | 0.357 | 0.938 |
| Pseudobutyrivibrio sp. MD2005 | 0 | Not detected | 0.143 | -0.225 | 0.511 | 0.447 |
| Pseudobutyrivibrio sp. UC1225 | 0 | Not detected | 0.020 | -0.253 | 0.293 | 0.887 |
| Pseudobutyrivibrio sp. YE44 | 0 | Not detected | -0.085 | -0.429 | 0.259 | 0.629 |
| Pseudobutyrivibrio xylanivorans | 0 | Not detected | 0.206 | -0.147 | 0.560 | 0.254 |

| Pseudoflavonifractor capillosus | 0 | Not detected | -0.107 | -0.310 | 0.096 | 0.304 |
| --- | --- | --- | --- | --- | --- | --- |
| Pseudopropionibacterium propionicum | 0 | Not detected | 0.134 | -0.265 | 0.532 | 0.512 |
| Pyramidobacter piscolens | 0 | Not detected | -0.215 | -0.776 | 0.345 | 0.452 |
| Ralstonia mannitolilytica | 0 | Not detected | -0.259 | -0.731 | 0.212 | 0.282 |
| Ralstonia pickettii | 0 | Not detected | -0.053 | -0.488 | 0.381 | 0.810 |
| Raoultella ornithinolytica | 0 | Not detected | -0.015 | -0.632 | 0.602 | 0.961 |
| Rhodococcus defluvii | 0 | Not detected | 0.064 | -0.284 | 0.412 | 0.720 |
| Rhodospirillum sp. UNK.MGS-17 | 0 | Not detected | -0.444 | -1.165 | 0.277 | 0.229 |
| Riemerella columbina | 0 | Not detected | -0.083 | -0.315 | 0.150 | 0.487 |
| Rikenella microfusus | 0 | Not detected | -0.093 | -0.325 | 0.140 | 0.436 |
| Risungbinella massiliensis | 0 | Not detected | -0.107 | -0.394 | 0.180 | 0.466 |
| Robinsoniella peoriensis | 0 | Not detected | -0.037 | -0.179 | 0.105 | 0.611 |
| Roseburia faecis | 0 | Not detected | -0.048 | -0.419 | 0.323 | 0.800 |
| Roseburia hominis | 0 | Not detected | -0.065 | -0.287 | 0.156 | 0.563 |
| Roseburia intestinalis | 0 | Not detected | 0.053 | -0.285 | 0.390 | 0.760 |
| Roseburia inulinivorans | 0 | Not detected | -0.011 | -0.283 | 0.261 | 0.936 |
| Roseburia sp. CAG:197_41_10 | 0 | Not detected | -0.113 | -0.377 | 0.150 | 0.401 |
| Roseburia sp. CAG:309 | 0 | Not detected | -0.075 | -0.670 | 0.520 | 0.805 |
| Roseburia sp. CAG:380 | 0 | Not detected | 0.211 | -0.255 | 0.676 | 0.376 |
| Rothia dentocariosa | 0 | Not detected | -0.249 | -0.753 | 0.255 | 0.333 |
| Rothia mucilaginosa | 0 | Not detected | 0.247 | -0.332 | 0.827 | 0.404 |
| Ruminococcaceae bacterium AE2021 | 0 | Not detected | -0.226 | -0.491 | 0.038 | 0.095 |
| Ruminococcaceae bacterium D16 | 0 | Not detected | -0.201 | -0.388 | -0.014 | 0.036 |
| Ruminococcaceae bacterium YRB3002 | 0 | Not detected | -0.058 | -0.395 | 0.280 | 0.738 |
| Ruminococcus albus | 0 | Not detected | 0.237 | -0.141 | 0.616 | 0.220 |
| Ruminococcus bicirculans | 0 | Not detected | 0.298 | -0.283 | 0.879 | 0.316 |
| Ruminococcus callidus | 0 | Not detected | 0.190 | -0.195 | 0.574 | 0.335 |
| Ruminococcus champanellensis | 0 | Not detected | 0.026 | -0.456 | 0.508 | 0.915 |
| Ruminococcus faecis | 0 | Not detected | -0.005 | -0.267 | 0.256 | 0.967 |
| Ruminococcus flavefaciens | 0 | Not detected | 0.138 | -0.223 | 0.499 | 0.454 |
| Ruminococcus gauvreauii | 0 | Not detected | 0.129 | -0.062 | 0.320 | 0.187 |
| Ruminococcus lactaris | 0 | Not detected | -0.159 | -0.455 | 0.137 | 0.292 |
| Ruminococcus obeum CAG:39 | 0 | Not detected | -0.018 | -0.365 | 0.329 | 0.920 |
| Ruminococcus sp. AT10 | 0 | Not detected | -0.084 | -0.255 | 0.087 | 0.336 |
| Ruminococcus sp. CAG:108-related_41_3 | 0 | Not detected | 0.299 | -0.309 | 0.907 | 0.336 |
| Ruminococcus sp. CAG:177 | 0 | Not detected | -0.060 | -0.969 | 0.848 | 0.897 |
| Ruminococcus sp. CAG:254 | 0 | Not detected | -0.452 | -1.214 | 0.310 | 0.246 |
| Ruminococcus sp. CAG:330 | 0 | Not detected | -0.042 | -0.861 | 0.776 | 0.920 |
| Ruminococcus sp. CAG:379 | 0 | Not detected | 0.225 | -0.293 | 0.743 | 0.395 |
| Ruminococcus sp. CAG:382 | 0 | Not detected | 0.024 | -0.529 | 0.577 | 0.933 |
| Ruminococcus sp. CAG:403 | 0 | Not detected | -0.024 | -0.654 | 0.606 | 0.940 |
| Ruminococcus sp. CAG:488 | 0 | Not detected | 0.044 | -0.432 | 0.519 | 0.856 |
| Ruminococcus sp. CAG:563 | 0 | Not detected | 0.107 | -0.377 | 0.591 | 0.664 |
| Ruminococcus sp. CAG:60 | 0 | Not detected | 0.169 | -0.155 | 0.493 | 0.307 |

| Ruminococcus sp. CAG:624 | 0 | Not detected | -0.183 | -0.658 | 0.291 | 0.450 |
| --- | --- | --- | --- | --- | --- | --- |
| Ruminococcus sp. CAG:724 | 0 | Not detected | -0.084 | -0.563 | 0.395 | 0.731 |
| Ruminococcus sp. FC2018 | 0 | Not detected | 0.230 | -0.146 | 0.606 | 0.231 |
| Ruminococcus sp. HUN007 | 0 | Not detected | -0.105 | -0.438 | 0.227 | 0.535 |
| Ruminococcus sp. NK3A76 | 0 | Not detected | 0.134 | -0.272 | 0.539 | 0.520 |
| Ruminococcus sp. Phil15 | 0 | Not detected | -0.187 | -0.689 | 0.315 | 0.466 |
| Ruminococcus sp. Zagget7 | 0 | Not detected | -0.102 | -0.388 | 0.183 | 0.483 |
| Ruminococcus torques CAG:61 | 0 | Not detected | -0.544 | -1.055 | -0.033 | 0.038 |
| Ruthenibacterium lactatiformans | 0 | Not detected | -0.047 | -0.335 | 0.240 | 0.747 |
| Salmonella enterica | 0 | Not detected | -0.171 | -0.755 | 0.413 | 0.567 |
| Sanguibacteroides justesenii | 0 | Not detected | -0.104 | -0.506 | 0.299 | 0.615 |
| Scardovia inopinata | 0 | Not detected | -0.128 | -0.474 | 0.219 | 0.471 |
| Scardovia wiggsiae | 0 | Not detected | 0.054 | -0.395 | 0.503 | 0.813 |
| Selenomonas bovis | 0 | Not detected | -0.039 | -0.355 | 0.276 | 0.809 |
| Selenomonas ruminantium | 0 | Not detected | 0.001 | -0.402 | 0.405 | 0.995 |
| Selenomonas sputigena | 0 | Not detected | 0.066 | -0.292 | 0.424 | 0.717 |
| Sellimonas intestinalis | 0 | Not detected | -0.155 | -0.467 | 0.157 | 0.331 |
| Senegalimassilia anaerobia | 0 | Not detected | -0.101 | -0.593 | 0.392 | 0.689 |
| Sharpea azabuensis | 0 | Not detected | 0.026 | -0.205 | 0.257 | 0.826 |
| Shigella dysenteriae | 0 | Not detected | -0.470 | -1.132 | 0.192 | 0.165 |
| Shigella flexneri | 0 | Not detected | -0.453 | -1.106 | 0.200 | 0.175 |
| Shimwellia blattae | 0 | Not detected | -0.077 | -0.493 | 0.339 | 0.717 |
| Shuttleworthia satelles | 0 | Not detected | -0.054 | -0.328 | 0.220 | 0.700 |
| Siccibacter turicensis | 0 | Not detected | -0.017 | -0.458 | 0.424 | 0.940 |
| Slackia exigua | 0 | Not detected | -0.318 | -0.702 | 0.066 | 0.106 |
| Slackia heliotrinireducens | 0 | Not detected | -0.235 | -0.547 | 0.077 | 0.140 |
| Slackia piriformis | 0 | Not detected | 0.123 | -0.516 | 0.763 | 0.705 |
| Solobacterium moorei | 0 | Not detected | -0.045 | -0.304 | 0.215 | 0.736 |
| Sphaerochaeta sp. Phil3 | 0 | Not detected | 0.127 | -0.356 | 0.611 | 0.607 |
| Sphingobacterium wenxiniae | 0 | Not detected | 0.019 | -0.447 | 0.486 | 0.935 |
| Spirochaetales bacterium Spiro_07 | 0 | Not detected | -0.148 | -0.460 | 0.165 | 0.355 |
| Sporobacter termitidis | 0 | Not detected | -0.007 | -0.382 | 0.369 | 0.972 |
| Sporomusa ovata | 0 | Not detected | 0.045 | -0.337 | 0.427 | 0.818 |
| Staphylococcus pseudintermedius | 0 | Not detected | -0.060 | -0.424 | 0.304 | 0.747 |
| Staphylococcus sp. CAG:324 | 0 | Not detected | -0.420 | -1.132 | 0.292 | 0.249 |
| Staphylococcus sp. DORA_6_22 | 0 | Not detected | 0.055 | -0.314 | 0.424 | 0.770 |
| Staphylococcus warneri | 0 | Not detected | 0.023 | -0.353 | 0.400 | 0.904 |
| Stomatobaculum longum | 0 | Not detected | -0.041 | -0.183 | 0.100 | 0.570 |
| Streptobacillus notomytis | 0 | Not detected | -0.116 | -0.461 | 0.228 | 0.509 |
| Streptococcus agalactiae | 0 | Not detected | -0.335 | -0.796 | 0.126 | 0.155 |
| Streptococcus anginosus | 0 | Not detected | 0.039 | -0.425 | 0.503 | 0.868 |
| Streptococcus caballi | 0 | Not detected | -0.168 | -0.569 | 0.233 | 0.412 |
| Streptococcus cristatus | 0 | Not detected | -0.031 | -0.402 | 0.341 | 0.872 |
| Streptococcus dysgalactiae | 0 | Not detected | -0.213 | -0.616 | 0.191 | 0.303 |

| Streptococcus entericus | 0 | Not detected | -0.254 | -0.616 | 0.108 | 0.171 |
| --- | --- | --- | --- | --- | --- | --- |
| Streptococcus gallolyticus | 0 | Not detected | 0.033 | -0.507 | 0.573 | 0.904 |
| Streptococcus gordonii | 0 | Not detected | 0.217 | -0.301 | 0.734 | 0.413 |
| Streptococcus halotolerans | 0 | Not detected | -0.036 | -0.342 | 0.270 | 0.819 |
| Streptococcus henryi | 0 | Not detected | -0.204 | -0.567 | 0.160 | 0.273 |
| Streptococcus ictaluri | 0 | Not detected | -0.199 | -0.528 | 0.130 | 0.237 |
| Streptococcus infantis | 0 | Not detected | 0.097 | -0.303 | 0.497 | 0.636 |
| Streptococcus iniae | 0 | Not detected | -0.248 | -0.595 | 0.100 | 0.164 |
| Streptococcus intermedius | 0 | Not detected | -0.009 | -0.469 | 0.450 | 0.969 |
| Streptococcus massiliensis | 0 | Not detected | -0.331 | -0.681 | 0.019 | 0.065 |
| Streptococcus mitis | 0 | Not detected | 0.153 | -0.159 | 0.465 | 0.339 |
| Streptococcus mutans | 0 | Not detected | -0.166 | -0.732 | 0.399 | 0.565 |
| Streptococcus oralis | 0 | Not detected | 0.184 | -0.233 | 0.601 | 0.388 |
| Streptococcus orisratti | 0 | Not detected | -0.010 | -0.417 | 0.397 | 0.962 |
| Streptococcus ovis | 0 | Not detected | -0.042 | -0.395 | 0.311 | 0.816 |
| Streptococcus parasanguinis | 0 | Not detected | 0.133 | -0.350 | 0.616 | 0.590 |
| Streptococcus parauberis | 0 | Not detected | -0.170 | -0.511 | 0.171 | 0.329 |
| Streptococcus plurextorum | 0 | Not detected | 0.082 | -0.172 | 0.336 | 0.529 |
| Streptococcus porci | 0 | Not detected | 0.060 | -0.218 | 0.337 | 0.672 |
| Streptococcus pseudoporcinus | 0 | Not detected | -0.092 | -0.499 | 0.315 | 0.657 |
| Streptococcus pyogenes | 0 | Not detected | -0.039 | -0.445 | 0.367 | 0.850 |
| Streptococcus salivarius | 0 | Not detected | 0.120 | -0.318 | 0.558 | 0.593 |
| Streptococcus sanguinis | 0 | Not detected | 0.243 | -0.270 | 0.756 | 0.354 |
| Streptococcus sobrinus | 0 | Not detected | -0.054 | -0.440 | 0.332 | 0.784 |
| Streptococcus suis | 0 | Not detected | -0.142 | -0.408 | 0.124 | 0.297 |
| Streptococcus thermophilus | 0 | Not detected | 0.092 | -0.394 | 0.578 | 0.712 |
| Streptococcus uberis | 0 | Not detected | -0.201 | -0.539 | 0.136 | 0.243 |
| Streptococcus urinalis | 0 | Not detected | -0.121 | -0.515 | 0.273 | 0.548 |
| Streptomyces albulus | 0 | Not detected | 0.009 | -0.321 | 0.340 | 0.955 |
| Streptomyces cattleya | 0 | Not detected | -0.196 | -0.629 | 0.237 | 0.375 |
| Subdoligranulum sp. CAG:314 | 0 | Not detected | 0.072 | -0.794 | 0.939 | 0.870 |
| Subdoligranulum variabile | 0 | Not detected | -0.046 | -0.238 | 0.146 | 0.640 |
| Succinatimonas sp. CAG:777 | 0 | Not detected | 0.129 | -0.540 | 0.797 | 0.706 |
| Succinispira mobilis | 0 | Not detected | 0.005 | -0.403 | 0.413 | 0.979 |
| Succinivibrio sp. Phil9 | 0 | Not detected | -0.056 | -0.699 | 0.586 | 0.864 |
| Sulfolobus acidocaldarius | 0 | Not detected | -0.087 | -0.605 | 0.430 | 0.741 |
| Sutterella parvirubra | 0 | Not detected | 0.185 | -0.283 | 0.652 | 0.439 |
| Sutterella sp. CAG:397 | 0 | Not detected | 0.103 | -0.701 | 0.907 | 0.802 |
| Sutterella sp. CAG:521 | 0 | Not detected | -0.140 | -0.522 | 0.242 | 0.474 |
| Sutterella wadsworthensis | 0 | Not detected | 0.620 | -0.215 | 1.455 | 0.147 |
| Sutterella wadsworthensis CAG:135 | 0 | Not detected | 0.644 | -0.362 | 1.650 | 0.211 |
| Sutterellaceae bacterium ND3 | 0 | Not detected | -0.326 | -0.904 | 0.251 | 0.269 |
| Synergistales bacterium Syner_02 | 0 | Not detected | -0.201 | -0.623 | 0.222 | 0.353 |
| Synergistes sp. Zagget9 | 0 | Not detected | -0.010 | -0.375 | 0.356 | 0.959 |

| Tannerella forsythia | 0 | Not detected | 0.037 | -0.251 | 0.325 | 0.803 |
| --- | --- | --- | --- | --- | --- | --- |
| Tepidanaerobacter sp. EBM-49 | 0 | Not detected | -0.300 | -0.885 | 0.285 | 0.316 |
| Terrisporobacter glycolicus | 0 | Not detected | -0.009 | -0.466 | 0.448 | 0.969 |
| Thermophagus xiamenensis | 0 | Not detected | 0.183 | -0.244 | 0.609 | 0.402 |
| Thermosinus carboxydivorans | 0 | Not detected | 0.177 | -0.131 | 0.486 | 0.261 |
| Tidjanibacter massiliensis | 0 | Not detected | -0.111 | -0.739 | 0.518 | 0.730 |
| Tissierellia bacterium S7-1-4 | 0 | Not detected | 0.035 | -0.393 | 0.464 | 0.872 |
| Trabulsiella odontotermitis | 0 | Not detected | -0.103 | -0.584 | 0.378 | 0.675 |
| Traorella massiliensis | 0 | Not detected | -0.017 | -0.185 | 0.152 | 0.845 |
| Treponema denticola | 0 | Not detected | 0.359 | 0.032 | 0.686 | 0.032 |
| Treponema medium | 0 | Not detected | -0.255 | -0.794 | 0.283 | 0.354 |
| Treponema putidum | 0 | Not detected | 0.175 | -0.131 | 0.481 | 0.265 |
| Treponema socranskii | 0 | Not detected | 0.050 | -0.291 | 0.390 | 0.775 |
| Treponema succinifaciens | 0 | Not detected | -0.021 | -0.228 | 0.185 | 0.841 |
| Treponema vincentii | 0 | Not detected | 0.213 | -0.101 | 0.527 | 0.185 |
| Trichococcus flocculiformis | 0 | Not detected | 0.001 | -0.365 | 0.366 | 0.997 |
| Trueperella pyogenes | 0 | Not detected | -0.230 | -0.626 | 0.165 | 0.254 |
| Turicibacter sanguinis | 0 | Not detected | -0.206 | -0.836 | 0.425 | 0.523 |
| Turicibacter sp. H121 | 0 | Not detected | -0.081 | -0.344 | 0.183 | 0.549 |
| Tyzzerella nexilis | 0 | Not detected | -0.077 | -0.240 | 0.086 | 0.355 |
| Urmitella timonensis | 0 | Not detected | 0.063 | -0.254 | 0.379 | 0.699 |
| Vaginella massiliensis | 0 | Not detected | 0.556 | 0.026 | 1.086 | 0.041 |
| Varibaculum cambriense | 0 | Not detected | 0.013 | -0.253 | 0.280 | 0.922 |
| Varibaculum sp. Marseille-P2802 | 0 | Not detected | -0.099 | -0.483 | 0.286 | 0.615 |
| Veillonella magna | 0 | Not detected | 0.018 | -0.170 | 0.206 | 0.851 |
| Veillonella parvula | 0 | Not detected | 0.515 | -0.099 | 1.130 | 0.101 |
| Veillonella seminalis | 0 | Not detected | 0.178 | -0.092 | 0.448 | 0.197 |
| Veillonella sp. ACP1 | 0 | Not detected | 0.411 | -0.270 | 1.091 | 0.238 |
| Veillonella sp. CAG:933 | 0 | Not detected | 0.137 | -0.273 | 0.547 | 0.513 |
| Veillonella sp. oral taxon 780 | 0 | Not detected | 0.130 | -0.283 | 0.542 | 0.538 |
| Waddlia chondrophila | 0 | Not detected | -0.329 | -0.834 | 0.175 | 0.202 |
| Weissella confusa | 0 | Not detected | 0.000 | -0.477 | 0.476 | 0.999 |
| Xanthomonas sp. Mitacek01 | 0 | Not detected | -0.097 | -0.353 | 0.159 | 0.458 |
| Yersinia enterocolitica | 0 | Not detected | -0.106 | -0.524 | 0.312 | 0.620 |
| Yersinia pestis | 0 | Not detected | -0.256 | -0.859 | 0.347 | 0.406 |
| Yokenella regensburgei | 0 | Not detected | -0.036 | -0.503 | 0.430 | 0.879 |
| [Bacteroides] pectinophilus | 0 | Not detected | 0.070 | -0.134 | 0.274 | 0.500 |
| [Clostridium] aminophilum | 0 | Not detected | -0.022 | -0.196 | 0.152 | 0.805 |
| [Clostridium] asparagiforme | 0 | Not detected | -0.149 | -0.393 | 0.094 | 0.230 |
| [Clostridium] bolteae | 0 | Not detected | -0.236 | -0.462 | -0.010 | 0.042 |
| [Clostridium] celerecrescens | 0 | Not detected | -0.034 | -0.187 | 0.120 | 0.668 |
| [Clostridium] cellobioparum | 0 | Not detected | -0.182 | -0.460 | 0.096 | 0.201 |
| [Clostridium] cellulolyticum | 0 | Not detected | 0.111 | -0.271 | 0.493 | 0.571 |
| [Clostridium] cellulosi | 0 | Not detected | -0.104 | -0.480 | 0.272 | 0.588 |

| [Clostridium] citroniae | 1 | Not detected | -0.217 | -0.368 | -0.065 | 0.005 |
| --- | --- | --- | --- | --- | --- | --- |
| [Clostridium] clariflavum | 0 | Not detected | -0.040 | -0.321 | 0.242 | 0.782 |
| [Clostridium] clostridioforme | 0 | Not detected | -0.126 | -0.391 | 0.140 | 0.355 |
| [Clostridium] dakarense | 0 | Not detected | 0.077 | -0.383 | 0.537 | 0.744 |
| [Clostridium] glycyrrhizinilyticum | 0 | Not detected | -0.028 | -0.184 | 0.127 | 0.721 |
| [Clostridium] hiranonis | 0 | Not detected | -0.207 | -0.704 | 0.291 | 0.416 |
| [Clostridium] hylemonae | 0 | Not detected | 0.064 | -0.307 | 0.434 | 0.736 |
| [Clostridium] innocuum | 0 | Not detected | -0.131 | -0.302 | 0.039 | 0.132 |
| [Clostridium] josui | 0 | Not detected | 0.133 | -0.266 | 0.532 | 0.515 |
| [Clostridium] lactatifermentans | 0 | Not detected | -0.126 | -0.344 | 0.092 | 0.258 |
| [Clostridium] leptum | 0 | Not detected | -0.205 | -0.513 | 0.103 | 0.194 |
| [Clostridium] methylpentosum | 0 | Not detected | 0.058 | -0.226 | 0.343 | 0.688 |
| [Clostridium] papyrosolvens | 0 | Not detected | 0.080 | -0.178 | 0.339 | 0.543 |
| [Clostridium] saccharogumia | 0 | Not detected | -0.005 | -0.224 | 0.214 | 0.964 |
| [Clostridium] saccharolyticum | 0 | Not detected | -0.097 | -0.335 | 0.140 | 0.421 |
| [Clostridium] scindens | 0 | Not detected | -0.127 | -0.333 | 0.080 | 0.230 |
| [Clostridium] spiroforme | 0 | Not detected | -0.202 | -0.667 | 0.262 | 0.394 |
| [Clostridium] sporosphaeroides | 0 | Not detected | 0.015 | -0.354 | 0.383 | 0.938 |
| [Clostridium] symbiosum | 0 | Not detected | -0.001 | -0.205 | 0.202 | 0.989 |
| [Clostridium] viride | 0 | Not detected | -0.217 | -0.559 | 0.125 | 0.214 |
| [Enterobacter] aerogenes | 0 | Not detected | -0.133 | -0.766 | 0.500 | 0.681 |
| [Eubacterium] brachy | 0 | Not detected | 0.544 | 0.116 | 0.973 | 0.013 |
| [Eubacterium] cellulosolvens | 0 | Not detected | -0.077 | -0.269 | 0.116 | 0.436 |
| [Eubacterium] contortum | 0 | Not detected | -0.057 | -0.209 | 0.094 | 0.459 |
| [Eubacterium] dolichum | 0 | Not detected | 0.015 | -0.154 | 0.183 | 0.866 |
| [Eubacterium] eligens | 0 | Not detected | 0.057 | -0.360 | 0.474 | 0.788 |
| [Eubacterium] fissicatena | 0 | Not detected | -0.065 | -0.259 | 0.129 | 0.510 |
| [Eubacterium] hallii | 0 | Not detected | -0.024 | -0.315 | 0.266 | 0.869 |
| [Eubacterium] nodatum | 0 | Not detected | 0.065 | -0.344 | 0.474 | 0.756 |
| [Eubacterium] rectale | 0 | Not detected | 0.006 | -0.391 | 0.402 | 0.977 |
| [Eubacterium] siraeum | 0 | Not detected | 0.039 | -0.537 | 0.616 | 0.894 |
| [Eubacterium] sulci | 0 | Not detected | -0.024 | -0.489 | 0.441 | 0.920 |
| [Haemophilus] parasuis | 0 | Not detected | 0.122 | -0.206 | 0.451 | 0.465 |
| [Ruminococcus] gnavus | 0 | Not detected | 0.073 | -0.258 | 0.403 | 0.668 |
| [Ruminococcus] torques | 1 | Not detected | -0.436 | -0.765 | -0.107 | 0.010 |
| bacterium F082 | 0 | Not detected | 0.016 | -0.241 | 0.274 | 0.903 |
| bacterium LF-3 | 0 | Not detected | -0.115 | -0.524 | 0.295 | 0.583 |
| bacterium MS4 | 0 | Not detected | -0.062 | -0.413 | 0.290 | 0.731 |
| bacterium OL-1 | 0 | Not detected | -0.272 | -0.739 | 0.195 | 0.255 |
| bacterium P201 | 0 | Not detected | -0.130 | -0.543 | 0.284 | 0.539 |
| bacterium P3 | 0 | Not detected | 0.385 | 0.006 | 0.765 | 0.047 |

| **Supplementary Table 21**. Association of current SEP with species in US-born participants (n=302). Analysis of Compositions of Microbiomes (ANCOM2) was used to identify species related to Lower current SEP. Regression coefficients were obtained by modelling the effect of Lower current SEP on centered log ratio (CLR)-transformed species in multivariable linear regression adjusted for age, gender, BMI, field center, Hispanic/Latino background, U.S. nativity, Bristol stool type, parental education, the alternative Healthy Eating Index 2010 score, cigarette smoking, alcohol use, depressive and anxiety symptoms, diabetes, hypertension, cardiovascular disease, dyslipidemia, and antibiotic use. | | | | | | |
| --- | --- | --- | --- | --- | --- | --- |
| **species** | **W_stat** | **ANCOM** | **beta** | **l95ci** | **u95ci** | **qval** |
| Acidaminococcus intestini | 860 | 0.7 | 1.61 | 0.451 | 2.778 | 0.007 |
| Acidaminococcus intestini CAG:325 | 781 | 0.7 | 2.15 | 0.530 | 3.770 | 0.010 |
| Bifidobacterium bifidum | 1050 | 0.9 | 1.97 | 0.871 | 3.068 | 0.001 |
| Collinsella aerofaciens | 834 | 0.7 | 1.20 | 0.336 | 2.060 | 0.007 |
| Collinsella sp. CAG:166 | 830 | 0.7 | 1.29 | 0.356 | 2.231 | 0.007 |
| Dialister sp. CAG:357 | 872 | 0.7 | 1.69 | 0.465 | 2.924 | 0.007 |
| Abiotrophia defectiva | 0 | Not detected | -0.15 | -0.757 | 0.448 | 0.616 |
| Acetanaerobacterium elongatum | 0 | Not detected | 0.26 | -0.237 | 0.761 | 0.304 |
| Acetitomaculum ruminis | 0 | Not detected | 0.03 | -0.321 | 0.383 | 0.863 |
| Acetivibrio cellulolyticus | 0 | Not detected | 0.16 | -0.339 | 0.658 | 0.532 |
| Acetivibrio ethanolgignens | 0 | Not detected | 0.08 | -0.194 | 0.347 | 0.581 |
| Acetobacter sp. 46_36 | 0 | Not detected | 0.49 | -0.678 | 1.650 | 0.414 |
| Acetobacter sp. CAG:267 | 0 | Not detected | -0.13 | -1.165 | 0.900 | 0.802 |
| Acholeplasma equifetale | 2 | Not detected | -0.15 | -0.738 | 0.433 | 0.610 |
| Acidaminococcus fermentans | 0 | Not detected | 0.55 | -0.049 | 1.147 | 0.073 |
| Acidaminococcus sp. CAG:542 | 0 | Not detected | 1.00 | 0.289 | 1.715 | 0.006 |
| Acinetobacter sp. CAG:196 | 0 | Not detected | 0.16 | -0.972 | 1.296 | 0.780 |
| Acinetobacter sp. CAG:196_36_41 | 0 | Not detected | 0.08 | -1.002 | 1.157 | 0.889 |
| Acinetobacter sp. N54.MGS-139 | 0 | Not detected | -0.27 | -0.947 | 0.411 | 0.440 |
| Acinetobacter venetianus | 0 | Not detected | 0.67 | 0.072 | 1.268 | 0.029 |
| Actinobaculum sp. oral taxon 183 | 0 | Not detected | -0.21 | -0.998 | 0.573 | 0.596 |
| Actinomyces dentalis | 0 | Not detected | 0.07 | -0.745 | 0.876 | 0.875 |
| Actinomyces gerencseriae | 0 | Not detected | -0.08 | -0.763 | 0.611 | 0.829 |
| Actinomyces graevenitzii | 0 | Not detected | 0.26 | -0.374 | 0.897 | 0.422 |
| Actinomyces hongkongensis | 0 | Not detected | 0.33 | -0.398 | 1.068 | 0.372 |
| Actinomyces israelii | 0 | Not detected | -0.07 | -0.825 | 0.679 | 0.850 |
| Actinomyces johnsonii | 0 | Not detected | 0.22 | -0.500 | 0.933 | 0.555 |
| Actinomyces massiliensis | 0 | Not detected | 0.30 | -0.410 | 1.018 | 0.405 |
| Actinomyces meyeri | 0 | Not detected | 0.69 | 0.107 | 1.280 | 0.021 |
| Actinomyces odontolyticus | 0 | Not detected | 0.29 | -0.334 | 0.915 | 0.362 |
| Actinomyces oris | 0 | Not detected | 0.58 | -0.090 | 1.250 | 0.091 |
| Actinomyces sp. HMSC065F12 | 0 | Not detected | 0.11 | -0.361 | 0.587 | 0.640 |
| Actinomyces sp. HMSC06A08 | 0 | Not detected | 0.13 | -0.336 | 0.592 | 0.590 |
| Actinomyces sp. oral taxon 181 | 0 | Not detected | 0.09 | -0.629 | 0.812 | 0.804 |
| Actinomyces sp. oral taxon 448 | 0 | Not detected | 0.31 | -0.534 | 1.152 | 0.473 |
| Actinomyces sp. oral taxon 848 | 0 | Not detected | 0.35 | -0.178 | 0.882 | 0.194 |
| Actinomyces sp. ph3 | 0 | Not detected | 0.19 | -0.529 | 0.905 | 0.608 |

| Actinomyces timonensis | 0 | Not detected | 0.37 | -0.186 | 0.921 | 0.194 |
| --- | --- | --- | --- | --- | --- | --- |
| Actinomyces turicensis | 727 | 0.6 | 1.62 | 0.876 | 2.358 | 0.000 |
| Adlercreutzia equolifaciens | 0 | Not detected | 0.14 | -0.808 | 1.090 | 0.772 |
| Aeromonas salmonicida | 0 | Not detected | 0.77 | 0.124 | 1.409 | 0.020 |
| Aggregatibacter aphrophilus | 0 | Not detected | 0.03 | -0.639 | 0.692 | 0.937 |
| Akkermansia glycaniphila | 0 | Not detected | 0.50 | -0.208 | 1.207 | 0.167 |
| Akkermansia muciniphila | 0 | Not detected | 0.29 | -0.754 | 1.342 | 0.583 |
| Akkermansia muciniphila CAG:154 | 0 | Not detected | 0.51 | -0.870 | 1.888 | 0.470 |
| Akkermansia sp. 54_46 | 0 | Not detected | 0.58 | -0.833 | 1.985 | 0.424 |
| Akkermansia sp. CAG:344 | 0 | Not detected | 1.02 | -0.371 | 2.403 | 0.152 |
| Akkermansia sp. KLE1605 | 0 | Not detected | 0.95 | -0.244 | 2.139 | 0.120 |
| Akkermansia sp. KLE1797 | 0 | Not detected | 0.92 | -0.268 | 2.115 | 0.130 |
| Akkermansia sp. KLE1798 | 0 | Not detected | 0.95 | -0.242 | 2.145 | 0.119 |
| Akkermansia sp. Phil8 | 0 | Not detected | 0.45 | -0.953 | 1.863 | 0.527 |
| Akkermansia sp. UNK.MGS-1 | 0 | Not detected | 0.60 | -0.794 | 1.991 | 0.400 |
| Alistipes finegoldii | 0 | Not detected | 0.12 | -0.460 | 0.707 | 0.679 |
| Alistipes ihumii | 0 | Not detected | 0.33 | -0.734 | 1.385 | 0.548 |
| Alistipes indistinctus | 0 | Not detected | 0.48 | -0.869 | 1.829 | 0.486 |
| Alistipes inops | 0 | Not detected | 0.34 | -0.640 | 1.319 | 0.498 |
| Alistipes obesi | 0 | Not detected | 0.15 | -0.472 | 0.771 | 0.638 |
| Alistipes putredinis | 0 | Not detected | 0.45 | -0.375 | 1.277 | 0.285 |
| Alistipes senegalensis | 0 | Not detected | 0.24 | -0.306 | 0.786 | 0.390 |
| Alistipes shahii | 0 | Not detected | 0.31 | -0.235 | 0.864 | 0.263 |
| Alistipes sp. CAG:435 | 0 | Not detected | -0.02 | -1.075 | 1.044 | 0.977 |
| Alistipes sp. CAG:514 | 0 | Not detected | 0.27 | -0.792 | 1.323 | 0.623 |
| Alistipes sp. Zagget8 | 0 | Not detected | 0.38 | -0.437 | 1.198 | 0.363 |
| Alistipes timonensis | 0 | Not detected | 0.13 | -0.799 | 1.056 | 0.787 |
| Alkaliflexus imshenetskii | 0 | Not detected | -0.05 | -0.573 | 0.464 | 0.838 |
| Allisonella histaminiformans | 0 | Not detected | 0.39 | -0.809 | 1.583 | 0.526 |
| Allobaculum stercoricanis | 0 | Not detected | 0.24 | -0.086 | 0.560 | 0.151 |
| Allofustis seminis | 0 | Not detected | 0.42 | -0.185 | 1.030 | 0.174 |
| Alloprevotella rava | 2 | Not detected | -0.17 | -0.684 | 0.347 | 0.522 |
| Alloprevotella tannerae | 0 | Not detected | -0.24 | -0.687 | 0.202 | 0.286 |
| Alloscardovia criceti | 0 | Not detected | 0.32 | -0.257 | 0.902 | 0.277 |
| Alloscardovia omnicolens | 0 | Not detected | -0.20 | -0.878 | 0.479 | 0.565 |
| Amphibacillus xylanus | 0 | Not detected | -0.07 | -0.675 | 0.530 | 0.814 |
| Anaerobium acetethylicum | 0 | Not detected | 0.10 | -0.205 | 0.414 | 0.510 |
| Anaerococcus hydrogenalis | 0 | Not detected | 0.52 | -0.026 | 1.056 | 0.063 |
| Anaerococcus lactolyticus | 0 | Not detected | 0.54 | -0.141 | 1.230 | 0.121 |
| Anaerococcus obesiensis | 0 | Not detected | 0.78 | 0.167 | 1.397 | 0.013 |
| Anaerococcus tetradius | 0 | Not detected | 0.45 | -0.101 | 1.003 | 0.111 |
| Anaerocolumna aminovalerica | 0 | Not detected | 0.02 | -0.556 | 0.599 | 0.942 |
| Anaerocolumna xylanovorans | 0 | Not detected | 0.09 | -0.441 | 0.626 | 0.734 |
| Anaerofustis stercorihominis | 0 | Not detected | -0.22 | -1.103 | 0.664 | 0.627 |

| Anaeroglobus geminatus | 0 | Not detected | -0.05 | -0.812 | 0.717 | 0.904 |
| --- | --- | --- | --- | --- | --- | --- |
| Anaeromassilibacillus senegalensis | 0 | Not detected | 0.16 | -0.382 | 0.695 | 0.569 |
| Anaerophaga thermohalophila | 0 | Not detected | 0.05 | -0.350 | 0.450 | 0.806 |
| Anaerosalibacter massiliensis | 0 | Not detected | -0.35 | -1.020 | 0.328 | 0.315 |
| Anaerosporobacter mobilis | 0 | Not detected | 0.16 | -0.207 | 0.530 | 0.391 |
| Anaerosporomusa subterranea | 0 | Not detected | 0.24 | -0.209 | 0.693 | 0.294 |
| Anaerostipes caccae | 0 | Not detected | -0.19 | -0.537 | 0.167 | 0.303 |
| Anaerostipes hadrus | 0 | Not detected | -0.07 | -0.653 | 0.506 | 0.803 |
| Anaerostipes sp. CAG:276 | 0 | Not detected | -0.96 | -2.107 | 0.184 | 0.101 |
| Anaerotruncus colihominis | 0 | Not detected | 0.16 | -0.182 | 0.510 | 0.354 |
| Anaerotruncus sp. CAG:390 | 0 | Not detected | 0.29 | -0.765 | 1.347 | 0.590 |
| Anaerotruncus sp. CAG:528 | 0 | Not detected | 0.76 | -0.109 | 1.635 | 0.087 |
| Anaerotruncus sp. G3(2012) | 0 | Not detected | 0.07 | -0.223 | 0.367 | 0.634 |
| Anaerovorax odorimutans | 0 | Not detected | -0.05 | -0.530 | 0.430 | 0.838 |
| Angelakisella massiliensis | 0 | Not detected | 0.01 | -0.302 | 0.312 | 0.973 |
| Aphanizomenon flos-aquae | 0 | Not detected | 0.15 | -0.274 | 0.577 | 0.486 |
| Aquabacterium parvum | 0 | Not detected | 0.33 | -0.152 | 0.820 | 0.180 |
| Arcanobacterium haemolyticum | 0 | Not detected | 0.24 | -0.341 | 0.812 | 0.424 |
| Asaccharospora irregularis | 0 | Not detected | 0.32 | -0.349 | 0.985 | 0.351 |
| Atlantibacter hermannii | 0 | Not detected | 0.30 | -0.463 | 1.071 | 0.438 |
| Atopobium deltae | 0 | Not detected | 0.40 | -0.232 | 1.042 | 0.214 |
| Atopobium minutum | 0 | Not detected | 0.59 | -0.055 | 1.228 | 0.074 |
| Atopobium parvulum | 0 | Not detected | -0.10 | -0.879 | 0.679 | 0.801 |
| Atopobium rimae | 0 | Not detected | -0.05 | -0.643 | 0.546 | 0.872 |
| Atopobium sp. BS2 | 0 | Not detected | 0.17 | -0.554 | 0.900 | 0.641 |
| Atopobium sp. HMSC064B08 | 2 | Not detected | -0.19 | -0.712 | 0.342 | 0.492 |
| Atopobium sp. ICM42b | 0 | Not detected | -0.28 | -1.015 | 0.457 | 0.458 |
| Atopobium vaginae | 0 | Not detected | 0.47 | -0.281 | 1.215 | 0.222 |
| Atopostipes suicloacalis | 0 | Not detected | 0.20 | -0.395 | 0.790 | 0.514 |
| Azospirillum sp. 47_25 | 0 | Not detected | 0.35 | -0.812 | 1.508 | 0.557 |
| Azospirillum sp. CAG:260 | 0 | Not detected | 0.44 | -0.663 | 1.538 | 0.437 |
| Bacillus acidiproducens | 0 | Not detected | -0.19 | -0.890 | 0.511 | 0.597 |
| Bacillus andreraoultii | 0 | Not detected | -0.26 | -0.932 | 0.409 | 0.446 |
| Bacillus azotoformans | 0 | Not detected | -0.03 | -0.628 | 0.576 | 0.933 |
| Bacillus bataviensis | 6 | Not detected | -0.48 | -1.076 | 0.118 | 0.117 |
| Bacillus coahuilensis | 0 | Not detected | 0.22 | -0.385 | 0.834 | 0.471 |
| Bacillus rubiinfantis | 0 | Not detected | 0.02 | -0.556 | 0.605 | 0.933 |
| Bacillus sp. CAG:988 | 0 | Not detected | -0.39 | -0.985 | 0.213 | 0.208 |
| Bacillus thermotolerans | 0 | Not detected | -0.19 | -0.828 | 0.455 | 0.570 |
| Bacteroidales bacterium 45-6 | 12 | Not detected | -0.59 | -1.184 | 0.000 | 0.051 |
| Bacteroidales bacterium 55_9 | 0 | Not detected | 0.27 | -0.168 | 0.702 | 0.230 |
| Bacteroidales bacterium Bact_08 | 0 | Not detected | 0.15 | -0.391 | 0.684 | 0.593 |
| Bacteroidales bacterium Bact_18 | 0 | Not detected | -0.02 | -0.526 | 0.482 | 0.932 |
| Bacteroidales bacterium Bact_23 | 3 | Not detected | -0.43 | -1.121 | 0.260 | 0.223 |

| Bacteroidales bacterium CF | 0 | Not detected | 0.42 | -0.087 | 0.935 | 0.105 |
| --- | --- | --- | --- | --- | --- | --- |
| Bacteroidales bacterium H10 | 0 | Not detected | -0.09 | -0.642 | 0.455 | 0.738 |
| Bacteroidales bacterium K1 | 0 | Not detected | -0.10 | -0.638 | 0.442 | 0.722 |
| Bacteroidales bacterium K10 | 0 | Not detected | -0.15 | -0.718 | 0.423 | 0.613 |
| Bacteroides barnesiae | 0 | Not detected | 0.09 | -0.313 | 0.503 | 0.649 |
| Bacteroides caccae CAG:21 | 484 | Not detected | 0.99 | 0.213 | 1.759 | 0.013 |
| Bacteroides cellulosilyticus | 0 | Not detected | -0.15 | -0.795 | 0.492 | 0.646 |
| Bacteroides coprocola | 0 | Not detected | -0.12 | -0.646 | 0.399 | 0.643 |
| Bacteroides coprophilus | 0 | Not detected | -0.03 | -0.422 | 0.353 | 0.860 |
| Bacteroides coprosuis | 0 | Not detected | -0.08 | -0.583 | 0.429 | 0.765 |
| Bacteroides dorei CAG:222 | 0 | Not detected | -0.16 | -0.776 | 0.454 | 0.609 |
| Bacteroides faecichinchillae | 0 | Not detected | 0.01 | -0.380 | 0.401 | 0.957 |
| Bacteroides faecis CAG:32 | 0 | Not detected | 0.04 | -0.655 | 0.731 | 0.914 |
| Bacteroides finegoldii | 0 | Not detected | -0.28 | -1.008 | 0.440 | 0.442 |
| Bacteroides finegoldii CAG:203 | 0 | Not detected | -0.28 | -0.958 | 0.406 | 0.428 |
| Bacteroides fluxus | 0 | Not detected | 0.03 | -0.384 | 0.451 | 0.874 |
| Bacteroides fragilis | 0 | Not detected | 0.25 | -0.269 | 0.765 | 0.348 |
| Bacteroides fragilis CAG:47 | 0 | Not detected | 0.11 | -0.732 | 0.951 | 0.799 |
| Bacteroides graminisolvens | 0 | Not detected | 0.20 | -0.535 | 0.931 | 0.597 |
| Bacteroides neonati | 0 | Not detected | 0.00 | -0.365 | 0.362 | 0.993 |
| Bacteroides oleiciplenus | 0 | Not detected | -0.07 | -0.531 | 0.389 | 0.762 |
| Bacteroides ovatus | 0 | Not detected | 0.03 | -0.509 | 0.566 | 0.918 |
| Bacteroides ovatus CAG:22 | 0 | Not detected | -0.09 | -0.679 | 0.492 | 0.754 |
| Bacteroides paurosaccharolyticus | 0 | Not detected | -0.01 | -0.463 | 0.447 | 0.972 |
| Bacteroides pectinophilus CAG:437 | 0 | Not detected | 0.15 | -0.326 | 0.619 | 0.544 |
| Bacteroides plebeius | 0 | Not detected | 0.02 | -0.512 | 0.557 | 0.934 |
| Bacteroides propionicifaciens | 16 | Not detected | -0.57 | -1.137 | 0.004 | 0.053 |
| Bacteroides pyogenes | 0 | Not detected | 0.16 | -0.248 | 0.570 | 0.442 |
| Bacteroides reticulotermitis | 0 | Not detected | -0.01 | -0.479 | 0.457 | 0.962 |
| Bacteroides salanitronis | 0 | Not detected | -0.26 | -0.673 | 0.158 | 0.226 |
| Bacteroides salyersiae | 0 | Not detected | -0.19 | -0.685 | 0.297 | 0.440 |
| Bacteroides sp. 41_26 | 0 | Not detected | -0.20 | -0.816 | 0.421 | 0.532 |
| Bacteroides sp. 43_46 | 565 | Not detected | 0.79 | 0.240 | 1.343 | 0.005 |
| Bacteroides sp. CAG:1060 | 0 | Not detected | 0.40 | -0.451 | 1.257 | 0.356 |
| Bacteroides sp. CAG:1076 | 0 | Not detected | -0.39 | -1.104 | 0.328 | 0.290 |
| Bacteroides sp. CAG:530 | 0 | Not detected | 0.17 | -0.321 | 0.667 | 0.493 |
| Bacteroides sp. CAG:545 | 0 | Not detected | -0.03 | -1.153 | 1.087 | 0.954 |
| Bacteroides sp. CAG:714 | 0 | Not detected | 0.17 | -0.677 | 1.015 | 0.695 |
| Bacteroides sp. CAG:770 | 0 | Not detected | 0.50 | -0.089 | 1.091 | 0.097 |
| Bacteroides sp. CAG:98 | 0 | Not detected | 0.45 | -0.489 | 1.393 | 0.348 |
| Bacteroides sp. D2 | 0 | Not detected | -0.10 | -0.691 | 0.494 | 0.745 |
| Bacteroides sp. Ga6A1 | 0 | Not detected | -0.09 | -0.894 | 0.706 | 0.819 |
| Bacteroides sp. Ga6A2 | 0 | Not detected | 0.15 | -0.613 | 0.918 | 0.696 |
| Bacteroides stercoris | 0 | Not detected | -0.51 | -1.244 | 0.223 | 0.174 |

| Bacteroides thetaiotaomicron | 0 | Not detected | 0.00 | -0.425 | 0.421 | 0.992 |
| --- | --- | --- | --- | --- | --- | --- |
| Bacteroides thetaiotaomicron CAG:40 | 0 | Not detected | -0.41 | -1.051 | 0.235 | 0.215 |
| Bacteroides uniformis | 0 | Not detected | 0.01 | -0.599 | 0.613 | 0.983 |
| Bacteroides vulgatus | 0 | Not detected | -0.09 | -0.633 | 0.448 | 0.737 |
| Bacteroides vulgatus CAG:6 | 0 | Not detected | -0.11 | -0.736 | 0.520 | 0.736 |
| Bacteroidetes bacterium OLB8 | 3 | Not detected | -0.36 | -0.969 | 0.246 | 0.245 |
| Bacteroidetes oral taxon 274 | 2 | Not detected | -0.12 | -0.560 | 0.312 | 0.577 |
| Bariatricus massiliensis | 0 | Not detected | -0.14 | -0.435 | 0.146 | 0.330 |
| Barnesiella intestinihominis | 0 | Not detected | 0.51 | -0.176 | 1.205 | 0.145 |
| Barnesiella viscericola | 0 | Not detected | -0.13 | -0.481 | 0.215 | 0.455 |
| Bavariicoccus seileri | 3 | Not detected | -0.32 | -0.845 | 0.196 | 0.223 |
| Beduini massiliensis | 0 | Not detected | -0.06 | -0.467 | 0.348 | 0.775 |
| Bibersteinia trehalosi | 0 | Not detected | -0.03 | -0.727 | 0.668 | 0.935 |
| Bifidobacterium actinocoloniiforme | 0 | Not detected | 0.74 | 0.091 | 1.390 | 0.026 |
| Bifidobacterium adolescentis | 0 | Not detected | 0.73 | -0.386 | 1.845 | 0.201 |
| Bifidobacterium aesculapii | 0 | Not detected | 0.37 | -0.264 | 1.008 | 0.253 |
| Bifidobacterium angulatum | 0 | Not detected | 0.97 | 0.022 | 1.913 | 0.046 |
| Bifidobacterium animalis | 0 | Not detected | 0.04 | -0.608 | 0.694 | 0.897 |
| Bifidobacterium asteroides | 0 | Not detected | 0.51 | -0.183 | 1.197 | 0.151 |
| Bifidobacterium biavatii | 0 | Not detected | 0.26 | -0.076 | 0.586 | 0.133 |
| Bifidobacterium bohemicum | 0 | Not detected | 0.42 | -0.202 | 1.049 | 0.186 |
| Bifidobacterium bombi | 0 | Not detected | 0.34 | -0.280 | 0.952 | 0.286 |
| Bifidobacterium breve | 0 | Not detected | 0.90 | -0.164 | 1.973 | 0.099 |
| Bifidobacterium callitrichos | 0 | Not detected | 0.86 | 0.013 | 1.710 | 0.048 |
| Bifidobacterium choerinum | 0 | Not detected | 0.55 | -0.143 | 1.237 | 0.121 |
| Bifidobacterium coryneforme | 1 | Not detected | 0.12 | -0.536 | 0.780 | 0.717 |
| Bifidobacterium crudilactis | 3 | Not detected | -0.21 | -0.742 | 0.324 | 0.443 |
| Bifidobacterium cuniculi | 0 | Not detected | 0.51 | -0.181 | 1.202 | 0.149 |
| Bifidobacterium dentium | 0 | Not detected | 0.49 | -0.486 | 1.458 | 0.328 |
| Bifidobacterium gallicum | 0 | Not detected | 0.56 | -0.153 | 1.263 | 0.126 |
| Bifidobacterium gallinarum | 0 | Not detected | 0.67 | -0.244 | 1.589 | 0.152 |
| Bifidobacterium longum | 0 | Not detected | 0.80 | -0.256 | 1.848 | 0.139 |
| Bifidobacterium magnum | 0 | Not detected | 0.11 | -0.370 | 0.595 | 0.647 |
| Bifidobacterium minimum | 0 | Not detected | 0.54 | -0.369 | 1.451 | 0.245 |
| Bifidobacterium mongoliense | 0 | Not detected | 0.24 | -0.410 | 0.884 | 0.474 |
| Bifidobacterium pseudolongum | 0 | Not detected | 0.37 | -0.361 | 1.108 | 0.320 |
| Bifidobacterium psychraerophilum | 0 | Not detected | 0.21 | -0.288 | 0.699 | 0.415 |
| Bifidobacterium reuteri | 0 | Not detected | 0.39 | -0.370 | 1.141 | 0.319 |
| Bifidobacterium saguini | 0 | Not detected | 0.57 | -0.376 | 1.524 | 0.238 |
| Bifidobacterium scardovii | 0 | Not detected | 1.03 | 0.059 | 1.993 | 0.039 |
| Bifidobacterium stellenboschense | 0 | Not detected | 0.31 | -0.044 | 0.657 | 0.088 |
| Bifidobacterium subtile | 0 | Not detected | 0.66 | -0.159 | 1.486 | 0.115 |
| Bifidobacterium thermophilum | 0 | Not detected | 0.17 | -0.344 | 0.685 | 0.516 |
| Bifidobacterium tsurumiense | 0 | Not detected | 0.60 | -0.120 | 1.328 | 0.103 |

| Bilophila wadsworthia | 0 | Not detected | 0.39 | -0.876 | 1.652 | 0.548 |
| --- | --- | --- | --- | --- | --- | --- |
| Bittarella massiliensis | 0 | Not detected | 0.03 | -0.257 | 0.324 | 0.820 |
| Blautia hansenii | 0 | Not detected | -0.25 | -0.650 | 0.145 | 0.215 |
| Blautia hydrogenotrophica | 0 | Not detected | -0.05 | -0.383 | 0.282 | 0.766 |
| Blautia hydrogenotrophica CAG:147 | 0 | Not detected | 0.39 | -0.487 | 1.264 | 0.385 |
| Blautia obeum | 0 | Not detected | 0.09 | -0.228 | 0.416 | 0.569 |
| Blautia producta | 0 | Not detected | -0.28 | -0.722 | 0.169 | 0.225 |
| Blautia schinkii | 0 | Not detected | 0.10 | -0.129 | 0.335 | 0.384 |
| Blautia sp. CAG:237 | 0 | Not detected | -0.07 | -0.748 | 0.601 | 0.831 |
| Blautia sp. CAG:37 | 0 | Not detected | 0.54 | -0.112 | 1.193 | 0.106 |
| Blautia sp. CAG:37_48_57 | 0 | Not detected | 0.25 | -0.217 | 0.723 | 0.293 |
| Blautia sp. KLE 1732 | 0 | Not detected | -0.01 | -0.406 | 0.387 | 0.963 |
| Bulleidia extructa | 0 | Not detected | -0.15 | -0.855 | 0.562 | 0.685 |
| Burkholderiales bacterium GJ-E10 | 0 | Not detected | 0.41 | -0.220 | 1.036 | 0.204 |
| Butyricicoccus desmolans | 0 | Not detected | 0.05 | -0.221 | 0.311 | 0.740 |
| Butyricicoccus pullicaecorum | 0 | Not detected | 0.07 | -0.276 | 0.411 | 0.701 |
| Butyricicoccus sp. K4410.MGS-46 | 0 | Not detected | 0.10 | -0.388 | 0.593 | 0.683 |
| Butyricimonas sp. Marseille-P2440 | 0 | Not detected | -0.25 | -0.928 | 0.435 | 0.479 |
| Butyricimonas synergistica | 0 | Not detected | 0.39 | -0.402 | 1.186 | 0.334 |
| Butyricimonas virosa | 0 | Not detected | 0.00 | -0.704 | 0.701 | 0.996 |
| Butyrivibrio crossotus | 0 | Not detected | -0.07 | -0.496 | 0.346 | 0.728 |
| Butyrivibrio crossotus CAG:259 | 0 | Not detected | 0.13 | -0.553 | 0.822 | 0.702 |
| Butyrivibrio fibrisolvens | 0 | Not detected | -0.26 | -0.785 | 0.262 | 0.329 |
| Butyrivibrio hungatei | 0 | Not detected | -0.23 | -0.777 | 0.313 | 0.405 |
| Butyrivibrio proteoclasticus | 0 | Not detected | 0.09 | -0.283 | 0.458 | 0.644 |
| Butyrivibrio sp. AD3002 | 0 | Not detected | 0.02 | -0.415 | 0.463 | 0.914 |
| Butyrivibrio sp. AE2005 | 13 | Not detected | -0.43 | -0.911 | 0.045 | 0.077 |
| Butyrivibrio sp. AE3003 | 0 | Not detected | -0.06 | -0.473 | 0.356 | 0.781 |
| Butyrivibrio sp. AE3004 | 0 | Not detected | 0.17 | -0.157 | 0.501 | 0.306 |
| Butyrivibrio sp. AE3009 | 0 | Not detected | -0.07 | -0.561 | 0.418 | 0.776 |
| Butyrivibrio sp. FCS006 | 0 | Not detected | 0.08 | -0.441 | 0.604 | 0.759 |
| Butyrivibrio sp. INlla16 | 0 | Not detected | 0.21 | -0.252 | 0.670 | 0.374 |
| Butyrivibrio sp. INlla21 | 0 | Not detected | 0.22 | -0.269 | 0.715 | 0.375 |
| Butyrivibrio sp. LB2008 | 0 | Not detected | 0.08 | -0.421 | 0.584 | 0.752 |
| Butyrivibrio sp. M55 | 16 | Not detected | -0.48 | -1.003 | 0.039 | 0.071 |
| Butyrivibrio sp. NC2002 | 0 | Not detected | 0.07 | -0.457 | 0.600 | 0.790 |
| Butyrivibrio sp. NC2007 | 0 | Not detected | -0.19 | -0.685 | 0.300 | 0.445 |
| Butyrivibrio sp. NC3005 | 4 | Not detected | -0.33 | -1.014 | 0.361 | 0.353 |
| Butyrivibrio sp. VCD2006 | 0 | Not detected | 0.09 | -0.401 | 0.578 | 0.723 |
| Butyrivibrio sp. WCD2001 | 0 | Not detected | -0.11 | -0.608 | 0.396 | 0.679 |
| Butyrivibrio sp. WCD3002 | 0 | Not detected | -0.13 | -0.600 | 0.342 | 0.592 |
| Butyrivibrio sp. ob235 | 2 | Not detected | -0.18 | -0.643 | 0.280 | 0.442 |
| Campylobacter coli | 0 | Not detected | -0.01 | -0.440 | 0.422 | 0.968 |
| Campylobacter hominis | 0 | Not detected | 0.36 | -0.360 | 1.071 | 0.331 |

| Candidatus Azobacteroides pseudotricho | 2 | Not detected | -0.12 | -0.567 | 0.328 | 0.602 |
| --- | --- | --- | --- | --- | --- | --- |
| Candidatus Gastranaerophilales bacteriu | 0 | Not detected | 0.01 | -0.736 | 0.747 | 0.989 |
| Candidatus Melainabacteria bacterium L | 0 | Not detected | 0.10 | -0.354 | 0.560 | 0.660 |
| Candidatus Melainabacteria bacterium M | 0 | Not detected | -0.03 | -0.854 | 0.785 | 0.934 |
| Candidatus Saccharibacteria oral taxon T | 0 | Not detected | -0.03 | -0.583 | 0.527 | 0.920 |
| Candidatus Soleaferrea massiliensis | 0 | Not detected | 0.17 | -0.292 | 0.638 | 0.468 |
| Candidatus Stoquefichus sp. SB1 | 0 | Not detected | 0.32 | -0.117 | 0.761 | 0.152 |
| Capnocytophaga granulosa | 0 | Not detected | 0.20 | -0.339 | 0.737 | 0.470 |
| Capnocytophaga sp. oral taxon 329 | 0 | Not detected | -0.14 | -0.913 | 0.628 | 0.718 |
| Capnocytophaga sp. oral taxon 863 | 0 | Not detected | 0.13 | -0.557 | 0.814 | 0.714 |
| Capnocytophaga sputigena | 2 | Not detected | -0.18 | -0.625 | 0.272 | 0.442 |
| Carnobacterium gallinarum | 0 | Not detected | 0.28 | -0.378 | 0.935 | 0.407 |
| Catabacter hongkongensis | 0 | Not detected | 0.07 | -0.632 | 0.770 | 0.847 |
| Catenibacterium mitsuokai | 426 | Not detected | 1.25 | 0.208 | 2.293 | 0.020 |
| Catenibacterium sp. CAG:290 | 0 | Not detected | 1.22 | -0.038 | 2.470 | 0.059 |
| Catonella morbi | 0 | Not detected | -0.07 | -0.416 | 0.286 | 0.717 |
| Cedecea neteri | 0 | Not detected | -0.02 | -0.686 | 0.638 | 0.944 |
| Cellulomonas carbonis | 0 | Not detected | -0.21 | -0.970 | 0.551 | 0.590 |
| Cellulomonas flavigena | 0 | Not detected | 0.26 | -0.471 | 0.986 | 0.488 |
| Cellulosilyticum lentocellum | 0 | Not detected | -0.06 | -0.593 | 0.482 | 0.841 |
| Cellulosilyticum ruminicola | 0 | Not detected | 0.26 | -0.283 | 0.806 | 0.348 |
| Chlamydia psittaci | 0 | Not detected | 0.10 | -0.434 | 0.631 | 0.718 |
| Christensenella sp. AF73-05CM02 | 0 | Not detected | 0.35 | -0.331 | 1.032 | 0.314 |
| Christensenellaceae bacterium Phil1 | 0 | Not detected | 0.13 | -0.394 | 0.649 | 0.632 |
| Christensenellaceae bacterium Phil10 | 0 | Not detected | 0.44 | -0.402 | 1.283 | 0.306 |
| Christensenellaceae bacterium Phil7 | 0 | Not detected | -0.10 | -0.927 | 0.729 | 0.815 |
| Chryseobacterium gallinarum | 0 | Not detected | 0.13 | -0.860 | 1.117 | 0.799 |
| Chryseobacterium greenlandense | 0 | Not detected | 0.17 | -0.669 | 1.012 | 0.689 |
| Citrobacter amalonaticus | 0 | Not detected | 0.37 | -0.595 | 1.332 | 0.454 |
| Citrobacter freundii | 0 | Not detected | 0.63 | -0.419 | 1.678 | 0.241 |
| Cloacibacillus evryensis | 0 | Not detected | -0.33 | -1.211 | 0.541 | 0.454 |
| Cloacibacillus porcorum | 0 | Not detected | -0.36 | -1.139 | 0.425 | 0.372 |
| Cloacibacterium normanense | 0 | Not detected | 0.65 | -0.088 | 1.397 | 0.085 |
| Clostridia bacterium UC5.1-1C12 | 0 | Not detected | 0.73 | -0.150 | 1.616 | 0.105 |
| Clostridia bacterium UC5.1-1D1 | 0 | Not detected | -0.01 | -0.318 | 0.294 | 0.939 |
| Clostridia bacterium UC5.1-1E11 | 0 | Not detected | 0.12 | -0.211 | 0.447 | 0.484 |
| Clostridiales bacterium 1_7_47FAA | 0 | Not detected | -0.07 | -0.397 | 0.264 | 0.694 |
| Clostridiales bacterium 59_14 | 0 | Not detected | 0.59 | -0.005 | 1.180 | 0.053 |
| Clostridiales bacterium DTU035 | 4 | Not detected | -0.37 | -0.880 | 0.134 | 0.151 |
| Clostridiales bacterium DTU036 | 0 | Not detected | -0.14 | -0.617 | 0.346 | 0.581 |
| Clostridiales bacterium DTU089 | 0 | Not detected | -0.17 | -0.736 | 0.393 | 0.552 |
| Clostridiales bacterium Firm_03 | 0 | Not detected | -0.45 | -1.212 | 0.320 | 0.255 |
| Clostridiales bacterium Firm_16 | 0 | Not detected | 0.00 | -0.803 | 0.800 | 0.998 |
| Clostridiales bacterium NK3B98 | 0 | Not detected | -0.09 | -0.705 | 0.529 | 0.780 |

| Clostridiales bacterium S5-A14a | 0 | Not detected | 0.10 | -0.238 | 0.435 | 0.566 |
| --- | --- | --- | --- | --- | --- | --- |
| Clostridiales bacterium SIT11 | 0 | Not detected | 0.59 | -0.009 | 1.189 | 0.055 |
| Clostridiales bacterium VE202-08 | 0 | Not detected | 0.30 | -0.043 | 0.642 | 0.088 |
| Clostridioides difficile | 0 | Not detected | -0.07 | -0.355 | 0.206 | 0.602 |
| Clostridioides mangenotii | 0 | Not detected | 0.06 | -0.469 | 0.597 | 0.813 |
| Clostridium baratii | 0 | Not detected | 0.46 | -0.105 | 1.031 | 0.111 |
| Clostridium beijerinckii | 0 | Not detected | 0.15 | -0.341 | 0.645 | 0.547 |
| Clostridium bolteae CAG:59 | 0 | Not detected | -0.05 | -0.581 | 0.482 | 0.854 |
| Clostridium botulinum | 0 | Not detected | 0.03 | -0.562 | 0.615 | 0.930 |
| Clostridium cadaveris | 0 | Not detected | -0.02 | -0.686 | 0.654 | 0.962 |
| Clostridium celatum | 0 | Not detected | -0.17 | -1.139 | 0.796 | 0.729 |
| Clostridium cellulovorans | 0 | Not detected | 0.02 | -0.429 | 0.470 | 0.928 |
| Clostridium clostridioforme CAG:132 | 0 | Not detected | -0.11 | -0.864 | 0.640 | 0.770 |
| Clostridium clostridioforme CAG:511 | 0 | Not detected | -0.40 | -1.091 | 0.297 | 0.264 |
| Clostridium disporicum | 0 | Not detected | -0.04 | -0.609 | 0.528 | 0.888 |
| Clostridium ihumii | 0 | Not detected | 0.33 | -0.178 | 0.839 | 0.204 |
| Clostridium kluyveri | 0 | Not detected | 0.09 | -0.593 | 0.783 | 0.787 |
| Clostridium leptum CAG:27 | 0 | Not detected | -0.20 | -1.089 | 0.694 | 0.665 |
| Clostridium lundense | 0 | Not detected | -0.04 | -0.734 | 0.652 | 0.908 |
| Clostridium nexile CAG:348 | 0 | Not detected | -0.10 | -0.693 | 0.498 | 0.750 |
| Clostridium paraputrificum | 0 | Not detected | 0.32 | -0.440 | 1.081 | 0.409 |
| Clostridium perfringens | 0 | Not detected | 0.18 | -0.742 | 1.104 | 0.701 |
| Clostridium phoceensis | 0 | Not detected | -0.21 | -0.667 | 0.252 | 0.378 |
| Clostridium polynesiense | 0 | Not detected | 0.20 | -0.502 | 0.912 | 0.571 |
| Clostridium sartagoforme | 0 | Not detected | -0.24 | -0.829 | 0.353 | 0.431 |
| Clostridium sp. 44_14 | 0 | Not detected | 0.14 | -0.486 | 0.771 | 0.656 |
| Clostridium sp. ASF356 | 0 | Not detected | 0.04 | -0.654 | 0.732 | 0.912 |
| Clostridium sp. CAG:122 | 0 | Not detected | 0.48 | -0.407 | 1.366 | 0.290 |
| Clostridium sp. CAG:12237_41 | 0 | Not detected | 0.45 | -0.252 | 1.148 | 0.211 |
| Clostridium sp. CAG:127 | 0 | Not detected | -0.34 | -1.358 | 0.671 | 0.508 |
| Clostridium sp. CAG:138 | 0 | Not detected | 1.05 | -0.223 | 2.333 | 0.107 |
| Clostridium sp. CAG:167 | 0 | Not detected | 0.25 | -0.808 | 1.302 | 0.646 |
| Clostridium sp. CAG:226 | 0 | Not detected | -0.24 | -1.198 | 0.728 | 0.633 |
| Clostridium sp. CAG:230 | 0 | Not detected | 0.15 | -0.554 | 0.851 | 0.679 |
| Clostridium sp. CAG:242 | 0 | Not detected | -0.04 | -0.976 | 0.900 | 0.937 |
| Clostridium sp. CAG:245 | 0 | Not detected | 0.53 | -0.829 | 1.883 | 0.447 |
| Clostridium sp. CAG:245_30_32 | 0 | Not detected | 0.30 | -0.350 | 0.957 | 0.364 |
| Clostridium sp. CAG:264 | 0 | Not detected | -0.09 | -0.962 | 0.789 | 0.847 |
| Clostridium sp. CAG:277 | 0 | Not detected | 0.28 | -0.357 | 0.915 | 0.391 |
| Clostridium sp. CAG:299 | 0 | Not detected | -0.18 | -1.054 | 0.693 | 0.686 |
| Clostridium sp. CAG:307_30_263 | 0 | Not detected | 0.22 | -0.394 | 0.838 | 0.481 |
| Clostridium sp. CAG:349 | 0 | Not detected | 0.30 | -0.736 | 1.338 | 0.570 |
| Clostridium sp. CAG:352 | 0 | Not detected | 0.16 | -0.688 | 1.013 | 0.708 |
| Clostridium sp. CAG:413 | 0 | Not detected | -0.04 | -1.017 | 0.928 | 0.928 |

| Clostridium sp. CAG:448 | 0 | Not detected | -0.38 | -1.179 | 0.413 | 0.347 |
| --- | --- | --- | --- | --- | --- | --- |
| Clostridium sp. CAG:451 | 0 | Not detected | -0.42 | -1.431 | 0.592 | 0.417 |
| Clostridium sp. CAG:465 | 0 | Not detected | 0.62 | -0.280 | 1.511 | 0.179 |
| Clostridium sp. CAG:505 | 0 | Not detected | 1.13 | 0.072 | 2.187 | 0.037 |
| Clostridium sp. CAG:568 | 0 | Not detected | 0.22 | -0.513 | 0.961 | 0.552 |
| Clostridium sp. CAG:58 | 0 | Not detected | 0.61 | -0.128 | 1.348 | 0.106 |
| Clostridium sp. CAG:590 | 0 | Not detected | 0.00 | -0.534 | 0.536 | 0.998 |
| Clostridium sp. CAG:594 | 0 | Not detected | 0.49 | -0.395 | 1.382 | 0.277 |
| Clostridium sp. CAG:628 | 0 | Not detected | -0.27 | -1.093 | 0.548 | 0.515 |
| Clostridium sp. CAG:62_40_43 | 0 | Not detected | 0.47 | -0.751 | 1.687 | 0.452 |
| Clostridium sp. CAG:632 | 0 | Not detected | 0.16 | -0.549 | 0.866 | 0.661 |
| Clostridium sp. CAG:678 | 0 | Not detected | -0.12 | -0.949 | 0.716 | 0.784 |
| Clostridium sp. CAG:768 | 0 | Not detected | 0.11 | -0.353 | 0.571 | 0.645 |
| Clostridium sp. CAG:780 | 0 | Not detected | 0.39 | -0.662 | 1.441 | 0.469 |
| Clostridium sp. CAG:793 | 0 | Not detected | 0.56 | -0.283 | 1.399 | 0.195 |
| Clostridium sp. CAG:81 | 0 | Not detected | -0.34 | -0.939 | 0.260 | 0.268 |
| Clostridium sp. CAG:91 | 0 | Not detected | 0.01 | -0.914 | 0.943 | 0.976 |
| Clostridium sp. CAG:964 | 0 | Not detected | 0.18 | -0.469 | 0.833 | 0.584 |
| Clostridium sp. HGF2 | 0 | Not detected | 0.07 | -0.247 | 0.379 | 0.680 |
| Clostridium sp. K4410.MGS-306 | 0 | Not detected | -0.14 | -0.552 | 0.282 | 0.526 |
| Clostridium sp. KLE 1755 | 0 | Not detected | 0.06 | -0.413 | 0.538 | 0.795 |
| Clostridium sp. KNHs205 | 0 | Not detected | -0.16 | -0.393 | 0.080 | 0.196 |
| Clostridium sp. KNHs209 | 0 | Not detected | -0.06 | -0.302 | 0.188 | 0.649 |
| Clostridium sp. KNHs214 | 0 | Not detected | 0.37 | -0.088 | 0.823 | 0.115 |
| Clostridium sp. L2-50 | 0 | Not detected | 0.25 | -0.072 | 0.566 | 0.131 |
| Clostridium sp. M62/1 | 0 | Not detected | 0.18 | -0.077 | 0.443 | 0.169 |
| Clostridium sp. SY8519 | 0 | Not detected | 0.08 | -0.223 | 0.386 | 0.601 |
| Clostridium sulfidigenes | 0 | Not detected | 0.06 | -0.446 | 0.569 | 0.812 |
| Collinsella intestinalis | 0 | Not detected | 0.68 | -0.354 | 1.716 | 0.199 |
| Collinsella sp. MS5 | 0 | Not detected | 0.77 | 0.122 | 1.423 | 0.021 |
| Collinsella stercoris | 0 | Not detected | 0.72 | -0.147 | 1.577 | 0.105 |
| Collinsella tanakaei | 0 | Not detected | 0.34 | -0.289 | 0.963 | 0.293 |
| Comamonas kerstersii | 0 | Not detected | 0.24 | -0.327 | 0.811 | 0.406 |
| Coprobacillus sp. CAG:605 | 0 | Not detected | 0.10 | -0.731 | 0.928 | 0.816 |
| Coprobacillus sp. D6 | 676 | 0.6 | 0.72 | 0.297 | 1.149 | 0.001 |
| Coprobacter secundus | 0 | Not detected | 0.18 | -0.193 | 0.556 | 0.344 |
| Coprococcus comes | 0 | Not detected | 0.22 | -0.112 | 0.544 | 0.199 |
| Coprococcus eutactus | 0 | Not detected | 0.13 | -0.221 | 0.481 | 0.469 |
| Coprococcus eutactus CAG:665 | 0 | Not detected | 0.75 | -0.096 | 1.597 | 0.084 |
| Coprococcus sp. 43_8 | 0 | Not detected | 0.51 | -0.202 | 1.227 | 0.161 |
| Coprococcus sp. CAG:131 | 0 | Not detected | 0.18 | -0.666 | 1.028 | 0.676 |
| Coprococcus sp. CAG:131_42_139 | 0 | Not detected | 0.35 | -0.538 | 1.231 | 0.443 |
| Coprococcus sp. HPP0048 | 0 | Not detected | 0.02 | -0.494 | 0.529 | 0.945 |
| Coriobacteriaceae bacterium 68-1-3 | 0 | Not detected | 0.37 | -0.241 | 0.977 | 0.237 |

| Coriobacteriaceae bacterium BV3Ac1 | 2 | Not detected | -0.51 | -1.288 | 0.269 | 0.201 |
| --- | --- | --- | --- | --- | --- | --- |
| Coriobacteriales bacterium DNF00809 | 0 | Not detected | 0.48 | -0.196 | 1.152 | 0.166 |
| Coriobacterium glomerans | 0 | Not detected | 0.81 | 0.250 | 1.378 | 0.005 |
| Corynebacterium amycolatum | 0 | Not detected | 0.06 | -0.465 | 0.576 | 0.835 |
| Corynebacterium durum | 0 | Not detected | 0.45 | -0.205 | 1.100 | 0.180 |
| Corynebacterium glucuronolyticum | 0 | Not detected | 0.10 | -0.495 | 0.699 | 0.738 |
| Corynebacterium urealyticum | 0 | Not detected | 0.13 | -0.354 | 0.622 | 0.591 |
| Criibacterium bergeronii | 0 | Not detected | 0.18 | -0.235 | 0.597 | 0.395 |
| Cronobacter sakazakii | 0 | Not detected | 0.48 | -0.468 | 1.433 | 0.321 |
| Culturomica massiliensis | 0 | Not detected | 0.08 | -0.347 | 0.512 | 0.707 |
| Cutibacterium acnes | 0 | Not detected | 0.45 | -0.174 | 1.083 | 0.158 |
| Defluviitalea phaphyphila | 0 | Not detected | -0.06 | -0.508 | 0.393 | 0.804 |
| Dendrosporobacter quercicolus | 0 | Not detected | -0.14 | -0.599 | 0.324 | 0.559 |
| Denitrobacterium detoxificans | 0 | Not detected | 0.13 | -0.429 | 0.685 | 0.652 |
| Desulfovibrio desulfuricans | 0 | Not detected | 0.72 | -0.199 | 1.631 | 0.126 |
| Desulfovibrio fairfieldensis | 0 | Not detected | 0.28 | -0.648 | 1.209 | 0.554 |
| Desulfovibrio piger | 0 | Not detected | 1.02 | -0.271 | 2.318 | 0.123 |
| Desulfovibrio vulgaris | 0 | Not detected | 0.42 | -0.112 | 0.956 | 0.123 |
| Dialister invisus | 0 | Not detected | 0.05 | -1.551 | 1.653 | 0.951 |
| Dialister invisus CAG:218 | 0 | Not detected | 0.00 | -1.649 | 1.657 | 0.996 |
| Dialister micraerophilus | 0 | Not detected | 0.55 | -0.282 | 1.379 | 0.197 |
| Dialister sp. CAG:486 | 0 | Not detected | 0.23 | -0.799 | 1.258 | 0.662 |
| Dialister sp. CAG:588 | 176 | Not detected | 1.13 | 0.444 | 1.808 | 0.001 |
| Dialister succinatiphilus | 0 | Not detected | 0.08 | -0.711 | 0.866 | 0.848 |
| Dielma fastidiosa | 0 | Not detected | 0.86 | 0.114 | 1.601 | 0.025 |
| Dorea formicigenerans | 0 | Not detected | 0.13 | -0.188 | 0.449 | 0.423 |
| Dorea longicatena | 0 | Not detected | -0.09 | -0.535 | 0.352 | 0.687 |
| Dorea sp. 42_8 | 0 | Not detected | -0.02 | -0.526 | 0.489 | 0.944 |
| Dorea sp. 5-2 | 0 | Not detected | 0.02 | -0.211 | 0.249 | 0.870 |
| Dorea sp. CAG:105 | 0 | Not detected | 0.40 | -0.228 | 1.030 | 0.213 |
| Draconibacterium orientale | 0 | Not detected | 0.05 | -0.383 | 0.479 | 0.827 |
| Drancourtella massiliensis | 0 | Not detected | 0.15 | -0.469 | 0.768 | 0.635 |
| Dubosiella newyorkensis | 2 | Not detected | -0.12 | -0.588 | 0.352 | 0.623 |
| Dysgonomonas gadei | 0 | Not detected | 0.00 | -0.401 | 0.392 | 0.983 |
| Dysgonomonas mossii | 0 | Not detected | -0.03 | -0.540 | 0.486 | 0.918 |
| Dysgonomonas sp. BGC7 | 3 | Not detected | -0.67 | -1.311 | -0.031 | 0.041 |
| Dysgonomonas sp. HGC4 | 2 | Not detected | -0.14 | -0.667 | 0.386 | 0.601 |
| Eggerthella lenta | 0 | Not detected | -0.29 | -1.067 | 0.494 | 0.473 |
| Eggerthella sp. 51_9 | 0 | Not detected | 0.52 | -0.263 | 1.308 | 0.193 |
| Eggerthella sp. CAG:209 | 0 | Not detected | 0.00 | -1.086 | 1.084 | 0.999 |
| Eggerthella sp. YY7918 | 0 | Not detected | -0.30 | -0.914 | 0.310 | 0.335 |
| Eggerthellaceae bacterium AT8 | 0 | Not detected | 0.19 | -0.197 | 0.569 | 0.342 |
| Eggerthia catenaformis | 0 | Not detected | 0.88 | 0.170 | 1.596 | 0.016 |
| Eisenbergiella tayi | 343 | Not detected | 0.84 | 0.145 | 1.529 | 0.019 |

| Elizabethkingia anophelis | 0 | Not detected | 0.68 | -0.030 | 1.383 | 0.062 |
| --- | --- | --- | --- | --- | --- | --- |
| Emergencia timonensis | 0 | Not detected | -0.01 | -0.310 | 0.294 | 0.959 |
| Empedobacter brevis | 0 | Not detected | 1.08 | 0.059 | 2.094 | 0.039 |
| Enorma massiliensis | 0 | Not detected | 0.07 | -0.298 | 0.444 | 0.701 |
| Enorma timonensis | 0 | Not detected | 0.62 | 0.082 | 1.159 | 0.025 |
| Enterobacter cloacae | 0 | Not detected | 0.58 | -0.468 | 1.628 | 0.279 |
| Enterobacter lignolyticus | 0 | Not detected | 0.28 | -0.581 | 1.135 | 0.527 |
| Enterobacter sp. 638 | 0 | Not detected | 0.38 | -0.388 | 1.140 | 0.336 |
| Enterococcus avium | 0 | Not detected | 0.09 | -0.591 | 0.775 | 0.792 |
| Enterococcus cecorum | 0 | Not detected | 0.15 | -0.443 | 0.747 | 0.617 |
| Enterococcus columbae | 0 | Not detected | 0.15 | -0.540 | 0.839 | 0.671 |
| Enterococcus durans | 0 | Not detected | -0.31 | -0.992 | 0.375 | 0.377 |
| Enterococcus faecalis | 0 | Not detected | -0.05 | -0.674 | 0.578 | 0.880 |
| Enterococcus faecium | 0 | Not detected | -0.24 | -0.893 | 0.406 | 0.463 |
| Enterococcus gallinarum | 0 | Not detected | 0.10 | -0.534 | 0.733 | 0.759 |
| Enterococcus gilvus | 0 | Not detected | 0.34 | -0.243 | 0.932 | 0.252 |
| Enterococcus italicus | 0 | Not detected | -0.11 | -0.741 | 0.516 | 0.726 |
| Enterococcus massiliensis | 0 | Not detected | 0.15 | -0.467 | 0.761 | 0.639 |
| Enterococcus sp. HMSC064A12 | 0 | Not detected | 0.30 | -0.378 | 0.985 | 0.384 |
| Enterorhabdus caecimuris | 0 | Not detected | -0.38 | -1.103 | 0.345 | 0.306 |
| Enterorhabdus mucosicola | 0 | Not detected | 0.55 | -0.111 | 1.215 | 0.104 |
| Erwinia oleae | 0 | Not detected | 0.55 | -0.156 | 1.257 | 0.128 |
| Erysipelatoclostridium ramosum | 0 | Not detected | -0.20 | -1.121 | 0.714 | 0.664 |
| Erysipelotrichaceae bacterium 21_3 | 0 | Not detected | -0.20 | -0.546 | 0.155 | 0.275 |
| Erysipelotrichaceae bacterium 5_2_54F | 0 | Not detected | 0.08 | -0.295 | 0.446 | 0.690 |
| Erysipelotrichaceae bacterium CAG:64 | 0 | Not detected | 0.08 | -0.555 | 0.721 | 0.799 |
| Erysipelotrichaceae bacterium I46 | 0 | Not detected | -0.09 | -0.458 | 0.270 | 0.614 |
| Escherichia coli | 0 | Not detected | 0.81 | -0.238 | 1.850 | 0.131 |
| Escherichia vulneris | 0 | Not detected | 0.10 | -0.611 | 0.807 | 0.787 |
| Ethanoligenens harbinense | 0 | Not detected | 0.60 | -0.033 | 1.242 | 0.064 |
| Eubacterium dolichum CAG:375 | 0 | Not detected | 0.53 | -0.525 | 1.589 | 0.325 |
| Eubacterium limosum | 0 | Not detected | 0.41 | -0.109 | 0.924 | 0.124 |
| Eubacterium plexicaudatum | 0 | Not detected | 0.06 | -0.192 | 0.302 | 0.661 |
| Eubacterium pyruvativorans | 0 | Not detected | 0.21 | -0.156 | 0.577 | 0.261 |
| Eubacterium ramulus | 0 | Not detected | 0.06 | -0.230 | 0.355 | 0.676 |
| Eubacterium sp. 14-2 | 0 | Not detected | -0.15 | -0.403 | 0.099 | 0.236 |
| Eubacterium sp. 3_1_31 | 0 | Not detected | 0.01 | -0.412 | 0.428 | 0.970 |
| Eubacterium sp. 45_250 | 0 | Not detected | 0.25 | -0.813 | 1.306 | 0.649 |
| Eubacterium sp. AB3007 | 0 | Not detected | 0.48 | -0.357 | 1.323 | 0.261 |
| Eubacterium sp. CAG:115 | 0 | Not detected | 0.91 | -0.490 | 2.314 | 0.204 |
| Eubacterium sp. CAG:146 | 0 | Not detected | 0.58 | -0.136 | 1.305 | 0.113 |
| Eubacterium sp. CAG:180 | 0 | Not detected | 1.47 | 0.248 | 2.700 | 0.019 |
| Eubacterium sp. CAG:192 | 0 | Not detected | 0.90 | -0.102 | 1.900 | 0.080 |
| Eubacterium sp. CAG:202 | 0 | Not detected | 0.56 | -0.335 | 1.446 | 0.223 |

| Eubacterium sp. CAG:248 | 0 | Not detected | 0.49 | -0.316 | 1.292 | 0.235 |
| --- | --- | --- | --- | --- | --- | --- |
| Eubacterium sp. CAG:251 | 0 | Not detected | 1.12 | -0.156 | 2.403 | 0.086 |
| Eubacterium sp. CAG:252 | 0 | Not detected | 0.45 | -0.318 | 1.216 | 0.252 |
| Eubacterium sp. CAG:581 | 0 | Not detected | 0.19 | -0.651 | 1.031 | 0.658 |
| Eubacterium sp. CAG:603 | 0 | Not detected | 0.04 | -0.500 | 0.588 | 0.874 |
| Eubacterium sp. CAG:786 | 0 | Not detected | 0.03 | -0.966 | 1.035 | 0.946 |
| Eubacterium sp. CAG:841 | 0 | Not detected | -0.13 | -1.141 | 0.878 | 0.799 |
| Eubacterium sp. ER2 | 0 | Not detected | 0.02 | -0.258 | 0.293 | 0.900 |
| Eubacterium ventriosum | 0 | Not detected | 0.13 | -0.290 | 0.553 | 0.541 |
| Eubacterium xylanophilum | 0 | Not detected | 0.23 | -0.305 | 0.762 | 0.401 |
| Facklamia hominis | 0 | Not detected | 0.54 | -0.094 | 1.171 | 0.097 |
| Facklamia ignava | 0 | Not detected | 0.24 | -0.359 | 0.841 | 0.432 |
| Facklamia languida | 0 | Not detected | 0.18 | -0.358 | 0.726 | 0.507 |
| Faecalibacterium prausnitzii | 0 | Not detected | 0.27 | -0.295 | 0.830 | 0.353 |
| Faecalibacterium sp. CAG:1138 | 0 | Not detected | 0.00 | -0.716 | 0.708 | 0.992 |
| Faecalibacterium sp. CAG:82-related_59 | 0 | Not detected | 0.39 | -0.460 | 1.234 | 0.371 |
| Faecalibaculum rodentium | 0 | Not detected | 0.56 | -0.117 | 1.230 | 0.106 |
| Faecalicoccus pleomorphus | 0 | Not detected | 0.03 | -0.414 | 0.473 | 0.897 |
| Faecalitalea cylindroides | 0 | Not detected | 0.05 | -0.216 | 0.322 | 0.700 |
| Fenollaria massiliensis | 747 | 0.6 | 1.43 | 0.696 | 2.167 | 0.000 |
| Fermentimonas caenicola | 0 | Not detected | -0.06 | -0.733 | 0.618 | 0.867 |
| Fibrobacter sp. UWB12 | 0 | Not detected | -0.08 | -0.504 | 0.350 | 0.725 |
| Fibrobacter sp. UWCM | 0 | Not detected | 0.27 | -0.264 | 0.813 | 0.318 |
| Fibrobacter sp. UWOV1 | 0 | Not detected | 0.10 | -0.363 | 0.563 | 0.672 |
| Fibrobacterales bacterium Fibro_02 | 8 | Not detected | -0.36 | -0.825 | 0.105 | 0.130 |
| Fibrobacteria bacterium AD80 | 0 | Not detected | -0.24 | -0.738 | 0.259 | 0.347 |
| Filifactor alocis | 0 | Not detected | 0.11 | -0.579 | 0.805 | 0.749 |
| Finegoldia magna | 0 | Not detected | 1.11 | 0.391 | 1.836 | 0.003 |
| Firmicutes bacterium ASF500 | 0 | Not detected | 0.01 | -0.277 | 0.298 | 0.943 |
| Firmicutes bacterium CAG:102 | 0 | Not detected | 0.20 | -0.864 | 1.272 | 0.708 |
| Firmicutes bacterium CAG:137 | 0 | Not detected | 1.17 | 0.004 | 2.335 | 0.050 |
| Firmicutes bacterium CAG:137_57_8 | 0 | Not detected | 0.61 | -0.379 | 1.590 | 0.229 |
| Firmicutes bacterium CAG:145 | 0 | Not detected | 0.96 | -0.138 | 2.054 | 0.088 |
| Firmicutes bacterium CAG:176 | 0 | Not detected | -0.29 | -1.464 | 0.889 | 0.632 |
| Firmicutes bacterium CAG:176_59_8 | 0 | Not detected | 0.02 | -1.066 | 1.108 | 0.970 |
| Firmicutes bacterium CAG:227 | 0 | Not detected | 0.63 | -0.206 | 1.468 | 0.141 |
| Firmicutes bacterium CAG:238 | 0 | Not detected | 0.05 | -0.865 | 0.956 | 0.922 |
| Firmicutes bacterium CAG:24 | 0 | Not detected | 0.27 | -0.492 | 1.032 | 0.488 |
| Firmicutes bacterium CAG:240 | 0 | Not detected | -0.04 | -1.088 | 1.008 | 0.941 |
| Firmicutes bacterium CAG:270 | 0 | Not detected | 0.44 | -0.370 | 1.240 | 0.290 |
| Firmicutes bacterium CAG:272 | 0 | Not detected | 0.30 | -0.589 | 1.186 | 0.510 |
| Firmicutes bacterium CAG:272_52_7 | 0 | Not detected | 0.34 | -0.396 | 1.080 | 0.364 |
| Firmicutes bacterium CAG:308 | 0 | Not detected | 0.11 | -0.454 | 0.667 | 0.711 |
| Firmicutes bacterium CAG:313 | 0 | Not detected | -0.65 | -1.769 | 0.478 | 0.261 |

| Firmicutes bacterium CAG:41 | 0 | Not detected | -0.02 | -0.957 | 0.924 | 0.972 |
| --- | --- | --- | --- | --- | --- | --- |
| Firmicutes bacterium CAG:466 | 0 | Not detected | 0.73 | -0.012 | 1.479 | 0.055 |
| Firmicutes bacterium CAG:475 | 0 | Not detected | -0.23 | -0.906 | 0.439 | 0.496 |
| Firmicutes bacterium CAG:536 | 0 | Not detected | 0.00 | -0.825 | 0.834 | 0.991 |
| Firmicutes bacterium CAG:555 | 0 | Not detected | 0.24 | -0.643 | 1.124 | 0.594 |
| Firmicutes bacterium CAG:65 | 0 | Not detected | 0.14 | -0.684 | 0.969 | 0.735 |
| Firmicutes bacterium CAG:791 | 0 | Not detected | 0.22 | -0.238 | 0.682 | 0.345 |
| Firmicutes bacterium CAG:83 | 0 | Not detected | -0.15 | -1.156 | 0.858 | 0.772 |
| Firmicutes bacterium CAG:882 | 0 | Not detected | -0.40 | -0.981 | 0.187 | 0.184 |
| Firmicutes bacterium CAG:884 | 0 | Not detected | 0.56 | -0.149 | 1.270 | 0.123 |
| Firmicutes bacterium CAG:94 | 0 | Not detected | 0.18 | -0.831 | 1.185 | 0.731 |
| Firmicutes bacterium M10-2 | 0 | Not detected | 0.55 | -0.149 | 1.240 | 0.125 |
| Firmicutes bacterium ZOR0006 | 0 | Not detected | 0.30 | -0.431 | 1.033 | 0.421 |
| Flavonifractor plautii | 0 | Not detected | 0.04 | -0.540 | 0.613 | 0.902 |
| Fournierella massiliensis | 0 | Not detected | 0.00 | -0.296 | 0.306 | 0.975 |
| Franconibacter pulveris | 0 | Not detected | 0.05 | -0.592 | 0.690 | 0.881 |
| Fusicatenibacter saccharivorans | 0 | Not detected | 0.08 | -0.253 | 0.420 | 0.627 |
| Fusobacterium mortiferum | 0 | Not detected | -0.16 | -1.163 | 0.836 | 0.749 |
| Fusobacterium necrophorum | 0 | Not detected | -0.30 | -0.958 | 0.349 | 0.362 |
| Fusobacterium nucleatum | 0 | Not detected | 0.49 | -0.365 | 1.338 | 0.264 |
| Fusobacterium periodonticum | 0 | Not detected | -0.14 | -0.871 | 0.590 | 0.706 |
| Gabonia massiliensis | 0 | Not detected | 0.13 | -0.473 | 0.725 | 0.681 |
| Gabonibacter massiliensis | 0 | Not detected | 0.45 | -0.212 | 1.112 | 0.184 |
| Gallibacterium anatis | 3 | Not detected | -0.21 | -0.767 | 0.353 | 0.469 |
| Gardnerella vaginalis | 0 | Not detected | 0.36 | -0.368 | 1.095 | 0.331 |
| Gemella haemolysans | 3 | Not detected | -0.49 | -1.178 | 0.199 | 0.165 |
| Gemella sanguinis | 0 | Not detected | -0.10 | -0.948 | 0.742 | 0.811 |
| Gemmata obscuriglobus | 0 | Not detected | 0.41 | -0.155 | 0.967 | 0.158 |
| Geofilum rubicundum | 0 | Not detected | 0.02 | -0.523 | 0.564 | 0.941 |
| Globicatella sanguinis | 0 | Not detected | 0.39 | -0.197 | 0.987 | 0.193 |
| Gordonibacter pamelaeae | 0 | Not detected | 0.21 | -0.332 | 0.744 | 0.454 |
| Granulicatella adiacens | 0 | Not detected | -0.32 | -1.104 | 0.473 | 0.434 |
| Granulicatella elegans | 0 | Not detected | -0.06 | -0.716 | 0.599 | 0.862 |
| Haemophilus influenzae | 0 | Not detected | 0.22 | -0.572 | 1.013 | 0.586 |
| Haemophilus parainfluenzae | 0 | Not detected | 0.35 | -0.763 | 1.460 | 0.539 |
| Haemophilus sputorum | 0 | Not detected | 0.16 | -0.748 | 1.078 | 0.724 |
| Hathewaya proteolytica | 0 | Not detected | 0.24 | -0.379 | 0.862 | 0.446 |
| Heliobacterium sp. N15.MGS-306 | 0 | Not detected | -0.12 | -0.477 | 0.233 | 0.502 |
| Hespellia stercorisuis | 0 | Not detected | -0.05 | -0.377 | 0.283 | 0.781 |
| Holdemanella biformis | 0 | Not detected | 0.33 | -0.204 | 0.858 | 0.229 |
| Holdemania filiformis | 0 | Not detected | 0.18 | -0.166 | 0.522 | 0.312 |
| Holdemania massiliensis | 0 | Not detected | -0.13 | -0.520 | 0.267 | 0.531 |
| Holdemania sp. Marseille-P2844 | 0 | Not detected | 0.26 | -0.147 | 0.676 | 0.209 |
| Hungatella hathewayi | 0 | Not detected | 0.32 | -0.023 | 0.660 | 0.069 |

| Hydrogenoanaerobacterium saccharovor | 0 | Not detected | 0.35 | -0.123 | 0.825 | 0.148 |
| --- | --- | --- | --- | --- | --- | --- |
| Idiomarina zobellii | 0 | Not detected | 0.10 | -0.715 | 0.908 | 0.815 |
| Ileibacterium valens | 0 | Not detected | -0.31 | -0.996 | 0.368 | 0.367 |
| Intestinibacter bartlettii | 0 | Not detected | 0.04 | -0.592 | 0.669 | 0.906 |
| Intestinimonas butyriciproducens | 0 | Not detected | 0.11 | -0.384 | 0.607 | 0.659 |
| Intestinimonas massiliensis | 0 | Not detected | -0.06 | -0.462 | 0.344 | 0.773 |
| Jeotgalibaca sp. PTS2502 | 0 | Not detected | 0.01 | -0.422 | 0.450 | 0.951 |
| Johnsonella ignava | 0 | Not detected | 0.00 | -0.367 | 0.361 | 0.988 |
| Kallipyga massiliensis | 0 | Not detected | 0.55 | -0.048 | 1.154 | 0.073 |
| Kandleria vitulina | 0 | Not detected | 0.94 | -0.115 | 1.994 | 0.082 |
| Klebsiella oxytoca | 0 | Not detected | 0.70 | -0.425 | 1.816 | 0.225 |
| Klebsiella pneumoniae | 0 | Not detected | 0.01 | -1.104 | 1.116 | 0.991 |
| Klebsiella sp. RIT-PI-d | 0 | Not detected | 0.06 | -0.657 | 0.778 | 0.869 |
| Kluyvera ascorbata | 0 | Not detected | 0.17 | -0.778 | 1.126 | 0.720 |
| Kluyvera cryocrescens | 0 | Not detected | 0.65 | -0.355 | 1.646 | 0.207 |
| Kluyvera intermedia | 0 | Not detected | 0.97 | -0.039 | 1.987 | 0.061 |
| Kocuria sp. ZOR0020 | 6 | Not detected | -0.66 | -1.178 | -0.132 | 0.015 |
| Kosakonia sacchari | 0 | Not detected | 0.25 | -0.548 | 1.057 | 0.534 |
| Lachnoanaerobaculum saburreum | 0 | Not detected | 0.04 | -0.350 | 0.422 | 0.855 |
| Lachnoanaerobaculum sp. OBRC5-5 | 0 | Not detected | 0.04 | -0.302 | 0.392 | 0.801 |
| Lachnobacterium bovis | 0 | Not detected | -0.18 | -0.522 | 0.156 | 0.291 |
| Lachnoclostridium phytofermentans | 0 | Not detected | 0.05 | -0.201 | 0.303 | 0.691 |
| Lachnospira multipara | 0 | Not detected | 0.00 | -0.490 | 0.488 | 0.996 |
| Lachnospiraceae bacterium 10-1 | 0 | Not detected | -0.05 | -0.268 | 0.165 | 0.641 |
| Lachnospiraceae bacterium 28-4 | 0 | Not detected | 0.01 | -0.267 | 0.293 | 0.928 |
| Lachnospiraceae bacterium 2_1_46FAA | 0 | Not detected | 0.21 | -0.158 | 0.585 | 0.262 |
| Lachnospiraceae bacterium 3-1 | 0 | Not detected | -0.04 | -0.319 | 0.239 | 0.778 |
| Lachnospiraceae bacterium 3-2 | 0 | Not detected | 0.03 | -0.255 | 0.320 | 0.825 |
| Lachnospiraceae bacterium A2 | 0 | Not detected | 0.15 | -0.122 | 0.423 | 0.279 |
| Lachnospiraceae bacterium A4 | 0 | Not detected | 0.00 | -0.245 | 0.241 | 0.984 |
| Lachnospiraceae bacterium AB2028 | 0 | Not detected | 0.12 | -0.539 | 0.787 | 0.715 |
| Lachnospiraceae bacterium AC2012 | 0 | Not detected | -0.05 | -0.403 | 0.303 | 0.782 |
| Lachnospiraceae bacterium AC2014 | 0 | Not detected | -0.22 | -0.747 | 0.302 | 0.407 |
| Lachnospiraceae bacterium AC2029 | 0 | Not detected | 0.01 | -0.373 | 0.390 | 0.966 |
| Lachnospiraceae bacterium AC2031 | 0 | Not detected | 0.02 | -0.478 | 0.521 | 0.932 |
| Lachnospiraceae bacterium AC3007 | 0 | Not detected | 0.28 | -0.214 | 0.778 | 0.266 |
| Lachnospiraceae bacterium AD3010 | 2 | Not detected | -0.31 | -0.899 | 0.284 | 0.310 |
| Lachnospiraceae bacterium C6A11 | 0 | Not detected | -0.17 | -0.654 | 0.314 | 0.493 |
| Lachnospiraceae bacterium COE1 | 0 | Not detected | 0.05 | -0.280 | 0.389 | 0.749 |
| Lachnospiraceae bacterium FD2005 | 0 | Not detected | -0.10 | -0.649 | 0.451 | 0.725 |
| Lachnospiraceae bacterium FE2018 | 0 | Not detected | 0.01 | -0.634 | 0.647 | 0.984 |
| Lachnospiraceae bacterium G41 | 4 | Not detected | -0.28 | -0.692 | 0.142 | 0.197 |
| Lachnospiraceae bacterium KHCPX20 | 0 | Not detected | 0.46 | -0.220 | 1.135 | 0.187 |
| Lachnospiraceae bacterium M18-1 | 0 | Not detected | -0.15 | -0.418 | 0.114 | 0.263 |

| Lachnospiraceae bacterium NC2004 | 0 | Not detected | 0.02 | -0.546 | 0.595 | 0.933 |
| --- | --- | --- | --- | --- | --- | --- |
| Lachnospiraceae bacterium NC2008 | 0 | Not detected | 0.15 | -0.436 | 0.734 | 0.619 |
| Lachnospiraceae bacterium ND2006 | 0 | Not detected | 0.15 | -0.380 | 0.670 | 0.588 |
| Lachnospiraceae bacterium NK3A20 | 0 | Not detected | -0.01 | -0.692 | 0.677 | 0.983 |
| Lachnospiraceae bacterium NK4A144 | 0 | Not detected | -0.12 | -0.548 | 0.303 | 0.573 |
| Lachnospiraceae bacterium NK4A179 | 4 | Not detected | -0.34 | -0.887 | 0.212 | 0.230 |
| Lachnospiraceae bacterium NLAE-zl-G2 | 552 | Not detected | 0.92 | 0.178 | 1.669 | 0.016 |
| Lachnospiraceae bacterium P6B14 | 0 | Not detected | 0.32 | -0.175 | 0.823 | 0.204 |
| Lachnospiraceae bacterium TF01-11 | 0 | Not detected | 0.03 | -0.328 | 0.392 | 0.862 |
| Lachnospiraceae bacterium TWA4 | 0 | Not detected | 0.22 | -0.146 | 0.589 | 0.238 |
| Lachnospiraceae bacterium YSB2008 | 0 | Not detected | -0.18 | -0.640 | 0.278 | 0.441 |
| Lachnospiraceae bacterium mt14 | 1 | Not detected | -0.25 | -0.726 | 0.230 | 0.310 |
| Lachnospiraceae bacterium oral taxon 50 | 0 | Not detected | 0.08 | -0.215 | 0.373 | 0.599 |
| Lactobacillus acidophilus | 0 | Not detected | 0.11 | -0.475 | 0.687 | 0.721 |
| Lactobacillus amylotrophicus | 0 | Not detected | -0.08 | -0.631 | 0.463 | 0.763 |
| Lactobacillus animalis | 0 | Not detected | 0.42 | -0.183 | 1.019 | 0.174 |
| Lactobacillus aviarius | 0 | Not detected | 0.05 | -0.350 | 0.447 | 0.811 |
| Lactobacillus casei | 0 | Not detected | -0.30 | -1.110 | 0.506 | 0.464 |
| Lactobacillus curvatus | 0 | Not detected | -0.06 | -0.647 | 0.534 | 0.851 |
| Lactobacillus delbrueckii | 0 | Not detected | -0.02 | -0.710 | 0.671 | 0.955 |
| Lactobacillus equi | 0 | Not detected | 0.40 | -0.237 | 1.033 | 0.220 |
| Lactobacillus fermentum | 0 | Not detected | -0.02 | -1.014 | 0.976 | 0.970 |
| Lactobacillus gasseri | 0 | Not detected | 0.04 | -0.948 | 1.030 | 0.936 |
| Lactobacillus helveticus | 0 | Not detected | 0.25 | -0.413 | 0.921 | 0.456 |
| Lactobacillus ingluviei | 0 | Not detected | 0.26 | -0.339 | 0.855 | 0.398 |
| Lactobacillus jensenii | 0 | Not detected | -0.19 | -0.824 | 0.439 | 0.551 |
| Lactobacillus manihotivorans | 0 | Not detected | 0.29 | -0.271 | 0.848 | 0.313 |
| Lactobacillus mucosae | 0 | Not detected | 0.60 | -0.023 | 1.214 | 0.060 |
| Lactobacillus paracasei | 0 | Not detected | -0.36 | -1.195 | 0.468 | 0.392 |
| Lactobacillus pasteurii | 0 | Not detected | 0.23 | -0.365 | 0.825 | 0.450 |
| Lactobacillus plantarum | 0 | Not detected | -0.09 | -0.785 | 0.611 | 0.807 |
| Lactobacillus reuteri | 0 | Not detected | 0.09 | -0.760 | 0.933 | 0.841 |
| Lactobacillus rhamnosus | 0 | Not detected | -0.03 | -0.818 | 0.755 | 0.937 |
| Lactobacillus ruminis | 739 | 0.6 | 1.03 | 0.321 | 1.733 | 0.005 |
| Lactobacillus ruminis CAG:367 | 702 | 0.6 | 1.06 | 0.336 | 1.777 | 0.004 |
| Lactobacillus sakei | 0 | Not detected | 0.02 | -0.551 | 0.595 | 0.940 |
| Lactobacillus salivarius | 0 | Not detected | 0.39 | -0.481 | 1.259 | 0.381 |
| Lactobacillus vaginalis | 0 | Not detected | 0.71 | -0.081 | 1.500 | 0.080 |
| Lactococcus garvieae | 6 | Not detected | -0.60 | -1.147 | -0.049 | 0.034 |
| Lactococcus lactis | 0 | Not detected | -0.69 | -1.668 | 0.290 | 0.169 |
| Lactococcus piscium | 0 | Not detected | -0.20 | -0.868 | 0.472 | 0.562 |
| Lactonifactor longoviformis | 0 | Not detected | 0.04 | -0.191 | 0.279 | 0.715 |
| Lamprocystis purpurea | 0 | Not detected | 0.37 | -0.359 | 1.095 | 0.322 |
| Lascolabacillus massiliensis | 5 | Not detected | -0.41 | -0.932 | 0.114 | 0.127 |

| Lautropia mirabilis | 0 | Not detected | 0.02 | -0.550 | 0.588 | 0.948 |
| --- | --- | --- | --- | --- | --- | --- |
| Lawsonia intracellularis | 0 | Not detected | 0.13 | -0.548 | 0.807 | 0.709 |
| Leclercia adecarboxylata | 0 | Not detected | 0.67 | -0.267 | 1.605 | 0.163 |
| Lelliottia amnigena | 0 | Not detected | 1.01 | -0.073 | 2.091 | 0.069 |
| Lentimicrobium saccharophilum | 0 | Not detected | -0.10 | -0.797 | 0.593 | 0.774 |
| Leuconostoc lactis | 3 | Not detected | -0.63 | -1.268 | 0.007 | 0.054 |
| Leuconostoc mesenteroides | 0 | Not detected | -0.22 | -0.889 | 0.447 | 0.517 |
| Levyella massiliensis | 0 | Not detected | 0.03 | -0.368 | 0.432 | 0.875 |
| Libanicoccus massiliensis | 0 | Not detected | 0.70 | 0.019 | 1.378 | 0.045 |
| Luteimonas sp. FCS-9 | 5 | Not detected | -0.26 | -0.722 | 0.200 | 0.268 |
| Lutispora thermophila | 0 | Not detected | -0.04 | -0.620 | 0.540 | 0.893 |
| Macellibacteroides sp. HH-ZS | 0 | Not detected | -0.36 | -0.806 | 0.085 | 0.114 |
| Macrococcus caseolyticus | 0 | Not detected | 0.20 | -0.367 | 0.774 | 0.484 |
| Mageeibacillus indolicus | 0 | Not detected | 0.64 | 0.117 | 1.167 | 0.017 |
| Mangrovibacter sp. MFB070 | 0 | Not detected | 0.13 | -0.517 | 0.773 | 0.698 |
| Mannheimia haemolytica | 2 | Not detected | -0.02 | -0.506 | 0.471 | 0.944 |
| Mariniphaga anaerophila | 0 | Not detected | -0.08 | -0.589 | 0.430 | 0.760 |
| Marvinbryantia formatexigens | 0 | Not detected | 0.17 | -0.173 | 0.518 | 0.328 |
| Massilioclostridium coli | 0 | Not detected | -0.12 | -0.456 | 0.220 | 0.495 |
| Massiliomicrobiota timonensis | 0 | Not detected | 0.42 | -0.225 | 1.068 | 0.203 |
| Mediterranea massiliensis | 0 | Not detected | -0.08 | -0.509 | 0.351 | 0.719 |
| Megamonas funiformis | 0 | Not detected | 0.31 | -1.005 | 1.627 | 0.643 |
| Megasphaera cerevisiae | 0 | Not detected | 1.27 | 0.377 | 2.154 | 0.006 |
| Megasphaera elsdenii | 0 | Not detected | 0.91 | -0.258 | 2.074 | 0.128 |
| Megasphaera genomosp. type_1 | 0 | Not detected | 0.12 | -0.145 | 0.380 | 0.381 |
| Megasphaera massiliensis | 0 | Not detected | 1.76 | 0.409 | 3.117 | 0.011 |
| Megasphaera micronuciformis | 0 | Not detected | 0.64 | -0.154 | 1.438 | 0.115 |
| Megasphaera sp. BV3C16-1 | 0 | Not detected | 0.85 | 0.143 | 1.562 | 0.019 |
| Megasphaera sp. UPII 135-E | 0 | Not detected | 0.03 | -0.256 | 0.309 | 0.854 |
| Merdibacter massiliensis | 0 | Not detected | 0.02 | -0.271 | 0.303 | 0.911 |
| Methanobrevibacter smithii | 0 | Not detected | 0.20 | -1.152 | 1.544 | 0.776 |
| Methanobrevibacter smithii CAG:186 | 0 | Not detected | 0.10 | -1.117 | 1.321 | 0.870 |
| Methanocorpusculaceae archaeon Phil4 | 0 | Not detected | -0.33 | -1.043 | 0.376 | 0.358 |
| Methanosarcina mazei | 0 | Not detected | 0.16 | -0.533 | 0.863 | 0.644 |
| Methylobacterium sp. ZNC0032 | 9 | Not detected | -0.61 | -1.134 | -0.091 | 0.022 |
| Mitsuokella multacida | 0 | Not detected | 1.11 | 0.005 | 2.210 | 0.050 |
| Mobilibacterium timonense | 0 | Not detected | 0.02 | -0.534 | 0.582 | 0.933 |
| Mobiluncus curtisii | 0 | Not detected | 0.10 | -0.217 | 0.418 | 0.535 |
| Mobiluncus mulieris | 0 | Not detected | 0.14 | -0.258 | 0.537 | 0.492 |
| Mogibacterium timidum | 0 | Not detected | 0.60 | -0.099 | 1.302 | 0.093 |
| Morganella morganii | 0 | Not detected | 0.21 | -0.439 | 0.855 | 0.529 |
| Mucinivorans hirudinis | 0 | Not detected | 0.47 | -0.023 | 0.953 | 0.063 |
| Murdochiella sp. Marseille-P2341 | 0 | Not detected | 0.03 | -0.349 | 0.416 | 0.863 |
| Mycoplasma sp. CAG:877 | 0 | Not detected | 0.15 | -0.583 | 0.874 | 0.696 |

| Mycoplasma sp. CAG:956 | 0 | Not detected | 0.05 | -0.605 | 0.713 | 0.873 |
| --- | --- | --- | --- | --- | --- | --- |
| Myroides injenensis | 0 | Not detected | 0.26 | -0.507 | 1.037 | 0.502 |
| Myroides odoratimimus | 0 | Not detected | 0.28 | -0.591 | 1.158 | 0.525 |
| Negativicoccus massiliensis | 0 | Not detected | 0.57 | -0.013 | 1.144 | 0.057 |
| Neglecta timonensis | 0 | Not detected | 0.14 | -0.365 | 0.641 | 0.591 |
| Neisseria cinerea | 0 | Not detected | 0.01 | -0.586 | 0.607 | 0.972 |
| Nissabacter archeti | 0 | Not detected | 0.27 | -0.362 | 0.909 | 0.399 |
| Novibacillus thermophilus | 0 | Not detected | 0.01 | -0.589 | 0.611 | 0.972 |
| Obesumbacterium proteus | 0 | Not detected | 0.78 | -0.074 | 1.629 | 0.075 |
| Odoribacter laneus | 0 | Not detected | -0.18 | -0.889 | 0.524 | 0.613 |
| Odoribacter laneus CAG:561 | 0 | Not detected | 0.34 | -0.514 | 1.203 | 0.432 |
| Odoribacter sp. 43_10 | 0 | Not detected | 0.14 | -0.479 | 0.767 | 0.652 |
| Odoribacter sp. CAG:788 | 0 | Not detected | -0.06 | -0.819 | 0.697 | 0.875 |
| Odoribacter splanchnicus | 0 | Not detected | -0.06 | -0.514 | 0.399 | 0.805 |
| Odoribacter splanchnicus CAG:14 | 0 | Not detected | 0.02 | -0.656 | 0.704 | 0.944 |
| Oligella ureolytica | 0 | Not detected | 0.13 | -0.478 | 0.731 | 0.681 |
| Olsenella sp. Marseille-P3197 | 0 | Not detected | 0.30 | -0.365 | 0.971 | 0.374 |
| Olsenella sp. oral taxon 809 | 0 | Not detected | 0.34 | -0.125 | 0.808 | 0.152 |
| Olsenella uli | 0 | Not detected | -0.14 | -0.812 | 0.536 | 0.689 |
| Olsenella umbonata | 0 | Not detected | 0.77 | 0.256 | 1.285 | 0.004 |
| Oribacterium parvum | 0 | Not detected | 0.03 | -0.211 | 0.270 | 0.808 |
| Oribacterium sinus | 0 | Not detected | -0.16 | -0.741 | 0.425 | 0.596 |
| Oribacterium sp. WCC10 | 0 | Not detected | 0.00 | -0.635 | 0.628 | 0.992 |
| Oribacterium sp. oral taxon 078 | 0 | Not detected | 0.17 | -0.249 | 0.599 | 0.420 |
| Oscillibacter ruminantium | 0 | Not detected | 0.19 | -0.349 | 0.732 | 0.488 |
| Oscillibacter sp. 1-3 | 0 | Not detected | 0.17 | -0.155 | 0.497 | 0.304 |
| Oscillibacter sp. 57_20 | 0 | Not detected | 0.26 | -0.810 | 1.325 | 0.637 |
| Oscillibacter sp. ER4 | 0 | Not detected | 0.22 | -0.410 | 0.847 | 0.496 |
| Oscillibacter sp. KLE 1745 | 0 | Not detected | 0.16 | -0.316 | 0.631 | 0.514 |
| Oscillibacter valericigenes | 0 | Not detected | 0.19 | -0.257 | 0.639 | 0.404 |
| Oscillospiraceae bacterium Zagget12 | 0 | Not detected | -0.32 | -0.920 | 0.286 | 0.304 |
| Oxalobacter formigenes | 0 | Not detected | 0.28 | -0.285 | 0.843 | 0.334 |
| Paeniclostridium sordellii | 0 | Not detected | -0.02 | -0.708 | 0.663 | 0.948 |
| Paludibacter propionicigenes | 2 | Not detected | -0.19 | -0.690 | 0.304 | 0.447 |
| Paludibacter sp. 47-17 | 0 | Not detected | 0.30 | -0.259 | 0.854 | 0.296 |
| Pantoea sp. PSNIH2 | 0 | Not detected | 0.81 | -0.109 | 1.731 | 0.085 |
| Parabacteroides distasonis | 0 | Not detected | -0.20 | -0.786 | 0.392 | 0.513 |
| Parabacteroides goldsteinii | 0 | Not detected | -0.09 | -0.705 | 0.520 | 0.767 |
| Parabacteroides gordonii | 0 | Not detected | 0.02 | -0.508 | 0.557 | 0.927 |
| Parabacteroides johnsonii | 0 | Not detected | -0.13 | -0.603 | 0.344 | 0.593 |
| Parabacteroides merdae CAG:48 | 0 | Not detected | -0.21 | -1.087 | 0.675 | 0.647 |
| Paraburkholderia sacchari | 0 | Not detected | 0.20 | -0.410 | 0.803 | 0.525 |
| Paraclostridium bifermentans | 0 | Not detected | 0.06 | -0.554 | 0.676 | 0.845 |
| Paraprevotella clara CAG:116 | 0 | Not detected | 0.09 | -0.874 | 1.063 | 0.848 |

| Paraprevotella xylaniphila | 0 | Not detected | 0.02 | -0.504 | 0.549 | 0.934 |
| --- | --- | --- | --- | --- | --- | --- |
| Parascardovia denticolens | 0 | Not detected | -0.14 | -0.796 | 0.523 | 0.685 |
| Parasporobacterium paucivorans | 0 | Not detected | 0.16 | -0.313 | 0.625 | 0.514 |
| Parasutterella excrementihominis | 0 | Not detected | 0.19 | -0.832 | 1.204 | 0.720 |
| Parasutterella excrementihominis CAG:2 | 0 | Not detected | 0.66 | -0.756 | 2.072 | 0.363 |
| Parvimonas micra | 0 | Not detected | -0.03 | -0.723 | 0.658 | 0.927 |
| Pelosinus fermentans | 0 | Not detected | -0.05 | -0.574 | 0.475 | 0.854 |
| Peptoanaerobacter stomatis | 12 | Not detected | -0.41 | -0.923 | 0.099 | 0.116 |
| Peptococcus niger | 0 | Not detected | 0.31 | -0.211 | 0.840 | 0.241 |
| Peptoniphilus duerdenii | 309 | Not detected | 0.92 | 0.448 | 1.400 | 0.000 |
| Peptoniphilus grossensis | 0 | Not detected | 1.01 | 0.338 | 1.681 | 0.004 |
| Peptoniphilus harei | 199 | Not detected | 1.03 | 0.335 | 1.721 | 0.004 |
| Peptoniphilus lacrimalis | 0 | Not detected | 0.87 | 0.113 | 1.631 | 0.025 |
| Peptoniphilus obesi | 0 | Not detected | 0.38 | -0.318 | 1.074 | 0.288 |
| Peptoniphilus rhinitidis | 0 | Not detected | 0.33 | -0.316 | 0.981 | 0.316 |
| Peptoniphilus senegalensis | 0 | Not detected | 0.66 | -0.045 | 1.368 | 0.068 |
| Peptoniphilus sp. BV3C26 | 0 | Not detected | 0.28 | -0.466 | 1.020 | 0.466 |
| Peptoniphilus sp. ING2-D1G | 0 | Not detected | -0.05 | -0.701 | 0.601 | 0.881 |
| Peptoniphilus sp. oral taxon 375 | 0 | Not detected | 0.19 | -0.164 | 0.549 | 0.290 |
| Peptostreptococcaceae bacterium VA2 | 0 | Not detected | 0.32 | -0.472 | 1.112 | 0.429 |
| Peptostreptococcus anaerobius | 0 | Not detected | 0.07 | -0.245 | 0.389 | 0.655 |
| Peptostreptococcus anaerobius CAG:621 | 0 | Not detected | 0.38 | -0.235 | 1.004 | 0.225 |
| Peptostreptococcus stomatis | 0 | Not detected | 0.55 | -0.075 | 1.169 | 0.086 |
| Petrimonas mucosa | 0 | Not detected | -0.08 | -0.708 | 0.551 | 0.807 |
| Phaeodactylibacter xiamenensis | 0 | Not detected | 0.05 | -0.808 | 0.917 | 0.902 |
| Phascolarctobacterium sp. CAG:207 | 0 | Not detected | 0.24 | -1.188 | 1.664 | 0.744 |
| Phascolarctobacterium sp. CAG:266 | 0 | Not detected | -0.15 | -0.981 | 0.675 | 0.717 |
| Phascolarctobacterium succinatutens | 0 | Not detected | 0.43 | -0.311 | 1.164 | 0.258 |
| Phocaeicola abscessus | 0 | Not detected | -0.04 | -0.512 | 0.432 | 0.868 |
| Phocea massiliensis | 0 | Not detected | 0.01 | -0.349 | 0.371 | 0.954 |
| Pluralibacter gergoviae | 0 | Not detected | 0.40 | -0.391 | 1.190 | 0.323 |
| Pontibacillus litoralis | 0 | Not detected | 0.18 | -0.732 | 1.091 | 0.699 |
| Porphyromonadaceae bacterium COT-18 | 0 | Not detected | 0.24 | -0.345 | 0.820 | 0.425 |
| Porphyromonadaceae bacterium KH3CP | 0 | Not detected | 0.10 | -0.391 | 0.592 | 0.689 |
| Porphyromonadaceae bacterium KH3R1 | 0 | Not detected | -0.21 | -0.761 | 0.339 | 0.453 |
| Porphyromonas asaccharolytica | 0 | Not detected | 0.53 | -0.250 | 1.302 | 0.185 |
| Porphyromonas bennonis | 0 | Not detected | 0.17 | -0.530 | 0.874 | 0.632 |
| Porphyromonas cangingivalis | 0 | Not detected | 0.23 | -0.323 | 0.774 | 0.421 |
| Porphyromonas canoris | 0 | Not detected | -0.17 | -0.788 | 0.449 | 0.592 |
| Porphyromonas catoniae | 0 | Not detected | -0.10 | -0.560 | 0.356 | 0.663 |
| Porphyromonas crevioricanis | 0 | Not detected | 0.12 | -0.403 | 0.642 | 0.654 |
| Porphyromonas endodontalis | 0 | Not detected | -0.19 | -0.811 | 0.440 | 0.562 |
| Porphyromonas gingivalis | 0 | Not detected | 0.07 | -0.451 | 0.591 | 0.793 |
| Porphyromonas gingivicanis | 0 | Not detected | 0.32 | -0.128 | 0.777 | 0.161 |

| Porphyromonas levii | 0 | Not detected | 0.78 | -0.105 | 1.669 | 0.085 |
| --- | --- | --- | --- | --- | --- | --- |
| Porphyromonas macacae | 0 | Not detected | 0.20 | -0.372 | 0.776 | 0.491 |
| Porphyromonas somerae | 0 | Not detected | -0.05 | -0.426 | 0.325 | 0.793 |
| Porphyromonas sp. CAG:1061 | 0 | Not detected | 0.09 | -0.660 | 0.835 | 0.819 |
| Porphyromonas sp. COT-239 OH1446 | 0 | Not detected | -0.22 | -1.103 | 0.658 | 0.620 |
| Porphyromonas sp. COT-290 OH3588 | 5 | Not detected | -0.43 | -1.006 | 0.150 | 0.148 |
| Prevotella albensis | 0 | Not detected | -0.01 | -1.116 | 1.100 | 0.989 |
| Prevotella amnii | 0 | Not detected | -0.09 | -0.469 | 0.291 | 0.646 |
| Prevotella aurantiaca | 0 | Not detected | 0.10 | -0.622 | 0.829 | 0.780 |
| Prevotella baroniae | 0 | Not detected | 0.05 | -0.391 | 0.495 | 0.819 |
| Prevotella bergensis | 0 | Not detected | 0.11 | -0.470 | 0.686 | 0.714 |
| Prevotella bivia | 0 | Not detected | 0.09 | -0.329 | 0.516 | 0.665 |
| Prevotella bryantii | 0 | Not detected | 0.20 | -0.909 | 1.315 | 0.721 |
| Prevotella buccae | 0 | Not detected | -0.17 | -0.990 | 0.648 | 0.683 |
| Prevotella buccalis | 0 | Not detected | -0.01 | -0.434 | 0.415 | 0.965 |
| Prevotella copri | 0 | Not detected | 0.29 | -0.941 | 1.529 | 0.642 |
| Prevotella corporis | 0 | Not detected | -0.08 | -0.526 | 0.360 | 0.715 |
| Prevotella dentalis | 0 | Not detected | 0.23 | -0.711 | 1.173 | 0.631 |
| Prevotella dentasini | 0 | Not detected | 0.15 | -0.648 | 0.951 | 0.710 |
| Prevotella denticola | 0 | Not detected | -0.24 | -1.189 | 0.710 | 0.621 |
| Prevotella disiens | 0 | Not detected | 0.07 | -0.339 | 0.473 | 0.746 |
| Prevotella enoeca | 0 | Not detected | 0.02 | -0.839 | 0.872 | 0.969 |
| Prevotella fusca | 0 | Not detected | 0.15 | -0.909 | 1.215 | 0.778 |
| Prevotella histicola | 0 | Not detected | -0.15 | -0.837 | 0.547 | 0.681 |
| Prevotella intermedia | 0 | Not detected | 0.23 | -0.418 | 0.871 | 0.492 |
| Prevotella loescheii | 0 | Not detected | -0.16 | -0.876 | 0.564 | 0.672 |
| Prevotella maculosa | 0 | Not detected | -0.23 | -0.981 | 0.524 | 0.552 |
| Prevotella marshii | 0 | Not detected | -0.09 | -0.564 | 0.376 | 0.695 |
| Prevotella melaninogenica | 0 | Not detected | 0.02 | -0.676 | 0.716 | 0.955 |
| Prevotella micans | 0 | Not detected | 0.13 | -0.683 | 0.939 | 0.757 |
| Prevotella multiformis | 0 | Not detected | 0.06 | -0.512 | 0.626 | 0.845 |
| Prevotella multisaccharivorax | 0 | Not detected | -0.64 | -1.687 | 0.410 | 0.234 |
| Prevotella nanceiensis | 0 | Not detected | -0.08 | -0.854 | 0.700 | 0.846 |
| Prevotella nigrescens | 0 | Not detected | 0.22 | -0.514 | 0.957 | 0.556 |
| Prevotella oralis | 0 | Not detected | -0.21 | -1.003 | 0.589 | 0.611 |
| Prevotella oris | 0 | Not detected | 0.13 | -0.807 | 1.066 | 0.787 |
| Prevotella oryzae | 0 | Not detected | -0.01 | -0.970 | 0.943 | 0.977 |
| Prevotella oulorum | 0 | Not detected | 0.42 | -0.584 | 1.423 | 0.413 |
| Prevotella pallens | 0 | Not detected | -0.17 | -0.660 | 0.323 | 0.503 |
| Prevotella paludivivens | 0 | Not detected | -0.10 | -0.652 | 0.446 | 0.713 |
| Prevotella ruminicola | 0 | Not detected | -0.09 | -1.020 | 0.833 | 0.843 |
| Prevotella saccharolytica | 0 | Not detected | 0.34 | -0.535 | 1.212 | 0.448 |
| Prevotella salivae | 0 | Not detected | -0.10 | -0.532 | 0.336 | 0.658 |
| Prevotella scopos | 0 | Not detected | -0.14 | -0.698 | 0.414 | 0.617 |

| Prevotella sp. 10(H) | 4 | Not detected | -0.21 | -0.722 | 0.295 | 0.412 |
| --- | --- | --- | --- | --- | --- | --- |
| Prevotella sp. 109 | 0 | Not detected | -0.06 | -0.484 | 0.372 | 0.798 |
| Prevotella sp. AGR2160 | 0 | Not detected | 0.24 | -0.859 | 1.347 | 0.665 |
| Prevotella sp. CAG:1058 | 0 | Not detected | 0.43 | -0.410 | 1.279 | 0.314 |
| Prevotella sp. CAG:1092 | 0 | Not detected | 0.43 | -0.598 | 1.455 | 0.414 |
| Prevotella sp. CAG:1124 | 0 | Not detected | -0.13 | -1.002 | 0.747 | 0.775 |
| Prevotella sp. CAG:1185 | 0 | Not detected | 0.54 | -0.426 | 1.510 | 0.274 |
| Prevotella sp. CAG:1320 | 0 | Not detected | 0.18 | -0.643 | 1.009 | 0.665 |
| Prevotella sp. CAG:255 | 0 | Not detected | -0.07 | -0.728 | 0.583 | 0.828 |
| Prevotella sp. CAG:386 | 0 | Not detected | 0.09 | -1.108 | 1.285 | 0.885 |
| Prevotella sp. CAG:487 | 0 | Not detected | 0.19 | -0.414 | 0.799 | 0.534 |
| Prevotella sp. CAG:520 | 0 | Not detected | 0.24 | -0.835 | 1.315 | 0.662 |
| Prevotella sp. CAG:5226 | 0 | Not detected | -0.39 | -1.421 | 0.633 | 0.453 |
| Prevotella sp. CAG:604 | 0 | Not detected | 0.63 | -0.710 | 1.969 | 0.358 |
| Prevotella sp. CAG:732 | 0 | Not detected | 0.41 | -0.818 | 1.628 | 0.517 |
| Prevotella sp. CAG:891 | 0 | Not detected | 0.19 | -0.781 | 1.166 | 0.699 |
| Prevotella sp. FD3004 | 0 | Not detected | -0.06 | -0.722 | 0.603 | 0.860 |
| Prevotella sp. HUN102 | 0 | Not detected | 0.12 | -0.382 | 0.618 | 0.645 |
| Prevotella sp. MSX73 | 3 | Not detected | -0.35 | -1.120 | 0.417 | 0.371 |
| Prevotella sp. P4-76 | 0 | Not detected | -0.07 | -0.718 | 0.572 | 0.824 |
| Prevotella sp. RM4 | 2 | Not detected | -0.24 | -0.779 | 0.295 | 0.378 |
| Prevotella sp. S7 MS 2 | 0 | Not detected | -0.14 | -0.596 | 0.314 | 0.545 |
| Prevotella sp. S7-1-8 | 0 | Not detected | 0.05 | -0.370 | 0.462 | 0.830 |
| Prevotella sp. kh1p2 | 0 | Not detected | -0.13 | -0.958 | 0.694 | 0.754 |
| Prevotella sp. khp1 | 0 | Not detected | -0.05 | -0.588 | 0.479 | 0.841 |
| Prevotella sp. ne3005 | 2 | Not detected | -0.16 | -0.614 | 0.290 | 0.483 |
| Prevotella sp. oral taxon 473 | 0 | Not detected | 0.40 | -0.418 | 1.221 | 0.338 |
| Prevotella stercorea | 0 | Not detected | 0.00 | -0.507 | 0.504 | 0.995 |
| Prevotella stercorea CAG:629 | 0 | Not detected | 0.58 | -0.523 | 1.676 | 0.305 |
| Prevotella timonensis | 0 | Not detected | -0.08 | -0.490 | 0.339 | 0.722 |
| Prevotellamassilia timonensis | 0 | Not detected | 0.20 | -0.369 | 0.773 | 0.489 |
| Propionibacterium acidifaciens | 0 | Not detected | -0.17 | -0.806 | 0.461 | 0.594 |
| Proteiniclasticum ruminis | 0 | Not detected | -0.18 | -0.633 | 0.279 | 0.448 |
| Proteiniphilum acetatigenes | 0 | Not detected | 0.04 | -0.459 | 0.529 | 0.889 |
| Proteiniphilum saccharofermentans | 0 | Not detected | -0.14 | -0.712 | 0.427 | 0.625 |
| Proteiniphilum sp. EBM-41 | 0 | Not detected | 0.03 | -0.790 | 0.840 | 0.952 |
| Proteobacteria bacterium CAG:139 | 0 | Not detected | 0.33 | -1.023 | 1.679 | 0.635 |
| Proteobacteria bacterium CAG:495 | 0 | Not detected | -0.65 | -1.790 | 0.493 | 0.266 |
| Providencia rettgeri | 0 | Not detected | 0.02 | -0.589 | 0.634 | 0.942 |
| Pseudobacteroides cellulosolvens | 0 | Not detected | 0.24 | -0.234 | 0.706 | 0.326 |
| Pseudobutyrivibrio ruminis | 0 | Not detected | -0.06 | -0.360 | 0.248 | 0.717 |
| Pseudobutyrivibrio sp. ACV-2 | 2 | Not detected | -0.18 | -0.727 | 0.369 | 0.523 |
| Pseudobutyrivibrio sp. JW11 | 3 | Not detected | -0.21 | -0.769 | 0.357 | 0.474 |
| Pseudobutyrivibrio sp. MD2005 | 0 | Not detected | 0.08 | -0.516 | 0.684 | 0.784 |

| Pseudobutyrivibrio sp. UC1225 | 0 | Not detected | 0.36 | -0.089 | 0.804 | 0.118 |
| --- | --- | --- | --- | --- | --- | --- |
| Pseudobutyrivibrio sp. YE44 | 6 | Not detected | -0.49 | -1.047 | 0.059 | 0.081 |
| Pseudobutyrivibrio xylanivorans | 0 | Not detected | -0.29 | -0.855 | 0.284 | 0.327 |
| Pseudoflavonifractor capillosus | 0 | Not detected | 0.01 | -0.320 | 0.336 | 0.961 |
| Pseudopropionibacterium propionicum | 0 | Not detected | 0.25 | -0.399 | 0.890 | 0.456 |
| Pyramidobacter piscolens | 0 | Not detected | 0.24 | -0.615 | 1.085 | 0.588 |
| Ralstonia mannitolilytica | 0 | Not detected | 0.08 | -0.696 | 0.858 | 0.839 |
| Ralstonia pickettii | 0 | Not detected | 0.14 | -0.572 | 0.854 | 0.698 |
| Raoultella ornithinolytica | 0 | Not detected | 0.53 | -0.506 | 1.563 | 0.318 |
| Rhodococcus defluvii | 0 | Not detected | -0.07 | -0.633 | 0.483 | 0.793 |
| Rhodospirillum sp. UNK.MGS-17 | 0 | Not detected | 0.37 | -0.805 | 1.554 | 0.534 |
| Riemerella columbina | 0 | Not detected | -0.14 | -0.550 | 0.274 | 0.512 |
| Rikenella microfusus | 0 | Not detected | -0.16 | -0.571 | 0.261 | 0.466 |
| Risungbinella massiliensis | 0 | Not detected | 0.27 | -0.187 | 0.725 | 0.249 |
| Robinsoniella peoriensis | 0 | Not detected | -0.12 | -0.357 | 0.109 | 0.298 |
| Roseburia faecis | 0 | Not detected | 0.03 | -0.569 | 0.631 | 0.920 |
| Roseburia hominis | 0 | Not detected | 0.17 | -0.197 | 0.532 | 0.369 |
| Roseburia intestinalis | 0 | Not detected | 0.02 | -0.519 | 0.556 | 0.946 |
| Roseburia inulinivorans | 0 | Not detected | 0.03 | -0.404 | 0.469 | 0.883 |
| Roseburia sp. CAG:197_41_10 | 0 | Not detected | 0.09 | -0.361 | 0.541 | 0.696 |
| Roseburia sp. CAG:309 | 0 | Not detected | -0.52 | -1.461 | 0.421 | 0.280 |
| Roseburia sp. CAG:380 | 0 | Not detected | -0.14 | -0.870 | 0.599 | 0.718 |
| Rothia dentocariosa | 0 | Not detected | -0.75 | -1.563 | 0.070 | 0.075 |
| Rothia mucilaginosa | 0 | Not detected | 0.16 | -0.777 | 1.105 | 0.733 |
| Ruminococcaceae bacterium AE2021 | 0 | Not detected | 0.06 | -0.345 | 0.472 | 0.761 |
| Ruminococcaceae bacterium D16 | 0 | Not detected | -0.13 | -0.435 | 0.166 | 0.382 |
| Ruminococcaceae bacterium YRB3002 | 0 | Not detected | -0.39 | -0.921 | 0.148 | 0.158 |
| Ruminococcus albus | 0 | Not detected | 0.70 | 0.078 | 1.332 | 0.029 |
| Ruminococcus bicirculans | 0 | Not detected | 0.73 | -0.234 | 1.696 | 0.139 |
| Ruminococcus callidus | 0 | Not detected | 0.62 | 0.013 | 1.219 | 0.046 |
| Ruminococcus champanellensis | 0 | Not detected | 0.37 | -0.425 | 1.167 | 0.362 |
| Ruminococcus faecis | 0 | Not detected | 0.16 | -0.259 | 0.580 | 0.454 |
| Ruminococcus flavefaciens | 0 | Not detected | 0.59 | -0.005 | 1.189 | 0.053 |
| Ruminococcus gauvreauii | 0 | Not detected | -0.11 | -0.414 | 0.199 | 0.492 |
| Ruminococcus lactaris | 0 | Not detected | -0.14 | -0.600 | 0.329 | 0.568 |
| Ruminococcus obeum CAG:39 | 0 | Not detected | 0.64 | 0.078 | 1.202 | 0.026 |
| Ruminococcus sp. AT10 | 0 | Not detected | 0.16 | -0.109 | 0.439 | 0.239 |
| Ruminococcus sp. CAG:108-related_41_ | 0 | Not detected | -0.16 | -1.149 | 0.836 | 0.758 |
| Ruminococcus sp. CAG:177 | 0 | Not detected | 0.01 | -1.449 | 1.465 | 0.992 |
| Ruminococcus sp. CAG:254 | 0 | Not detected | 1.15 | -0.070 | 2.371 | 0.066 |
| Ruminococcus sp. CAG:330 | 0 | Not detected | 1.56 | 0.303 | 2.810 | 0.016 |
| Ruminococcus sp. CAG:379 | 0 | Not detected | 0.14 | -0.703 | 0.986 | 0.743 |
| Ruminococcus sp. CAG:382 | 0 | Not detected | -0.80 | -1.692 | 0.096 | 0.082 |
| Ruminococcus sp. CAG:403 | 363 | Not detected | 1.18 | 0.208 | 2.153 | 0.018 |

| Ruminococcus sp. CAG:488 | 0 | Not detected | 0.09 | -0.653 | 0.833 | 0.812 |
| --- | --- | --- | --- | --- | --- | --- |
| Ruminococcus sp. CAG:563 | 0 | Not detected | 0.28 | -0.489 | 1.049 | 0.477 |
| Ruminococcus sp. CAG:60 | 0 | Not detected | 0.37 | -0.185 | 0.923 | 0.193 |
| Ruminococcus sp. CAG:624 | 0 | Not detected | 0.54 | -0.235 | 1.319 | 0.173 |
| Ruminococcus sp. CAG:724 | 0 | Not detected | -0.01 | -0.766 | 0.748 | 0.981 |
| Ruminococcus sp. FC2018 | 0 | Not detected | 0.78 | 0.155 | 1.401 | 0.015 |
| Ruminococcus sp. HUN007 | 0 | Not detected | 0.58 | 0.028 | 1.124 | 0.041 |
| Ruminococcus sp. NK3A76 | 0 | Not detected | 0.68 | 0.004 | 1.351 | 0.050 |
| Ruminococcus sp. Phil15 | 0 | Not detected | -0.15 | -0.947 | 0.654 | 0.720 |
| Ruminococcus sp. Zagget7 | 0 | Not detected | 0.11 | -0.352 | 0.575 | 0.638 |
| Ruminococcus torques CAG:61 | 0 | Not detected | 0.93 | 0.093 | 1.757 | 0.030 |
| Ruthenibacterium lactatiformans | 0 | Not detected | 0.20 | -0.269 | 0.661 | 0.409 |
| Salmonella enterica | 0 | Not detected | 0.74 | -0.226 | 1.707 | 0.134 |
| Sanguibacteroides justesenii | 0 | Not detected | -0.25 | -0.887 | 0.396 | 0.454 |
| Scardovia inopinata | 0 | Not detected | 0.19 | -0.354 | 0.739 | 0.491 |
| Scardovia wiggsiae | 0 | Not detected | -0.05 | -0.751 | 0.648 | 0.885 |
| Selenomonas bovis | 196 | Not detected | 0.70 | 0.219 | 1.190 | 0.005 |
| Selenomonas ruminantium | 0 | Not detected | 0.77 | 0.116 | 1.428 | 0.022 |
| Selenomonas sputigena | 0 | Not detected | 0.53 | -0.052 | 1.104 | 0.076 |
| Sellimonas intestinalis | 0 | Not detected | 0.14 | -0.364 | 0.649 | 0.582 |
| Senegalimassilia anaerobia | 0 | Not detected | 0.77 | -0.011 | 1.552 | 0.055 |
| Sharpea azabuensis | 0 | Not detected | 0.35 | -0.002 | 0.708 | 0.052 |
| Shigella dysenteriae | 0 | Not detected | 0.91 | -0.172 | 1.990 | 0.101 |
| Shigella flexneri | 0 | Not detected | 0.80 | -0.266 | 1.869 | 0.143 |
| Shimwellia blattae | 0 | Not detected | -0.02 | -0.717 | 0.673 | 0.951 |
| Shuttleworthia satelles | 0 | Not detected | 0.39 | -0.049 | 0.827 | 0.083 |
| Siccibacter turicensis | 0 | Not detected | 0.14 | -0.598 | 0.873 | 0.714 |
| Slackia exigua | 2 | Not detected | 0.02 | -0.577 | 0.627 | 0.935 |
| Slackia heliotrinireducens | 0 | Not detected | 0.14 | -0.348 | 0.636 | 0.567 |
| Slackia piriformis | 0 | Not detected | 0.88 | -0.165 | 1.920 | 0.100 |
| Solobacterium moorei | 0 | Not detected | -0.17 | -0.596 | 0.250 | 0.424 |
| Sphaerochaeta sp. Phil3 | 0 | Not detected | 0.10 | -0.683 | 0.886 | 0.800 |
| Sphingobacterium wenxiniae | 0 | Not detected | 0.19 | -0.559 | 0.933 | 0.625 |
| Spirochaetales bacterium Spiro_07 | 2 | Not detected | -0.16 | -0.665 | 0.341 | 0.529 |
| Sporobacter termitidis | 0 | Not detected | 0.33 | -0.287 | 0.941 | 0.297 |
| Sporomusa ovata | 0 | Not detected | 0.16 | -0.451 | 0.775 | 0.605 |
| Staphylococcus pseudintermedius | 0 | Not detected | 0.22 | -0.370 | 0.814 | 0.463 |
| Staphylococcus sp. CAG:324 | 0 | Not detected | 0.28 | -0.819 | 1.382 | 0.617 |
| Staphylococcus sp. DORA_6_22 | 0 | Not detected | -0.25 | -0.849 | 0.342 | 0.404 |
| Staphylococcus warneri | 0 | Not detected | -0.20 | -0.826 | 0.416 | 0.519 |
| Stomatobaculum longum | 0 | Not detected | 0.06 | -0.175 | 0.291 | 0.624 |
| Streptobacillus notomytis | 0 | Not detected | -0.02 | -0.585 | 0.546 | 0.946 |
| Streptococcus agalactiae | 0 | Not detected | 0.42 | -0.301 | 1.151 | 0.253 |
| Streptococcus anginosus | 0 | Not detected | 0.72 | -0.044 | 1.487 | 0.066 |

| Streptococcus caballi | 0 | Not detected | 0.27 | -0.377 | 0.908 | 0.419 |
| --- | --- | --- | --- | --- | --- | --- |
| Streptococcus cristatus | 0 | Not detected | 0.32 | -0.275 | 0.910 | 0.294 |
| Streptococcus dysgalactiae | 0 | Not detected | -0.07 | -0.714 | 0.580 | 0.839 |
| Streptococcus entericus | 0 | Not detected | 0.33 | -0.270 | 0.925 | 0.284 |
| Streptococcus gallolyticus | 0 | Not detected | 0.49 | -0.366 | 1.355 | 0.261 |
| Streptococcus gordonii | 0 | Not detected | 0.63 | -0.190 | 1.453 | 0.133 |
| Streptococcus halotolerans | 3 | Not detected | -0.30 | -0.788 | 0.183 | 0.223 |
| Streptococcus henryi | 0 | Not detected | 0.10 | -0.476 | 0.681 | 0.729 |
| Streptococcus ictaluri | 0 | Not detected | -0.04 | -0.570 | 0.488 | 0.880 |
| Streptococcus infantis | 0 | Not detected | -0.09 | -0.721 | 0.543 | 0.783 |
| Streptococcus iniae | 3 | Not detected | -0.27 | -0.819 | 0.279 | 0.336 |
| Streptococcus intermedius | 0 | Not detected | 0.14 | -0.608 | 0.881 | 0.720 |
| Streptococcus massiliensis | 0 | Not detected | -0.07 | -0.640 | 0.509 | 0.823 |
| Streptococcus mitis | 0 | Not detected | -0.01 | -0.506 | 0.479 | 0.958 |
| Streptococcus mutans | 0 | Not detected | 0.74 | -0.149 | 1.629 | 0.104 |
| Streptococcus oralis | 0 | Not detected | 0.20 | -0.482 | 0.881 | 0.567 |
| Streptococcus orisratti | 0 | Not detected | -0.41 | -1.070 | 0.253 | 0.228 |
| Streptococcus ovis | 0 | Not detected | 0.04 | -0.521 | 0.605 | 0.884 |
| Streptococcus parasanguinis | 0 | Not detected | 0.23 | -0.557 | 1.013 | 0.569 |
| Streptococcus parauberis | 3 | Not detected | -0.27 | -0.820 | 0.284 | 0.343 |
| Streptococcus plurextorum | 0 | Not detected | 0.13 | -0.275 | 0.536 | 0.528 |
| Streptococcus porci | 0 | Not detected | -0.10 | -0.554 | 0.349 | 0.658 |
| Streptococcus pseudoporcinus | 0 | Not detected | 0.59 | -0.075 | 1.255 | 0.083 |
| Streptococcus pyogenes | 0 | Not detected | 0.06 | -0.590 | 0.714 | 0.852 |
| Streptococcus salivarius | 0 | Not detected | 0.03 | -0.669 | 0.725 | 0.937 |
| Streptococcus sanguinis | 0 | Not detected | 0.61 | -0.209 | 1.425 | 0.146 |
| Streptococcus sobrinus | 0 | Not detected | 0.18 | -0.422 | 0.776 | 0.563 |
| Streptococcus suis | 0 | Not detected | 0.20 | -0.220 | 0.617 | 0.353 |
| Streptococcus thermophilus | 0 | Not detected | -0.14 | -0.919 | 0.629 | 0.714 |
| Streptococcus uberis | 2 | Not detected | -0.21 | -0.732 | 0.320 | 0.444 |
| Streptococcus urinalis | 0 | Not detected | -0.07 | -0.692 | 0.562 | 0.839 |
| Streptomyces albulus | 10 | Not detected | -0.39 | -0.908 | 0.135 | 0.147 |
| Streptomyces cattleya | 0 | Not detected | 0.24 | -0.452 | 0.938 | 0.493 |
| Subdoligranulum sp. CAG:314 | 0 | Not detected | -0.12 | -1.521 | 1.287 | 0.870 |
| Subdoligranulum variabile | 0 | Not detected | 0.27 | -0.043 | 0.591 | 0.091 |
| Succinatimonas sp. CAG:777 | 0 | Not detected | -0.08 | -1.154 | 1.002 | 0.891 |
| Succiniclasticum ruminis | 0 | Not detected | 0.39 | -0.211 | 0.995 | 0.204 |
| Succinispira mobilis | 0 | Not detected | 0.19 | -0.452 | 0.835 | 0.560 |
| Succinivibrio sp. Phil9 | 0 | Not detected | 0.15 | -0.881 | 1.179 | 0.777 |
| Sulfolobus acidocaldarius | 0 | Not detected | 0.22 | -0.599 | 1.049 | 0.593 |
| Sutterella parvirubra | 0 | Not detected | 0.71 | -0.019 | 1.435 | 0.058 |
| Sutterella sp. CAG:397 | 0 | Not detected | -0.86 | -2.158 | 0.431 | 0.192 |
| Sutterella sp. CAG:521 | 0 | Not detected | -0.20 | -0.783 | 0.380 | 0.498 |
| Sutterella wadsworthensis | 763 | 0.6 | 1.80 | 0.461 | 3.138 | 0.009 |

| Sutterella wadsworthensis CAG:135 | 0 | Not detected | 1.31 | -0.301 | 2.926 | 0.112 |
| --- | --- | --- | --- | --- | --- | --- |
| Sutterellaceae bacterium ND3 | 0 | Not detected | 0.91 | 0.015 | 1.800 | 0.047 |
| Synergistales bacterium Syner_02 | 0 | Not detected | 0.27 | -0.436 | 0.978 | 0.453 |
| Synergistes sp. 3_1_syn1 | 0 | Not detected | -0.40 | -1.288 | 0.480 | 0.371 |
| Synergistes sp. Zagget9 | 0 | Not detected | -0.15 | -0.722 | 0.421 | 0.606 |
| Tannerella forsythia | 0 | Not detected | -0.02 | -0.483 | 0.449 | 0.943 |
| Tepidanaerobacter sp. EBM-49 | 0 | Not detected | 0.21 | -0.739 | 1.163 | 0.662 |
| Terrisporobacter glycolicus | 0 | Not detected | -0.09 | -0.823 | 0.641 | 0.807 |
| Thermophagus xiamenensis | 3 | Not detected | -0.39 | -1.062 | 0.281 | 0.255 |
| Thermosinus carboxydivorans | 0 | Not detected | 0.16 | -0.339 | 0.659 | 0.530 |
| Tidjanibacter massiliensis | 0 | Not detected | 0.51 | -0.490 | 1.509 | 0.319 |
| Tissierellia bacterium S7-1-4 | 0 | Not detected | 0.76 | 0.058 | 1.462 | 0.035 |
| Trabulsiella odontotermitis | 0 | Not detected | 0.29 | -0.514 | 1.103 | 0.476 |
| Traorella massiliensis | 0 | Not detected | 0.12 | -0.147 | 0.396 | 0.369 |
| Treponema denticola | 0 | Not detected | 0.34 | -0.184 | 0.873 | 0.203 |
| Treponema medium | 0 | Not detected | -0.37 | -1.236 | 0.492 | 0.400 |
| Treponema putidum | 0 | Not detected | 0.54 | 0.043 | 1.028 | 0.034 |
| Treponema socranskii | 0 | Not detected | 0.35 | -0.196 | 0.902 | 0.209 |
| Treponema succinifaciens | 0 | Not detected | -0.10 | -0.429 | 0.236 | 0.570 |
| Treponema vincentii | 0 | Not detected | 0.10 | -0.405 | 0.605 | 0.698 |
| Trichococcus flocculiformis | 0 | Not detected | 0.20 | -0.393 | 0.796 | 0.506 |
| Trueperella pyogenes | 0 | Not detected | 0.02 | -0.607 | 0.651 | 0.946 |
| Turicibacter sanguinis | 0 | Not detected | 0.62 | -0.395 | 1.626 | 0.234 |
| Turicibacter sp. H121 | 0 | Not detected | -0.11 | -0.521 | 0.297 | 0.592 |
| Tyzzerella nexilis | 0 | Not detected | -0.09 | -0.356 | 0.169 | 0.486 |
| Urmitella timonensis | 98 | Not detected | 0.69 | 0.193 | 1.185 | 0.007 |
| Vaginella massiliensis | 0 | Not detected | 0.28 | -0.562 | 1.121 | 0.516 |
| Varibaculum cambriense | 218 | Not detected | 0.49 | 0.059 | 0.927 | 0.027 |
| Varibaculum sp. Marseille-P2802 | 0 | Not detected | 0.58 | -0.038 | 1.198 | 0.067 |
| Veillonella magna | 0 | Not detected | -0.16 | -0.462 | 0.149 | 0.316 |
| Veillonella parvula | 0 | Not detected | -0.61 | -1.598 | 0.387 | 0.233 |
| Veillonella seminalis | 0 | Not detected | 0.26 | -0.181 | 0.702 | 0.249 |
| Veillonella sp. ACP1 | 0 | Not detected | -0.21 | -1.300 | 0.881 | 0.707 |
| Veillonella sp. CAG:933 | 0 | Not detected | 0.06 | -0.595 | 0.712 | 0.862 |
| Veillonella sp. oral taxon 780 | 0 | Not detected | -0.28 | -0.936 | 0.380 | 0.408 |
| Waddlia chondrophila | 0 | Not detected | 0.66 | -0.138 | 1.461 | 0.106 |
| Weissella cibaria | 0 | Not detected | 0.03 | -0.606 | 0.672 | 0.918 |
| Weissella confusa | 0 | Not detected | -0.03 | -0.794 | 0.743 | 0.948 |
| Xanthomonas sp. Mitacek01 | 2 | Not detected | -0.13 | -0.529 | 0.273 | 0.532 |
| Yersinia enterocolitica | 0 | Not detected | 0.63 | -0.072 | 1.325 | 0.080 |
| Yersinia pestis | 0 | Not detected | 0.60 | -0.378 | 1.586 | 0.229 |
| Yokenella regensburgei | 0 | Not detected | 0.27 | -0.511 | 1.059 | 0.495 |
| [Bacteroides] pectinophilus | 0 | Not detected | -0.01 | -0.333 | 0.321 | 0.972 |
| [Clostridium] aminophilum | 0 | Not detected | 0.15 | -0.131 | 0.433 | 0.295 |

| [Clostridium] asparagiforme | 0 | Not detected | 0.06 | -0.326 | 0.454 | 0.748 |
| --- | --- | --- | --- | --- | --- | --- |
| [Clostridium] bolteae | 0 | Not detected | -0.06 | -0.418 | 0.301 | 0.750 |
| [Clostridium] celerecrescens | 0 | Not detected | 0.04 | -0.207 | 0.293 | 0.735 |
| [Clostridium] cellobioparum | 0 | Not detected | 0.15 | -0.303 | 0.597 | 0.523 |
| [Clostridium] cellulolyticum | 0 | Not detected | 0.05 | -0.569 | 0.667 | 0.876 |
| [Clostridium] cellulosi | 0 | Not detected | 0.27 | -0.340 | 0.873 | 0.389 |
| [Clostridium] citroniae | 0 | Not detected | 0.16 | -0.074 | 0.402 | 0.177 |
| [Clostridium] clariflavum | 2 | Not detected | -0.18 | -0.641 | 0.276 | 0.436 |
| [Clostridium] clostridioforme | 0 | Not detected | -0.24 | -0.669 | 0.183 | 0.265 |
| [Clostridium] dakarense | 0 | Not detected | 0.14 | -0.593 | 0.880 | 0.703 |
| [Clostridium] glycyrrhizinilyticum | 0 | Not detected | 0.10 | -0.145 | 0.349 | 0.420 |
| [Clostridium] hiranonis | 0 | Not detected | 0.09 | -0.712 | 0.899 | 0.821 |
| [Clostridium] hylemonae | 0 | Not detected | 0.15 | -0.434 | 0.737 | 0.613 |
| [Clostridium] innocuum | 0 | Not detected | -0.05 | -0.327 | 0.228 | 0.727 |
| [Clostridium] josui | 0 | Not detected | -0.18 | -0.831 | 0.462 | 0.577 |
| [Clostridium] lactatifermentans | 0 | Not detected | 0.12 | -0.235 | 0.470 | 0.513 |
| [Clostridium] leptum | 0 | Not detected | -0.33 | -0.827 | 0.172 | 0.200 |
| [Clostridium] methylpentosum | 0 | Not detected | -0.24 | -0.696 | 0.221 | 0.311 |
| [Clostridium] papyrosolvens | 0 | Not detected | 0.19 | -0.226 | 0.606 | 0.373 |
| [Clostridium] saccharogumia | 0 | Not detected | -0.05 | -0.411 | 0.304 | 0.769 |
| [Clostridium] saccharolyticum | 0 | Not detected | -0.14 | -0.522 | 0.240 | 0.468 |
| [Clostridium] scindens | 0 | Not detected | -0.26 | -0.594 | 0.075 | 0.130 |
| [Clostridium] spiroforme | 0 | Not detected | -0.15 | -0.927 | 0.623 | 0.701 |
| [Clostridium] sporosphaeroides | 0 | Not detected | 0.30 | -0.286 | 0.893 | 0.314 |
| [Clostridium] symbiosum | 0 | Not detected | 0.21 | -0.125 | 0.536 | 0.225 |
| [Clostridium] viride | 0 | Not detected | 0.14 | -0.413 | 0.692 | 0.621 |
| [Enterobacter] aerogenes | 0 | Not detected | -0.11 | -1.145 | 0.934 | 0.842 |
| [Eubacterium] brachy | 0 | Not detected | 0.13 | -0.570 | 0.824 | 0.722 |
| [Eubacterium] cellulosolvens | 0 | Not detected | 0.07 | -0.243 | 0.378 | 0.670 |
| [Eubacterium] contortum | 0 | Not detected | 0.14 | -0.105 | 0.378 | 0.269 |
| [Eubacterium] dolichum | 0 | Not detected | 0.10 | -0.173 | 0.371 | 0.475 |
| [Eubacterium] eligens | 0 | Not detected | -0.07 | -0.737 | 0.600 | 0.841 |
| [Eubacterium] fissicatena | 0 | Not detected | 0.08 | -0.224 | 0.378 | 0.617 |
| [Eubacterium] hallii | 0 | Not detected | 0.10 | -0.377 | 0.577 | 0.681 |
| [Eubacterium] nodatum | 0 | Not detected | -0.15 | -0.816 | 0.519 | 0.664 |
| [Eubacterium] rectale | 0 | Not detected | 0.09 | -0.544 | 0.732 | 0.773 |
| [Eubacterium] siraeum | 0 | Not detected | 0.07 | -0.849 | 0.984 | 0.885 |
| [Eubacterium] sulci | 0 | Not detected | 0.11 | -0.634 | 0.858 | 0.769 |
| [Ruminococcus] gnavus | 0 | Not detected | -0.01 | -0.542 | 0.515 | 0.960 |
| [Ruminococcus] torques | 0 | Not detected | 0.68 | 0.143 | 1.227 | 0.014 |
| bacterium F082 | 0 | Not detected | 0.03 | -0.391 | 0.444 | 0.902 |
| bacterium LF-3 | 0 | Not detected | -0.31 | -0.973 | 0.344 | 0.350 |
| bacterium MS4 | 0 | Not detected | -0.19 | -0.753 | 0.379 | 0.518 |
| bacterium OL-1 | 0 | Not detected | 0.79 | 0.019 | 1.558 | 0.046 |

| bacterium P201 | 0 | Not detected | 0.04 | -0.640 | 0.716 | 0.913 |
| --- | --- | --- | --- | --- | --- | --- |
| bacterium P3 | 0 | Not detected | 0.02 | -0.600 | 0.630 | 0.962 |
